# The Transcriptome and Methylome of the Developing and Aging Brain and Their Relations to Gliomas and Psychological Disorders

**DOI:** 10.3390/cells11030362

**Published:** 2022-01-21

**Authors:** Henry Loeffler-Wirth, Lydia Hopp, Maria Schmidt, Roksana Zakharyan, Arsen Arakelyan, Hans Binder

**Affiliations:** 1 Interdisciplinary Centre for Bioinformatics, Universität Leipzig, Härtelstr. 16–18, 04107 Leipzig, Germany; wirth@izbi.uni-leipzig.de (H.L.-W.); lydia.hopp@gmx.ne (L.H.); schmidt@izbi.uni-leipzig.de (M.S.); 2 Institute of Molecular Biology, the National Academy of Sciences of the Republic of Armenia, 7 Hasratyan Str., Yerevan 0014, Armenia; roksana.zakharyan@rau.am (R.Z.); aarakelyan@sci.am (A.A.); 3 Institute of Biomedicine and Pharmacy, Russian-Armenian University, 123 Hovsep Emin Str., Yerevan 0051, Armenia; 4 Armenian Bioinformatics Institute (ABI), 7 Hasratyan Str., Yerevan 0014, Armenia

**Keywords:** human brain, development and aging, gene expression, DNA methylation, chromatin remodeling, epigenetics, bioinformatics, machine learning

## Abstract

Mutually linked expression and methylation dynamics in the brain govern genome regulation over the whole lifetime with an impact on cognition, psychological disorders, and cancer. We performed a joint study of gene expression and DNA methylation of brain tissue originating from the human prefrontal cortex of individuals across the lifespan to describe changes in cellular programs and their regulation by epigenetic mechanisms. The analysis considers previous knowledge in terms of functional gene signatures and chromatin states derived from independent studies, aging profiles of a battery of chromatin modifying enzymes, and data of gliomas and neuropsychological disorders for a holistic view on the development and aging of the brain. Expression and methylation changes from babies to elderly adults decompose into different modes associated with the serial activation of (brain) developmental, learning, metabolic and inflammatory functions, where methylation in gene promoters mostly represses transcription. Expression of genes encoding methylome modifying enzymes is very diverse reflecting complex regulations during lifetime which also associates with the marked remodeling of chromatin between permissive and restrictive states. Data of brain cancer and psychotic disorders reveal footprints of pathophysiologies related to brain development and aging. Comparison of aging brains with gliomas supports the view that glioblastoma-like and astrocytoma-like tumors exhibit higher cellular plasticity activated in the developing healthy brain while oligodendrogliomas have a more stable differentiation hierarchy more resembling the aged brain. The balance and specific shifts between volatile and stable and between more irreversible and more plastic epigenomic networks govern the development and aging of healthy and diseased brain.

## 1. Introduction

The human brain relies on the lifelong function of its diverse neuronal cell types. Most neurons emerge during development in the fetal and baby’s brain and need to be maintained throughout adulthood. Neuronal health is governed by cellular programs, which need to be flexible to mediate neuronal plasticity, and yet stable to express a constant set of cell-type-specific genes throughout the life of the neurons [1]. During aging, the mechanisms that normally maintain health, stress resistance, and brain function decline, resulting in decrepitude, frailty, and the increased risk of aging-associated psychotic and neurodegenerative disorders and also of cancer [2]. The dysregulation of transcriptional and chromatin networks is a crucial component of aging [3]. Hereby, epigenetic, changes including DNA methylation, histone modifications, and chromatin remodeling, profoundly affect cellular function, thereby contributing to the progression of aging and to age-related declines in cognition. Additionally, neuronal differentiation in early life is tightly regulated by transcriptional and epigenetic mechanisms [4] with impact also for high-order cognitive functions such as learning and memory [5]. Molecular shaping during prenatal development via DNA methylation, histone modifications, and other molecular constituents of the epigenome are likely to play a critical role in the maintenance of neuronal health and function throughout the entire lifespan [6]. The dysregulation of any of these mechanisms can result in neuropsychiatric disorders, such as depression, schizophrenia, and autism spectrum disorders [2,7]. Previous studies show that gene expression and DNA-methylation dynamics are largest from birth through infancy, after which these molecular profiles transition to a relatively stable state by young adulthood [8,9]. Later in life, DNA methylation drifts with age [10] with impact on other epigenetic marks, such as histone modification, in turn affecting chromatin states [11]. Overall, altered DNA methylation is among the central mechanisms in development, aging, and cellular senescence [12].

The aging and development of brain cancer are two interrelated processes, with aging being a major risk factor for the initiation of cancer. Analysis of epigenetic aging signatures of 25 cancer types revealed that DNA methylation patterns hardly reflect chronological age of cancer patients, but they are coherently modified in a non-stochastic manner, particularly at CpG (CG rich; see list of abbreviations below) islands that become hypermethylated upon aging in non-malignant tissues [13]. Brain cancer cells are diverse in their genetic, metabolic. and micro-environmental compositions, accounting for their phenotypic heterogeneity and disparate responses to therapy. These factors converge at the level of the epigenome, where aberrant epigenomes define many childhood and adult brain cancers, as demonstrated by widespread changes of DNA methylation patterns, redistribution of histone marks. and disruption of chromatin structure [14]. This coordinated regulation of cancer signatures seems to resemble aspects of aging and development.

**Figure 1:**
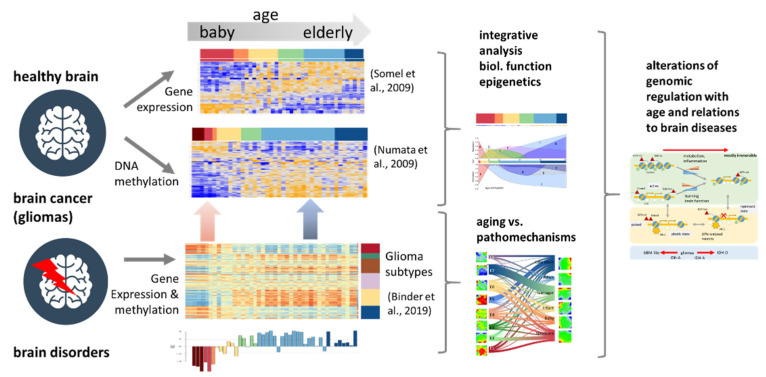
Schematic overview: Gene expression and DNA-methylation data of the prefrontal cortex have been analyzed as a function of age and compared with data taken from gliomas and brain disorders in order to study alterations of modes of (epi-)genomic regulation [9,17,21].

In a wider context, mutually linked expression and methylation dynamics in the brain govern regulatory mechanisms in the genome during development and aging occurring over the whole lifetime and determining the developmental stage and later the onset and the progression of aging [15]. Quantifying the extent and identity of transcriptional and epigenetic changes is therefore important for understanding development, aging. and related disorders and cancer [16]. In this publication, we have analyzed transcriptome and DNA-methylation data collected separately from human brain samples over the whole lifespan [9,17] (Figure 1). Our integrated analysis aims at systematically linking transcriptional programs and DNA-methylation changes as a function of age between early childhood and late adulthood. We decomposed molecular aging profiles into characteristic modes using self-organizing maps data portrayal, a machine learning approach proven in previous large-scale studies on transcriptome [18,19] and joint transcriptome/methylome data [20,21,22]. Hereby, we aimed to identify the functional context of the modes and their relation to epigenetic mechanisms such as the expression of genes encoding chromatin-modifying enzymes and of genes located in different chromatin states [23,24]. Finally, we compare the aging profiles of the healthy brain with expression and methylation data of brain cancer (lower-grade gliomas) and with gene signatures derived from psychotic disorders [16] in order to identify footprints of the respective pathophysiologies related to brain development and aging.

## 2. Materials and Methods

### 2.1. Gene Expression Data

Microarray-based gene expression data (Affymetrix HT-HG-U133A arrays) of postmortem human dorsolateral prefrontal cortex (named for short “brain” throughout the paper) were taken from the GEO database under accession number GSE11512 (see also [9,25]) covering the expression of, in total, 52,656 Affymetrix probe sets. We divided the 44 brain tissue samples into different age groups ranging from Newborn to Late adult: newborn (0–4 months), baby (5–12 months), infant (1–9 years), teenager (10–19 years), adult (20–59 years), and late adult (>60 years).

### 2.2. DNA Methylation Data

Microarray-derived DNA methylation data (Infinium HumanMethylation27 BeadChips) were taken from Numata et al. [17] in units of beta values of ~27,000 CpG’s located in the range of −1500 bp to +500 bp around the transcription start sites of 13,540 genes, thus serving as markers for their promoter methylation. They refer to, in total, 43 prefrontal cortex samples obtained from postmortem human brains of non-psychiatric controls. Mean beta values averaged over the CpGs in the promoter range estimate the level of methylation between values of zero (no methylation) and unity (full methylation) for each gene promoter. Genes located on chromosomes X and Y were excluded from further analyses to minimize gender-specific effects. The brain methylation samples were classified into age groups as follows: Fetal (before birth), Newborn, Baby, Infant, Teenager, Adult, Late adult using the same age ranges applied to gene expression samples. Only individuals of Caucasian ethnicity were considered.

### 2.3. Preprocessing of the Data

We used preprocessed gene expression and methylation data obtained from microarray experiments as provided by the data sources. Methylation data were rescaled from beta-scale (fraction of methylated CpG) into ratio-scale (ratio of methylated to unmethylated CpG in each promoter range). Finally, expression and methylation data were log-transformed and centralized with respect to the mean value of each gene averaged over all samples.

### 2.4. SOM Portrayal

For downstream data processing (feature cluster selection and functional analysis), we made use of the omics “portrayal” method “oposSOM” developed by our group, a machine learning based pipeline using self-organizing maps (SOM) [26,27]. “oposSOM” was separately applied to the preprocessed expression and methylation data. It transferred the high-dimensional genomic data into data of reduced dimensionality by simultaneously keeping their intrinsic data structure unchanged. Genes were clustered into so-called metagenes obtained via iterative machine learning based on their expression and methylation profiles over all samples. The metagenes arrange in a quadratic grid of size 40 × 40 for each sample thus providing one “personalized” SOM image of its expression or methylation landscape after coloring the metagenes according to their expression or methylation level using a color scale ranging from dark blue (low expression/methylation) to maroon (high expression/methylation). After supervised clustering into metagenes, we performed unsupervised clustering of metagenes into so-called spot-modules representing clusters of co-expressed/co-methylated genes [26]. Functional analysis was performed using gene set analysis using a collection of more than 6000 gene sets available in “oposSOM” [27,28]. Specific applications of SOM-portrayal to methylation array data inclusive preprocessing were described previously [21,22,23,29].

### 2.5. Chromatin States

For the analysis of epigenetic regulation, we used different chromatin states of the fetal and adult (prefrontal cortex) human brain, which were established previously on a genome-wide scale [30] (see [31] for methodical details). First, we identified the genes in the genomic regions which were assigned to different states such as TssA, TssP (active and poised gene promoters, respectively), Het (heterochromatin), or RepPRC (genes, repressed via polycomb repressive complex) separately for the two developmental stages (fetal and adult brain). Then, genes were divided into three strata for each state, namely into those which change their chromatin state upon development and were found either in fetal or adult brain only, and into “overlap” genes, which do not change their state and were observed in both states. Finally, we considered genes in each of the strata of each state as a gene set and calculated their mean expression and methylation values as a function of age.

### 2.6. Extension SOM (exSOM) of Glioma Data

The extension SOM method (exSOM) [32] aims at adding secondary data to an already existing SOM without changing its metagene structure. We used exSOM to add expression and methylation data of lower-grade glioma (LGG) taken from [21] as secondary data to the respective expression and methylation SOM of the healthy developing brain.

## 3. Results

### 3.1. The Transcriptome of the Aging Brain

**Figure 2:**
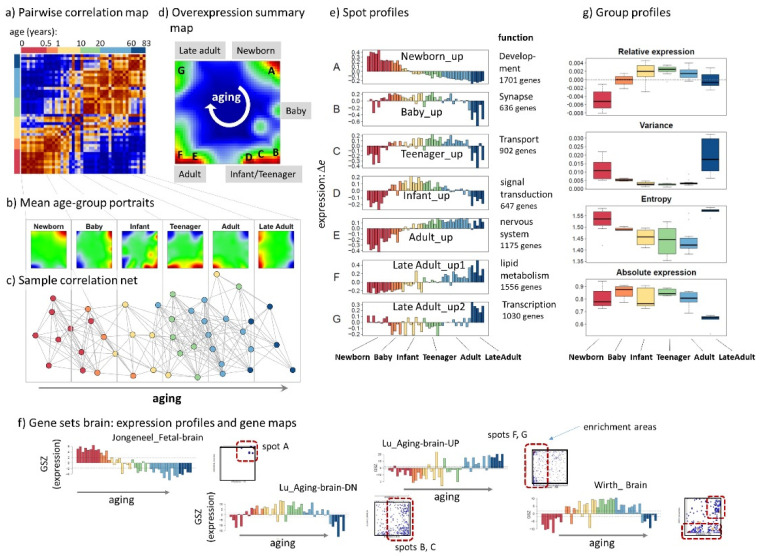
Gene expression characteristics of the developing and aging brain: (**a**) The pairwise correlation map (PCM) of samples’ SOM portraits indicates continuous alterations of the brain transcriptome upon aging in terms of a “stripe” of mutually correlated samples along the diagonal. (**b**) Mean SOM portraits of different age strata reveal distinct spot patterns of activated genes. (**c**) The sample similarity net reflects a virtually one-dimensional development of the brain from the left to the right. (**d**) The overexpression summary map indicates seven spots (“A”–“G”) of co-expressed genes. Upon aging spots activate in a clockwise direction as indicated by the arrow. (**e**) The spot profiles reveal specific over- and under-expression in different age intervals. Δe is the differential log-expression with respect to the mean expression of each gene averaged over all samples (see Table A1). (**f**) Expression profiles of gene sets of brain aging are supported by our age courses of the brain transcriptome. Gene sets were taken from [26,33,34]. (**g**) Boxplots of different expression measures as a function of the age-strata indicate a transition between developmental and aging regimes in juveniles (infant, teenager) evident as an “hourglass”-like shape of relative expression, variance, and entropy along the age axis.

**Figure A1:**
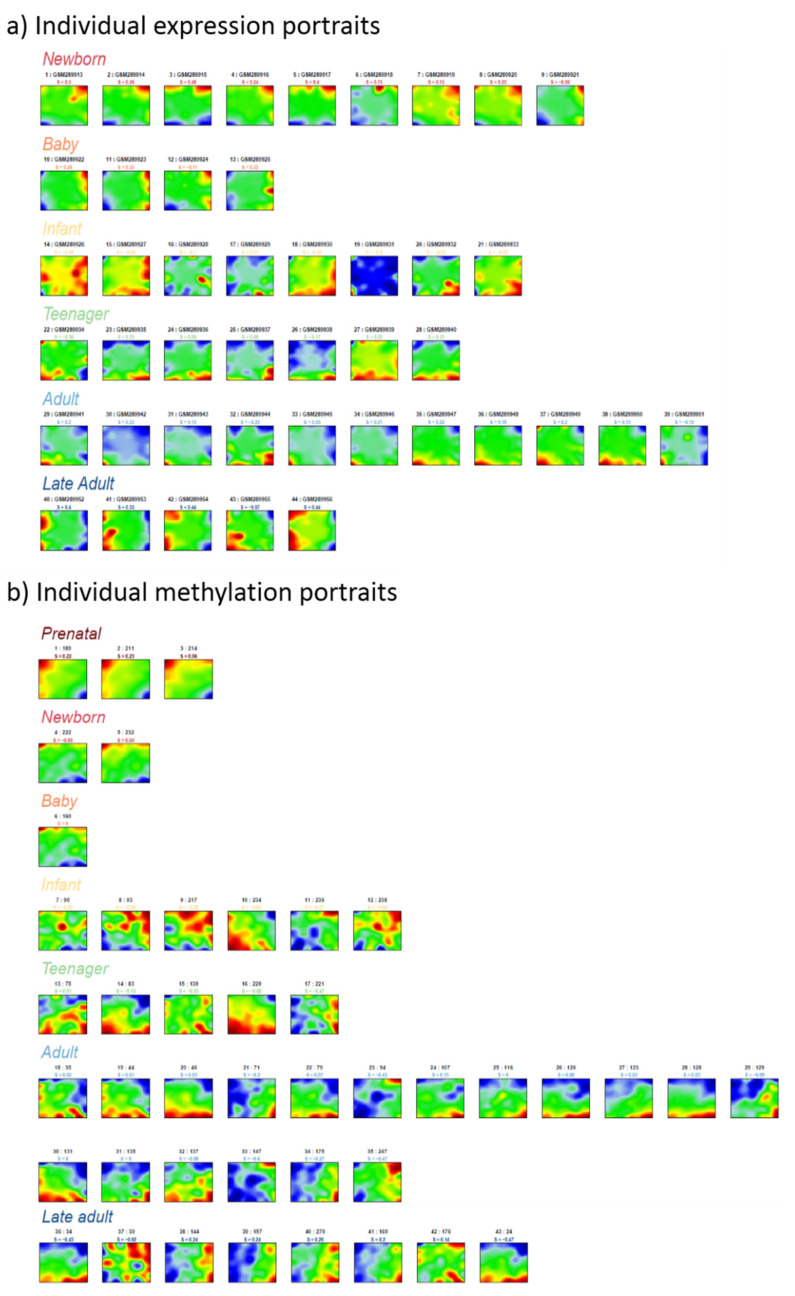
Gallery of individual SOM portraits of (**a**) expression and (**b**) methylation samples divided into age groups.

We generated SOM portraits of all brain transcriptomes studied (Figure A1a). The pairwise correlation map (PCM, Figure 2a) visualizes mutual correlations between all these SOM portraits using a color code ranging from dark blue to maroon for negative to positive values of Pearson correlation coefficient, respectively. Samples were sorted along the axes with increasing age from the left to the right and from bottom to top and grouped into six age intervals ranging from newborn to late adults as indicated in the color bar above the PCM. The maroon ribbon along the diagonal reveals continuous alterations of the expression landscape with age starting from newborn to late adults. Mean portraits of each group are characterized by red and blue over- and underexpression spots (Figure 2b). They represent clusters of correlated metagenes, whose expression strength changes in an age-specific fashion: The pattern of red overexpression spots “rotates” clockwise with increasing age, which reflects activation of transcriptional programs in consecutive order. Moreover, one finds antagonistic switching of spot patterns of newborn and adults (and also of baby and late adult) meaning that transcriptional programs which are activated in early phases of development become deactivated upon aging and vice versa. The correlation net visualizes the similarity relations between the brain samples forming a virtually one-dimensional stripe along the age axis (Figure 2c). The overexpression summary map provides an overview of all spots observed in the group-averaged portraits. Overall, we find seven spots labelled by capital letters A–G (Figure 2d). Their expression profiles reveal different courses with maxima ranging from newborns to late adults age, respectively (Figure 2e). For example, spot A activates in newborns and deactivates in late adults, while spot D shows maximum expression during childhood and adolescence and virtually low expression in newborns and elderly people. The functional context of the spots was estimated using gene set enrichment analysis (Table A1). Activated transcriptional programs shift from developmental, nervous function (e.g., “synapse”) via metabolism towards inflammatory responses upon aging. Gene expression signatures taken from independent studies agree with these results (Figure 2f). For example, “fetal brain genes” [33] show maximum expression in newborns and accumulate in spot A, while gene expression signatures decreasing (aging_DN) and increasing (aging_UP) with age in a previous study [34] indeed show a similar trend in our analysis.

Finally, we were interested in expression characteristics of the age groups describing their mean expression levels and variability (Figure 2g). The respective boxplots as a function of age show U-shaped courses with extremal values of relative (centralized) expression and of variance for teenagers. Such hourglass shapes suggest an overlap of two complementary processes related either to brain development, maturation, and learning in childhood at younger age on one hand and to functions related to maintenance of brain tasks, metabolism, and immunity in the aging brain on the other hand. The courses seem to characterize the transition between development and aging with a tipping point in the adolescent phase. They are previously reported for brain transcriptomics and morphological features [35]. Increased variance in late adults shows that increased expression heterogeneity is a characteristic of the aging human brain [36]. In summary, expression changes upon aging from babies to elderly adults decompose into different modes of monotonously increasing, decaying and U and inverse-U shaped courses referring to different functions thus reflecting genomic regulation via activation and de-activation of transcriptional programs in an age-dependent fashion.

### 3.2. The Methylome of the Aging Brain

**Figure 3:**
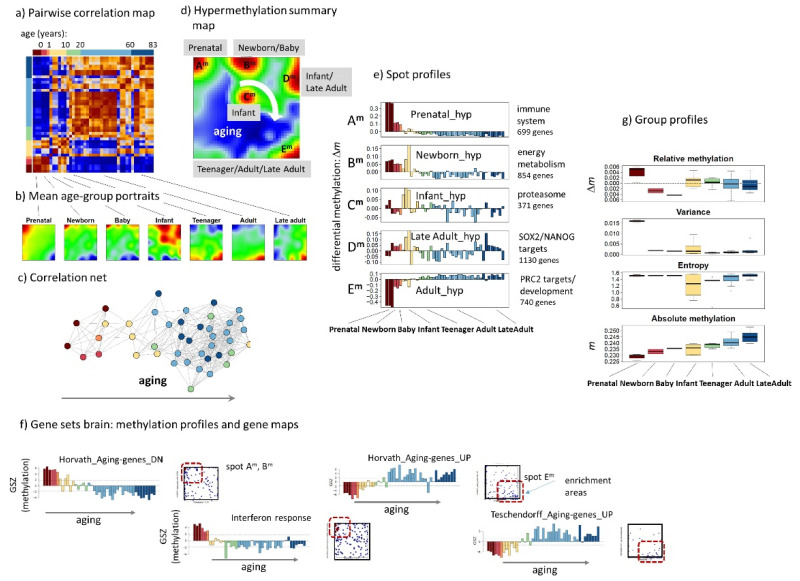
DNA methylation characteristics of the human brain (prefrontal cortex). (**a**) The pairwise correlation map indicates a virtually binary pattern of the aging brain in terms of two major clusters referring to prenatal and early childhood and ages from late childhood to late adults. (**b**) Mean SOM portraits averaged over intervals of increasing age. (**c**) The sample similarity net reveals a virtually one-dimensional development of promoter methylation in the brain samples. (**d**) “Spot” clusters of co-methylated genes are summarized in the overview map. Overall five hyper-methylation spots were detected (“A^m^”–“E^m^”). Upon aging hyper-methylation spots activate in a clockwise direction as indicated by the arrow. (**e**) The spot profiles reveal specific hyper- and hypo-methylation in different age intervals. Δm is the centralized log-ratio of methylated-versus-non-methylated CpGs in the gene promoters (Table A2). (**f**) Methylation profiles and gene maps of aging gene sets taken from [10,37,38] show decaying and increasing methylation levels as expected. The respective genes accumulate in map areas referring to spots A^m^ and E^m^. (**g**) Boxplots of different methylation measures as a function of the age strata. The absolute methylation level continuously increases with age. The color code for the aging groups is used throughout the paper.

SOM methylome portraits of 43 brain (prefrontal cortex) samples of age ranging from prenatal to late adult individuals were generated independently of the expression portraits, meaning that the genes are arranged differently in the E(xpression)- and M(ethylation)-SOM. SOM methylation portraits (Figure A1b) divide into two major clusters dominated by either prenatal and early childhood samples or by teenagers to late adult brains in the pairwise correlation map (Figure 3a). Methylation portraits (Figure 3b) group along the age axis in the sample similarity net from prenatal to late adults (Figure 3c). Five major spot-clusters of co-methylated gene promoters labelled A^m^ to E^m^ were detected. They reflect a series of methylation modes appearing upon aging in clockwise order as indicated in the summary map (Figure 3d, Table A2). The respective methylation profiles show either strong hyper- or hypomethylation in early lifetime, which associates with inflammatory and interferon related functions or with developmental functionalities of the included genes, respectively (Table A2). Previous methylation signatures of aging [10,37,38] agree with the methylation data analyzed here (Figure 3e,f). Overall, gene promotors accumulate methylation in CpG islands with age (Figure 3f) [39]. Taken together, we find strong alterations of methylation in the prenatal and early postnatal brains owing to differentiation of brain tissue followed by a “slow” methylation shift during the rest of the lifetime resembling “methylation clocks” of biological age [40].

### 3.3. Couplings between Expression and Methylation

**Figure 4:**
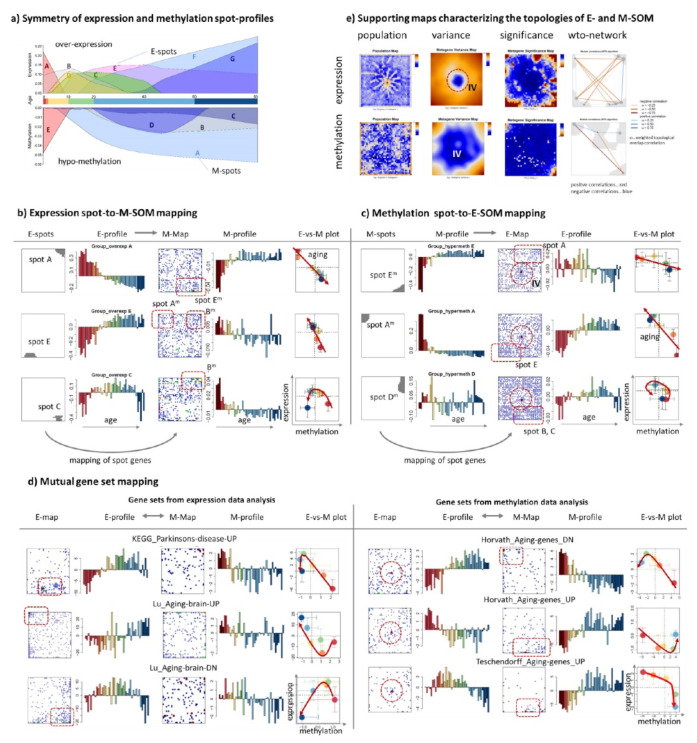
Couplings between expression and methylation upon development and aging. (**a**) Spot profiles of expression and methylation suggest antagonistic changes. Only overexpression (Δe > 0) and hypomethylation (Δm < 0) parts of the curves are shown. Mutual mapping of spot genes, (**b**) of E-spots into M-SOM and, (**c**) vice versa of M-spots into E-SOM reveal spot “melting” as indication of partial de-coupling between expression and methylation. The E- and M-profiles reveal antagonistic changes upon aging. (**d**) Mutual mapping of gene sets taken from previous expression (left part) and methylation (right part) studies. It reveals melting (increased disorder) upon mapping into the map not referring to the experimental origin of the data in similarity to results shown in part (**b**,**c**). The arrows in the correlation plots in part (**b**,**d**) point in direction of increasing age (the circles refer to mean values averaged over the age groups) and serve as guides for the eyes. (**e**) Supporting maps [26] visualize numbers of genes per metagene (population map), variance of metagene expression/methylation, their significance (*p*-values derived from *t*-statistics of metagene expression/methylation), and wto (weighted topological overlap) correlation coefficients between spots [42]. One finds a cluster of invariant genes (IV) in the middle of the E-SOM. The wto map reflects anticorrelated expression/methylation in opposite corners of the map.

**Figure A2:**
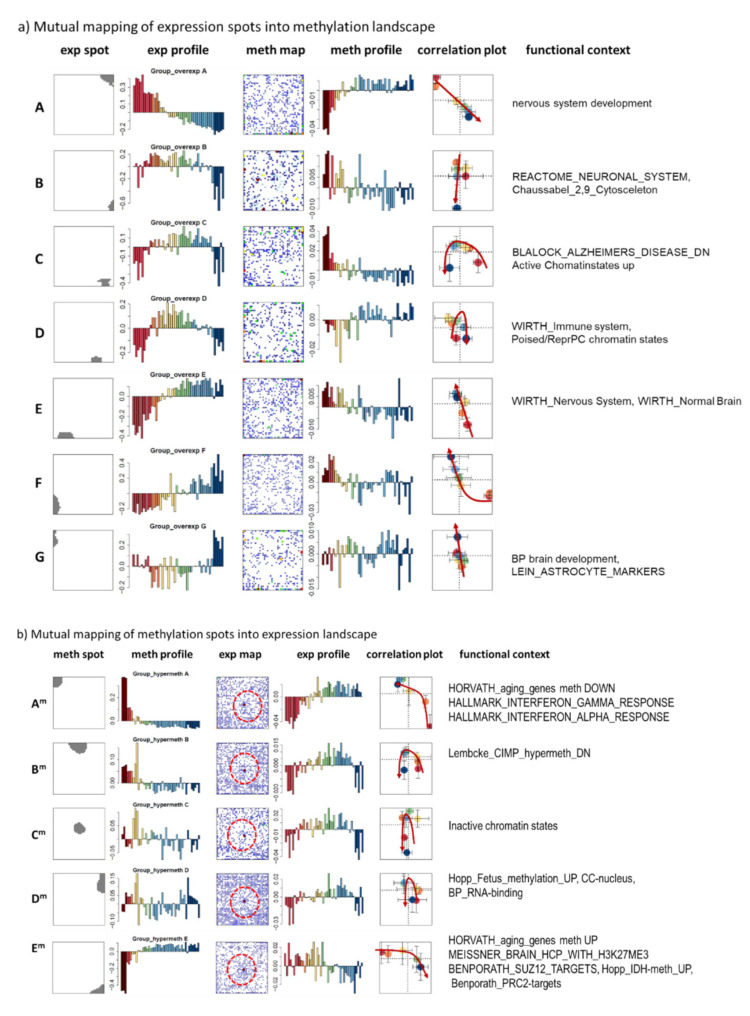
Mutual mapping of genes of the expression spot modules into methylation SOM (part **a**) and vice versa, of genes of the methylation spot modules into expression SOM (part **b**). Spots virtually “melt” in both directions reflecting the lack of one-to-one relationships between co-expressed and co-methylated spot modules, meaning that expression of part of genes is not or weakly affected by methylation and vice versa. In expression space part of co-methylated genes show virtually invariant expression and accumulate in the middle of the expression map (red circles in part b; see also Figure 3e). The functional context was estimated by means of gene set enrichment analysis of a collection of about 6000 gene sets taken from literature and implemented in oposSOM [116] using a threshold of *p* < 10^−5^ (Fisher’s exact test for enrichment in the respective spot genes). Age groups are colored as assigned in the main paper. The correlation plots show means values over the respective age groups as circles.

So far, we separately analyzed expression and methylation data, which generate two independent series clusters of genes with different time courses of mean expression and methylation levels, respectively. The comparison and joint plot of expression and methylation spot profiles reveals virtually mirror symmetrical courses especially for strongly variant spots at early childhood (spots A/E^m^ for expression/methylation, respectively; see Figure 4a) and for spots slowly varying upon aging (spots E, F, G/C^m^, B^m^, A^m^). Particularly, expression of genes in spot A associating with developmental functionalities (see below) strongly decays in the first decade of life while gene promoters in spot E^m^ gain their methylation in the same period of childhood. The inverse relation, i.e., increasing expression and loss of methylation applies to later lifetime, e.g., to spots F and A^m^. To better understand these changes on gene-level we selected genes from single E(xpression)-spots and generated their M(ethylation)-profiles together with gene maps, indicating positions of the E-spot genes in the M-map (Figure 4b; see Figure A2 for plots of all spots). Comparison of the E- and M-profiles of the same genes reveals overall antagonistic changes of expression and methylation, which is clearly supported by the negative slopes in E-versus-M correlation plots using age-group averaged values (Figure A2). High expression is found either at early or late ages for different spots (see red arrows in Figure 4b pointing in direction of aging). In some situations, E-versus-M relations are not linear and change sign and slope with age, e.g., showing markedly reduced variations of methylation at later ages on one hand, but still marked changes of expression which suggests overlay of different modes of genomic regulation such as transcription factor networks and epigenetic remodeling of chromatin (see below).

Interestingly, localized E-spot genes “melt” in the M-map, i.e., they distribute over large areas of the M-map. This “spot-melting” reveals partial decoupling of expression and methylation, meaning that only part of the expression of genes from the E-spots associates with anti-correlated alterations of methylation. The inverse mapping of M-spots into the E-map shows an analogous behavior, i.e., antagonistic changes of expression and methylation, spot melting, and accumulation of part of the genes in E-spot areas (Figure 4c). Similar results were obtained for gene sets either derived from transcriptomic or methylation data (Figure 4d). Set genes are more localized in E-spots if derived from expression data but “melt” in the M-map and vice versa for gene sets obtained from methylation data.

Overall, these results reflect the lack of a one-to-one relation between expression and methylation changes, as reported previously in cancer studies [20,21,22]. Additionally, aging studies reveal only weak correlation between DNA-methylation and transcriptome age predictors [41], suggesting that transcriptomics and epigenetics partly describe different aspects of biological aging. Interestingly, M-spot genes and methylation data derived gene sets often accumulate near the center of the map (see dashed circles in Figure 4c), referring typically to invariant genes (IV; see the variance and entropy maps in Figure 4e). The IV regions in the E-maps indicate that a considerable part of genes that change their promoter methylation upon aging do not alter their expression in parallel. In turn, invariant methylation of genes changing expression seems weaker as suggested by the more distributed IV region in the M-map. Hence, transcriptome and methylome alter in an asymmetrical fashion: Differential methylation is the more common phenomenon, which seems not to be paralleled by differential expression of part of the genes.

Taken together, the joint view on expression and promoter methylation revealed antagonistic changes over large age intervals, which suggests repression of expression by promoter methylation of many genes. Expression and methylation of another fraction of genes, however, clearly decouples or shows other than antagonistic relationships. Associations between expression and methylation obviously can change with age; i.e., it can be different at early, middle, and late ages.

### 3.4. Functional Context

**Figure 5:**
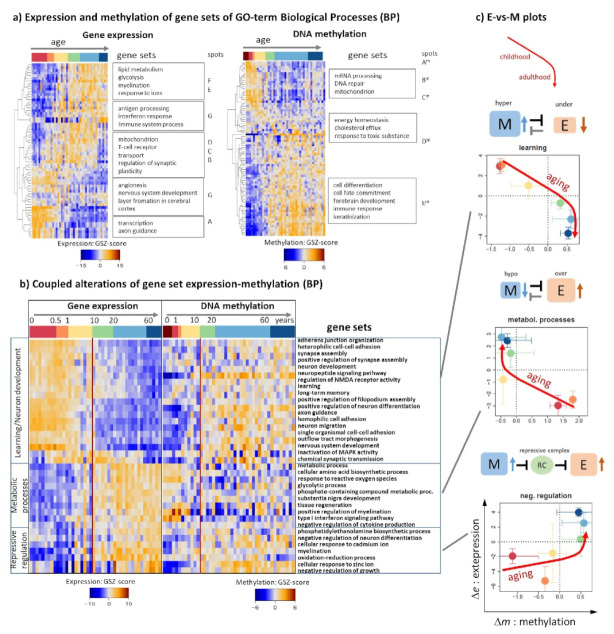
Gene function, expression, and methylation: (**a**) Gene expression and DNA promoter methylation of gene sets of the GO-category biological function (BP) provide an overview of functional changes upon aging. Expression and methylation were addressed independently. (**b**) Joint gene set analysis of gene expression and methylation as a function of age. Affected functions roughly divide into three major categories as indicated at the left axis of the heatmap. (**c**) Expression (E) versus methylation (M) plots of the three categories. Dots are mean values over all gene sets per category. Arrows serve as a guide for the eye and illustrate alterations upon aging. Anti-correlated relations between M and E suggest mutual repression while positive correlation can be rationalized in terms of repressive complexes (RC) which mediate interactions between M and E.

**Figure A3:**
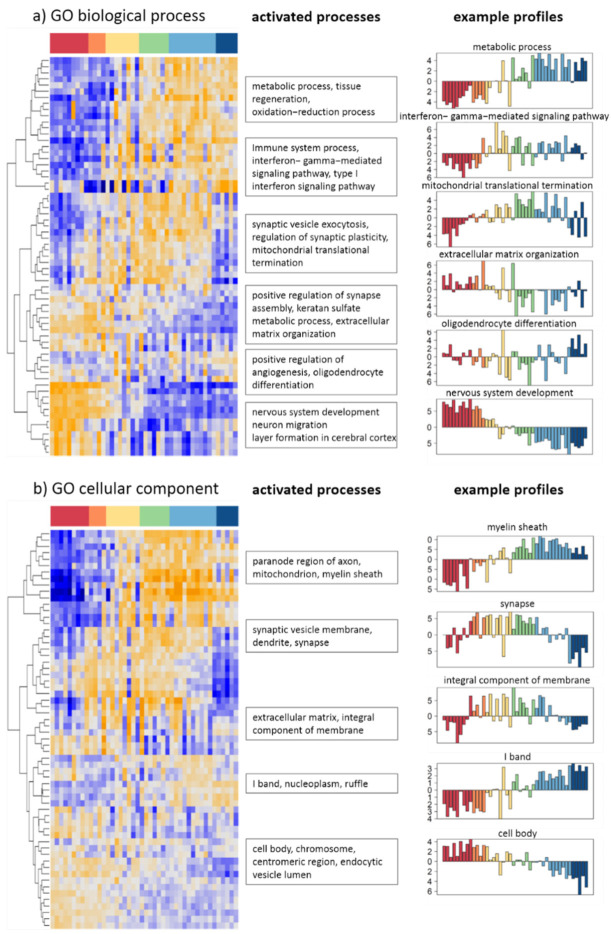
Heatmap of gene expression activity of gene sets taken from GO biological process (**a**) and GO cellular component (**b**). Selected aging profiles are shown as bar plots in the right part. Age groups are colored as assigned in the main paper.

Heatmaps of gene sets taken from the GO (gene ontology) biological process domain (BP, [43]) associate brain development and aging with underlying biology separately for expression and methylation data (Figure 5a, selected profiles and gene sets of GO cellular component are shown in Figure A3). Highly expressed cellular programs shift from “transcription” and “nervous development” (early childhood), via the “regulation of synaptic plasticity” and immune response (T-cells, interferon alpha/gamma response) towards lipid metabolism (late adults). Next, assuming deactivation of expression by promoter methylation as the basic effect, BP-sets were sorted with decreasing methylation upon aging (Figure 5a, right part). One finds promoter hypermethylation of genes involved in “cell fate commitment” and “differentiation” (early ages), then “energy homeostasis” and later “DNA repair” and “mitochondrion” at later ages. For a better match of functions, we generated a joint heatmap of expression and methylation levels of the same set in each of the rows (Figure 5b). The sets can be divided roughly into three groups: (i) Gene sets showing increasing methylation and decaying expression with age were subsumed as “learning”-related involving developmental functionalities. (ii) The inverse relation, e.g., decaying methylation and increasing expression is observed for metabolic and inflammatory processes. (iii) Virtually positive correlations between expression and methylation were subsumed as repressive regulation including “negative regulation of differentiation” and “responses to zinc and cadmium ions”. The E-versus-M plots reveal curved courses where methylation changes level off at later ages while expression still changes. In terms of simple interactions (i) and (ii) trivially can be considered as repression of expression by promoter methylation while (iii) can be rationalized by inserting an intermediate repressive complex which effectively de-represses expression (RC, Figure 5b, right part). The latter interaction is partly compatible with mutually exclusive DNA methylation and PRC2 -mediated repression since PRC2 binds to hypomethylated CpGs [44].

**Figure A4:**
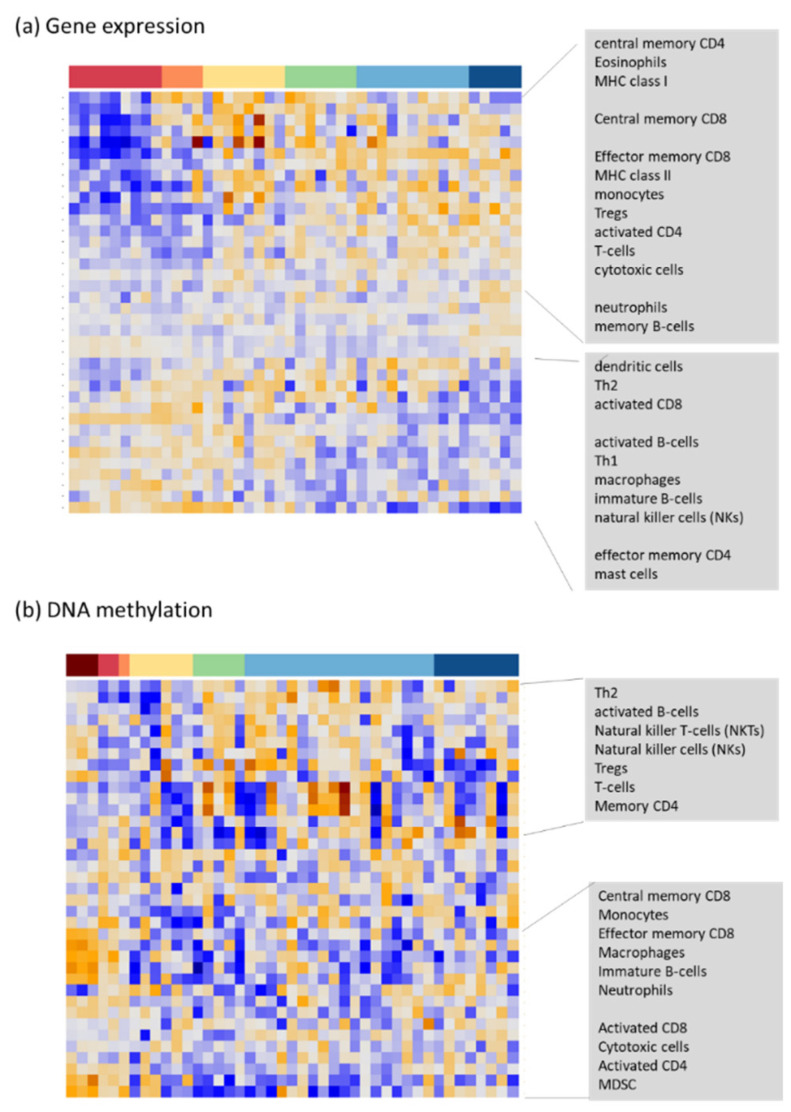
Immune cell and inflammatory characteristics of brain aging. Gene sets of immune cells were taken from [117]. Transcription signatures of mast cells, macrophages, natural killer cells and of activated CD8 cells activate in childhood and adolescent age while activated CD4, monocytes, Tregs, cytotoxic cells, neutrophils and T-cells show high expression in adults and elderly people. A series of signatures show high methylation level in the fetal brain (see grey list below).

The aging of healthy tissues is often accompanied by increasing inflammation (“immunoaging”) and senescence at the cell level [45]. A series of immunity-related signatures indeed activate with age, e.g., activated CD4 cells, regulatory T cells, memory CD8 cells, and MHC I and II related signatures (Figure A4a). Other immune-related signatures, such as mast cells, activated CD8 cells, immature and activated B cells, macrophages, natural killer cells, however, decay with age showing maximum expression in childhood and/or early adulthood this way reflecting complex alterations of the immune environment in the brain. Interestingly, the accompanying methylation data reveal hypermethylation of a series of signatures (e.g., diverse CD8 cells, macrophages; see Figure A4b) in the fetal and babies’ brains suggesting their transcriptional repression in the early development phase. Others, such as activated B cells and natural killer cells show decaying methylation in elderly people which associates with their transcriptional activation.

**Figure A5:**
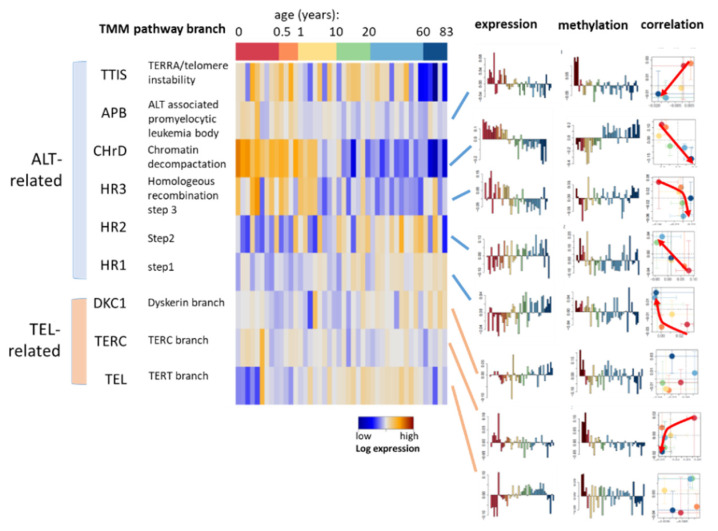
Telomerase (TEL) and alternative (ALT) telomere maintenance (TM) related pathway activities taken from [46]. The heatmap illustrates heterogeneous expression changes with age of genes taken from different branches of the TM pathways. Expression and methylation profiles of part of the processes and their bi-plot are shown in the right part of the figure. Expression of most ALT-related processes (except HR1 and HR2) decay with age while the TEL-related processes DKC1 and TEL increases. DNA virtually changes in an anticorrelated fashion.

Next, we considered gene expression signatures related to telomere length maintenance (TM) via telomerase (TEL) and alternative (ALT) mechanisms [46], which possibly associate with human aging of brain tissue and neurodegenerative diseases as indicated by human cell line experiments and animal models [47,48,49]. TEL-TM and particularly the TERT branch of this process gains in gene expression until adult age and afterwards decays while methylation decreases, thus suggesting increasing TM potential in adults and a decay for elderly people (Figure A5). Part of the ALT processes show the opposite trend, particularly, mechanisms leading to chromatin decompaction deactivate with age accompanied by anticorrelated methylation. Note, however, that these pathways also fulfill non-telomeric functions [46]. For example, TERT might be able to ameliorate the effects of toxic proteins such as amyloid-β, pathological tau, and α-synuclein involved in neurodegenerative disorders such as Alzheimer’s and Parkinson’s disease possibly by activation of autophagy [50].

**Figure A6:**
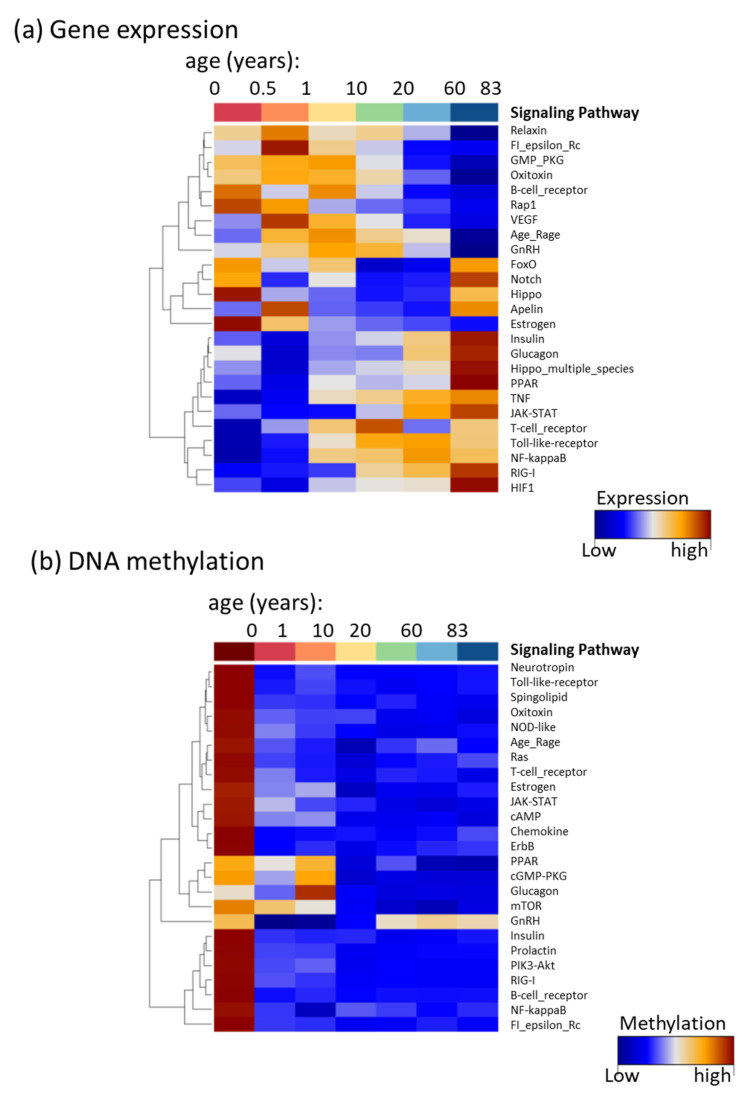
Pathway signal flow analysis of expression (**a**) and methylation (**b**). In total 51 KEGG-pathways were considered by means of pathway signal flow (PSF) analysis [51] using either gene expression (in a) or gene-promoter methylation (in b) values. The heatmaps show the top 25 pathways where the mean PSF-value averaged over all nodes of each pathway is shown. (**a**) According to expression activity pathways divide into three major clusters, activated in babies and children (e.g., oxitoxin, B-cell receptor), activated in adults and late adults (e.g., HIF1, Jak-Stat) or in both and deactivated in between (e.g., Foxo, Notch). (**b**) High methylation PSF values were found especially in prenatal brains for most pathways. Detailed pathway plots showing node activation for all age ranges were provided in Supplementary Material S3 (gene expression) and S4 (DNA-methylation).

Finally, we analyzed expression and methylation patterns of more than 50 KEGG-pathways using pathway signal flow values [51] (Figure A6 and Supplementary Figures S3 and S4). Pathways show high transcriptomic activity either in babies and children, adults and late adults, or both with low activity at intermediate ages. In contrast, high methylation dominates in prenatal brains associating with their deactivation upon fetal development. In summary, we find serial activation of (brain) developmental, learning, metabolic and inflammatory functions upon aging, which mostly associate with anticorrelated methylation and expression changes and therefore suggest that changing methylation governs transcriptional programs mainly by direct repression or mediated via intermediate steps. Gene sets related to immune response and telomere maintenance show a complex behavior but overall seem to support immuno-aging and TM destabilization.

### 3.5. Methylome Modifying Enzymes Govern Gene Activity and Methylation

**Figure 6:**
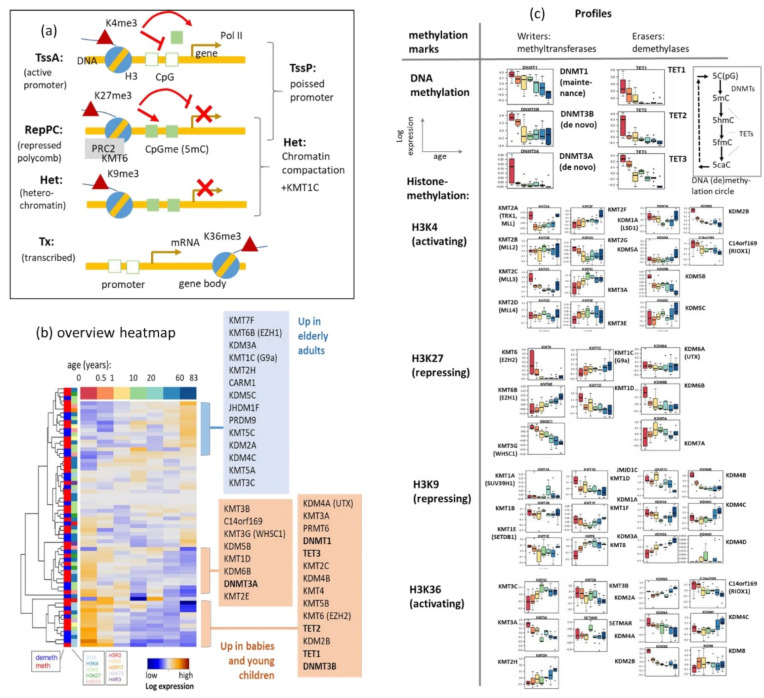
Aging profiles of gene expression of a series of enzymes acting as writers or erasers of methylation marks at DNA and histone side chains. (**a**) Schematic sketch of the joint effect of histone lysine side chain and DNA methylation on the transcription of downstream genes and their relation to different chromatin states. (**b**) The overview heatmap shows that most of the genes, among them the DNA methyltransferases DNMT1 and DNMT3A,B and the demethylases Tet1–Tet3 (acting along the cytosine oxidation cycle) show large expression in babies and young children, which then decays with age, while another, smaller group gains expression upon aging, among them the H3K27me3 writing and chromatin compacting enzyme G9a (KMT1c). (**c**) Boxplots of the aging profiles of part of the enzymes reveal a variety of courses either increasing or decreasing with age. The different marks can have activating (e.g., triple methylation of lysine side chain H3K4 or H3K36), repressing (e.g., triple methylation at H3K27 or at H3K9) or poising (combination of H3K4 and H3K27) and chromatin compacting (H3K27 and H3K9) effect on transcription of the associated gene as schematically illustrated in part c. Combination of H3K4me3 and H3K27me3 gives rise to poised (“ready to go” and/plastic state) transcription while combination of H3K27me3 and H3K9me3 leads to compacted chromatin. For a detailed description of the enzymes and the histone code, see [24,68,69,70,71] and [66,67,72] for mechanisms in brain development. Maps of the different enzyme classes in the expression landscape of the brain are shown in Figure A7. DNA cytosine (CpG) methylation and de-methylation oxidation cycle are shown in part b.

Dynamic chromatin regulation is a fundamental determinant of the developing and aging neural network. Chromatin remodelers are critical determinants of this process with impact on health and disease [52]. Particularly, methylome modifying enzymes are an essential ingredient of the epigenomic machinery. They modulate the methylation of DNA and of a series of proteins affecting gene activity. For example, methylation of lysine (symbol K) side chains of the histone subunit 3 (symbol H3) at positions 4, 9, 27, or 36 (symbols H3K4, H3K9, etc.) affect the chromatin state, gene expression and, in turn, also DNA methylation mainly in the promoter region of the respective genes (see below and Figure 6a for a schematic overview). Methylation is regulated via methylating and de-methylating enzymes writing or erasing the methylation marks to CpG islands, respectively [53,54]. Gene expression of a series of such enzymes shows pronounced changes either decaying or gaining with age (Figure 6b; see the two clusters marked in the heatmap). Expression of the DNA methylation maintenance methyltransferases Dnmt1 continuously decays with age on a logarithmic scale, while expression of the de novo methyltransferases Dnmt3a and Dnmt3b is high in babies and then sharply drops, suggesting that de novo methylation along the DNA associates with brain development at early age while methylation maintenance acts during whole lifetime (Figure 6c). Indeed, previous studies suggest an intimate link between synaptic events and nuclear Dnmt activity [55]. However, it is still unclear how neuronal activity regulates dynamic and reversible DNA methylation and how this can be influenced by Dnmts.

A similar pattern as for methyltransferases is observed for DNA demethylases Tet1 –Tet3, where Tet3 shows the more continuous decay than Tet1 and 2. Note that demethylation proceeds via a series of oxidation steps as sketched in Figure 6c right above. Tet activity has a stronger effect on the demethylation rate than Dnmt1 infidelity [56]. On the other hand, one cannot distinguish whether the effect of Tet activity on demethylation rate is due to bona fide active demethylation (base excision), or incomplete maintenance of oxidized methyl groups. Both, DNA methylases and demethylases are shown to play specific roles upon neuronal differentiation [7,57,58,59,60,61]. The decreasing transcriptional activity of maintenance methyltransferase during lifetime possibly associates with passive DNA-hypomethylation owing to the decaying restoration of methylation after cell division. Dnmt1 expression in post-mitotic neurons might serve to maintain DNA methylation after base-excision repair and it is required for maintaining DNA methylation [5]. On the other hand, it seems that decaying Dnmt1 activity adjusts to decreasing cell division rates and stemness-activity in the brain with age (Figure A11). The virtually constant methylation levels of stemness genes during adulthood support this view. The parallel decay of gene expression of writers and erasers of DNA methylation presumably reflects a coupled equilibrium of methylating and de-methylating activity where, e.g., Dnmt1 antagonizes DNA demethylation and Tet3 antagonized hypermethylation, which, in turn, varies during lifetime. Indeed, a recent study on genome-scale methylation kinetics revealed highly variable and context-specific activity for the DNA methylation machinery [56].

**Figure A7:**
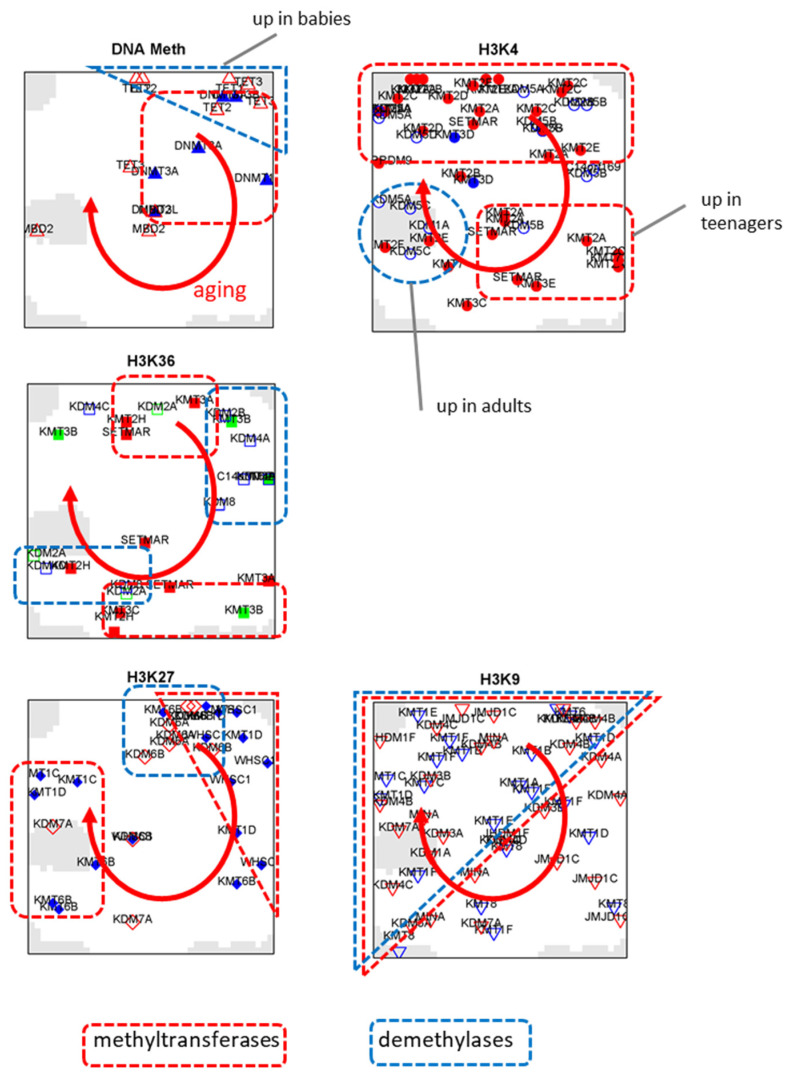
Mapping of epigenetic modifiers into gene expression SOM (see also Figure 5b). Methyltransferases (red frames) and demethylases (blue frames) for part of modifications accumulate in different regions of the map referring to their activation in different phases of lifetime. Particularly, H3K4me3 writers show often high expression in early and intermediate phases of lifetime possibly reflecting transcriptional activation of the associated genes. For writers of the repressive H3K27me3 marks this relation reverses, i.e., writers activate with delay to erasers. A similar but smaller delay is observed for writers of DNA methylation, which also associate with repression of gene expression. Hence, modifications activating transcription seem to precede repressive modifications for H3K4, H3K27, DNA and, to a lesser degree, H3K36. Note that SOM mapping of the respective genes reveals these subtle shifts not immediately evident from the aging profiles (Figure 5b,c).

Tri-methylation of lysine side chains of histone H3 at different positions can either activate (H3K4me3, H3K36me3) or repress (H3K9me3, H3K27me3) transcription according to the histone code (see Figure 6a and [31,62]). It further associates with different chromatin states such as active or repressed promoters (TssA, RepPC), heterochromatin (Het), and transcribed genes (Tx). The methylation of the K-side chains is regulated by a battery of lysine methyltransferases (symbol KTM) and demethylases (symbol KDM) showing overall very diverse aging profiles of their gene expression (Figure 6c). Typical courses reflect continuous gains (e.g., KMT6B alias EZH1) or decays (e.g., KMT3G alias WHSC1) or marked changes at early developmental stages (e.g., KMT6 alias EZH2 and KMT3C) or at later stages of aging (e.g., KDM5B and KDM5C). The detailed regulatory tasks of these enzymes are overall complex and beyond the scope of this publication (see, e.g., [24,63,64,65]). They comprise mono-, bi- and trimethylation steps, coupled functions affecting lysines at different positions, e.g., at K9 and K27, or along the gene, e.g., in the promoter or gene body regions. For example, modifiers of H3K4 methylation are needed for memory formation, shown through animal studies, and many of the same modifiers are mutated in human cognitive diseases and have been associated with impaired cognition in neurologic and psychiatric disorders [66]. Methylation of histone H3 on lysine 27 (H3K27me3) is frequently found in the heterochromatin and conceives a repressive marker that is linked with gene silencing [67]. H3K27me3 and H3K9me3 associate with PRC2-repression and constitutive heterochromatin, respectively. Both contribute to stable gene repression through interactions with protein complexes involved in chromatin compaction. Mapping of the genes encoding the enzymes into the expression SOM, comparison of their positions with the different spots and their aging profiles suggests that modifications associating with transcriptional activation (i.e., methylation of H3K4 and H3K36 and demethylation of H3K27 and DNA) slightly precede modifications associating with transcriptional repression along the lifetime scale (Figure A7). In summary, expression of genes encoding methylome modifying enzymes is very diverse, reflecting complex regulations over one’s lifetime. A larger part of them shows maximum expression in babies and children reflecting a governing role in brain development. Activating modifications seem to precede deactivating ones along the lifetime scale. Another part of the enzymes is activated in later phases of lifetime playing a role in aging.

### 3.6. Remodeling of Chromatin States

**Figure 7:**
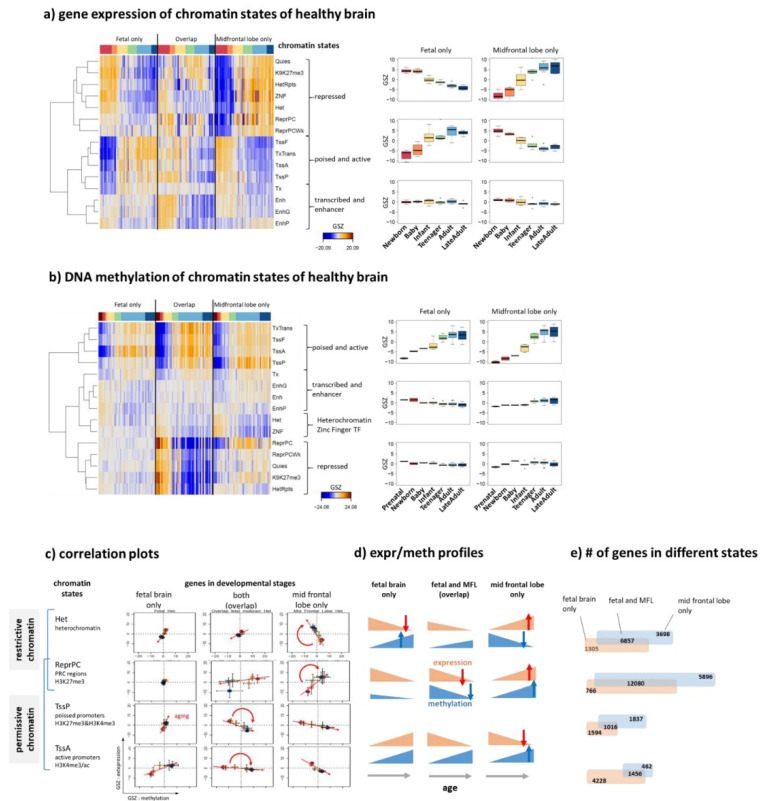
Gene expression (**a**) and DNA methylation (**b**) of chromatin states in the fetal and adult brain. “Overlap” genes do not change their state between fetal and adult brain. Chromatin states refer to active promoters (TssA), transcribed genes (Tx), weak active enhancer and enhancer-like states (Enh, EnhG, EnhP), zinc finger proteins (ZNF), quiescent (Quies), heterochromatin (Het), heterochromatin repeats (HetRpts), poised promoters (TssP), repressed polycomb states (RepPC, weak: RepPCWk) and were taken from [30]. The profiles can be divided into three major groups, referring to repressed, active/poised genes and heterochromatin/enhancers. The boxplots in the right part of (**a**,**b**) show profiles of selected groups of chromatin states across the age strata in GSZ (gene set Z-) score. (**c**) Correlation plots between expression and methylation for selected chromatin states (columns) and brain developmental stages (rows) indicate changing slopes and directions of the trajectories. (**d**) Schematic illustration of the expression (red) and methylation (blue) profiles across age. The red arrows indicate expected (nominal) expression in the respective states, e.g., low expression in repressed and high expression in transcribed states. (**e**) Number of genes in the different states. The largest fraction of genes in the fetal brain only refer to active states (TssA) while genes in adult brain (only and overlap) are mostly found in the other states.

Neuronal specification is driven by transcriptional programs, which are regulated by an interplay of epigenetics and transcription factor networks [73,74]. It is mediated by chromatin organization [75,76], which can be characterized by a series of chromatin states defined via combinations of histone modification at the affected nucleosomes [30,77]. For a more detailed view on epigenetic regulation, we analyzed expression and methylation levels of genes assigned to these different chromatin states referring to either the developing fetal or aging adult (midfrontal lobe) human brain only, or to both (overlap genes), which were taken from previous studies on a genome-wide scale [30,77]. Clustering of the expression and DNA-methylation profiles of the genes taken from the different states roughly provided three major groups including genes with (i) active (TssA) and poised (TssP) promoters, (ii) repressed (RepPC) promoters, and (iii) transcribed genes (Tx) and enhancer (Enh) states (Figure 7a,b). The genes of groups (i) and (ii) switch their expression levels from high to low upon aging, between fetal and adult brain states and between the two groups, respectively (Figure 7a). This means that, e.g., repressed states in the fetal brain show similar aging profiles as active and poised states in the adult brain and vice versa, and that expression of group (ii) in the adult brain resembles expression of group (i) in the fetal brain. In other words, expression of the genes from active and repressive states switch antagonistically between the developing fetal brain and the aging adult brain. These expression changes suggest the remodeling of chromatin states between the developing and aging brain in early and later lifetimes, respectively.

In contrast to gene expression, DNA methylation of active and poised promoter states (group (i)) gains with age for all developmental groups (fetal only, overlap, and adult brain only, Figure 7b). Contrarily, methylation of overlap genes with repressed promoters (group (ii)) decays with age. A similar trend of hypomethylation was found for heterochromatin (Het state). The methylation changes of other states are less pronounced. The gene expression versus methylation correlation plots result in positive and negative slopes for repressive and active/poised states, respectively, which in turn can vary between the developmental stages (Figure 7c,d for a schematic summary). Polycomb group proteins generally mediate repression of gene expression via RepPC states. However, recent studies have revealed that PRC2 may contribute to gene activation via the establishment of PRC1-mediated enhancer–promoter interactions during brain development ([76] and references cited therein).

The gene numbers in repressive promoter states and heterochromatin are relatively low in fetal brain only, but much larger in the overlap and adult brain which reflects the fact that a large number of developmental and stemness genes become switched off in differentiated brain tissue (Figure 7d). This relation reverses for active promoter states where the majority of genes are found in the fetal brain where development proceeds mainly under control of transcription factors regulating networks of active genes. Genes with poised promoters distribute roughly equally over fetal only, overlap and adult brain data suggesting that this promoter state plays a functional role during all stages of brain development and aging. Poised promoters are characterized by bivalent marks, the simultaneous presence of H3K4me3 and H3K27me3, i.e., histone modifications associated with transcriptionally active and repressed chromatin, respectively. Bivalency is postulated to poise lineage-controlling of developmental genes for rapid activation during embryogenesis while maintaining a transcriptionally repressed state in the absence of activation cues. Recent studies show that bivalent chromatin however does not poise genes for rapid activation in the first instance but protects promoters from de novo DNA methylation [73]. This way bivalency represents a distinct regulatory mechanism for maintaining epigenetic plasticity by protecting reversibly repressed genes from irreversible silencing via DNA methylation. The similar gene expression and methylation profiles of poised/bivalent and active promoter states reflect a closely related functional impact where TssP states presumably form a reservoir of regulatory modes poised for reprogramming tasks of the cells.

Chromatin and associated epigenetic mechanisms stabilize gene expression and cellular states while also facilitating appropriate responses to environmental and developmental cues upon aging. They can induce restrictive or permissive epigenetic landscapes, where the former ones are formed by condensed chromatin states blocking differentiation [78] (Figure 7c–e). By contrast, permissive or “plastic” chromatin allows cell fate transitions. Upon aging restrictive chromatin in terms of heterochromatin states hypomethylates while permissive chromatin (TssA and TssP states) hypermethylate accompanied by anticorrelated expression changes thus suggesting less pronounced definition of the underlying cellular programs. Indeed, in young and healthy individuals, neural cells exhibit intact heterochromatin while aged cells show loss of heterochromatin as indicated by its hypomethylation accompanied by DNA damage accumulation, and the expression of aberrant transcripts [72]. The methylation of repressive states (RepPC) changes in both directions for overlap (hypomethylation) and adult brain only (hypermethylation) states which suggests their assignment to restrictive and permissive chromatin due to excess and suppressed activity, respectively [78]. In summary, the aging profiles of gene expression and DNA methylation of genes from different chromatin states reflect their remodeling between developing and aging phases of lifetime which includes repressive, active, and poised plasticity retaining states overall playing permissive and restrictive roles for the underlying cellular programs. Chromatin state remodeling contributes to the emerging patterns of cell fate specification leading to celltype diversity during brain development in early childhood indicating that neuronal plasticity depends on the availability of the DNA modification machinery [75].

### 3.7. Relations between the Aging Brain and Glioma Heterogeneity

**Figure 8:**
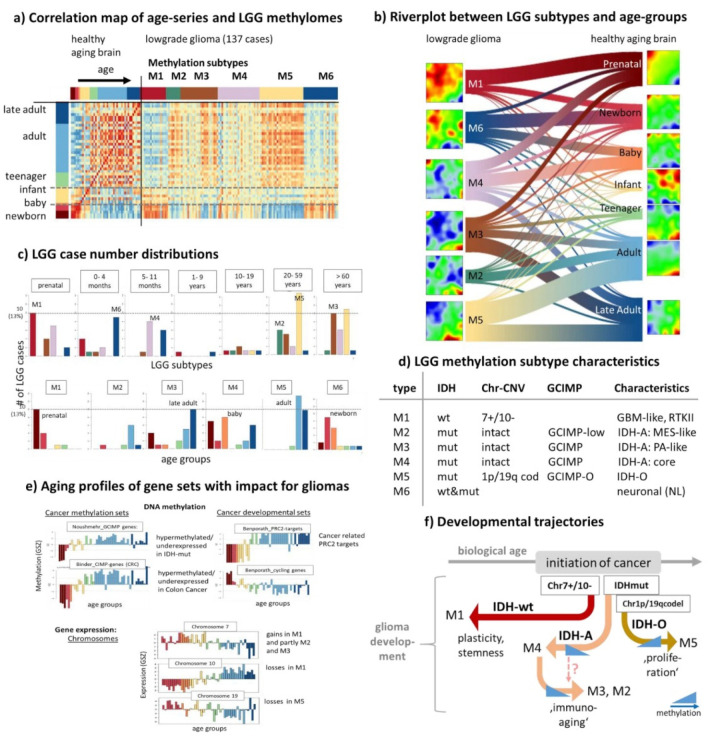
Comparison of the aging methylomes of healthy brain and that of lower-grade glioma (LGG). (**a**) The pairwise correlation heatmap between the samples indicates a strong correlation between different LGG-methylation and aging groups (red), e.g., between M1 (*IDH*-wt group) and the prenatal brain. The LGG methylation data were trained together with the healthy brain methylomes using exSOM transfer learning [32]. (**b**) The river plot visualizes the distribution of LGG tumor methylomes of the different M-groups among the age groups. The SOM portraits of the LGG were obtained using exSOM method applied to the aging data. (**c**) Distribution of samples of the M-groups among the age groups (part above) and vice versa, of the age groups among the LGG-groups (part below). (**d**) Characteristics of the M(ethylation) subtypes regarding *IDH* mutation status, chromosomal aberrations (7+/10−: gains at chromosome 7 and losses at chromosome 10, 1p/19q cod: codeletion at chromosomes 1p and 19q), GCIMP type and characteristics (GBM: glioblastoma, MES: mesenchymal, PA: pilocytic astrozytoma, IDH-A: *IDH* mutated astrocytoma, IDH-O: *IDH* mutated oligodendroglioma). (**e**) Aging profiles of DNA methylation of genes hypermethylated in the GCIMP [81] and CIMP colon cancer [91], of PRC2 targets and cycling genes [92], and of the integral expression of genes at chromosomes 7, 10, and 19 (for further glioma related signatures see Figure A10). (**f**) Schematic summary: Cancer is initiated by genetic lesions usually in later adulthood (>45 years). Developmental trajectories point towards induction of stemness-like cancer cells (M1 and partly M4), subsequent immuno-aging (M2, M3), or metabo-aging (M5) which resemble different ages of the healthy brain. Development of the *IDH*-mut gliomas is shaped by altered DNA methylation.

**Figure A8:**
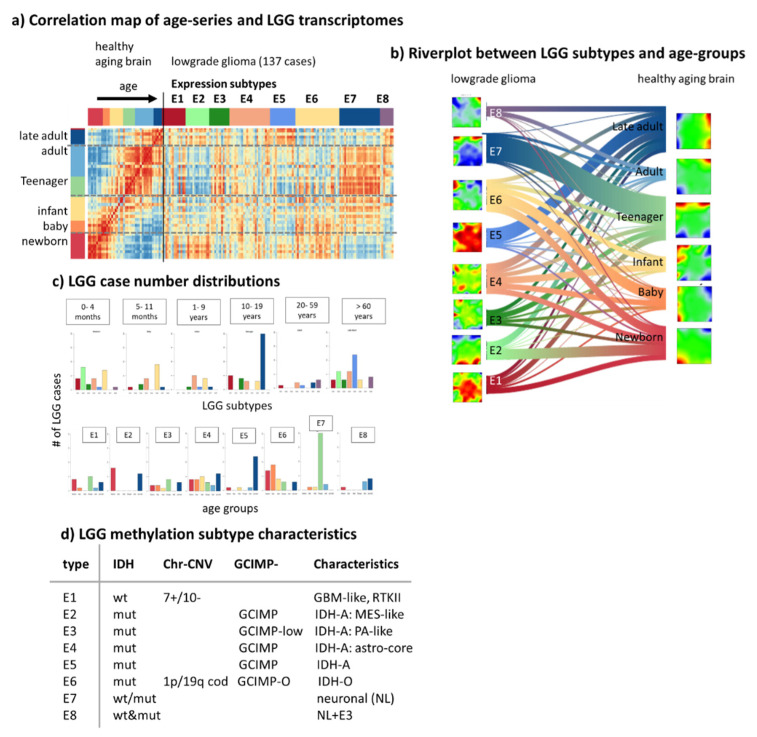
Comparison of the aging transcriptome of healthy brain and that of lower-grade glioma (LGG). (**a**) The pairwise correlation heatmap between the samples indicates correlation between different LGG-methylation and aging groups (red), e.g., between E1, E2 and E6 and the newborn brain. (**b**) The river plot visualizes distribution of LGG tumor methylomes of the different E-groups among the age groups. The SOM portraits of the LGG were obtained using exSOM method applied to the aging data. (**c**) Distribution of samples of the E-groups among the age groups (part above) and vice versa, of the age groups among the LGG-groups (part below). (**d**) Characteristics of the E(xpression) subtypes regarding *IDH* mutation status, chromosomal aberrations (7+/10−gains at chromosome 7 and losses at chromosome 10, 1p/19q cod codeletion at chromosomes 1p and 19q). Comparison of the aging methylomes of healthy brain and that of lower-grade glioma (LGG). The pairwise correlation heatmap between the samples indicates strong correlation between different LGG-methylation and aging groups (red), e.g., between M1 (*IDH*-wt group) and the prenatal brain. The LGG methylation data were trained together with the healthy brain methylomes using exSOM transfer learning [32]. The river plot visualizes distribution of LGG tumor methylomes of the different M-groups among the age groups. The SOM portraits of the LGG were obtained using exSOM method applied to the aging data. Distribution of samples of the M-groups among the age groups (part above) and vice versa, of the age groups among the LGG-groups (part below). Characteristics of the M(ethylation) subtypes regarding *IDH* mutation status, chromosomal aberrations (7+/10−gains at chromosome 7 and losses at chromosome 10, 1p/19q cod codeletion at chromosomes 1p and 19q), GCIMP type and characteristics (GBM glioblastoma, MES mesenchymal, PA pilocytic astrocytoma, IDH-A *IDH* mutated astrocytoma, IDH-O *IDH* mutated oligodendroglioma). Aging profiles of DNA-methylation of genes hypermethylated in the GCIMP [81] and CIMP colon cancer [91], of *PRC2* targets and cycling genes [92] and of the integral expression of genes at chromosomes 7, 10 and 19.

Aberrant DNA methylation, redistribution of histone marks, and disruption of chromatin structure in brain cancer, and particularly gliomas, are among the best described epigenetic changes underlying human pathology [22,29,79,80,81,82,83,84,85]. Hereby, cancer cells capitalize on cellular plasticity to acquire developmental programs that endow on self-renewal capacity with parallels to brain development. A particular subgroup of gliomas carrying mutation(s) in the *IDH1/2* gene (*IDH*-mut) express the CpG island methylation phenotype (GCIMP), which is characterized by large-scale hypermethylation patterns mainly due to the oncometabolic repression of demethylating enzymes such as the Tet-type DNA-methylases but also selected histone modifiers. The aberrant methylation of gliomas seems to resemble, at least partly, the age-related methylation patterns of the healthy brain [20,29]. Lower-grade diffuse gliomas (LGG) are mainly *IDH*-mut tumors (about 85%), which makes them suited for comparing age and cancer-related effects. We here compare the whole genome methylation and expression patterns of the healthy brain with that of LGG taken from our previous studies using the classification of LGG into six methylation subtypes M1–M6 of different characteristics including glioblastoma (GBM)-like *IDH*-wt (M1), *IDH*-mut astrocytoma-like (IDH-A, M2–M4) and oligodendroglioma-like (IDH-O, M5) as well as neuronal (NL, M6) tumors (Figure 8; for the gene expression analysis see Figure A8) [20,21].

Comparison of the methylation landscapes by means of the pairwise correlation map, river flow plot, and number distributions reveals a clear shift with age from prenatal and newborns brain with similarity to M1 (*IDH*-wt), of babies and young children resembling partly astrocytoma-like IDH-A of the M4 group (highly methylated IDH-A core group), of adults with similarity to GCIMP-low IDH-A LGG of the M2–M3 groups showing inflammatory characteristics of the tumor microenvironment and of elderly people with IDH-O LGG possessing relatively high proliferative characteristics (M5) (Figure 8a–d). The NL subtype M6 is partly dominated by *IDH*-wt properties what explains its similarity with M1 methylation patterns. The observed similarity can be mainly attributed to the age profile of specific GCIMP genes differentially methylated between *IDH*-mut and *IDH*-wt gliomas [81] which resembles the aging profile of PRC2-targets weakly methylated in early developmental stages of the brain (Figure 8e). Recent studies show that PRC2-targets associate with open chromatin states as well as with hypomethylation which is a key determinant of glioma stem cells ([86] and references cited therein). This epigenetic encoding of glioma supports the parallels between glioma differentiation and physiological neurodevelopment where stemness is also marked by PRC2 target hypomethylation. This maintains PRC2-targets in a hypomethylated, glioma stem-like state. Interestingly, also genes found hypermethylated in colon cancer (CRC CIMP genes) show a similar profile as GCIMP genes, thus reflecting cross-cancer similarities between gliomas and CRC and aging as well.

Cycling and inflammatory genes show an antagonistic profile of decaying methylation with age (Figure 3, Figure 5a and Figure 8d), which anti-correlates with the respective expression profiles (Figure 5b). In consequence, the inflammatory IDH-A types M2 and M3 and the proliferating IDH-O type M5 (see [20]) resemble the aging stage of the adult and later adult brain, respectively. Note also that these results agree with a recent view that glioma progression along the M4-M2-M3 axis of immunogenic subtypes shows certain similarity with immunoaging and senescence at the cell level [45] driven by hypomethylation of associated genes and particularly of the olfactory subgenome involving G-protein coupled receptors (*GPCR*; see below) [20,21]. Recent single-cell transcriptomics studies [87] showed that *IDH*-mut gliomas are characterized by a developmental hierarchy from neuronal progenitors (NPC)-like cells towards two separate IDH-O and IDH-A cancer cell branches which corresponds to the increased age of healthy brain upon comparison with the tumors (Figure 8f). In contrast, *IDH*-wt are assumed to refer more to neurodevelopmental NPC-like plastic states resembling more the young healthy brain as observed. Note that this correspondence between glioma subtypes and the aging healthy brain refers to developmental stages of the underlying cellular programs and not to the age of incidence of the tumors, which appears in a different order, namely of largest age for *IDH*-wt gliomas (median age of incidence above 50 years) followed by IDH-O (about 45 years) and IDH-A observed in youngest patients (about 40 years) [21,88].

**Figure A9:**
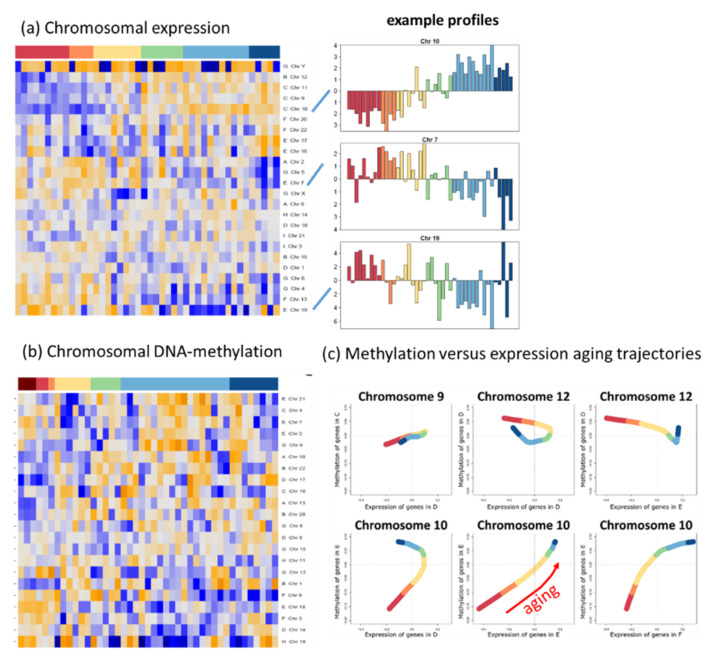
Chromosomal gene expression and methylation: (**a**) Chromosomal expression partly show pronounced age courses, particularly of Chr. 10, Chr. 7 and Chr. 19. Aberrant copy numbers of these chromosomes govern gliomagenesis into different subtypes (see main text). (**b**) Changes in chromosomal DNA methylation are much less pronounced. (**c**) Methylation versus expression trajectories (see [118] for method) reveal turns into different directions at a “breakpoint” in the adolescent–early adult age range. The different trajectories of Chr. 10 refer to genes located in spots D, E and F. The show similar courses in childhood but different courses mainly due to different expression changes in adulthood.

The major glioma subtypes show key-chromosomal defects, namely gains at Chr. 7 and losses at Chr. 10 for GBM-like *IDH*-wt gliomas and co-deletions at Chr. 1p/19q for IDH-O tumors resulting in dose–response relations of genes located at these loci [20]. Interestingly, aging profiles of genes from these chromosomes of the healthy brain show overexpression for Chr.7 and underexpression for Chr.10 in babies and adolescent thus resembling the Chr.7+/Chr.10− dose responses of *IDH*-wt (M1) gliomas, while the aging profile of Chr.19 shows underexpression in elderly thus resembling the Chr.19− dose effect in IDH-O (M5) gliomas (Figure 8e and Figure A9). In other words, the correspondence between age-related characteristics of the healthy brain and LGG is also reflected in age-dependent chromosomal expression of healthy brain thus resembling the dose effects due to aberrant copy numbers in gliomas. The analogous similarity analysis between the aging brain and glioma subtypes using gene expression instead of DNA methylation delivers no clear correspondence between aging intervals and LGG subtypes (Figure A8). This result does not surprise on one hand because active cellular programs in neoplastic brain tissue differ substantially from that in the healthy brain. On the other hand, the observed correspondence of DNA-methylation patterns confirms the fact that cell-of-origin DNA methylation signatures are largely maintained during carcinogenesis [86,89,90]. In summary, comparison of aging brain with LGG supports the view that GBM-like *IDH*-wt LGG exhibit higher cellular plasticity activated in the developing healthy brain while *IDH*-mut LGG have a more stable differentiation hierarchy more resembling the aged brain (Figure 8f).

### 3.8. Association with Brain Disorders

**Figure 9:**
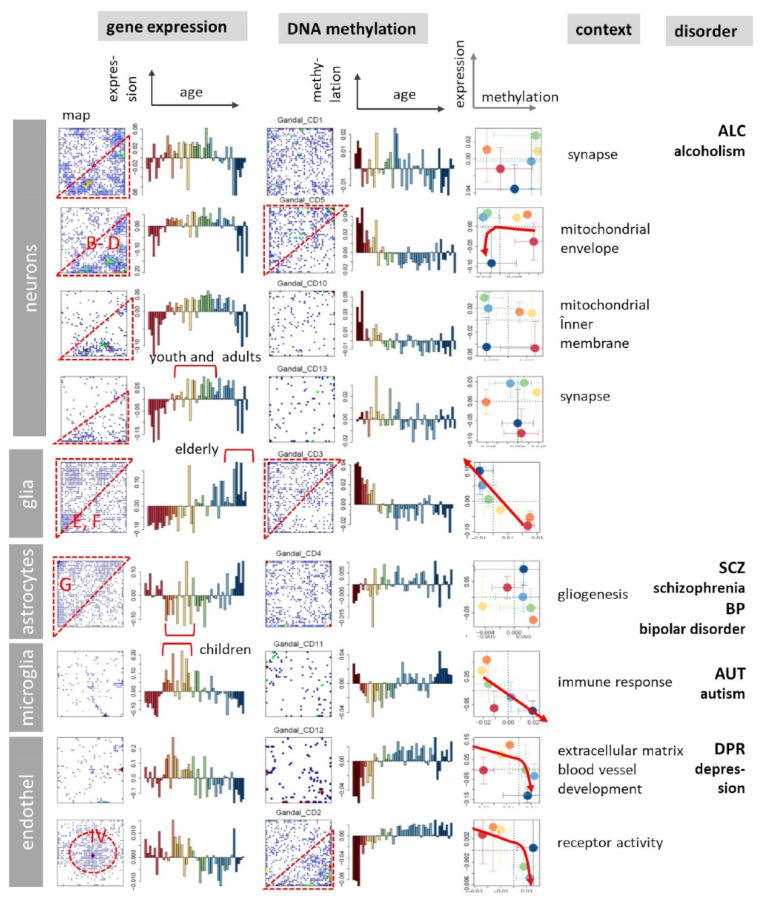
Comparison of expression and methylation effects of gene sets characterizing psychotic disorders extracted from [16] using gene maps (distributions of set genes in the E- and M-SOMs) and expression and methylation profiles and their functional and cellular context. The sets were obtained from transcriptomic studies of autism, schizophrenia, bipolar disorder, depression, alcoholism, and matched controls (see [16] for details). The red frames in the maps indicate regions of increased gene density. The arrows point in direction of increasing age and serve as a guide for the eye. For age-related color code see Figure 2a.

**Figure A11:**
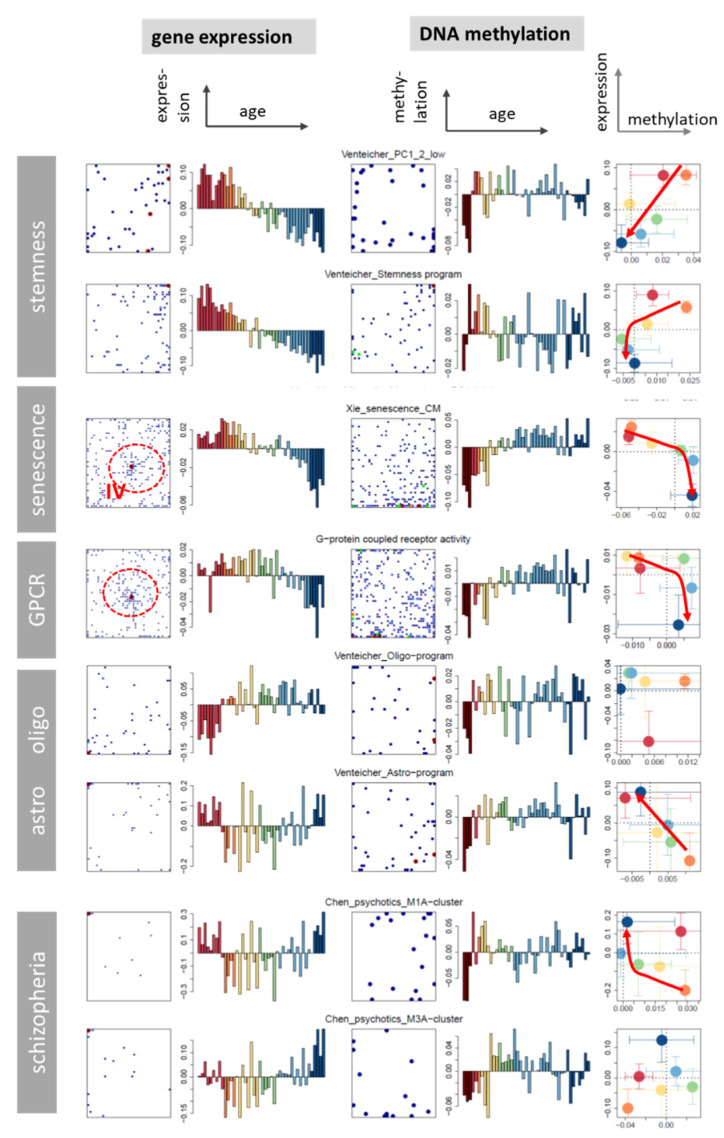
Maps and profiles of brain-related gene sets taken from [93,94,119]. Stemness sets were taken from single cell transcriptomics [93]. They resemble courses of *PRC2*-repressed genes that govern stemness properties. Senescence genes were selected by hypermethylation criteria and virtually anticorrelate with expression [94]. Senescence genes and genes encoding *GPCR* accumulate in the IV-area of the expression map thus reflecting invariant gene expression upon aging. Single cell profiles of astrocytes and oligodendrocytes show mirror symmetric expression profiles with a minimum/maximum for children and adolescents. Both cell types seem to hypermethylate with age. Schizophrenia related sets taken from [119] resemble that of astrocytes.

Next, we ask about relations between aging brain characteristics and psychiatric disorders. We made use of gene signatures taken from a comparative study of autism (AUT), schizophrenia (SCZ), bipolar disorder (BPD), major depression (DPR), and alcoholism (ALC) [16], and analyzed their gene maps and aging profiles in the expression and methylation domains of the aging brain (Figure 9). Most expression profiles show U- or inverse U-shaped profiles with maximum/minimum values in the ranges between infants, teenagers, and young adults and accumulation of the respective genes in the lower-right/upper-left part of the maps, respectively. The methylation profiles are relatively noisy because genes were selected from transcriptomic analyses (see above). Overall, expression and methylation profiles are mostly mirror-symmetrical thus reflecting virtually anti-correlation. The respective gene sets were assigned to different cell types such as neurons, astrocytes, microglia, and endothelial cells and associate with ALC, SCZ, AUT, and DPR, respectively (see [16] for details). The U-shaped characteristics resemble the hourglass-like courses of variability measures suggesting maximum vulnerability of relevant molecular processes in and around the adolescent age. Profiles of independent gene signatures confirm these results (Figure A11). For example, genes related to SCZ associate with astrocytes showing minimum activity in childhood-to-adolescent-young adult age ranges. Transcription of neurons (related to ALC), microglia (related to AUT) and oligodendrocytes, on the other hand is maximum in childhood and adolescent age ranges and decays with age. A monotonous decay of gene expression with age is observed for stemness and partly for senescence expression signatures (Figure A11). Note that the stemness signature was obtained from single cell transcriptomics of gliomas [93] while the senescence genes were extracted as senescence-associated hypermethylation signature [94] giving rise to decaying gene expression with age in both cases. Interestingly, both signatures however show different methylation courses suggesting different mechanisms acting via repression (senescence) and via intermediate repressive complexes (stemness), respectively (see above).

**Figure A12:**
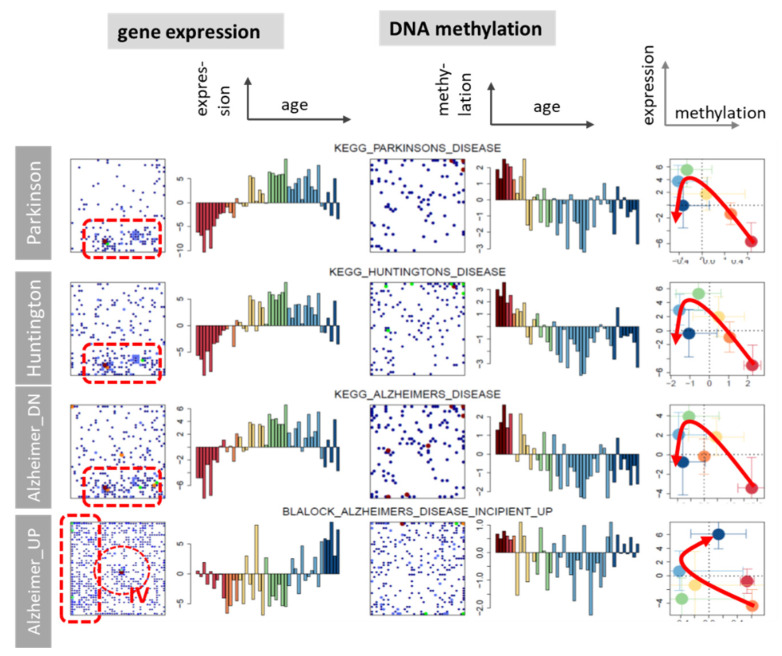
Expression and methylation characteristics of gene sets related to brain disorders taken from [43]. Genes associated with Parkinson, Huntington and Alzheimer’s diseases show virtually identical profiles with an expression maximum for teenagers/young adults and decaying methylation during childhood. Interestingly, genes associating with incipient Alzheimer’s disease show a virtually mirror symmetrical expression profile with a minimum at adolescent age. The authors suggest that pathogenesis of this disease is genomically orchestrated by the up-regulation of tumor suppressor-mediated differentiation which induces the spread of pathology along myelinated axons [120]. These genes possibly describe active drivers while the KEGG genes seem to associate with ubiquitous loss of function processes upon aging which are common between Alzheimer, Huntington and Parkinson.

**Figure A13:**
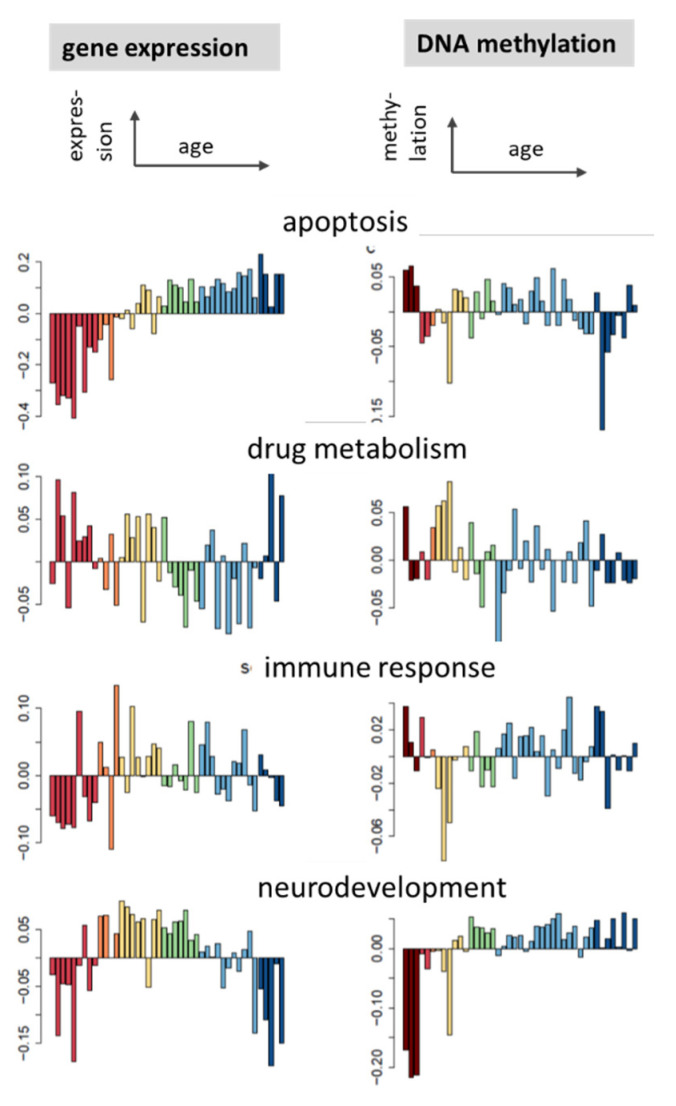
Expression and methylation characteristics of gene sets of different functional context related to schizophrenia taken from [121,122,123]. In healthy subjects, inflammatory processes are activated during the first years of life and then decreased and became overexpressed in oldness (regulation of inflammatory response). U-shaped changes upon aging are shown for secretion of neurotransmission in brain disorders and healthy controls as well. The same applied for neuronal death related processes with the maximum at childhood. U-shaped changes are common for presynapse assembly disrupted in mental and behavioral disorders. No significant changes depending on age in case of processes involved in the innate immune response in healthy subjects are shown while several studies indicated altered innate immunity both in psychiatric disorders and ALC [124,125]. The main player of immunity, the compliment system suggested association with SCZ implicated a new risk factor for disease development [122]. On the other hand, evidence suggested contribution of abnormal apoptosis in brain and behavioral disorder [126]. Interestingly, cytokine and chemokine network implicated in the abovementioned disorders seems to be constantly expressed regardless the age [121,127,128]. In particular, a strong contribution of chemo-cytokine network to SCZ development indicated [121,123,129]. In contrast, genes enrolled in long-term neuronal synaptic plasticity regulation are expressed almost constantly over the time. Despite alterations in neurodevelopment are involved in mental disorders, genes underlying these processes seem to have no age-dependence.

U-shaped expression courses are also found for a series of additional gene signatures of different brain disorders (Figure A12) and, particularly, SCZ (Figure A13 and discussion in the legend). Note that U-shaped courses mean that a trend in early ages reverses in elderly people. Brain neuronal development strongly implicated in mental disorders [95] and alcohol abuse, undergo age-dependent changes with a marked decrease in oldness. Activation of neurotrophin signaling pathway during the first years of life and U-shaped activation profile in healthy subjects are detected where our previous studies indicated association of neurodevelopmental genes including neurotrophins with SCZ [96,97,98]. U-shaped expression profiles found for neurogenesis suggest a negative regulation indicative for alterations in neurotransmitter system in brain disorders in contrast to healthy individuals [99].

In summary, transcriptomic signatures of psychiatric disorders typically show U- or inverse U-shaped profiles with maximum/minimum in the children-to-young adult age ranges resembling that of spots B–D or G, respectively. They associate with the maximum vulnerability of molecular processes in different brain cellular compounds such as neurons, microglia, or astrocytes, respectively. Assignment to distinct neuronal cell types based on previous knowledge and consideration of the corresponding age profiles of methylation provided different disease characteristics for AUT, SCZ, BPD, DPR, and ALC complementing the unique module-activity spectrum proposed in the original publication [16]. These data provide a quantitative, genome-wide characterization of the cortical pathology across major neuropsychiatric disorders.

### 3.9. G-Protein Coupled Receptors (GPCR)

**Figure 10:**
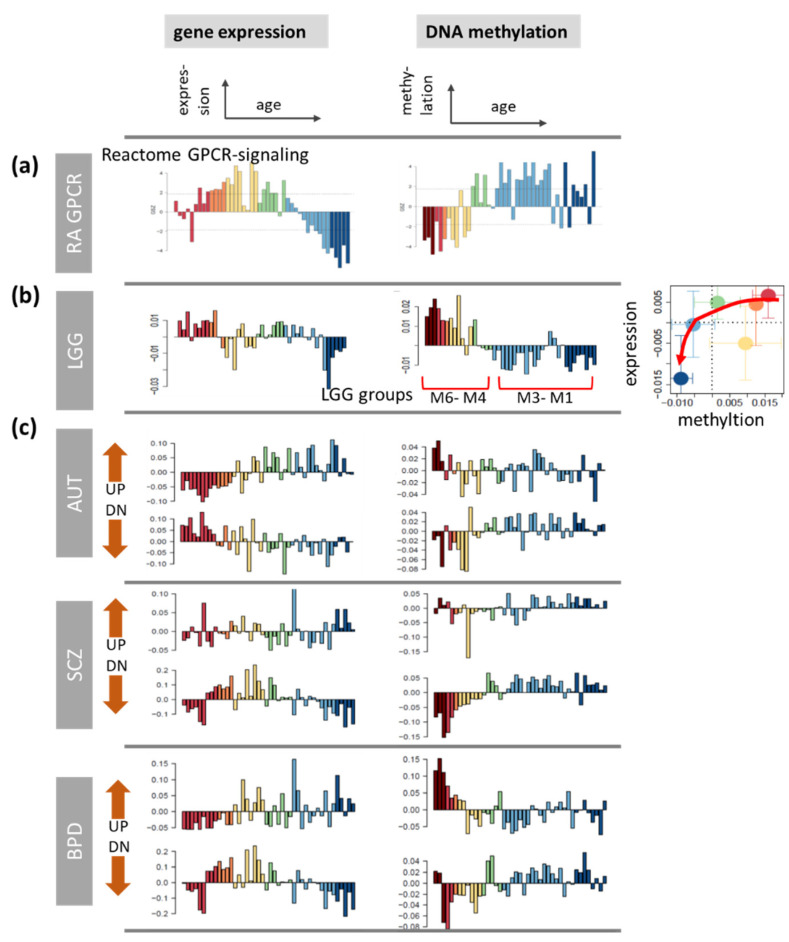
Age profiles of gene expression and DNA methylation of G-protein coupled receptors (*GPCR*). (**a**) *GPCR* genes collected in the set Reactome *GPCR* signaling, (**b**) the set of *GPCR* genes showing pronounced differential methylation in LGG methylation groups (spot B’ in [20]), and (**c**) sets of *GPCR* differentially expressed (up or down) in patients of different psychic disorders compared with healthy controls taken from [101]. The age ranges referring to the respective differential expression are marked by red marks.

Cells receive and translate extracellular signals to regulate processes such as differentiation, metabolism, and proliferation, via transmembrane receptors, where G protein-coupled receptors (*GPCR*s) belong to the largest family of such proteins, with several hundreds of members [100]. During normal aging, alterations in the activity of *GPCR*s in the brain associate with neuroderegeneration, increased vulnerability to neuropathologies [101] including the development of brain cancer [102]. For an overview, we generated aging profiles of *GPCR* collected in the Reactome (RA) *GPCR* signaling (Figure 10a) virtually agreeing with the profiles of the set GO BP *GPCR* activation (Figure A11). Gene expression shows an inverse U-shaped profile with its maximum at infant/childhood age followed by a strong decay upon further aging while methylation monotonously increases with age virtually leveling off for adults and elderly people. Part of *GPCR* of these sets accumulate in the central region of the expression SOM referring to age-invariant genes (Figure A11). Overall these profiles resemble the general aging profiles subsumed in spot B and the LU-aging_DN signature (Figure 2e,f) and spot E^m^ and the HORVATH-aging_UP (Figure 3e,f) signatures for expression and methylation, respectively.

As a next type of age profile, we studied a set of *GPCR* which were found to hypomethylate in part of the gliomas, particularly the *IDH*-wt group M1 and the IDH-A groups M2–M3 comprising the low-GCIMP tumors of inferior prognosis among the *IDH*-mut gliomas (see above Figure 8 and Figure 10b). While expression of this LGG-set is only weakly affected, its methylation is high at early childhood and then it markedly decays resembling the decay in developing gliomas. Hence, demethylation of these *GPCR* set seems to associate with tumor age of IDH-A type LGG, i.e., its developmental stage. In the healthy brain, this trend goes parallel with hypomethylation during aging especially during child- and adulthood. In LGG hypomethylation of these genes associates with a more inferior prognosis and activation of inflammatory processes resembling immunoaging [20,103,104].

Next, we studied age profiles of *GPCR*s of relevance for psychiatric disorders. Particularly, age profiles of sets of *GPCR* differentially expressed in AUT, SCZ, and BPD taken from [101] are considered. Genes encoding these *GPCR* were shown to be overrepresented in the lists of differentially expressed genes compared with normal controls and they partly overlap across the psychiatric disorders [101]. The age profiles reveal similar courses for up and downregulated genes where expression and methylation predominantly anticorrelate. The decay of the expression_DN profile of AUT starts at earlier ages than those of SCZ and BPD. Note that the *GPCR* extracted from all brain disorders show similar methylation profiles for up- and down-regulation, respectively, which throughout suggest repression of their expression. In summary, disease related *GPCR* show pronounced age dependence in the expression and especially methylation domains, where changes are most pronounced in early childhood.

## 4. Discussion

We performed a joint study of gene expression and DNA methylation of brain tissue collected from the human prefrontal cortex of individuals across the lifespan in order to describe changes of cellular programs and their regulation by epigenetic mechanisms. We analyzed previous knowledge in terms of functional gene signatures and chromatin states derived from independent studies, aging profiles of a battery of chromatin modifying profiles and data about gliomas and neuropsychological disorders for a holistic view on (epi-)genomic development and aging of the brain. Despite this wide perspective, our study has a number of limitations: We used bulk omics data from only one brain region (prefrontal cortex) without cellular resolution obtained by means of microarray measurement platforms of unmatched expression and methylation samples. Other brain regions such as the hippocampus also plays a large role in the processes of learning and synaptic plasticity not considered here. Gene expression and DNA methylation studies on hyppocampus tissues in humans and model animals reported different aspects also including aging but need integration into a more holistic view and comparison with other brain regions [105,106,107,108,109]. Further, DNA methylation characterizes only promoter regions of the genes, and prenatal age (3 methylation samples) were not matched with expression data. Hence, our analysis might miss a number of regulatory interactions and the observation scale is relatively coarse within the different age intervals and also with respect to cellular and genetic resolution. Observed age-dependent changes likely point more to changes in cellular composition rather than developmental dynamics of a single cell type and thus demonstrate the predicaments when heterogeneous cell populations are examined [61]. Further, our analysis focused on DNA methylation leaving aging factors such as histone acetylation and DNA loopings formed by cohesin and CTCF unconsidered [110].

Despite these drawbacks, our analysis provides relatively highly resolved patterns of developmental and aging dynamics of brains gene expression and DNA methylation. It decomposes into a manifold of modes of different aging profiles such as monotonously and smoothly increasing, decaying, U- and inverse U-shaped (with maximum/minimum at intermediate ages), and profiles of steep changes at early and later ages as well. Promoter methylation and expression of downstream genes are anti-correlated as a rule of thumb, however, a large number of genes deviate from this simple relation. Overall, a clear one-to-one relation is missing presumably because of the overlay of different regulatory effects not directly affected by methylation such as transcription factor networks [74], genetic defects [20,21], and/or passenger genes regulated indirectly by methylation changes in upstream drivers. We also observe positively correlated interactions where methylation activates expression. Such interactions possibly indicate interactions via an intermediate repressive complex such as PRC2 often acting mutually exclusive from methylation [44].

Along the lifetime scale, serial activation of these modes associates with functions such as neuronal development, maturated neuronal function such as synaptic transmission in babies and children, and later immune response and inflammation, metabolic and transcriptional activity in adults and elderly persons. Most changes, especially of the methylome, are established during development and childhood, while methylomes are less dynamic later in life. We find an hourglass-shaped transition of gene expression variance between two processes in adolescents’ brains, which presumably refer to the finalization of neuronal development and maturation and the onset of aging in early adulthood. Another hourglass-shaped “neurotypic” region around birth was recently reported [35], where transcription specialization of processes involved in constructing brain circuitry in prenatal development decreases but afterwards post-natal development starts largely due to specialization of plasticity and neural activity processes subsumed as “learning”. Although resolution of our data is insufficient for clearly resolving this neurotypic region, we see strong changes of methylation in prenatal brains quickly levelling off after birth.

U-shaped aging profiles indicate that gene expression changes with impact, e.g., for brain development and synaptic transmission. They reflect a partial reversal of developmental patterns upon aging, suggesting a link between developmental regulation and expression changes taking place in aging meaning that gene expression changes attributed to aging, such as down-regulation of neural genes, are initiated in early childhood. Hence, a number of regulatory processes continue throughout the entire life span where processes, beneficial in development, reverse direction and might be detrimental in aging [25].

**Figure 11:**
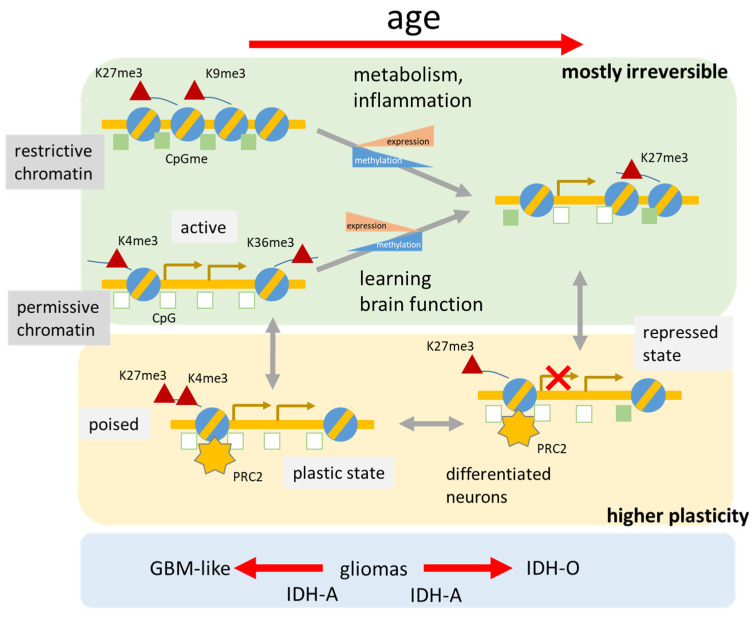
Combined changes of DNA methylation, gene expression, and chromatin states relate to brain development and aging. DNA demethylation of restrictive chromatin and methylation of permissive chromatin activate/deactivate different cell functions with age in a mostly irreversible fashion. Differentiation of neuronal tissue proceeds in active states which then become stabilized after repression of developmental genes. Poised states maintain plasticity while PRC2-repressed states maintain cellular identity of differentiated neuronal tissue. In particular, transitions between the latter states are partly reversible due to (de-)methylation equilibria governed by the activity of methyl writing, erasing and reading enzymes, and a large battery of cofactors such as PRC2.

Although aging induces diverse epigenetic changes in the brain, methylation appears to play a prominent regulatory role (Figure 11). The fastest changes occur during the prenatal period, slow down markedly after birth, and continue to slow further with aging [111]. The transition from fetal to postnatal life is typified by a reversal of direction, from demethylation prenatally to increased methylation postnatally. DNA methylation is strongly associated with expression of genes involved in brain development and in de novo DNA methylation. The methylation status can change bidirectionally, where restrictive DNA regions tend to become hypomethylated while many developmental genes in permissive chromatin regions become hypermethylated [11,13]. This way aging seems to disturb mechanisms of proper gene repression and activation. Importantly, utilizing genomic segments assigned to different chromatin states rather than individual CpGs will provide more comprehensive (epi-)genomic contexts to understand the intricate associations between genomic neighborhoods and developmental and aging processes.

In contrast to most genetic lesions, DNA methylation is considered a reversible epigenetic mark which can vary during differentiation and in diseases such as cancer. Methyl groups can be added, removed, and interpreted by various classes of proteins collectively known as “writers”, “erasers” and “readers”, respectively. The disruption of these epigenetic mechanisms and their molecular machinery can have catastrophic consequences on the nervous system. The local levels of DNA methylation result from opposing enzymatic activities, the rates of which are largely unknown. We here provided a glimpse at this area of regulatory mechanisms. The expression levels of writers, erasers, and readers of methylation marks at DNA and histone side chains are indeed highly dynamic in the developing and aging brain (Figure 11). Consequences for the particular genomic contexts are difficult to access because enzymatic rates can vary as much as two orders of magnitude between CpGs with identical steady-state DNA methylation outcome [56]. De novo methylation, as well as passive and active demethylation activities, is affected by local variations in chromatin, transcriptional activity, and transcription factor binding, leading to complex rate patterns which result in the steady-state methylation levels observed. Our data reveal a highly variable and presumably context-specific activity of the DNA and histone methylation machinery during lifetime.

We observed accumulation of methylation in bivalent (poised, TssP) genomic regions with age which is likely to be a common process that occurs across tissue types [112,113]. In the aging brain, this accumulation might be targeted to loci with important roles in cell differentiation and development, and the closing off of these developmental pathways. On the other hand, for proper homeostasis and functioning of the adult brain, a balanced control of neural stem/progenitor cell self-renewal, differentiation, production of neurons and glia (known as “adult neurogenesis”), repair, learning, and memory, are important which requires a residual level of developmental plasticity [2]. Hereby *PRC2* in adult neurons suppress the transcription of bivalent genes which protects neurons against degeneration. In other words, *PRC2*s contribute to the suppression of a transcriptional program that is detrimental to adult neuron function and survival. *PRC2* deficiency in consequence leads to the de-repression of predominantly bivalent *PRC2* target genes normally suppressed in these neurons leading to neuronal degeneration and dysfunction [73]. Recent results show that DNA methylation and *PRC2* can coexist in several contexts suggesting that *PRC2* might not necessarily have any preference for DNA methylation ([44] and references cited therein). The mutual exclusion of DNA methylation and H3K27me3 in the genome is probably dependent on context with unknown factors.

Histone modifications affect chromatin states which trigger transcriptional processes that set neurons on a path of neurodegeneration [114]. The role of higher-order chromatin structure and DNA methylation in transcriptional regulation of brain disorders has been shown with considerable overlap of the genetic and epigenetic risk architecture, e.g., between SCZ, DPR, and BPD [55,115]. Comparison of aging brain with LGG revealed parallels, particularly that GBM-like *IDH*-wt LGG exhibit higher cellular plasticity activated in the developing healthy brain while *IDH*-mut LGG more resemble the aged brain. Hence, glioma development can be partly understood in terms of analogies of brain development along the aging axis in both directions depending on the glioma subtype. Aberrant methylation of the olfactory subgenome and particularly of G-protein coupled receptors observed in LGG [20,21] seems to be a more general, but largely not understood phenomenon with an impact on diverse brain disorders.

## 5. Conclusions

Our analysis highlighted aspects of the dynamic nature of the developing and aging epigenome and of associated cellular functions. A more systematic understanding of the role and precise timing of age-associated changes in molecular players is required for a deeper insight into these processes. One important point in the role of epigenomic changes with age is the balance between volatile and stable and between more irreversible and more plastic networks. Understanding how genetic aberrations and environmental stimuli modulate these networks is required not only to increase our understanding of aging, but also of the initiation and courses of neuropsychological disorders and of cancer. Larger-scale analyses and increased cellular resolution are needed to link molecular players such as gene expression and DNA methylation with neuronal nets and cognitive functions and their development and aging.

## Figures and Tables

**Figure A10:**
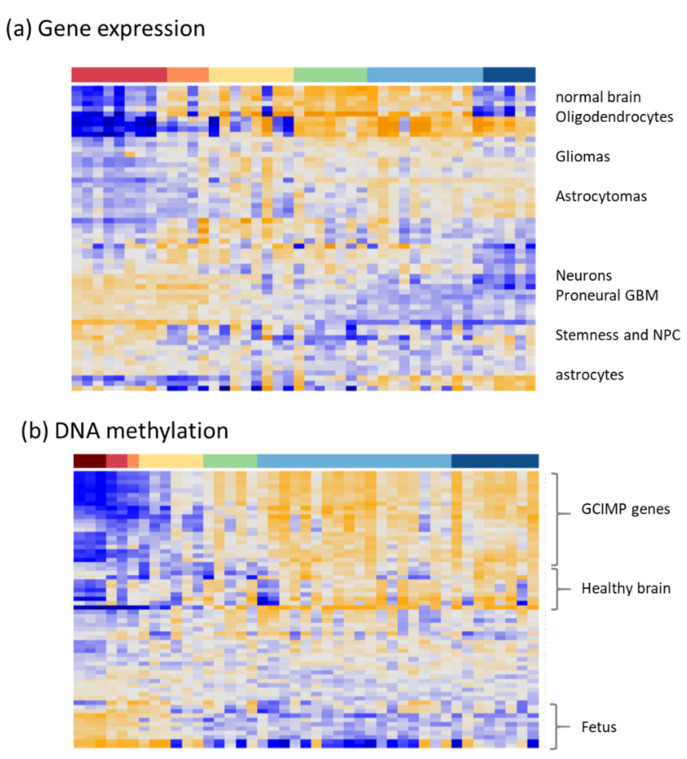
Gene expression (part **a**) and DNA-methylation (part **b**) of gene signatures published in previous glioma studies for different glioma subtypes, methylation classes and healthy brain signatures (see [21,29] and references cited therein).

## Data Availability

Not applicable.

## Supplementary Materials

The following are available online at https://www.mdpi.com/article/10.3390/cells11030362/s1, Table S1: Lists of genes referring to the expression spots, Table S2: Lists of genes referring to the methylation spots, File S3: PDF-files of KEGG pathway expression activities, File S4: PDF-files of KEGG pathway methylation levels.

## List of Abbreviations

ALC	alcoholism
ALT	alternative lengthening of telomeres
AUT	autism
BP	gene sets related to biological processes; part of Gene Ontology (GO) data base
BPD	bipolar disorder
bp	base pairs (DNA nucleobases)
CD4 & 8	cluster of differentiation 4 and 8; co-receptors of T cells
CG	cytosin and guanine; see CpG
Chr	chromosome
CIMP	CpG island methylator phenotype in colorectal cancer
CpG island	genomic regions with high frequency of cytosine and guanine
CRC	colorectal cancer
CTCF	transcriptional repressor; also known as 11-zinc finger protein
DKC1	dyskerin pseudouridine synthase 1
DN	down-regulation in gene set or pathway analyses
DNA	deoxyribonucleic acid
Dnmt	DNA methylation maintenance methyltransferases
DPR	major depression
E-	expression- (profiles, SOM, spots, or portrait)
E1–E8	expression subtypes of LGG
exSOM	extension SOM transfer learning approach
EZH1	histone-lysine N-methyltransferase
GBM	glioblastoma WHO grade IV
GEO	Gene Expression Omnibus repository for Transcriptome data
GCIMP	glioma CpG island hypermethylation phenotype; associated with IHD mutation
*GPCR*	G-protein-coupled receptors
GO	Gene Ontology database
GSZ	gene set Z-score
H3K	methylation of lysine side chains (histone subunit 3) at positions 4, 9, 27 or 36
Het	heterochromatin
HetRpts	heterochromatin repeats
HR	homologous recombination
*IDH*	isocitrate dehydrogenase
IDH-A	*IDH*-mutated astrocytoma-like subset of gliomas with chromosome 1p19q intact
*IDH*-mut	gliomas carrying mutation in *IDH* genes
IDH-O	*IDH*-mutated oligodendroglioma-like subset of gliomas with chromosome 1p19q intact
*IDH*-wt	gliomas with wildtype *IDH* genes
KEGG	Kyoto Encyclopedia of Genes and Genomes; database of cellular pathways
KTM	lysine methyltransferases
KDM	lysine demethylases
LGG	lower-grade diffuse gliomas (WHO grade II and III)
M-	methylation- (profiles, SOM, spots, or portrait)
M1-M6	methylation subtypes of LGG
MES	mesenchymal subtype of gliobalstomas according to Verhaak et al. (2010)
MHC	Major Histocompatibility Complex
NL	neuronal-like subset of gliomas according to Verhaak et al. (2010)
NPC	neuronal progenitors
PA	pilocytic astrozytoma
PCM	pairwise correlation map
PRC1&2	polycomb repressive complex 1 and 2
PSF	pathway signal flow algorithm
RA	Reactome cellular pathway database
RC	repressive complexes
RepPRC	repressed polycomb states
RepPCWk	repressed polycomb states, weak
SCZ	schizophrenia
SOM	Self-organizing map
TEL	telomerase
TERT	telomerase reverse transcriptase
Tet1–Tet3	DNA demethylases
TM	telomere length maintenance
Treg	regulatory T cell
TssA/TssP	active/poised gene promoters
Tx	transcribed genes
UP	up-regulation in gene set or pathway analyses
WHSC1	histone-lysine N-methyltransferase
ZNF	zinc finger proteins

## Appendix B. Tables

**Table A1:** Functional context of expression spots.

Spot	Notation	Up/DN	Top Genes ^(a)^	Biological Function/Enriched Gene Sets ^(b)^
A	Newborn_up; “development”	Newborn/Teenager, Adult, Late Adult	*SOX11, DCX, CRMP1, TENM1, STMN2, KIF21B, DPYSL3, RPL12, TUBB, JPT1*	BP_nervous-system-development, BP_cell-adhesion, MEISSNER_BRAIN_HCP_ WITH_H3K4ME3_ AND_H3K27ME3, Benporath_ES-with_H3K27me3, VAQUERIZAS_Fetal brain_TF, Hebenstreit_low-expression-TFs, Verhaak_Glioblastoma_proneural
B	Baby_up; “synaptic transmission”	Baby, Infant, Teenager/Late Adult	*SCAMP1, TBC1D9, CNTN1, PRKCB, SUMO3, RAB6A, FKBP1A, FYTTD1, MYT1L, NDFIP1*	BP_chemical-synaptic-transmission, LU_AGING_BRAIN_DN, REACTOME_NEURONAL_ SYSTEM, repressed chromatin states fetal brain, Reifenberger_Glioblastoma_CL&PNwt, Chaussabel_cytoskeleton, GO_CC_Synapse
C	Teenager_up; “transport”	Infant, Teenager/Late Adult	*PCMT1, GHITM, LMBR1, C8orf46, SKP1, TRIM23, OXR1, RGS4, IDH3A, GABARAPL1*	BP_transport, BLALOCK_ALZHEIMERS_ DISEASE_ DN, Reifenberger_Glioblastoma_CL&PNwt, KIM_Myc-targets, LU_AGING_BRAIN_DN
D	Infant_up; “signal transduc-tion”	Infant, Teenager/Late Adult	*NRGN, ARPP19, NGEF, ARHGDIB, OLFM1, PIN1, JCAD, ATG101, LRPAP1, YJEFN3*	BP_signal-transduction, CC_plasma-membrane, WILLSCHER_GBM_Verhaak−CL & MES_up, Lembcke_CIMP_H, Verhaak_Glioblastoma-Mesenchymal
E	Adult_up; “nervous system”	Adult/Newborn, Baby	*ITPR1, RCAN2, KCNAB2, BCL2L2, CABP1, STEAP2, ITPR1, RIMS3, EIF5A2, ATP6V1E1*	Wirth_nervous-system; Blalock_Alzheimer-disease-DN; KIM_ALL_DISORDERS_OLIGODENDROCYTE_ NUMBER_CORR_UP, Glioblastoma_DN; CC_mylin-sheath
F	Late Adult_up1; “lipid metabolism”	Adult, Late Adult/ Baby, Infant	*PIP4K2A, USP54, HSPA2, GPRC5B, PLEKHB1, PLEKHH1, ANP32B, CLDND1, MAN2A1, TMEM144*	BP_lipid-metabolic process; BP_myelination, ASTON_MAJOR_DEPRESSIVE_DISORDER_DN, BLALOCK_ALZHEIMERS_DISEASE_UP, LU_AGING_BRAIN_UP, MEISSNER_BRAIN_ HCP_ WITH_H3K4ME3_AND_H3K27ME3, OL versus OPC
G	Late Adult_up2; “transcription”	Late Adult/ Baby, Infant, Teenager	*DTNA, ADD3, PLPP3, BMPR1B, PLPP3, RHOBTB3, ADD3, ALDH9A1, METTL7A, ACSS1*	BP_positive-regulation of-transcription, LU_AGING_BRAIN_UP, BLALOCK_ALZHEIMERS_DISEASE_UP, Lein_astrocyte-markers, Verhaak_Glioblastoma-classical, Chaussabel_Interferon-inducable

(a) Top 10 genes with largest correlation coefficient of their expression profile with spot profile (r > 0.9). See Supplementary Materials, Supplementary Table S1, for full gene list, gene names and further details. (b) Gene sets were implemented in oposSOM ([27,28] and references cited therein). Enrichment was calculated using Fisher’s exact test [51]. Only gene sets with enrichment *p* < 10^−6^ were considered.

**Table A2:** Functional context of methylation spots.

Spot	Notation	Up/DN	Top Genes ^(a)^	Biological Function/Enriched Gene Sets ^(b)^
A^m^	Fetal_up;	Newborn/Teenager, Adult, Late Adult	*MPG, IPO8, WFDC3, DNTTIP1, GOT2, RARRES3, SSRP1, ABHD14B, FAM162A, CCDC58*	Horvath_aging-genes_DN, Hallmark_Interferon-gamma/alpha_response, Lembcke_CIMP_hypermeth_UP, Hopp_IDH-meth-DN
B^m^	Baby_up;	Baby	*NUP98, ZDHHC4, HLTF, EAPP, RNASEH2A, TKFC, TMEM175, GPS2, HYAL3, CCNB1*	Lembcke_CIMP_hypermeth_DN,
C^m^	Infant;	Infant	*SYF2, ADAM17, PHC1, SECISBP2, MTDH, CHPT1, ZRANB2, USP49, MRPL51*	No set enriched
D^m^	Infant_up; late-adult_up	Infant, Late Adult	*LAD1, HESX1, PYY, DKK4, SEL1L3, TP73, FOXD4L1, PIP5KL1, MYL4, WDR86*	Hopp_Fetus_methylation_UP, CC-nucleus, BP_RNA-binding
E^m^	Late adult	Adult/Newborn, Baby	*SALL4, GPR17, MIXL1, SYTL1, CFTR, NRK, IL18, DCT, SYNE2, CCDC69*	Hopp_IDH-meth_UP, Benporath_ES-H3K27me3, Benporath_PRC2-targets

(a) Top 10 genes with largest correlation coefficient of their methylation profile with spot profile (r > 0.9). See Supplementary Materia, Supplementary Table S2 for full gene list, gene names and further details. (b) Gene sets were implemented in oposSOM ([27,28] and references cited therein). Enrichment was calculated using Fisher’s exact test [104]. Only gene sets with enrichment *p* < 10^−6^ were considered.

## References

[1] Yang X.W. (2016). Life and death rest on a bivalent chromatin state. Nat. Neurosci..

[2] Kim-Ha J., Kim Y.-J. (2016). Age-related epigenetic regulation in the brain and its role in neuronal diseases. BMB Rep..

[3] Booth L.N., Brunet A. (2016). The Aging Epigenome. Mol. Cell.

[4] Zhang W., Qu J., Liu G.H., Belmonte J.C.I. (2020). The ageing epigenome and its rejuvenation. Nat. Rev. Mol. Cell Biol..

[5] Feng J., Fouse S., Fan G. (2007). Epigenetic Regulation of Neural Gene Expression and Neuronal Function. Pediatr. Res..

[6] Akbarian S., Beeri M.S., Haroutunian V. (2013). Epigenetic Determinants of Healthy and Diseased Brain Aging and Cognition. JAMA Neurol..

[7] Kuehner J.N., Bruggeman E.C., Wen Z., Yao B. (2019). Epigenetic Regulations in Neuropsychiatric Disorders. Front. Genet..

[8] Bakken T.E., Miller J.A., Luo R., Bernard A., Bennett J.L., Lee C.-K., Lein E.S. (2015). Spatiotemporal dynamics of the postnatal developing primate brain transcriptome. Hum. Mol. Genet..

[9] Somel M., Franz H., Yan Z., Lorenc A., Guo S., Giger T., Khaitovich P. (2009). Transcriptional neoteny in the human brain. Proc. Natl. Acad. Sci. USA.

[10] Teschendorff A.E., West J., Beck S. (2013). Age-associated epigenetic drift: Implications, and a case of epigenetic thrift?. Hum. Mol. Genet..

[11] Sun D., Yi S.V. (2015). Impacts of Chromatin States and Long-Range Genomic Segments on Aging and DNA Methylation. PLoS ONE.

[12] Hernandez D.G., Nalls M.A., Gibbs J.R., Arepalli S., van der Brug M., Chong S., Singleton A.B. (2011). Distinct DNA methylation changes highly correlated with chronological age in the human brain. Hum. Mol. Genet..

[13] Lin Q., Wagner W. (2015). Epigenetic Aging Signatures Are Coherently Modified in Cancer. PLoS Genet..

[14] Mack S.C., Hubert C.G., Miller T.E., Taylor M.D., Rich J.N. (2016). An epigenetic gateway to brain tumor cell identity. Nat. Neurosci..

[15] Bakken T.E., Miller J.A., Ding S.-L., Sunkin S.M., Smith K.A., Ng L., Lein E.S. (2016). A comprehensive transcriptional map of primate brain development. Nature.

[16] Gandal M.J., Haney J.R., Parikshak N.N., Leppa V., Ramaswami G., Hartl C., Geschwind D.H. (2018). Shared molecular neuropathology across major psychiatric disorders parallels polygenic overlap. Science.

[17] Numata S., Ye T., Hyde T.M., Guitart-Navarro X., Tao R., Wininger M., Lipska B.K. (2012). DNA methylation signatures in development and aging of the human prefrontal cortex. Am. J. Hum. Genet..

[18] Schmidt M., Hopp L., Arakelyan A., Kirsten H., Engel C., Wirkner K., Binder H. (2020). The Human Blood Transcriptome in a Large Population Cohort and Its Relation to Aging and Health. Front. Big Data.

[19] Loeffler-Wirth H., Kreuz M., Hopp L., Arakelyan A., Haake A., Cogliatti S.B., Feller A.C., Hansmann M., Lenze D., Möller P. (2019). A modular transcriptome map of mature B cell lymphomas. Genome Med..

[20] Willscher E., Hopp L., Kreuz M., Schmidt M., Hakobyan S., Arakelyan A., Binder H. (2021). High-Resolution Cartography of the Transcriptome and Methylome Landscapes of Diffuse Gliomas. Cancers.

[21] Binder H., Willscher E., Loeffler-Wirth H., Hopp L., Jones D.T.W., Pfister S.M., Loeffler M. (2019). DNA methylation, transcriptome and genetic copy number signatures of diffuse cerebral WHO grade II/III gliomas resolve cancer heterogeneity and development. Acta Neuropathol. Commun..

[22] Hopp L., Löffler-Wirth H., Galle J., Binder H. (2018). Combined SOM-portrayal of gene expression and DNA methylation landscapes disentangles modes of epigenetic regulation in glioblastoma. Epigenomics.

[23] Hopp L., Wirth-Loeffler H., Binder H. (2015). Epigenetic heterogeneity of B-cell lymphoma: DNA-methylation, gene expression and chromatin states. Genes.

[24] Hopp L., Nersisyan L., Löffler-Wirth H., Arakelyan A., Binder H. (2015). Epigenetic Heterogeneity of B-Cell Lymphoma: Chromatin Modifiers. Genes.

[25] Somel M., Guo S., Fu N., Yan Z., Hu H.Y., Xu Y., Khaitovich P. (2010). MicroRNA, mRNA, and protein expression link development and aging in human and macaque brain. Genome Res..

[26] Wirth H., Löffler M., von Bergen M., Binder H. (2011). Expression cartography of human tissues using self organizing maps. BMC Bioinform..

[27] Löffler-Wirth H., Kalcher M., Binder H. (2015). oposSOM: R-package for high-dimensional portraying of genome-wide expression landscapes on bioconductor. Bioinformatics.

[28] Wirth H., von Bergen M., Binder H. (2012). Mining SOM expression portraits: Feature selection and integrating concepts of molecular function. BioData Min..

[29] Hopp L., Willscher E., Wirth-Loeffler H., Binder H. (2015). Function Shapes Content: DNA-Methylation Marker Genes and their Impact for Molecular Mechanisms of Glioma. J. Cancer Res. Updates.

[30] Kundaje A., Meuleman W., Ernst J., Bilenky M., Yen A., Kellis M., Roadmap Epigenomics Consortium (2015). Integrative analysis of 111 reference human epigenomes. Nature.

[31] Ernst J., Kheradpour P., Mikkelsen T.S., Shoresh N., Ward L.D., Epstein C.B., Bernstein B.E. (2011). Mapping and analysis of chromatin state dynamics in nine human cell types. Nature.

[32] Schmidt M., Arshad M., Bernhart S.H., Hakobyan S., Arakelyan A., Loeffler-Wirth H., Binder H. (2021). The Evolving Faces of the SARS-CoV-2 Genome. Viruses.

[33] Jongeneel C.V., Delorenzi M., Iseli C., Zhou D., Haudenschild C.D., Khrebtukova I., Vasicek T.J. (2005). An atlas of human gene expression from massively parallel signature sequencing (MPSS). Genome Res..

[34] Lu T., Pan Y., Kao S.-Y., Li C., Kohane I., Chan J., Yankner B.A. (2004). Gene regulation and DNA damage in the ageing human brain. Nature.

[35] Liscovitch N., Chechik G. (2013). Specialization of gene expression during mouse brain development. PLoS Comput. Biol..

[36] Işıldak U., Somel M., Thornton J.M., Dönertaş H.M. (2020). Temporal changes in the gene expression heterogeneity during brain development and aging. Sci. Rep..

[37] Teschendorff A.E., Menon U., Gentry-Maharaj A., Ramus S.J., Weisenberger D.J., Shen H., Widschwendter M. (2010). Age-dependent DNA methylation of genes that are suppressed in stem cells is a hallmark of cancer. Genome Res..

[38] Horvath S. (2013). DNA methylation age of human tissues and cell types. Genome Biol..

[39] Johnson A.A., Akman K., Calimport S.R.G., Wuttke D., Stolzing A., de Magalhães J.P. (2012). The role of DNA methylation in aging, rejuvenation, and age-related disease. Rejuvenation Res..

[40] Bell C.G., Lowe R., Adams P.D., Baccarelli A.A., Beck S., Bell J.T., Rakyan V.K. (2019). DNA methylation aging clocks: Challenges and recommendations. Genome Biol..

[41] Peters M.J., Joehanes R., Pilling L.C., Schurmann C., Conneely K.N., Powell J., Johnson A.D. (2015). The transcriptional landscape of age in human peripheral blood. Nat. Commun..

[42] Hopp L., Wirth H., Fasold M., Binder H. (2013). Portraying the expression landscapes of cancer subtypes: A glioblastoma multiforme and prostate cancer case study. Syst. Biomed..

[43] Subramanian A., Tamayo P., Mootha V.K., Mukherjee S., Ebert B.L., Gillette M.A., Mesirov J.P. (2005). Gene set enrichment analysis: A knowledge-based approach for interpreting genome-wide expression profiles. Proc. Natl. Acad. Sci. USA.

[44] Yang Y., Li G. (2020). Post-translational modifications of PRC2: Signals directing its activity. Epigenetics Chromatin.

[45] Lowe R., Overhoff M.G., Ramagopalan S.V., Garbe J.C., Koh J., Stampfer M.R., Bishop C.L. (2015). The senescent methylome and its relationship with cancer, ageing and germline genetic variation in humans. Genome Biol..

[46] Nersisyan L., Hopp L., Loeffler-Wirth H., Galle J., Loeffler M., Arakelyan A., Binder H. (2019). Telomere Length Maintenance and Its Transcriptional Regulation in Lynch Syndrome and Sporadic Colorectal Carcinoma. Front. Oncol..

[47] Thomas P., O’ Callaghan N.J., Fenech M. (2008). Telomere length in white blood cells, buccal cells and brain tissue and its variation with ageing and Alzheimer’s disease. Mech. Ageing Dev..

[48] Palmos A.B., Duarte R.R.R., Smeeth D.M., Hedges E.C., Nixon D.F., Thuret S., Powell T.R. (2020). Telomere length and human hippocampal neurogenesis. Neuropsychopharmacology.

[49] Tsoukalas D., Buga A.M., Docea A.O., Sarandi E., Mitrut R., Renieri E., Calina D. (2021). Reversal of brain aging by targeting telomerase: A nutraceutical approach. Int, J. Mol. Med..

[50] Saretzki G., Wan T. (2021). Telomerase in Brain: The New Kid on the Block and Its Role in Neurodegenerative Diseases. BioMed.

[51] Nersisyan L., Loeffler-Wirth H., Arakelyan A., Binder H. (2016). Gene set- and pathway- centered knowledge discovery assigns transcriptional activation patterns in brain, blood and colon cancer-A bioinformatics perspective. J. Bioinform. Knowl. Min..

[52] D’Souza L., Channakkar A.S., Muralidharan B. (2021). Chromatin remodelling complexes in cerebral cortex development and neurodevelopmental disorders. Neurochem. Int..

[53] Arand J., Spieler D., Karius T., Branco M.R., Meilinger D., Meissner A., Walter J. (2012). In Vivo Control of CpG and Non-CpG DNA Methylation by DNA Methyltransferases. PLoS Genet..

[54] Fu A.Q., Genereux D.P., Stöger R., Burden A.F., Laird C.D., Stephens M. (2012). Statistical Inference of In Vivo Properties of Human DNA Methyltransferases from Double-Stranded Methylation Patterns. PLoS ONE.

[55] Bayraktar G., Kreutz M.R. (2017). Neuronal DNA Methyltransferases: Epigenetic Mediators between Synaptic Activity and Gene Expression?. Neuroscience.

[56] Ginno P.A., Gaidatzis D., Feldmann A., Hoerner L., Imanci D., Burger L., Schübeler D. (2020). A genome-scale map of DNA methylation turnover identifies site-specific dependencies of DNMT and TET activity. Nat. Commun..

[57] Cui D., Xu X. (2018). DNA Methyltransferases DNA Methylation, and Age-Associated Cognitive Function. Int. J. Mol. Sci..

[58] Hahn A., Pensold D., Bayer C., Tittelmeier J., González-Bermúdez L., Marx-Blümel L., Zimmer-Bensch G. (2020). DNA Methyltransferase 1 (DNMT1) Function Is Implicated in the Age-Related Loss of Cortical Interneurons. Front. Cell Dev. Biol..

[59] Santiago M., Antunes C., Guedes M., Sousa N., Marques C.J. (2014). TET enzymes and DNA hydroxymethylation in neural development and function—How critical are they?. Genom..

[60] Prasad R., Jho E.H. (2019). A concise review of human brain methylome during aging and neurodegenerative diseases. BMB Rep..

[61] Stricker S.H., Götz M. (2018). DNA-Methylation: Master or Slave of Neural Fate Decisions?. Front. Neurosci..

[62] Ernst J., Kellis M. (2010). Discovery and characterization of chromatin states for systematic annotation of the human genome. Nat. Biotech..

[63] Rohlf T., Steiner L., Przybilla J., Prohaska S., Binder H., Galle J. (2012). Modeling the dynamic epigenome: From histone modifications towards self-organizing chromatin. Epigenomics.

[64] Steiner L., Hopp L., Wirth H., Galle J., Binder H., Prohaska S.J., Rohlf T. (2012). A Global Genome Segmentation Method for Exploration of Epigenetic Patterns. PLoS ONE.

[65] Binder H., Steiner L., Wirth H., Rohlf T., Prohaska S., Galle J. (2013). Transcriptional regulation by histone modifications: Towards a theory of chromatin re-organization during stem cell differentiation. Phys. Biol.

[66] Collins B.E., Greer C.B., Coleman B.C., Sweatt J.D. (2019). Histone H3 lysine K4 methylation and its role in learning and memory. Epigenetics Chromatin.

[67] Pan M.-R., Hsu M.-C., Chen L.-T., Hung W.-C. (2018). Orchestration of H3K27 methylation: Mechanisms and therapeutic implication. Cell. Mol. Life Sci..

[68] Strahl B.D., Allis C.D. (2000). The language of covalent histone modifications. Nature.

[69] Kim Y.Z. (2014). Altered Histone Modifications in Gliomas. Brain Tumor Res. Treat..

[70] Barth T.K., Imhof A. (2010). Fast signals and slow marks: The dynamics of histone modifications. Trends Biochem. Sci..

[71] Montavon T., Shukeir N., Erikson G., Engist B., Onishi-Seebacher M., Ryan D., Jenuwein T. (2021). Complete loss of H3K9 methylation dissolves mouse heterochromatin organization. Nat. Commun..

[72] Lee J.-H., Kim E.W., Croteau D.L., Bohr V.A. (2020). Heterochromatin: An epigenetic point of view in aging. Exp. Mol. Med..

[73] Von Schimmelmann M., Feinberg P.A., Sullivan J.M., Ku S.M., Badimon A., Duff M.K., Schaefer A. (2016). Polycomb repressive complex 2 (PRC2) silences genes responsible for neurodegeneration. Nat. Neurosci..

[74] Thalheim T., Hopp L., Binder H., Aust G., Galle J. (2018). On the Cooperation between Epigenetics and Transcription Factor Networks in the Specification of Tissue Stem Cells. Epigenomes.

[75] Ziffra R.S., Kim C.N., Ross J.M., Wilfert A., Turner T.N., Haeussler M., Nowakowski T.J. (2021). Single-cell epigenomics reveals mechanisms of human cortical development. Nature.

[76] Kishi Y., Gotoh Y. (2018). Regulation of Chromatin Structure During Neural Development. Front. Neurosci..

[77] Ernst J., Kellis M. (2017). Chromatin-state discovery and genome annotation with ChromHMM. Nat. Protoc..

[78] Flavahan W.A., Gaskell E., Bernstein B.E. (2017). Epigenetic plasticity and the hallmarks of cancer. Science.

[79] Ceccarelli M., Barthel F.P., Malta M.T., Sabedot T.S., Salama S.R., Murray B.A., Verhaak R.G.W. (2016). Molecular Profiling Reveals Biologically Discrete Subsets and Pathways of Progression in Diffuse Glioma. Cell.

[80] Christensen B.C., Smith A.A., Zheng S., Koestler D.C., Houseman E.A., Marsit C.J., Wiencke J.K. (2011). DNA Methylation, Isocitrate Dehydrogenase Mutation, and Survival in Glioma. J. Natl. Cancer Inst..

[81] Noushmehr H., Weisenberger D.J., Diefes K., Phillips H.S., Pujara K., Berman B.P., Aldape K. (2010). Identification of a CpG Island Methylator Phenotype that Defines a Distinct Subgroup of Glioma. Cancer Cell.

[82] Dabrowski J.M., Wojtas B. (2019). Global DNA Methylation Patterns in Human Gliomas and Their Interplay with Other Epigenetic Modifications. Int. J. Mol. Sci..

[83] Capper D., Jones D.T.W., Sill M., Hovestadt V., Schrimpf D., Sturm D., Pfister S.M. (2018). DNA methylation-based classification of central nervous system tumours. Nature.

[84] Sturm D., Bender S., Jones D.T.W., Lichter P., Grill J., Becher O., Pfister S.M. (2014). Paediatric and adult glioblastoma: Multiform (epi)genomic culprits emerge. Nat. Rev. Cancer.

[85] Sturm D., Witt H., Hovestadt V., Khuong-Quang D.-A., Jones D.T.W., Konermann C., Pfister S.M. (2012). Hotspot Mutations in H3F3A and IDH1 Define Distinct Epigenetic and Biological Subgroups of Glioblastoma. Cancer Cell.

[86] Chaligne R., Gaiti F., Silverbush D., Schiffman J.S., Weisman H.R., Kluegel L., Landau D.A. (2021). Epigenetic encoding, heritability and plasticity of glioma transcriptional cell states. Nat. Genet..

[87] Suvà M.L., Tirosh I. (2020). The Glioma Stem Cell Model in the Era of Single-Cell Genomics. Cancer Cell.

[88] Weller M., Weber R.G., Willscher E., Riehmer V., Hentschel B., Kreuz M., Reifenberger G. (2015). Molecular classification of diffuse cerebral WHO grade II/III gliomas using genome- and transcriptome-wide profiling improves stratification of prognostically distinct patient groups. Acta Neuropathol..

[89] Bormann F., Rodríguez-Paredes M., Lasitschka F., Edelmann D., Musch T., Benner A., Lyko F. (2018). Cell-of-Origin DNA Methylation Signatures Are Maintained during Colorectal Carcinogenesis. Cell Rep..

[90] Hoadley K.A., Yau C., Hinoue T., Wolf D.M., Lazar A.J., Drill E., Laird P.W. (2018). Cell-of-Origin Patterns Dominate the Molecular Classification of 10,000 Tumors from 33 Types of Cancer. Cell.

[91] Binder H., Hopp L., Lembcke K., Wirth H., Baoying W., Ruowang L., William P. (2015). Big Data Analytics in Bioinformatics and Healthcare.

[92] Ben-Porath I., Thomson M.W., Carey V.J., Ge R., Bell G.W., Regev A., Weinberg R.A. (2008). An embryonic stem cell-like gene expression signature in poorly differentiated aggressive human tumors. Nat. Genet..

[93] Venteicher A.S., Tirosh I., Hebert C., Yizhak K., Neftel C., Filbin M.G., Suvà M.L. (2017). Decoupling genetics, lineages, and microenvironment in IDH-mutant gliomas by single-cell RNA-seq. Science.

[94] Xie W., Kagiampakis I., Pan L., Zhang Y.W., Murphy L., Tao Y., Easwaran H. (2018). DNA Methylation Patterns Separate Senescence from Transformation Potential and Indicate Cancer Risk. Cancer Cell.

[95] Levitt P., Veenstra-VanderWeele J. (2015). Neurodevelopment and the origins of brain disorders. Neuropsychopharmacol..

[96] Zakharyan R., Atshemyan S., Boyajyan A. (2014). Risk and protective effects of the complexin-2 gene and gene-environment interactions in schizophrenia. Recent Adv. DNA Gene Seq..

[97] Zakharyan R., Atshemyan S., Gevorgyan A., Boyajyan A. (2014). Nerve growth factor and its receptor in schizophrenia. BBA Clin..

[98] Zakharyan R., Boyajyan A. (2014). Brain-derived neurotrophic factor blood levels are decreased in schizophrenia patients and associate with rs6265 genotypes. Clin. Biochem..

[99] Sarter M., Bruno J.P., Parikh V. (2007). Abnormal neurotransmitter release underlying behavioral and cognitive disorders: Toward concepts of dynamic and function-specific dysregulation. Neuropsychopharmacology.

[100] De Oliveira P.G., Ramos M.L.S., Amaro A.J., Dias R.A., Vieira S.I. (2019). Gi/o-Protein Coupled Receptors in the Aging Brain. Front. Aging Neurosci..

[101] Monfared R.V., Alhassen W., Truong T.M., Gonzales M.A.M., Vachirakorntong V., Chen S., Alachkar A. (2021). Transcriptome Profiling of Dysregulated GPCRs Reveals Overlapping Patterns across Psychiatric Disorders and Age-Disease Interactions. Cells.

[102] Cherry A.E., Stella N. (2014). G protein-coupled receptors as oncogenic signals in glioma: Emerging therapeutic avenues. Neuroscience.

[103] Karantza V. (2011). Keratins in health and cancer: More than mere epithelial cell markers. Oncogene.

[104] Polioudaki H., Agelaki S., Chiotaki R., Politaki E., Mavroudis D., Matikas A., Theodoropoulos P.A. (2015). Variable expression levels of keratin and vimentin reveal differential EMT status of circulating tumor cells and correlation with clinical characteristics and outcome of patients with metastatic breast cancer. BMC Cancer.

[105] Lanke V., Moolamalla S.T.R., Roy D., Vinod P.K. (2018). Integrative Analysis of Hippocampus Gene Expression Profiles Identifies Network Alterations in Aging and Alzheimer’s Disease. Front. Aging Neurosci..

[106] Bienkowski M.S., Bowman I., Song M.Y., Gou L., Ard T., Cotter K., Dong H.-W. (2018). Integration of gene expression and brain-wide connectivity reveals the multiscale organization of mouse hippocampal networks. Nat. Neurosci..

[107] Altuna M., Urdánoz-Casado A., Sánchez-Ruiz de Gordoa J., Zelaya M.V., Labarga A., Lepesant J.M.J. (2019). Mendioroz, M. DNA methylation signature of human hippocampus in Alzheimer’s disease is linked to neurogenesis. Clin. Epigenetics.

[108] Alberry B.L.J., Singh S.M. (2020). Hippocampal DNA Methylation in a Mouse Model of Fetal Alcohol Spectrum Disorder That Includes Maternal Separation Stress Only Partially Explains Changes in Gene Expression. Front. Genet..

[109] Harris C.J., Davis B.A., Zweig J.A., Nevonen K.A., Quinn J.F., Carbone L., Gray N.E. (2020). Age-Associated DNA Methylation Patterns Are Shared Between the Hippocampus and Peripheral Blood Cells. Front. Genet..

[110] Michalak E.M., Burr M.L., Bannister A.J., Dawson M.A. (2019). The roles of DNA, RNA and histone methylation in ageing and cancer. Nat. Rev. Mol. Cell Biol..

[111] Price A.J., Collado-Torres L., Ivanov N.A., Xia W., Burke E.E., Shin J.H., Jaffe A.E. (2019). Divergent neuronal DNA methylation patterns across human cortical development reveal critical periods and a unique role of CpH methylation. Genome Biol..

[112] Watson C.T., Disanto G., Sandve G.K., Breden F., Giovannoni G., Ramagopalan S.V. (2012). Age-Associated Hyper-Methylated Regions in the Human Brain Overlap with Bivalent Chromatin Domains. PLoS ONE.

[113] Liu P.-P., Xu Y.-J., Teng Z.-Q., Liu C.-M. (2018). Polycomb Repressive Complex 2: Emerging Roles in the Central Nervous System. Neuroscience.

[114] Kozlenkov A., Wang M., Roussos P., Rudchenko S., Barbu M., Bibikova M., Dracheva S. (2016). Substantial DNA methylation differences between two major neuronal subtypes in human brain. Nucleic Acids Res..

[115] Nestler E.J., Peña C.J., Kundakovic M., Mitchell A., Akbarian S. (2015). Epigenetic Basis of Mental Illness. Neuroscience.

[116] Loeffler-Wirth H., Reikowski J., Hakobyan S., Wagner J., Binder H. (2020). oposSOM-Browser: An interactive tool to explore omics data landscapes in health science. BMC Bioinform..

[117] Angelova M., Charoentong P., Hackl H., Fischer M.L., Snajder R., Krogsdam A.M., Trajanoski Z. (2015). Characterization of the immunophenotypes and antigenomes of colorectal cancers reveals distinct tumor escape mechanisms and novel targets for immunotherapy. Genome Biol..

[118] Loeffler-Wirth H., Binder H., Willscher E., Gerber T., Kunz M. (2018). Pseudotime Dynamics in Melanoma Single-Cell Transcriptomes Reveals Different Mechanisms of Tumor Progression. Biology.

[119] Chen C., Cheng L., Grennan K., Pibiri F., Zhang C., Badner J.A., Members of the Bipolar Disorder Genome Study C. (2013). Two gene co-expression modules differentiate psychotics and controls. Mol. Psychiatry.

[120] Blalock E.M., Geddes J.W., Chen K.C., Porter N.M., Markesbery W.R., Landfield P.W. (2004). Incipient Alzheimer’s disease: Microarray correlation analyses reveal major transcriptional and tumor suppressor responses. Proc. Natl. Acad. Sci. USA.

[121] Zakharyan R., Boyajyan A. (2014). Inflammatory cytokine network in schizophrenia. World J. Biol Psychiatry.

[122] Zakharyan R., Khoyetsyan A., Arakelyan A., Boyajyan A., Gevorgyan A., Stahelova A., Petrek M. (2011). Association of C1QB gene polymorphism with schizophrenia in Armenian population. BMC Med. Genet..

[123] Zakharyan R., Petrek M., Arakelyan A., Mrazek F., Atshemyan S., Boyajyan A. (2012). Interleukin-6 promoter polymorphism and plasma levels in patients with schizophrenia. Tissue Antigens.

[124] Vasile C. (2020). Mental health and immunity (Review). Exp. Ther Med..

[125] Kany S., Janicova A., Relja B. (2019). Innate Immunity and Alcohol. J. Clin. Med..

[126] Margolis R.L., Chuang D.M., Post R.M. (1994). Programmed cell death: Implications for neuropsychiatric disorders. Biol Psychiatry.

[127] Santoft F., Hedman-Lagerlöf E., Salomonsson S., Lindsäter E., Ljótsson B., Kecklund G., Andreasson A. (2020). Inflammatory cytokines in patients with common mental disorders treated with cognitive behavior therapy. Brain Behav. Immun. Health.

[128] Crews F.T., Bechara R., Brown L.A., Guidot D.M., Mandrekar P., Oak S., Zou J. (2006). Cytokines and alcohol. Alcohol Clin. Exp. Res..

[129] Zakharyan R., Boyajyan A., Arakelyan A., Melkumova M., Mrazek F., Petrek M. (2012). Monocyte chemoattractant protein-1 in schizophrenia: -2518A/G genetic variant and protein levels in Armenian population. Cytokine.

