# Supplementary material for: The Transcriptome and Methylome of the Developing and Aging Brain and Their Relations to Gliomas and Psychological Disorders
Source: Cells. 2022 Jan 21;11(3):362. doi: 10.3390/cells11030362 (PMC8834030; doi:10.3390/cells11030362)
Supplement: Supplementary file 1 [file cells-11-00362-s001.zip › ReadMe.pdf]

ReadMe

Supplementary Table S1:

List of spot genes taken from the expression SOM. Each sheet refers to one spot providing the Affymetrix probe ID, the rank according to p-value, the gene symbol, the mean expression (log_10), standard deviation, the minimum and maximum expression along the age profile, the correlation coefficient of the gene-profile with the mean spot profile, the t-score (correlation statistics), p-value, position of the gene in the SOM (X x Y coordinate; X= 1...40; Y=1...40) and the full gene name

Supplementary Table S2:

List of spot genes taken from the methylation SOM. Each sheet refers to one spot providing the Affymetrix probe ID, the rank according to p-value, the gene symbol, the mean methylation (log M-value, log_10), standard deviation, the minimum and maximum methylation along the age profile, the correlation coefficient of the gene-profile with the mean spot profile, the t-score (correlation statistics), p-value, position of the gene in the SOM (X x Y coordinate; X= 1...40; Y=1...40) and the full gene name

Supplementary Material S3:

KEGG-pathway gene expression activation patterns: Each of the 51 KEGG pathways is considered in one PDF-report showing details about the pathway topology and pathway signal flow (PSF) activities calculated based on gene expression for each of the age strata. Red colored nodes refer to activated, blue nodes to deactivated nodes.

Supplementary Material S4:

KEGG-pathway methylation patterns: Each of the 51 KEGG pathways is considered in one PDF-report showing details about the pathway topology and pathway signal flow (PSF) calculated based on promoter methylation for each of the age strata. Red colored nodes refer to methylated, blue nodes to non-methylated nodes.
