# Supplementary material for: The Transcriptome and Methylome of the Developing and Aging Brain and Their Relations to Gliomas and Psychological Disorders
Source: Cells. 2022 Jan 21;11(3):362. doi: 10.3390/cells11030362 (PMC8834030; doi:10.3390/cells11030362)
Supplement: Supplementary file 1 [file cells-11-00362-s001.zip › Suppl-Material-S3-Pathways-PSF_Expression/0verview.html]

PSF Analysis Summary of Nov17\_Developmental\_Brain\_Human+ dataset


# General Information

Number of Pathways
:   51

- PSF signal Heatmaps (PDF)


# Pathways

Population maps of the node and of the sink genes, and profile plots
of the mean and maximum sink node signals are shown.
Additionally the signal flow in each sample is given for the pathways.

| Pathway | Report sheet |
| --- | --- |
| Adipocytokine\_signaling\_pathway | PDF |
| Adrenergic\_signaling\_in\_cardiomyocytes | PDF |
| AGE\_RAGE\_signaling\_pathway\_in\_diabetic\_complications | PDF |
| AMPK\_signaling\_pathway | PDF |
| Apelin\_signaling\_pathway | PDF |
| B\_cell\_receptor\_signaling\_pathway | PDF |
| C\_type\_lectin\_receptor\_signaling\_pathway | PDF |
| Calcium\_signaling\_pathway | PDF |
| cAMP\_signaling\_pathway | PDF |
| cGMP\_PKG\_signaling\_pathway | PDF |
| Chemokine\_signaling\_pathway | PDF |
| Epithelial\_cell\_signaling\_in\_Helicobacter\_pylori\_infection | PDF |
| ErbB\_signaling\_pathway | PDF |
| Estrogen\_signaling\_pathway | PDF |
| Fc\_epsilon\_RI\_signaling\_pathway | PDF |
| FoxO\_signaling\_pathway | PDF |
| Glucagon\_signaling\_pathway | PDF |
| GnRH\_signaling\_pathway | PDF |
| Hedgehog\_signaling\_pathway | PDF |
| HIF\_1\_signaling\_pathway | PDF |
| Hippo\_signaling\_pathway | PDF |
| Hippo\_signaling\_pathway\_multiple\_species | PDF |
| IL\_17\_signaling\_pathway | PDF |
| Insulin\_signaling\_pathway | PDF |
| JAK\_STAT\_signaling\_pathway | PDF |
| MAPK\_signaling\_pathway | PDF |
| mTOR\_signaling\_pathway | PDF |
| Neurotrophin\_signaling\_pathway | PDF |
| NF\_kappa\_B\_signaling\_pathway | PDF |
| NOD\_like\_receptor\_signaling\_pathway | PDF |
| Notch\_signaling\_pathway | PDF |
| Oxytocin\_signaling\_pathway | PDF |
| p53\_signaling\_pathway | PDF |
| Phosphatidylinositol\_signaling\_system | PDF |
| Phospholipase\_D\_signaling\_pathway | PDF |
| PI3K\_Akt\_signaling\_pathway | PDF |
| PPAR\_signaling\_pathway | PDF |
| Prolactin\_signaling\_pathway | PDF |
| Rap1\_signaling\_pathway | PDF |
| Ras\_signaling\_pathway | PDF |
| Relaxin\_signaling\_pathway | PDF |
| Retrograde\_endocannabinoid\_signaling | PDF |
| RIG\_I\_like\_receptor\_signaling\_pathway | PDF |
| Sphingolipid\_signaling\_pathway | PDF |
| T\_cell\_receptor\_signaling\_pathway | PDF |
| TGF\_beta\_signaling\_pathway | PDF |
| Thyroid\_hormone\_signaling\_pathway | PDF |
| TNF\_signaling\_pathway | PDF |
| Toll\_like\_receptor\_signaling\_pathway | PDF |
| VEGF\_signaling\_pathway | PDF |
| Wnt\_signaling\_pathway | PDF |
