# Supplementary material for: The Transcriptome and Methylome of the Developing and Aging Brain and Their Relations to Gliomas and Psychological Disorders
Source: Cells. 2022 Jan 21;11(3):362. doi: 10.3390/cells11030362 (PMC8834030; doi:10.3390/cells11030362)
Supplement: Supplementary file 1 [file cells-11-00362-s001.zip › Suppl-Material-S3-Pathways-PSF_Expression/AGE_RAGE_signaling_pathway_in_diabetic_complications.pdf]

AGE−RAGE signaling pathway in dia

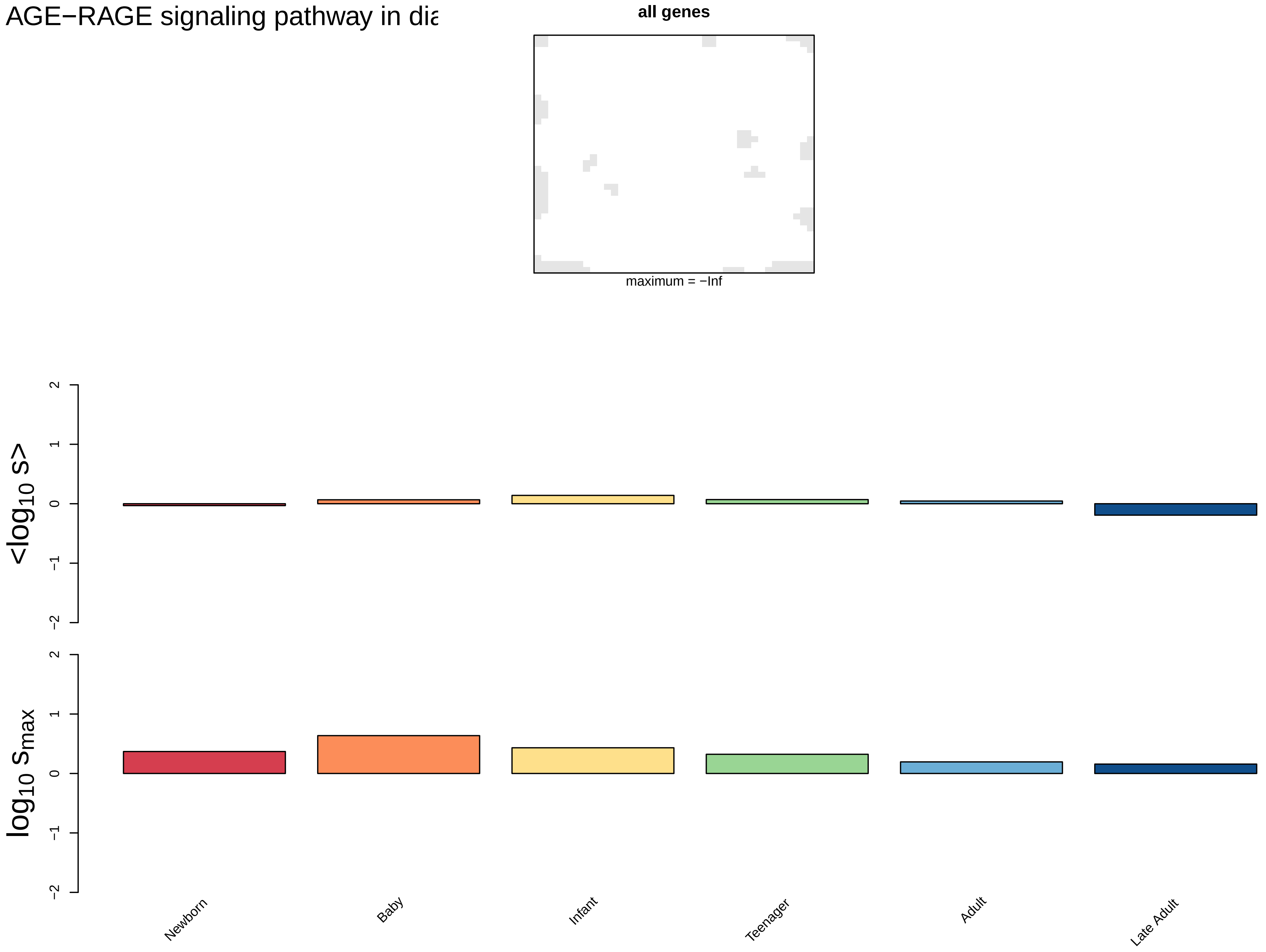

AGE_RAGE_signaling_pathway_in_diabetic_complications
genes with data

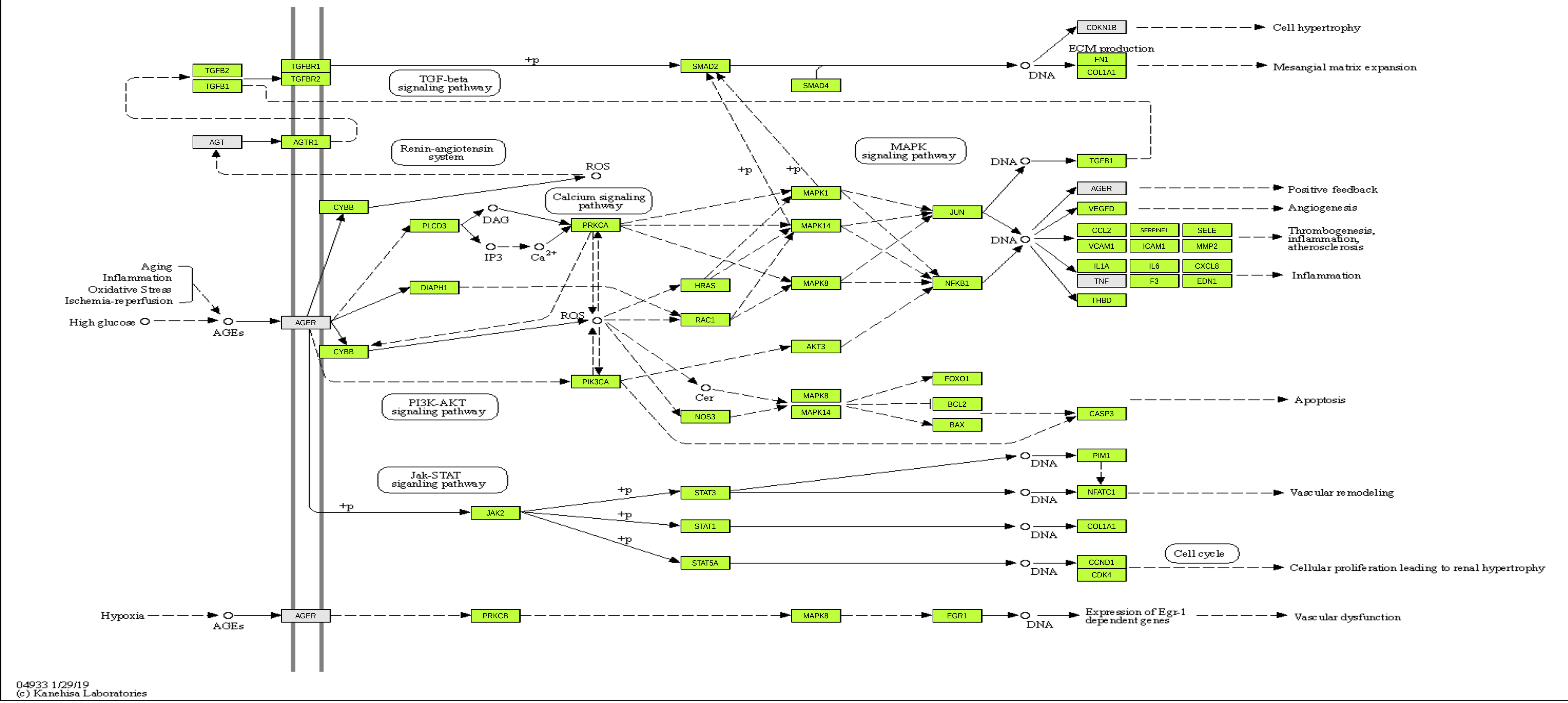

AGE_RAGE_signaling_pathway_in_diabetic_complications
sink nodes

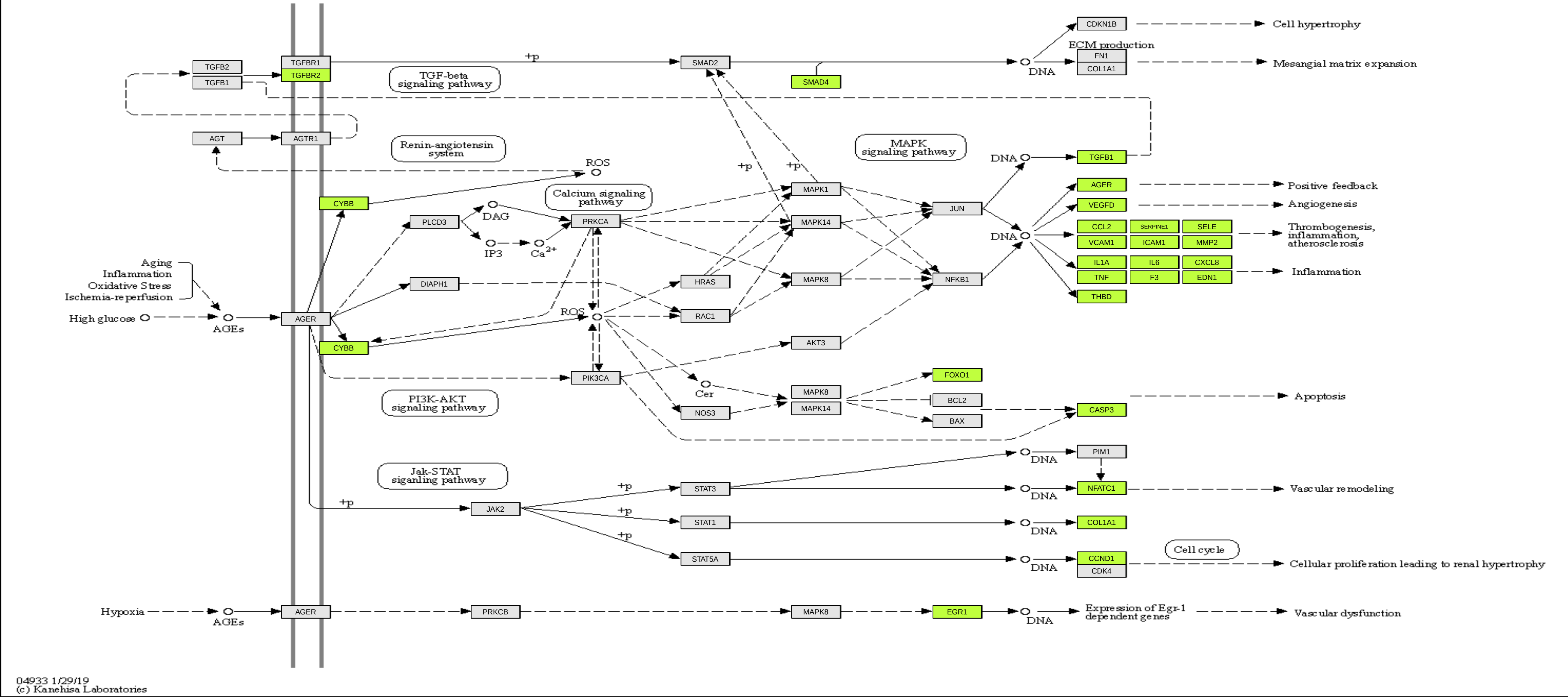

AGE_RAGE_signaling_pathway_in_diabetic_complications
Newborn

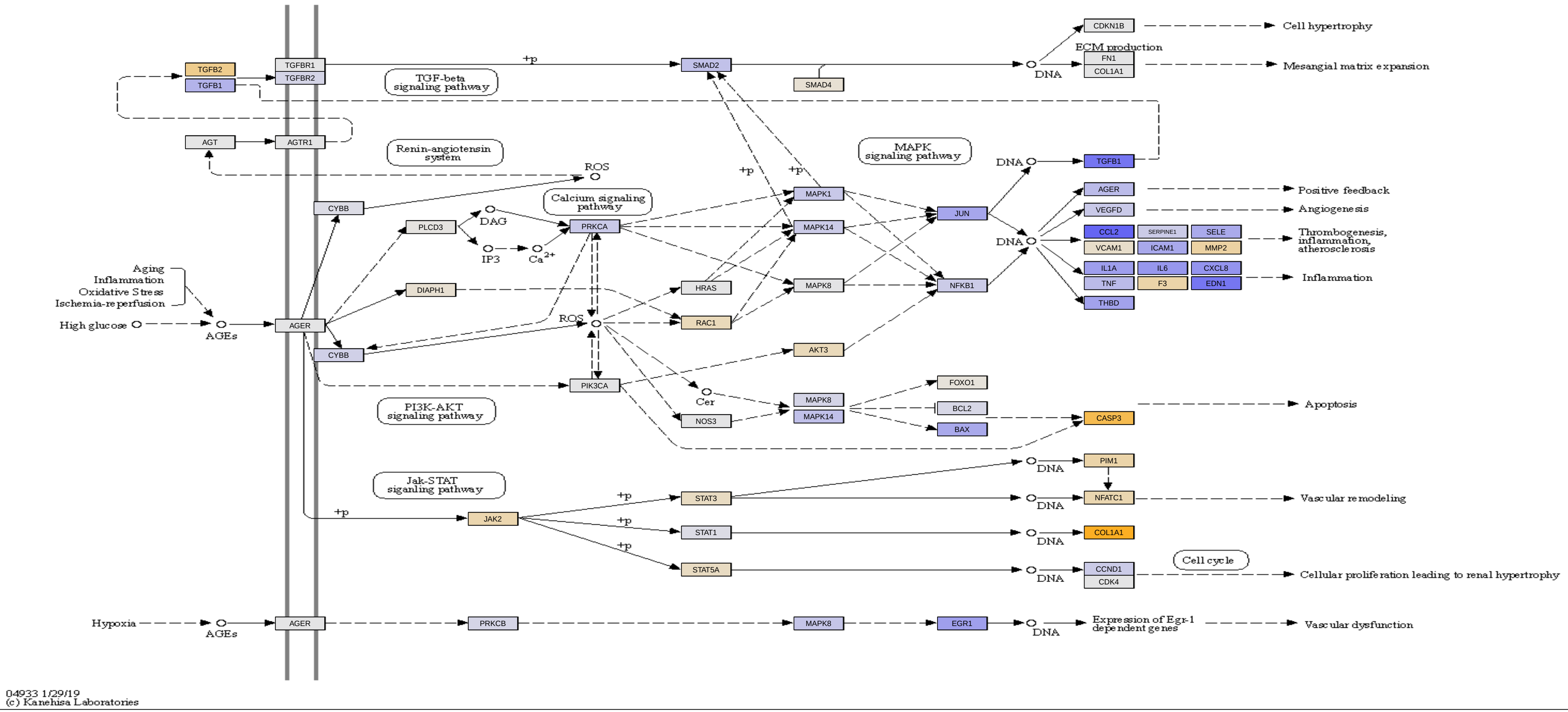

AGE_RAGE_signaling_pathway_in_diabetic_complications
Baby

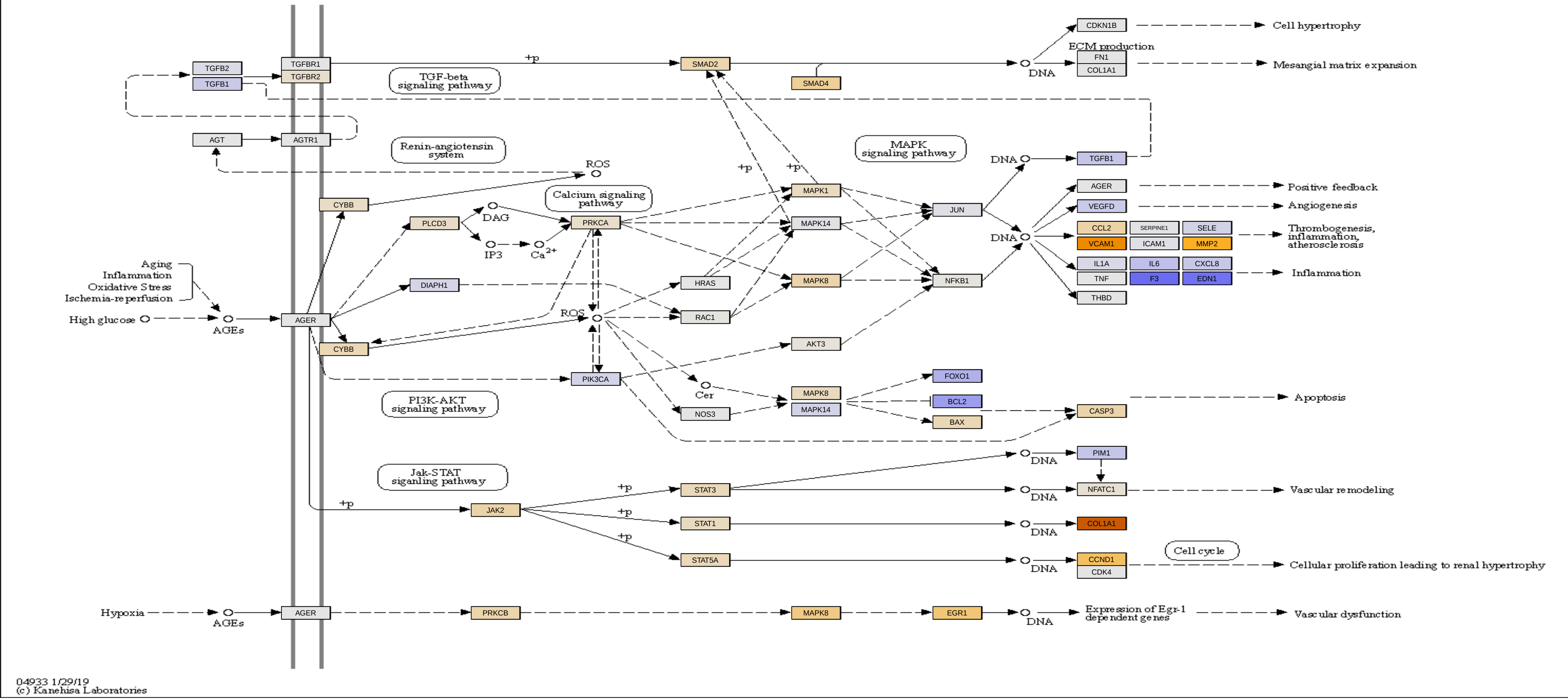

AGE_RAGE_signaling_pathway_in_diabetic_complications
Infant

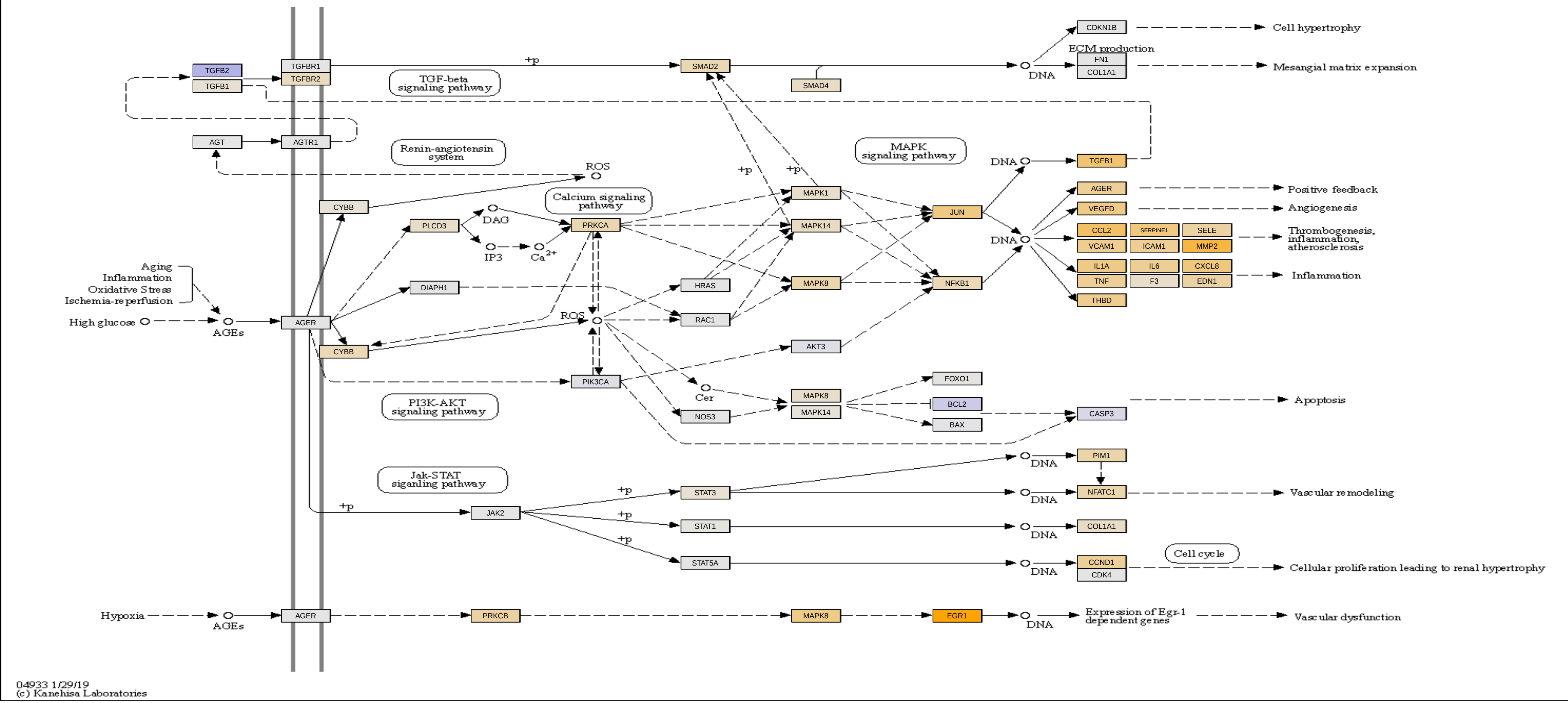

AGE_RAGE_signaling_pathway_in_diabetic_complications
Teenager

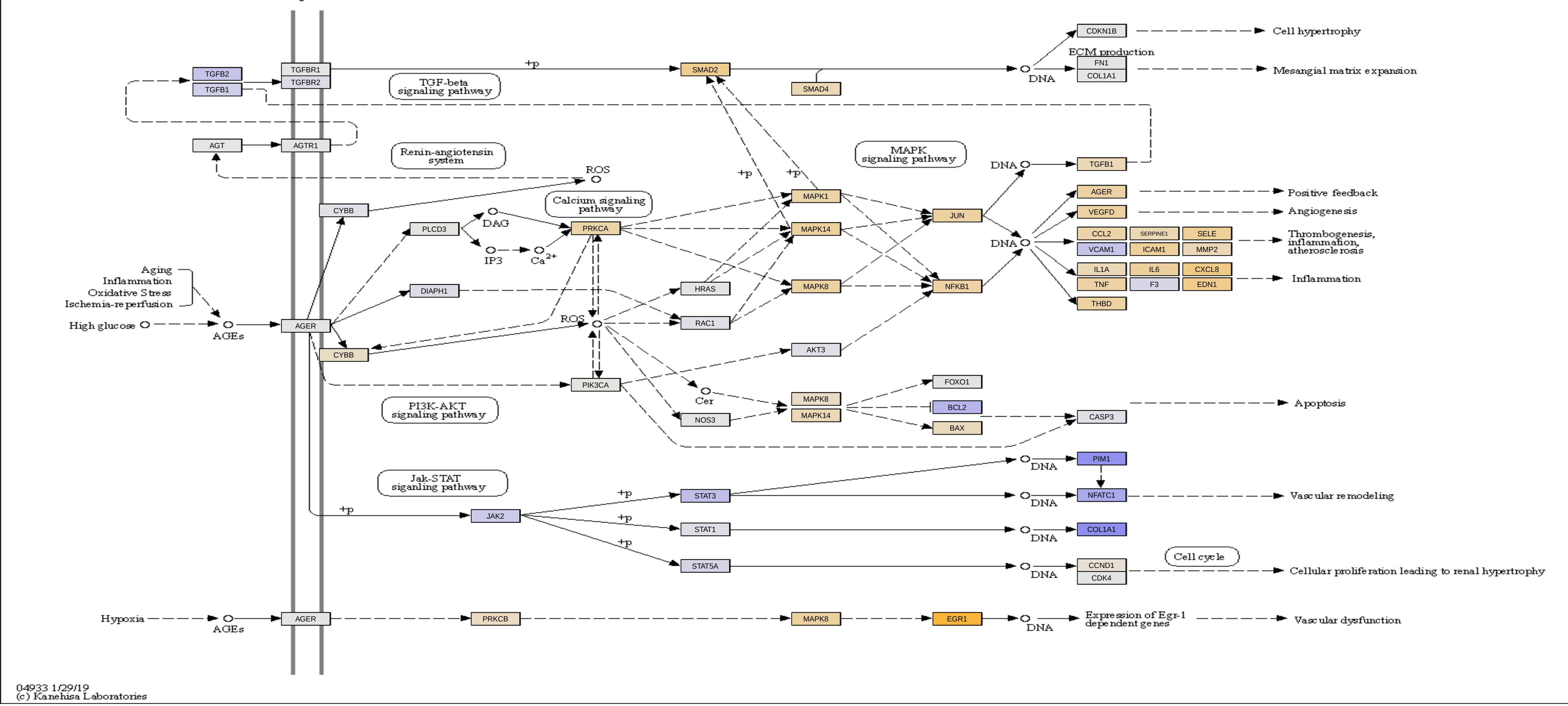

AGE_RAGE_signaling_pathway_in_diabetic_complications
Adult

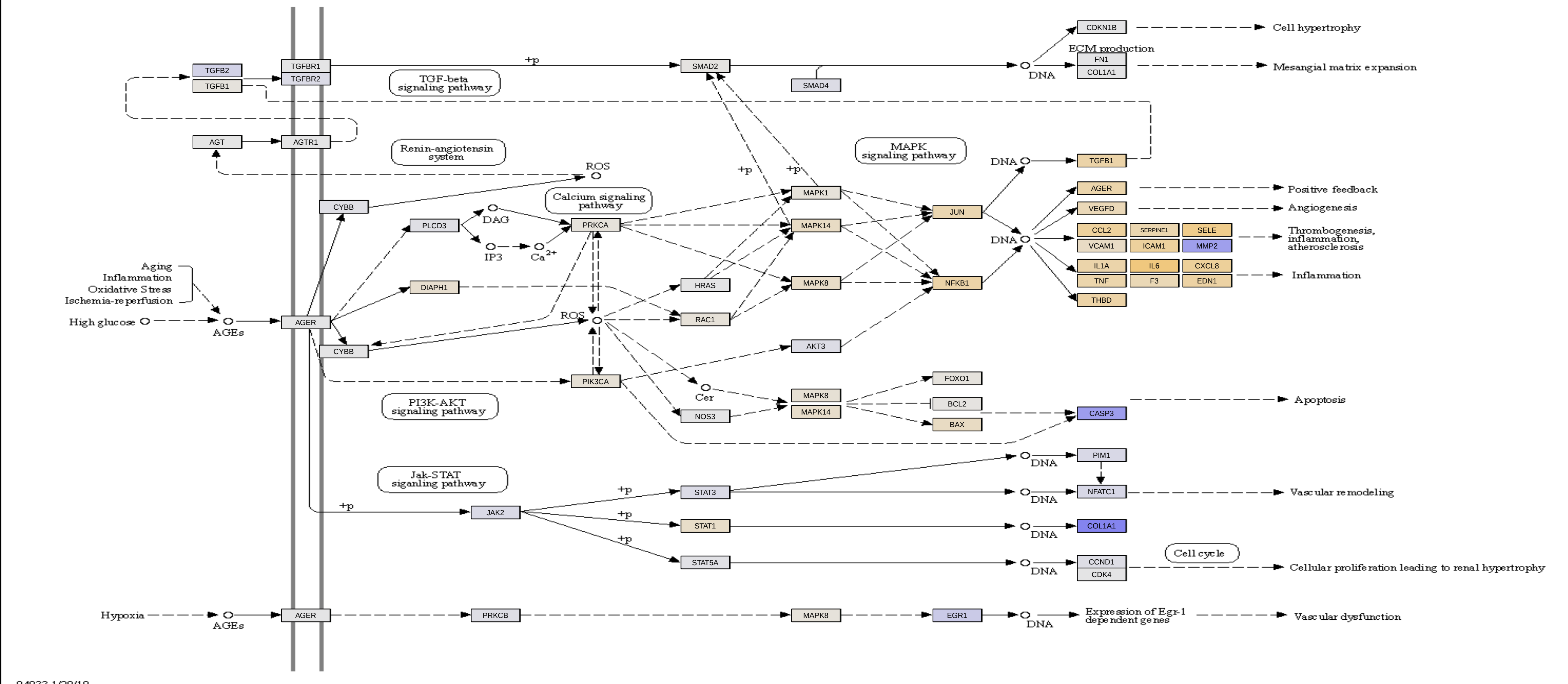

04933 1/29/19
(c) Kanehisa Laboratories

AGE_RAGE_signaling_pathway_in_diabetic_complications
Late Adult

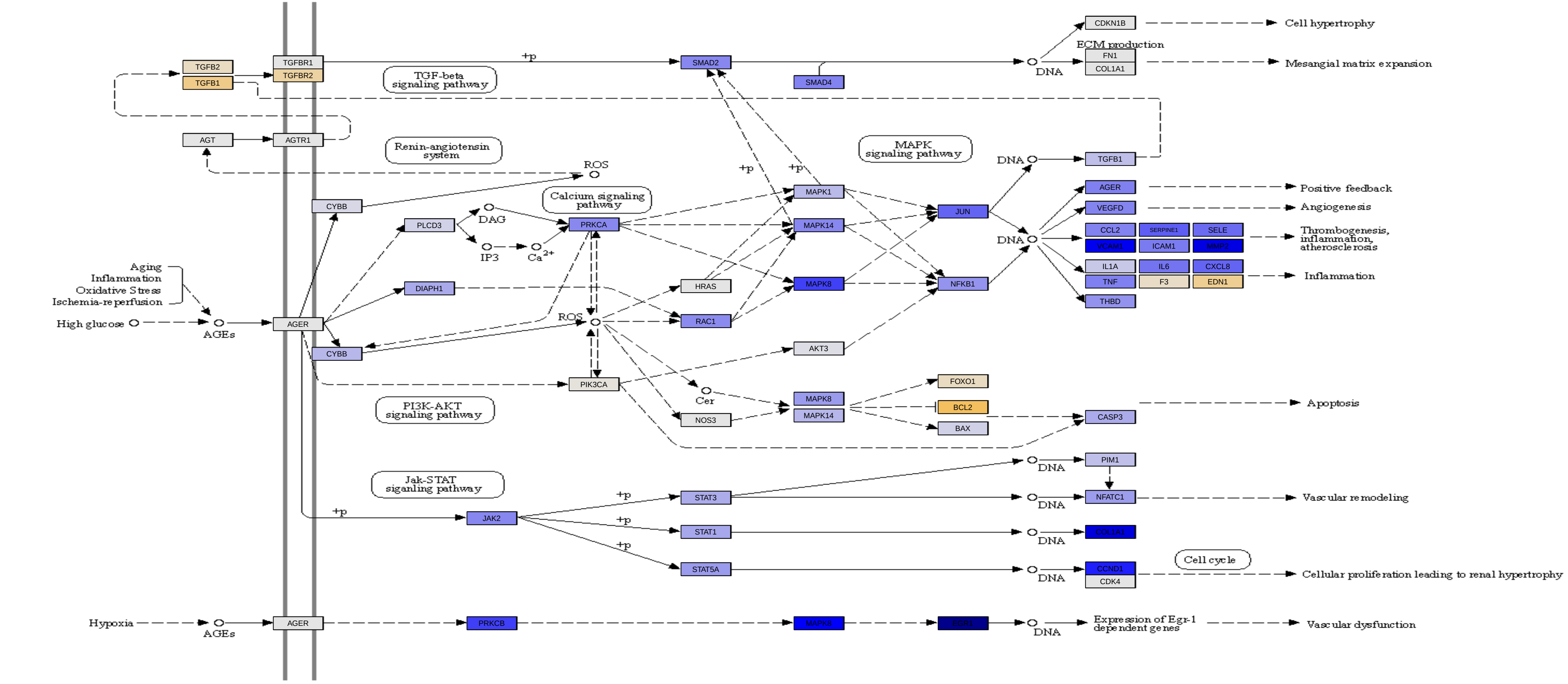

04933 1/29/19
(c) Kanehisa Laboratories
