# Supplementary material for: The Transcriptome and Methylome of the Developing and Aging Brain and Their Relations to Gliomas and Psychological Disorders
Source: Cells. 2022 Jan 21;11(3):362. doi: 10.3390/cells11030362 (PMC8834030; doi:10.3390/cells11030362)
Supplement: Supplementary file 1 [file cells-11-00362-s001.zip › Suppl-Material-S3-Pathways-PSF_Expression/AMPK_signaling_pathway.pdf]

AMPK signaling pathway

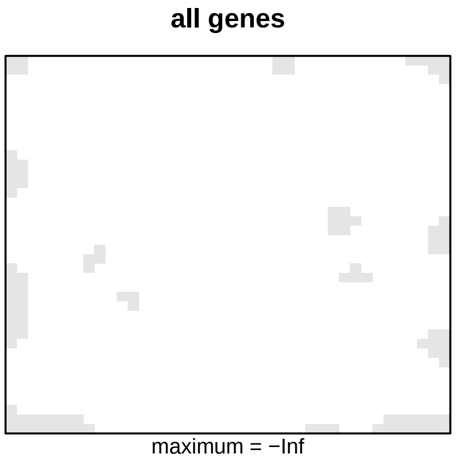

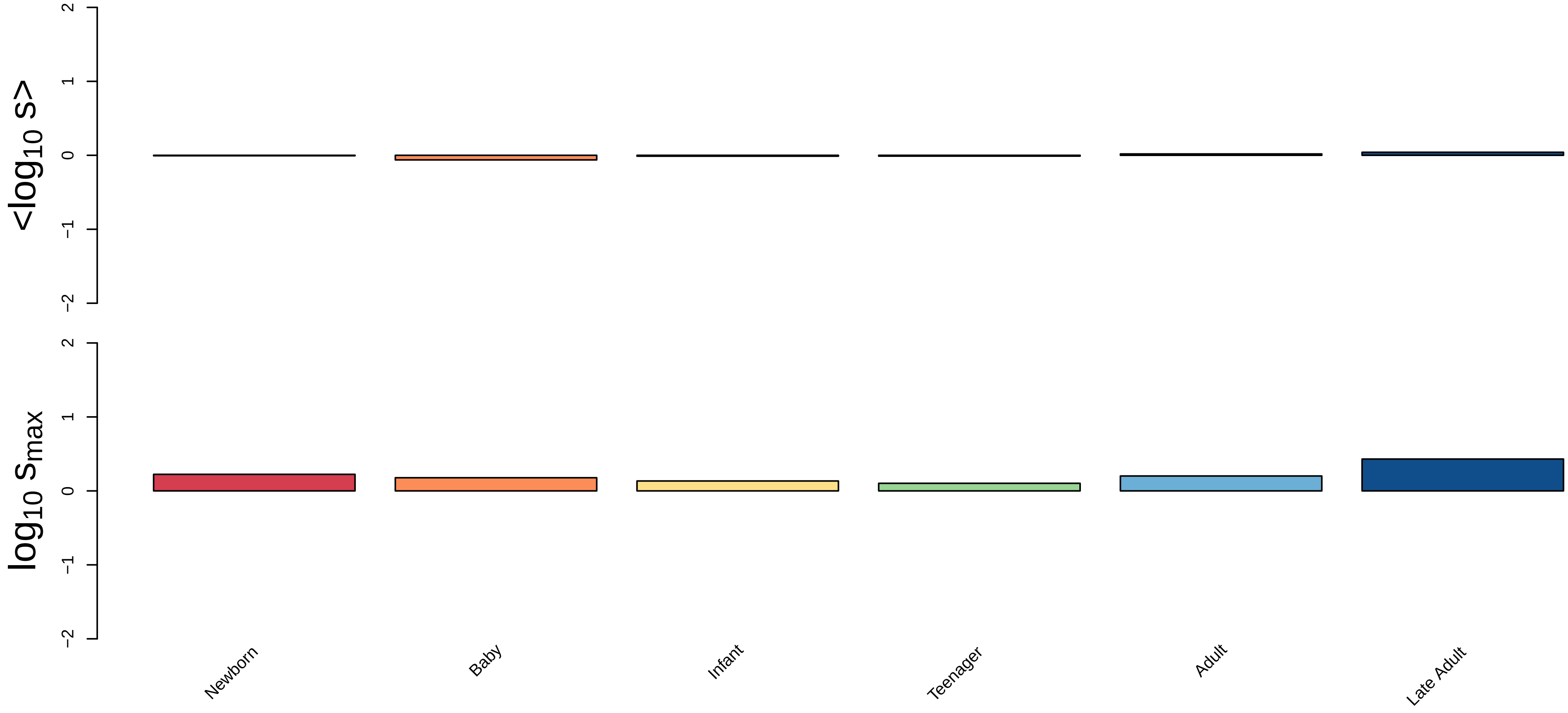

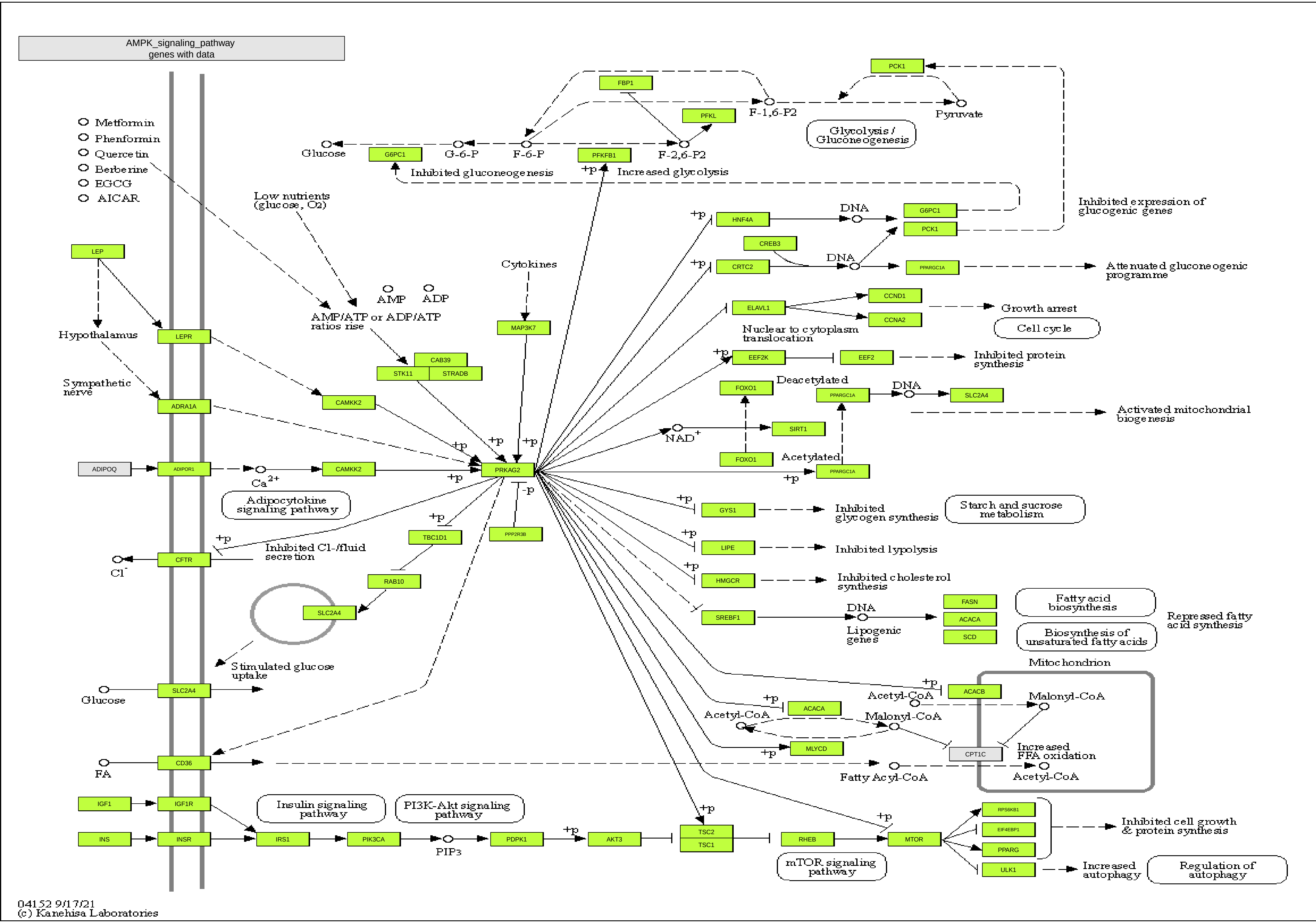
AMPK_signaling_pathway
genes with data
Metformin
Phenformin
Quercetin
Berberine
EGCG
AICAR
LEP
Hypothalamus
LEPR
Sympathetic nerve
ADRA1A
ADIPOQ
ADIPOR1
Ca2+
CAMKK2
Adipocytokine signaling pathway
CFTR
Cl-
Inhibited Cl-/fluid secretion
TBC1D1
RAB10
SLC2A4
Stimulated glucose uptake
Glucose
CD36
FA
IGF1
IGF1R
INS
INSR
IRS1
PIK3CA
PIP3
PDPK1
AKT3
Insulin signaling pathway
PI3K-Akt signaling pathway
Low nutrients (glucose, O2)
AMP
ADP
AMP/ATP or ADP/ATP ratios rise
CAB39
STK11
STRADB
CAMKK2
Cytokines
MAP3K7
PRKAG2
PPP2R3B
+p
-p
Glucose
G6PC1
G-6-P
F-6-P
FBP1
PFKFB1
F-2,6-P2
PFKL
F-1,6-P2
Pyruvate
PCK1
Glycolysis / Gluconeogenesis
Inhibited gluconeogenesis
Increased glycolysis
HNF4A
DNA
G6PC1
PCK1
Inhibited expression of glucogenic genes
CREB3
CRTC2
PPARGC1A
Attenuated gluconeogenic programme
ELAVL1
CCND1
CCNA2
Growth arrest
Cell cycle
Nuclear to cytoplasm translocation
EEF2K
EEF2
Inhibited protein synthesis
FOXO1
Deacetylated
PPARGC1A
SLC2A4
Activated mitochondrial biogenesis
NAD+
SIRT1
FOXO1
Acetylated
PPARGC1A
GYS1
Inhibited glycogen synthesis
Starch and sucrose metabolism
LIPE
Inhibited lypolysis
HMGCR
Inhibited cholesterol synthesis
SREBF1
Lipogenic genes
FASN
ACACA
SCD
Fatty acid biosynthesis
Biosynthesis of unsaturated fatty acids
Repressed fatty acid synthesis
Mitochondrion
ACACB
Acetyl-CoA
Malonyl-CoA
ACACA
MLYCD
CPT1C
Increased FFA oxidation
Fatty Acyl-CoA
TSC2
TSC1
RHEB
MTOR
mTOR signaling pathway
RPS6KB1
EIF4EBP1
PPARG
ULK1
Inhibited cell growth & protein synthesis
Increased autophagy
Regulation of autophagy
04152 9/17/21
(c) Kanehisa Laboratories

AMPK_signaling_pathway
sink nodes

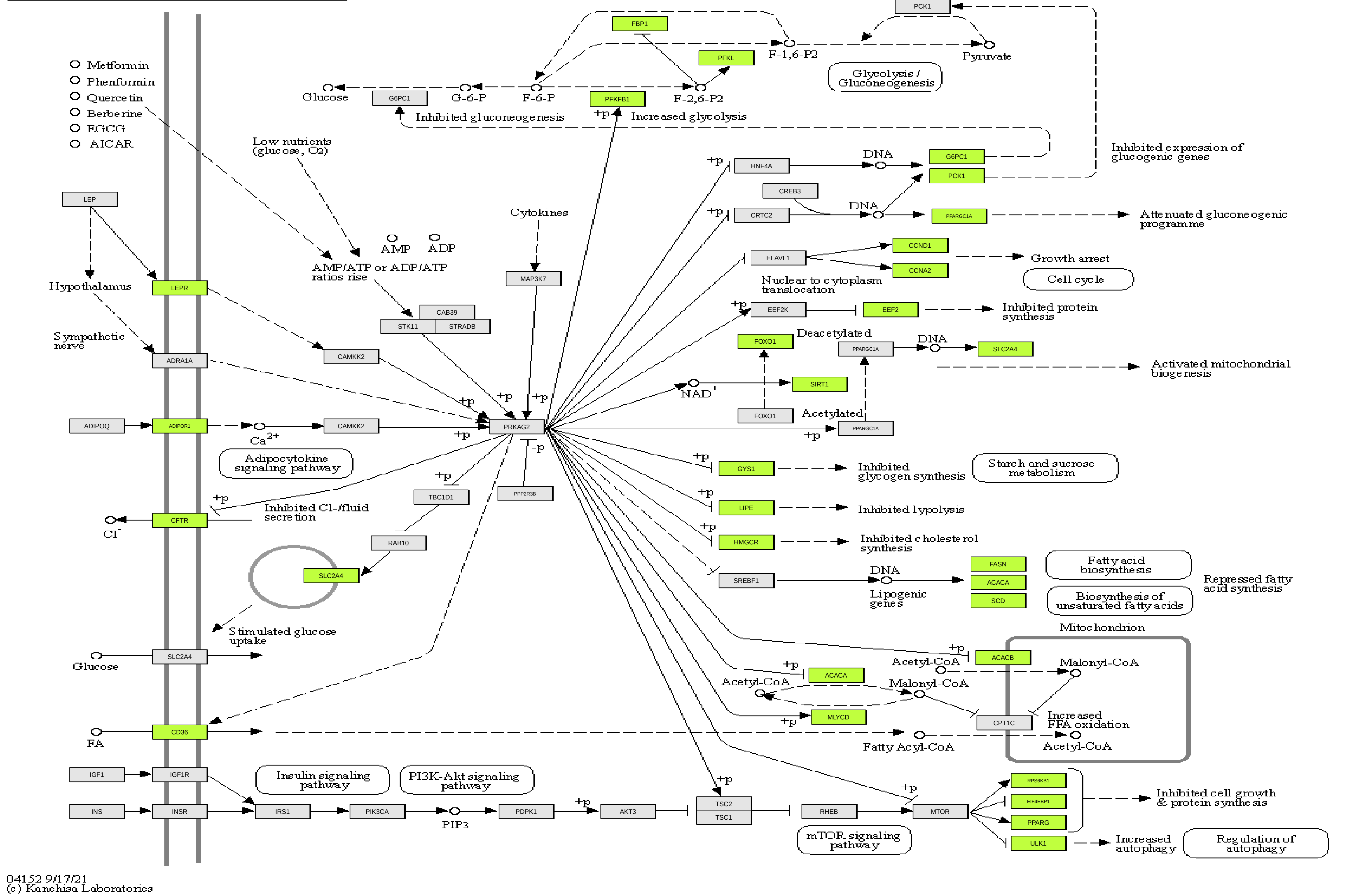

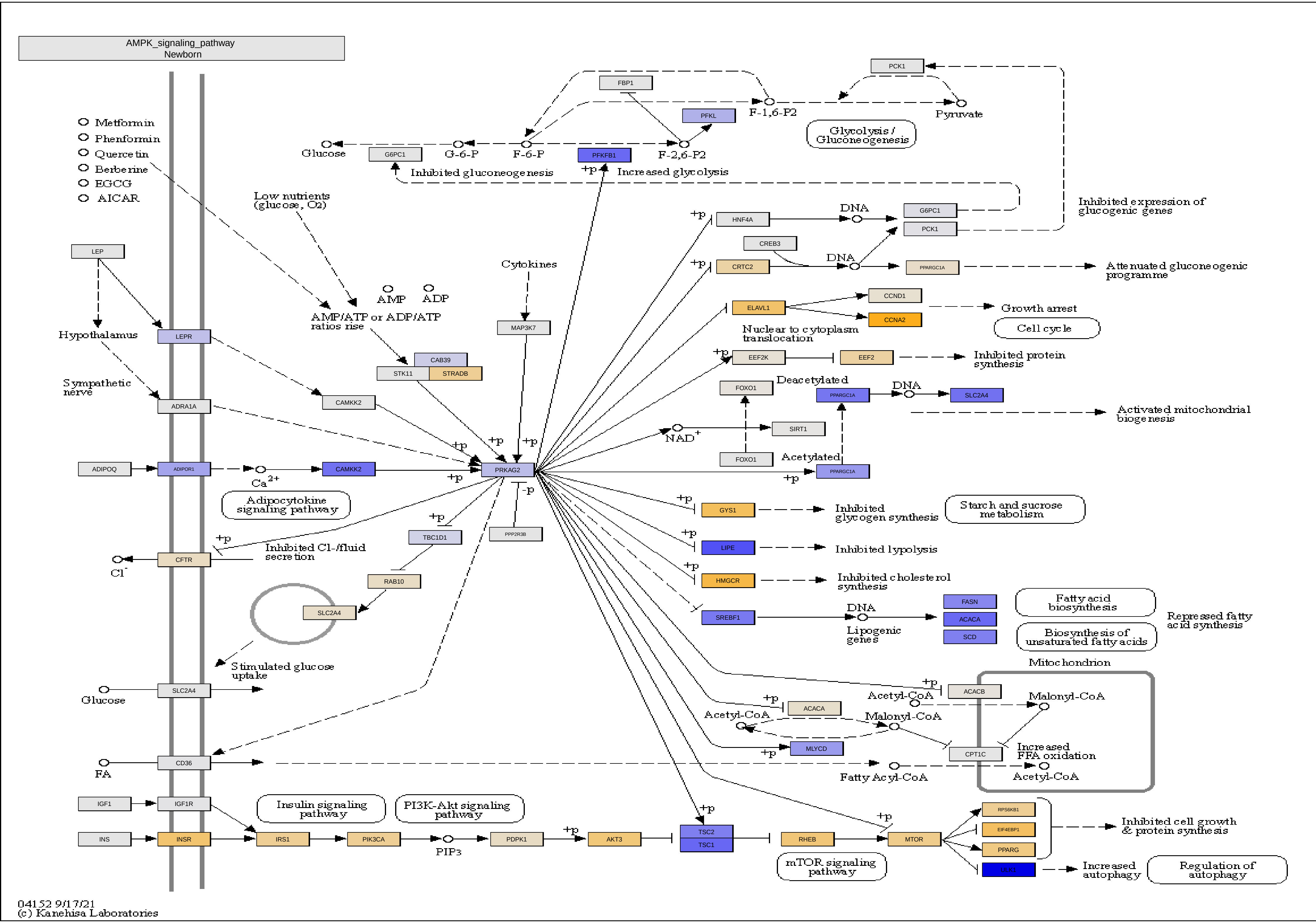
AMPK_signaling_pathway
Newborn
Metformin
Phenformin
Quercetin
Berberine
EGCG
AICAR
Low nutrients (glucose, O2)
AMP
ADP
AMP/ATP or ADP/ATP ratios rise
Cytokines
LEP
LEPR
Hypothalamus
Sympathetic nerve
ADRA1A
CAMKK2
CAB39
STK11
STRADB
MAP3K7
ADIPOQ
ADIPOR1
Ca2+
CAMKK2
PRKAG2
Adipocytokine signaling pathway
+p
-p
PPP2R3B
TBC1D1
RAB10
SLC2A4
CFTR
Cl-
Inhibited Cl-/fluid secretion
Stimulated glucose uptake
Glucose
SLC2A4
FA
CD36
IGF1
IGF1R
INS
INSR
IRS1
PIK3CA
PIP3
PDPK1
AKT3
TSC2
TSC1
RHEB
MTOR
Insulin signaling pathway
PI3K-Akt signaling pathway
mTOR signaling pathway
RPS6KB1
EIF4EBP1
PPARG
ULK1
Inhibited cell growth & protein synthesis
Increased autophagy
Regulation of autophagy
FBP1
PFKL
PCK1
Glucose
G6PC1
G-6-P
F-6-P
PFKFB1
F-2,6-P2
F-1,6-P2
Pyruvate
Glycolysis / Gluconeogenesis
Inhibited gluconeogenesis
Increased glycolysis
HNF4A
DNA
G6PC1
PCK1
Inhibited expression of glucogenic genes
CREB3
CRTC2
PPARGC1A
Attenuated gluconeogenic programme
ELAVL1
CCND1
CCNA2
Growth arrest
Cell cycle
Nuclear to cytoplasm translocation
EEF2K
EEF2
Inhibited protein synthesis
FOXO1
Deacetylated
PPARGC1A
SLC2A4
Activated mitochondrial biogenesis
NAD+
SIRT1
Acetylated
GYS1
Inhibited glycogen synthesis
Starch and sucrose metabolism
LIPE
Inhibited lypolysis
HMGCR
Inhibited cholesterol synthesis
SREBF1
Lipogenic genes
FASN
ACACA
SCD
Fatty acid biosynthesis
Biosynthesis of unsaturated fatty acids
Repressed fatty acid synthesis
Mitochondrion
ACACB
Acetyl-CoA
Malonyl-CoA
ACACA
MLYCD
CPT1C
Increased FFA oxidation
Fatty Acyl-CoA
04152 9/17/21
(c) Kanehisa Laboratories

AMPK_signaling_pathway
Baby

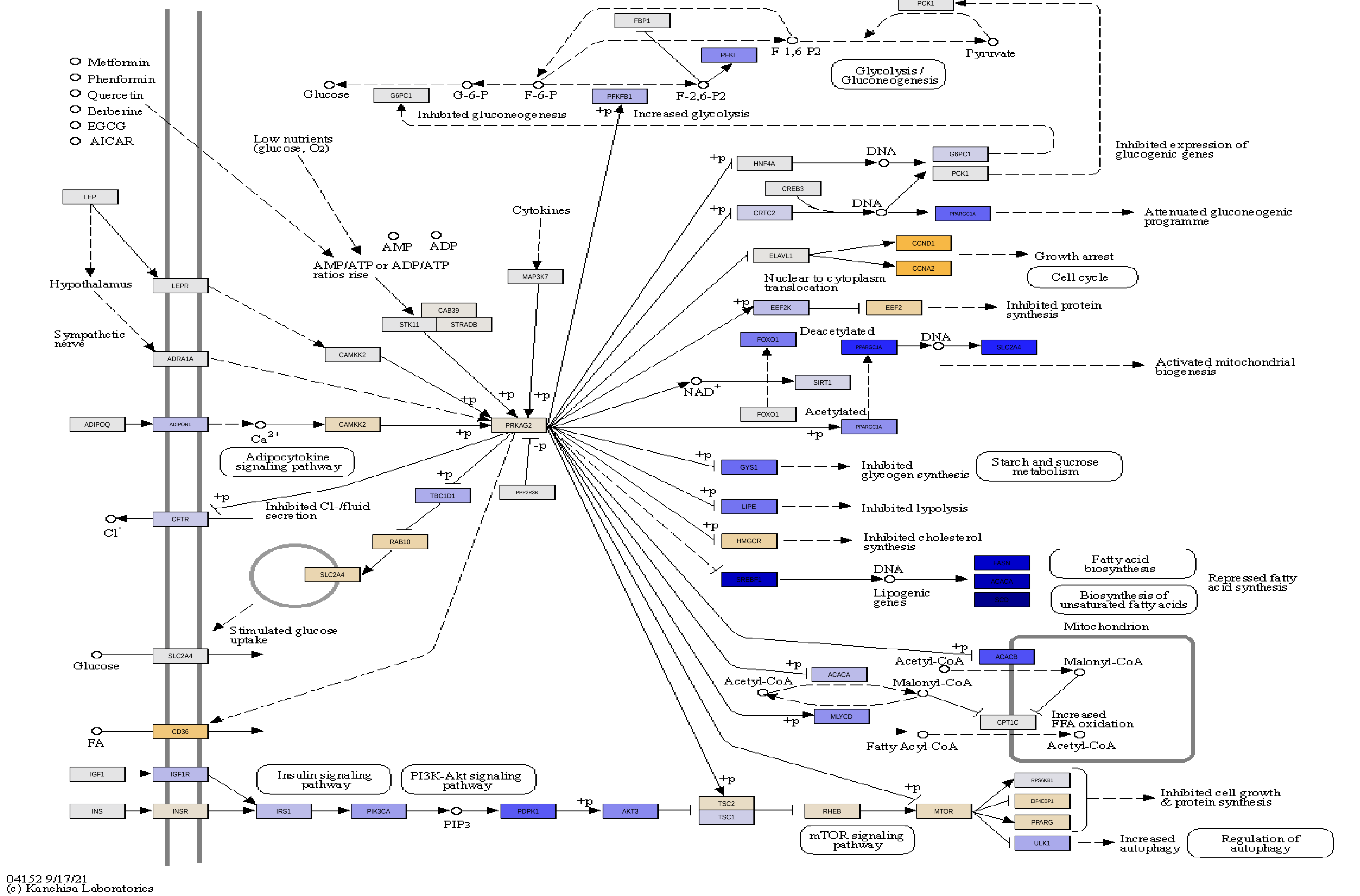

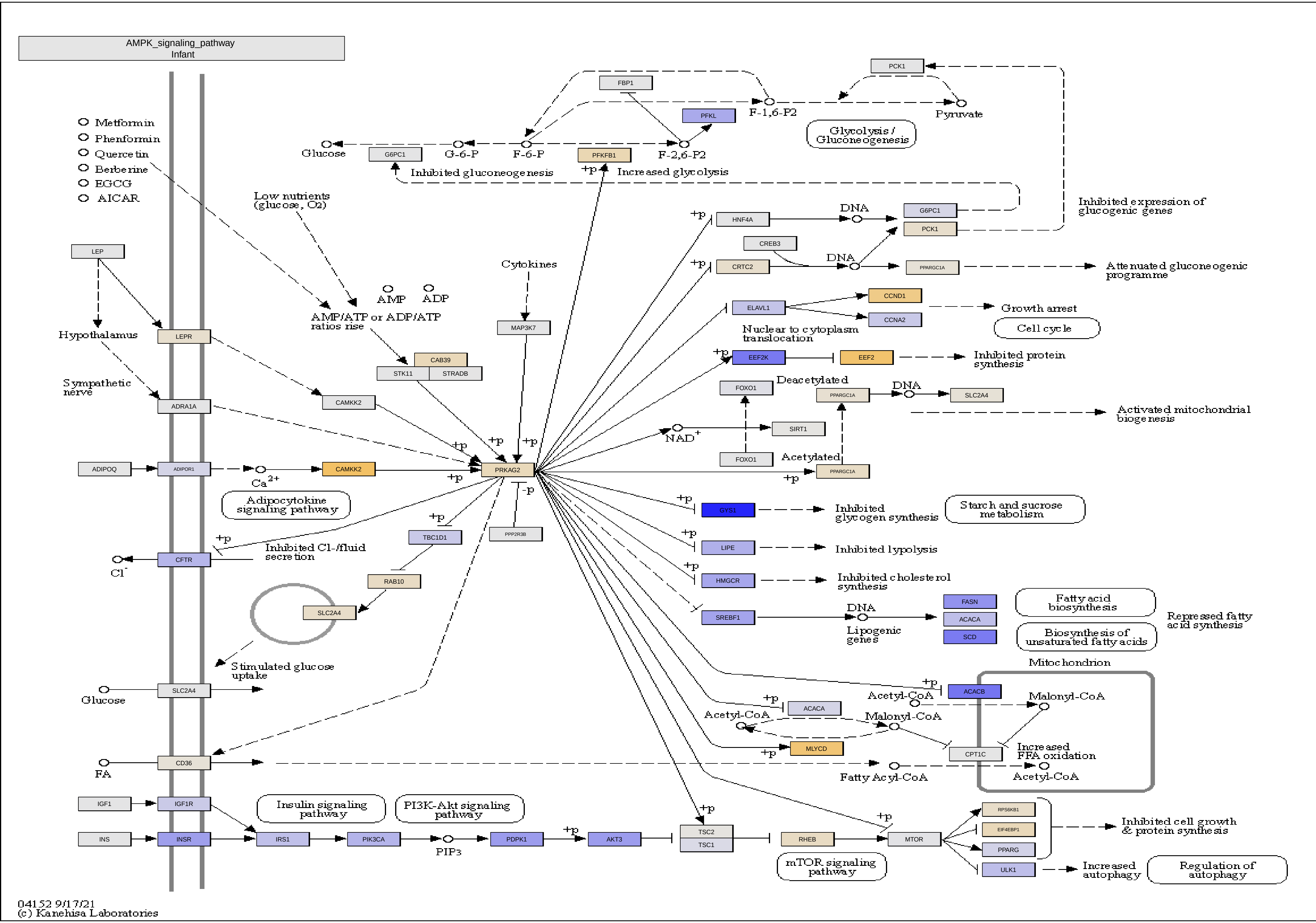
AMPK_signaling_pathway
Infant
Metformin
Phenformin
Quercetin
Berberine
EGCG
AICAR
Low nutrients
(glucose, O2)
AMP
ADP
AMP/ATP or ADP/ATP
ratios rise
Cytokines
LEP
Hypothalamus
LEPR
Sympathetic
nerve
ADRA1A
ADIPOQ
ADIPOR1
Ca2+
CAMKK2
CAMKK2
CAB39
STK11
STRADB
MAP3K7
PRKAG2
+p
-p
PPP2R3B
Adipocytokine
signaling pathway
CFTR
Cl-
Inhibited Cl-/fluid
secretion
TBC1D1
RAB10
SLC2A4
Stimulated glucose
uptake
SLC2A4
Glucose
CD36
FA
IGF1
IGF1R
INS
INSR
IRS1
PIK3CA
PIP3
PDPK1
AKT3
TSC2
TSC1
RHEB
MTOR
Insulin signaling
pathway
PI3K-Akt signaling
pathway
mTOR signaling
pathway
Glucose
G6PC1
G-6-P
F-6-P
PFKFB1
F-2,6-P2
FBP1
PFKL
F-1,6-P2
PCK1
Pyruvate
Glycolysis /
Gluconeogenesis
Inhibited gluconeogenesis
Increased glycolysis
HNF4A
DNA
G6PC1
PCK1
Inhibited expression of
glucogenic genes
CREB3
CRTC2
DNA
PPARGC1A
Attenuated gluconeogenic
programme
ELAVL1
CCND1
CCNA2
Growth arrest
Cell cycle
Nuclear to cytoplasm
translocation
EEF2K
EEF2
Inhibited protein
synthesis
FOXO1
Deacetylated
PPARGC1A
DNA
SLC2A4
Activated mitochondrial
biogenesis
NAD+
SIRT1
FOXO1
Acetylated
PPARGC1A
GYS1
Inhibited
glycogen synthesis
Starch and sucrose
metabolism
LIPE
Inhibited lypolysis
HMGCR
Inhibited cholesterol
synthesis
SREBF1
DNA
Lipogenic
genes
FASN
ACACA
SCD
Fatty acid
biosynthesis
Biosynthesis of
unsaturated fatty acids
Repressed fatty
acid synthesis
Mitochondrion
ACACB
Acetyl-CoA
Malonyl-CoA
ACACA
Acetyl-CoA
Malonyl-CoA
MLYCD
CPT1C
Increased
FFA oxidation
Acetyl-CoA
Fatty Acyl-CoA
RPS6KB1
EIF4EBP1
PPARG
ULK1
Inhibited cell growth
& protein synthesis
Increased
autophagy
Regulation of
autophagy
04152 9/17/21
(c) Kanehisa Laboratories

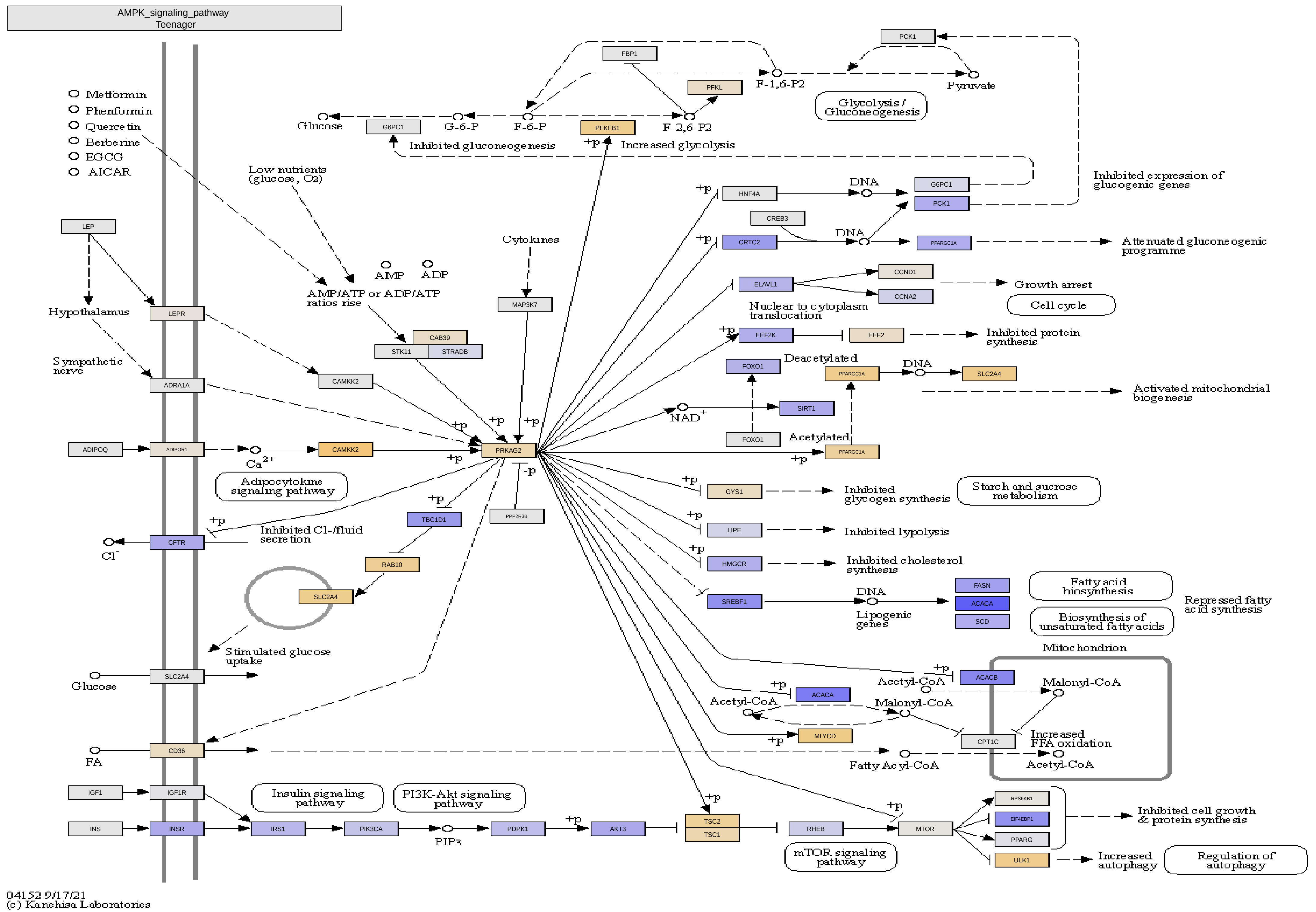

AMPK_signaling_pathway
Teenager
Metformin
Phenformin
Quercetin
Berberine
EGCG
AICAR
Low nutrients (glucose, O2)
AMP
ADP
AMP/ATP or ADP/ATP ratios rise
Cytokines
LEP
LEPR
Hypothalamus
Sympathetic nerve
ADRA1A
CAMKK2
CAB39
STK11
STRADB
MAP3K7
ADIPOQ
ADIPOR1
Ca2+
CAMKK2
PRKAG2
Adipocytokine signaling pathway
PPP2R3B
CFTR
Cl-
Inhibited Cl-/fluid secretion
TBC1D1
RAB10
SLC2A4
Stimulated glucose uptake
SLC2A4
Glucose
CD36
FA
IGF1
IGF1R
INS
INSR
IRS1
PIK3CA
PIP3
PDPK1
AKT3
TSC2
TSC1
RHEB
MTOR
Insulin signaling pathway
PI3K-Akt signaling pathway
mTOR signaling pathway
RPS6KB1
EIF4EBP1
PPARG
ULK1
Inhibited cell growth & protein synthesis
Increased autophagy
Regulation of autophagy
FBP1
PCK1
F-1,6-P2
Pyruvate
Glycolysis / Gluconeogenesis
PFKL
Glucose
G6PC1
G-6-P
F-6-P
PFKFB1
F-2,6-P2
Inhibited gluconeogenesis
Increased glycolysis
HNF4A
DNA
G6PC1
PCK1
Inhibited expression of glucogenic genes
CREB3
CRTC2
DNA
PPARGC1A
Attenuated gluconeogenic programme
ELAVL1
CCND1
CCNA2
Growth arrest
Cell cycle
Nuclear to cytoplasm translocation
EEF2K
EEF2
Inhibited protein synthesis
FOXO1
Deacetylated
PPARGC1A
DNA
SLC2A4
Activated mitochondrial biogenesis
NAD+
SIRT1
FOXO1
Acetylated
PPARGC1A
GYS1
Inhibited glycogen synthesis
Starch and sucrose metabolism
LIPE
Inhibited lypolysis
HMGCR
Inhibited cholesterol synthesis
SREBF1
DNA
Lipogenic genes
FASN
ACACA
SCD
Fatty acid biosynthesis
Biosynthesis of unsaturated fatty acids
Repressed fatty acid synthesis
Mitochondrion
ACACB
Acetyl-CoA
Malonyl-CoA
ACACA
Acetyl-CoA
Malonyl-CoA
MLYCD
CPT1C
Increased FFA oxidation
Fatty Acyl-CoA
Acetyl-CoA
+p
-p
04152 9/17/21
(c) Kanehisa Laboratories

AMPK_signaling_pathway
Adult

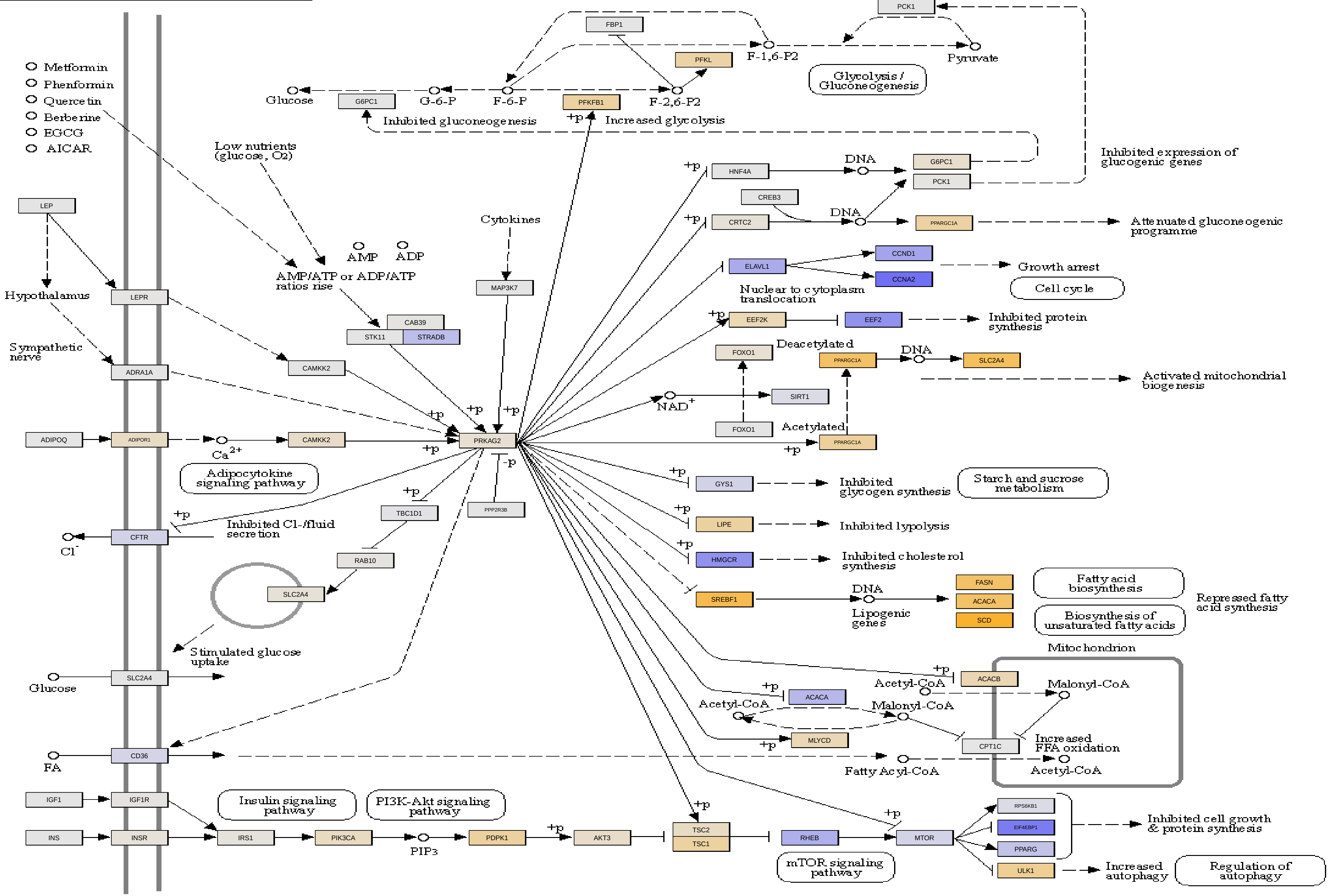

04152 9/17/21
(c) Kanehisa Laboratories

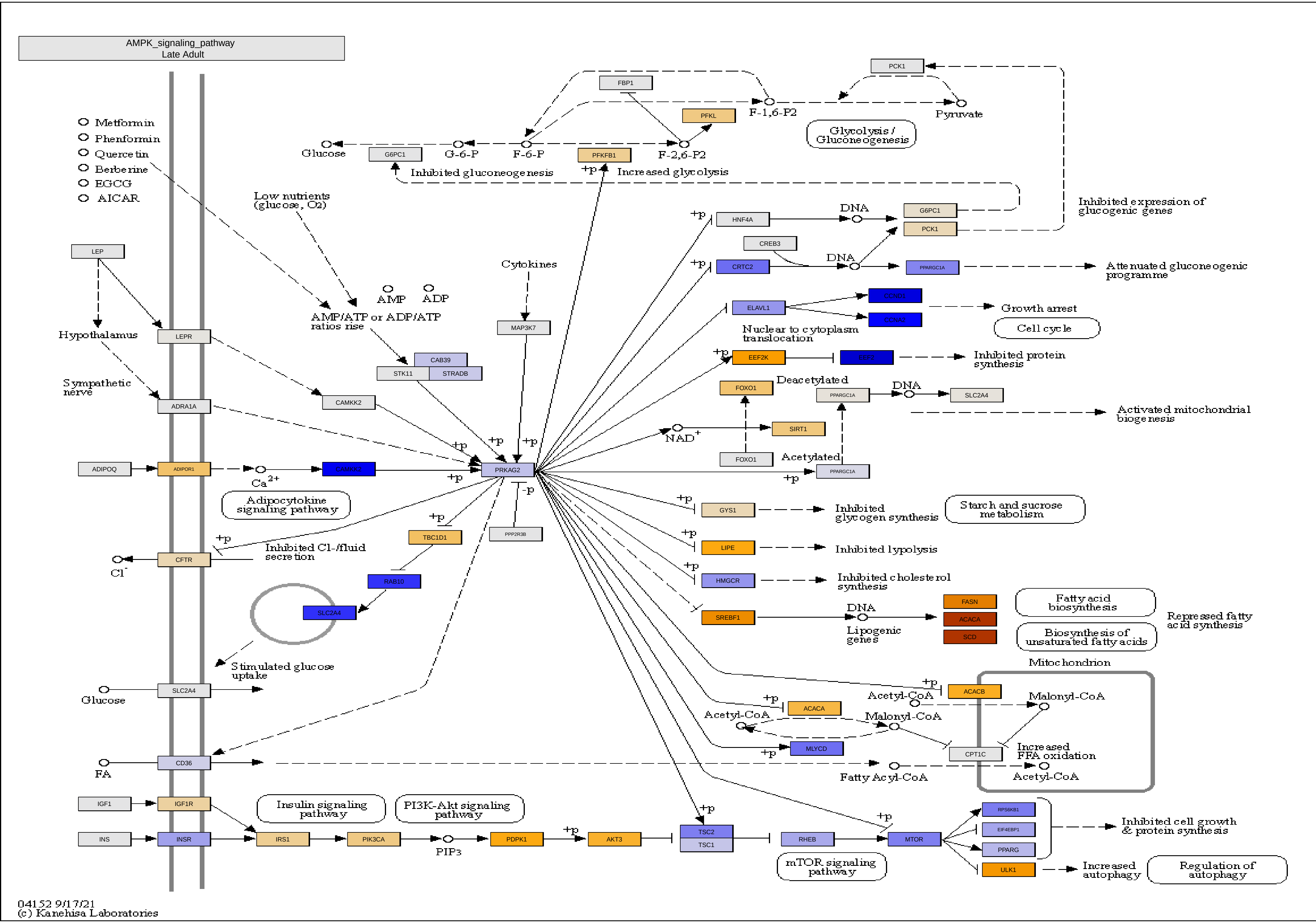
AMPK_signaling_pathway
Late Adult
Metformin
Phenformin
Quercetin
Berberine
EGCG
AICAR
Low nutrients (glucose, O2)
AMP
ADP
AMP/ATP or ADP/ATP ratios rise
Cytokines
LEP
LEPR
Hypothalamus
Sympathetic nerve
ADRA1A
ADIPOQ
ADIPOR1
Ca2+
CAMKK2
CAB39
STK11
STRADB
MAP3K7
PRKAG2
PPP2R3B
Adipocytokine signaling pathway
CFTR
Cl-
Inhibited Cl-/fluid secretion
TBC1D1
RAB10
SLC2A4
Stimulated glucose uptake
Glucose
FA
CD36
IGF1
IGF1R
INS
INSR
IRS1
PIK3CA
PIP3
PDPK1
AKT3
TSC2
TSC1
RHEB
MTOR
Insulin signaling pathway
PI3K-Akt signaling pathway
mTOR signaling pathway
RPS6KB1
EIF4EBP1
PPARG
ULK1
Inhibited cell growth & protein synthesis
Increased autophagy
Regulation of autophagy
FBP1
PFKL
PFKFB1
G6PC1
G-6-P
F-6-P
F-2,6-P2
F-1,6-P2
Pyruvate
PCK1
Glycolysis / Gluconeogenesis
Inhibited gluconeogenesis
Increased glycolysis
HNF4A
CREB3
CRTC2
DNA
G6PC1
PCK1
PPARGC1A
Inhibited expression of glucogenic genes
Attenuated gluconeogenic programme
ELAVL1
CCND1
CCNA2
Nuclear to cytoplasm translocation
Growth arrest
Cell cycle
EEF2K
EEF2
Inhibited protein synthesis
FOXO1
Deacetylated
Acetylated
SIRT1
NAD+
SLC2A4
Activated mitochondrial biogenesis
GYS1
Inhibited glycogen synthesis
Starch and sucrose metabolism
LIPE
Inhibited lypolysis
HMGCR
Inhibited cholesterol synthesis
SREBF1
Lipogenic genes
FASN
ACACA
SCD
Fatty acid biosynthesis
Biosynthesis of unsaturated fatty acids
Repressed fatty acid synthesis
Mitochondrion
ACACB
ACACA
Acetyl-CoA
Malonyl-CoA
MLYCD
CPT1C
Increased FFA oxidation
Fatty Acyl-CoA
+p
-p
04152 9/17/21
(c) Kanehisa Laboratories
