# Supplementary material for: The Transcriptome and Methylome of the Developing and Aging Brain and Their Relations to Gliomas and Psychological Disorders
Source: Cells. 2022 Jan 21;11(3):362. doi: 10.3390/cells11030362 (PMC8834030; doi:10.3390/cells11030362)
Supplement: Supplementary file 1 [file cells-11-00362-s001.zip › Suppl-Material-S3-Pathways-PSF_Expression/B_cell_receptor_signaling_pathway.pdf]

B cell receptor signaling pathway

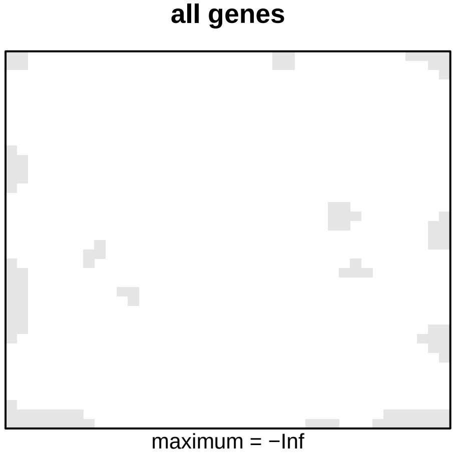

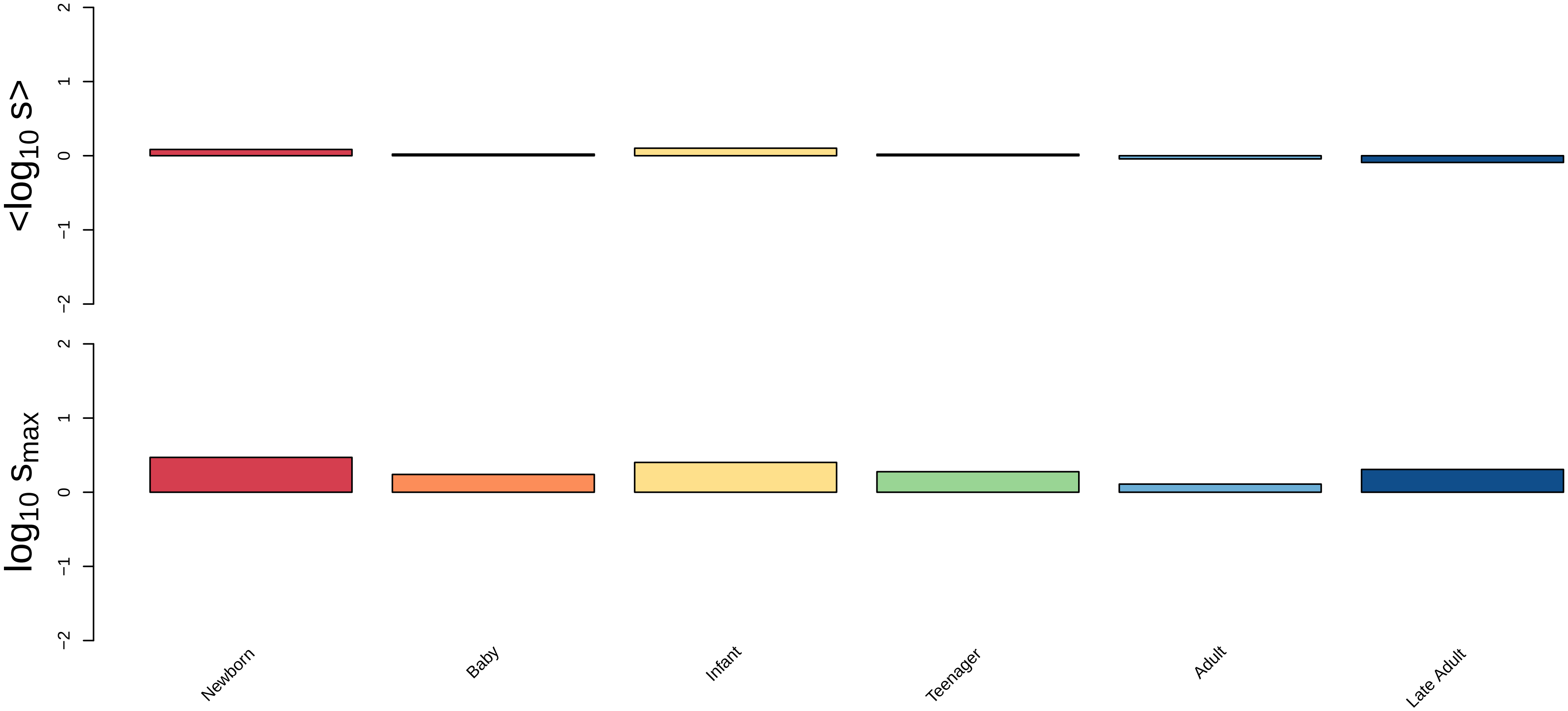

B_cell_receptor_signaling_pathway
genes with data

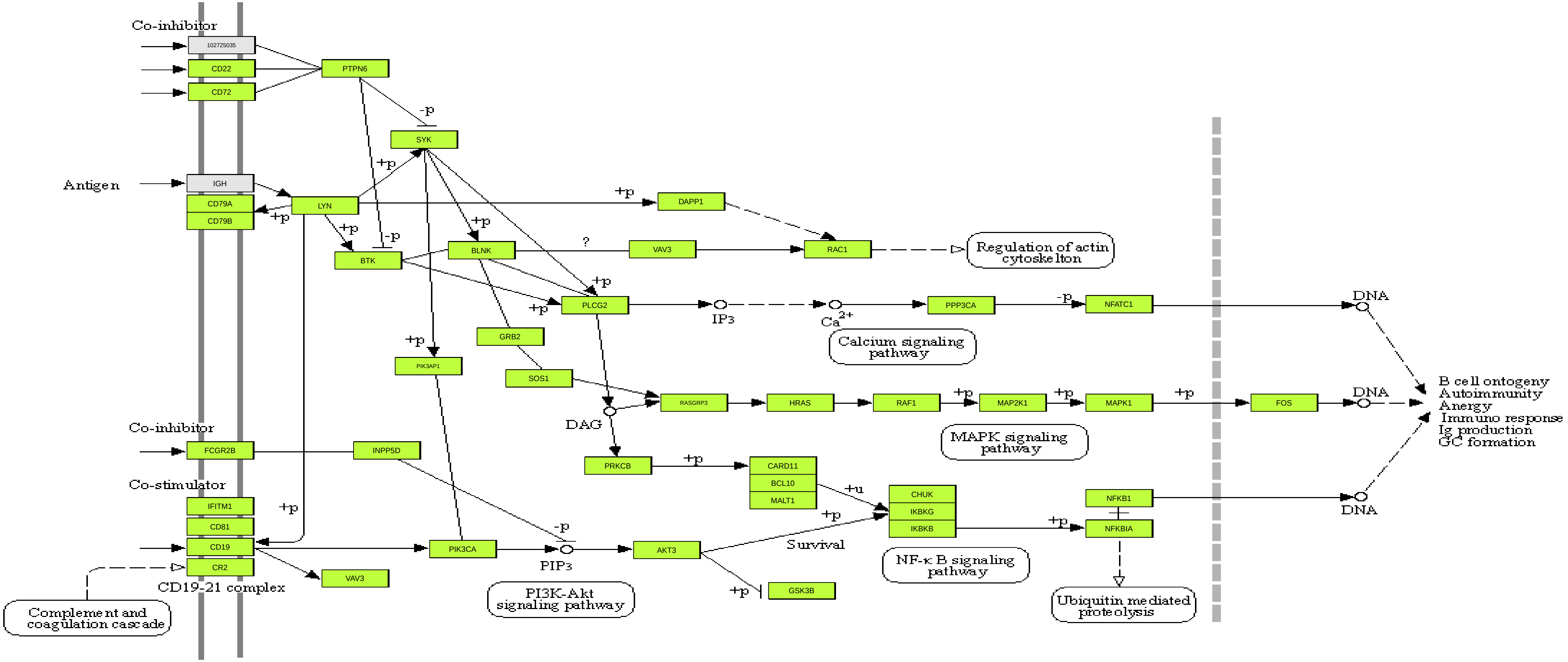

04662 3/31/21
(c) Kanehisa Laboratories

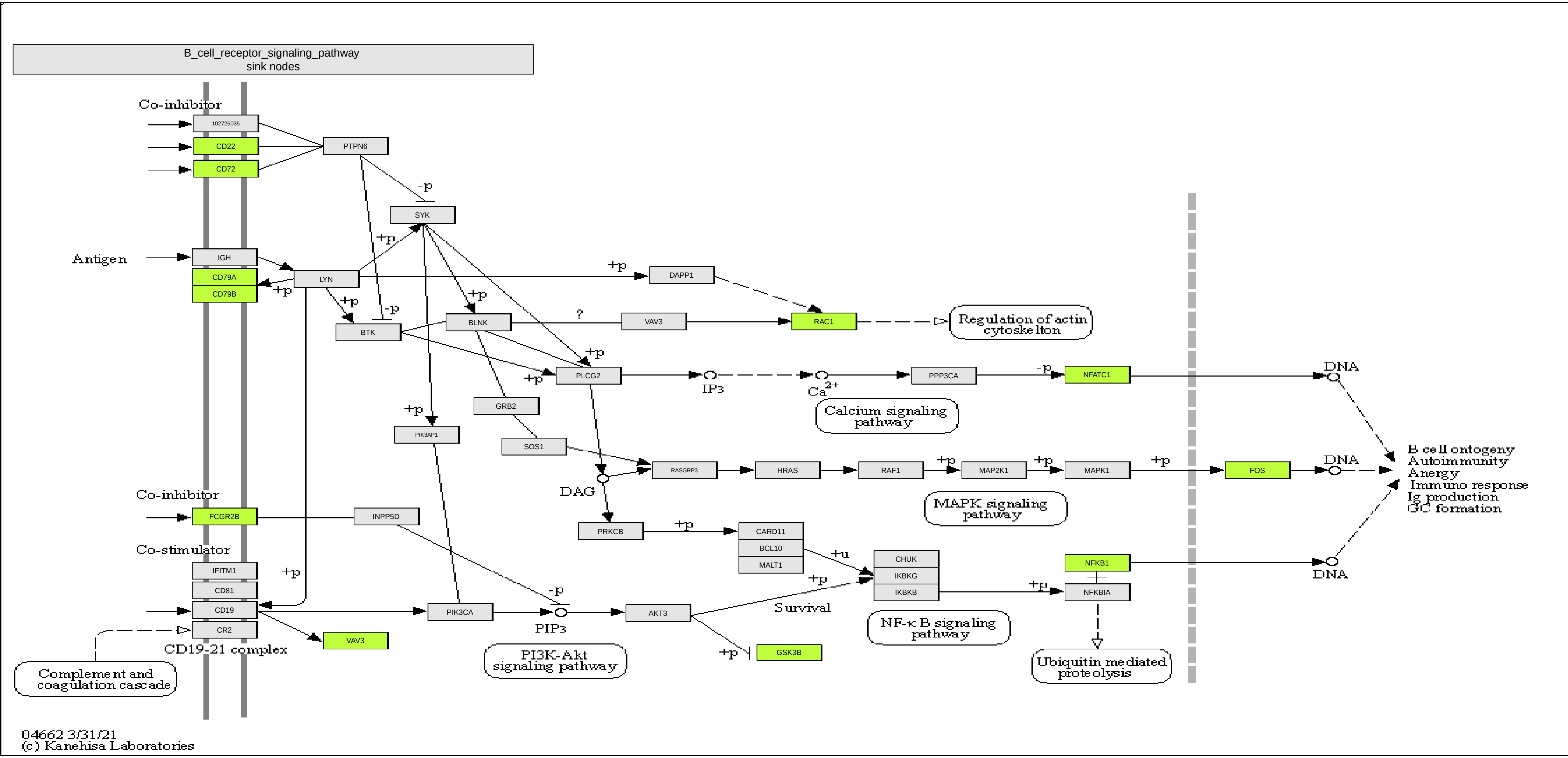
B_cell_receptor_signaling_pathway
sink nodes
Co-inhibitor
102725035
CD22
CD72
PTPN6
-p
SYK
+p
Antigen
IGH
CD79A
CD79B
LYN
+p
DAPP1
+p
BTK
-p
BLNK
?
VAV3
RAC1
Regulation of actin cytoskelton
+p
PLCG2
IP3
Ca2+
PPP3CA
-p
NFATC1
DNA
Calcium signaling pathway
GRB2
PIK3AP1
SOS1
RASGRP3
HRAS
RAF1
MAP2K1
MAPK1
FOS
DNA
DAG
MAPK signaling pathway
B cell ontogeny
Autoimmunity
Anergy
Immuno response
Ig production
GC formation
Co-inhibitor
FCGR2B
INPP5D
PRKCB
CARD11
BCL10
MALT1
+u
CHUK
IKBKG
IKBKB
NFKB1
NFKBIA
DNA
Co-stimulator
IFITM1
CD81
CD19
CR2
CD19-21 complex
VAV3
PIK3CA
PIP3
AKT3
Survival
GSK3B
PI3K-Akt signaling pathway
NF-κ B signaling pathway
Ubiquitin mediated proteolysis
Complement and coagulation cascade
04662 3/31/21
(c) Kanehisa Laboratories

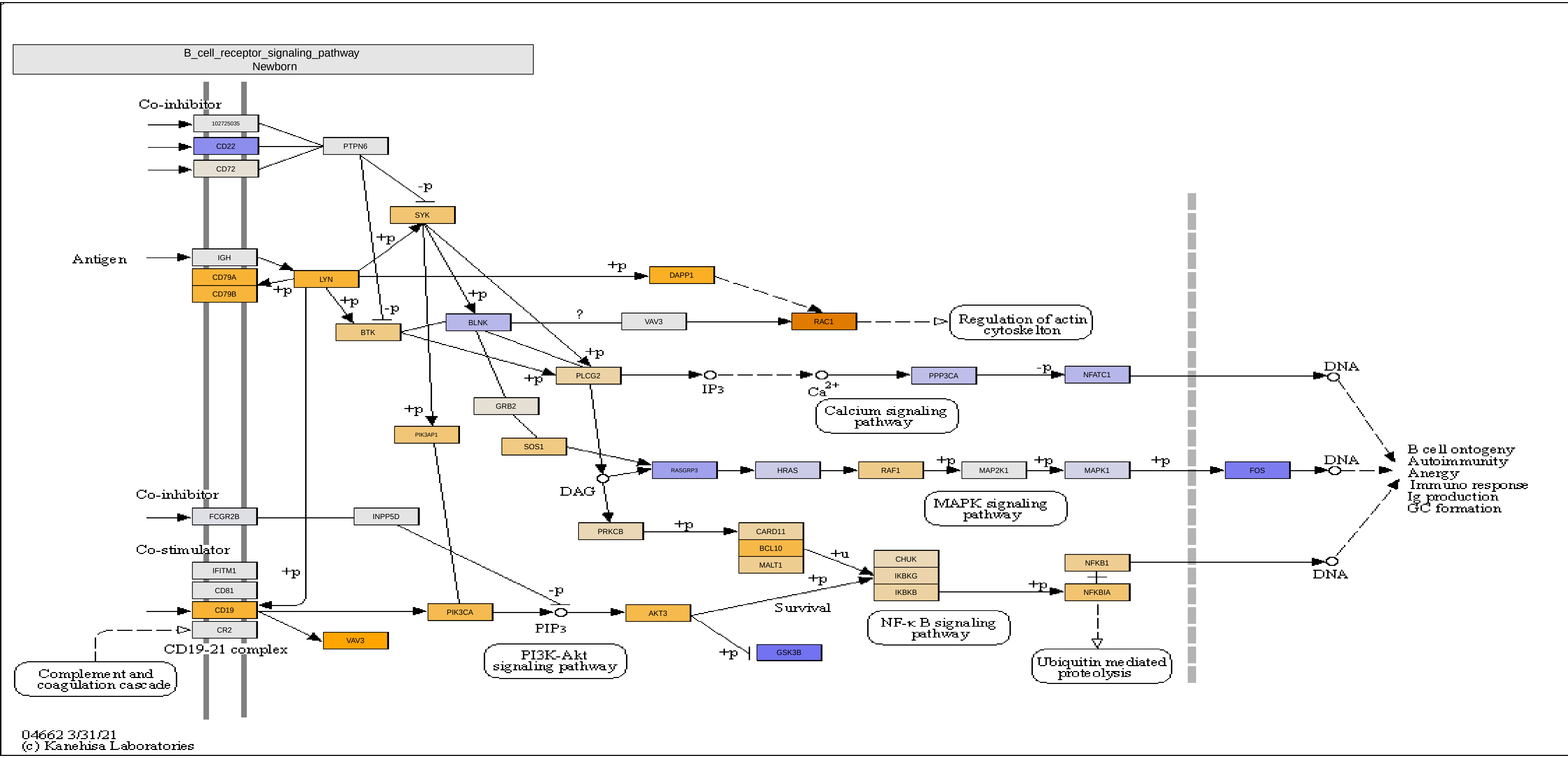
B_cell_receptor_signaling_pathway
Newborn
Co-inhibitor
102725035
CD22
CD72
PTPN6
-p
SYK
+p
Antigen
IGH
CD79A
CD79B
LYN
DAPP1
BTK
BLNK
?
VAV3
RAC1
Regulation of actin cytoskelton
PLCG2
IP3
Ca^{2+}
PPP3CA
NFATC1
DNA
Calcium signaling pathway
GRB2
PIK3AP1
SOS1
RASGRP3
HRAS
RAF1
MAP2K1
MAPK1
FOS
DAG
MAPK signaling pathway
B cell ontogeny
Autoimmunity
Anergy
Immuno response
Ig production
GC formation
Co-inhibitor
FCGR2B
INPP5D
Co-stimulator
IFITM1
CD81
CD19
CR2
CD19-21 complex
PRKCB
CARD11
BCL10
MALT1
+u
CHUK
IKBKG
IKBKB
NFKB1
NFKBIA
PIK3CA
PIP3
AKT3
Survival
GSK3B
VAV3
PI3K-Akt signaling pathway
NF-κ B signaling pathway
Ubiquitin mediated proteolysis
Complement and coagulation cascade
04662 3/31/21
(c) Kanehisa Laboratories

B_cell_receptor_signaling_pathway
Baby

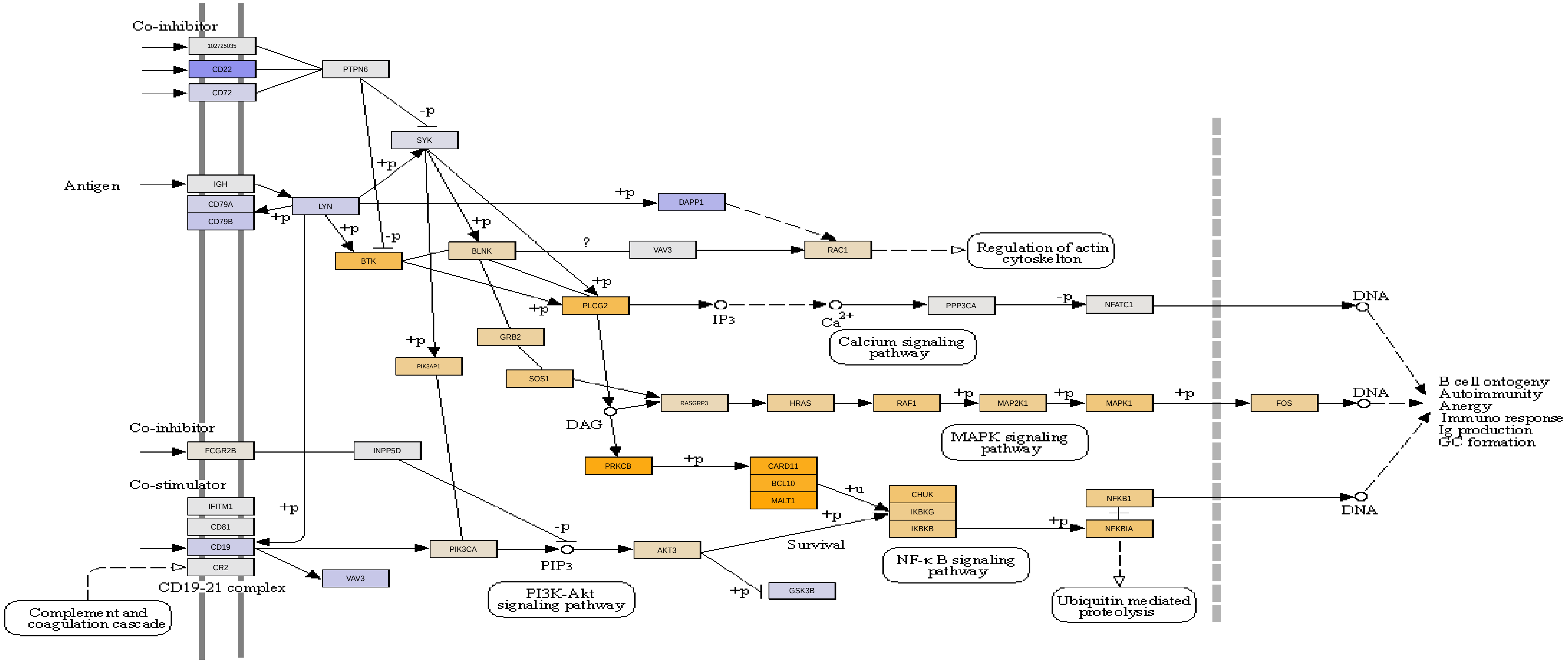

04662 3/31/21
(c) Kanehisa Laboratories

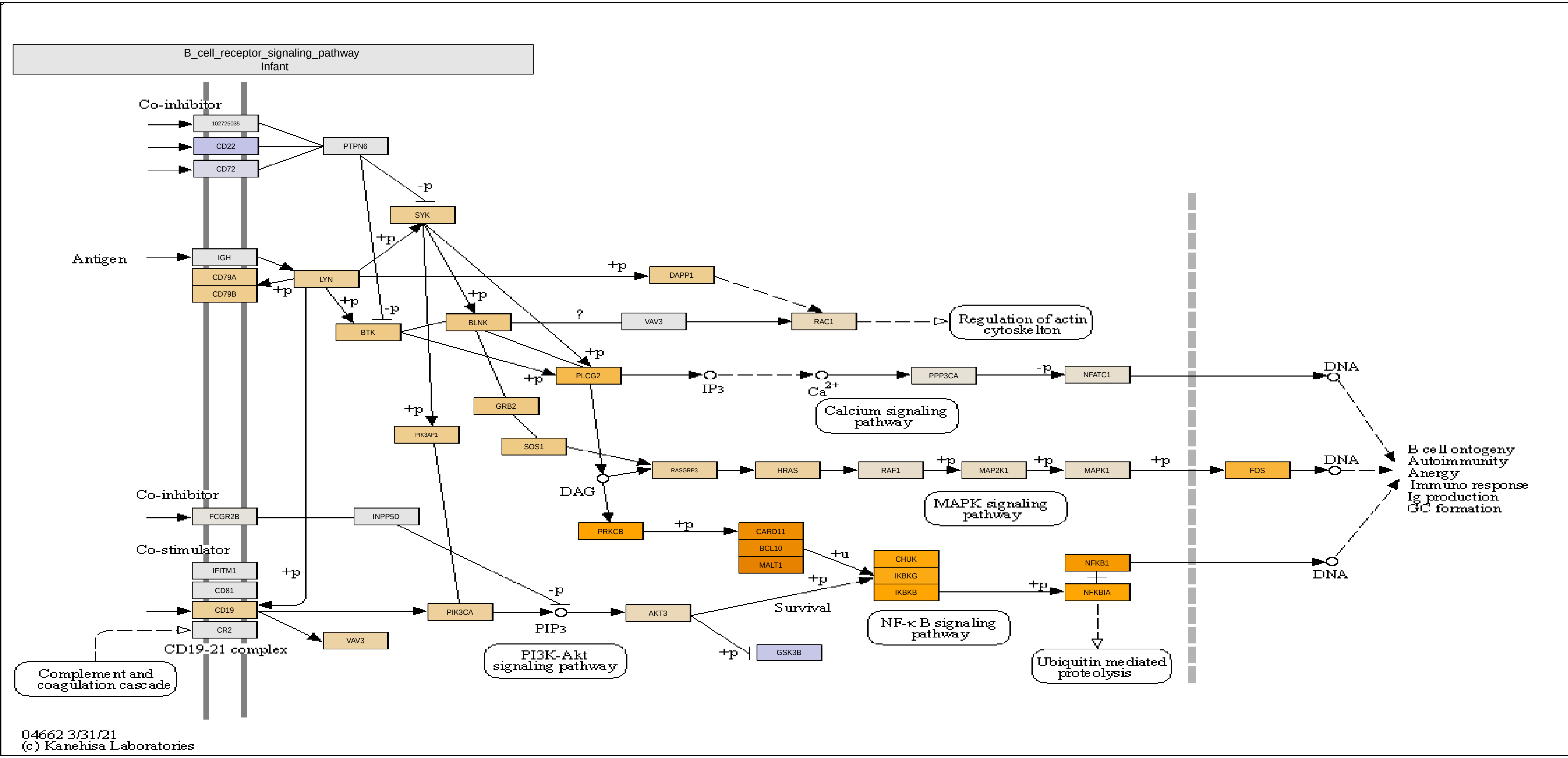
B_cell_receptor_signaling_pathway
Infant
Co-inhibitor
102725035
CD22
CD72
PTPN6
-p
SYK
+p
Antigen
IGH
CD79A
CD79B
LYN
+p
DAPP1
+p
BTK
-p
BLNK
?
VAV3
RAC1
Regulation of actin cytoskeleton
PLCG2
IP3
Ca2+
PPP3CA
-p
NFATC1
DNA
Calcium signaling pathway
GRB2
PIK3AP1
SOS1
RASGRP3
HRAS
RAF1
MAP2K1
MAPK1
FOS
DAG
MAPK signaling pathway
B cell ontogeny
Autoimmunity
Anergy
Immuno response
Ig production
GC formation
Co-inhibitor
FCGR2B
INPP5D
PRKCB
CARD11
BCL10
MALT1
+u
CHUK
IKBKG
IKBKB
NFKB1
NFKBIA
Co-stimulator
IFITM1
CD81
CD19
CR2
PIK3CA
PIP3
AKT3
Survival
GSK3B
VAV3
CD19-21 complex
Complement and coagulation cascade
PI3K-Akt signaling pathway
NF-κB signaling pathway
Ubiquitin mediated proteolysis
04662 3/31/21
(c) Kanehisa Laboratories

B_cell_receptor_signaling_pathway
Teenager

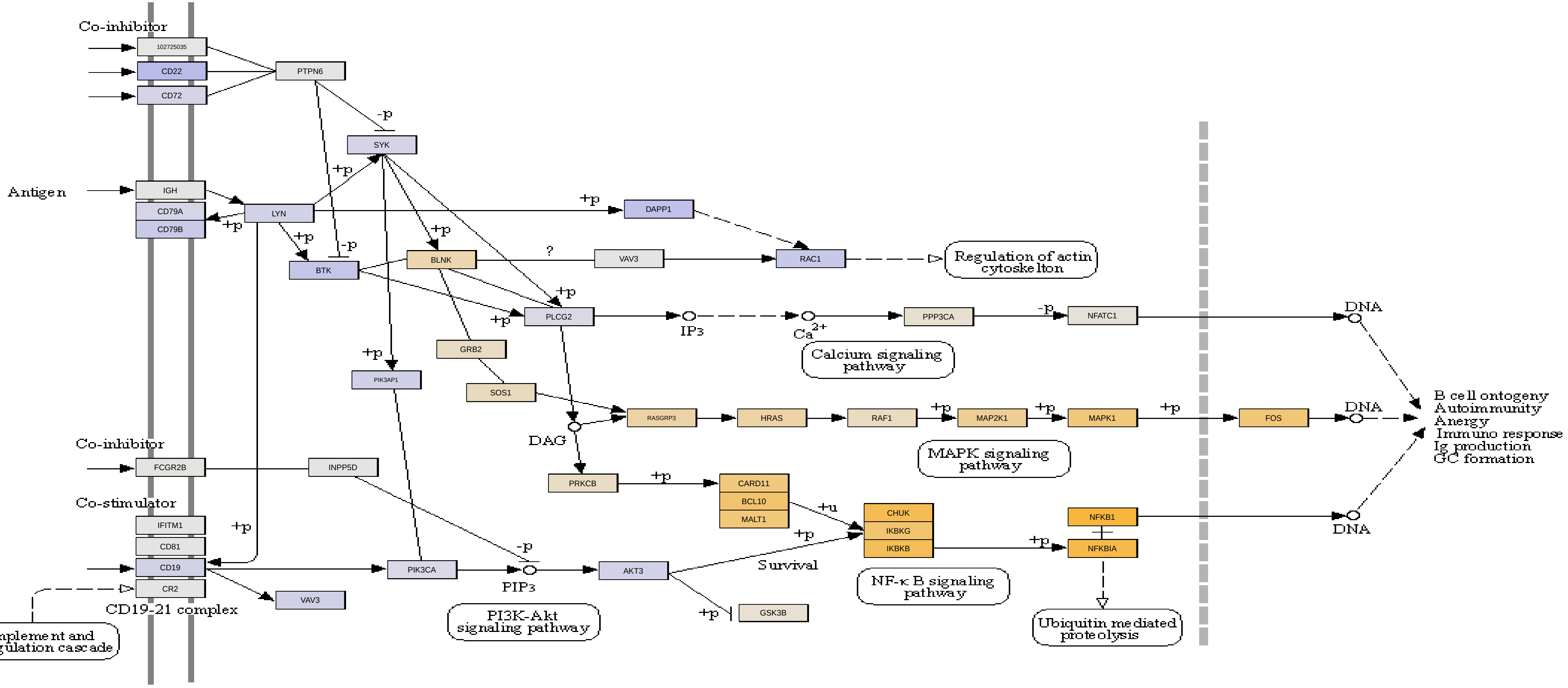

04662 3/31/21
(c) Kanehisa Laboratories

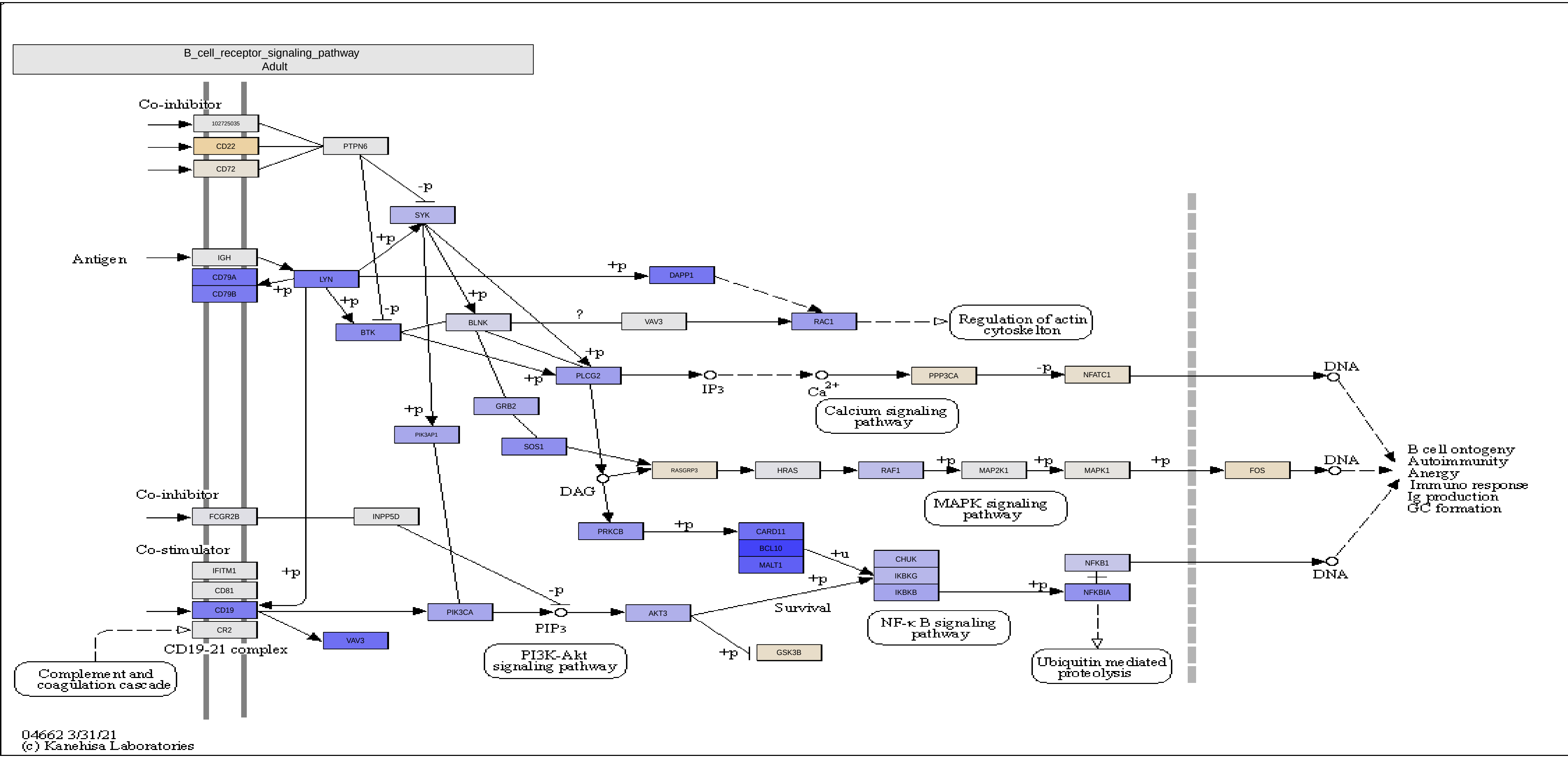
B_cell_receptor_signaling_pathway
Adult
Co-inhibitor
102725035
CD22
CD72
PTPN6
-p
SYK
+p
Antigen
IGH
CD79A
CD79B
LYN
+p
DAPP1
BTK
-p
BLNK
?
VAV3
RAC1
Regulation of actin cytoskelton
PLCG2
IP3
Ca2+
PPP3CA
NFATC1
DNA
Calcium signaling pathway
GRB2
PIK3AP1
SOS1
RASGRP3
HRAS
RAF1
MAP2K1
MAPK1
FOS
DAG
MAPK signaling pathway
B cell ontogeny
Autoimmunity
Anergy
Immuno response
Ig production
GC formation
Co-inhibitor
FCGR2B
INPP5D
Co-stimulator
PRKCB
CARD11
BCL10
MALT1
+u
CHUK
IKBKG
IKBKB
NFKB1
NFKBIA
IFITM1
CD81
CD19
CR2
PIK3CA
PIP3
AKT3
Survival
GSK3B
VAV3
CD19-21 complex
Complement and coagulation cascade
PI3K-Akt signaling pathway
NF-κ B signaling pathway
Ubiquitin mediated proteolysis
04662 3/31/21
(c) Kanehisa Laboratories

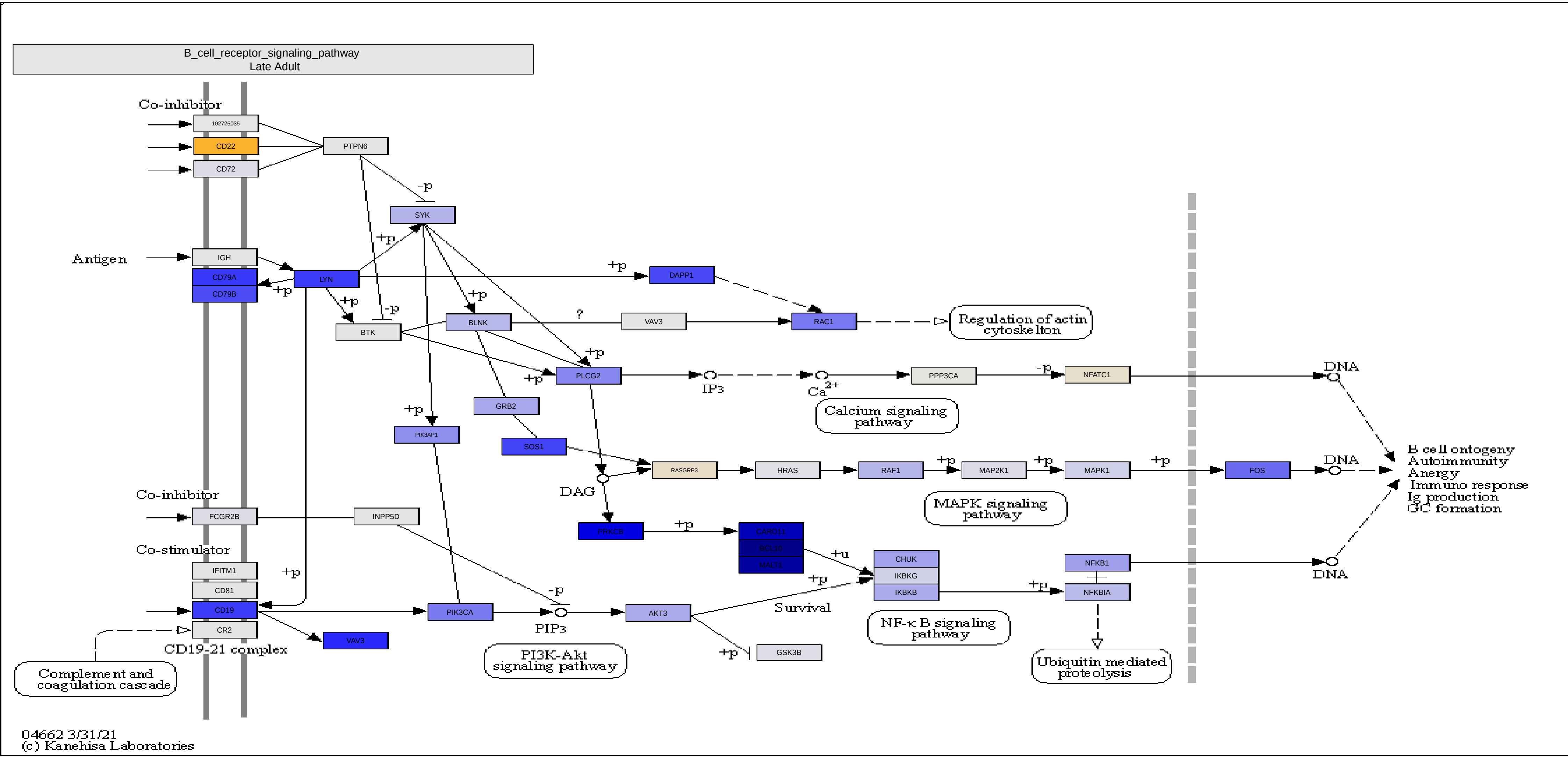
B_cell_receptor_signaling_pathway
Late Adult
Co-inhibitor
102725035
CD22
CD72
PTPN6
-p
SYK
+p
Antigen
IGH
CD79A
CD79B
LYN
+p
DAPP1
BTK
BLNK
?
VAV3
RAC1
Regulation of actin cytoskelton
PLCG2
IP3
Ca^{2+}
PPP3CA
NFATC1
DNA
Calcium signaling pathway
GRB2
PIK3AP1
SOS1
RASGRP3
HRAS
RAF1
MAP2K1
MAPK1
FOS
DAG
MAPK signaling pathway
B cell ontogeny
Autoimmunity
Anergy
Immuno response
Ig production
GC formation
Co-inhibitor
FCGR2B
INPP5D
PRKCB
CARD11
BCL10
MALT1
+u
CHUK
IKBKG
IKBKB
NFKB1
NFKBIA
Co-stimulator
IFITM1
CD81
CD19
CR2
PIK3CA
PIP3
AKT3
Survival
GSK3B
VAV3
CD19-21 complex
Complement and coagulation cascade
PI3K-Akt signaling pathway
NF-κ B signaling pathway
Ubiquitin mediated proteolysis
04662 3/31/21
(c) Kanehisa Laboratories
