# Supplementary material for: The Transcriptome and Methylome of the Developing and Aging Brain and Their Relations to Gliomas and Psychological Disorders
Source: Cells. 2022 Jan 21;11(3):362. doi: 10.3390/cells11030362 (PMC8834030; doi:10.3390/cells11030362)
Supplement: Supplementary file 1 [file cells-11-00362-s001.zip › Suppl-Material-S3-Pathways-PSF_Expression/Calcium_signaling_pathway.pdf]

Calcium signaling pathway

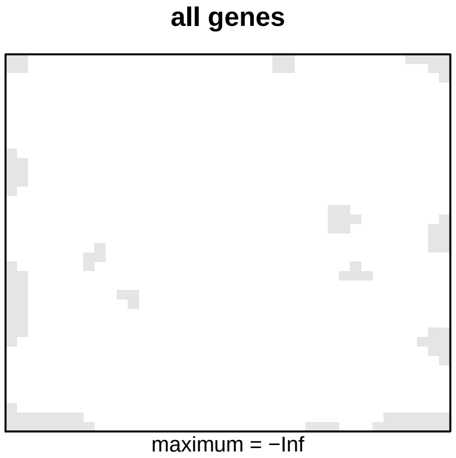

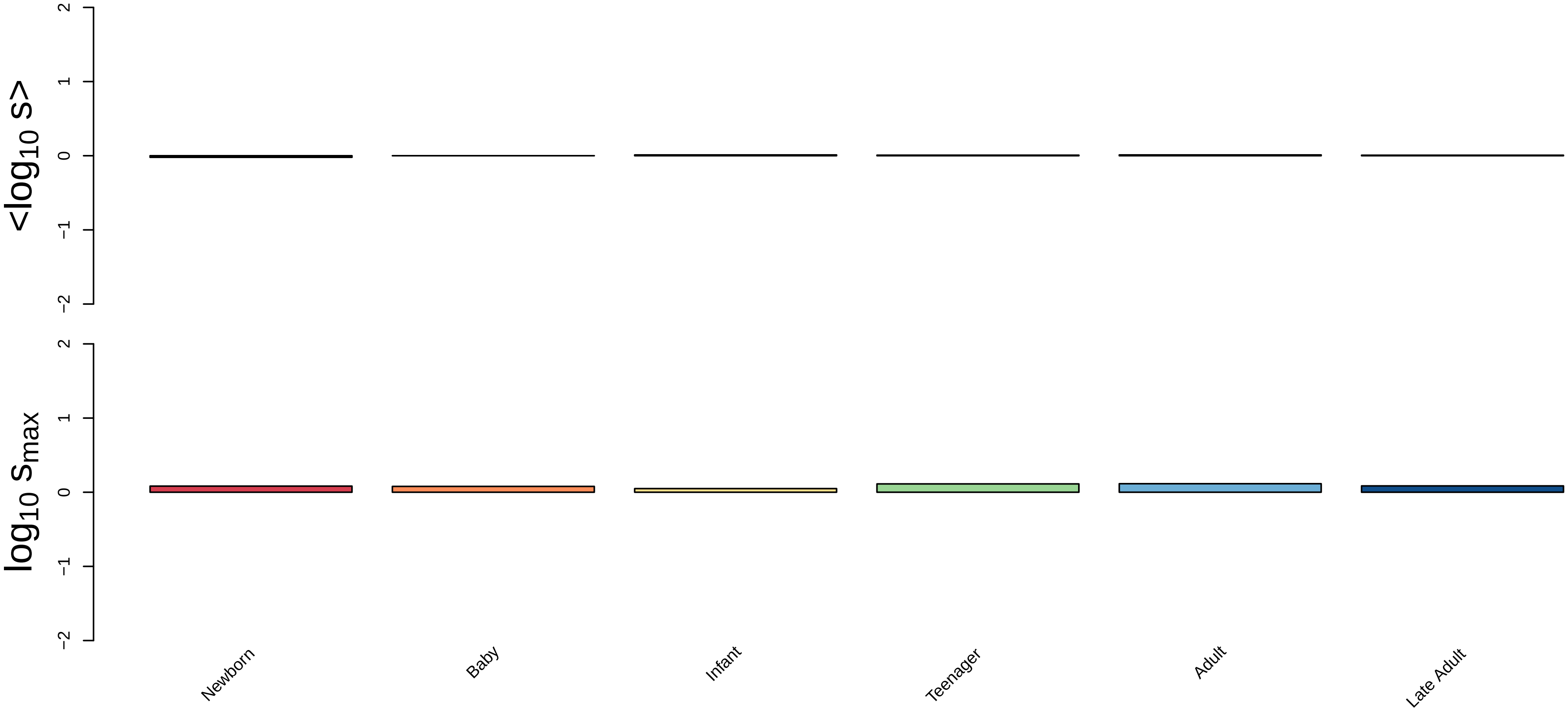

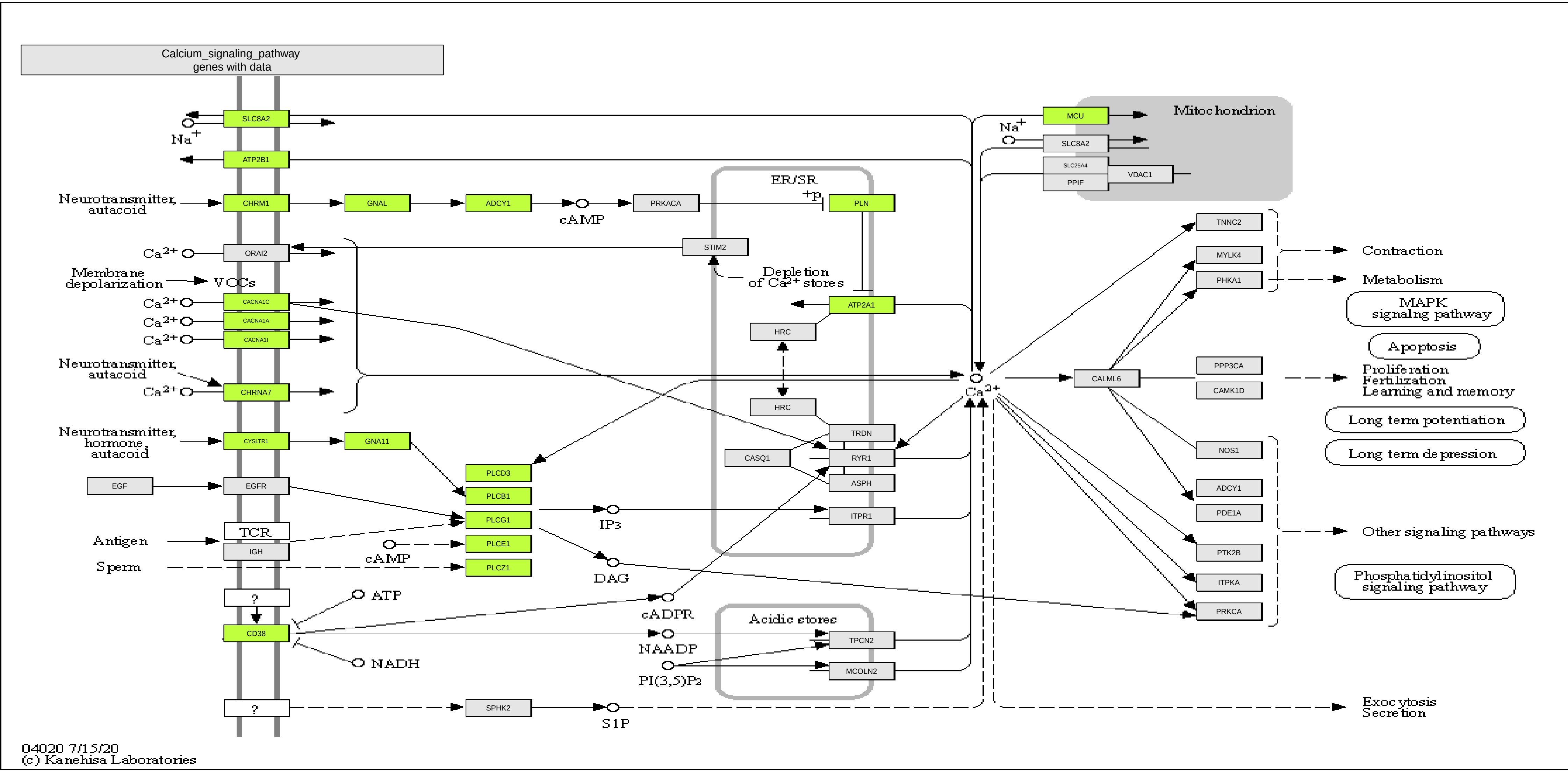
Calcium_signaling_pathway
genes with data
SLC8A2
Na+
ATP2B1
Neurotransmitter, autacoid
CHRM1
GNAL
ADCY1
cAMP
PRKACA
ER/SR
+p
PLN
Ca2+
ORAI2
STIM2
Membrane depolarization
VOCs
Depletion of Ca2+ stores
CACNA1C
CACNA1A
CACNA1I
ATP2A1
HRC
Neurotransmitter, autacoid
CHRNA7
HRC
TRDN
Neurotransmitter, hormone, autacoid
CYSLTR1
GNA11
CASQ1
RYR1
ASPH
PLCD3
PLCB1
EGF
EGFR
PLCG1
IP3
ITPR1
TCR
IGH
Antigen
PLCE1
Sperm
PLCZ1
DAG
?
ATP
CD38
cADPR
NAADP
NADH
Acidic stores
TPCN2
MCOLN2
PI(3,5)P2
SPHK2
S1P
Mitochondrion
MCU
SLC8A2
SLC25A4
PPIF
VDAC1
TNNC2
MYLK4
PHKA1
Contraction
Metabolism
MAPK signalng pathway
Apoptosis
CALML6
PPP3CA
CAMK1D
Proliferation
Fertilization
Learning and memory
Long term potentiation
Long term depression
NOS1
ADCY1
PDE1A
Other signaling pathways
PTK2B
ITPKA
Phosphatidylinositol signaling pathway
PRKCA
Exocytosis
Secretion
04020 7/15/20
(c) Kanehisa Laboratories

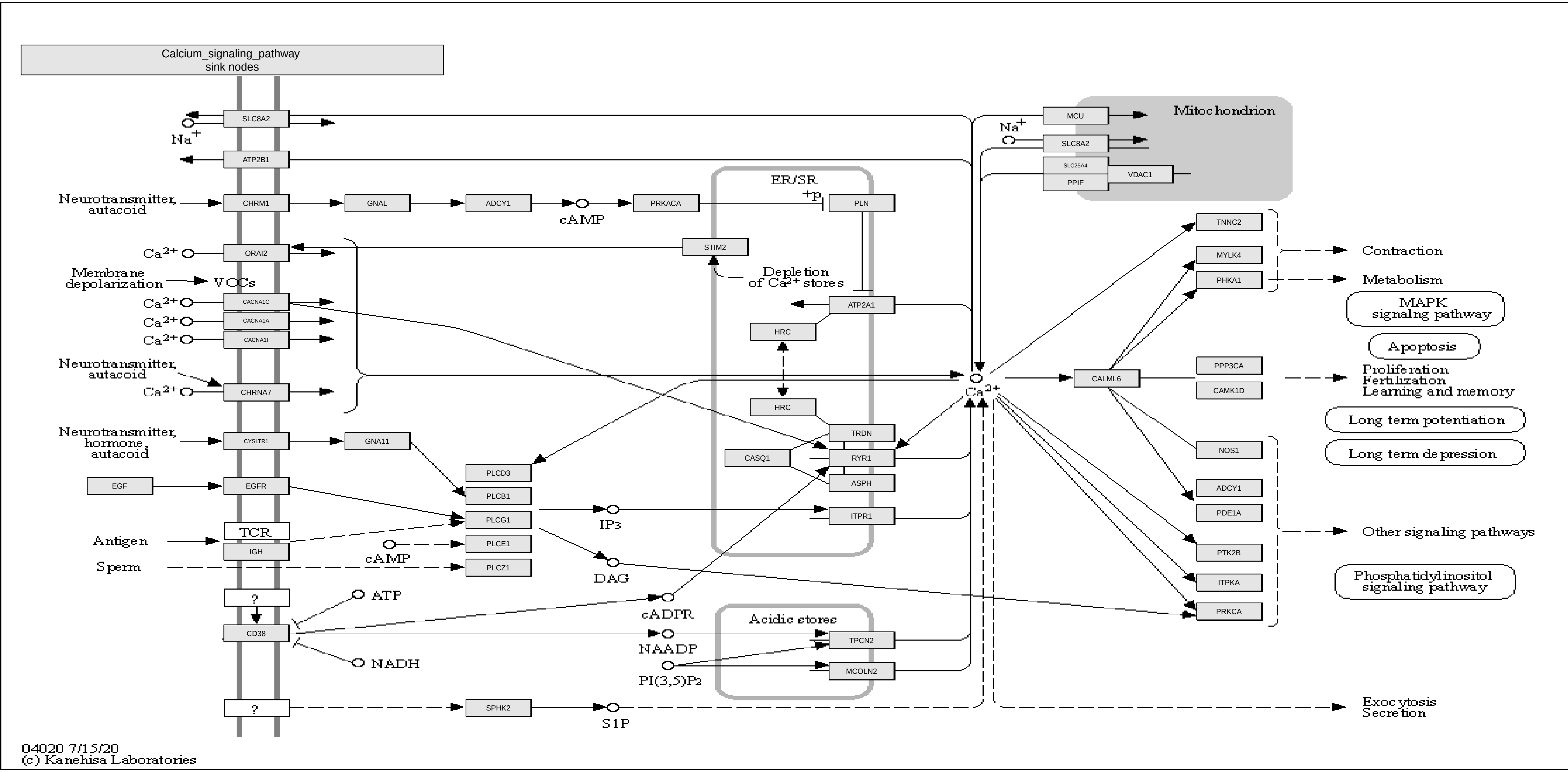

Calcium_signaling_pathway
sink nodes
SLC8A2
Na+
ATP2B1
Neurotransmitter, autacoid
CHRM1
GNAL
ADCY1
cAMP
PRKACA
+p
PLN
ER/SR
Ca2+
ORAI2
STIM2
Depletion of Ca2+ stores
Membrane depolarization
VOCs
CACNA1C
CACNA1A
CACNA1I
ATP2A1
HRC
HRC
Neurotransmitter, autacoid
CHRNA7
Neurotransmitter, hormone, autacoid
CYSLTR1
GNA11
TRDN
CASQ1
RYR1
ASPH
EGF
EGFR
PLCD3
PLCB1
PLCG1
IP3
ITPR1
Antigen
TCR
IGH
PLCE1
Sperm
PLCZ1
DAG
?
ATP
cADPR
CD38
NAADP
Acidic stores
TPCN2
NADH
PI(3,5)P2
MCOLN2
?
SPHK2
S1P
Mitochondrion
MCU
SLC8A2
SLC25A4
PPIF
VDAC1
TNNC2
MYLK4
PHKA1
Contraction
Metabolism
MAPK signalng pathway
Apoptosis
CALML6
PPP3CA
CAMK1D
Proliferation
Fertilization
Learning and memory
Long term potentiation
Long term depression
NOS1
ADCY1
PDE1A
Other signaling pathways
PTK2B
ITPKA
Phosphatidylinositol signaling pathway
PRKCA
Exocytosis
Secretion
04020 7/15/20
(c) Kanehisa Laboratories

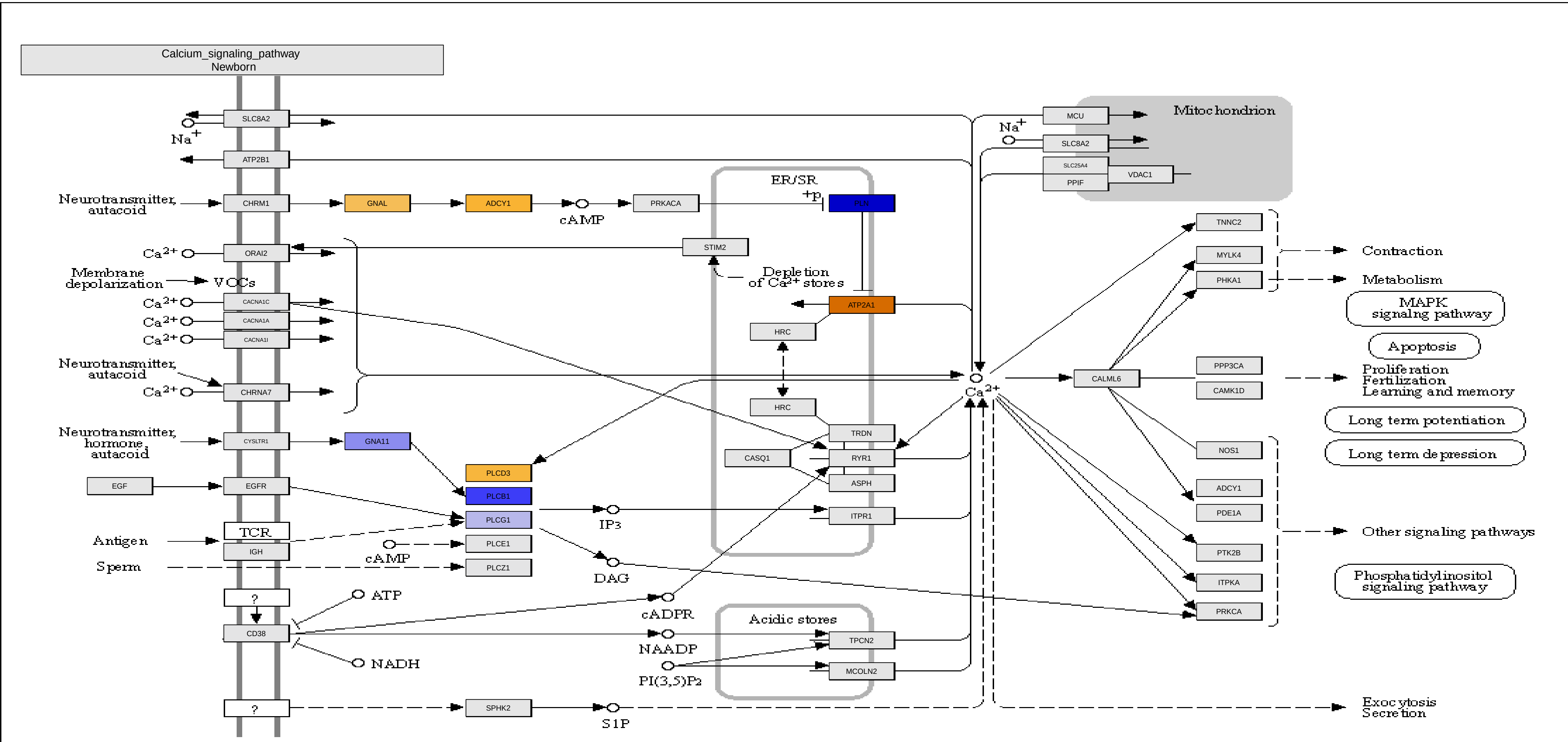

04020 7/15/20

(c) Kanehisa Laboratories

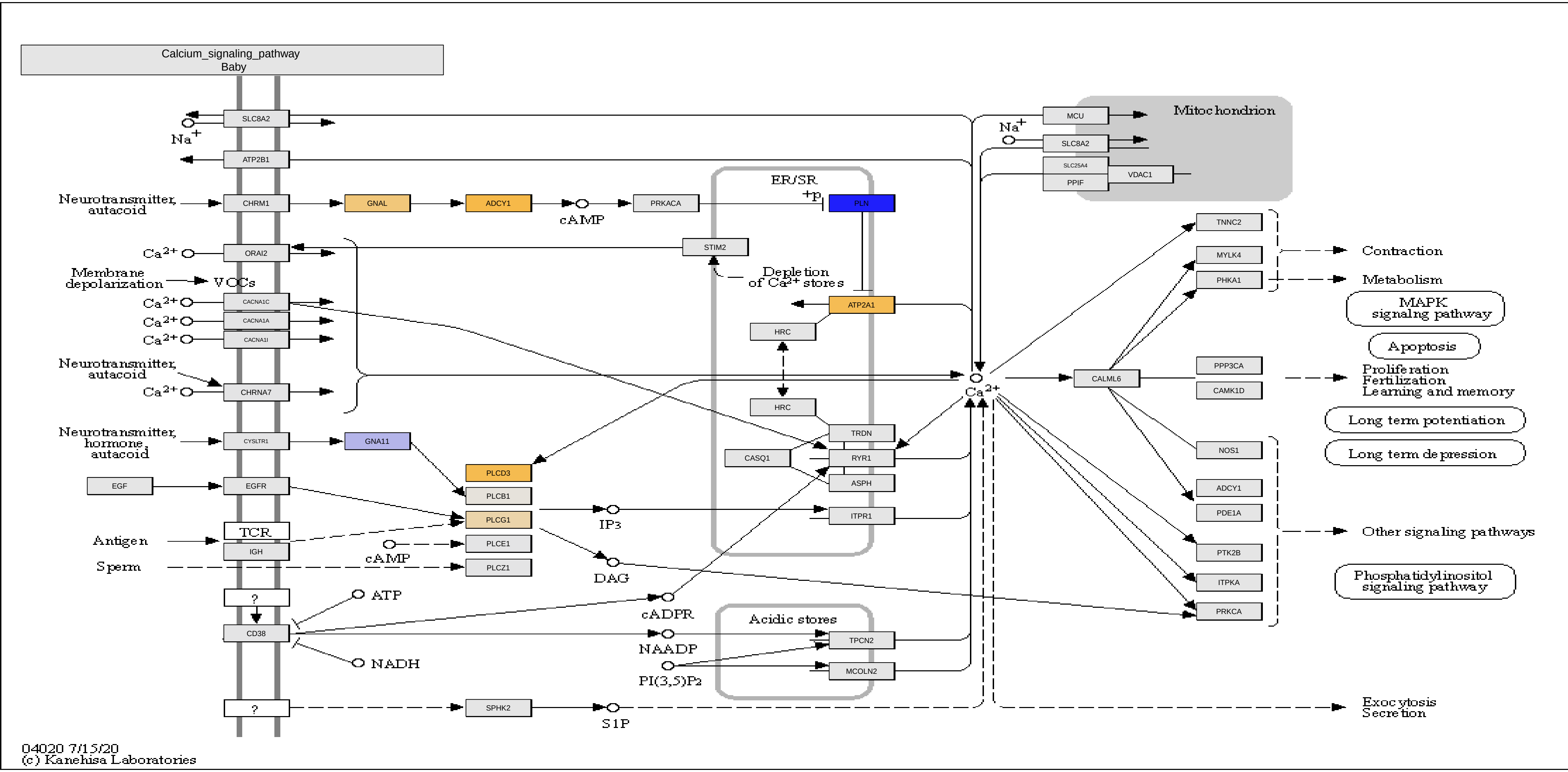
Calcium_signaling_pathway
Baby
SLC8A2
Na+
ATP2B1
Neurotransmitter, autacoid
CHRM1
GNAL
ADCY1
cAMP
PRKACA
ER/SR
+p
PLN
Ca2+
ORAI2
STIM2
Depletion of Ca2+ stores
Membrane depolarization
VOCs
CACNA1C
CACNA1A
CACNA1I
ATP2A1
HRC
Neurotransmitter, autacoid
CHRNA7
HRC
TRDN
CASQ1
RYR1
ASPH
Neurotransmitter, hormone, autacoid
CYSLTR1
GNA11
PLCD3
EGF
EGFR
PLCB1
PLCG1
IP3
ITPR1
TCR
Antigen
IGH
cAMP
PLCE1
Sperm
PLCZ1
DAG
?
ATP
CD38
cADPR
Acidic stores
NAADP
TPCN2
NADH
PI(3,5)P2
MCOLN2
?
SPHK2
S1P
Mitochondrion
MCU
Na+
SLC8A2
SLC25A4
PPIF
VDAC1
Ca2+
TNNC2
MYLK4
PHKA1
Contraction
Metabolism
MAPK signalng pathway
Apoptosis
CALML6
PPP3CA
CAMK1D
Proliferation
Fertilization
Learning and memory
Long term potentiation
Long term depression
NOS1
ADCY1
PDE1A
PTK2B
ITPKA
PRKCA
Other signaling pathways
Phosphatidylinositol signaling pathway
Exocytosis
Secretion
04020 7/15/20
(c) Kanehisa Laboratories

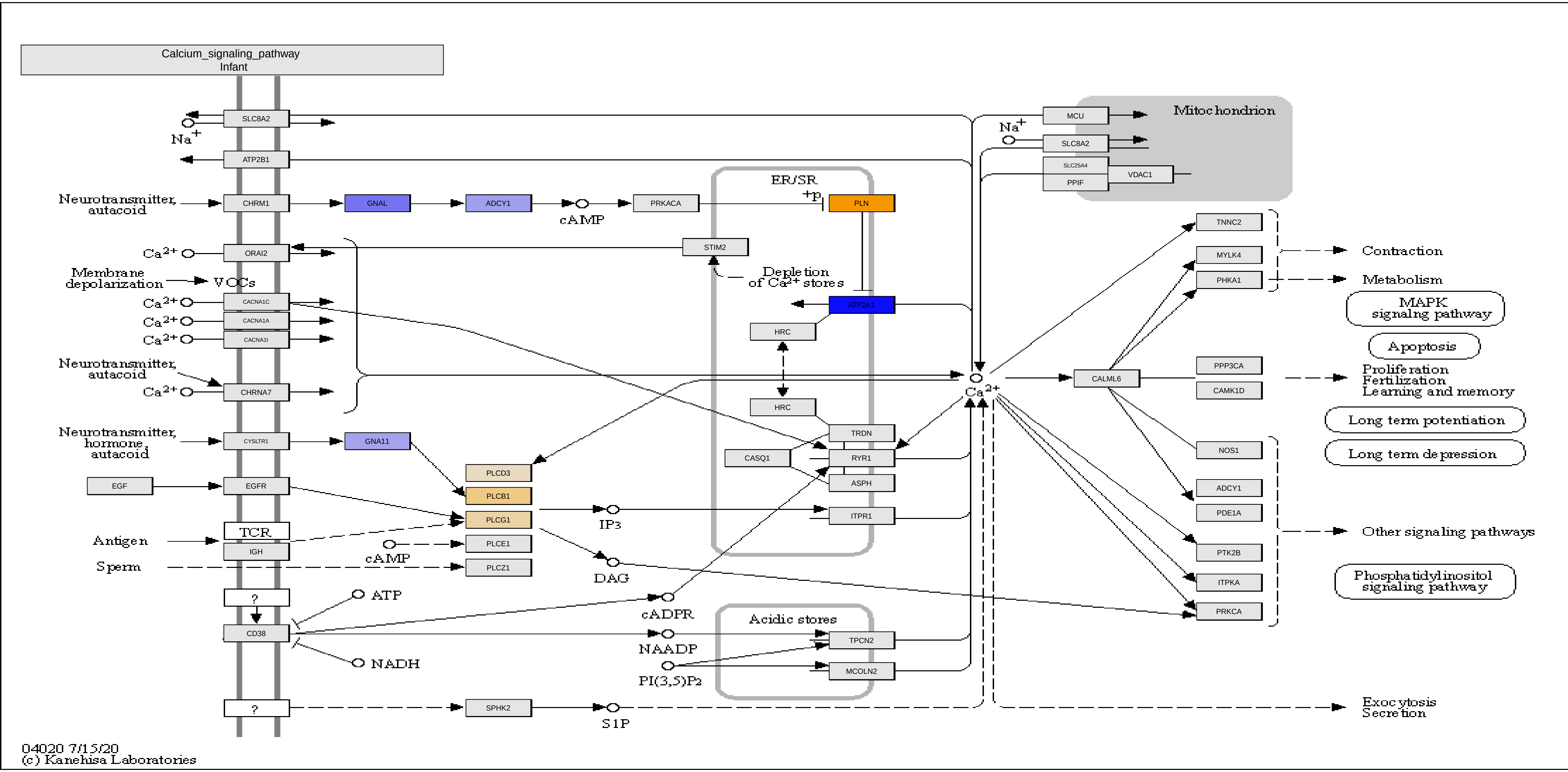
Calcium_signaling_pathway
Infant
SLC8A2
Na+
ATP2B1
Neurotransmitter, autacoid
CHRM1
GNAL
ADCY1
cAMP
PRKACA
ER/SR
+p
PLN
Ca2+
ORAI2
STIM2
Membrane depolarization
VCCs
Depletion of Ca2+ stores
CACNA1C
CACNA1A
CACNA1I
ATP2A1
HRC
Neurotransmitter, autacoid
CHRNA7
HRC
TRDN
Neurotransmitter, hormone, autacoid
CYSLTR1
GNA11
CASQ1
RYR1
ASPH
EGF
EGFR
PLCD3
PLCB1
PLCG1
IP3
ITPR1
TCR
Antigen
IGH
PLCE1
cAMP
Sperm
PLCZ1
DAG
?
ATP
cADPR
CD38
Acidic stores
NAADP
TPCN2
NADH
PI(3,5)P2
MCOLN2
?
SPHK2
S1P
MCU
Mitochondrion
Na+
SLC8A2
SLC25A4
VDAC1
PPIF
TNNC2
MYLK4
Contraction
PHKA1
Metabolism
MAPK signalng pathway
Apoptosis
PPP3CA
CALML6
Proliferation
Fertilization
Learning and memory
CAMK1D
Long term potentiation
NOS1
Long term depression
ADCY1
PDE1A
Other signaling pathways
PTK2B
ITPKA
Phosphatidylinositol signaling pathway
PRKCA
Exocytosis
Secretion
04020 7/15/20
(c) Kanehisa Laboratories

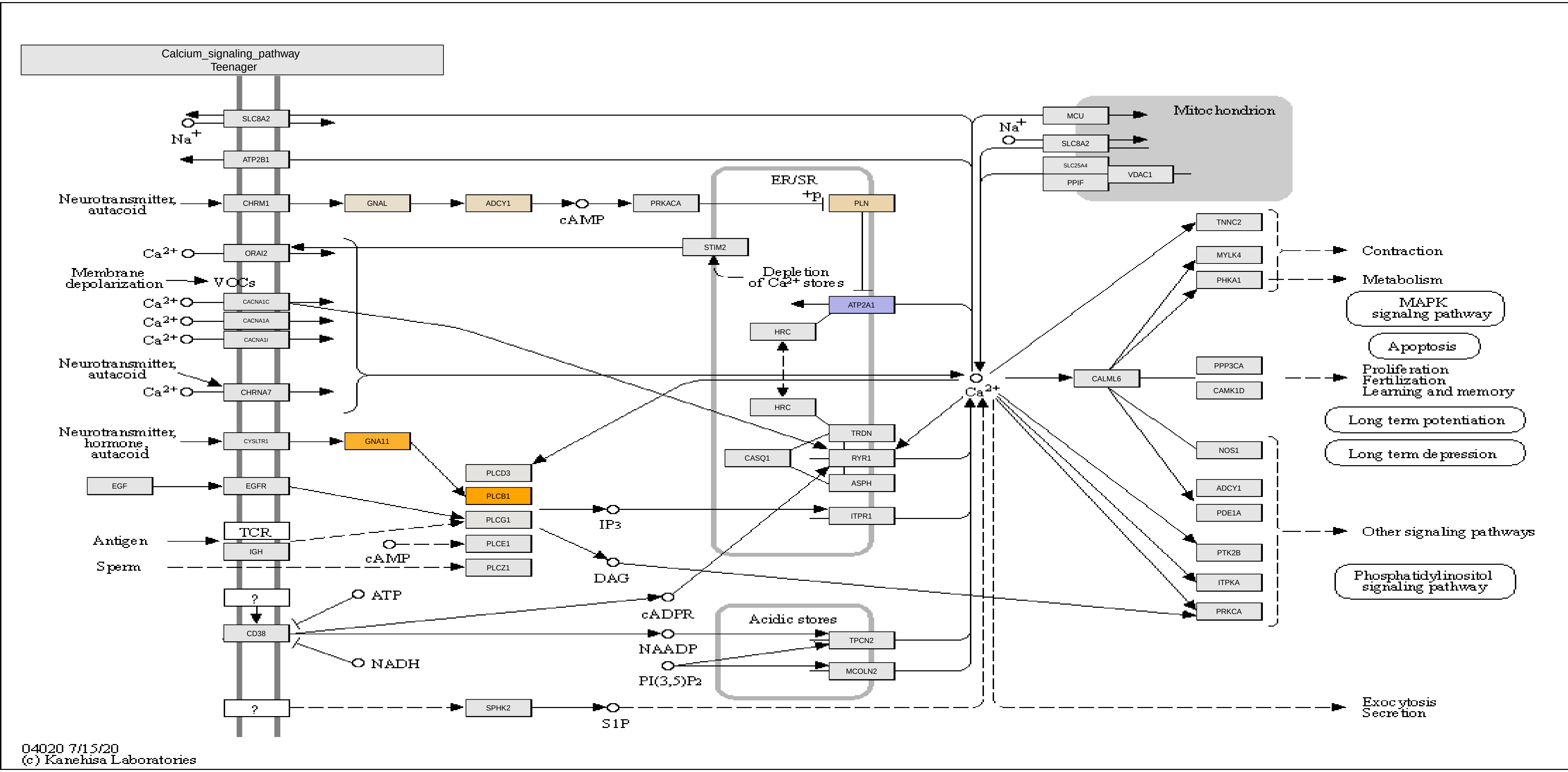

Calcium_signaling_pathway
Teenager
SLC8A2
Na+
ATP2B1
Neurotransmitter, autacoid
CHRM1
GNAL
ADCY1
cAMP
PRKACA
ER/SR
+p
PLN
Ca2+
ORAI2
STIM2
Depletion of Ca2+ stores
Membrane depolarization
VOCs
CACNA1C
CACNA1A
CACNA1I
ATP2A1
HRC
HRC
Neurotransmitter, autacoid
CHRNA7
TRDN
CASQ1
RYR1
ASPH
Neurotransmitter, hormone, autacoid
CYSLTR1
GNA11
PLCD3
PLCB1
EGF
EGFR
PLCG1
IP3
ITPR1
TCR
Antigen
IGH
cAMP
PLCE1
Sperm
PLCZ1
DAG
?
ATP
CD38
cADPR
NAADP
Acidic stores
TPCN2
NADH
PI(3,5)P2
MCOLN2
?
SPHK2
S1P
Mitochondrion
MCU
SLC8A2
SLC25A4
PPIF
VDAC1
TNNC2
MYLK4
PHKA1
Contraction
Metabolism
MAPK signalng pathway
Apoptosis
PPP3CA
CALML6
CAMK1D
Proliferation
Fertilization
Learning and memory
Long term potentiation
Long term depression
NOS1
ADCY1
PDE1A
PTK2B
ITPKA
PRKCA
Other signaling pathways
Phosphatidylinositol signaling pathway
Exocytosis
Secretion
04020 7/15/20
(c) Kanehisa Laboratories

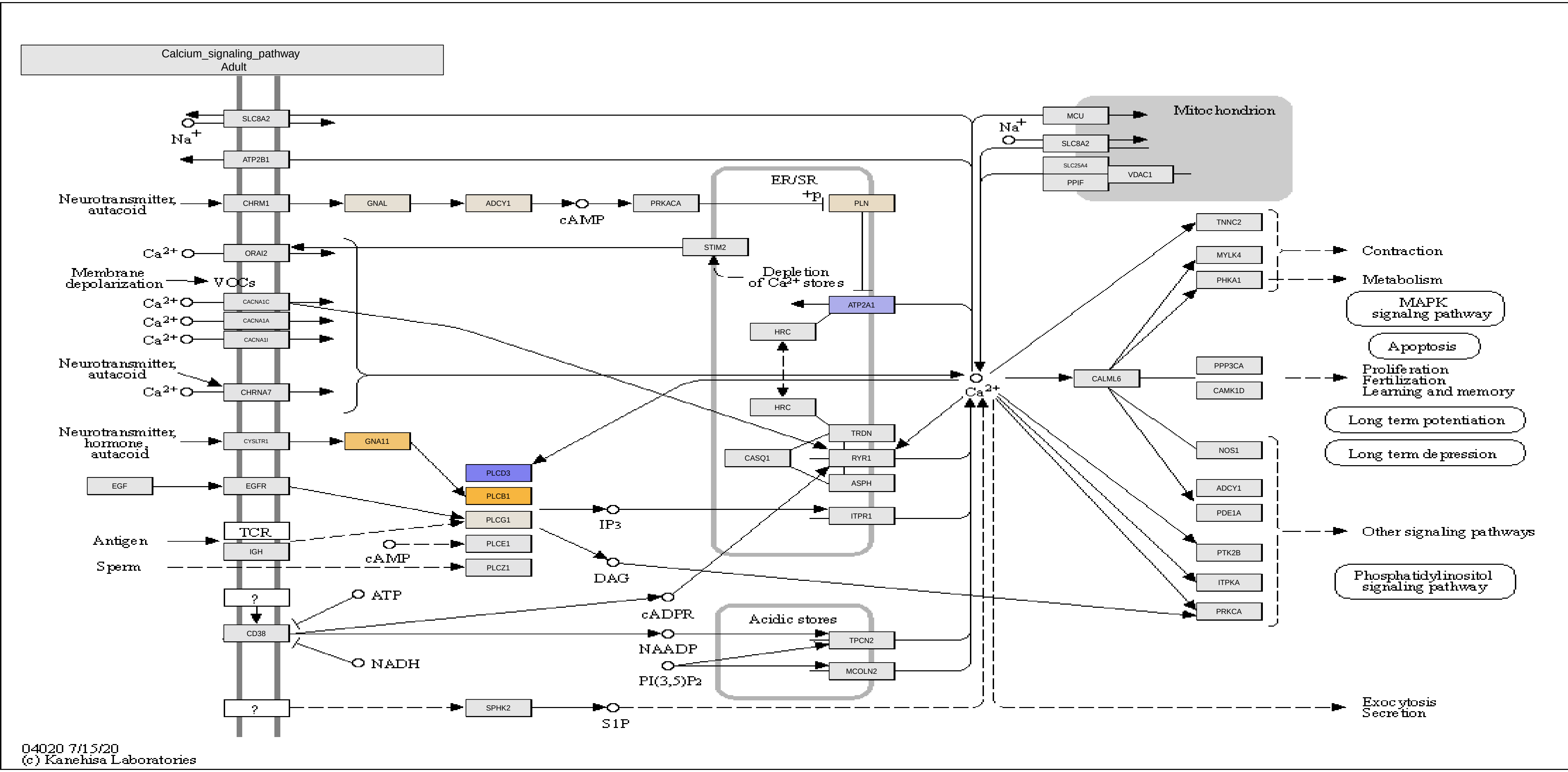
Calcium_signaling_pathway
Adult
SLC8A2
Na+
ATP2B1
Neurotransmitter, autacoid
CHRM1
GNAL
ADCY1
cAMP
PRKACA
ER/SR
+p
PLN
Ca2+
ORAI2
STIM2
Membrane depolarization
VOCs
Depletion of Ca2+ stores
CACNA1C
CACNA1A
CACNA1I
ATP2A1
HRC
Neurotransmitter, autacoid
CHRNA7
HRC
TRDN
Neurotransmitter, hormone, autacoid
CYSLTR1
GNA11
CASQ1
RYR1
ASPH
EGF
EGFR
PLCD3
PLCB1
PLCG1
IP3
ITPR1
TCR
Antigen
IGH
cAMP
PLCE1
Sperm
PLCZ1
DAG
?
ATP
CD38
cADPR
Acidic stores
NAADP
TPCN2
NADH
PI(3,5)P2
MCOLN2
?
SPHK2
S1P
Mitochondrion
MCU
Na+
SLC8A2
SLC25A4
VDAC1
PPIF
Ca2+
CALML6
TNNC2
MYLK4
PHKA1
PPP3CA
CAMK1D
NOS1
ADCY1
PDE1A
PTK2B
ITPKA
PRKCA
Contraction
Metabolism
MAPK signalng pathway
Apoptosis
Proliferation
Fertilization
Learning and memory
Long term potentiation
Long term depression
Other signaling pathways
Phosphatidylinositol signaling pathway
Exocytosis
Secretion
04020 7/15/20
(c) Kanehisa Laboratories

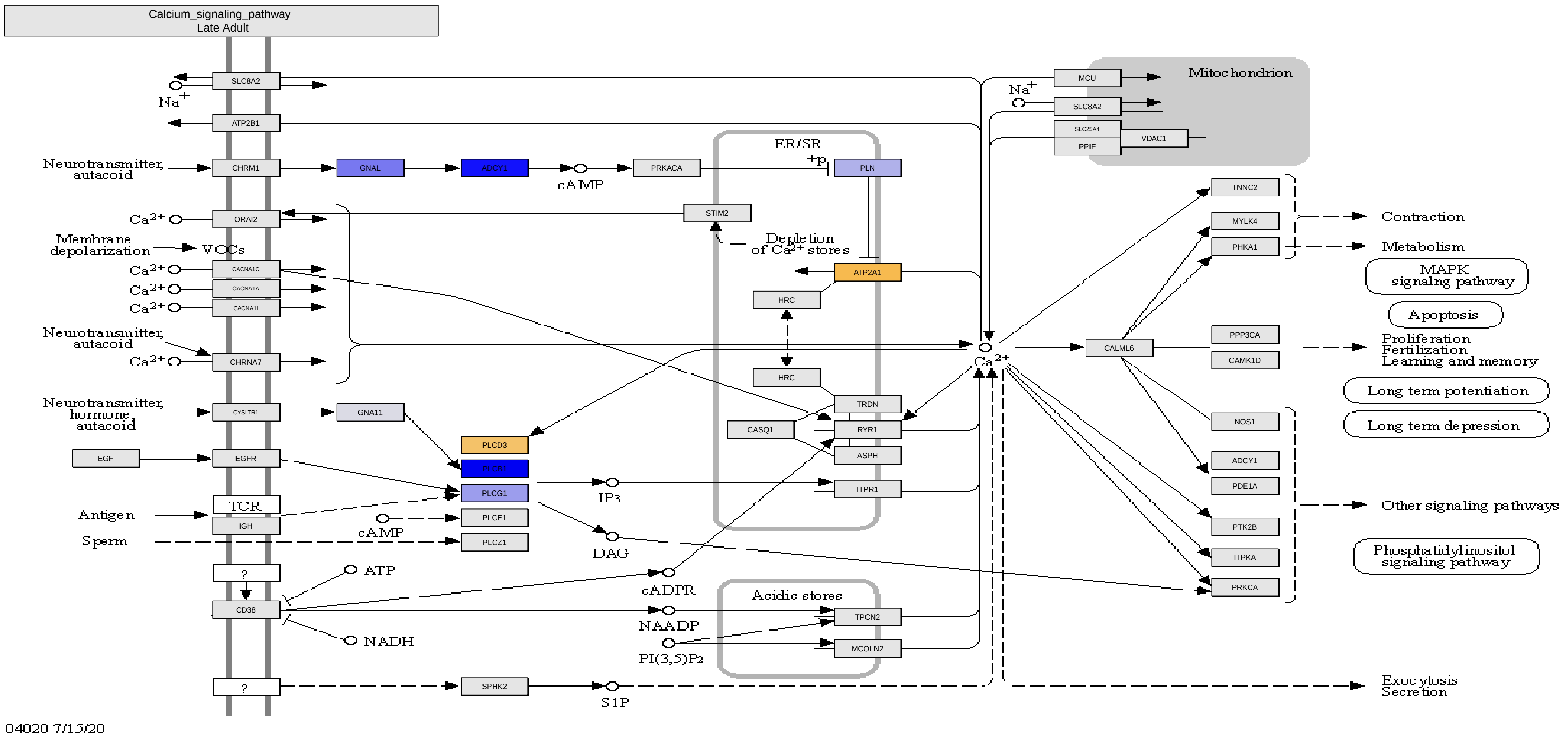

04020 7/15/20

(c) Kanehisa Laboratories
