# Supplementary material for: The Transcriptome and Methylome of the Developing and Aging Brain and Their Relations to Gliomas and Psychological Disorders
Source: Cells. 2022 Jan 21;11(3):362. doi: 10.3390/cells11030362 (PMC8834030; doi:10.3390/cells11030362)
Supplement: Supplementary file 1 [file cells-11-00362-s001.zip › Suppl-Material-S3-Pathways-PSF_Expression/cGMP_PKG_signaling_pathway.pdf]

# cGMP−PKG signaling pathway

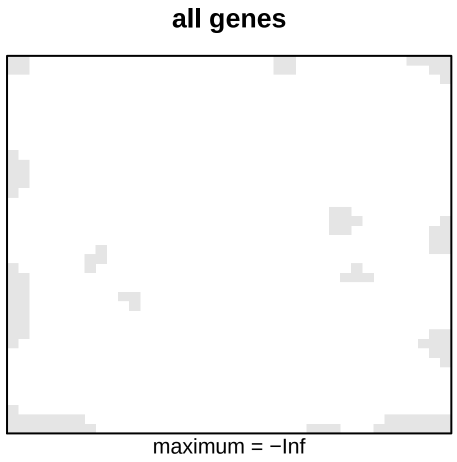


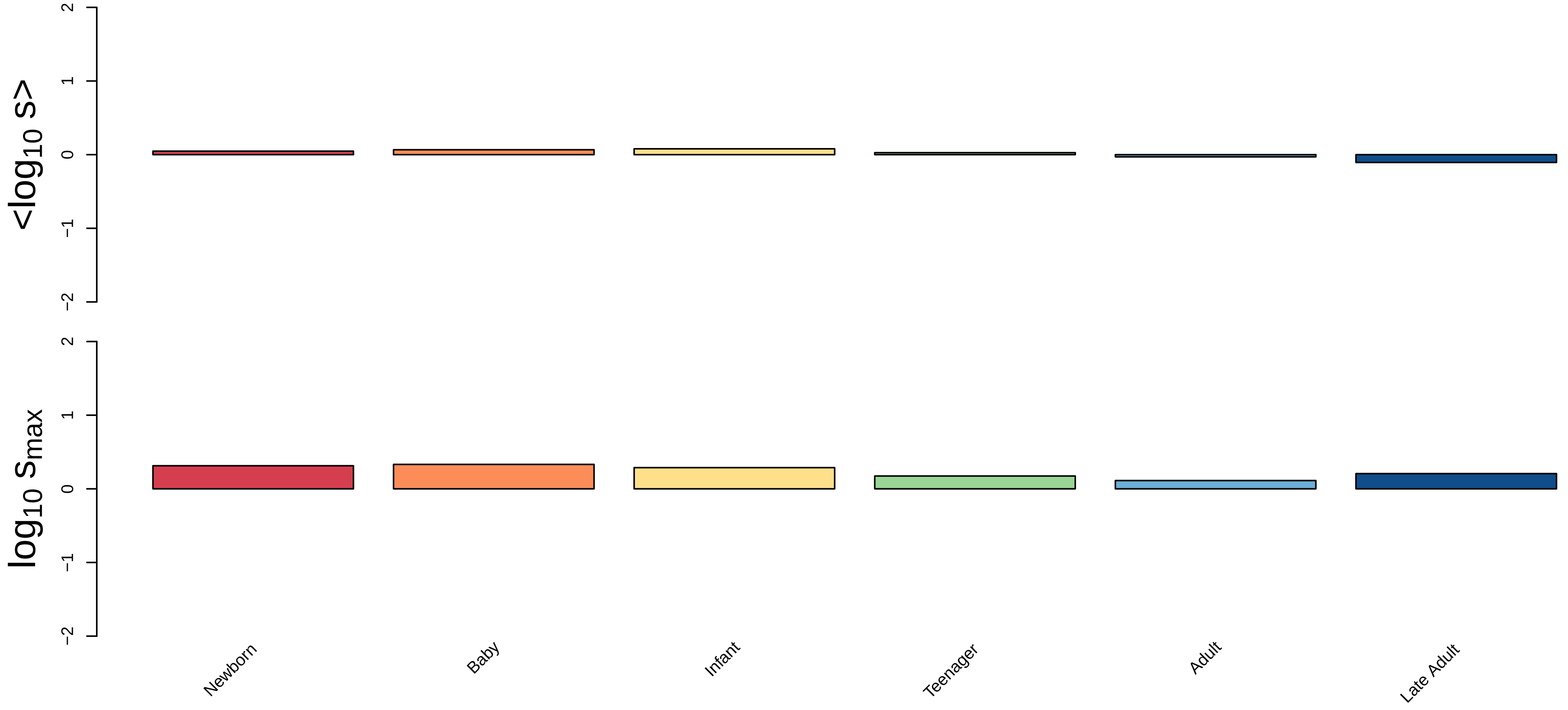

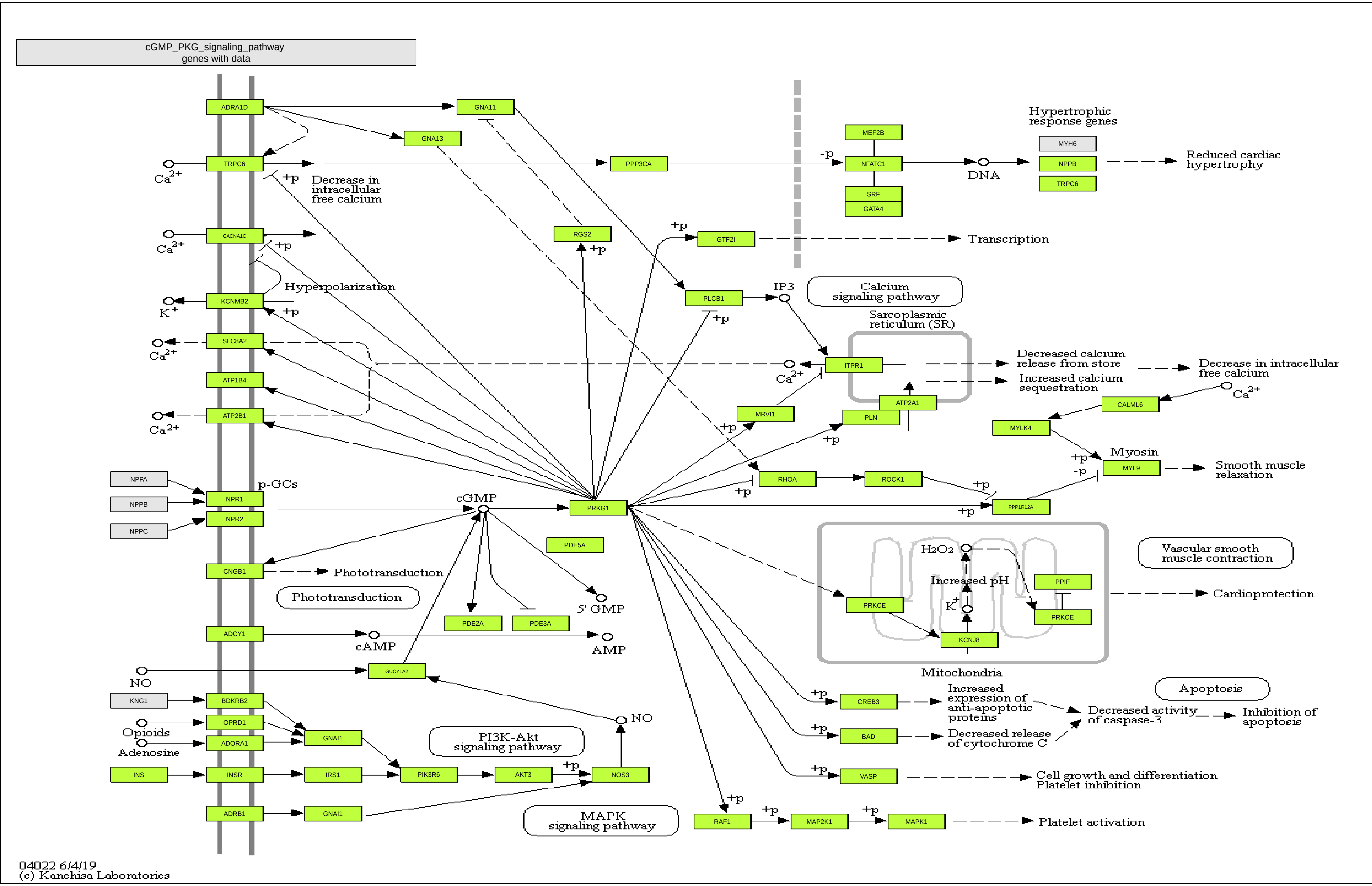
cGMP_PKG_signaling_pathway
genes with data
ADRA1D
GNA11
GNA13
TRPC6
Ca2+
Decrease in intracellular free calcium
PPP3CA
MEF2B
NFATC1
SRF
GATA4
-p
DNA
Hypertrophic response genes
MYH6
NPPB
TRPC6
Reduced cardiac hypertrophy
CACNA1C
RGS2
+p
GTF2I
Transcription
KCNMB2
K+
Hyperpolarization
PLCB1
IP3
Calcium signaling pathway
Sarcoplasmic reticulum (SR)
SLC8A2
ITPR1
Decreased calcium release from store
Increased calcium sequestration
Decrease in intracellular free calcium
ATP1B4
ATP2A1
MRVI1
PLN
CALML6
ATP2B1
MYLK4
Myosin
MYL9
Smooth muscle relaxation
NPPA
NPPB
NPPC
p-GCs
NPR1
NPR2
cGMP
PRKG1
RHOA
ROCK1
PPP1R12A
PDE5A
CNGB1
Phototransduction
PDE2A
PDE3A
5' GMP
H2O2
Increased pH
PPIF
PRKCE
KCNJ8
Mitochondria
Vascular smooth muscle contraction
Cardioprotection
ADCY1
cAMP
AMP
NO
GUCY1A2
KNG1
BDKRB2
OPRD1
Opioids
ADORA1
Adenosine
GNAI1
PI3K-Akt signaling pathway
CREB3
Increased expression of anti-apoptotic proteins
Decreased activity of caspase-3
Apoptosis
Inhibition of apoptosis
BAD
Decreased release of cytochrome C
INS
INSR
IRS1
PIK3R6
AKT3
NOS3
VASP
Cell growth and differentiation
Platelet inhibition
ADRB1
MAPK signaling pathway
RAF1
MAP2K1
MAPK1
Platelet activation
04022 6/4/19
(c) Kanehisa Laboratories

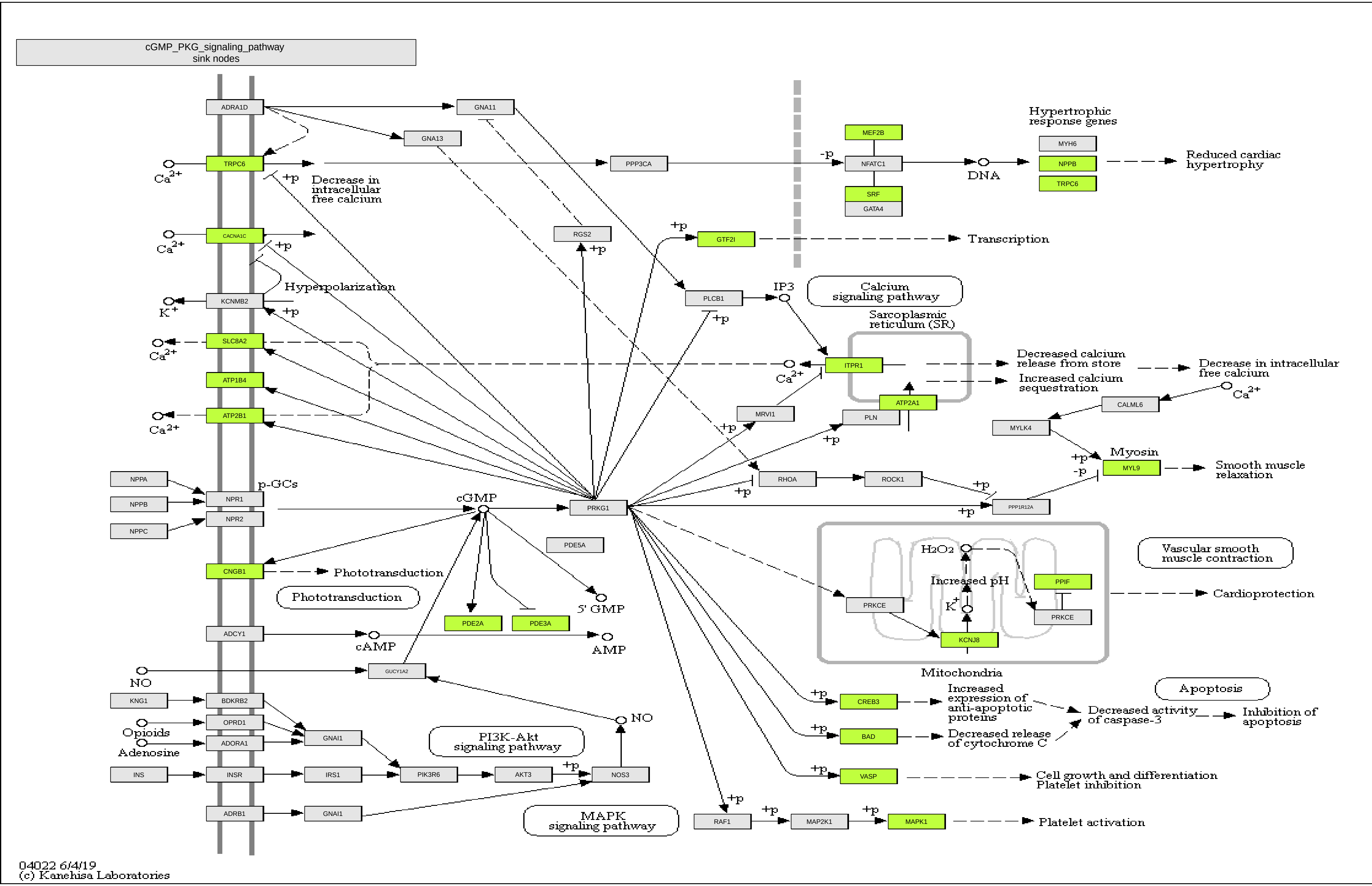

cGMP_PKG_signaling_pathway
sink nodes
ADRA1D
GNA11
GNA13
TRPC6
PPP3CA
MEF2B
NFATC1
SRF
GATA4
-p
DNA
Hypertrophic response genes
MYH6
NPPB
TRPC6
Reduced cardiac hypertrophy
Ca2+
+p
Decrease in intracellular free calcium
CACNA1C
RGS2
GTF2I
Transcription
KCNMB2
K+
Hyperpolarization
PLCB1
IP3
Calcium signaling pathway
Sarcoplasmic reticulum (SR)
SLC8A2
ITPR1
Decreased calcium release from store
Increased calcium sequestration
Decrease in intracellular free calcium
ATP1B4
ATP2A1
ATP2B1
MRVI1
PLN
CALML6
MYLK4
Myosin
MYL9
Smooth muscle relaxation
NPPA
NPPB
NPPC
NPR1
NPR2
p-GCs
cGMP
PRKG1
RHOA
ROCK1
PPP1R12A
PDE5A
H2O2
Increased pH
PPIF
PRKCE
KCNJ8
Mitochondria
Vascular smooth muscle contraction
Cardioprotection
CNGB1
Phototransduction
PDE2A
PDE3A
5' GMP
ADCY1
cAMP
AMP
NO
GUCY1A2
KNG1
BDKRB2
OPRD1
Opioids
ADORA1
Adenosine
GNAI1
PI3K-Akt signaling pathway
INS
INSR
IRS1
PIK3R6
AKT3
NOS3
ADRB1
MAPK signaling pathway
CREB3
Increased expression of anti-apoptotic proteins
Apoptosis
Decreased activity of caspase-3
Inhibition of apoptosis
BAD
Decreased release of cytochrome C
VASP
Cell growth and differentiation
Platelet inhibition
RAF1
MAP2K1
MAPK1
Platelet activation
04022 6/4/19
(c) Kanehisa Laboratories

cGMP_PKG_signaling_pathway
Newborn

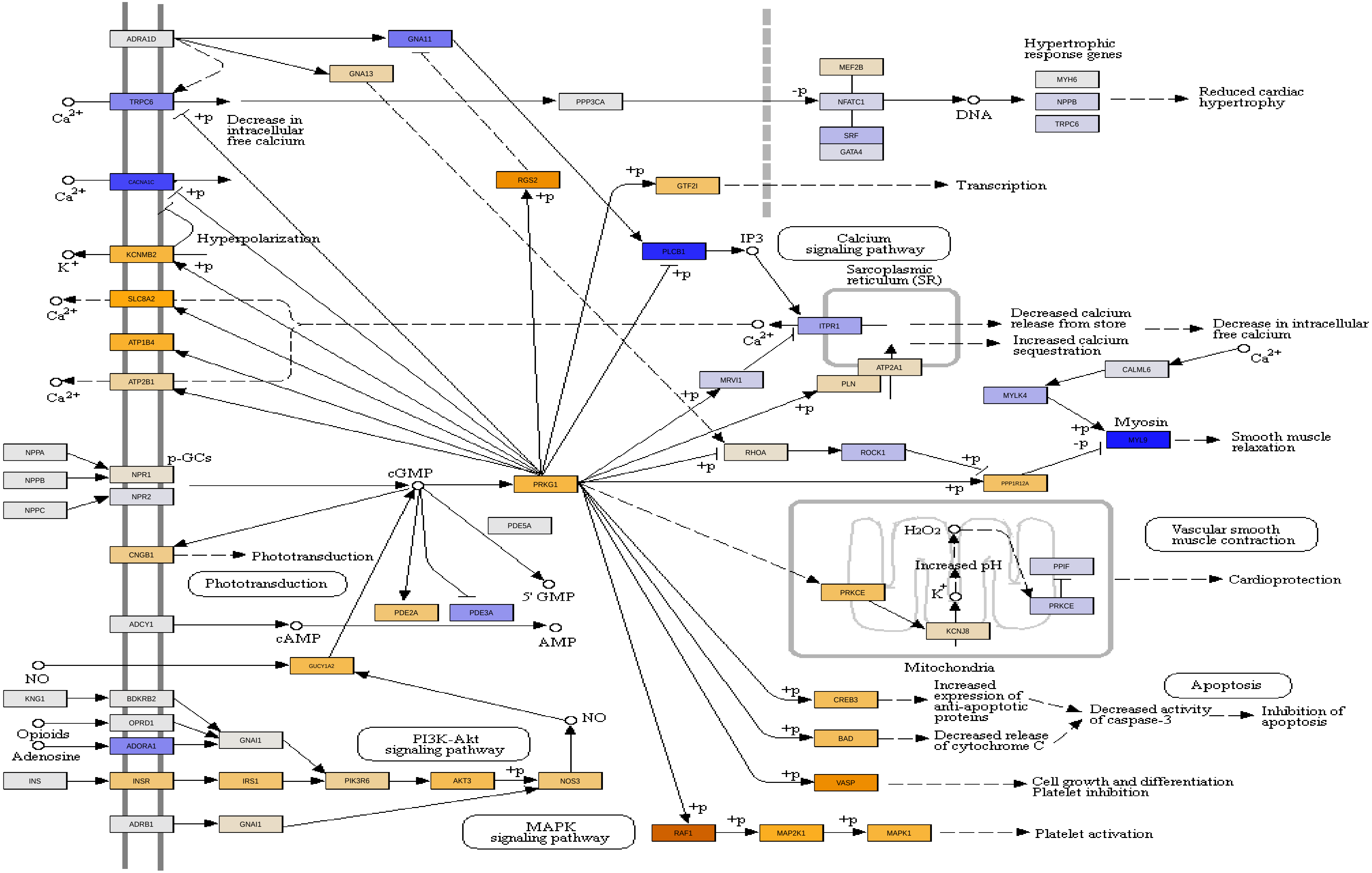


04022 6/4/19
(c) Kanehisa Laboratories

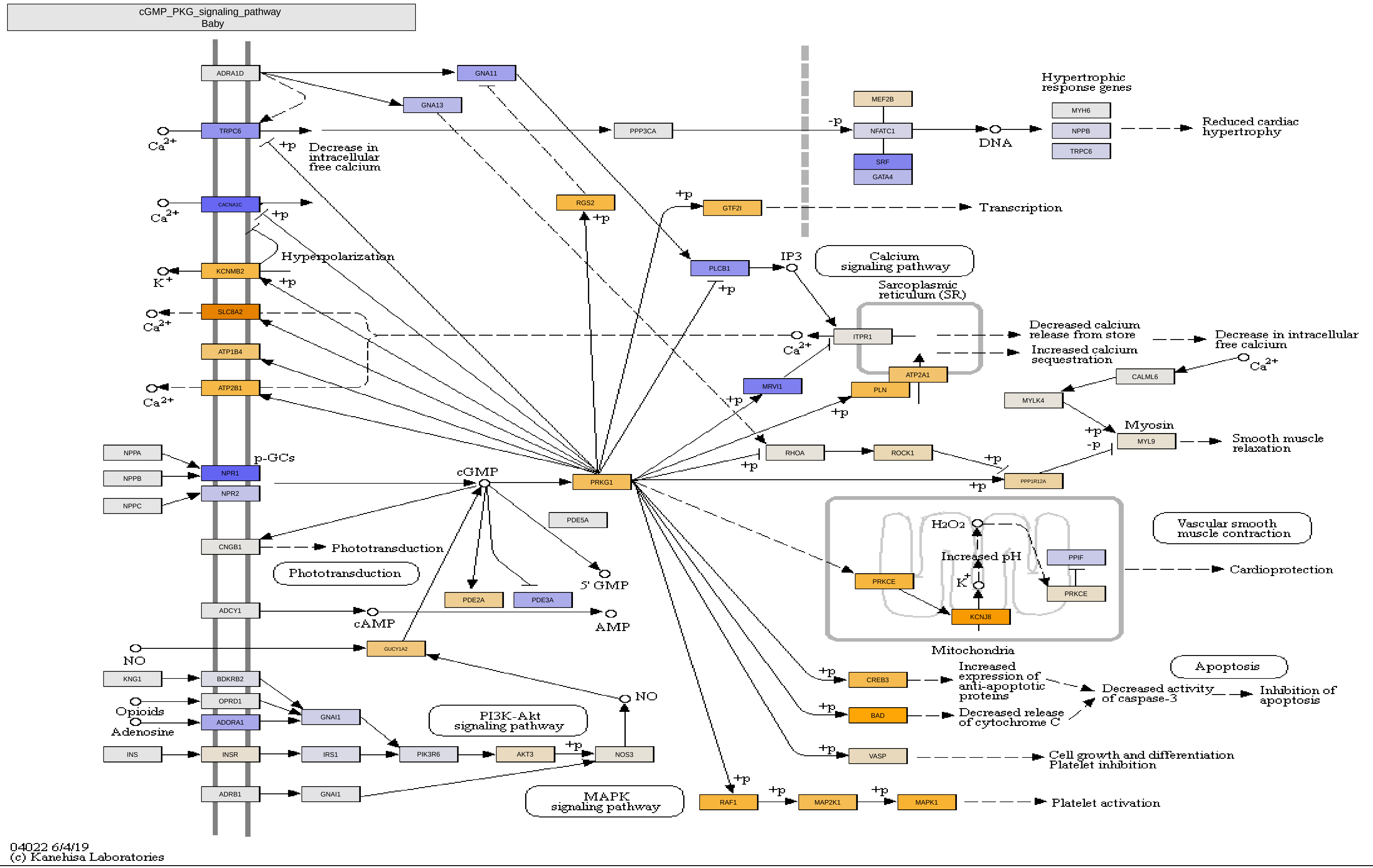

cGMP_PKG_signaling_pathway
Baby
ADRA1D
GNA11
GNA13
TRPC6
Ca2+
Decrease in intracellular free calcium
PPP3CA
MEF2B
NFATC1
SRF
GATA4
DNA
Hypertrophic response genes
MYH6
NPPB
TRPC6
Reduced cardiac hypertrophy
CACNA1C
RGS2
GTF2I
Transcription
KCNMB2
K+
Hyperpolarization
PLCB1
IP3
Calcium signaling pathway
Sarcoplasmic reticulum (SR)
SLC8A2
ATP1B4
ATP2B1
ITPR1
Decreased calcium release from store
Increased calcium sequestration
Decrease in intracellular free calcium
CALML6
ATP2A1
MRVI1
PLN
MYLK4
Myosin
MYL9
Smooth muscle relaxation
NPPA
NPPB
NPPC
NPR1
NPR2
p-GCs
cGMP
PRKG1
RHOA
ROCK1
PPP1R12A
PDE5A
CNGB1
Phototransduction
PDE2A
PDE3A
5' GMP
H2O2
Increased pH
PPIF
PRKCE
KCNJ8
Vascular smooth muscle contraction
Cardioprotection
Mitochondria
ADCY1
cAMP
AMP
NO
GUCY1A2
KNG1
BDKRB2
OPRD1
Opioids
Adenosine
ADORA1
GNAI1
PI3K-Akt signaling pathway
INS
INSR
IRS1
PIK3R6
AKT3
NOS3
ADRB1
MAPK signaling pathway
CREB3
BAD
VASP
Increased expression of anti-apoptotic proteins
Decreased release of cytochrome C
Decreased activity of caspase-3
Apoptosis
Inhibition of apoptosis
Cell growth and differentiation
Platelet inhibition
RAF1
MAP2K1
MAPK1
Platelet activation
04022 6/4/19
(c) Kanehisa Laboratories

cGMP_PKG_signaling_pathway
Infant

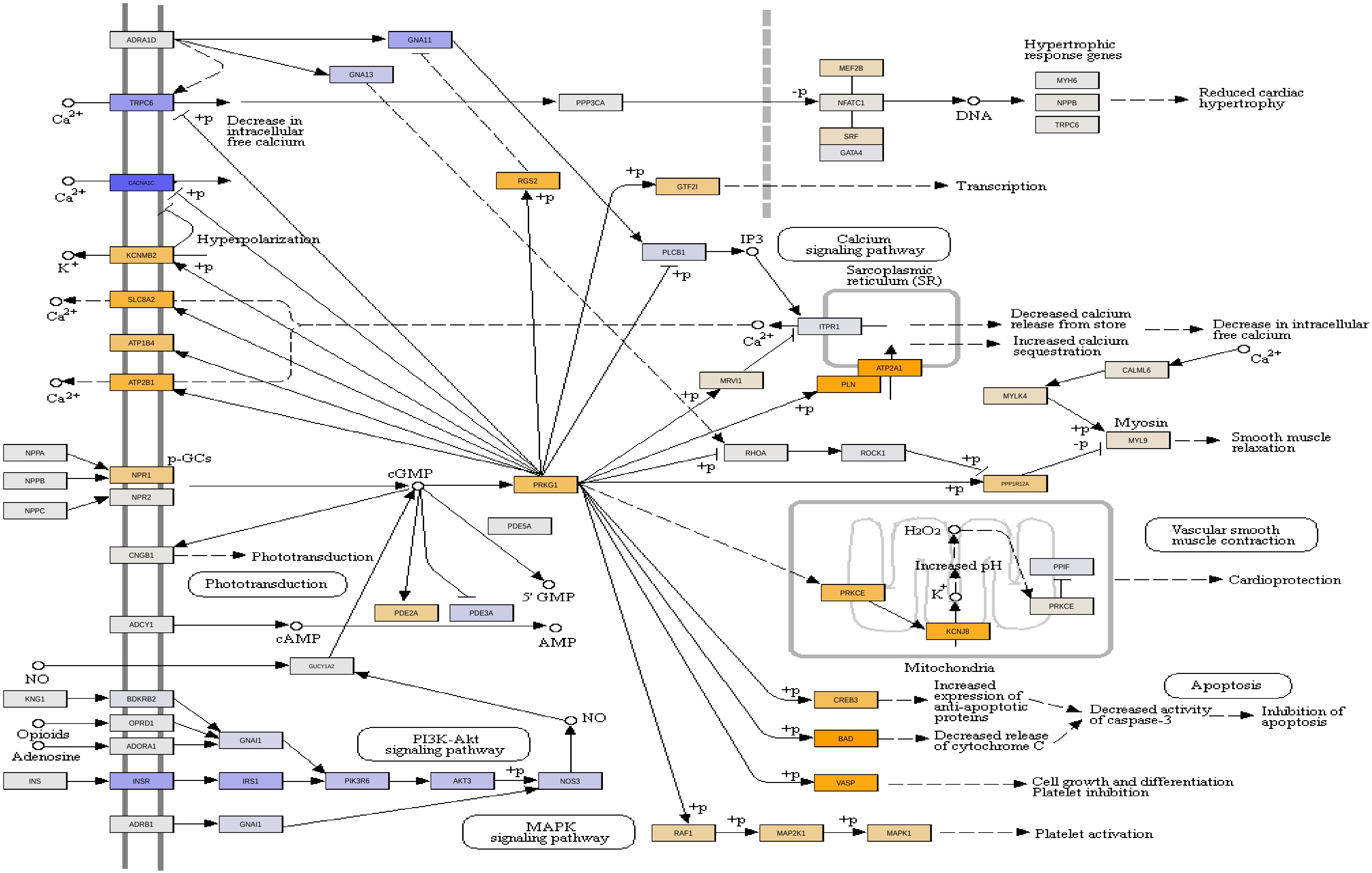


04022 6/4/19
(c) Kanehisa Laboratories

cGMP_PKG_signaling_pathway
Teenager

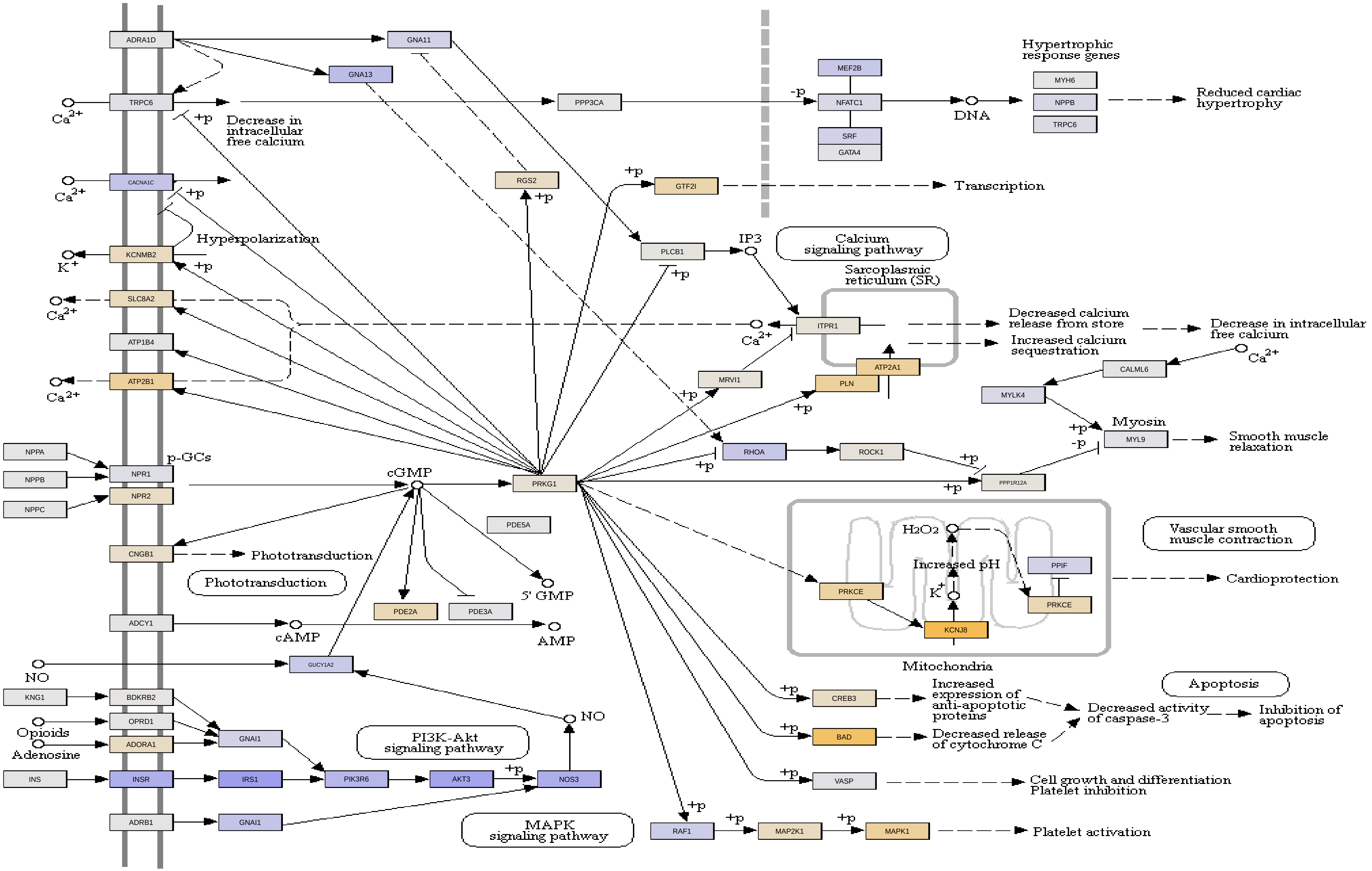


04022 6/4/19
(c) Kanehisa Laboratories

cGMP_PKG_signaling_pathway
Adult

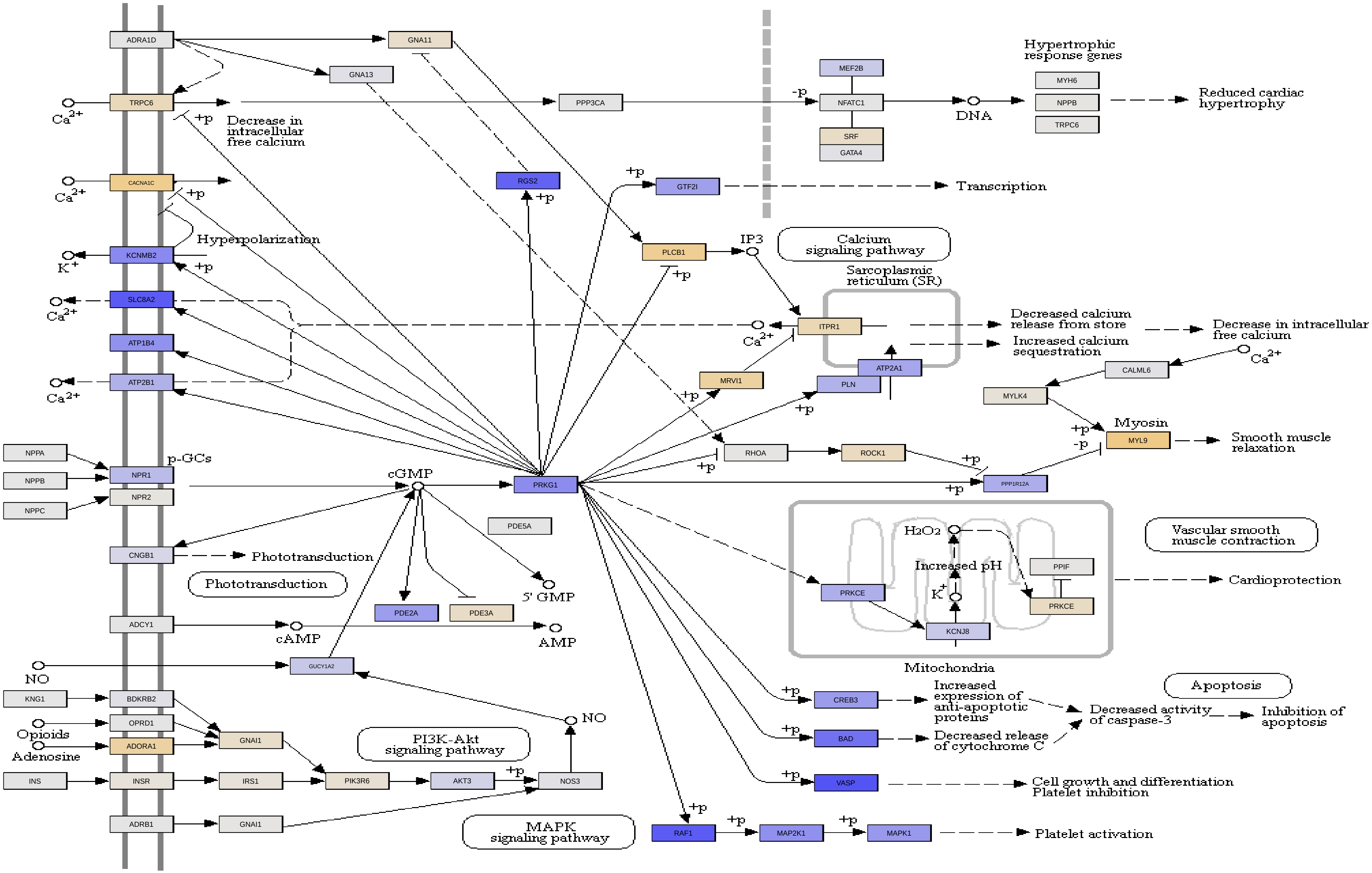


04022 6/4/19
(c) Kanehisa Laboratories

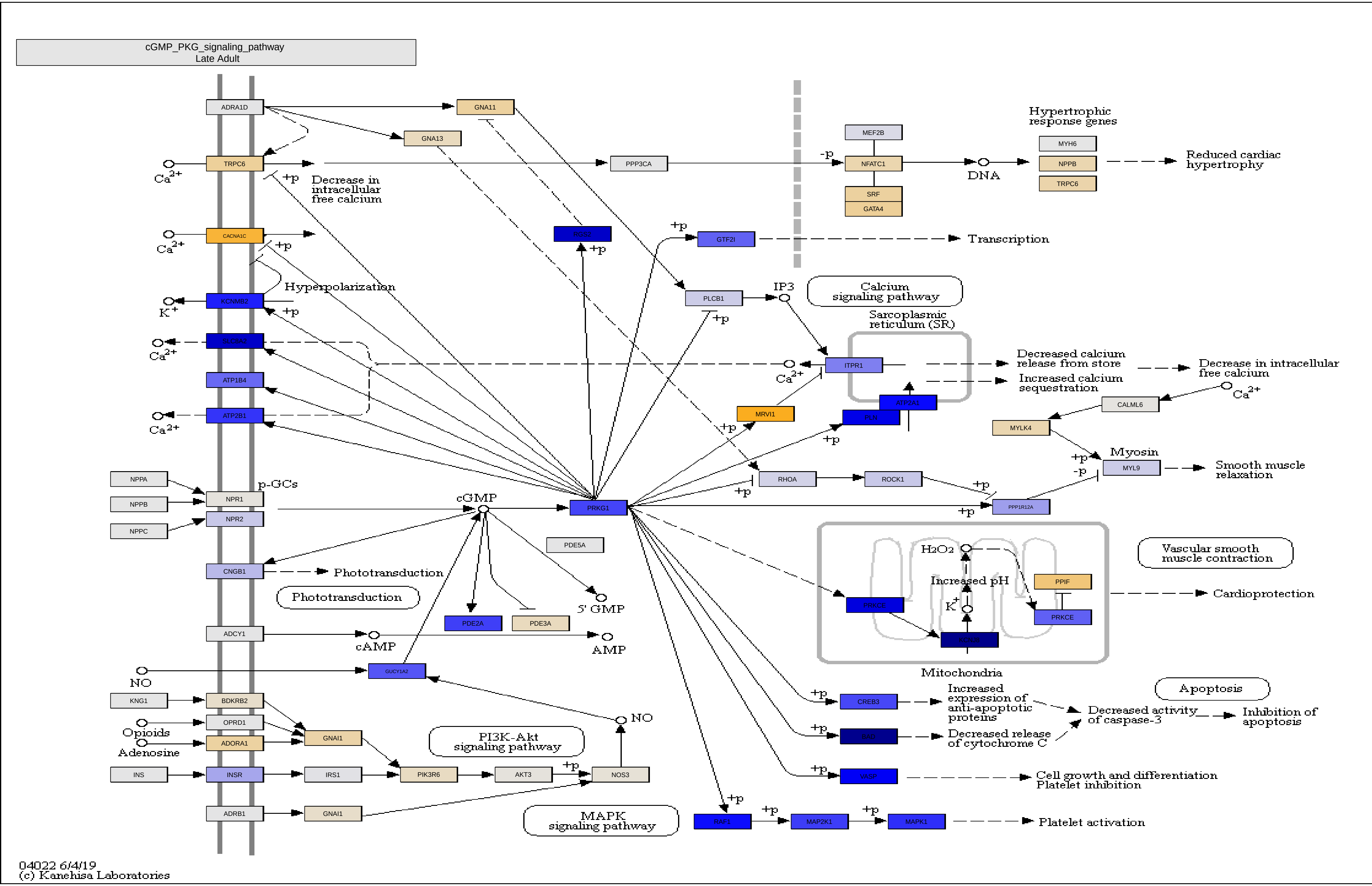
cGMP_PKG_signaling_pathway
Late Adult
ADRA1D
GNA11
GNA13
TRPC6
Ca2+
Decrease in intracellular free calcium
PPP3CA
MEF2B
NFATC1
SRF
GATA4
DNA
Hypertrophic response genes
MYH6
NPPB
TRPC6
Reduced cardiac hypertrophy
CACNA1C
RGS2
GTF2I
Transcription
KCNMB2
K+
Hyperpolarization
PLCB1
IP3
Calcium signaling pathway
Sarcoplasmic reticulum (SR)
SLC8A2
ITPR1
Decreased calcium release from store
Increased calcium sequestration
Decrease in intracellular free calcium
ATP1B4
ATP2A1
MRVI1
PLN
CALML6
ATP2B1
MYLK4
Myosin
MYL9
Smooth muscle relaxation
NPPA
NPPB
NPPC
NPR1
NPR2
p-GCs
cGMP
PRKG1
RHOA
ROCK1
PPP1R12A
+p
-p
PDE5A
CNGB1
Phototransduction
PDE2A
PDE3A
5' GMP
H2O2
Increased pH
PPIF
PRKCE
Mitochondria
Vascular smooth muscle contraction
Cardioprotection
ADCY1
cAMP
AMP
NO
GUCY1A2
KNG1
BDKRB2
OPRD1
Opioids
Adenosine
ADORA1
GNAI1
PI3K-Akt signaling pathway
INS
INSR
IRS1
PIK3R6
AKT3
NOS3
ADRB1
MAPK signaling pathway
CREB3
Increased expression of anti-apoptotic proteins
BAD
Decreased release of cytochrome C
Decreased activity of caspase-3
Apoptosis
Inhibition of apoptosis
VASP
Cell growth and differentiation
Platelet inhibition
RAF1
MAP2K1
MAPK1
Platelet activation
04022 6/4/19
(c) Kanehisa Laboratories
