# Supplementary material for: The Transcriptome and Methylome of the Developing and Aging Brain and Their Relations to Gliomas and Psychological Disorders
Source: Cells. 2022 Jan 21;11(3):362. doi: 10.3390/cells11030362 (PMC8834030; doi:10.3390/cells11030362)
Supplement: Supplementary file 1 [file cells-11-00362-s001.zip › Suppl-Material-S3-Pathways-PSF_Expression/ErbB_signaling_pathway.pdf]

ErbB signaling pathway

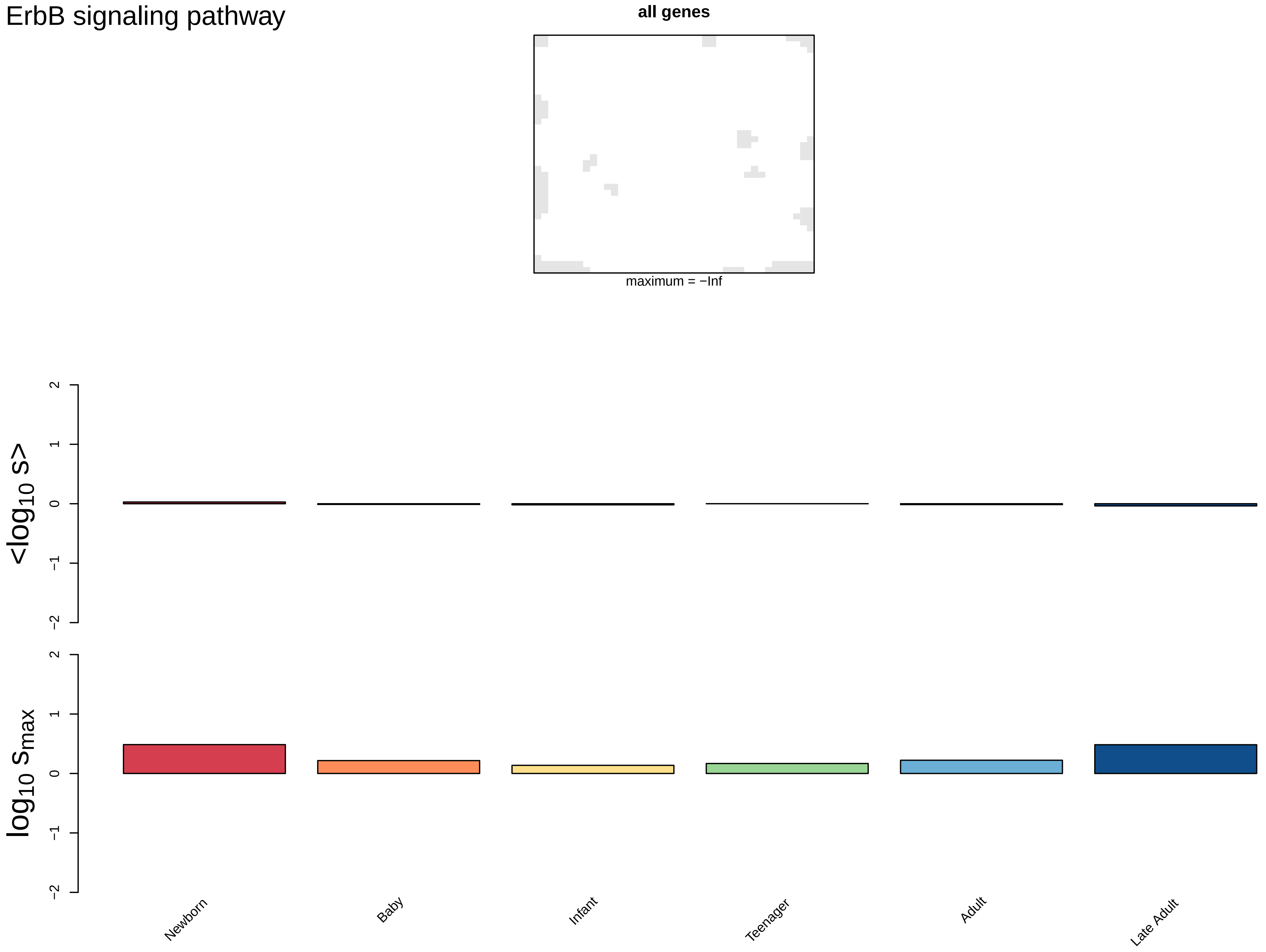

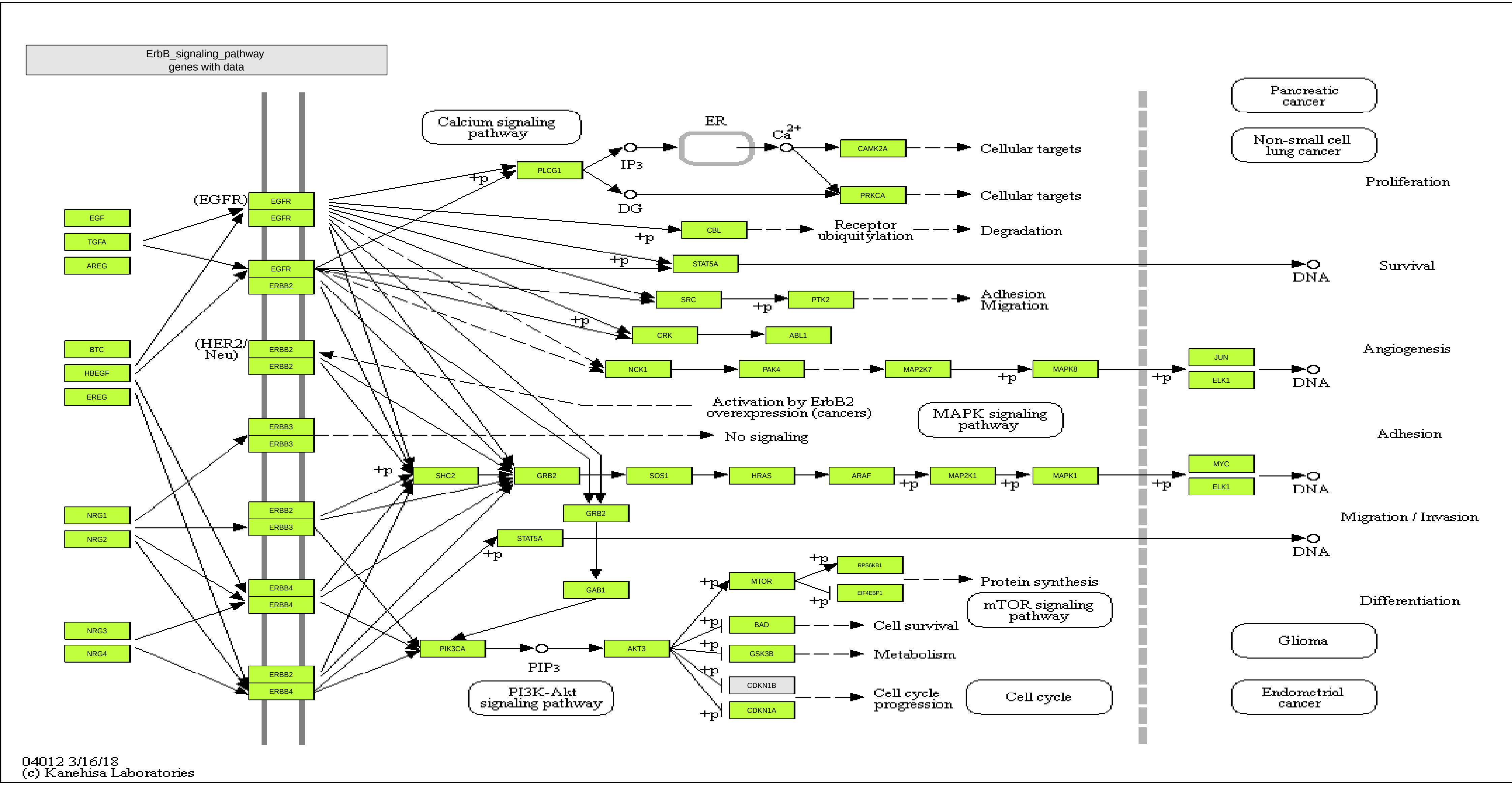

ErbB_signaling_pathway
genes with data
EGF
TGFA
AREG
BTC
HBEGF
EREG
NRG1
NRG2
NRG3
NRG4
(EGFR)
EGFR
EGFR
EGFR
ERBB2
(HER2/Neu)
ERBB2
ERBB2
ERBB3
ERBB3
ERBB2
ERBB3
ERBB4
ERBB4
ERBB2
ERBB4
Calcium signaling pathway
PLCG1
IP3
DG
ER
Ca2+
CAMK2A
PRKCA
Cellular targets
Cellular targets
CBL
Receptor ubiquitylation
Degradation
STAT5A
DNA
SRC
PTK2
Adhesion Migration
CRK
ABL1
NCK1
PAK4
MAP2K7
MAPK8
JUN
ELK1
DNA
Activation by ErbB2 overexpression (cancers)
MAPK signaling pathway
No signaling
SHC2
GRB2
SOS1
HRAS
ARAF
MAP2K1
MAPK1
MYC
ELK1
DNA
GRB2
STAT5A
DNA
GAB1
MTOR
RPS6KB1
EIF4EBP1
Protein synthesis
mTOR signaling pathway
PIK3CA
PIP3
AKT3
BAD
Cell survival
GSK3B
Metabolism
CDKN1B
CDKN1A
Cell cycle progression
Cell cycle
PI3K-Akt signaling pathway
+p
Pancreatic cancer
Non-small cell lung cancer
Proliferation
Survival
Angiogenesis
Adhesion
Migration / Invasion
Differentiation
Glioma
Endometrial cancer
04012 3/16/18
(c) Kanehisa Laboratories

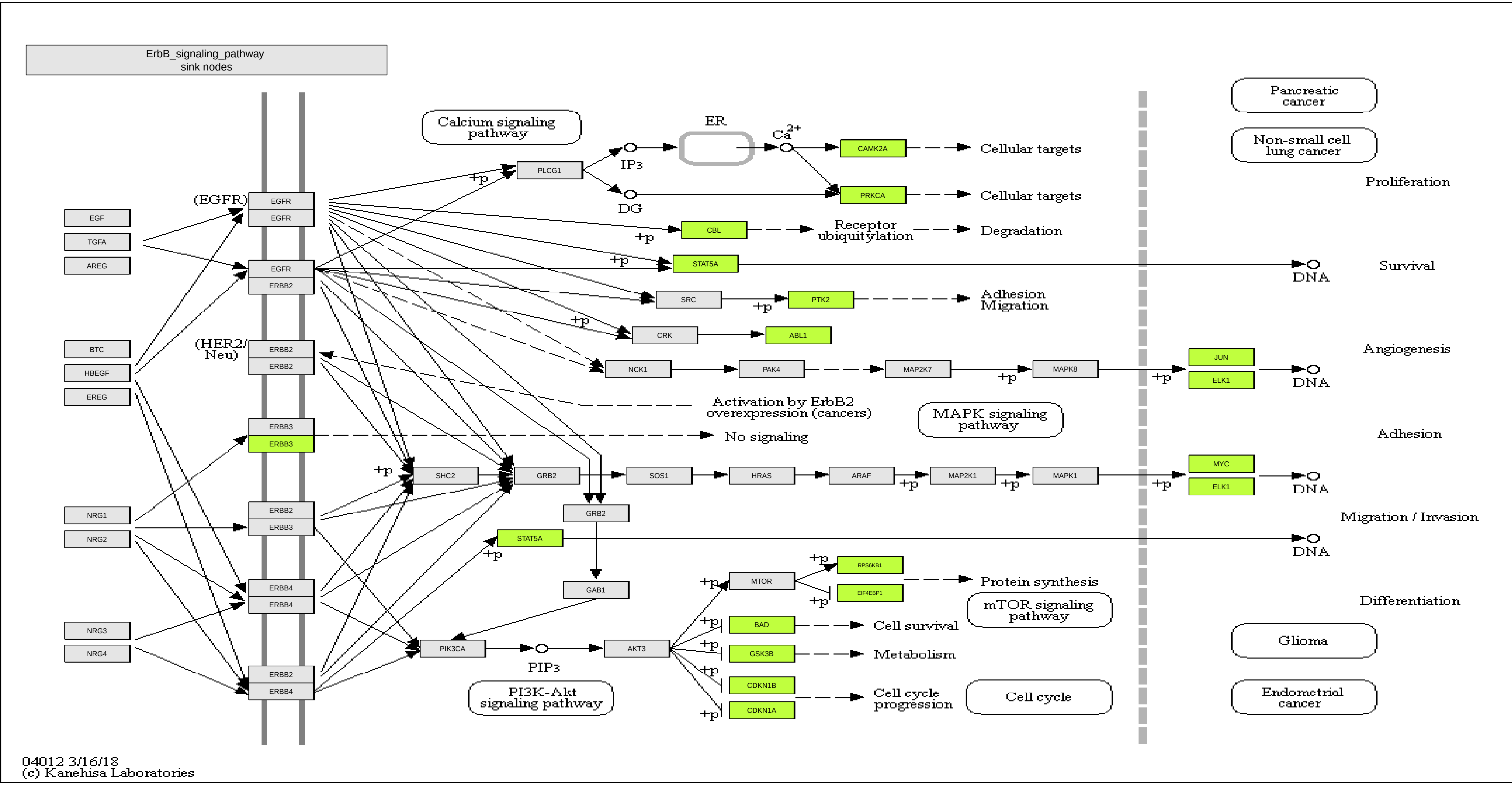

ErbB_signaling_pathway
sink nodes
EGF
TGFA
AREG
BTC
HBEGF
EREG
NRG1
NRG2
NRG3
NRG4
(EGFR)
EGFR
EGFR
EGFR
ERBB2
(HER2/
Neu)
ERBB2
ERBB2
ERBB3
ERBB3
ERBB2
ERBB3
ERBB4
ERBB4
ERBB2
ERBB4
Calcium signaling
pathway
PLCG1
IP3
DG
ER
Ca2+
CAMK2A
PRKCA
Cellular targets
Cellular targets
CBL
Receptor
ubiquitylation
Degradation
STAT5A
DNA
SRC
PTK2
Adhesion
Migration
CRK
ABL1
NCK1
PAK4
MAP2K7
MAPK8
JUN
ELK1
DNA
Activation by ErbB2
overexpression (cancers)
MAPK signaling
pathway
No signaling
SHC2
GRB2
SOS1
HRAS
ARAF
MAP2K1
MAPK1
MYC
ELK1
DNA
GRB2
STAT5A
DNA
GAB1
MTOR
RPS6KB1
EIF4EBP1
Protein synthesis
mTOR signaling
pathway
BAD
Cell survival
GSK3B
Metabolism
CDKN1B
CDKN1A
Cell cycle
progression
Cell cycle
PIK3CA
PIP3
AKT3
PI3K-Akt
signaling pathway
+p
Pancreatic
cancer
Non-small cell
lung cancer
Proliferation
Survival
Angiogenesis
Adhesion
Migration / Invasion
Differentiation
Glioma
Endometrial
cancer
04012 3/16/18
(c) Kanehisa Laboratories

ErbB_signaling_pathway
Newborn

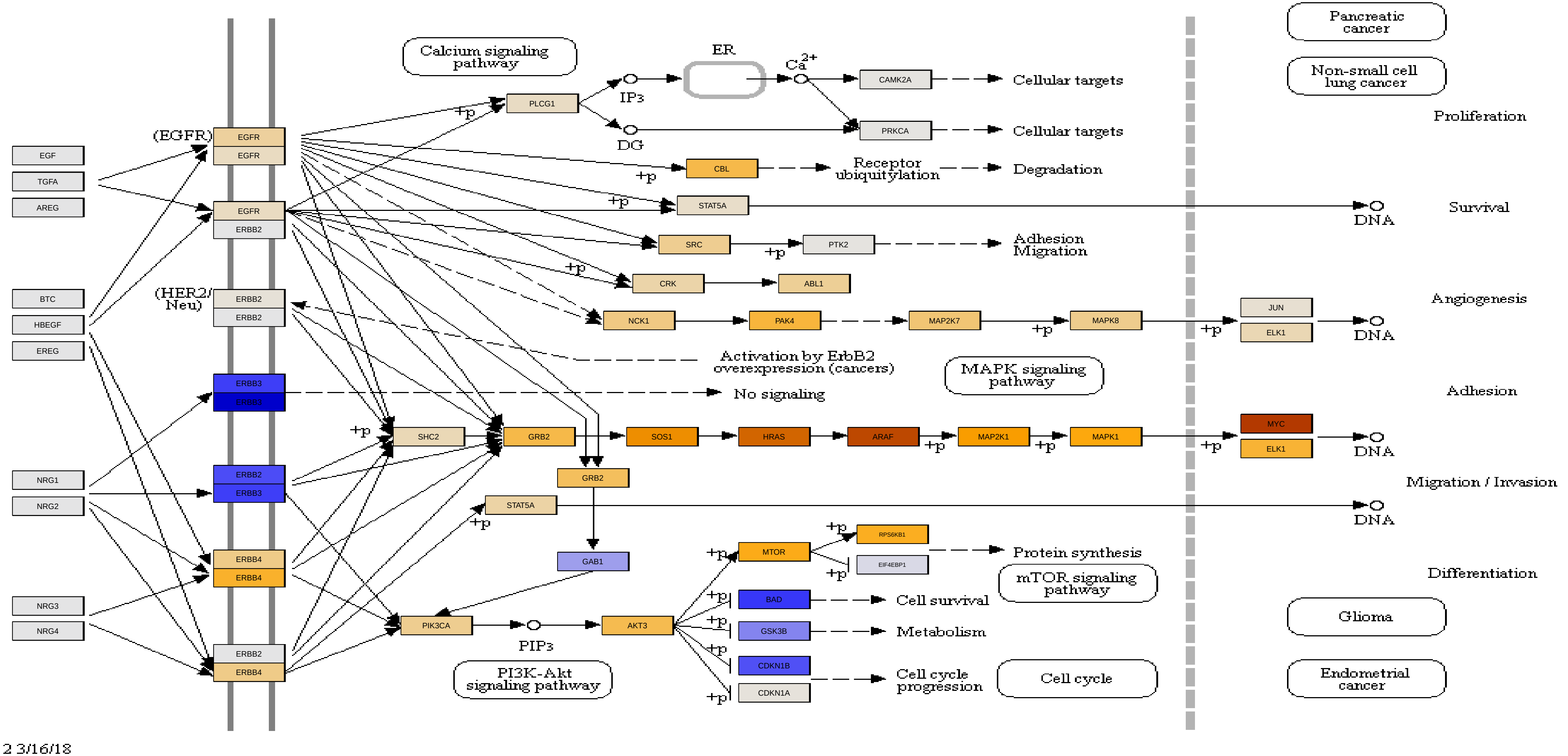

(c) Kanehisa Laboratories

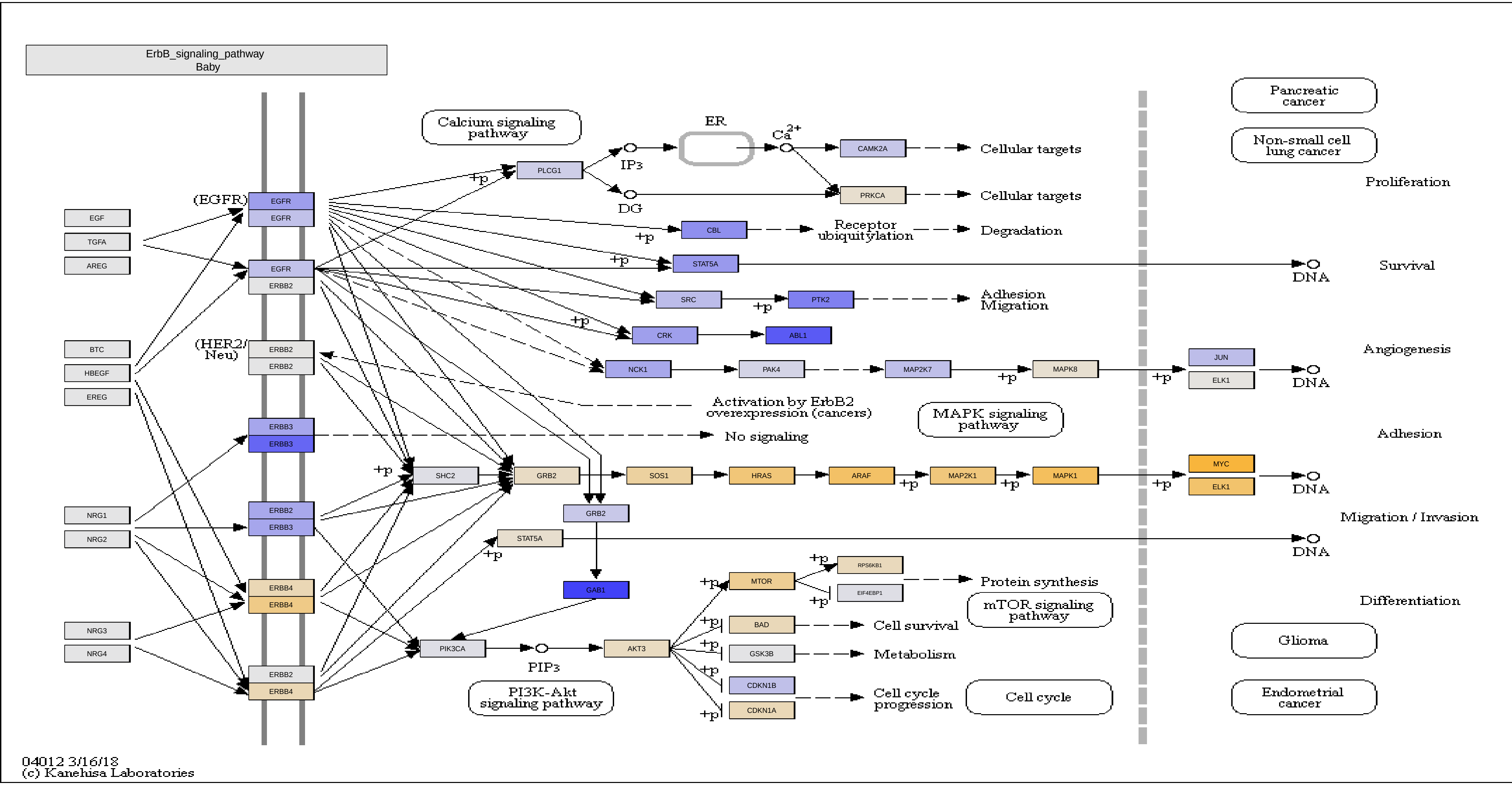
ErbB_signaling_pathway
Baby
(EGFR)
EGFR
EGFR
EGFR
ERBB2
(HER2/ Neu)
ERBB2
ERBB2
ERBB3
ERBB3
ERBB2
ERBB3
ERBB4
ERBB4
ERBB2
ERBB4
EGF
TGFA
AREG
BTC
HBEGF
EREG
NRG1
NRG2
NRG3
NRG4
Calcium signaling pathway
PLCG1
IP3
DG
ER
Ca2+
CAMK2A
PRKCA
Cellular targets
Cellular targets
CBL
Receptor ubiquitylation
Degradation
STAT5A
DNA
SRC
PTK2
Adhesion Migration
CRK
ABL1
NCK1
PAK4
MAP2K7
MAPK8
JUN
ELK1
DNA
Activation by ErbB2 overexpression (cancers)
MAPK signaling pathway
No signaling
SHC2
GRB2
SOS1
HRAS
ARAF
MAP2K1
MAPK1
MYC
ELK1
DNA
GRB2
STAT5A
DNA
GAB1
MTOR
RPS6KB1
EIF4EBP1
Protein synthesis
mTOR signaling pathway
BAD
Cell survival
PIK3CA
PIP3
AKT3
GSK3B
Metabolism
CDKN1B
CDKN1A
Cell cycle progression
Cell cycle
PI3K-Akt signaling pathway
+p
Pancreatic cancer
Non-small cell lung cancer
Proliferation
Survival
Angiogenesis
Adhesion
Migration / Invasion
Differentiation
Glioma
Endometrial cancer
04012 3/16/18
(c) Kanehisa Laboratories

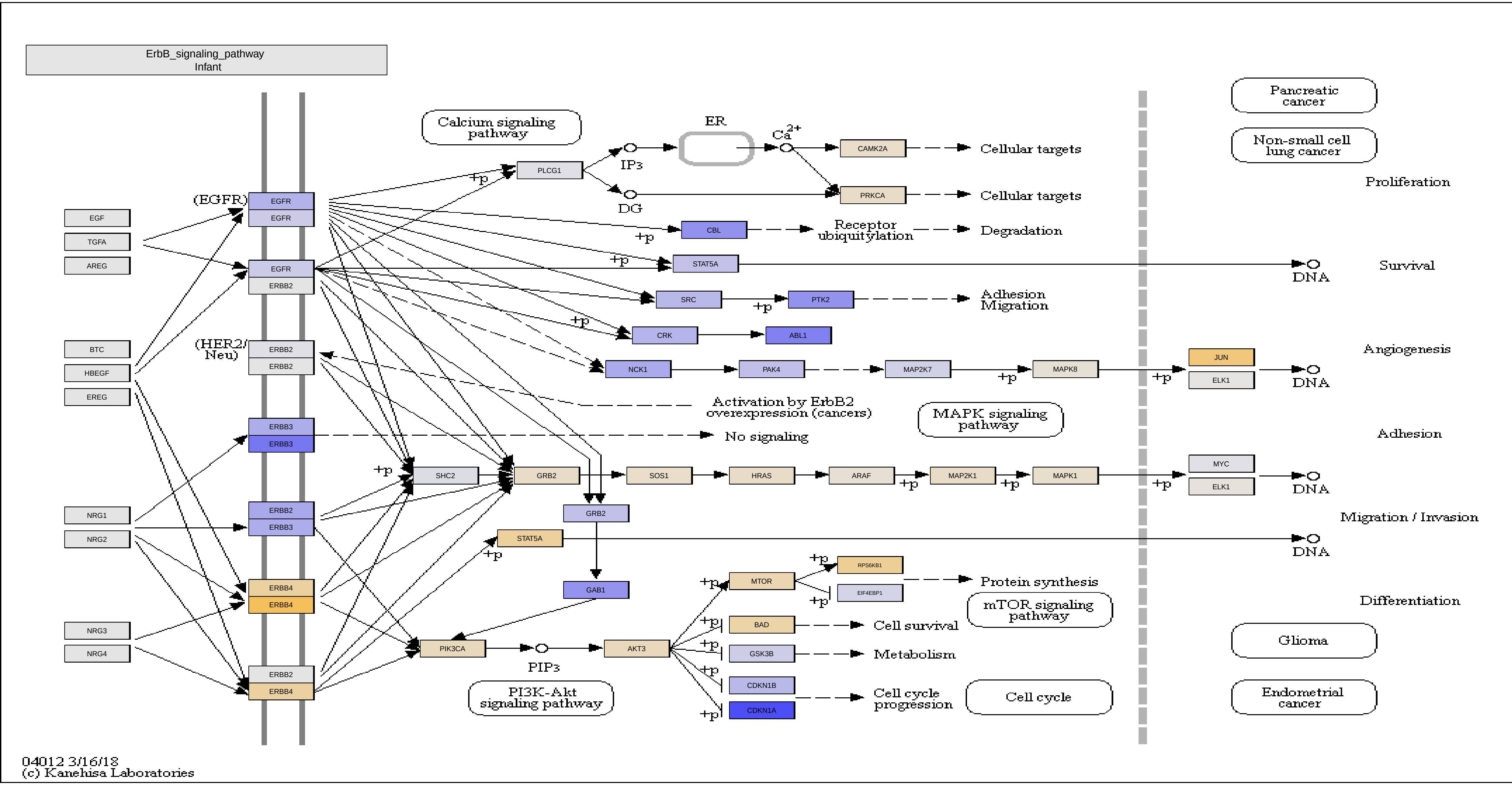
ErbB_signaling_pathway
Infant
EGF
TGFA
AREG
BTC
HBEGF
EREG
NRG1
NRG2
NRG3
NRG4
(EGFR)
EGFR
EGFR
EGFR
ERBB2
(HER2/ Neu)
ERBB2
ERBB2
ERBB3
ERBB3
ERBB2
ERBB3
ERBB4
ERBB4
ERBB2
ERBB4
Calcium signaling pathway
PLCG1
IP3
DG
ER
Ca2+
CAMK2A
PRKCA
Cellular targets
Cellular targets
CBL
Receptor ubiquitylation
Degradation
STAT5A
DNA
SRC
PTK2
Adhesion Migration
CRK
ABL1
NCK1
PAK4
MAP2K7
MAPK8
JUN
ELK1
DNA
Activation by ErbB2 overexpression (cancers)
MAPK signaling pathway
No signaling
SHC2
GRB2
SOS1
HRAS
ARAF
MAP2K1
MAPK1
MYC
ELK1
DNA
GRB2
STAT5A
DNA
GAB1
MTOR
RPS6KB1
EIF4EBP1
Protein synthesis
mTOR signaling pathway
BAD
Cell survival
GSK3B
Metabolism
CDKN1B
CDKN1A
Cell cycle progression
Cell cycle
PIK3CA
PIP3
AKT3
PI3K-Akt signaling pathway
+p
Pancreatic cancer
Non-small cell lung cancer
Proliferation
Survival
Angiogenesis
Adhesion
Migration / Invasion
Differentiation
Glioma
Endometrial cancer
04012 3/16/18
(c) Kanehisa Laboratories

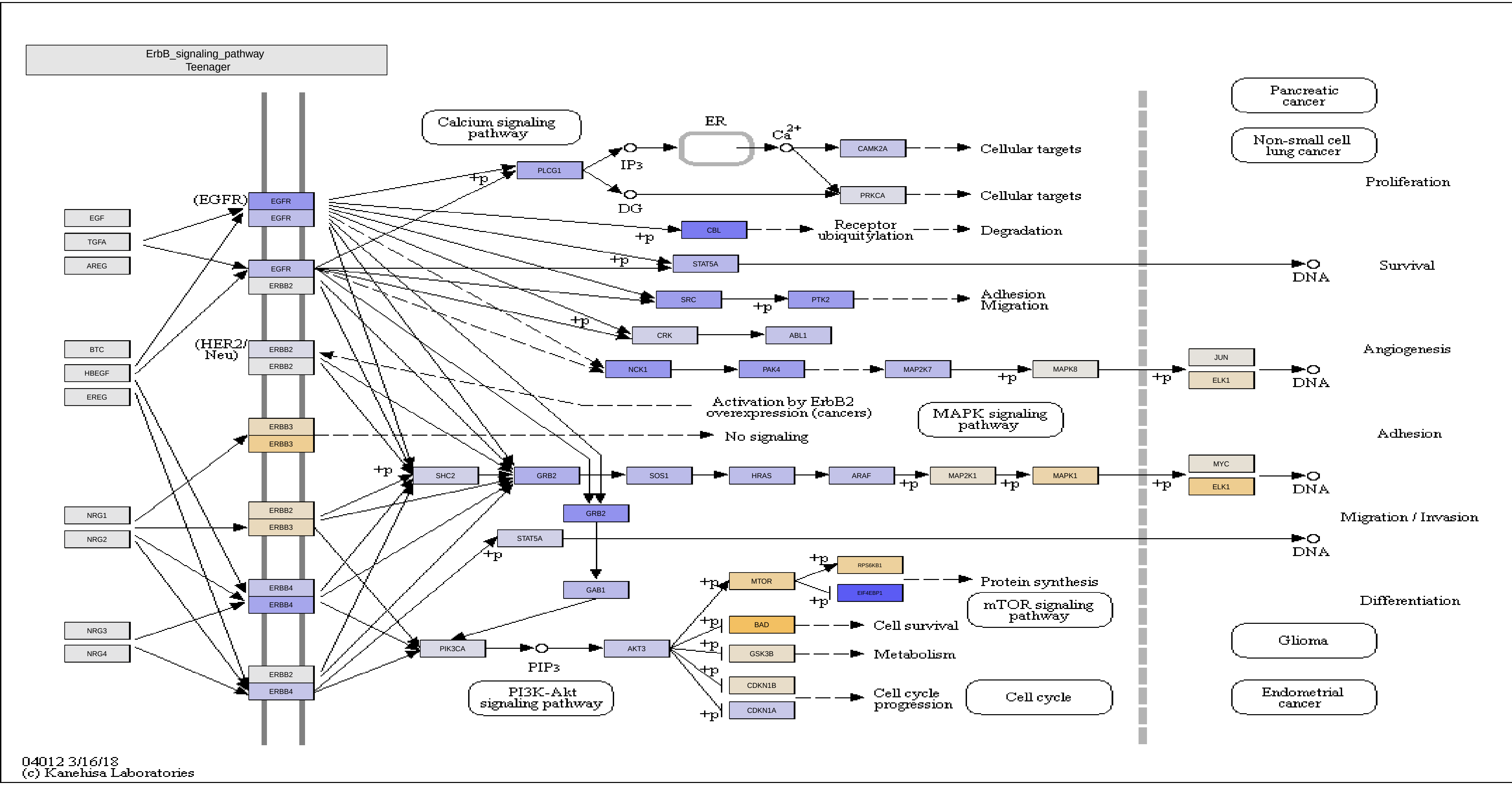

ErbB_signaling_pathway
Teenager
Calcium signaling pathway
ER
IP3
DG
Ca^{2+}
PLCG1
CAMK2A
PRKCA
Cellular targets
Cellular targets
Pancreatic cancer
Non-small cell lung cancer
Proliferation
(EGFR)
EGFR
EGFR
EGFR
ERBB2
EGF
TGFA
AREG
CBL
Receptor ubiquitylation
Degradation
STAT5A
DNA
Survival
SRC
PTK2
Adhesion
Migration
CRK
ABL1
(HER2/ Neu)
ERBB2
ERBB2
BTC
HBEGF
EREG
NCK1
PAK4
MAP2K7
MAPK8
JUN
ELK1
DNA
Angiogenesis
Activation by ErbB2 overexpression (cancers)
MAPK signaling pathway
ERBB3
ERBB3
No signaling
Adhesion
SHC2
GRB2
SOS1
HRAS
ARAF
MAP2K1
MAPK1
MYC
ELK1
DNA
NRG1
NRG2
ERBB2
ERBB3
GRB2
STAT5A
DNA
Migration / Invasion
GAB1
MTOR
RPS6KB1
EIF4EBP1
Protein synthesis
mTOR signaling pathway
Differentiation
ERBB4
ERBB4
NRG3
NRG4
BAD
Cell survival
Glioma
PIK3CA
PIP3
AKT3
GSK3B
Metabolism
ERBB2
ERBB4
CDKN1B
CDKN1A
Cell cycle progression
Cell cycle
Endometrial cancer
PI3K-Akt signaling pathway
+p
04012 3/16/18
(c) Kanehisa Laboratories

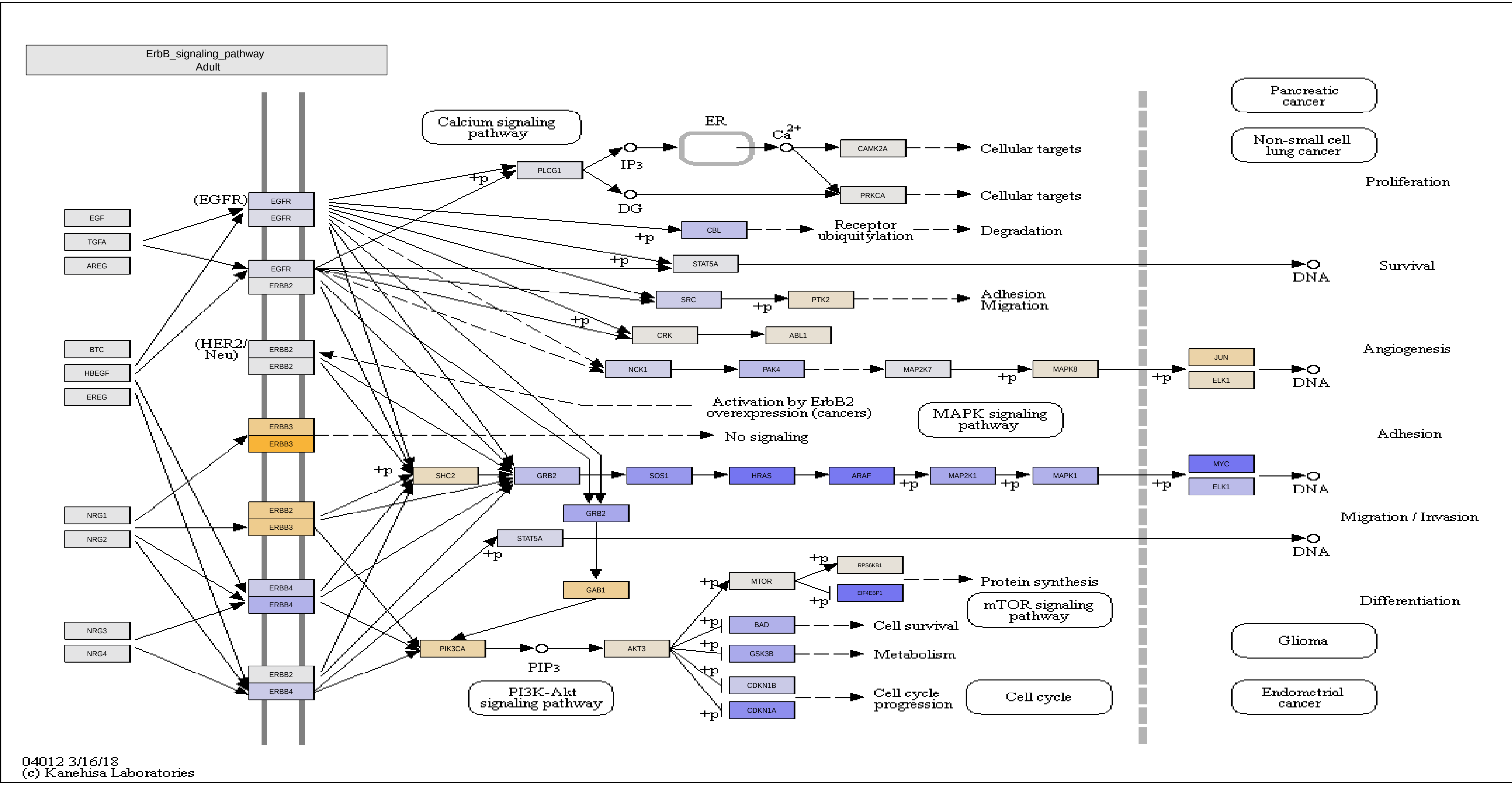
ErbB_signaling_pathway
Adult
EGF
TGFA
AREG
BTC
HBEGF
EREG
NRG1
NRG2
NRG3
NRG4
(EGFR)
EGFR
EGFR
EGFR
ERBB2
(HER2/Neu)
ERBB2
ERBB2
ERBB3
ERBB3
ERBB2
ERBB3
ERBB4
ERBB4
ERBB2
ERBB4
Calcium signaling pathway
PLCG1
IP3
DG
ER
Ca2+
CAMK2A
PRKCA
Cellular targets
Cellular targets
CBL
Receptor ubiquitylation
Degradation
STAT5A
DNA
SRC
PTK2
Adhesion Migration
CRK
ABL1
NCK1
PAK4
MAP2K7
MAPK8
JUN
ELK1
DNA
Activation by ErbB2 overexpression (cancers)
MAPK signaling pathway
No signaling
SHC2
GRB2
SOS1
HRAS
ARAF
MAP2K1
MAPK1
MYC
ELK1
DNA
GRB2
STAT5A
DNA
GAB1
MTOR
RPS6KB1
EIF4EBP1
Protein synthesis
mTOR signaling pathway
BAD
Cell survival
GSK3B
Metabolism
CDKN1B
CDKN1A
Cell cycle progression
Cell cycle
PIK3CA
PIP3
AKT3
PI3K-Akt signaling pathway
+p
Pancreatic cancer
Non-small cell lung cancer
Proliferation
Survival
Angiogenesis
Adhesion
Migration / Invasion
Differentiation
Glioma
Endometrial cancer
04012 3/16/18
(c) Kanehisa Laboratories

ErbB_signaling_pathway
Late Adult

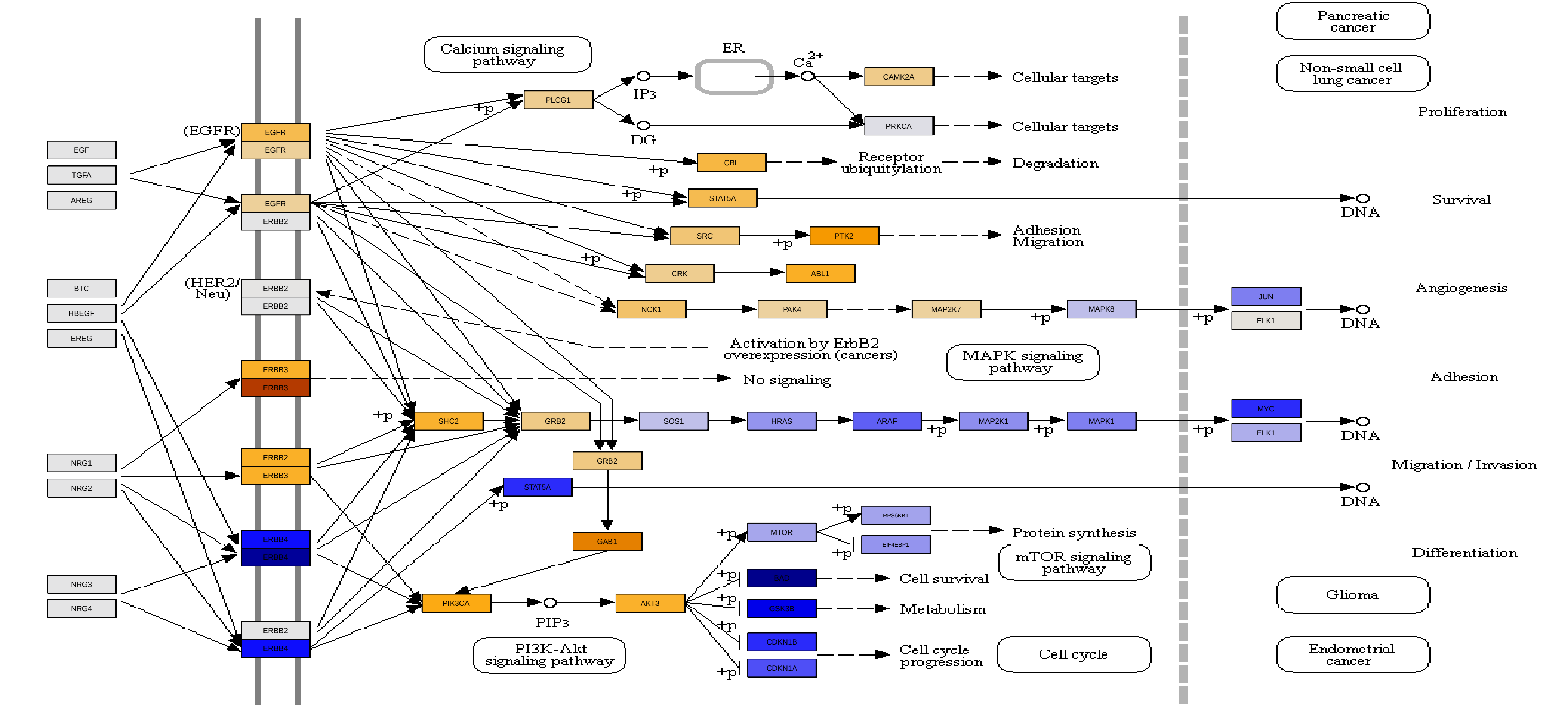

04012 3/16/18
(c) Kanehisa Laboratories
