# Supplementary material for: The Transcriptome and Methylome of the Developing and Aging Brain and Their Relations to Gliomas and Psychological Disorders
Source: Cells. 2022 Jan 21;11(3):362. doi: 10.3390/cells11030362 (PMC8834030; doi:10.3390/cells11030362)
Supplement: Supplementary file 1 [file cells-11-00362-s001.zip › Suppl-Material-S3-Pathways-PSF_Expression/Estrogen_signaling_pathway.pdf]

Estrogen signaling pathway

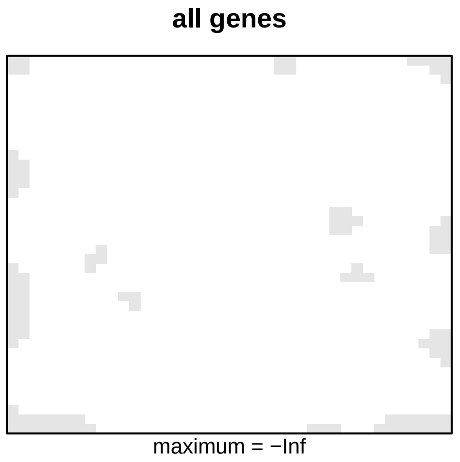

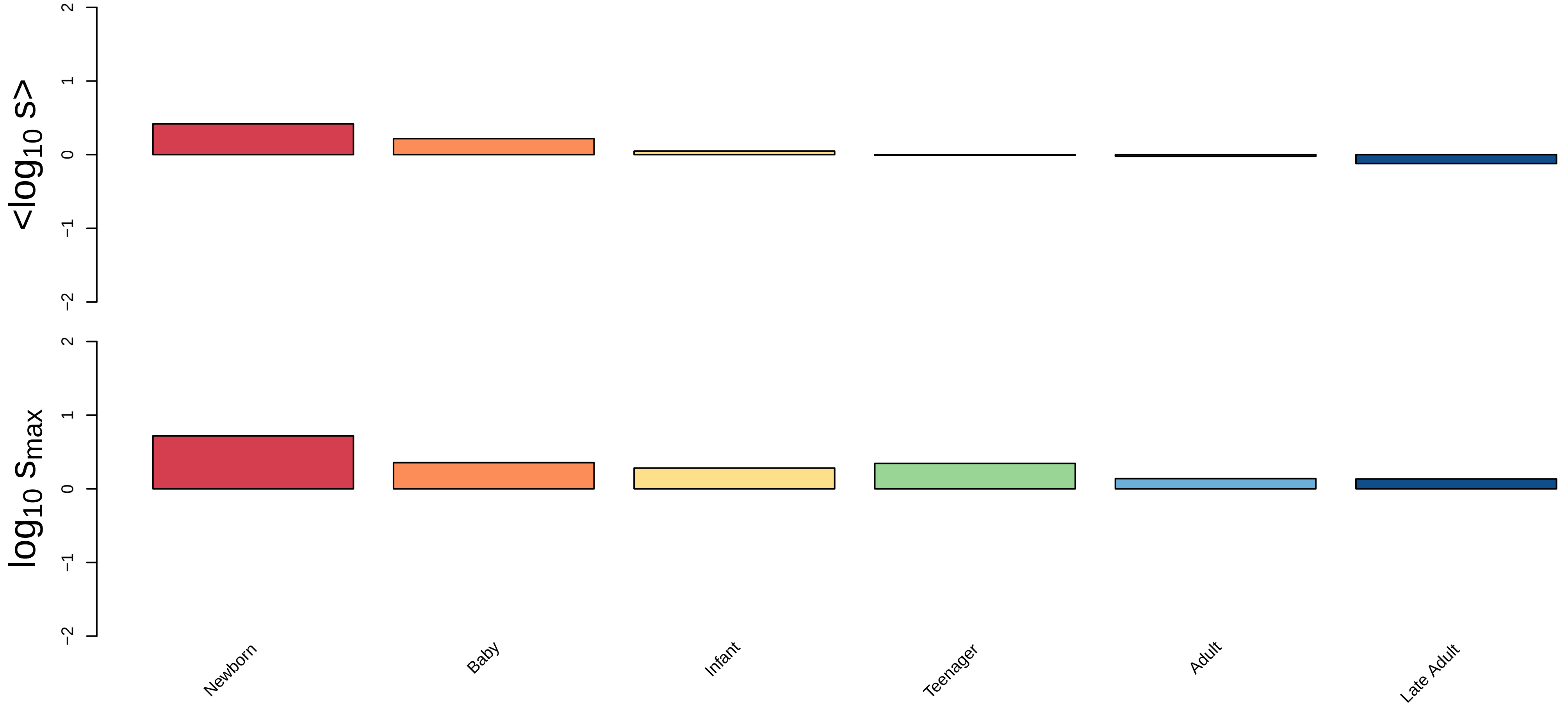

Estrogen_signaling_pathway
genes with data

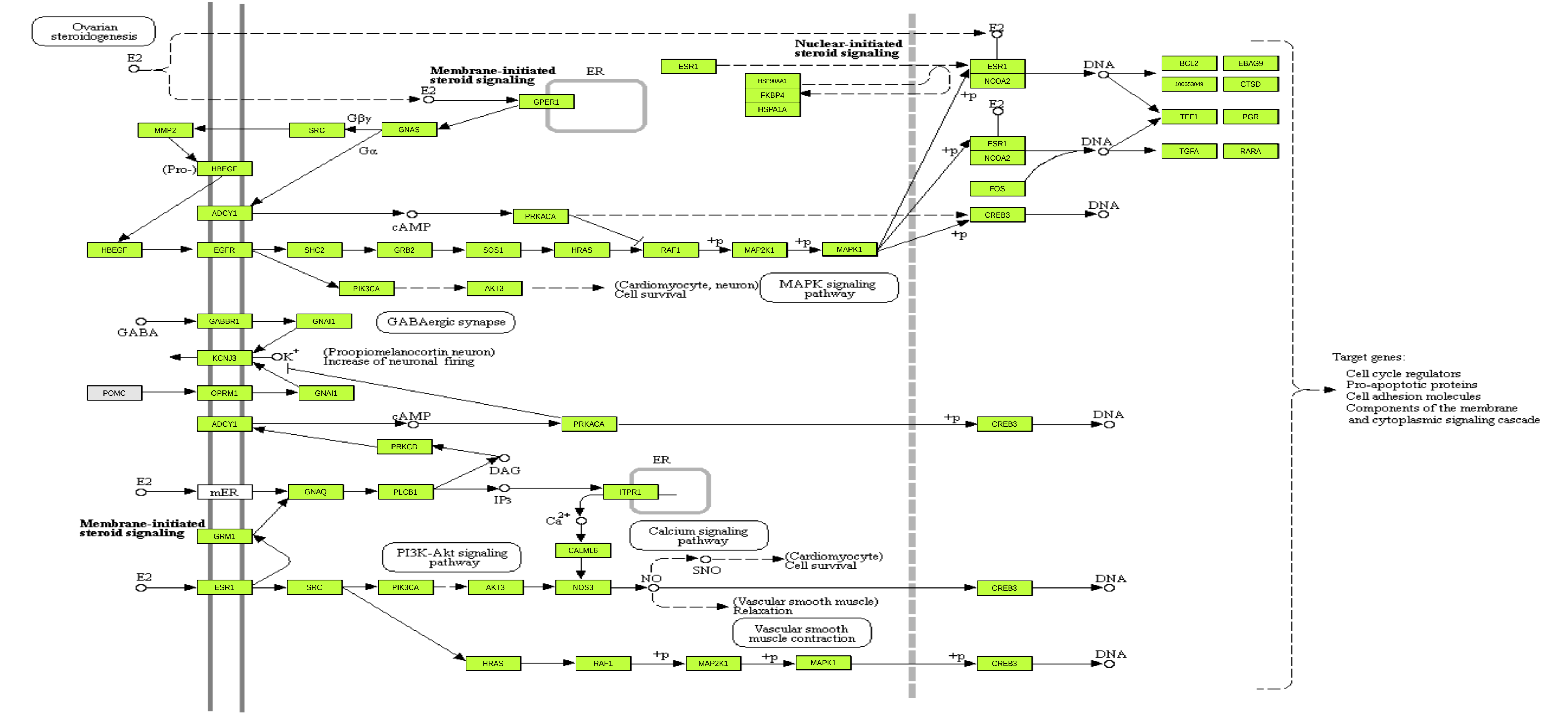

04915 2/5/19
(c) Kanehisa Laboratories

Estrogen_signaling_pathway
sink nodes

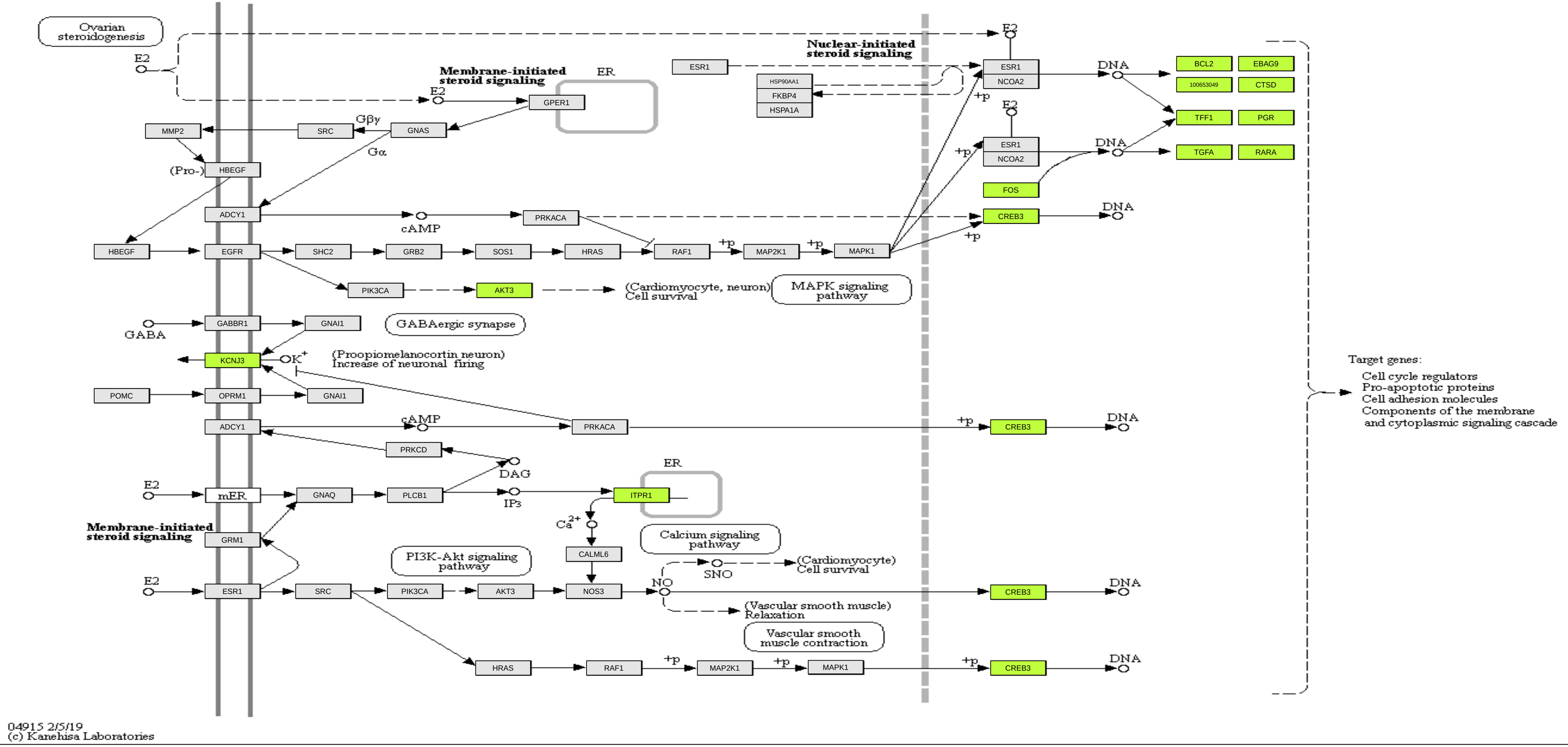

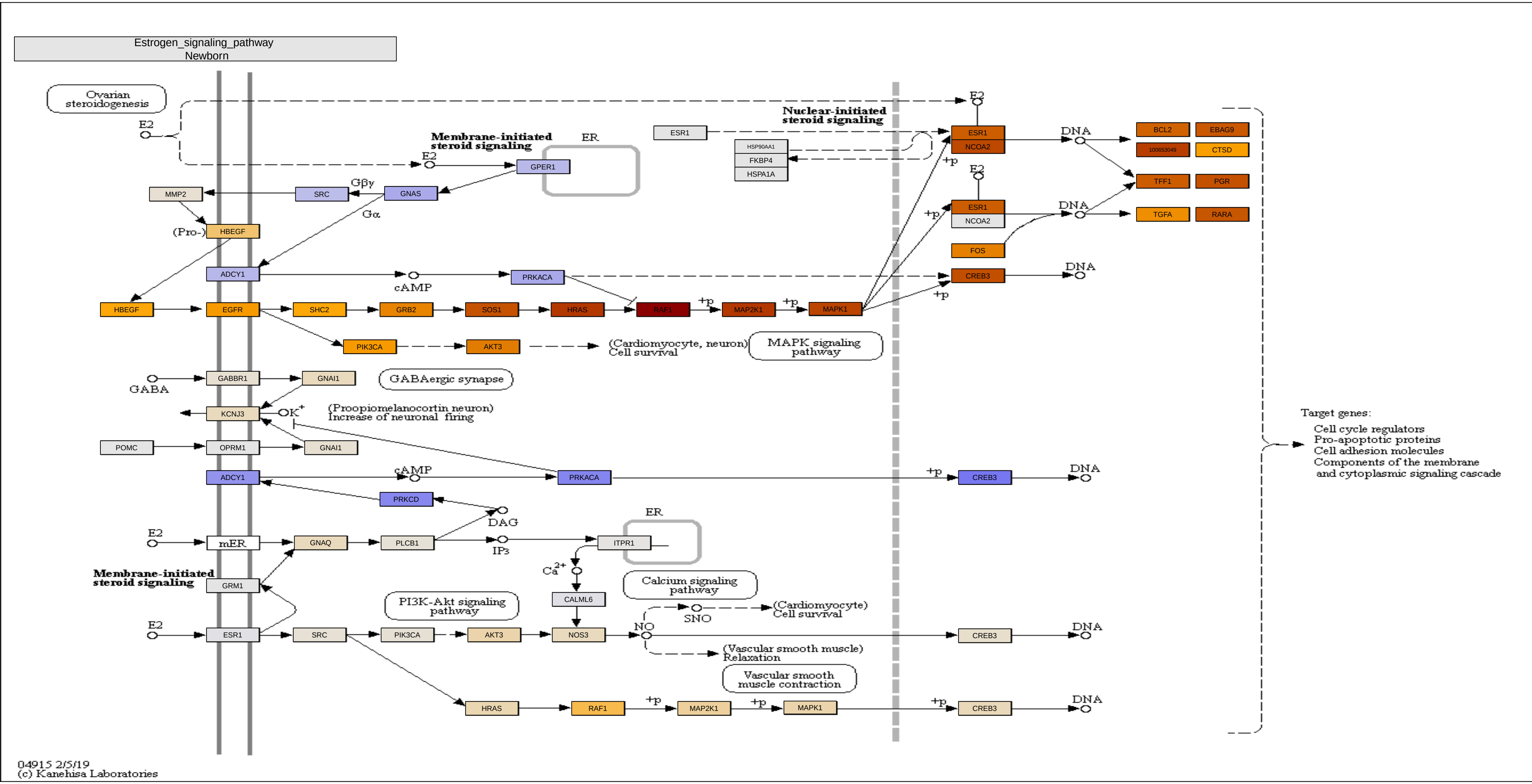

Estrogen_signaling_pathway
Newborn
Ovarian steroidogenesis
E2
Membrane-initiated steroid signaling
ER
GPER1
GNAS
SRC
MMP2
Gβγ
Gα
(Pro-) HBEGF
ADCY1
cAMP
PRKACA
HBEGF
EGFR
SHC2
GRB2
SOS1
HRAS
RAF1
MAP2K1
MAPK1
+p
PIK3CA
AKT3
(Cardiomyocyte, neuron) Cell survival
MAPK signaling pathway
GABA
GABBR1
GNAI1
GABAergic synapse
KCNJ3
K+
(Proopiomelanocortin neuron) Increase of neuronal firing
POMC
OPRM1
PRKCD
DAG
mER
GNAQ
PLCB1
IP3
ITPR1
Ca2+
CALML6
Calcium signaling pathway
GRM1
ESR1
NOS3
NO
SNO
(Cardiomyocyte) Cell survival
(Vascular smooth muscle) Relaxation
Vascular smooth muscle contraction
PI3K-Akt signaling pathway
Nuclear-initiated steroid signaling
HSP90AA1
FKBP4
HSPA1A
NCOA2
FOS
CREB3
DNA
BCL2
EBAG9
100653049
CTSD
TFF1
PGR
TGFA
RARA
Target genes:
Cell cycle regulators
Pro-apoptotic proteins
Cell adhesion molecules
Components of the membrane and cytoplasmic signaling cascade
04915 2/5/19
(c) Kanehisa Laboratories

Estrogen_signaling_pathway
Baby

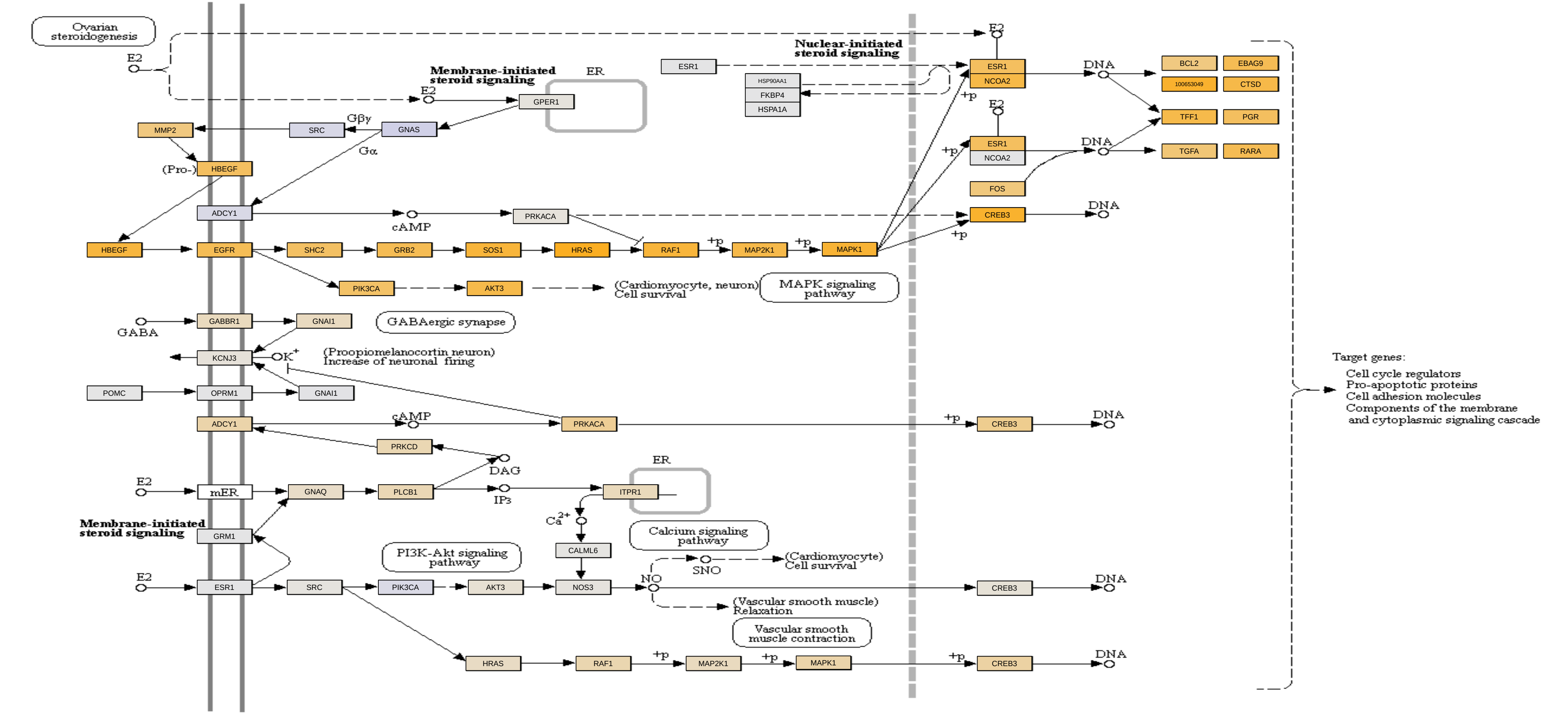

04915 2/5/19
(c) Kanehisa Laboratories

Estrogen_signaling_pathway
Infant

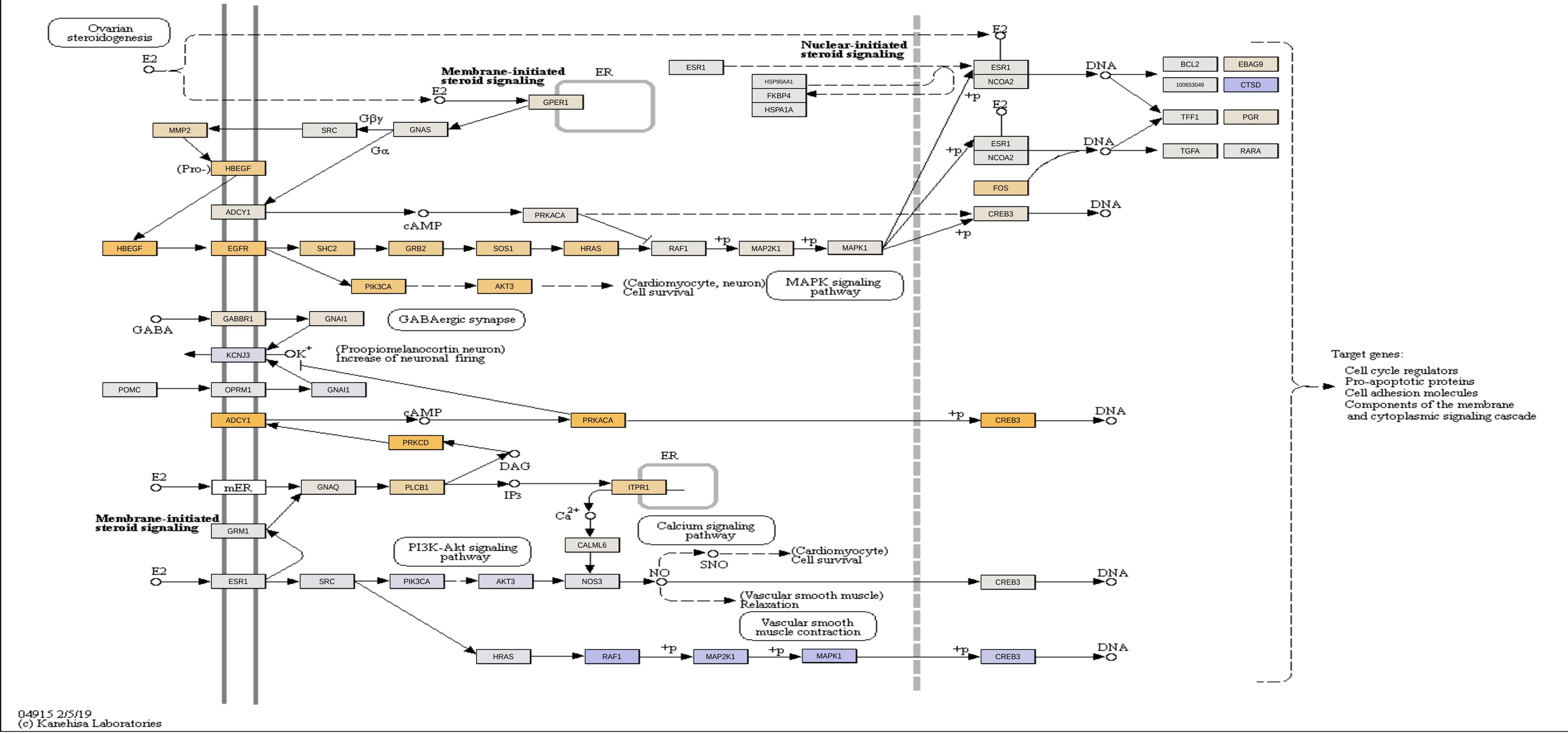

Estrogen_signaling_pathway
Teenager

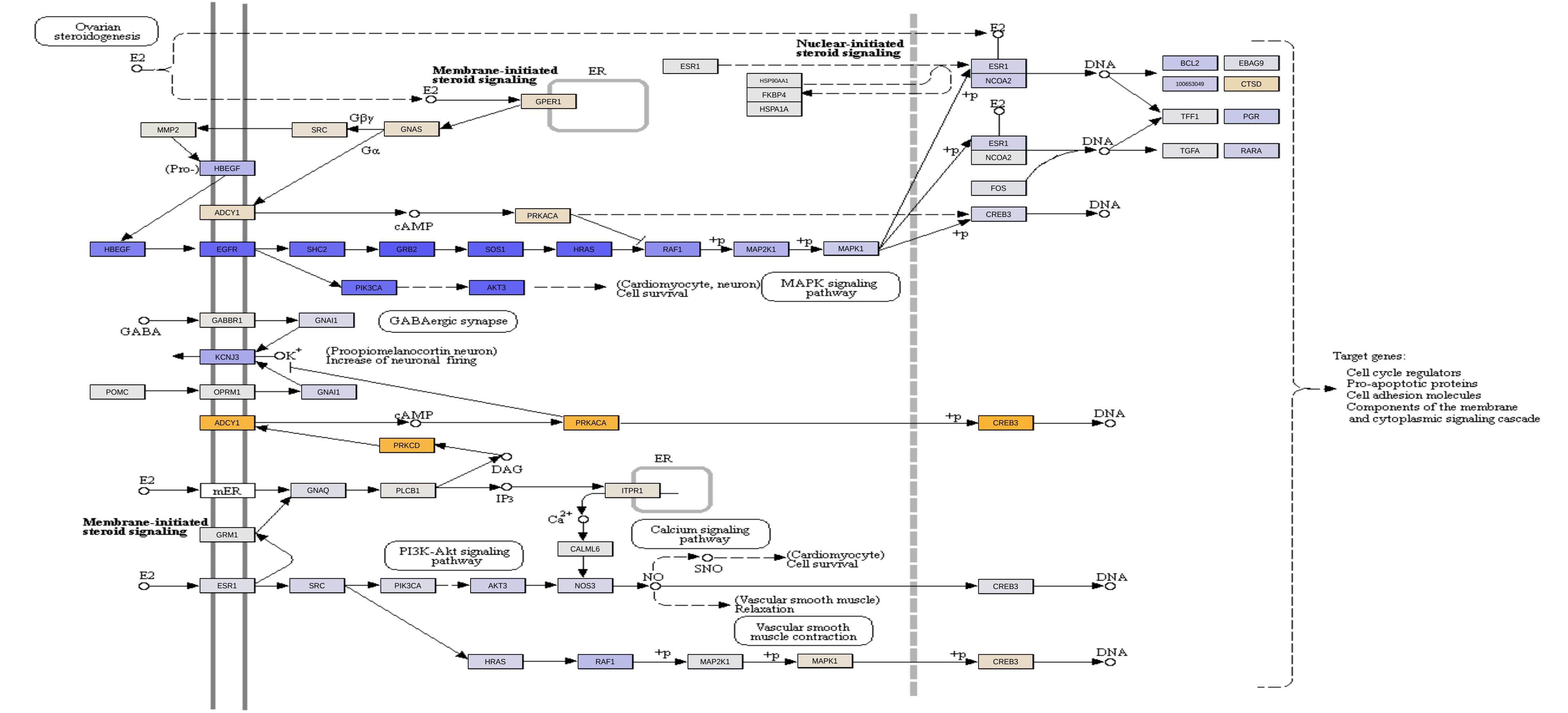

04915 2/5/19
(c) Kanehisa Laboratories

Estrogen_signaling_pathway
Adult

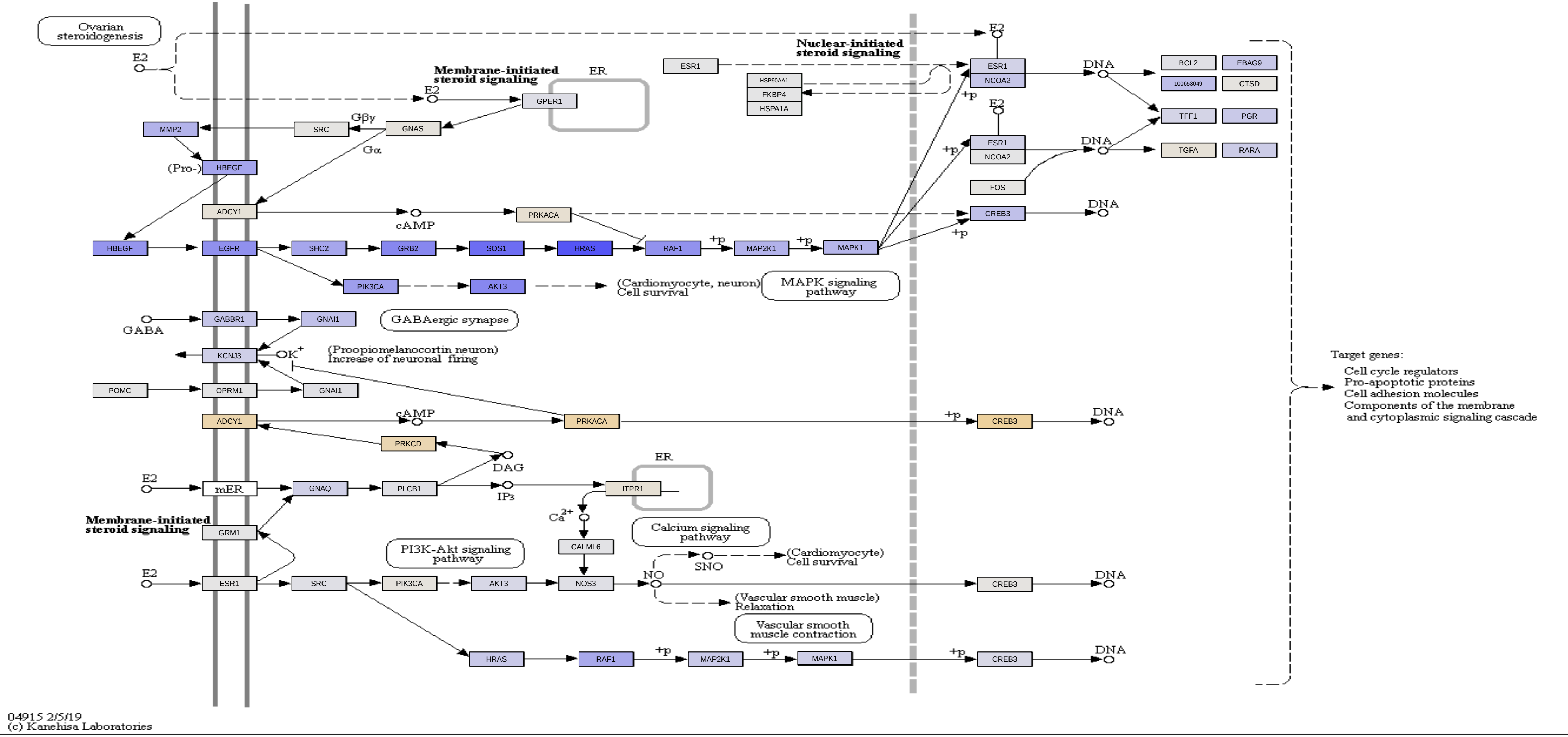

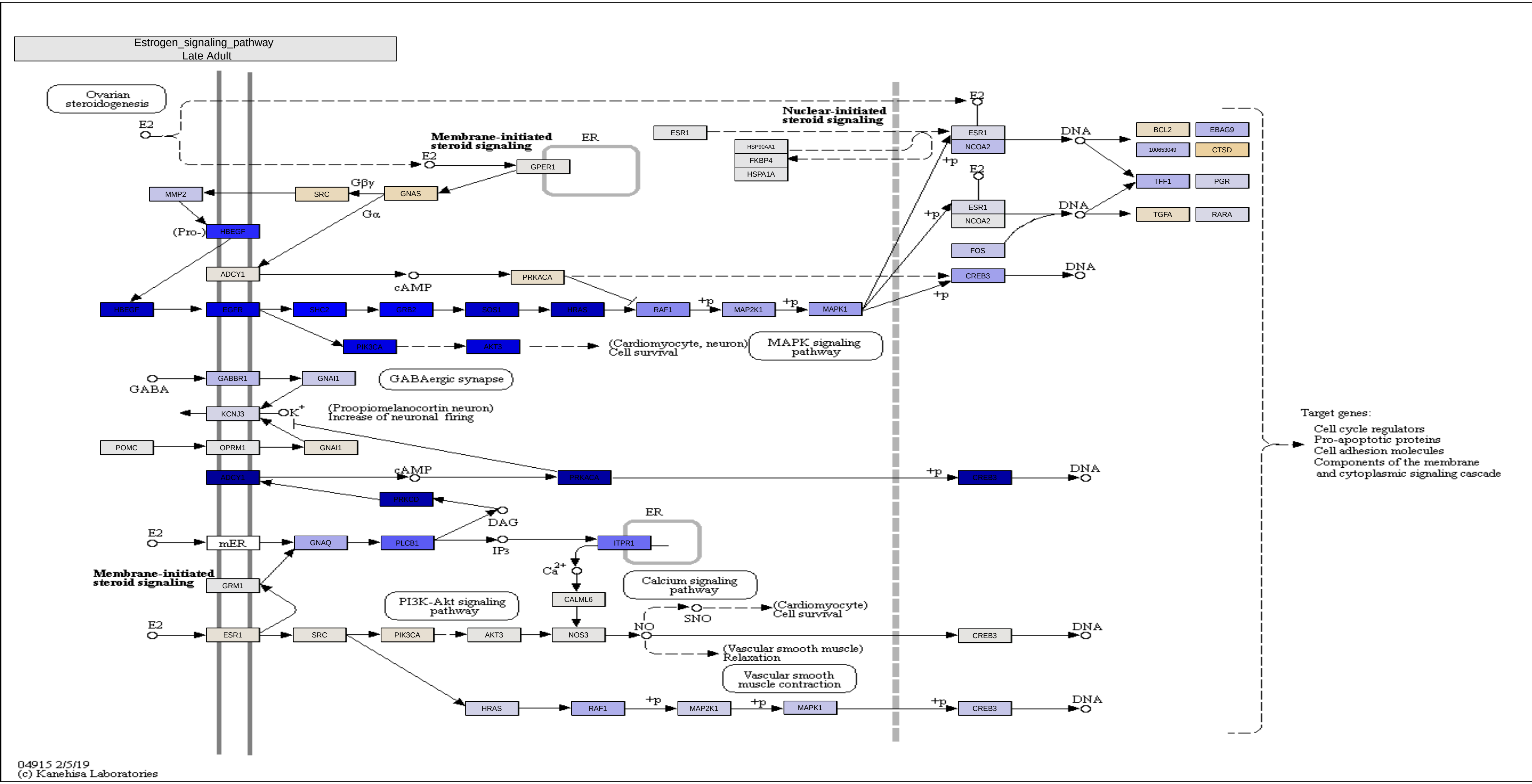
Estrogen_signaling_pathway
Late Adult
Ovarian steroidogenesis
E2
Membrane-initiated steroid signaling
ER
GPER1
GNAS
SRC
MMP2
Gβγ
Gα
(Pro-) HBEGF
ADCY1
cAMP
PRKACA
HBEGF
EGFR
SHC2
GRB2
SOS1
HRAS
RAF1
MAP2K1
MAPK1
+p
PIK3CA
AKT3
(Cardiomyocyte, neuron) Cell survival
MAPK signaling pathway
GABA
GABBR1
GNAI1
GABAergic synapse
KCNJ3
K+
(Proopiomelanocortin neuron) Increase of neuronal firing
POMC
OPRM1
PRKCD
DAG
mER
GNAQ
PLCB1
IP3
ITPR1
Ca2+
CALML6
Calcium signaling pathway
GRM1
PI3K-Akt signaling pathway
ESR1
NOS3
NO
SNO
(Cardiomyocyte) Cell survival
(Vascular smooth muscle) Relaxation
Vascular smooth muscle contraction
Nuclear-initiated steroid signaling
HSP90AA1
FKBP4
HSPA1A
NCOA2
FOS
CREB3
CREB1
DNA
BCL2
EBAG9
100653049
CTSD
TFF1
PGR
TGFA
RARA
Target genes:
Cell cycle regulators
Pro-apoptotic proteins
Cell adhesion molecules
Components of the membrane and cytoplasmic signaling cascade
04915 2/5/19
(c) Kanehisa Laboratories
