# Supplementary material for: The Transcriptome and Methylome of the Developing and Aging Brain and Their Relations to Gliomas and Psychological Disorders
Source: Cells. 2022 Jan 21;11(3):362. doi: 10.3390/cells11030362 (PMC8834030; doi:10.3390/cells11030362)
Supplement: Supplementary file 1 [file cells-11-00362-s001.zip › Suppl-Material-S3-Pathways-PSF_Expression/Fc_epsilon_RI_signaling_pathway.pdf]

Fc epsilon RI signaling pathway

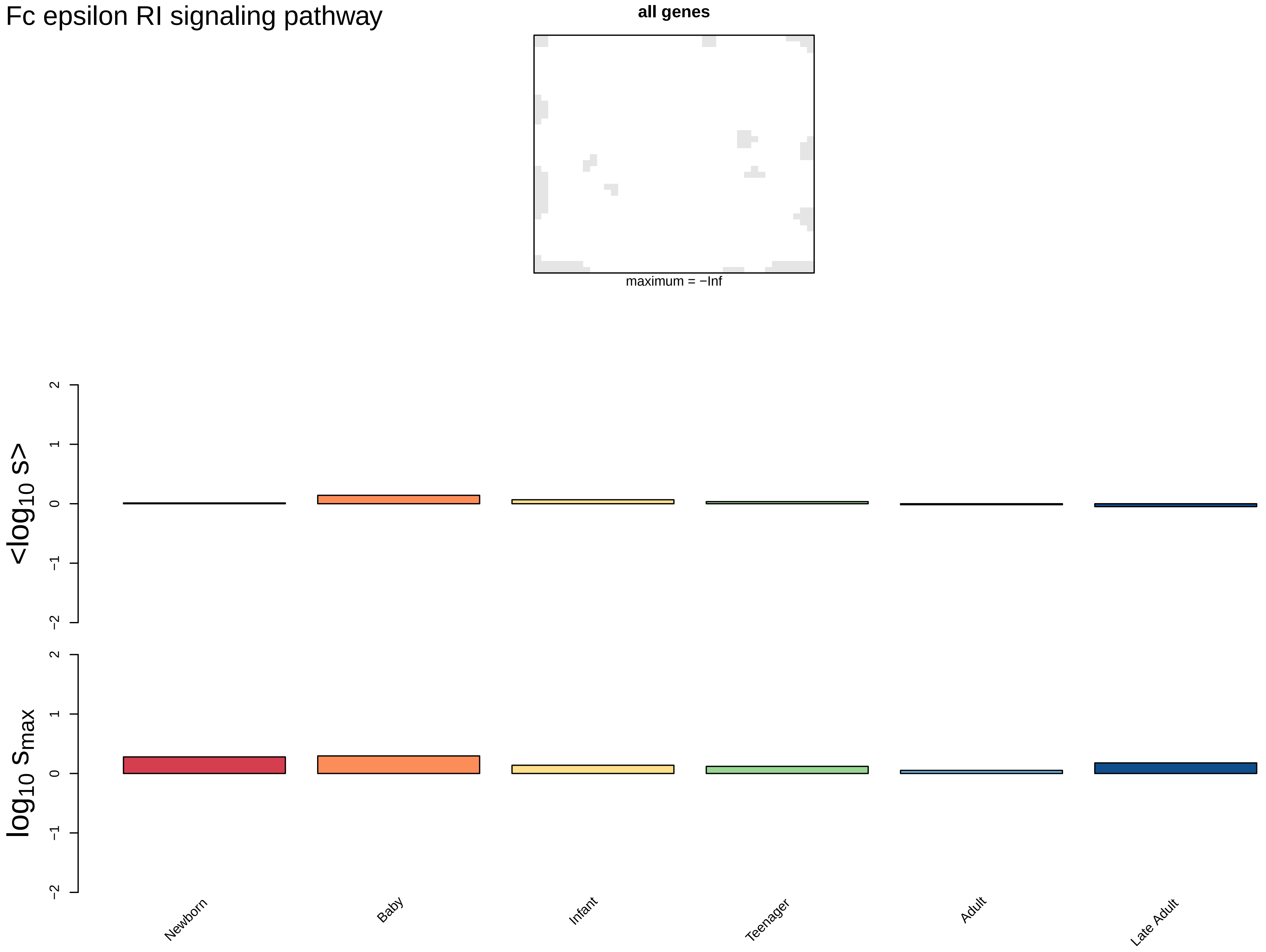

Fc_epsilon_RI_signaling_pathway
genes with data

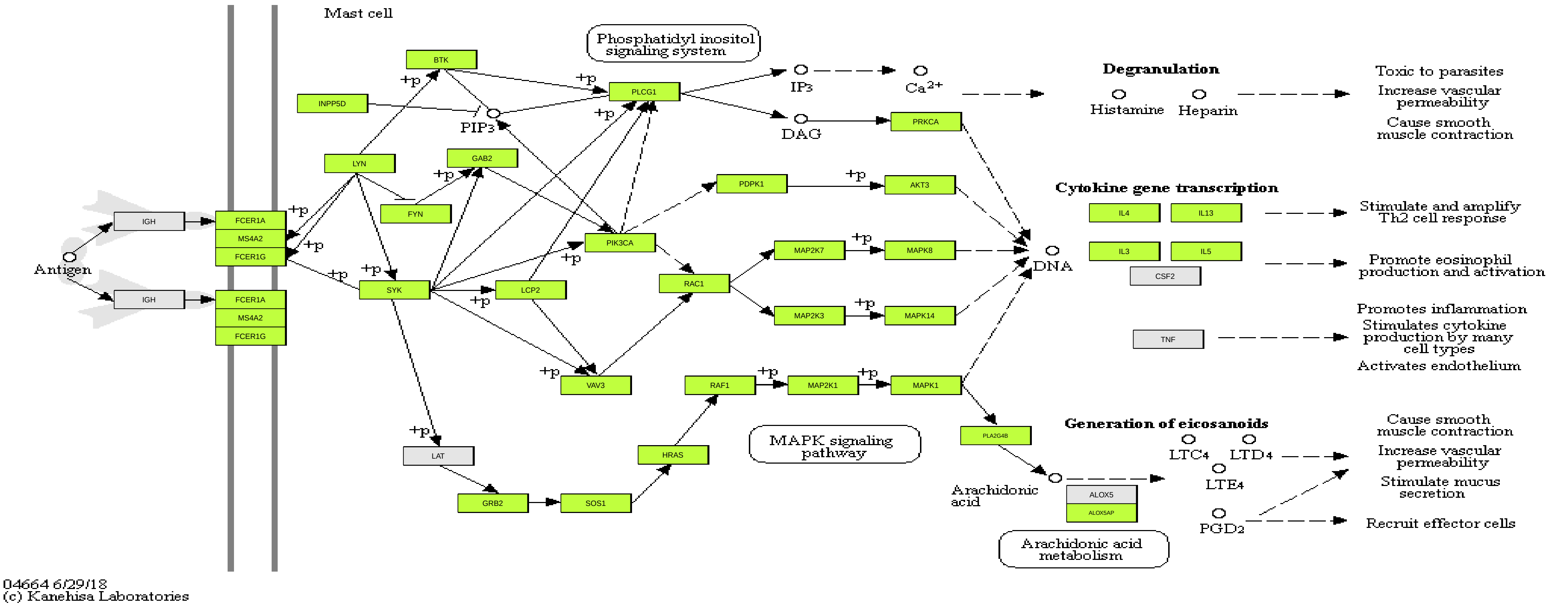

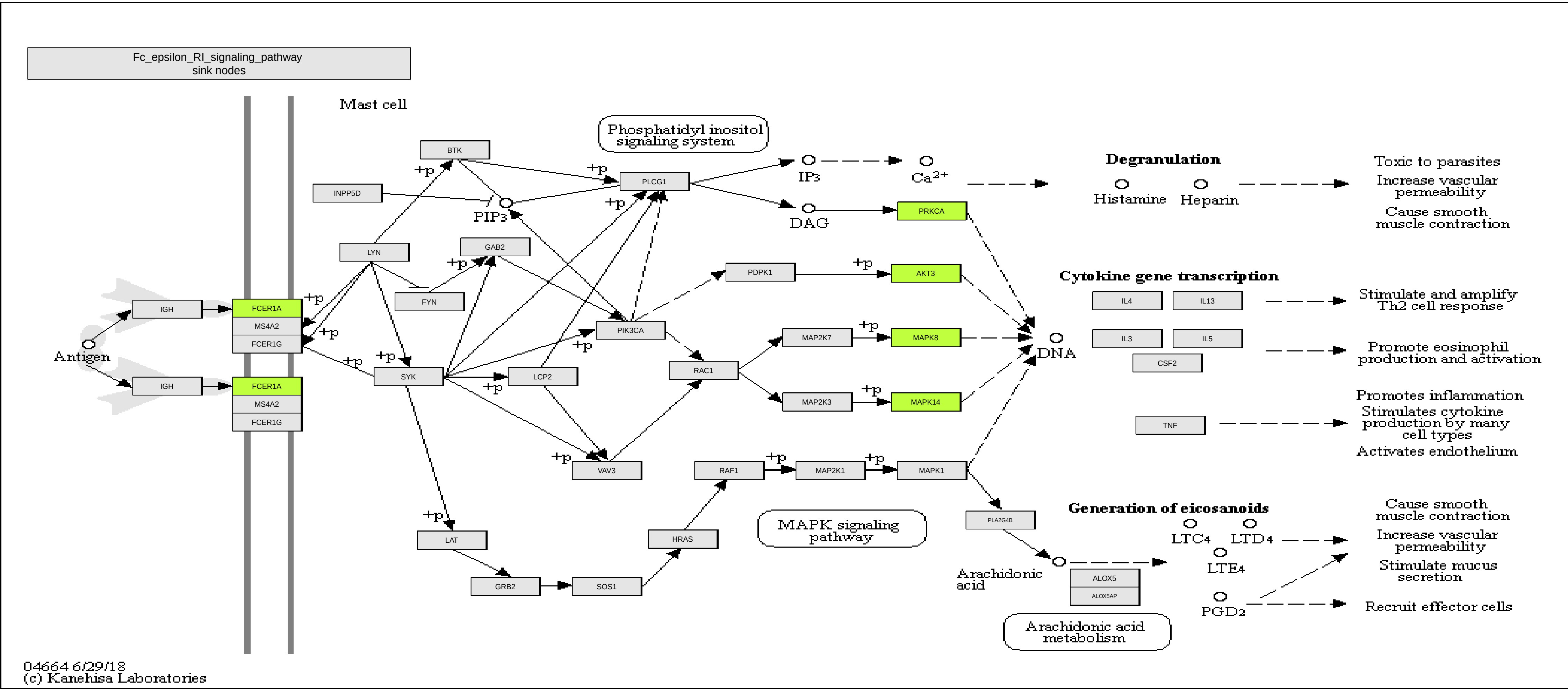

Fc_epsilon_RI_signaling_pathway
sink nodes
Mast cell
Phosphatidyl inositol signaling system
BTK
INPP5D
PLCG1
PIP3
IP3
Ca2+
DAG
PRKCA
Degranulation
Histamine
Heparin
Toxic to parasites
Increase vascular permeability
Cause smooth muscle contraction
LYN
GAB2
FYN
PIK3CA
PDPK1
AKT3
Cytokine gene transcription
IL4
IL13
IL3
IL5
CSF2
TNF
Stimulate and amplify Th2 cell response
Promote eosinophil production and activation
Promotes inflammation
Stimulates cytokine production by many cell types
Activates endothelium
Antigen
IGH
FCER1A
MS4A2
FCER1G
SYK
LCP2
RAC1
MAP2K7
MAPK8
MAP2K3
MAPK14
DNA
VAV3
RAF1
MAP2K1
MAPK1
PLA2G4B
LAT
HRAS
GRB2
SOS1
MAPK signaling pathway
Generation of eicosanoids
LTC4
LTD4
LTE4
PGD2
Arachidonic acid
ALOX5
ALOX5AP
Arachidonic acid metabolism
Cause smooth muscle contraction
Increase vascular permeability
Stimulate mucus secretion
Recruit effector cells
+p
04664 6/29/18
(c) Kanehisa Laboratories

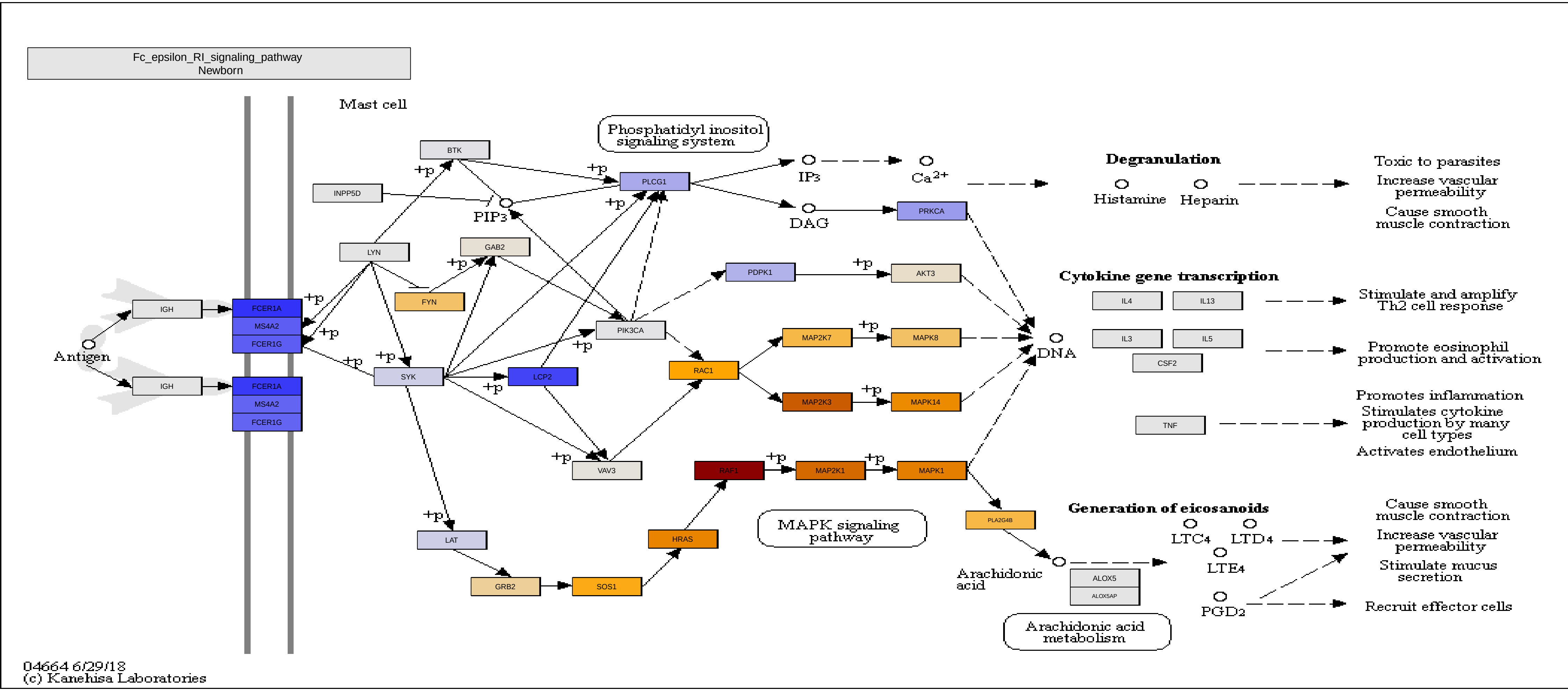
Fc_epsilon_RI_signaling_pathway
Newborn
Mast cell
Phosphatidyl inositol signaling system
BTK
INPP5D
PLCG1
PIP3
IP3
Ca2+
DAG
PRKCA
Degranulation
Histamine
Heparin
Toxic to parasites
Increase vascular permeability
Cause smooth muscle contraction
LYN
GAB2
FYN
PDPK1
AKT3
Cytokine gene transcription
IL4
IL13
IL3
IL5
CSF2
TNF
Stimulate and amplify Th2 cell response
Promote eosinophil production and activation
Promotes inflammation
Stimulates cytokine production by many cell types
Activates endothelium
IGH
FCER1A
MS4A2
FCER1G
Antigen
PIK3CA
SYK
LCP2
RAC1
MAP2K7
MAPK8
MAP2K3
MAPK14
DNA
VAV3
RAF1
MAP2K1
MAPK1
PLA2G4B
LAT
HRAS
GRB2
SOS1
MAPK signaling pathway
Generation of eicosanoids
LTC4
LTD4
LTE4
PGD2
Arachidonic acid
ALOX5
ALOX5AP
Arachidonic acid metabolism
Cause smooth muscle contraction
Increase vascular permeability
Stimulate mucus secretion
Recruit effector cells
+p
04664 6/29/18
(c) Kanehisa Laboratories

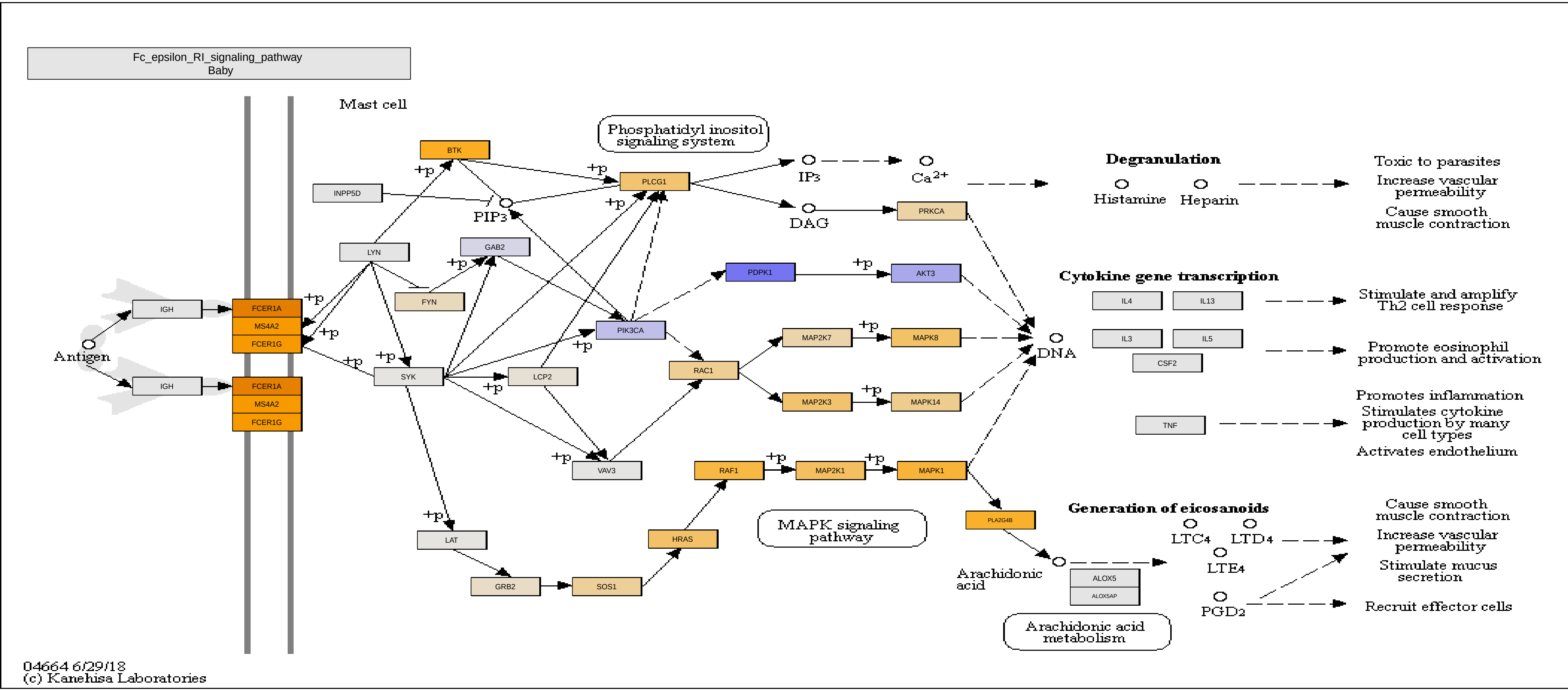

Fc_epsilon_RI_signaling_pathway
Baby
Mast cell
Antigen
IGH
FCER1A
MS4A2
FCER1G
LYN
FYN
SYK
BTK
INPP5D
GAB2
PIP3
PIK3CA
PLCG1
LCP2
VAV3
LAT
GRB2
SOS1
HRAS
RAF1
MAP2K1
MAPK1
RAC1
MAP2K7
MAPK8
MAP2K3
MAPK14
PDPK1
AKT3
PRKCA
PLA2G4B
Phosphatidyl inositol signaling system
MAPK signaling pathway
IP3
Ca2+
DAG
DNA
Degranulation
Histamine
Heparin
Toxic to parasites
Increase vascular permeability
Cause smooth muscle contraction
Cytokine gene transcription
IL4
IL13
IL3
IL5
CSF2
TNF
Stimulate and amplify Th2 cell response
Promote eosinophil production and activation
Promotes inflammation
Stimulates cytokine production by many cell types
Activates endothelium
Generation of eicosanoids
LTC4
LTD4
LTE4
PGD2
ALOX5
ALOX5AP
Arachidonic acid
Arachidonic acid metabolism
Cause smooth muscle contraction
Increase vascular permeability
Stimulate mucus secretion
Recruit effector cells
+p
04664 6/29/18
(c) Kanehisa Laboratories

Fc_epsilon_RI_signaling_pathway
Infant

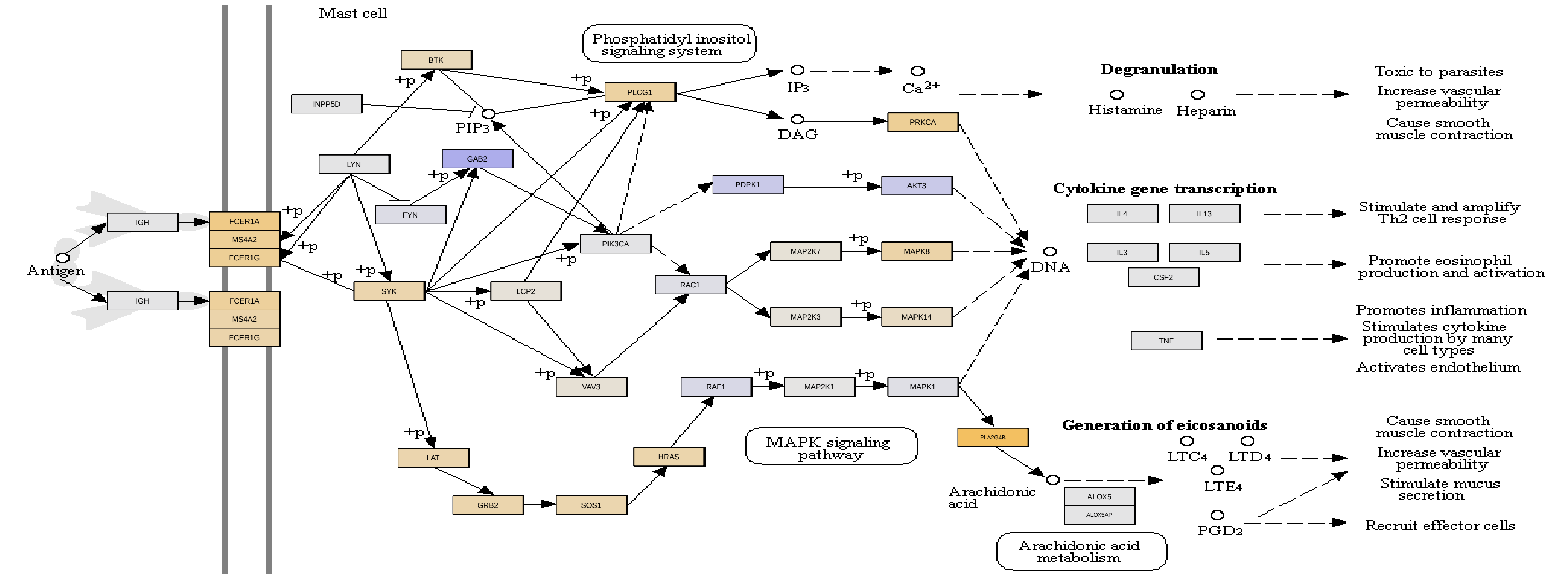

04664 6/29/18
(c) Kanehisa Laboratories

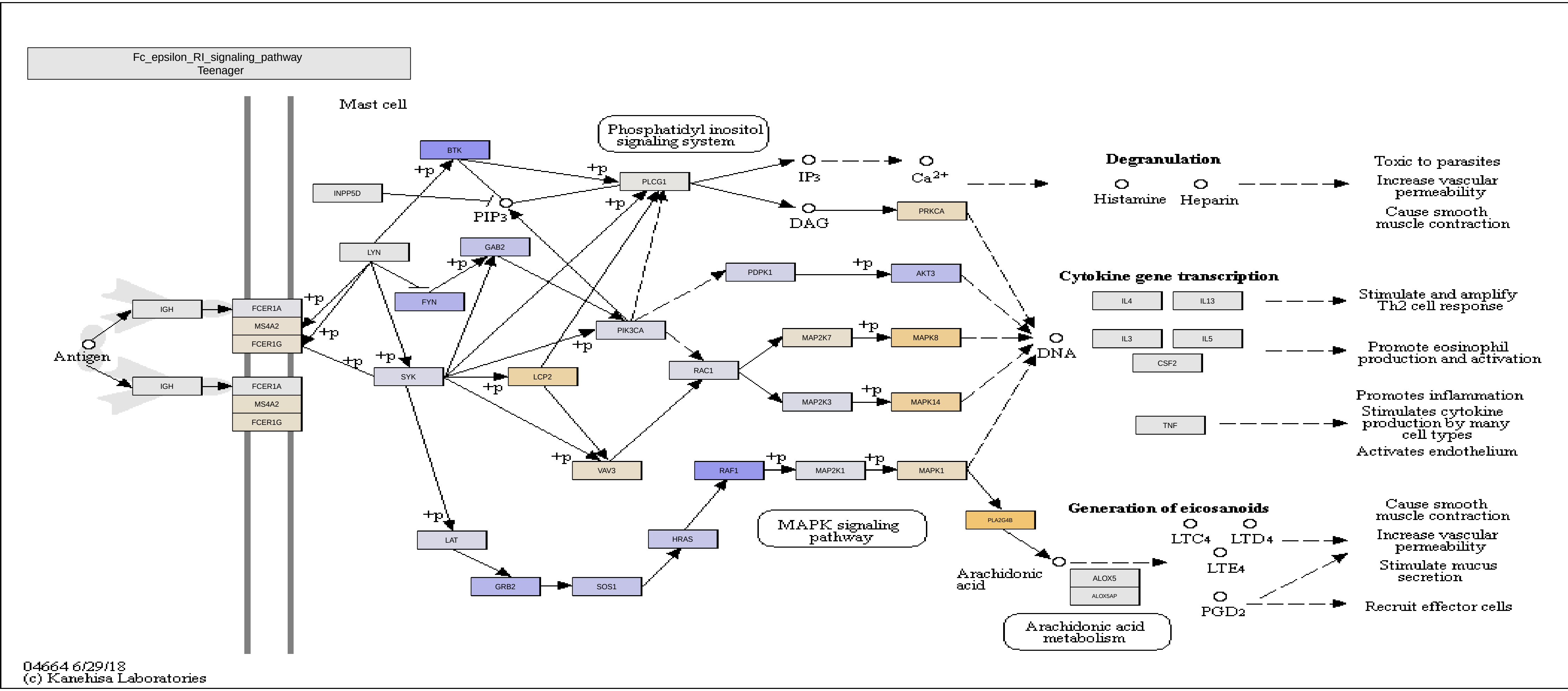

Fc_epsilon_RI_signaling_pathway
Teenager
Mast cell
Antigen
IGH
FCER1A
MS4A2
FCER1G
LYN
BTK
INPP5D
FYN
GAB2
SYK
LCP2
VAV3
LAT
GRB2
SOS1
HRAS
RAF1
MAP2K1
MAPK1
PLA2G4B
PIP3
PLCG1
PIK3CA
RAC1
PDPK1
AKT3
MAP2K7
MAPK8
MAP2K3
MAPK14
Phosphatidyl inositol signaling system
IP3
Ca2+
DAG
PRKCA
DNA
MAPK signaling pathway
Arachidonic acid
ALOX5
ALOX5AP
Arachidonic acid metabolism
Degranulation
Histamine
Heparin
Cytokine gene transcription
IL4
IL13
IL3
IL5
CSF2
TNF
Generation of eicosanoids
LTC4
LTD4
LTE4
PGD2
Toxic to parasites
Increase vascular permeability
Cause smooth muscle contraction
Stimulate and amplify Th2 cell response
Promote eosinophil production and activation
Promotes inflammation
Stimulates cytokine production by many cell types
Activates endothelium
Cause smooth muscle contraction
Increase vascular permeability
Stimulate mucus secretion
Recruit effector cells
+p
04664 6/29/18
(c) Kanehisa Laboratories

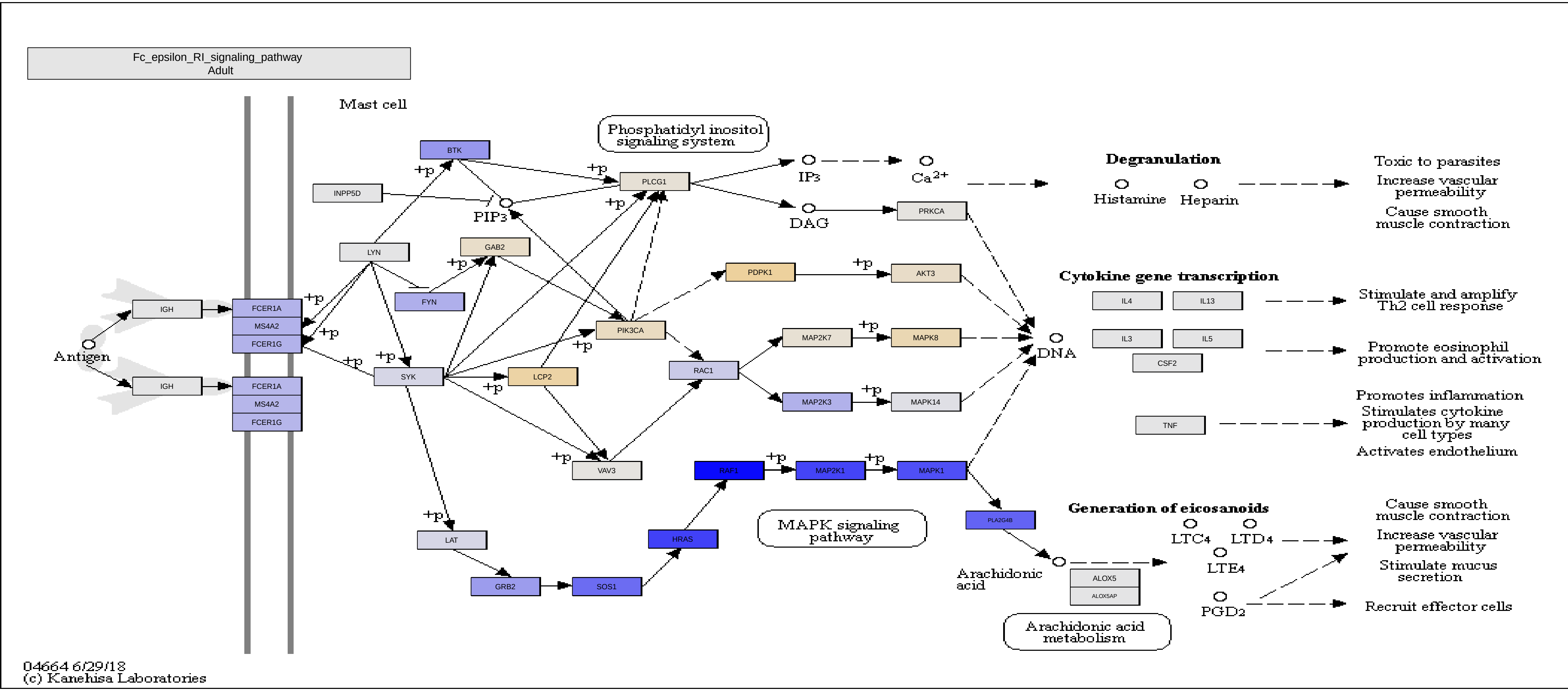
Fc_epsilon_RI_signaling_pathway
Adult
Mast cell
Phosphatidyl inositol signaling system
BTK
INPP5D
PLCG1
IP3
Ca2+
DAG
PRKCA
PIP3
LYN
GAB2
FYN
PIK3CA
PDPK1
AKT3
Antigen
IGH
FCER1A
MS4A2
FCER1G
SYK
LCP2
RAC1
MAP2K7
MAPK8
MAP2K3
MAPK14
VAV3
RAF1
MAP2K1
MAPK1
LAT
GRB2
SOS1
HRAS
MAPK signaling pathway
PLA2G4B
Arachidonic acid
ALOX5
ALOX5AP
Arachidonic acid metabolism
DNA
Degranulation
Histamine
Heparin
Toxic to parasites
Increase vascular permeability
Cause smooth muscle contraction
Cytokine gene transcription
IL4
IL13
IL3
IL5
CSF2
TNF
Stimulate and amplify Th2 cell response
Promote eosinophil production and activation
Promotes inflammation
Stimulates cytokine production by many cell types
Activates endothelium
Generation of eicosanoids
LTC4
LTD4
LTE4
PGD2
Cause smooth muscle contraction
Increase vascular permeability
Stimulate mucus secretion
Recruit effector cells
+p
04664 6/29/18
(c) Kanehisa Laboratories

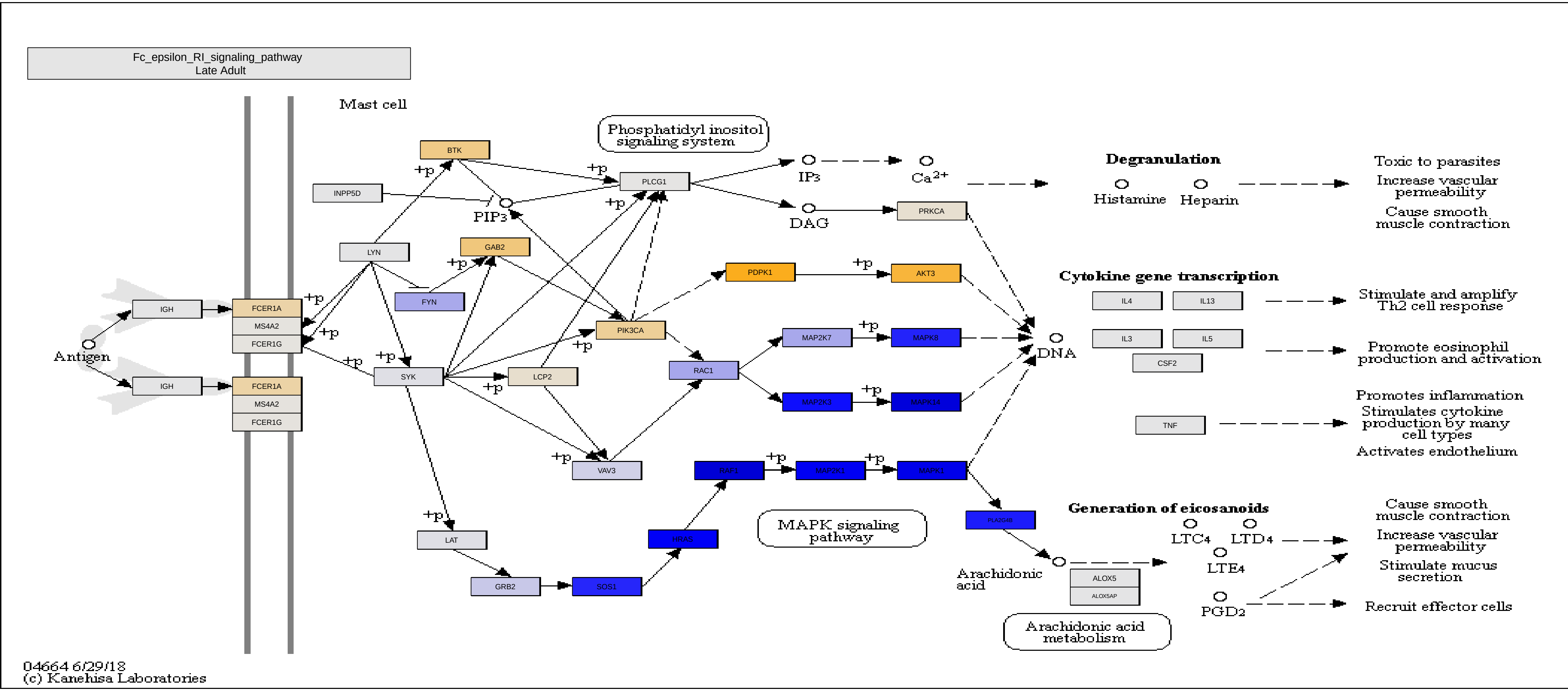
Fc_epsilon_RI_signaling_pathway
Late Adult
Mast cell
Antigen
IGH
IGH
FCER1A
MS4A2
FCER1G
FCER1A
MS4A2
FCER1G
LYN
FYN
SYK
BTK
INPP5D
GAB2
PIP3
PIK3CA
LCP2
VAV3
LAT
GRB2
SOS1
HRAS
RAF1
MAP2K1
MAPK1
RAC1
MAP2K7
MAPK8
MAP2K3
MAPK14
PLA2G4B
PLCG1
Phosphatidyl inositol signaling system
IP3
Ca2+
DAG
PRKCA
PDPK1
AKT3
DNA
+p
Degranulation
Histamine
Heparin
Toxic to parasites
Increase vascular permeability
Cause smooth muscle contraction
Cytokine gene transcription
IL4
IL13
IL3
IL5
CSF2
TNF
Stimulate and amplify Th2 cell response
Promote eosinophil production and activation
Promotes inflammation
Stimulates cytokine production by many cell types
Activates endothelium
MAPK signaling pathway
Generation of eicosanoids
LTC4
LTD4
LTE4
PGD2
Arachidonic acid
ALOX5
ALOX5AP
Arachidonic acid metabolism
Cause smooth muscle contraction
Increase vascular permeability
Stimulate mucus secretion
Recruit effector cells
04664 6/29/18
(c) Kanehisa Laboratories
