# Supplementary material for: The Transcriptome and Methylome of the Developing and Aging Brain and Their Relations to Gliomas and Psychological Disorders
Source: Cells. 2022 Jan 21;11(3):362. doi: 10.3390/cells11030362 (PMC8834030; doi:10.3390/cells11030362)
Supplement: Supplementary file 1 [file cells-11-00362-s001.zip › Suppl-Material-S3-Pathways-PSF_Expression/Glucagon_signaling_pathway.pdf]

Glucagon signaling pathway

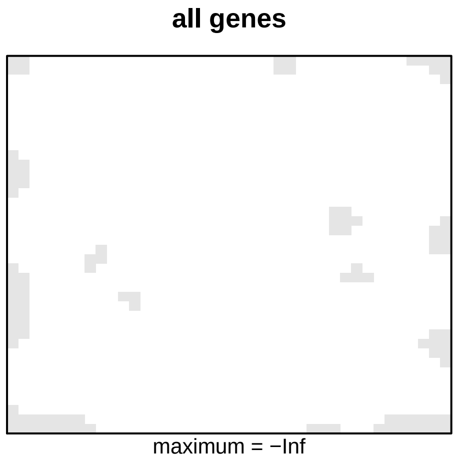

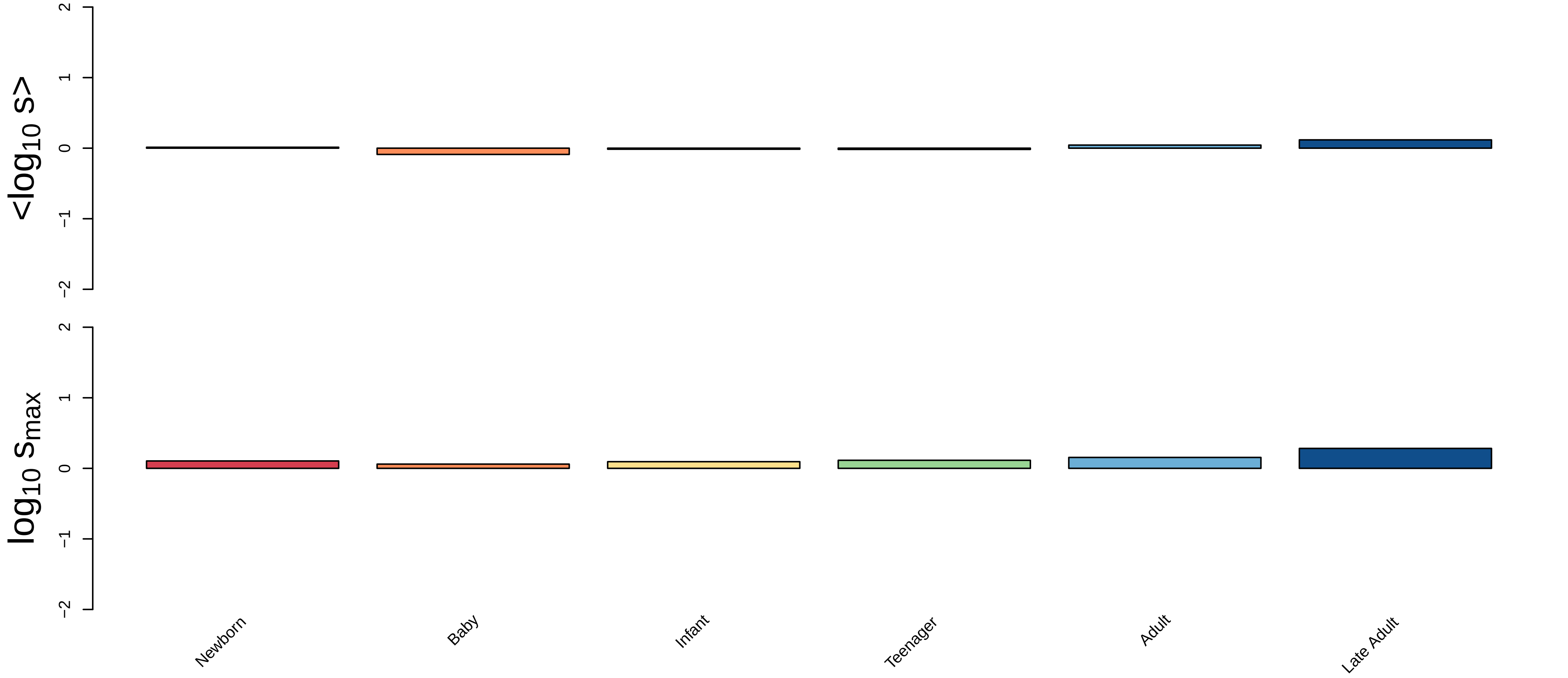

Glucagon_signaling_pathway
genes with data

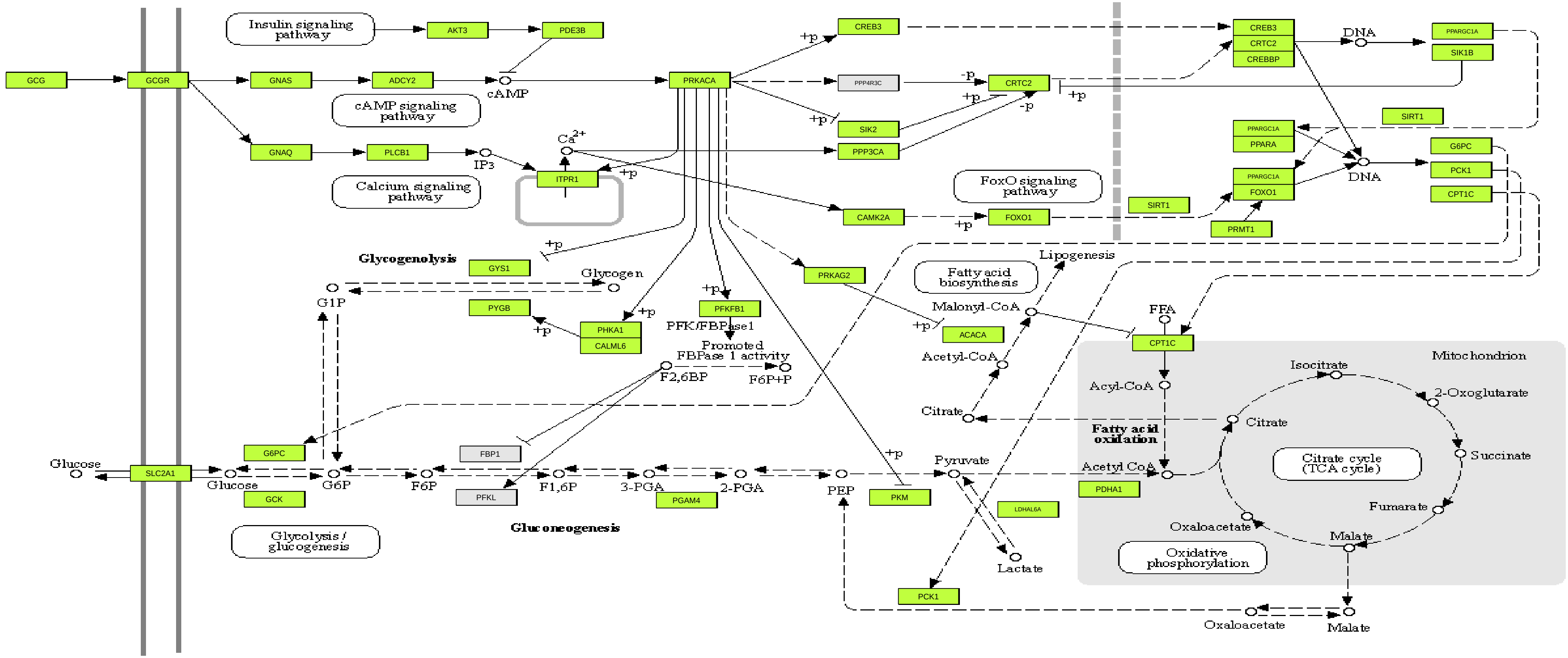

04922 7/5/19
(c) Kanehisa Laboratories

Glucagon_signaling_pathway
sink nodes

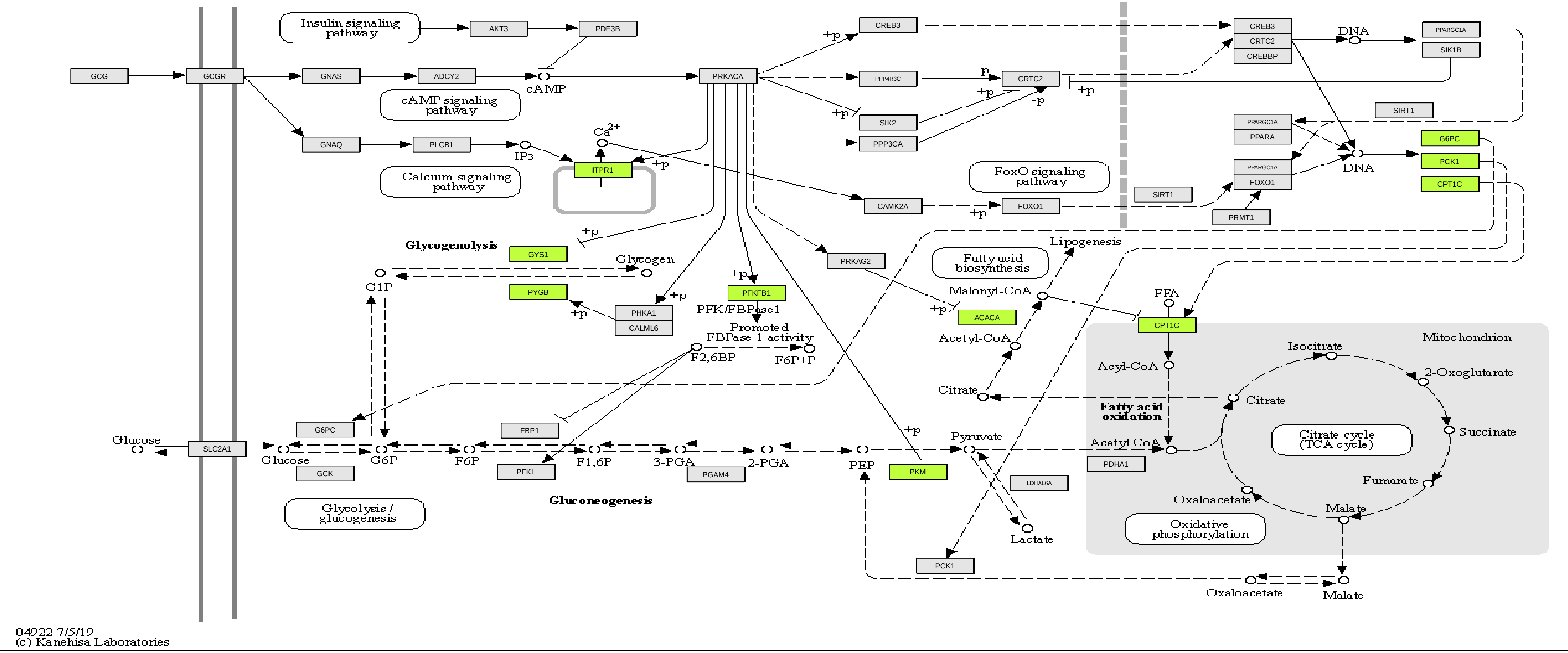

Glucagon_signaling_pathway
Newborn

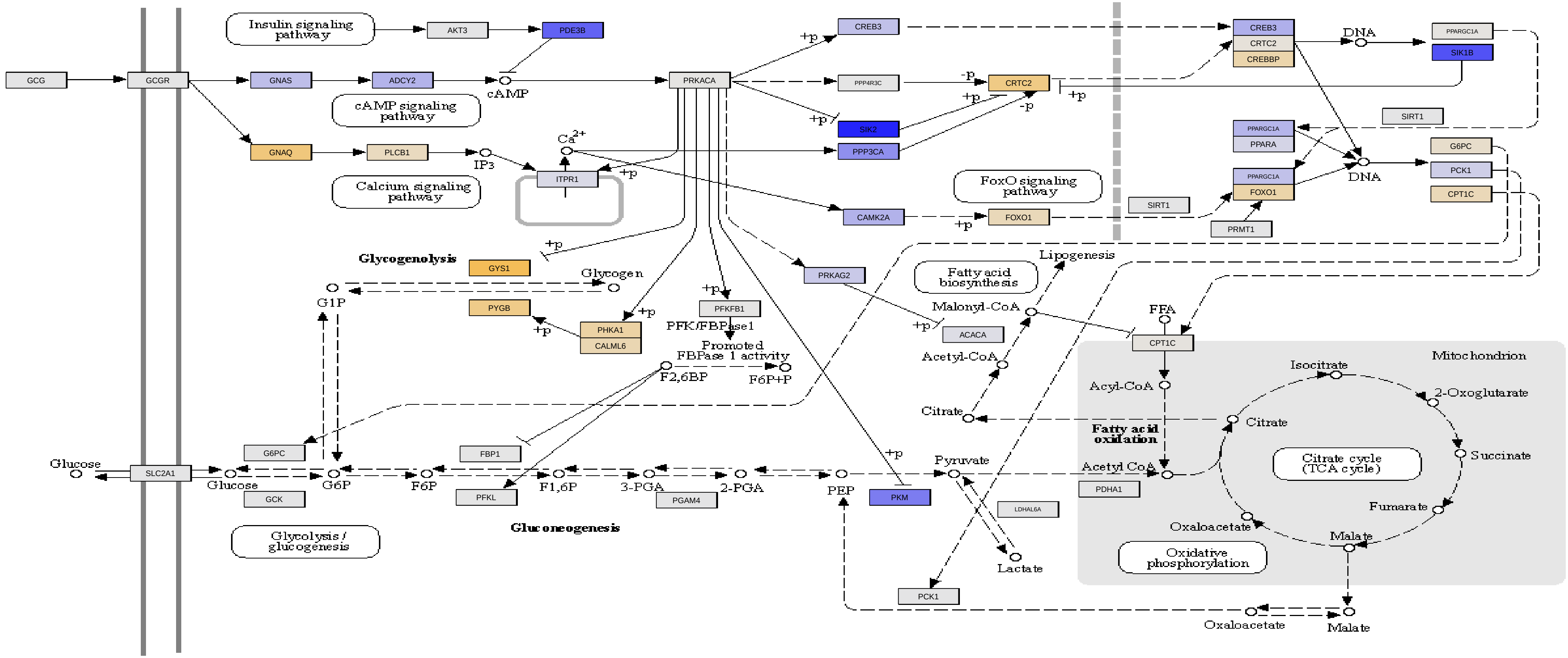

04922 7/5/19
(c) Kanehisa Laboratories

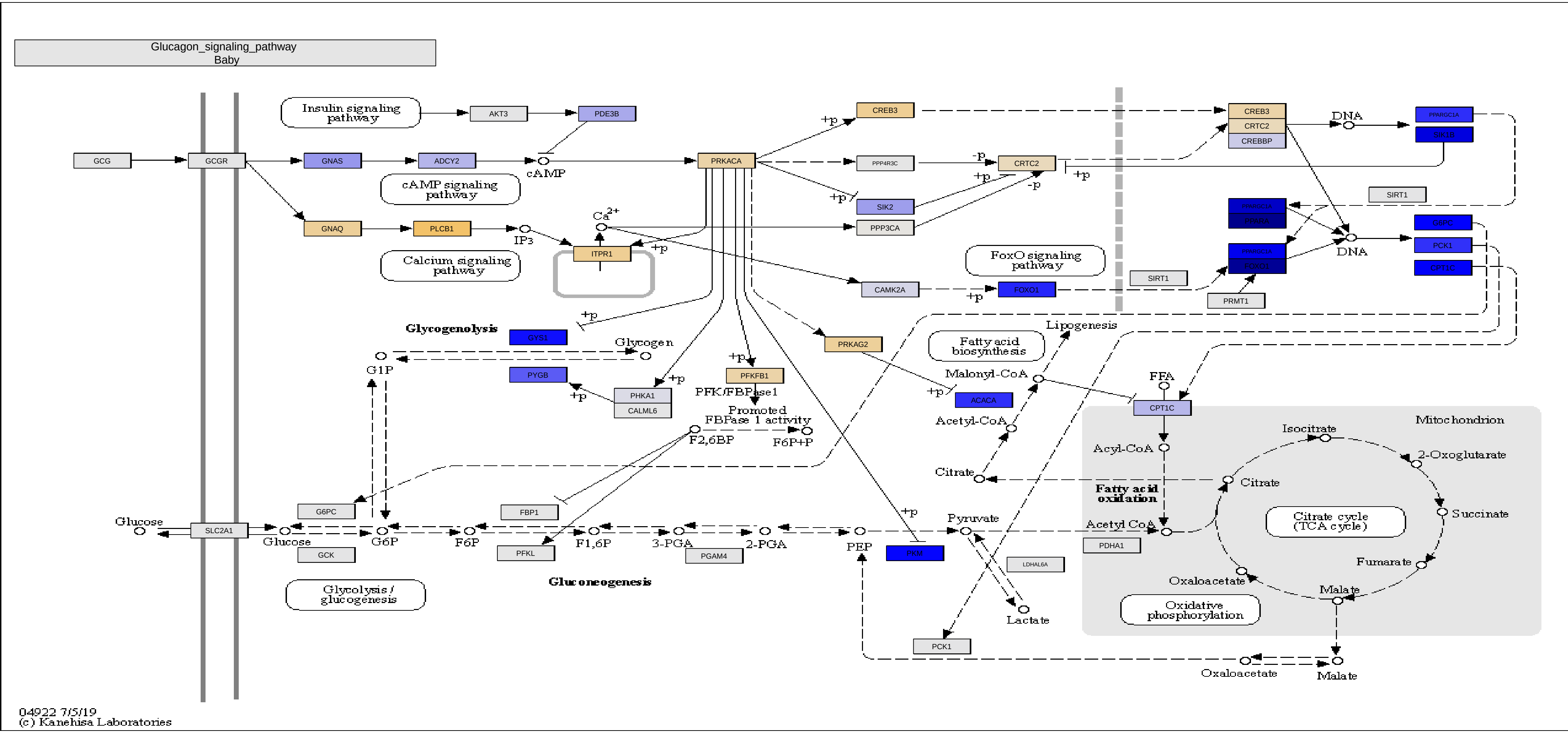

Glucagon_signaling_pathway
Baby
Insulin signaling pathway
AKT3
PDE3B
GCG
GCGR
GNAS
ADCY2
cAMP
cAMP signaling pathway
PRKACA
CREB3
PPP4R3C
CRTC2
SIK2
PPP3CA
GNAQ
PLCB1
IP3
Ca2+
ITPR1
Calcium signaling pathway
CAMK2A
FOXO1
FoxO signaling pathway
CREB3
CRTC2
CREBBP
DNA
PPARGC1A
SIK1B
SIRT1
PPARGC1A
PPARA
G6PC
PCK1
CPT1C
PRMT1
Glycogenolysis
GYS1
Glycogen
G1P
PYGB
PHKA1
CALML6
PFKFB1
PFK/FBPase1
Promoted FBPase 1 activity
F2,6BP
F6P+P
PRKAG2
Fatty acid biosynthesis
Lipogenesis
Malonyl-CoA
ACACA
Acetyl-CoA
Citrate
FFA
Mitochondrion
Acyl-CoA
Isocitrate
2-Oxoglutarate
Fatty acid oxidation
Succinate
Citrate cycle (TCA cycle)
Acetyl CoA
PDHA1
Fumarate
Oxaloacetate
Malate
Oxidative phosphorylation
Glucose
SLC2A1
G6PC
GCK
G6P
FBP1
F6P
PFKL
F1,6P
3-PGA
PGAM4
2-PGA
PEP
PKM
Pyruvate
LDHAL6A
Lactate
PCK1
Oxaloacetate
Malate
Glycolysis / glucogenesis
Gluconeogenesis
+p
-p
04922 7/5/19
(c) Kanehisa Laboratories

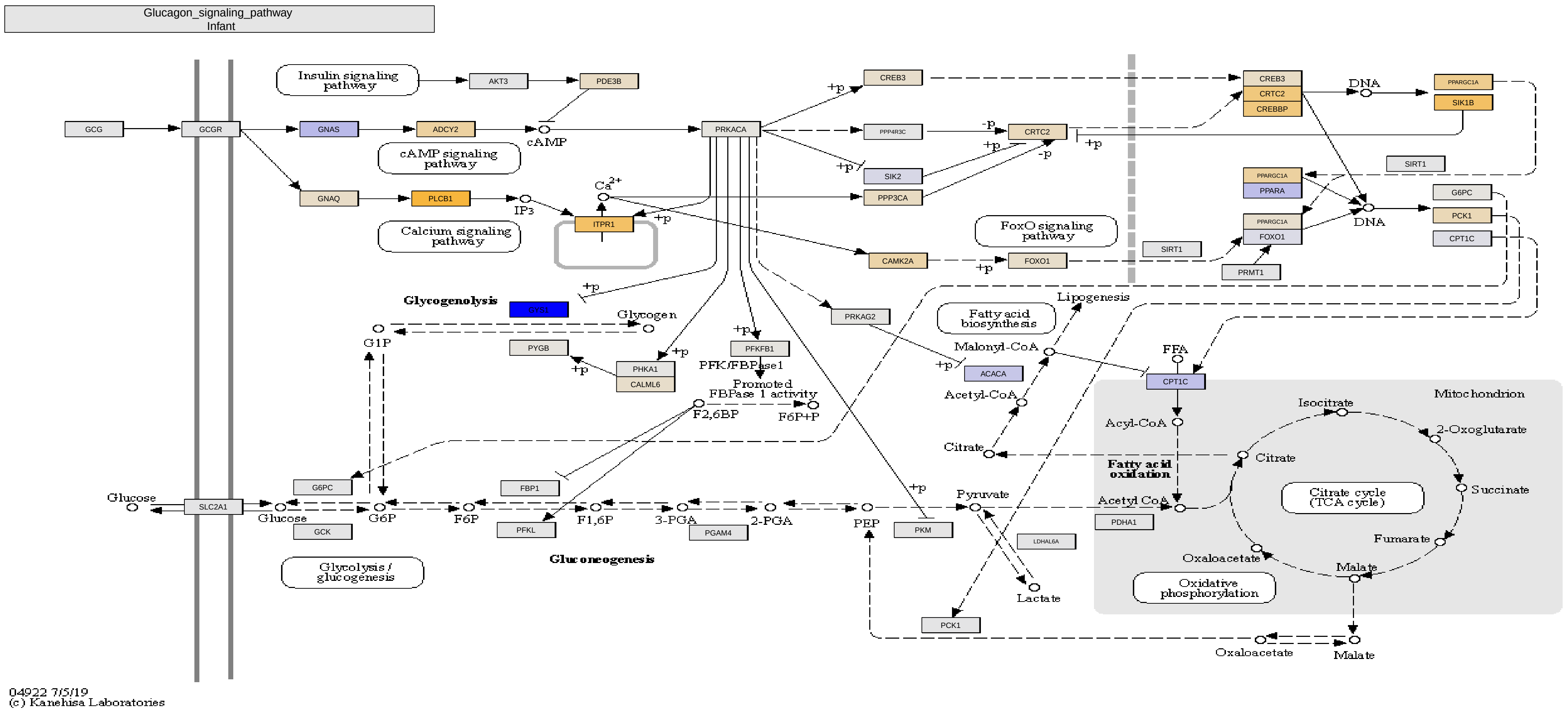
Glucagon_signaling_pathway
Infant
Insulin signaling pathway
AKT3
PDE3B
GCG
GCGR
GNAS
ADCY2
cAMP
PRKACA
cAMP signaling pathway
GNAQ
PLCB1
IP3
Ca2+
ITPR1
Calcium signaling pathway
CREB3
PPP4R3C
SIK2
PPP3CA
CRTC2
CAMK2A
FOXO1
FoxO signaling pathway
CREB3
CRTC2
CREBBP
DNA
PPARGC1A
SIK1B
SIRT1
PPARGC1A
PPARA
PPARGC1A
FOXO1
SIRT1
PRMT1
G6PC
PCK1
CPT1C
Glycogenolysis
GYS1
Glycogen
G1P
PYGB
PHKA1
CALML6
PFKFB1
PFK/FBPase1
Promoted FBPase 1 activity
F2,6BP
F6P+P
PRKAG2
Lipogenesis
Fatty acid biosynthesis
Malonyl-CoA
ACACA
Acetyl-CoA
Citrate
FFA
CPT1C
Mitochondrion
Acyl-CoA
Isocitrate
2-Oxoglutarate
Citrate
Fatty acid oxidation
Succinate
Citrate cycle (TCA cycle)
Acetyl CoA
PDHA1
Fumarate
Oxaloacetate
Malate
Oxidative phosphorylation
Glucose
SLC2A1
G6PC
Glucose
GCK
G6P
FBP1
F6P
PFKL
F1,6P
3-PGA
PGAM4
2-PGA
PEP
PKM
Pyruvate
LDHAL6A
Lactate
PCK1
Oxaloacetate
Malate
Glycolysis / glucogenesis
Gluconeogenesis
04922 7/5/19
(c) Kanehisa Laboratories

Glucagon_signaling_pathway
Teenager

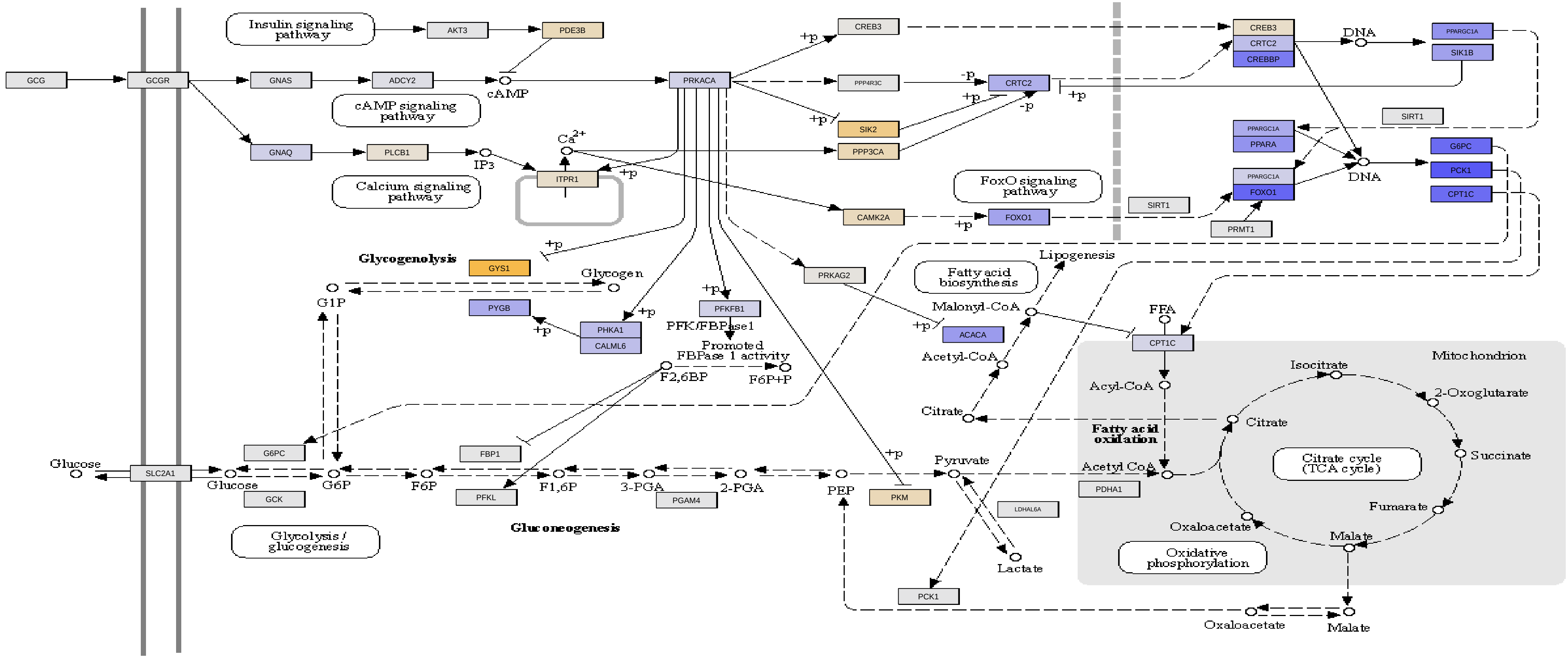

04922 7/5/19
(c) Kanehisa Laboratories

Glucagon_signaling_pathway
Adult

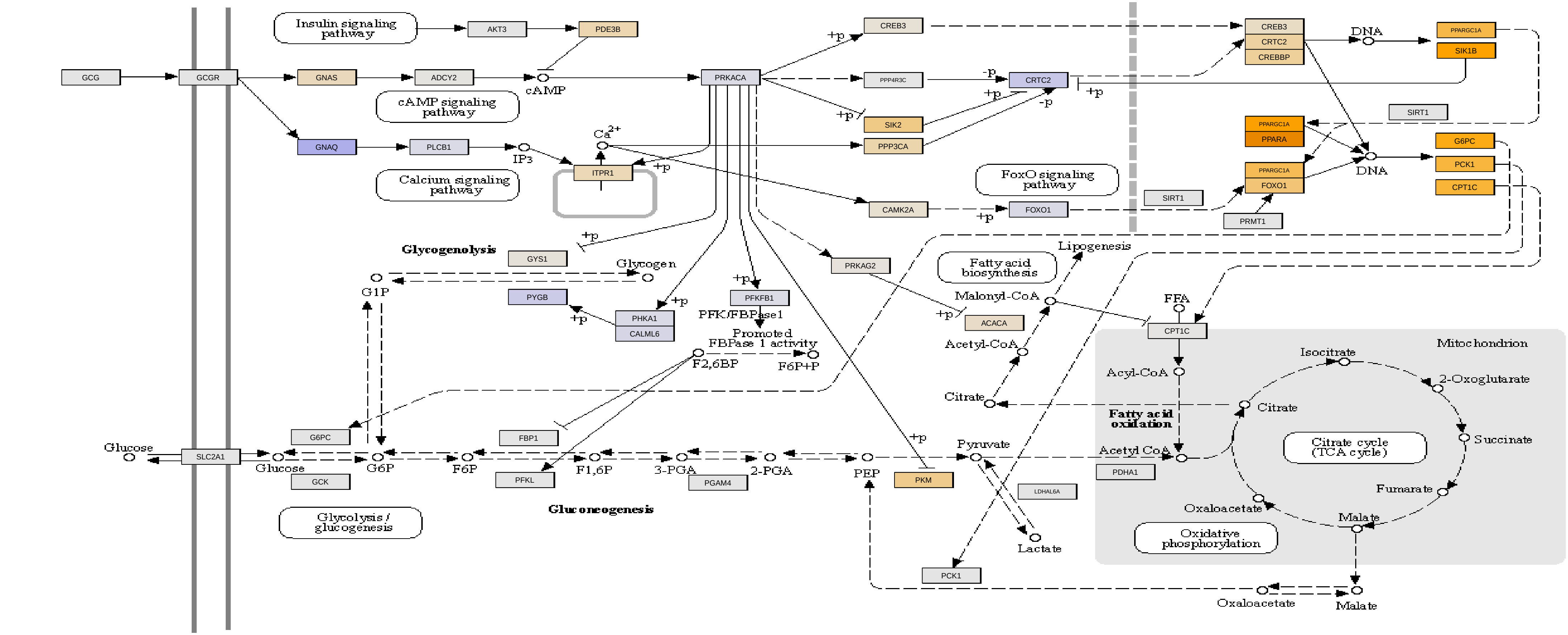

04922 7/5/19
(c) Kanehisa Laboratories

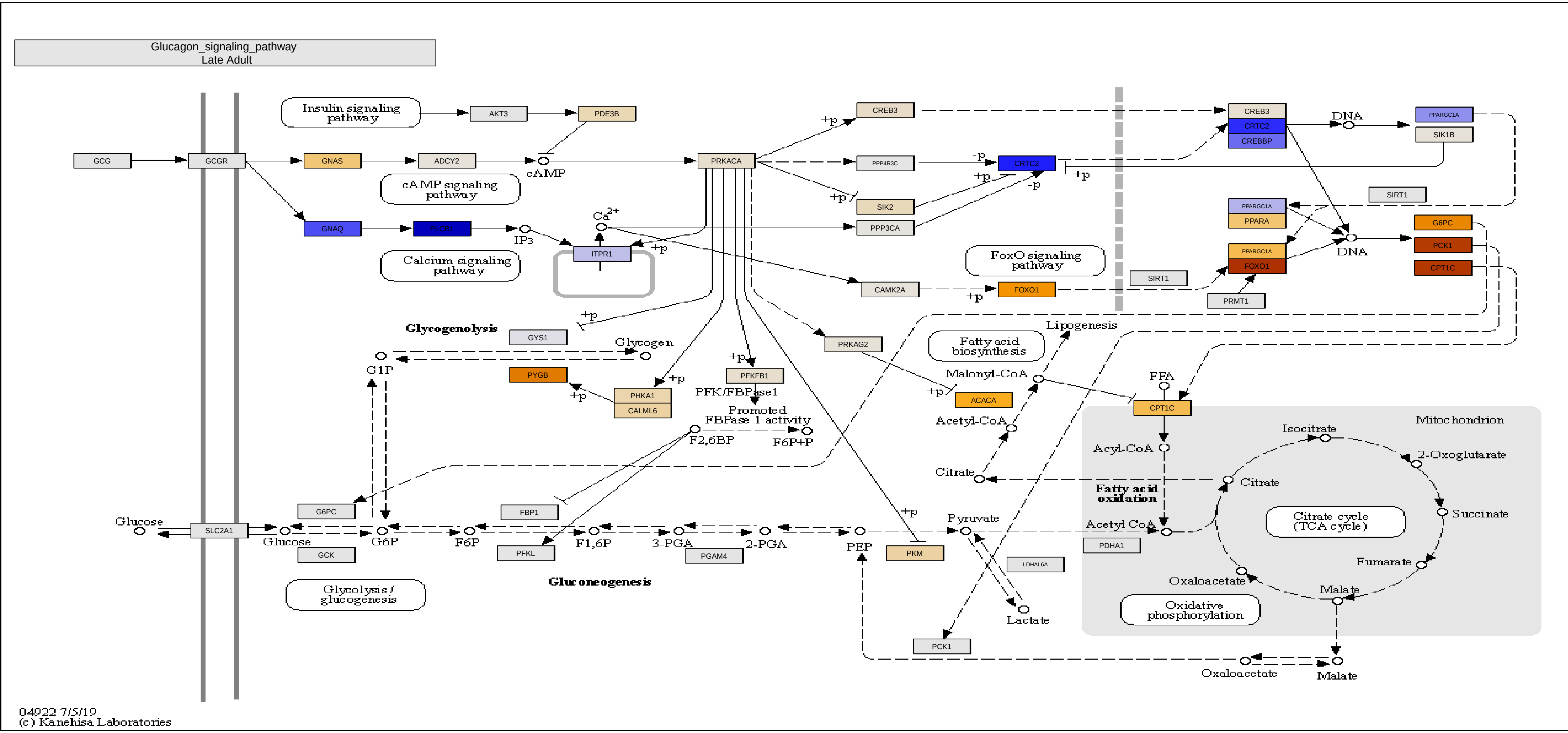
Glucagon_signaling_pathway
Late Adult
Insulin signaling pathway
AKT3
PDE3B
GCG
GCGR
GNAS
ADCY2
cAMP
PRKACA
CREB3
PPP4R3C
SIK2
PPP3CA
CRTC2
CREBBP
DNA
PPARGC1A
SIK1B
SIRT1
PPARA
FOXO1
PRMT1
G6PC
PCK1
CPT1C
cAMP signaling pathway
GNAQ
PLCB1
IP3
Ca2+
ITPR1
Calcium signaling pathway
CAMK2A
FoxO signaling pathway
+p
-p
Glycogenolysis
GYS1
Glycogen
G1P
PYGB
PHKA1
CALML6
PFKFB1
PFK/FBPase1
Promoted FBPase 1 activity
F2,6BP
F6P+P
PRKAG2
Fatty acid biosynthesis
Lipogenesis
Malonyl-CoA
ACACA
Acetyl-CoA
Citrate
FFA
Mitochondrion
Isocitrate
2-Oxoglutarate
Acyl-CoA
Fatty acid oxidation
Succinate
Citrate cycle (TCA cycle)
Acetyl CoA
PDHA1
Fumarate
Oxaloacetate
Malate
Oxidative phosphorylation
Glucose
SLC2A1
G6PC
FBP1
G6P
F6P
F1,6P
3-PGA
2-PGA
PEP
Pyruvate
PKM
LDHAL6A
Lactate
GCK
PFKL
PGAM4
Glycolysis / glucogenesis
Gluconeogenesis
PCK1
04922 7/5/19
(c) Kanehisa Laboratories
