# Supplementary material for: The Transcriptome and Methylome of the Developing and Aging Brain and Their Relations to Gliomas and Psychological Disorders
Source: Cells. 2022 Jan 21;11(3):362. doi: 10.3390/cells11030362 (PMC8834030; doi:10.3390/cells11030362)
Supplement: Supplementary file 1 [file cells-11-00362-s001.zip › Suppl-Material-S3-Pathways-PSF_Expression/Hedgehog_signaling_pathway.pdf]

Hedgehog signaling pathway

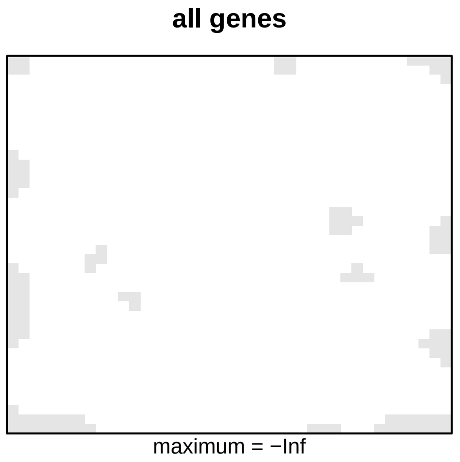

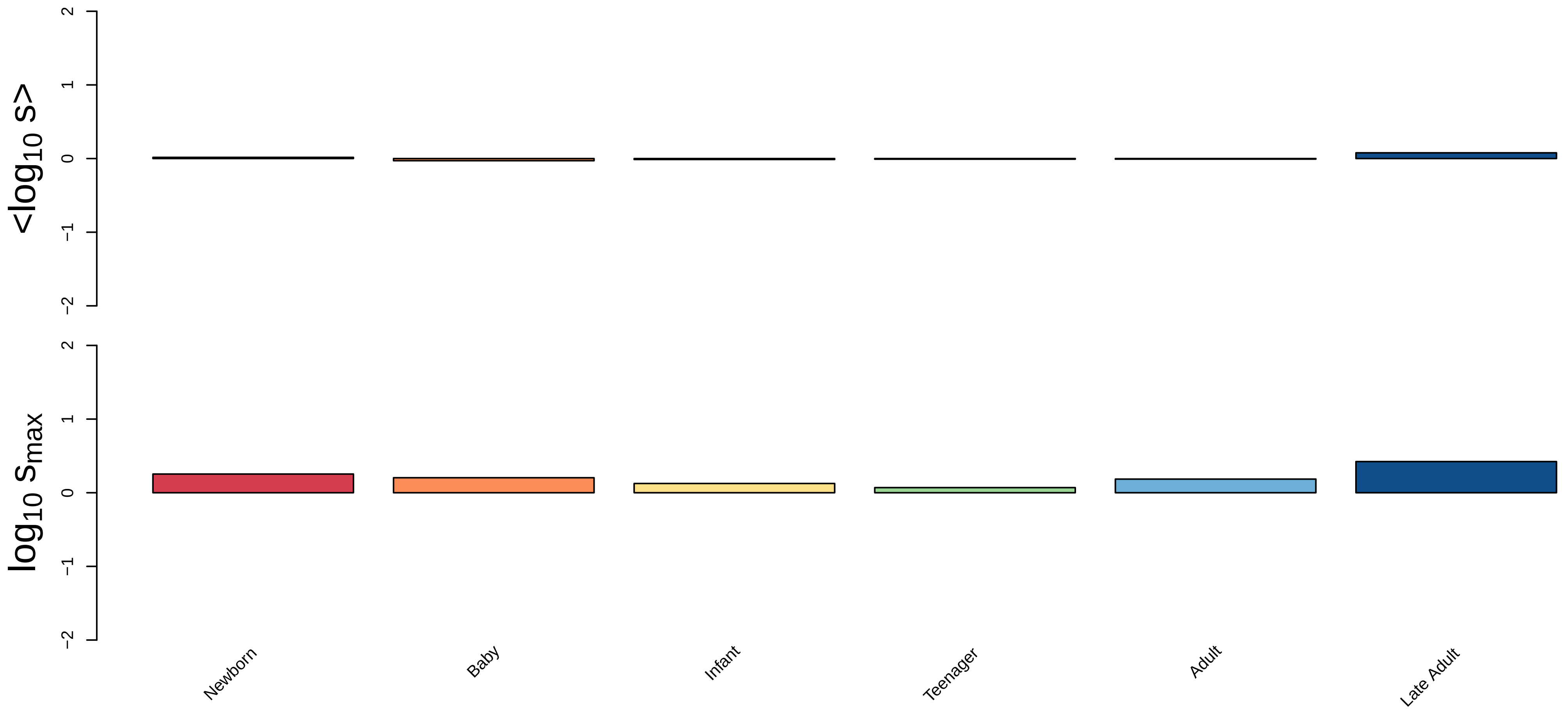

Hedgehog_signaling_pathway
genes with data

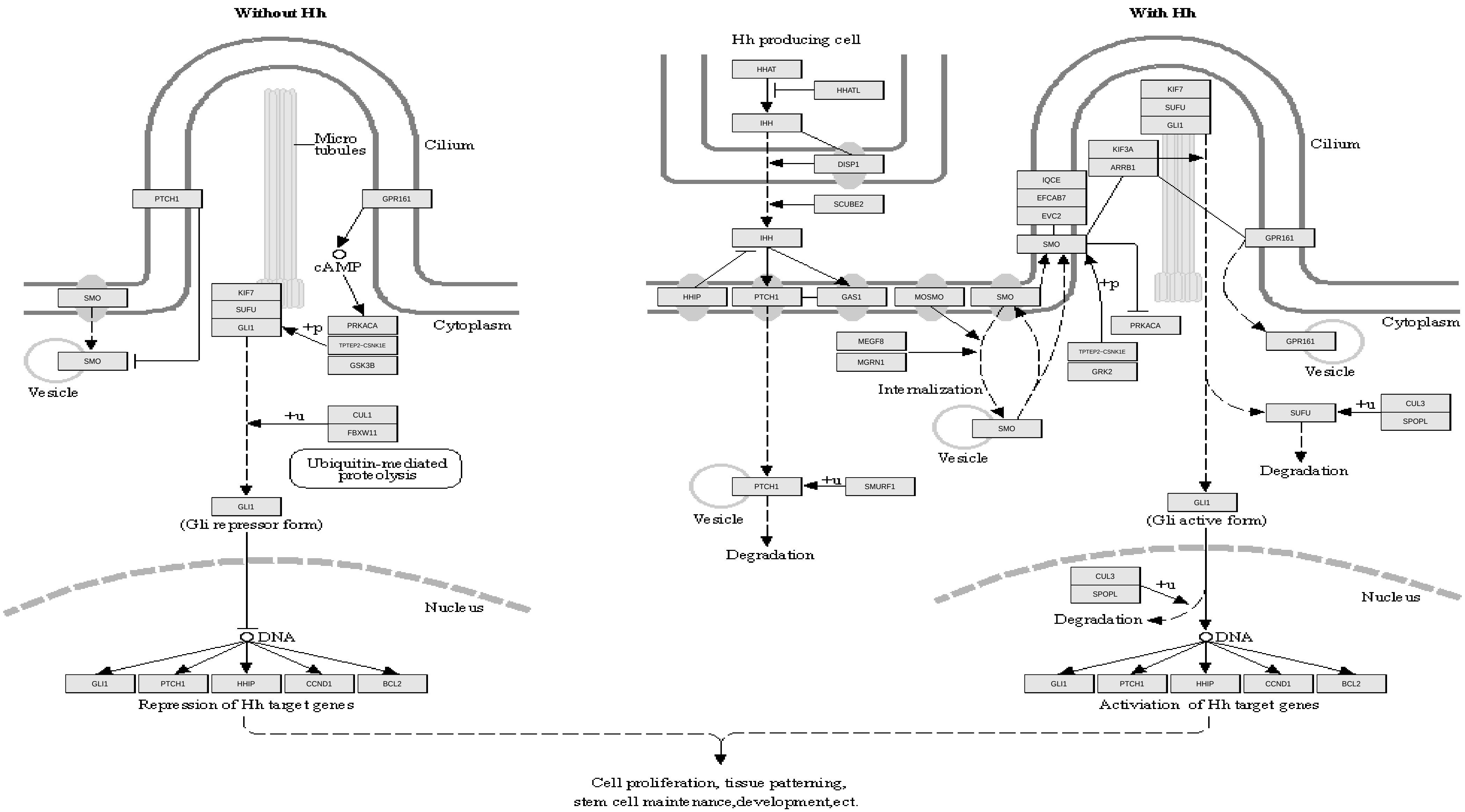

04340 2/2/21
(c) Kanehisa Laboratories

Hedgehog_signaling_pathway
sink nodes

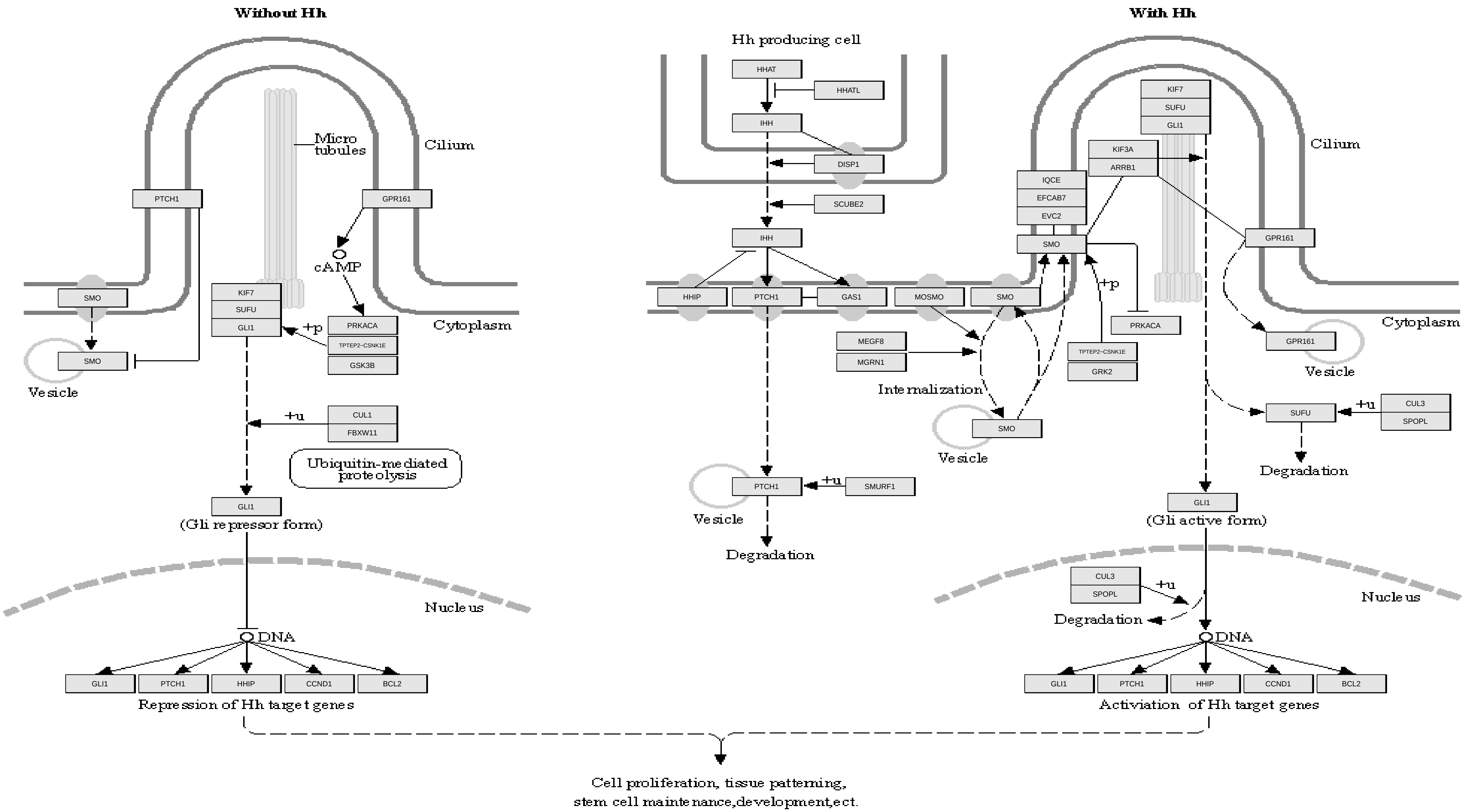

04340 2/2/21
(c) Kanehisa Laboratories

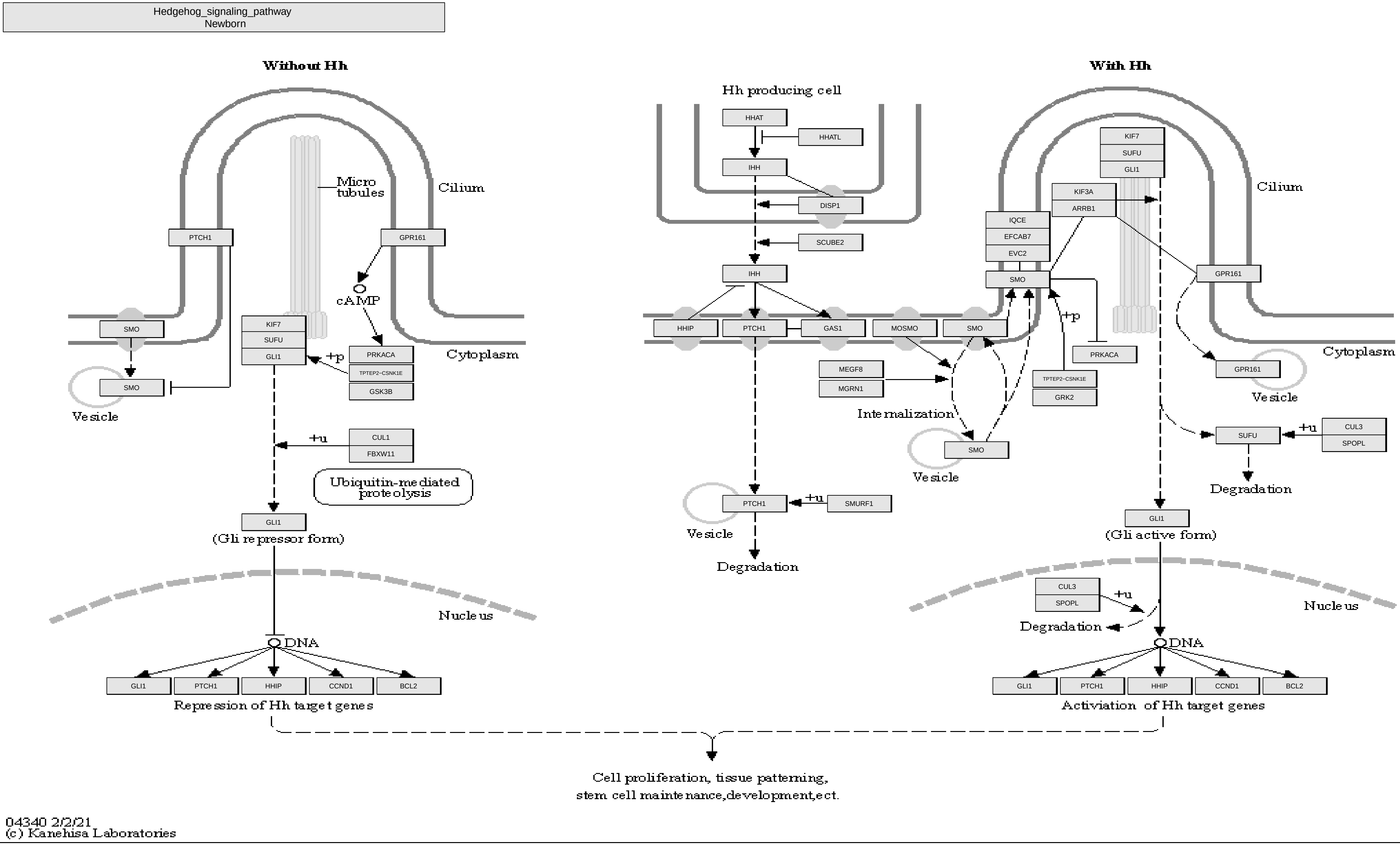
Hedgehog_signaling_pathway
Newborn
Without Hh
With Hh
Hh producing cell
Micro tubules
Cilium
PTCH1
GPR161
cAMP
SMO
KIF7
SUFU
GLI1
PRKACA
TPTEP2-CSNK1E
GSK3B
+p
Cytoplasm
Vesicle
+u
CUL1
FBXW11
Ubiquitin-mediated proteolysis
(Gli repressor form)
Nucleus
DNA
HHIP
CCND1
BCL2
Repression of Hh target genes
HHAT
HHATL
IHH
DISP1
SCUBE2
GAS1
MOSMO
MEGF8
MGRN1
Internalization
SMURF1
Degradation
KIF3A
ARRB1
IQCE
EFCAB7
EVC2
GRK2
CUL3
SPOPL
(Gli active form)
Activiation of Hh target genes
Cell proliferation, tissue patterning,
stem cell maintenance,development,ect.
04340 2/2/21
(c) Kanehisa Laboratories

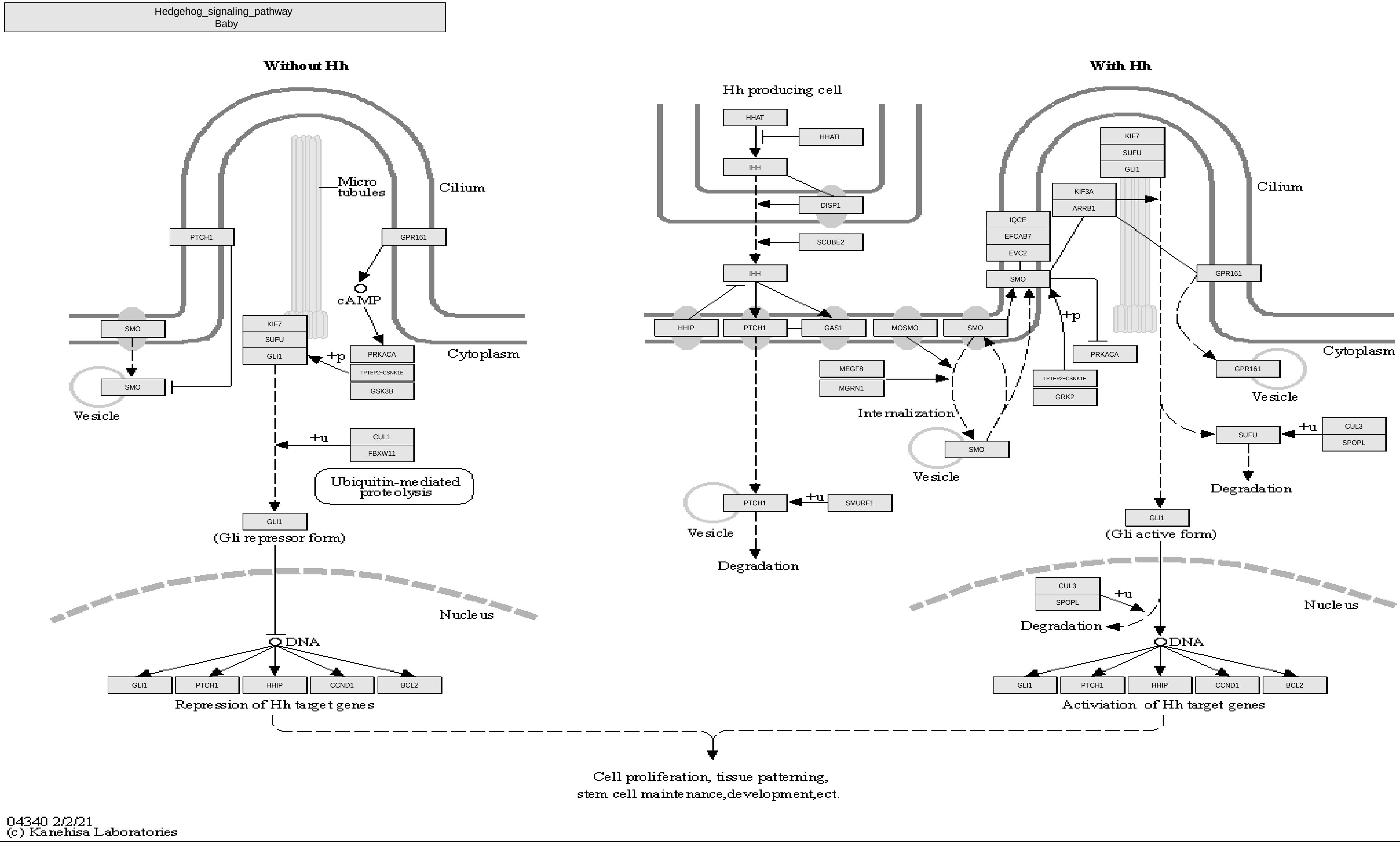
Hedgehog_signaling_pathway
Baby
Without Hh
With Hh
Hh producing cell
Micro tubules
Cilium
Cytoplasm
Vesicle
Nucleus
PTCH1
GPR161
SMO
KIF7
SUFU
GLI1
cAMP
+p
PRKACA
TPTEP2-CSNK1E
GSK3B
+u
CUL1
FBXW11
Ubiquitin-mediated proteolysis
(Gli repressor form)
DNA
GLI1
PTCH1
HHIP
CCND1
BCL2
Repression of Hh target genes
HHAT
HHATL
IHH
DISP1
SCUBE2
GAS1
MOSMO
MEGF8
MGRN1
Internalization
SMURF1
Degradation
IQCE
EFCAB7
EVC2
KIF3A
ARRB1
GRK2
CUL3
SPOPL
(Gli active form)
Activiation of Hh target genes
Cell proliferation, tissue patterning,
stem cell maintenance,development,ect.
04340 2/2/21
(c) Kanehisa Laboratories

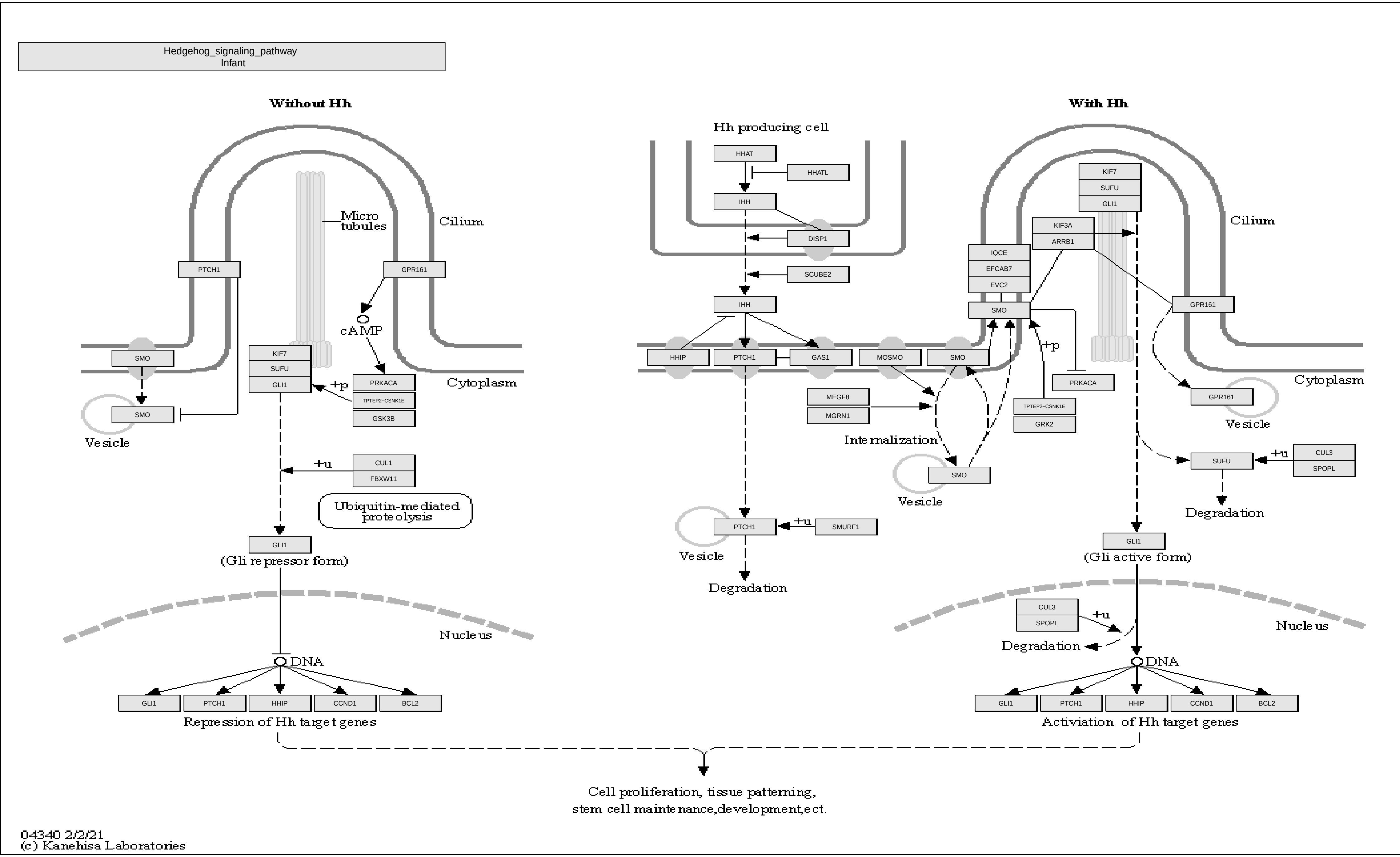
Hedgehog_signaling_pathway
Infant
Without Hh
With Hh
Hh producing cell
HHAT
HHATL
IHH
DISP1
SCUBE2
IHH
HHIP
PTCH1
GAS1
MOSMO
SMO
MEGF8
MGRN1
Internalization
SMO
Vesicle
PTCH1
+u
SMURF1
Vesicle
Degradation
Micro tubules
Cilium
PTCH1
GPR161
cAMP
PRKACA
TPTEP2-CSNK1E
GSK3B
+p
KIF7
SUFU
GLI1
SMO
SMO
Vesicle
Cytoplasm
+u
CUL1
FBXW11
Ubiquitin-mediated proteolysis
GLI1
(Gli repressor form)
Nucleus
DNA
GLI1
PTCH1
HHIP
CCND1
BCL2
Repression of Hh target genes
KIF7
SUFU
GLI1
KIF3A
ARRB1
IQCE
EFCAB7
EVC2
SMO
GPR161
+p
PRKACA
TPTEP2-CSNK1E
GRK2
Cilium
GPR161
Vesicle
Cytoplasm
SUFU
+u
CUL3
SPOPL
Degradation
GLI1
(Gli active form)
CUL3
SPOPL
+u
Degradation
Nucleus
DNA
GLI1
PTCH1
HHIP
CCND1
BCL2
Activiation of Hh target genes
Cell proliferation, tissue patterning,
stem cell maintenance,development,ect.
04340 2/2/21
(c) Kanehisa Laboratories

Hedgehog_signaling_pathway
Teenager

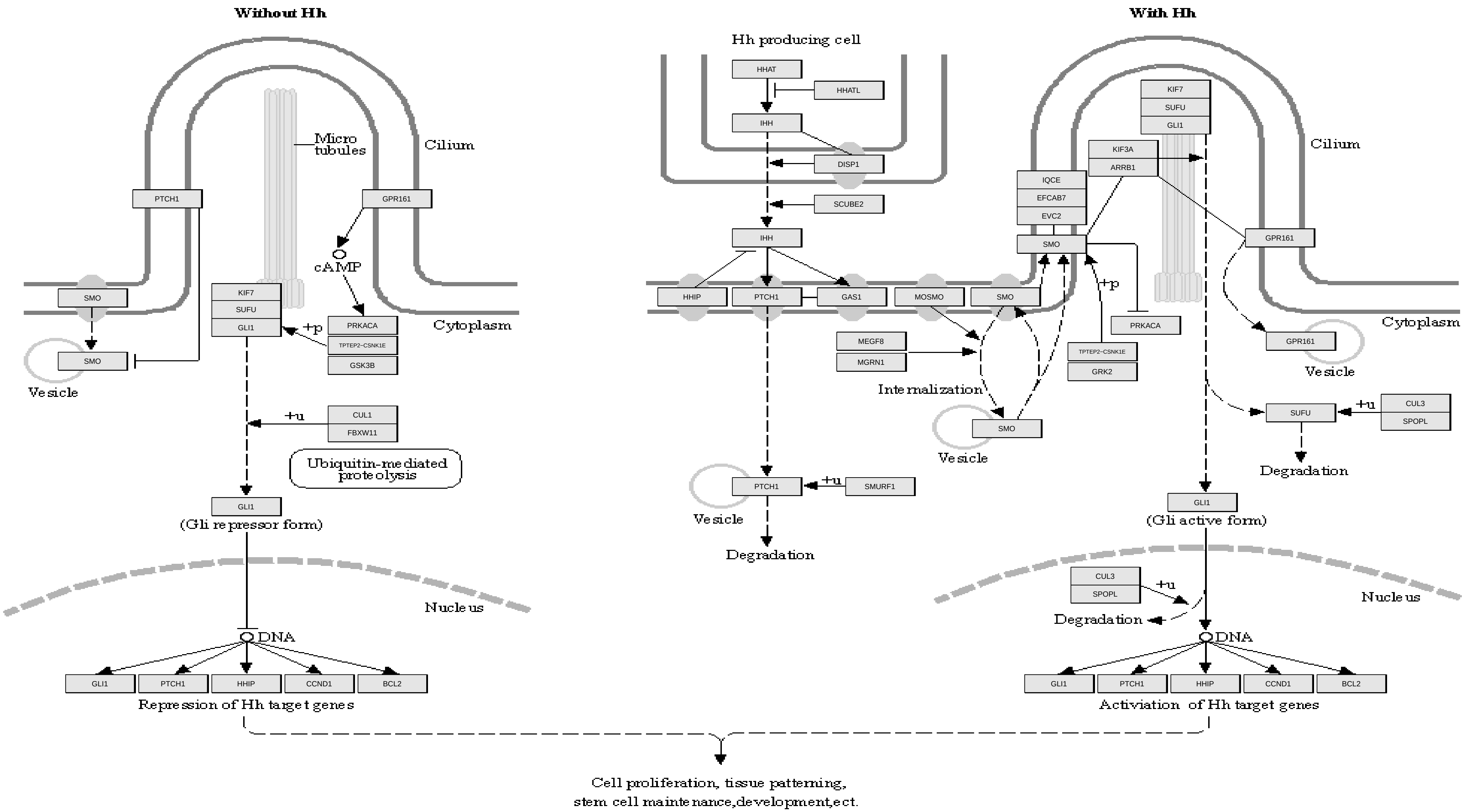

04340 2/2/21
(c) Kanehisa Laboratories

Hedgehog_signaling_pathway
Adult

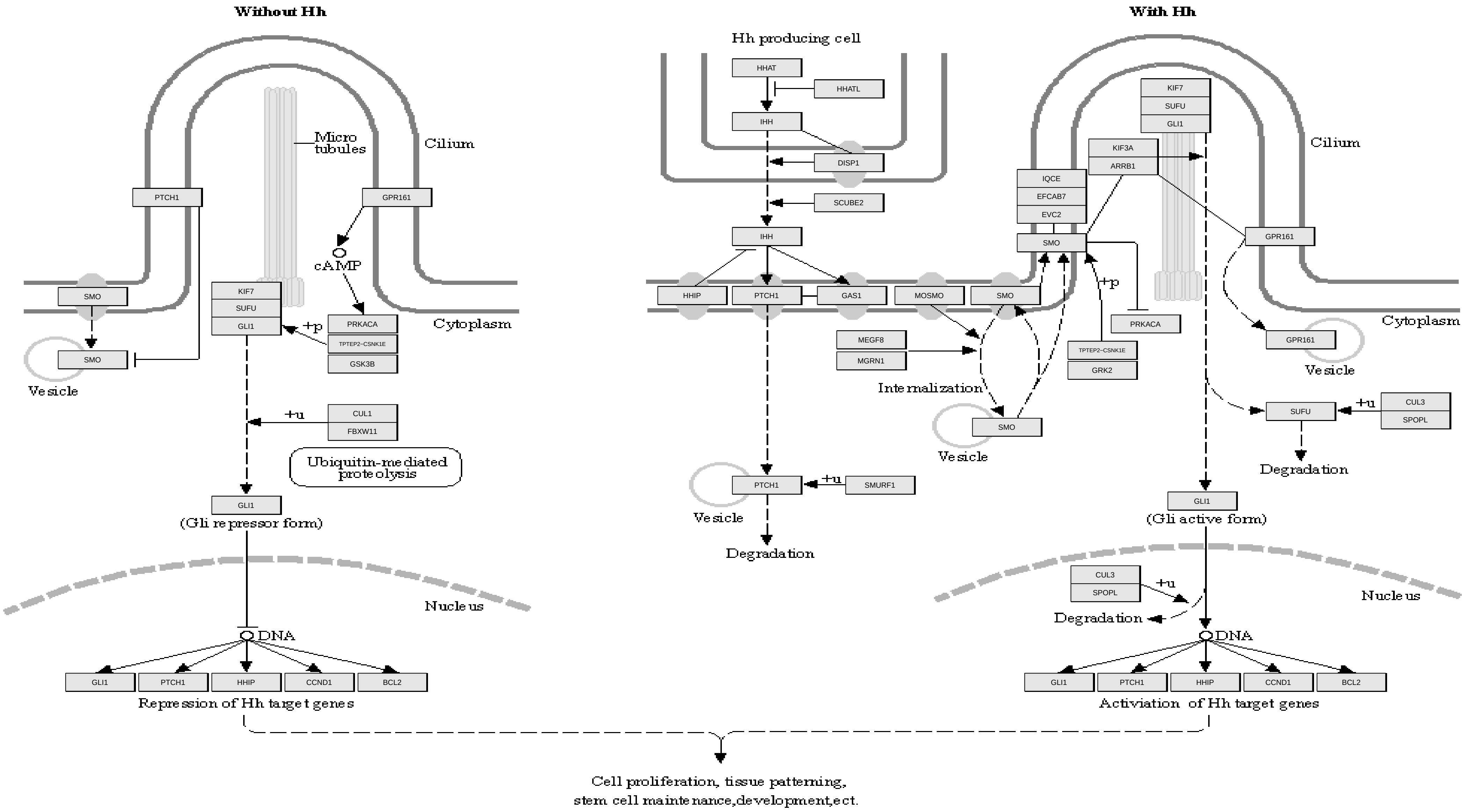

04340 2/2/21
(c) Kanehisa Laboratories

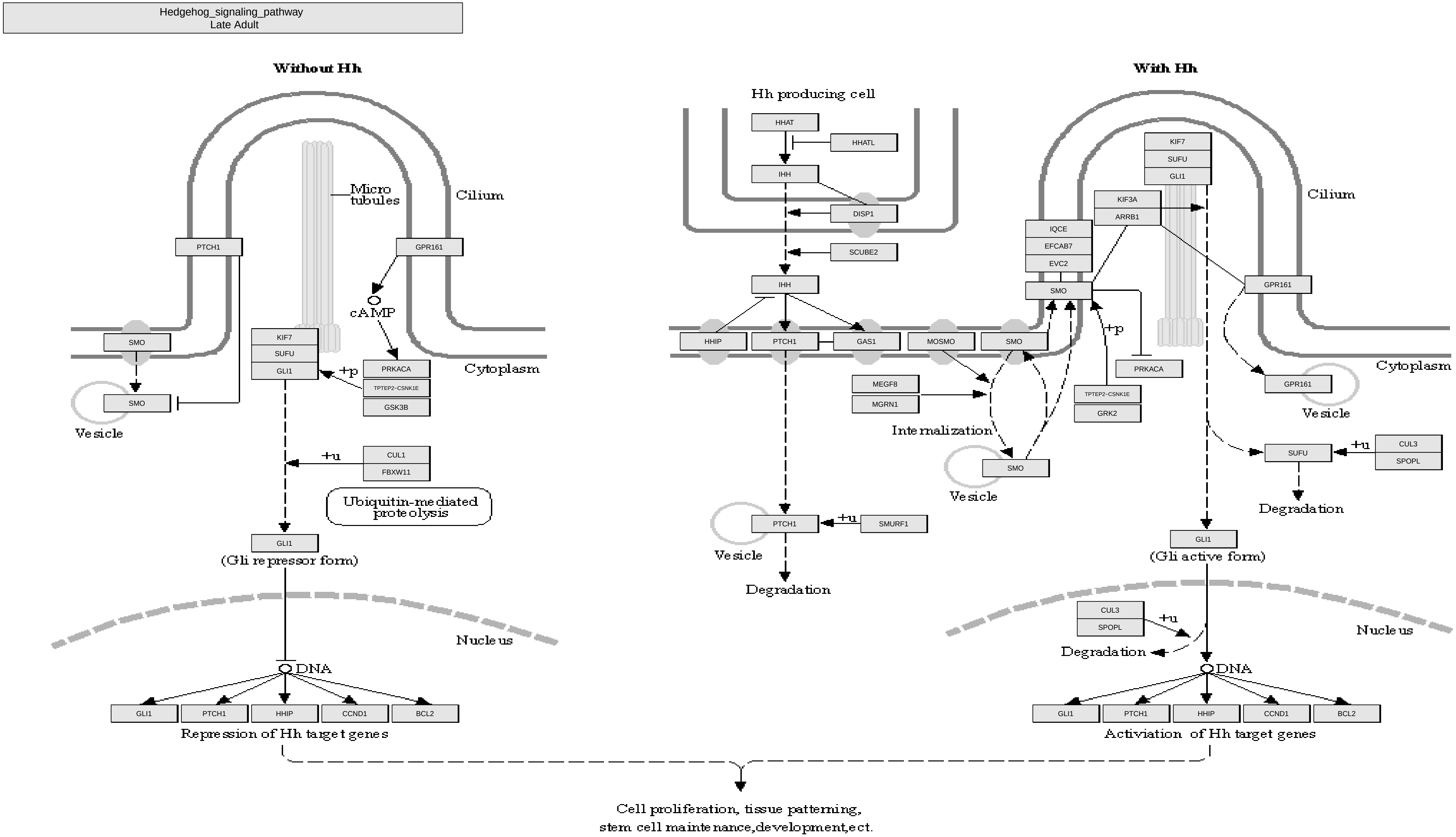

04340 2/2/21

(c) Kanehisa Laboratories
