# Supplementary material for: The Transcriptome and Methylome of the Developing and Aging Brain and Their Relations to Gliomas and Psychological Disorders
Source: Cells. 2022 Jan 21;11(3):362. doi: 10.3390/cells11030362 (PMC8834030; doi:10.3390/cells11030362)
Supplement: Supplementary file 1 [file cells-11-00362-s001.zip › Suppl-Material-S3-Pathways-PSF_Expression/HIF_1_signaling_pathway.pdf]

HIF−1 signaling pathway

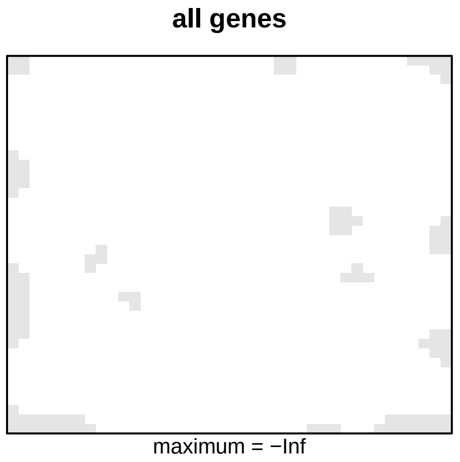

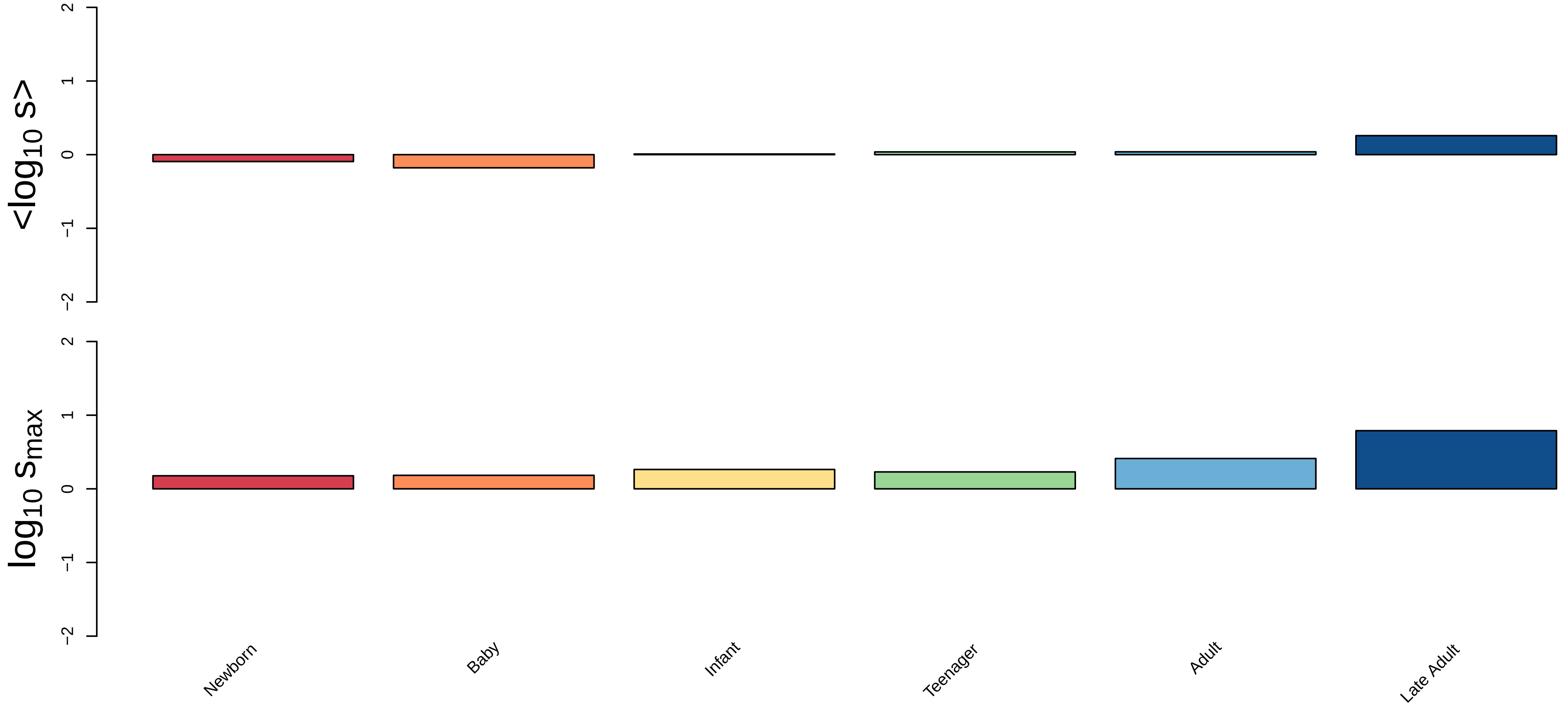

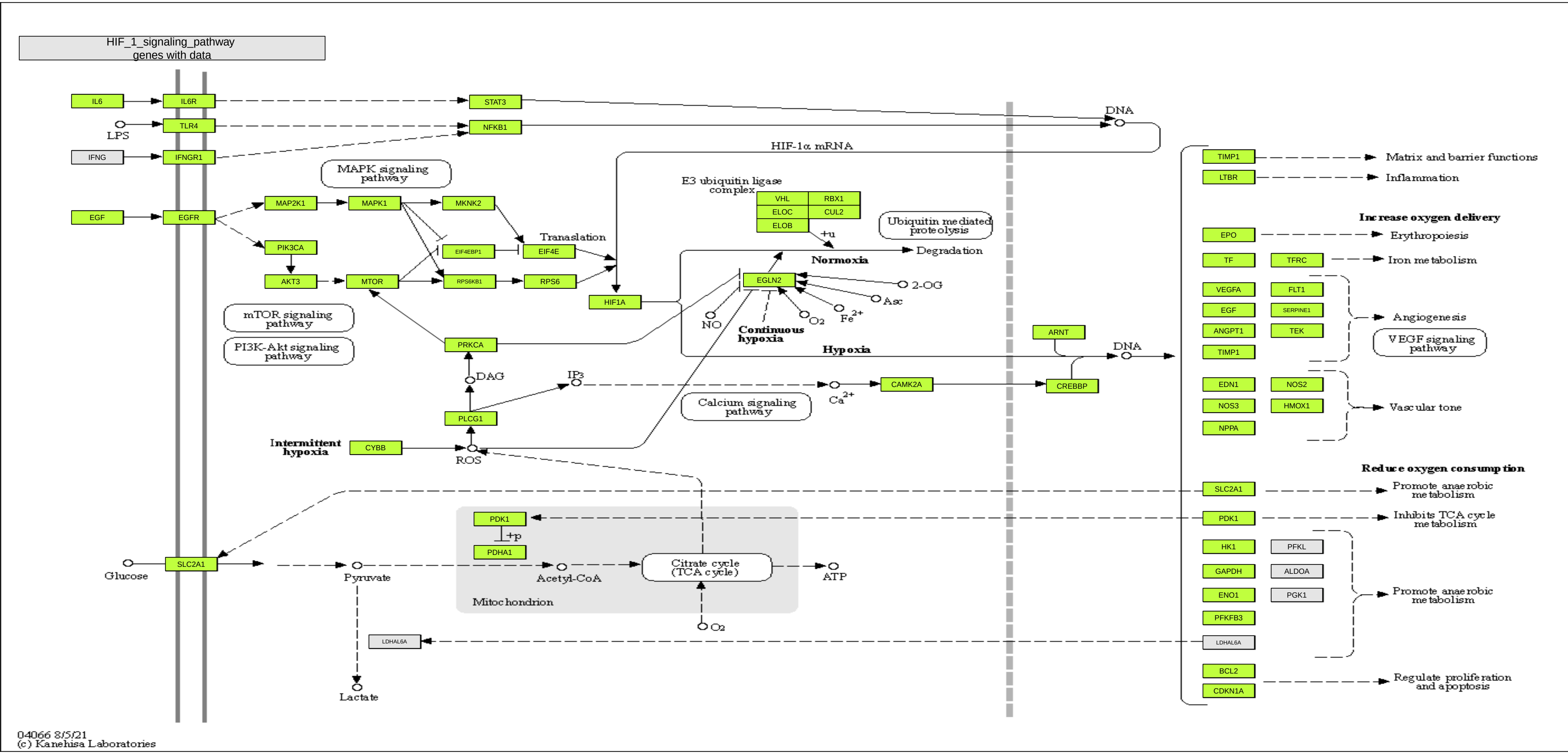

HIF_1_signaling_pathway
genes with data
IL6
IL6R
STAT3
LPS
TLR4
NFKB1
IFNG
IFNGR1
DNA
HIF-1α mRNA
MAPK signaling pathway
EGF
EGFR
MAP2K1
MAPK1
MKNK2
PIK3CA
EIF4EBP1
EIF4E
Translation
AKT3
MTOR
RPS6KB1
RPS6
HIF1A
E3 ubiquitin ligase complex
VHL
RBX1
ELOC
CUL2
ELOB
+u
Ubiquitin mediated proteolysis
Normoxia
Degradation
EGLN2
2-OG
Asc
Fe^{2+}
O_2
NO
Continuous hypoxia
Hypoxia
mTOR signaling pathway
PI3K-Akt signaling pathway
PRKCA
DAG
IP_3
PLCG1
Calcium signaling pathway
Ca^{2+}
CAMK2A
ARNT
CREBBP
Intermittent hypoxia
CYBB
ROS
TIMP1
Matrix and barrier functions
LTBR
Inflammation
Increase oxygen delivery
EPO
Erythropoiesis
TF
TFRC
Iron metabolism
VEGFA
FLT1
EGF
SERPINE1
Angiogenesis
ANGPT1
TEK
VEGF signaling pathway
TIMP1
EDN1
NOS2
NOS3
HMOX1
Vascular tone
NPPA
Reduce oxygen consumption
SLC2A1
Promote anaerobic metabolism
PDK1
Inhibits TCA cycle metabolism
HK1
PFKL
GAPDH
ALDOA
ENO1
PGK1
Promote anaerobic metabolism
PFKFB3
LDHAL6A
BCL2
CDKN1A
Regulate proliferation and apoptosis
Glucose
SLC2A1
Pyruvate
PDK1
+p
PDHA1
Acetyl-CoA
Citrate cycle (TCA cycle)
ATP
Mitochondrion
O_2
LDHAL6A
Lactate
04066 8/5/21
(c) Kanehisa Laboratories

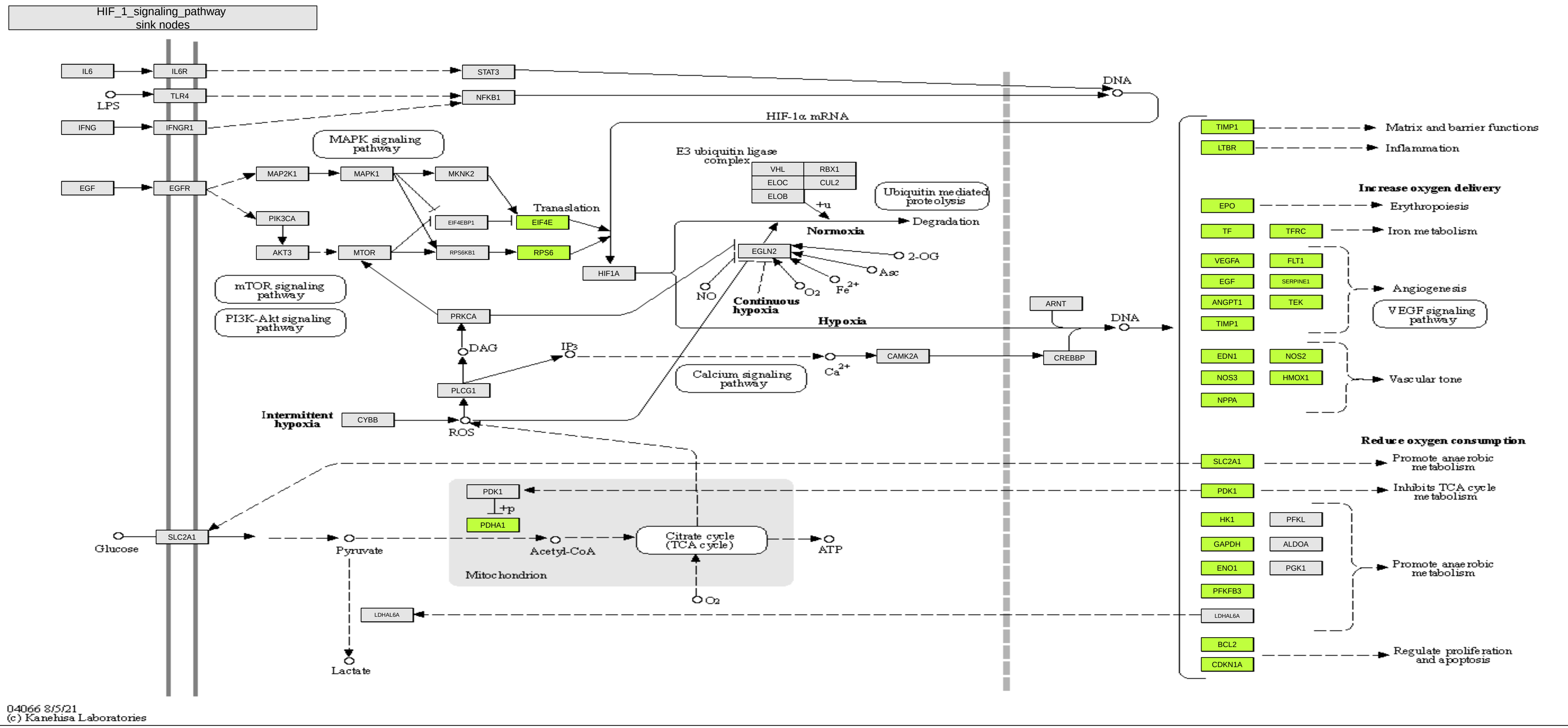
HIF_1_signaling_pathway
sink nodes
IL6
IL6R
STAT3
LPS
TLR4
NFKB1
IFNG
IFNGR1
DNA
HIF-1α mRNA
MAPK signaling pathway
EGF
EGFR
MAP2K1
MAPK1
MKNK2
PIK3CA
EIF4EBP1
EIF4E
Translation
AKT3
MTOR
RPS6KB1
RPS6
HIF1A
E3 ubiquitin ligase complex
VHL
RBX1
ELOC
CUL2
ELOB
+u
Ubiquitin mediated proteolysis
Normoxia
Degradation
EGLN2
2-OG
Asc
O_2
Fe^{2+}
NO
Continuous hypoxia
Hypoxia
mTOR signaling pathway
PI3K-Akt signaling pathway
PRKCA
ARNT
DNA
DAG
IP_3
CAMK2A
CREBBP
Ca^{2+}
Calcium signaling pathway
PLCG1
Intermittent hypoxia
CYBB
ROS
PDK1
+p
PDHA1
Glucose
SLC2A1
Pyruvate
Acetyl-CoA
Citrate cycle (TCA cycle)
ATP
Mitochondrion
O_2
LDHAL6A
Lactate
TIMP1
Matrix and barrier functions
LTBR
Inflammation
Increase oxygen delivery
EPO
Erythropoiesis
TF
TFRC
Iron metabolism
VEGFA
FLT1
EGF
SERPINE1
Angiogenesis
ANGPT1
TEK
VEGF signaling pathway
TIMP1
EDN1
NOS2
NOS3
HMOX1
Vascular tone
NPPA
Reduce oxygen consumption
SLC2A1
Promote anaerobic metabolism
PDK1
Inhibits TCA cycle metabolism
HK1
PFKL
GAPDH
ALDOA
ENO1
PGK1
Promote anaerobic metabolism
PFKFB3
LDHAL6A
BCL2
Regulate proliferation and apoptosis
CDKN1A
04066 8/5/21
(c) Kanehisa Laboratories

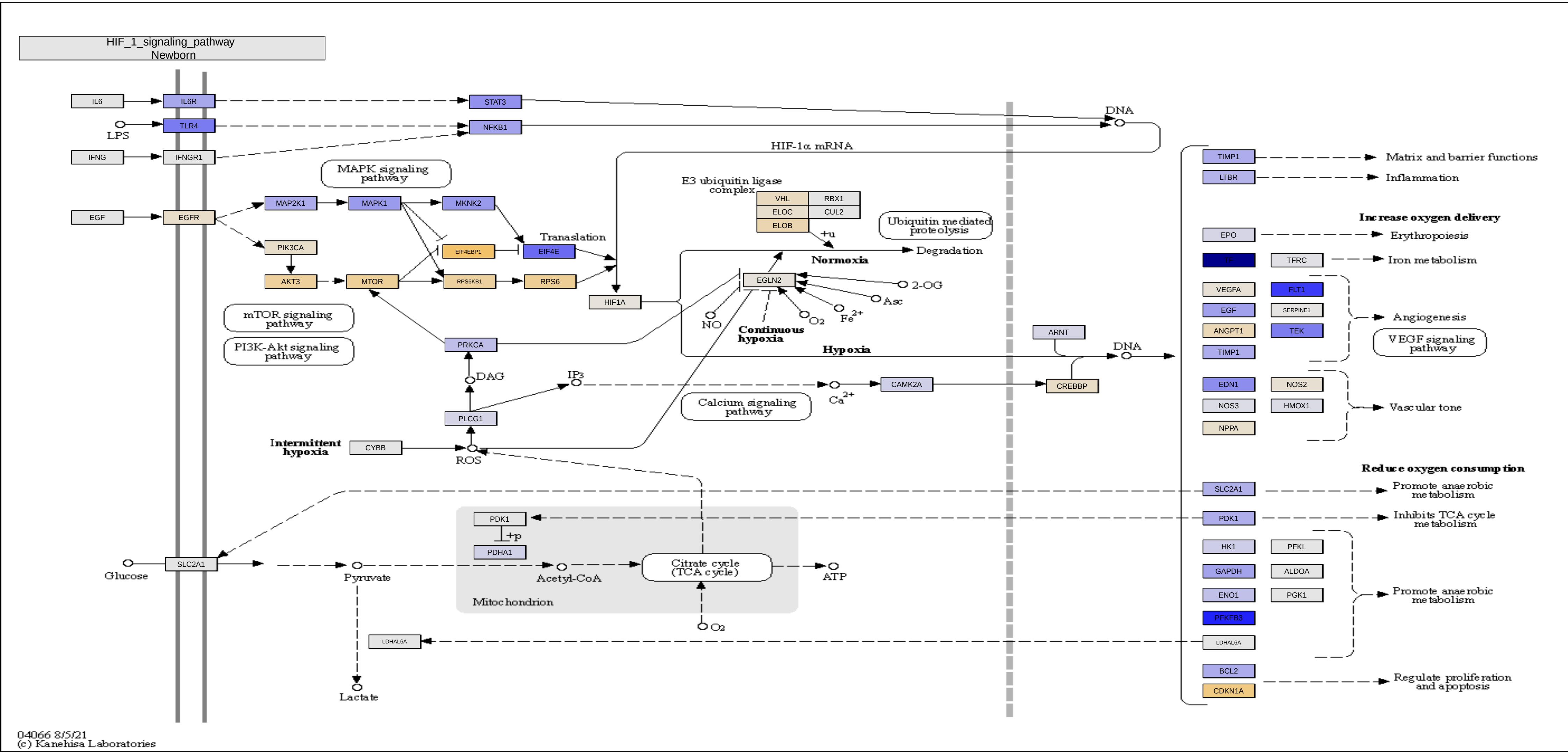
HIF_1_signaling_pathway
Newborn
IL6
IL6R
STAT3
LPS
TLR4
NFKB1
IFNG
IFNGR1
DNA
HIF-1α mRNA
MAPK signaling pathway
EGF
EGFR
MAP2K1
MAPK1
MKNK2
PIK3CA
AKT3
MTOR
EIF4EBP1
EIF4E
RPS6KB1
RPS6
Translation
HIF1A
E3 ubiquitin ligase complex
VHL
RBX1
ELOC
CUL2
ELOB
+u
Ubiquitin mediated proteolysis
Normoxia
Degradation
EGLN2
2-OG
Asc
O2
Fe2+
NO
Continuous hypoxia
Hypoxia
mTOR signaling pathway
PI3K-Akt signaling pathway
PRKCA
DAG
IP3
PLCG1
Ca2+
CAMK2A
CREBBP
ARNT
Calcium signaling pathway
Intermittent hypoxia
CYBB
ROS
Glucose
SLC2A1
Pyruvate
PDK1
+p
PDHA1
Acetyl-CoA
Citrate cycle (TCA cycle)
ATP
Mitochondrion
O2
LDHAL6A
Lactate
TIMP1
Matrix and barrier functions
LTBR
Inflammation
Increase oxygen delivery
EPO
Erythropoiesis
TFRC
Iron metabolism
VEGFA
FLT1
EGF
SERPINE1
Angiogenesis
ANGPT1
TEK
VEGF signaling pathway
TIMP1
EDN1
NOS2
NOS3
HMOX1
Vascular tone
NPPA
Reduce oxygen consumption
SLC2A1
Promote anaerobic metabolism
PDK1
Inhibits TCA cycle metabolism
HK1
PFKL
GAPDH
ALDOA
ENO1
PGK1
PFKFB3
Promote anaerobic metabolism
LDHAL6A
BCL2
Regulate proliferation and apoptosis
CDKN1A
04066 8/5/21
(c) Kanehisa Laboratories

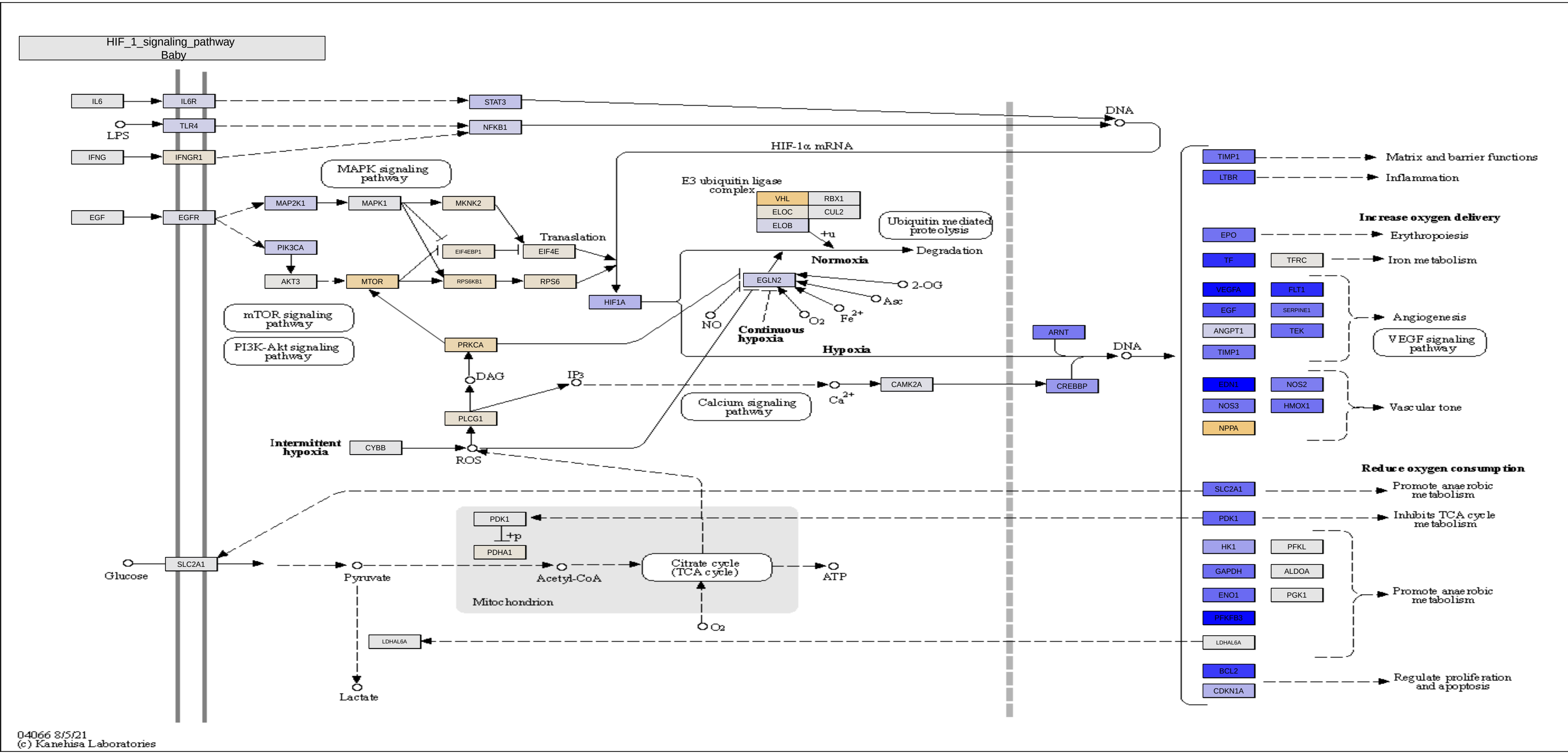
HIF_1_signaling_pathway
Baby
IL6
IL6R
STAT3
LPS
TLR4
NFKB1
IFNG
IFNGR1
DNA
HIF-1α mRNA
MAPK signaling pathway
E3 ubiquitin ligase complex
VHL
RBX1
ELOC
CUL2
ELOB
+u
Ubiquitin mediated proteolysis
EGF
EGFR
MAP2K1
MAPK1
MKNK2
PIK3CA
EIF4EBP1
EIF4E
Translation
AKT3
MTOR
RPS6KB1
RPS6
HIF1A
Normoxia
Degradation
EGLN2
2-OG
Asc
Fe2+
O2
NO
Continuous hypoxia
Hypoxia
mTOR signaling pathway
PI3K-Akt signaling pathway
PRKCA
DAG
IP3
PLCG1
Calcium signaling pathway
Ca2+
CAMK2A
ARNT
CREBBP
DNA
Intermittent hypoxia
CYBB
ROS
Glucose
SLC2A1
Pyruvate
PDK1
+p
PDHA1
Acetyl-CoA
Citrate cycle (TCA cycle)
ATP
Mitochondrion
O2
LDHAL6A
Lactate
TIMP1
Matrix and barrier functions
LTBR
Inflammation
Increase oxygen delivery
EPO
Erythropoiesis
TF
TFRC
Iron metabolism
VEGFA
FLT1
EGF
SERPINE1
Angiogenesis
ANGPT1
TEK
VEGF signaling pathway
TIMP1
EDN1
NOS2
NOS3
HMOX1
Vascular tone
NPPA
Reduce oxygen consumption
SLC2A1
Promote anaerobic metabolism
PDK1
Inhibits TCA cycle metabolism
HK1
PFKL
GAPDH
ALDOA
ENO1
PGK1
Promote anaerobic metabolism
PFKFB3
LDHAL6A
BCL2
Regulate proliferation and apoptosis
CDKN1A
04066 8/5/21
(c) Kanehisa Laboratories

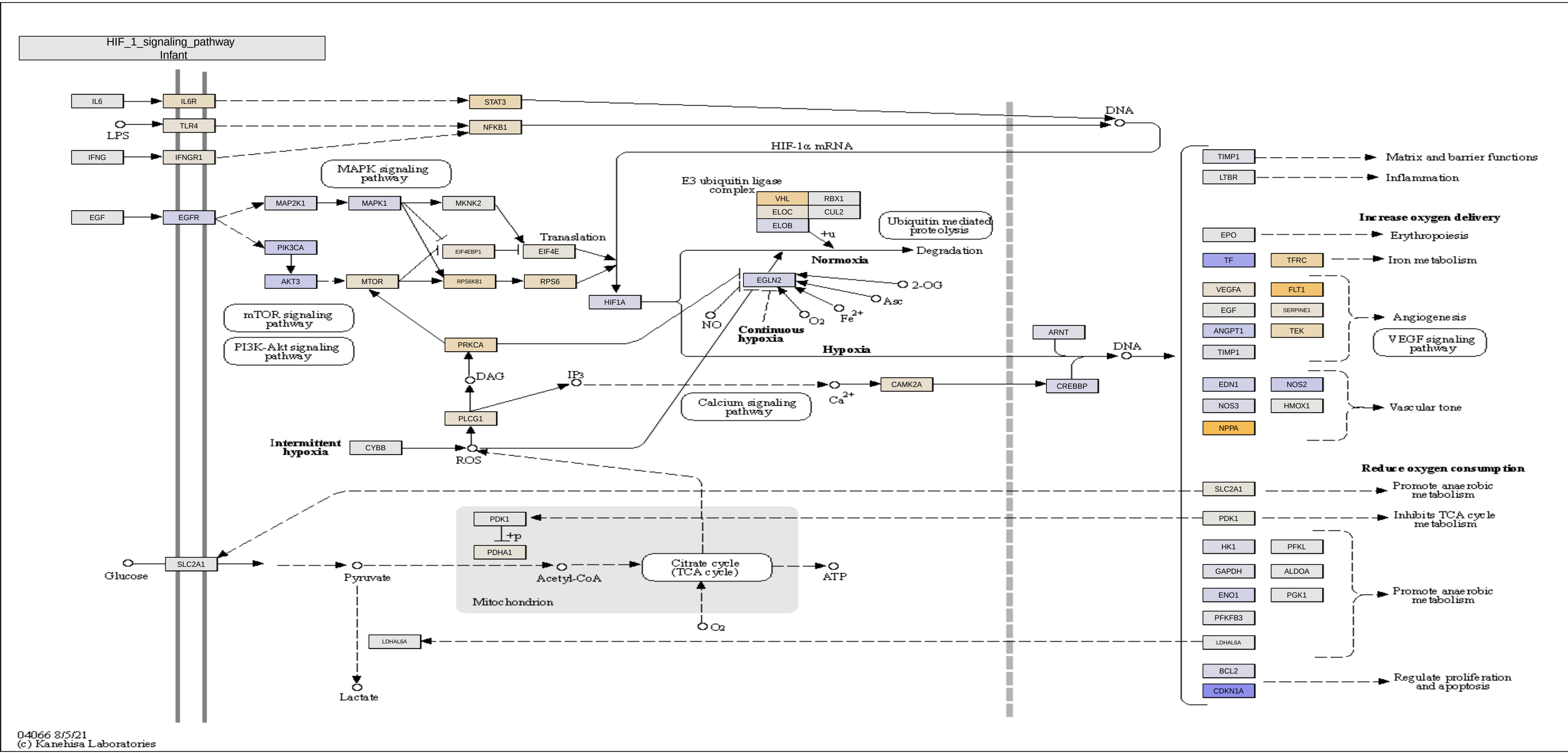
HIF_1_signaling_pathway
Infant
IL6
IL6R
STAT3
LPS
TLR4
NFKB1
IFNG
IFNGR1
DNA
HIF-1α mRNA
MAPK signaling pathway
EGF
EGFR
MAP2K1
MAPK1
MKNK2
PIK3CA
AKT3
MTOR
EIF4EBP1
EIF4E
RPS6KB1
RPS6
Translation
HIF1A
mTOR signaling pathway
PI3K-Akt signaling pathway
PRKCA
DAG
IP3
PLCG1
Intermittent hypoxia
CYBB
ROS
E3 ubiquitin ligase complex
VHL
RBX1
ELOC
CUL2
ELOB
+u
Ubiquitin mediated proteolysis
Normoxia
Degradation
EGLN2
2-OG
Asc
Fe2+
O2
NO
Continuous hypoxia
Hypoxia
ARNT
DNA
CREBBP
CAMK2A
Ca2+
Calcium signaling pathway
Glucose
SLC2A1
Pyruvate
PDK1
+p
PDHA1
Acetyl-CoA
Citrate cycle (TCA cycle)
ATP
Mitochondrion
O2
LDHAL6A
Lactate
TIMP1
Matrix and barrier functions
LTBR
Inflammation
Increase oxygen delivery
EPO
Erythropoiesis
TF
TFRC
Iron metabolism
VEGFA
FLT1
EGF
SERPINE1
Angiogenesis
ANGPT1
TEK
VEGF signaling pathway
TIMP1
EDN1
NOS2
NOS3
HMOX1
Vascular tone
NPPA
Reduce oxygen consumption
SLC2A1
Promote anaerobic metabolism
PDK1
Inhibits TCA cycle metabolism
HK1
PFKL
GAPDH
ALDOA
ENO1
PGK1
PFKFB3
LDHAL6A
Promote anaerobic metabolism
BCL2
CDKN1A
Regulate proliferation and apoptosis
04066 8/5/21
(c) Kanehisa Laboratories

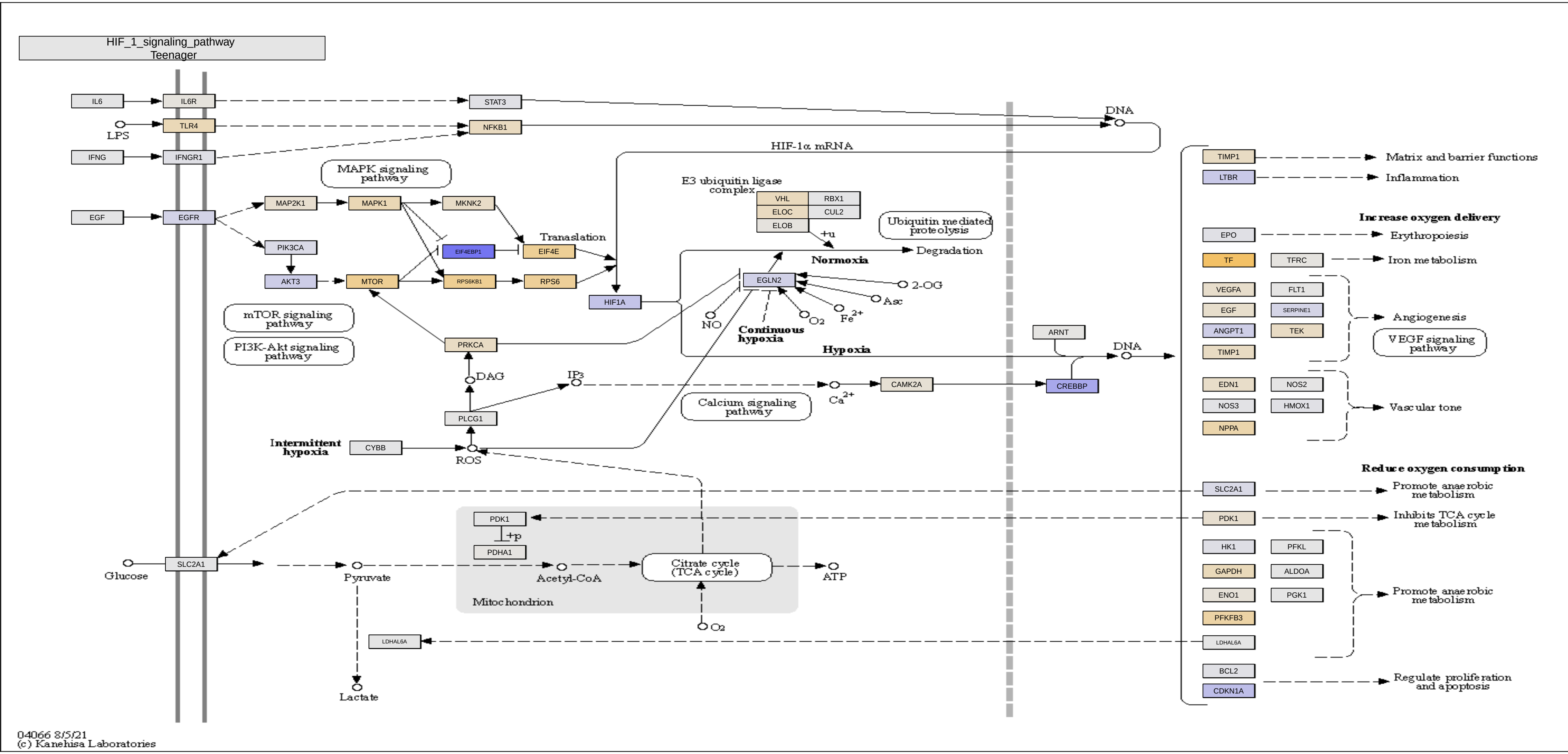
HIF_1_signaling_pathway
Teenager
IL6
IL6R
STAT3
LPS
TLR4
NFKB1
IFNG
IFNGR1
DNA
HIF-1α mRNA
MAPK signaling pathway
EGF
EGFR
MAP2K1
MAPK1
MKNK2
PIK3CA
AKT3
MTOR
EIF4EBP1
EIF4E
RPS6KB1
RPS6
Translation
E3 ubiquitin ligase complex
VHL
RBX1
ELOC
CUL2
ELOB
+u
Ubiquitin mediated proteolysis
Normoxia
Degradation
EGLN2
2-OG
Asc
O2
Fe2+
NO
Continuous hypoxia
Hypoxia
HIF1A
mTOR signaling pathway
PI3K-Akt signaling pathway
PRKCA
DAG
IP3
PLCG1
Ca2+
CAMK2A
Calcium signaling pathway
ARNT
CREBBP
Intermittent hypoxia
CYBB
ROS
PDK1
+p
PDHA1
Glucose
SLC2A1
Pyruvate
Acetyl-CoA
Citrate cycle (TCA cycle)
ATP
Mitochondrion
O2
LDHAL6A
Lactate
TIMP1
Matrix and barrier functions
LTBR
Inflammation
Increase oxygen delivery
EPO
Erythropoiesis
TF
TFRC
Iron metabolism
VEGFA
FLT1
EGF
SERPINE1
Angiogenesis
ANGPT1
TEK
VEGF signaling pathway
TIMP1
EDN1
NOS2
NOS3
HMOX1
Vascular tone
NPPA
Reduce oxygen consumption
SLC2A1
Promote anaerobic metabolism
PDK1
Inhibits TCA cycle metabolism
HK1
PFKL
GAPDH
ALDOA
ENO1
PGK1
Promote anaerobic metabolism
PFKFB3
LDHAL6A
BCL2
Regulate proliferation and apoptosis
CDKN1A
04066 8/5/21
(c) Kanehisa Laboratories

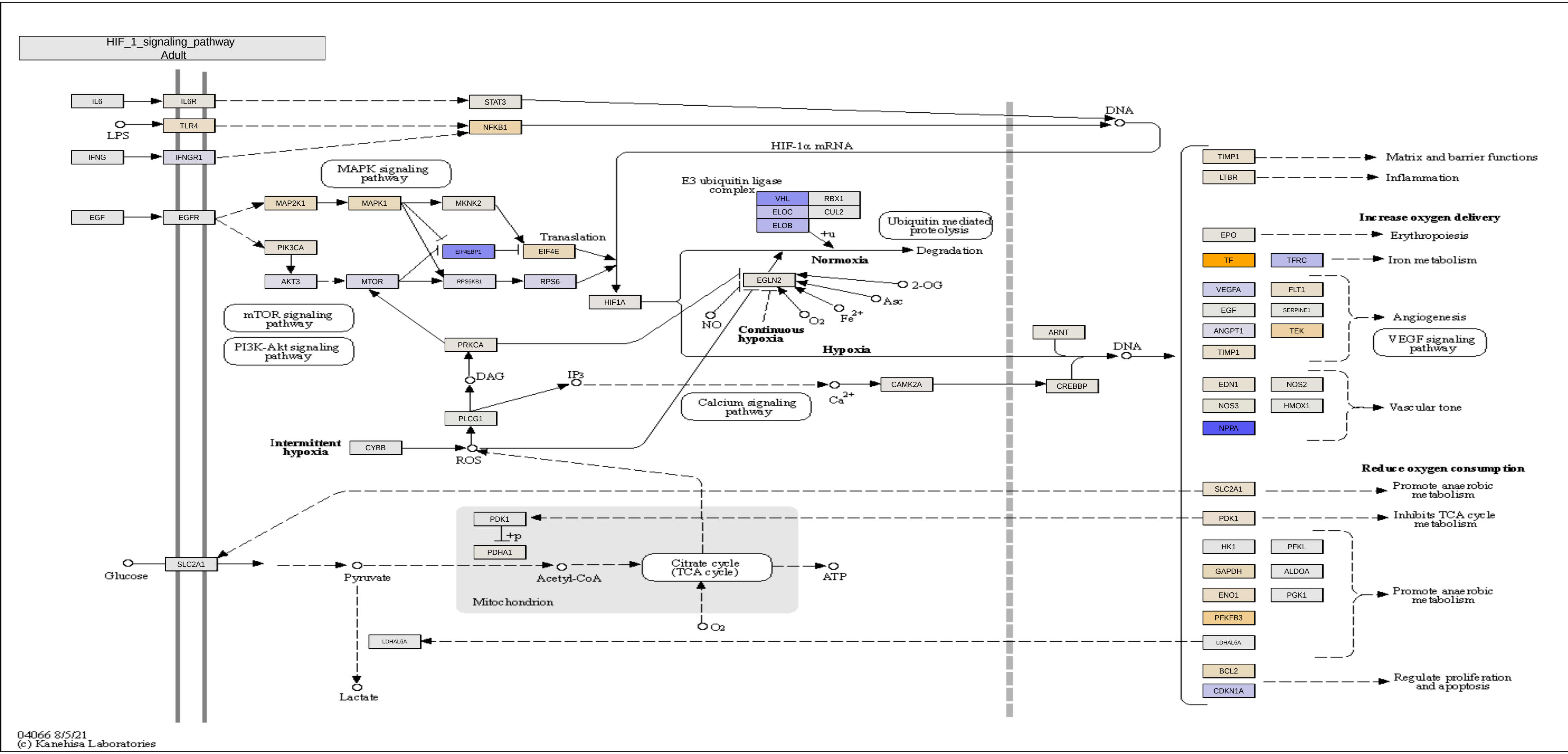
HIF_1_signaling_pathway
Adult
IL6
IL6R
STAT3
LPS
TLR4
NFKB1
IFNG
IFNGR1
DNA
HIF-1α mRNA
MAPK signaling pathway
EGF
EGFR
MAP2K1
MAPK1
MKNK2
E3 ubiquitin ligase complex
VHL
RBX1
ELOC
CUL2
ELOB
+u
Ubiquitin mediated proteolysis
Translation
PIK3CA
EIF4EBP1
EIF4E
Normoxia
Degradation
AKT3
MTOR
RPS6KB1
RPS6
EGLN2
2-OG
Asc
HIF1A
NO
O2
Fe2+
Continuous hypoxia
Hypoxia
mTOR signaling pathway
PI3K-Akt signaling pathway
PRKCA
ARNT
DNA
DAG
IP3
Calcium signaling pathway
Ca2+
CAMK2A
CREBBP
PLCG1
Intermittent hypoxia
CYBB
ROS
PDK1
+p
PDHA1
Glucose
SLC2A1
Pyruvate
Acetyl-CoA
Citrate cycle (TCA cycle)
ATP
Mitochondrion
O2
LDHAL6A
Lactate
TIMP1
Matrix and barrier functions
LTBR
Inflammation
Increase oxygen delivery
EPO
Erythropoiesis
TF
TFRC
Iron metabolism
VEGFA
FLT1
EGF
SERPINE1
Angiogenesis
ANGPT1
TEK
VEGF signaling pathway
TIMP1
EDN1
NOS2
NOS3
HMOX1
Vascular tone
NPPA
Reduce oxygen consumption
SLC2A1
Promote anaerobic metabolism
PDK1
Inhibits TCA cycle metabolism
HK1
PFKL
GAPDH
ALDOA
ENO1
PGK1
PFKFB3
Promote anaerobic metabolism
LDHAL6A
BCL2
Regulate proliferation and apoptosis
CDKN1A
04066 8/5/21
(c) Kanehisa Laboratories

HIF_1_signaling_pathway
Late Adult

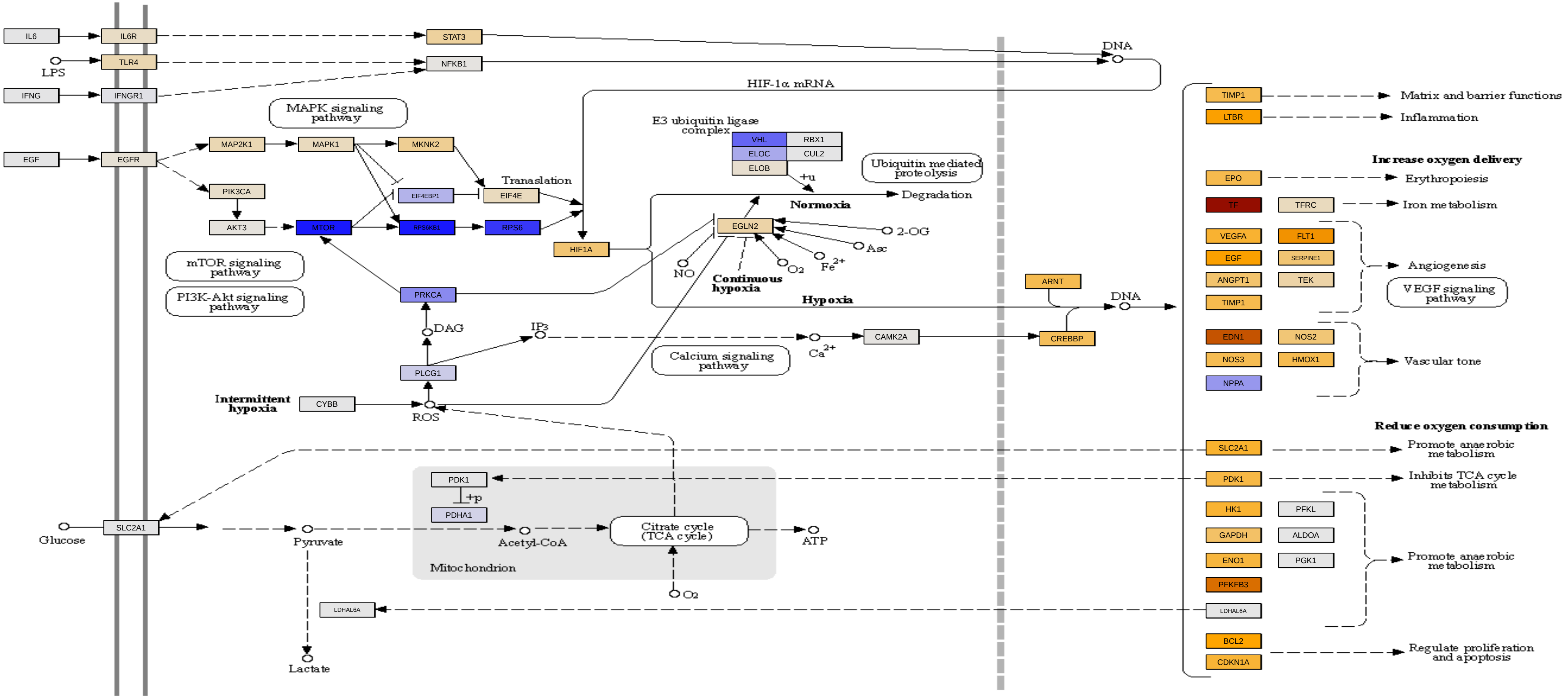

04066 8/5/21
(c) Kanehisa Laboratories
