# Supplementary material for: The Transcriptome and Methylome of the Developing and Aging Brain and Their Relations to Gliomas and Psychological Disorders
Source: Cells. 2022 Jan 21;11(3):362. doi: 10.3390/cells11030362 (PMC8834030; doi:10.3390/cells11030362)

Hippo signaling pathway

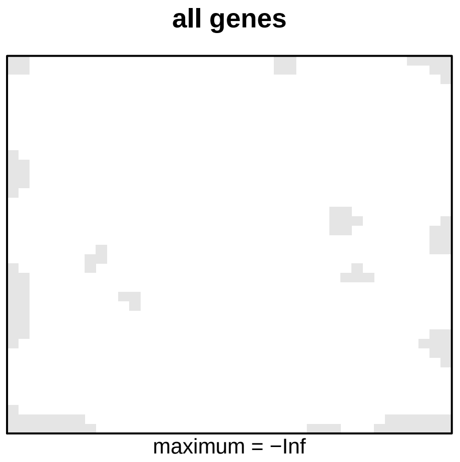

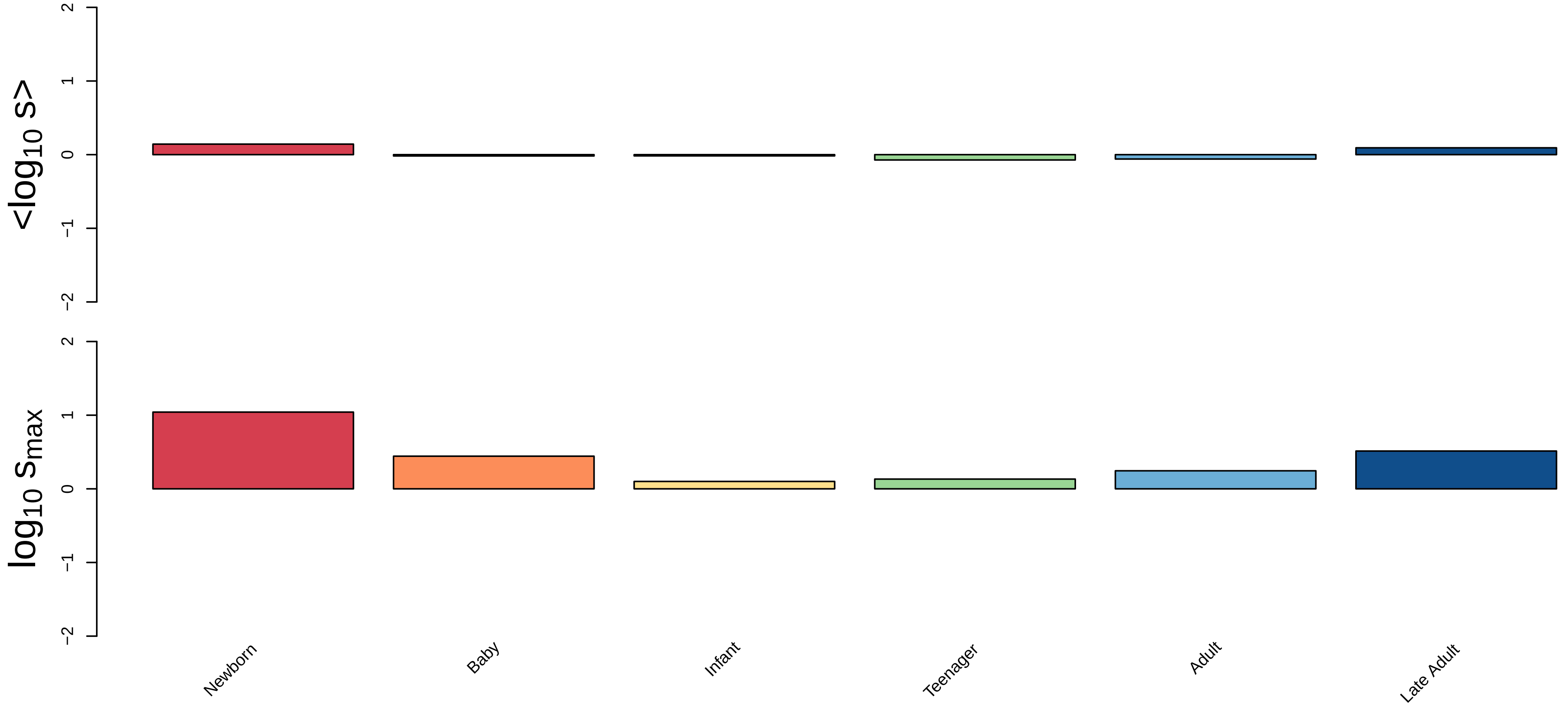

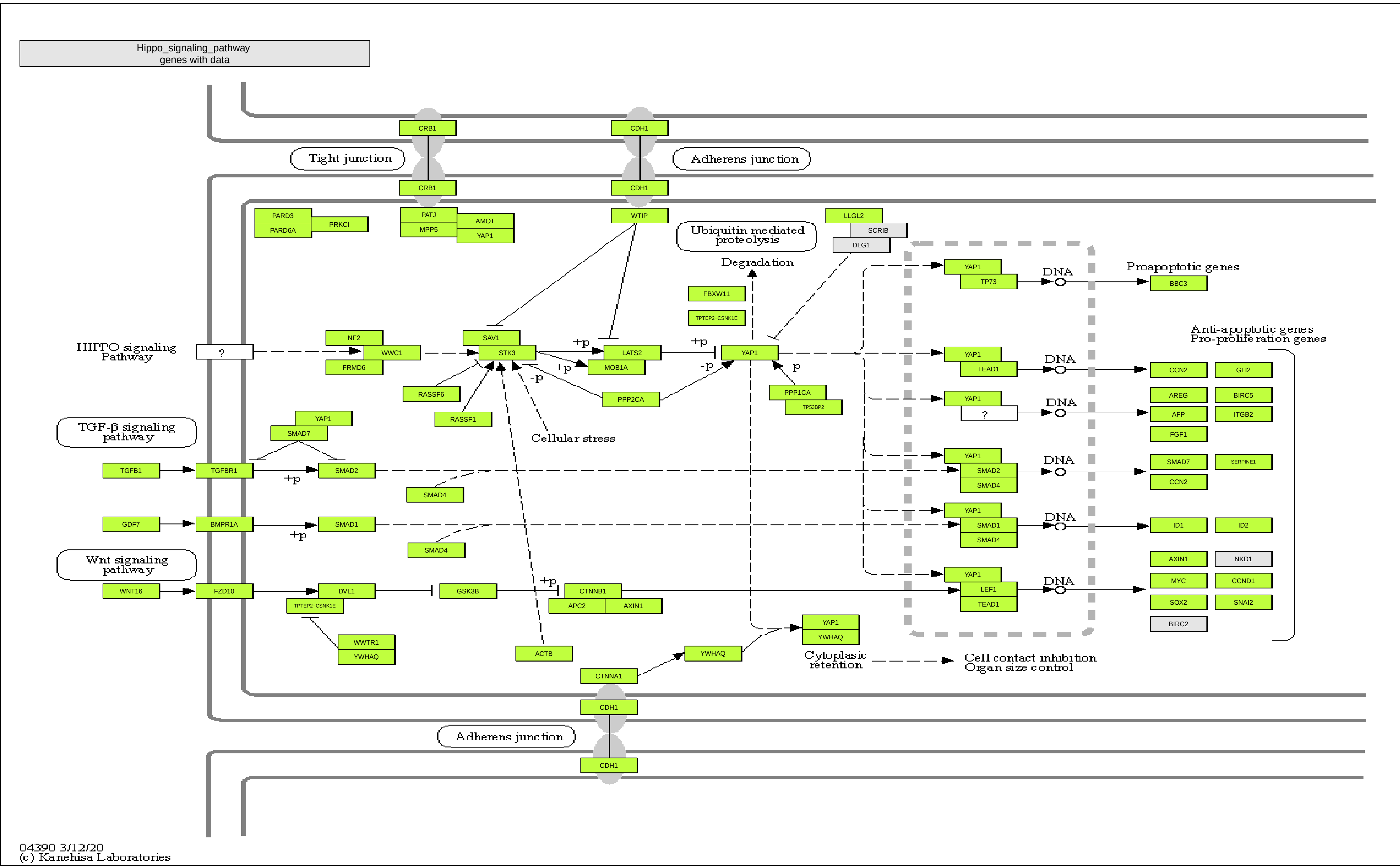
Hippo_signaling_pathway
genes with data
CRB1
CDH1
Tight junction
Adherens junction
CRB1
CDH1
PARD3
PRKCI
PARD6A
PATJ
AMOT
MPP5
YAP1
WTIP
Ubiquitin mediated proteolysis
LLGL2
SCRIB
DLG1
Degradation
FBXW11
TPTEP2-CSNK1E
HIPPO signaling Pathway
?
NF2
WWC1
FRMD6
SAV1
STK3
+p
LATS2
MOB1A
+p
YAP1
-p
-p
+p
-p
RASSF6
RASSF1
PPP2CA
PPP1CA
TP53BP2
Cellular stress
YAP1
TP73
DNA
Proapoptotic genes
BBC3
Anti-apoptotic genes
Pro-proliferation genes
YAP1
TEAD1
DNA
CCN2
GLI2
YAP1
?
DNA
AREG
BIRC5
AFP
ITGB2
FGF1
YAP1
SMAD2
SMAD4
DNA
SMAD7
SERPINE1
CCN2
YAP1
SMAD1
SMAD4
DNA
ID1
ID2
YAP1
LEF1
TEAD1
DNA
AXIN1
NKD1
MYC
CCND1
SOX2
SNAI2
BIRC2
TGF-β signaling pathway
YAP1
SMAD7
TGFB1
TGFBR1
+p
SMAD2
SMAD4
GDF7
BMPR1A
+p
SMAD1
SMAD4
Wnt signaling pathway
WNT16
FZD10
DVL1
TPTEP2-CSNK1E
GSK3B
+p
CTNNB1
APC2
AXIN1
WWTR1
YWHAQ
ACTB
YWHAQ
CTNNA1
YAP1
YWHAQ
Cytoplasic retention
Cell contact inhibition
Organ size control
CDH1
Adherens junction
CDH1
04390 3/12/20
(c) Kanehisa Laboratories

Hippo_signaling_pathway
sink nodes

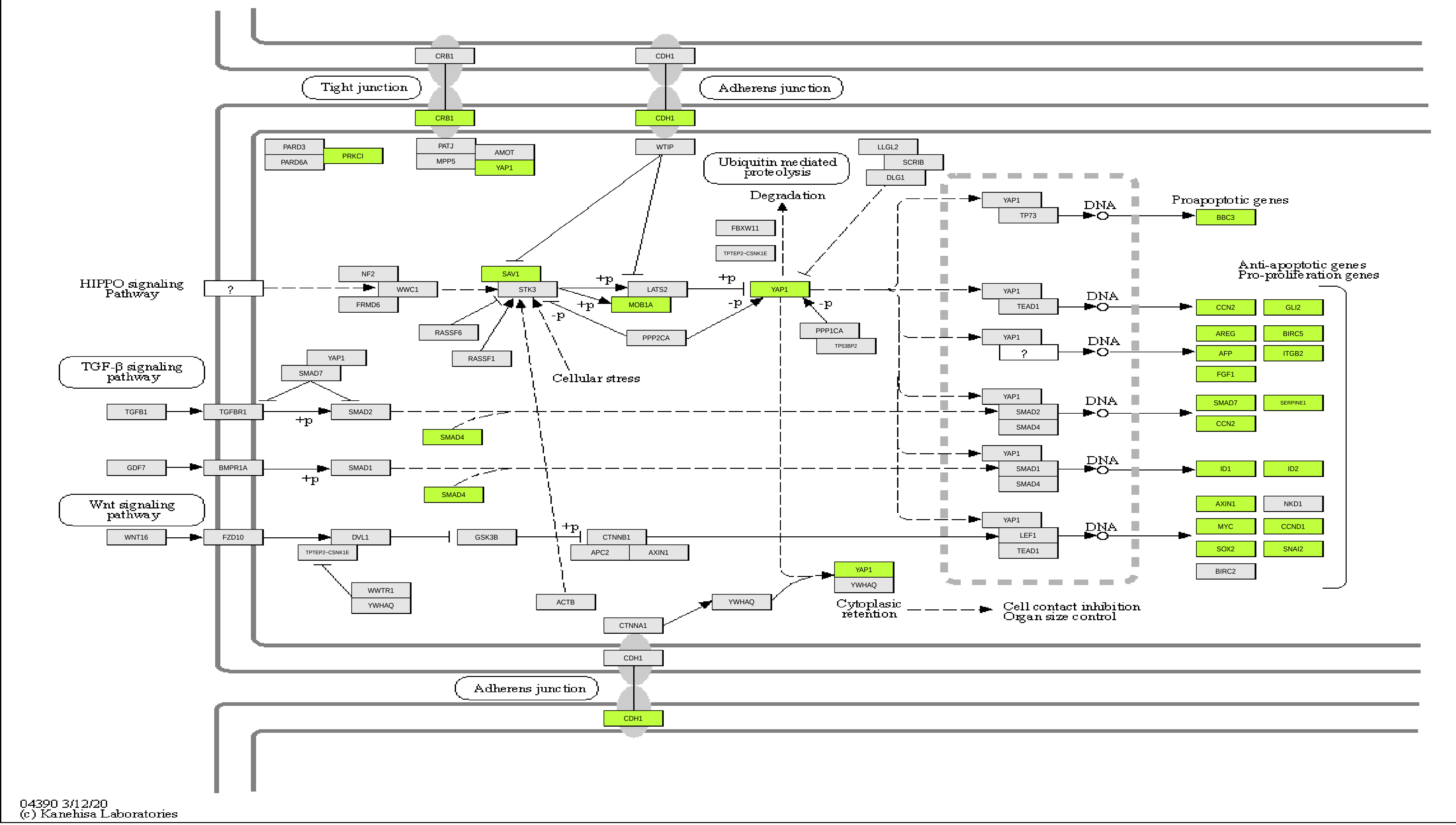

Hippo_signaling_pathway
Newborn

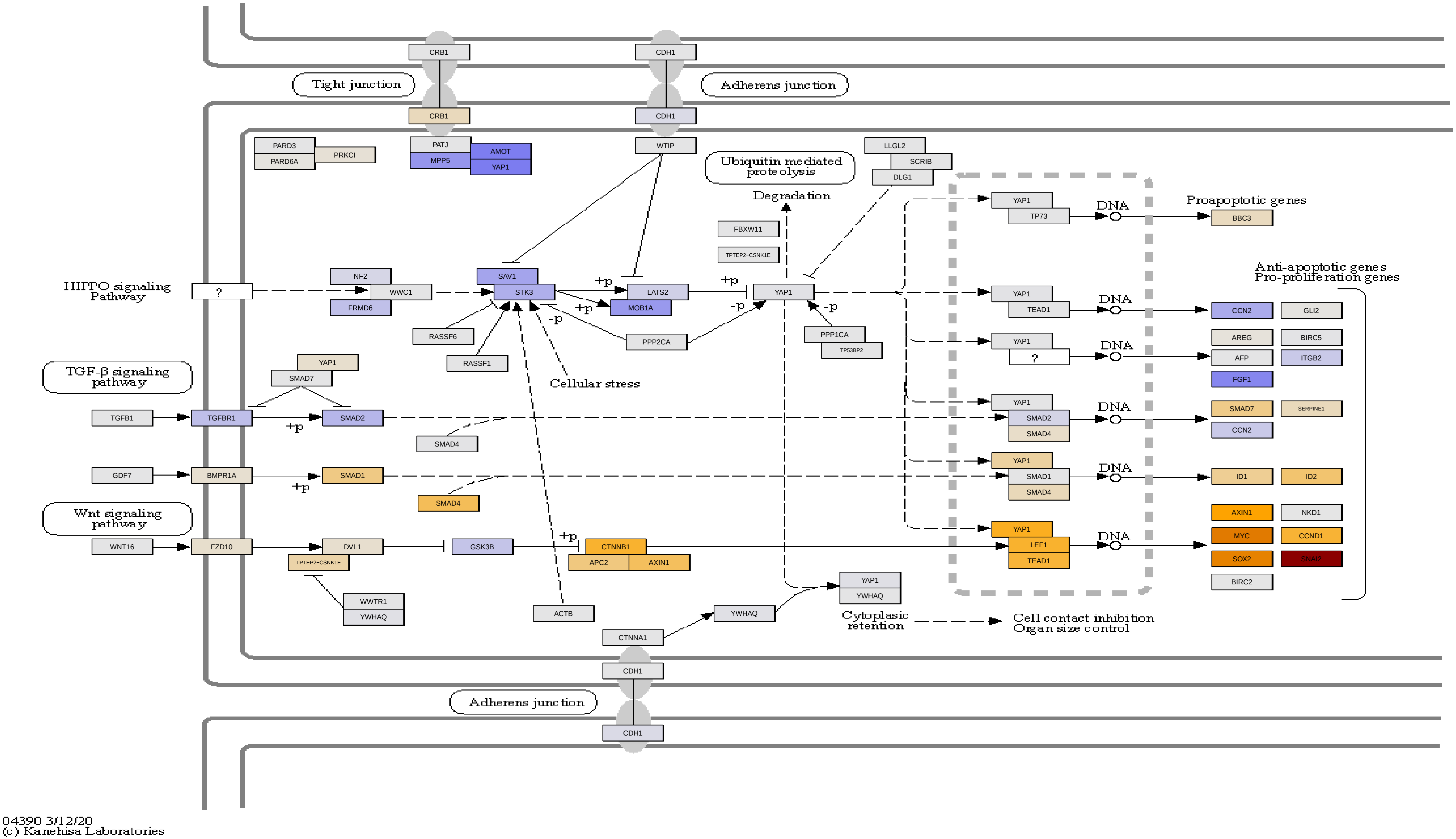

Hippo_signaling_pathway
Baby

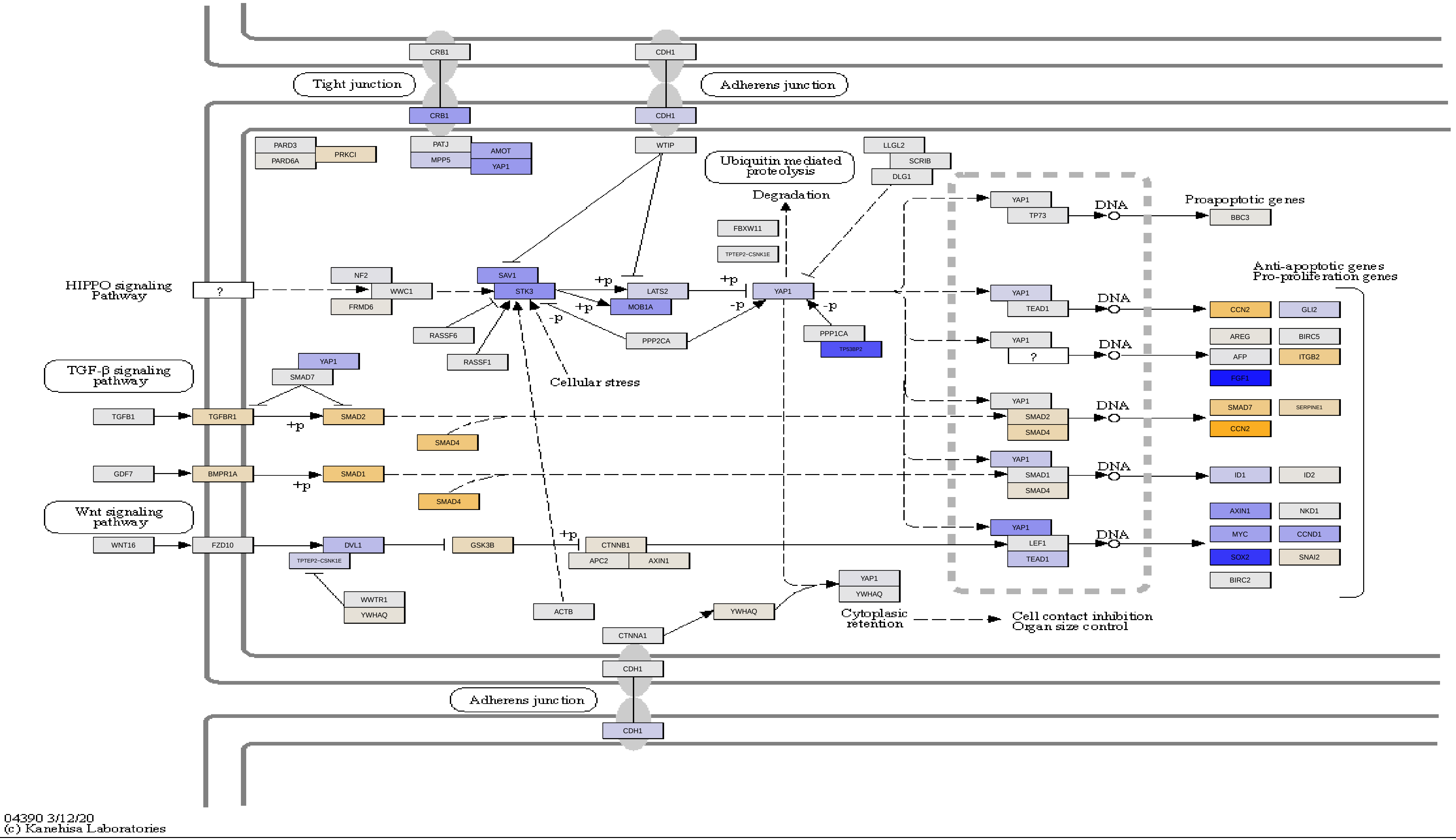

Hippo_signaling_pathway
Infant

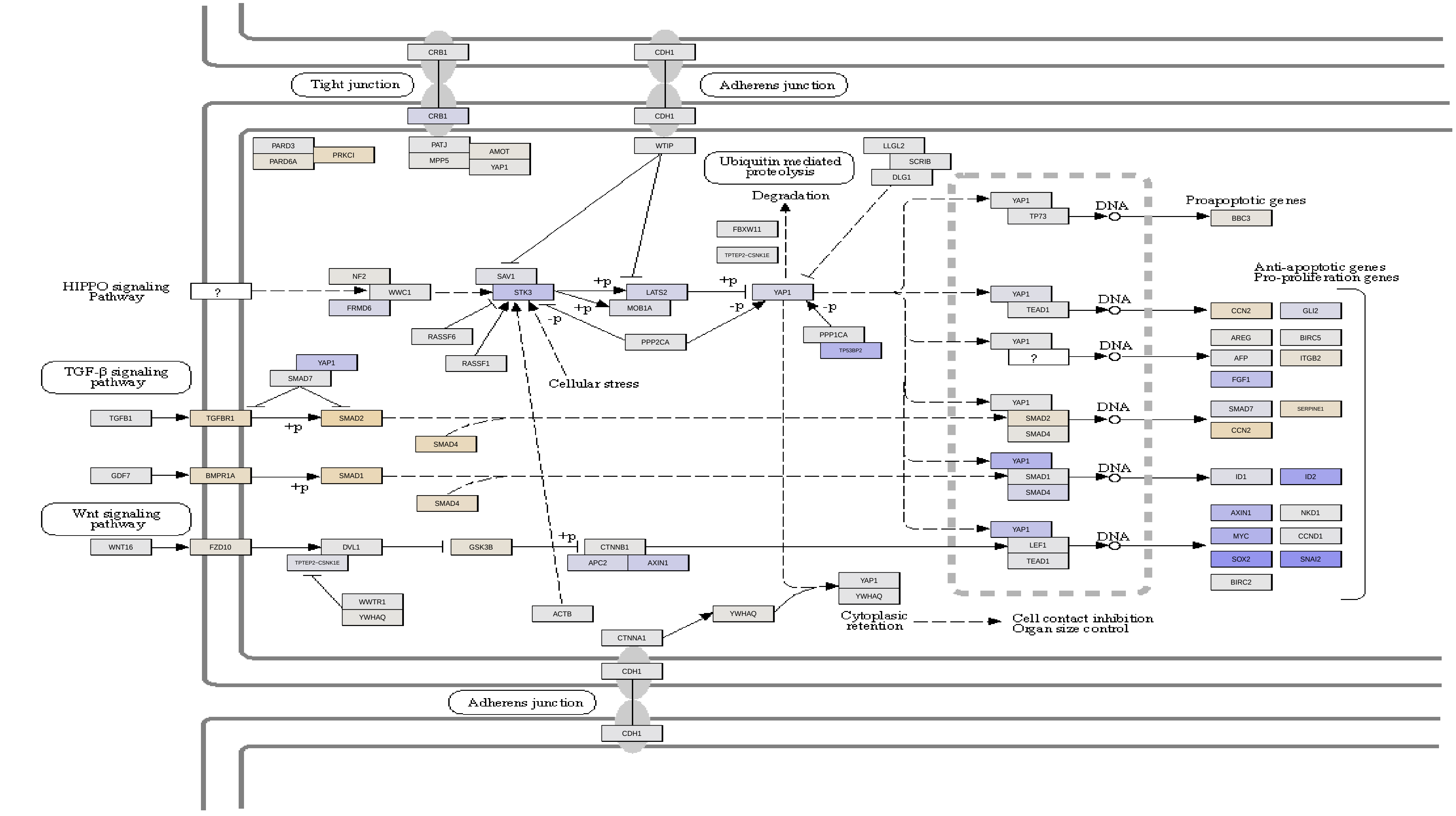

04390 3/12/20
(c) Kanehisa Laboratories

Hippo_signaling_pathway
Teenager

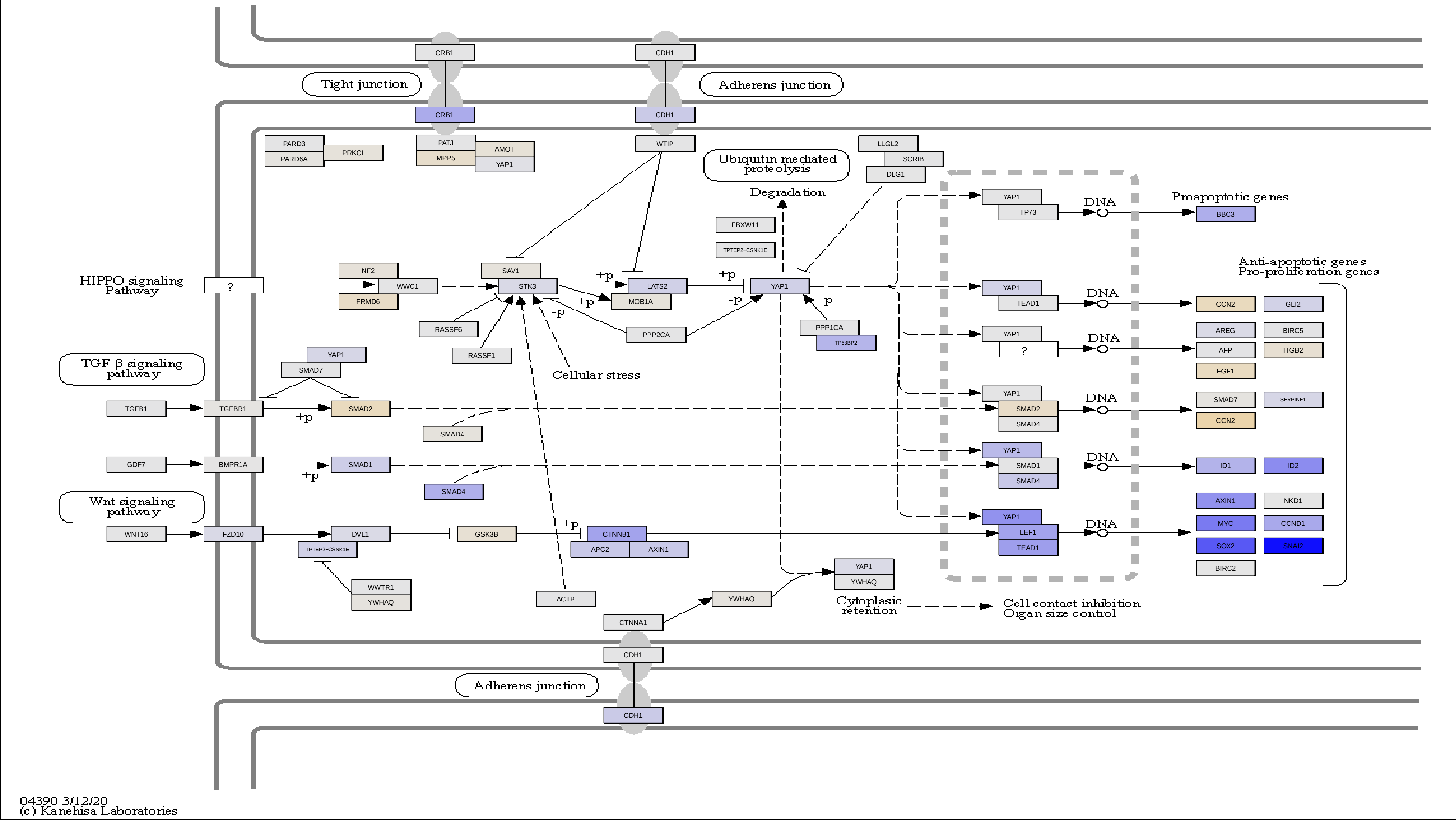

Hippo_signaling_pathway
Adult

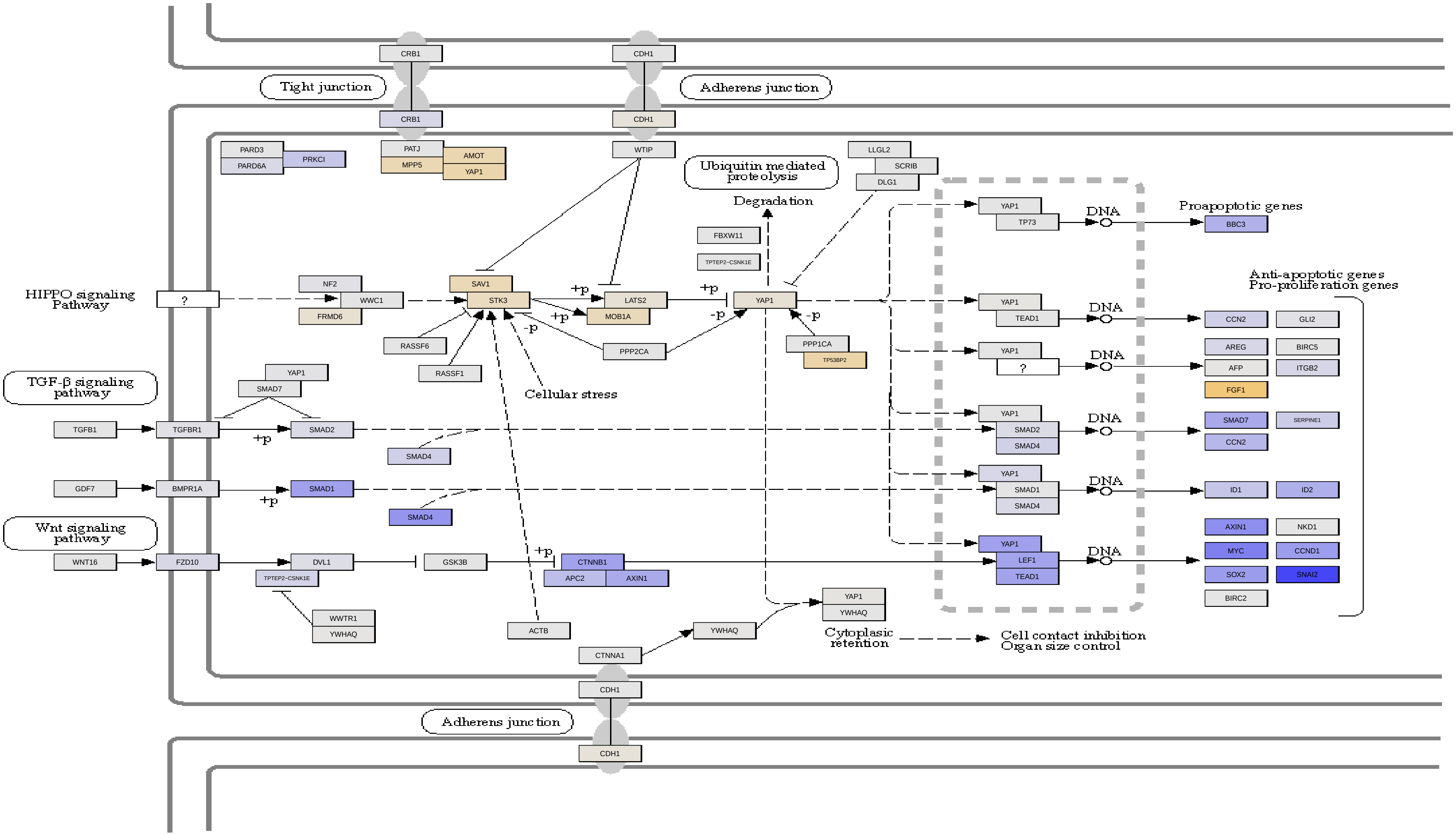

04390 3/12/20
(c) Kanehisa Laboratories

Hippo_signaling_pathway
Late Adult

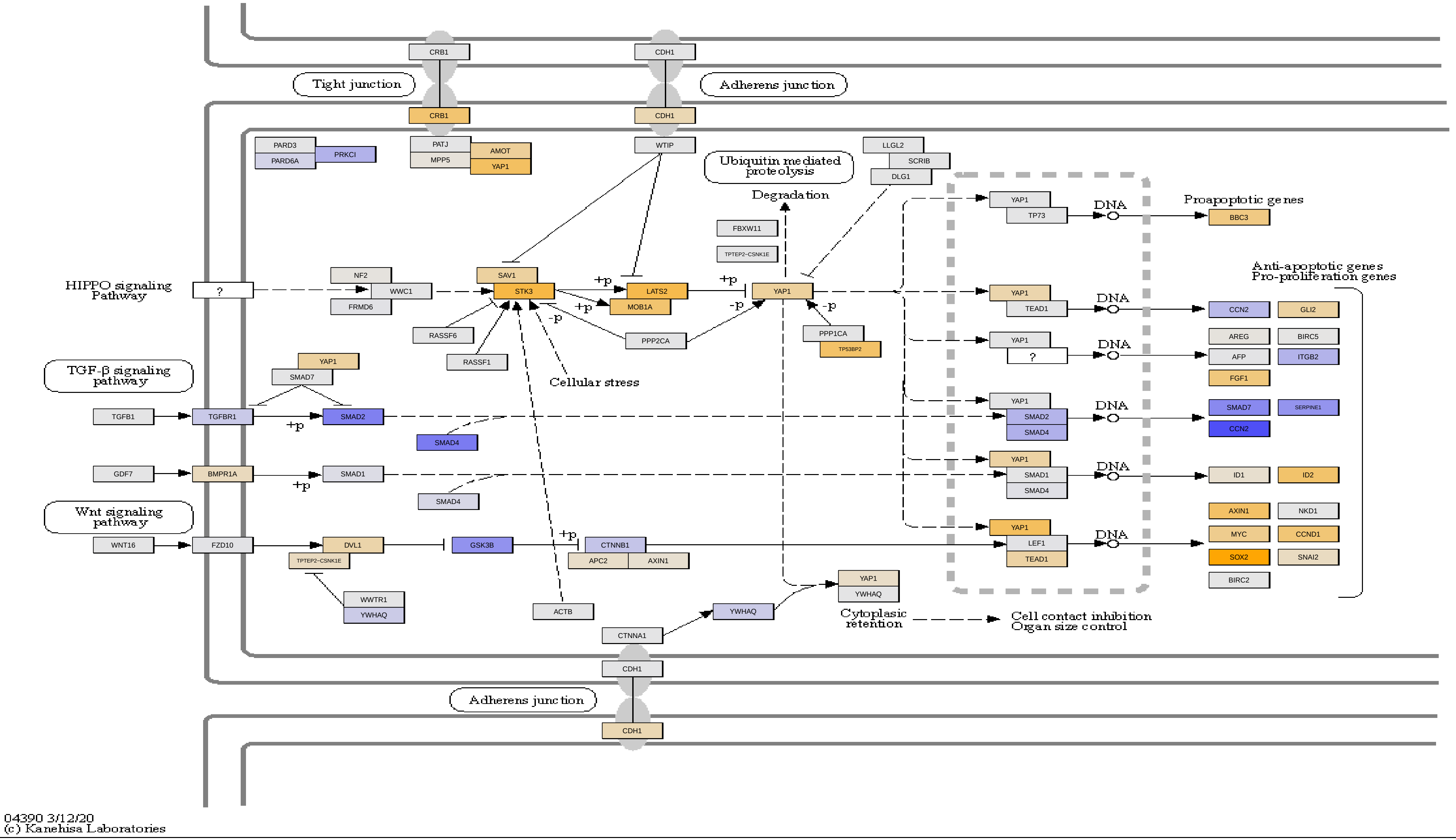

Supplement: Supplementary file 1 [file cells-11-00362-s001.zip › Suppl-Material-S3-Pathways-PSF_Expression/Hippo_signaling_pathway.pdf]
