# Supplementary material for: The Transcriptome and Methylome of the Developing and Aging Brain and Their Relations to Gliomas and Psychological Disorders
Source: Cells. 2022 Jan 21;11(3):362. doi: 10.3390/cells11030362 (PMC8834030; doi:10.3390/cells11030362)
Supplement: Supplementary file 1 [file cells-11-00362-s001.zip › Suppl-Material-S3-Pathways-PSF_Expression/Hippo_signaling_pathway_multiple_species.pdf]

Hippo signaling pathway − multiple s

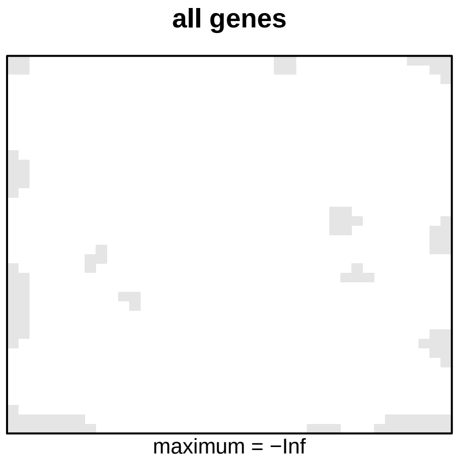

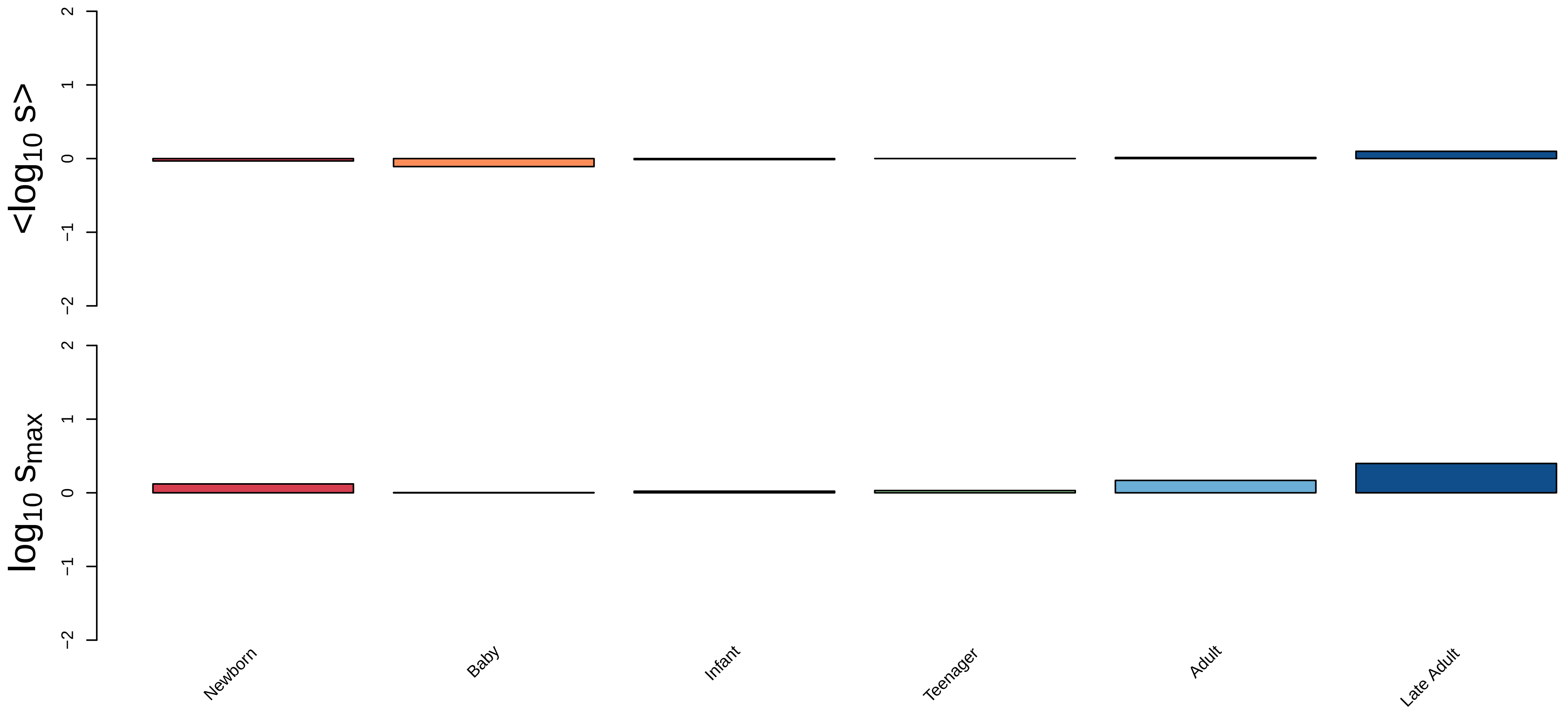

Hippo_signaling_pathway_multiple_species
genes with data

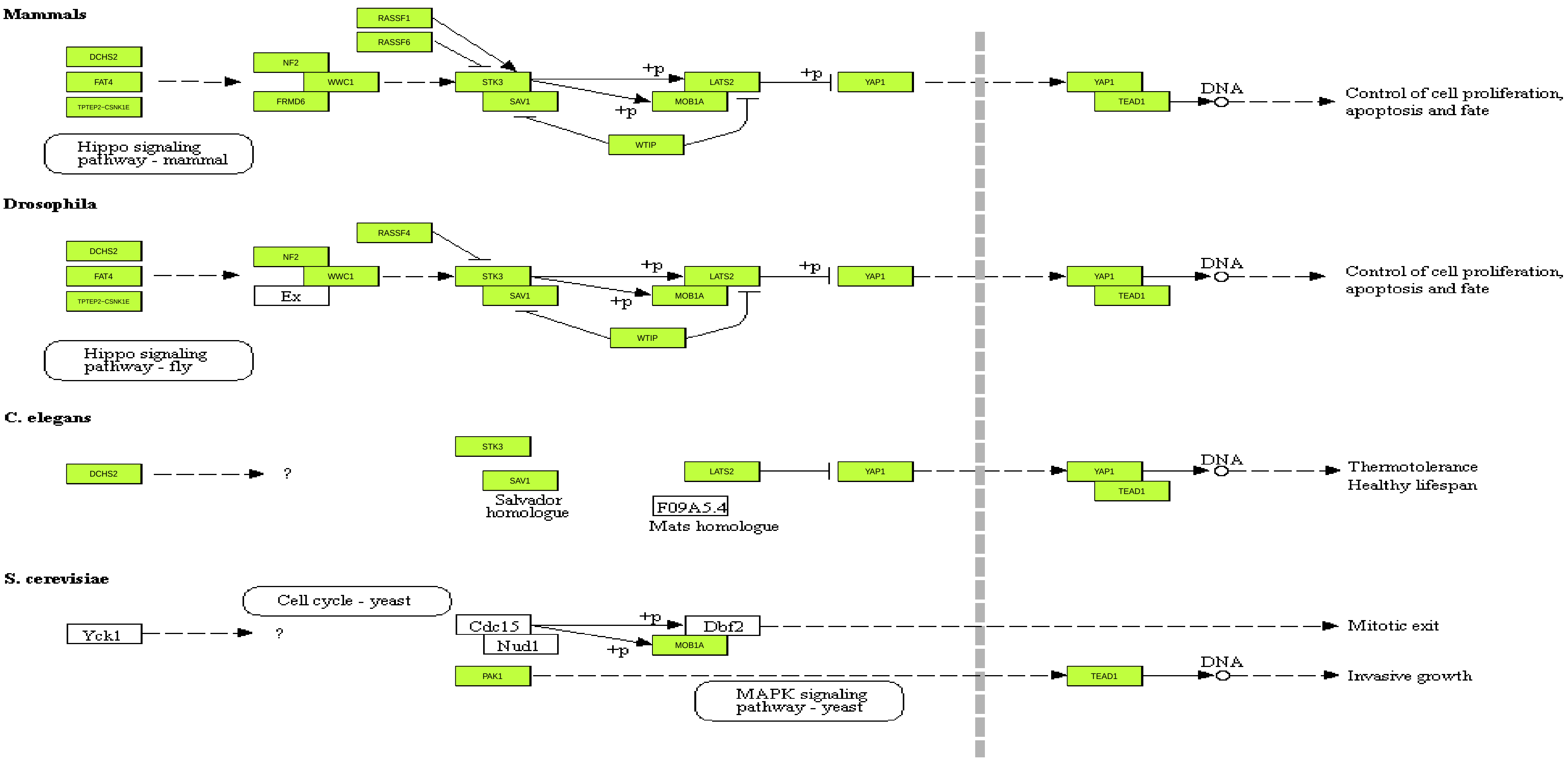

04392 5/27/16
(c) Kanehisa Laboratories

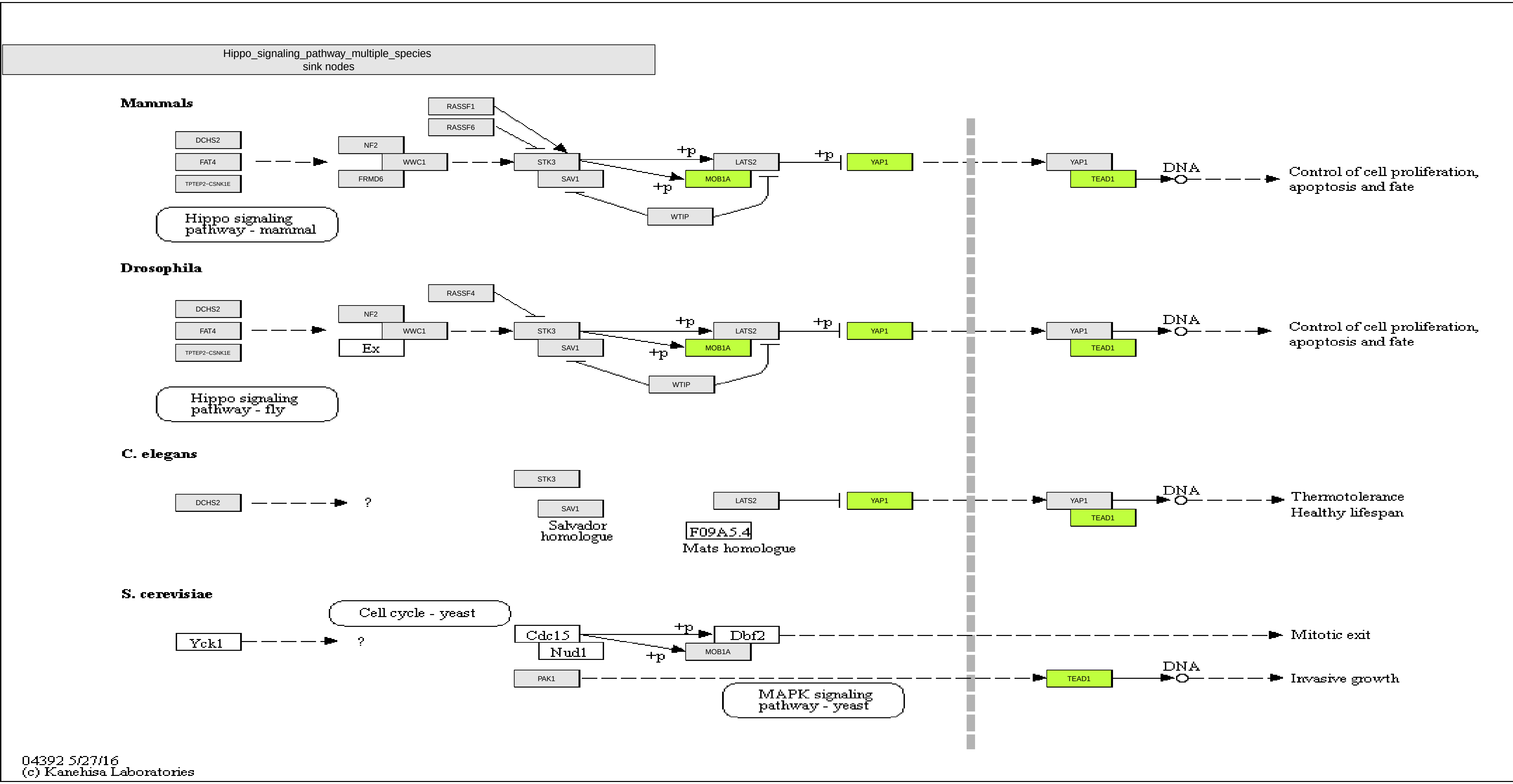
Hippo_signaling_pathway_multiple_species
sink nodes
Mammals
DCHS2
FAT4
TPTEP2-CSNK1E
NF2
WWC1
FRMD6
RASSF1
RASSF6
STK3
SAV1
+p
LATS2
MOB1A
+p
WTIP
+p
YAP1
YAP1
TEAD1
DNA
Control of cell proliferation, apoptosis and fate
Hippo signaling pathway - mammal
Drosophila
DCHS2
FAT4
TPTEP2-CSNK1E
NF2
WWC1
Ex
RASSF4
STK3
SAV1
+p
LATS2
MOB1A
+p
WTIP
+p
YAP1
YAP1
TEAD1
DNA
Control of cell proliferation, apoptosis and fate
Hippo signaling pathway - fly
C. elegans
DCHS2
?
STK3
SAV1
Salvador homologue
LATS2
F09A5.4
Mats homologue
YAP1
YAP1
TEAD1
DNA
Thermotolerance
Healthy lifespan
S. cerevisiae
Cell cycle - yeast
Yck1
?
Cdc15
Nud1
+p
Dbf2
MOB1A
+p
Mitotic exit
PAK1
TEAD1
DNA
Invasive growth
MAPK signaling pathway - yeast
04392 5/27/16
(c) Kanehisa Laboratories

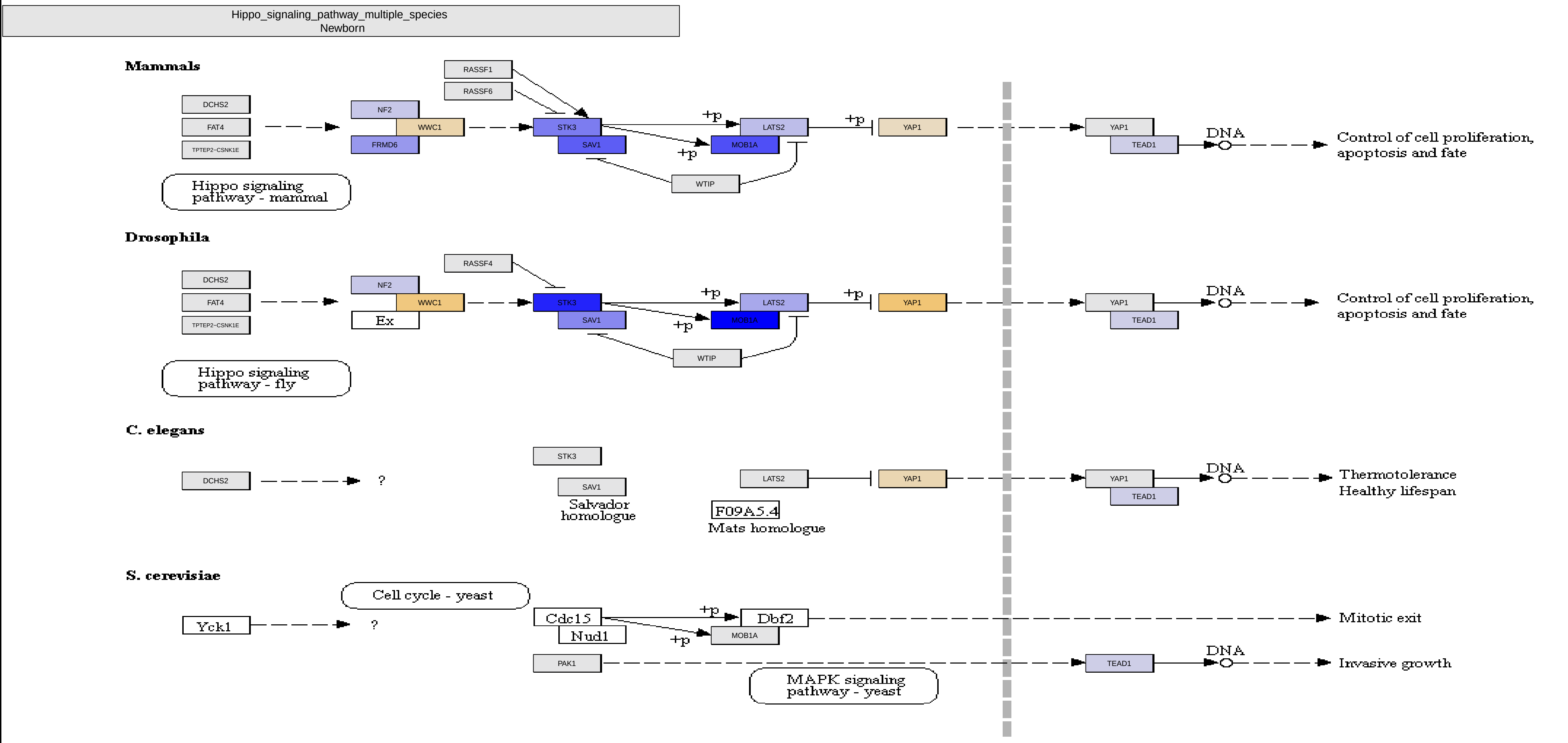

04392 5/27/16

(c) Kanehisa Laboratories

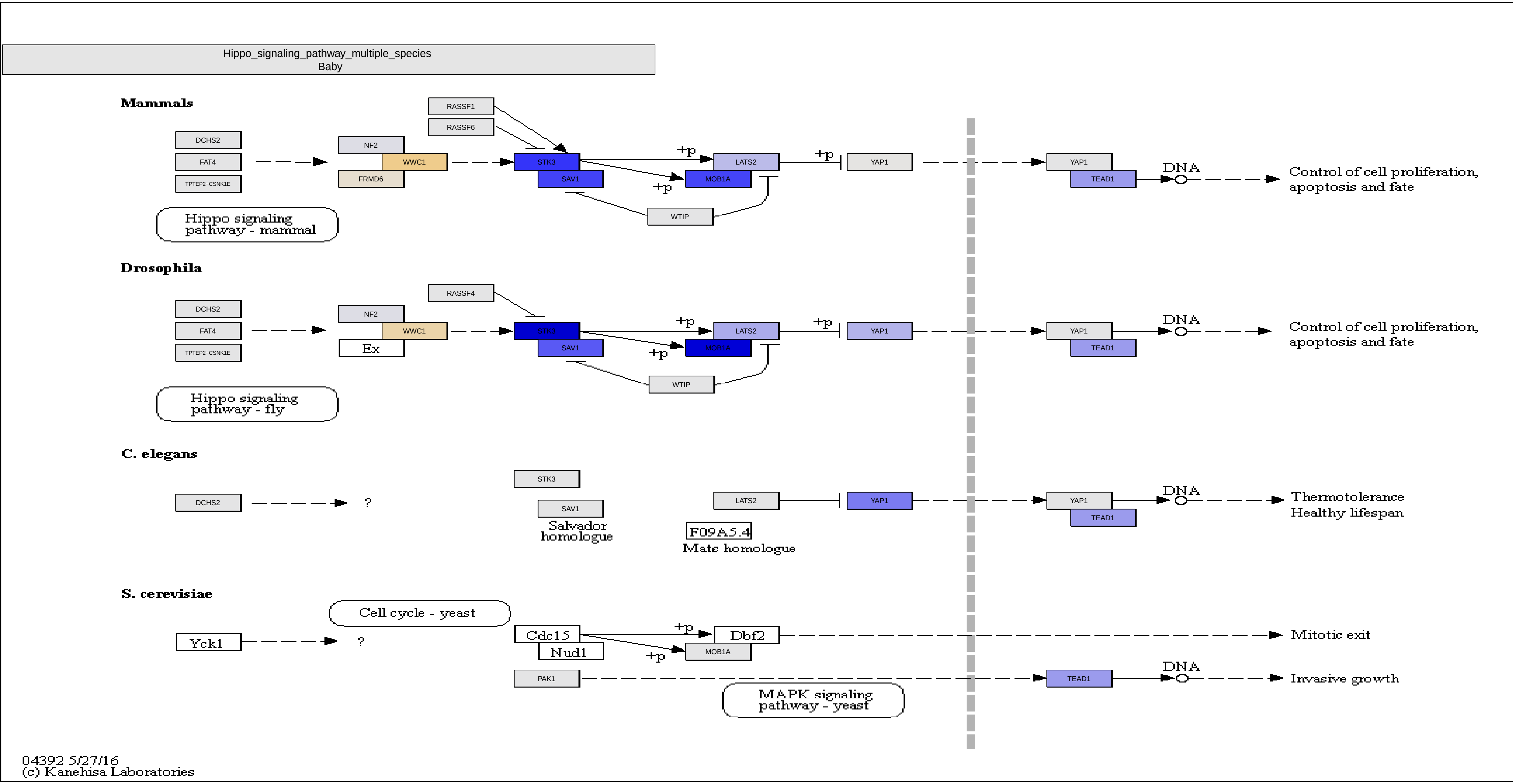
Hippo_signaling_pathway_multiple_species
Baby
Mammals
RASSF1
RASSF6
DCHS2
FAT4
TPTEP2-CSNK1E
NF2
WWC1
FRMD6
STK3
SAV1
+p
LATS2
MOB1A
+p
+p
YAP1
YAP1
TEAD1
DNA
Control of cell proliferation, apoptosis and fate
WTIP
Hippo signaling pathway - mammal
Drosophila
RASSF4
DCHS2
FAT4
TPTEP2-CSNK1E
NF2
WWC1
Ex
STK3
SAV1
+p
LATS2
MOB1A
+p
+p
YAP1
YAP1
TEAD1
DNA
Control of cell proliferation, apoptosis and fate
WTIP
Hippo signaling pathway - fly
C. elegans
STK3
DCHS2
?
SAV1
Salvador homologue
LATS2
YAP1
F09A5.4
Mats homologue
YAP1
TEAD1
DNA
Thermotolerance
Healthy lifespan
S. cerevisiae
Cell cycle - yeast
Yck1
?
Cdc15
Nud1
+p
Dbf2
MOB1A
+p
Mitotic exit
PAK1
TEAD1
DNA
Invasive growth
MAPK signaling pathway - yeast
04392 5/27/16
(c) Kanehisa Laboratories

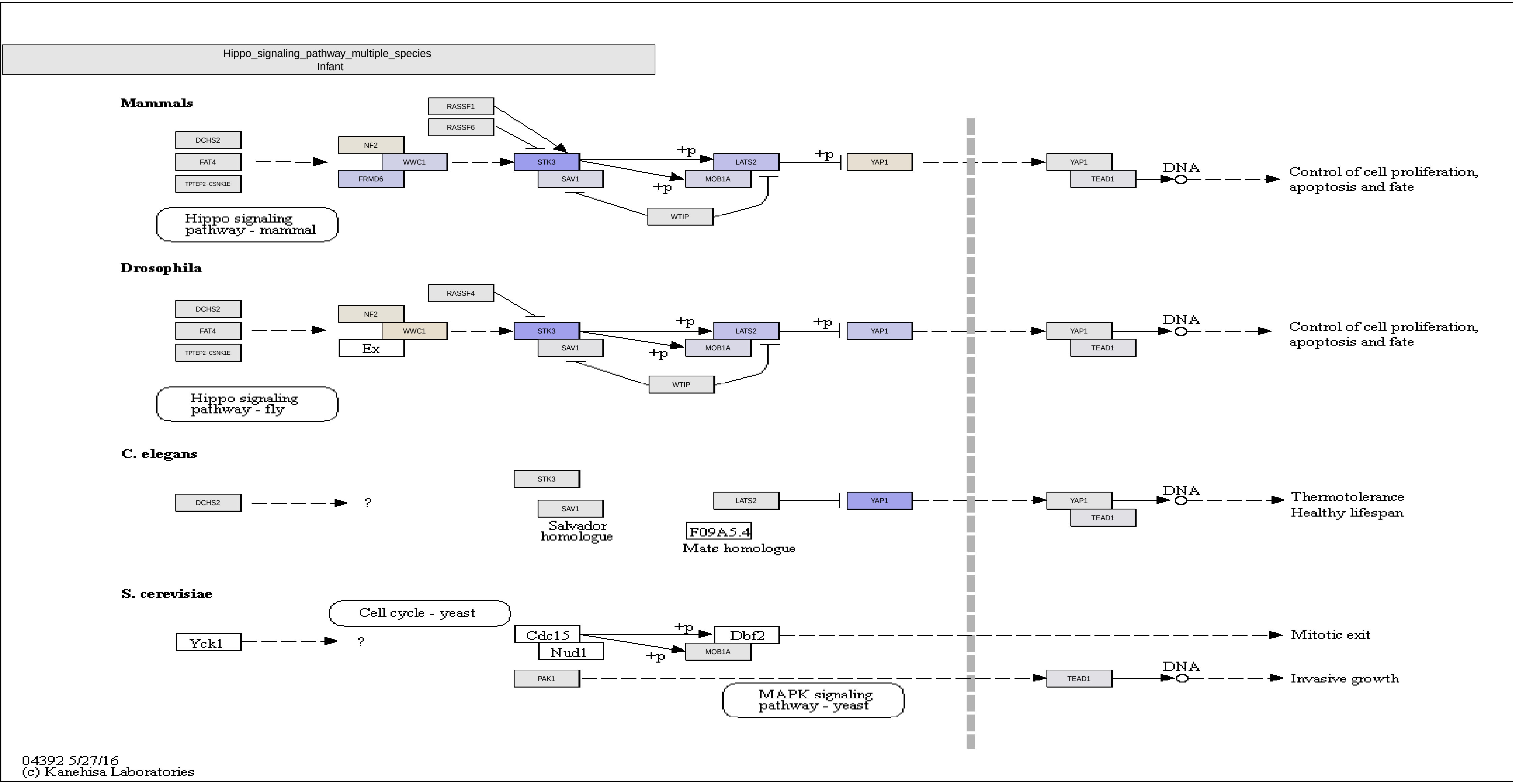

Hippo_signaling_pathway_multiple_species
Infant
Mammals
RASSF1
RASSF6
DCHS2
FAT4
TPTEP2-CSNK1E
NF2
WWC1
FRMD6
STK3
SAV1
+p
LATS2
MOB1A
+p
WTIP
+p
YAP1
YAP1
TEAD1
DNA
Control of cell proliferation, apoptosis and fate
Hippo signaling pathway - mammal
Drosophila
RASSF4
DCHS2
FAT4
TPTEP2-CSNK1E
NF2
WWC1
Ex
STK3
SAV1
+p
LATS2
MOB1A
+p
WTIP
+p
YAP1
YAP1
TEAD1
DNA
Control of cell proliferation, apoptosis and fate
Hippo signaling pathway - fly
C. elegans
DCHS2
?
STK3
SAV1
Salvador homologue
LATS2
F09A5.4
Mats homologue
YAP1
YAP1
TEAD1
DNA
Thermotolerance Healthy lifespan
S. cerevisiae
Cell cycle - yeast
Yck1
?
Cdc15
Nud1
+p
Dbf2
MOB1A
+p
Mitotic exit
PAK1
TEAD1
DNA
Invasive growth
MAPK signaling pathway - yeast
04392 5/27/16
(c) Kanehisa Laboratories

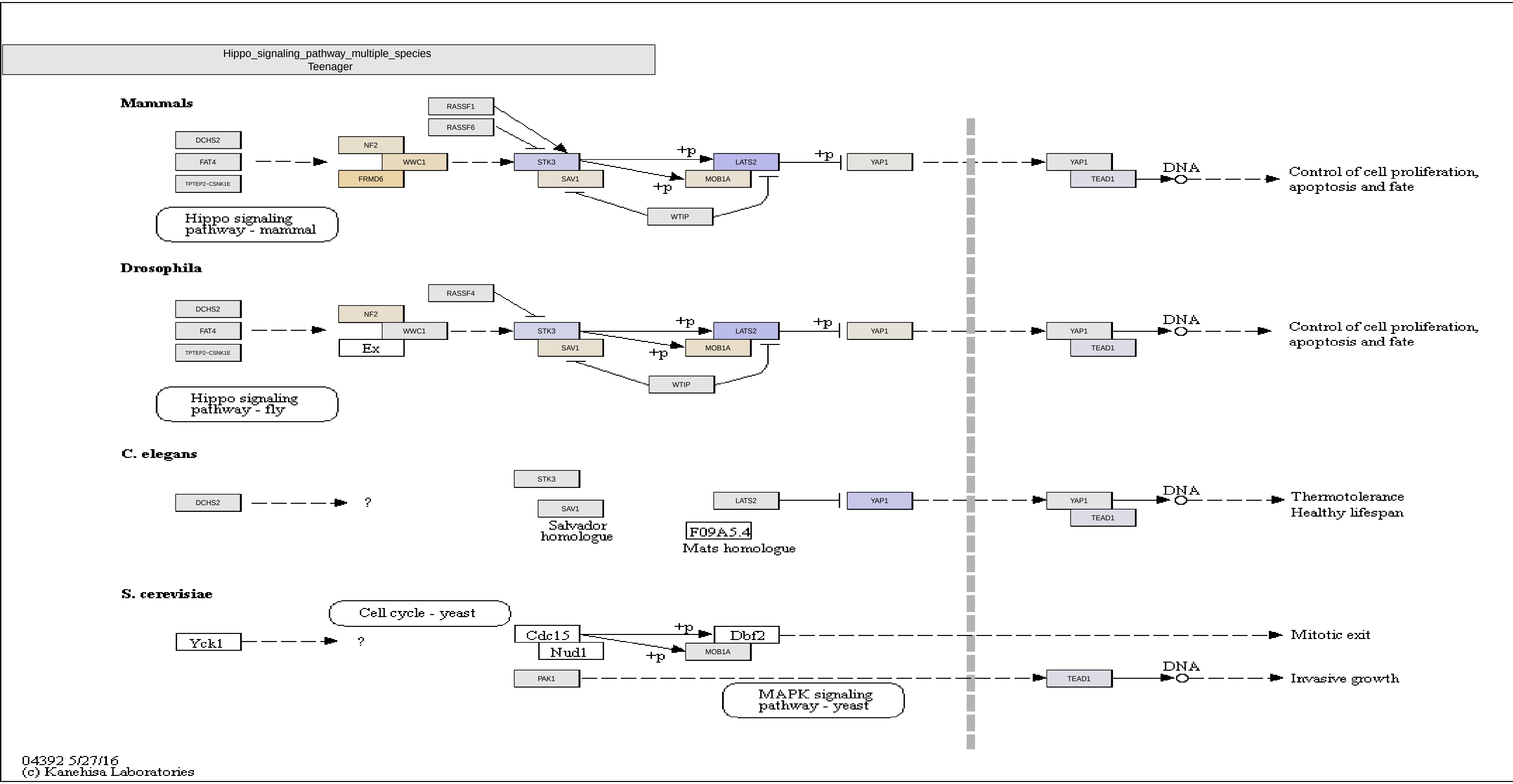

Hippo_signaling_pathway_multiple_species
Teenager
Mammals
RASSF1
RASSF6
DCHS2
FAT4
TPTEP2-CSNK1E
NF2
WWC1
FRMD6
STK3
SAV1
+p
LATS2
MOB1A
+p
+p
YAP1
YAP1
TEAD1
DNA
Control of cell proliferation, apoptosis and fate
WTIP
Hippo signaling pathway - mammal
Drosophila
RASSF4
DCHS2
FAT4
TPTEP2-CSNK1E
NF2
WWC1
Ex
STK3
SAV1
+p
LATS2
MOB1A
+p
+p
YAP1
YAP1
TEAD1
DNA
Control of cell proliferation, apoptosis and fate
WTIP
Hippo signaling pathway - fly
C. elegans
STK3
DCHS2
?
SAV1
Salvador homologue
LATS2
F09A5.4
Mats homologue
YAP1
YAP1
TEAD1
DNA
Thermotolerance
Healthy lifespan
S. cerevisiae
Cell cycle - yeast
Yck1
?
Cdc15
Nud1
+p
Dbf2
MOB1A
+p
Mitotic exit
PAK1
TEAD1
DNA
Invasive growth
MAPK signaling pathway - yeast
04392 5/27/16
(c) Kanehisa Laboratories

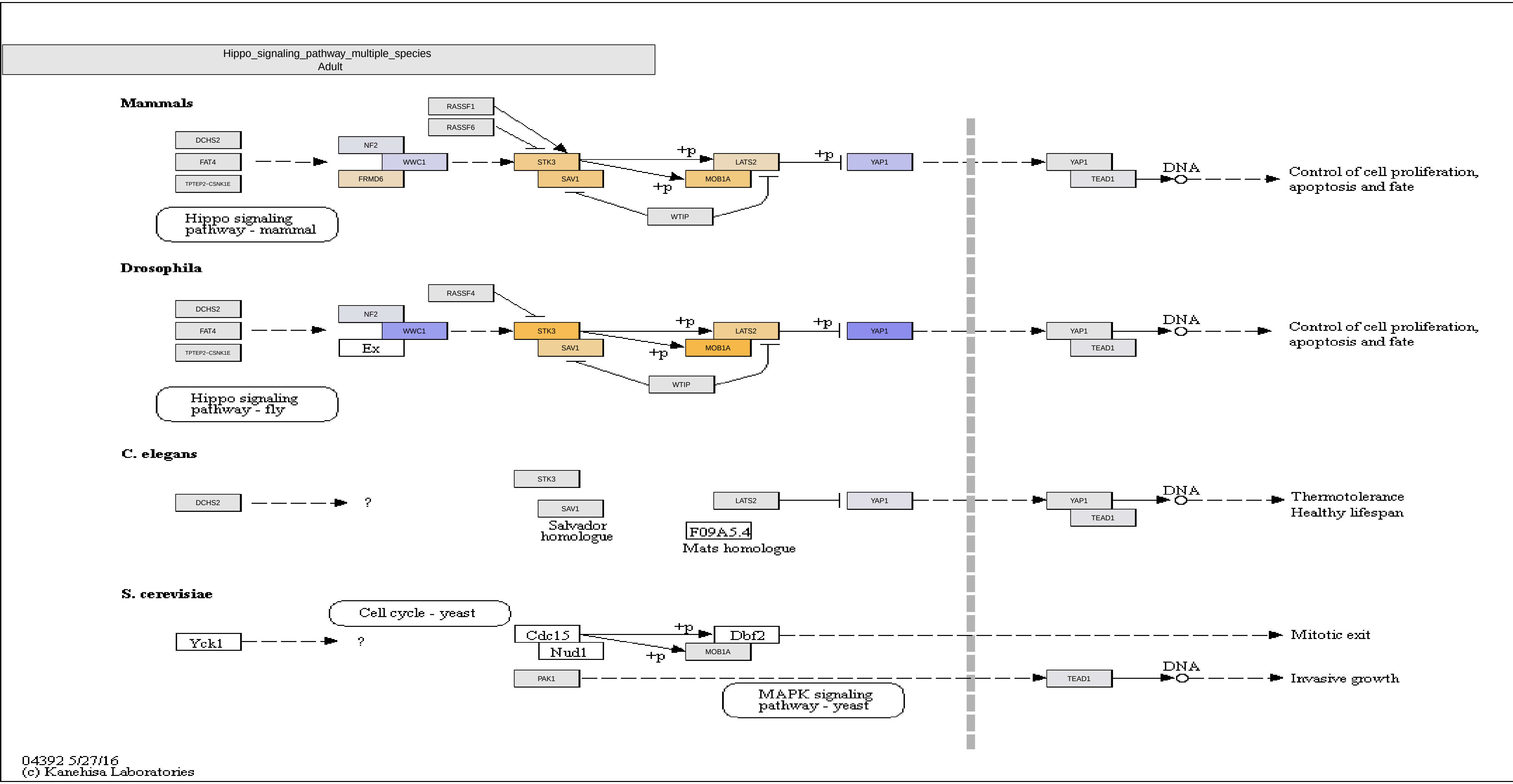

Hippo_signaling_pathway_multiple_species
Adult
Mammals
DCHS2
FAT4
TPTEP2-CSNK1E
NF2
WWC1
FRMD6
RASSF1
RASSF6
STK3
SAV1
+p
LATS2
MOB1A
+p
WTIP
+p
YAP1
YAP1
TEAD1
DNA
Control of cell proliferation, apoptosis and fate
Hippo signaling pathway - mammal
Drosophila
DCHS2
FAT4
TPTEP2-CSNK1E
NF2
WWC1
Ex
RASSF4
STK3
SAV1
+p
LATS2
MOB1A
+p
WTIP
+p
YAP1
YAP1
TEAD1
DNA
Control of cell proliferation, apoptosis and fate
Hippo signaling pathway - fly
C. elegans
DCHS2
?
STK3
SAV1
Salvador homologue
LATS2
YAP1
F09A5.4
Mats homologue
YAP1
TEAD1
DNA
Thermotolerance
Healthy lifespan
S. cerevisiae
Yck1
?
Cell cycle - yeast
Cdc15
Nud1
+p
Dbf2
MOB1A
+p
Mitotic exit
PAK1
MAPK signaling pathway - yeast
TEAD1
DNA
Invasive growth
04392 5/27/16
(c) Kanehisa Laboratories

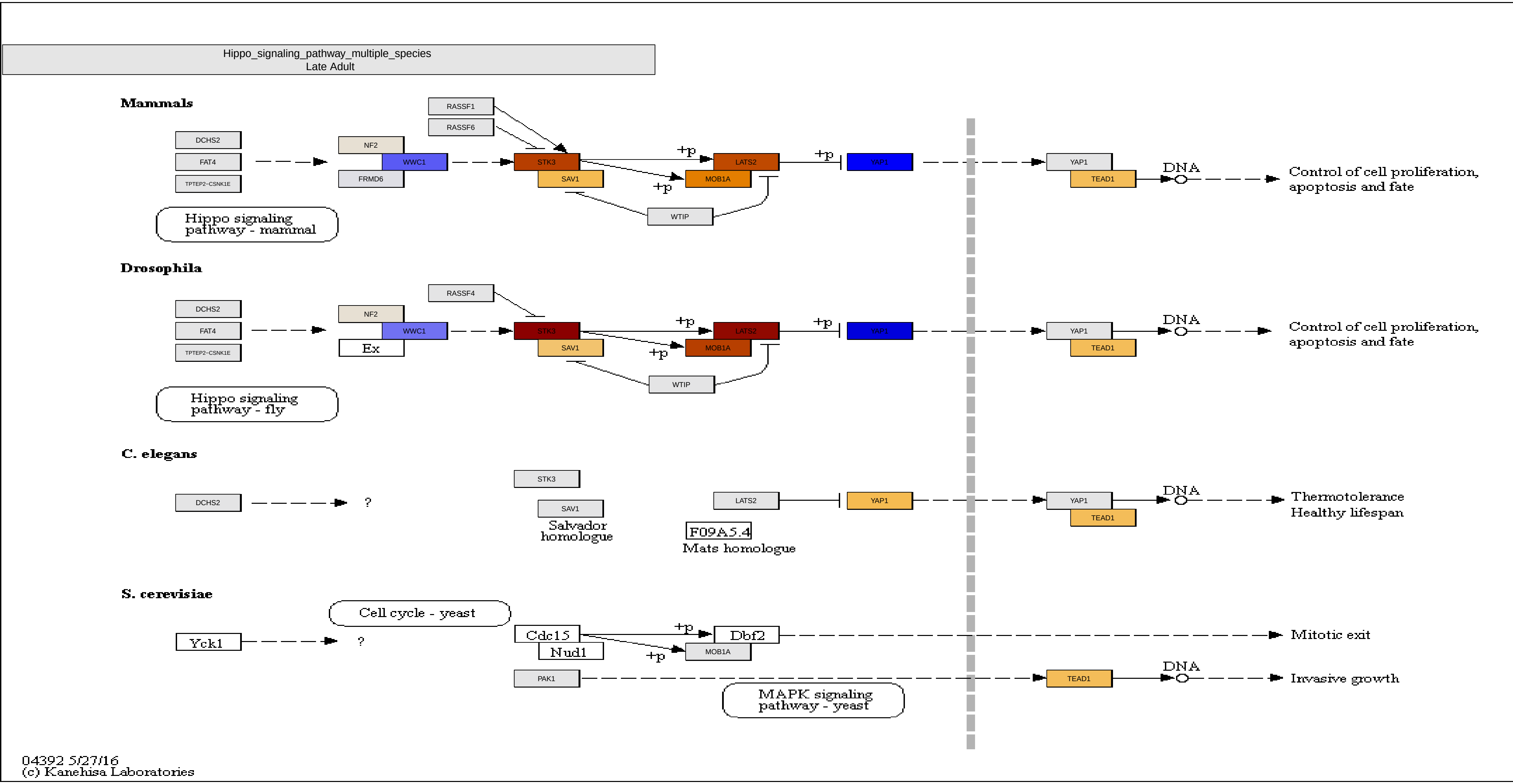
Hippo_signaling_pathway_multiple_species
Late Adult
Mammals
DCHS2
FAT4
TPTEP2-CSNK1E
NF2
WWC1
FRMD6
RASSF1
RASSF6
STK3
SAV1
+p
LATS2
MOB1A
+p
WTIP
+p
YAP1
YAP1
TEAD1
DNA
Control of cell proliferation, apoptosis and fate
Hippo signaling pathway - mammal
Drosophila
DCHS2
FAT4
TPTEP2-CSNK1E
NF2
WWC1
Ex
RASSF4
STK3
SAV1
LATS2
MOB1A
WTIP
YAP1
YAP1
TEAD1
DNA
Control of cell proliferation, apoptosis and fate
Hippo signaling pathway - fly
C. elegans
DCHS2
?
STK3
SAV1
Salvador homologue
LATS2
F09A5.4
Mats homologue
YAP1
YAP1
TEAD1
DNA
Thermotolerance
Healthy lifespan
S. cerevisiae
Cell cycle - yeast
Yck1
?
Cdc15
Nud1
Dbf2
MOB1A
Mitotic exit
PAK1
TEAD1
DNA
Invasive growth
MAPK signaling pathway - yeast
04392 5/27/16
(c) Kanehisa Laboratories
