# Supplementary material for: The Transcriptome and Methylome of the Developing and Aging Brain and Their Relations to Gliomas and Psychological Disorders
Source: Cells. 2022 Jan 21;11(3):362. doi: 10.3390/cells11030362 (PMC8834030; doi:10.3390/cells11030362)

IL−17 signaling pathway

all genes

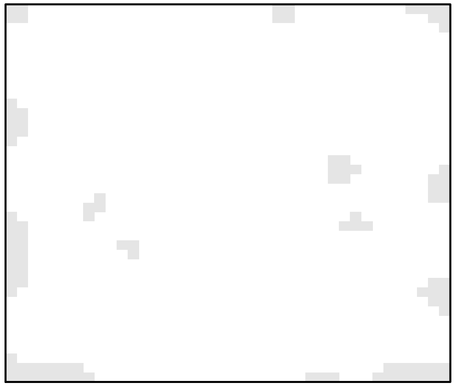

maximum = −Inf

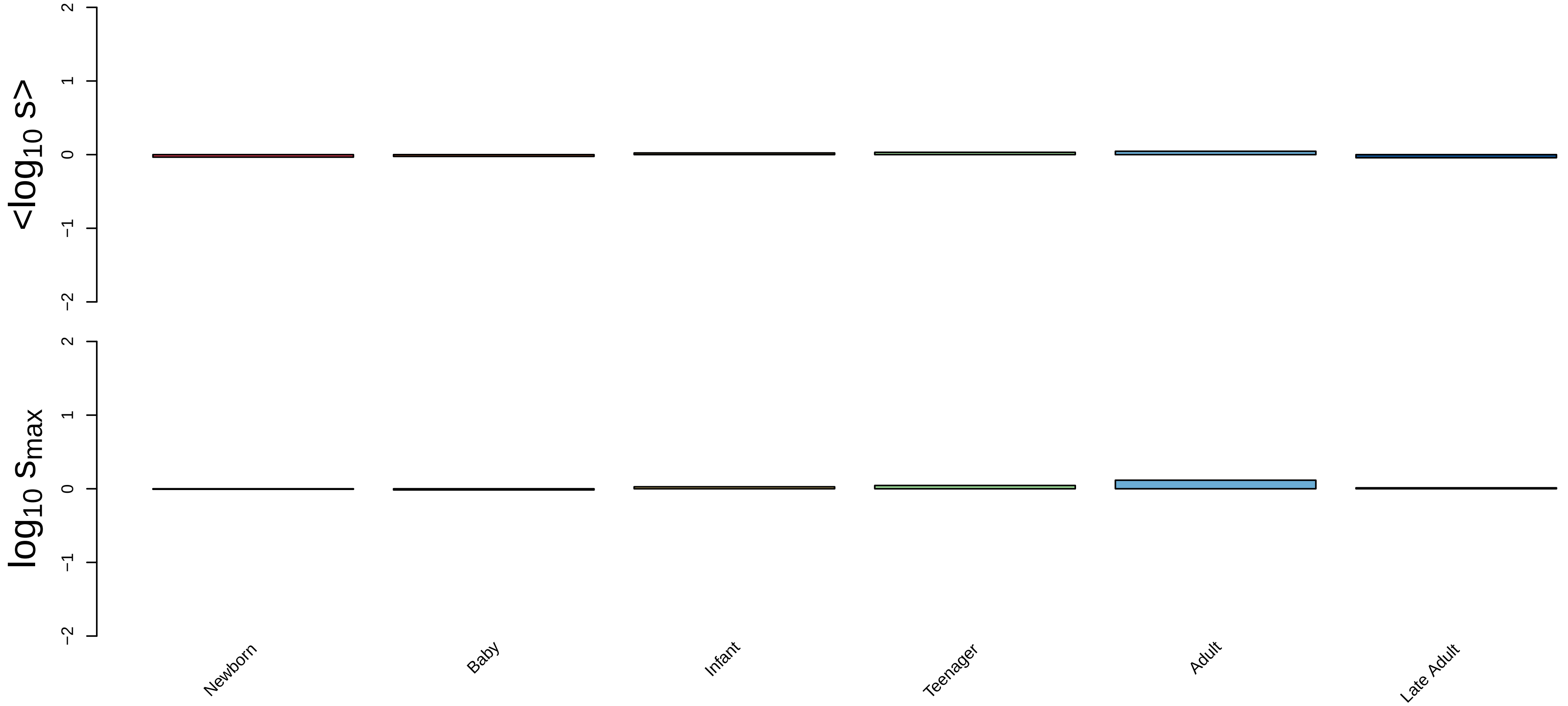

IL_17_signaling_pathway
genes with data

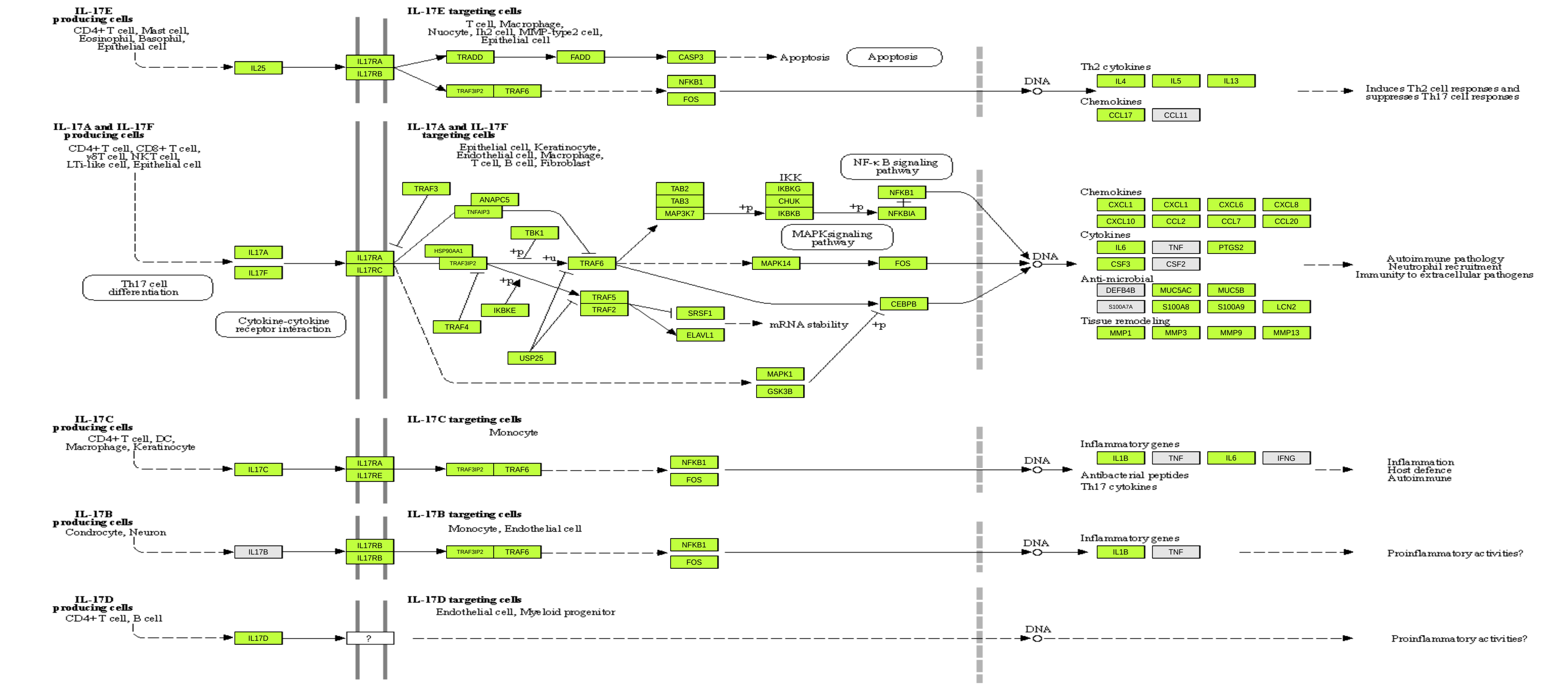

04657 1/12/21

(c) Kanehisa Laboratories

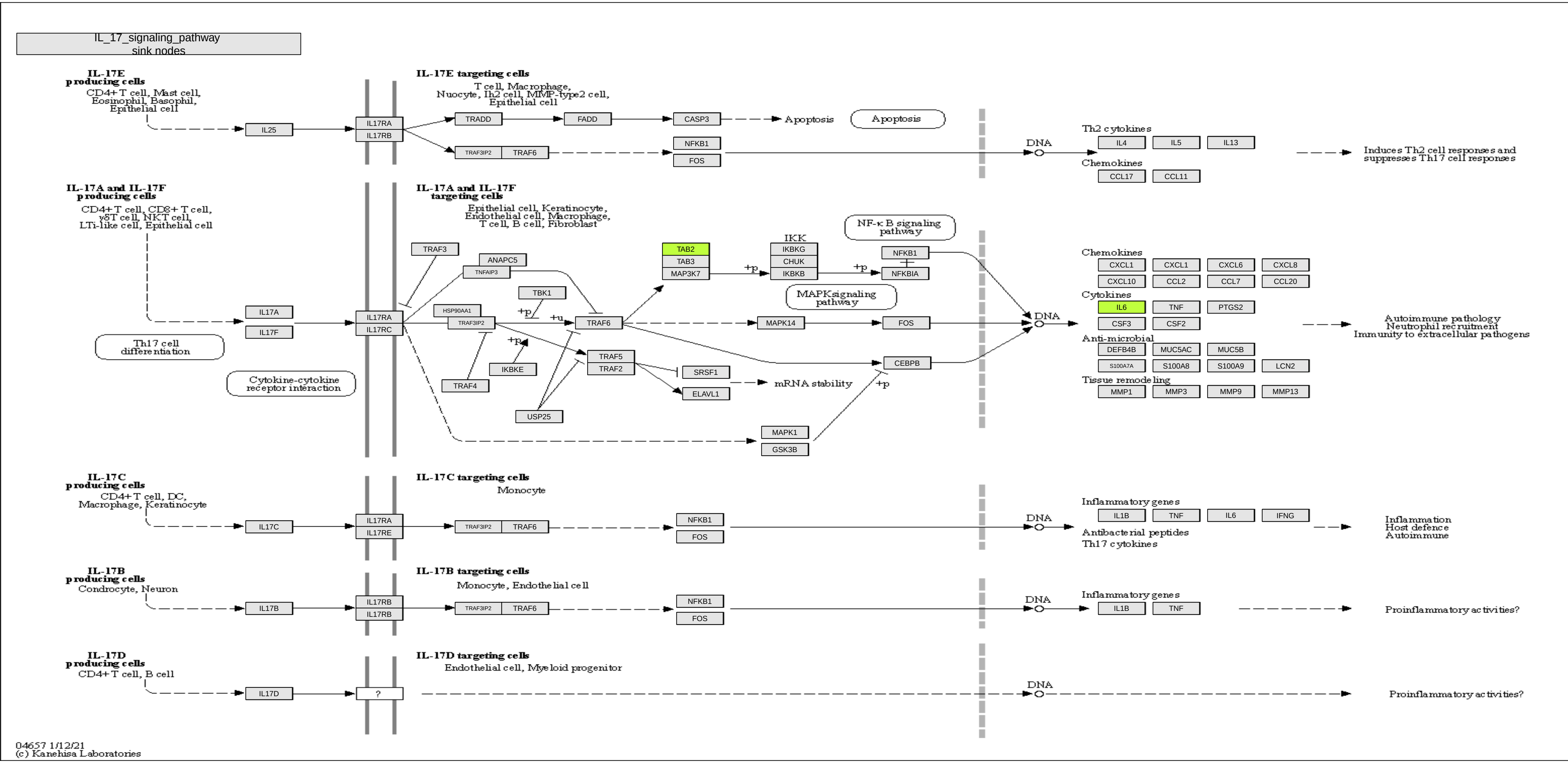
IL_17_signaling_pathway
sink nodes
IL-17E producing cells
CD4+ T cell, Mast cell, Eosinophil, Basophil, Epithelial cell
IL25
IL17RA
IL17RB
IL-17E targeting cells
T cell, Macrophage, Nuocyte, Ih2 cell, MMP-type2 cell, Epithelial cell
TRADD
FADD
CASP3
Apoptosis
Apoptosis
TRAF3IP2
TRAF6
NFKB1
FOS
DNA
Th2 cytokines
IL4
IL5
IL13
Chemokines
CCL17
CCL11
Induces Th2 cell responses and suppresses Th17 cell responses
IL-17A and IL-17F producing cells
CD4+ T cell, CD8+ T cell, γδT cell, NKT cell, LTi-like cell, Epithelial cell
IL-17A and IL-17F targeting cells
Epithelial cell, Keratinocyte, Endothelial cell, Macrophage, T cell, B cell, Fibroblast
IL17A
IL17F
IL17RA
IL17RC
Th17 cell differentiation
Cytokine-cytokine receptor interaction
TRAF3
ANAPC5
TNFAIP3
HSP90AA1
TRAF3IP2
TBK1
+p
+u
TRAF6
IKBKE
+p
TRAF4
TRAF5
TRAF2
USP25
TAB2
TAB3
MAP3K7
+p
IKK
IKBKG
CHUK
IKBKB
+p
NF-κ B signaling pathway
NFKB1
NFKBIA
MAPKsignaling pathway
MAPK14
FOS
CEBPB
SRSF1
ELAVL1
mRNA stability
+p
MAPK1
GSK3B
DNA
Chemokines
CXCL1
CXCL1
CXCL6
CXCL8
CXCL10
CCL2
CCL7
CCL20
Cytokines
IL6
TNF
PTGS2
CSF3
CSF2
Anti-microbial
DEFB4B
MUC5AC
MUC5B
S100A7A
S100A8
S100A9
LCN2
Tissue remodeling
MMP1
MMP3
MMP9
MMP13
Autoimmune pathology
Neutrophil recruitment
Immunity to extracellular pathogens
IL-17C producing cells
CD4+ T cell, DC, Macrophage, Keratinocyte
IL17C
IL17RA
IL17RE
IL-17C targeting cells
Monocyte
TRAF3IP2
TRAF6
NFKB1
FOS
DNA
Inflammatory genes
IL1B
TNF
IL6
IFNG
Antibacterial peptides
Th17 cytokines
Inflammation
Host defence
Autoimmune
IL-17B producing cells
Condrocyte, Neuron
IL17B
IL17RB
IL17RB
IL-17B targeting cells
Monocyte, Endothelial cell
TRAF3IP2
TRAF6
NFKB1
FOS
DNA
Inflammatory genes
IL1B
TNF
Proinflammatory activities?
IL-17D producing cells
CD4+ T cell, B cell
IL17D
?
IL-17D targeting cells
Endothelial cell, Myeloid progenitor
DNA
Proinflammatory activities?
04657 1/12/21
(c) Kanehisa Laboratories

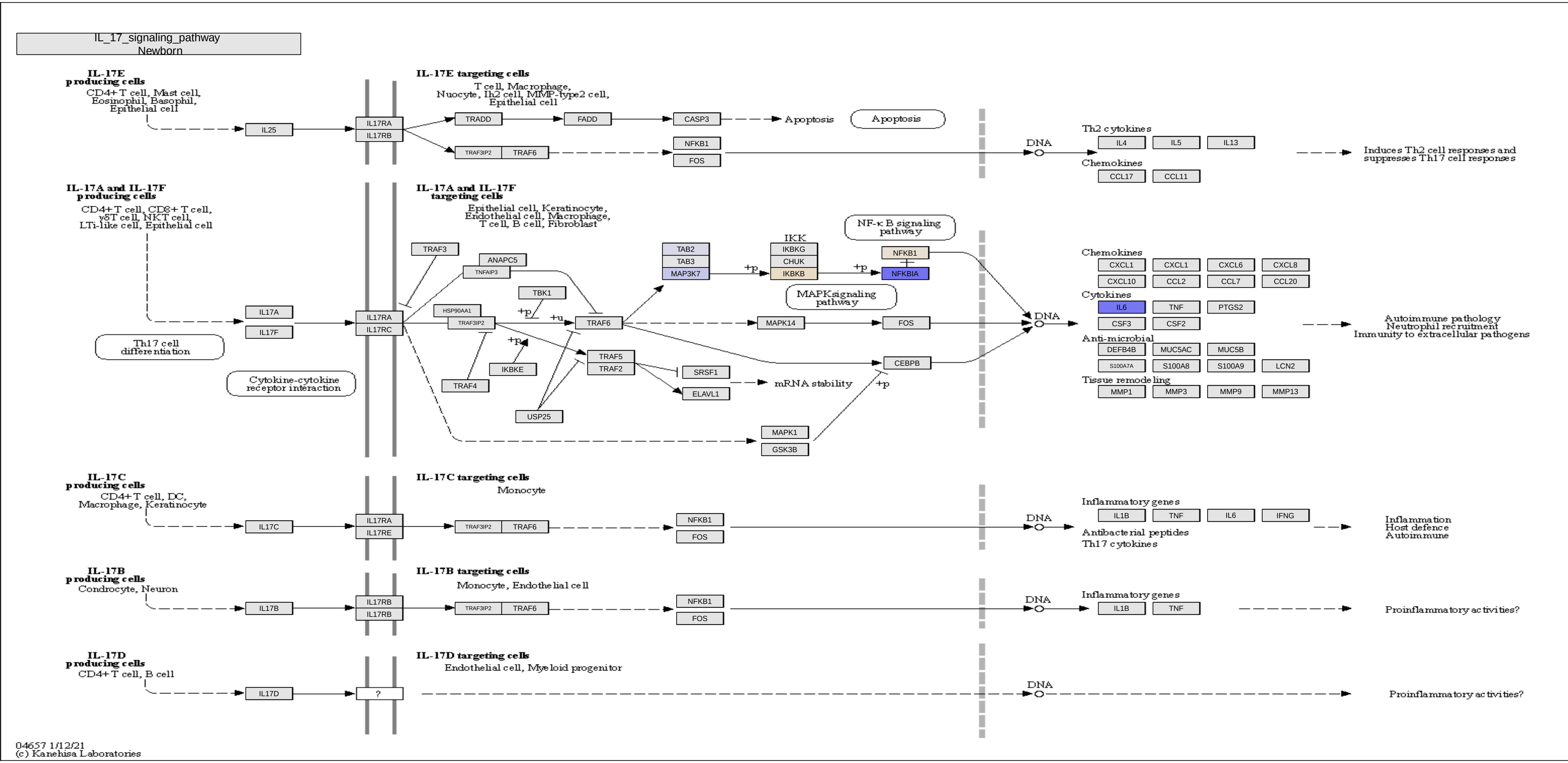

IL_17_signaling_pathway
Newborn
IL-17E producing cells
CD4+ T cell, Mast cell, Eosinophil, Basophil, Epithelial cell
IL-17E targeting cells
T cell, Macrophage, Nuocyte, Ih2 cell, MMP-type2 cell, Epithelial cell
IL25
IL17RA
IL17RB
TRADD
FADD
CASP3
Apoptosis
TRAF3IP2
TRAF6
NFKB1
FOS
DNA
Th2 cytokines
IL4
IL5
IL13
Chemokines
CCL17
CCL11
Induces Th2 cell responses and suppresses Th17 cell responses
IL-17A and IL-17F producing cells
CD4+ T cell, CD8+ T cell, γδT cell, NKT cell, LTi-like cell, Epithelial cell
IL-17A and IL-17F targeting cells
Epithelial cell, Keratinocyte, Endothelial cell, Macrophage, T cell, B cell, Fibroblast
IL17A
IL17F
IL17RC
TRAF3
ANAPC5
TNFAIP3
HSP90AA1
TBK1
IKBKE
TRAF4
USP25
TRAF5
TRAF2
SRSF1
ELAVL1
mRNA stability
TAB2
TAB3
MAP3K7
IKK
IKBKG
CHUK
IKBKB
NFKBIA
NF-κB signaling pathway
MAPKsignaling pathway
MAPK14
CEBPB
MAPK1
GSK3B
+p
+u
Th17 cell differentiation
Cytokine-cytokine receptor interaction
CXCL1
CXCL6
CXCL8
CXCL10
CCL2
CCL7
CCL20
Cytokines
IL6
TNF
PTGS2
CSF3
CSF2
Anti-microbial
DEFB4B
MUC5AC
MUC5B
S100A7A
S100A8
S100A9
LCN2
Tissue remodeling
MMP1
MMP3
MMP9
MMP13
Autoimmune pathology
Neutrophil recruitment
Immunity to extracellular pathogens
IL-17C producing cells
CD4+ T cell, DC, Macrophage, Keratinocyte
IL-17C targeting cells
Monocyte
IL17C
IL17RE
Inflammatory genes
IL1B
IFNG
Antibacterial peptides
Th17 cytokines
Inflammation
Host defence
Autoimmune
IL-17B producing cells
Condrocyte, Neuron
IL-17B targeting cells
Monocyte, Endothelial cell
IL17B
Proinflammatory activities?
IL-17D producing cells
CD4+ T cell, B cell
IL-17D targeting cells
Endothelial cell, Myeloid progenitor
IL17D
?
04657 1/12/21
(c) Kanehisa Laboratories

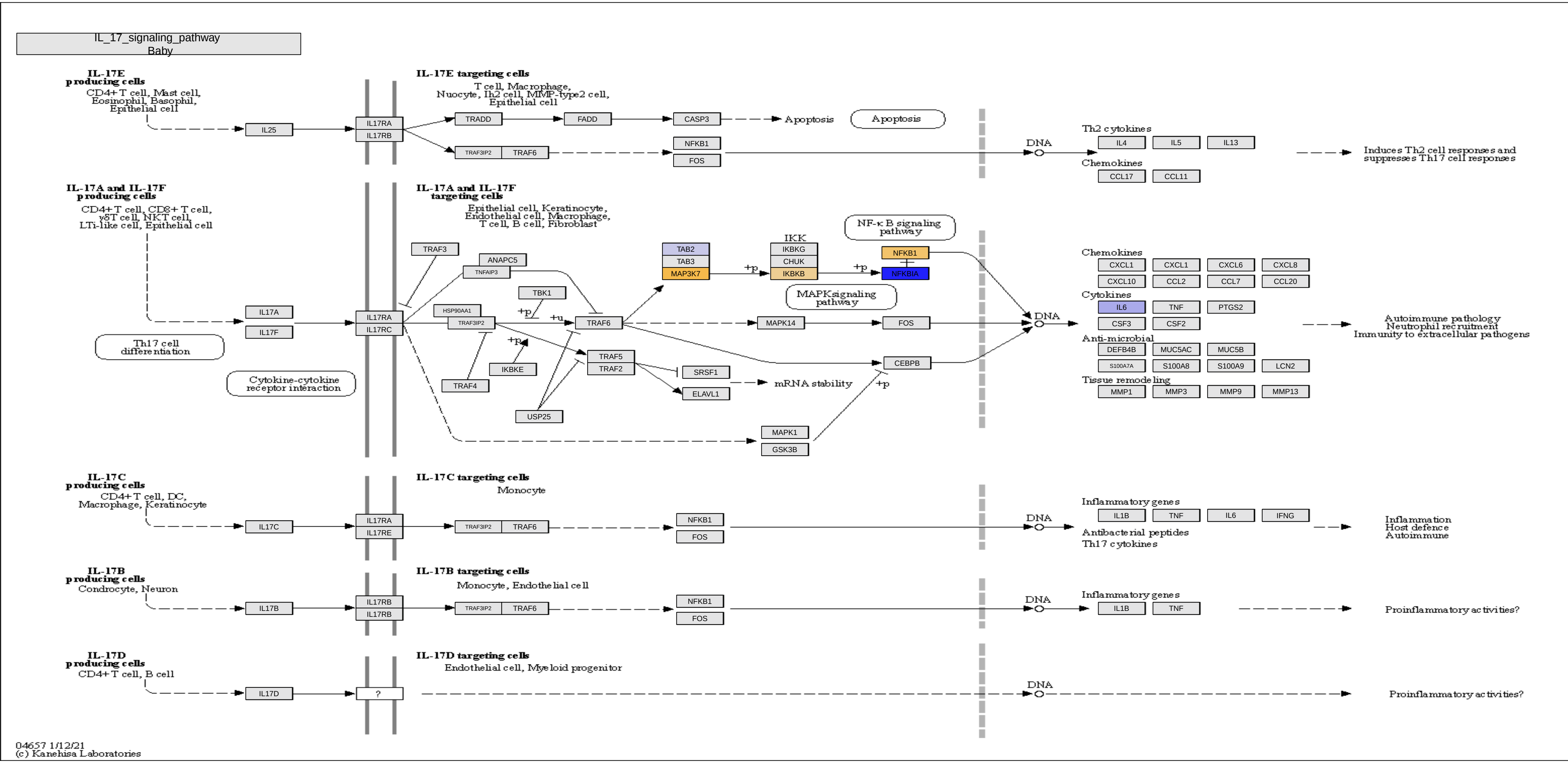
IL_17_signaling_pathway
Baby
IL-17E producing cells
CD4+ T cell, Mast cell, Eosinophil, Basophil, Epithelial cell
IL25
IL17RA
IL17RB
IL-17E targeting cells
T cell, Macrophage, Nuocyte, Ih2 cell, MMP-type2 cell, Epithelial cell
TRADD
FADD
CASP3
Apoptosis
Apoptosis
TRAF3IP2
TRAF6
NFKB1
FOS
DNA
Th2 cytokines
IL4
IL5
IL13
Chemokines
CCL17
CCL11
Induces Th2 cell responses and suppresses Th17 cell responses
IL-17A and IL-17F producing cells
CD4+ T cell, CD8+ T cell, γδT cell, NKT cell, LTi-like cell, Epithelial cell
IL-17A and IL-17F targeting cells
Epithelial cell, Keratinocyte, Endothelial cell, Macrophage, T cell, B cell, Fibroblast
IL17A
IL17F
IL17RA
IL17RC
Th17 cell differentiation
Cytokine-cytokine receptor interaction
TRAF3
ANAPC5
TNFAIP3
HSP90AA1
TRAF3IP2
TBK1
+p
+u
TRAF6
+p
IKBKE
TRAF4
TRAF5
TRAF2
USP25
TAB2
TAB3
MAP3K7
+p
IKK
IKBKG
CHUK
IKBKB
+p
NF-κB signaling pathway
NFKB1
NFKBIA
MAPKsignaling pathway
MAPK14
FOS
CEBPB
+p
SRSF1
ELAVL1
mRNA stability
MAPK1
GSK3B
DNA
Chemokines
CXCL1
CXCL1
CXCL6
CXCL8
CXCL10
CCL2
CCL7
CCL20
Cytokines
IL6
TNF
PTGS2
CSF3
CSF2
Anti-microbial
DEFB4B
MUC5AC
MUC5B
S100A7A
S100A8
S100A9
LCN2
Tissue remodeling
MMP1
MMP3
MMP9
MMP13
Autoimmune pathology
Neutrophil recruitment
Immunity to extracellular pathogens
IL-17C producing cells
CD4+ T cell, DC, Macrophage, Keratinocyte
IL17C
IL17RA
IL17RE
IL-17C targeting cells
Monocyte
TRAF3IP2
TRAF6
NFKB1
FOS
DNA
Inflammatory genes
IL1B
TNF
IL6
IFNG
Antibacterial peptides
Th17 cytokines
Inflammation
Host defence
Autoimmune
IL-17B producing cells
Condrocyte, Neuron
IL17B
IL17RB
IL17RB
IL-17B targeting cells
Monocyte, Endothelial cell
TRAF3IP2
TRAF6
NFKB1
FOS
DNA
Inflammatory genes
IL1B
TNF
Proinflammatory activities?
IL-17D producing cells
CD4+ T cell, B cell
IL17D
?
IL-17D targeting cells
Endothelial cell, Myeloid progenitor
DNA
Proinflammatory activities?
04657 1/12/21
(c) Kanehisa Laboratories

IL_17_signaling_pathway
Infant

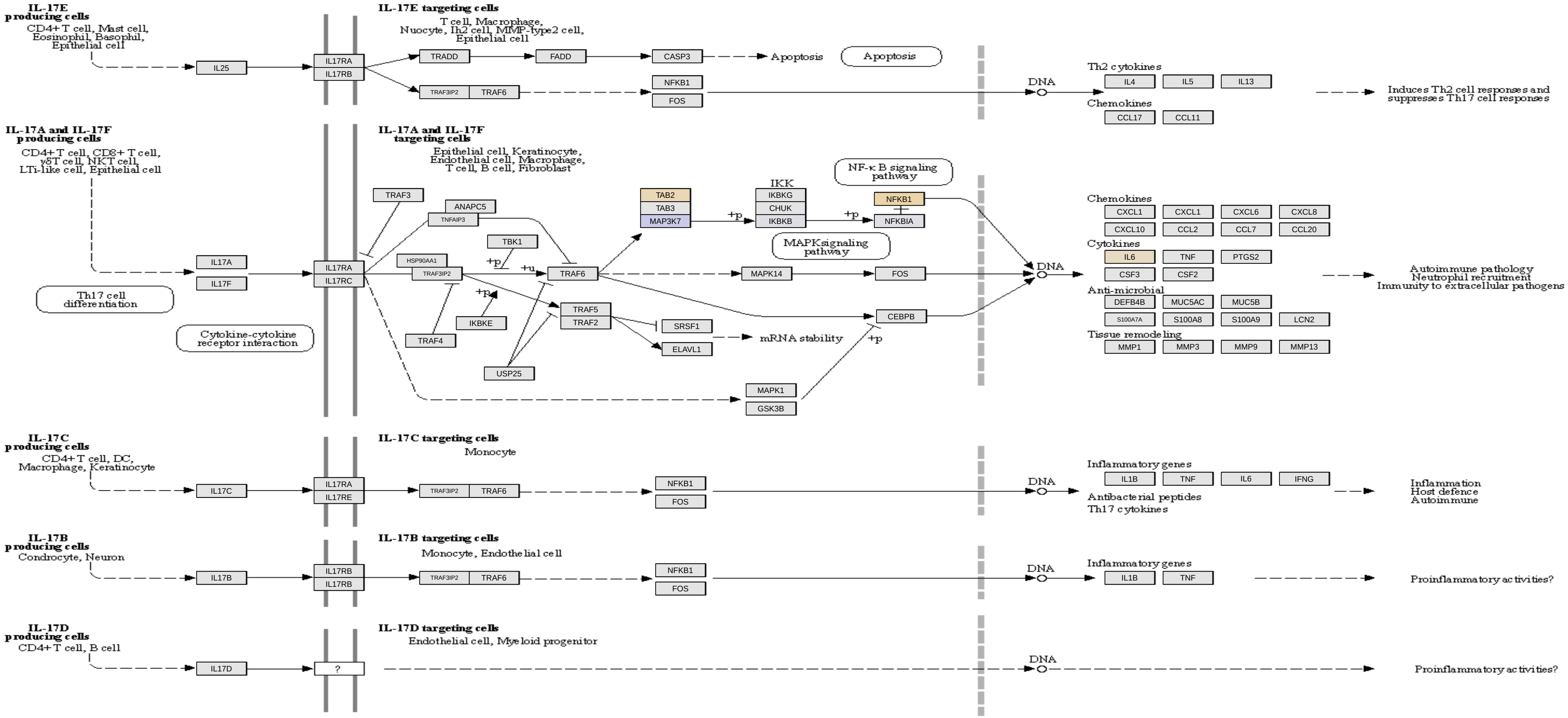

04657 1/12/21
(c) Kanehisa Laboratories

IL_17_signaling_pathway
Teenager

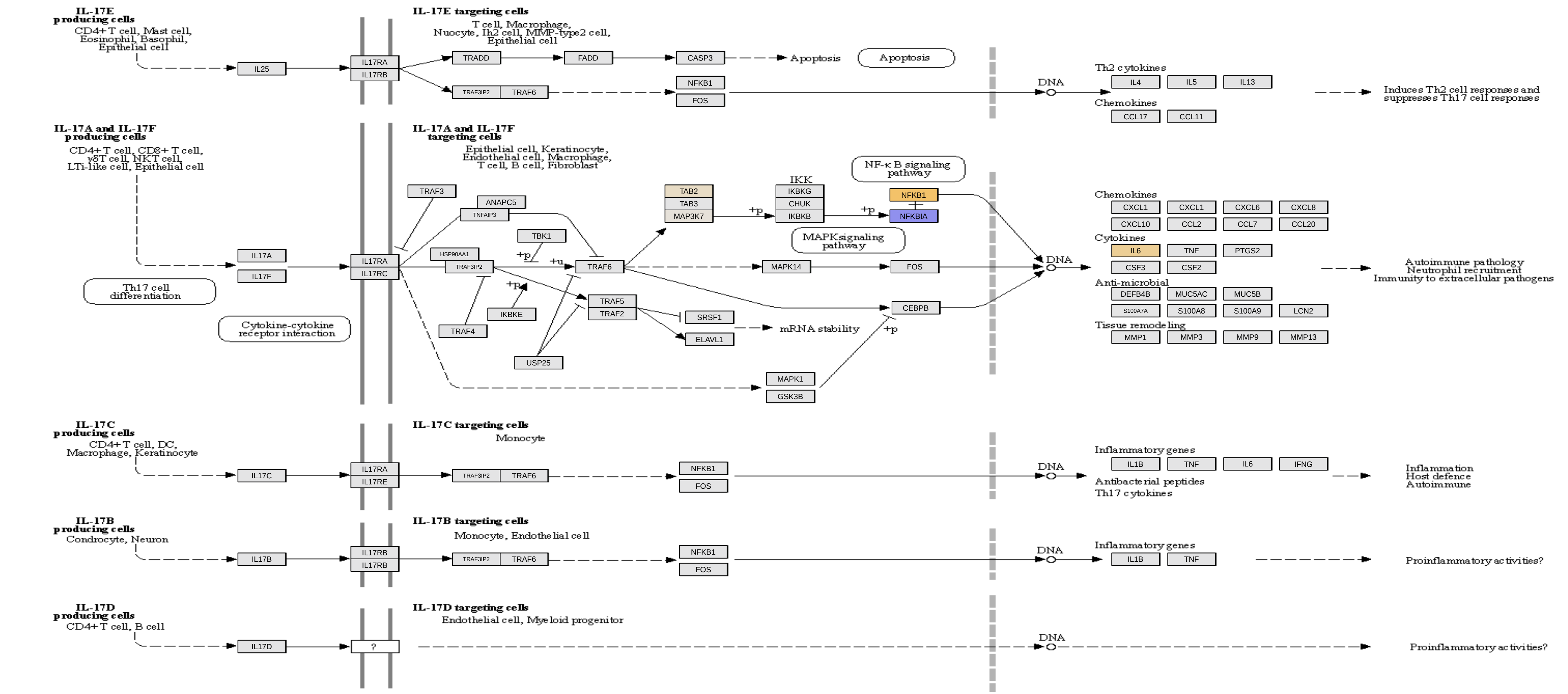

04657 1/12/21
(c) Kanehisa Laboratories

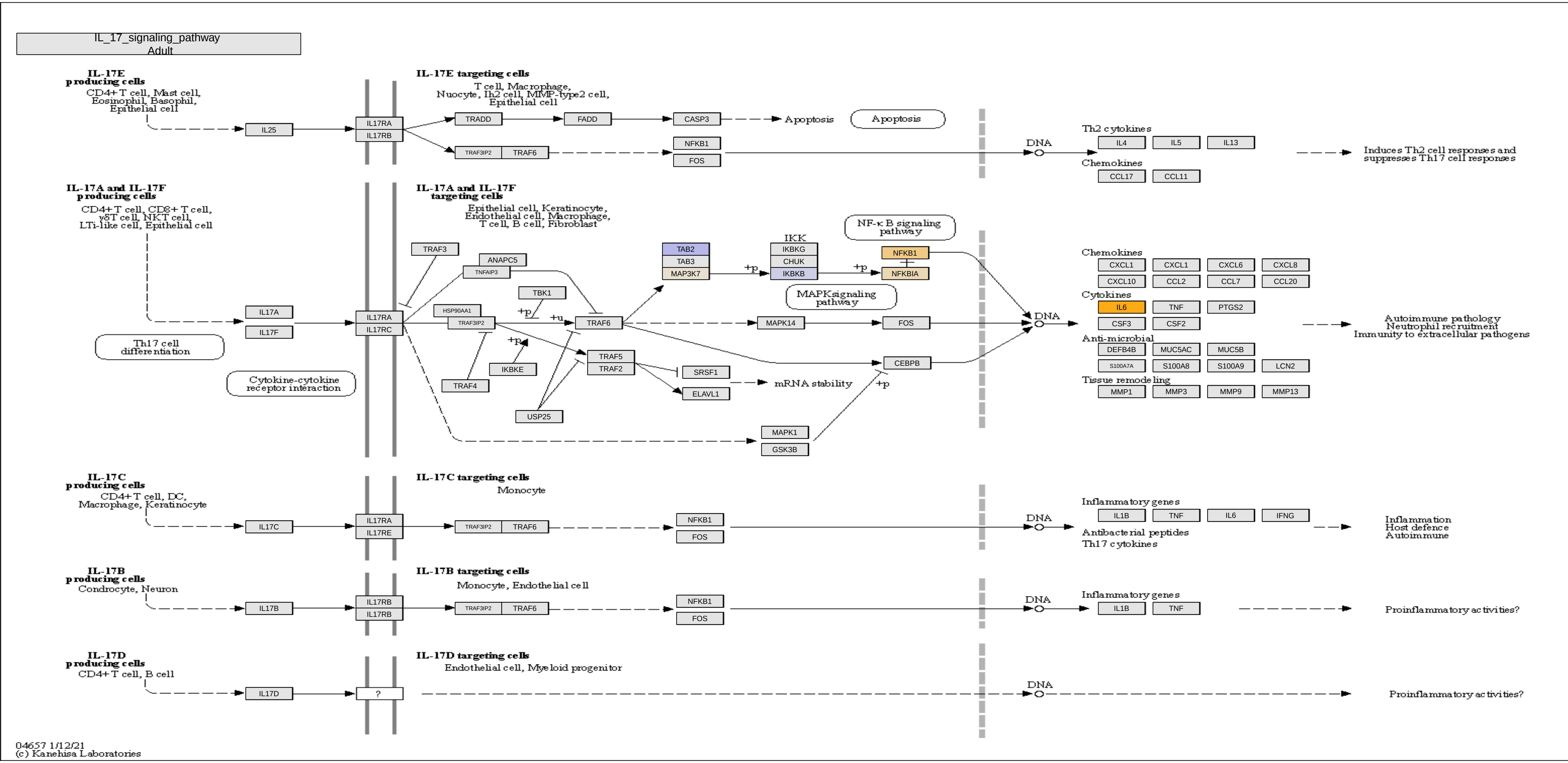
IL_17_signaling_pathway
Adult
IL-17E producing cells
CD4+ T cell, Mast cell, Eosinophil, Basophil, Epithelial cell
IL25
IL17RA
IL17RB
IL-17E targeting cells
T cell, Macrophage, Nuocyte, Ih2 cell, MMP-type2 cell, Epithelial cell
TRADD
FADD
CASP3
Apoptosis
Apoptosis
TRAF3IP2
TRAF6
NFKB1
FOS
DNA
Th2 cytokines
IL4
IL5
IL13
Chemokines
CCL17
CCL11
Induces Th2 cell responses and suppresses Th17 cell responses
IL-17A and IL-17F producing cells
CD4+ T cell, CD8+ T cell, γδT cell, NKT cell, LTi-like cell, Epithelial cell
IL17A
IL17F
Th17 cell differentiation
Cytokine-cytokine receptor interaction
IL17RA
IL17RC
IL-17A and IL-17F targeting cells
Epithelial cell, Keratinocyte, Endothelial cell, Macrophage, T cell, B cell, Fibroblast
TRAF3
ANAPC5
TNFAIP3
HSP90AA1
TRAF3IP2
TBK1
+p
+u
+p
IKBKE
TRAF4
USP25
TRAF6
TRAF5
TRAF2
SRSF1
ELAVL1
mRNA stability
TAB2
TAB3
MAP3K7
+p
IKK
IKBKG
CHUK
IKBKB
+p
NF-κB signaling pathway
NFKB1
NFKBIA
MAPKsignaling pathway
MAPK14
FOS
CEBPB
+p
MAPK1
GSK3B
DNA
Chemokines
CXCL1
CXCL2
CXCL6
CXCL8
CXCL10
CCL2
CCL7
CCL20
Cytokines
IL6
TNF
PTGS2
CSF3
CSF2
Anti-microbial
DEFB4B
MUC5AC
MUC5B
S100A7A
S100A8
S100A9
LCN2
Tissue remodeling
MMP1
MMP3
MMP9
MMP13
Autoimmune pathology
Neutrophil recruitment
Immunity to extracellular pathogens
IL-17C producing cells
CD4+ T cell, DC, Macrophage, Keratinocyte
IL17C
IL17RA
IL17RE
IL-17C targeting cells
Monocyte
TRAF3IP2
TRAF6
NFKB1
FOS
DNA
Inflammatory genes
IL1B
TNF
IL6
IFNG
Antibacterial peptides
Th17 cytokines
Inflammation
Host defence
Autoimmune
IL-17B producing cells
Condrocyte, Neuron
IL17B
IL17RB
IL17RB
IL-17B targeting cells
Monocyte, Endothelial cell
TRAF3IP2
TRAF6
NFKB1
FOS
DNA
Inflammatory genes
IL1B
TNF
Proinflammatory activities?
IL-17D producing cells
CD4+ T cell, B cell
IL17D
?
IL-17D targeting cells
Endothelial cell, Myeloid progenitor
DNA
Proinflammatory activities?
04657 1/12/21
(c) Kanehisa Laboratories

IL_17_signaling_pathway
Late Adult

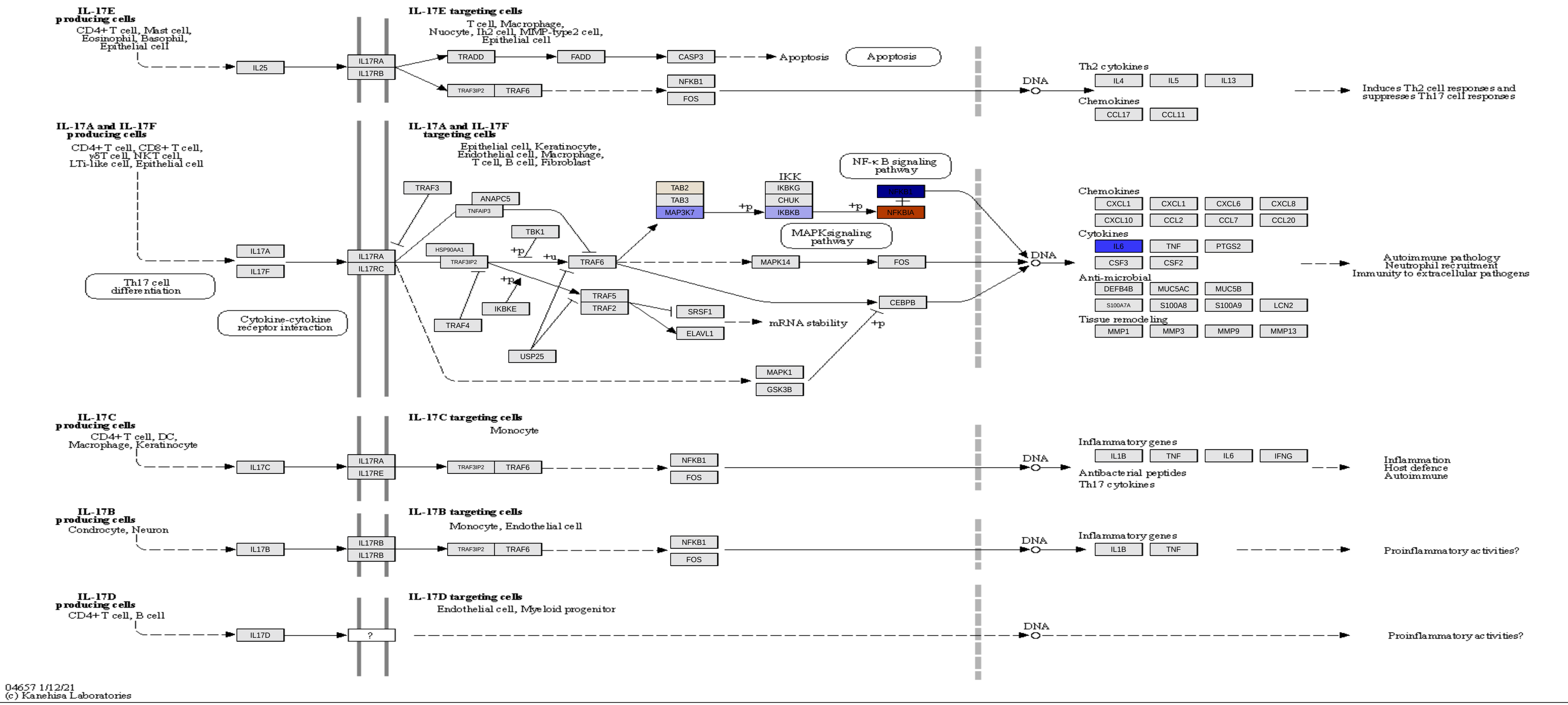

Supplement: Supplementary file 1 [file cells-11-00362-s001.zip › Suppl-Material-S3-Pathways-PSF_Expression/IL_17_signaling_pathway.pdf]
