# Supplementary material for: The Transcriptome and Methylome of the Developing and Aging Brain and Their Relations to Gliomas and Psychological Disorders
Source: Cells. 2022 Jan 21;11(3):362. doi: 10.3390/cells11030362 (PMC8834030; doi:10.3390/cells11030362)
Supplement: Supplementary file 1 [file cells-11-00362-s001.zip › Suppl-Material-S3-Pathways-PSF_Expression/Insulin_signaling_pathway.pdf]

Insulin signaling pathway

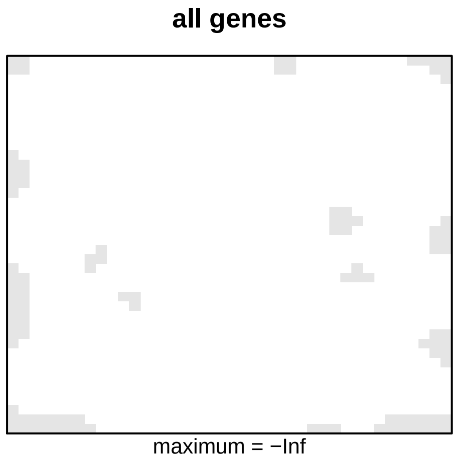

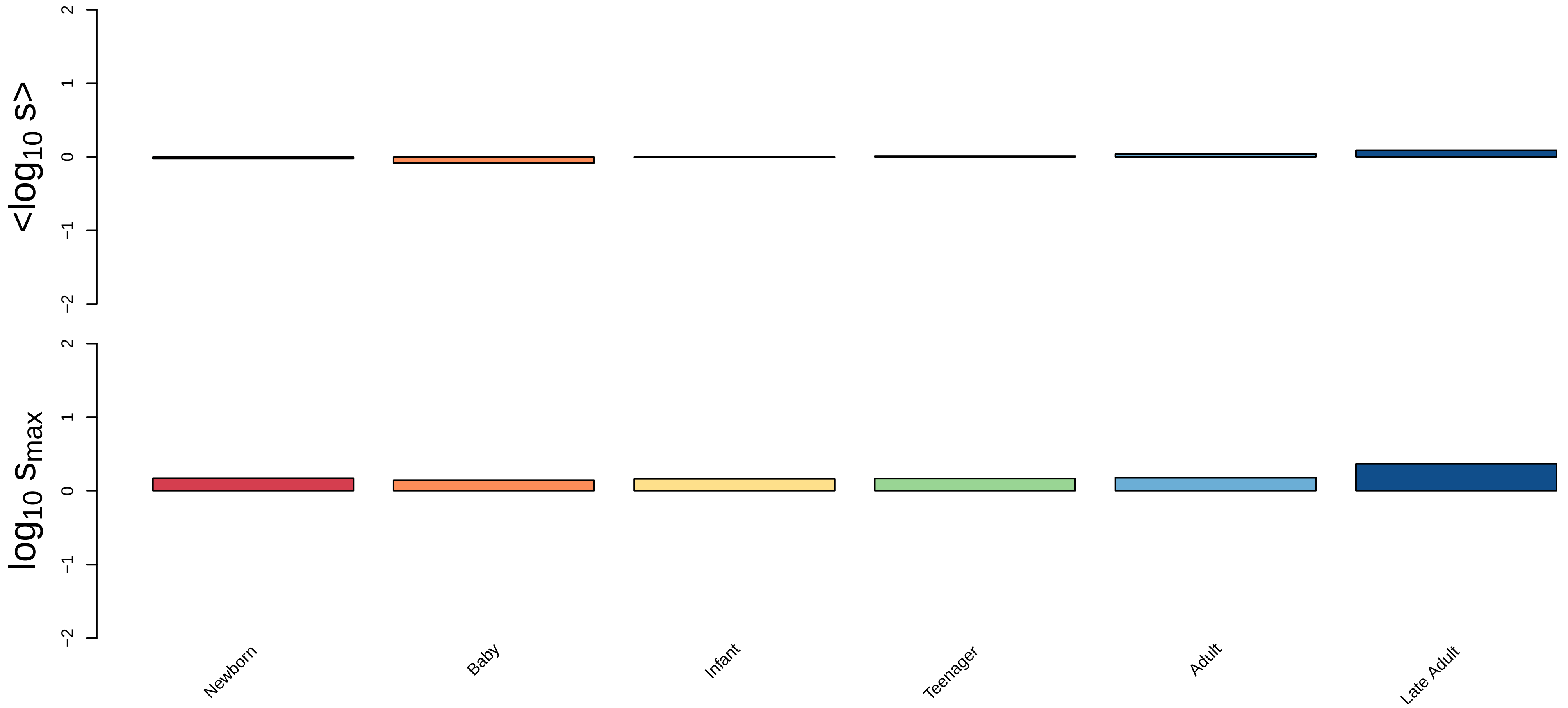

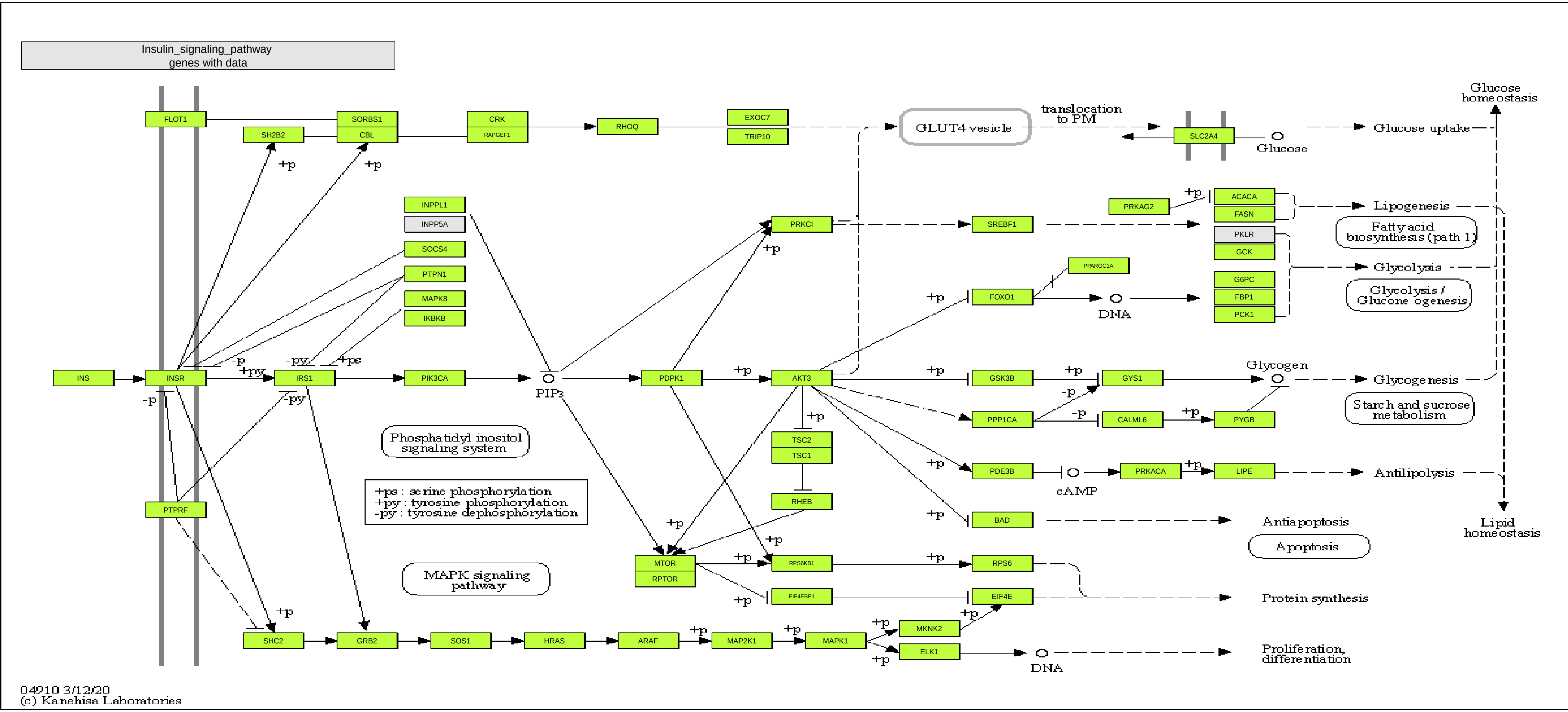
Insulin_signaling_pathway
genes with data
FLOT1
SORBS1
SH2B2
CBL
CRK
RAPGEF1
RHOQ
EXOC7
TRIP10
GLUT4 vesicle
translocation
to PM
SLC2A4
Glucose
Glucose uptake
Glucose
homeostasis
INPPL1
INPP5A
SOCS4
PTPN1
MAPK8
IKBKB
PRKCI
SREBF1
PRKAG2
ACACA
FASN
Lipogenesis
Fatty acid
biosynthesis (path 1)
PKLR
GCK
Glycolysis
PPARGC1A
G6PC
FBP1
PCK1
Glycolysis /
Gluconeogenesis
FOXO1
DNA
INS
INSR
IRS1
PIK3CA
PIP3
PDPK1
AKT3
GSK3B
GYS1
Glycogen
Glycogenesis
PPP1CA
CALML6
PYGB
Starch and sucrose
metabolism
TSC2
TSC1
RHEB
PDE3B
cAMP
PRKACA
LIPE
Antilipolysis
Lipid
homeostasis
BAD
Antiapoptosis
Apoptosis
PTPRF
Phosphatidyl inositol
signaling system
+ps : serine phosphorylation
+py : tyrosine phosphorylation
-py : tyrosine dephosphorylation
MTOR
RPTOR
RPS6KB1
RPS6
EIF4EBP1
EIF4E
Protein synthesis
MAPK signaling
pathway
SHC2
GRB2
SOS1
HRAS
ARAF
MAP2K1
MAPK1
MKNK2
ELK1
DNA
Proliferation,
differentiation
04910 3/12/20
(c) Kanehisa Laboratories

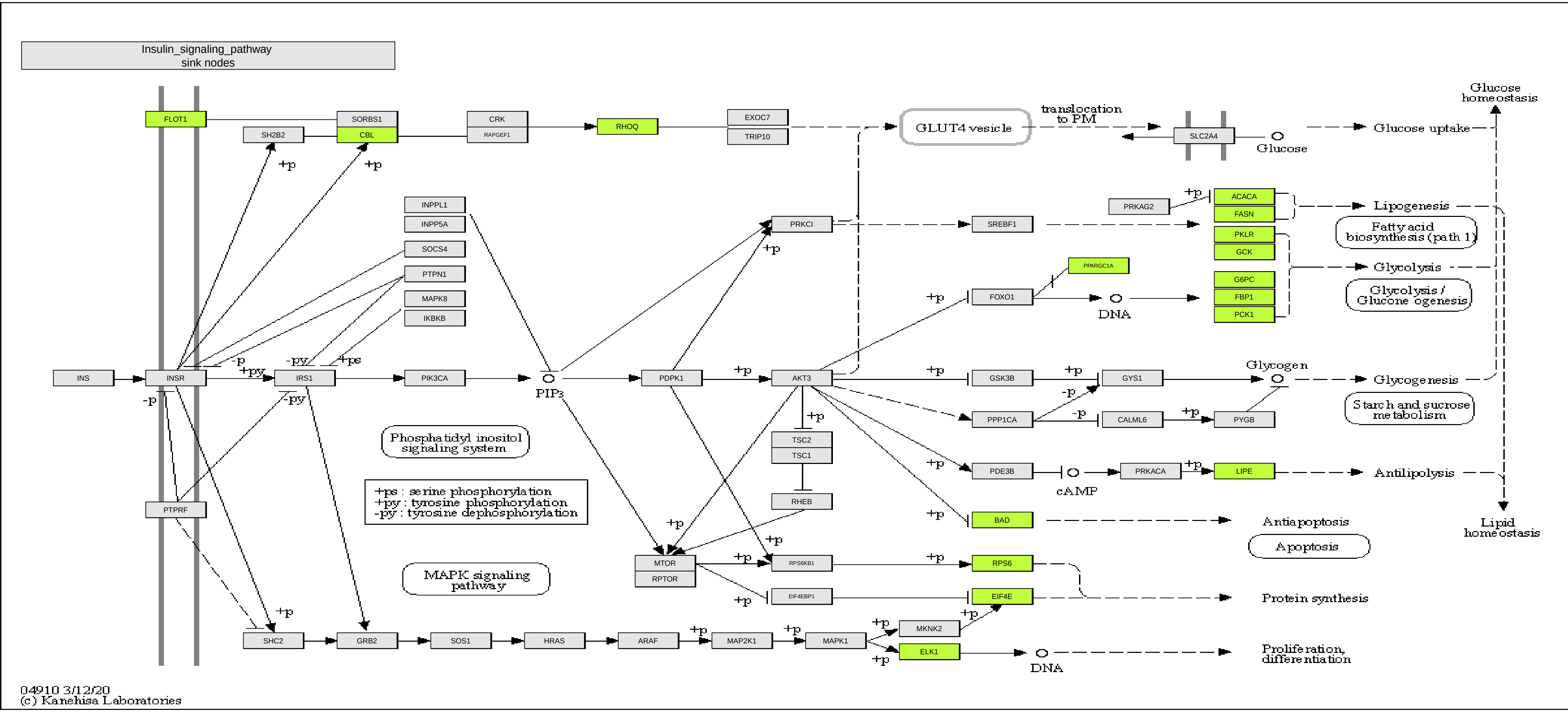

Insulin_signaling_pathway
sink nodes
FLOT1
SORBS1
SH2B2
CBL
CRK
RAPGEF1
RHOQ
EXOC7
TRIP10
GLUT4 vesicle
translocation to PM
SLC2A4
Glucose
Glucose uptake
Glucose homeostasis
INPPL1
INPP5A
SOCS4
PTPN1
MAPK8
IKBKB
PRKCI
SREBF1
PRKAG2
ACACA
FASN
Lipogenesis
Fatty acid biosynthesis (path 1)
PKLR
GCK
PPARGC1A
G6PC
FBP1
PCK1
Glycolysis
Glycolysis / Gluconeogenesis
FOXO1
DNA
INS
INSR
IRS1
PIK3CA
PIP3
PDPK1
AKT3
GSK3B
GYS1
Glycogen
Glycogenesis
PPP1CA
CALML6
PYGB
Starch and sucrose metabolism
TSC2
TSC1
RHEB
PDE3B
cAMP
PRKACA
LIPE
Antilipolysis
Lipid homeostasis
BAD
Antiapoptosis
Apoptosis
Phosphatidyl inositol signaling system
+ps : serine phosphorylation
+py : tyrosine phosphorylation
-py : tyrosine dephosphorylation
PTPRF
MTOR
RPTOR
RPS6KB1
RPS6
EIF4EBP1
EIF4E
Protein synthesis
MAPK signaling pathway
SHC2
GRB2
SOS1
HRAS
ARAF
MAP2K1
MAPK1
MKNK2
ELK1
DNA
Proliferation, differentiation
04910 3/12/20
(c) Kanehisa Laboratories

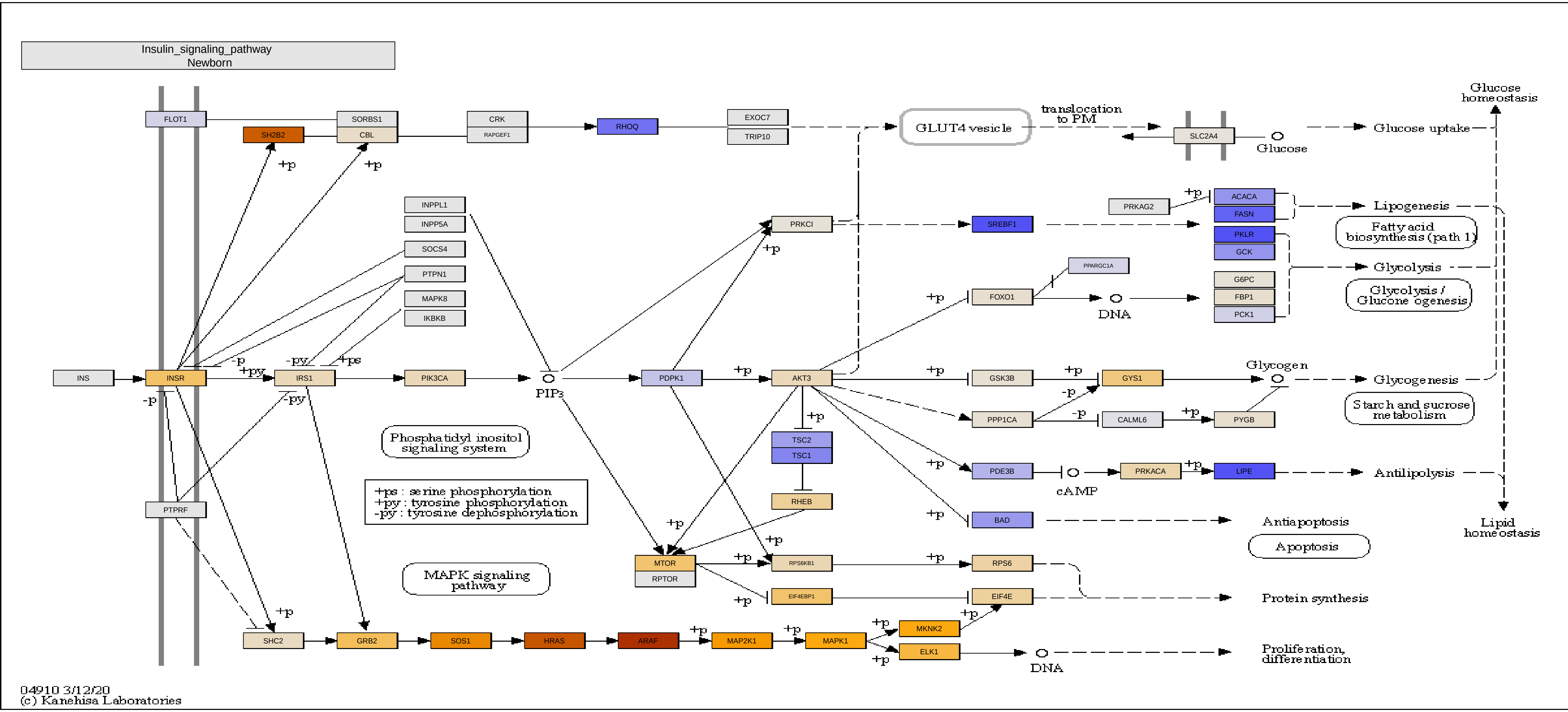
Insulin_signaling_pathway
Newborn
FLOT1
SORBS1
CBL
SH2B2
CRK
RAPGEF1
RHOQ
EXOC7
TRIP10
GLUT4 vesicle
translocation to PM
SLC2A4
Glucose
Glucose uptake
Glucose homeostasis
INPPL1
INPP5A
SOCS4
PTPN1
MAPK8
IKBKB
PRKCI
SREBF1
PRKAG2
ACACA
FASN
Lipogenesis
Fatty acid biosynthesis (path 1)
PKLR
GCK
Glycolysis
PPARGC1A
G6PC
FBP1
PCK1
Glycolysis / Gluconeogenesis
FOXO1
DNA
INS
INSR
IRS1
PIK3CA
PIP3
PDPK1
AKT3
GSK3B
GYS1
Glycogen
Glycogenesis
Starch and sucrose metabolism
PPP1CA
CALML6
PYGB
TSC2
TSC1
RHEB
PDE3B
cAMP
PRKACA
LIPE
Antilipolysis
Lipid homeostasis
BAD
Antiapoptosis
Apoptosis
PTPRF
Phosphatidyl inositol signaling system
+ps : serine phosphorylation
+py : tyrosine phosphorylation
-py : tyrosine dephosphorylation
MAPK signaling pathway
MTOR
RPTOR
RPS6KB1
RPS6
EIF4EBP1
EIF4E
Protein synthesis
SHC2
GRB2
SOS1
HRAS
ARAF
MAP2K1
MAPK1
MKNK2
ELK1
Proliferation, differentiation
04910 3/12/20
(c) Kanehisa Laboratories

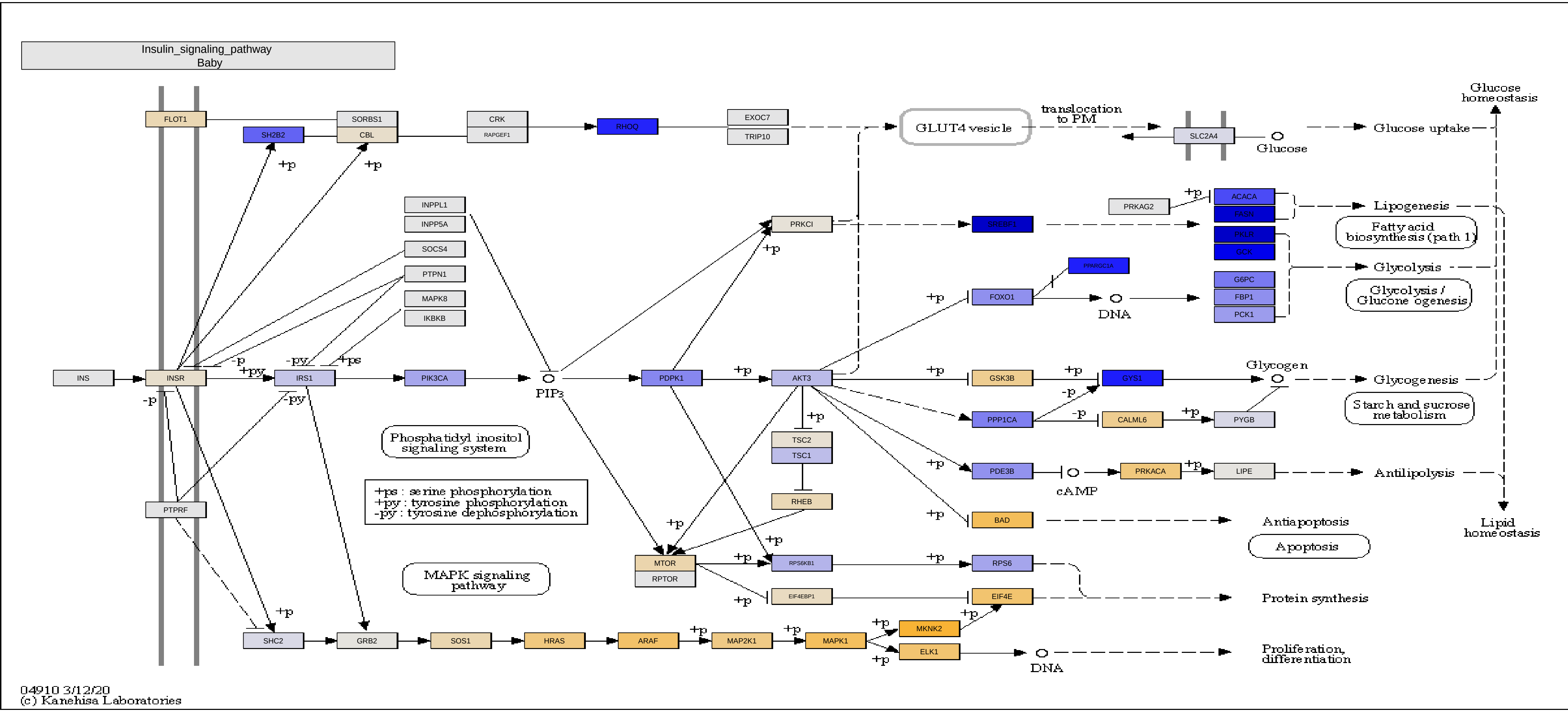

Insulin_signaling_pathway
Baby
FLOT1
SORBS1
CBL
SH2B2
CRK
RAPGEF1
RHOQ
EXOC7
TRIP10
GLUT4 vesicle
translocation to PM
SLC2A4
Glucose
Glucose uptake
Glucose homeostasis
PRKAG2
ACACA
FASN
Lipogenesis
Fatty acid biosynthesis (path 1)
PKLR
GCK
Glycolysis
Glycolysis / Gluconeogenesis
PPARGC1A
G6PC
FBP1
PCK1
SREBF1
PRKCI
FOXO1
DNA
INPPL1
INPP5A
SOCS4
PTPN1
MAPK8
IKBKB
INS
INSR
IRS1
PIK3CA
PIP3
PDPK1
AKT3
GSK3B
GYS1
Glycogen
Glycogenesis
Starch and sucrose metabolism
PPP1CA
CALML6
PYGB
TSC2
TSC1
RHEB
PDE3B
cAMP
PRKACA
LIPE
Antilipolysis
Lipid homeostasis
BAD
Antiapoptosis
Apoptosis
Phosphatidyl inositol signaling system
+ps : serine phosphorylation
+py : tyrosine phosphorylation
-py : tyrosine dephosphorylation
PTPRF
MTOR
RPTOR
RPS6KB1
RPS6
EIF4EBP1
EIF4E
Protein synthesis
MAPK signaling pathway
SHC2
GRB2
SOS1
HRAS
ARAF
MAP2K1
MAPK1
MKNK2
ELK1
DNA
Proliferation, differentiation
04910 3/12/20
(c) Kanehisa Laboratories

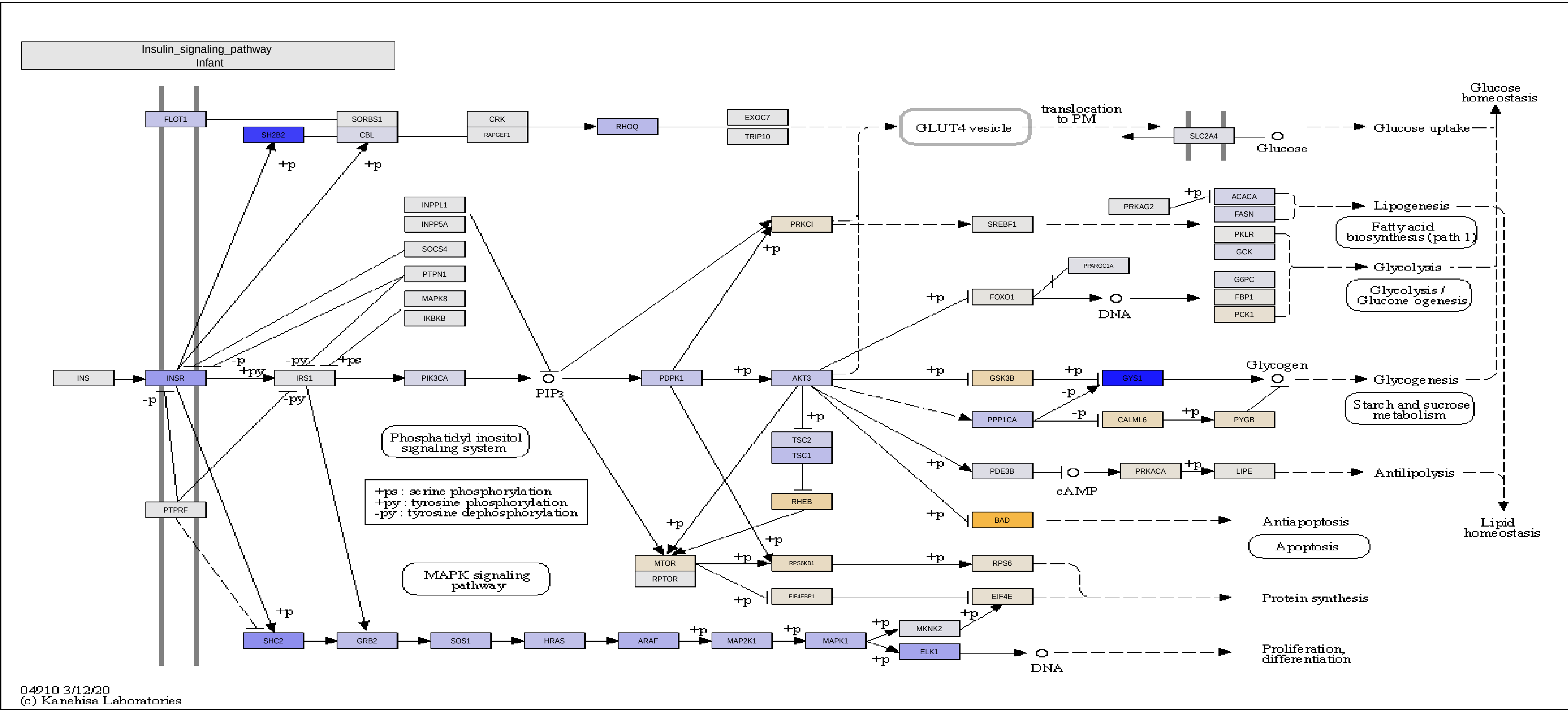
Insulin_signaling_pathway
Infant
FLOT1
SORBS1
SH2B2
CBL
CRK
RAPGEF1
RHOQ
EXOC7
TRIP10
GLUT4 vesicle
translocation to PM
SLC2A4
Glucose
Glucose uptake
Glucose homeostasis
INPPL1
INPP5A
SOCS4
PTPN1
MAPK8
IKBKB
PRKCI
SREBF1
PRKAG2
ACACA
FASN
Lipogenesis
Fatty acid biosynthesis (path 1)
PKLR
GCK
Glycolysis
PPARGC1A
FOXO1
DNA
G6PC
FBP1
PCK1
Glycolysis / Gluconeogenesis
INS
INSR
IRS1
PIK3CA
PIP3
PDPK1
AKT3
GSK3B
GYS1
Glycogen
Glycogenesis
PPP1CA
CALML6
PYGB
Starch and sucrose metabolism
TSC2
TSC1
RHEB
PDE3B
cAMP
PRKACA
LIPE
Antilipolysis
Lipid homeostasis
BAD
Antiapoptosis
Apoptosis
PTPRF
Phosphatidyl inositol signaling system
+ps : serine phosphorylation
+py : tyrosine phosphorylation
-py : tyrosine dephosphorylation
MAPK signaling pathway
MTOR
RPTOR
RPS6KB1
RPS6
EIF4EBP1
EIF4E
Protein synthesis
SHC2
GRB2
SOS1
HRAS
ARAF
MAP2K1
MAPK1
MKNK2
ELK1
Proliferation, differentiation
+p
-p
+py
-py
+ps
04910 3/12/20
(c) Kanehisa Laboratories

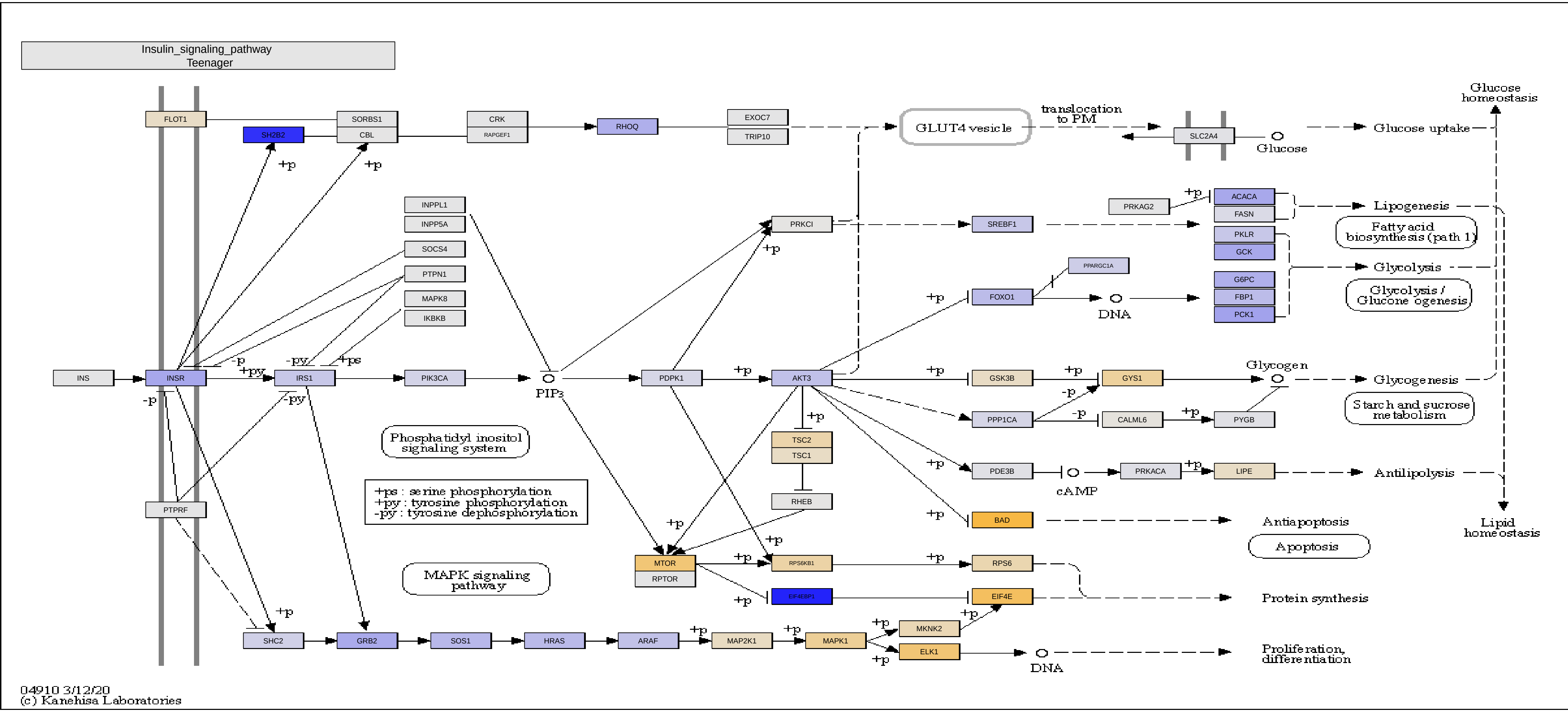
Insulin_signaling_pathway
Teenager
FLOT1
SORBS1
CBL
SH2B2
CRK
RAPGEF1
RHOQ
EXOC7
TRIP10
GLUT4 vesicle
translocation to PM
SLC2A4
Glucose
Glucose uptake
Glucose homeostasis
INPPL1
INPP5A
SOCS4
PTPN1
MAPK8
IKBKB
PRKCI
SREBF1
PRKAG2
ACACA
FASN
Lipogenesis
Fatty acid biosynthesis (path 1)
PKLR
GCK
Glycolysis
PPARGC1A
G6PC
FBP1
PCK1
Glycolysis / Gluconeogenesis
FOXO1
DNA
INS
INSR
IRS1
PIK3CA
PIP3
PDPK1
AKT3
GSK3B
GYS1
Glycogen
Glycogenesis
Starch and sucrose metabolism
PPP1CA
CALML6
PYGB
TSC2
TSC1
RHEB
PDE3B
cAMP
PRKACA
LIPE
Antilipolysis
Lipid homeostasis
BAD
Antiapoptosis
Apoptosis
Phosphatidyl inositol signaling system
+ps : serine phosphorylation
+py : tyrosine phosphorylation
-py : tyrosine dephosphorylation
PTPRF
MTOR
RPTOR
RPS6KB1
RPS6
EIF4EBP1
EIF4E
Protein synthesis
MAPK signaling pathway
SHC2
GRB2
SOS1
HRAS
ARAF
MAP2K1
MAPK1
MKNK2
ELK1
Proliferation, differentiation
04910 3/12/20
(c) Kanehisa Laboratories

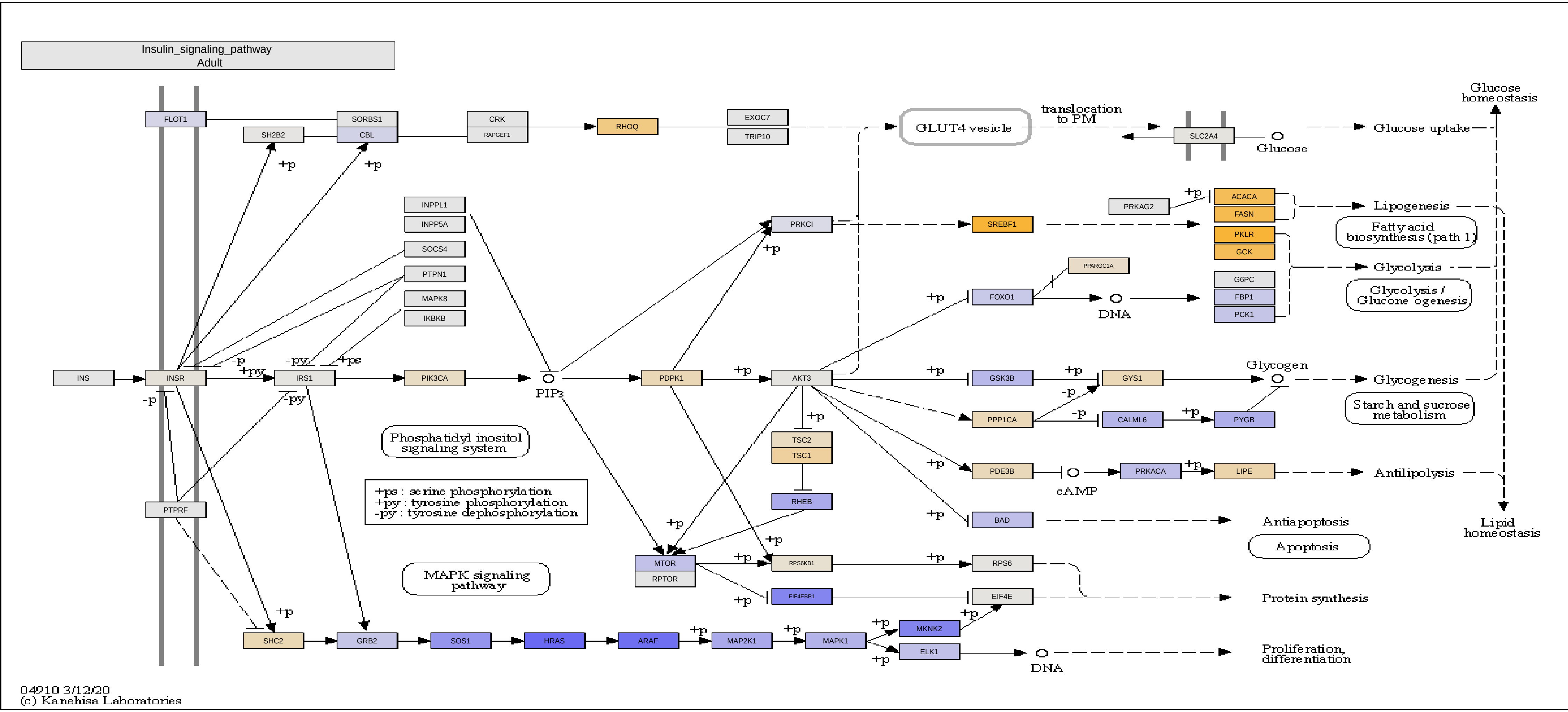

Insulin_signaling_pathway
Adult
FLOT1
SORBS1
SH2B2
CBL
CRK
RAPGEF1
RHOQ
EXOC7
TRIP10
GLUT4 vesicle
translocation to PM
SLC2A4
Glucose
Glucose uptake
Glucose homeostasis
INPPL1
INPP5A
SOCS4
PTPN1
MAPK8
IKBKB
PRKCI
SREBF1
PRKAG2
ACACA
FASN
Lipogenesis
Fatty acid biosynthesis (path 1)
PKLR
GCK
Glycolysis
PPARGC1A
G6PC
FBP1
PCK1
Glycolysis / Gluconeogenesis
FOXO1
DNA
INS
INSR
IRS1
PIK3CA
PIP3
PDPK1
AKT3
GSK3B
GYS1
Glycogen
Glycogenesis
Starch and sucrose metabolism
PPP1CA
CALML6
PYGB
TSC2
TSC1
RHEB
PDE3B
cAMP
PRKACA
LIPE
Antilipolysis
Lipid homeostasis
BAD
Antiapoptosis
Apoptosis
PTPRF
Phosphatidyl inositol signaling system
+ps : serine phosphorylation
+py : tyrosine phosphorylation
-py : tyrosine dephosphorylation
MTOR
RPTOR
RPS6KB1
RPS6
EIF4EBP1
EIF4E
Protein synthesis
MAPK signaling pathway
SHC2
GRB2
SOS1
HRAS
ARAF
MAP2K1
MAPK1
MKNK2
ELK1
Proliferation, differentiation
04910 3/12/20
(c) Kanehisa Laboratories

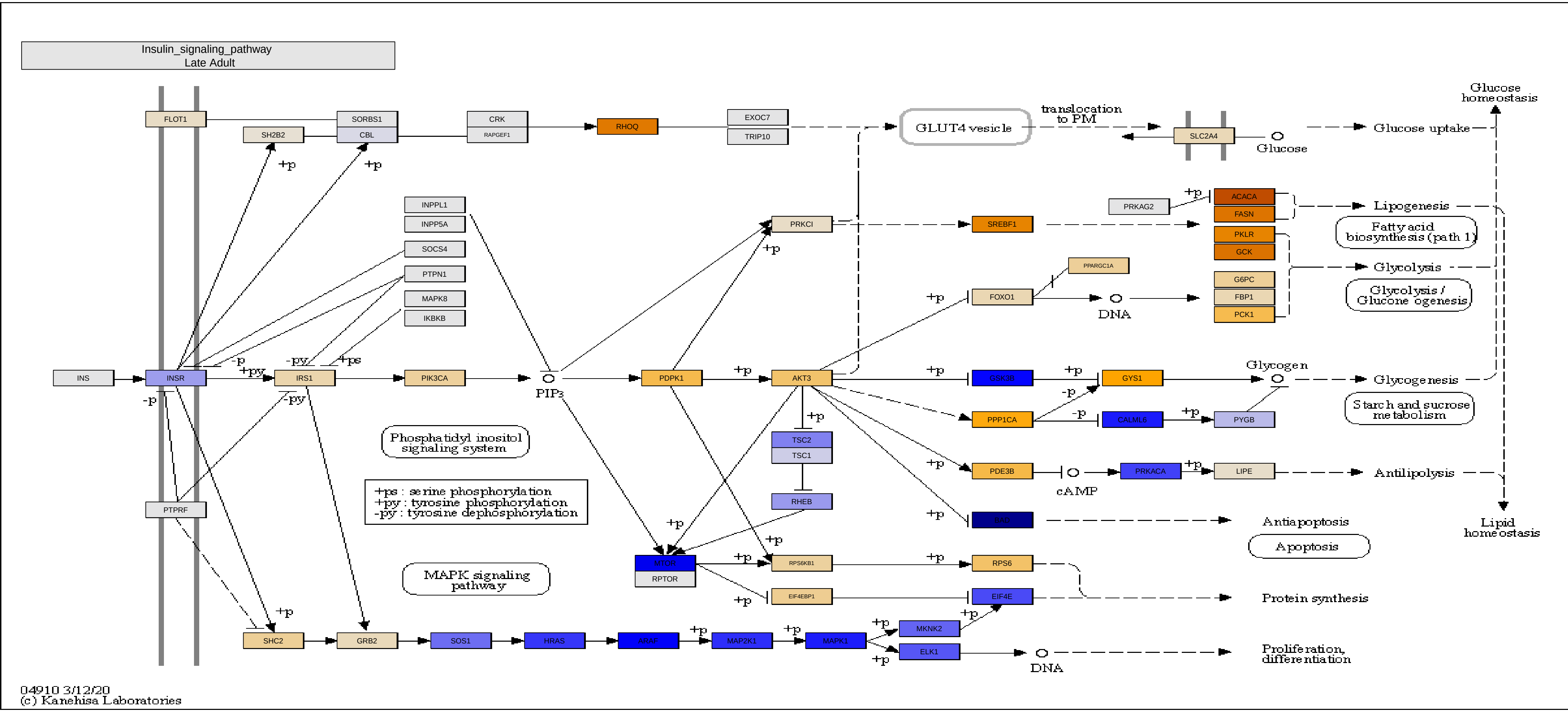
Insulin_signaling_pathway
Late Adult
FLOT1
SORBS1
CBL
SH2B2
CRK
RAPGEF1
RHOQ
EXOC7
TRIP10
GLUT4 vesicle
translocation to PM
SLC2A4
Glucose
Glucose uptake
Glucose homeostasis
INPPL1
INPP5A
SOCS4
PTPN1
MAPK8
IKBKB
PRKCI
SREBF1
PRKAG2
ACACA
FASN
PKLR
GCK
Lipogenesis
Fatty acid biosynthesis (path 1)
Glycolysis
PPARGC1A
FOXO1
DNA
G6PC
FBP1
PCK1
Glycolysis / Gluconeogenesis
INS
INSR
IRS1
PIK3CA
PIP3
PDPK1
AKT3
GSK3B
GYS1
Glycogen
Glycogenesis
PPP1CA
CALML6
PYGB
Starch and sucrose metabolism
TSC2
TSC1
RHEB
PDE3B
cAMP
PRKACA
LIPE
Antilipolysis
Lipid homeostasis
BAD
Antiapoptosis
Apoptosis
Phosphatidyl inositol signaling system
+ps : serine phosphorylation
+py : tyrosine phosphorylation
-py : tyrosine dephosphorylation
PTPRF
MTOR
RPTOR
RPS6KB1
RPS6
EIF4EBP1
EIF4E
Protein synthesis
MAPK signaling pathway
SHC2
GRB2
SOS1
HRAS
ARAF
MAP2K1
MAPK1
MKNK2
ELK1
Proliferation, differentiation
+p
-p
+py
-py
+ps
04910 3/12/20
(c) Kanehisa Laboratories
