# Supplementary material for: The Transcriptome and Methylome of the Developing and Aging Brain and Their Relations to Gliomas and Psychological Disorders
Source: Cells. 2022 Jan 21;11(3):362. doi: 10.3390/cells11030362 (PMC8834030; doi:10.3390/cells11030362)
Supplement: Supplementary file 1 [file cells-11-00362-s001.zip › Suppl-Material-S3-Pathways-PSF_Expression/JAK_STAT_signaling_pathway.pdf]

JAK−STAT signaling pathway

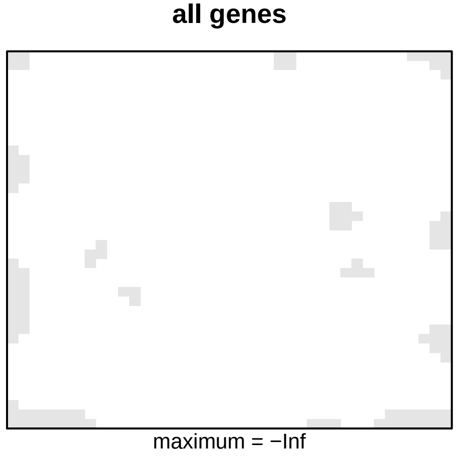

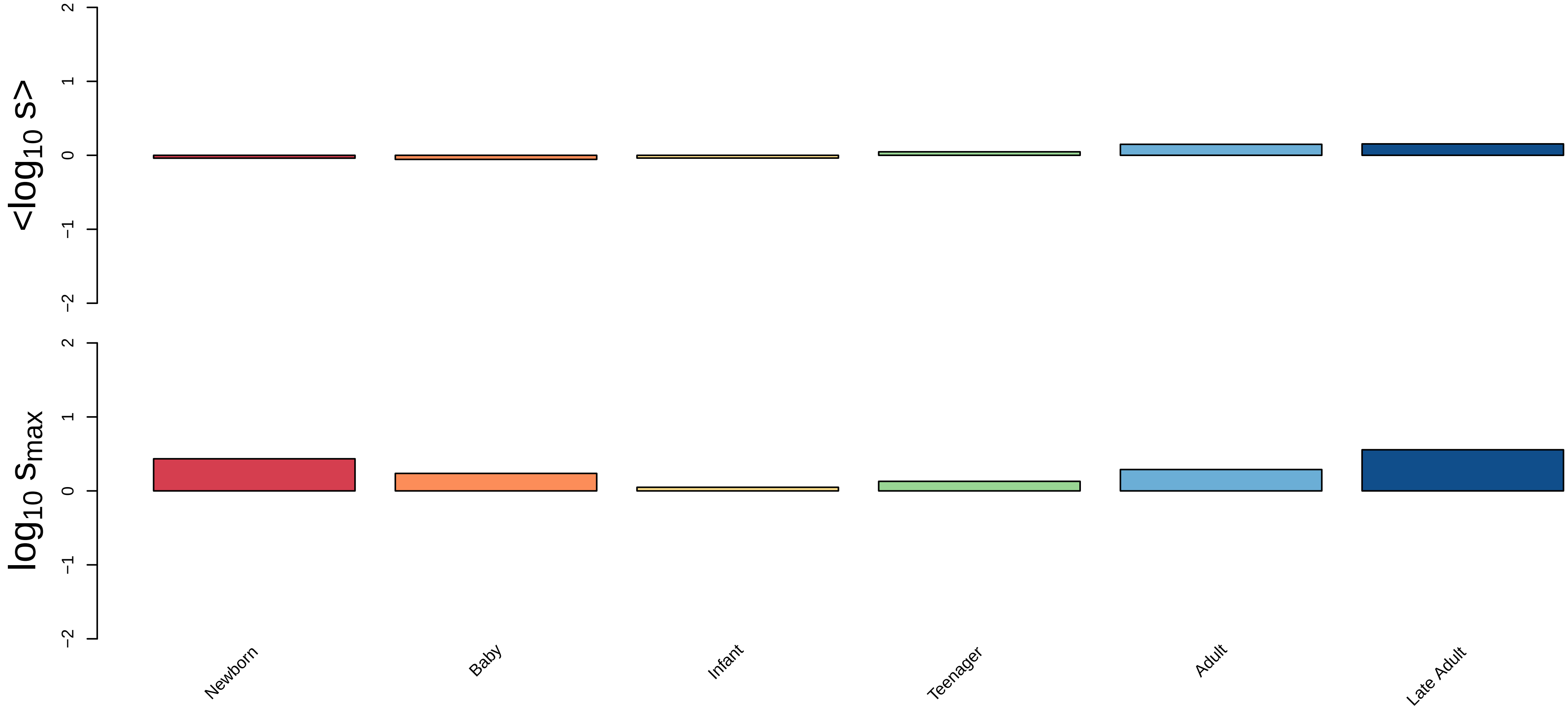

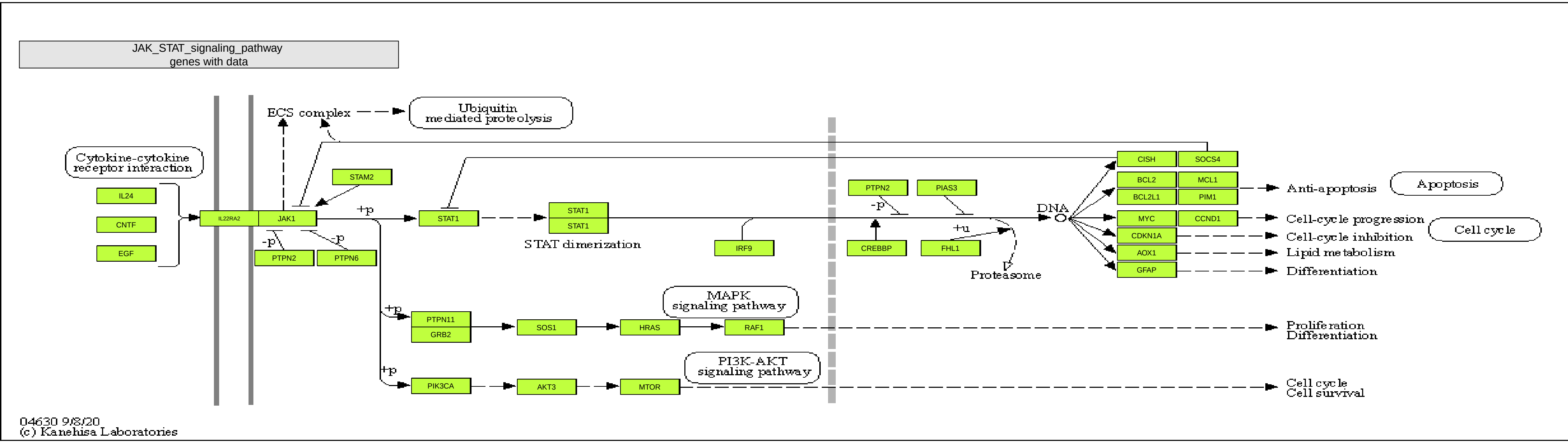
JAK_STAT_signaling_pathway
genes with data
ECS complex
Ubiquitin mediated proteolysis
Cytokine-cytokine receptor interaction
IL24
CNTF
EGF
IL22RA2
JAK1
STAM2
PTPN2
PTPN6
+p
-p
STAT1
STAT1
STAT1
STAT dimerization
IRF9
PTPN2
PIAS3
CREBBP
FHL1
+u
Proteasome
DNA
CISH
SOCS4
BCL2
MCL1
BCL2L1
PIM1
MYC
CCND1
CDKN1A
AOX1
GFAP
Anti-apoptosis
Apoptosis
Cell-cycle progression
Cell-cycle inhibition
Cell cycle
Lipid metabolism
Differentiation
PTPN11
GRB2
SOS1
HRAS
RAF1
MAPK signaling pathway
Proliferation
Differentiation
PIK3CA
AKT3
MTOR
PI3K-AKT signaling pathway
Cell cycle
Cell survival
04630 9/8/20
(c) Kanehisa Laboratories

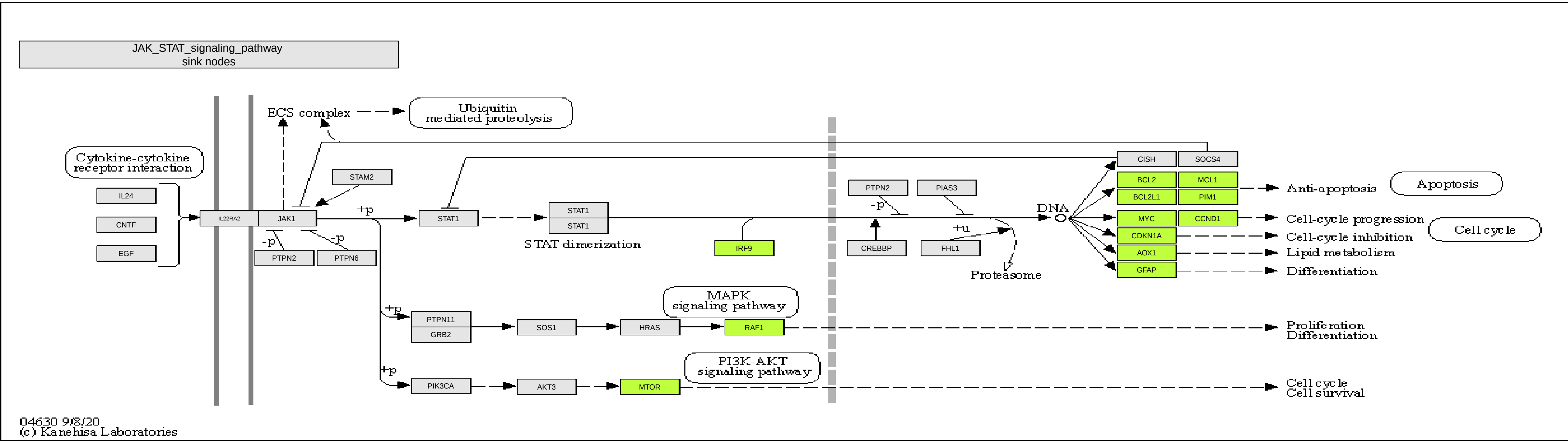
JAK_STAT_signaling_pathway
sink nodes
ECS complex
Ubiquitin mediated proteolysis
Cytokine-cytokine receptor interaction
IL24
CNTF
EGF
IL22RA2
JAK1
STAM2
PTPN2
PTPN6
+p
-p
STAT1
STAT1
STAT1
STAT dimerization
IRF9
PTPN2
PIAS3
CREBBP
FHL1
+u
Proteasome
DNA
CISH
SOCS4
BCL2
MCL1
BCL2L1
PIM1
MYC
CCND1
CDKN1A
AOX1
GFAP
Anti-apoptosis
Apoptosis
Cell-cycle progression
Cell cycle
Cell-cycle inhibition
Lipid metabolism
Differentiation
MAPK signaling pathway
PTPN11
GRB2
SOS1
HRAS
RAF1
Proliferation
Differentiation
PI3K-AKT signaling pathway
PIK3CA
AKT3
MTOR
Cell cycle
Cell survival
04630 9/8/20
(c) Kanehisa Laboratories

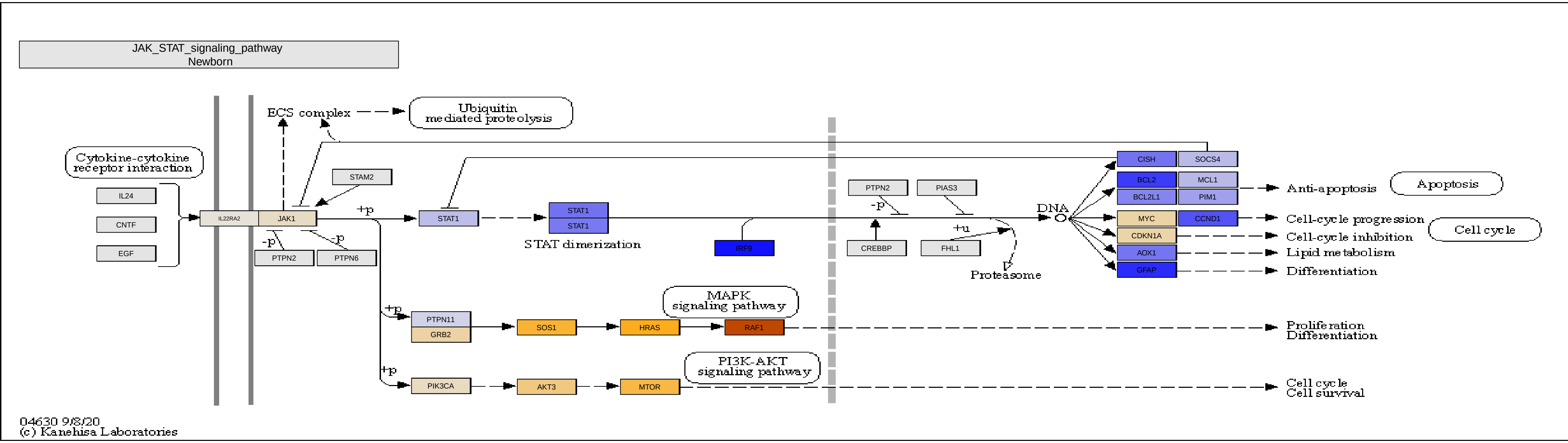

JAK_STAT_signaling_pathway
Newborn
Cytokine-cytokine receptor interaction
IL24
CNTF
EGF
IL22RA2
JAK1
ECS complex
Ubiquitin mediated proteolysis
STAM2
+p
-p
PTPN2
PTPN6
STAT1
STAT1
STAT1
STAT dimerization
IRF9
PTPN2
PIAS3
-P
CREBBP
FHL1
+u
Proteasome
DNA
CISH
SOCS4
BCL2
MCL1
BCL2L1
PIM1
MYC
CCND1
CDKN1A
AOX1
GFAP
Anti-apoptosis
Apoptosis
Cell-cycle progression
Cell-cycle inhibition
Cell cycle
Lipid metabolism
Differentiation
MAPK signaling pathway
PTPN11
GRB2
SOS1
HRAS
RAF1
Proliferation
Differentiation
PI3K-AKT signaling pathway
PIK3CA
AKT3
MTOR
Cell cycle
Cell survival
04630 9/8/20
(c) Kanehisa Laboratories

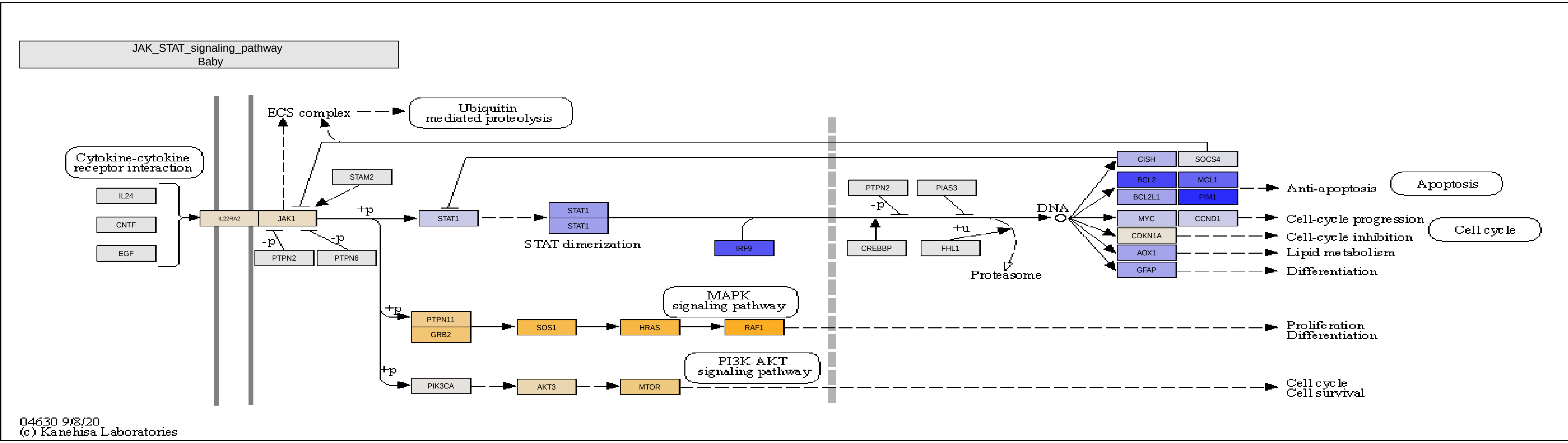
JAK_STAT_signaling_pathway
Baby
ECS complex
Ubiquitin mediated proteolysis
Cytokine-cytokine receptor interaction
IL24
CNTF
EGF
IL22RA2
JAK1
STAM2
-p
PTPN2
-p
PTPN6
+p
STAT1
STAT1
STAT1
STAT dimerization
IRF9
PTPN2
-p
PIAS3
+u
CREBBP
FHL1
Proteasome
DNA
CISH
SOCS4
BCL2
MCL1
BCL2L1
PIM1
MYC
CCND1
CDKN1A
AOX1
GFAP
Anti-apoptosis
Apoptosis
Cell-cycle progression
Cell-cycle inhibition
Cell cycle
Lipid metabolism
Differentiation
+p
PTPN11
GRB2
SOS1
HRAS
RAF1
MAPK signaling pathway
Proliferation
Differentiation
+p
PIK3CA
AKT3
MTOR
PI3K-AKT signaling pathway
Cell cycle
Cell survival
04630 9/8/20
(c) Kanehisa Laboratories

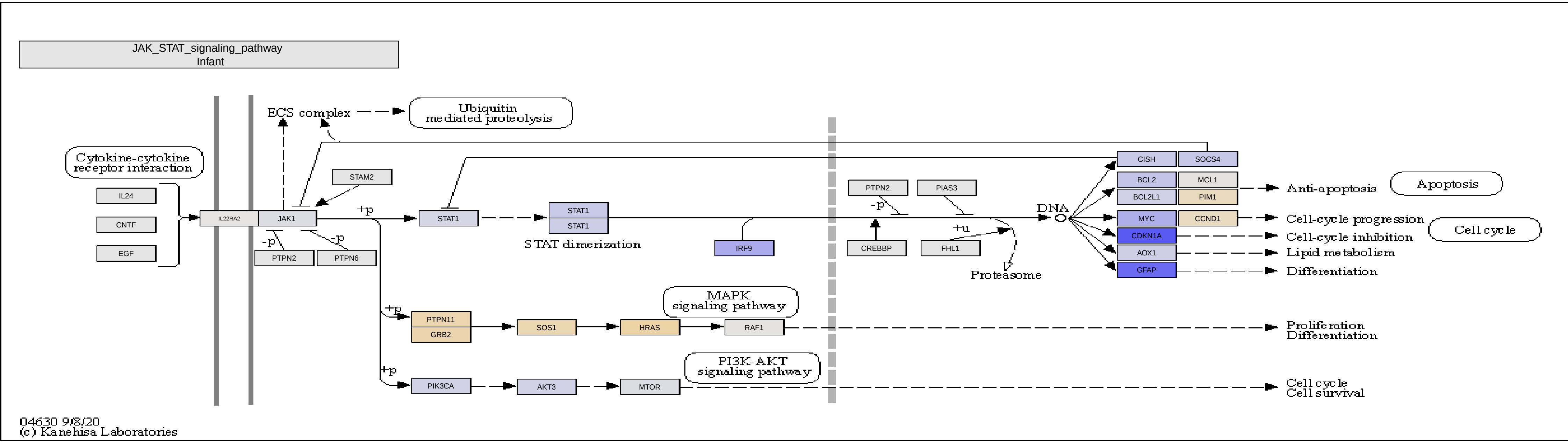

JAK_STAT_signaling_pathway
Infant
ECS complex
Ubiquitin mediated proteolysis
Cytokine-cytokine receptor interaction
IL24
CNTF
EGF
IL22RA2
JAK1
STAM2
+p
-p
PTPN2
PTPN6
STAT1
STAT dimerization
IRF9
PTPN2
PIAS3
CREBBP
FHL1
+u
Proteasome
DNA
CISH
SOCS4
BCL2
MCL1
BCL2L1
PIM1
MYC
CCND1
CDKN1A
AOX1
GFAP
Anti-apoptosis
Apoptosis
Cell-cycle progression
Cell cycle
Cell-cycle inhibition
Lipid metabolism
Differentiation
PTPN11
GRB2
SOS1
HRAS
RAF1
MAPK signaling pathway
Proliferation
Differentiation
PIK3CA
AKT3
MTOR
PI3K-AKT signaling pathway
Cell cycle
Cell survival
04630 9/8/20
(c) Kanehisa Laboratories

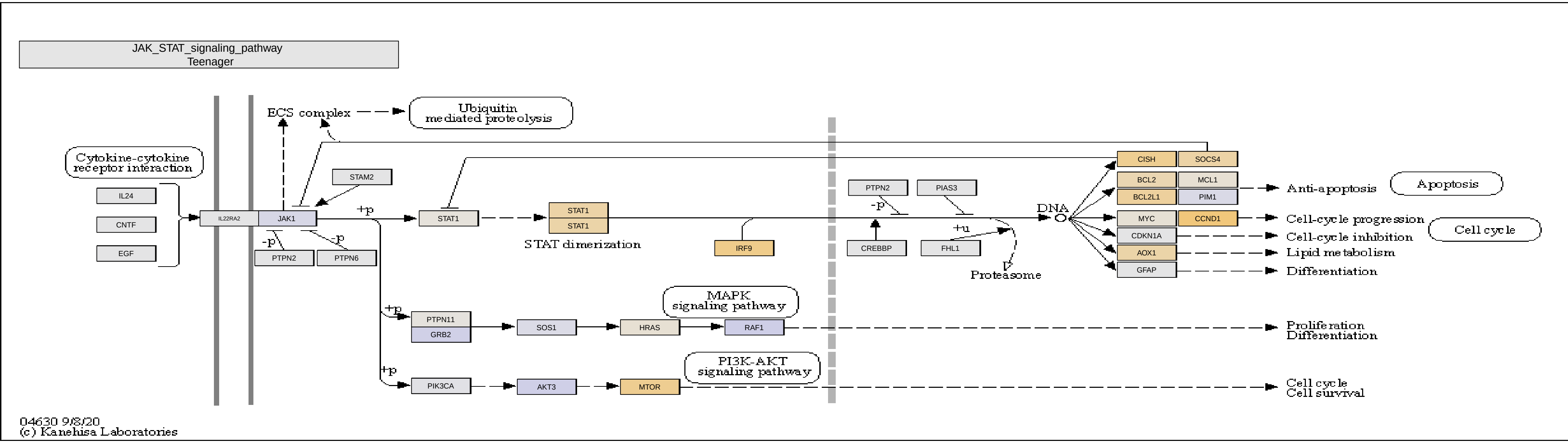
JAK_STAT_signaling_pathway
Teenager
ECS complex
Ubiquitin mediated proteolysis
Cytokine-cytokine receptor interaction
IL24
CNTF
EGF
IL22RA2
JAK1
STAM2
+p
-p
-p
PTPN2
PTPN6
STAT1
STAT1
STAT1
STAT dimerization
IRF9
PTPN2
PIAS3
-p
CREBBP
FHL1
+u
Proteasome
DNA
CISH
SOCS4
BCL2
MCL1
BCL2L1
PIM1
MYC
CCND1
CDKN1A
AOX1
GFAP
Anti-apoptosis
Apoptosis
Cell-cycle progression
Cell-cycle inhibition
Cell cycle
Lipid metabolism
Differentiation
+p
PTPN11
GRB2
SOS1
HRAS
RAF1
MAPK signaling pathway
Proliferation
Differentiation
+p
PIK3CA
AKT3
MTOR
PI3K-AKT signaling pathway
Cell cycle
Cell survival
04630 9/8/20
(c) Kanehisa Laboratories

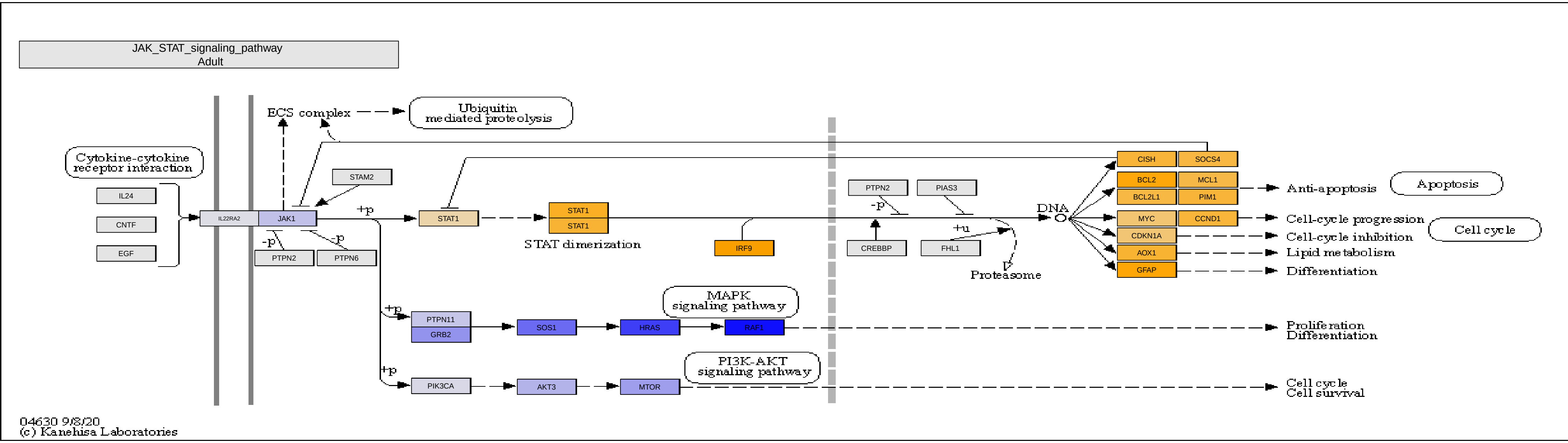
JAK_STAT_signaling_pathway
Adult
ECS complex
Ubiquitin mediated proteolysis
Cytokine-cytokine receptor interaction
IL24
CNTF
EGF
IL22RA2
JAK1
STAM2
+p
-p
-p
PTPN2
PTPN6
STAT1
STAT1
STAT1
STAT dimerization
IRF9
PTPN2
PIAS3
-p
CREBBP
FHL1
+u
Proteasome
DNA
CISH
SOCS4
BCL2
MCL1
BCL2L1
PIM1
MYC
CCND1
CDKN1A
AOX1
GFAP
Anti-apoptosis
Apoptosis
Cell-cycle progression
Cell-cycle inhibition
Cell cycle
Lipid metabolism
Differentiation
+p
PTPN11
GRB2
SOS1
HRAS
RAF1
MAPK signaling pathway
Proliferation
Differentiation
+p
PIK3CA
AKT3
MTOR
PI3K-AKT signaling pathway
Cell cycle
Cell survival
04630 9/8/20
(c) Kanehisa Laboratories

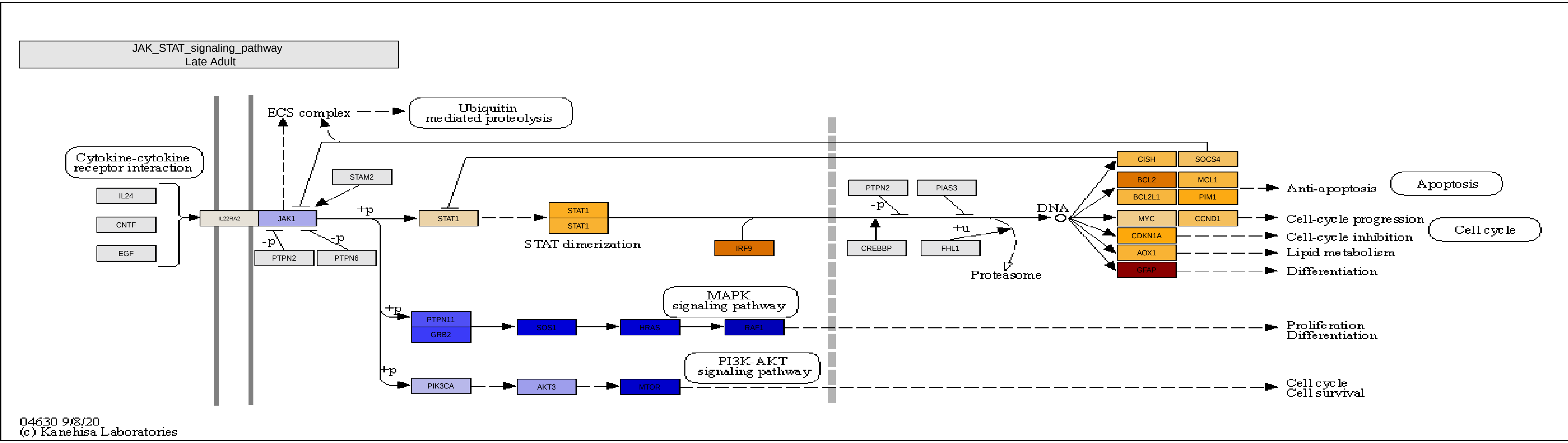
JAK_STAT_signaling_pathway
Late Adult
ECS complex
Ubiquitin mediated proteolysis
Cytokine-cytokine receptor interaction
IL24
CNTF
EGF
IL22RA2
JAK1
STAM2
+p
-p
-p
PTPN2
PTPN6
STAT1
STAT1
STAT1
STAT dimerization
IRF9
PTPN2
PIAS3
-p
+u
CREBBP
FHL1
Proteasome
DNA
CISH
SOCS4
BCL2
MCL1
BCL2L1
PIM1
MYC
CCND1
CDKN1A
AOX1
GFAP
Anti-apoptosis
Apoptosis
Cell-cycle progression
Cell-cycle inhibition
Cell cycle
Lipid metabolism
Differentiation
+p
PTPN11
GRB2
SOS1
HRAS
RAF1
MAPK signaling pathway
Proliferation
Differentiation
+p
PIK3CA
AKT3
MTOR
PI3K-AKT signaling pathway
Cell cycle
Cell survival
04630 9/8/20
(c) Kanehisa Laboratories
