# Supplementary material for: The Transcriptome and Methylome of the Developing and Aging Brain and Their Relations to Gliomas and Psychological Disorders
Source: Cells. 2022 Jan 21;11(3):362. doi: 10.3390/cells11030362 (PMC8834030; doi:10.3390/cells11030362)
Supplement: Supplementary file 1 [file cells-11-00362-s001.zip › Suppl-Material-S3-Pathways-PSF_Expression/mTOR_signaling_pathway.pdf]

# mTOR signaling pathway

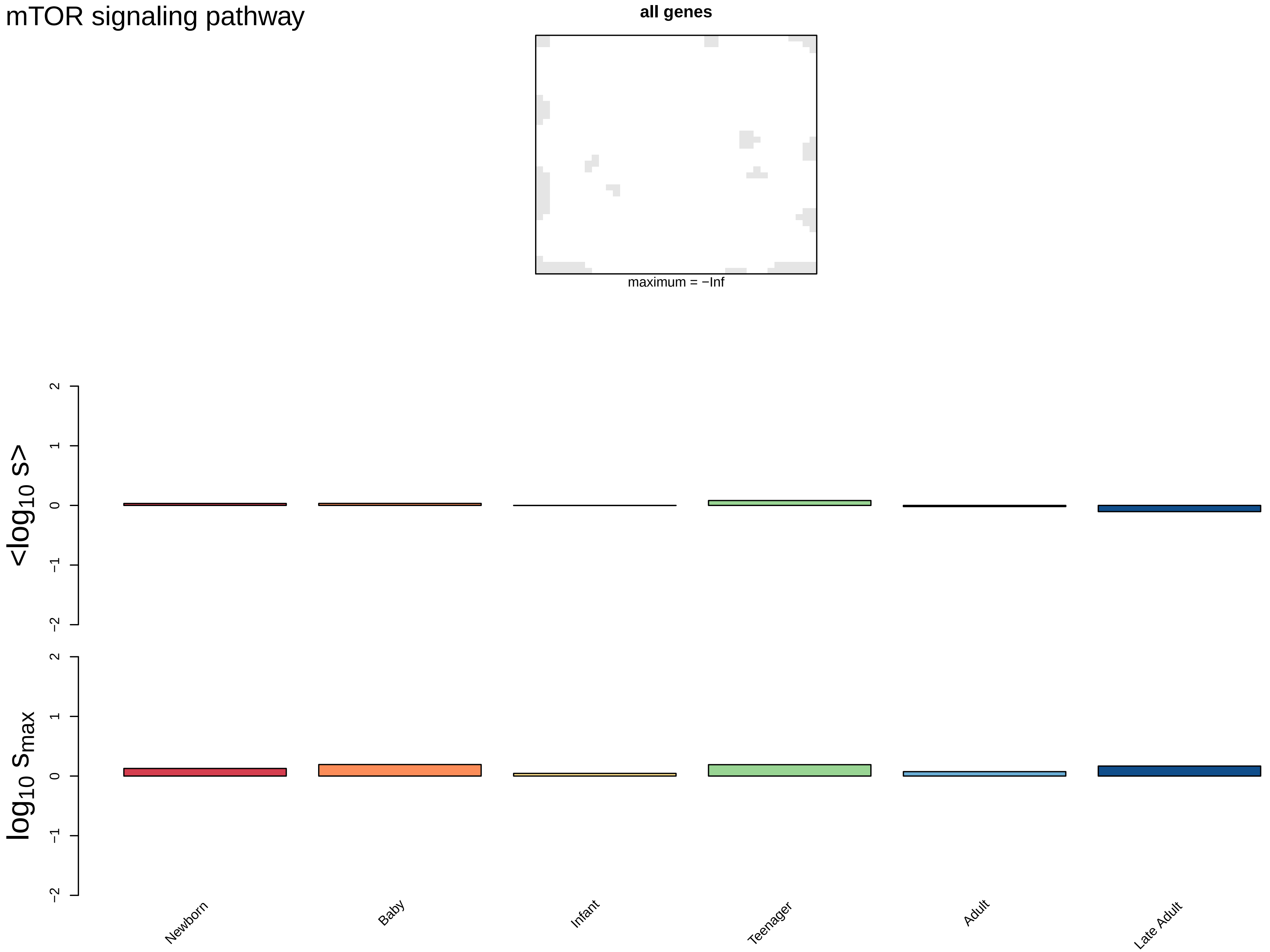

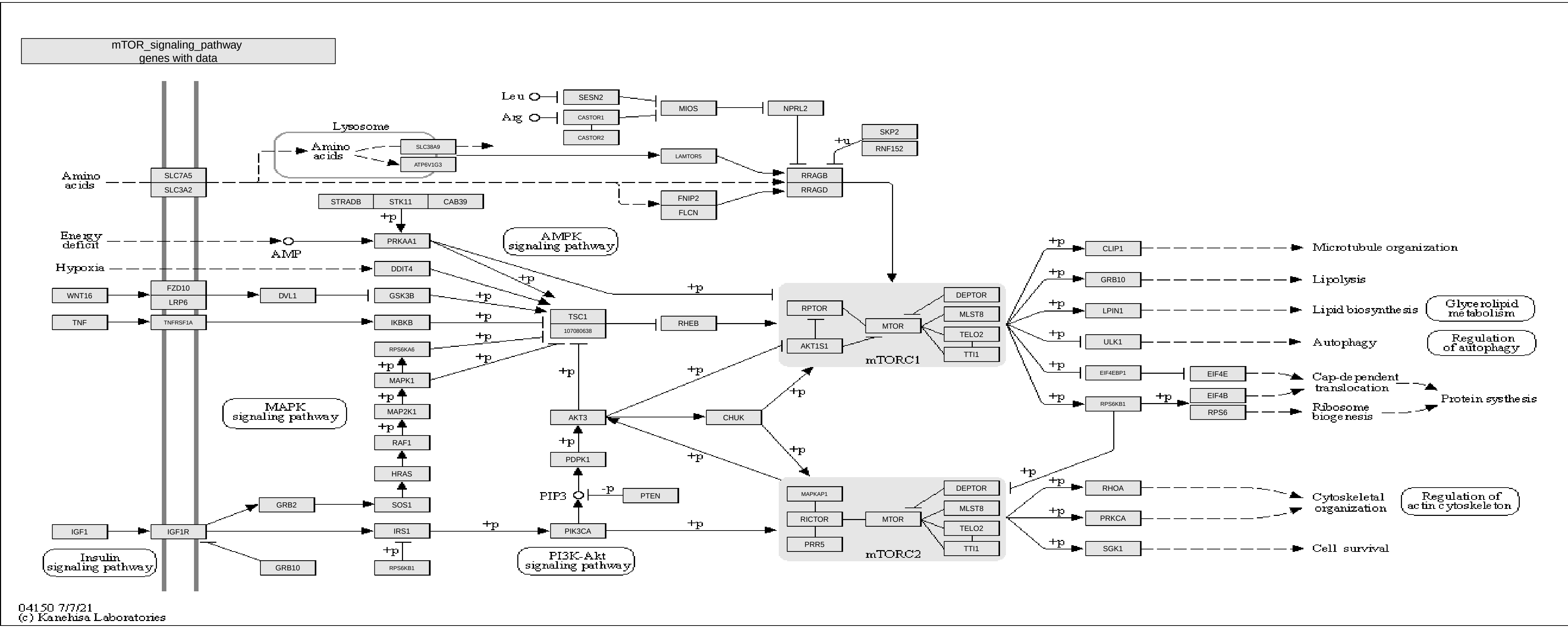
mTOR_signaling_pathway
genes with data
Leu
Arg
SESN2
CASTOR1
CASTOR2
MIOS
NPRL2
SKP2
RNF152
+u
Lysosome
Amino acids
SLC38A9
ATP6V1G3
LAMTOR5
RRAGB
RRAGD
Amino acids
SLC7A5
SLC3A2
FNIP2
FLCN
STRADB
STK11
CAB39
+p
Energy deficit
AMP
PRKAA1
AMPK signaling pathway
Hypoxia
DDIT4
WNT16
FZD10
LRP6
DVL1
GSK3B
TNF
TNFRSF1A
IKBKB
RPS6KA6
MAPK1
MAP2K1
RAF1
HRAS
SOS1
GRB2
MAPK signaling pathway
TSC1
107080638
RHEB
AKT3
PDPK1
PIP3
-p
PTEN
CHUK
PIK3CA
IRS1
IGF1
IGF1R
GRB10
RPS6KB1
Insulin signaling pathway
PI3K-Akt signaling pathway
RPTOR
AKT1S1
MTOR
DEPTOR
MLST8
TELO2
TTI1
mTORC1
MAPKAP1
RICTOR
PRR5
MTOR
DEPTOR
MLST8
TELO2
TTI1
mTORC2
CLIP1
GRB10
LPIN1
ULK1
EIF4EBP1
EIF4E
RPS6KB1
EIF4B
RPS6
RHOA
PRKCA
SGK1
Microtubule organization
Lipolysis
Lipid biosynthesis
Glycerolipid metabolism
Autophagy
Regulation of autophagy
Cap-dependent translocation
Ribosome biogenesis
Protein systhesis
Cytoskeletal organization
Regulation of actin cytoskeleton
Cell survival
04150 7/7/21
(c) Kanehisa Laboratories

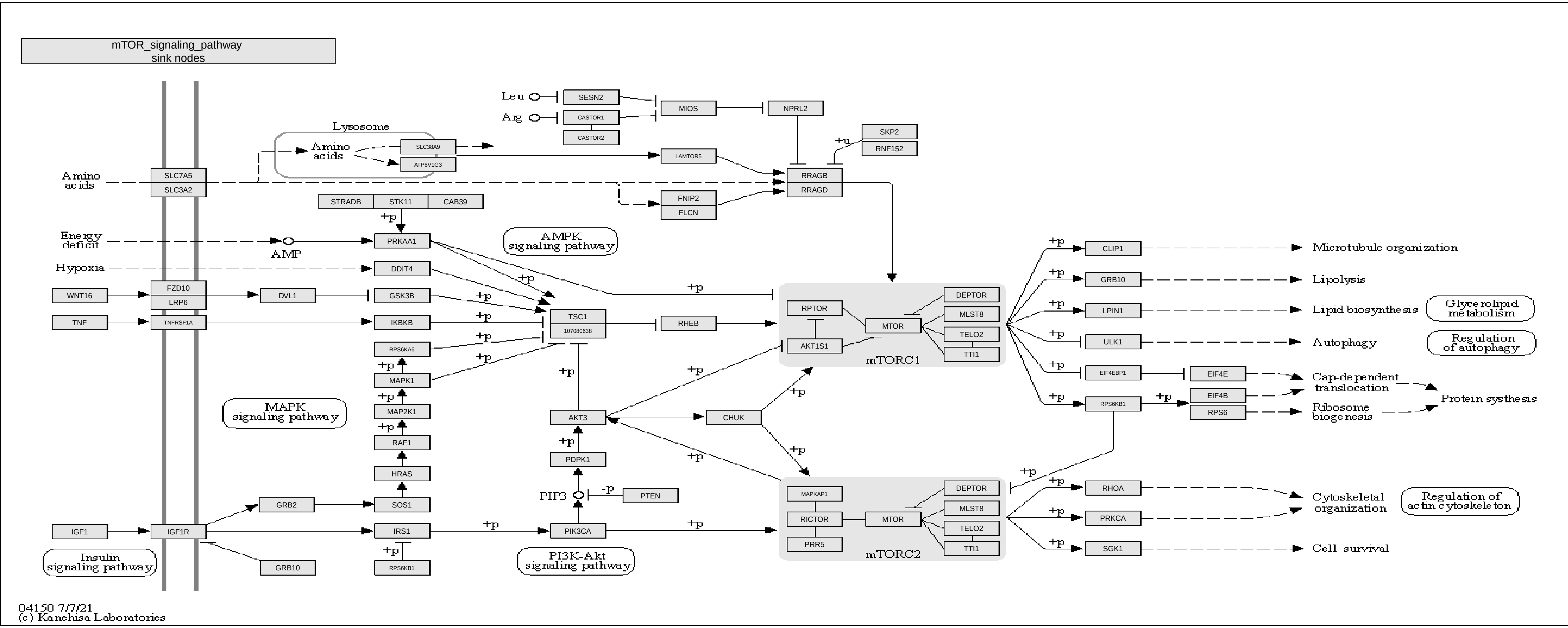
mTOR_signaling_pathway
sink nodes
Leu
Arg
SESN2
CASTOR1
CASTOR2
MIOS
NPRL2
SKP2
RNF152
+u
Lysosome
Amino acids
SLC38A9
ATP6V1G3
LAMTOR5
RRAGB
RRAGD
FNIP2
FLCN
Amino acids
SLC7A5
SLC3A2
STRADB
STK11
CAB39
+p
Energy deficit
AMP
PRKAA1
AMPK signaling pathway
Hypoxia
DDIT4
WNT16
FZD10
LRP6
DVL1
GSK3B
TNF
TNFRSF1A
IKBKB
RPS6KA6
MAPK1
MAP2K1
RAF1
HRAS
SOS1
GRB2
MAPK signaling pathway
TSC1
107080638
RHEB
AKT3
PDPK1
PIP3
-p
PTEN
CHUK
PIK3CA
IRS1
RPS6KB1
IGF1
IGF1R
GRB10
Insulin signaling pathway
PI3K-Akt signaling pathway
RPTOR
AKT1S1
MTOR
DEPTOR
MLST8
TELO2
TTI1
mTORC1
MAPKAP1
RICTOR
PRR5
mTORC2
CLIP1
GRB10
LPIN1
ULK1
EIF4EBP1
EIF4E
EIF4B
RPS6
RPS6KB1
RHOA
PRKCA
SGK1
Microtubule organization
Lipolysis
Lipid biosynthesis
Glycerolipid metabolism
Autophagy
Regulation of autophagy
Cap-dependent translocation
Protein systhesis
Ribosome biogenesis
Cytoskeletal organization
Regulation of actin cytoskeleton
Cell survival
04150 7/7/21
(c) Kanehisa Laboratories

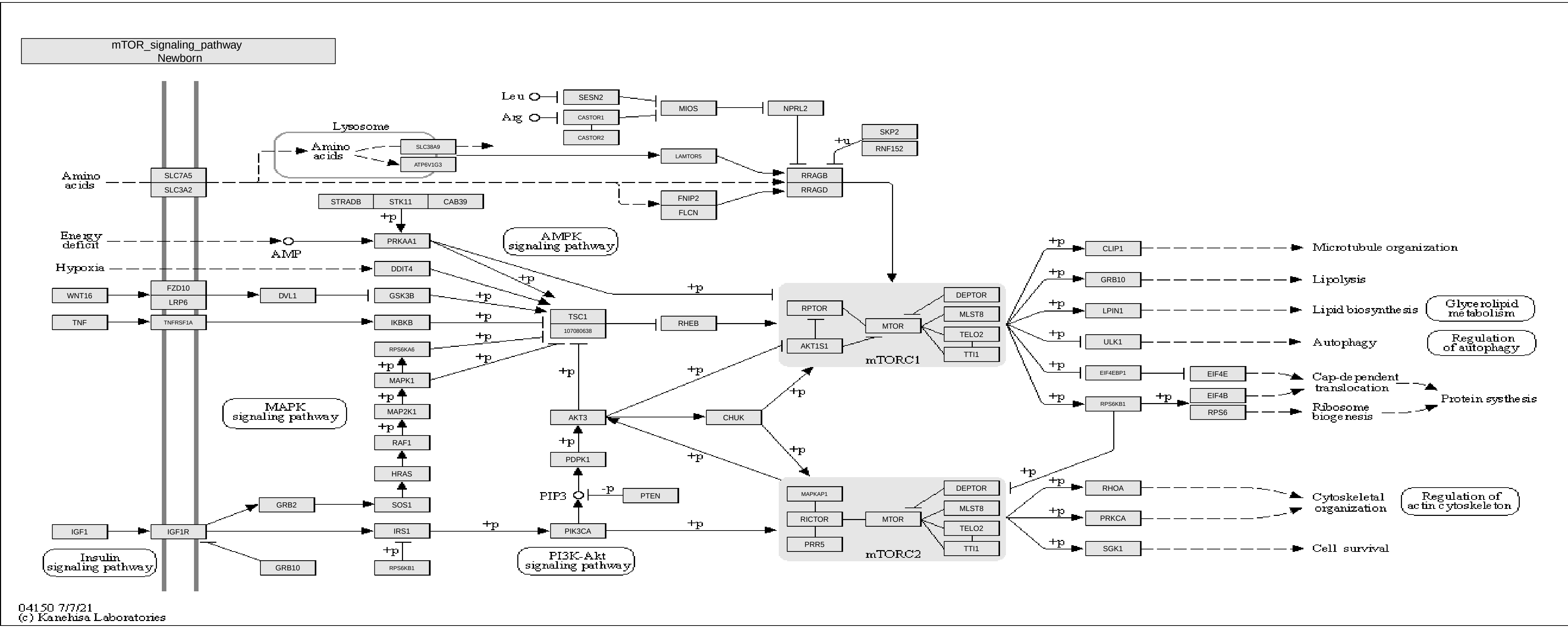

mTOR_signaling_pathway
Newborn
Leu
Arg
SESN2
CASTOR1
CASTOR2
MIOS
NPRL2
Lysosome
Amino acids
SLC38A9
ATP6V1G3
LAMTOR5
SKP2
RNF152
+u
Amino acids
SLC7A5
SLC3A2
RRAGB
RRAGD
FNIP2
FLCN
STRADB
STK11
CAB39
+p
Energy deficit
AMP
PRKAA1
AMPK signaling pathway
Hypoxia
DDIT4
WNT16
FZD10
LRP6
DVL1
GSK3B
TNF
TNFRSF1A
IKBKB
TSC1
107080638
RHEB
RPS6KA6
MAPK1
MAP2K1
RAF1
HRAS
SOS1
GRB2
MAPK signaling pathway
AKT3
PDPK1
PIP3
-p
PTEN
PIK3CA
CHUK
IGF1
IGF1R
IRS1
GRB10
RPS6KB1
Insulin signaling pathway
PI3K-Akt signaling pathway
RPTOR
AKT1S1
MTOR
DEPTOR
MLST8
TELO2
TTI1
mTORC1
MAPKAP1
RICTOR
PRR5
mTORC2
CLIP1
Microtubule organization
GRB10
Lipolysis
LPIN1
Lipid biosynthesis
Glycerolipid metabolism
ULK1
Autophagy
Regulation of autophagy
EIF4EBP1
EIF4E
Cap-dependent translocation
RPS6KB1
EIF4B
RPS6
Ribosome biogenesis
Protein systhesis
RHOA
PRKCA
Cytoskeletal organization
Regulation of actin cytoskeleton
SGK1
Cell survival
04150 7/7/21
(c) Kanehisa Laboratories

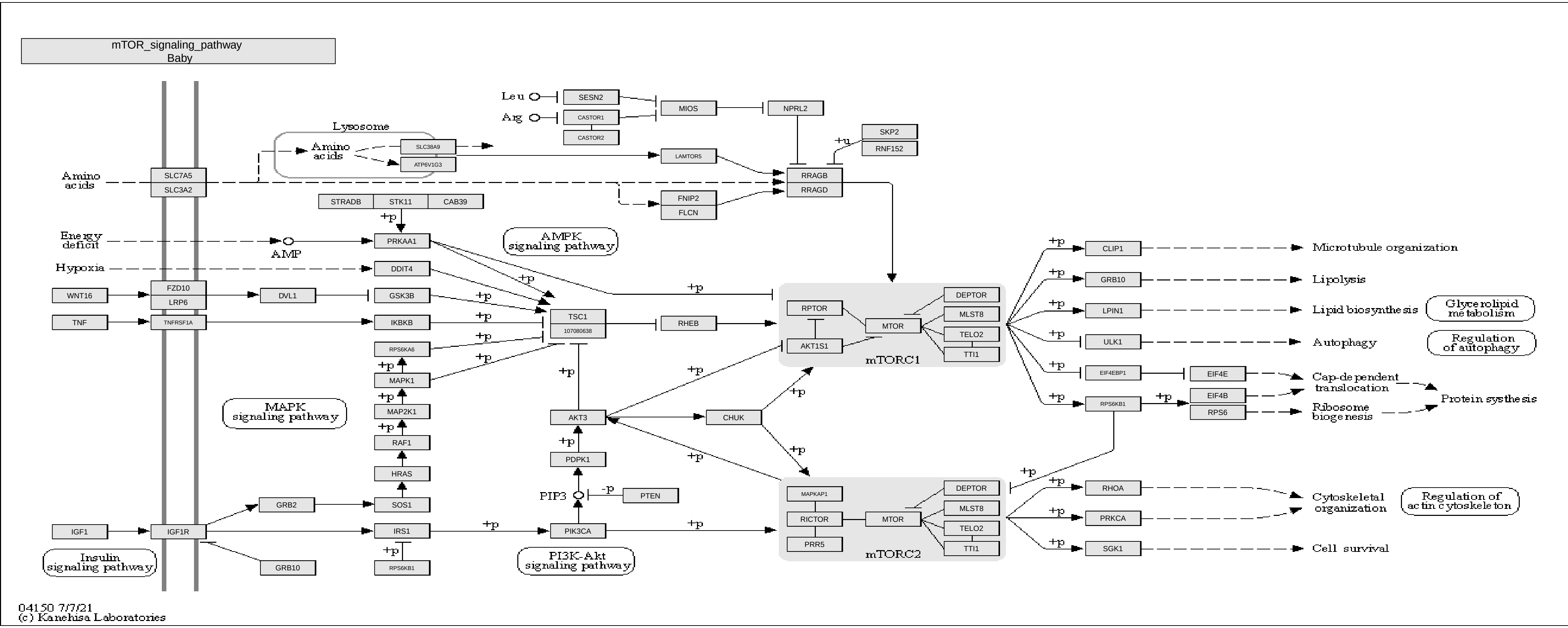
mTOR_signaling_pathway
Baby
Leu
Arg
SESN2
CASTOR1
CASTOR2
MIOS
NPRL2
SKP2
RNF152
+u
Lysosome
Amino acids
SLC38A9
ATP6V1G3
LAMTOR5
RRAGB
RRAGD
FNIP2
FLCN
Amino acids
SLC7A5
SLC3A2
STRADB
STK11
CAB39
+p
Energy deficit
AMP
PRKAA1
AMPK signaling pathway
Hypoxia
DDIT4
WNT16
FZD10
LRP6
DVL1
GSK3B
TNF
TNFRSF1A
IKBKB
TSC1
107080638
RHEB
RPS6KA6
MAPK1
MAP2K1
RAF1
HRAS
SOS1
GRB2
MAPK signaling pathway
AKT3
CHUK
PDPK1
PIP3
-p
PTEN
PIK3CA
IRS1
IGF1
IGF1R
GRB10
RPS6KB1
Insulin signaling pathway
PI3K-Akt signaling pathway
RPTOR
AKT1S1
MTOR
DEPTOR
MLST8
TELO2
TTI1
mTORC1
MAPKAP1
RICTOR
PRR5
mTORC2
CLIP1
Microtubule organization
GRB10
Lipolysis
LPIN1
Lipid biosynthesis
Glycerolipid metabolism
ULK1
Autophagy
Regulation of autophagy
EIF4EBP1
EIF4E
Cap-dependent translocation
EIF4B
RPS6
RPS6KB1
Ribosome biogenesis
Protein systhesis
RHOA
PRKCA
SGK1
Cytoskeletal organization
Regulation of actin cytoskeleton
Cell survival
04150 7/7/21
(c) Kanehisa Laboratories

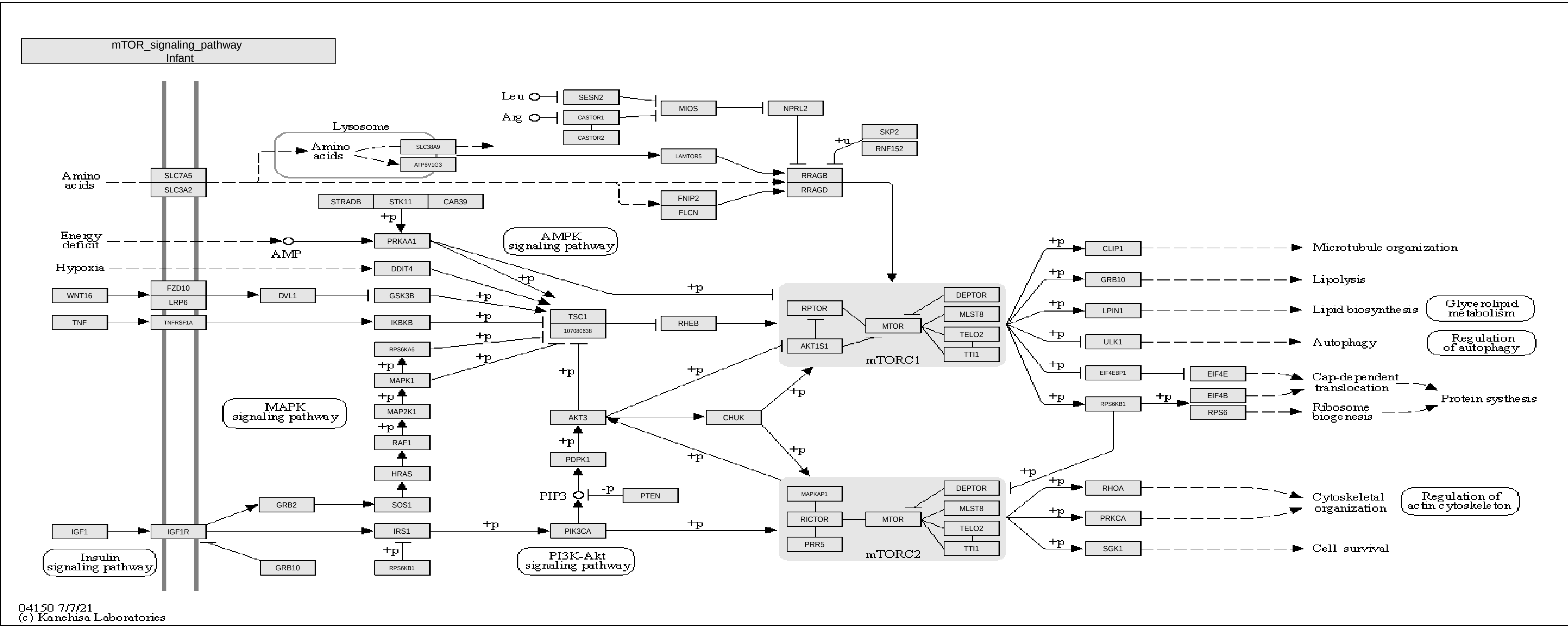
mTOR_signaling_pathway
Infant
Leu
Arg
SESN2
CASTOR1
CASTOR2
MIOS
NPRL2
SKP2
RNF152
+u
Lysosome
Amino acids
SLC38A9
ATP6V1G3
LAMTOR5
RRAGB
RRAGD
FNIP2
FLCN
Amino acids
SLC7A5
SLC3A2
STRADB
STK11
CAB39
Energy deficit
AMP
PRKAA1
AMPK signaling pathway
Hypoxia
DDIT4
WNT16
FZD10
LRP6
DVL1
GSK3B
TNF
TNFRSF1A
IKBKB
TSC1
107080638
RHEB
RPS6KA6
MAPK1
MAP2K1
RAF1
HRAS
SOS1
GRB2
MAPK signaling pathway
AKT3
PDPK1
PIP3
PTEN
CHUK
IGF1
IGF1R
IRS1
PIK3CA
GRB10
RPS6KB1
Insulin signaling pathway
PI3K-Akt signaling pathway
RPTOR
AKT1S1
MTOR
DEPTOR
MLST8
TELO2
TTI1
mTORC1
MAPKAP1
RICTOR
PRR5
MTOR
DEPTOR
MLST8
TELO2
TTI1
mTORC2
CLIP1
GRB10
LPIN1
ULK1
EIF4EBP1
EIF4E
RPS6KB1
EIF4B
RPS6
RHOA
PRKCA
SGK1
Microtubule organization
Lipolysis
Lipid biosynthesis
Glycerolipid metabolism
Autophagy
Regulation of autophagy
Cap-dependent translocation
Ribosome biogenesis
Protein systhesis
Cytoskeletal organization
Regulation of actin cytoskeleton
Cell survival
04150 7/7/21
(c) Kanehisa Laboratories

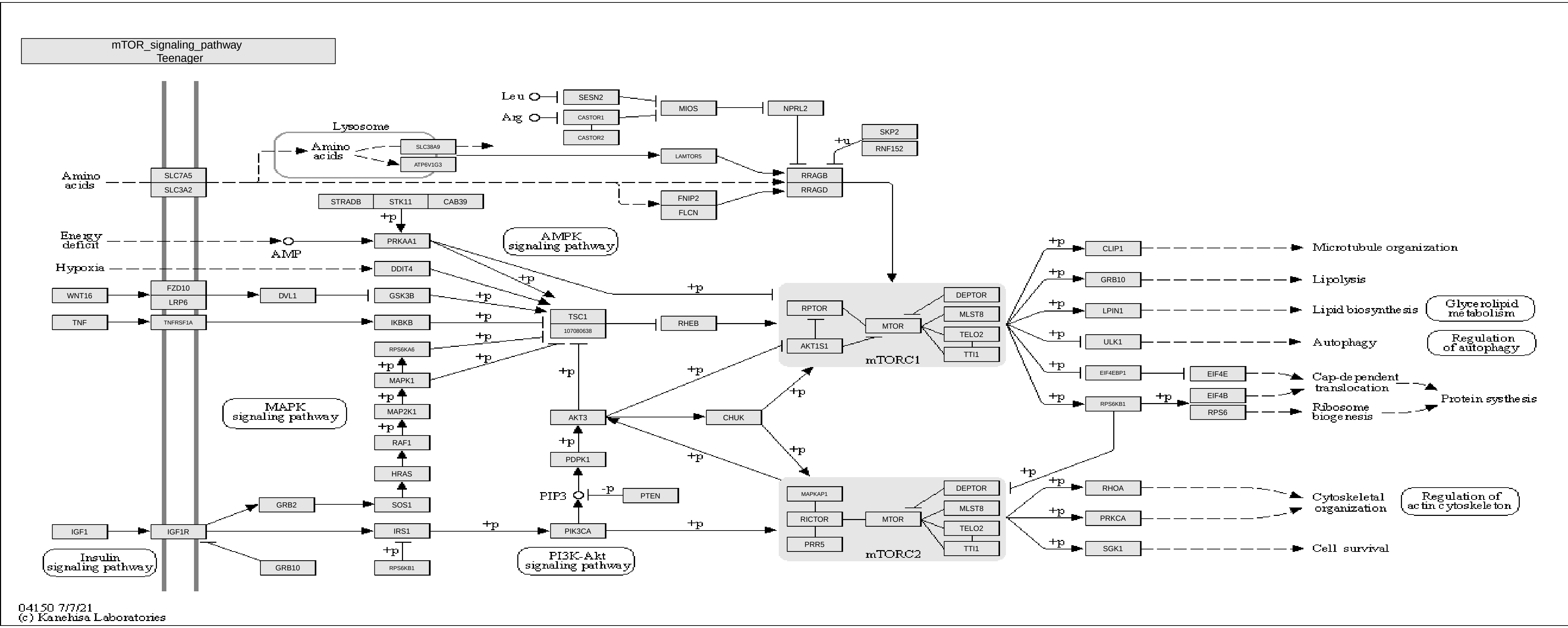

mTOR_signaling_pathway
Teenager
Leu
Arg
SESN2
CASTOR1
CASTOR2
MIOS
NPRL2
SKP2
RNF152
+u
Lysosome
Amino acids
SLC38A9
ATP6V1G3
LAMTOR5
Amino acids
SLC7A5
SLC3A2
RRAGB
RRAGD
FNIP2
FLCN
STRADB
STK11
CAB39
Energy deficit
AMP
PRKAA1
AMPK signaling pathway
Hypoxia
DDIT4
WNT16
FZD10
LRP6
DVL1
GSK3B
TNF
TNFRSF1A
IKBKB
TSC1
107080638
RHEB
RPS6KA6
MAPK1
MAP2K1
RAF1
HRAS
SOS1
GRB2
MAPK signaling pathway
AKT3
PDPK1
PIP3
PTEN
PIK3CA
IRS1
RPS6KB1
IGF1
IGF1R
GRB10
Insulin signaling pathway
PI3K-Akt signaling pathway
CHUK
RPTOR
AKT1S1
MTOR
DEPTOR
MLST8
TELO2
TTI1
mTORC1
MAPKAP1
RICTOR
PRR5
mTORC2
CLIP1
GRB10
LPIN1
ULK1
EIF4EBP1
EIF4E
RPS6KB1
EIF4B
RPS6
RHOA
PRKCA
SGK1
Microtubule organization
Lipolysis
Lipid biosynthesis
Glycerolipid metabolism
Autophagy
Regulation of autophagy
Cap-dependent translocation
Ribosome biogenesis
Protein systhesis
Cytoskeletal organization
Regulation of actin cytoskeleton
Cell survival
04150 7/7/21
(c) Kanehisa Laboratories

mTOR_signaling_pathway
Adult

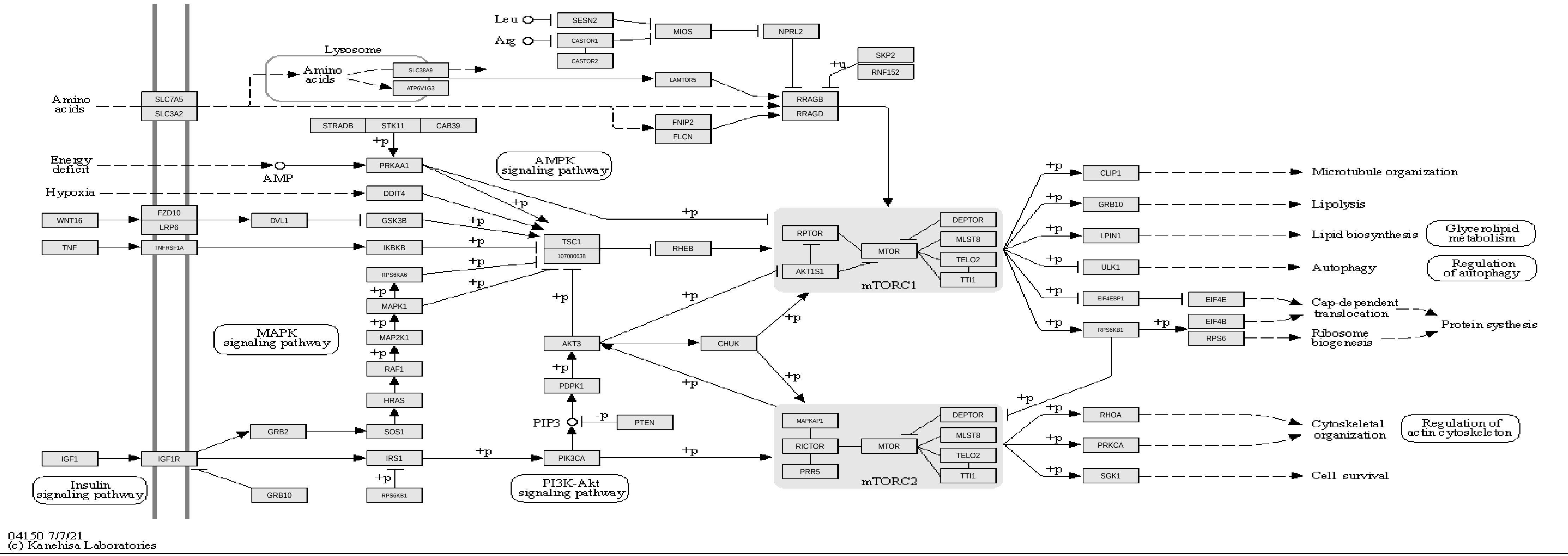

mTOR_signaling_pathway
Late Adult

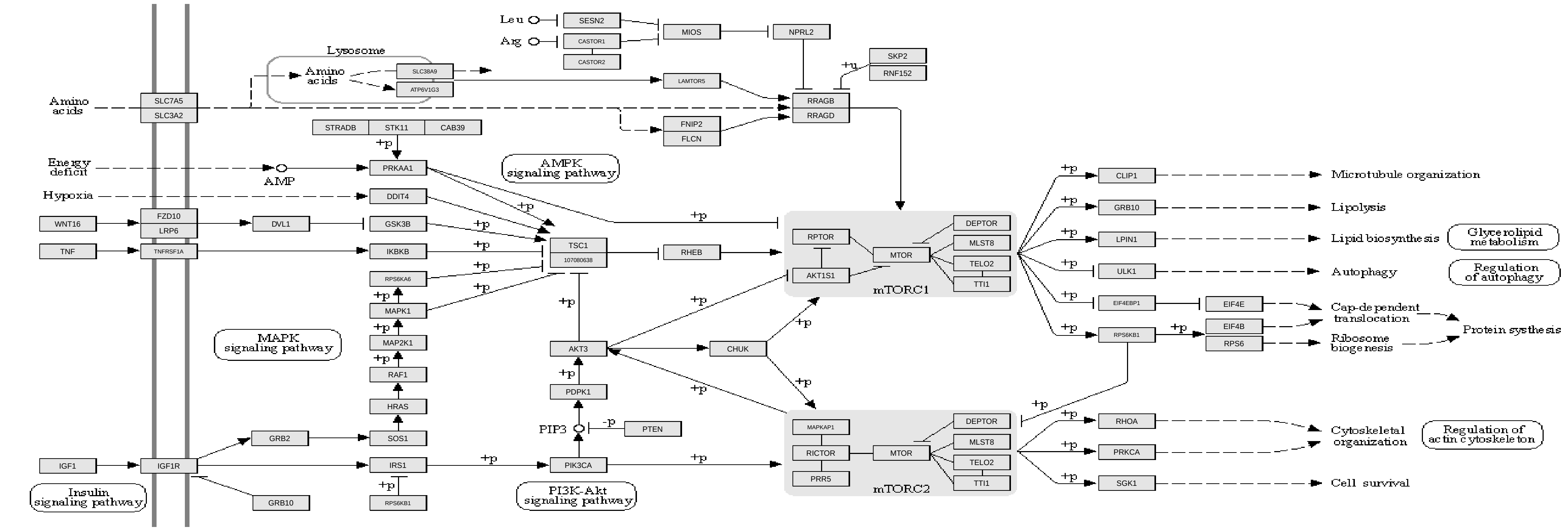


04150 7/7/21
(c) Kanehisa Laboratories
