# Supplementary material for: The Transcriptome and Methylome of the Developing and Aging Brain and Their Relations to Gliomas and Psychological Disorders
Source: Cells. 2022 Jan 21;11(3):362. doi: 10.3390/cells11030362 (PMC8834030; doi:10.3390/cells11030362)

NF−kappa B signaling pathway

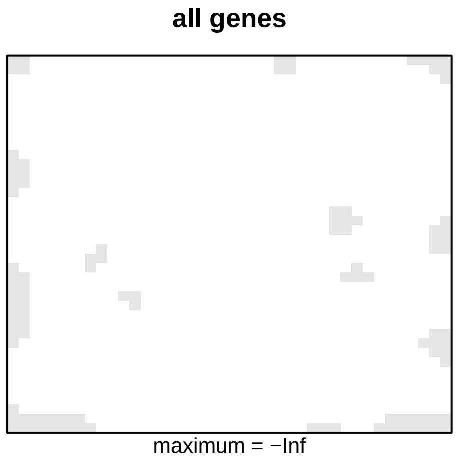

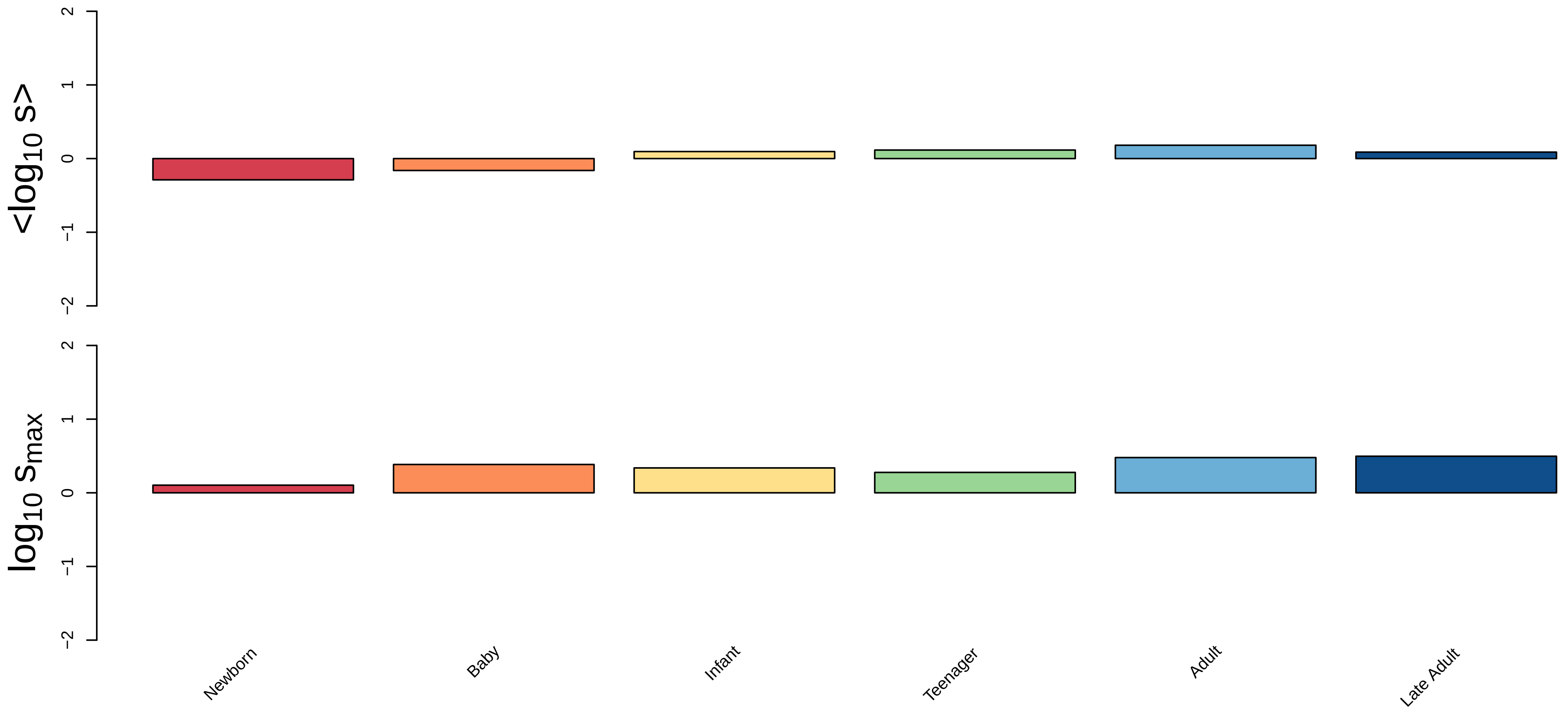

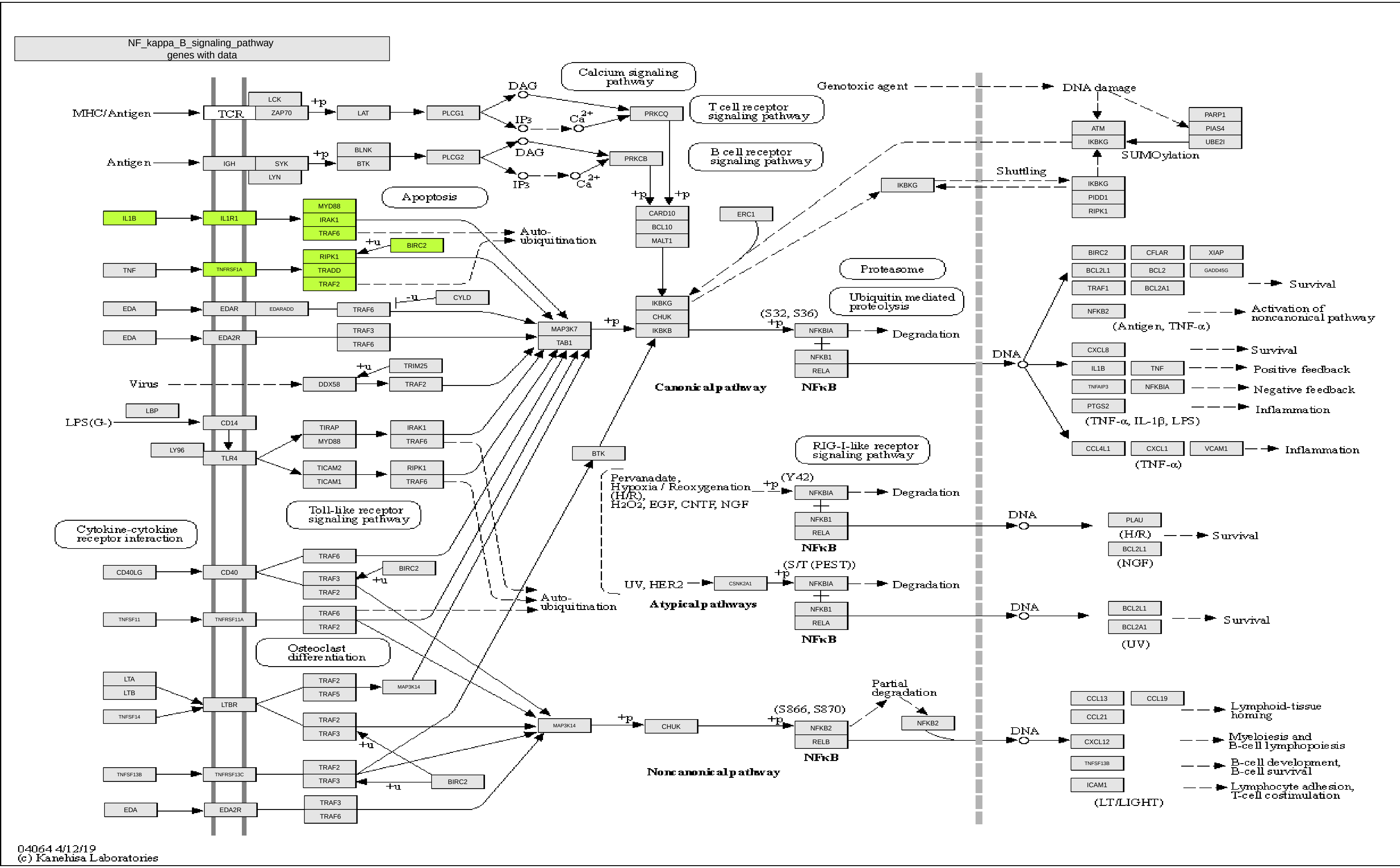
NF_kappa_B_signaling_pathway
genes with data
MHC/Antigen
TCR
LCK
ZAP70
+p
LAT
PLCG1
DAG
IP3
Ca2+
PRKCQ
Calcium signaling pathway
T cell receptor signaling pathway
B cell receptor signaling pathway
Antigen
IGH
SYK
LYN
BLNK
BTK
PLCG2
PRKCB
Genotoxic agent
DNA damage
PARP1
PIAS4
UBE2I
ATM
IKBKG
SUMOylation
Shuttling
PIDD1
RIPK1
IL1B
IL1R1
MYD88
IRAK1
TRAF6
Apoptosis
Auto-ubiquitination
+u
BIRC2
TNF
TNFRSF1A
TRADD
TRAF2
CARD10
BCL10
MALT1
ERC1
+p
EDA
EDAR
EDARADD
-u
CYLD
EDA2R
TRAF3
MAP3K7
TAB1
CHUK
IKBKB
Proteasome
Ubiquitin mediated proteolysis
(S32, S36)
NFKBIA
Degradation
NFKB1
RELA
NFκB
Canonical pathway
DNA
CFLAR
XIAP
BCL2L1
BCL2
GADD45G
TRAF1
BCL2A1
Survival
NFKB2
Activation of noncanonical pathway
(Antigen, TNF-α)
CXCL8
Positive feedback
TNFAIP3
Negative feedback
PTGS2
Inflammation
(TNF-α, IL-1β, LPS)
CCL4L1
CXCL1
VCAM1
(TNF-α)
Virus
DDX58
TRIM25
LPS(G-)
LBP
CD14
LY96
TLR4
TIRAP
TICAM2
TICAM1
BTK
RIG-I-like receptor signaling pathway
Pervanadate, Hypoxia / Reoxygenation (H/R), H2O2, EGF, CNTF, NGF
(Y42)
PLAU
(H/R)
(NGF)
Toll-like receptor signaling pathway
Cytokine-cytokine receptor interaction
CD40LG
CD40
UV, HER2
CSNK2A1
(S/T (PEST))
Atypical pathways
(UV)
TNFSF11
TNFRSF11A
Osteoclast differentiation
LTA
LTB
TNFSF14
LTBR
TRAF5
MAP3K14
Partial degradation
(S866, S870)
RELB
Noncanonical pathway
CCL13
CCL19
CCL21
CXCL12
TNFSF13B
ICAM1
(LT/LIGHT)
Lymphoid-tissue homing
Myeloiesis and B-cell lymphopoiesis
B-cell development, B-cell survival
Lymphocyte adhesion, T-cell costimulation
TNFRSF13C
04064 4/12/19
(c) Kanehisa Laboratories

NF_kappa_B_signaling_pathway
sink nodes

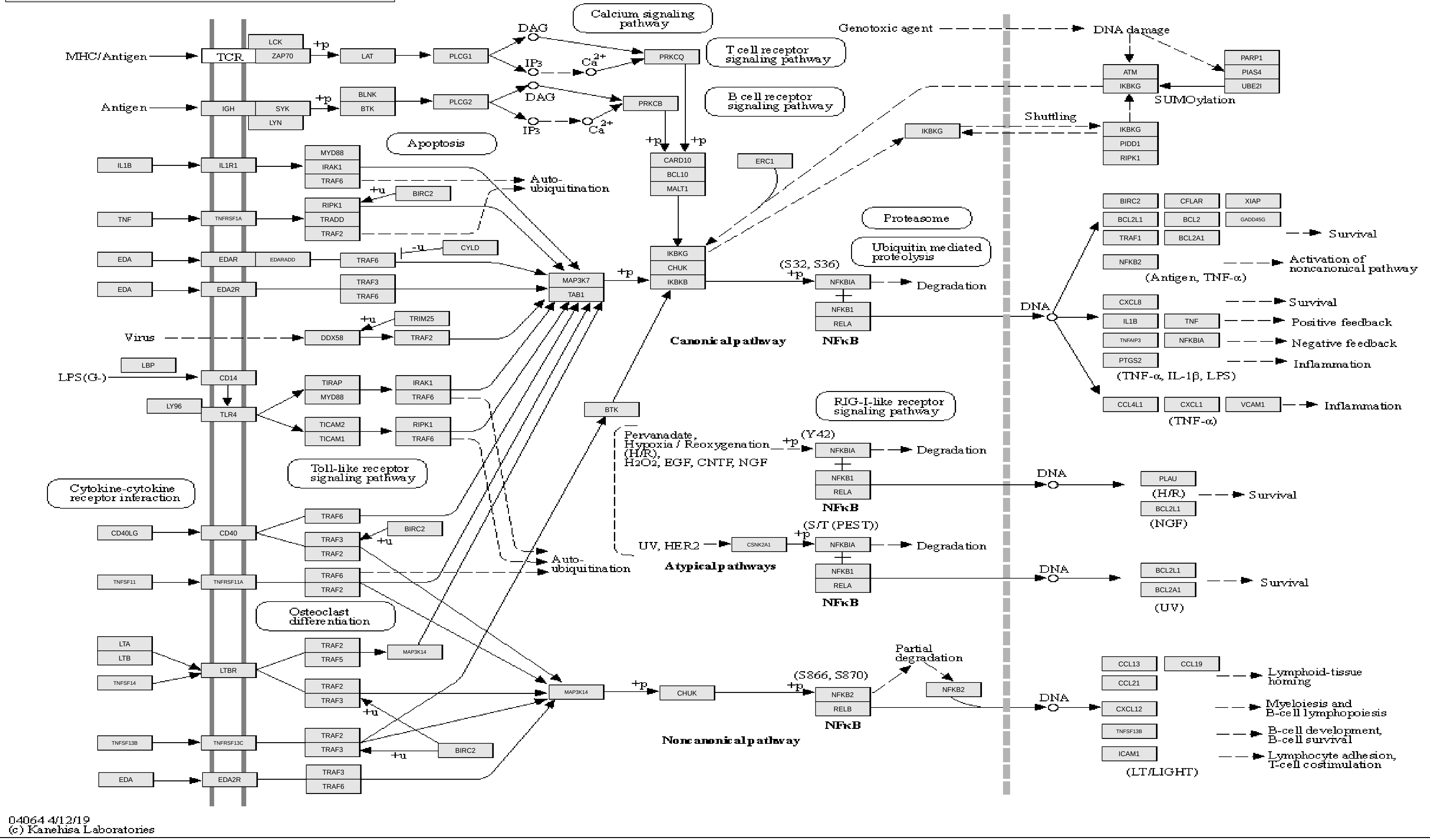

NF_kappa_B_signaling_pathway
Newborn

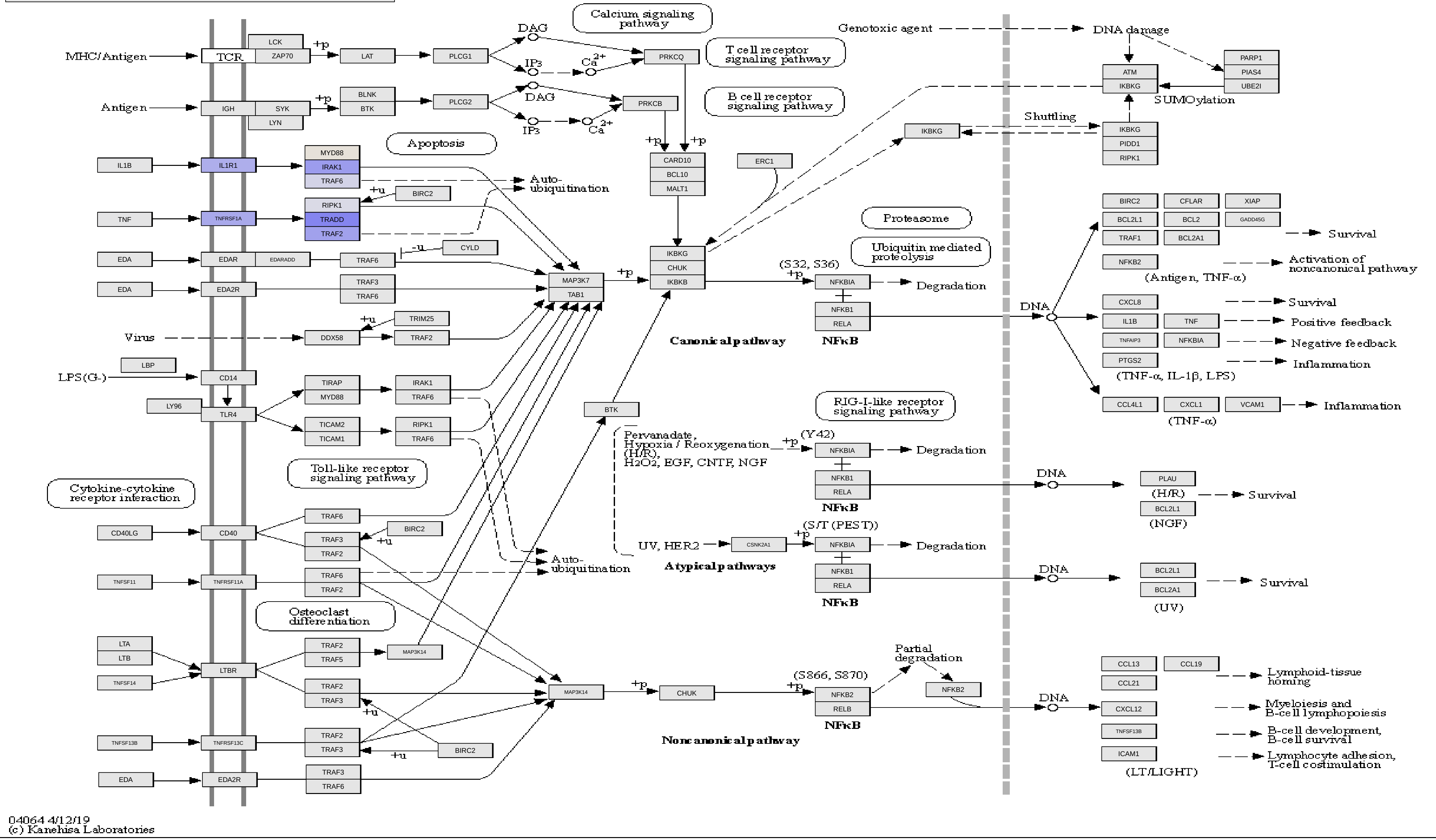

NF_kappa_B_signaling_pathway
Baby

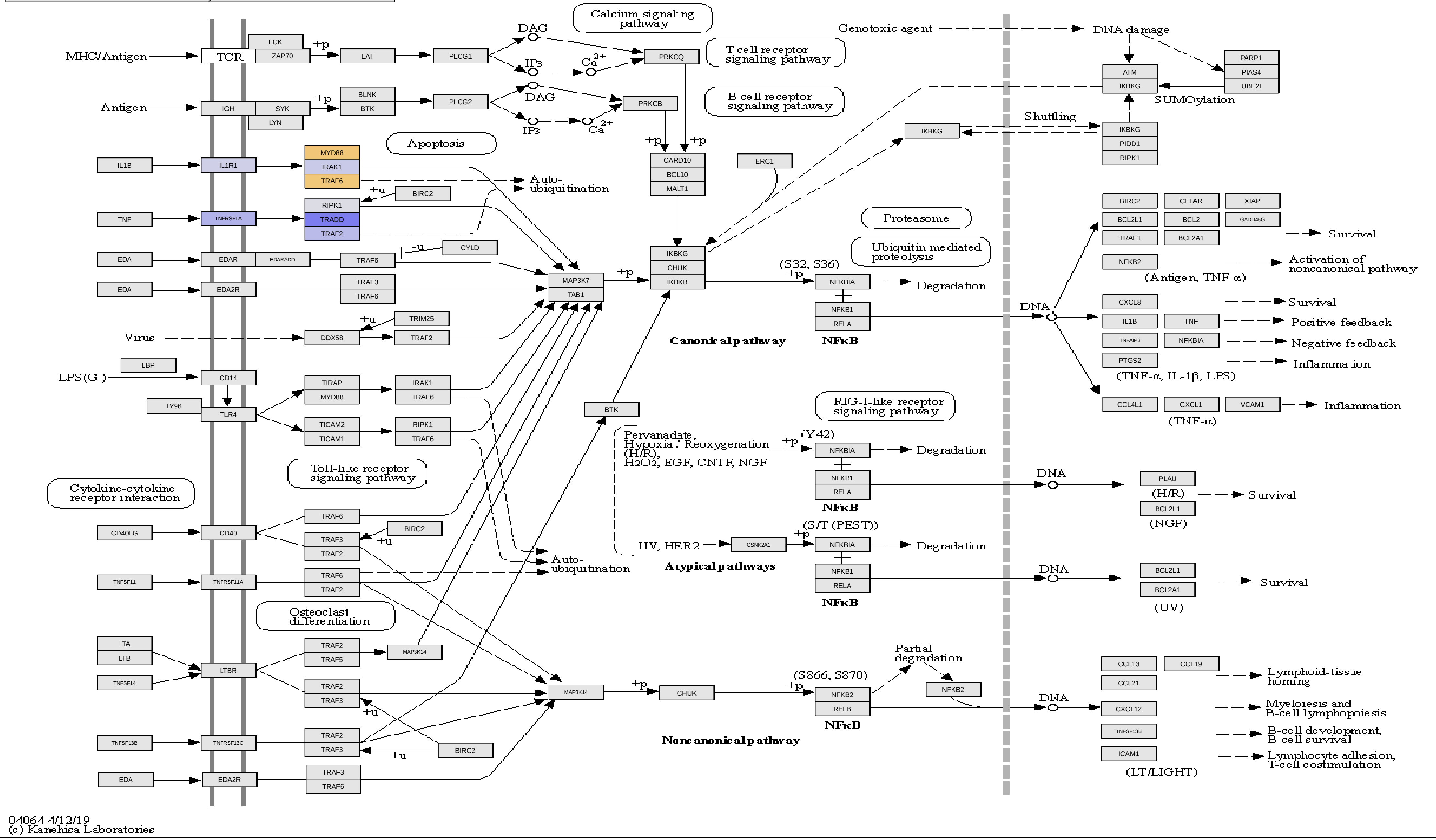

NF_kappa_B_signaling_pathway
Infant

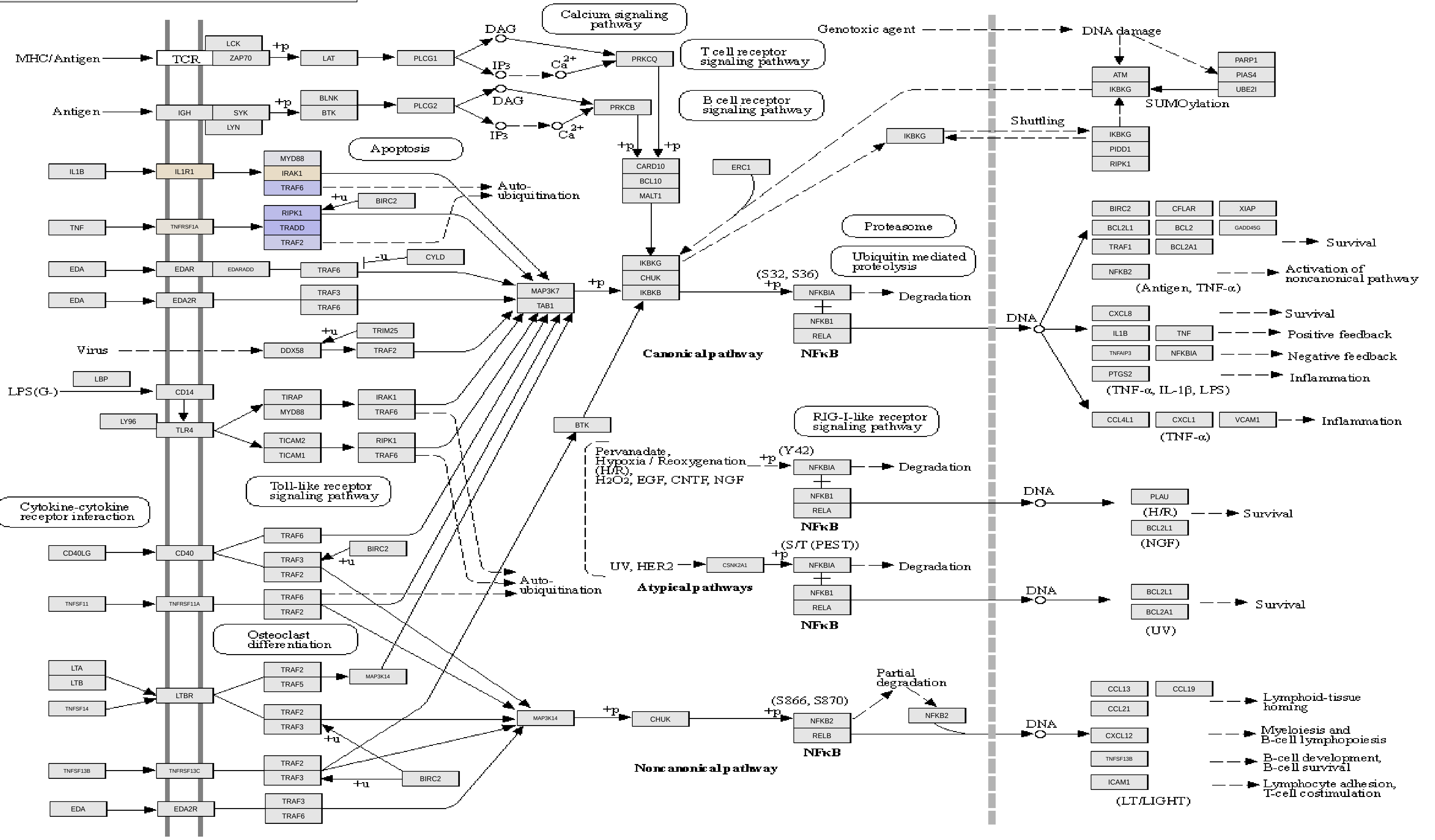

04064 4/12/19
(c) Kanehisa Laboratories

NF_kappa_B_signaling_pathway
Teenager

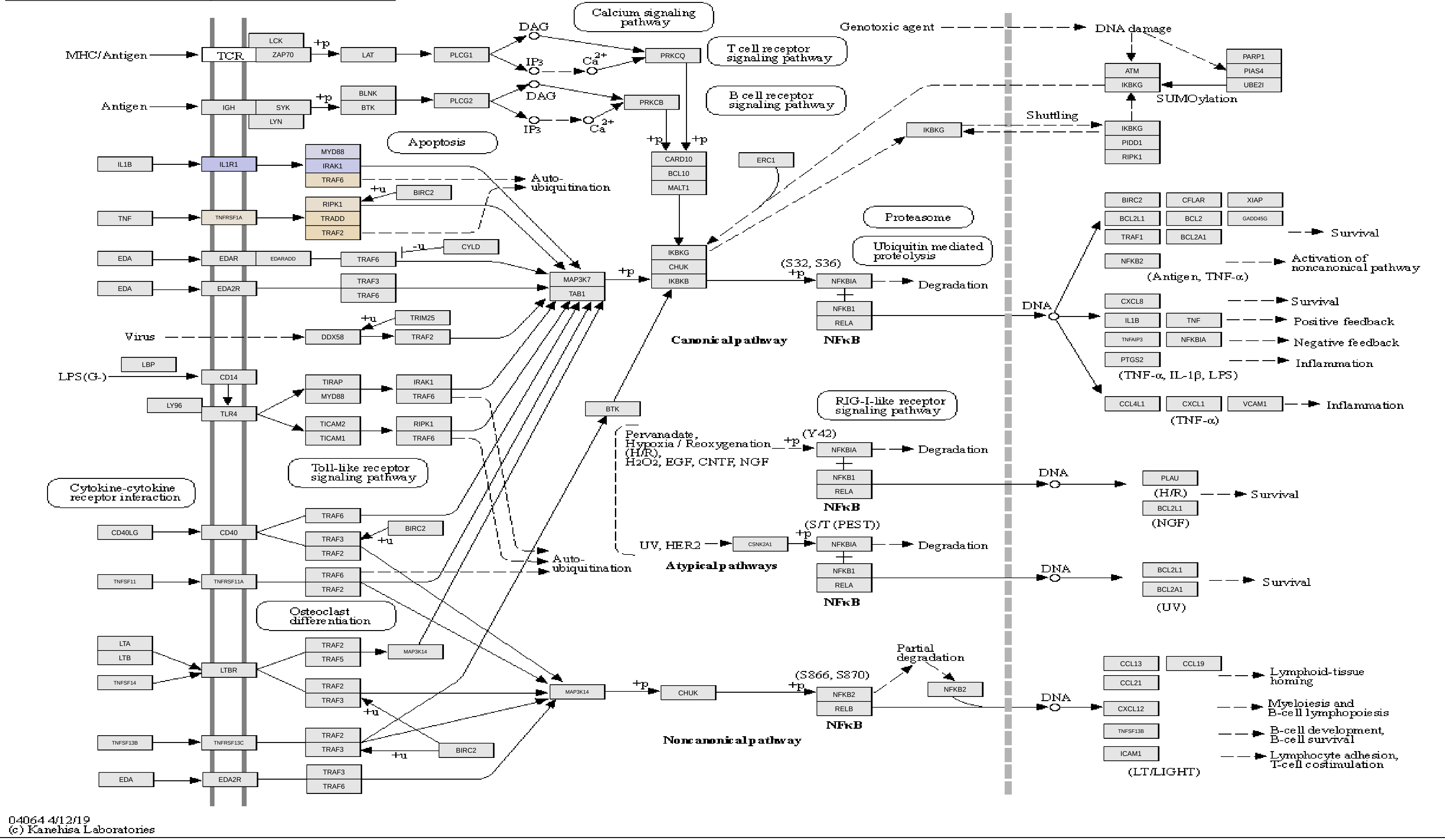

NF_kappa_B_signaling_pathway
Adult

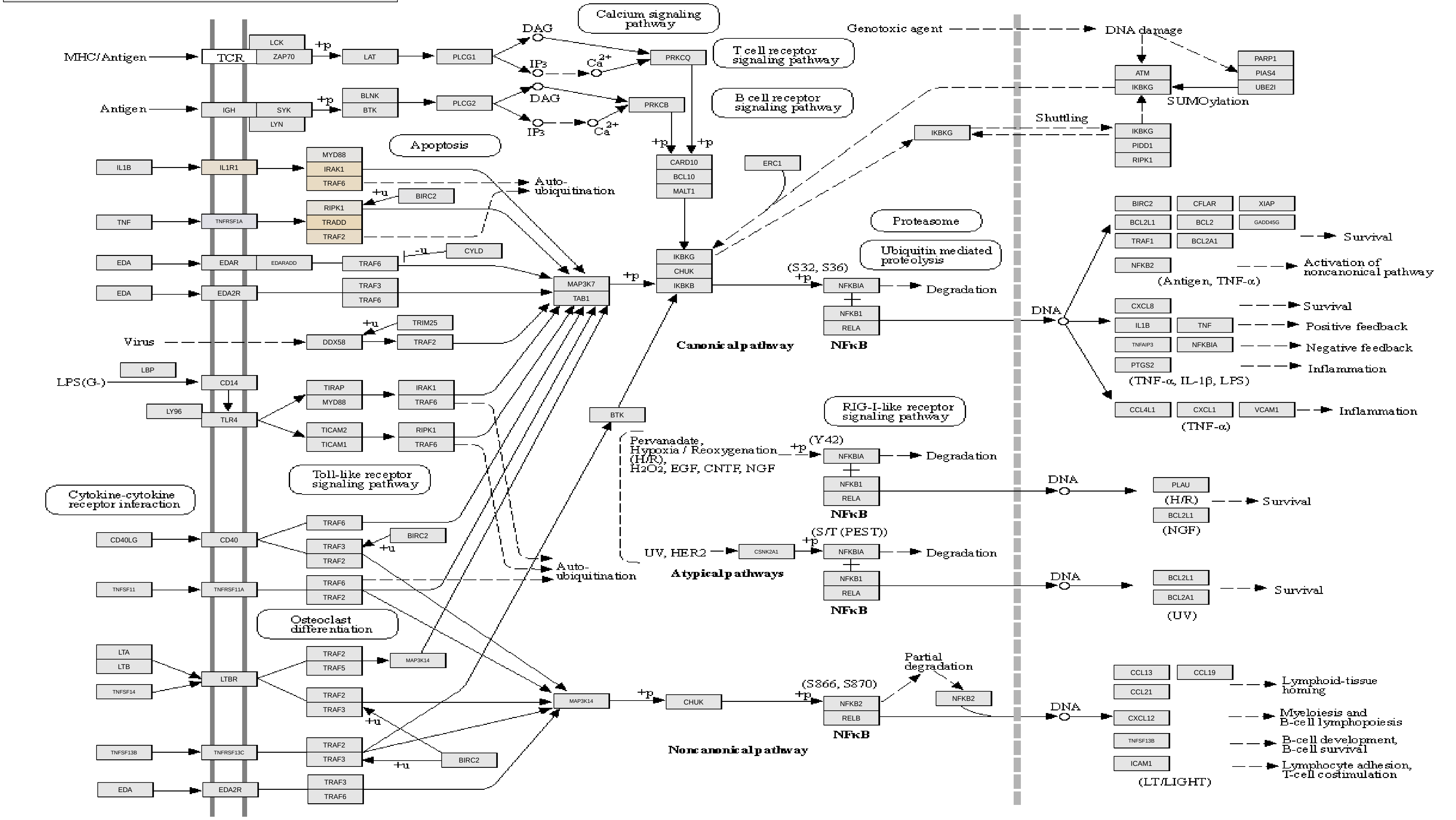

04064 4/12/19
(c) Kanehisa Laboratories

NF_kappa_B_signaling_pathway
Late Adult

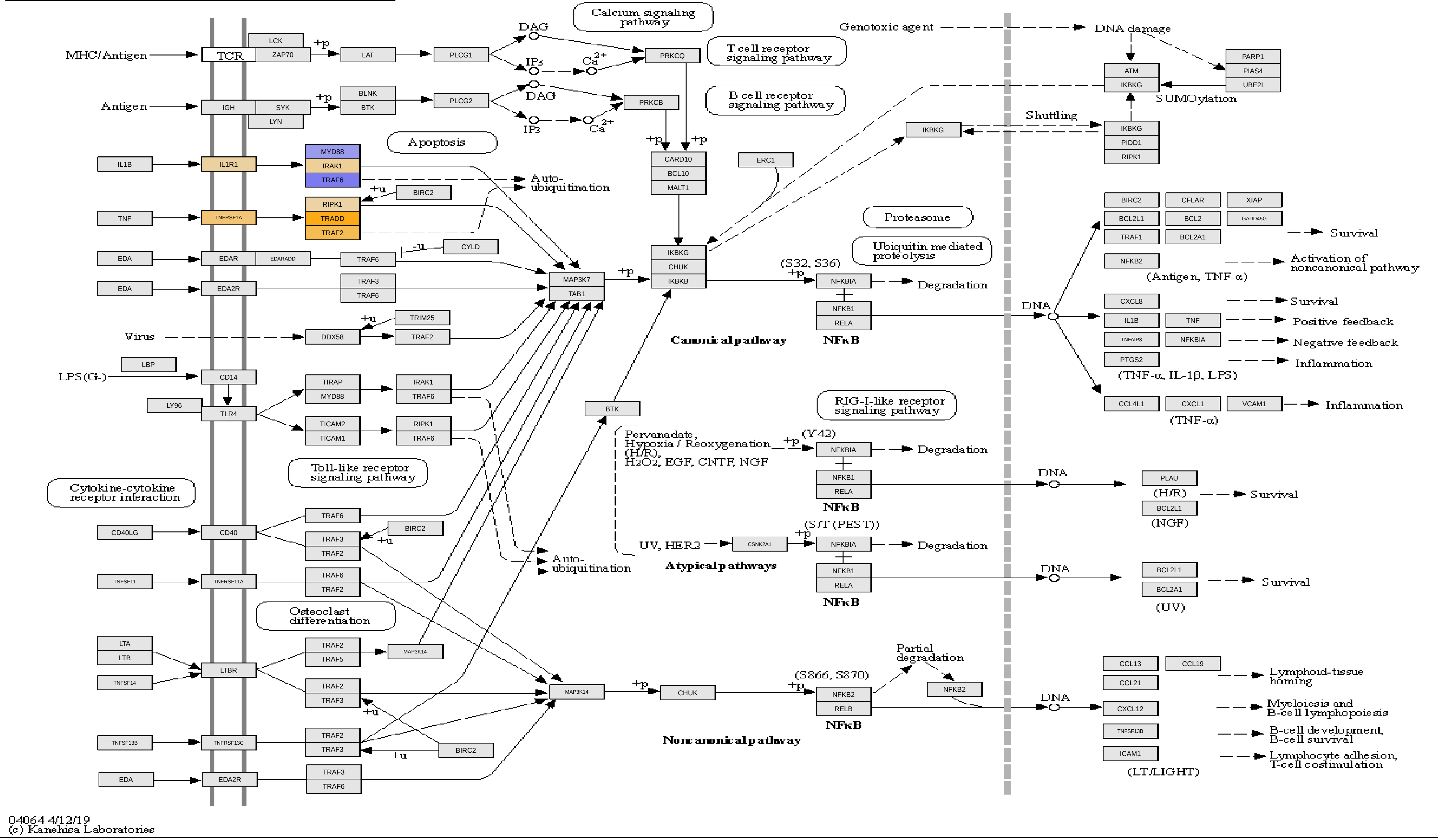

Supplement: Supplementary file 1 [file cells-11-00362-s001.zip › Suppl-Material-S3-Pathways-PSF_Expression/NF_kappa_B_signaling_pathway.pdf]
