# Supplementary material for: The Transcriptome and Methylome of the Developing and Aging Brain and Their Relations to Gliomas and Psychological Disorders
Source: Cells. 2022 Jan 21;11(3):362. doi: 10.3390/cells11030362 (PMC8834030; doi:10.3390/cells11030362)
Supplement: Supplementary file 1 [file cells-11-00362-s001.zip › Suppl-Material-S3-Pathways-PSF_Expression/NOD_like_receptor_signaling_pathway.pdf]

NOD−like receptor signaling pathway

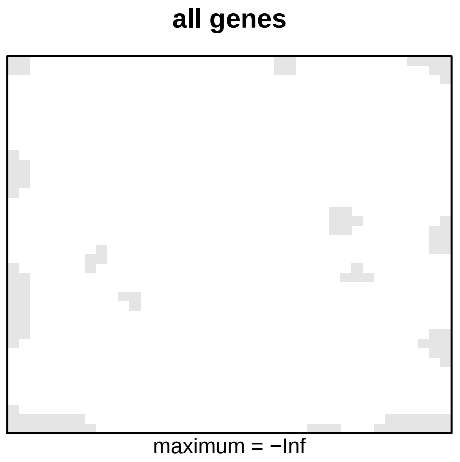

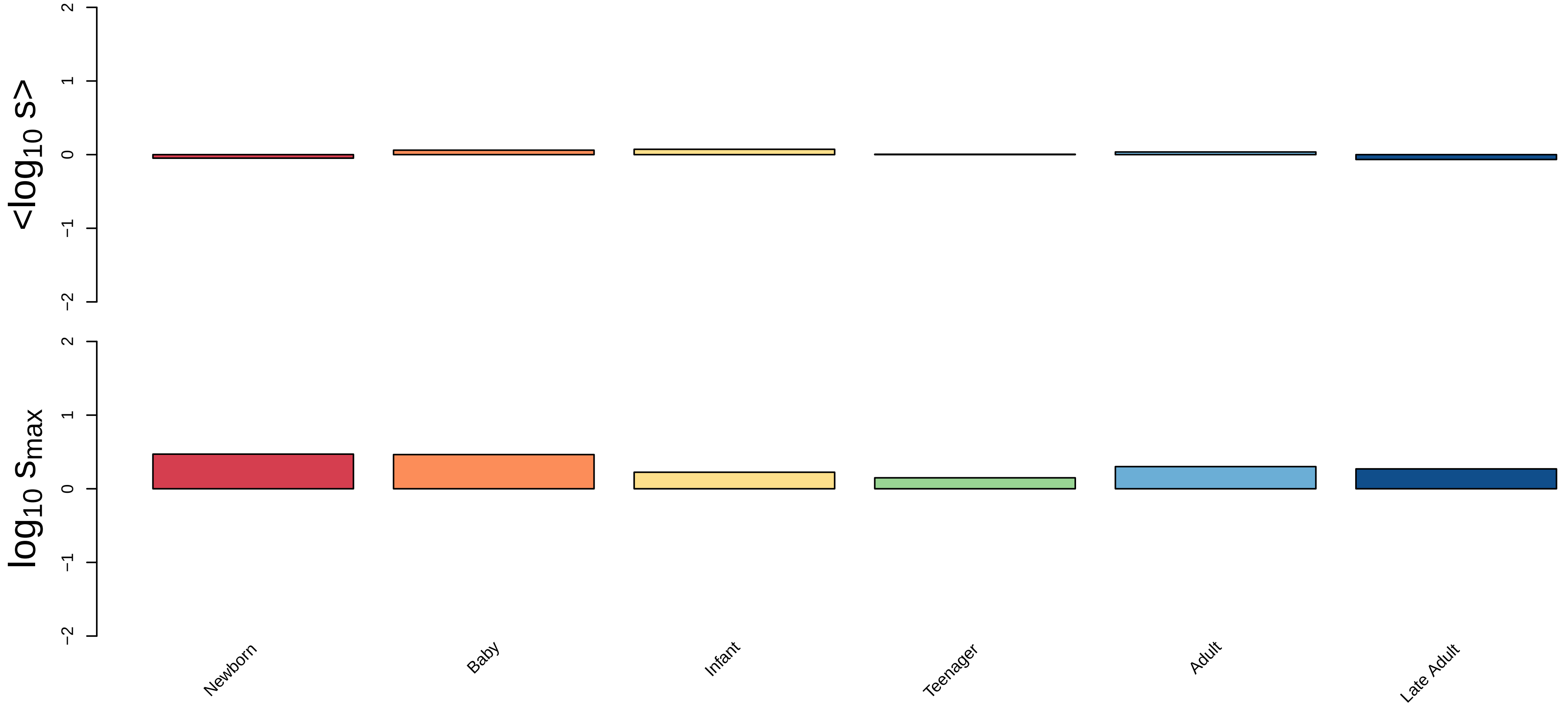

NOD_like_receptor_signaling_pathway
genes with data

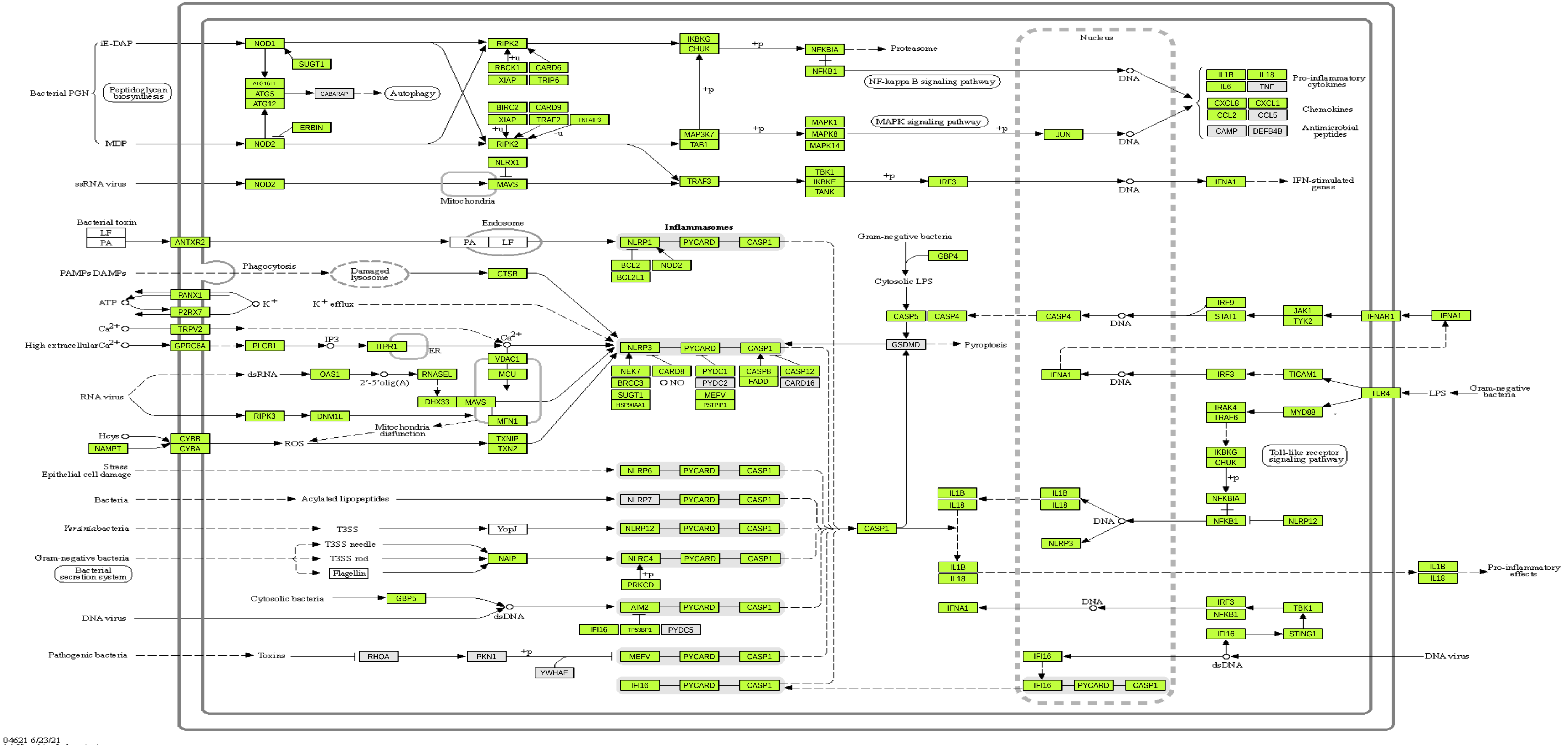

04621 6/23/21
(c) Kanehisa Laboratories

NOD_like_receptor_signaling_pathway
sink nodes

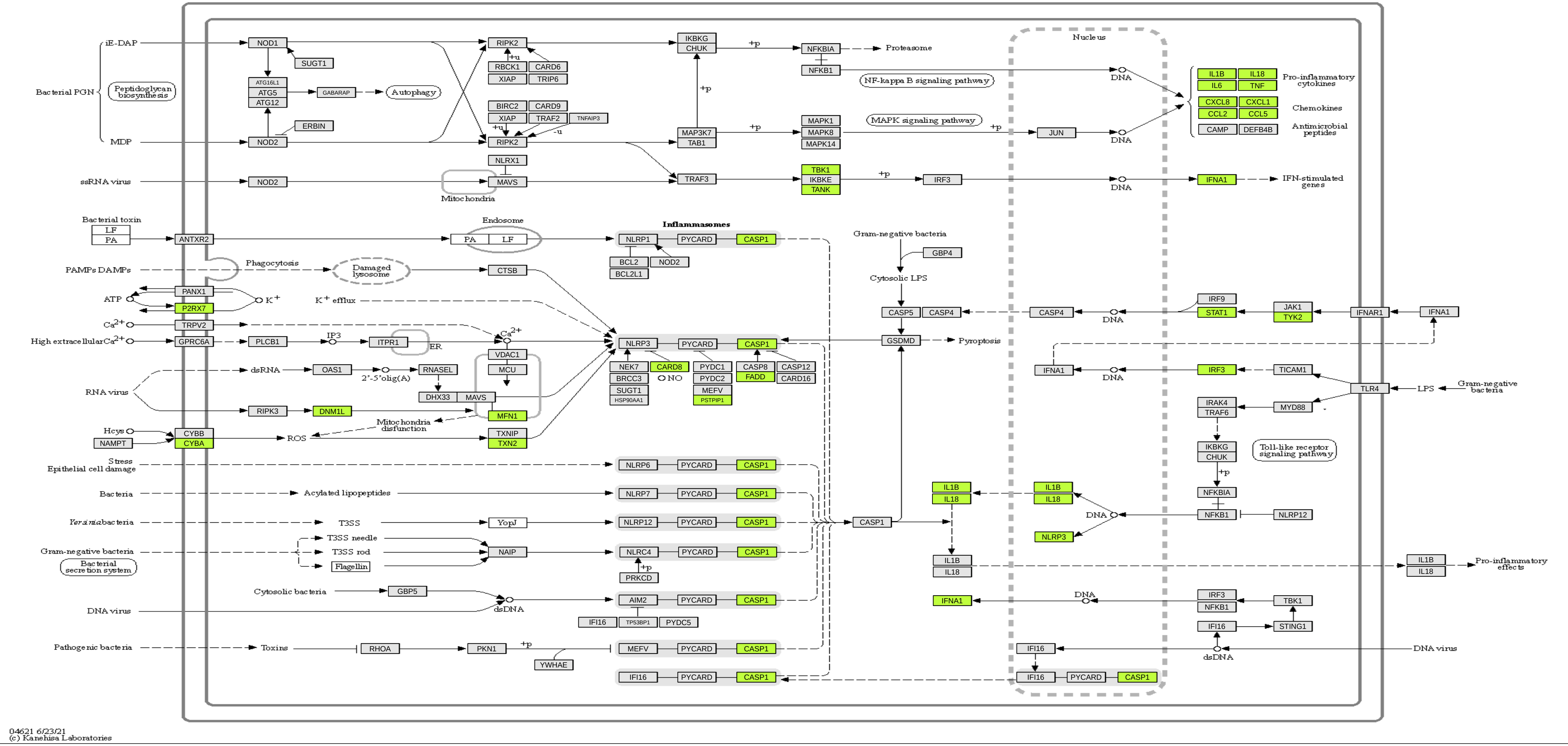

NOD_like_receptor_signaling_pathway
Newborn

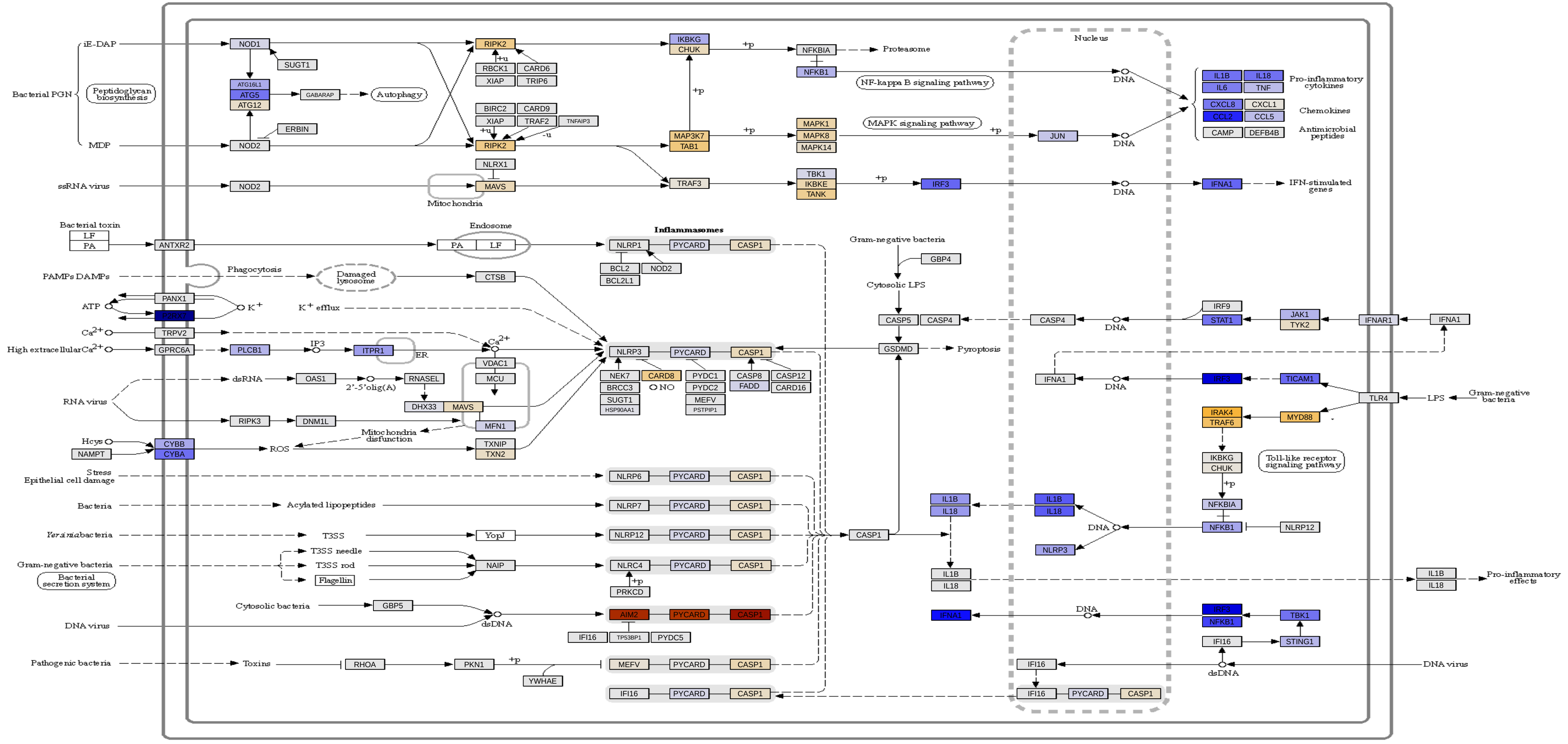

04621 6/23/21
(c) Kanehisa Laboratories

NOD_like_receptor_signaling_pathway
Baby

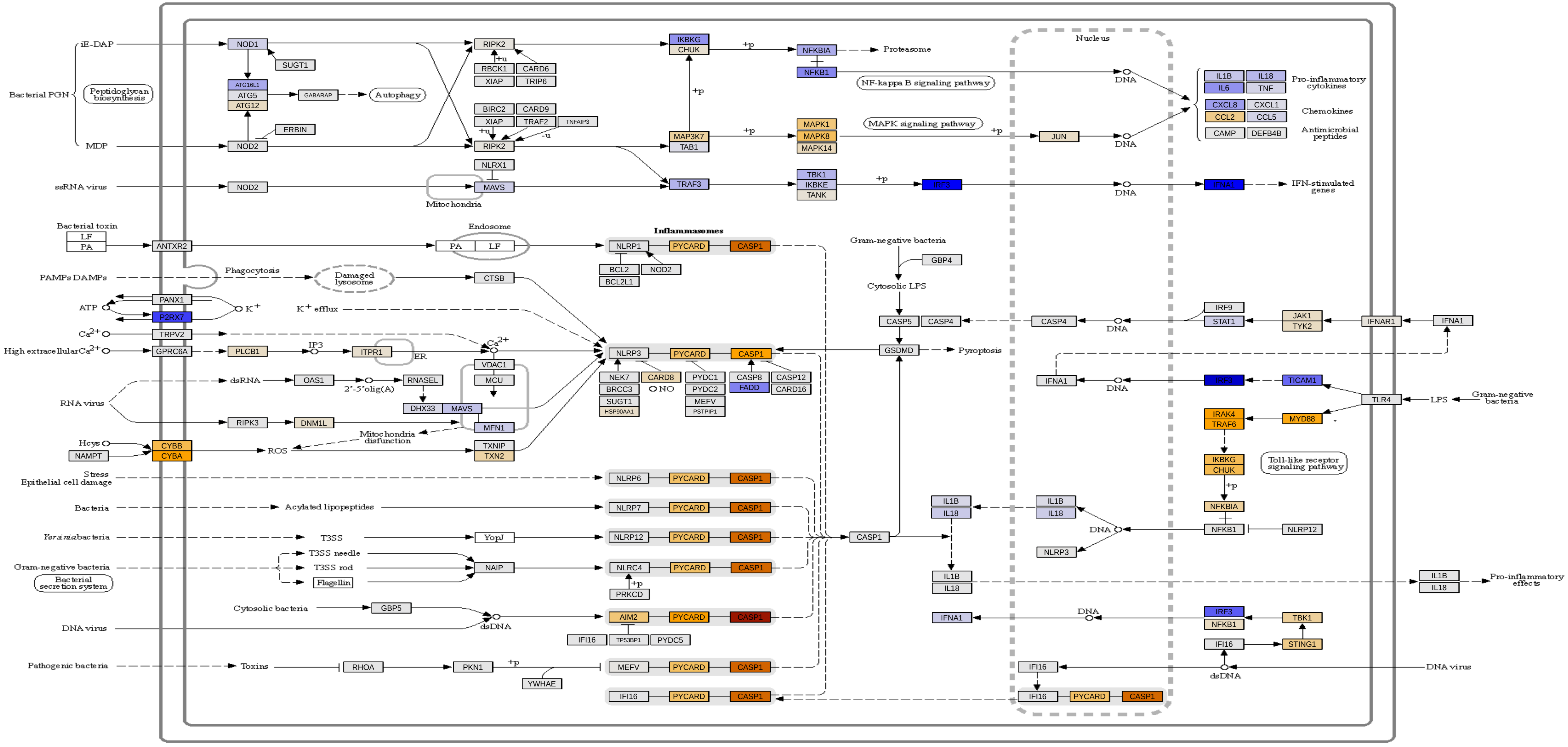

04621 6/23/21
(c) Kanehisa Laboratories

NOD_like_receptor_signaling_pathway
Infant

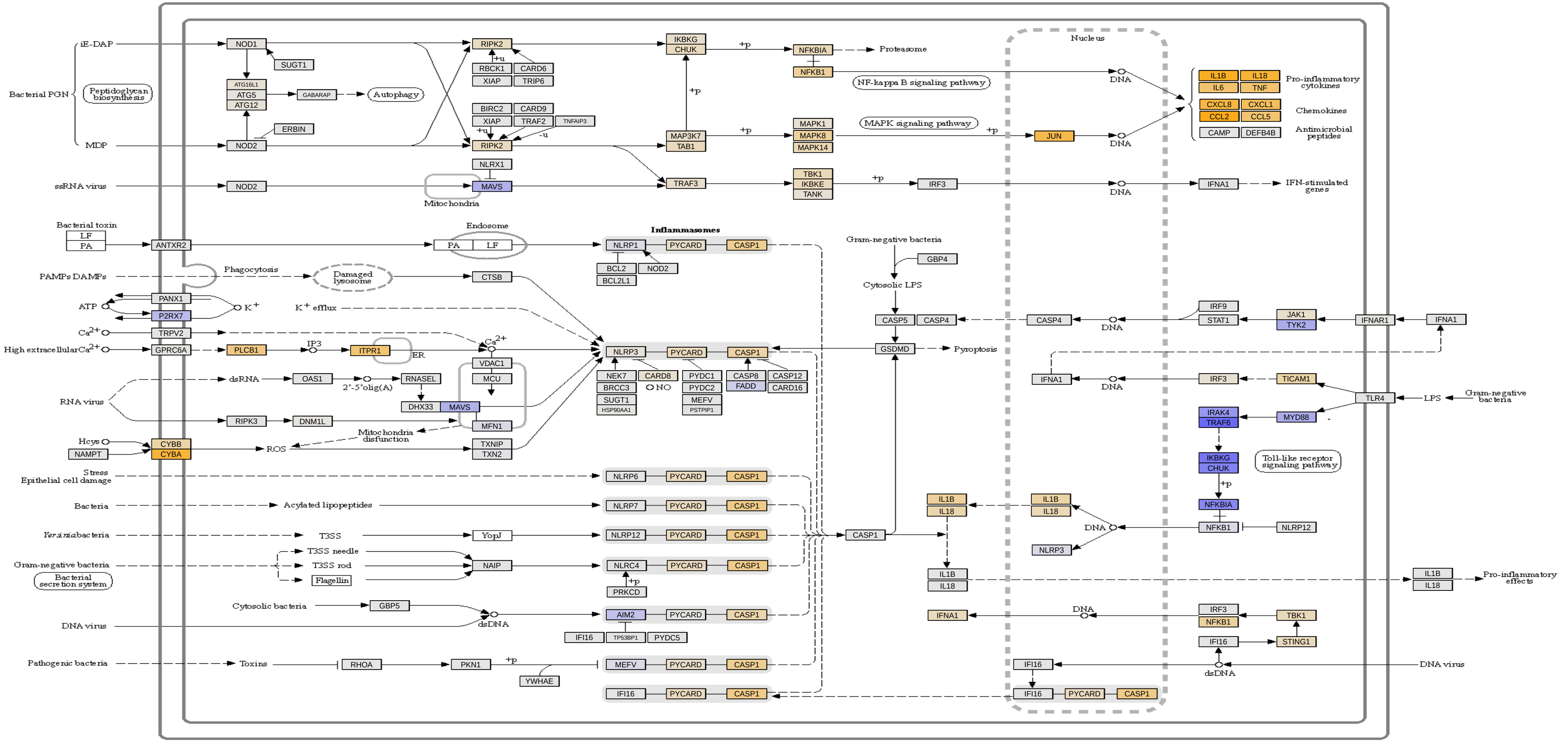

04621 6/23/21
(c) Kanehisa Laboratories

NOD_like_receptor_signaling_pathway
Teenager

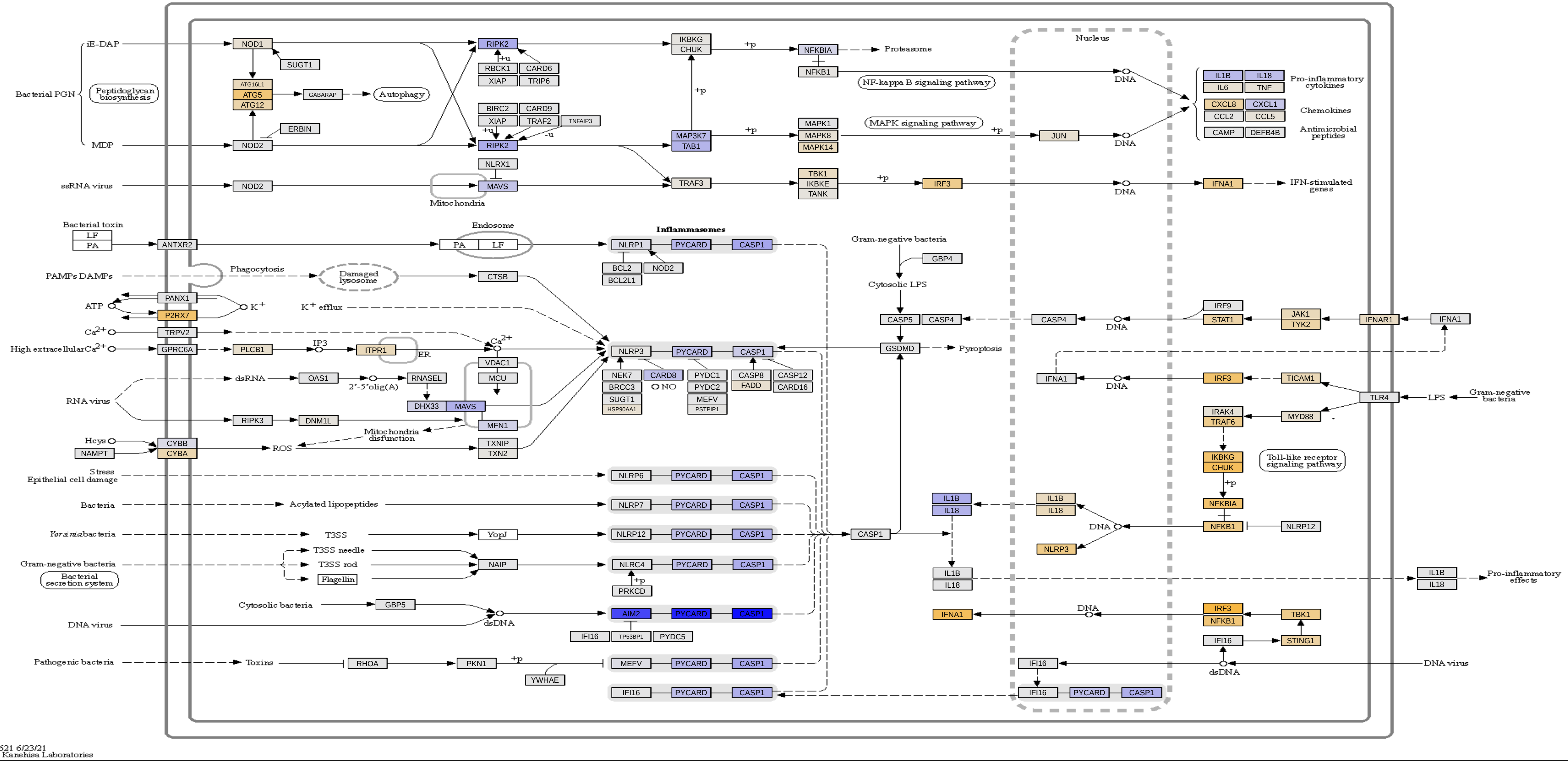

NOD_like_receptor_signaling_pathway
Adult

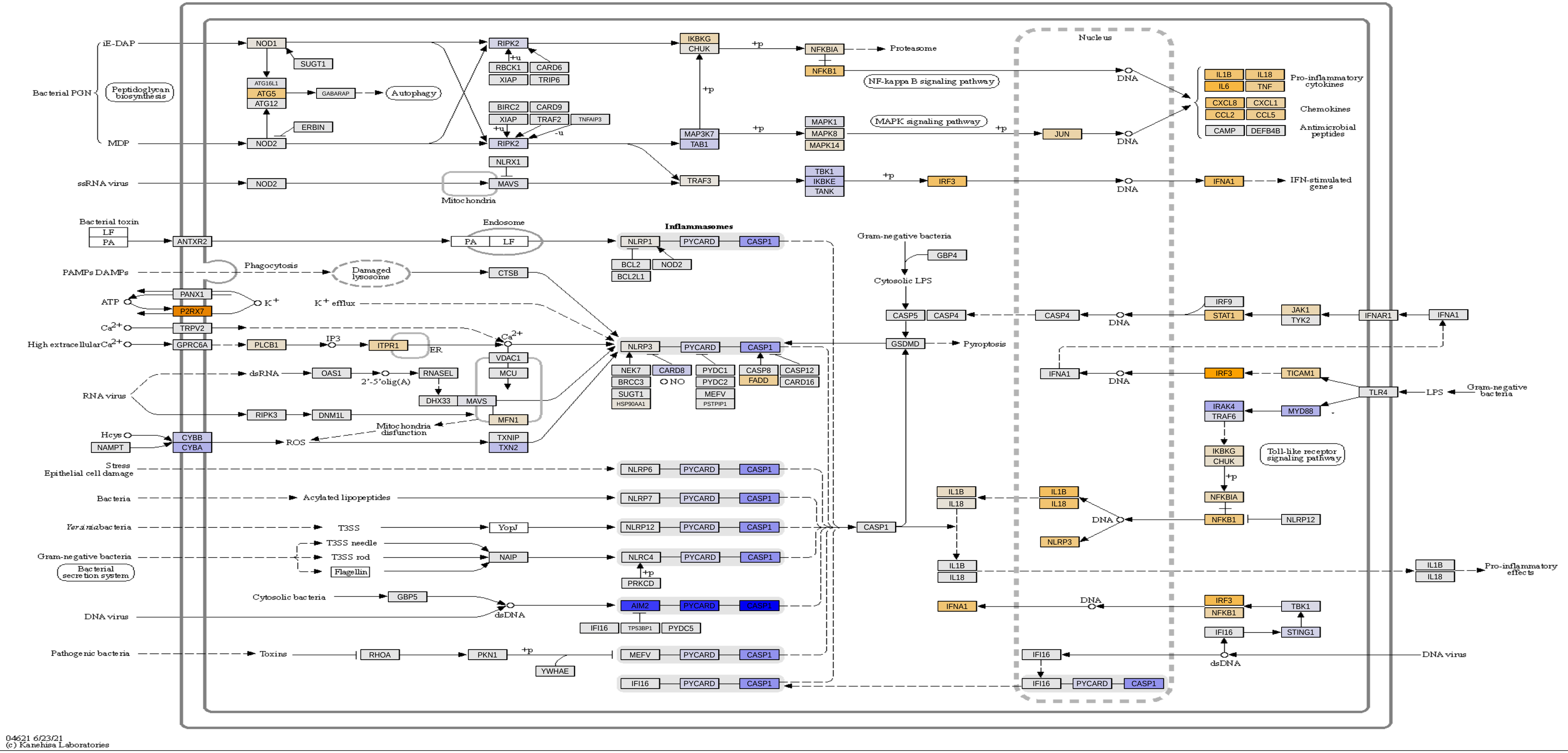

NOD_like_receptor_signaling_pathway
Late Adult

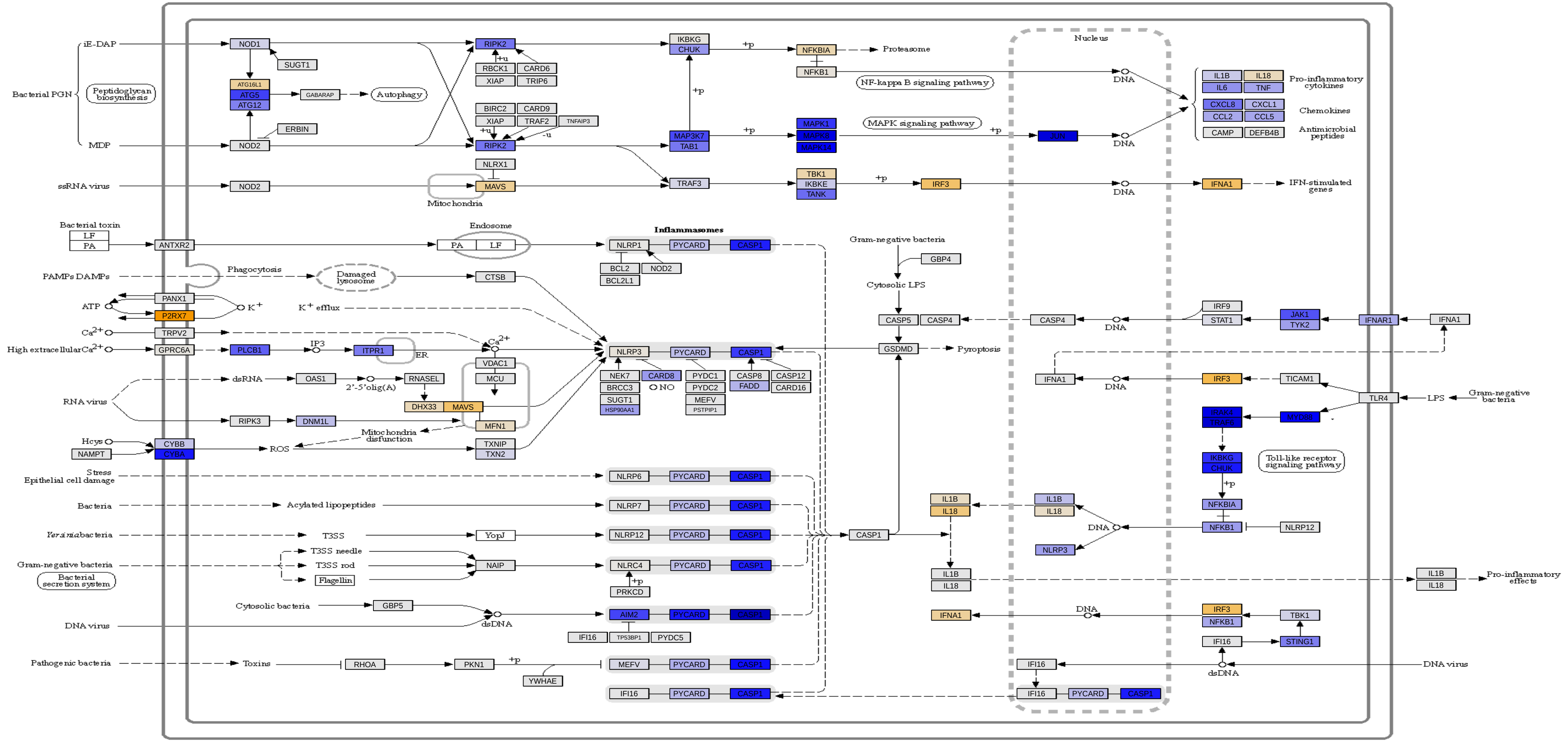

04621 6/23/21
(c) Kanehisa Laboratories
