# Supplementary material for: The Transcriptome and Methylome of the Developing and Aging Brain and Their Relations to Gliomas and Psychological Disorders
Source: Cells. 2022 Jan 21;11(3):362. doi: 10.3390/cells11030362 (PMC8834030; doi:10.3390/cells11030362)
Supplement: Supplementary file 1 [file cells-11-00362-s001.zip › Suppl-Material-S3-Pathways-PSF_Expression/Oxytocin_signaling_pathway.pdf]

Oxytocin signaling pathway

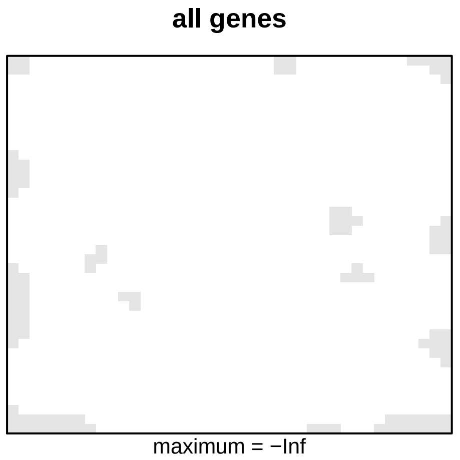

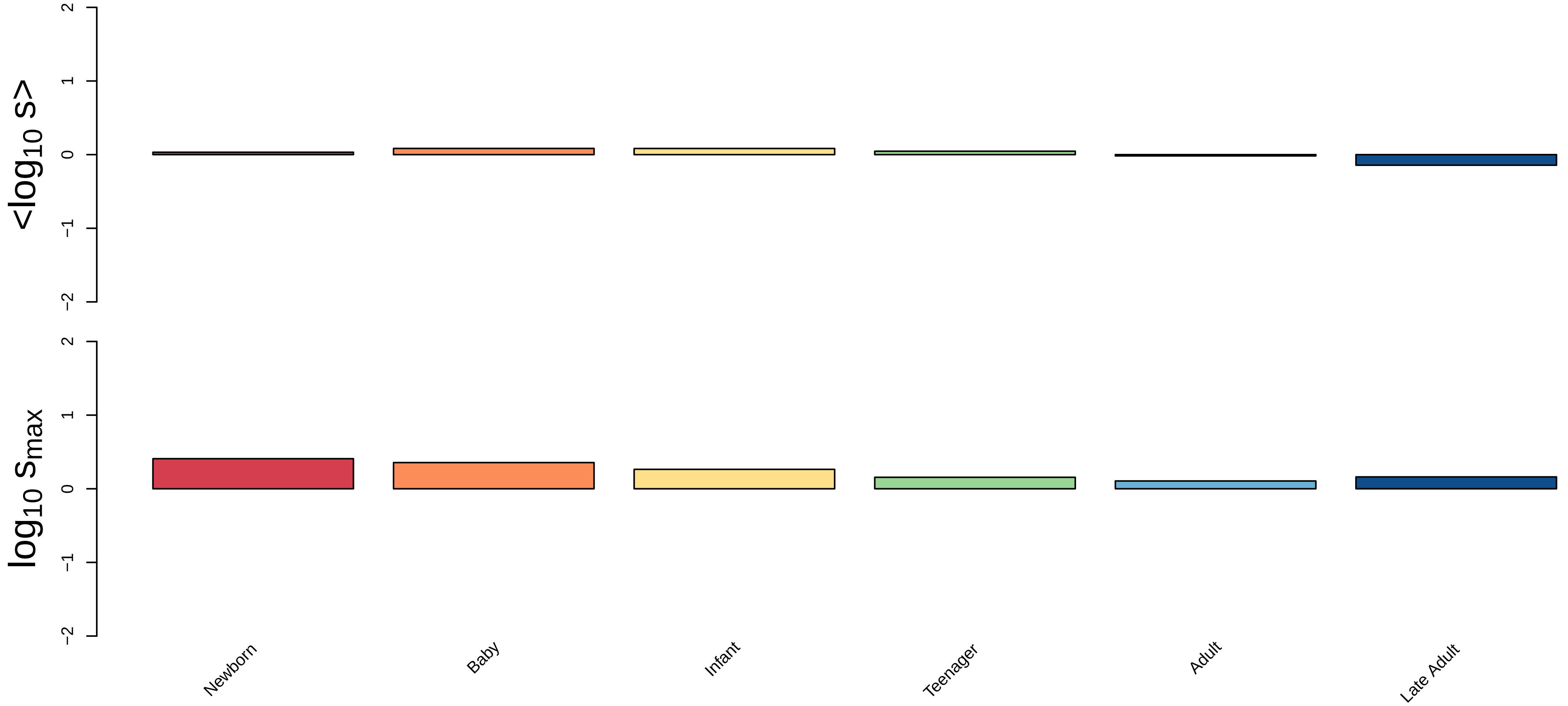

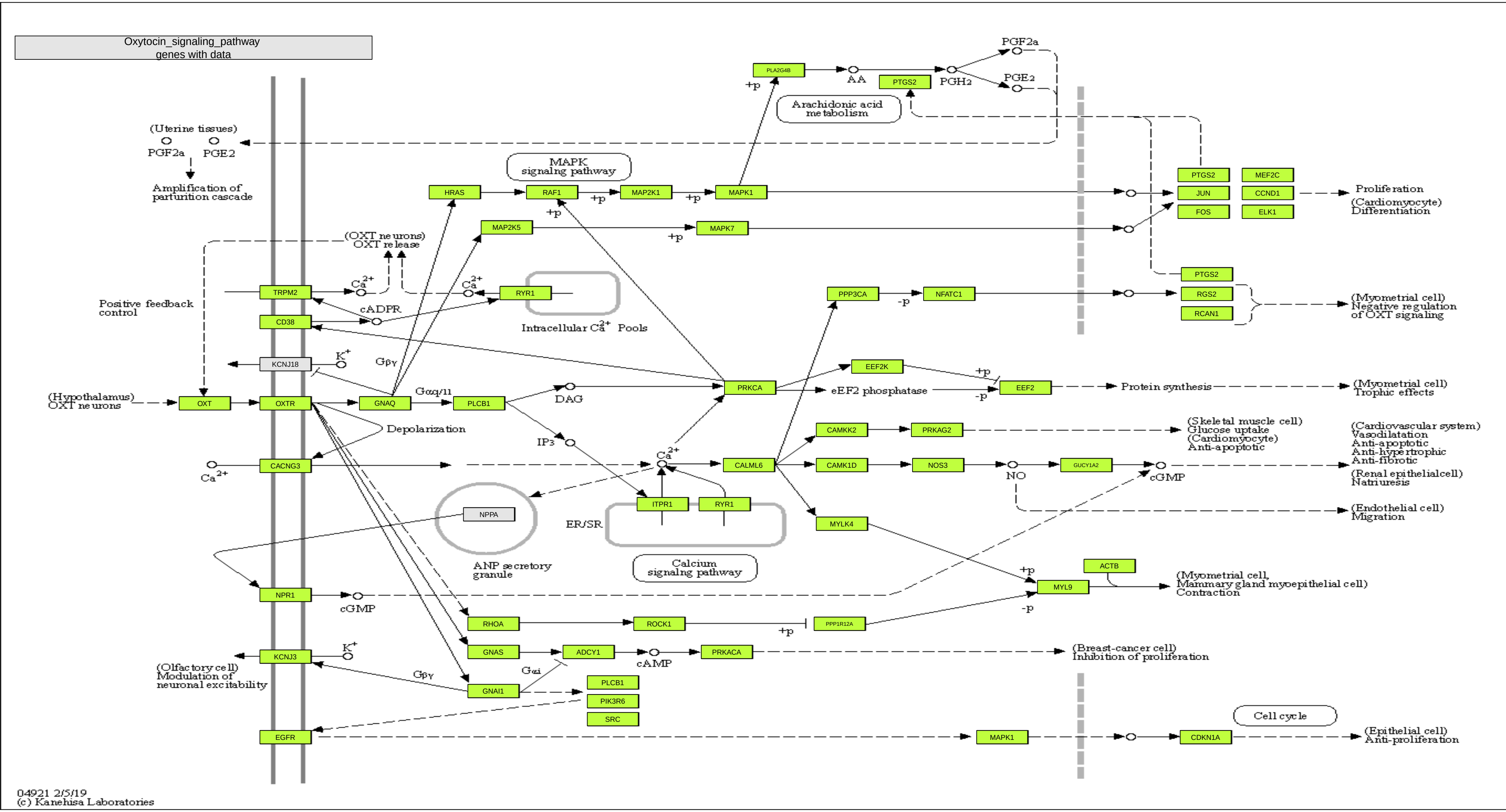
Oxytocin_signaling_pathway
genes with data
(Uterine tissues)
PGF2a
PGE2
Amplification of parturition cascade
PLA2G4B
AA
PTGS2
PGH2
PGF2a
PGE2
Arachidonic acid metabolism
MAPK signalng pathway
HRAS
RAF1
MAP2K1
MAPK1
+p
MAP2K5
MAPK7
(OXT neurons) OXT release
Positive feedback control
TRPM2
CD38
cADPR
RYR1
Intracellular Ca2+ Pools
KCNJ18
K+
Gβγ
(Hypothalamus) OXT neurons
OXT
OXTR
GNAQ
Gαq/11
PLCB1
DAG
PRKCA
Depolarization
IP3
CACNG3
Ca2+
CALML6
NPPA
ANP secretory granule
ER/SR
ITPR1
RYR1
Calcium signalng pathway
NPR1
cGMP
RHOA
ROCK1
PPP1R12A
GNAS
ADCY1
cAMP
PRKACA
KCNJ3
(Olfactory cell) Modulation of neuronal excitability
Gαi
GNAI1
PLCB1
PIK3R6
SRC
EGFR
MAPK1
PTGS2
JUN
FOS
MEF2C
CCND1
ELK1
Proliferation
(Cardiomyocyte) Differentiation
PPP3CA
-p
NFATC1
PTGS2
RGS2
RCAN1
(Myometrial cell) Negative regulation of OXT signaling
EEF2K
eEF2 phosphatase
EEF2
Protein synthesis
(Myometrial cell) Trophic effects
CAMKK2
PRKAG2
(Skeletal muscle cell) Glucose uptake
(Cardiomyocyte) Anti-apoptotic
CAMK1D
NOS3
NO
GUCY1A2
cGMP
(Cardiovascular system) Vasodilatation Anti-apoptotic Anti-hypertrophic Anti-fibrotic
(Renal epithelialcell) Natriuresis
(Endothelial cell) Migration
MYLK4
ACTB
MYL9
(Myometrial cell, Mammary gland myoepithelial cell) Contraction
(Breast-cancer cell) Inhibition of proliferation
Cell cycle
CDKN1A
(Epithelial cell) Anti-proliferation
04921 2/5/19
(c) Kanehisa Laboratories

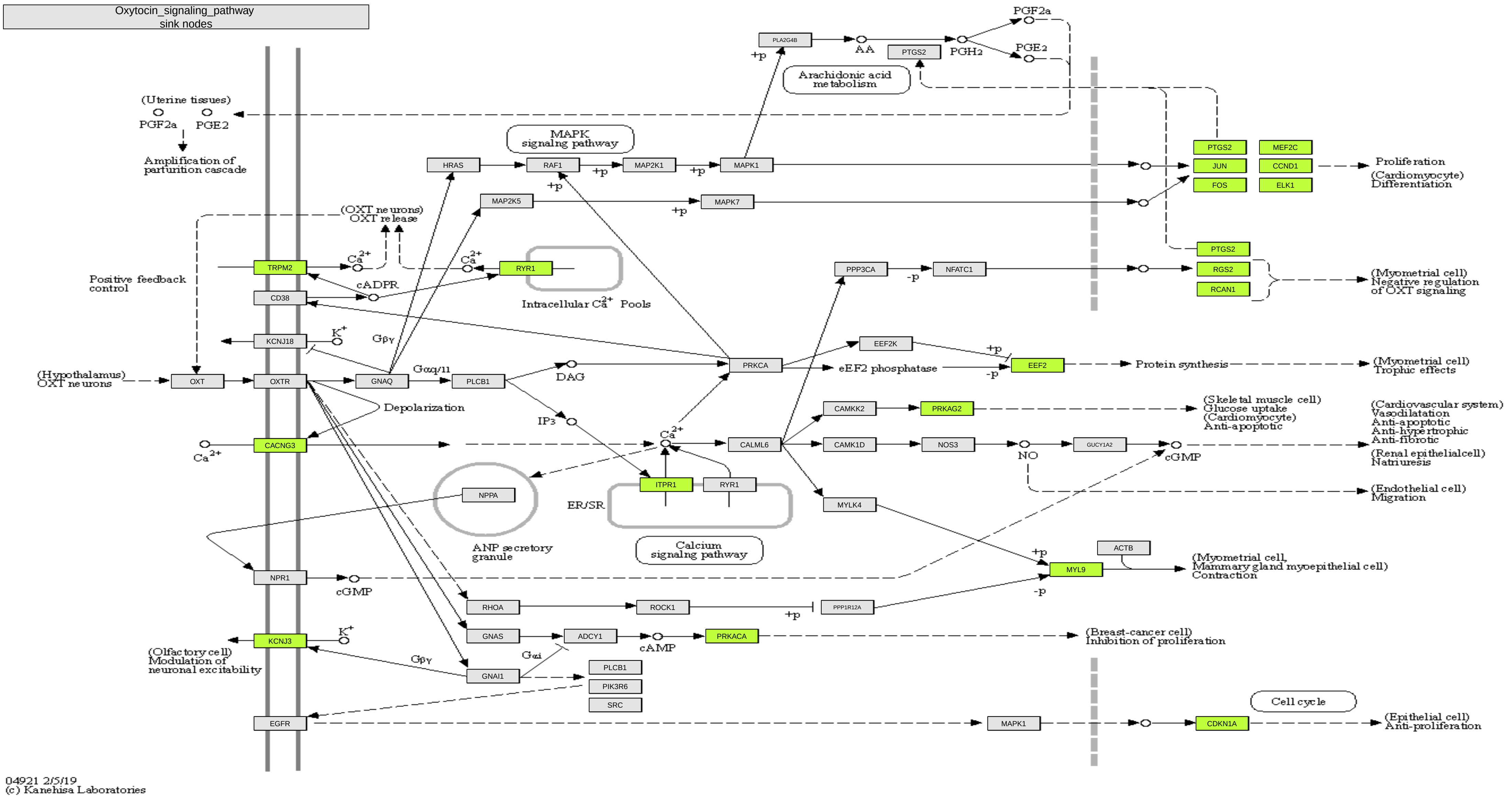
Oxytocin_signaling_pathway
sink nodes
(Uterine tissues)
PGF2a
PGE2
Amplification of parturition cascade
(OXT neurons) OXT release
Positive feedback control
(Hypothalamus) OXT neurons
(Olfactory cell) Modulation of neuronal excitability
TRPM2
CD38
KCNJ18
OXT
OXTR
CACNG3
NPR1
KCNJ3
EGFR
cADPR
Depolarization
NPPA
ANP secretory granule
GNAQ
PLCB1
DAG
IP3
HRAS
RAF1
MAP2K1
MAPK1
MAP2K5
MAPK7
MAPK signalng pathway
RYR1
Intracellular Ca2+ Pools
PRKCA
ITPR1
ER/SR
Calcium signalng pathway
CALML6
CAMKK2
PRKAG2
CAMK1D
NOS3
GUCY1A2
NO
cGMP
MYLK4
RHOA
ROCK1
PPP1R12A
GNAS
ADCY1
cAMP
PRKACA
GNAI1
PLCB1
PIK3R6
SRC
PLA2G4B
AA
PTGS2
PGH2
PGF2a
PGE2
Arachidonic acid metabolism
PPP3CA
NFATC1
EEF2K
EEF2
eEF2 phosphatase
Protein synthesis
ACTB
MYL9
MEF2C
JUN
CCND1
FOS
ELK1
RGS2
RCAN1
CDKN1A
Cell cycle
Proliferation
(Cardiomyocyte) Differentiation
(Myometrial cell) Negative regulation of OXT signaling
(Myometrial cell) Trophic effects
(Skeletal muscle cell) Glucose uptake
(Cardiomyocyte) Anti-apoptotic
(Cardiovascular system) Vasodilatation
Anti-apoptotic
Anti-hypertrophic
Anti-fibrotic
(Renal epithelial cell) Natriuresis
(Endothelial cell) Migration
(Myometrial cell, Mammary gland myoepithelial cell) Contraction
(Breast-cancer cell) Inhibition of proliferation
(Epithelial cell) Anti-proliferation
04921 2/5/19
(c) Kanehisa Laboratories

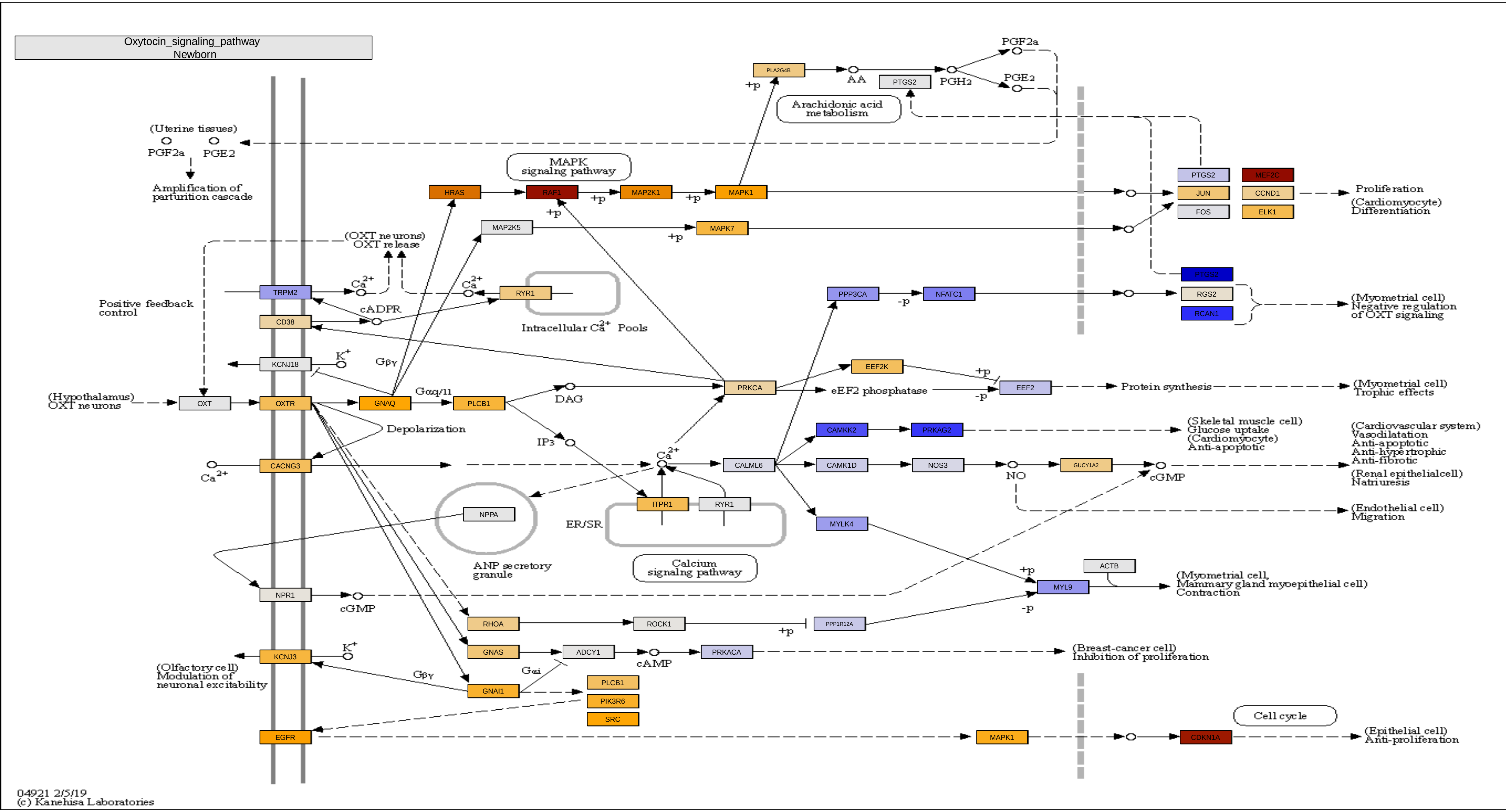
Oxytocin_signaling_pathway
Newborn
PGF2a
PLA2G4B
AA
PTGS2
PGH2
PGE2
+p
Arachidonic acid metabolism
(Uterine tissues)
PGF2a
PGE2
Amplification of parturition cascade
MAPK signalng pathway
HRAS
RAF1
+p
MAP2K1
+p
MAPK1
+p
PTGS2
MEF2C
JUN
CCND1
FOS
ELK1
Proliferation
(Cardiomyocyte) Differentiation
MAP2K5
MAPK7
+p
(OXT neurons) OXT release
Positive feedback control
TRPM2
Ca^{2+}
Ca^{2+}
RYR1
cADPR
CD38
Intracellular Ca^{2+} Pools
PPP3CA
-p
NFATC1
RGS2
RCAN1
(Myometrial cell) Negative regulation of OXT signaling
KCNJ18
K^{+}
$G\beta\gamma$
$G\alpha q/11$
(Hypothalamus) OXT neurons
OXT
OXTR
GNAQ
PLCB1
DAG
PRKCA
EEF2K
+p
eEF2 phosphatase
-p
EEF2
Protein synthesis
(Myometrial cell) Trophic effects
Depolarization
IP_3
CAMKK2
PRKAG2
(Skeletal muscle cell) Glucose uptake
(Cardiomyocyte) Anti-apoptotic
(Cardiovascular system) Vasodilatation
Anti-apoptotic
Anti-hypertrophic
Anti-fibrotic
CACNG3
Ca^{2+}
Ca^{2+}
CALML6
CAMK1D
NOS3
NO
GUCY1A2
cGMP
(Renal epithelial cell) Natriuresis
(Endothelial cell) Migration
NPPA
ITPR1
RYR1
ER/SR
MYLK4
ANP secretory granule
Calcium signalng pathway
ACTB
+p
MYL9
(Myometrial cell, Mammary gland myoepithelial cell) Contraction
NPR1
cGMP
-p
RHOA
ROCK1
+p
PPP1R12A
GNAS
ADCY1
cAMP
PRKACA
(Breast-cancer cell) Inhibition of proliferation
KCNJ3
K^{+}
$G\beta\gamma$
$G\alpha i$
(Olfactory cell) Modulation of neuronal excitability
GNAI1
PLCB1
PIK3R6
SRC
Cell cycle
EGFR
MAPK1
CDKN1A
(Epithelial cell) Anti-proliferation
04921 2/5/19
(c) Kanehisa Laboratories

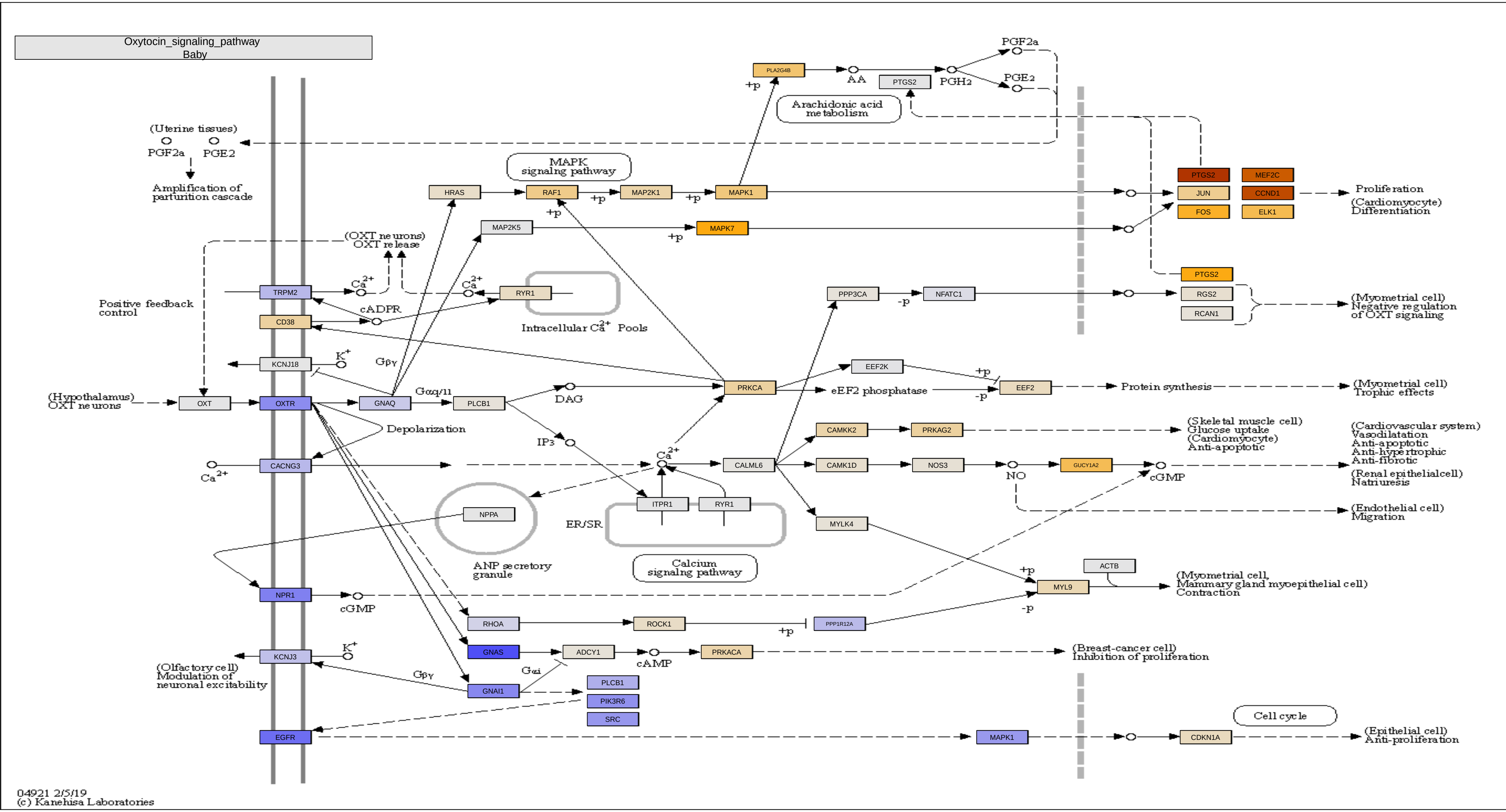

Oxytocin_signaling_pathway
Baby
(Uterine tissues)
PGF2a
PGE2
Amplification of parturition cascade
PLA2G4B
+p
AA
PTGS2
PGH2
PGF2a
PGE2
Arachidonic acid metabolism
MAPK signalng pathway
HRAS
RAF1
+p
MAP2K1
+p
MAPK1
PTGS2
MEF2C
JUN
CCND1
FOS
ELK1
Proliferation
(Cardiomyocyte) Differentiation
MAP2K5
MAPK7
+p
(OXT neurons) OXT release
Positive feedback control
TRPM2
Ca^{2+}
Ca^{2+}
RYR1
cADPR
CD38
Intracellular Ca^{2+} Pools
PTGS2
PPP3CA
-p
NFATC1
RGS2
RCAN1
(Myometrial cell) Negative regulation of OXT signaling
KCNJ18
K^{+}
$G\beta\gamma$
EEF2K
+p
EEF2
-p
PRKCA
eEF2 phosphatase
Protein synthesis
(Myometrial cell) Trophic effects
(Hypothalamus) OXT neurons
OXT
OXTR
GNAQ
$G\alpha q/11$
PLCB1
DAG
Depolarization
CAMKK2
PRKAG2
(Skeletal muscle cell) Glucose uptake (Cardiomyocyte) Anti-apoptotic
(Cardiovascular system) Vasodilatation Anti-apoptotic Anti-hypertrophic Anti-fibrotic
IP3
Ca^{2+}
CACNG3
Ca^{2+}
CALML6
CAMK1D
NOS3
NO
GUCY1A2
cGMP
(Renal epithelial cell) Natriuresis
NPPA
ER/SR
ITPR1
RYR1
MYLK4
(Endothelial cell) Migration
ANP secretory granule
Calcium signalng pathway
ACTB
+p
MYL9
(Myometrial cell, Mammary gland myoepithelial cell) Contraction
NPR1
cGMP
-p
RHOA
ROCK1
+p
PPP1R12A
KCNJ3
K^{+}
GNAS
ADCY1
cAMP
PRKACA
(Breast-cancer cell) Inhibition of proliferation
(Olfactory cell) Modulation of neuronal excitability
$G\beta\gamma$
$G\alpha i$
GNAI1
PLCB1
PIK3R6
SRC
Cell cycle
EGFR
MAPK1
CDKN1A
(Epithelial cell) Anti-proliferation
04921 2/5/19
(c) Kanehisa Laboratories

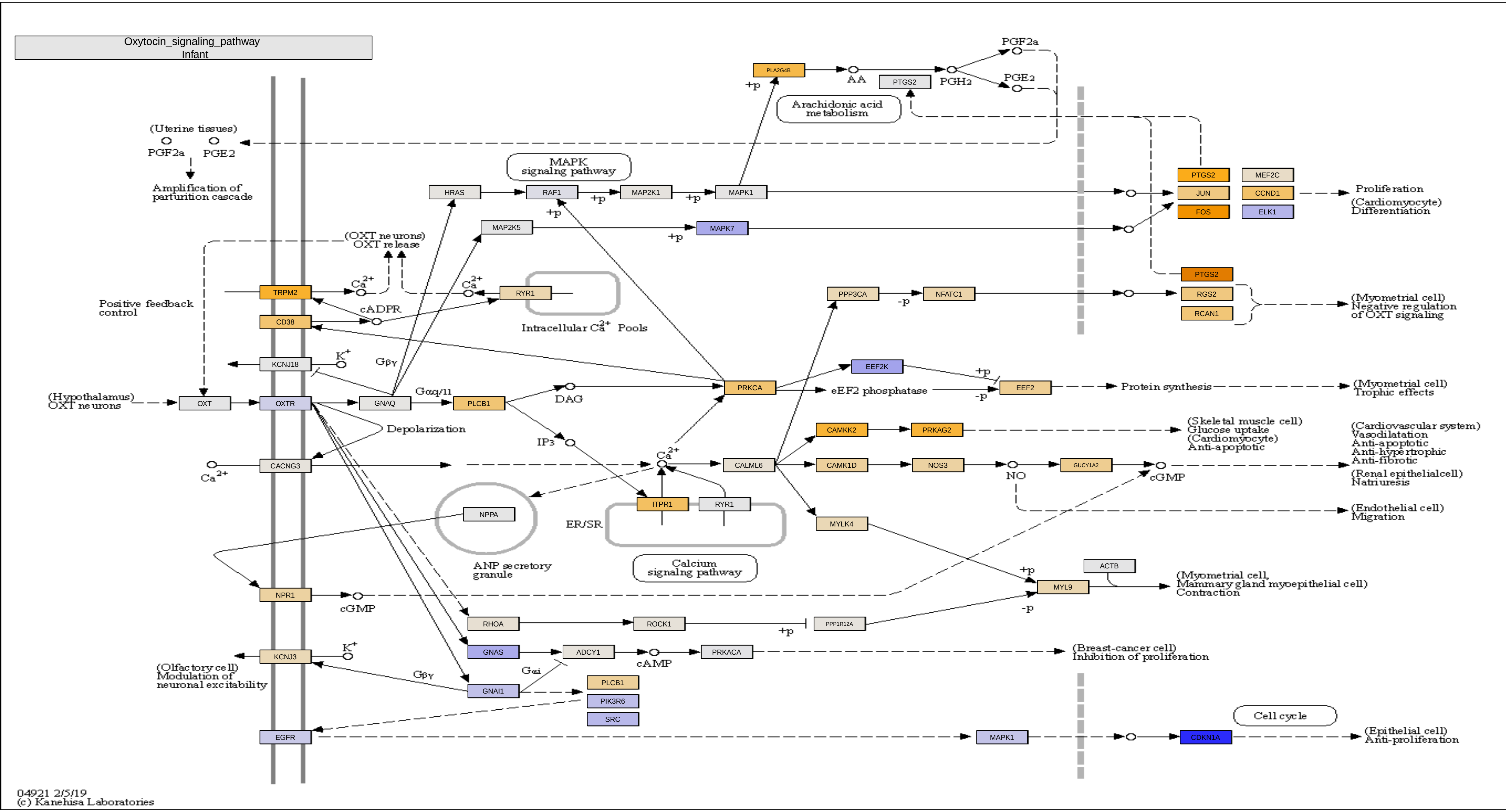

Oxytocin_signaling_pathway
Infant
PGF2a
PGE2
PLA2G4B
+p
AA
PTGS2
PGH2
Arachidonic acid metabolism
(Uterine tissues)
PGF2a
PGE2
Amplification of parturition cascade
MAPK signalng pathway
HRAS
RAF1
+p
MAP2K1
+p
MAPK1
PTGS2
MEF2C
JUN
CCND1
FOS
ELK1
Proliferation
(Cardiomyocyte) Differentiation
MAP2K5
MAPK7
+p
(OXT neurons) OXT release
Positive feedback control
TRPM2
Ca^{2+}
RYR1
cADPR
CD38
Intracellular Ca^{2+} Pools
PTGS2
RGS2
RCAN1
PPP3CA
-p
NFATC1
(Myometrial cell) Negative regulation of OXT signaling
KCNJ18
K^{+}
$G\beta\gamma$
EEF2K
+p
EEF2
-p
eEF2 phosphatase
Protein synthesis
(Myometrial cell) Trophic effects
(Hypothalamus) OXT neurons
OXT
OXTR
GNAQ
$G\alpha q/11$
PLCB1
DAG
PRKCA
Depolarization
IP3
CAMKK2
PRKAG2
(Skeletal muscle cell) Glucose uptake (Cardiomyocyte) Anti-apoptotic
(Cardiovascular system) Vasodilatation Anti-apoptotic Anti-hypertrophic Anti-fibrotic
CACNG3
Ca^{2+}
CALML6
CAMK1D
NOS3
NO
GUCY1A2
cGMP
(Renal epithelial cell) Natriuresis
NPPA
ER/SR
ITPR1
RYR1
(Endothelial cell) Migration
MYLK4
ANP secretory granule
Calcium signalng pathway
ACTB
+p
MYL9
(Myometrial cell, Mammary gland myoepithelial cell) Contraction
NPR1
cGMP
-p
RHOA
ROCK1
PPP1R12A
+p
KCNJ3
K^{+}
GNAS
ADCY1
cAMP
PRKACA
(Breast-cancer cell) Inhibition of proliferation
(Olfactory cell) Modulation of neuronal excitability
$G\beta\gamma$
$G\alpha i$
GNAI1
PLCB1
PIK3R6
SRC
Cell cycle
EGFR
MAPK1
CDKN1A
(Epithelial cell) Anti-proliferation
04921 2/5/19
(c) Kanehisa Laboratories

Oxytocin_signaling_pathway
Teenager

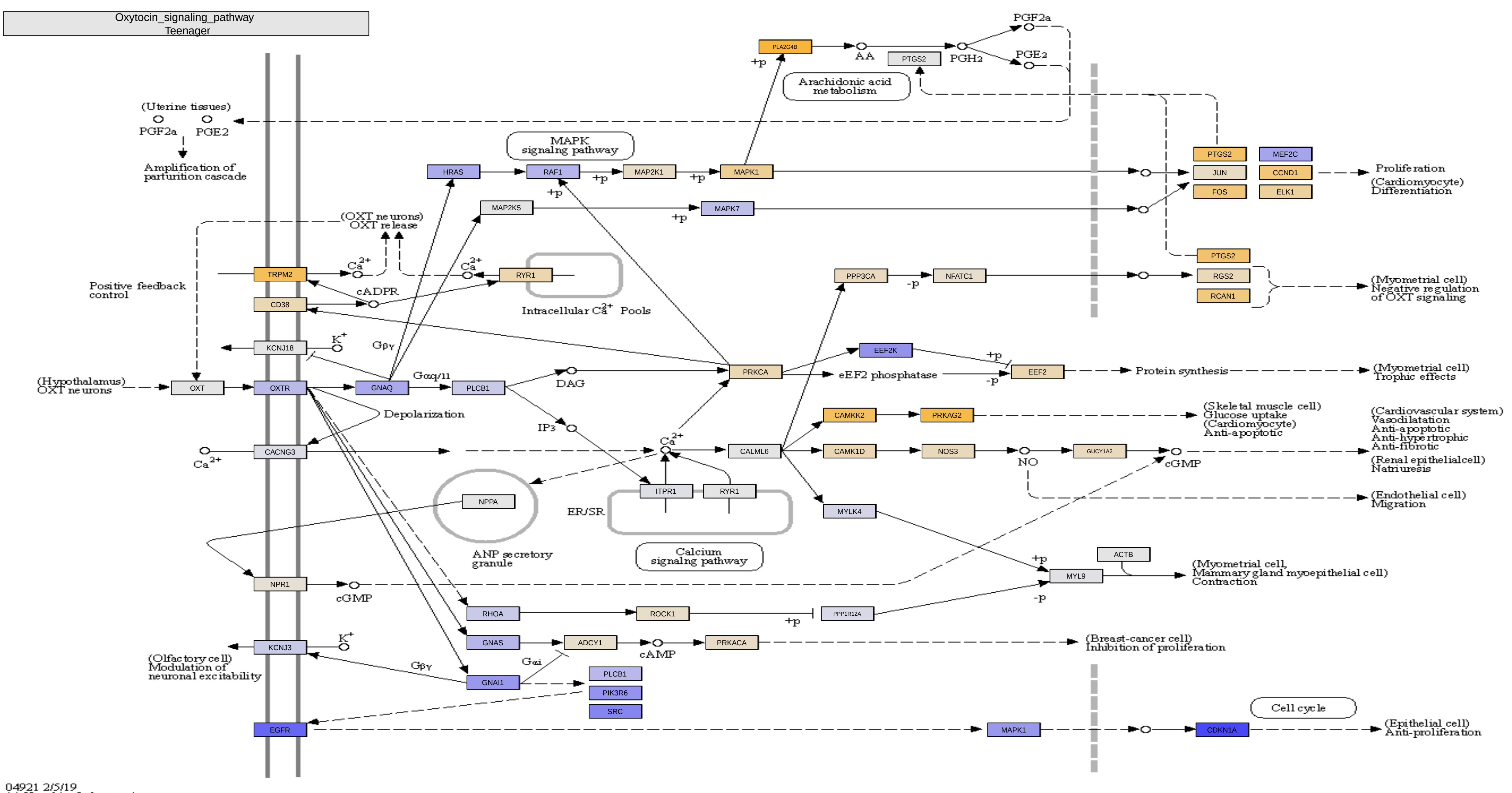

04921 2/5/19
(c) Kanehisa Laboratories

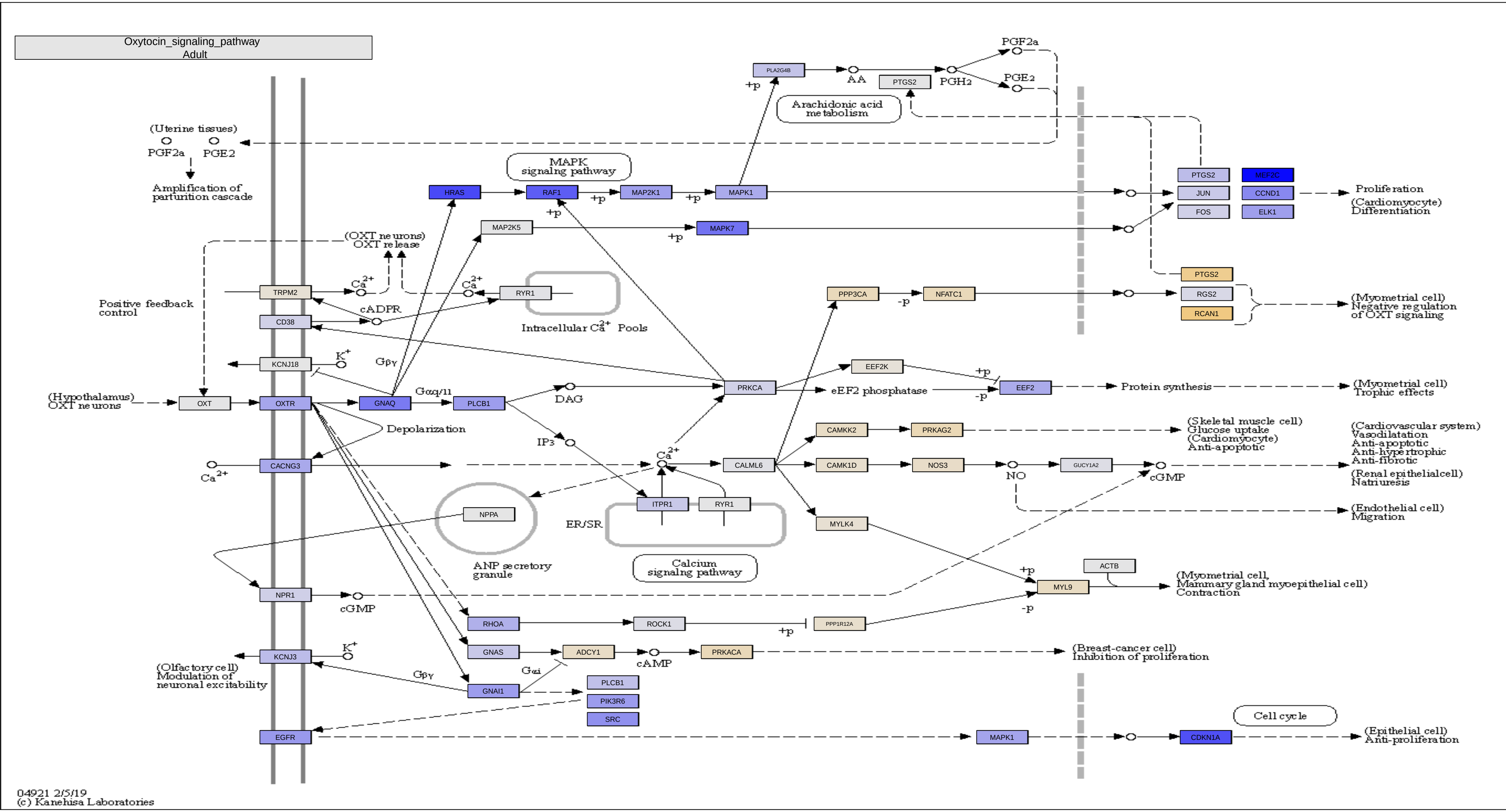
Oxytocin_signaling_pathway
Adult
(Uterine tissues)
PGF2a
PGE2
Amplification of parturition cascade
(OXT neurons) OXT release
Positive feedback control
(Hypothalamus) OXT neurons
OXT
TRPM2
CD38
KCNJ18
OXTR
CACNG3
NPR1
KCNJ3
EGFR
Ca2+
cADPR
K+
Gβγ
Gαq/11
GNAQ
PLCB1
DAG
IP3
Depolarization
RYR1
Intracellular Ca2+ Pools
HRAS
RAF1
MAP2K1
MAPK1
MAP2K5
MAPK7
MAPK signalng pathway
+p
-p
PLA2G4B
AA
PTGS2
PGH2
PGF2a
PGE2
Arachidonic acid metabolism
PTGS2
JUN
FOS
MEF2C
CCND1
ELK1
Proliferation
(Cardiomyocyte) Differentiation
PTGS2
RGS2
RCAN1
PPP3CA
NFATC1
(Myometrial cell) Negative regulation of OXT signaling
PRKCA
EEF2K
eEF2 phosphatase
EEF2
Protein synthesis
(Myometrial cell) Trophic effects
CAMKK2
PRKAG2
(Skeletal muscle cell) Glucose uptake
(Cardiomyocyte) Anti-apoptotic
CALML6
CAMK1D
NOS3
NO
GUCY1A2
cGMP
(Cardiovascular system) Vasodilatation
Anti-apoptotic
Anti-hypertrophic
Anti-fibrotic
(Renal epithelial cell) Natriuresis
(Endothelial cell) Migration
NPPA
ANP secretory granule
ITPR1
RYR1
ER/SR
Calcium signalng pathway
MYLK4
ACTB
MYL9
(Myometrial cell, Mammary gland myoepithelial cell) Contraction
RHOA
ROCK1
PPP1R12A
GNAS
ADCY1
cAMP
PRKACA
(Breast-cancer cell) Inhibition of proliferation
GNAI1
Gαi
PLCB1
PIK3R6
SRC
(Olfactory cell) Modulation of neuronal excitability
MAPK1
CDKN1A
Cell cycle
(Epithelial cell) Anti-proliferation
04921 2/5/19
(c) Kanehisa Laboratories

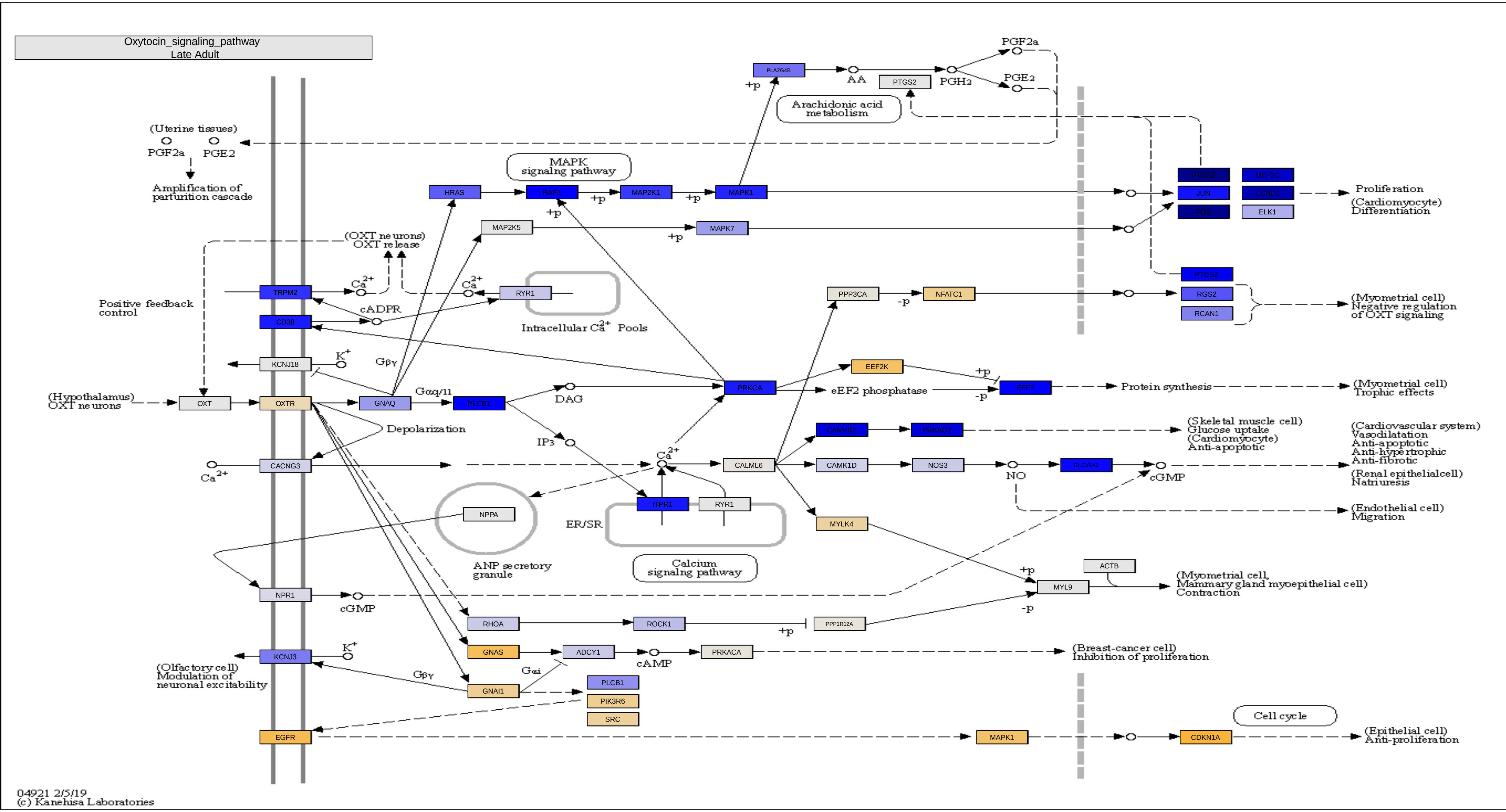
Oxytocin_signaling_pathway
Late Adult
(Uterine tissues)
PGF2a PGE2
Amplification of parturition cascade
PLA2G4B
+p
AA
PTGS2
PGH2
PGF2a
PGE2
Arachidonic acid metabolism
MAPK signalng pathway
HRAS
RAF1
+p
MAP2K1
+p
MAPK1
+p
MAP2K5
MAPK7
+p
JUN
FOS
MEF2C
CCND1
ELK1
Proliferation
(Cardiomyocyte) Differentiation
(OXT neurons) OXT release
Positive feedback control
TRPM2
CD38
Ca^{2+}
cADPR
Ca^{2+}
RYR1
Intracellular Ca^{2+} Pools
PPP3CA
-p
NFATC1
PTGS2
RGS2
RCAN1
(Myometrial cell) Negative regulation of OXT signaling
KCNJ18
K^{+}
Gβγ
(Hypothalamus) OXT neurons
OXT
OXTR
GNAQ
Gαq/11
PLCB1
DAG
PRKCA
EEF2K
+p
EEF2
eEF2 phosphatase
-p
Protein synthesis
(Myometrial cell) Trophic effects
Depolarization
IP3
CAMKK2
PRKAG1
(Skeletal muscle cell) Glucose uptake (Cardiomyocyte) Anti-apoptotic
(Cardiovascular system) Vasodilatation Anti-apoptotic Anti-hypertrophic Anti-fibrotic
CACNG3
Ca^{2+}
Ca^{2+}
CALML6
CAMK1D
NOS3
NO
GUCY1A2
cGMP
(Renal epithelialcell) Natriuresis
NPPA
ER/SR
ITPR1
RYR1
MYLK4
(Endothelial cell) Migration
ANP secretory granule
Calcium signalng pathway
ACTB
+p
MYL9
-p
(Myometrial cell, Mammary gland myoepithelial cell) Contraction
NPR1
cGMP
RHOA
ROCK1
+p
PPP1R12A
KCNJ3
K^{+}
GNAS
ADCY1
cAMP
PRKACA
(Breast-cancer cell) Inhibition of proliferation
(Olfactory cell) Modulation of neuronal excitability
Gβγ
Gαi
GNAI1
PLCB1
PIK3R6
SRC
EGFR
MAPK1
CDKN1A
Cell cycle
(Epithelial cell) Anti-proliferation
04921 2/5/19
(c) Kanehisa Laboratories
