# Supplementary material for: The Transcriptome and Methylome of the Developing and Aging Brain and Their Relations to Gliomas and Psychological Disorders
Source: Cells. 2022 Jan 21;11(3):362. doi: 10.3390/cells11030362 (PMC8834030; doi:10.3390/cells11030362)
Supplement: Supplementary file 1 [file cells-11-00362-s001.zip › Suppl-Material-S3-Pathways-PSF_Expression/Phospholipase_D_signaling_pathway.pdf]

Phospholipase D signaling pathway

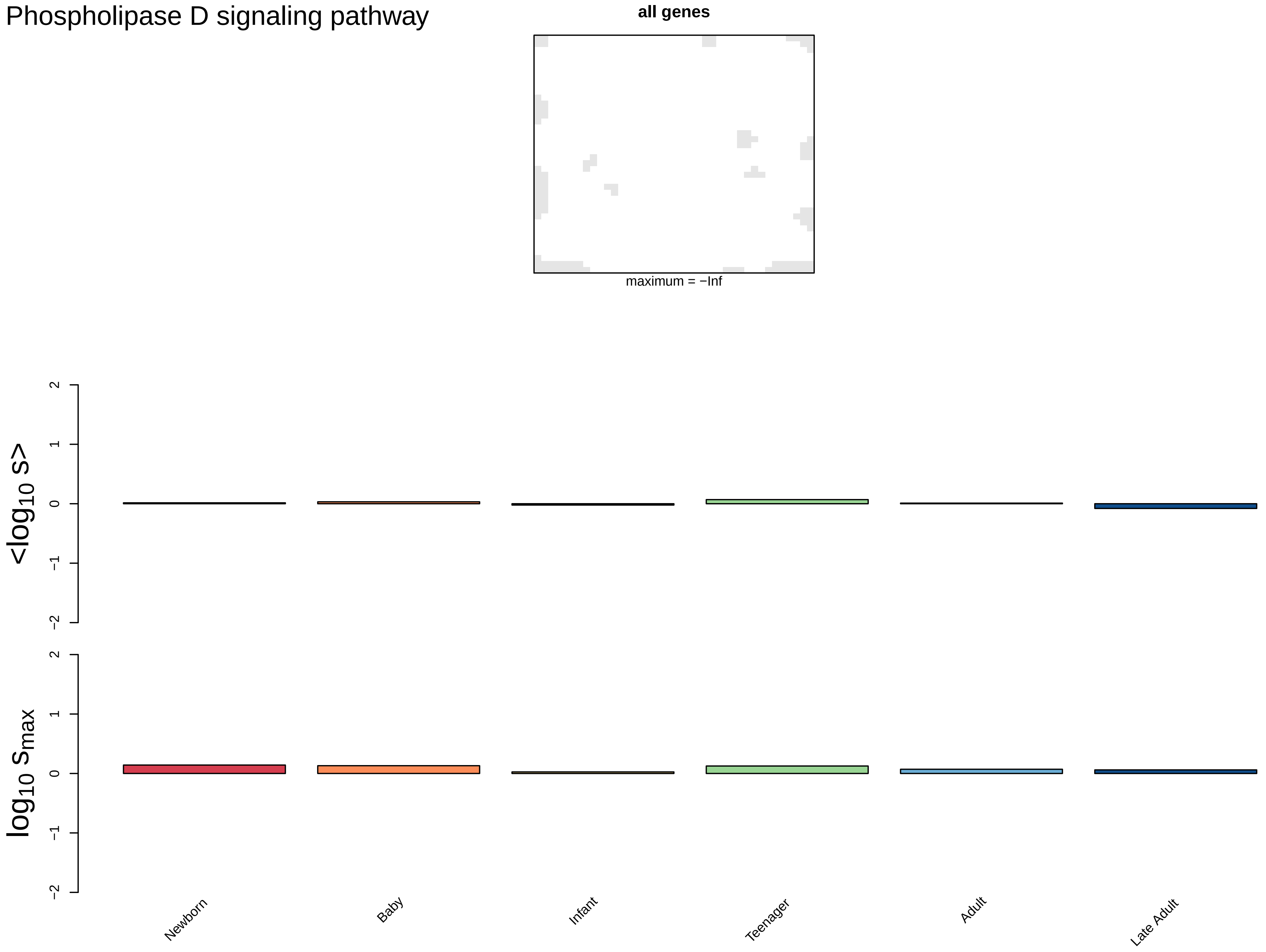

Phospholipase_D_signaling_pathway
genes with data

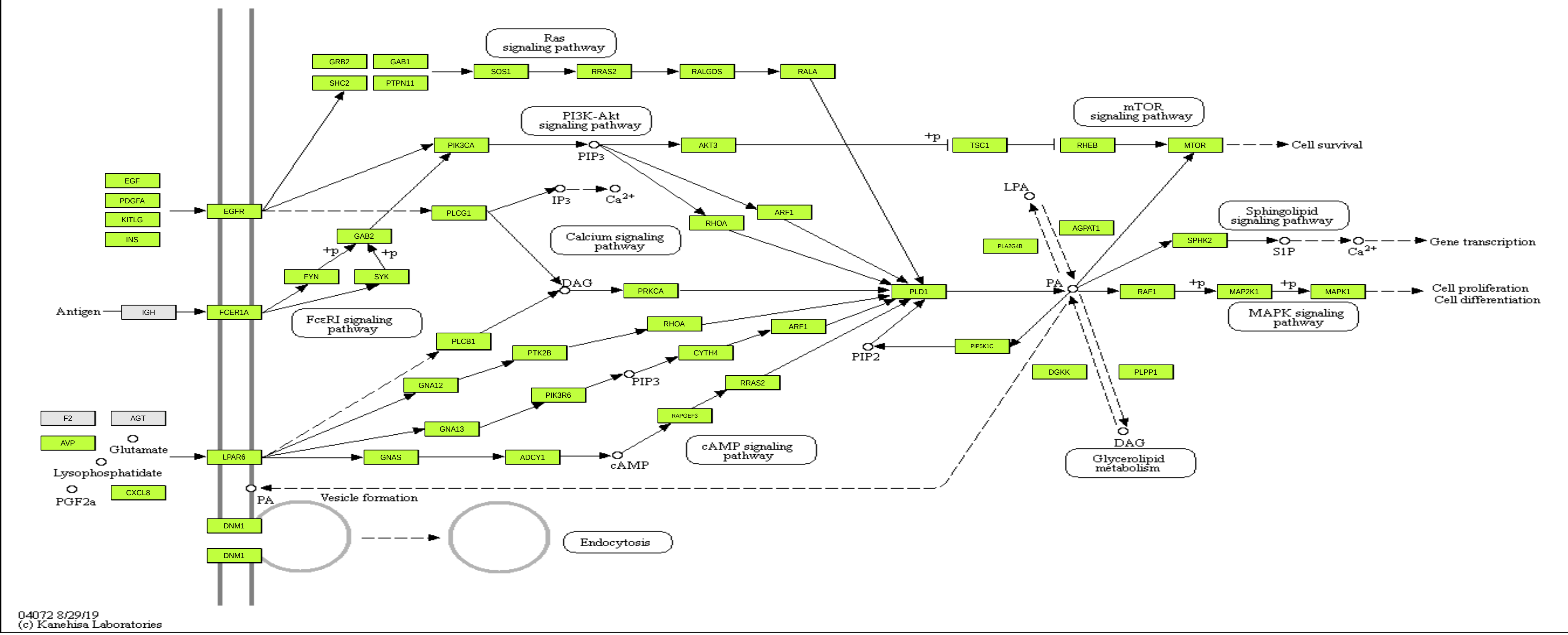

Phospholipase_D_signaling_pathway
sink nodes

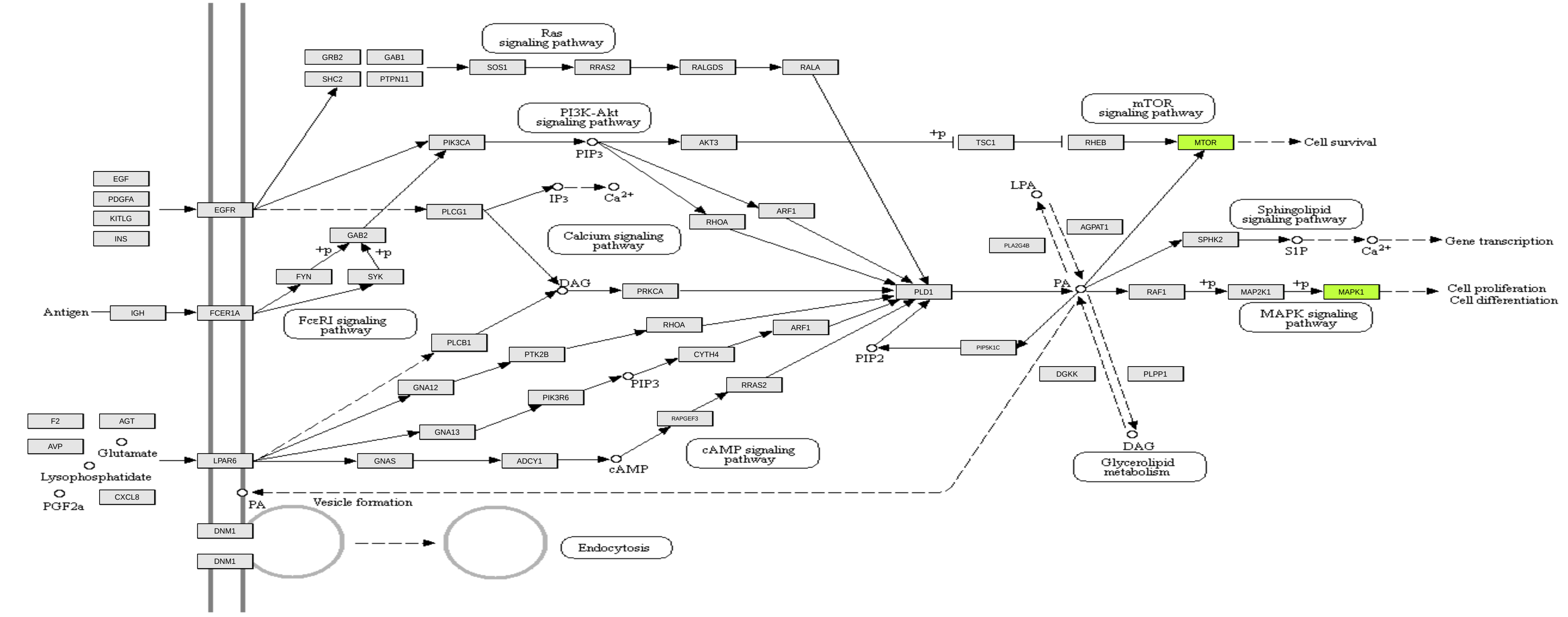

04072 8/29/19
(c) Kanehisa Laboratories

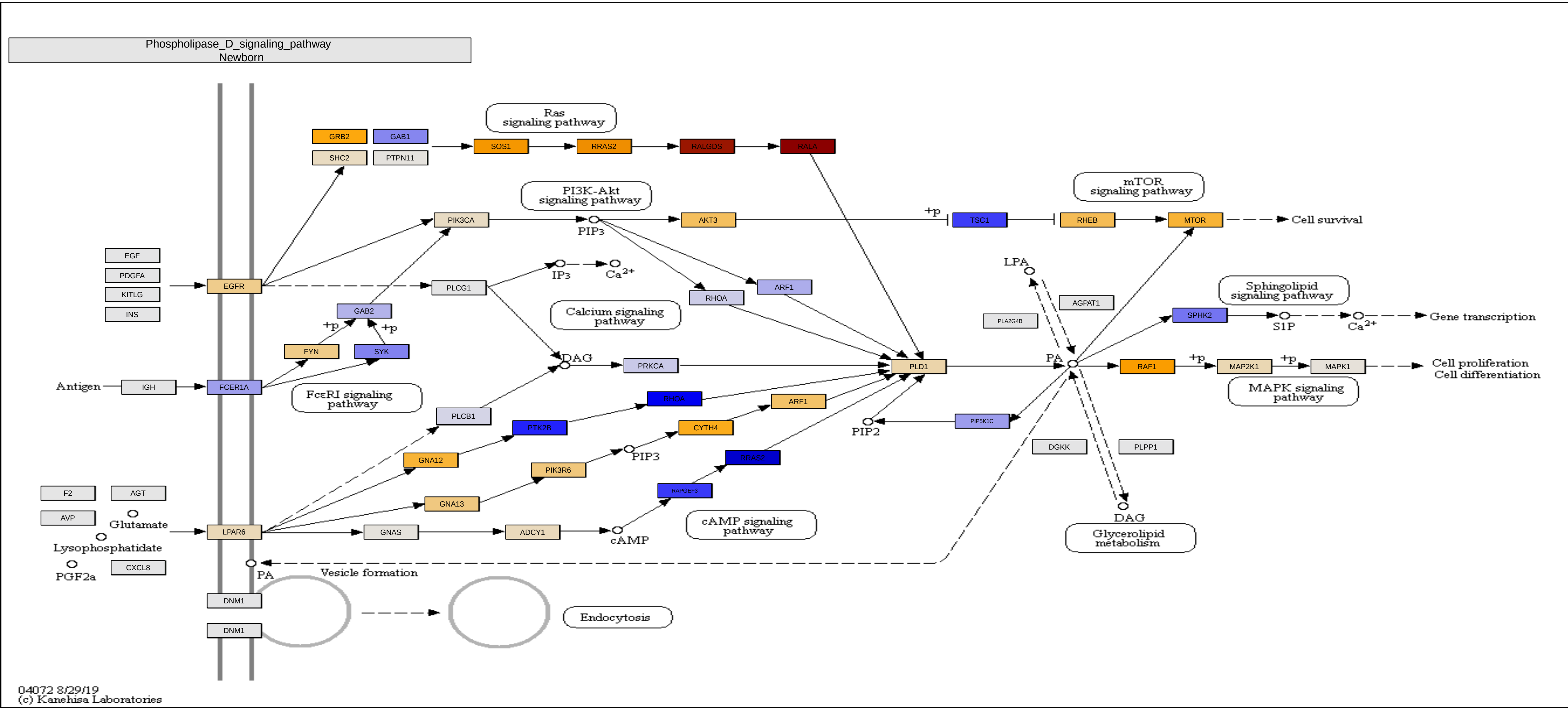

Phospholipase_D_signaling_pathway
Newborn
Ras
signaling pathway
GRB2
GAB1
SHC2
PTPN11
SOS1
RRAS2
RALGDS
RALA
PI3K-Akt
signaling pathway
PIK3CA
PIP3
AKT3
+p
TSC1
RHEB
MTOR
mTOR
signaling pathway
Cell survival
EGF
PDGFA
KITLG
INS
EGFR
PLCG1
IP3
Ca2+
RHOA
ARF1
LPA
AGPAT1
PLA2G4B
Sphingolipid
signaling pathway
SPHK2
S1P
Ca2+
Gene transcription
GAB2
+p
+p
FYN
SYK
Calcium signaling
pathway
DAG
PRKCA
PLD1
PA
RAF1
+p
MAP2K1
+p
MAPK1
Cell proliferation
Cell differentiation
Antigen
IGH
FCER1A
FcεRI signaling
pathway
PLCB1
RHOA
ARF1
PTK2B
CYTH4
PIP2
PIP5K1C
MAPK signaling
pathway
GNA12
PIP3
PIK3R6
RRAS2
DGKK
PLPP1
F2
AGT
AVP
Glutamate
GNA13
RAPGEF3
DAG
Lysophosphatidate
LPAR6
GNAS
ADCY1
cAMP
cAMP signaling
pathway
Glycerolipid
metabolism
PGF2a
CXCL8
PA
Vesicle formation
DNM1
Endocytosis
DNM1
04072 8/29/19
(c) Kanehisa Laboratories

Phospholipase_D_signaling_pathway
Baby

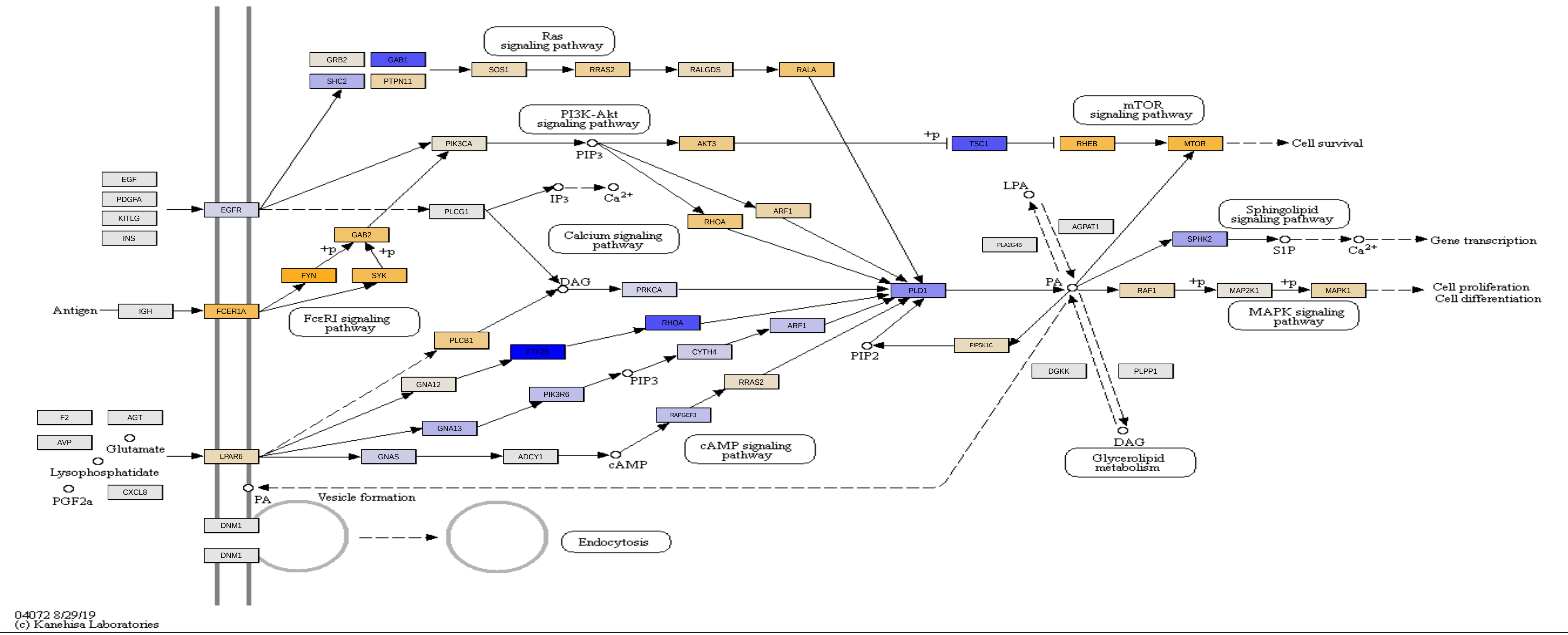

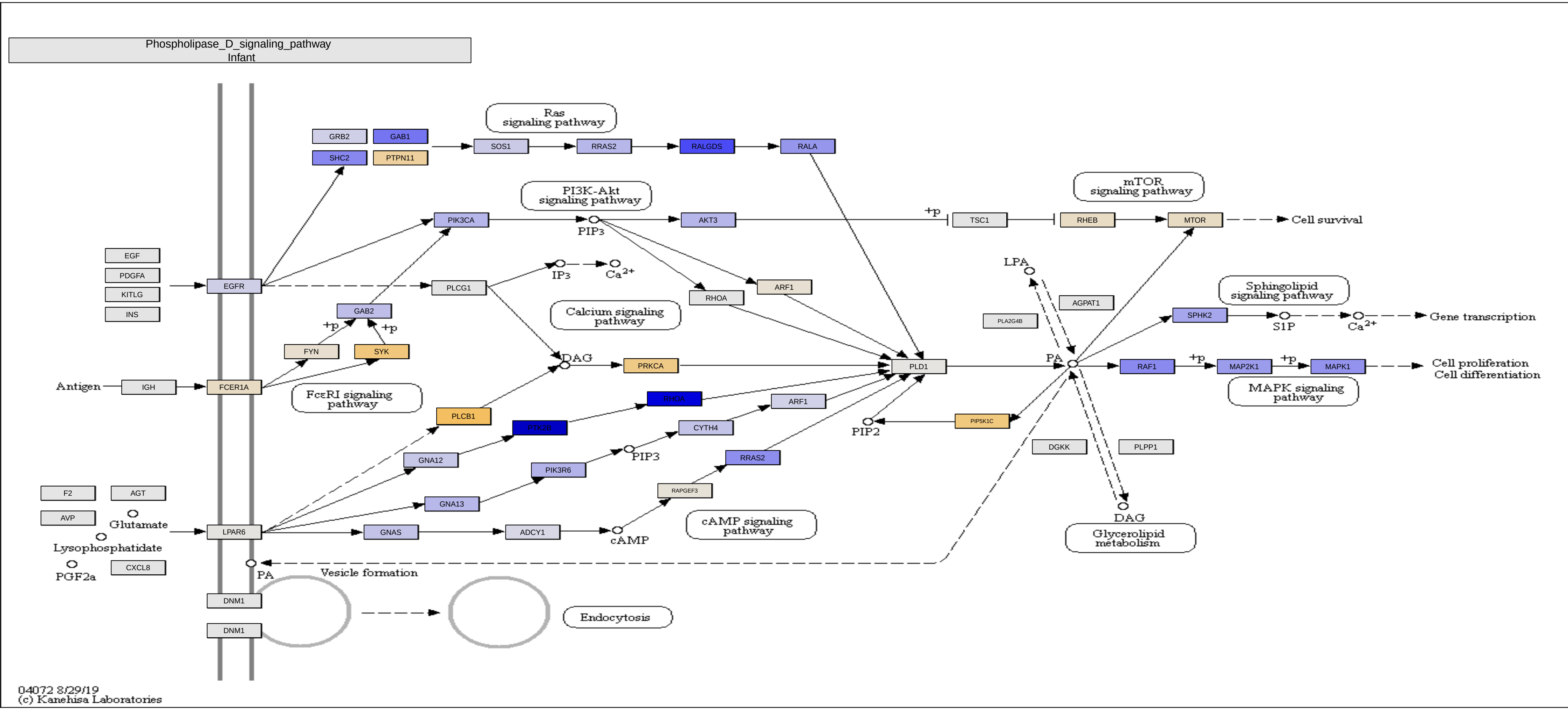
Phospholipase_D_signaling_pathway
Infant
Ras signaling pathway
GRB2
GAB1
SHC2
PTPN11
SOS1
RRAS2
RALGDS
RALA
PI3K-Akt signaling pathway
PIK3CA
PIP3
AKT3
+p
TSC1
RHEB
MTOR
mTOR signaling pathway
Cell survival
EGF
PDGFA
KITLG
INS
EGFR
PLCG1
IP3
Ca2+
RHOA
ARF1
LPA
AGPAT1
PLA2G4B
Sphingolipid signaling pathway
SPHK2
S1P
Ca2+
Gene transcription
GAB2
+p
+p
FYN
SYK
Calcium signaling pathway
DAG
PRKCA
PLD1
PA
RAF1
+p
MAP2K1
+p
MAPK1
Cell proliferation
Cell differentiation
MAPK signaling pathway
Antigen
IGH
FCER1A
FcεRI signaling pathway
PLCB1
RHOA
PTK2B
ARF1
CYTH4
PIP2
PIP5K1C
DGKK
PLPP1
GNA12
PIK3R6
PIP3
RRAS2
RAPGEF3
F2
AGT
AVP
Glutamate
Lysophosphatidate
PGF2a
CXCL8
LPAR6
GNA13
GNAS
ADCY1
cAMP
cAMP signaling pathway
DAG
Glycerolipid metabolism
PA
Vesicle formation
DNM1
DNM1
Endocytosis
04072 8/29/19
(c) Kanehisa Laboratories

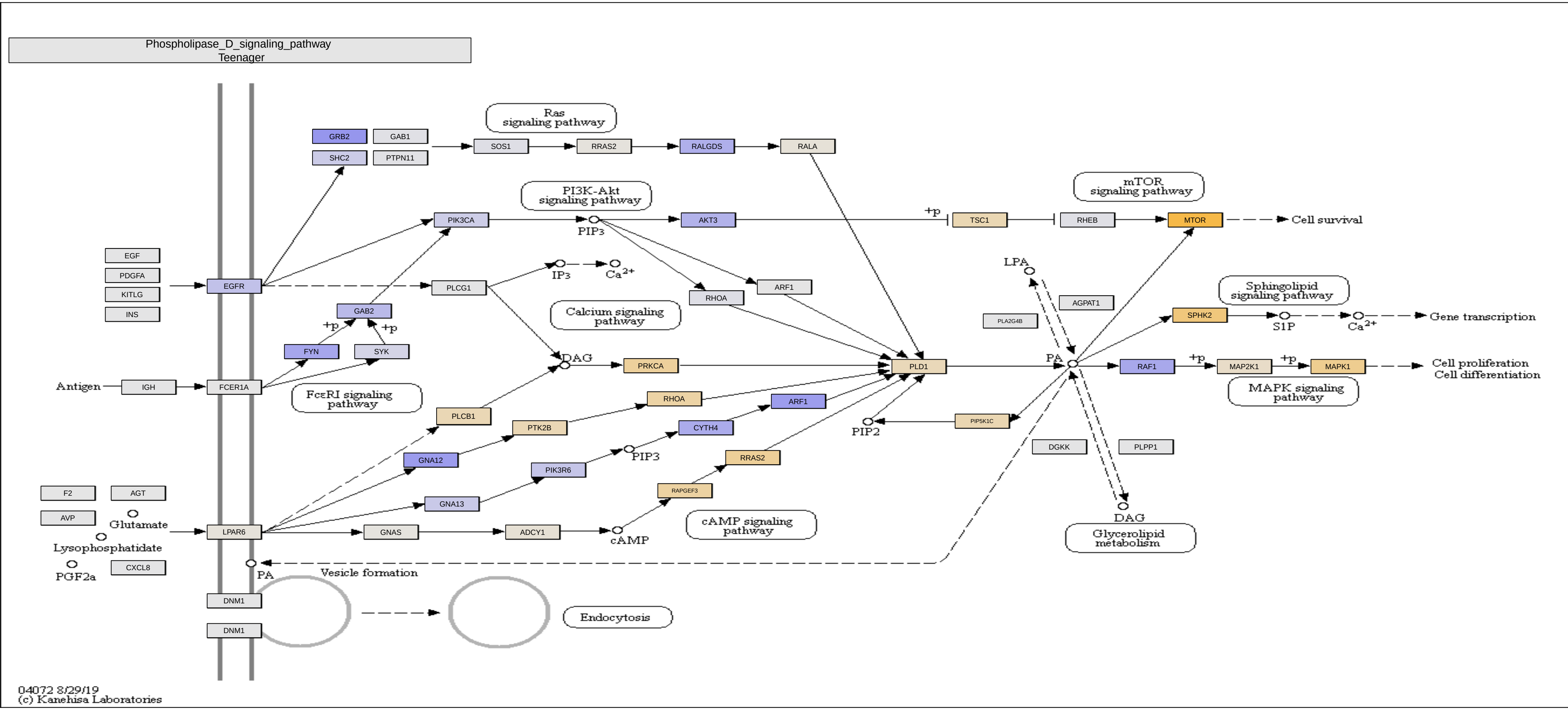
Phospholipase_D_signaling_pathway
Teenager
Ras signaling pathway
GRB2
GAB1
SHC2
PTPN11
SOS1
RRAS2
RALGDS
RALA
PI3K-Akt signaling pathway
PIK3CA
PIP3
AKT3
+p
TSC1
RHEB
MTOR
Cell survival
mTOR signaling pathway
EGF
PDGFA
KITLG
INS
EGFR
PLCG1
IP3
Ca2+
GAB2
+p
FYN
SYK
Calcium signaling pathway
RHOA
ARF1
DAG
PRKCA
PLD1
LPA
AGPAT1
PLA2G4B
PA
Sphingolipid signaling pathway
SPHK2
S1P
Ca2+
Gene transcription
RAF1
MAP2K1
MAPK1
Cell proliferation
Cell differentiation
MAPK signaling pathway
Antigen
IGH
FCER1A
FcεRI signaling pathway
PLCB1
RHOA
ARF1
PTK2B
CYTH4
PIP2
PIP5K1C
GNA12
PIP3
PIK3R6
RRAS2
DGKK
PLPP1
RAPGEF3
F2
AGT
AVP
Glutamate
Lysophosphatidate
PGF2a
CXCL8
GNA13
LPAR6
GNAS
ADCY1
cAMP
cAMP signaling pathway
DAG
Glycerolipid metabolism
PA
Vesicle formation
DNM1
DNM1
Endocytosis
04072 8/29/19
(c) Kanehisa Laboratories

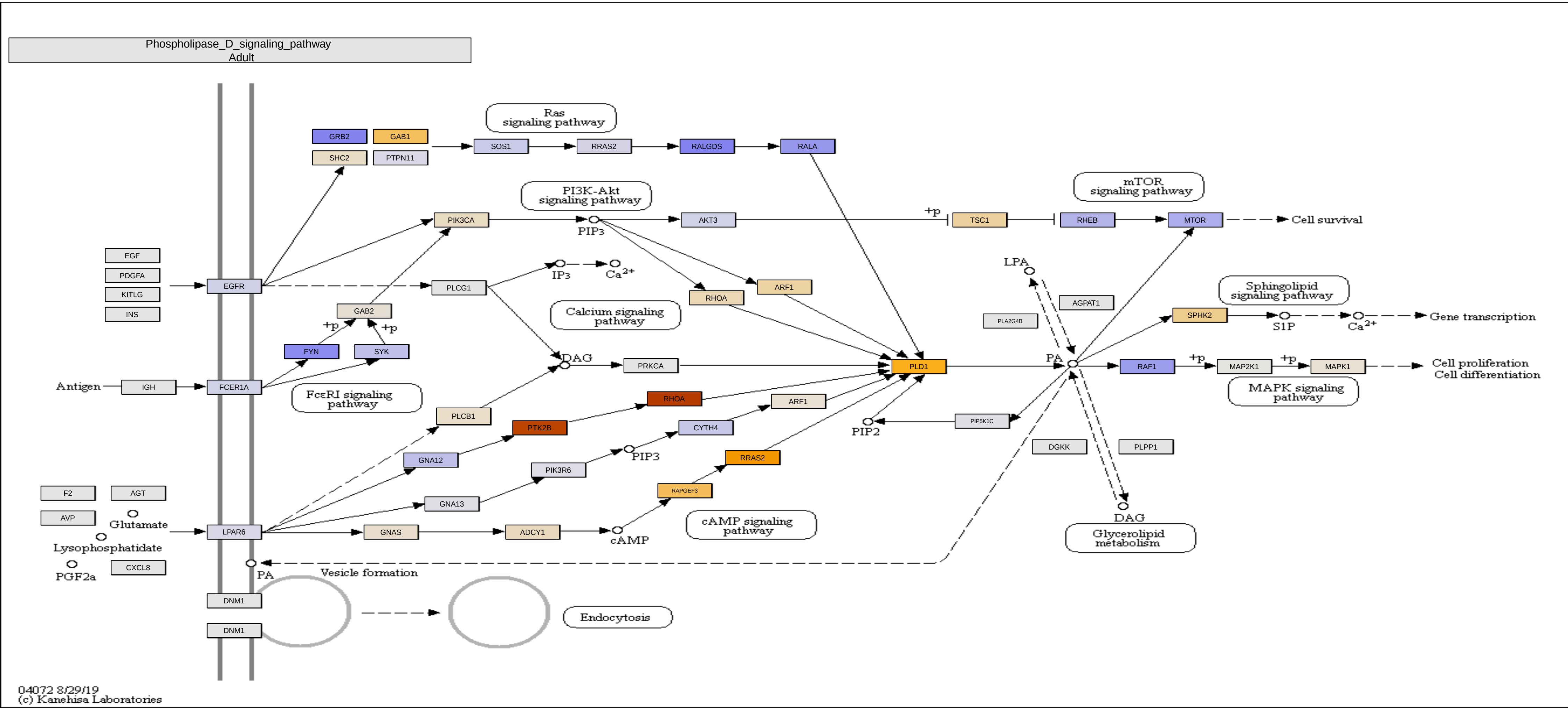

Phospholipase_D_signaling_pathway
Adult
Ras signaling pathway
GRB2
GAB1
SHC2
PTPN11
SOS1
RRAS2
RALGDS
RALA
PI3K-Akt signaling pathway
PIK3CA
PIP3
AKT3
+p
TSC1
RHEB
MTOR
Cell survival
mTOR signaling pathway
EGF
PDGFA
KITLG
INS
EGFR
PLCG1
IP3
Ca2+
GAB2
+p
FYN
SYK
Calcium signaling pathway
RHOA
ARF1
DAG
PRKCA
PLD1
LPA
AGPAT1
PLA2G4B
PA
Sphingolipid signaling pathway
SPHK2
S1P
Gene transcription
RAF1
MAP2K1
MAPK1
Cell proliferation
Cell differentiation
MAPK signaling pathway
Antigen
IGH
FCER1A
FcεRI signaling pathway
PLCB1
RHOA
PTK2B
ARF1
CYTH4
GNA12
PIK3R6
PIP3
PIP2
PIP5K1C
DGKK
PLPP1
RRAS2
RAPGEF3
GNA13
F2
AGT
AVP
Glutamate
Lysophosphatidate
PGF2a
CXCL8
LPAR6
GNAS
ADCY1
cAMP
cAMP signaling pathway
DAG
Glycerolipid metabolism
PA
Vesicle formation
DNM1
DNM1
Endocytosis
04072 8/29/19
(c) Kanehisa Laboratories

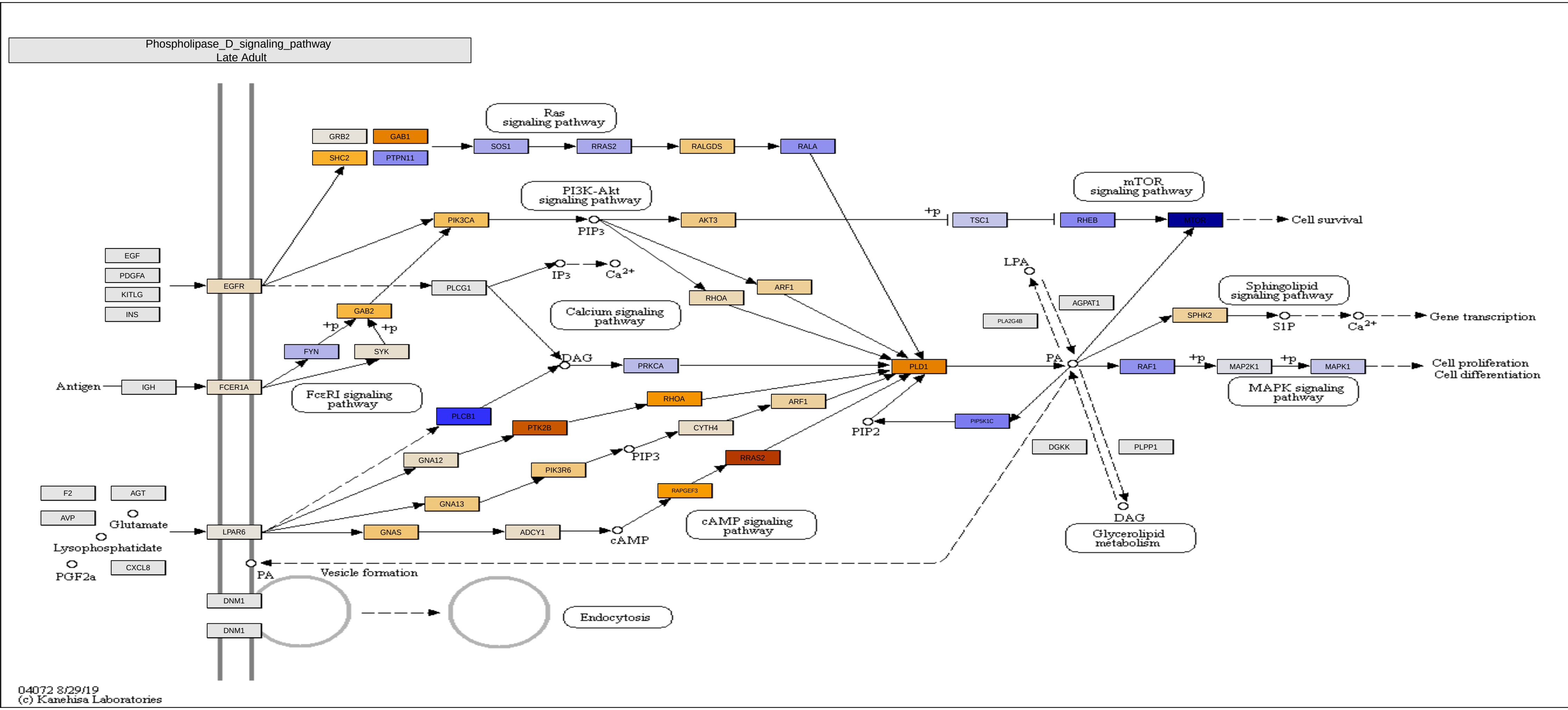
Phospholipase_D_signaling_pathway
Late Adult
Ras signaling pathway
GRB2
GAB1
SHC2
PTPN11
SOS1
RRAS2
RALGDS
RALA
PI3K-Akt signaling pathway
PIK3CA
PIP3
AKT3
+p
TSC1
RHEB
MTOR
Cell survival
mTOR signaling pathway
EGF
PDGFA
KITLG
INS
EGFR
PLCG1
IP3
Ca2+
GAB2
+p
+p
FYN
SYK
Calcium signaling pathway
RHOA
ARF1
DAG
PRKCA
PLD1
LPA
AGPAT1
PLA2G4B
PA
Sphingolipid signaling pathway
SPHK2
S1P
Ca2+
Gene transcription
RAF1
+p
MAP2K1
+p
MAPK1
Cell proliferation
Cell differentiation
MAPK signaling pathway
Antigen
IGH
FCER1A
FcεRI signaling pathway
PLCB1
RHOA
PTK2B
ARF1
GNA12
CYTH4
PIP3
PIK3R6
RRAS2
PIP2
PIP5K1C
DGKK
PLPP1
F2
AGT
AVP
Glutamate
Lysophosphatidate
PGF2a
CXCL8
LPAR6
GNA13
GNAS
ADCY1
cAMP
RAPGEF3
cAMP signaling pathway
DAG
Glycerolipid metabolism
PA
Vesicle formation
DNM1
DNM1
Endocytosis
04072 8/29/19
(c) Kanehisa Laboratories
