# Supplementary material for: The Transcriptome and Methylome of the Developing and Aging Brain and Their Relations to Gliomas and Psychological Disorders
Source: Cells. 2022 Jan 21;11(3):362. doi: 10.3390/cells11030362 (PMC8834030; doi:10.3390/cells11030362)
Supplement: Supplementary file 1 [file cells-11-00362-s001.zip › Suppl-Material-S3-Pathways-PSF_Expression/PI3K_Akt_signaling_pathway.pdf]

PI3K−Akt signaling pathway

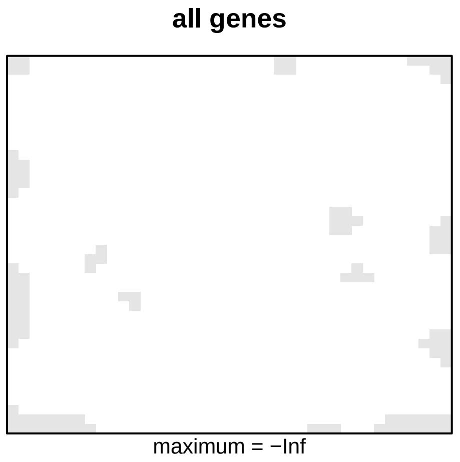

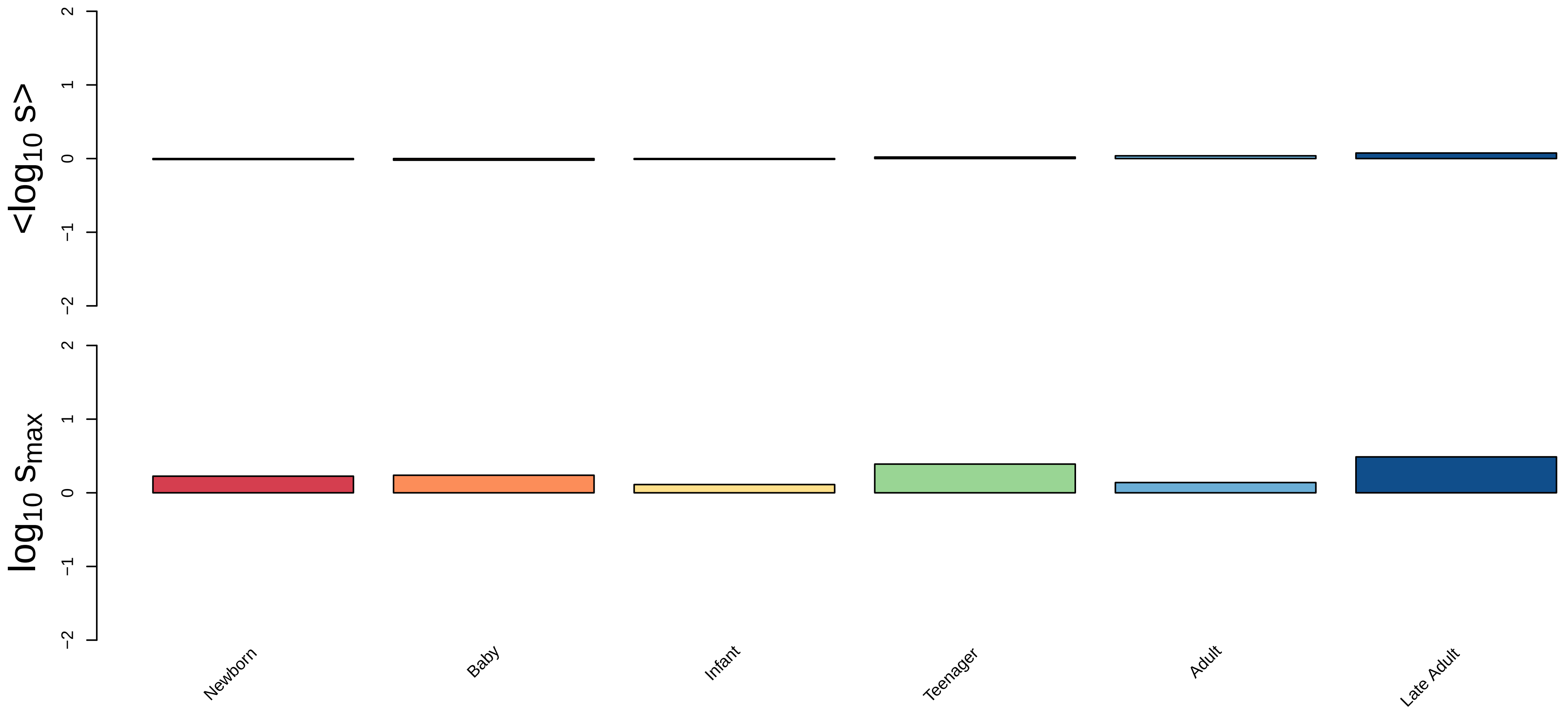

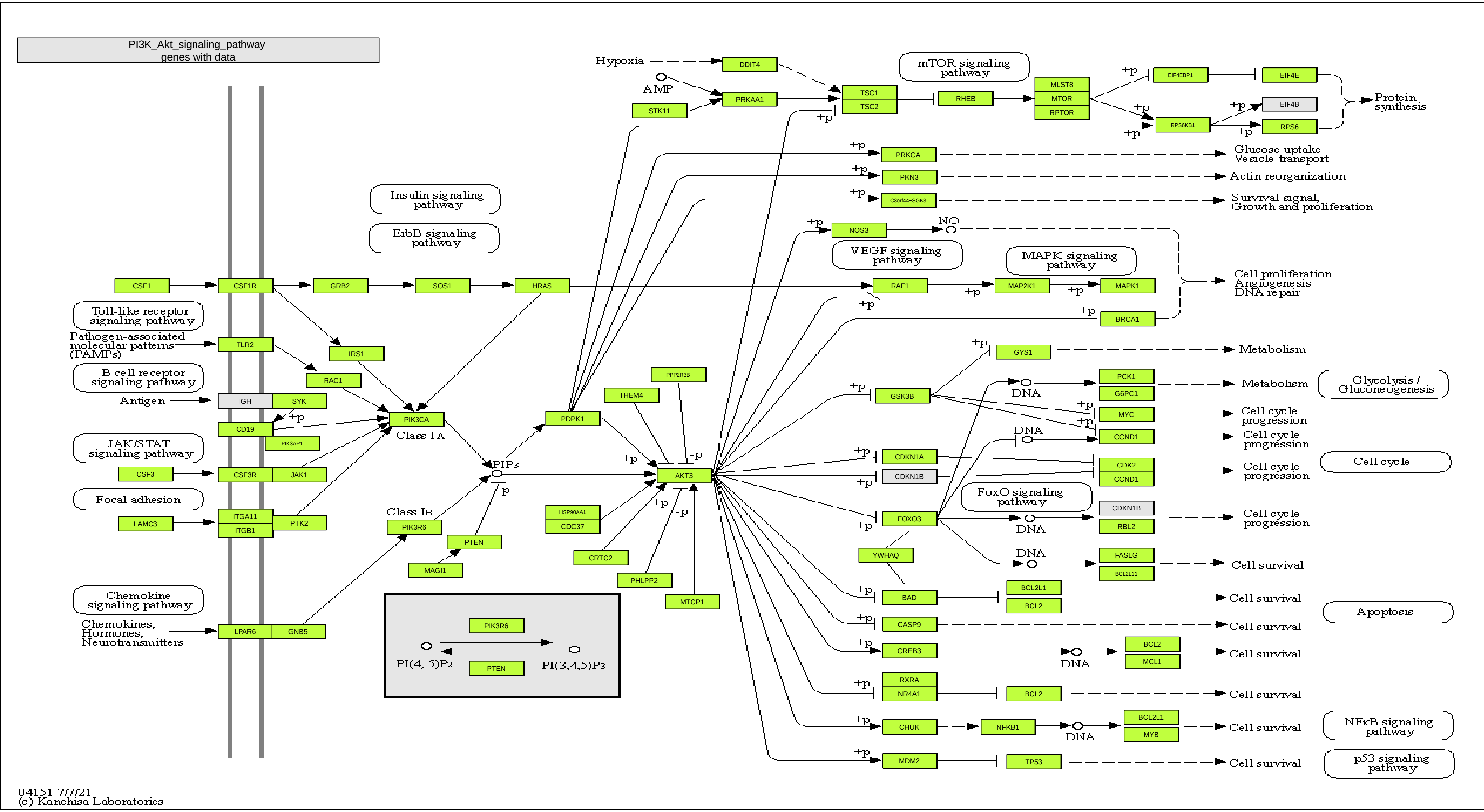

PI3K_Akt_signaling_pathway
genes with data
Hypoxia
DDIT4
AMP
PRKAA1
STK11
TSC1
TSC2
mTOR signaling pathway
RHEB
MLST8
MTOR
RPTOR
EIF4EBP1
EIF4E
EIF4B
RPS6KB1
RPS6
Protein synthesis
PRKCA
Glucose uptake
Vesicle transport
PKN3
Actin reorganization
C8orf44-SGK3
Survival signal,
Growth and proliferation
Insulin signaling pathway
ErbB signaling pathway
NOS3
NO
VEGF signaling pathway
MAPK signaling pathway
Cell proliferation
Angiogenesis
DNA repair
CSF1
CSF1R
GRB2
SOS1
HRAS
RAF1
MAP2K1
MAPK1
BRCA1
Toll-like receptor signaling pathway
Pathogen-associated molecular patterns (PAMPs)
TLR2
IRS1
RAC1
GYS1
Metabolism
B cell receptor signaling pathway
Antigen
IGH
SYK
CD19
PIK3AP1
PIK3CA
Class IA
PDPK1
THEM4
PPP2R3B
GSK3B
PCK1
G6PC1
Glycolysis / Gluconeogenesis
MYC
Cell cycle progression
CCND1
JAK/STAT signaling pathway
CSF3
CSF3R
JAK1
PIP3
AKT3
CDKN1A
CDKN1B
CDK2
Cell cycle
Focal adhesion
LAMC3
ITGA11
ITGB1
PTK2
Class IB
PIK3R6
HSP90AA1
CDC37
FoxO signaling pathway
FOXO3
RBL2
YWHAQ
FASLG
BCL2L11
Cell survival
PTEN
MAGI1
CRTC2
PHLPP2
MTCP1
BAD
BCL2L1
BCL2
Apoptosis
Chemokine signaling pathway
Chemokines, Hormones, Neurotransmitters
LPAR6
GNB5
PI(4, 5)P2
PI(3,4,5)P3
CASP9
CREB3
MCL1
RXRA
NR4A1
CHUK
NFKB1
MYB
NFκB signaling pathway
MDM2
TP53
p53 signaling pathway
DNA
+p
-p
04151 7/7/21
(c) Kanehisa Laboratories

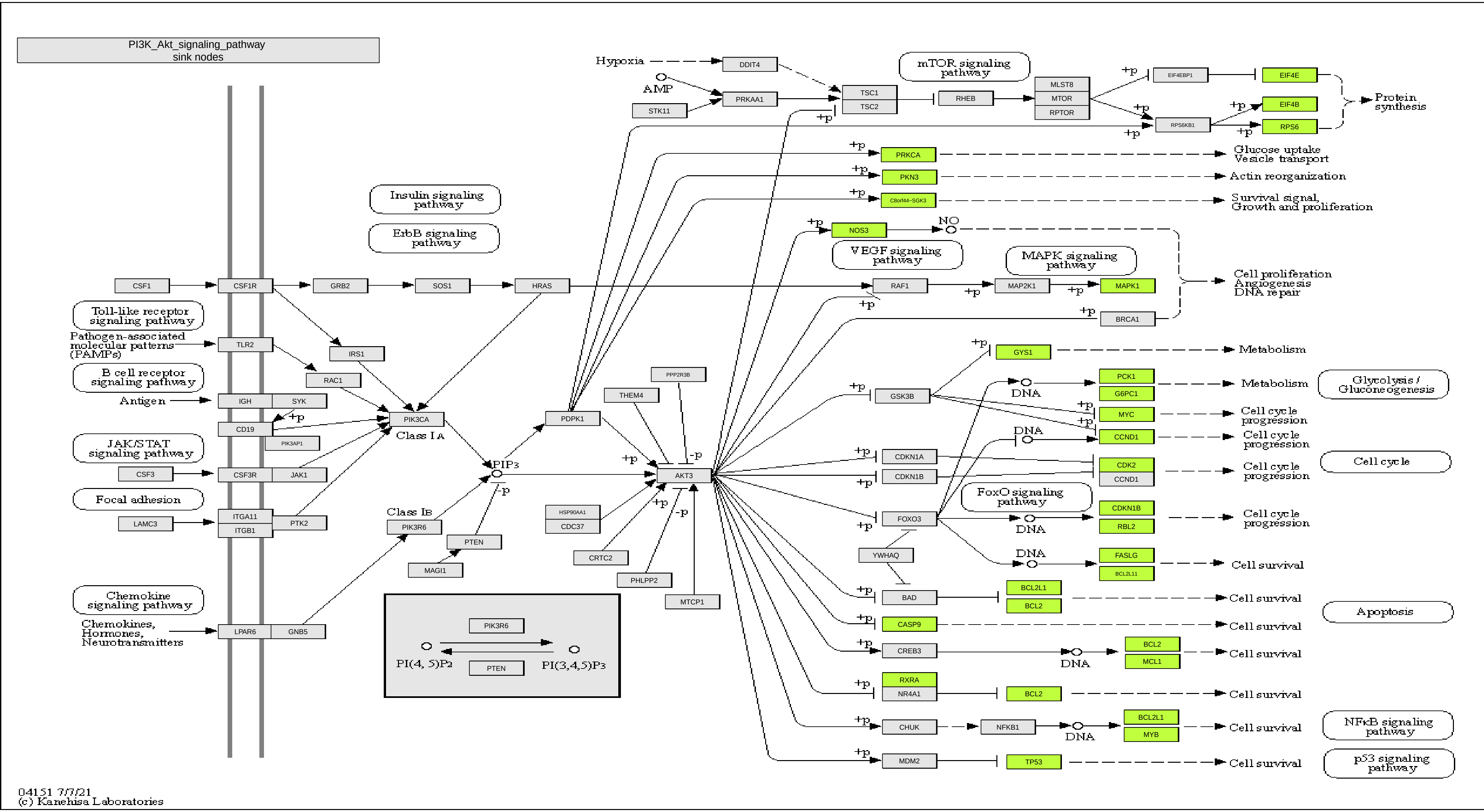
PI3K_Akt_signaling_pathway
sink nodes
Hypoxia
DDIT4
AMP
PRKAA1
STK11
TSC1
TSC2
mTOR signaling pathway
RHEB
MLST8
MTOR
RPTOR
EIF4EBP1
EIF4E
EIF4B
RPS6KB1
RPS6
Protein synthesis
PRKCA
Glucose uptake
Vesicle transport
PKN3
Actin reorganization
C8orf44-SGK3
Survival signal,
Growth and proliferation
Insulin signaling pathway
ErbB signaling pathway
NOS3
NO
VEGF signaling pathway
MAPK signaling pathway
CSF1
CSF1R
GRB2
SOS1
HRAS
RAF1
MAP2K1
MAPK1
Cell proliferation
Angiogenesis
DNA repair
BRCA1
Toll-like receptor signaling pathway
Pathogen-associated molecular patterns (PAMPs)
TLR2
IRS1
RAC1
B cell receptor signaling pathway
Antigen
IGH
SYK
CD19
PIK3AP1
PIK3CA
Class IA
JAK/STAT signaling pathway
CSF3
CSF3R
JAK1
Focal adhesion
LAMC3
ITGA11
ITGB1
PTK2
Class IB
PIK3R6
PIP3
PTEN
MAGI1
PDPK1
THEM4
PPP2R3B
AKT3
HSP90AA1
CDC37
CRTC2
PHLPP2
MTCP1
GYS1
Metabolism
GSK3B
DNA
PCK1
G6PC1
Glycolysis / Gluconeogenesis
MYC
Cell cycle progression
CCND1
CDKN1A
CDKN1B
CDK2
Cell cycle
FoxO signaling pathway
FOXO3
CDKN1B
RBL2
YWHAQ
FASLG
BCL2L11
Cell survival
BAD
BCL2L1
BCL2
CASP9
Apoptosis
CREB3
MCL1
RXRA
NR4A1
CHUK
NFKB1
MYB
NFκB signaling pathway
MDM2
TP53
p53 signaling pathway
Chemokine signaling pathway
Chemokines, Hormones, Neurotransmitters
LPAR6
GNB5
PI(4, 5)P2
PI(3,4,5)P3
+p
-p
04151 7/7/21
(c) Kanehisa Laboratories

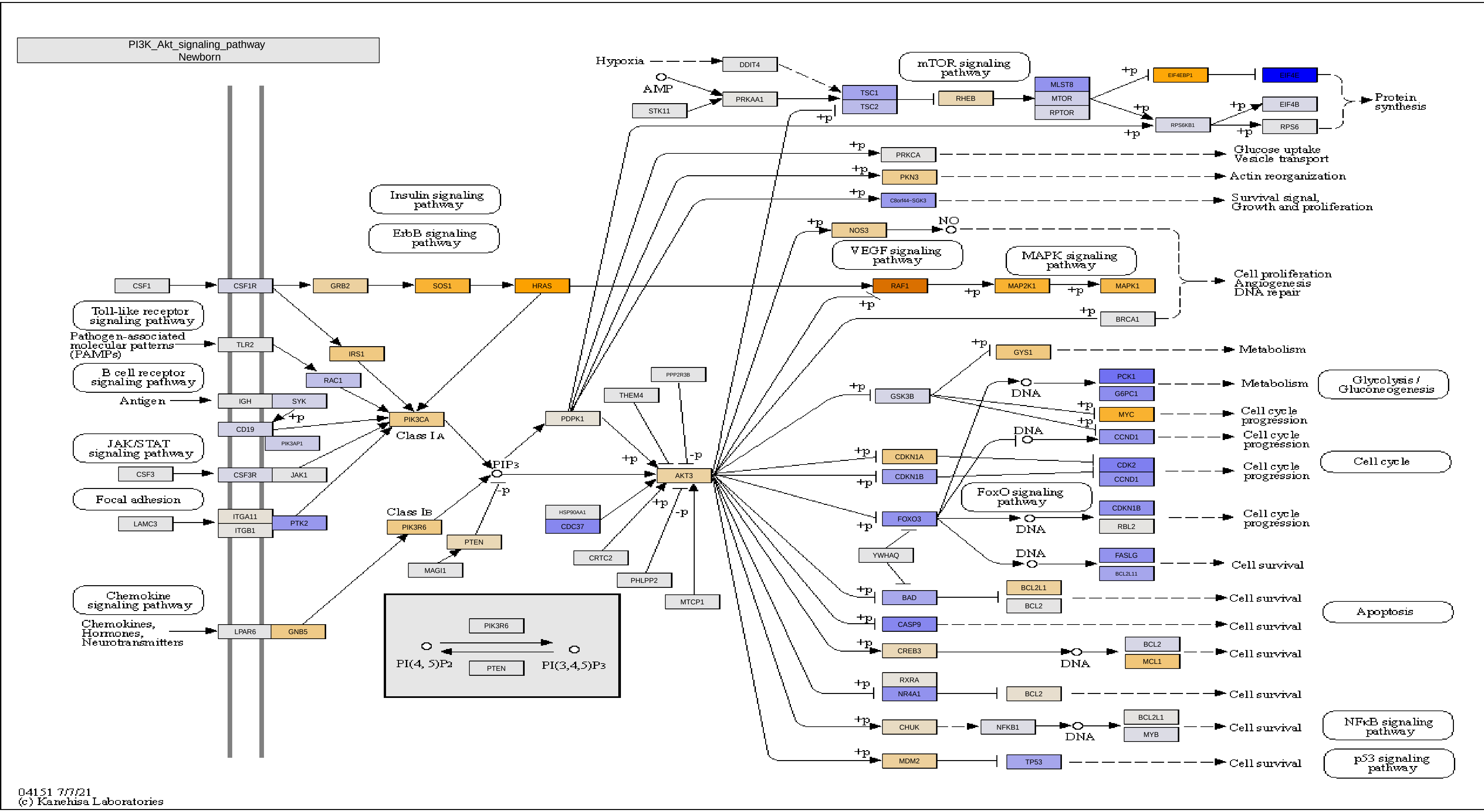
PI3K_Akt_signaling_pathway
Newborn
Hypoxia
DDIT4
AMP
PRKAA1
STK11
mTOR signaling pathway
TSC1
TSC2
RHEB
MLST8
MTOR
RPTOR
EIF4EBP1
EIF4E
EIF4B
RPS6KB1
RPS6
Protein synthesis
PRKCA
Glucose uptake Vesicle transport
PKN3
Actin reorganization
C8orf44-SGK3
Survival signal, Growth and proliferation
Insulin signaling pathway
ErbB signaling pathway
NOS3
NO
VEGF signaling pathway
MAPK signaling pathway
Cell proliferation Angiogenesis DNA repair
CSF1
CSF1R
GRB2
SOS1
HRAS
RAF1
MAP2K1
MAPK1
BRCA1
Toll-like receptor signaling pathway
Pathogen-associated molecular patterns (PAMPs)
TLR2
IRS1
RAC1
B cell receptor signaling pathway
Antigen
IGH
SYK
CD19
PIK3AP1
PIK3CA
Class IA
GYS1
Metabolism
GSK3B
PCK1
G6PC1
Metabolism
Glycolysis / Gluconeogenesis
MYC
Cell cycle progression
CCND1
Cell cycle progression
DNA
JAK/STAT signaling pathway
CSF3
CSF3R
JAK1
PIP3
PDPK1
THEM4
PPP2R3B
AKT3
CDKN1A
CDKN1B
CDK2
CCND1
Cell cycle progression
Cell cycle
Focal adhesion
ITGA11
ITGB1
PTK2
LAMC3
Class IB
PIK3R6
PTEN
MAGI1
HSP90AA1
CDC37
CRTC2
PHLPP2
MTCP1
FoxO signaling pathway
FOXO3
CDKN1B
RBL2
Cell cycle progression
YWHAQ
FASLG
BCL2L11
Cell survival
BAD
BCL2L1
BCL2
Cell survival
Apoptosis
CASP9
Cell survival
CREB3
BCL2
MCL1
Cell survival
RXRA
NR4A1
BCL2
Cell survival
CHUK
NFKB1
BCL2L1
MYB
Cell survival
NFκB signaling pathway
MDM2
TP53
Cell survival
p53 signaling pathway
Chemokine signaling pathway
Chemokines, Hormones, Neurotransmitters
LPAR6
GNB5
PIK3R6
PI(4,5)P2
PTEN
PI(3,4,5)P3
04151 7/7/21
(c) Kanehisa Laboratories

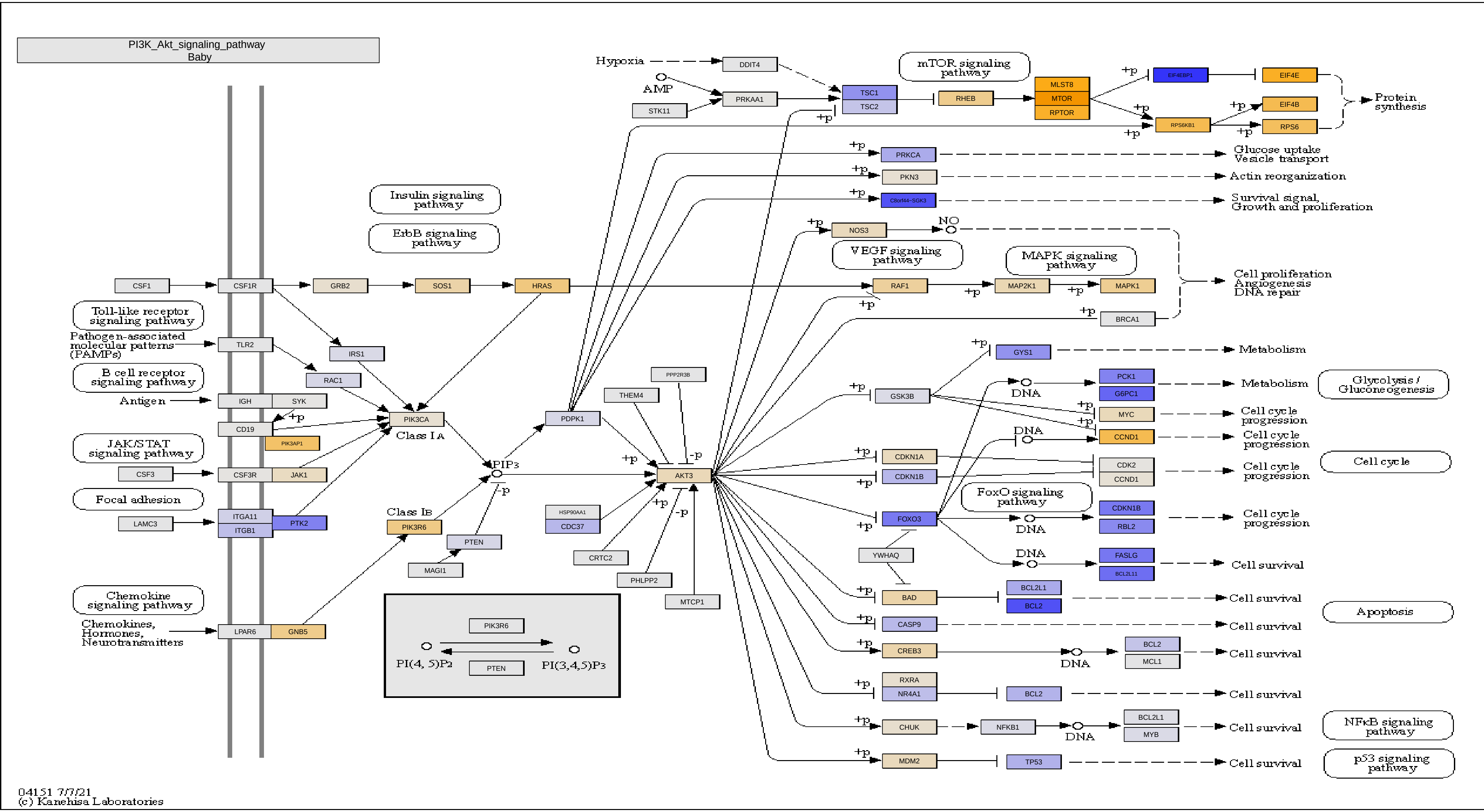
PI3K_Akt_signaling_pathway
Baby
Hypoxia
DDIT4
AMP
PRKAA1
STK11
mTOR signaling pathway
TSC1
TSC2
RHEB
MLST8
MTOR
RPTOR
EIF4EBP1
EIF4E
EIF4B
RPS6KB1
RPS6
Protein synthesis
+p
PRKCA
Glucose uptake
Vesicle transport
PKN3
Actin reorganization
C8orf44-SGK3
Survival signal, Growth and proliferation
Insulin signaling pathway
ErbB signaling pathway
NOS3
NO
VEGF signaling pathway
MAPK signaling pathway
CSF1
CSF1R
GRB2
SOS1
HRAS
RAF1
MAP2K1
MAPK1
Cell proliferation
Angiogenesis
DNA repair
BRCA1
Toll-like receptor signaling pathway
Pathogen-associated molecular patterns (PAMPs)
TLR2
IRS1
RAC1
B cell receptor signaling pathway
Antigen
IGH
SYK
CD19
PIK3AP1
PIK3CA
Class IA
JAK/STAT signaling pathway
CSF3
CSF3R
JAK1
Focal adhesion
LAMC3
ITGA11
ITGB1
PTK2
Chemokine signaling pathway
Chemokines, Hormones, Neurotransmitters
LPAR6
GNB5
Class IB
PIK3R6
PIP3
-p
PTEN
MAGI1
PDPK1
THEM4
PPP2R3B
AKT3
HSP90AA1
CDC37
CRTC2
PHLPP2
MTCP1
GYS1
Metabolism
GSK3B
DNA
PCK1
G6PC1
Metabolism
Glycolysis / Gluconeogenesis
MYC
Cell cycle progression
CCND1
Cell cycle progression
CDKN1A
CDKN1B
CDK2
CCND1
Cell cycle progression
Cell cycle
FoxO signaling pathway
FOXO3
CDKN1B
RBL2
Cell cycle progression
YWHAQ
FASLG
BCL2L11
Cell survival
BAD
BCL2L1
BCL2
Cell survival
Apoptosis
CASP9
Cell survival
CREB3
BCL2
MCL1
Cell survival
RXRA
NR4A1
BCL2
Cell survival
CHUK
NFKB1
BCL2L1
MYB
Cell survival
NFκB signaling pathway
MDM2
TP53
Cell survival
p53 signaling pathway
PIK3R6
PI(4,5)P2
PTEN
PI(3,4,5)P3
04151 7/7/21
(c) Kanehisa Laboratories

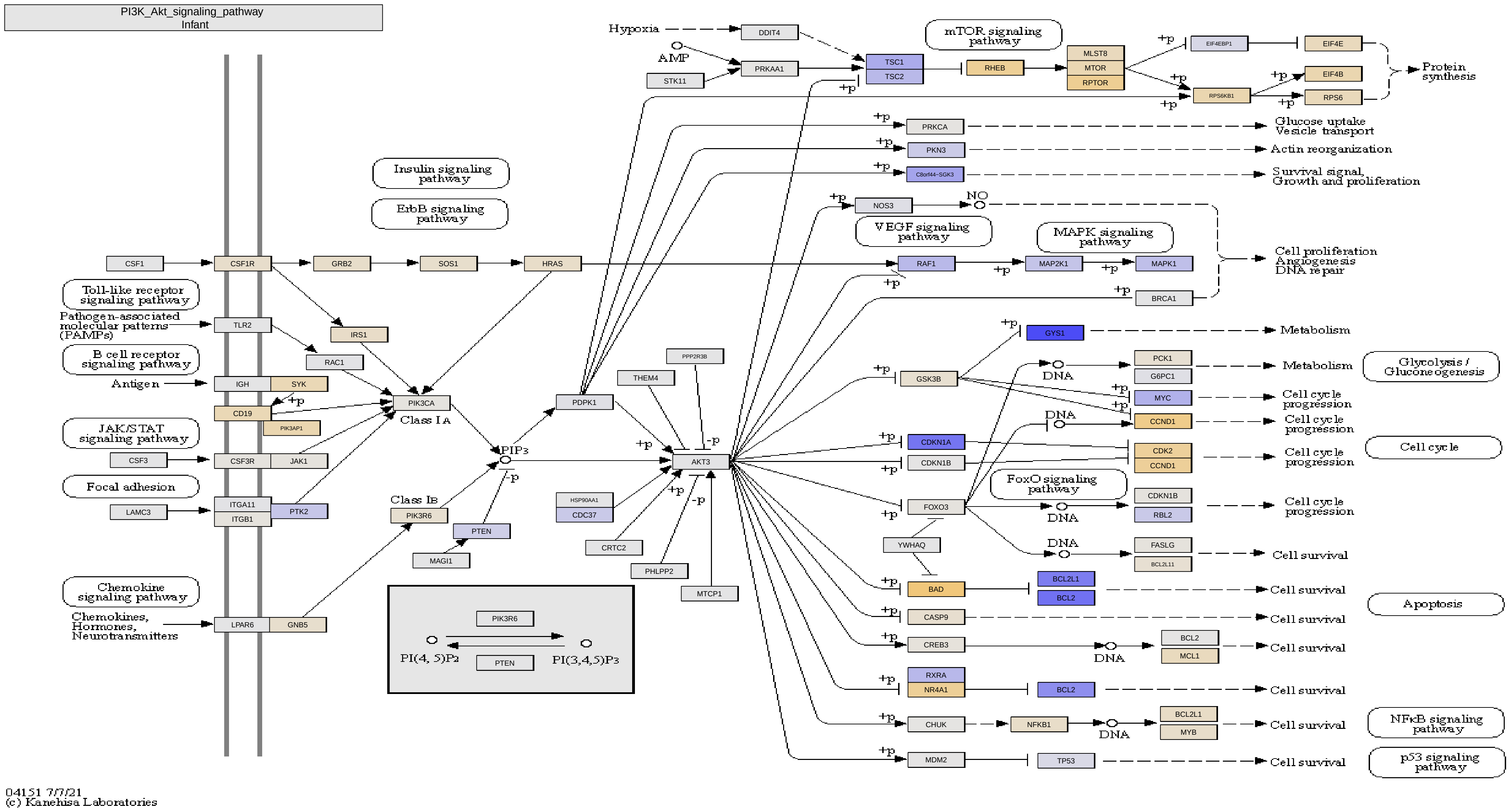

PI3K_Akt_signaling_pathway
Infant
Hypoxia
DDIT4
AMP
PRKAA1
STK11
TSC1
TSC2
mTOR signaling pathway
RHEB
MLST8
MTOR
RPTOR
EIF4EBP1
EIF4E
EIF4B
RPS6KB1
RPS6
Protein synthesis
PRKCA
Glucose uptake
Vesicle transport
PKN3
Actin reorganization
C8orf44-SGK3
Survival signal,
Growth and proliferation
Insulin signaling pathway
ErbB signaling pathway
NOS3
NO
VEGF signaling pathway
MAPK signaling pathway
Cell proliferation
Angiogenesis
DNA repair
CSF1
CSF1R
GRB2
SOS1
HRAS
RAF1
MAP2K1
MAPK1
BRCA1
Toll-like receptor signaling pathway
Pathogen-associated molecular patterns (PAMPs)
TLR2
IRS1
RAC1
GYS1
Metabolism
B cell receptor signaling pathway
Antigen
IGH
SYK
CD19
PIK3AP1
PIK3CA
Class IA
PDPK1
THEM4
PPP2R3B
GSK3B
PCK1
G6PC1
Metabolism
Glycolysis / Gluconeogenesis
MYC
Cell cycle progression
CCND1
Cell cycle progression
DNA
JAK/STAT signaling pathway
CSF3
CSF3R
JAK1
PIP3
AKT3
CDKN1A
CDKN1B
CDK2
CCND1
Cell cycle progression
Cell cycle
Focal adhesion
LAMC3
ITGA11
ITGB1
PTK2
Class IB
PIK3R6
HSP90AA1
CDC37
FOXO3
FoxO signaling pathway
CDKN1B
RBL2
Cell cycle progression
PTEN
MAGI1
CRTC2
PHLPP2
MTCP1
YWHAQ
FASLG
BCL2L11
Cell survival
Chemokine signaling pathway
Chemokines, Hormones, Neurotransmitters
LPAR6
GNB5
BAD
BCL2L1
BCL2
Cell survival
Apoptosis
CASP9
Cell survival
PIK3R6
PI(4, 5)P2
PTEN
PI(3,4,5)P3
CREB3
BCL2
MCL1
Cell survival
RXRA
NR4A1
BCL2
Cell survival
CHUK
NFKB1
BCL2L1
MYB
Cell survival
NFκB signaling pathway
MDM2
TP53
Cell survival
p53 signaling pathway
+p
-p
04151 7/7/21
(c) Kanehisa Laboratories

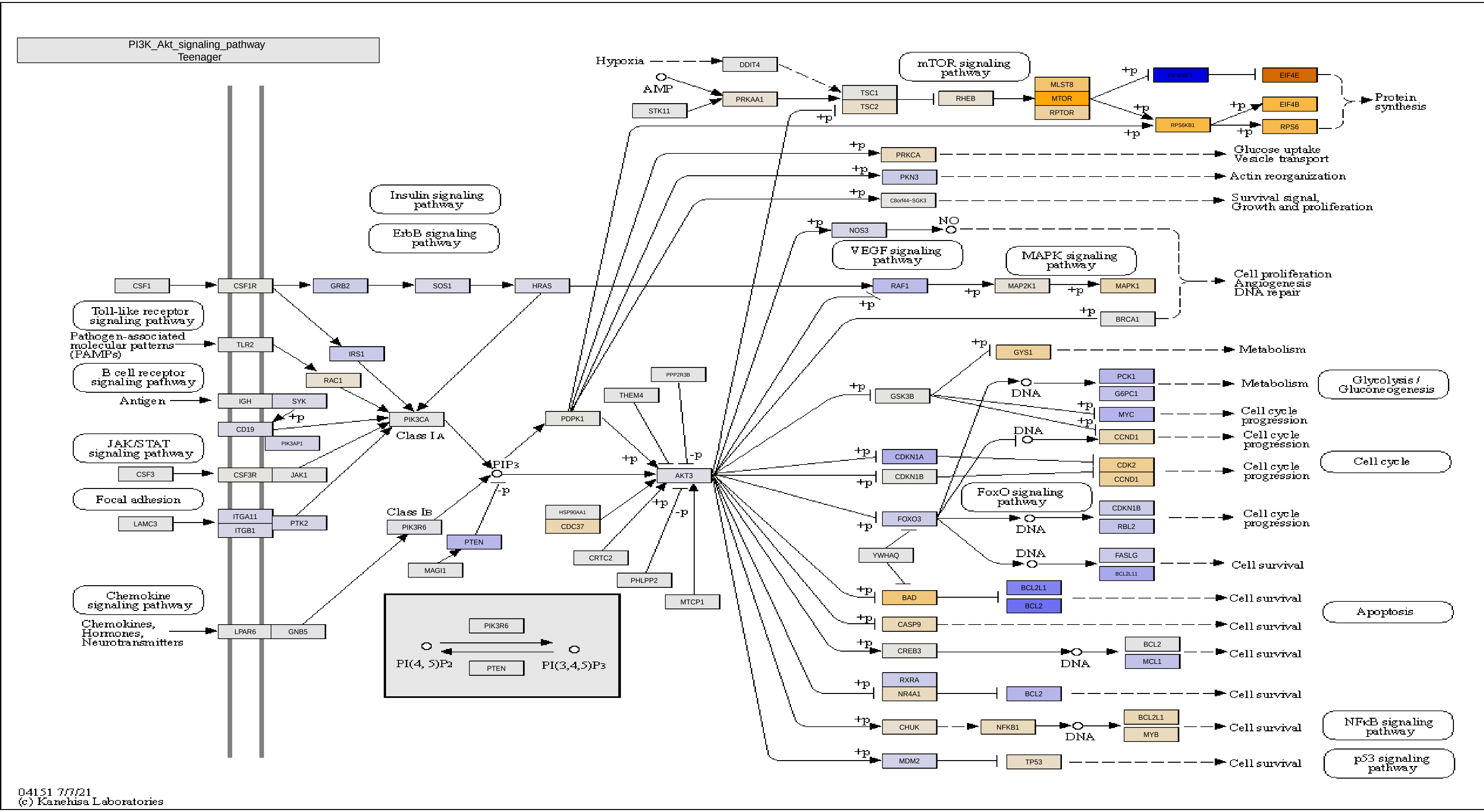

PI3K_Akt_signaling_pathway
Teenager
Hypoxia
DDIT4
AMP
PRKAA1
STK11
TSC1
TSC2
mTOR signaling pathway
RHEB
MLST8
MTOR
RPTOR
EIF4E
EIF4B
RPS6KB1
RPS6
Protein synthesis
PRKCA
Glucose uptake
Vesicle transport
PKN3
Actin reorganization
Survival signal, Growth and proliferation
Insulin signaling pathway
ErbB signaling pathway
NOS3
NO
VEGF signaling pathway
MAPK signaling pathway
CSF1
CSF1R
GRB2
SOS1
HRAS
RAF1
MAP2K1
MAPK1
Cell proliferation
Angiogenesis
DNA repair
BRCA1
Toll-like receptor signaling pathway
Pathogen-associated molecular patterns (PAMPs)
TLR2
IRS1
RAC1
GYS1
Metabolism
B cell receptor signaling pathway
Antigen
IGH
SYK
CD19
PIK3AP1
PIK3CA
Class IA
PDPK1
THEM4
PPP2R3B
GSK3B
PCK1
G6PC1
Glycolysis / Gluconeogenesis
MYC
CCND1
Cell cycle progression
DNA
JAK/STAT signaling pathway
CSF3
CSF3R
JAK1
PIP3
AKT3
CDKN1A
CDKN1B
CDK2
Cell cycle
HSP90AA1
CDC37
FoxO signaling pathway
FOXO3
RBL2
Focal adhesion
LAMC3
ITGA11
ITGB1
PTK2
Class IB
PIK3R6
PTEN
CRTC2
YWHAQ
FASLG
BCL2L11
Cell survival
MAGI1
PHLPP2
MTCP1
BAD
BCL2L1
BCL2
Chemokine signaling pathway
Chemokines, Hormones, Neurotransmitters
LPAR6
GNB5
PI(4,5)P2
PI(3,4,5)P3
CASP9
Apoptosis
CREB3
MCL1
RXRA
NR4A1
CHUK
NFKB1
MYB
NFκB signaling pathway
MDM2
TP53
p53 signaling pathway
+p
-p
04151 7/7/21
(c) Kanehisa Laboratories

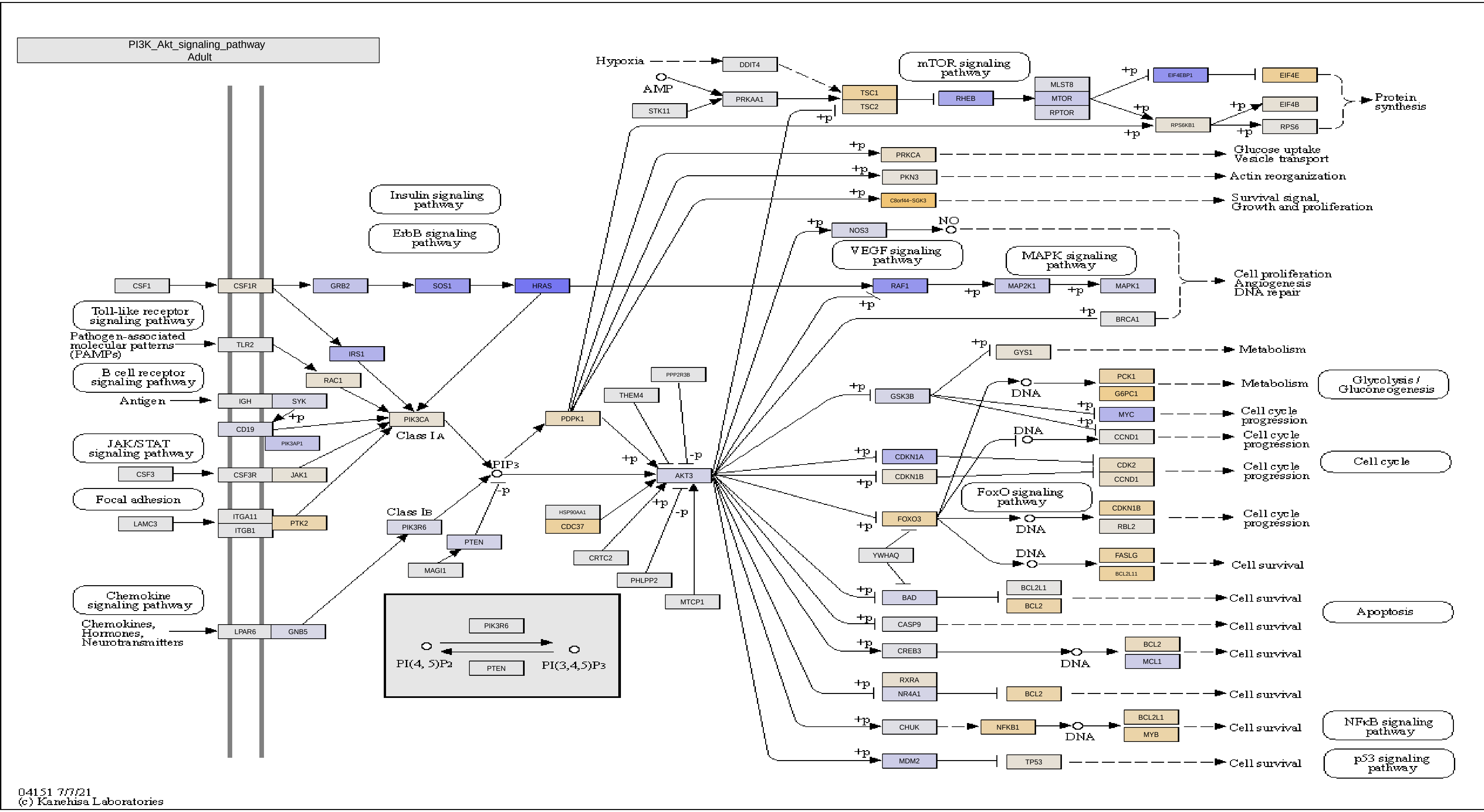
PI3K_Akt_signaling_pathway
Adult
Hypoxia
DDIT4
AMP
PRKAA1
STK11
TSC1
TSC2
mTOR signaling pathway
RHEB
MLST8
MTOR
RPTOR
EIF4EBP1
EIF4E
EIF4B
RPS6KB1
RPS6
Protein synthesis
PRKCA
Glucose uptake
Vesicle transport
PKN3
Actin reorganization
C8orf44-SGK3
Survival signal, Growth and proliferation
Insulin signaling pathway
ErbB signaling pathway
NOS3
NO
VEGF signaling pathway
MAPK signaling pathway
CSF1
CSF1R
GRB2
SOS1
HRAS
RAF1
MAP2K1
MAPK1
Cell proliferation
Angiogenesis
DNA repair
BRCA1
Toll-like receptor signaling pathway
Pathogen-associated molecular patterns (PAMPs)
TLR2
IRS1
RAC1
B cell receptor signaling pathway
Antigen
IGH
SYK
CD19
PIK3AP1
PIK3CA
Class IA
PDPK1
THEM4
PPP2R3B
GYS1
Metabolism
GSK3B
PCK1
G6PC1
Metabolism
Glycolysis / Gluconeogenesis
MYC
Cell cycle progression
CCND1
Cell cycle progression
JAK/STAT signaling pathway
CSF3
CSF3R
JAK1
PIP3
AKT3
HSP90AA1
CDC37
CDKN1A
CDKN1B
CDK2
CCND1
Cell cycle progression
Cell cycle
Focal adhesion
LAMC3
ITGA11
ITGB1
PTK2
Class IB
PIK3R6
PTEN
MAGI1
CRTC2
PHLPP2
MTCP1
FoxO signaling pathway
FOXO3
CDKN1B
RBL2
Cell cycle progression
YWHAQ
FASLG
BCL2L11
Cell survival
BAD
BCL2L1
BCL2
Cell survival
Apoptosis
Chemokine signaling pathway
Chemokines, Hormones, Neurotransmitters
LPAR6
GNB5
CASP9
Cell survival
CREB3
BCL2
MCL1
Cell survival
PIK3R6
PI(4, 5)P2
PI(3,4,5)P3
PTEN
RXRA
NR4A1
BCL2
Cell survival
CHUK
NFKB1
BCL2L1
MYB
Cell survival
NFκB signaling pathway
MDM2
TP53
Cell survival
p53 signaling pathway
+p
-p
DNA
04151 7/7/21
(c) Kanehisa Laboratories

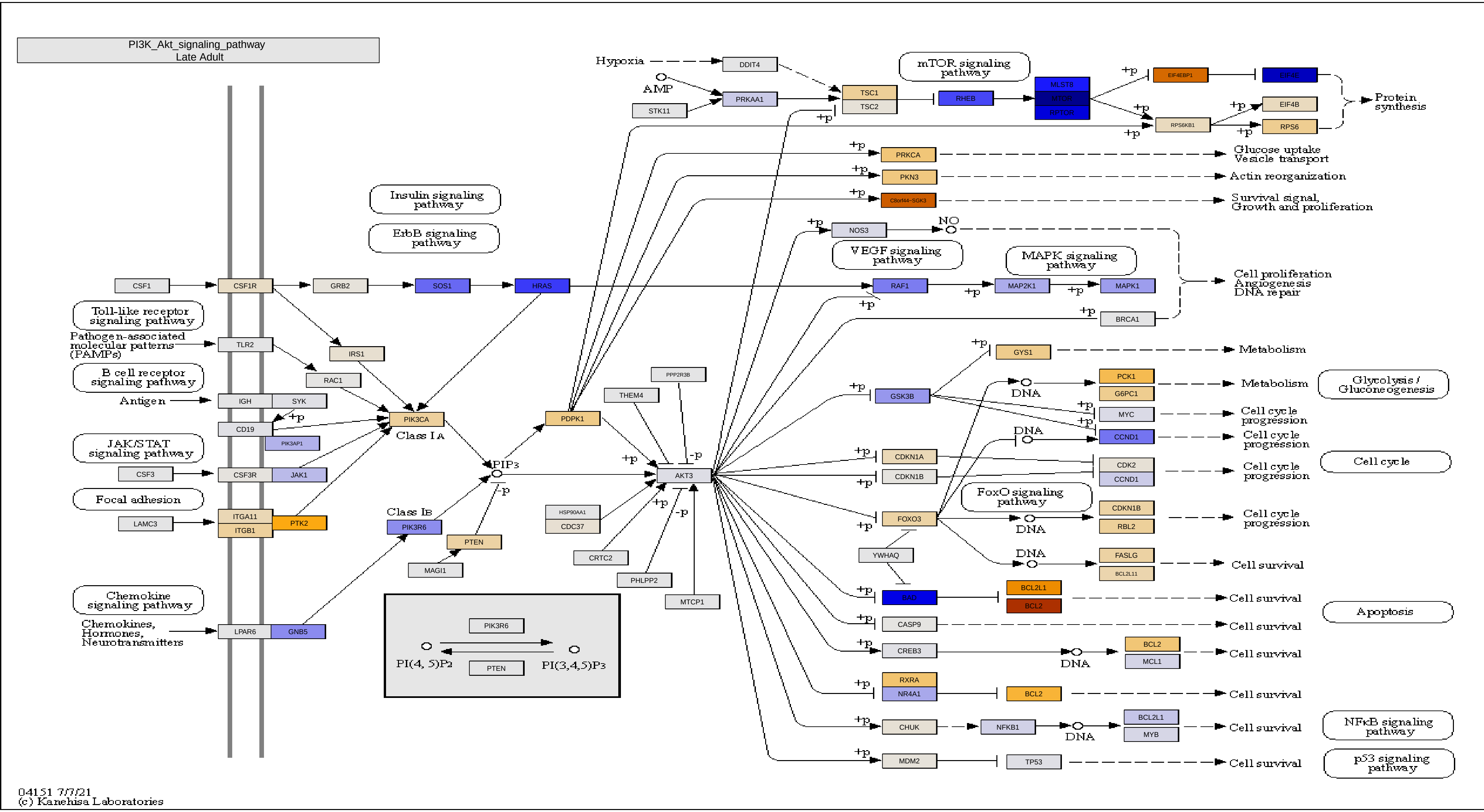
PI3K_Akt_signaling_pathway
Late Adult
Hypoxia
DDIT4
AMP
PRKAA1
STK11
mTOR signaling pathway
TSC1
TSC2
RHEB
MLST8
MTOR
RPTOR
EIF4EBP1
EIF4E
RPS6KB1
EIF4B
RPS6
Protein synthesis
PRKCA
Glucose uptake
Vesicle transport
PKN3
Actin reorganization
C8orf44-SGK3
Survival signal, Growth and proliferation
Insulin signaling pathway
ErbB signaling pathway
NOS3
NO
VEGF signaling pathway
MAPK signaling pathway
CSF1
CSF1R
GRB2
SOS1
HRAS
RAF1
MAP2K1
MAPK1
Cell proliferation
Angiogenesis
DNA repair
BRCA1
Toll-like receptor signaling pathway
Pathogen-associated molecular patterns (PAMPs)
TLR2
IRS1
RAC1
B cell receptor signaling pathway
Antigen
IGH
SYK
CD19
PIK3AP1
PIK3CA
Class IA
PDPK1
THEM4
PPP2R3B
GYS1
Metabolism
GSK3B
PCK1
G6PC1
Metabolism
Glycolysis / Gluconeogenesis
MYC
Cell cycle progression
CCND1
Cell cycle progression
JAK/STAT signaling pathway
CSF3
CSF3R
JAK1
PIP3
AKT3
CDKN1A
CDKN1B
CDK2
CCND1
Cell cycle progression
Cell cycle
Focal adhesion
ITGA11
ITGB1
PTK2
LAMC3
Class IB
PIK3R6
PTEN
HSP90AA1
CDC37
FoxO signaling pathway
FOXO3
CDKN1B
RBL2
Cell cycle progression
YWHAQ
FASLG
BCL2L11
Cell survival
MAGI1
CRTC2
PHLPP2
MTCP1
BAD
BCL2L1
BCL2
Cell survival
Apoptosis
Chemokine signaling pathway
Chemokines, Hormones, Neurotransmitters
LPAR6
GNB5
PIK3R6
PI(4, 5)P2
PI(3,4,5)P3
PTEN
CASP9
Cell survival
CREB3
BCL2
MCL1
Cell survival
RXRA
NR4A1
BCL2
Cell survival
CHUK
NFKB1
BCL2L1
MYB
Cell survival
NFκB signaling pathway
MDM2
TP53
Cell survival
p53 signaling pathway
DNA
+p
-p
04151 7/7/21
(c) Kanehisa Laboratories
