# Supplementary material for: The Transcriptome and Methylome of the Developing and Aging Brain and Their Relations to Gliomas and Psychological Disorders
Source: Cells. 2022 Jan 21;11(3):362. doi: 10.3390/cells11030362 (PMC8834030; doi:10.3390/cells11030362)
Supplement: Supplementary file 1 [file cells-11-00362-s001.zip › Suppl-Material-S3-Pathways-PSF_Expression/PPAR_signaling_pathway.pdf]

PPAR signaling pathway

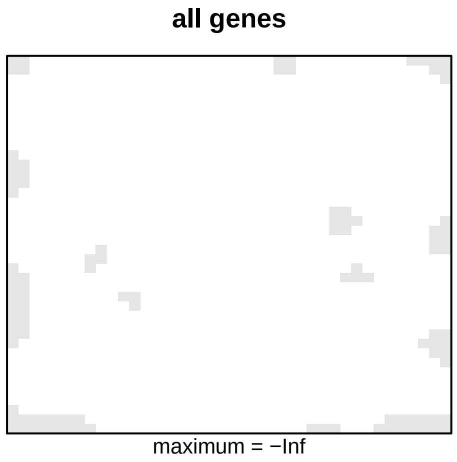

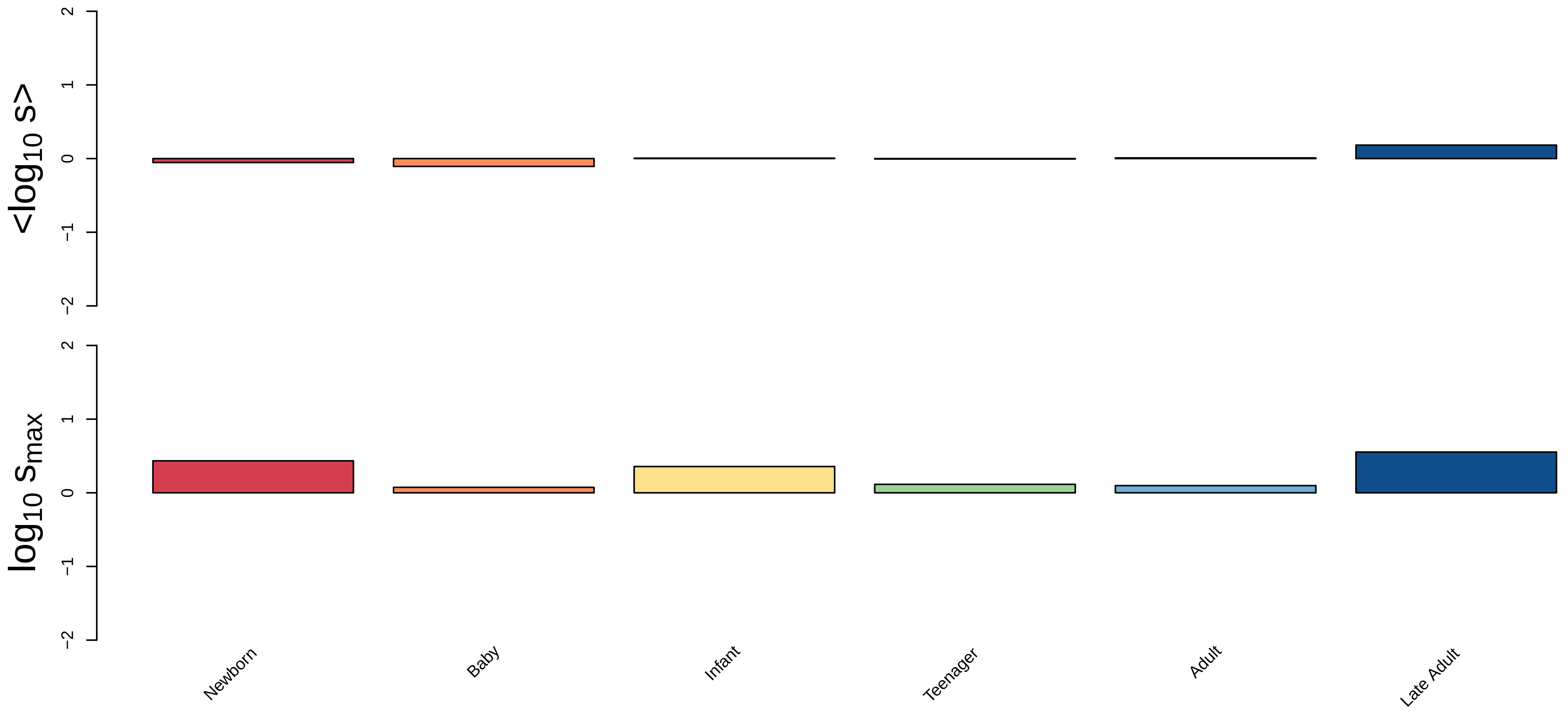

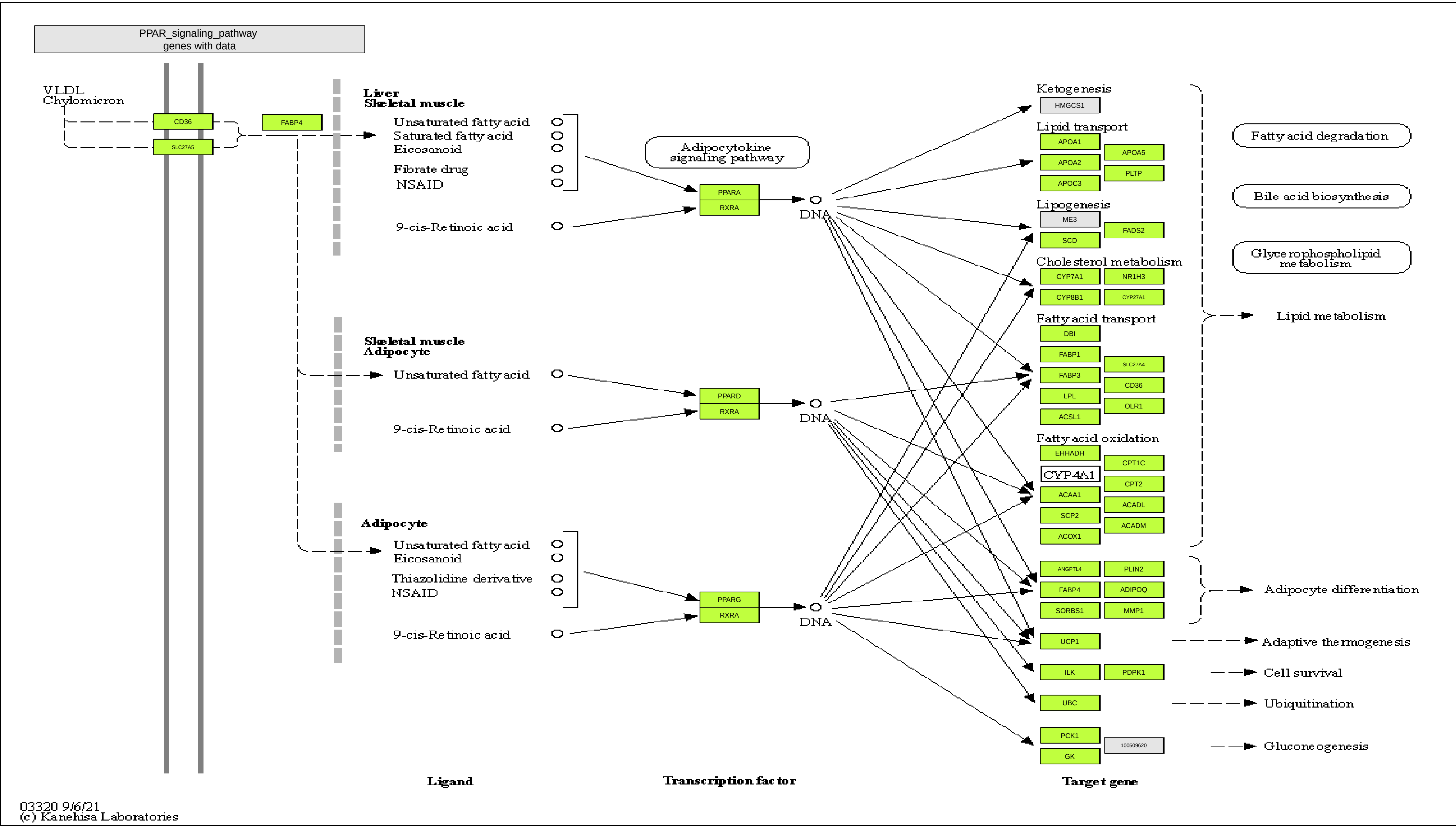
PPAR_signaling_pathway
genes with data
VLDL
Chylomicron
CD36
SLC27A5
FABP4
Liver
Skeletal muscle
Unsaturated fatty acid
Saturated fatty acid
Eicosanoid
Fibrate drug
NSAID
9-cis-Retinoic acid
Adipocytokine signaling pathway
PPARA
RXRA
DNA
Skeletal muscle
Adipocyte
Unsaturated fatty acid
9-cis-Retinoic acid
PPARD
RXRA
DNA
Adipocyte
Unsaturated fatty acid
Eicosanoid
Thiazolidine derivative
NSAID
9-cis-Retinoic acid
PPARG
RXRA
DNA
Ketogenesis
HMGCS1
Lipid transport
APOA1
APOA2
APOC3
APOA5
PLTP
Lipogenesis
ME3
SCD
FADS2
Cholesterol metabolism
CYP7A1
CYP8B1
NR1H3
CYP27A1
Fatty acid transport
DBI
FABP1
FABP3
LPL
ACSL1
SLC27A4
CD36
OLR1
Fatty acid oxidation
EHHADH
CYP4A1
ACAA1
SCP2
ACOX1
CPT1C
CPT2
ACADL
ACADM
ANGPTL4
FABP4
SORBS1
PLIN2
ADIPOQ
MMP1
UCP1
ILK
PDPK1
UBC
PCK1
GK
100509620
Fatty acid degradation
Bile acid biosynthesis
Glycerophospholipid metabolism
Lipid metabolism
Adipocyte differentiation
Adaptive thermogenesis
Cell survival
Ubiquitination
Gluconeogenesis
Ligand
Transcription factor
Target gene
03320 9/6/21
(c) Kanehisa Laboratories

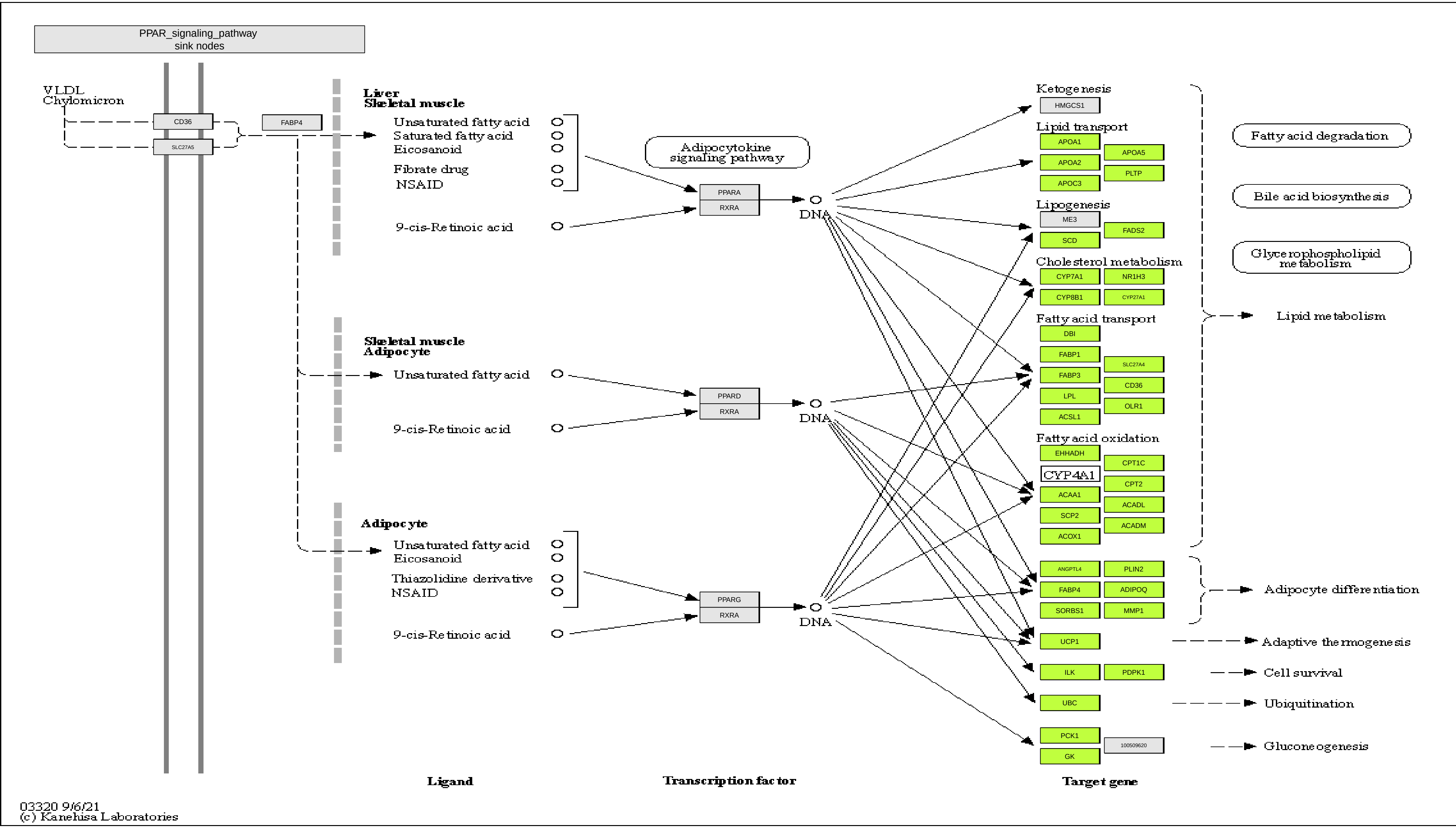
PPAR_signaling_pathway
sink nodes
VLDL
Chylomicron
CD36
SLC27A5
FABP4
Liver
Skeletal muscle
Unsaturated fatty acid
Saturated fatty acid
Eicosanoid
Fibrate drug
NSAID
9-cis-Retinoic acid
Adipocytokine signaling pathway
PPARA
RXRA
DNA
Skeletal muscle
Adipocyte
Unsaturated fatty acid
9-cis-Retinoic acid
PPARD
RXRA
DNA
Adipocyte
Unsaturated fatty acid
Eicosanoid
Thiazolidine derivative
NSAID
9-cis-Retinoic acid
PPARG
RXRA
DNA
Ketogenesis
HMGCS1
Lipid transport
APOA1
APOA2
APOC3
APOA5
PLTP
Lipogenesis
ME3
SCD
FADS2
Cholesterol metabolism
CYP7A1
CYP8B1
NR1H3
CYP27A1
Fatty acid transport
DBI
FABP1
FABP3
LPL
ACSL1
SLC27A4
CD36
OLR1
Fatty acid oxidation
EHHADH
CYP4A1
ACAA1
SCP2
ACOX1
CPT1C
CPT2
ACADL
ACADM
ANGPTL4
FABP4
SORBS1
PLIN2
ADIPOQ
MMP1
UCP1
ILK
PDPK1
UBC
PCK1
GK
100509620
Fatty acid degradation
Bile acid biosynthesis
Glycerophospholipid metabolism
Lipid metabolism
Adipocyte differentiation
Adaptive thermogenesis
Cell survival
Ubiquitination
Gluconeogenesis
Ligand
Transcription factor
Target gene
03320 9/6/21
(c) Kanehisa Laboratories

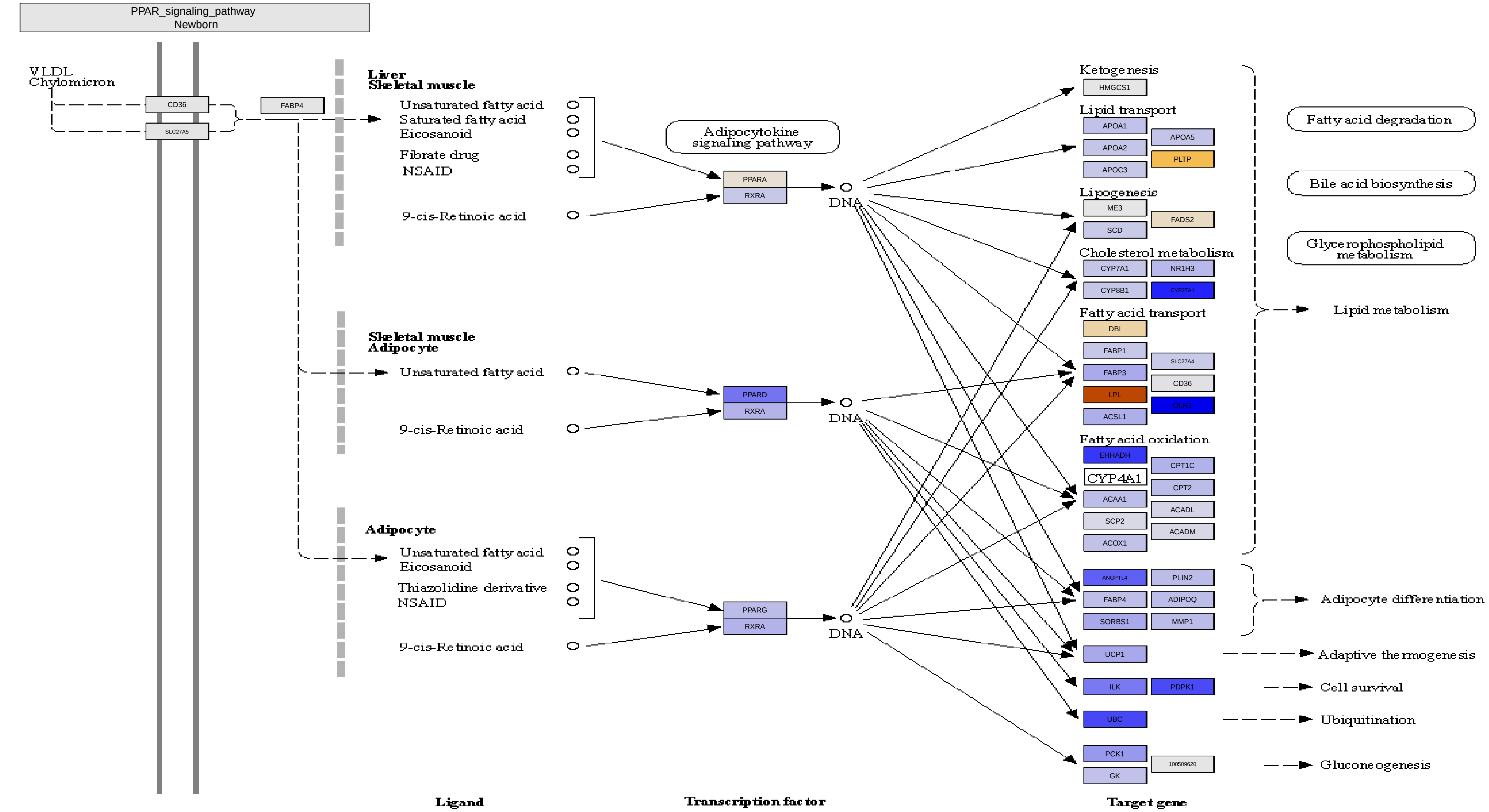

03320 9/6/21
(c) Kanehisa Laboratories

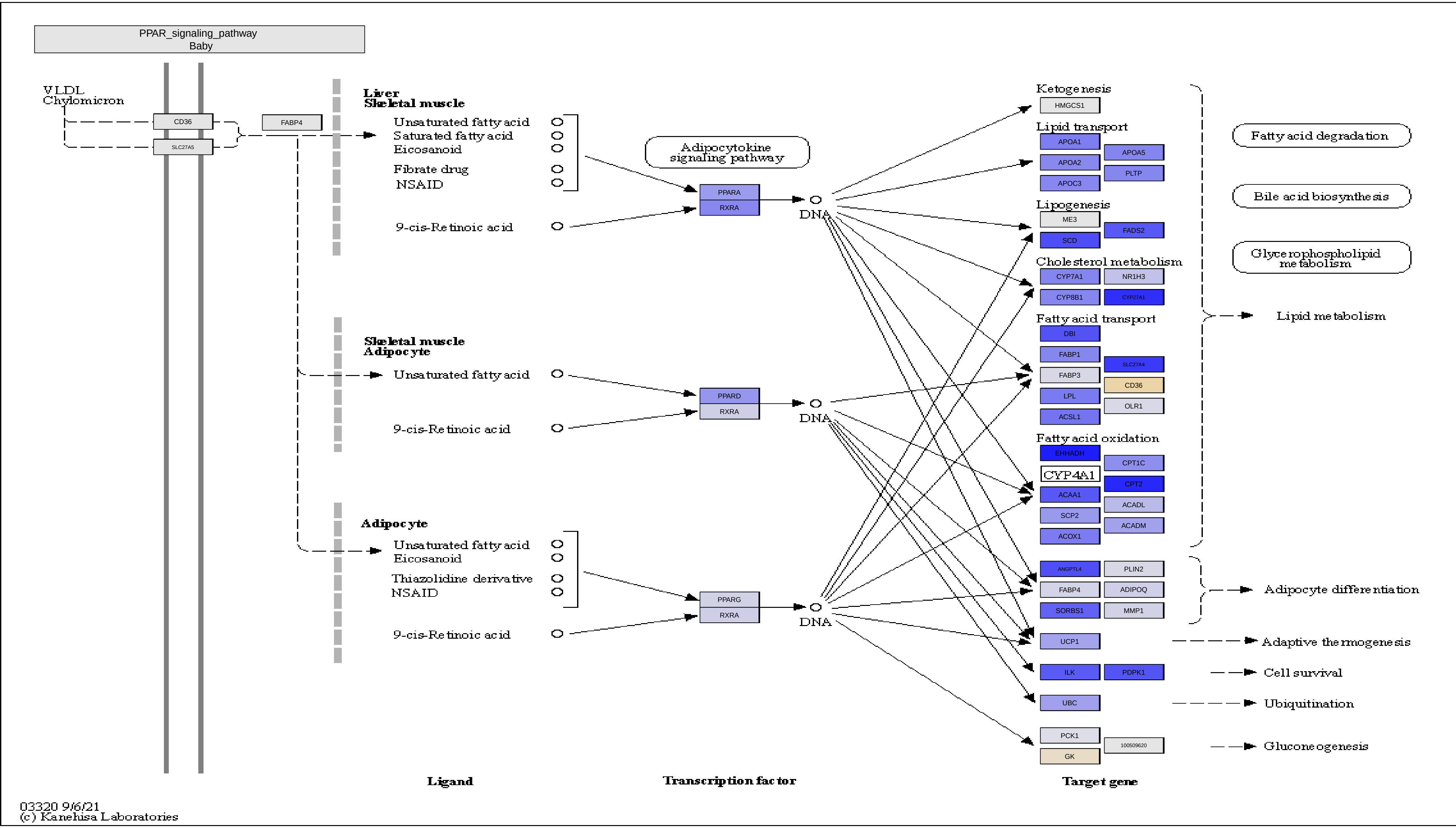
PPAR_signaling_pathway
Baby
VLDL
Chylomicron
CD36
SLC27A5
FABP4
Liver
Skeletal muscle
Unsaturated fatty acid
Saturated fatty acid
Eicosanoid
Fibrate drug
NSAID
9-cis-Retinoic acid
Adipocytokine signaling pathway
PPARA
RXRA
DNA
Skeletal muscle
Adipocyte
Unsaturated fatty acid
9-cis-Retinoic acid
PPARD
RXRA
DNA
Adipocyte
Unsaturated fatty acid
Eicosanoid
Thiazolidine derivative
NSAID
9-cis-Retinoic acid
PPARG
RXRA
DNA
Ketogenesis
HMGCS1
Lipid transport
APOA1
APOA5
APOA2
PLTP
APOC3
Lipogenesis
ME3
FADS2
SCD
Cholesterol metabolism
CYP7A1
NR1H3
CYP8B1
CYP27A1
Fatty acid transport
DBI
FABP1
SLC27A4
FABP3
CD36
LPL
OLR1
ACSL1
Fatty acid oxidation
EHHADH
CPT1C
CYP4A1
CPT2
ACAA1
ACADL
SCP2
ACADM
ACOX1
ANGPTL4
PLIN2
FABP4
ADIPOQ
SORBS1
MMP1
UCP1
ILK
PDPK1
UBC
PCK1
100509620
GK
Fatty acid degradation
Bile acid biosynthesis
Glycerophospholipid metabolism
Lipid metabolism
Adipocyte differentiation
Adaptive thermogenesis
Cell survival
Ubiquitination
Gluconeogenesis
Ligand
Transcription factor
Target gene
03320 9/6/21
(c) Kanehisa Laboratories

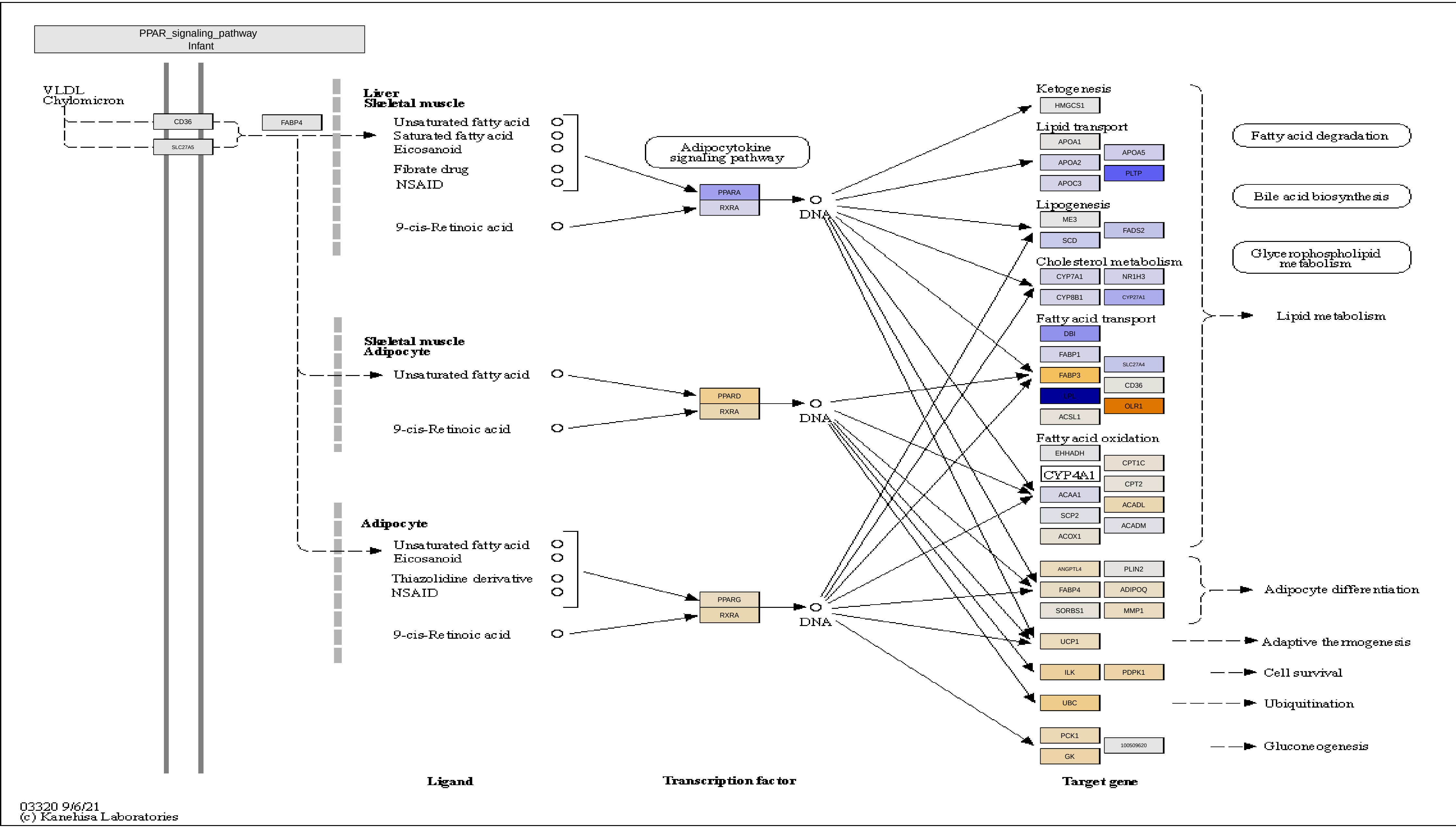
PPAR_signaling_pathway
Infant
VLDL
Chylomicron
CD36
SLC27A5
FABP4
Liver
Skeletal muscle
Unsaturated fatty acid
Saturated fatty acid
Eicosanoid
Fibrate drug
NSAID
9-cis-Retinoic acid
Adipocytokine signaling pathway
PPARA
RXRA
DNA
Skeletal muscle
Adipocyte
Unsaturated fatty acid
9-cis-Retinoic acid
PPARD
RXRA
DNA
Adipocyte
Unsaturated fatty acid
Eicosanoid
Thiazolidine derivative
NSAID
9-cis-Retinoic acid
PPARG
RXRA
DNA
Ketogenesis
HMGCS1
Lipid transport
APOA1
APOA2
APOC3
APOA5
PLTP
Lipogenesis
ME3
SCD
FADS2
Cholesterol metabolism
CYP7A1
CYP8B1
NR1H3
CYP27A1
Fatty acid transport
DBI
FABP1
FABP3
ACSL1
SLC27A4
CD36
OLR1
Fatty acid oxidation
EHHADH
CYP4A1
ACAA1
SCP2
ACOX1
CPT1C
CPT2
ACADL
ACADM
ANGPTL4
FABP4
SORBS1
PLIN2
ADIPOQ
MMP1
UCP1
ILK
PDPK1
UBC
PCK1
GK
100509620
Fatty acid degradation
Bile acid biosynthesis
Glycerophospholipid metabolism
Lipid metabolism
Adipocyte differentiation
Adaptive thermogenesis
Cell survival
Ubiquitination
Gluconeogenesis
Ligand
Transcription factor
Target gene
03320 9/6/21
(c) Kanehisa Laboratories

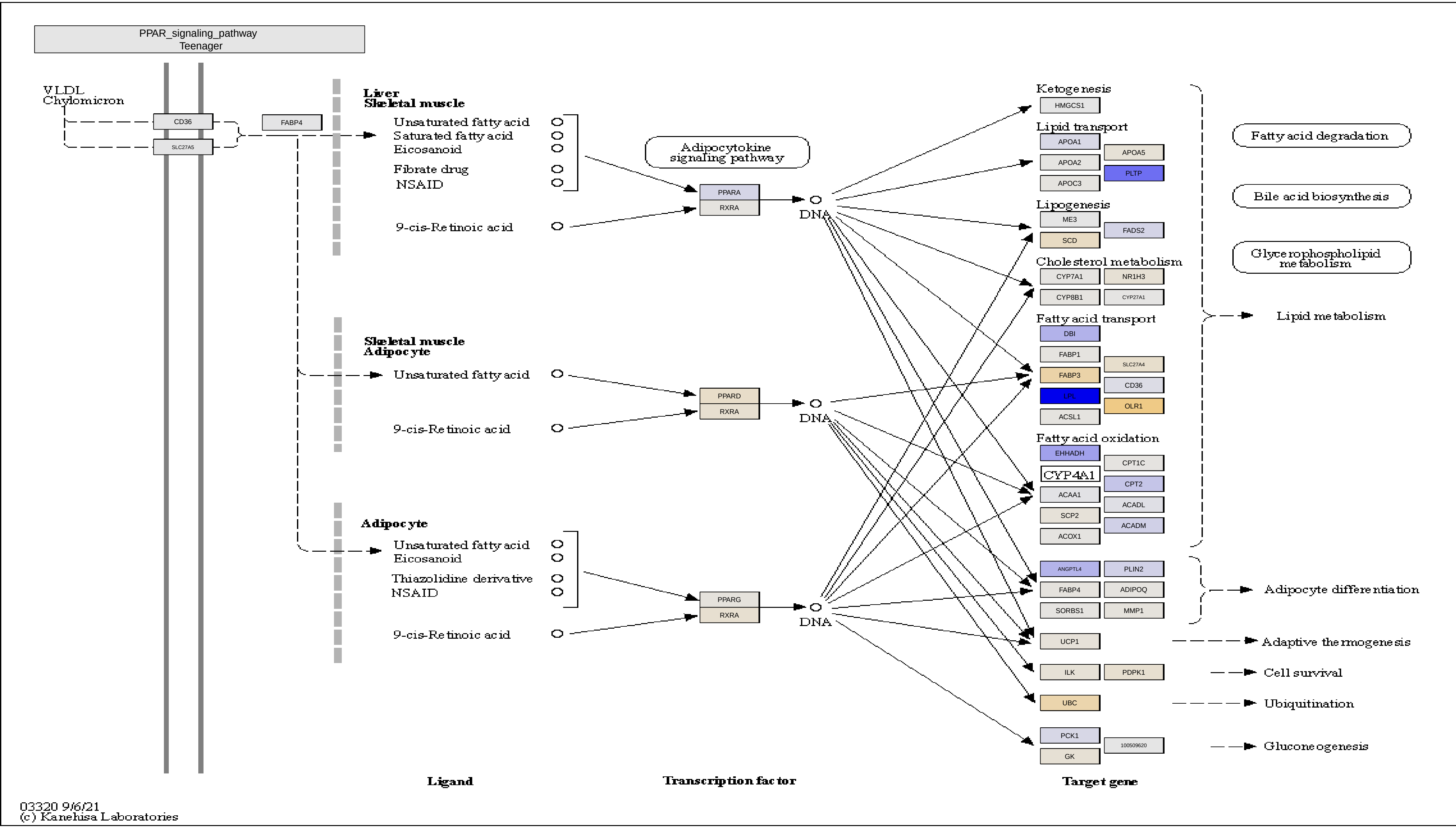
PPAR_signaling_pathway
Teenager
VLDL
Chylomicron
CD36
SLC27A5
FABP4
Liver
Skeletal muscle
Unsaturated fatty acid
Saturated fatty acid
Eicosanoid
Fibrate drug
NSAID
9-cis-Retinoic acid
Adipocytokine signaling pathway
PPARA
RXRA
DNA
Skeletal muscle
Adipocyte
Unsaturated fatty acid
9-cis-Retinoic acid
PPARD
RXRA
DNA
Adipocyte
Unsaturated fatty acid
Eicosanoid
Thiazolidine derivative
NSAID
9-cis-Retinoic acid
PPARG
RXRA
DNA
Ketogenesis
HMGCS1
Lipid transport
APOA1
APOA2
APOC3
APOA5
PLTP
Lipogenesis
ME3
SCD
FADS2
Cholesterol metabolism
CYP7A1
CYP8B1
NR1H3
CYP27A1
Fatty acid transport
DBI
FABP1
FABP3
LPL
ACSL1
SLC27A4
CD36
OLR1
Fatty acid oxidation
EHHADH
CYP4A1
ACAA1
SCP2
ACOX1
CPT1C
CPT2
ACADL
ACADM
ANGPTL4
FABP4
SORBS1
PLIN2
ADIPOQ
MMP1
UCP1
ILK
PDPK1
UBC
PCK1
GK
100509620
Fatty acid degradation
Bile acid biosynthesis
Glycerophospholipid metabolism
Lipid metabolism
Adipocyte differentiation
Adaptive thermogenesis
Cell survival
Ubiquitination
Gluconeogenesis
Ligand
Transcription factor
Target gene
03320 9/6/21
(c) Kanehisa Laboratories

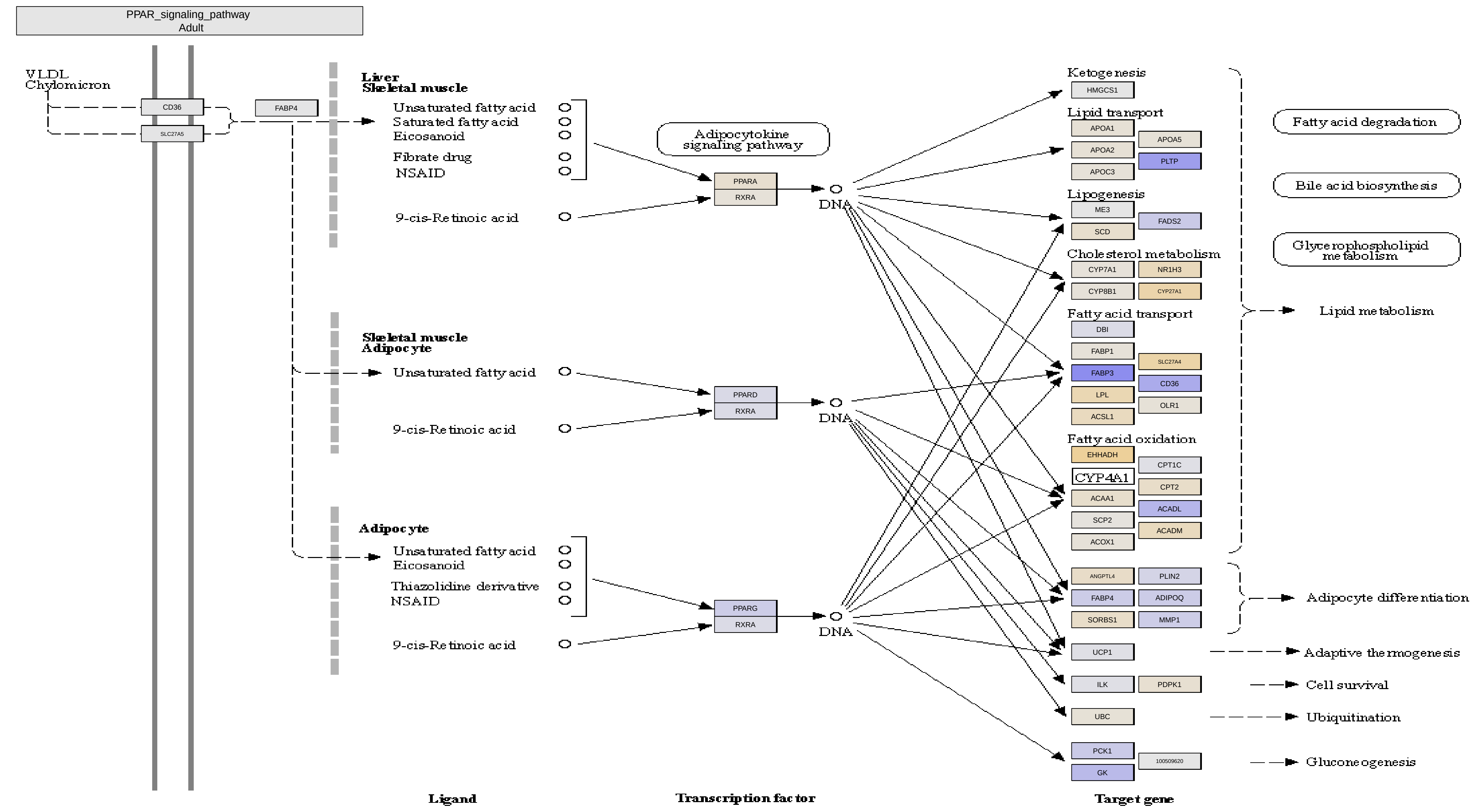

03320 9/6/21
(c) Kanehisa Laboratories

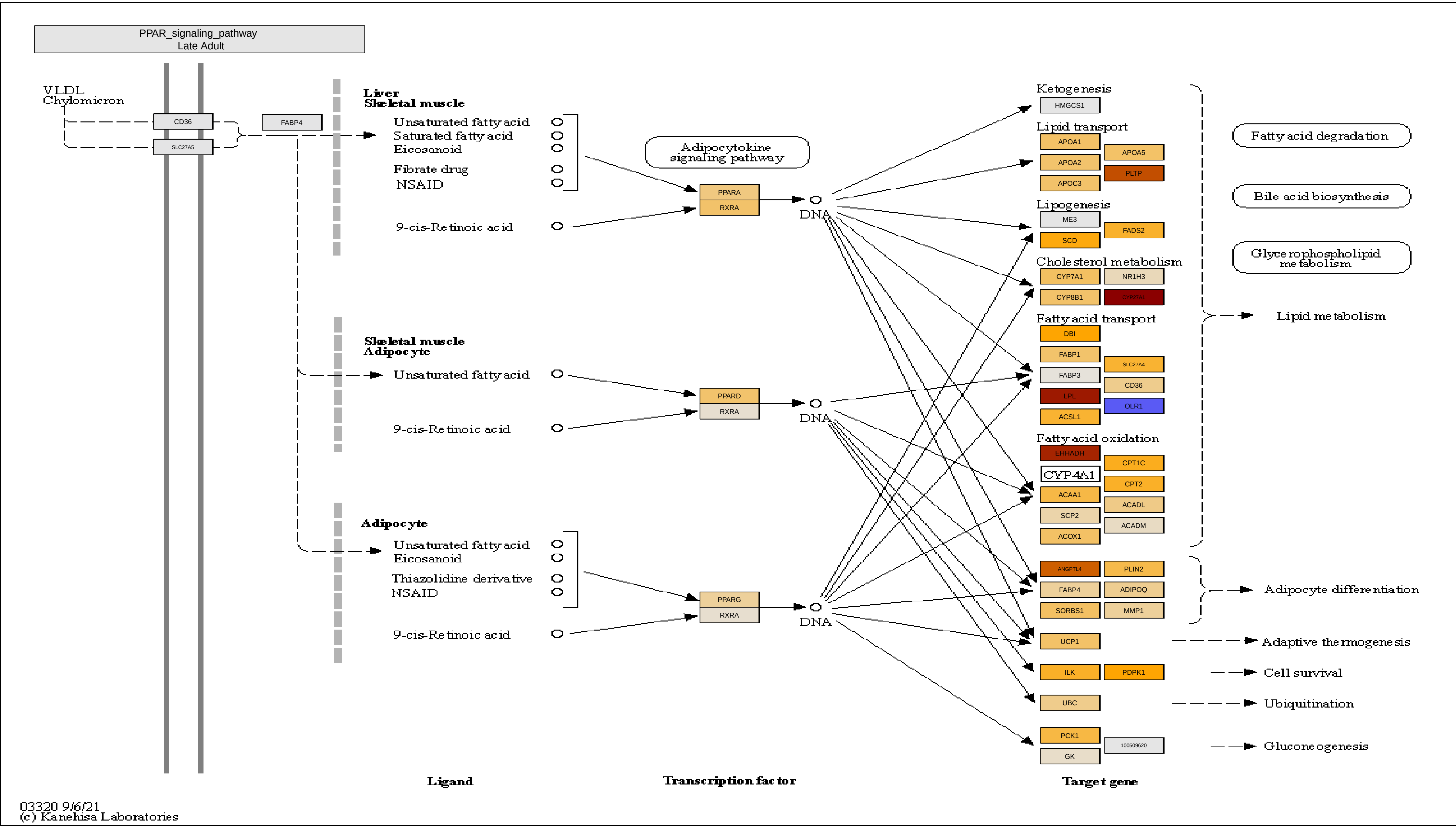

PPAR_signaling_pathway
Late Adult
VLDL
Chylomicron
CD36
SLC27A5
FABP4
Liver
Skeletal muscle
Unsaturated fatty acid
Saturated fatty acid
Eicosanoid
Fibrate drug
NSAID
9-cis-Retinoic acid
Adipocytokine signaling pathway
PPARA
RXRA
DNA
Skeletal muscle
Adipocyte
Unsaturated fatty acid
9-cis-Retinoic acid
PPARD
RXRA
DNA
Adipocyte
Unsaturated fatty acid
Eicosanoid
Thiazolidine derivative
NSAID
9-cis-Retinoic acid
PPARG
RXRA
DNA
Ketogenesis
HMGCS1
Lipid transport
APOA1
APOA2
APOC3
APOA5
PLTP
Lipogenesis
ME3
SCD
FADS2
Cholesterol metabolism
CYP7A1
CYP8B1
NR1H3
Fatty acid transport
DBI
FABP1
FABP3
LPL
ACSL1
SLC27A4
CD36
OLR1
Fatty acid oxidation
EHHADH
CYP4A1
ACAA1
SCP2
ACOX1
CPT1C
CPT2
ACADL
ACADM
ANGPTL4
FABP4
SORBS1
PLIN2
ADIPOQ
MMP1
UCP1
ILK
PDPK1
UBC
PCK1
GK
Fatty acid degradation
Bile acid biosynthesis
Glycerophospholipid metabolism
Lipid metabolism
Adipocyte differentiation
Adaptive thermogenesis
Cell survival
Ubiquitination
Gluconeogenesis
Ligand
Transcription factor
Target gene
03320 9/6/21
(c) Kanehisa Laboratories
