# Supplementary material for: The Transcriptome and Methylome of the Developing and Aging Brain and Their Relations to Gliomas and Psychological Disorders
Source: Cells. 2022 Jan 21;11(3):362. doi: 10.3390/cells11030362 (PMC8834030; doi:10.3390/cells11030362)
Supplement: Supplementary file 1 [file cells-11-00362-s001.zip › Suppl-Material-S3-Pathways-PSF_Expression/Prolactin_signaling_pathway.pdf]

Prolactin signaling pathway

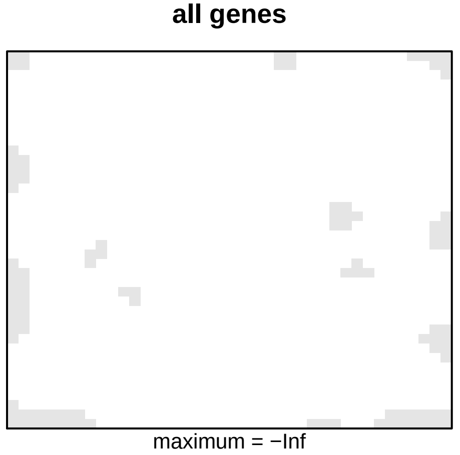

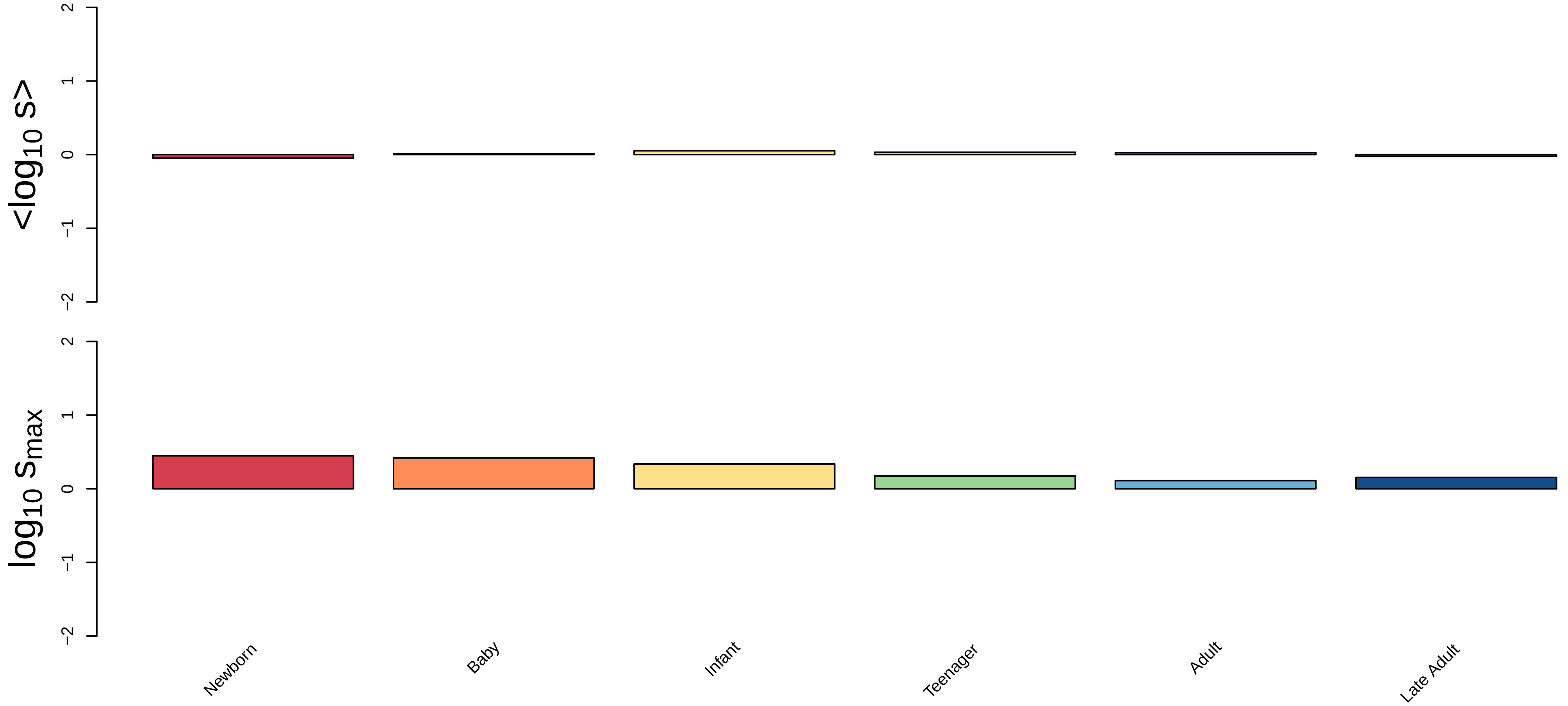

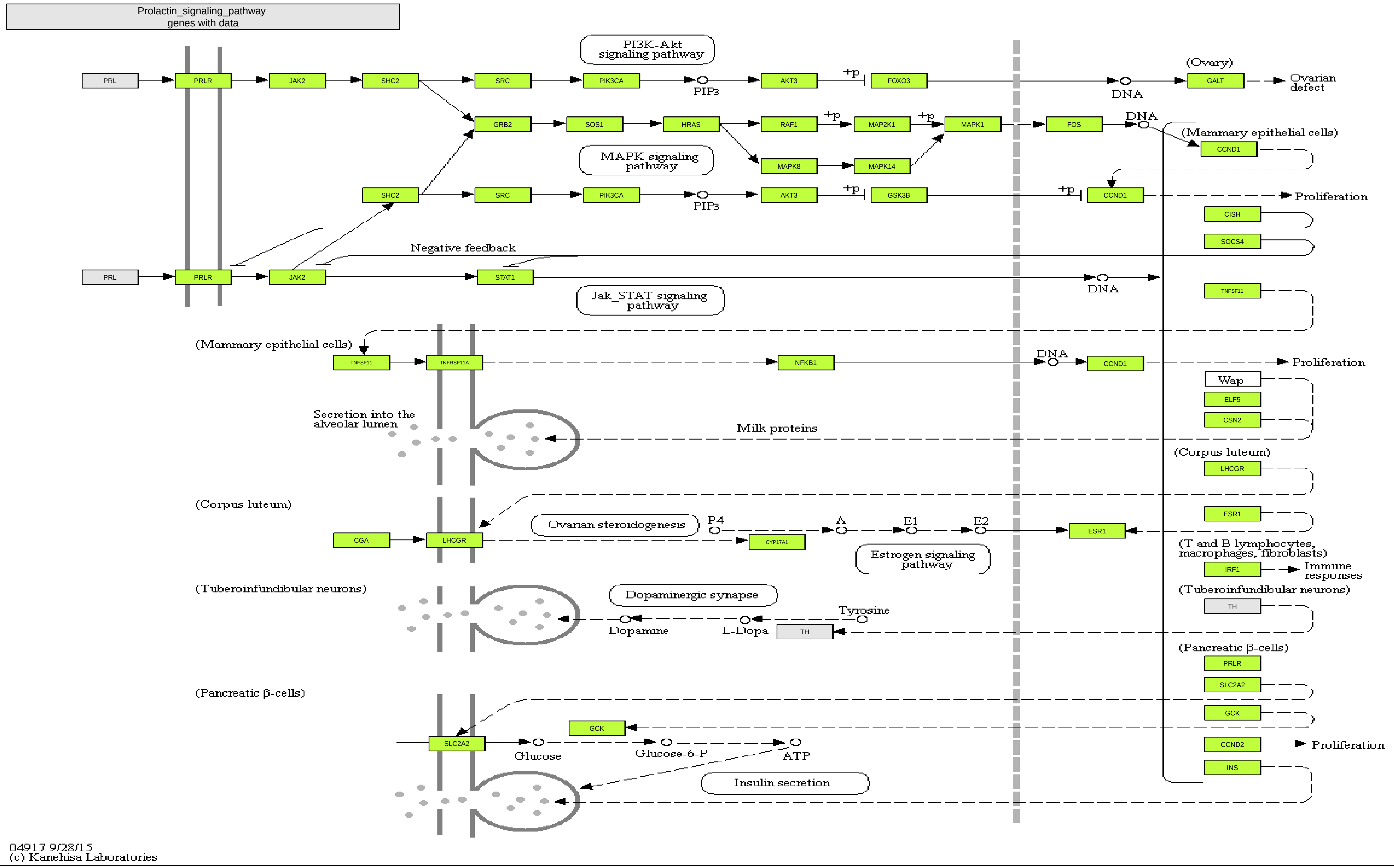

Prolactin_signaling_pathway
genes with data
PI3K-Akt signaling pathway
MAPK signaling pathway
Jak_STAT signaling pathway
PRL
PRLR
JAK2
SHC2
SRC
PIK3CA
PIP3
AKT3
+p
FOXO3
DNA
(Ovary)
GALT
Ovarian defect
GRB2
SOS1
HRAS
RAF1
MAP2K1
MAPK1
FOS
MAPK8
MAPK14
(Mammary epithelial cells)
CCND1
GSK3B
Proliferation
CISH
SOCS4
Negative feedback
STAT1
TNFSF11
TNFRSF11A
NFKB1
Secretion into the alveolar lumen
Milk proteins
Wap
ELF5
CSN2
(Corpus luteum)
LHCGR
CGA
Ovarian steroidogenesis
P4
CYP17A1
A
E1
E2
ESR1
Estrogen signaling pathway
(T and B lymphocytes, macrophages, fibroblasts)
IRF1
Immune responses
(Tuberoinfundibular neurons)
Dopaminergic synapse
Tyrosine
Dopamine
L-Dopa
TH
(Pancreatic β-cells)
SLC2A2
GCK
Glucose
Glucose-6-P
ATP
CCND2
INS
Insulin secretion
04917 9/28/15
(c) Kanehisa Laboratories

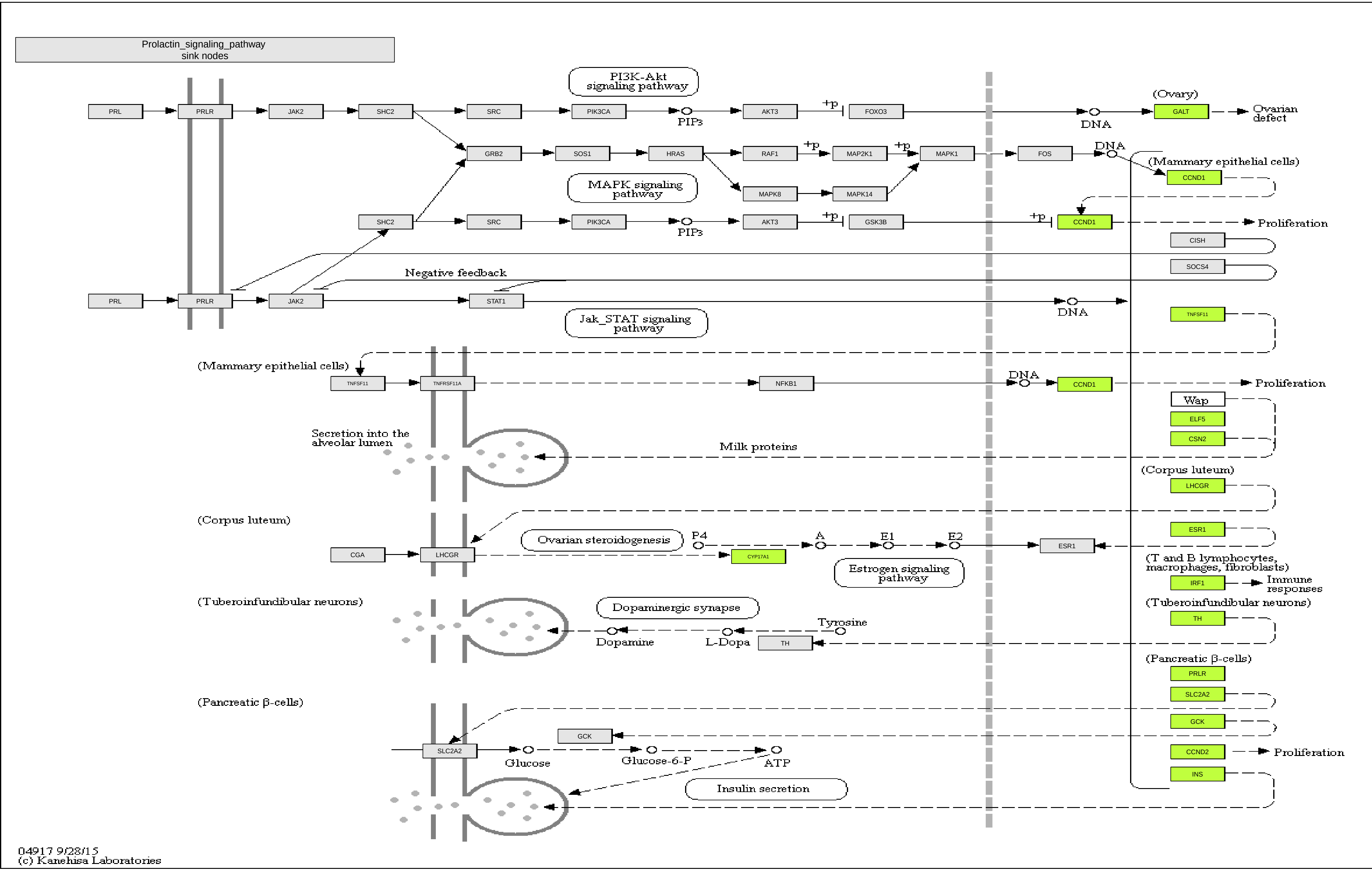
Prolactin_signaling_pathway
sink nodes
PI3K-Akt signaling pathway
MAPK signaling pathway
Jak_STAT signaling pathway
PRL
PRLR
JAK2
SHC2
SRC
PIK3CA
PIP3
AKT3
+p
FOXO3
DNA
(Ovary)
GALT
Ovarian defect
GRB2
SOS1
HRAS
RAF1
MAP2K1
MAPK1
FOS
(Mammary epithelial cells)
CCND1
MAPK8
MAPK14
GSK3B
Proliferation
CISH
SOCS4
Negative feedback
STAT1
TNFSF11
TNFRSF11A
NFKB1
Wap
ELF5
CSN2
Secretion into the alveolar lumen
Milk proteins
(Corpus luteum)
LHCGR
ESR1
CGA
Ovarian steroidogenesis
P4
A
E1
E2
CYP17A1
Estrogen signaling pathway
(T and B lymphocytes, macrophages, fibroblasts)
IRF1
Immune responses
(Tuberoinfundibular neurons)
TH
Dopaminergic synapse
Tyrosine
Dopamine
L-Dopa
(Pancreatic β-cells)
SLC2A2
GCK
CCND2
INS
Glucose
Glucose-6-P
ATP
Insulin secretion
04917 9/28/15
(c) Kanehisa Laboratories

Prolactin_signaling_pathway
Newborn

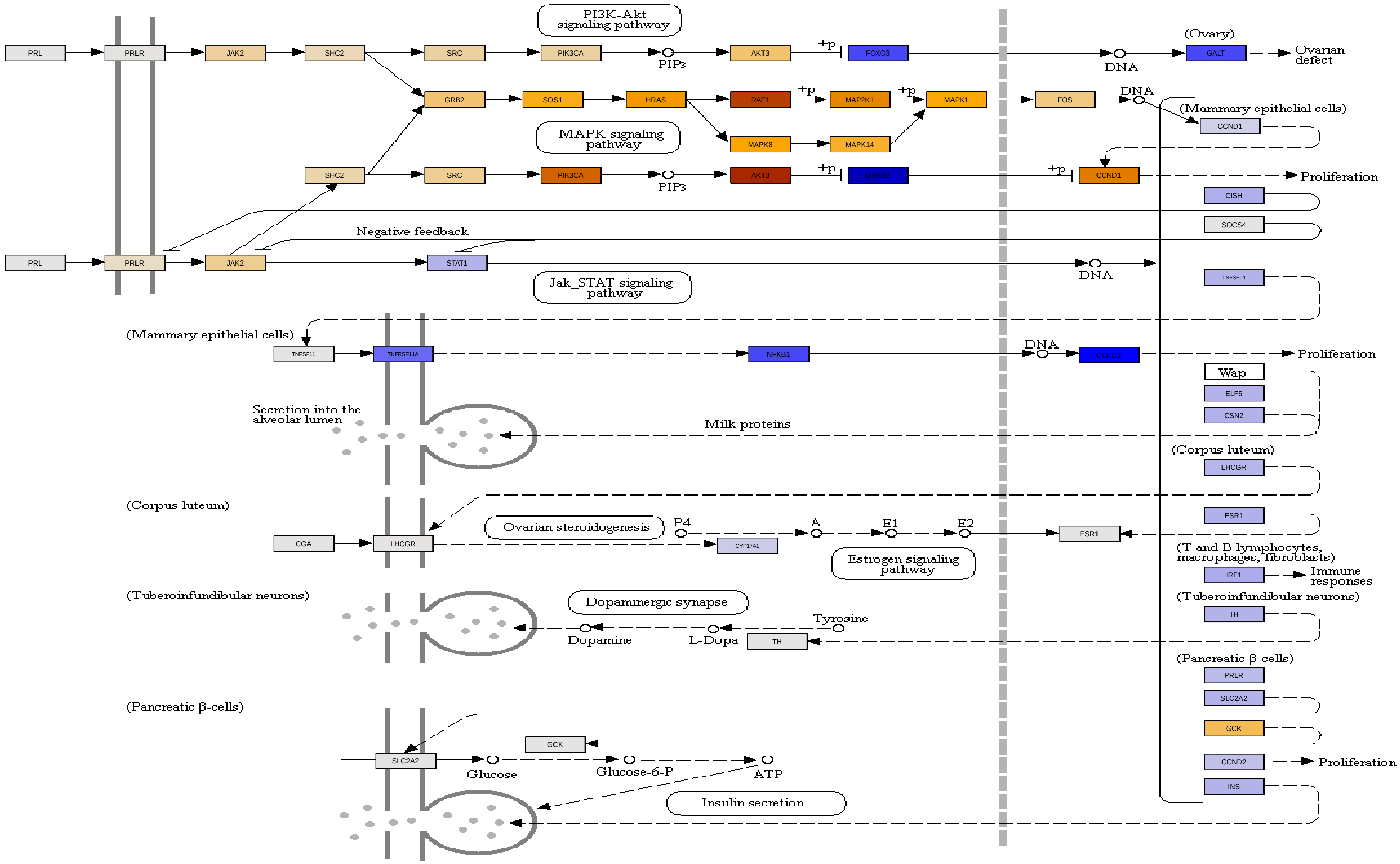

04917 9/28/15
(c) Kanehisa Laboratories

Prolactin_signaling_pathway
Baby

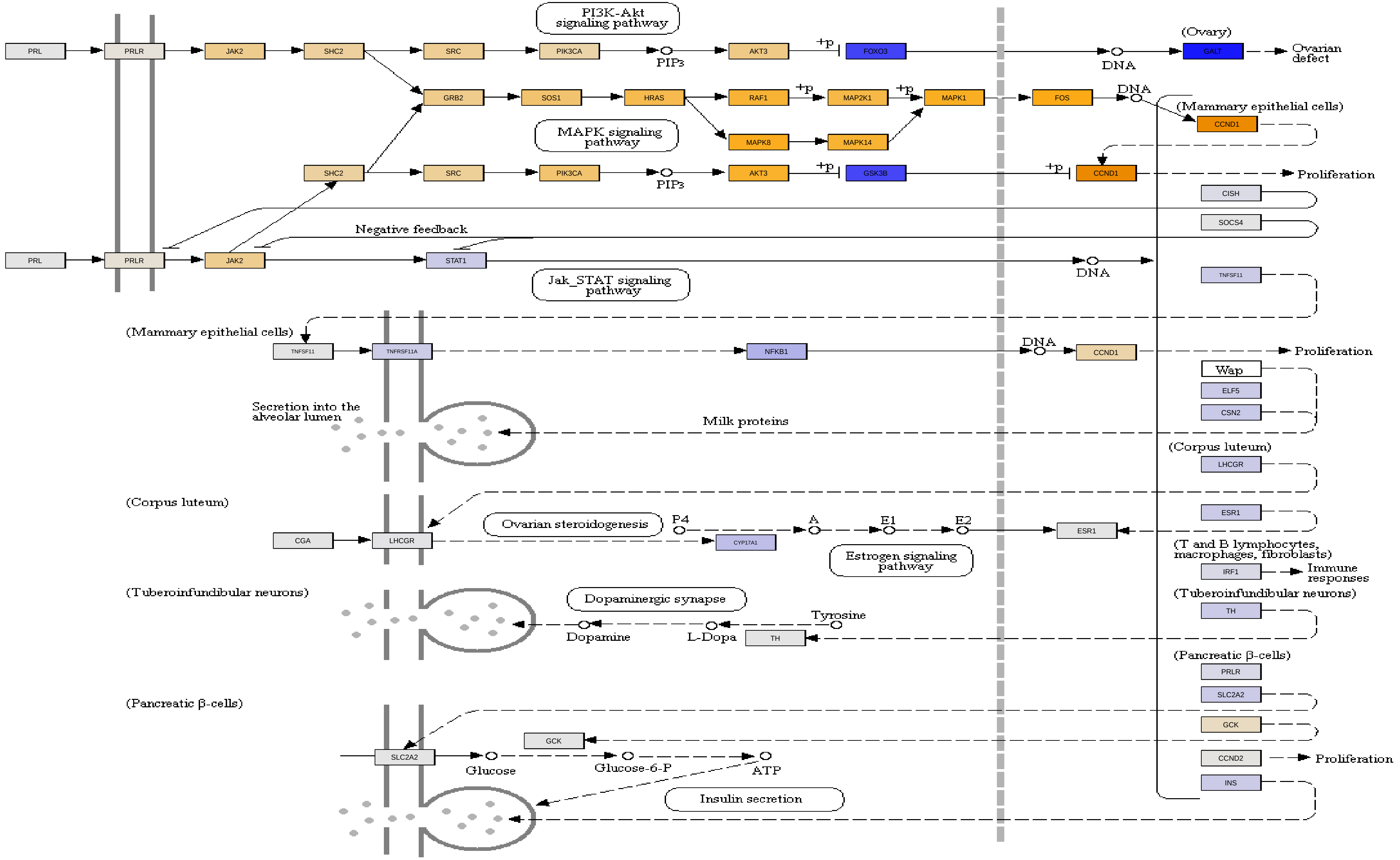

04917 9/28/15
(c) Kanehisa Laboratories

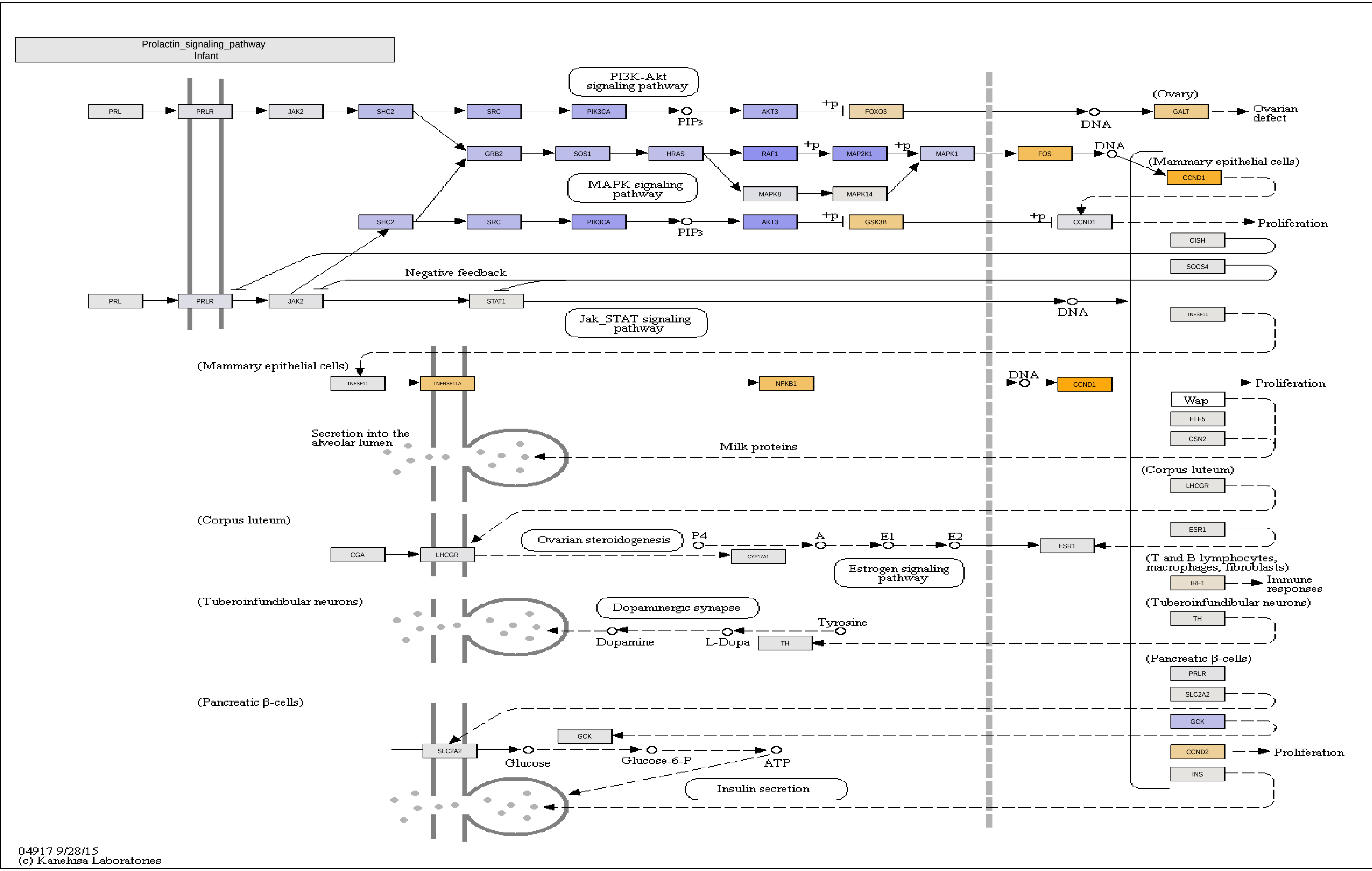
Prolactin_signaling_pathway
Infant
PRL
PRLR
JAK2
SHC2
SRC
GRB2
SOS1
HRAS
PIK3CA
PI3K-Akt signaling pathway
MAPK signaling pathway
PIP3
AKT3
+p
FOXO3
RAF1
MAP2K1
MAPK1
MAPK8
MAPK14
FOS
GSK3B
DNA
(Ovary)
GALT
Ovarian defect
(Mammary epithelial cells)
CCND1
Proliferation
CISH
SOCS4
Negative feedback
STAT1
Jak_STAT signaling pathway
TNFSF11
TNFRSF11A
NFKB1
Wap
ELF5
CSN2
Secretion into the alveolar lumen
Milk proteins
(Corpus luteum)
LHCGR
CGA
Ovarian steroidogenesis
P4
CYP17A1
A
E1
E2
ESR1
Estrogen signaling pathway
(T and B lymphocytes, macrophages, fibroblasts)
IRF1
Immune responses
(Tuberoinfundibular neurons)
Dopaminergic synapse
Dopamine
L-Dopa
Tyrosine
TH
(Pancreatic β-cells)
SLC2A2
GCK
Glucose
Glucose-6-P
ATP
Insulin secretion
CCND2
INS
04917 9/28/15
(c) Kanehisa Laboratories

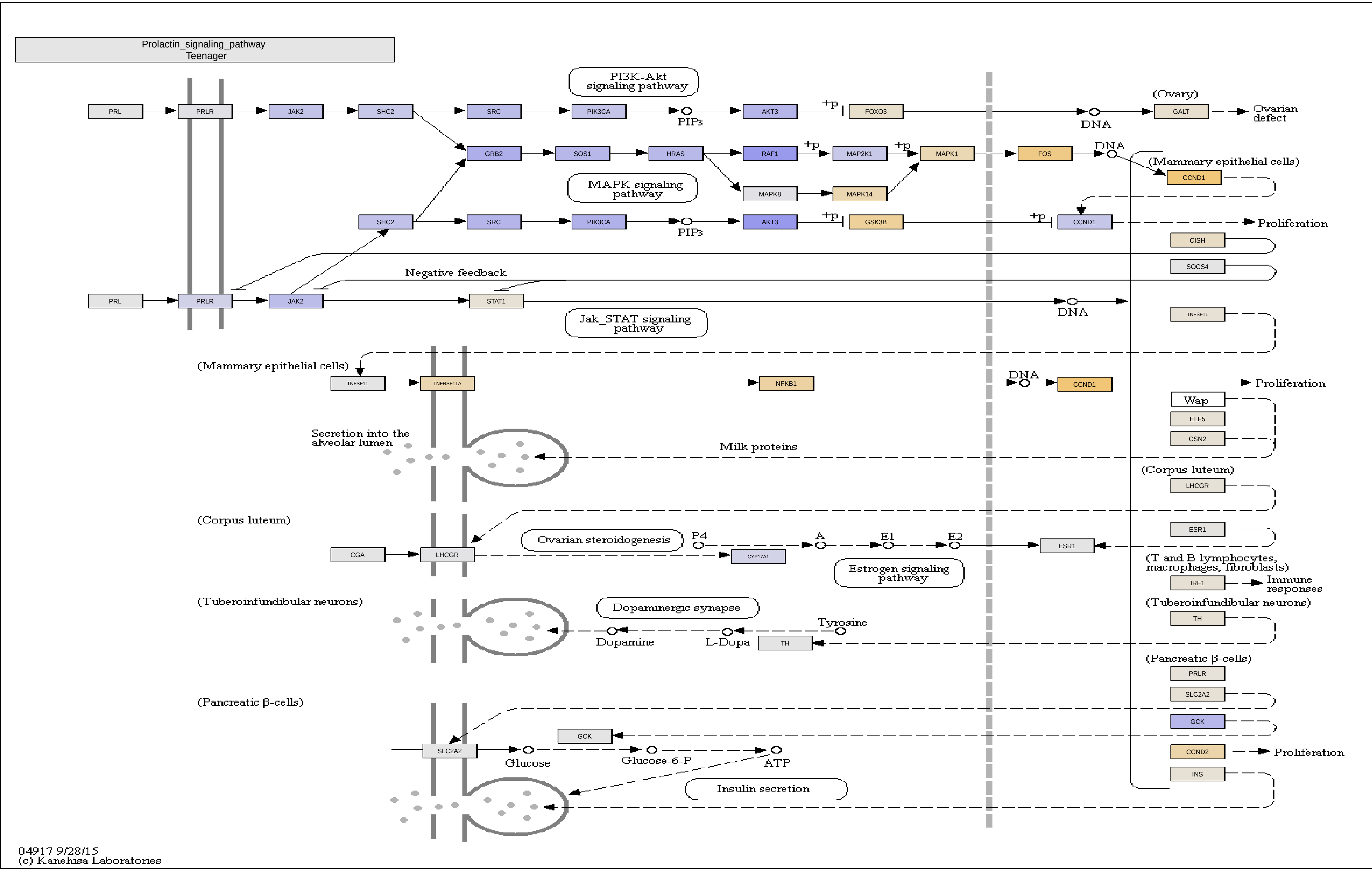
Prolactin_signaling_pathway
Teenager
PRL
PRLR
JAK2
SHC2
SRC
PIK3CA
PIP3
AKT3
+p
FOXO3
DNA
GALT
(Ovary)
Ovarian defect
PI3K-Akt signaling pathway
GRB2
SOS1
HRAS
RAF1
MAP2K1
MAPK1
FOS
MAPK8
MAPK14
MAPK signaling pathway
(Mammary epithelial cells)
CCND1
GSK3B
Proliferation
CISH
SOCS4
Negative feedback
STAT1
Jak_STAT signaling pathway
TNFSF11
TNFRSF11A
NFKB1
Wap
ELF5
CSN2
Secretion into the alveolar lumen
Milk proteins
(Corpus luteum)
LHCGR
CGA
Ovarian steroidogenesis
P4
A
E1
E2
CYP17A1
ESR1
Estrogen signaling pathway
(T and B lymphocytes, macrophages, fibroblasts)
IRF1
Immune responses
(Tuberoinfundibular neurons)
Dopaminergic synapse
Dopamine
L-Dopa
Tyrosine
TH
(Pancreatic β-cells)
SLC2A2
GCK
Glucose
Glucose-6-P
ATP
Insulin secretion
CCND2
INS
04917 9/28/15
(c) Kanehisa Laboratories

Prolactin_signaling_pathway
Adult

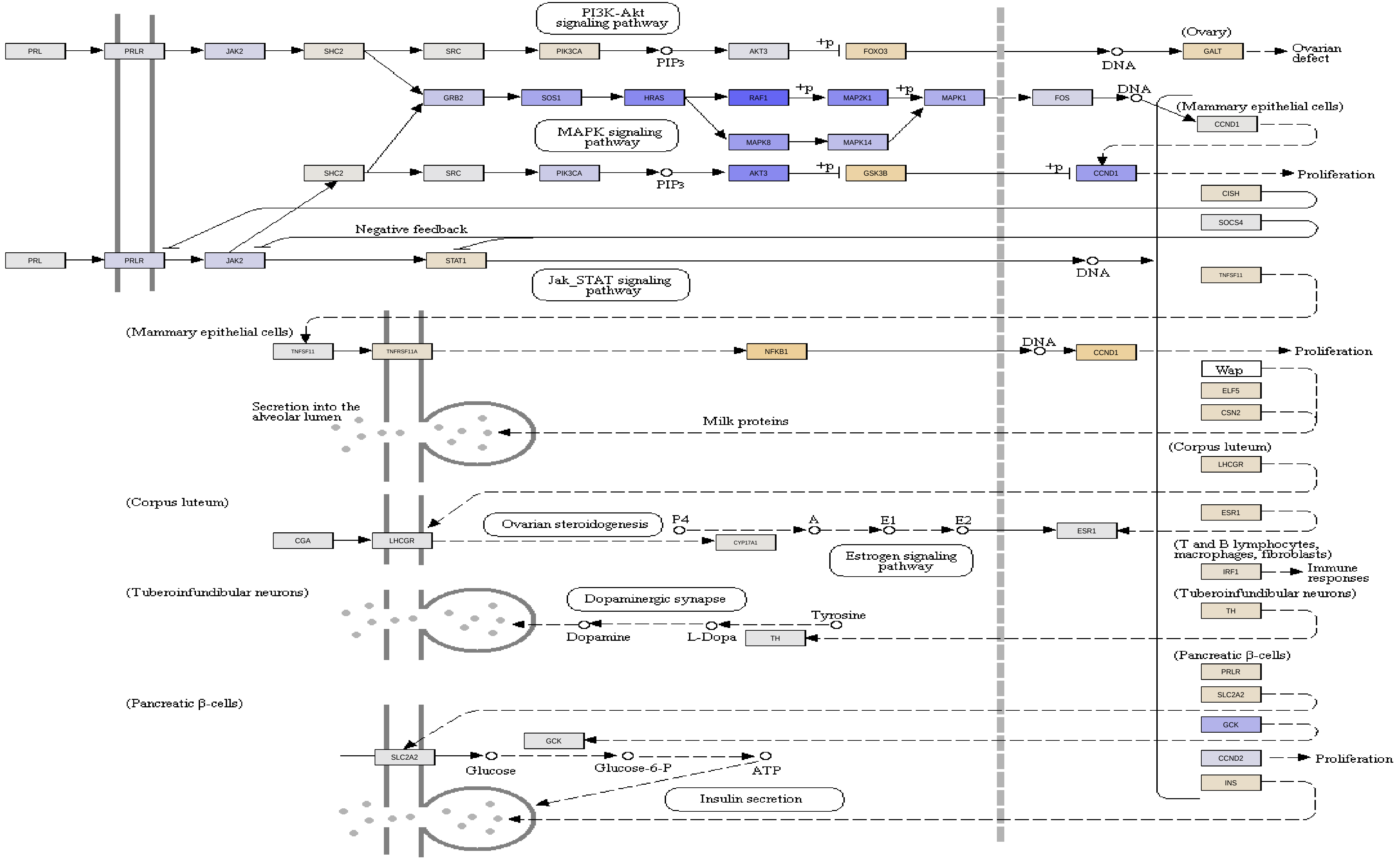

04917 9/28/15
(c) Kanehisa Laboratories

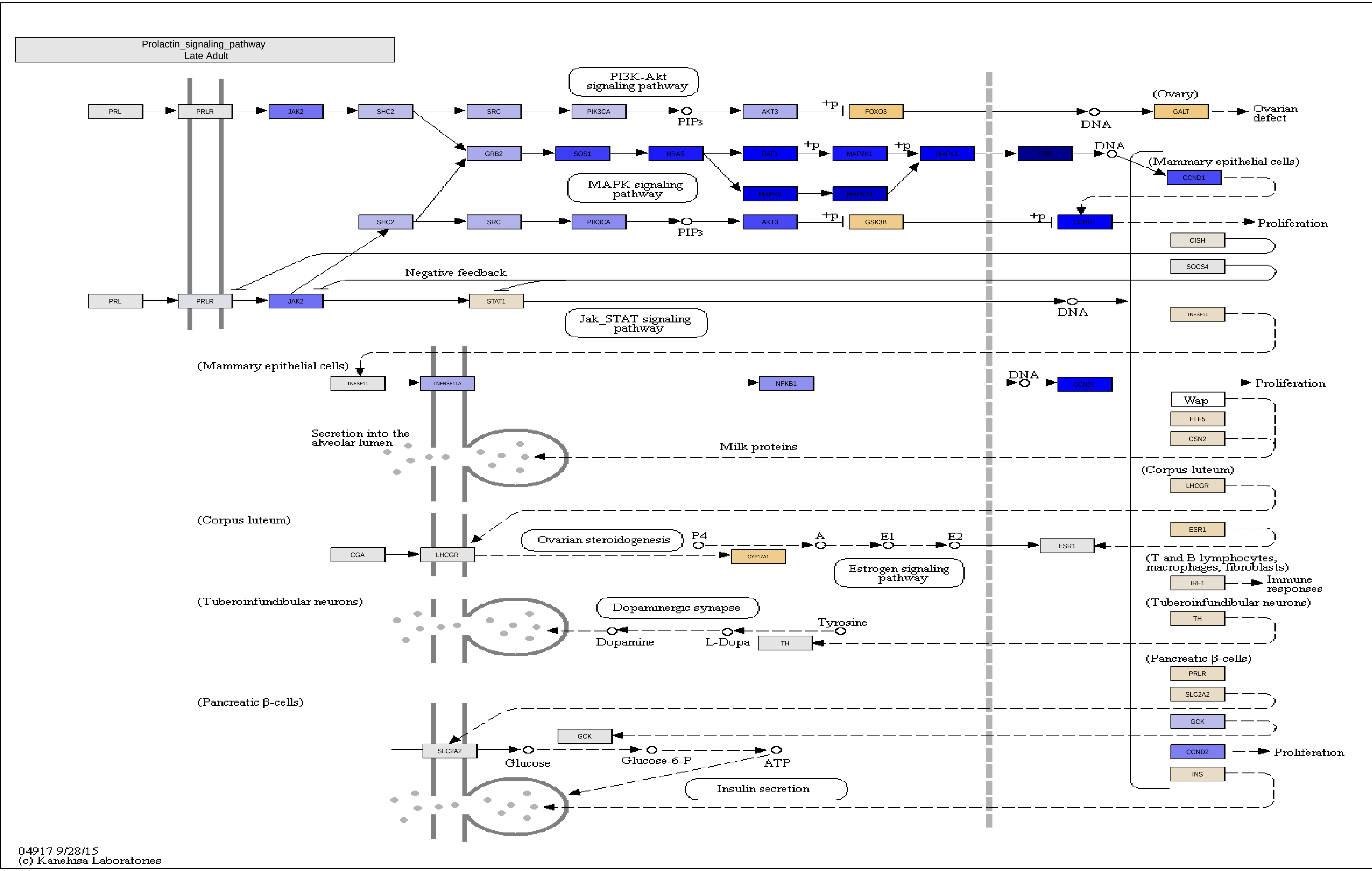
Prolactin_signaling_pathway
Late Adult
PRL
PRLR
JAK2
SHC2
SRC
PIK3CA
PI3K-Akt signaling pathway
PIP3
AKT3
+p
FOXO3
DNA
(Ovary)
GALT
Ovarian defect
GRB2
SOS1
HRAS
RAF1
MAP2K1
MAPK1
FOS
MAPK signaling pathway
MAPK8
MAPK14
(Mammary epithelial cells)
CCND1
GSK3B
Proliferation
CISH
SOCS4
Negative feedback
STAT1
Jak_STAT signaling pathway
TNFSF11
TNFRSF11A
NFKB1
Wap
ELF5
CSN2
Secretion into the alveolar lumen
Milk proteins
(Corpus luteum)
LHCGR
ESR1
CGA
Ovarian steroidogenesis
P4
A
E1
E2
CYP17A1
Estrogen signaling pathway
(T and B lymphocytes, macrophages, fibroblasts)
IRF1
Immune responses
(Tuberoinfundibular neurons)
TH
Dopaminergic synapse
Tyrosine
Dopamine
L-Dopa
(Pancreatic β-cells)
SLC2A2
GCK
CCND2
INS
Glucose
Glucose-6-P
ATP
Insulin secretion
04917 9/28/15
(c) Kanehisa Laboratories
