# Supplementary material for: The Transcriptome and Methylome of the Developing and Aging Brain and Their Relations to Gliomas and Psychological Disorders
Source: Cells. 2022 Jan 21;11(3):362. doi: 10.3390/cells11030362 (PMC8834030; doi:10.3390/cells11030362)
Supplement: Supplementary file 1 [file cells-11-00362-s001.zip › Suppl-Material-S3-Pathways-PSF_Expression/Rap1_signaling_pathway.pdf]

Rap1 signaling pathway

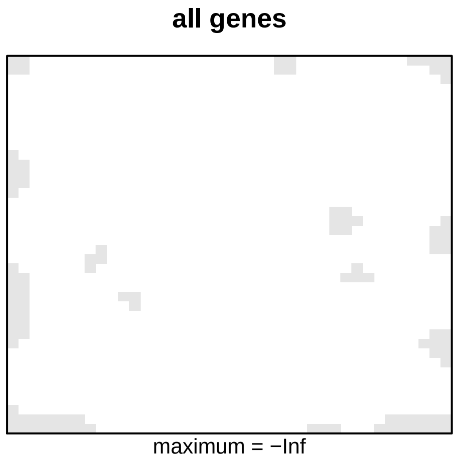

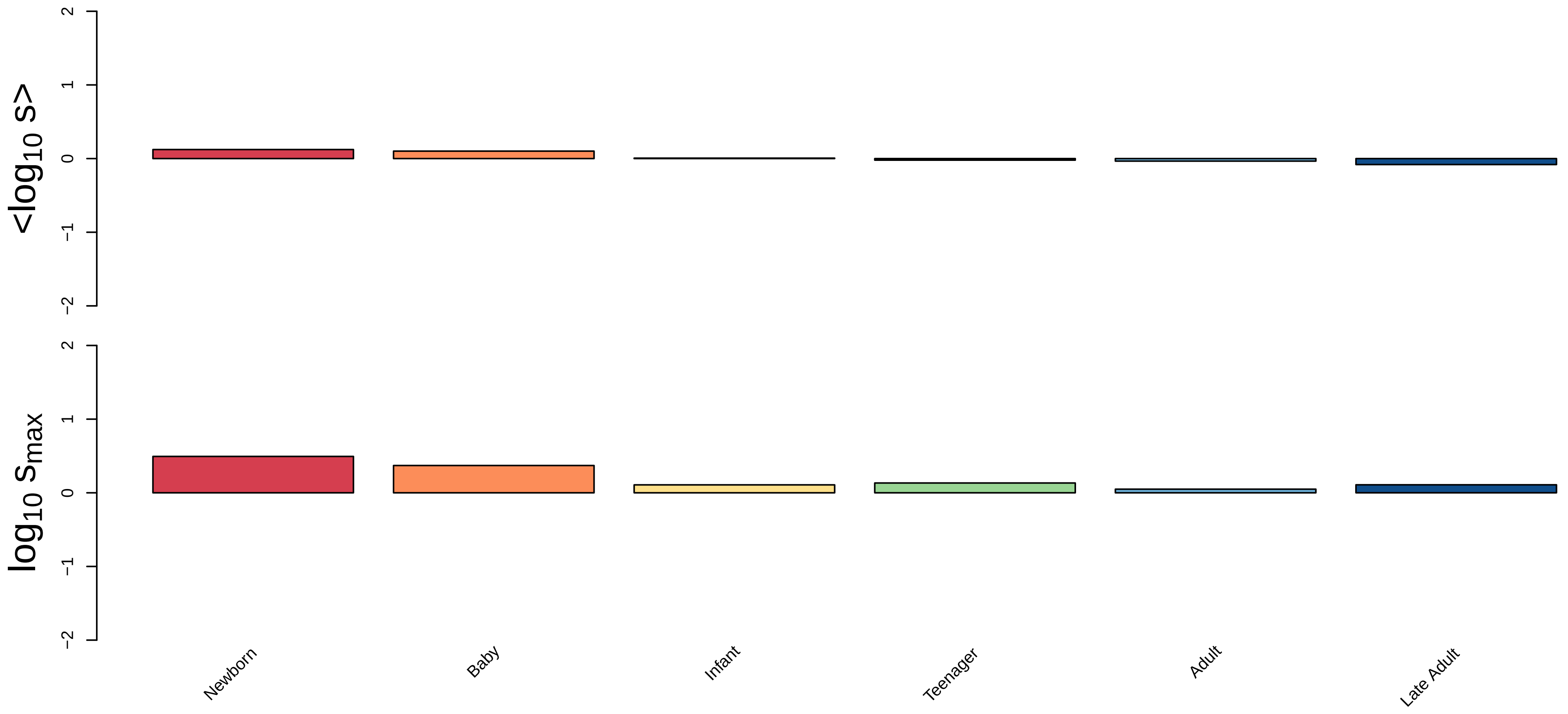

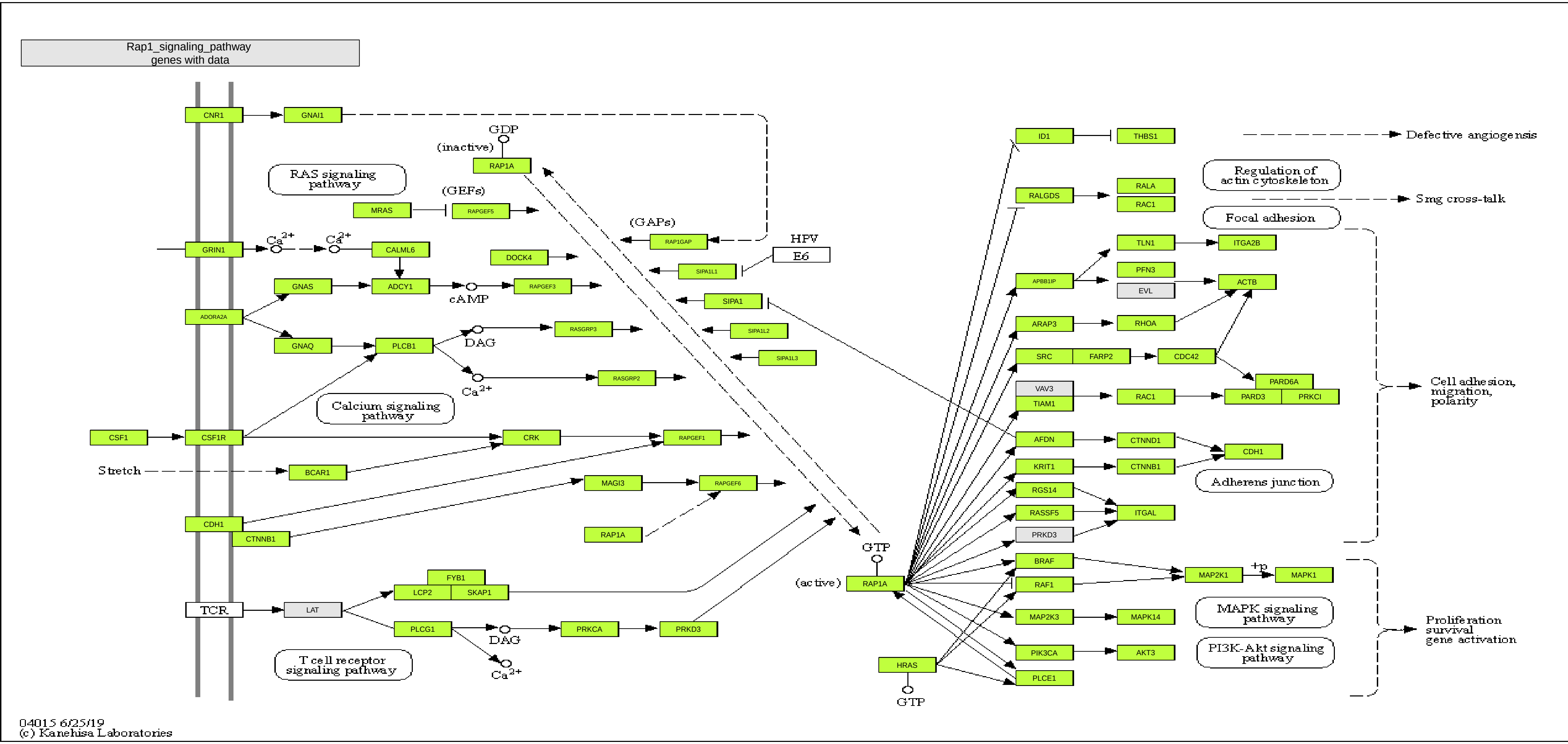

Rap1_signaling_pathway
genes with data
CNR1
GNAI1
GDP
(inactive)
RAP1A
RAS signaling pathway
(GEFs)
MRAS
RAPGEF5
(GAPs)
RAP1GAP
HPV
E6
GRIN1
Ca2+
Ca2+
CALML6
DOCK4
SIPA1L1
GNAS
ADCY1
cAMP
RAPGEF3
SIPA1
ADORA2A
SIPA1L2
GNAQ
PLCB1
DAG
RASGRP3
SIPA1L3
Ca2+
RASGRP2
Calcium signaling pathway
CSF1
CSF1R
CRK
RAPGEF1
Stretch
BCAR1
MAGI3
RAPGEF6
CDH1
CTNNB1
RAP1A
FYB1
LCP2
SKAP1
TCR
LAT
PLCG1
DAG
PRKCA
PRKD3
Ca2+
T cell receptor signaling pathway
GTP
(active)
RAP1A
HRAS
GTP
ID1
THBS1
Defective angiogensis
RALGDS
RALA
RAC1
Regulation of actin cytoskeleton
Smg cross-talk
Focal adhesion
TLN1
ITGA2B
APBB1IP
PFN3
EVL
ACTB
ARAP3
RHOA
SRC
FARP2
CDC42
PARD6A
VAV3
TIAM1
RAC1
PARD3
PRKCI
Cell adhesion, migration, polarity
AFDN
CTNND1
CDH1
KRIT1
CTNNB1
Adherens junction
RGS14
RASSF5
ITGAL
PRKD3
BRAF
MAP2K1
+p
MAPK1
RAF1
MAPK signaling pathway
MAP2K3
MAPK14
PIK3CA
AKT3
PI3K-Akt signaling pathway
Proliferation survival gene activation
PLCE1
04015 6/25/19
(c) Kanehisa Laboratories

Rap1_signaling_pathway
sink nodes

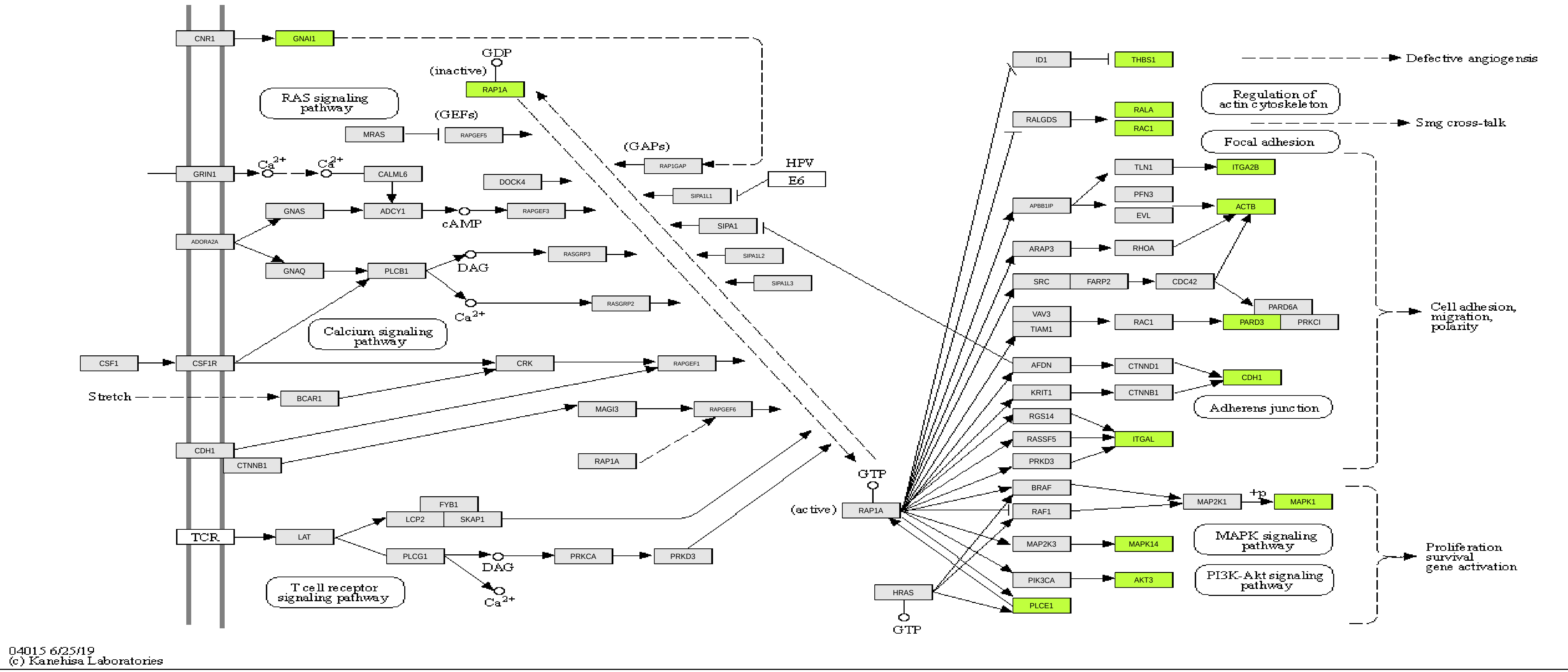

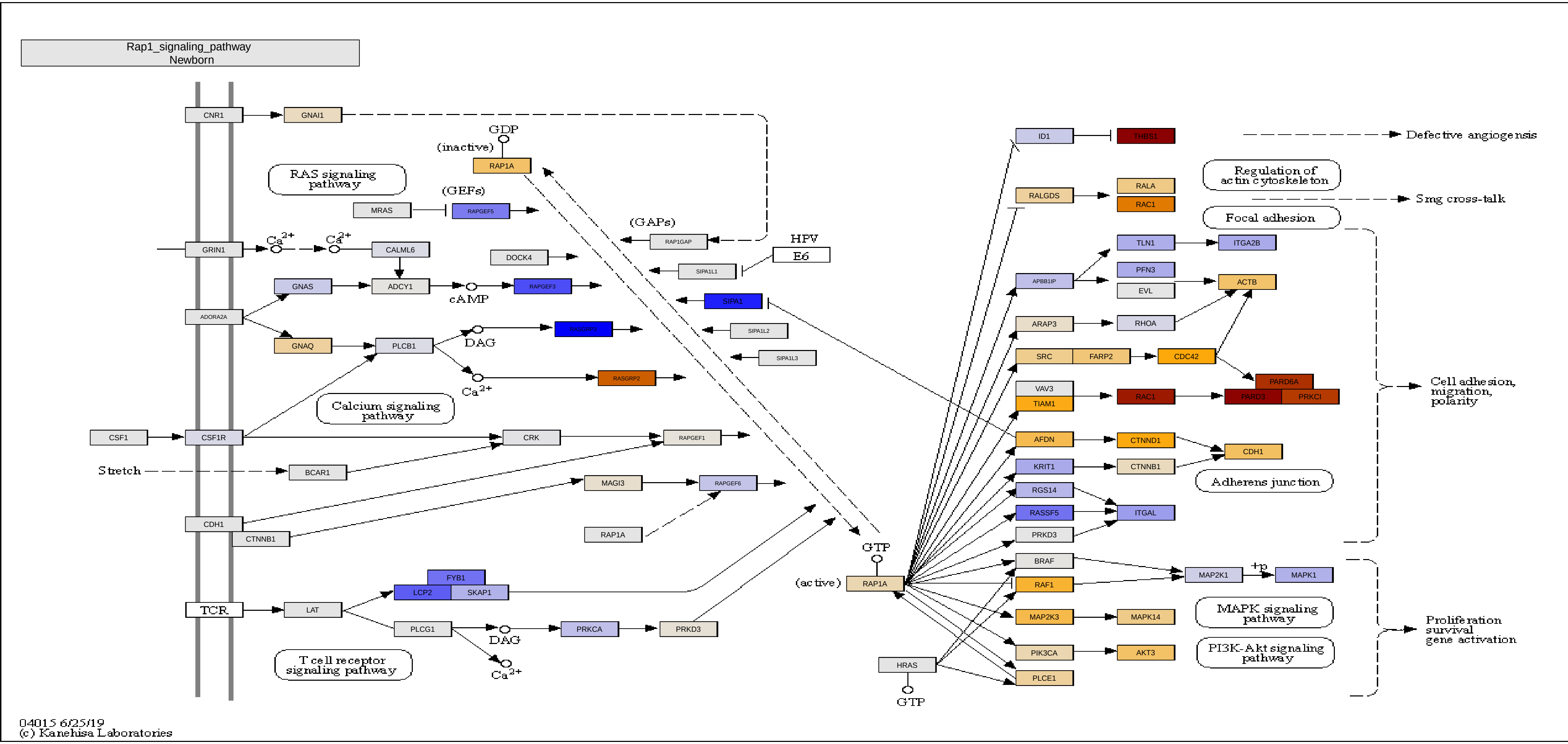
Rap1_signaling_pathway
Newborn
CNR1
GNAI1
GDP
(inactive)
RAP1A
RAS signaling pathway
(GEFs)
MRAS
RAPGEF5
(GAPs)
RAP1GAP
HPV
E6
GRIN1
Ca2+
CALML6
DOCK4
SIPA1L1
GNAS
ADCY1
cAMP
RAPGEF3
SIPA1
ADORA2A
SIPA1L2
GNAQ
PLCB1
DAG
RASGRP3
SIPA1L3
RASGRP2
Calcium signaling pathway
CSF1
CSF1R
CRK
RAPGEF1
Stretch
BCAR1
MAGI3
RAPGEF6
CDH1
CTNNB1
RAP1A
FYB1
LCP2
SKAP1
TCR
LAT
PLCG1
PRKCA
PRKD3
T cell receptor signaling pathway
GTP
(active)
HRAS
ID1
THBS1
Defective angiogensis
RALGDS
RALA
RAC1
Regulation of actin cytoskeleton
Smg cross-talk
Focal adhesion
TLN1
ITGA2B
APBB1IP
PFN3
EVL
ACTB
ARAP3
RHOA
SRC
FARP2
CDC42
PARD6A
VAV3
TIAM1
PARD3
PRKCI
Cell adhesion, migration, polarity
AFDN
CTNND1
CDH1
KRIT1
CTNNB1
Adherens junction
RGS14
RASSF5
ITGAL
PRKD3
BRAF
RAF1
MAP2K1
+p
MAPK1
MAP2K3
MAPK14
MAPK signaling pathway
PIK3CA
AKT3
PI3K-Akt signaling pathway
PLCE1
Proliferation survival gene activation
04015 6/25/19
(c) Kanehisa Laboratories

Rap1_signaling_pathway
Baby

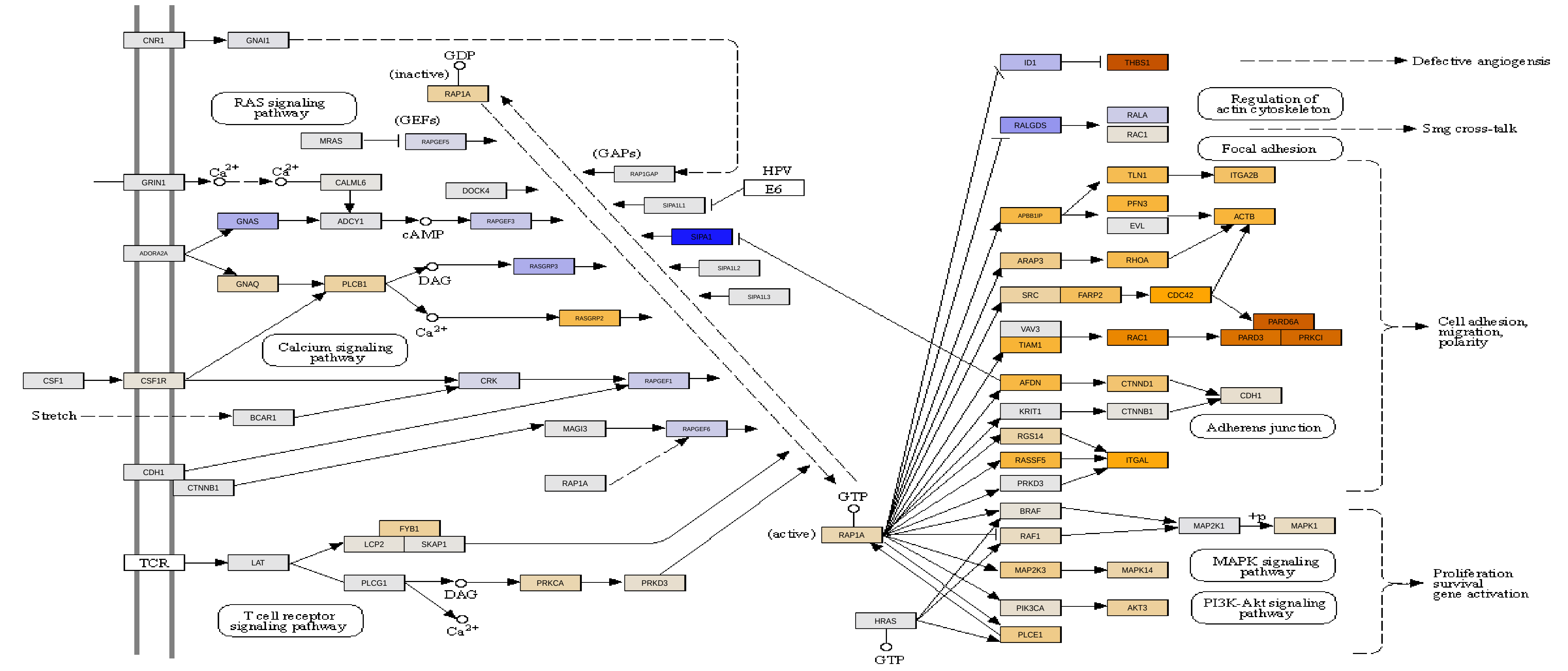

04015 6/25/19
(c) Kanehisa Laboratories

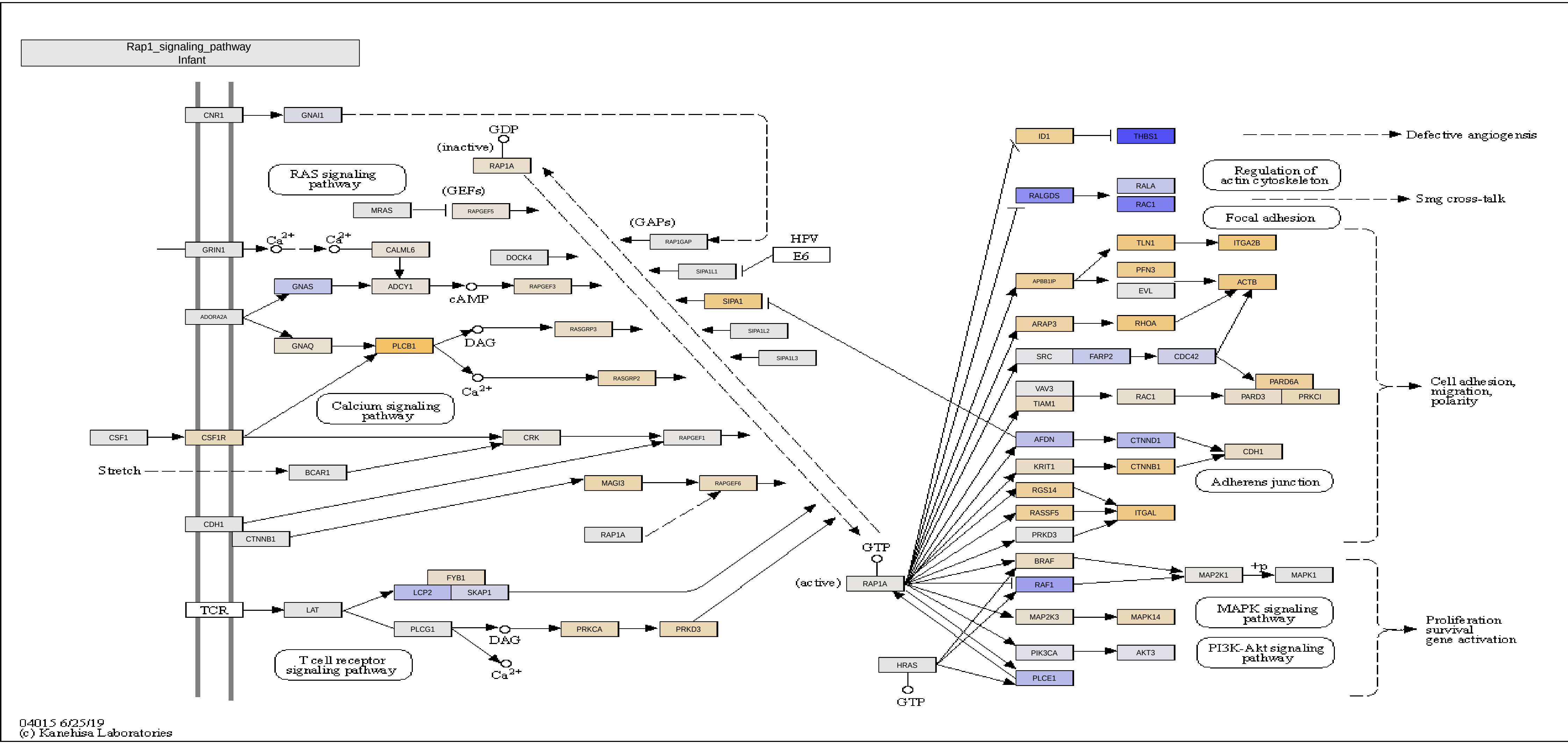
Rap1_signaling_pathway
Infant
CNR1
GNAI1
GDP
(inactive)
RAP1A
RAS signaling pathway
(GEFs)
MRAS
RAPGEF5
(GAPs)
RAP1GAP
HPV
E6
GRIN1
Ca2+
Ca2+
CALML6
DOCK4
SIPA1L1
GNAS
ADCY1
cAMP
RAPGEF3
SIPA1
ADORA2A
SIPA1L2
GNAQ
PLCB1
DAG
RASGRP3
SIPA1L3
Ca2+
RASGRP2
Calcium signaling pathway
CSF1
CSF1R
CRK
RAPGEF1
Stretch
BCAR1
MAGI3
RAPGEF6
CDH1
CTNNB1
RAP1A
FYB1
LCP2
SKAP1
TCR
LAT
PLCG1
DAG
PRKCA
PRKD3
Ca2+
T cell receptor signaling pathway
GTP
(active)
RAP1A
HRAS
GTP
ID1
THBS1
Defective angiogensis
RALGDS
RALA
RAC1
Regulation of actin cytoskeleton
Smg cross-talk
Focal adhesion
TLN1
ITGA2B
APBB1IP
PFN3
EVL
ACTB
ARAP3
RHOA
SRC
FARP2
CDC42
PARD6A
VAV3
TIAM1
RAC1
PARD3
PRKCI
Cell adhesion, migration, polarity
AFDN
CTNND1
CDH1
KRIT1
CTNNB1
Adherens junction
RGS14
RASSF5
ITGAL
PRKD3
BRAF
MAP2K1
+p
MAPK1
RAF1
MAPK signaling pathway
MAP2K3
MAPK14
PIK3CA
AKT3
PI3K-Akt signaling pathway
PLCE1
Proliferation survival gene activation
04015 6/25/19
(c) Kanehisa Laboratories

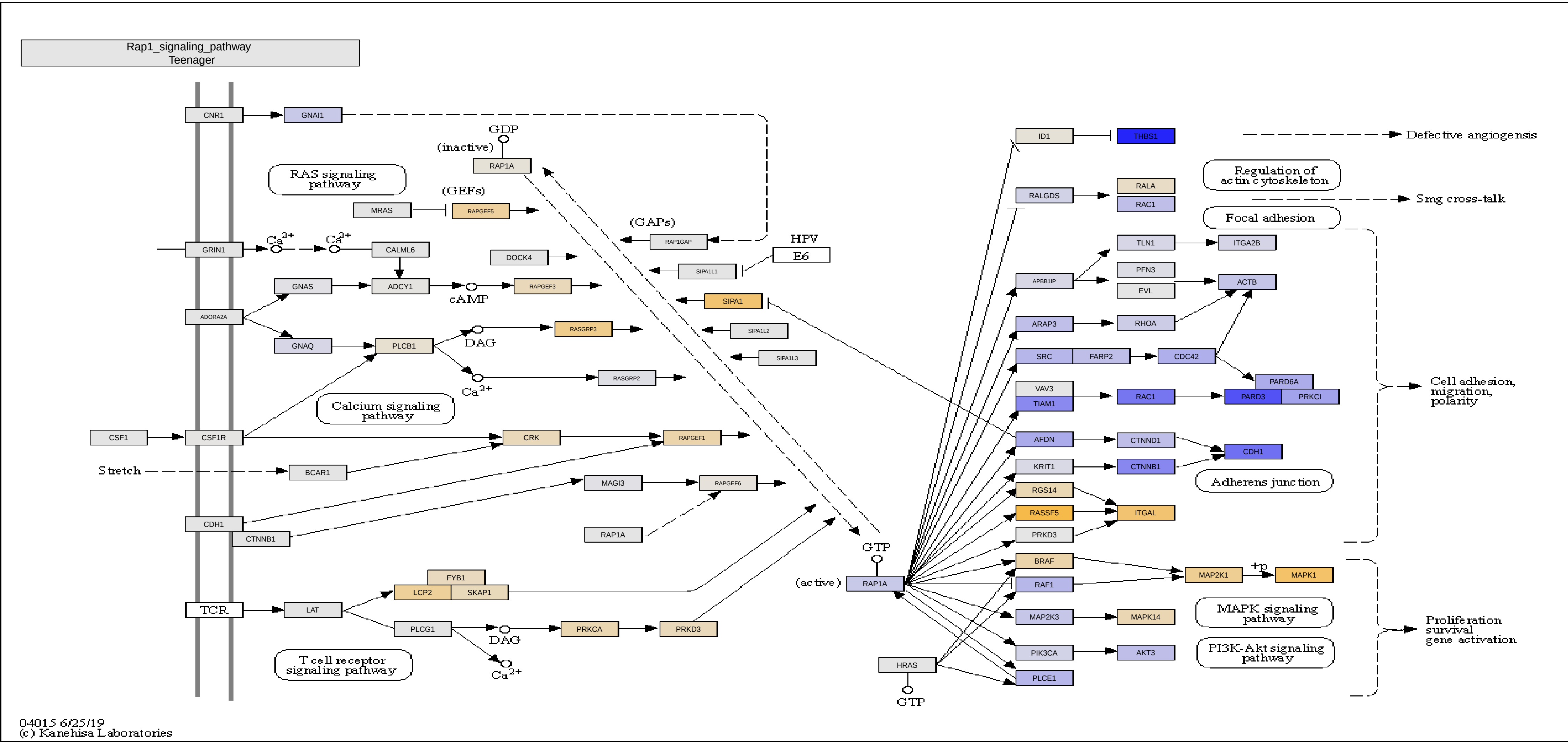

Rap1_signaling_pathway
Teenager
CNR1
GNAI1
GDP
(inactive)
RAP1A
RAS signaling pathway
(GEFs)
MRAS
RAPGEF5
(GAPs)
RAP1GAP
HPV
E6
GRIN1
Ca2+
Ca2+
CALML6
DOCK4
SIPA1L1
GNAS
ADCY1
cAMP
RAPGEF3
SIPA1
ADORA2A
SIPA1L2
GNAQ
PLCB1
DAG
RASGRP3
SIPA1L3
Ca2+
RASGRP2
Calcium signaling pathway
CSF1
CSF1R
CRK
RAPGEF1
Stretch
BCAR1
MAGI3
RAPGEF6
CDH1
CTNNB1
RAP1A
GTP
(active)
RAP1A
FYB1
LCP2
SKAP1
TCR
LAT
PLCG1
DAG
PRKCA
PRKD3
Ca2+
T cell receptor signaling pathway
HRAS
GTP
ID1
THBS1
Defective angiogensis
RALGDS
RALA
RAC1
Regulation of actin cytoskeleton
Smg cross-talk
Focal adhesion
TLN1
ITGA2B
APBB1IP
PFN3
EVL
ACTB
ARAP3
RHOA
SRC
FARP2
CDC42
PARD6A
VAV3
TIAM1
RAC1
PARD3
PRKCI
Cell adhesion, migration, polarity
AFDN
CTNND1
CDH1
KRIT1
CTNNB1
Adherens junction
RGS14
RASSF5
ITGAL
PRKD3
BRAF
MAP2K1
+p
MAPK1
RAF1
MAP2K3
MAPK14
MAPK signaling pathway
PIK3CA
AKT3
PI3K-Akt signaling pathway
PLCE1
Proliferation survival gene activation
04015 6/25/19
(c) Kanehisa Laboratories

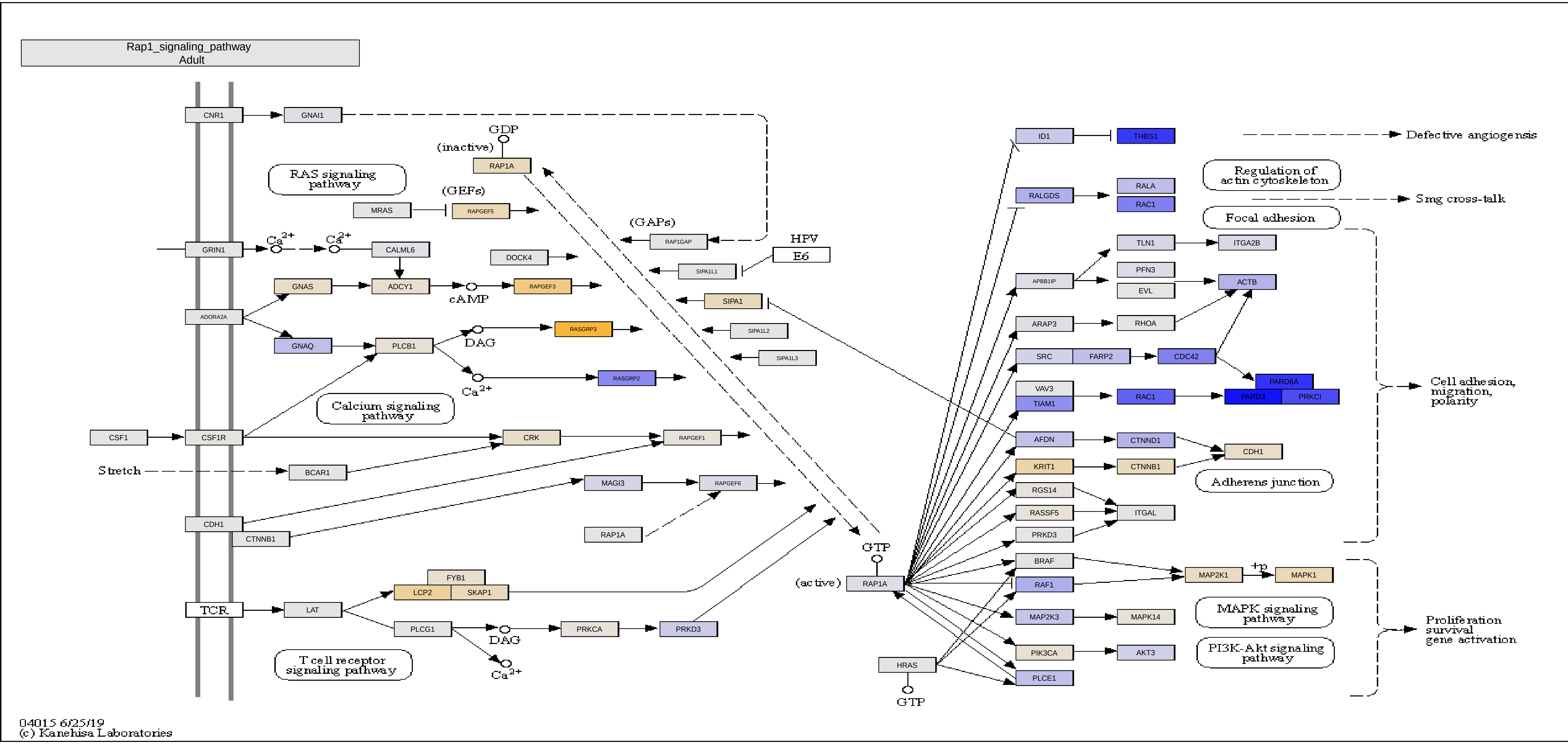
Rap1_signaling_pathway
Adult
CNR1
GNAI1
GDP
(inactive)
RAP1A
RAS signaling pathway
(GEFs)
MRAS
RAPGEF5
(GAPs)
RAP1GAP
HPV
E6
SIPA1L1
GRIN1
Ca2+
Ca2+
CALML6
DOCK4
GNAS
ADCY1
cAMP
RAPGEF3
SIPA1
ADORA2A
SIPA1L2
SIPA1L3
GNAQ
PLCB1
DAG
RASGRP3
Ca2+
RASGRP2
Calcium signaling pathway
CSF1
CSF1R
CRK
RAPGEF1
Stretch
BCAR1
MAGI3
RAPGEF6
CDH1
CTNNB1
RAP1A
FYB1
LCP2
SKAP1
TCR
LAT
PLCG1
DAG
PRKCA
PRKD3
Ca2+
T cell receptor signaling pathway
GTP
(active)
RAP1A
HRAS
GTP
ID1
THBS1
Defective angiogensis
Regulation of actin cytoskeleton
RALGDS
RALA
RAC1
Smg cross-talk
Focal adhesion
TLN1
ITGA2B
APBB1IP
PFN3
EVL
ACTB
ARAP3
RHOA
SRC
FARP2
CDC42
PARD6A
VAV3
TIAM1
RAC1
PARD3
PRKCI
Cell adhesion, migration, polarity
AFDN
CTNND1
CDH1
KRIT1
CTNNB1
Adherens junction
RGS14
RASSF5
ITGAL
PRKD3
BRAF
MAP2K1
+p
MAPK1
RAF1
MAP2K3
MAPK14
MAPK signaling pathway
PIK3CA
AKT3
PI3K-Akt signaling pathway
PLCE1
Proliferation survival gene activation
04015 6/25/19
(c) Kanehisa Laboratories

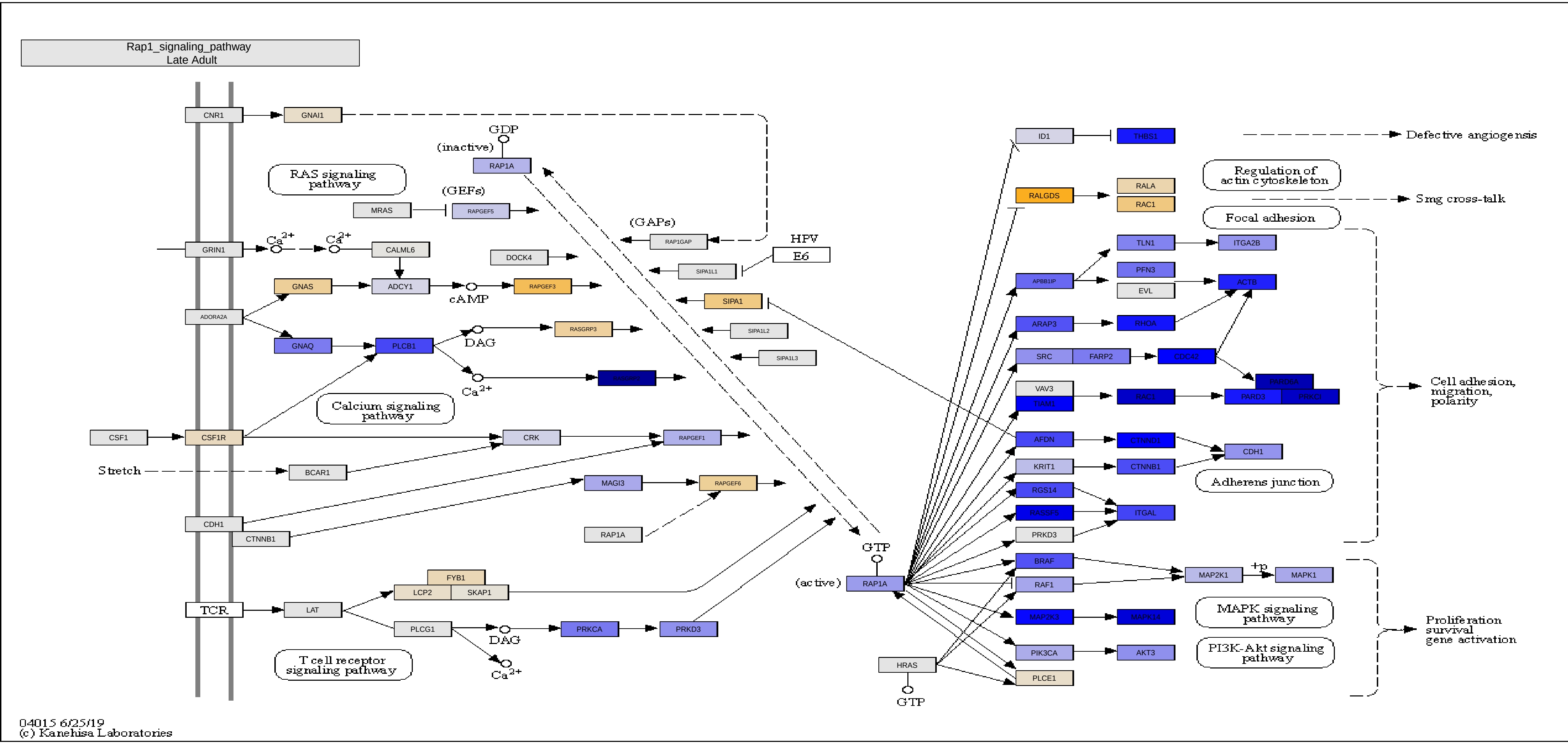
Rap1_signaling_pathway
Late Adult
CNR1
GNAI1
GDP
(inactive)
RAP1A
RAS signaling pathway
(GEFs)
MRAS
RAPGEF5
(GAPs)
RAP1GAP
HPV
E6
GRIN1
Ca2+
CALML6
DOCK4
SIPA1L1
GNAS
ADCY1
cAMP
RAPGEF3
SIPA1
ADORA2A
SIPA1L2
GNAQ
PLCB1
DAG
RASGRP3
SIPA1L3
Calcium signaling pathway
CSF1
CSF1R
CRK
RAPGEF1
Stretch
BCAR1
MAGI3
RAPGEF6
CDH1
CTNNB1
RAP1A
GTP
(active)
FYB1
LCP2
SKAP1
TCR
LAT
PLCG1
PRKCA
PRKD3
T cell receptor signaling pathway
HRAS
ID1
THBS1
Defective angiogenesis
RALGDS
RALA
RAC1
Regulation of actin cytoskeleton
Smg cross-talk
Focal adhesion
TLN1
ITGA2B
APBB1IP
PFN3
EVL
ACTB
ARAP3
RHOA
SRC
FARP2
CDC42
PARD6A
VAV3
TIAM1
PARD3
PRKCI
Cell adhesion, migration, polarity
AFDN
CTNND1
CDH1
KRIT1
CTNNB1
Adherens junction
RGS14
RASSF5
ITGAL
PRKD3
BRAF
RAF1
MAP2K1
+p
MAPK1
MAP2K3
MAPK14
MAPK signaling pathway
PIK3CA
AKT3
PI3K-Akt signaling pathway
PLCE1
Proliferation survival gene activation
04015 6/25/19
(c) Kanehisa Laboratories
