# Supplementary material for: The Transcriptome and Methylome of the Developing and Aging Brain and Their Relations to Gliomas and Psychological Disorders
Source: Cells. 2022 Jan 21;11(3):362. doi: 10.3390/cells11030362 (PMC8834030; doi:10.3390/cells11030362)
Supplement: Supplementary file 1 [file cells-11-00362-s001.zip › Suppl-Material-S3-Pathways-PSF_Expression/Relaxin_signaling_pathway.pdf]

Relaxin signaling pathway

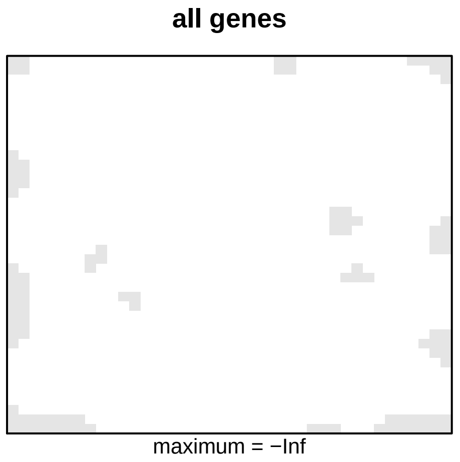

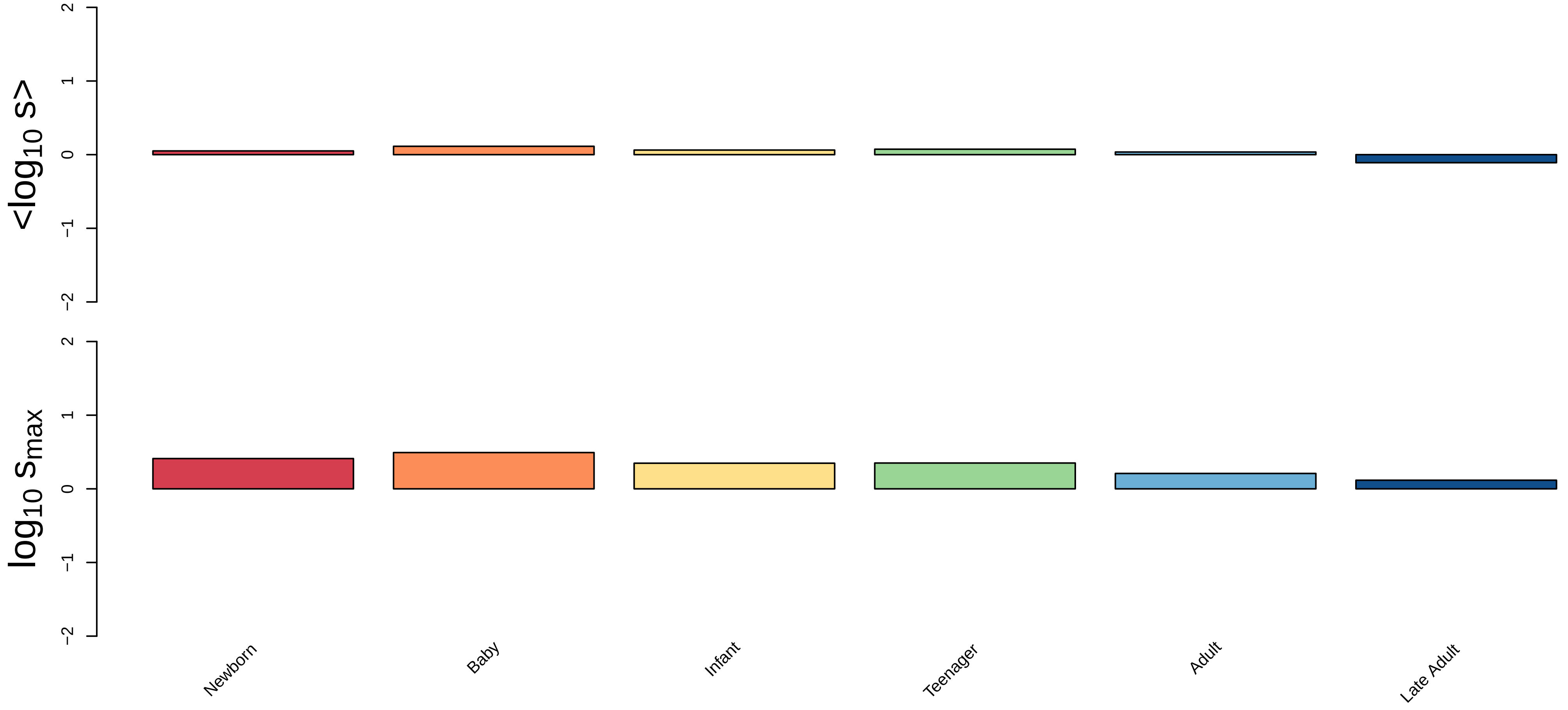

Relaxin_signaling_pathway
genes with data

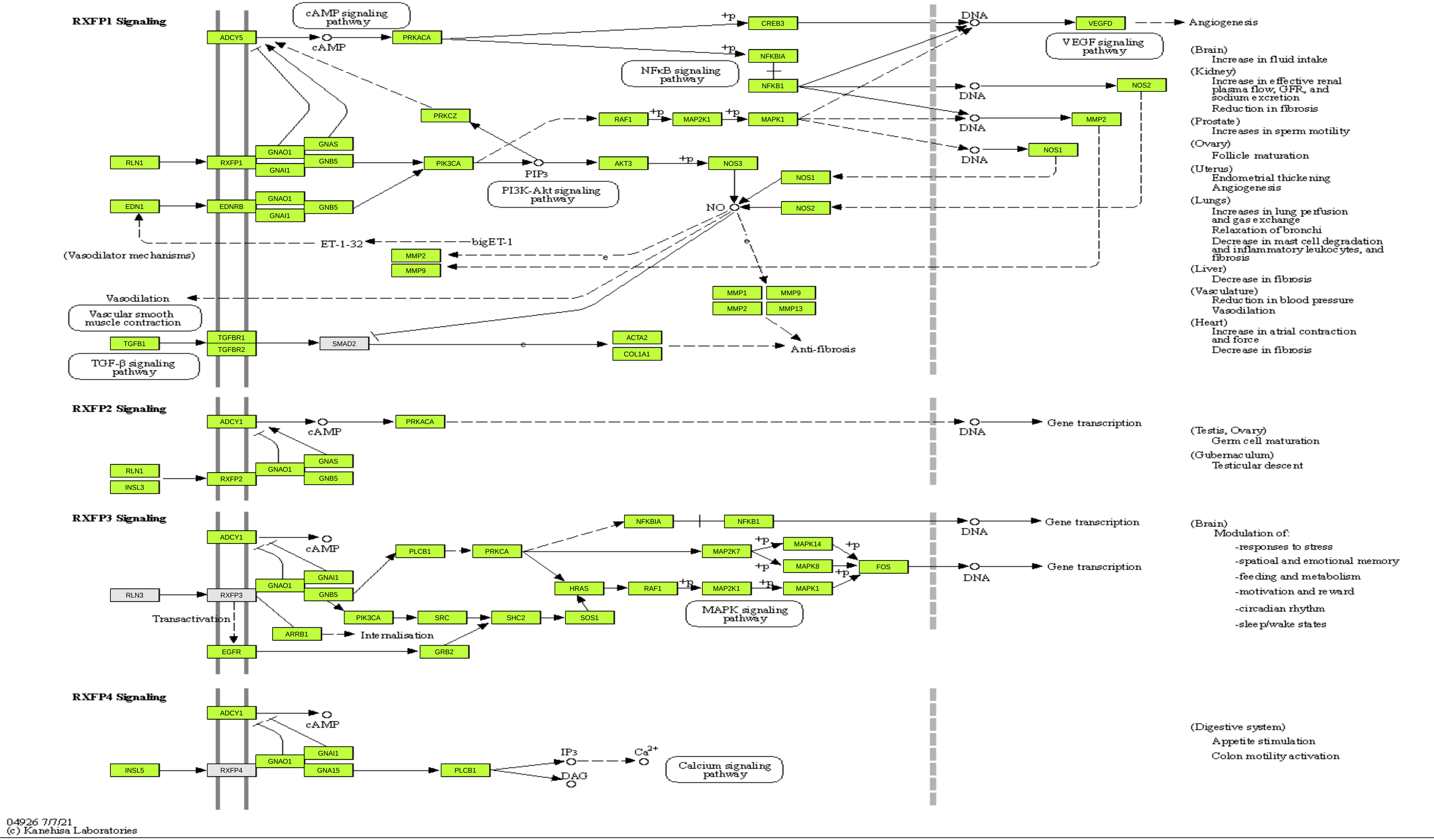

Relaxin_signaling_pathway
sink nodes

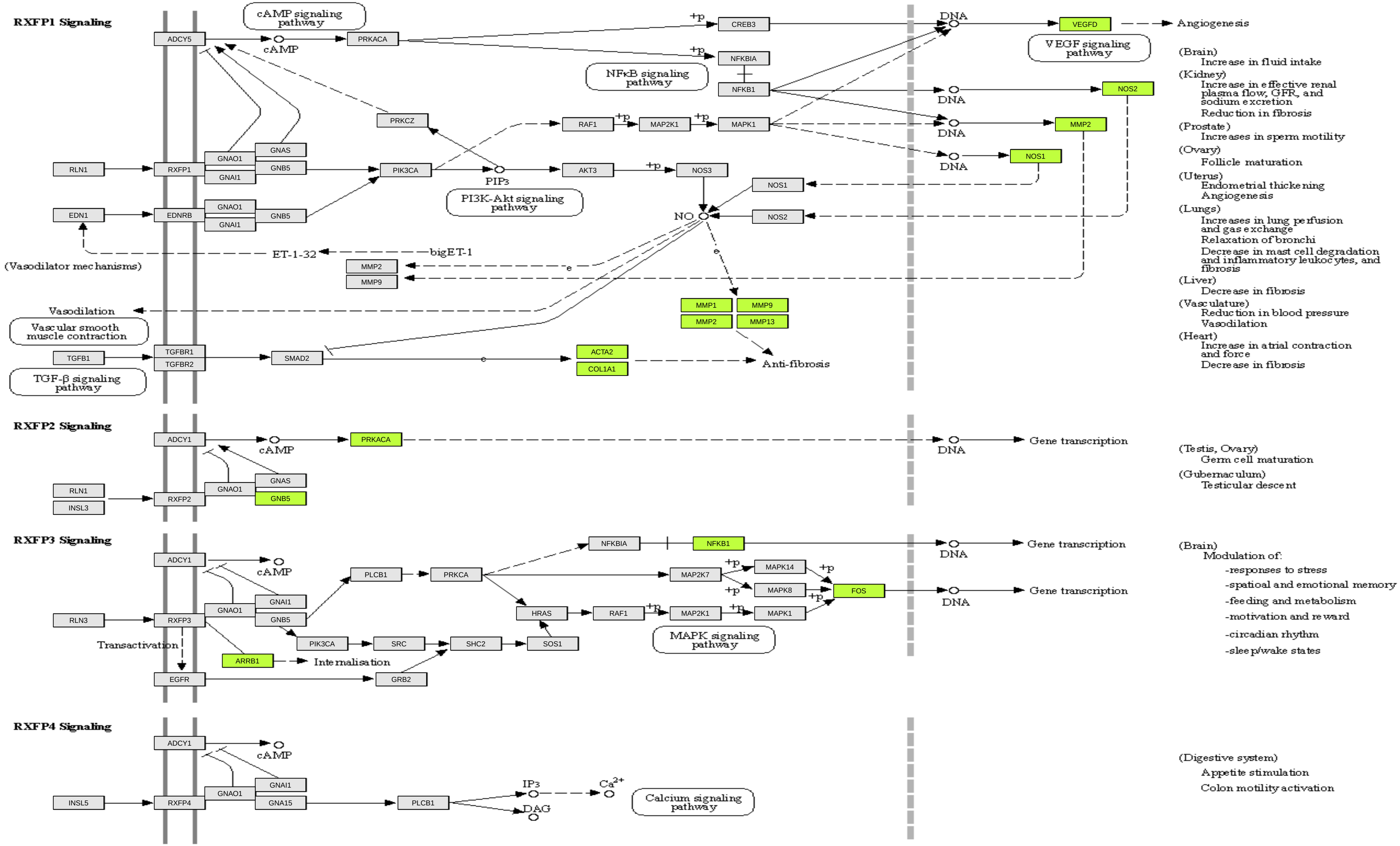

04926 7/7/21
(c) Kanehisa Laboratories

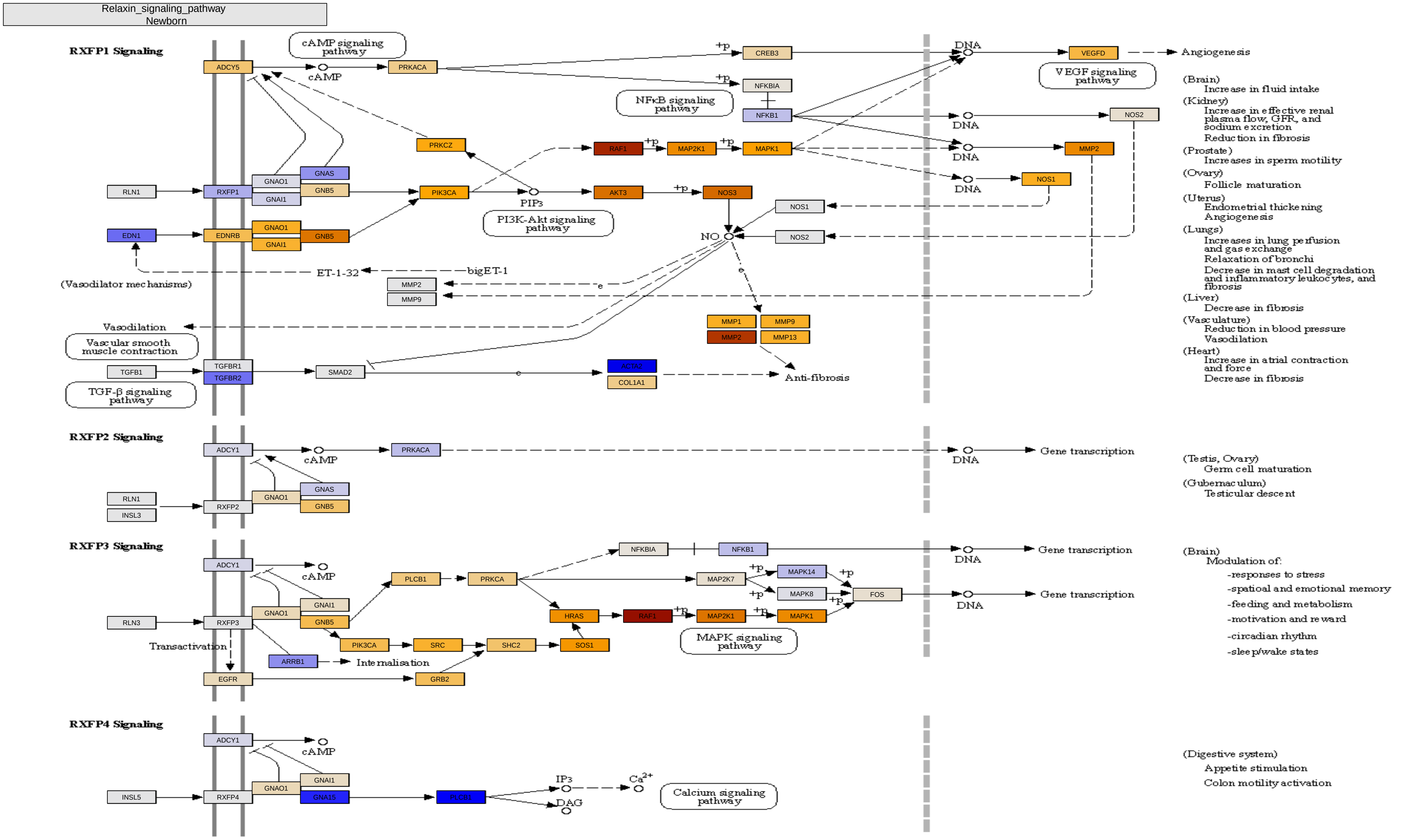

04926 7/7/21

(c) Kanehisa Laboratories

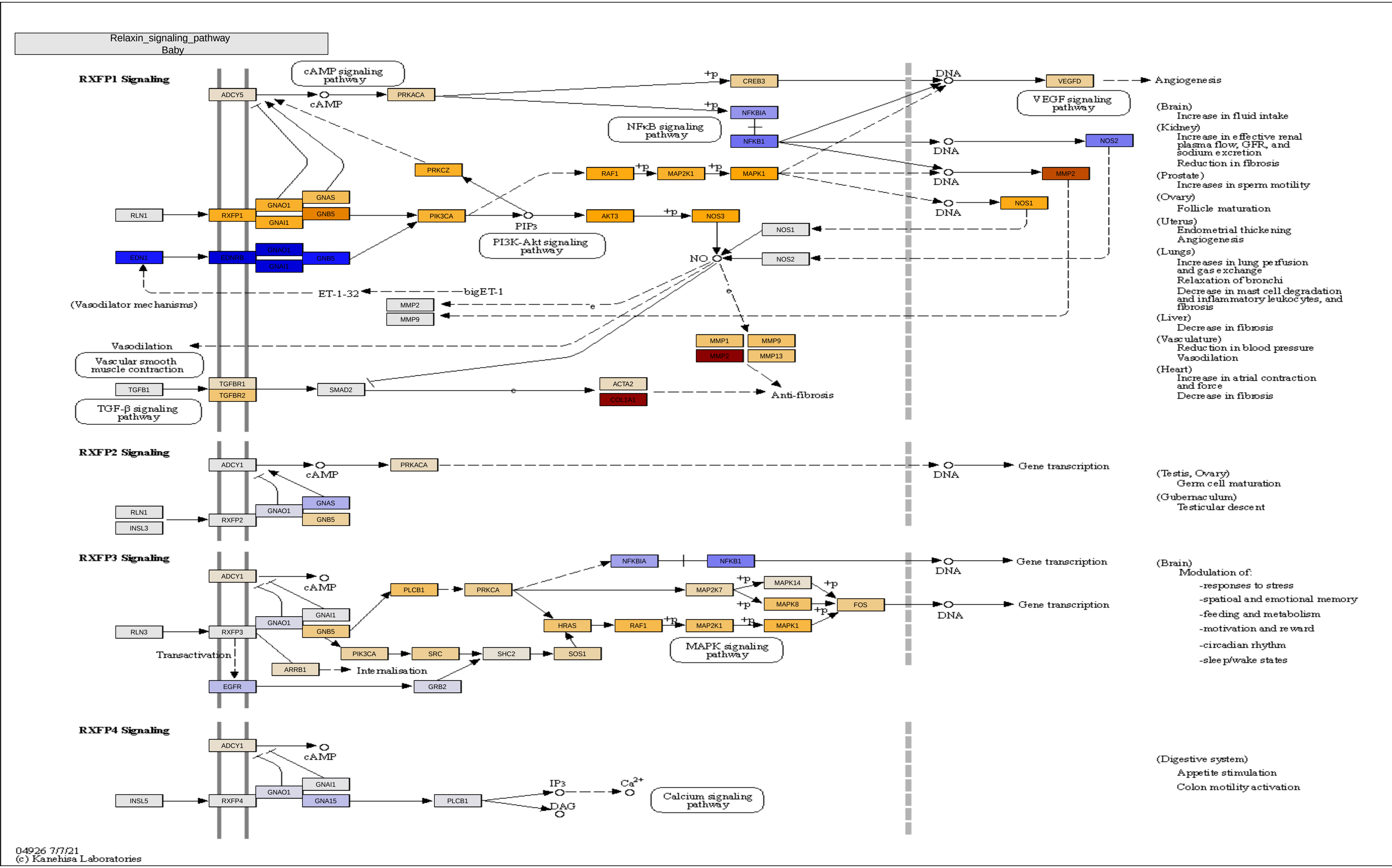

Relaxin_signaling_pathway
Baby
RXFP1 Signaling
ADCY5
cAMP
PRKACA
cAMP signaling pathway
+p
CREB3
NFKBIA
NFKB1
NFκB signaling pathway
DNA
VEGFD
VEGF signaling pathway
Angiogenesis
NOS2
MMP2
NOS1
PRKCZ
RAF1
MAP2K1
MAPK1
RLN1
RXFP1
GNAO1
GNAS
GNB5
GNAI1
PIK3CA
PIP3
AKT3
NOS3
PI3K-Akt signaling pathway
EDN1
EDNRB
NO
NOS1
NOS2
ET-1-32
bigET-1
(Vasodilator mechanisms)
MMP2
MMP9
MMP1
MMP9
MMP2
MMP13
Vasodilation
Vascular smooth muscle contraction
TGFB1
TGFBR1
TGFBR2
SMAD2
ACTA2
COL1A1
Anti-fibrosis
TGF-β signaling pathway
(Brain)
Increase in fluid intake
(Kidney)
Increase in effective renal plasma flow, GFR, and sodium excretion
Reduction in fibrosis
(Prostate)
Increases in sperm motility
(Ovary)
Follicle maturation
(Uterus)
Endometrial thickening
Angiogenesis
(Lungs)
Increases in lung perfusion and gas exchange
Relaxation of bronchi
Decrease in mast cell degradation and inflammatory leukocytes, and fibrosis
(Liver)
Decrease in fibrosis
(Vasculature)
Reduction in blood pressure
Vasodilation
(Heart)
Increase in atrial contraction and force
Decrease in fibrosis
RXFP2 Signaling
ADCY1
cAMP
PRKACA
RLN1
INSL3
RXFP2
GNAO1
GNAS
GNB5
DNA
Gene transcription
(Testis, Ovary)
Germ cell maturation
(Gubernaculum)
Testicular descent
RXFP3 Signaling
ADCY1
cAMP
PLCB1
PRKCA
NFKBIA
NFKB1
MAP2K7
MAPK14
MAPK8
FOS
HRAS
RAF1
MAP2K1
MAPK1
MAPK signaling pathway
RLN3
RXFP3
GNAO1
GNAI1
GNB5
PIK3CA
SRC
SHC2
SOS1
Transactivation
ARRB1
Internalisation
EGFR
GRB2
DNA
Gene transcription
(Brain)
Modulation of:
-responses to stress
-spatial and emotional memory
-feeding and metabolism
-motivation and reward
-circadian rhythm
-sleep/wake states
RXFP4 Signaling
ADCY1
cAMP
INSL5
RXFP4
GNAO1
GNAI1
GNA15
PLCB1
IP3
DAG
Ca2+
Calcium signaling pathway
(Digestive system)
Appetite stimulation
Colon motility activation
04926 7/7/21
(c) Kanehisa Laboratories

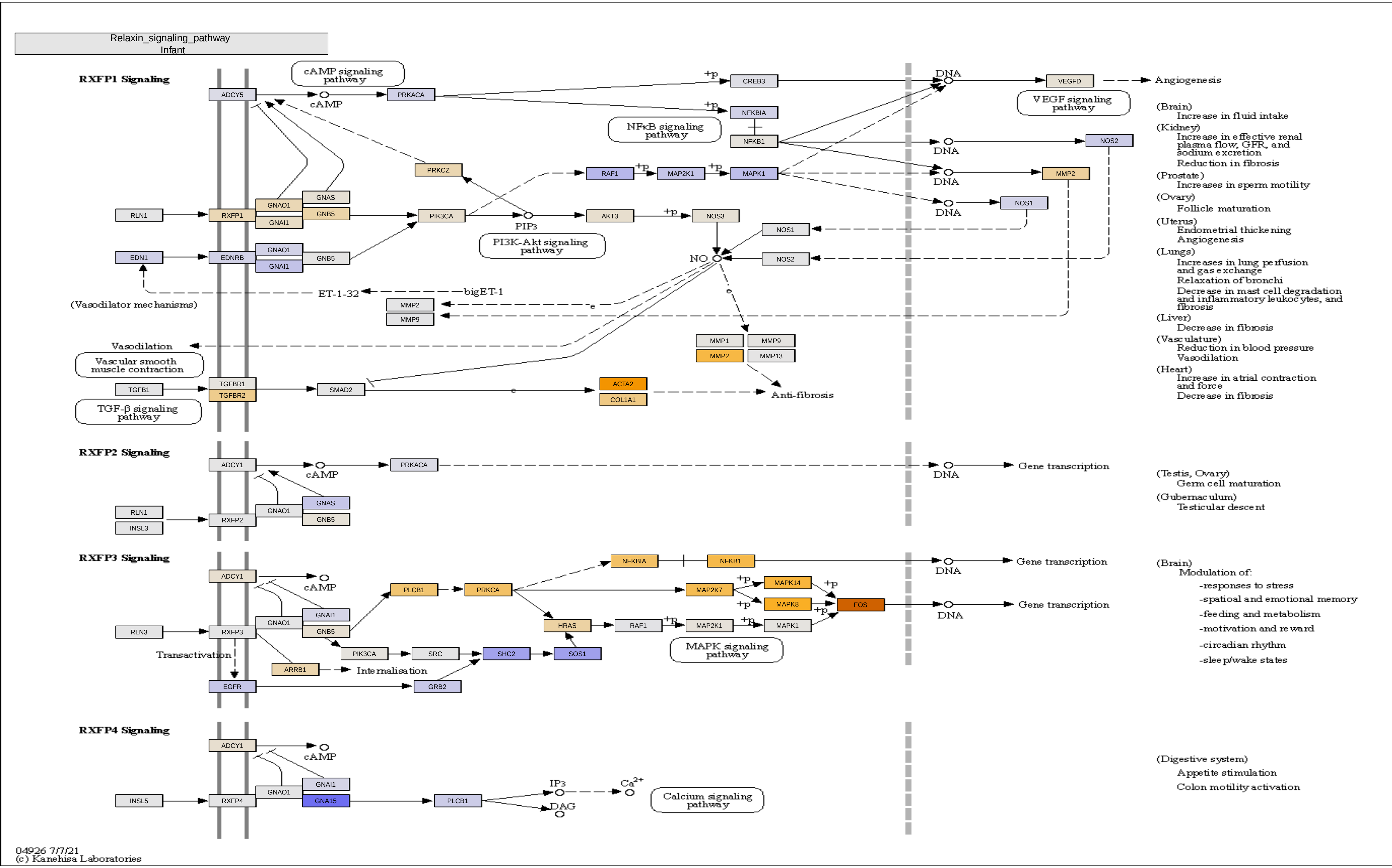
Relaxin_signaling_pathway
Infant
RXFP1 Signaling
ADCY5
cAMP
PRKACA
cAMP signaling pathway
CREB3
NFKBIA
NFKB1
NFκB signaling pathway
DNA
VEGFD
VEGF signaling pathway
Angiogenesis
NOS2
PRKCZ
RAF1
MAP2K1
MAPK1
MMP2
NOS1
RLN1
RXFP1
GNAO1
GNAI1
GNAS
GNB5
PIK3CA
PIP3
AKT3
NOS3
PI3K-Akt signaling pathway
EDN1
EDNRB
NO
ET-1-32
bigET-1
(Vasodilator mechanisms)
MMP9
MMP1
MMP13
Vasodilation
Vascular smooth muscle contraction
TGFB1
TGFBR1
TGFBR2
SMAD2
ACTA2
COL1A1
Anti-fibrosis
TGF-β signaling pathway
(Brain)
Increase in fluid intake
(Kidney)
Increase in effective renal plasma flow, GFR, and sodium excretion
Reduction in fibrosis
(Prostate)
Increases in sperm motility
(Ovary)
Follicle maturation
(Uterus)
Endometrial thickening
Angiogenesis
(Lungs)
Increases in lung perfusion and gas exchange
Relaxation of bronchi
Decrease in mast cell degradation and inflammatory leukocytes, and fibrosis
(Liver)
Decrease in fibrosis
(Vasculature)
Reduction in blood pressure
Vasodilation
(Heart)
Increase in atrial contraction and force
Decrease in fibrosis
RXFP2 Signaling
ADCY1
RXFP2
INSL3
Gene transcription
(Testis, Ovary)
Germ cell maturation
(Gubernaculum)
Testicular descent
RXFP3 Signaling
RLN3
RXFP3
PLCB1
PRKCA
MAP2K7
MAPK14
MAPK8
FOS
HRAS
SRC
SHC2
SOS1
Transactivation
ARRB1
Internalisation
EGFR
GRB2
MAPK signaling pathway
(Brain)
Modulation of:
-responses to stress
-spatial and emotional memory
-feeding and metabolism
-motivation and reward
-circadian rhythm
-sleep/wake states
RXFP4 Signaling
INSL5
RXFP4
GNA15
IP3
DAG
Ca^{2+}
Calcium signaling pathway
(Digestive system)
Appetite stimulation
Colon motility activation
04926 7/7/21
(c) Kanehisa Laboratories

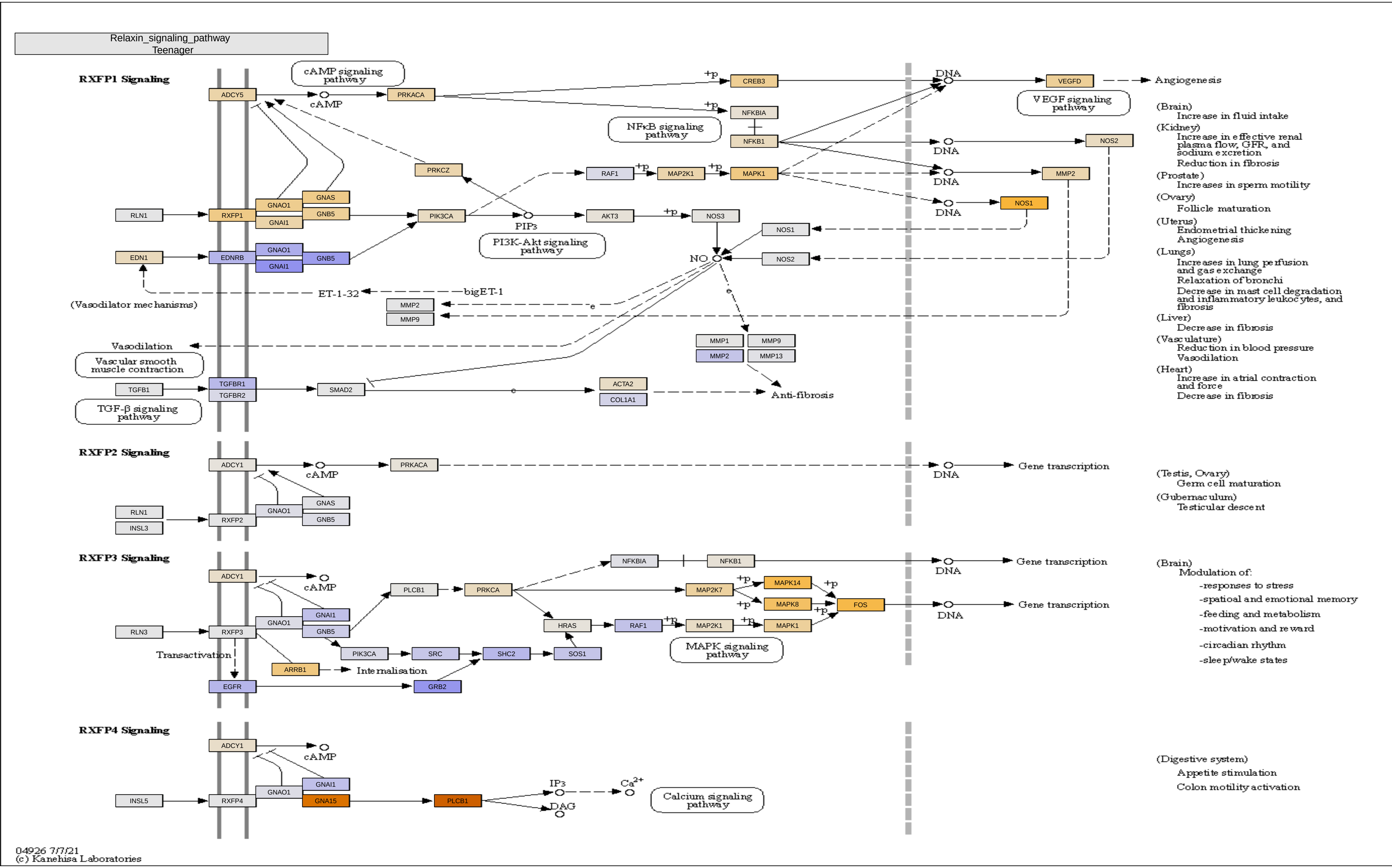
Relaxin_signaling_pathway
Teenager
RXFP1 Signaling
cAMP signaling pathway
ADCY5
cAMP
PRKACA
+p
CREB3
DNA
VEGFD
Angiogenesis
VEGF signaling pathway
NFKBIA
NFκB signaling pathway
NFKB1
NOS2
PRKCZ
RAF1
MAP2K1
MAPK1
MMP2
NOS1
GNAO1
GNAS
GNAI1
GNB5
RLN1
RXFP1
PIK3CA
PIP3
AKT3
NOS3
PI3K-Akt signaling pathway
EDN1
EDNRB
NO
ET-1-32
bigET-1
(Vasodilator mechanisms)
MMP9
MMP1
MMP13
Vasodilation
Vascular smooth muscle contraction
TGFB1
TGFBR1
TGFBR2
SMAD2
ACTA2
COL1A1
Anti-fibrosis
TGF-β signaling pathway
(Brain)
Increase in fluid intake
(Kidney)
Increase in effective renal plasma flow, GFR, and sodium excretion
Reduction in fibrosis
(Prostate)
Increases in sperm motility
(Ovary)
Follicle maturation
(Uterus)
Endometrial thickening
Angiogenesis
(Lungs)
Increases in lung perfusion and gas exchange
Relaxation of bronchi
Decrease in mast cell degradation and inflammatory leukocytes, and fibrosis
(Liver)
Decrease in fibrosis
(Vasculature)
Reduction in blood pressure
Vasodilation
(Heart)
Increase in atrial contraction and force
Decrease in fibrosis
RXFP2 Signaling
ADCY1
RLN1
INSL3
RXFP2
Gene transcription
(Testis, Ovary)
Germ cell maturation
(Gubernaculum)
Testicular descent
RXFP3 Signaling
PLCB1
PRKCA
MAP2K7
MAPK14
MAPK8
FOS
RLN3
RXFP3
HRAS
Transactivation
SRC
SHC2
SOS1
ARRB1
Internalisation
EGFR
GRB2
MAPK signaling pathway
(Brain)
Modulation of:
-responses to stress
-spatial and emotional memory
-feeding and metabolism
-motivation and reward
-circadian rhythm
-sleep/wake states
RXFP4 Signaling
INSL5
RXFP4
GNA15
IP3
DAG
Ca2+
Calcium signaling pathway
(Digestive system)
Appetite stimulation
Colon motility activation
04926 7/7/21
(c) Kanehisa Laboratories

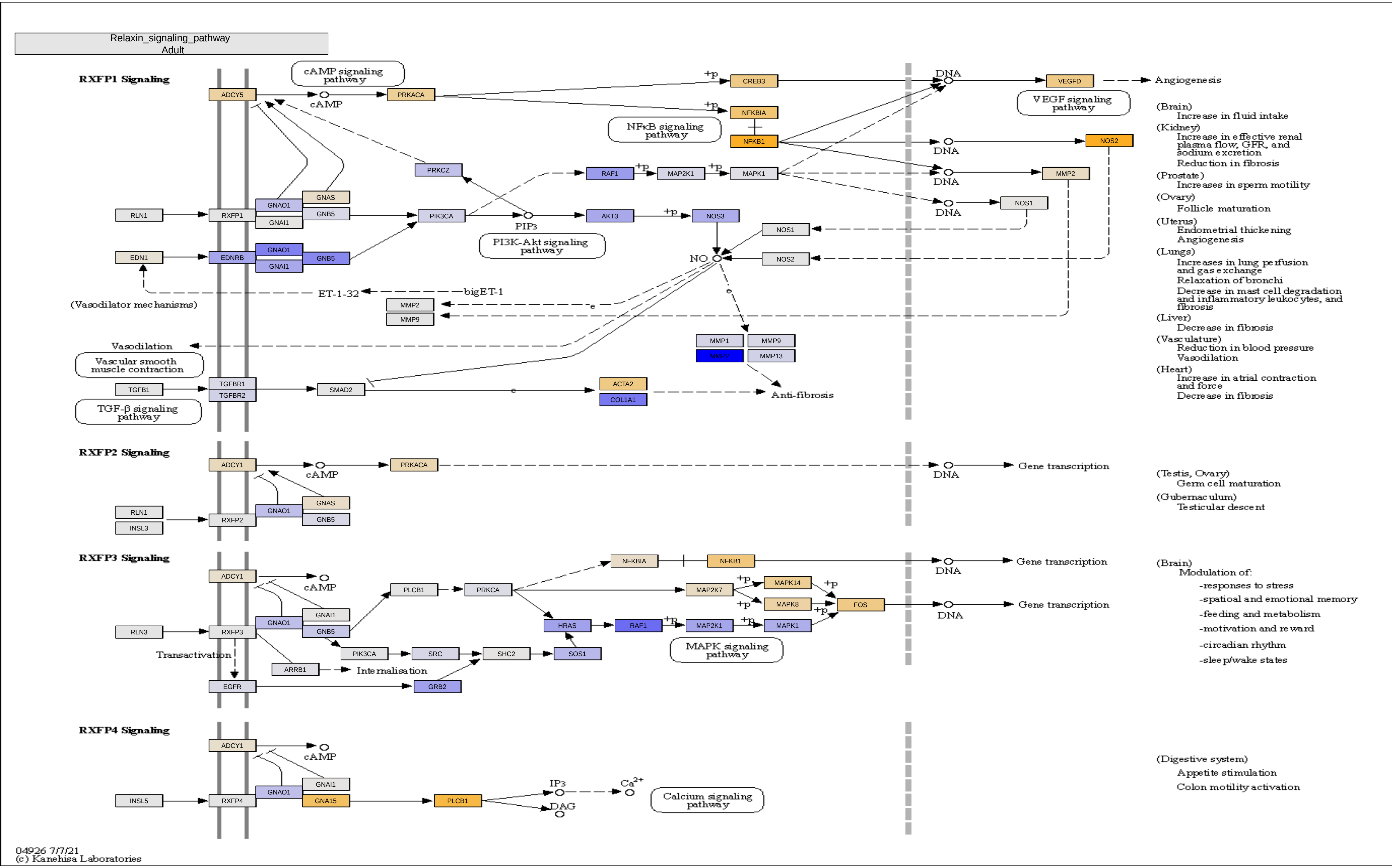

Relaxin_signaling_pathway
Adult
RXFP1 Signaling
ADCY5
cAMP
PRKACA
cAMP signaling pathway
CREB3
NFKBIA
NFKB1
NFκB signaling pathway
DNA
VEGFD
VEGF signaling pathway
Angiogenesis
NOS2
MMP2
NOS1
PRKCZ
RAF1
MAP2K1
MAPK1
GNAS
GNAO1
GNB5
GNAI1
RLN1
RXFP1
PIK3CA
PIP3
PI3K-Akt signaling pathway
AKT3
NOS3
EDN1
EDNRB
NO
ET-1-32
bigET-1
(Vasodilator mechanisms)
MMP9
MMP1
MMP13
Vasodilation
Vascular smooth muscle contraction
TGFB1
TGFBR1
TGFBR2
SMAD2
ACTA2
COL1A1
Anti-fibrosis
TGF-β signaling pathway
+p
e
(Brain)
Increase in fluid intake
(Kidney)
Increase in effective renal plasma flow, GFR, and sodium excretion
Reduction in fibrosis
(Prostate)
Increases in sperm motility
(Ovary)
Follicle maturation
(Uterus)
Endometrial thickening
Angiogenesis
(Lungs)
Increases in lung perfusion and gas exchange
Relaxation of bronchi
Decrease in mast cell degradation and inflammatory leukocytes, and fibrosis
(Liver)
Decrease in fibrosis
(Vasculature)
Reduction in blood pressure
Vasodilation
(Heart)
Increase in atrial contraction and force
Decrease in fibrosis
RXFP2 Signaling
ADCY1
RXFP2
INSL3
Gene transcription
(Testis, Ovary)
Germ cell maturation
(Gubernaculum)
Testicular descent
RXFP3 Signaling
RLN3
RXFP3
PLCB1
PRKCA
MAP2K7
MAPK14
MAPK8
FOS
HRAS
SRC
SHC2
SOS1
ARRB1
Internalisation
EGFR
GRB2
Transactivation
MAPK signaling pathway
(Brain)
Modulation of:
-responses to stress
-spatial and emotional memory
-feeding and metabolism
-motivation and reward
-circadian rhythm
-sleep/wake states
RXFP4 Signaling
INSL5
RXFP4
GNA15
IP3
DAG
Ca^{2+}
Calcium signaling pathway
(Digestive system)
Appetite stimulation
Colon motility activation
04926 7/7/21
(c) Kanehisa Laboratories

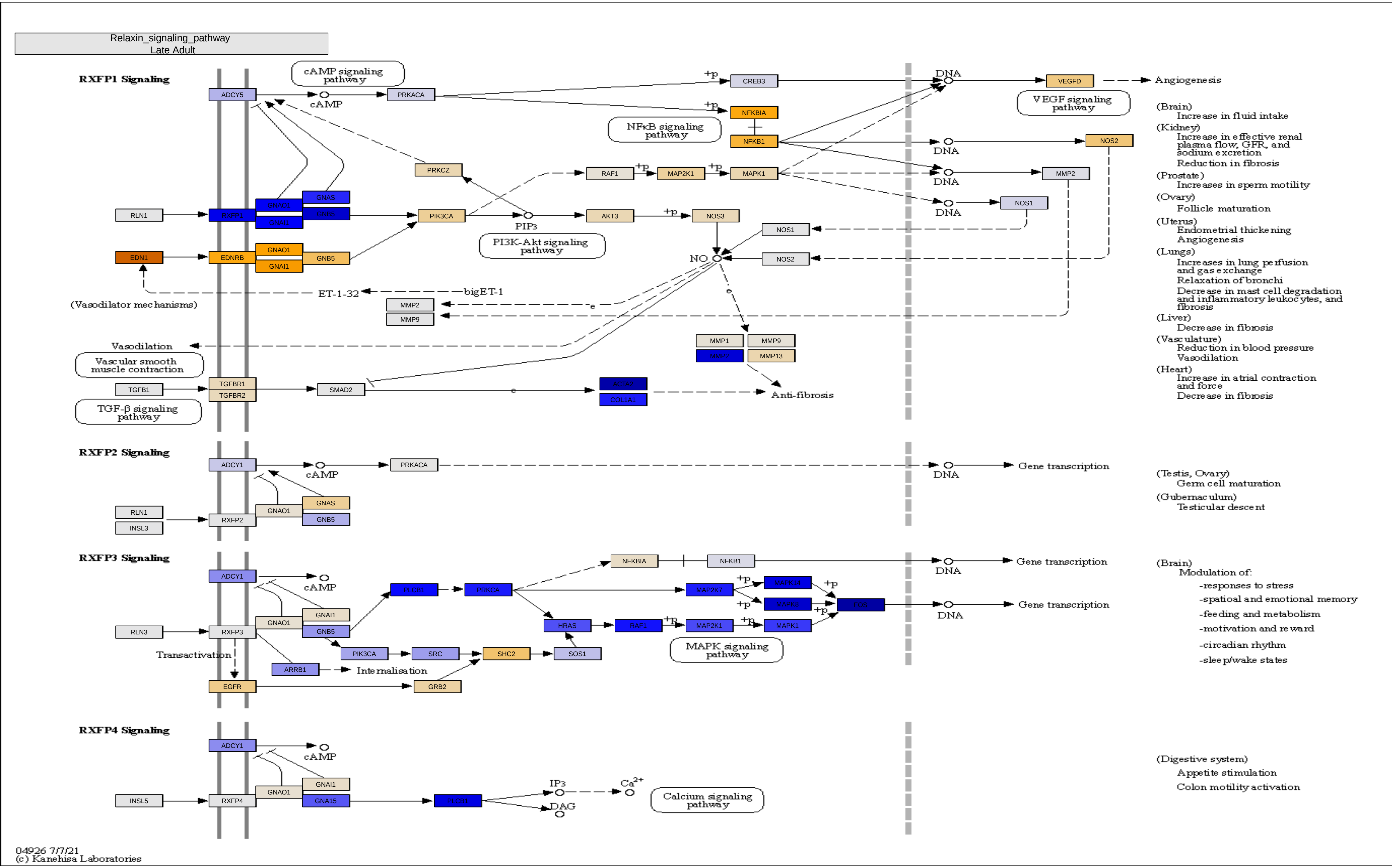
Relaxin_signaling_pathway
Late Adult
RXFP1 Signaling
cAMP signaling pathway
ADCY5
cAMP
PRKACA
CREB3
NFKBIA
NFKB1
NFκB signaling pathway
DNA
VEGFD
VEGF signaling pathway
Angiogenesis
NOS2
MMP2
NOS1
PRKCZ
RAF1
MAP2K1
MAPK1
+p
GNAS
GNAO1
GNB5
GNAI1
RLN1
PIK3CA
PIP3
PI3K-Akt signaling pathway
AKT3
NOS3
NO
EDN1
EDNRB
ET-1-32
bigET-1
(Vasodilator mechanisms)
MMP9
MMP1
MMP13
Vasodilation
Vascular smooth muscle contraction
TGFB1
TGFBR1
TGFBR2
SMAD2
COL1A1
Anti-fibrosis
TGF-β signaling pathway
(Brain)
Increase in fluid intake
(Kidney)
Increase in effective renal plasma flow, GFR, and sodium excretion
Reduction in fibrosis
(Prostate)
Increases in sperm motility
(Ovary)
Follicle maturation
(Uterus)
Endometrial thickening
Angiogenesis
(Lungs)
Increases in lung perfusion and gas exchange
Relaxation of bronchi
Decrease in mast cell degradation and inflammatory leukocytes, and fibrosis
(Liver)
Decrease in fibrosis
(Vasculature)
Reduction in blood pressure
Vasodilation
(Heart)
Increase in atrial contraction and force
Decrease in fibrosis
RXFP2 Signaling
ADCY1
RXFP2
INSL3
Gene transcription
(Testis, Ovary)
Germ cell maturation
(Gubernaculum)
Testicular descent
RXFP3 Signaling
RLN3
RXFP3
PLCB1
PRKCA
MAP2K7
HRAS
SRC
SHC2
SOS1
ARRB1
Internalisation
Transactivation
EGFR
GRB2
MAPK signaling pathway
(Brain)
Modulation of:
-responses to stress
-spatial and emotional memory
-feeding and metabolism
-motivation and reward
-circadian rhythm
-sleep/wake states
RXFP4 Signaling
INSL5
RXFP4
GNA15
IP3
DAG
Ca2+
Calcium signaling pathway
(Digestive system)
Appetite stimulation
Colon motility activation
04926 7/7/21
(c) Kanehisa Laboratories
