# Supplementary material for: The Transcriptome and Methylome of the Developing and Aging Brain and Their Relations to Gliomas and Psychological Disorders
Source: Cells. 2022 Jan 21;11(3):362. doi: 10.3390/cells11030362 (PMC8834030; doi:10.3390/cells11030362)
Supplement: Supplementary file 1 [file cells-11-00362-s001.zip › Suppl-Material-S3-Pathways-PSF_Expression/RIG_I_like_receptor_signaling_pathway.pdf]

RIG−I−like receptor signaling pathwa

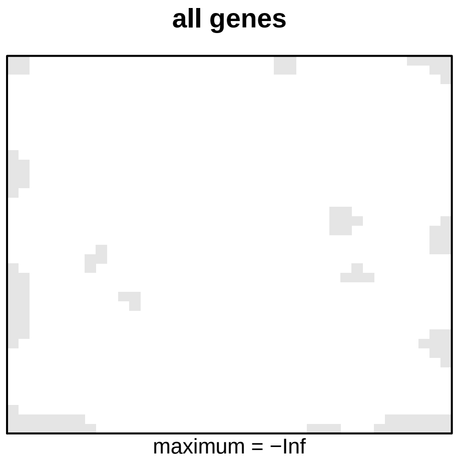

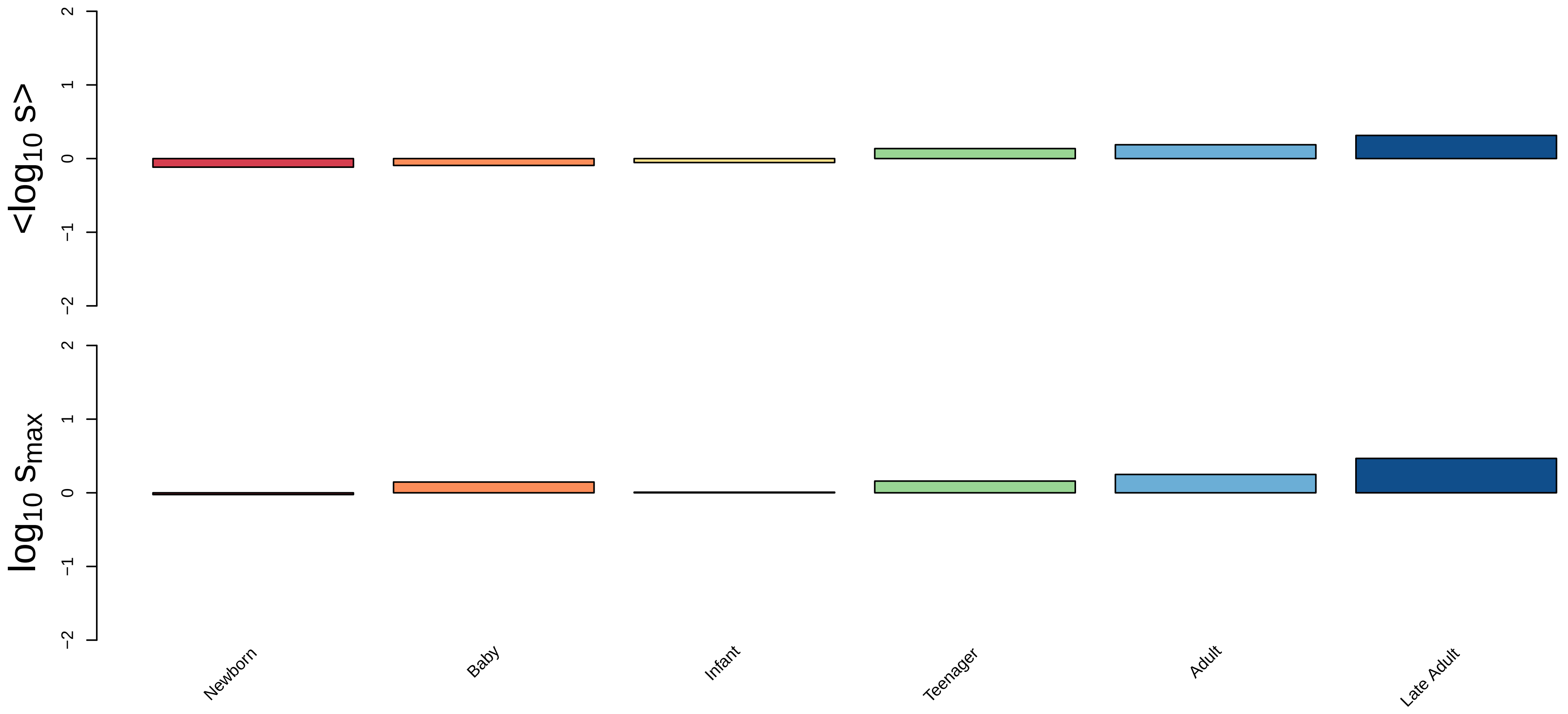

RIG_I_like_receptor_signaling_pathway
genes with data

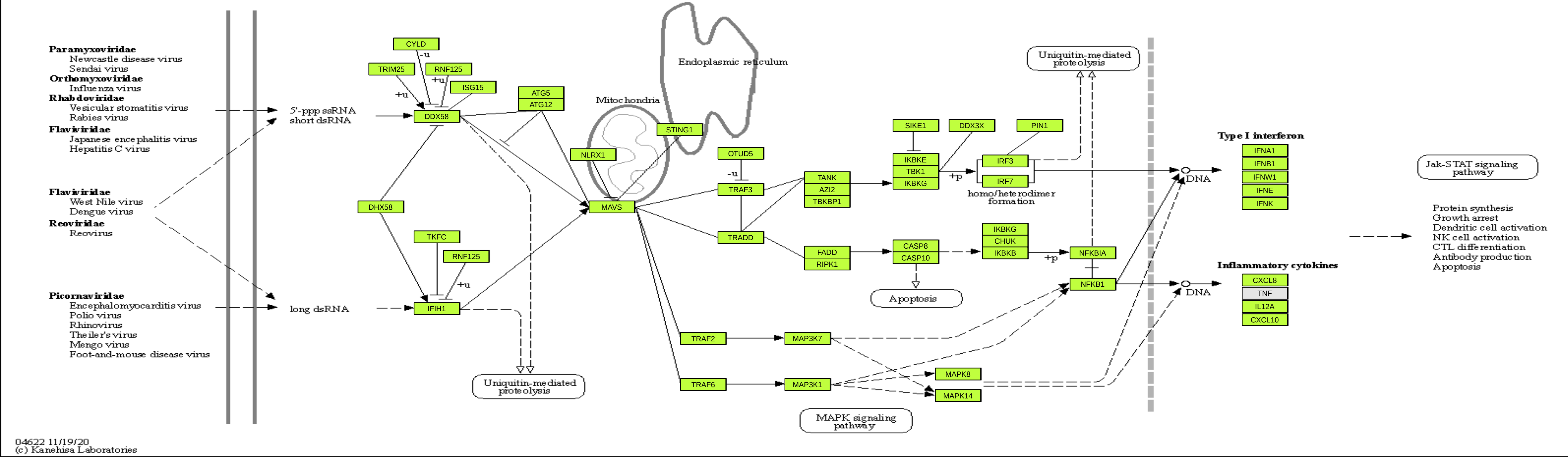

RIG_I_like_receptor_signaling_pathway
sink nodes

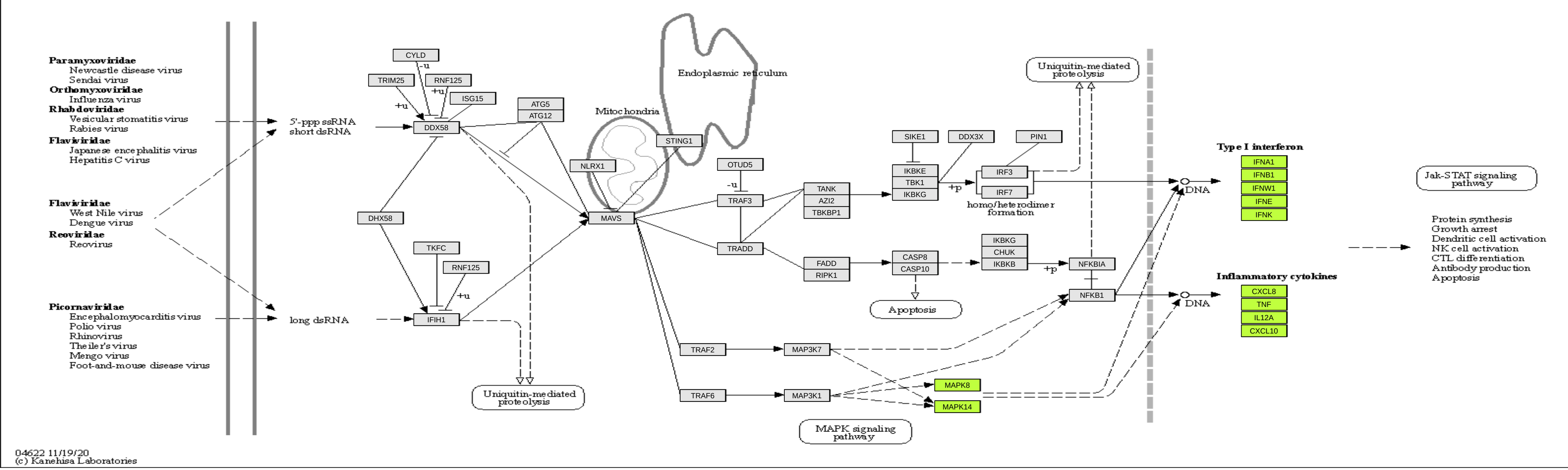

RIG_I_like_receptor_signaling_pathway
Newborn

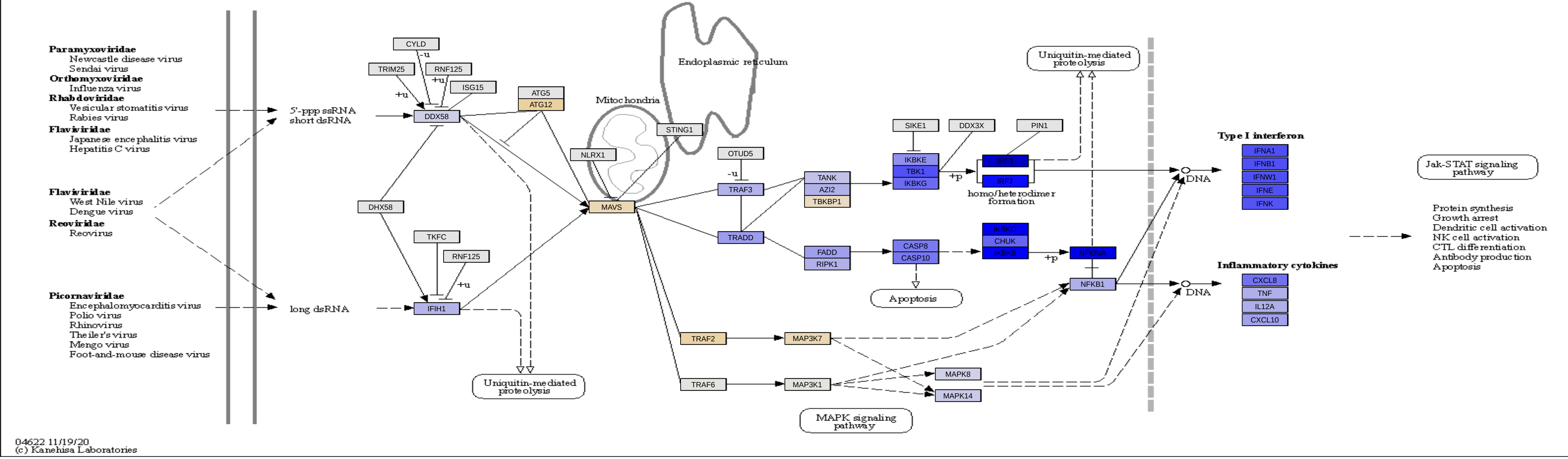

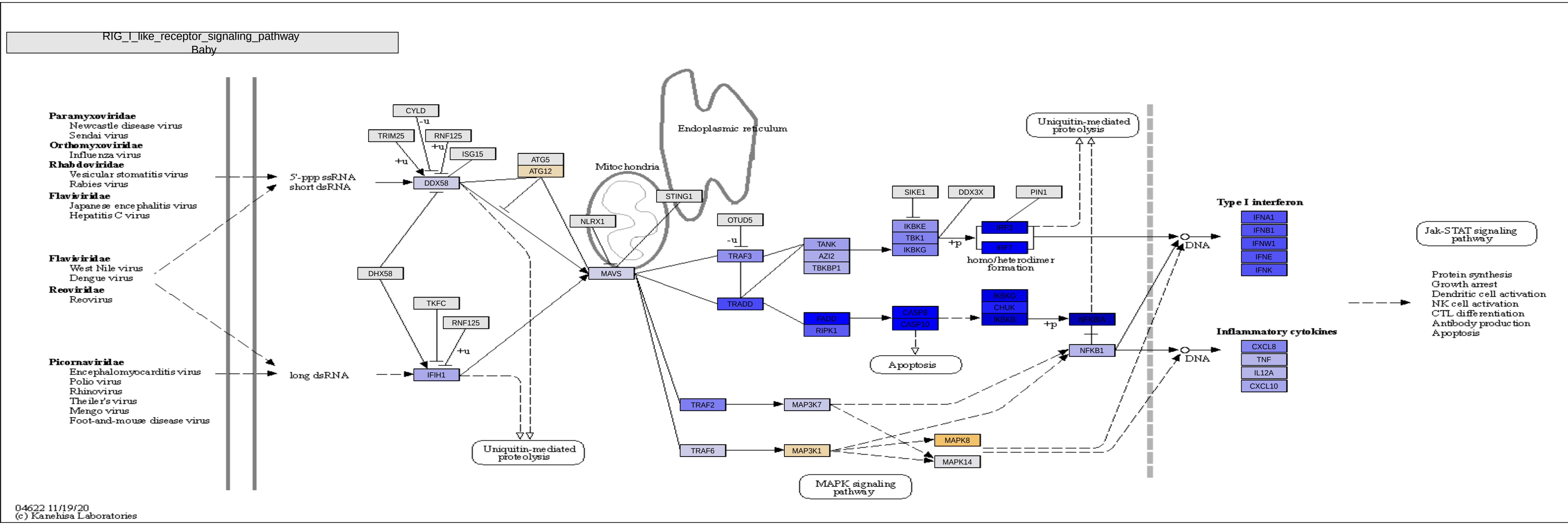
RIG_I_like_receptor_signaling_pathway
Baby
Paramyxoviridae
Newcastle disease virus
Sendai virus
Orthomyxoviridae
Influenza virus
Rhabdoviridae
Vesicular stomatitis virus
Rabies virus
Flaviviridae
Japanese encephalitis virus
Hepatitis C virus
Flaviviridae
West Nile virus
Dengue virus
Reoviridae
Reovirus
Picornaviridae
Encephalomyocarditis virus
Polio virus
Rhinovirus
Theiler's virus
Mengo virus
Foot-and-mouse disease virus
5'-ppp ssRNA
short dsRNA
long dsRNA
CYLD
-u
TRIM25
RNF125
+u
ISG15
+u
DDX58
ATG5
ATG12
DHX58
TKFC
RNF125
+u
IFIH1
Uniquitin-mediated
proteolysis
Mitochondria
Endoplasmic reticulum
NLRX1
STING1
MAVS
OTUD5
-u
TRAF3
TRADD
TANK
AZI2
TBKBP1
SIKE1
DDX3X
PIN1
IKBKE
TBK1
IKBKG
+p
IRF3
IRF7
homo/heterodimer
formation
Uniquitin-mediated
proteolysis
FADD
RIPK1
CASP8
CASP10
Apoptosis
IKBKG
CHUK
IKBKB
+p
NFKBIA
NFKB1
TRAF2
MAP3K7
TRAF6
MAP3K1
MAPK8
MAPK14
MAPK signaling
pathway
DNA
DNA
Type I interferon
IFNA1
IFNB1
IFNW1
IFNE
IFNK
Inflammatory cytokines
CXCL8
TNF
IL12A
CXCL10
Jak-STAT signaling
pathway
Protein synthesis
Growth arrest
Dendritic cell activation
NK cell activation
CTL differentiation
Antibody production
Apoptosis
04622 11/19/20
(c) Kanehisa Laboratories

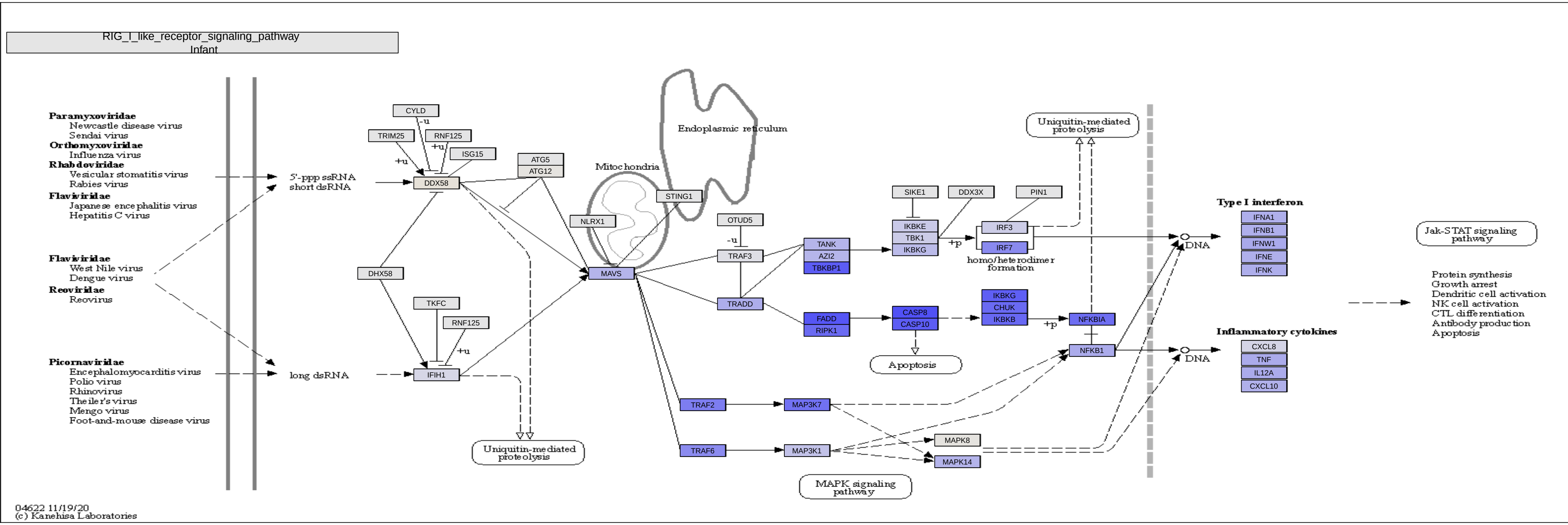
RIG_I_like_receptor_signaling_pathway
Infant
Paramyxoviridae
Newcastle disease virus
Sendai virus
Orthomyxoviridae
Influenza virus
Rhabdoviridae
Vesicular stomatitis virus
Rabies virus
Flaviviridae
Japanese encephalitis virus
Hepatitis C virus
Flaviviridae
West Nile virus
Dengue virus
Reoviridae
Reovirus
Picornaviridae
Encephalomyocarditis virus
Polio virus
Rhinovirus
Theiler's virus
Mengo virus
Foot-and-mouse disease virus
5'-ppp ssRNA
short dsRNA
long dsRNA
CYLD
TRIM25
RNF125
ISG15
ATG5
ATG12
DDX58
DHX58
TKFC
RNF125
IFIH1
Mitochondria
Endoplasmic reticulum
NLRX1
STING1
MAVS
OTUD5
TRAF3
TRADD
TANK
AZI2
TBKBP1
SIKE1
DDX3X
PIN1
IKBKE
TBK1
IKBKG
IRF3
IRF7
homo/heterodimer formation
Uniquitin-mediated proteolysis
FADD
RIPK1
CASP8
CASP10
Apoptosis
IKBKG
CHUK
IKBKB
NFKBIA
NFKB1
TRAF2
MAP3K7
TRAF6
MAP3K1
MAPK8
MAPK14
MAPK signaling pathway
Uniquitin-mediated proteolysis
DNA
DNA
Type I interferon
IFNA1
IFNB1
IFNW1
IFNE
IFNK
Inflammatory cytokines
CXCL8
TNF
IL12A
CXCL10
Jak-STAT signaling pathway
Protein synthesis
Growth arrest
Dendritic cell activation
NK cell activation
CTL differentiation
Antibody production
Apoptosis
04622 11/19/20
(c) Kanehisa Laboratories

RIG_I_like_receptor_signaling_pathway
Teenager

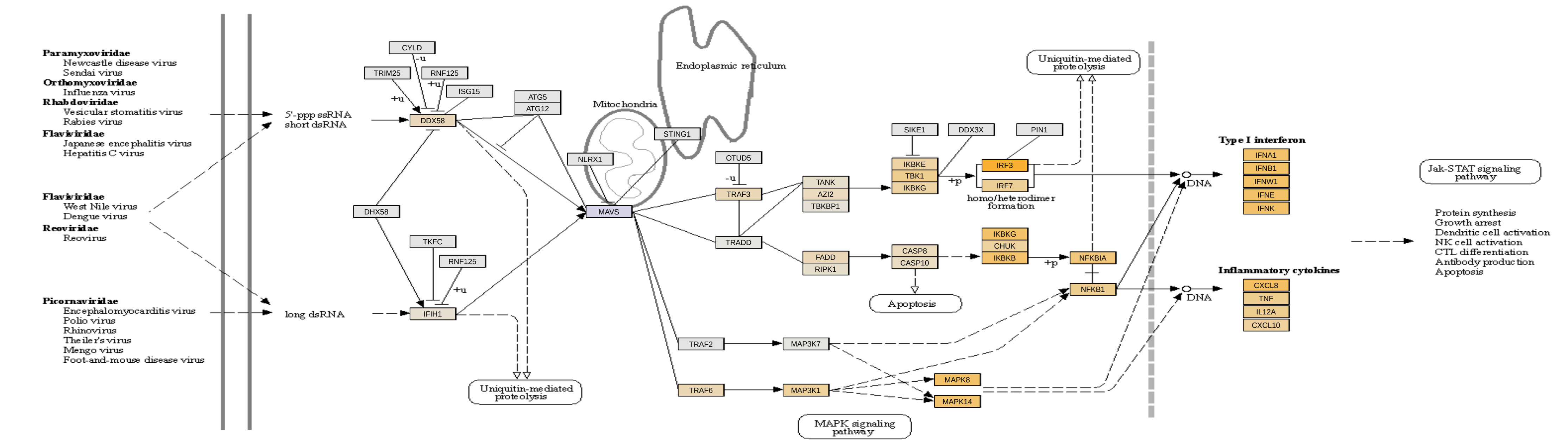

04622 11/19/20
(c) Kanehisa Laboratories

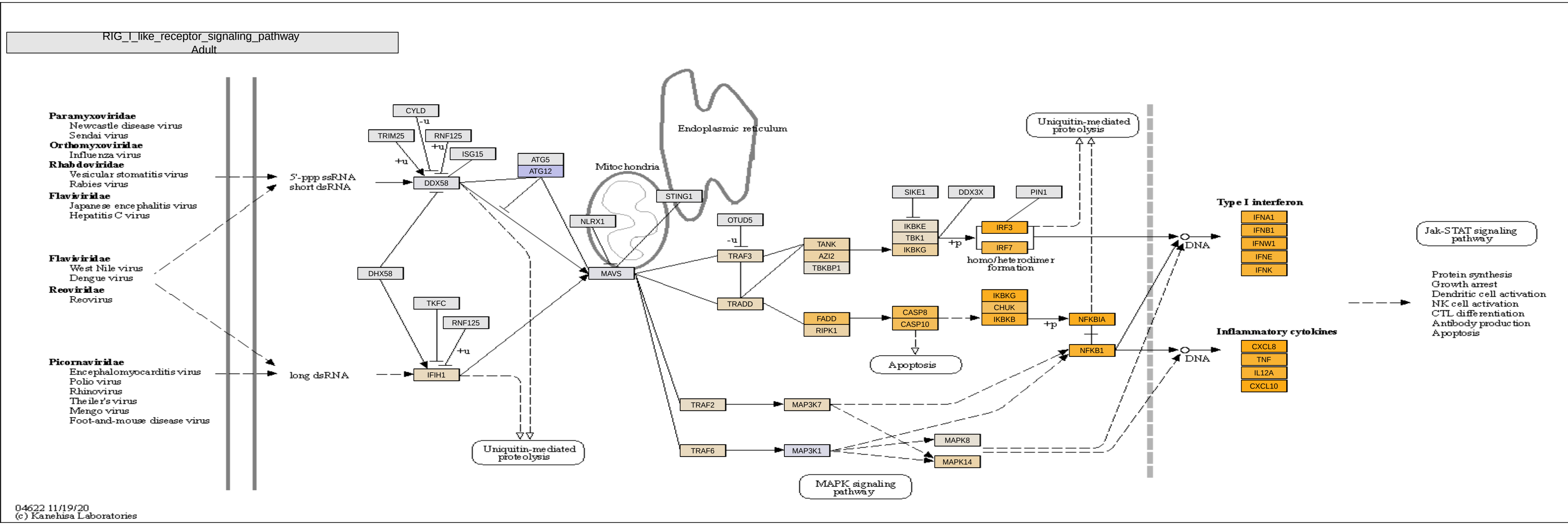

RIG_I_like_receptor_signaling_pathway
Adult
Paramyxoviridae
Newcastle disease virus
Sendai virus
Orthomyxoviridae
Influenza virus
Rhabdoviridae
Vesicular stomatitis virus
Rabies virus
Flaviviridae
Japanese encephalitis virus
Hepatitis C virus
Flaviviridae
West Nile virus
Dengue virus
Reoviridae
Reovirus
Picornaviridae
Encephalomyocarditis virus
Polio virus
Rhinovirus
Theiler's virus
Mengo virus
Foot-and-mouse disease virus
5'-ppp ssRNA
short dsRNA
long dsRNA
CYLD
TRIM25
RNF125
ISG15
ATG5
ATG12
DDX58
DHX58
TKFC
RNF125
IFIH1
Mitochondria
Endoplasmic reticulum
NLRX1
STING1
MAVS
OTUD5
TRAF3
TRADD
TANK
AZI2
TBKBP1
SIKE1
DDX3X
PIN1
IKBKE
TBK1
IKBKG
IRF3
IRF7
homo/heterodimer formation
Uniquitin-mediated proteolysis
FADD
RIPK1
CASP8
CASP10
Apoptosis
IKBKG
CHUK
IKBKB
NFKBIA
NFKB1
TRAF2
TRAF6
MAP3K7
MAP3K1
MAPK8
MAPK14
MAPK signaling pathway
Uniquitin-mediated proteolysis
DNA
DNA
Type I interferon
IFNA1
IFNB1
IFNW1
IFNE
IFNK
Inflammatory cytokines
CXCL8
TNF
IL12A
CXCL10
Jak-STAT signaling pathway
Protein synthesis
Growth arrest
Dendritic cell activation
NK cell activation
CTL differentiation
Antibody production
Apoptosis
04622 11/19/20
(c) Kanehisa Laboratories

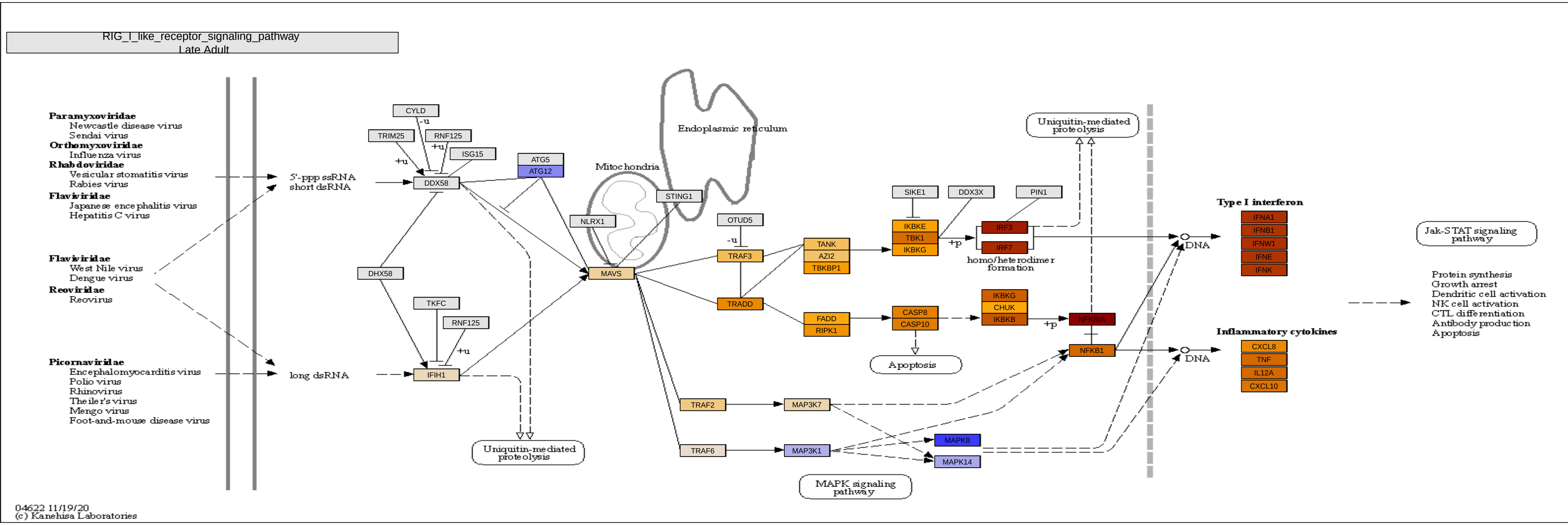

RIG_I_like_receptor_signaling_pathway
Late Adult
Paramyxoviridae
Newcastle disease virus
Sendai virus
Orthomyxoviridae
Influenza virus
Rhabdoviridae
Vesicular stomatitis virus
Rabies virus
Flaviviridae
Japanese encephalitis virus
Hepatitis C virus
Flaviviridae
West Nile virus
Dengue virus
Reoviridae
Reovirus
Picornaviridae
Encephalomyocarditis virus
Polio virus
Rhinovirus
Theiler's virus
Mengo virus
Foot-and-mouse disease virus
5'-ppp ssRNA
short dsRNA
long dsRNA
CYLD
-u
TRIM25
RNF125
+u
+u
ISG15
DDX58
ATG5
ATG12
DHX58
TKFC
RNF125
+u
IFIH1
Uniquitin-mediated proteolysis
Mitochondria
Endoplasmic reticulum
NLRX1
STING1
MAVS
OTUD5
-u
TRAF3
TRADD
TANK
AZI2
TBKBP1
FADD
RIPK1
TRAF2
MAP3K7
TRAF6
MAP3K1
MAPK8
MAPK14
MAPK signaling pathway
SIKE1
DDX3X
PIN1
IKBKE
TBK1
IKBKG
+p
IRF3
IRF7
homo/heterodimer formation
Uniquitin-mediated proteolysis
CASP8
CASP10
Apoptosis
IKBKG
CHUK
IKBKB
+p
NFKBIA
NFKB1
DNA
DNA
Type I interferon
IFNA1
IFNB1
IFNW1
IFNE
IFNK
Inflammatory cytokines
CXCL8
TNF
IL12A
CXCL10
Jak-STAT signaling pathway
Protein synthesis
Growth arrest
Dendritic cell activation
NK cell activation
CTL differentiation
Antibody production
Apoptosis
04622 11/19/20
(c) Kanehisa Laboratories
