# Supplementary material for: The Transcriptome and Methylome of the Developing and Aging Brain and Their Relations to Gliomas and Psychological Disorders
Source: Cells. 2022 Jan 21;11(3):362. doi: 10.3390/cells11030362 (PMC8834030; doi:10.3390/cells11030362)
Supplement: Supplementary file 1 [file cells-11-00362-s001.zip › Suppl-Material-S3-Pathways-PSF_Expression/TNF_signaling_pathway.pdf]

TNF signaling pathway

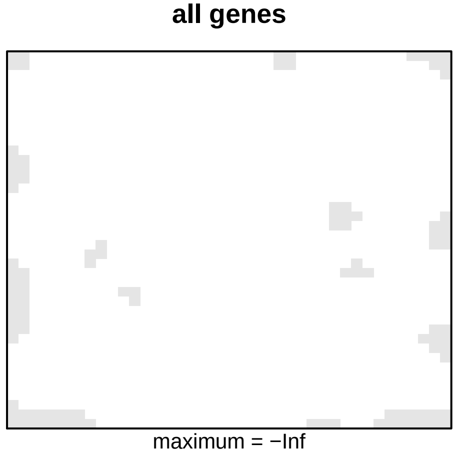

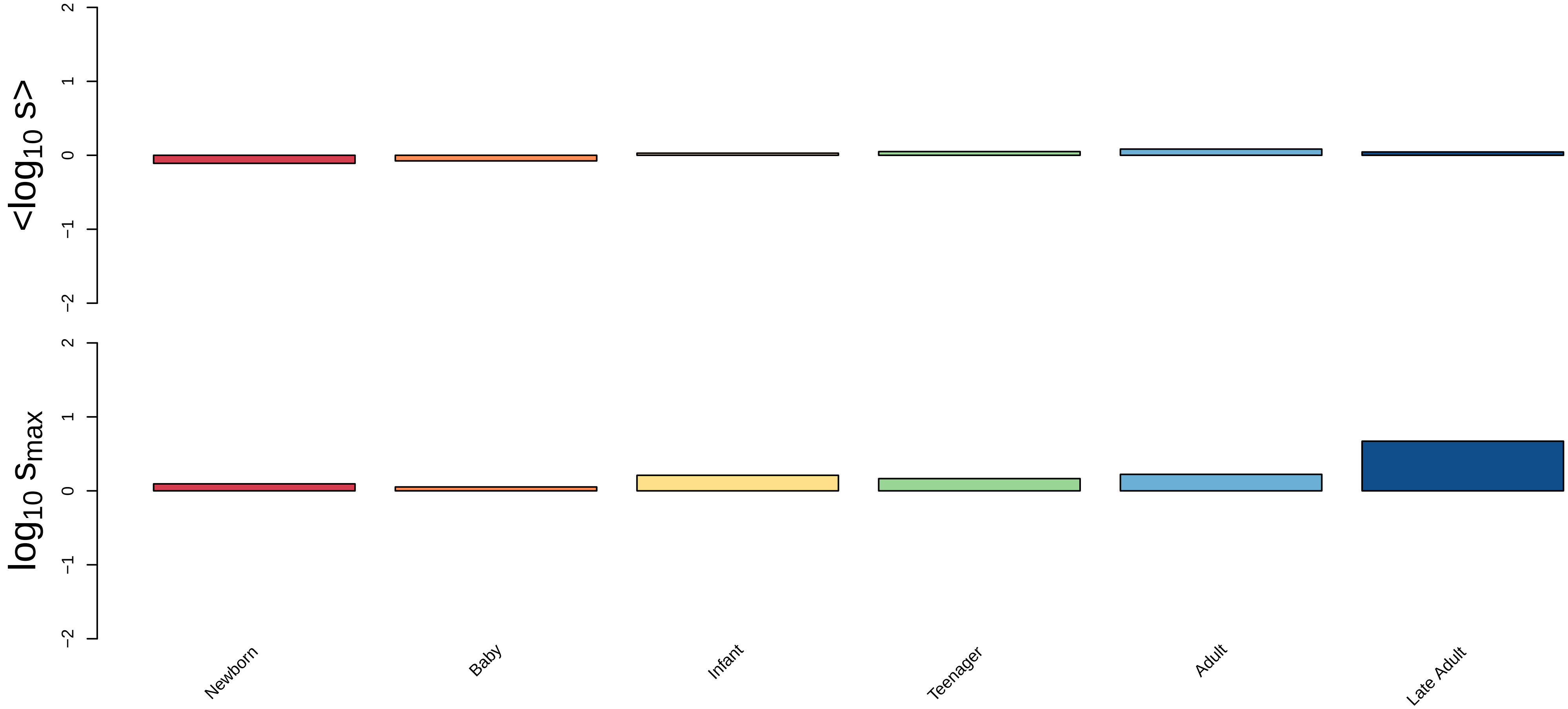

TNF_signaling_pathway
genes with data

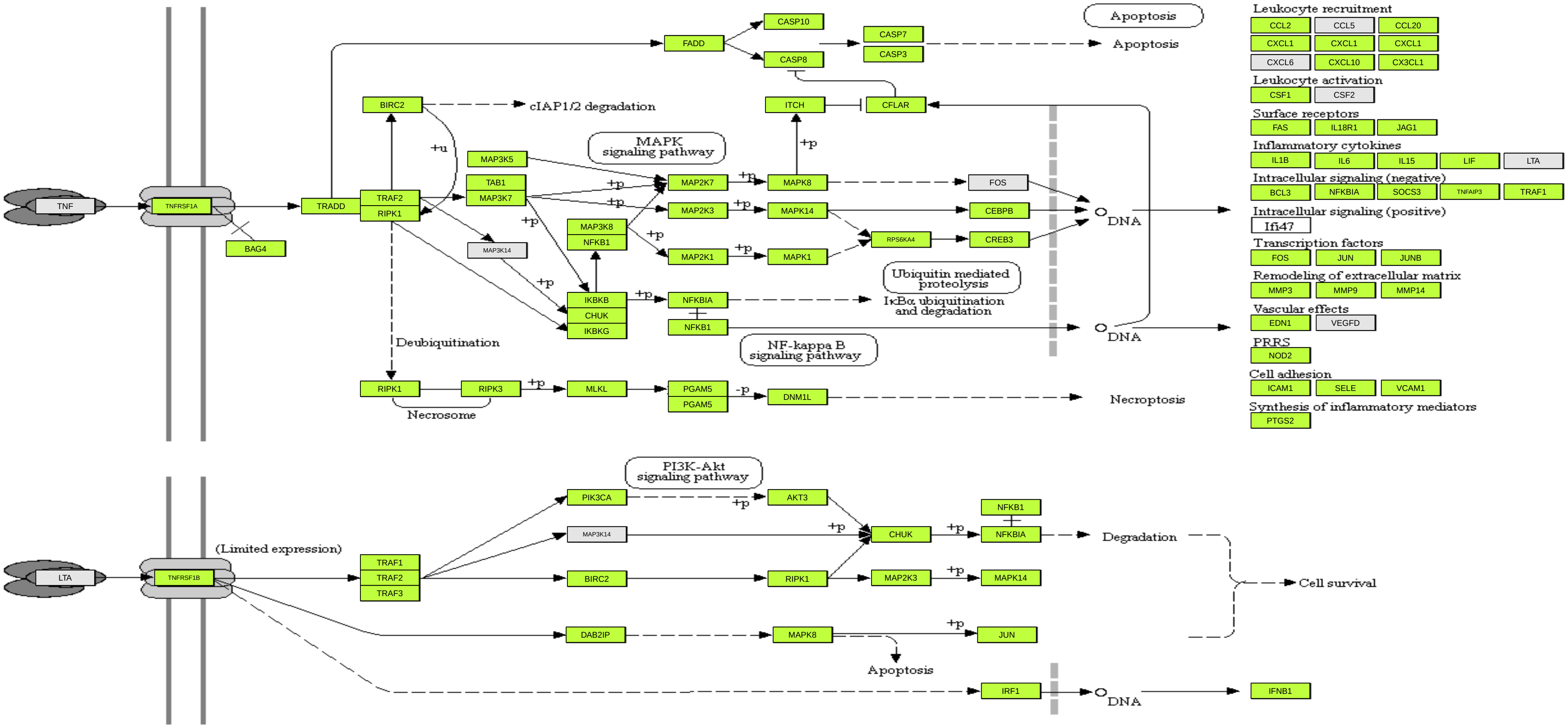

04668 7/5/19
(c) Kanehisa Laboratories

TNF_signaling_pathway
sink nodes

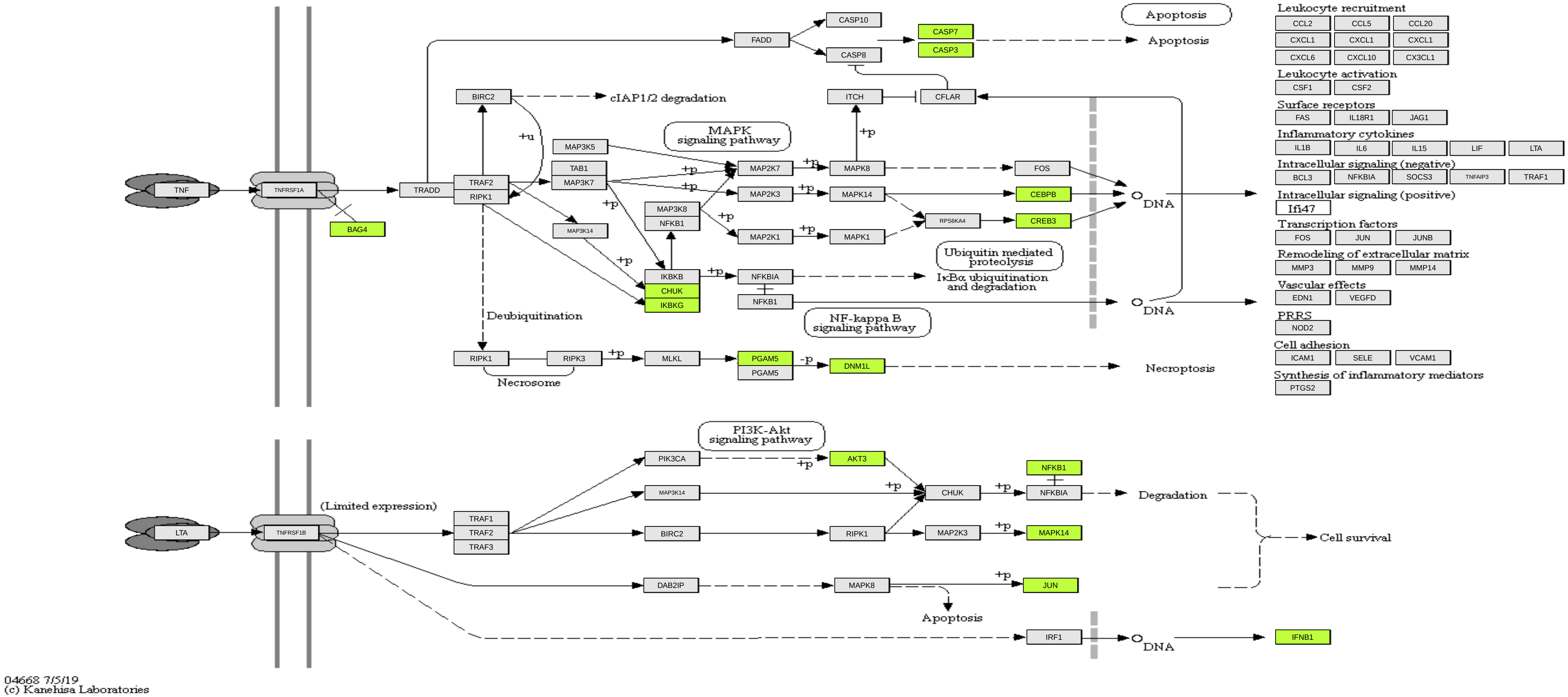

TNF_signaling_pathway
Newborn

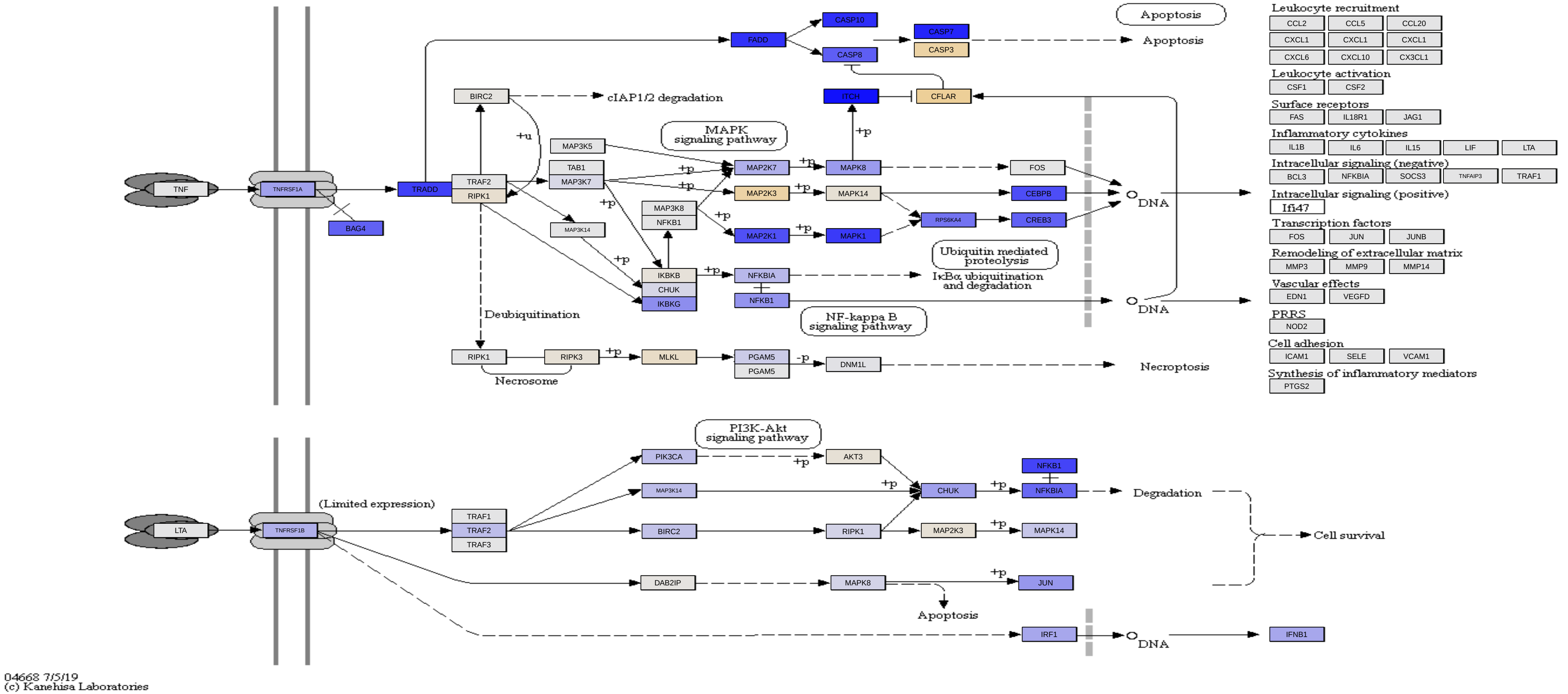

TNF_signaling_pathway
Baby

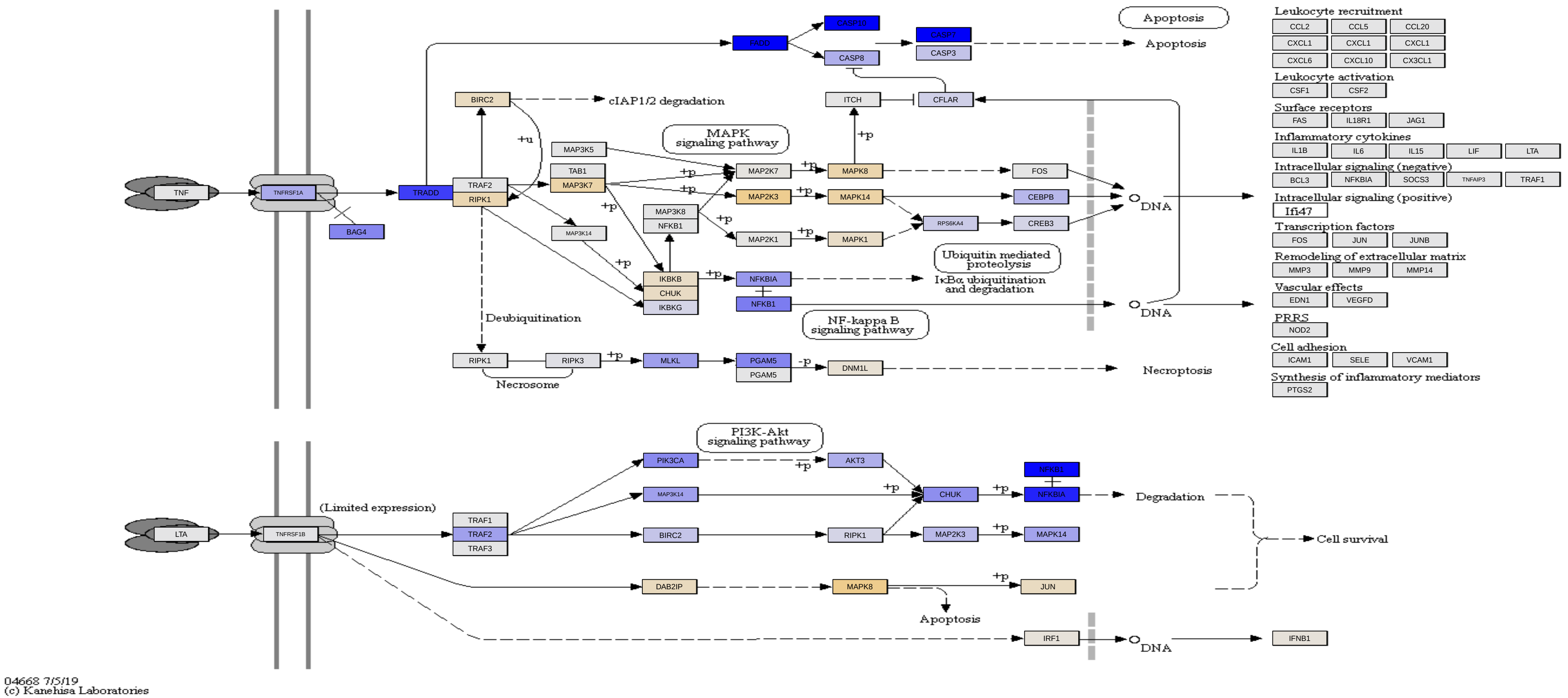

TNF_signaling_pathway
Infant

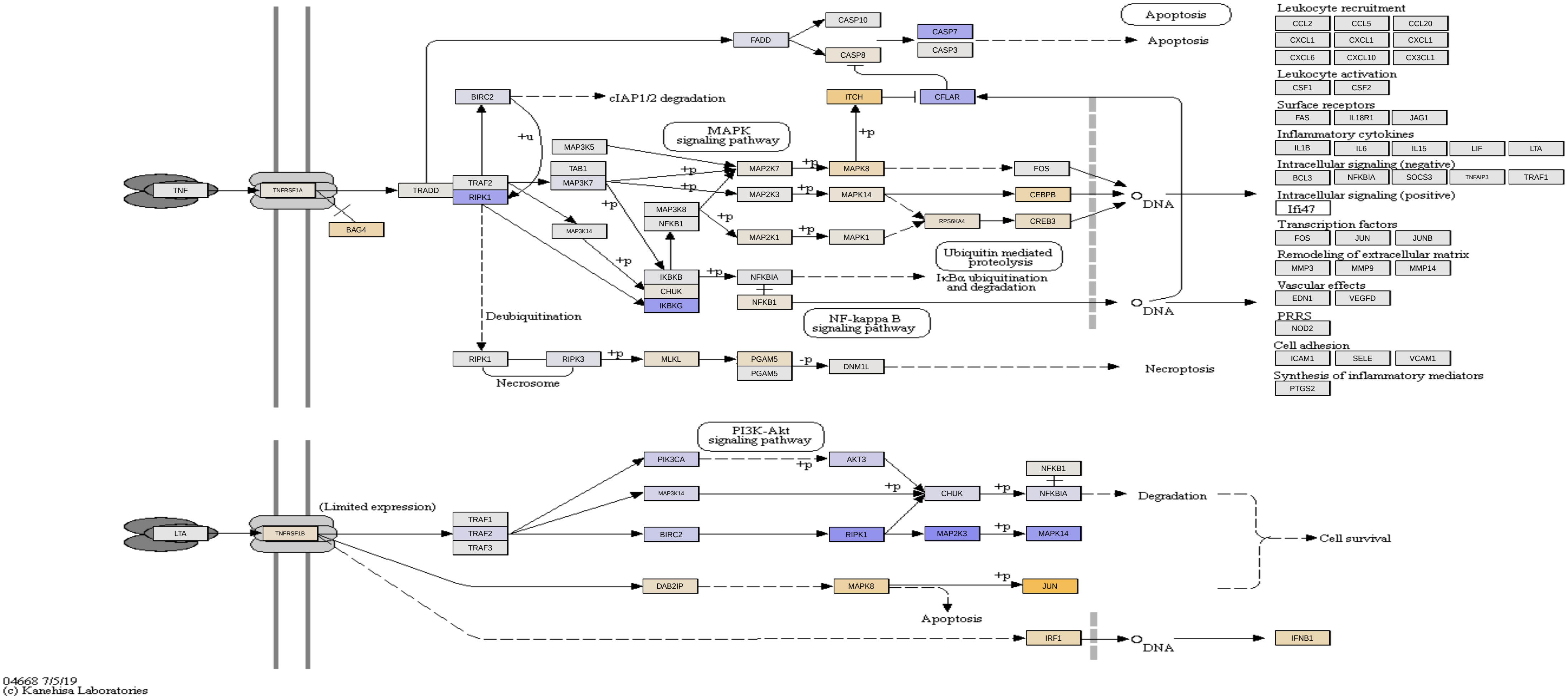

TNF_signaling_pathway
Teenager

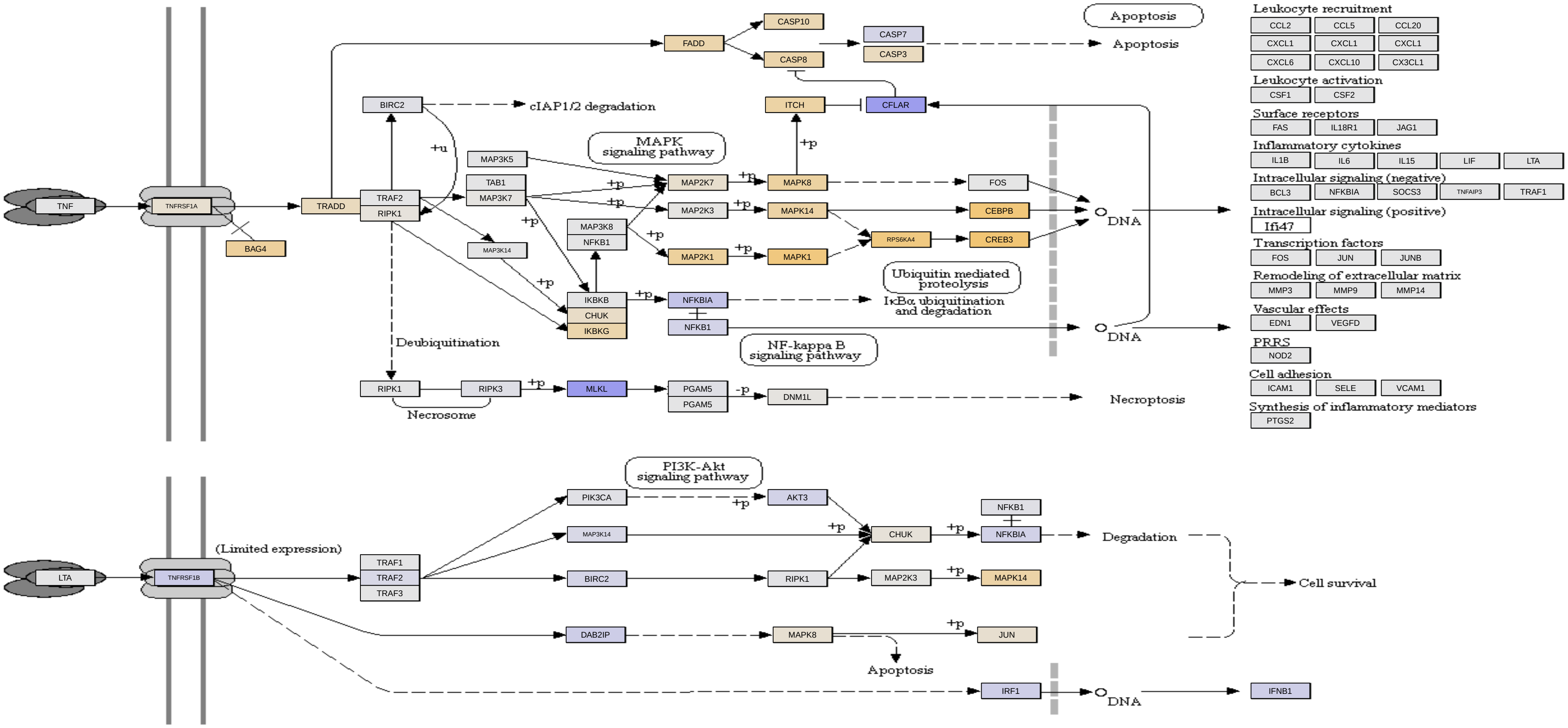

04668 7/5/19
(c) Kanehisa Laboratories

TNF_signaling_pathway
Adult

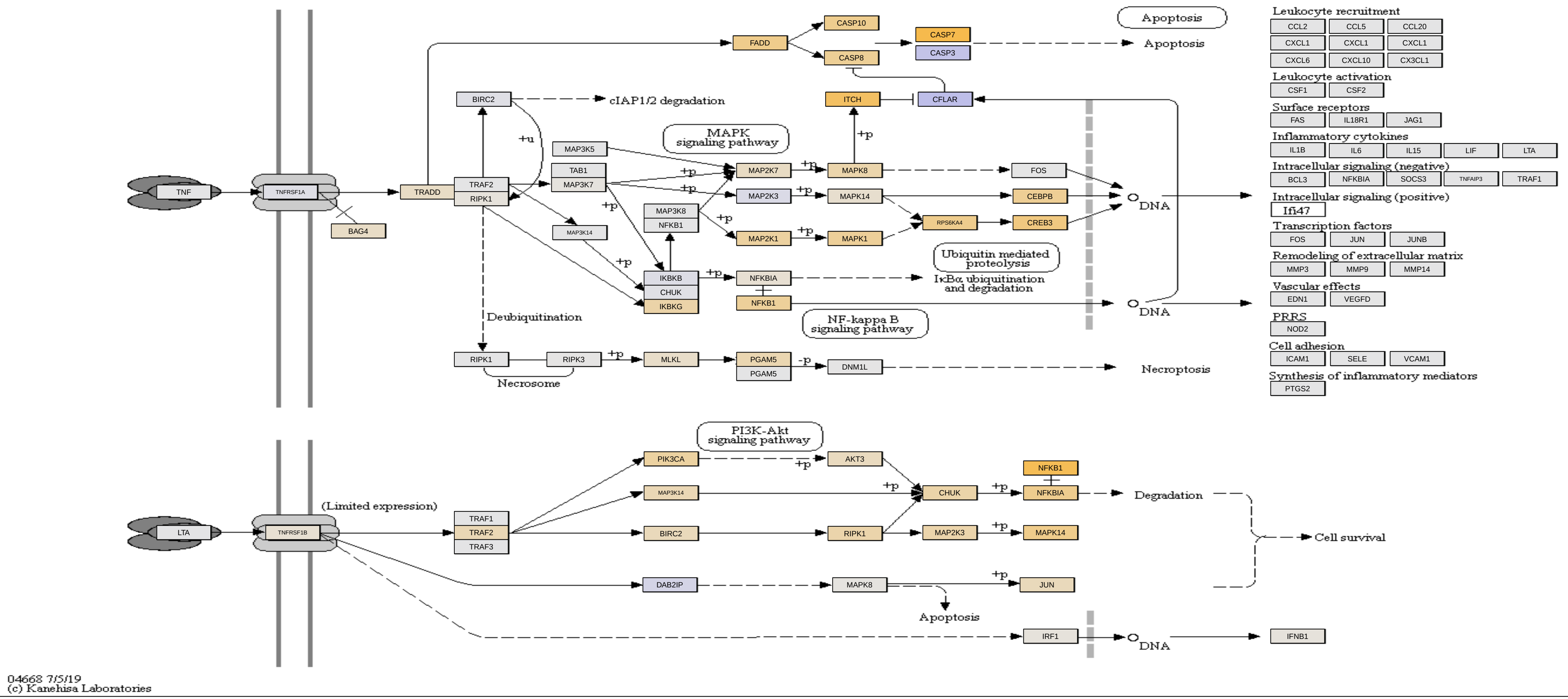

TNF_signaling_pathway
Late Adult

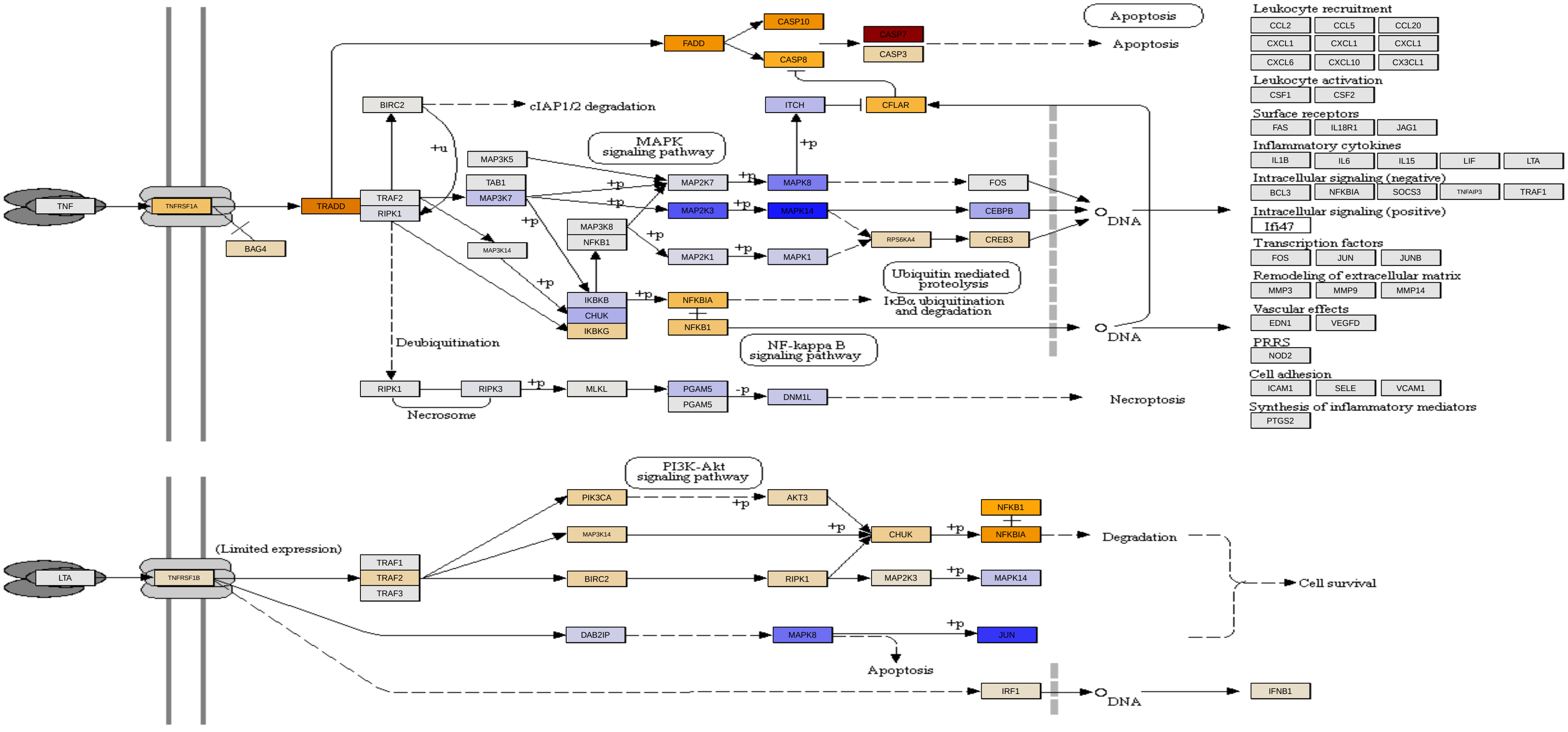

04668 7/5/19
(c) Kanehisa Laboratories
