# Supplementary material for: The Transcriptome and Methylome of the Developing and Aging Brain and Their Relations to Gliomas and Psychological Disorders
Source: Cells. 2022 Jan 21;11(3):362. doi: 10.3390/cells11030362 (PMC8834030; doi:10.3390/cells11030362)
Supplement: Supplementary file 1 [file cells-11-00362-s001.zip › Suppl-Material-S3-Pathways-PSF_Expression/VEGF_signaling_pathway.pdf]

VEGF signaling pathway

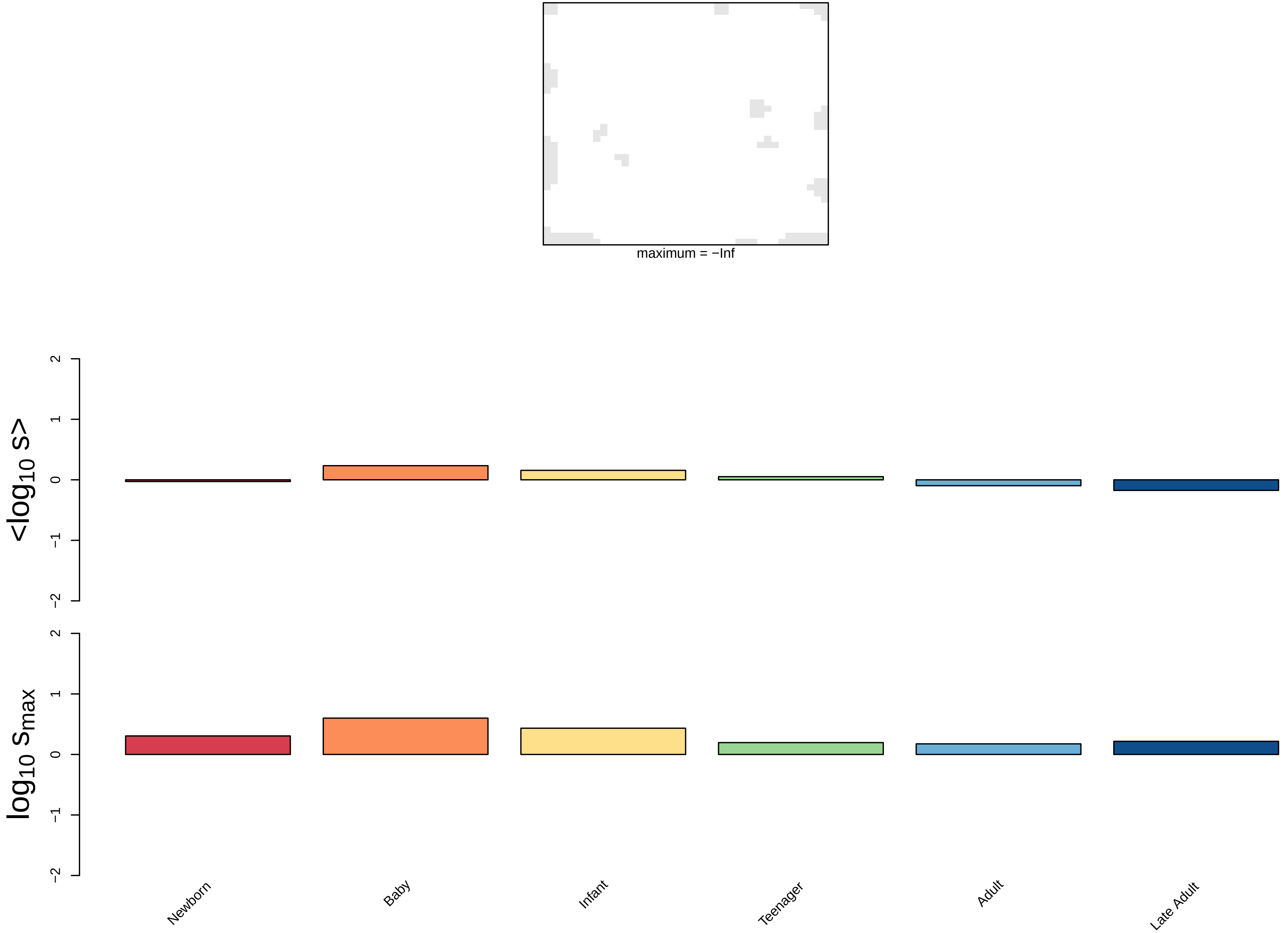

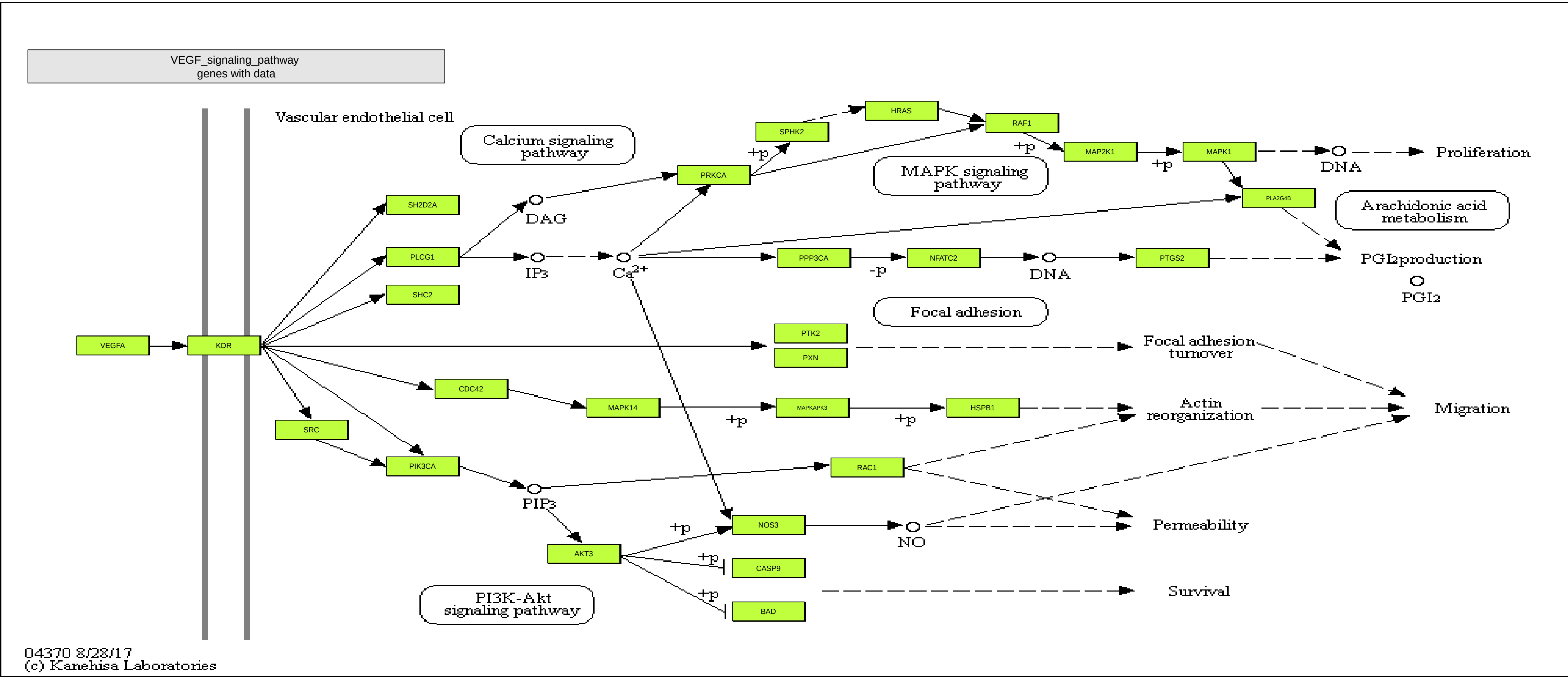
VEGF_signaling_pathway
genes with data
Vascular endothelial cell
Calcium signaling pathway
MAPK signaling pathway
Arachidonic acid metabolism
Focal adhesion
PI3K-Akt signaling pathway
VEGFA
KDR
SH2D2A
PLCG1
SHC2
SRC
PIK3CA
CDC42
MAPK14
MAPKAPK3
HSPB1
PRKCA
SPHK2
HRAS
RAF1
MAP2K1
MAPK1
PLA2G4B
PPP3CA
NFATC2
PTGS2
PTK2
PXN
RAC1
AKT3
NOS3
CASP9
BAD
DAG
IP3
Ca2+
PIP3
NO
DNA
PGI2
+p
-p
Proliferation
PGI2 production
Focal adhesion turnover
Actin reorganization
Migration
Permeability
Survival
04370 8/28/17
(c) Kanehisa Laboratories

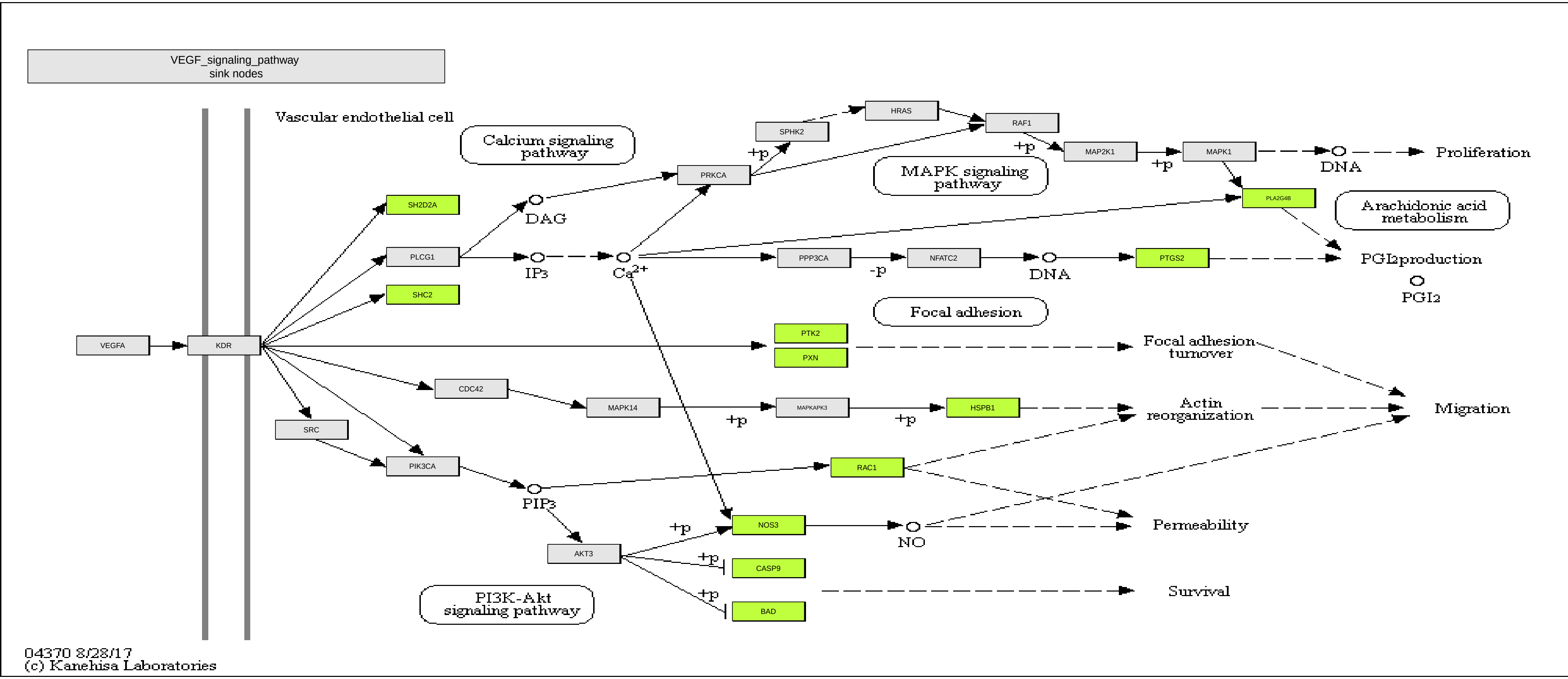
VEGF_signaling_pathway
sink nodes
Vascular endothelial cell
Calcium signaling pathway
MAPK signaling pathway
Arachidonic acid metabolism
Focal adhesion
PI3K-Akt signaling pathway
VEGFA
KDR
SH2D2A
PLCG1
SHC2
SRC
PIK3CA
CDC42
MAPK14
MAPKAPK3
HSPB1
PTK2
PXN
RAC1
NOS3
CASP9
BAD
AKT3
PRKCA
SPHK2
HRAS
RAF1
MAP2K1
MAPK1
PLA2G4B
PPP3CA
NFATC2
PTGS2
DAG
IP3
Ca2+
PIP3
NO
DNA
PGI2
+p
-p
Proliferation
PGI2production
Focal adhesion turnover
Actin reorganization
Migration
Permeability
Survival
04370 8/28/17
(c) Kanehisa Laboratories

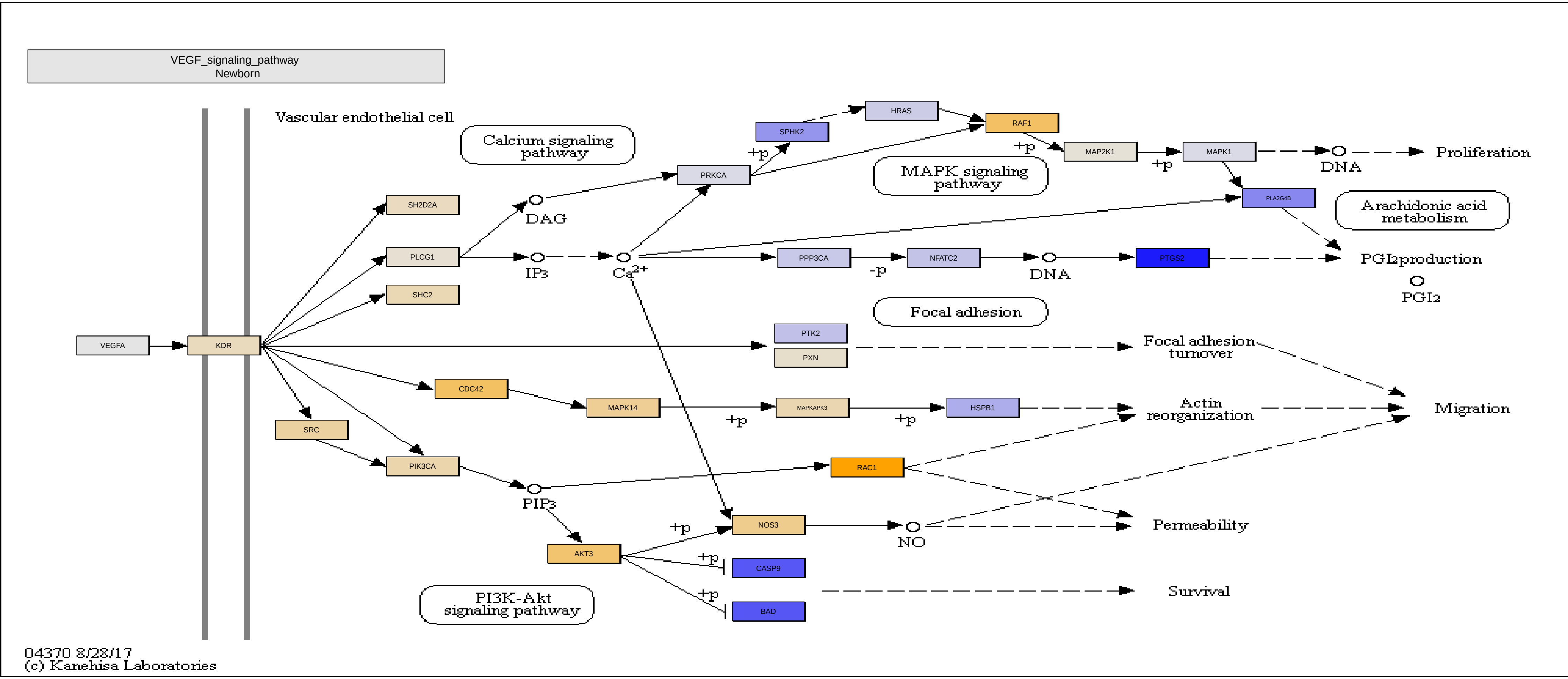
VEGF_signaling_pathway
Newborn
Vascular endothelial cell
Calcium signaling pathway
MAPK signaling pathway
Arachidonic acid metabolism
Focal adhesion
PI3K-Akt signaling pathway
VEGFA
KDR
SH2D2A
PLCG1
SHC2
SRC
PIK3CA
CDC42
MAPK14
MAPKAPK3
HSPB1
PTK2
PXN
PRKCA
SPHK2
HRAS
RAF1
MAP2K1
MAPK1
PLA2G4B
PPP3CA
NFATC2
PTGS2
RAC1
AKT3
NOS3
CASP9
BAD
DAG
IP3
Ca2+
PIP3
DNA
NO
PGI2
+p
-p
Proliferation
PGI2production
Focal adhesion turnover
Actin reorganization
Migration
Permeability
Survival
04370 8/28/17
(c) Kanehisa Laboratories

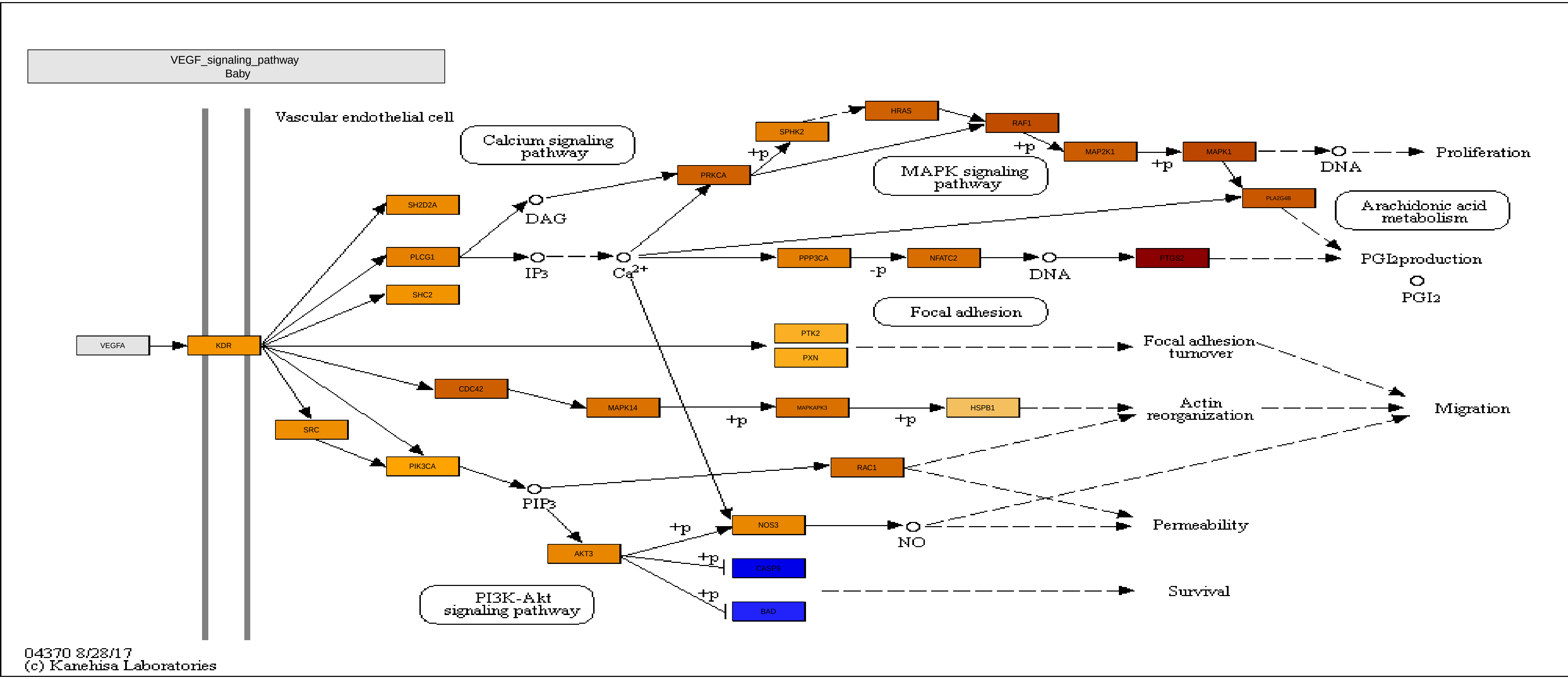
VEGF_signaling_pathway
Baby
Vascular endothelial cell
Calcium signaling pathway
MAPK signaling pathway
Arachidonic acid metabolism
Focal adhesion
PI3K-Akt signaling pathway
VEGFA
KDR
SH2D2A
PLCG1
SHC2
SRC
PIK3CA
CDC42
MAPK14
MAPKAPK3
HSPB1
PTK2
PXN
PRKCA
SPHK2
HRAS
RAF1
MAP2K1
MAPK1
PLA2G4B
PPP3CA
NFATC2
PTGS2
RAC1
NOS3
AKT3
CASP9
BAD
DAG
IP3
Ca2+
PIP3
NO
DNA
PGI2
+p
-p
Proliferation
PGI2 production
Focal adhesion turnover
Actin reorganization
Migration
Permeability
Survival
04370 8/28/17
(c) Kanehisa Laboratories

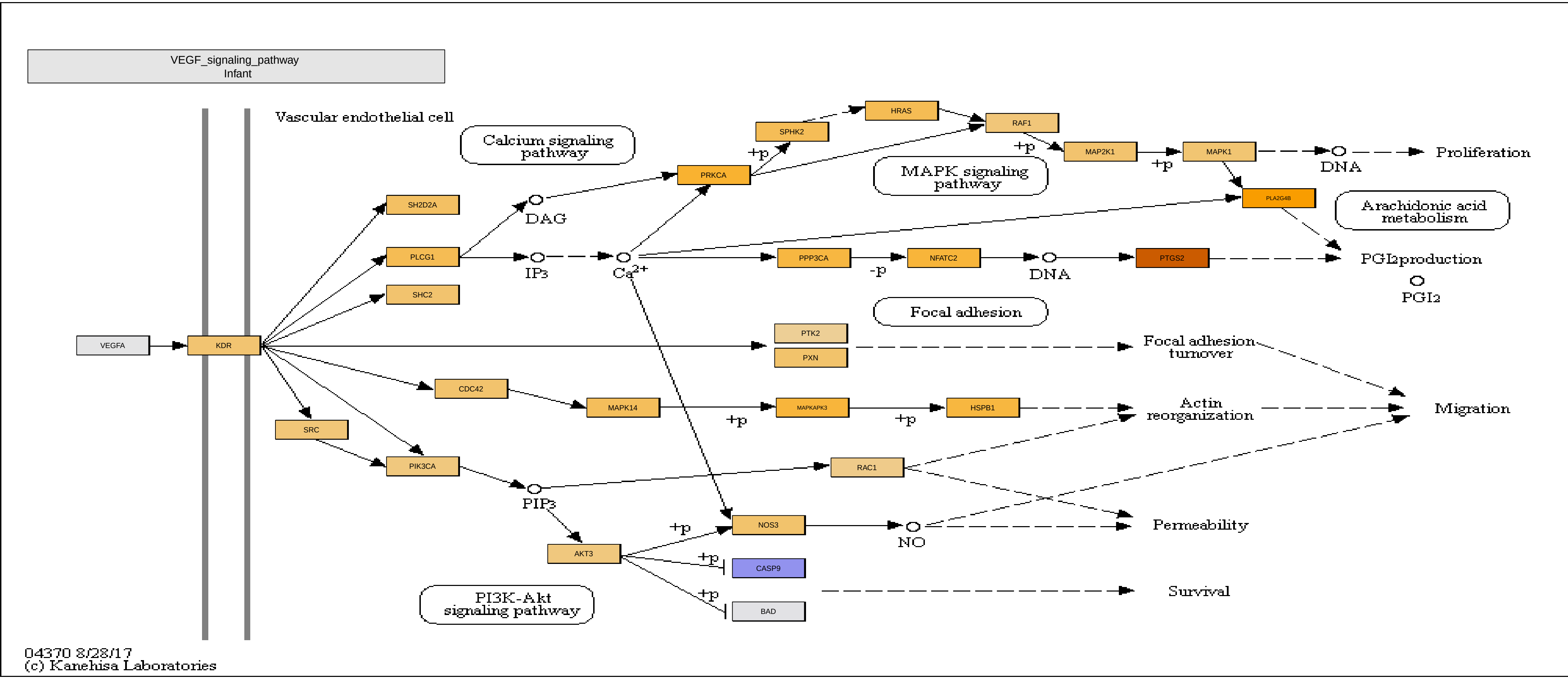
VEGF_signaling_pathway
Infant
Vascular endothelial cell
Calcium signaling pathway
MAPK signaling pathway
Arachidonic acid metabolism
Focal adhesion
PI3K-Akt signaling pathway
VEGFA
KDR
SH2D2A
PLCG1
SHC2
SRC
PIK3CA
CDC42
MAPK14
MAPKAPK3
HSPB1
PTK2
PXN
RAC1
AKT3
NOS3
CASP9
BAD
PRKCA
SPHK2
HRAS
RAF1
MAP2K1
MAPK1
PLA2G4B
PPP3CA
NFATC2
PTGS2
DAG
IP3
Ca2+
PIP3
NO
DNA
PGI2production
PGI2
Proliferation
Focal adhesion turnover
Actin reorganization
Migration
Permeability
Survival
+p
-p
04370 8/28/17
(c) Kanehisa Laboratories

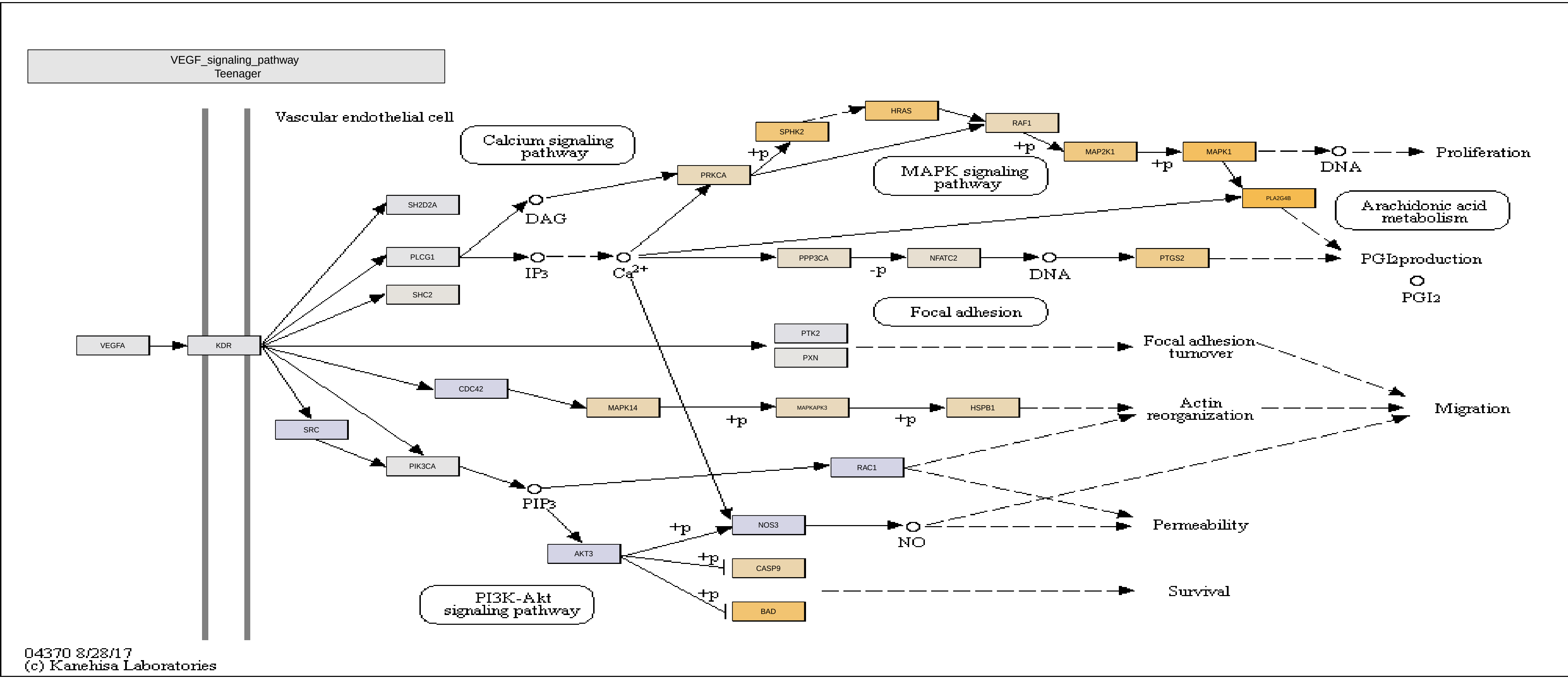

VEGF_signaling_pathway
Teenager
Vascular endothelial cell
Calcium signaling pathway
MAPK signaling pathway
Arachidonic acid metabolism
Focal adhesion
PI3K-Akt signaling pathway
VEGFA
KDR
SH2D2A
PLCG1
SHC2
SRC
PIK3CA
CDC42
MAPK14
MAPKAPK3
HSPB1
PTK2
PXN
DAG
IP3
Ca2+
PRKCA
SPHK2
HRAS
RAF1
MAP2K1
MAPK1
PLA2G4B
PPP3CA
NFATC2
PTGS2
DNA
Proliferation
PGI2production
PGI2
Focal adhesion turnover
Actin reorganization
Migration
RAC1
PIP3
AKT3
NOS3
NO
Permeability
CASP9
BAD
Survival
+p
-p
04370 8/28/17
(c) Kanehisa Laboratories

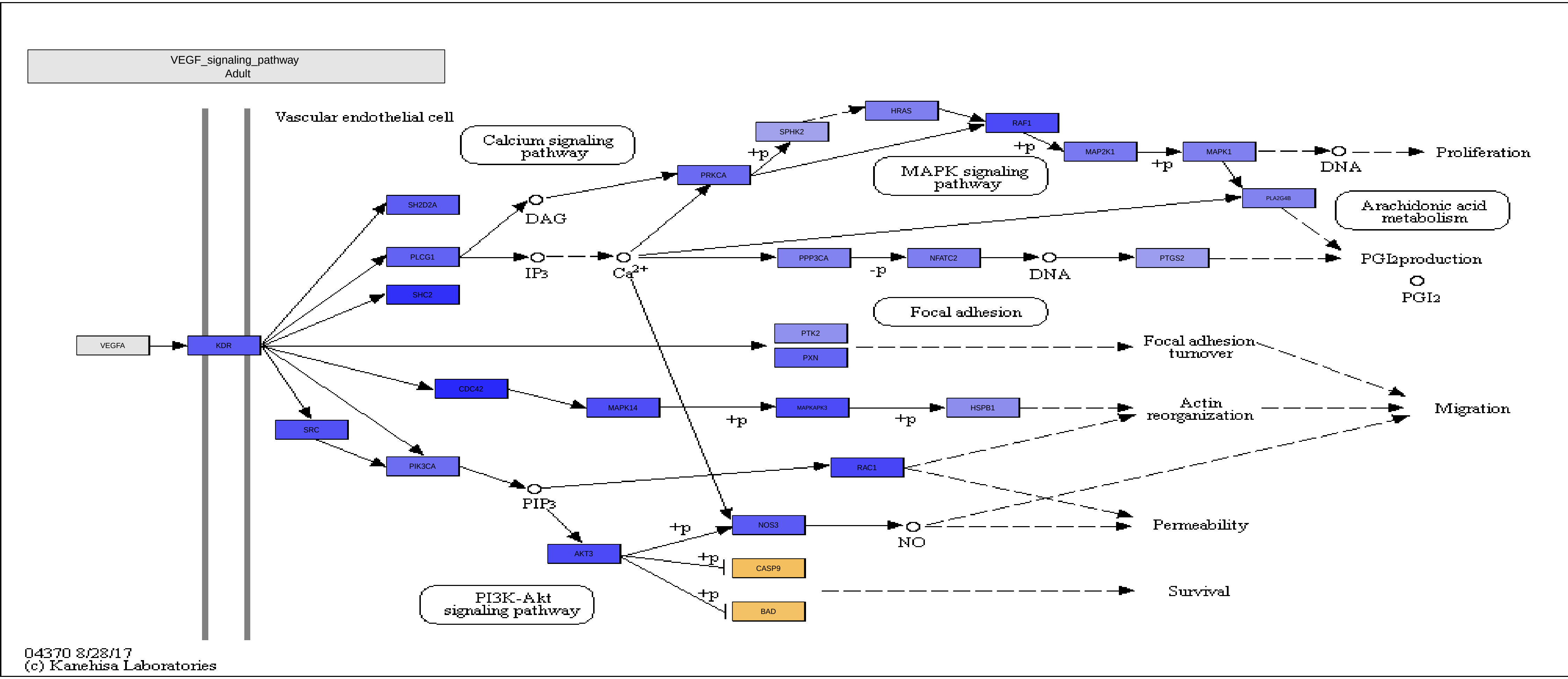
VEGF_signaling_pathway
Adult
Vascular endothelial cell
Calcium signaling pathway
MAPK signaling pathway
Arachidonic acid metabolism
Focal adhesion
PI3K-Akt signaling pathway
VEGFA
KDR
SH2D2A
PLCG1
SHC2
SRC
CDC42
PIK3CA
DAG
IP3
Ca2+
PIP3
PRKCA
SPHK2
HRAS
RAF1
MAP2K1
MAPK1
PLA2G4B
PPP3CA
NFATC2
PTGS2
PTK2
PXN
MAPK14
MAPKAPK3
HSPB1
RAC1
AKT3
NOS3
CASP9
BAD
NO
DNA
PGI2
+p
-p
Proliferation
PGI2production
Focal adhesion turnover
Actin reorganization
Migration
Permeability
Survival
04370 8/28/17
(c) Kanehisa Laboratories

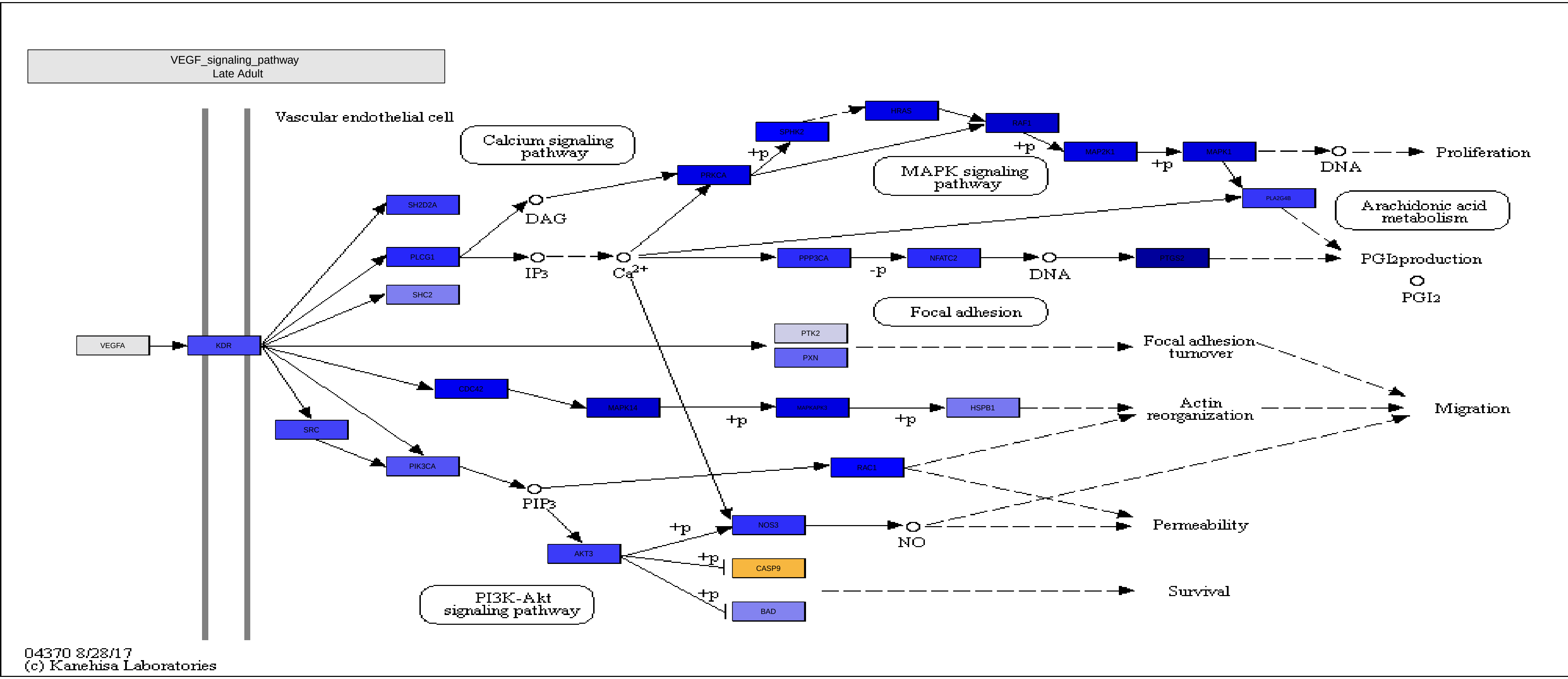
VEGF_signaling_pathway
Late Adult
Vascular endothelial cell
Calcium signaling pathway
MAPK signaling pathway
Arachidonic acid metabolism
Focal adhesion
PI3K-Akt signaling pathway
VEGFA
KDR
SH2D2A
PLCG1
SHC2
PTK2
PXN
SRC
PIK3CA
AKT3
NOS3
CASP9
BAD
PPP3CA
NFATC2
HSPB1
RAC1
DAG
IP3
Ca2+
PIP3
DNA
NO
PGI2
+p
-p
Proliferation
PGI2 production
Focal adhesion turnover
Actin reorganization
Migration
Permeability
Survival
04370 8/28/17
(c) Kanehisa Laboratories
