# Supplementary material for: The Transcriptome and Methylome of the Developing and Aging Brain and Their Relations to Gliomas and Psychological Disorders
Source: Cells. 2022 Jan 21;11(3):362. doi: 10.3390/cells11030362 (PMC8834030; doi:10.3390/cells11030362)
Supplement: Supplementary file 1 [file cells-11-00362-s001.zip › Suppl-Material-S4-Pathways-PSF_Methylation/Adipocytokine_signaling_pathway.pdf]

Adipocytokine signaling pathway

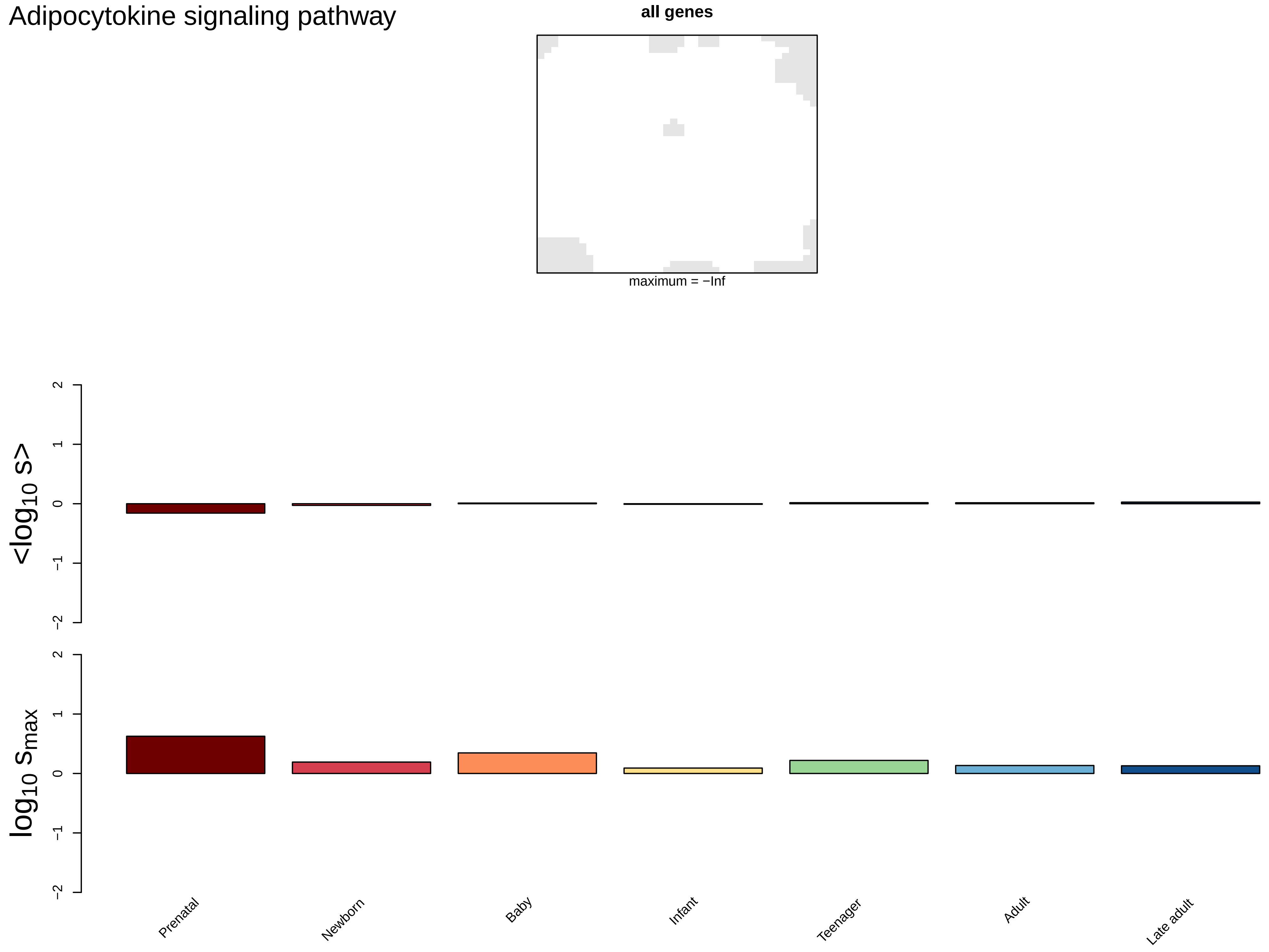

Adipocytokine_signaling_pathway
genes with data

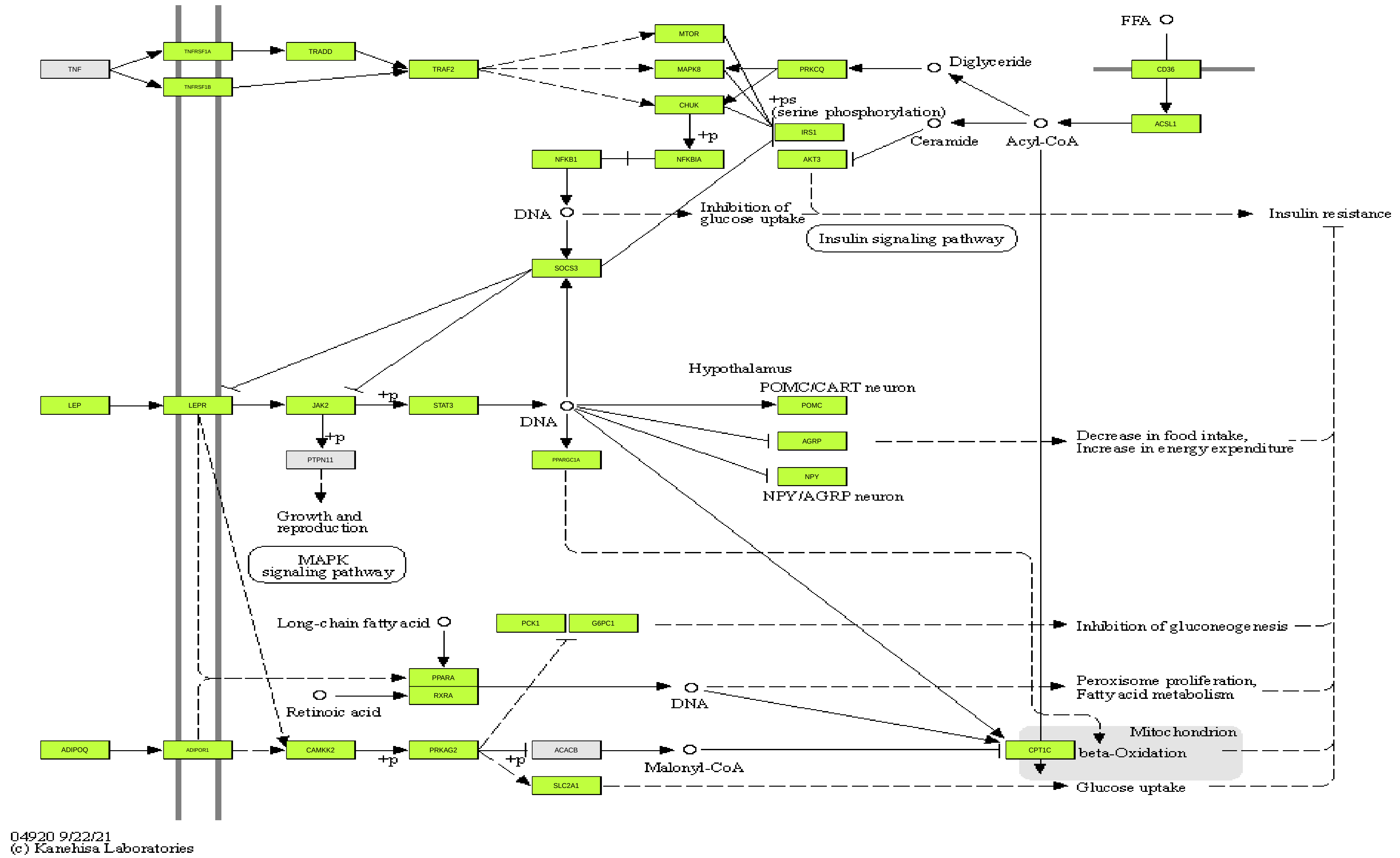

Adipocytokine_signaling_pathway
sink nodes

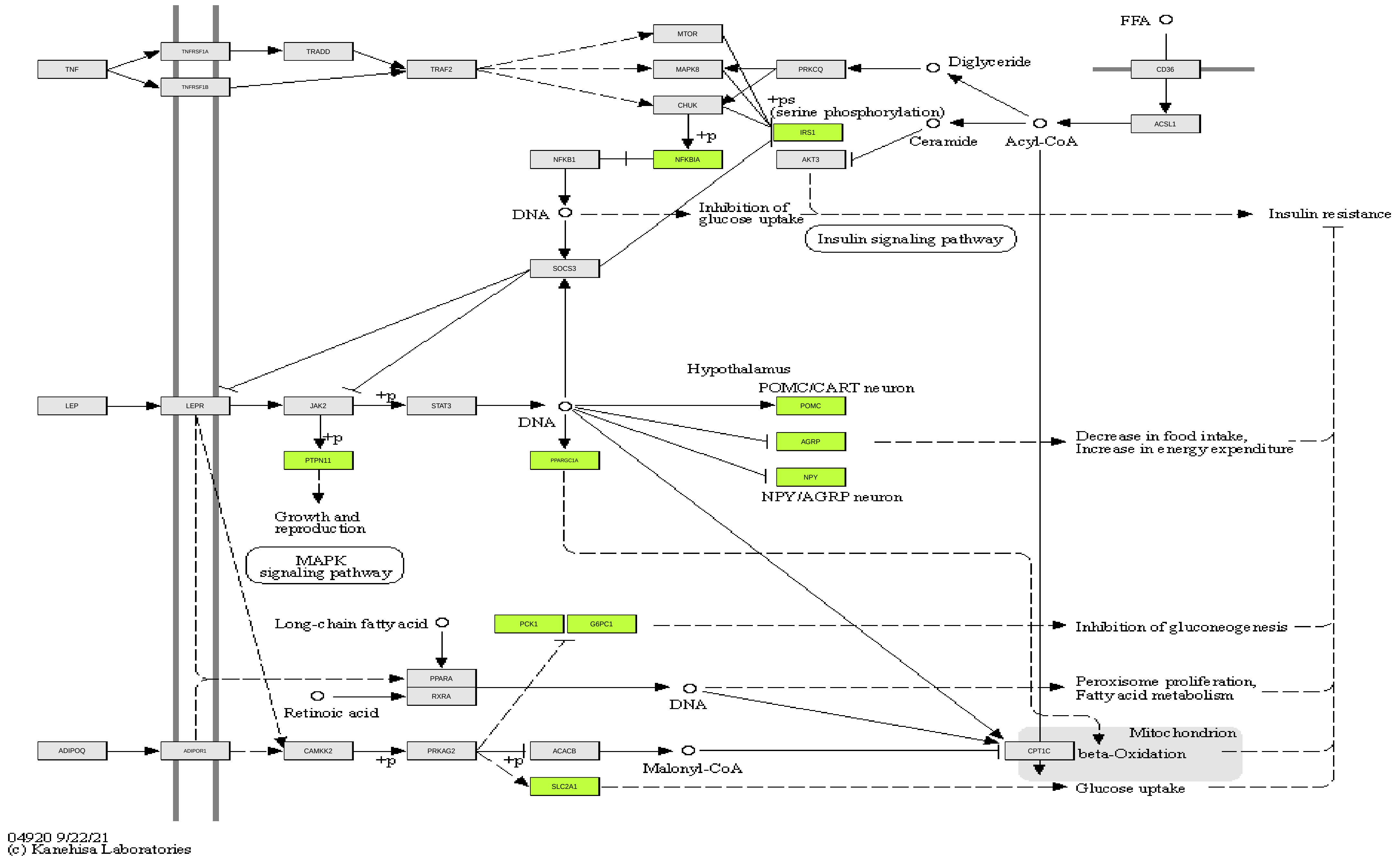

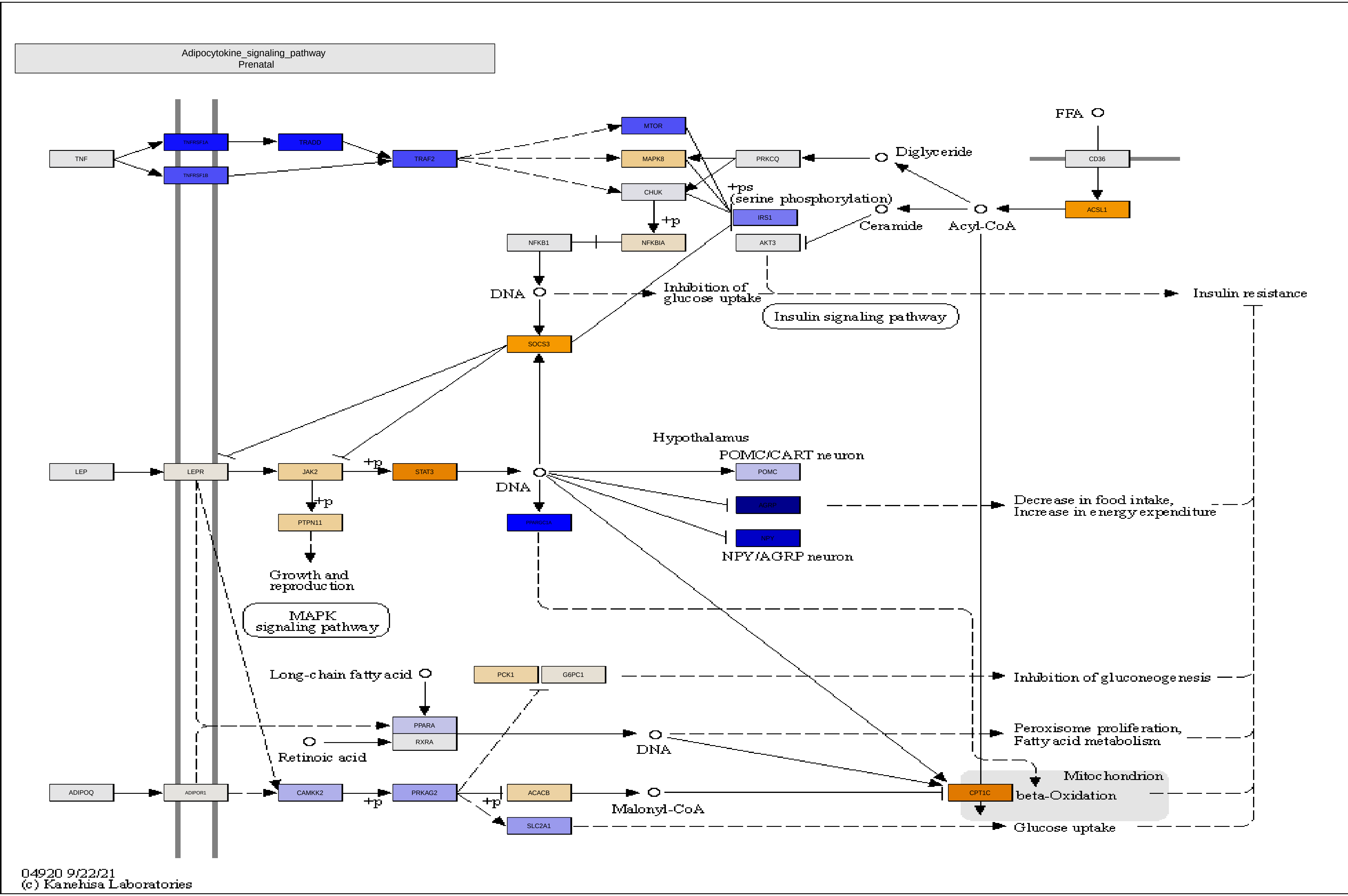
Adipocytokine_signaling_pathway
Prenatal
TNF
TNFRSF1A
TNFRSF1B
TRADD
TRAF2
MTOR
MAPK8
CHUK
PRKCQ
Diglyceride
FFA
CD36
ACSL1
Acyl-CoA
Ceramide
+ps
(serine phosphorylation)
IRS1
AKT3
NFKB1
NFKBIA
+p
DNA
Inhibition of
glucose uptake
Insulin resistance
Insulin signaling pathway
SOCS3
Hypothalamus
POMC/CART neuron
LEP
LEPR
JAK2
+p
STAT3
DNA
POMC
AGRP
NPY
NPY/AGRP neuron
PPARGC1A
Decrease in food intake,
Increase in energy expenditure
PTPN11
Growth and
reproduction
MAPK
signaling pathway
Long-chain fatty acid
PCK1
G6PC1
Inhibition of gluconeogenesis
PPARA
RXRA
Retinoic acid
DNA
Peroxisome proliferation,
Fatty acid metabolism
Mitochondrion
ADIPOQ
ADIPOR1
CAMKK2
PRKAG2
ACACB
Malonyl-CoA
CPT1C
beta-Oxidation
SLC2A1
Glucose uptake
04920 9/22/21
(c) Kanehisa Laboratories

Adipocytokine_signaling_pathway
Newborn

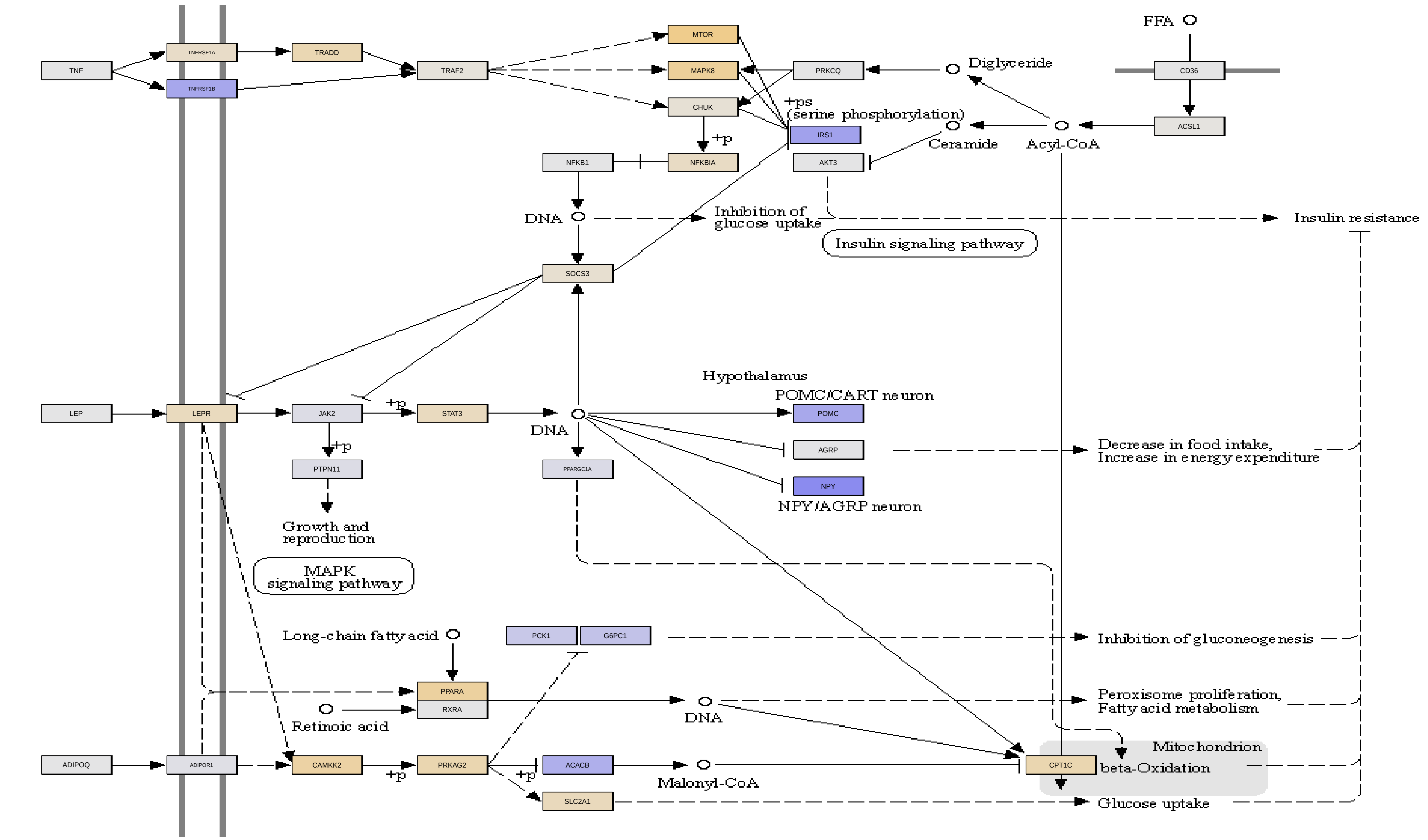

04920 9/22/21
(c) Kanehisa Laboratories

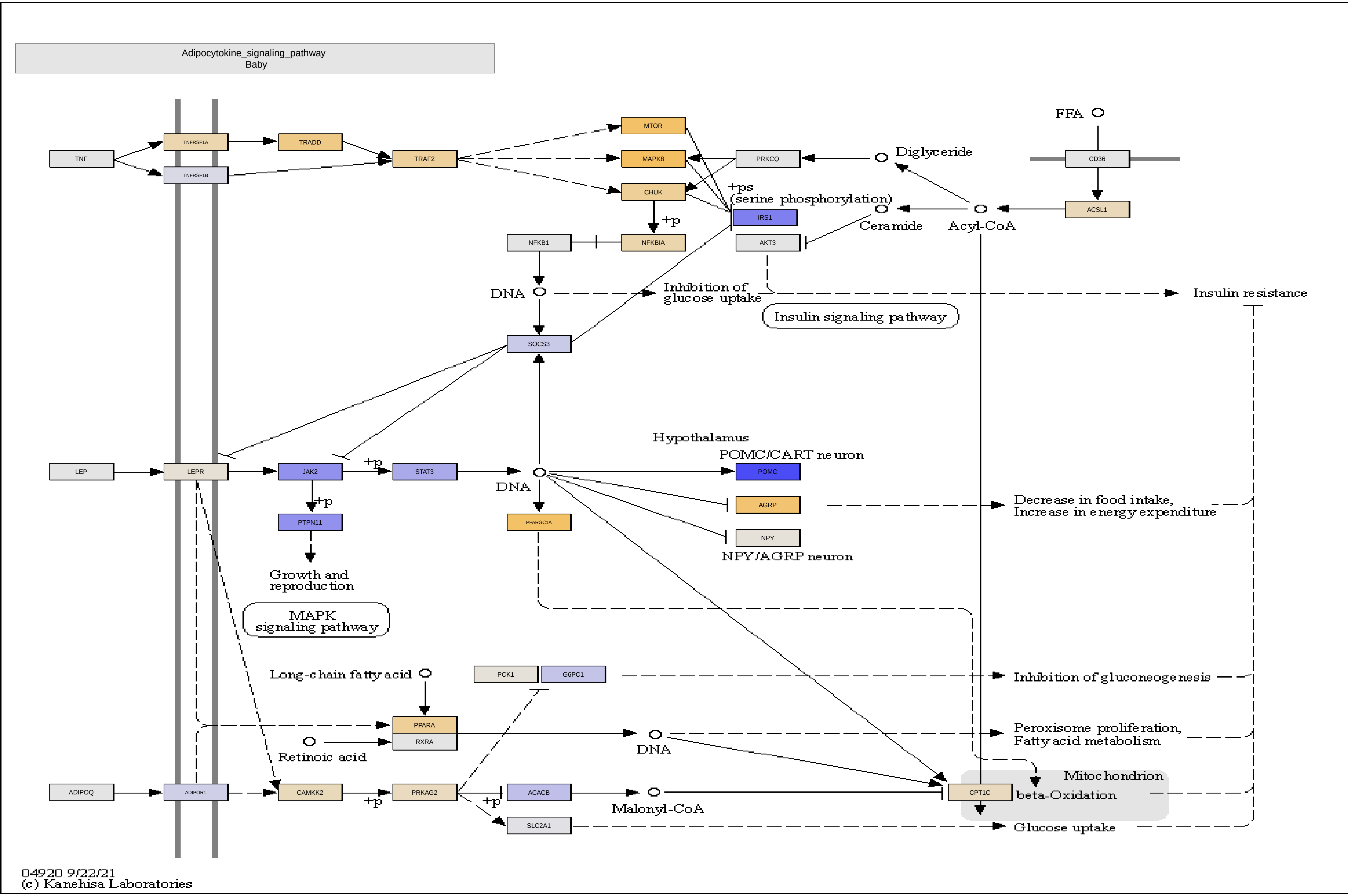

Adipocytokine_signaling_pathway
Baby
TNF
TNFRSF1A
TNFRSF1B
TRADD
TRAF2
MTOR
MAPK8
CHUK
PRKCQ
Diglyceride
FFA
CD36
ACSL1
Acyl-CoA
Ceramide
+ps
(serine phosphorylation)
IRS1
AKT3
NFKB1
NFKBIA
+p
DNA
Inhibition of
glucose uptake
Insulin resistance
Insulin signaling pathway
SOCS3
Hypothalamus
POMC/CART neuron
POMC
AGRP
NPY
NPY/AGRP neuron
Decrease in food intake,
Increase in energy expenditure
LEP
LEPR
JAK2
+p
STAT3
DNA
PTPN11
+p
PPARGC1A
Growth and
reproduction
MAPK
signaling pathway
Long-chain fatty acid
PCK1
G6PC1
Inhibition of gluconeogenesis
PPARA
RXRA
Retinoic acid
DNA
Peroxisome proliferation,
Fatty acid metabolism
Mitochondrion
ADIPOQ
ADIPOR1
CAMKK2
+p
PRKAG2
+p
ACACB
Malonyl-CoA
CPT1C
beta-Oxidation
SLC2A1
Glucose uptake
04920 9/22/21
(c) Kanehisa Laboratories

Adipocytokine_signaling_pathway
Infant

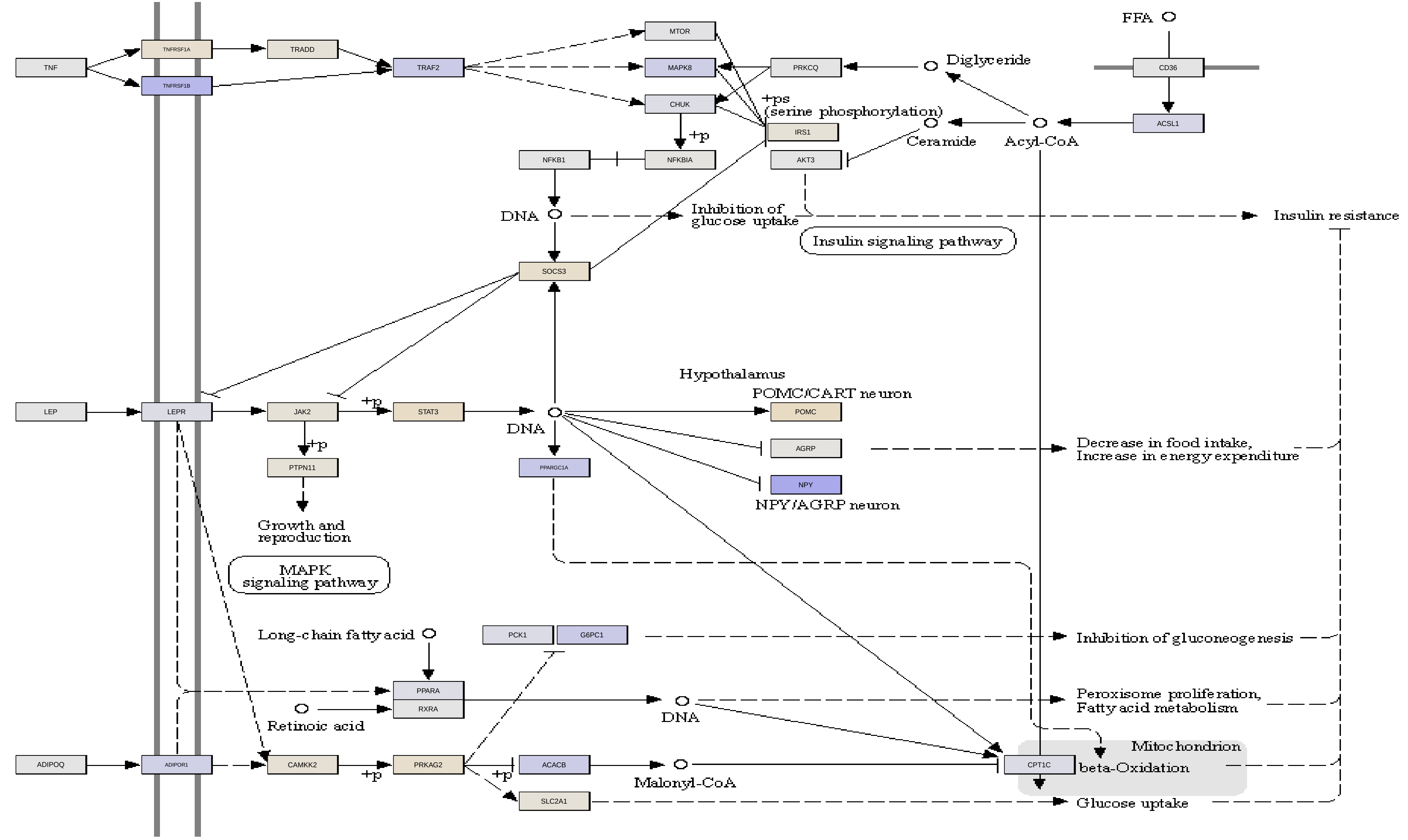

04920 9/22/21
(c) Kanehisa Laboratories

Adipocytokine_signaling_pathway
Teenager

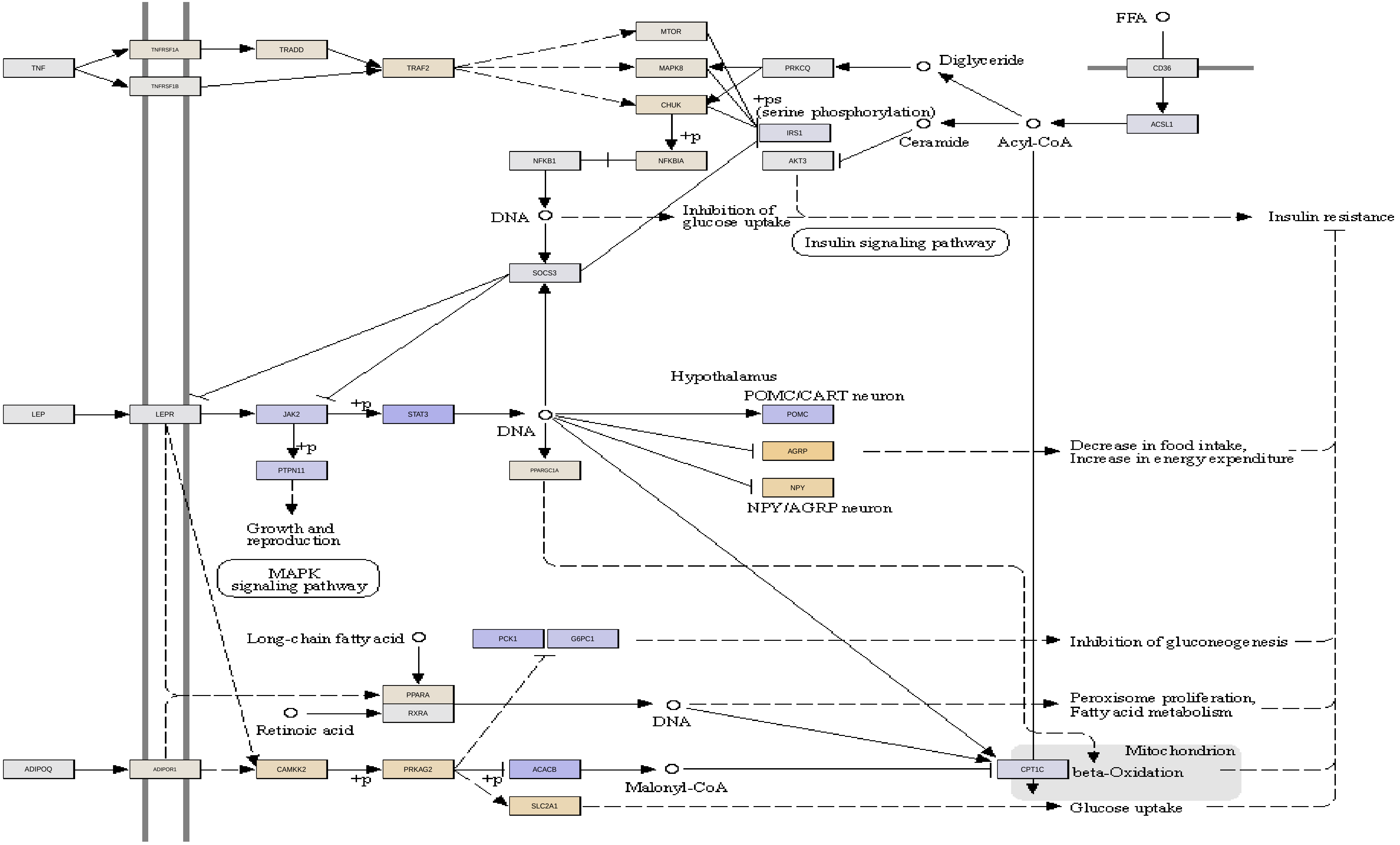

04920 9/22/21
(c) Kanehisa Laboratories

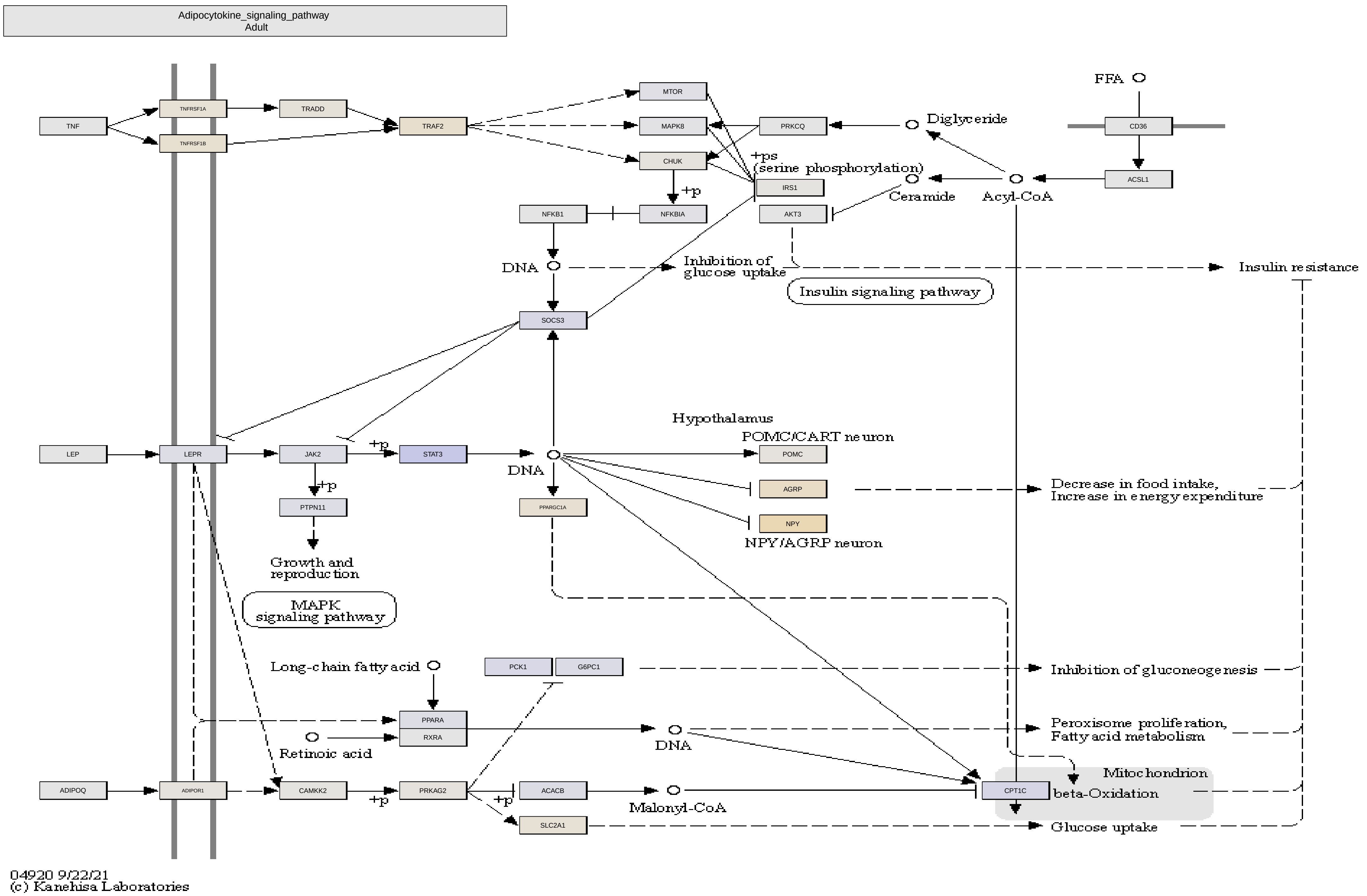
Adipocytokine_signaling_pathway
Adult
TNF
TNFRSF1A
TNFRSF1B
TRADD
TRAF2
MTOR
MAPK8
CHUK
NFKBIA
NFKB1
PRKCQ
Diglyceride
FFA
CD36
ACSL1
+ps
(serine phosphorylation)
IRS1
AKT3
Ceramide
Acyl-CoA
+p
DNA
Inhibition of
glucose uptake
Insulin signaling pathway
Insulin resistance
SOCS3
Hypothalamus
POMC/CART neuron
LEP
LEPR
JAK2
+p
STAT3
DNA
POMC
AGRP
NPY
NPY/AGRP neuron
Decrease in food intake,
Increase in energy expenditure
+p
PTPN11
PPARGC1A
Growth and
reproduction
MAPK
signaling pathway
Long-chain fatty acid
PCK1
G6PC1
Inhibition of gluconeogenesis
PPARA
RXRA
Retinoic acid
DNA
Peroxisome proliferation,
Fatty acid metabolism
Mitochondrion
ADIPOQ
ADIPOR1
CAMKK2
+p
PRKAG2
+p
ACACB
Malonyl-CoA
CPT1C
beta-Oxidation
SLC2A1
Glucose uptake
04920 9/22/21
(c) Kanehisa Laboratories

Adipocytokine_signaling_pathway
Late adult

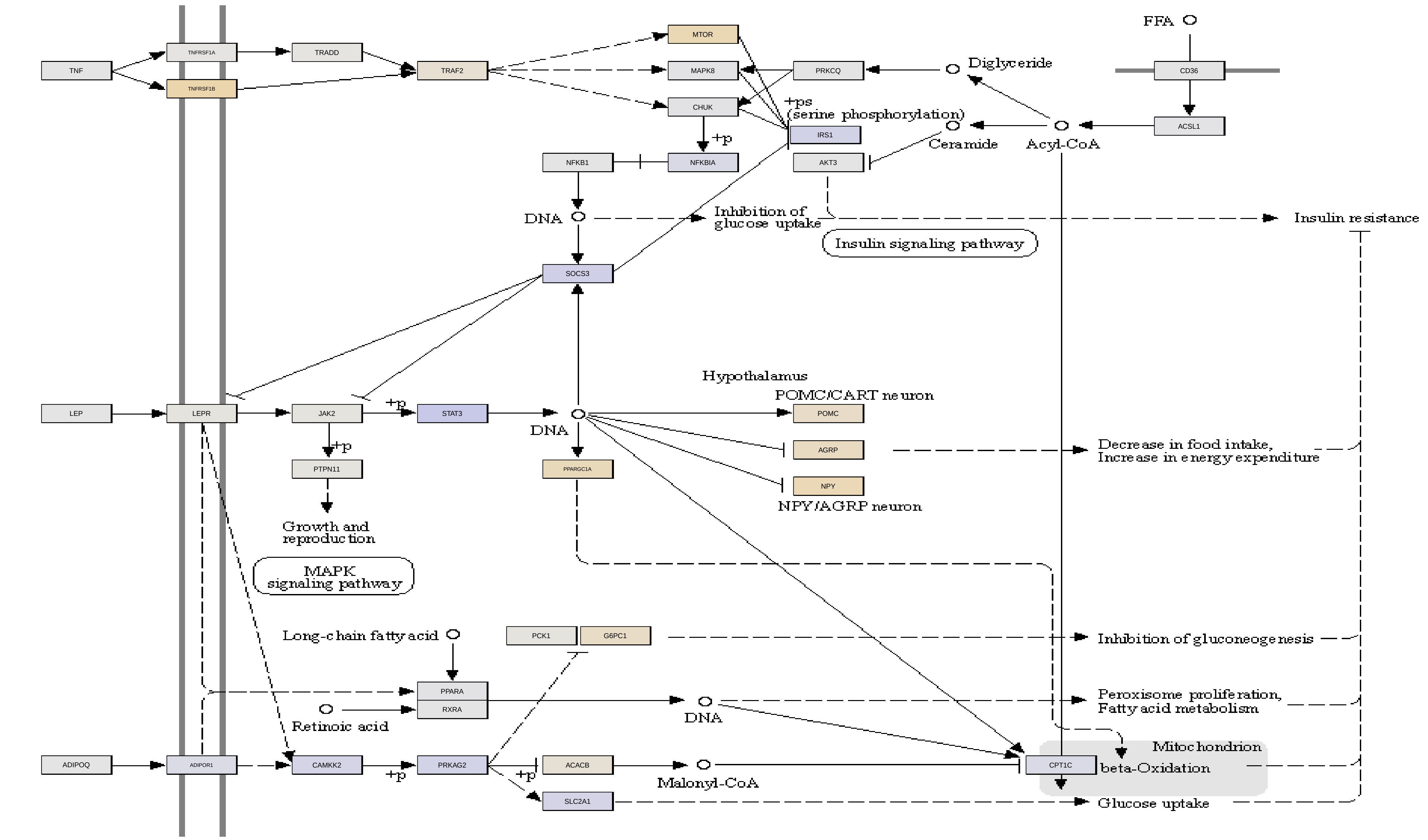

04920 9/22/21
(c) Kanehisa Laboratories
