# Supplementary material for: The Transcriptome and Methylome of the Developing and Aging Brain and Their Relations to Gliomas and Psychological Disorders
Source: Cells. 2022 Jan 21;11(3):362. doi: 10.3390/cells11030362 (PMC8834030; doi:10.3390/cells11030362)
Supplement: Supplementary file 1 [file cells-11-00362-s001.zip › Suppl-Material-S4-Pathways-PSF_Methylation/Adrenergic_signaling_in_cardiomyocytes.pdf]

Adrenergic signaling in cardiomyocyt

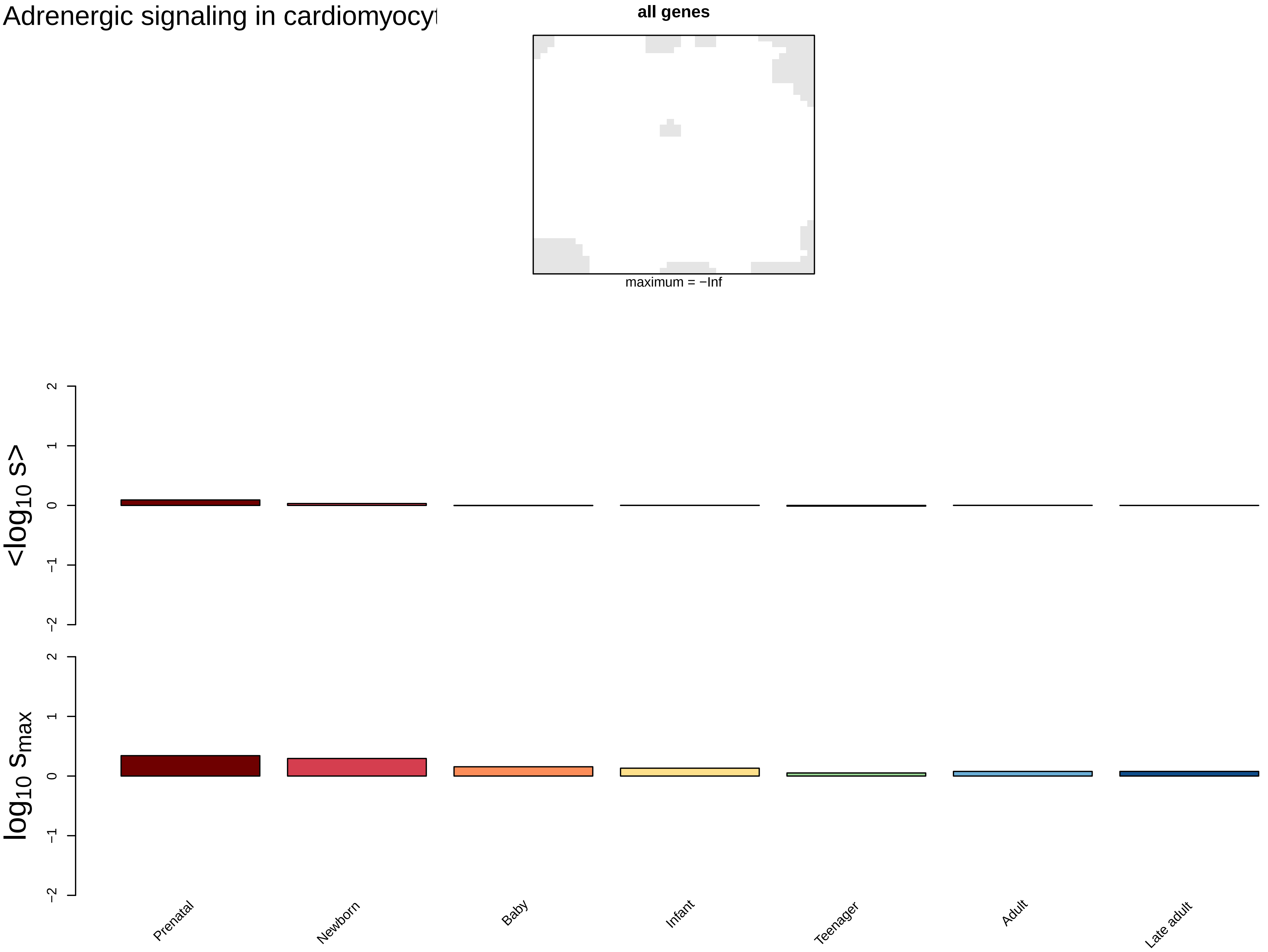

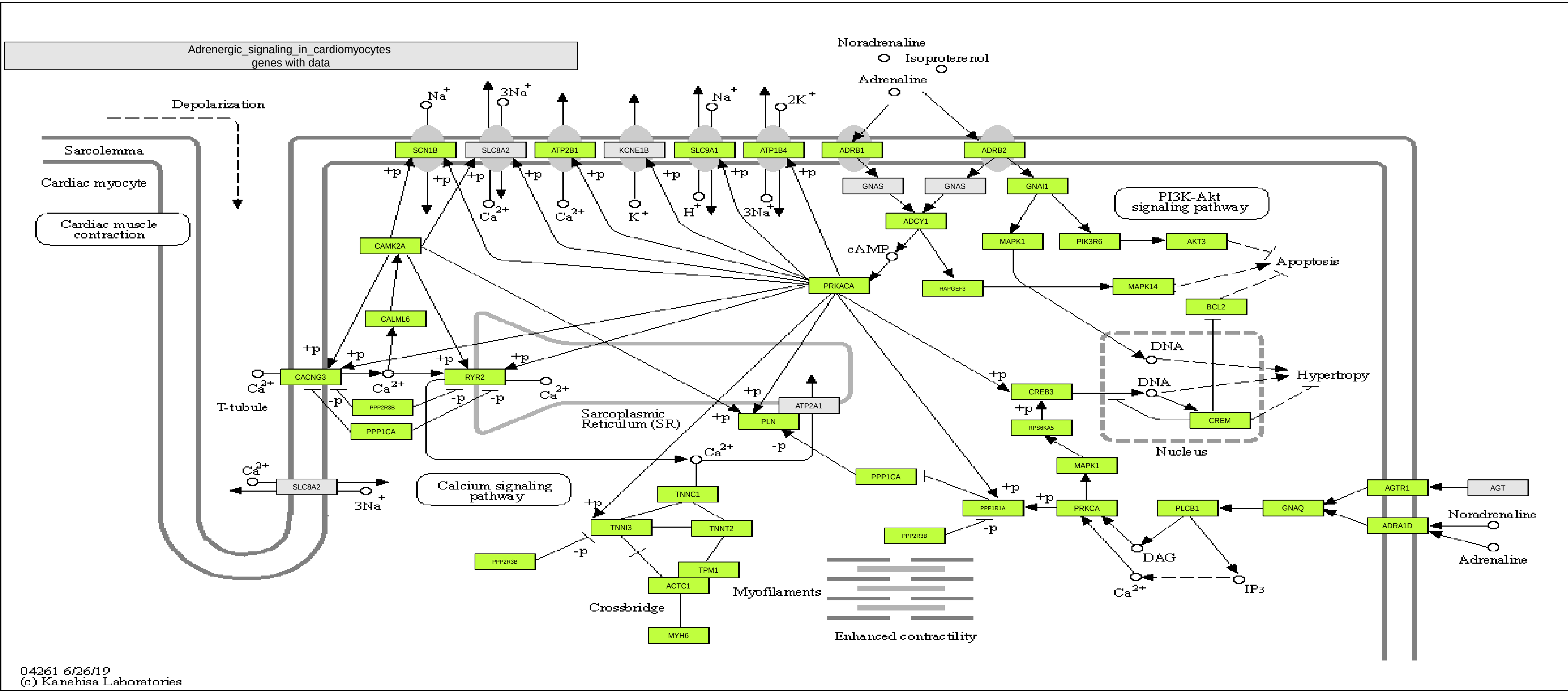
Adrenergic_signaling_in_cardiomyocytes
genes with data
Depolarization
Sarcolemma
Cardiac myocyte
Cardiac muscle contraction
Noradrenaline
Isoproterenol
Adrenaline
SCN1B
SLC8A2
ATP2B1
KCNE1B
SLC9A1
ATP1B4
ADRB1
ADRB2
GNAS
GNAS
GNAI1
ADCY1
cAMP
PRKACA
RAPGEF3
MAPK1
PIK3R6
AKT3
MAPK14
BCL2
PI3K-Akt signaling pathway
Apoptosis
DNA
DNA
Hypertropy
CREB3
RPS6KA5
CREM
Nucleus
MAPK1
PRKCA
PPP1R1A
PPP2R3B
PLCB1
GNAQ
AGTR1
AGT
ADRA1D
Noradrenaline
Adrenaline
DAG
IP3
CAMK2A
CALML6
CACNG3
RYR2
PPP2R3B
PPP1CA
T-tubule
Sarcoplasmic Reticulum (SR)
ATP2A1
PLN
PPP1CA
SLC8A2
3Na+
Calcium signaling pathway
TNNC1
TNNI3
TNNT2
TPM1
ACTC1
MYH6
PPP2R3B
Crossbridge
Myofilaments
Enhanced contractility
+p
-p
Na+
3Na+
2K+
K+
H+
Ca2+
04261 6/26/19
(c) Kanehisa Laboratories

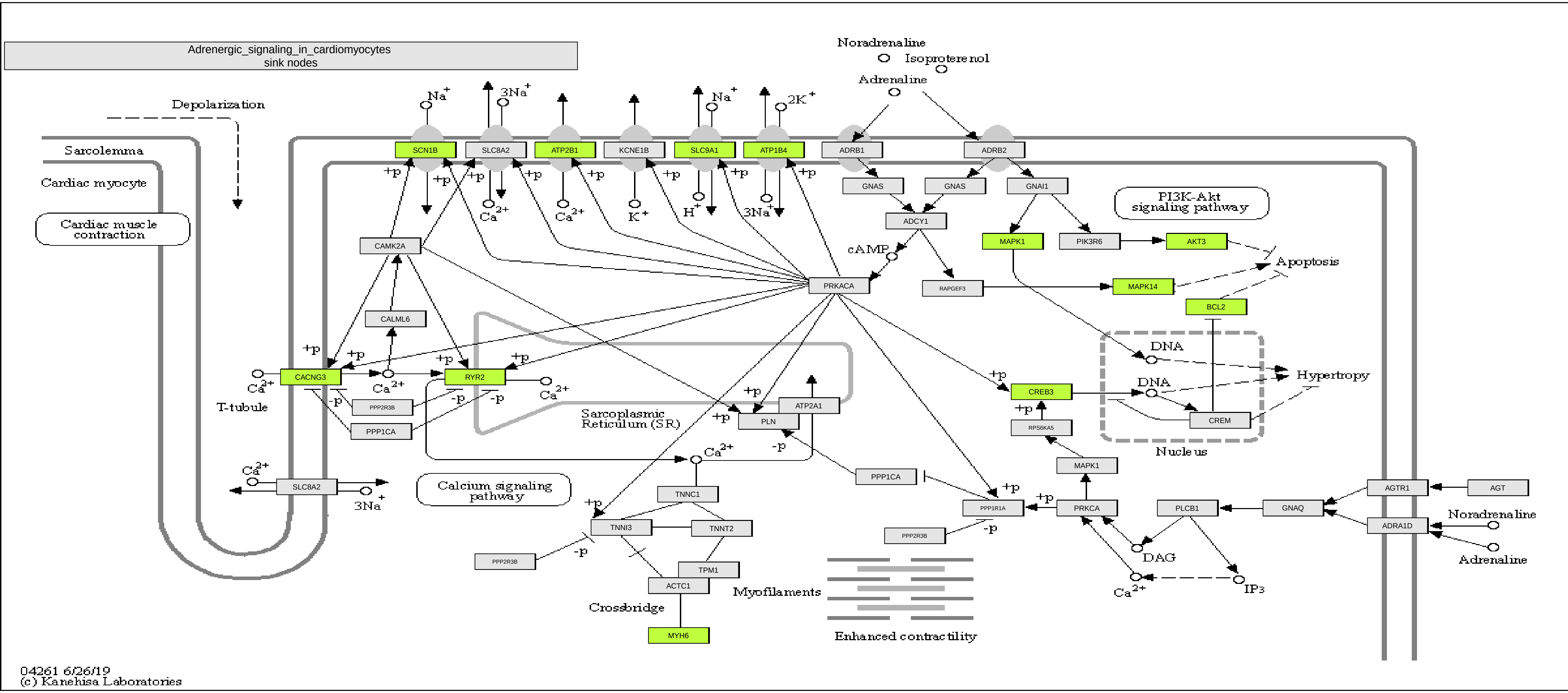
Adrenergic_signaling_in_cardiomyocytes
sink nodes
Depolarization
Sarcolemma
Cardiac myocyte
Cardiac muscle contraction
Noradrenaline
Isoproterenol
Adrenaline
SCN1B
SLC8A2
ATP2B1
KCNE1B
SLC9A1
ATP1B4
ADRB1
ADRB2
GNAS
GNAS
GNAI1
ADCY1
cAMP
MAPK1
PIK3R6
AKT3
PI3K-Akt signaling pathway
Apoptosis
RAPGEF3
MAPK14
BCL2
PRKACA
CAMK2A
CALML6
CACNG3
RYR2
T-tubule
PPP2R3B
PPP1CA
Sarcoplasmic Reticulum (SR)
ATP2A1
PLN
CREB3
RPS6KA5
DNA
DNA
Hypertropy
CREM
Nucleus
SLC8A2
3Na
Calcium signaling pathway
TNNC1
TNNI3
TNNT2
PPP2R3B
TPM1
ACTC1
Crossbridge
MYH6
Myofilaments
Enhanced contractility
PPP1CA
PPP1R1A
PPP2R3B
MAPK1
PRKCA
PLCB1
GNAQ
AGTR1
AGT
ADRA1D
Noradrenaline
Adrenaline
DAG
IP3
04261 6/26/19
(c) Kanehisa Laboratories

Adrenergic_signaling_in_cardiomyocytes
Prenatal

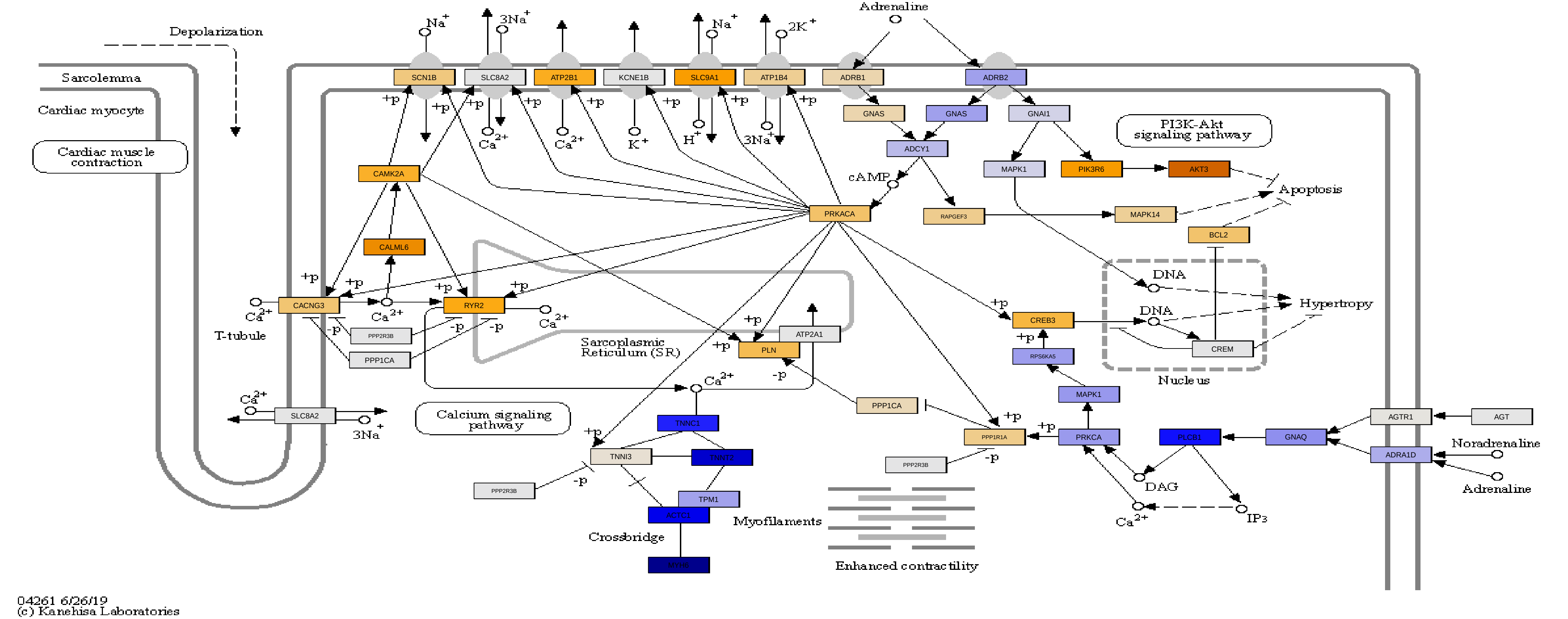

Adrenergic_signaling_in_cardiomyocytes
Newborn

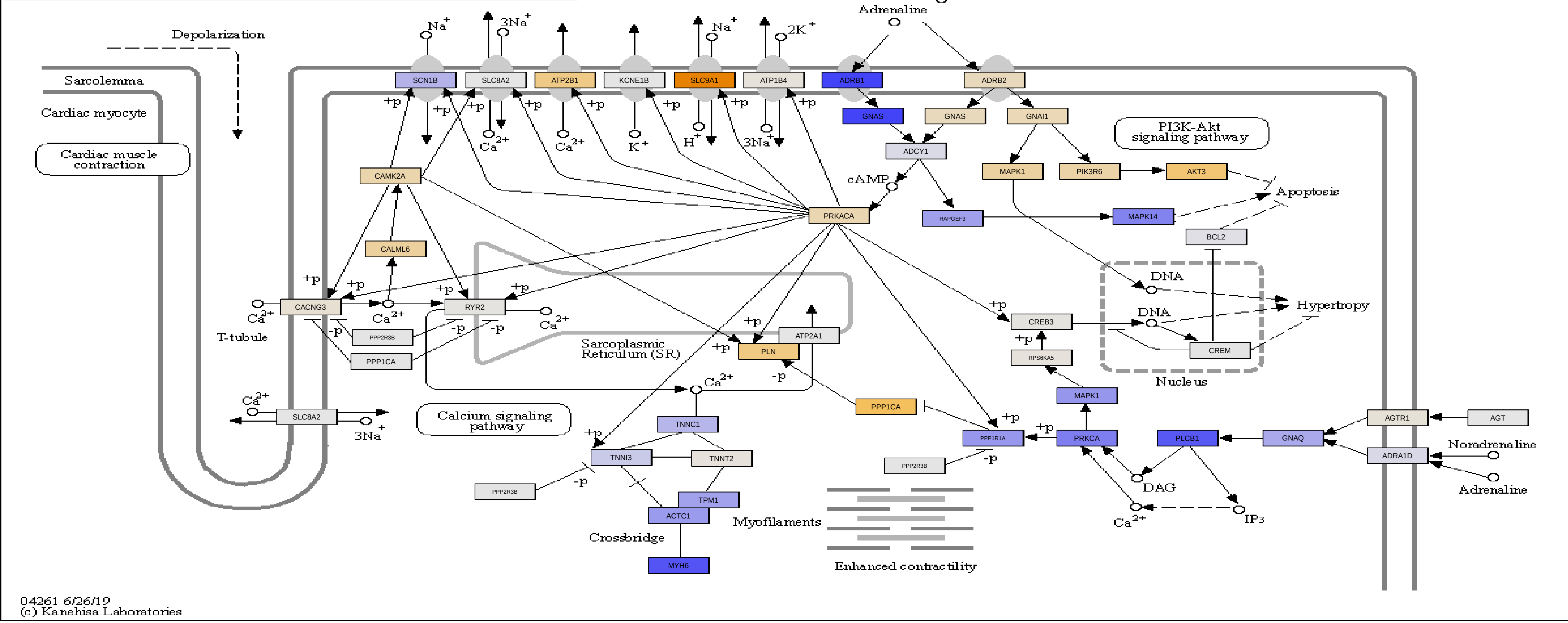

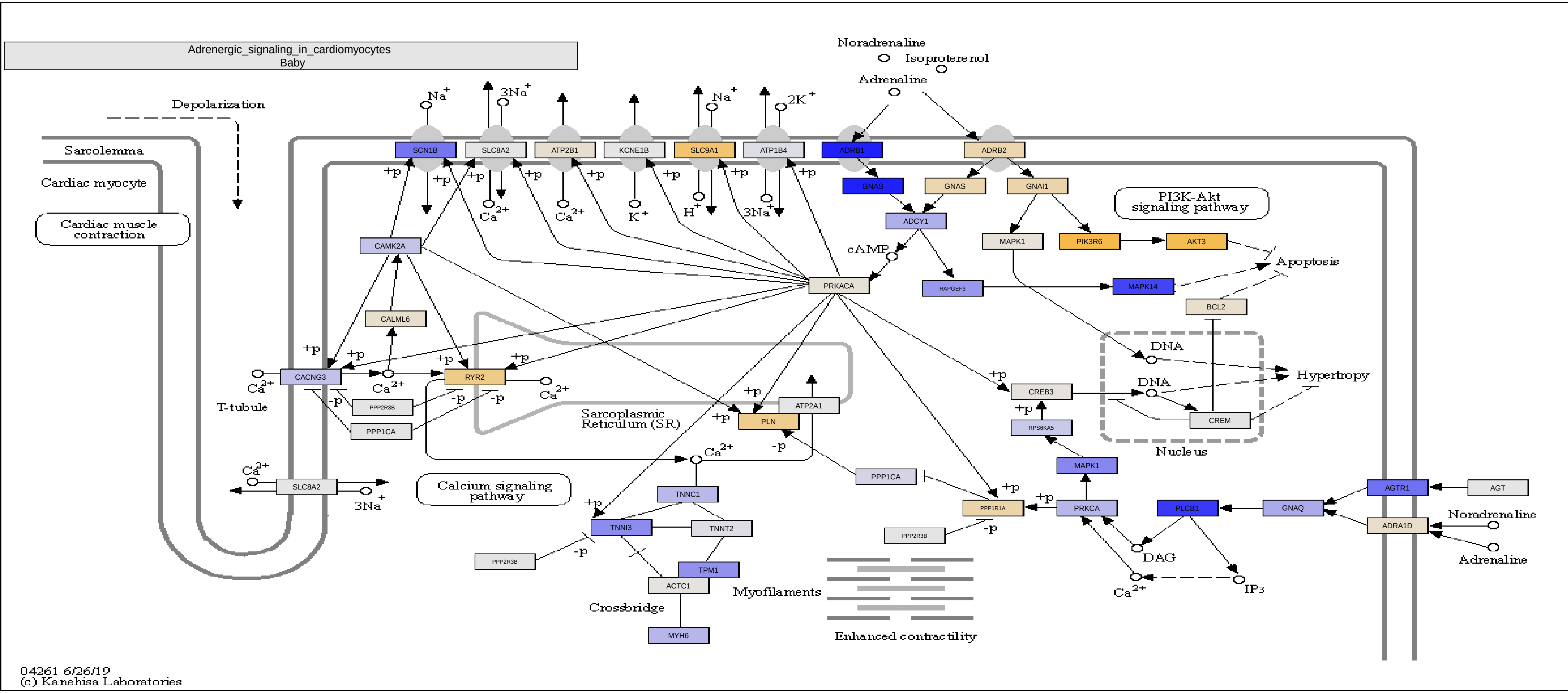

Adrenergic_signaling_in_cardiomyocytes
Baby
Depolarization
Sarcolemma
Cardiac myocyte
Cardiac muscle contraction
Noradrenaline
Isoproterenol
Adrenaline
SCN1B
SLC8A2
ATP2B1
KCNE1B
SLC9A1
ATP1B4
ADRB1
ADRB2
GNAS
GNAS
GNAI1
ADCY1
PI3K-Akt signaling pathway
cAMP
MAPK1
PIK3R6
AKT3
Apoptosis
RAPGEF3
MAPK14
PRKACA
CAMK2A
CALML6
BCL2
DNA
DNA
Hypertropy
CACNG3
RYR2
T-tubule
PPP2R3B
PPP1CA
Sarcoplasmic Reticulum (SR)
ATP2A1
PLN
CREB3
RPS6KA5
CREM
Nucleus
MAPK1
SLC8A2
3Na
Calcium signaling pathway
PPP1CA
PPP1R1A
PRKCA
PLCB1
GNAQ
AGTR1
AGT
ADRA1D
Noradrenaline
Adrenaline
TNNC1
TNNI3
TNNT2
PPP2R3B
PPP2R3B
TPM1
ACTC1
DAG
IP3
Crossbridge
Myofilaments
MYH6
Enhanced contractility
04261 6/26/19
(c) Kanehisa Laboratories

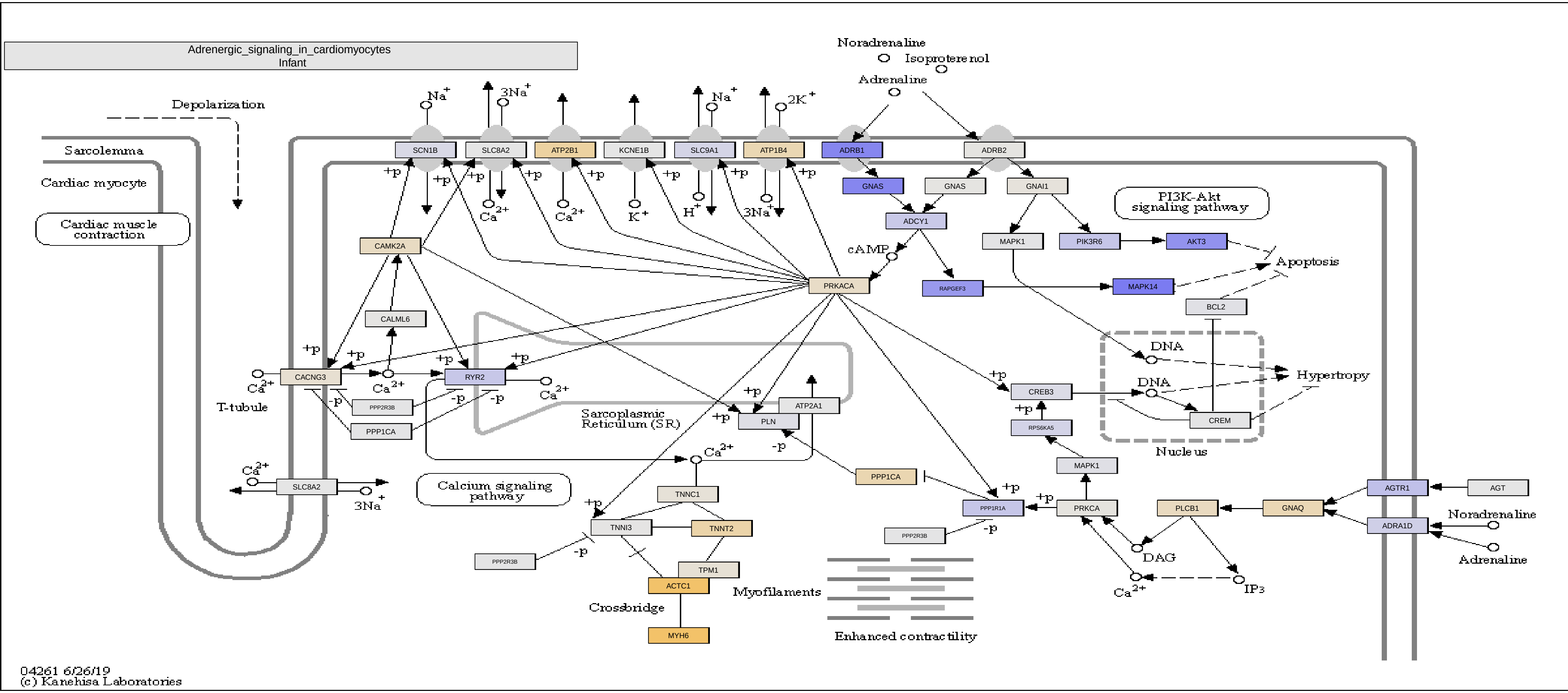

Adrenergic_signaling_in_cardiomyocytes
Infant
Depolarization
Sarcolemma
Cardiac myocyte
Cardiac muscle contraction
Noradrenaline
Isoproterenol
Adrenaline
SCN1B
SLC8A2
ATP2B1
KCNE1B
SLC9A1
ATP1B4
ADRB1
ADRB2
GNAS
GNAS
GNAI1
ADCY1
cAMP
PI3K-Akt signaling pathway
MAPK1
PIK3R6
AKT3
Apoptosis
RAPGEF3
MAPK14
PRKACA
CAMK2A
CALML6
BCL2
DNA
DNA
Hypertropy
CACNG3
RYR2
T-tubule
PPP2R3B
PPP1CA
Sarcoplasmic Reticulum (SR)
ATP2A1
PLN
CREB3
RPS6KA5
CREM
Nucleus
MAPK1
SLC8A2
Calcium signaling pathway
TNNC1
PPP1CA
PPP1R1A
PRKCA
PLCB1
GNAQ
AGTR1
AGT
ADRA1D
Noradrenaline
Adrenaline
TNNI3
TNNT2
PPP2R3B
PPP2R3B
TPM1
ACTC1
MYH6
Crossbridge
Myofilaments
Enhanced contractility
DAG
IP3
04261 6/26/19
(c) Kanehisa Laboratories

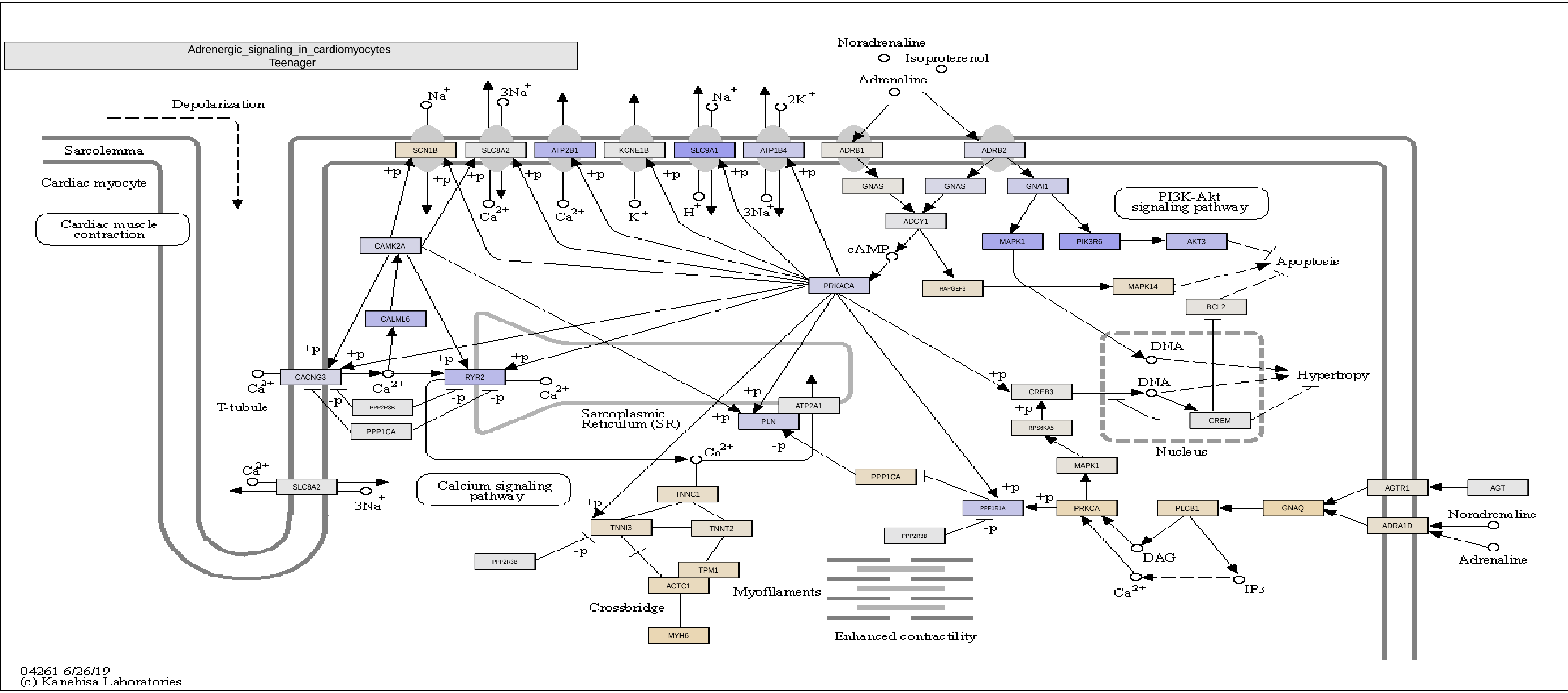
Adrenergic_signaling_in_cardiomyocytes
Teenager
Depolarization
Sarcolemma
Cardiac myocyte
Cardiac muscle contraction
Noradrenaline
Isoproterenol
Adrenaline
SCN1B
SLC8A2
ATP2B1
KCNE1B
SLC9A1
ATP1B4
ADRB1
ADRB2
GNAS
GNAS
GNAI1
ADCY1
PI3K-Akt signaling pathway
cAMP
MAPK1
PIK3R6
AKT3
Apoptosis
PRKACA
RAPGEF3
MAPK14
BCL2
CAMK2A
CALML6
CACNG3
RYR2
T-tubule
PPP2R3B
PPP1CA
Sarcoplasmic Reticulum (SR)
PLN
ATP2A1
DNA
DNA
Hypertropy
CREB3
RPS6KA5
CREM
Nucleus
MAPK1
SLC8A2
Calcium signaling pathway
TNNC1
PPP1CA
PPP1R1A
PRKCA
PLCB1
GNAQ
AGTR1
AGT
ADRA1D
Noradrenaline
Adrenaline
TNNI3
TNNT2
PPP2R3B
PPP2R3B
TPM1
ACTC1
DAG
IP3
Crossbridge
Myofilaments
MYH6
Enhanced contractility
04261 6/26/19
(c) Kanehisa Laboratories

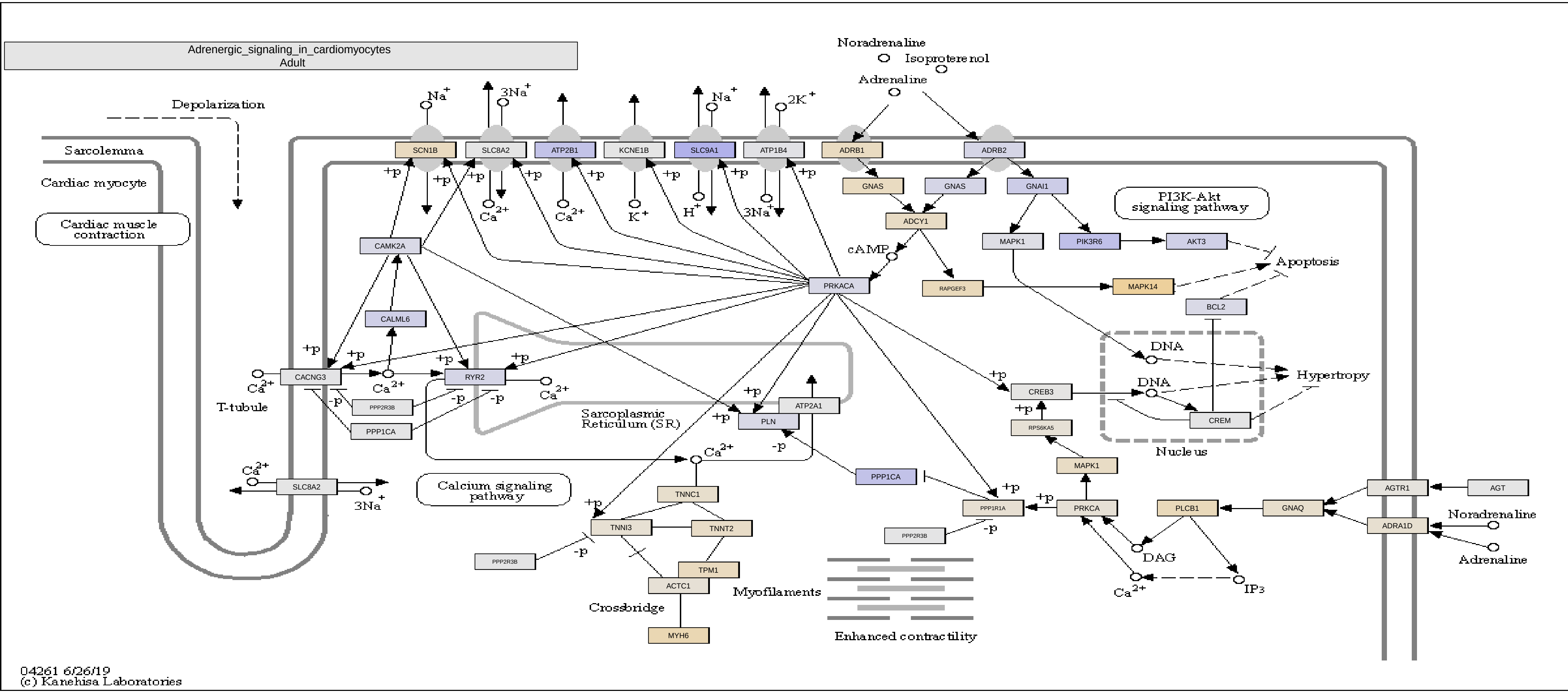
Adrenergic_signaling_in_cardiomyocytes
Adult
Depolarization
Sarcolemma
Cardiac myocyte
Cardiac muscle contraction
Noradrenaline
Isoproterenol
Adrenaline
Na+
3Na+
Na+
2K+
SCN1B
SLC8A2
ATP2B1
KCNE1B
SLC9A1
ATP1B4
ADRB1
ADRB2
+p
Ca2+
K+
H+
3Na+
GNAS
GNAS
GNAI1
ADCY1
PI3K-Akt signaling pathway
MAPK1
PIK3R6
AKT3
cAMP
RAPGEF3
MAPK14
Apoptosis
CAMK2A
PRKACA
BCL2
CALML6
DNA
Hypertropy
CACNG3
RYR2
CREB3
T-tubule
-p
PPP2R3B
PPP1CA
Sarcoplasmic Reticulum (SR)
ATP2A1
PLN
RPS6KA5
CREM
Nucleus
SLC8A2
3Na+
Calcium signaling pathway
PPP1CA
MAPK1
AGTR1
AGT
TNNC1
PPP1R1A
PRKCA
PLCB1
GNAQ
ADRA1D
Noradrenaline
Adrenaline
TNNI3
TNNT2
PPP2R3B
PPP2R3B
DAG
TPM1
ACTC1
Myofilaments
IP3
Crossbridge
MYH6
Enhanced contractility
04261 6/26/19
(c) Kanehisa Laboratories

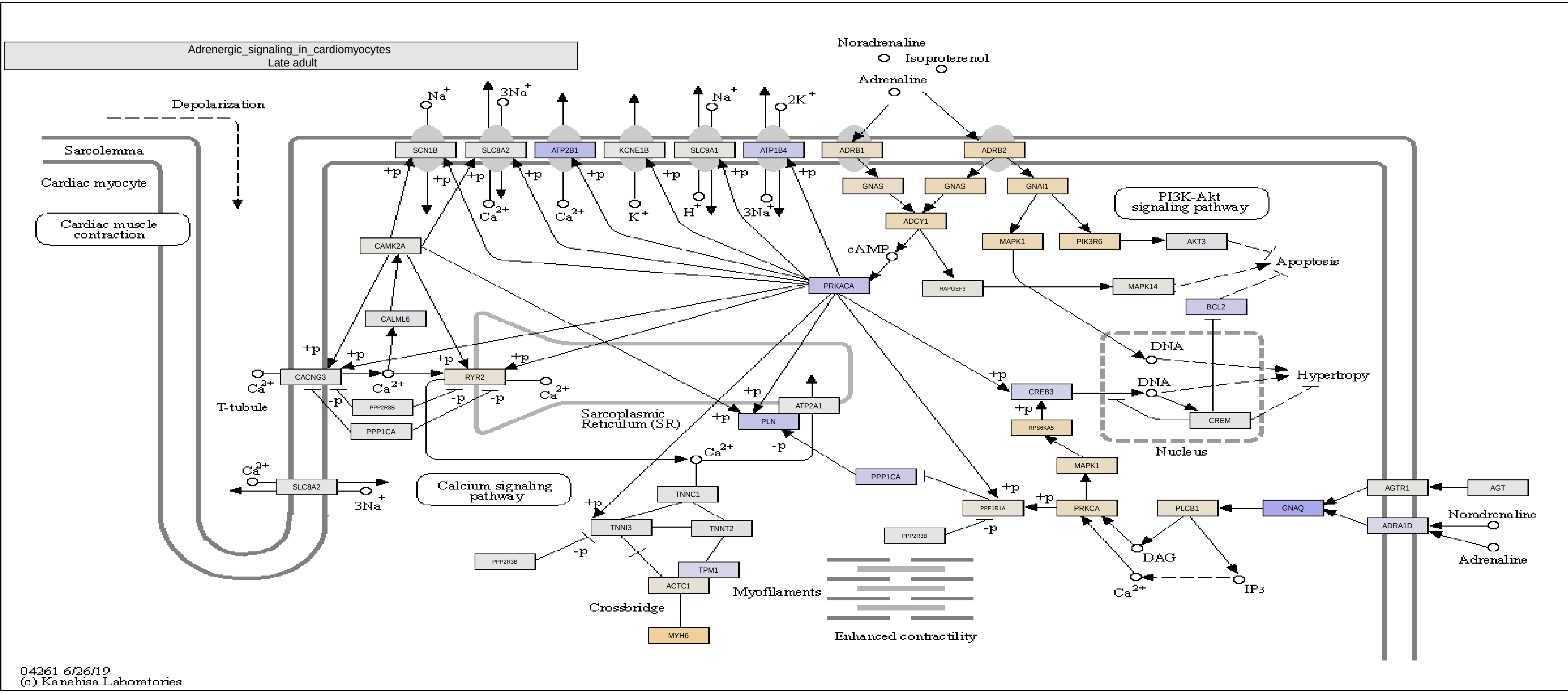
Adrenergic_signaling_in_cardiomyocytes
Late adult
Depolarization
Sarcolemma
Cardiac myocyte
Cardiac muscle contraction
T-tubule
Noradrenaline
Isoproterenol
Adrenaline
SCN1B
SLC8A2
ATP2B1
KCNE1B
SLC9A1
ATP1B4
ADRB1
ADRB2
GNAS
GNAS
GNAI1
ADCY1
cAMP
PRKACA
RAPGEF3
MAPK1
PIK3R6
AKT3
MAPK14
BCL2
PI3K-Akt signaling pathway
Apoptosis
CAMK2A
CALML6
CACNG3
RYR2
PPP2R3B
PPP1CA
Sarcoplasmic Reticulum (SR)
ATP2A1
PLN
PPP1CA
Calcium signaling pathway
TNNC1
TNNI3
TNNT2
TPM1
ACTC1
MYH6
PPP2R3B
Crossbridge
Myofilaments
Enhanced contractility
CREB3
RPS6KA5
DNA
DNA
CREM
Hypertropy
Nucleus
MAPK1
PRKCA
PPP1R1A
PPP2R3B
PLCB1
GNAQ
AGTR1
AGT
ADRA1D
Noradrenaline
Adrenaline
DAG
IP3
Ca2+
K+
H+
Na+
3Na+
2K+
+p
-p
04261 6/26/19
(c) Kanehisa Laboratories
