# Supplementary material for: The Transcriptome and Methylome of the Developing and Aging Brain and Their Relations to Gliomas and Psychological Disorders
Source: Cells. 2022 Jan 21;11(3):362. doi: 10.3390/cells11030362 (PMC8834030; doi:10.3390/cells11030362)
Supplement: Supplementary file 1 [file cells-11-00362-s001.zip › Suppl-Material-S4-Pathways-PSF_Methylation/Apelin_signaling_pathway.pdf]

Apelin signaling pathway

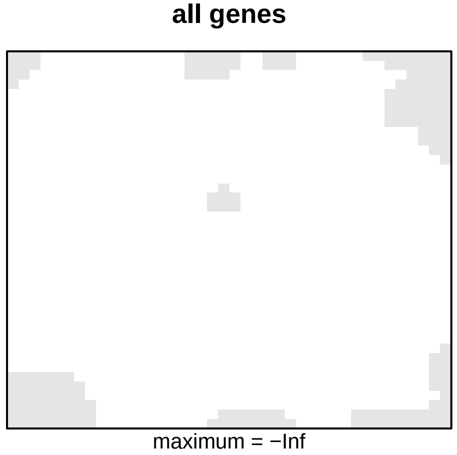

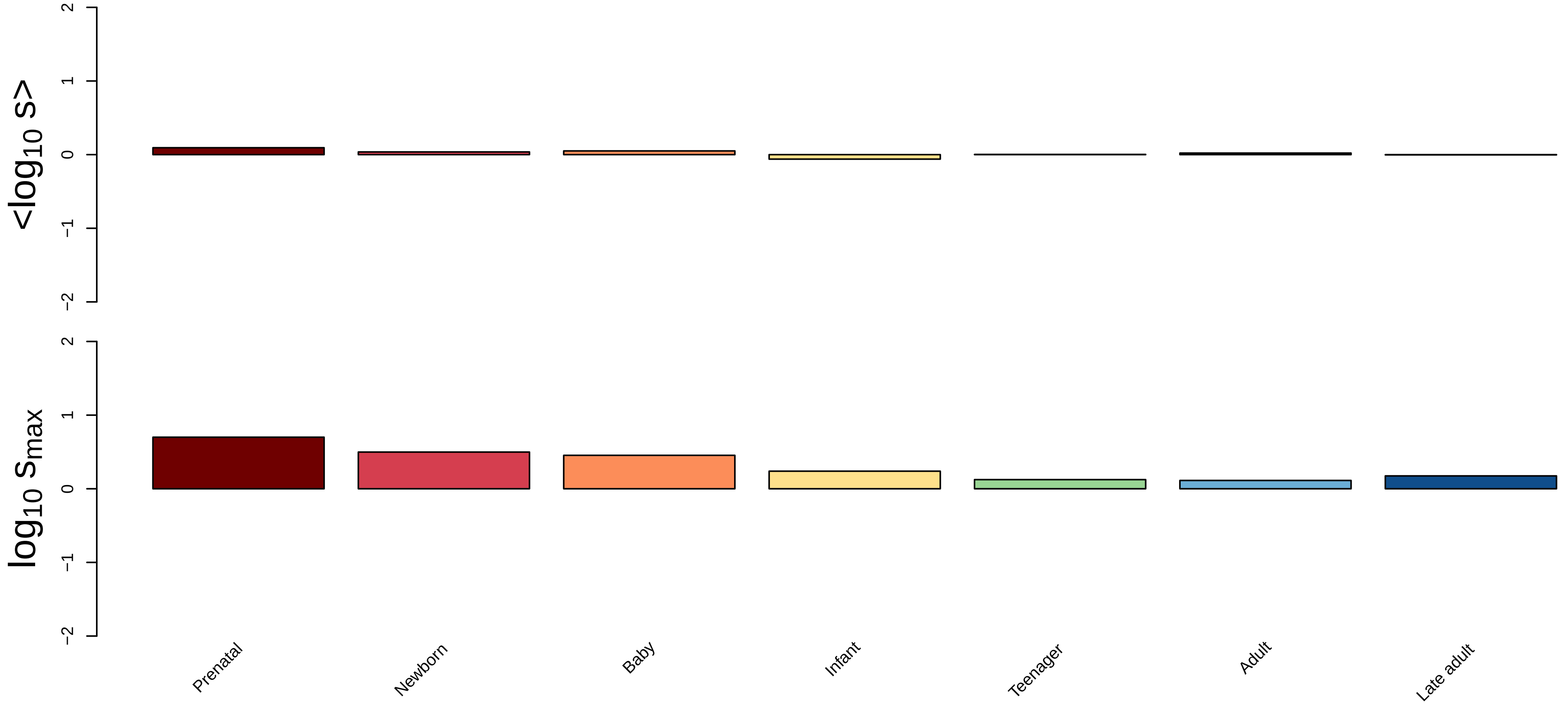

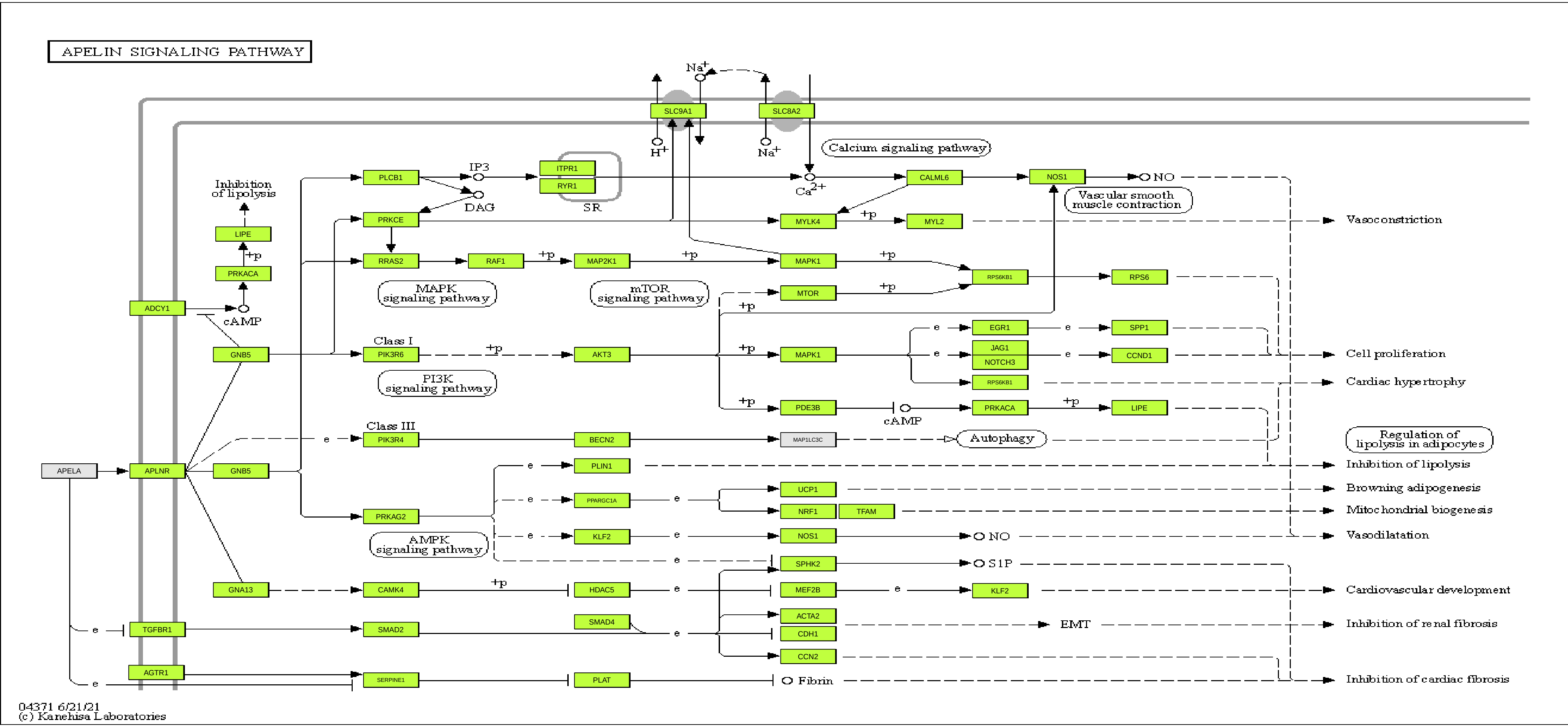
APELIN SIGNALING PATHWAY
Na+
SLC9A1
SLC8A2
H+
Na+
Calcium signaling pathway
IP3
ITPR1
RYR1
SR
Ca2+
CALML6
NOS1
NO
Vascular smooth muscle contraction
PLCB1
DAG
PRKCE
MYLK4
+p
MYL2
Vasoconstriction
Inhibition of lipolysis
LIPE
+p
PRKACA
RRAS2
RAF1
+p
MAP2K1
+p
MAPK1
+p
RPS6KB1
RPS6
MAPK signaling pathway
mTOR signaling pathway
MTOR
+p
ADCY1
cAMP
+p
EGR1
e
SPP1
Class I
JAG1
NOTCH3
e
CCND1
Cell proliferation
GNB5
PIK3R6
+p
AKT3
+p
MAPK1
e
RPS6KB1
Cardiac hypertrophy
PI3K signaling pathway
+p
PDE3B
cAMP
PRKACA
+p
LIPE
Class III
PIK3R4
BECN2
MAP1LC3C
Autophagy
Regulation of lipolysis in adipocytes
e
APELA
APLNR
GNB5
e
PLIN1
Inhibition of lipolysis
e
PPARGC1A
e
UCP1
Browning adipogenesis
NRF1
TFAM
Mitochondrial biogenesis
PRKAG2
AMPK signaling pathway
e
KLF2
e
NOS1
NO
Vasodilatation
e
SPHK2
S1P
GNA13
CAMK4
+p
HDAC5
e
MEF2B
e
KLF2
Cardiovascular development
SMAD4
ACTA2
EMT
e
TGFBR1
SMAD2
e
CDH1
Inhibition of renal fibrosis
CCN2
AGTR1
e
SERPINE1
PLAT
Fibrin
Inhibition of cardiac fibrosis
04371 6/21/21
(c) Kanehisa Laboratories

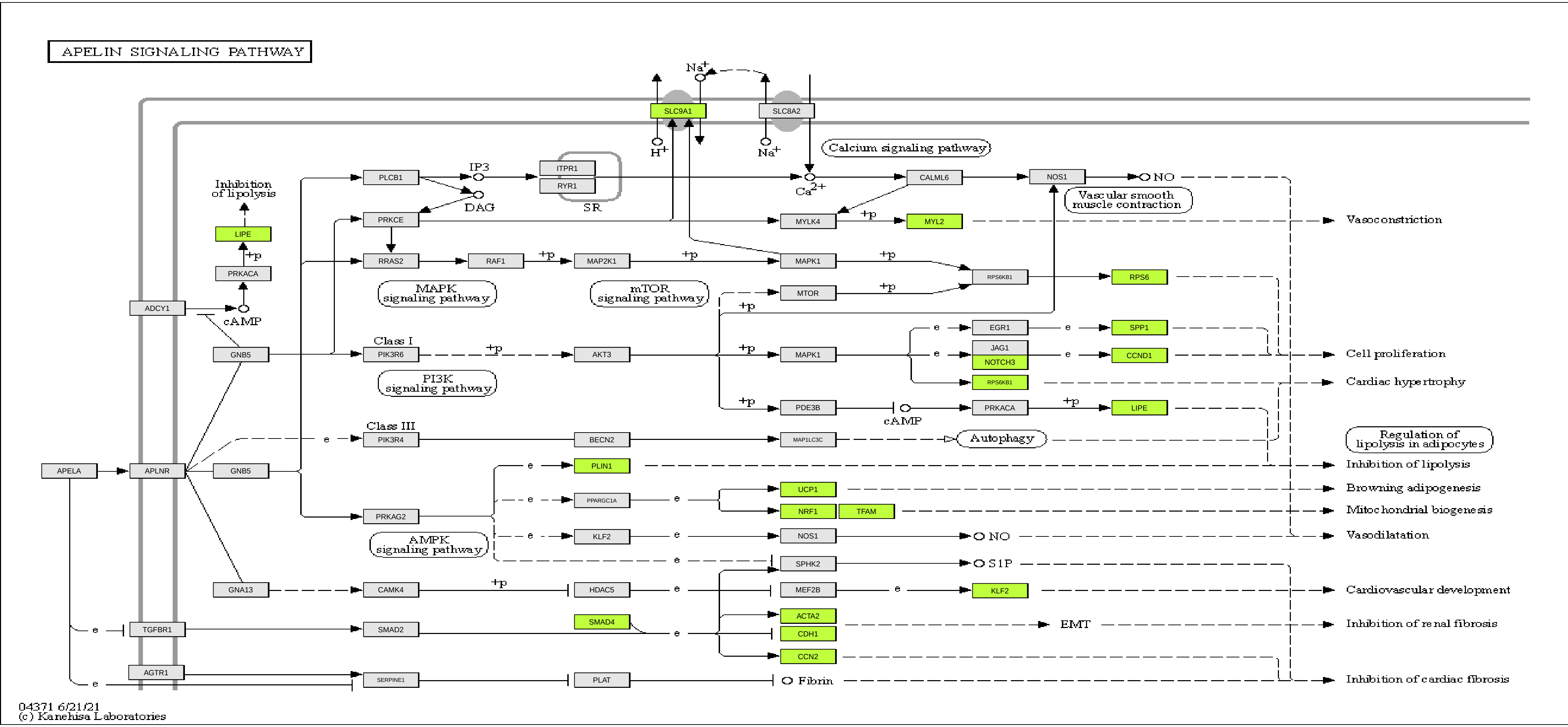
APELIN SIGNALING PATHWAY
Na+
SLC9A1
SLC8A2
H+
Na+
Calcium signaling pathway
Inhibition of lipolysis
PLCB1
IP3
ITPR1
RYR1
SR
DAG
Ca2+
CALML6
NOS1
NO
Vascular smooth muscle contraction
LIPE
PRKCE
MYLK4
+p
MYL2
Vasoconstriction
PRKACA
RRAS2
RAF1
MAP2K1
MAPK1
RPS6KB1
RPS6
ADCY1
cAMP
MAPK signaling pathway
mTOR signaling pathway
MTOR
GNB5
Class I
PIK3R6
AKT3
EGR1
SPP1
JAG1
NOTCH3
CCND1
Cell proliferation
Cardiac hypertrophy
PI3K signaling pathway
PDE3B
Class III
PIK3R4
BECN2
MAP1LC3C
Autophagy
Regulation of lipolysis in adipocytes
APELA
APLNR
PLIN1
Inhibition of lipolysis
PPARGC1A
UCP1
Browning adipogenesis
NRF1
TFAM
Mitochondrial biogenesis
PRKAG2
AMPK signaling pathway
KLF2
NOS1
Vasodilatation
SPHK2
S1P
GNA13
CAMK4
HDAC5
MEF2B
Cardiovascular development
TGFBR1
SMAD2
SMAD4
ACTA2
CDH1
EMT
Inhibition of renal fibrosis
CCN2
AGTR1
SERPINE1
PLAT
Fibrin
Inhibition of cardiac fibrosis
04371 6/21/21
(c) Kanehisa Laboratories

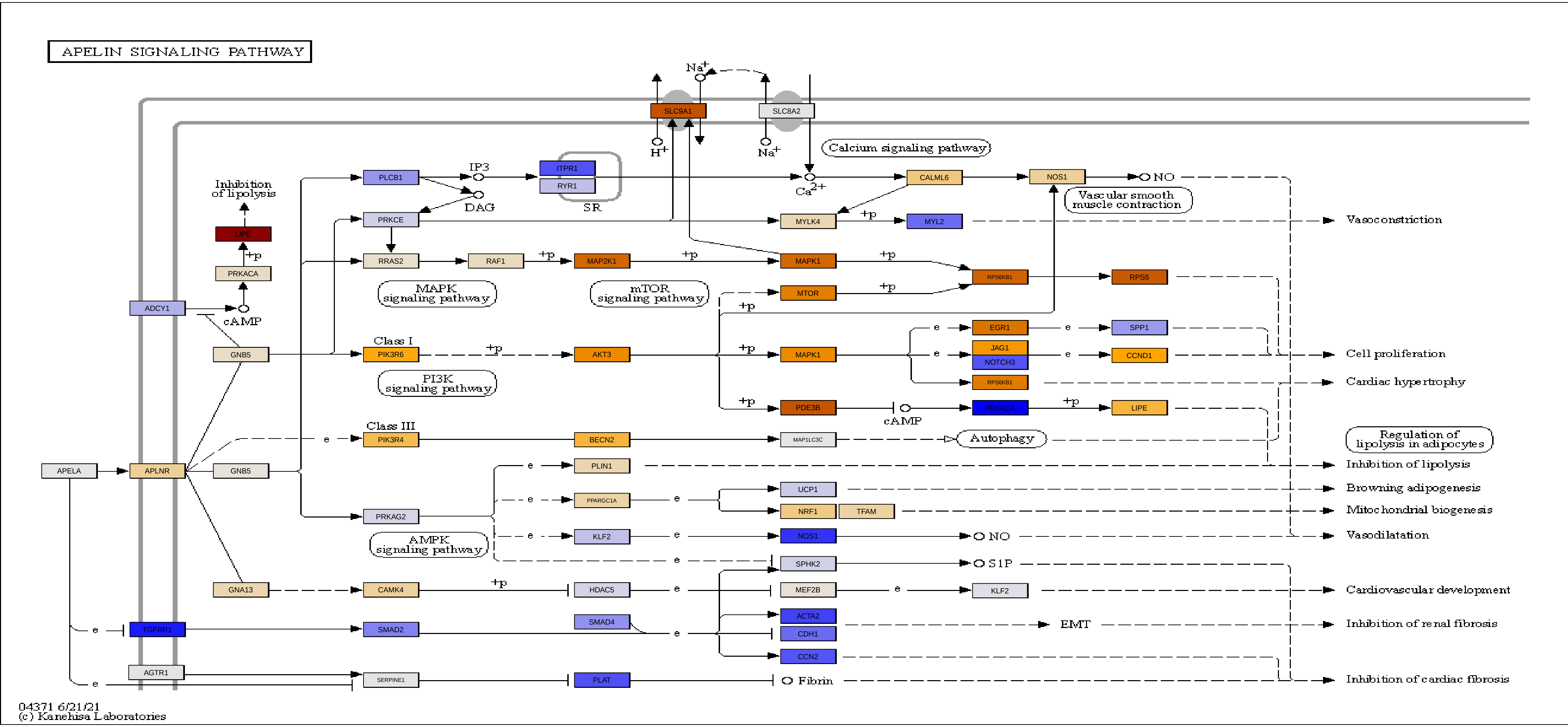
APELIN SIGNALING PATHWAY
Na+
H+
Na+
SLC9A1
SLC8A2
Calcium signaling pathway
Inhibition of lipolysis
LIPE
+p
PRKACA
ADCY1
cAMP
PLCB1
IP3
DAG
ITPR1
RYR1
SR
Ca2+
CALML6
NOS1
NO
Vascular smooth muscle contraction
PRKCE
MYLK4
+p
MYL2
Vasoconstriction
RRAS2
RAF1
+p
MAP2K1
+p
MAPK1
+p
RPS6KB1
RPS6
MAPK signaling pathway
mTOR signaling pathway
MTOR
+p
+p
GNB5
Class I
PIK3R6
+p
AKT3
+p
MAPK1
e
EGR1
e
SPP1
JAG1
NOTCH3
e
CCND1
RPS6KB1
Cell proliferation
Cardiac hypertrophy
PI3K signaling pathway
+p
PDE3B
cAMP
PRKACA
+p
LIPE
Class III
PIK3R4
e
BECN2
MAP1LC3C
Autophagy
Regulation of lipolysis in adipocytes
APELA
APLNR
GNB5
e
PLIN1
Inhibition of lipolysis
PPARGC1A
e
UCP1
Browning adipogenesis
NRF1
TFAM
Mitochondrial biogenesis
PRKAG2
AMPK signaling pathway
e
KLF2
e
NOS1
NO
Vasodilatation
e
SPHK2
S1P
GNA13
CAMK4
+p
HDAC5
e
MEF2B
e
KLF2
Cardiovascular development
e
TGFBR1
SMAD2
SMAD4
e
ACTA2
CDH1
EMT
Inhibition of renal fibrosis
CCN2
AGTR1
e
SERPINE1
PLAT
Fibrin
Inhibition of cardiac fibrosis
04371 6/21/21
(c) Kanehisa Laboratories

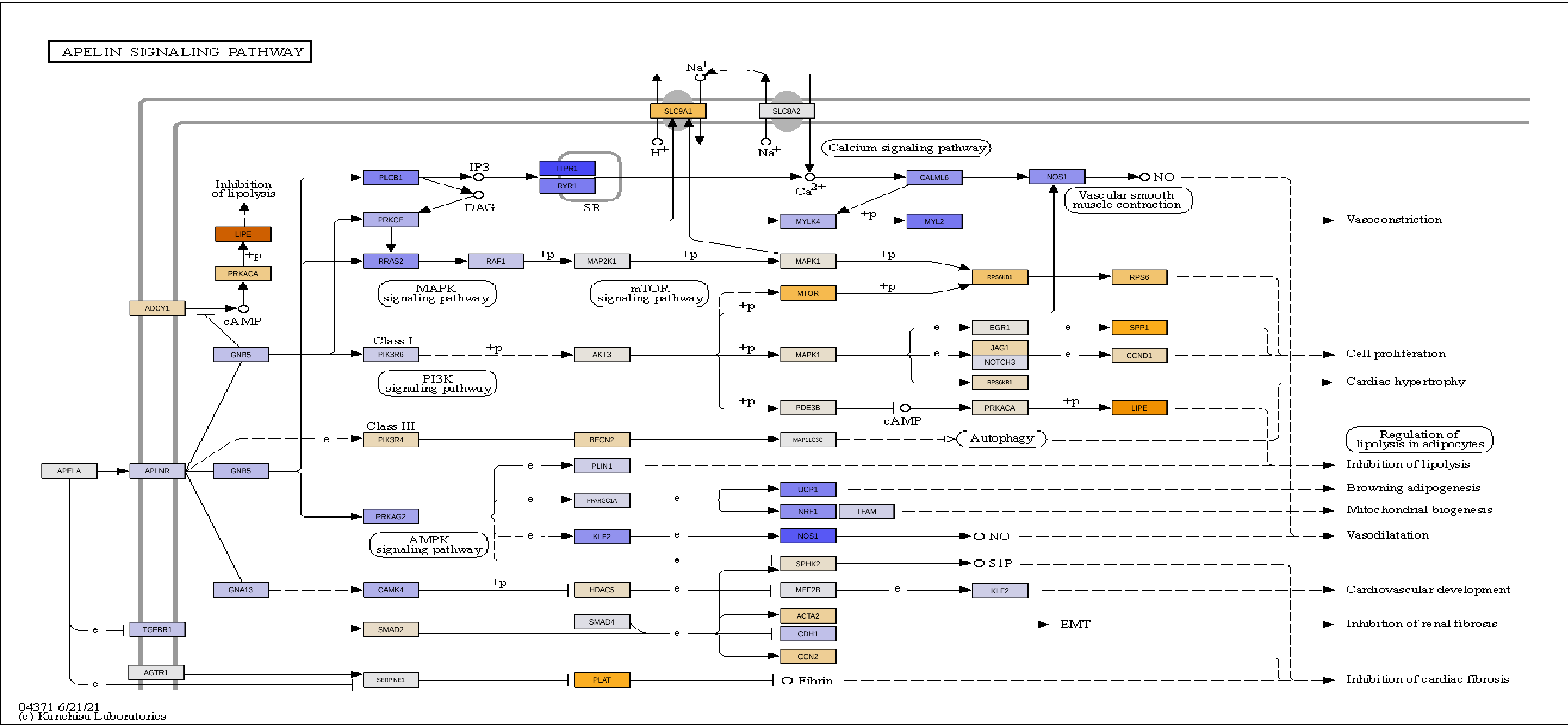
APELIN SIGNALING PATHWAY
Na+
SLC9A1
SLC8A2
H+
Na+
Calcium signaling pathway
PLCB1
IP3
ITPR1
RYR1
SR
DAG
Ca2+
CALML6
NOS1
NO
Vascular smooth muscle contraction
Inhibition of lipolysis
LIPE
+p
PRKACA
PRKCE
MYLK4
+p
MYL2
Vasoconstriction
RRAS2
RAF1
+p
MAP2K1
+p
MAPK1
+p
RPS6KB1
RPS6
MAPK signaling pathway
mTOR signaling pathway
MTOR
+p
ADCY1
cAMP
+p
EGR1
e
SPP1
GNB5
Class I
PIK3R6
+p
AKT3
+p
MAPK1
e
JAG1
NOTCH3
e
CCND1
Cell proliferation
RPS6KB1
Cardiac hypertrophy
PI3K signaling pathway
+p
PDE3B
cAMP
PRKACA
+p
LIPE
Class III
e
PIK3R4
BECN2
MAP1LC3C
Autophagy
Regulation of lipolysis in adipocytes
APELA
APLNR
GNB5
e
PLIN1
Inhibition of lipolysis
e
PPARGC1A
e
UCP1
Browning adipogenesis
NRF1
TFAM
Mitochondrial biogenesis
PRKAG2
AMPK signaling pathway
e
KLF2
e
NOS1
NO
Vasodilatation
e
SPHK2
S1P
GNA13
CAMK4
+p
HDAC5
e
MEF2B
e
KLF2
Cardiovascular development
ACTA2
EMT
Inhibition of renal fibrosis
e
TGFBR1
SMAD2
SMAD4
e
CDH1
CCN2
AGTR1
e
SERPINE1
PLAT
Fibrin
Inhibition of cardiac fibrosis
04371 6/21/21
(c) Kanehisa Laboratories

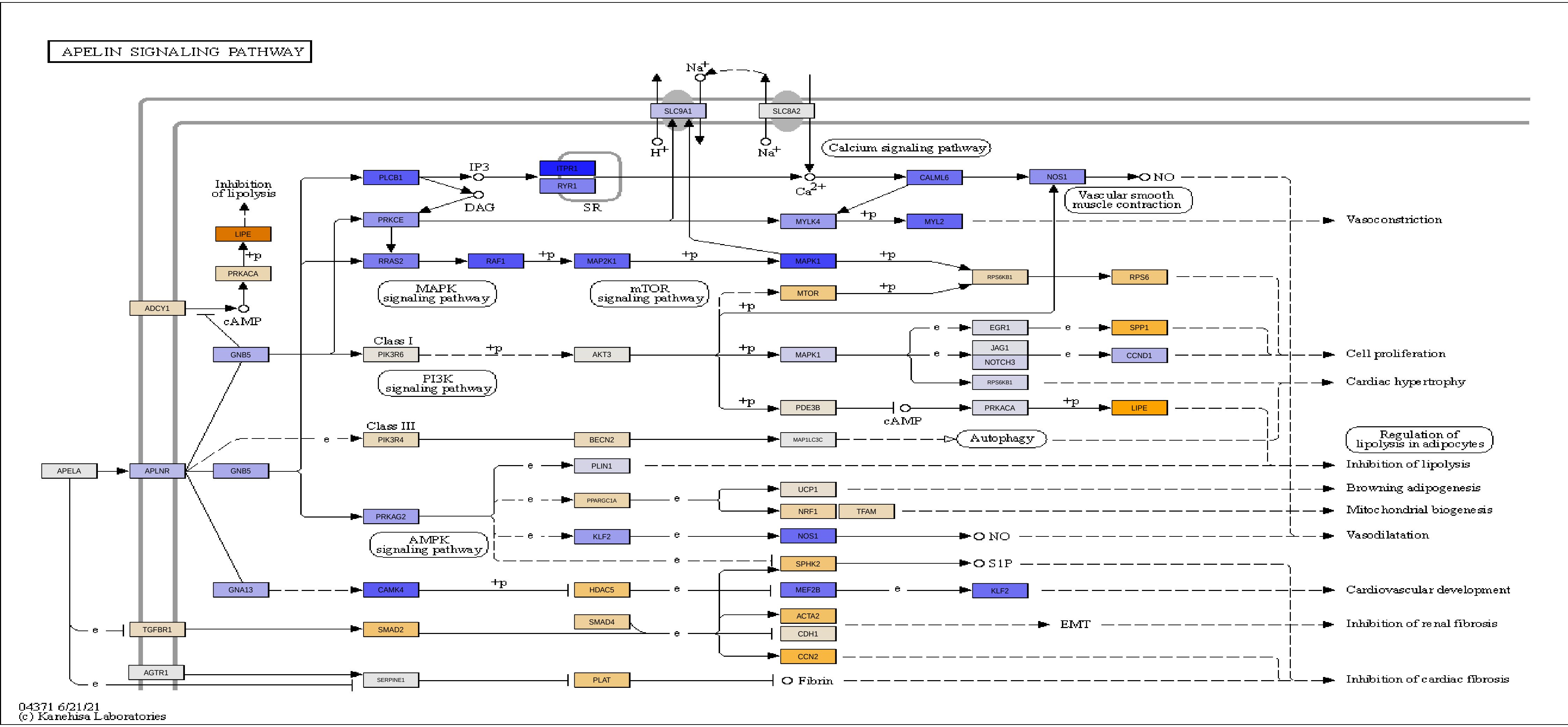

APELIN SIGNALING PATHWAY
Na+
SLC9A1
SLC8A2
H+
Na+
Calcium signaling pathway
Inhibition of lipolysis
PLCB1
IP3
ITPR1
RYR1
SR
DAG
Ca2+
CALML6
NOS1
NO
Vascular smooth muscle contraction
LIPE
PRKCE
MYLK4
+p
MYL2
Vasoconstriction
PRKACA
+p
RRAS2
RAF1
+p
MAP2K1
+p
MAPK1
+p
RPS6KB1
RPS6
ADCY1
cAMP
MAPK signaling pathway
mTOR signaling pathway
MTOR
+p
+p
e
EGR1
e
SPP1
GNB5
Class I
PIK3R6
+p
AKT3
+p
MAPK1
e
JAG1
NOTCH3
e
CCND1
Cell proliferation
RPS6KB1
Cardiac hypertrophy
PI3K signaling pathway
+p
PDE3B
cAMP
PRKACA
+p
LIPE
Class III
e
PIK3R4
BECN2
MAP1LC3C
Autophagy
Regulation of lipolysis in adipocytes
APELA
APLNR
GNB5
e
PLIN1
Inhibition of lipolysis
e
PPARGC1A
e
UCP1
Browning adipogenesis
PRKAG2
NRF1
TFAM
Mitochondrial biogenesis
AMPK signaling pathway
e
KLF2
e
NOS1
NO
Vasodilatation
e
SPHK2
S1P
GNA13
CAMK4
+p
HDAC5
e
MEF2B
e
KLF2
Cardiovascular development
SMAD4
ACTA2
EMT
Inhibition of renal fibrosis
e
TGFBR1
SMAD2
e
CDH1
CCN2
AGTR1
e
SERPINE1
PLAT
Fibrin
Inhibition of cardiac fibrosis
04371 6/21/21
(c) Kanehisa Laboratories

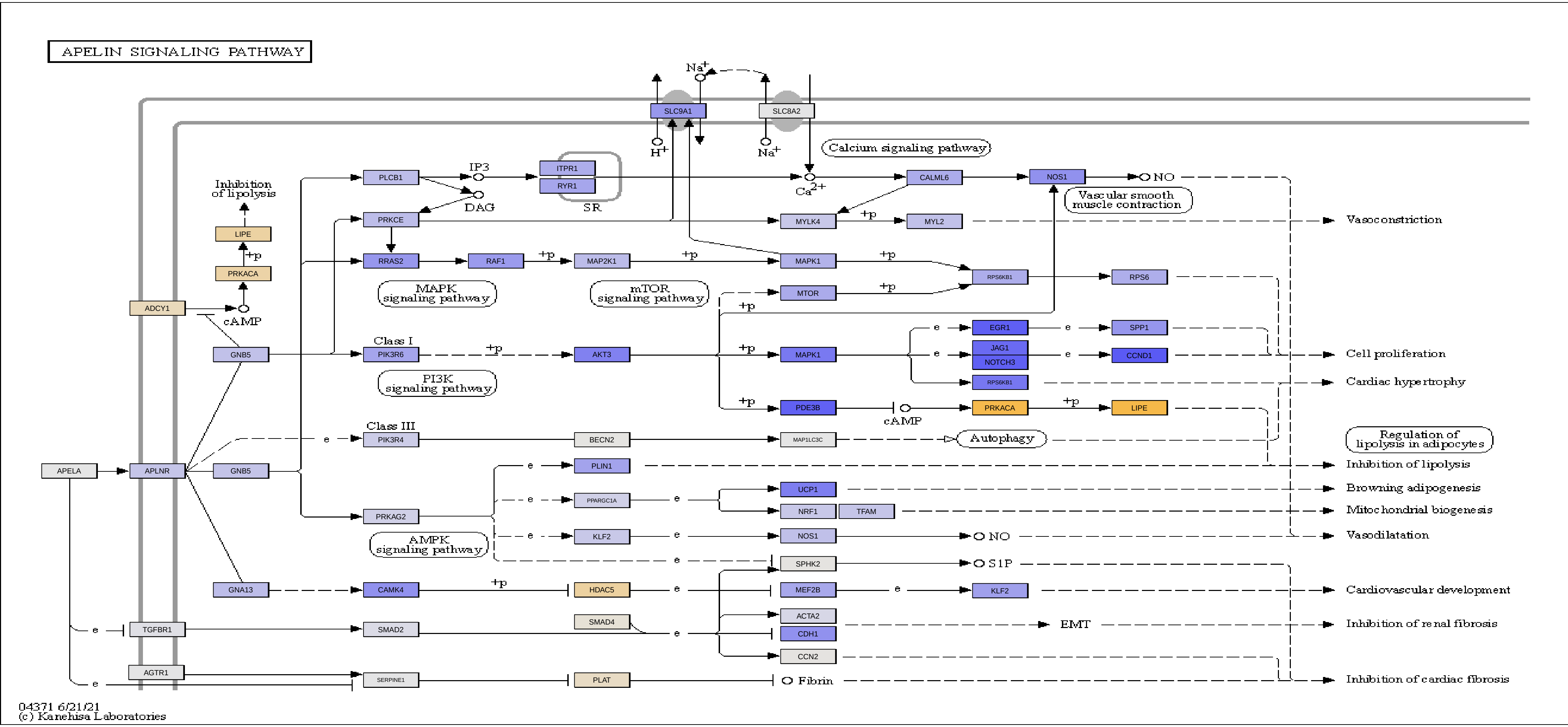
APELIN SIGNALING PATHWAY
Na+
SLC9A1
SLC8A2
H+
Na+
Calcium signaling pathway
PLCB1
IP3
ITPR1
RYR1
SR
DAG
Ca2+
CALML6
NOS1
NO
Vascular smooth muscle contraction
Inhibition of lipolysis
LIPE
+p
PRKACA
ADCY1
cAMP
PRKCE
MYLK4
+p
MYL2
Vasoconstriction
RRAS2
RAF1
+p
MAP2K1
+p
MAPK1
+p
RPS6KB1
RPS6
MAPK signaling pathway
mTOR signaling pathway
MTOR
+p
+p
GNB5
Class I
PIK3R6
+p
AKT3
e
EGR1
e
SPP1
+p
MAPK1
e
JAG1
NOTCH3
e
CCND1
Cell proliferation
RPS6KB1
Cardiac hypertrophy
PI3K signaling pathway
+p
PDE3B
cAMP
PRKACA
+p
LIPE
Class III
e
PIK3R4
BECN2
MAP1LC3C
Autophagy
Regulation of lipolysis in adipocytes
APELA
APLNR
GNB5
e
PLIN1
Inhibition of lipolysis
e
PPARGC1A
e
UCP1
Browning adipogenesis
NRF1
TFAM
Mitochondrial biogenesis
PRKAG2
AMPK signaling pathway
e
KLF2
e
NOS1
NO
Vasodilatation
e
SPHK2
S1P
GNA13
CAMK4
+p
HDAC5
e
MEF2B
e
KLF2
Cardiovascular development
ACTA2
SMAD4
e
TGFBR1
SMAD2
e
CDH1
EMT
Inhibition of renal fibrosis
CCN2
AGTR1
e
SERPINE1
PLAT
Fibrin
Inhibition of cardiac fibrosis
04371 6/21/21
(c) Kanehisa Laboratories

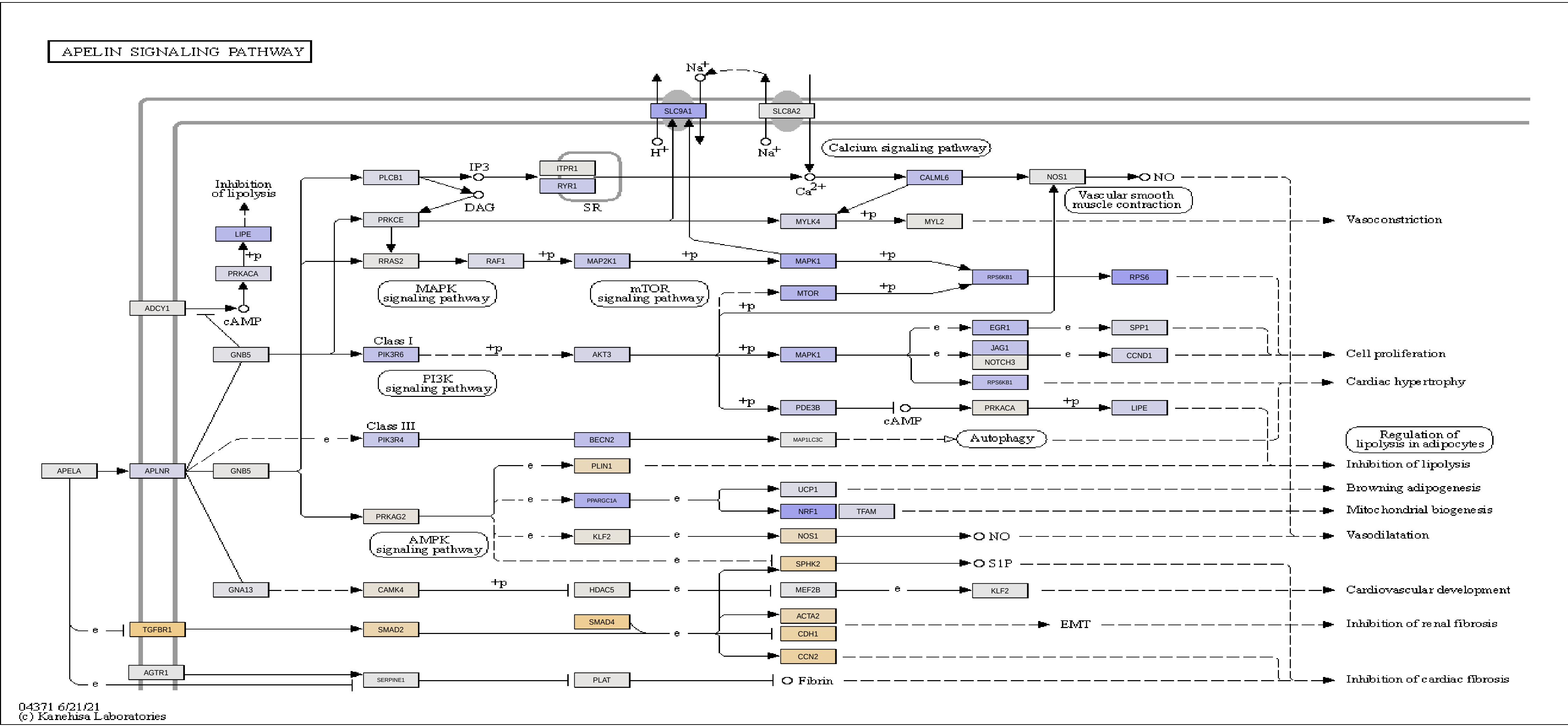
APELIN SIGNALING PATHWAY
Na+
SLC9A1
SLC8A2
H+
Na+
Calcium signaling pathway
PLCB1
IP3
ITPR1
RYR1
SR
Ca2+
CALML6
NOS1
NO
Vascular smooth muscle contraction
DAG
Inhibition of lipolysis
PRKCE
MYLK4
+p
MYL2
Vasoconstriction
LIPE
+p
PRKACA
RRAS2
RAF1
MAP2K1
MAPK1
RPS6KB1
RPS6
MAPK signaling pathway
mTOR signaling pathway
MTOR
ADCY1
cAMP
Class I
GNB5
PIK3R6
AKT3
EGR1
SPP1
JAG1
NOTCH3
CCND1
Cell proliferation
Cardiac hypertrophy
PI3K signaling pathway
PDE3B
cAMP
LIPE
Class III
PIK3R4
BECN2
MAP1LC3C
Autophagy
Regulation of lipolysis in adipocytes
APELA
APLNR
GNB5
PLIN1
Inhibition of lipolysis
PPARGC1A
UCP1
Browning adipogenesis
NRF1
TFAM
Mitochondrial biogenesis
PRKAG2
AMPK signaling pathway
KLF2
NOS1
NO
Vasodilatation
SPHK2
S1P
GNA13
CAMK4
HDAC5
MEF2B
KLF2
Cardiovascular development
SMAD4
ACTA2
EMT
Inhibition of renal fibrosis
TGFBR1
SMAD2
CDH1
CCN2
AGTR1
SERPINE1
PLAT
Fibrin
Inhibition of cardiac fibrosis
04371 6/21/21
(c) Kanehisa Laboratories

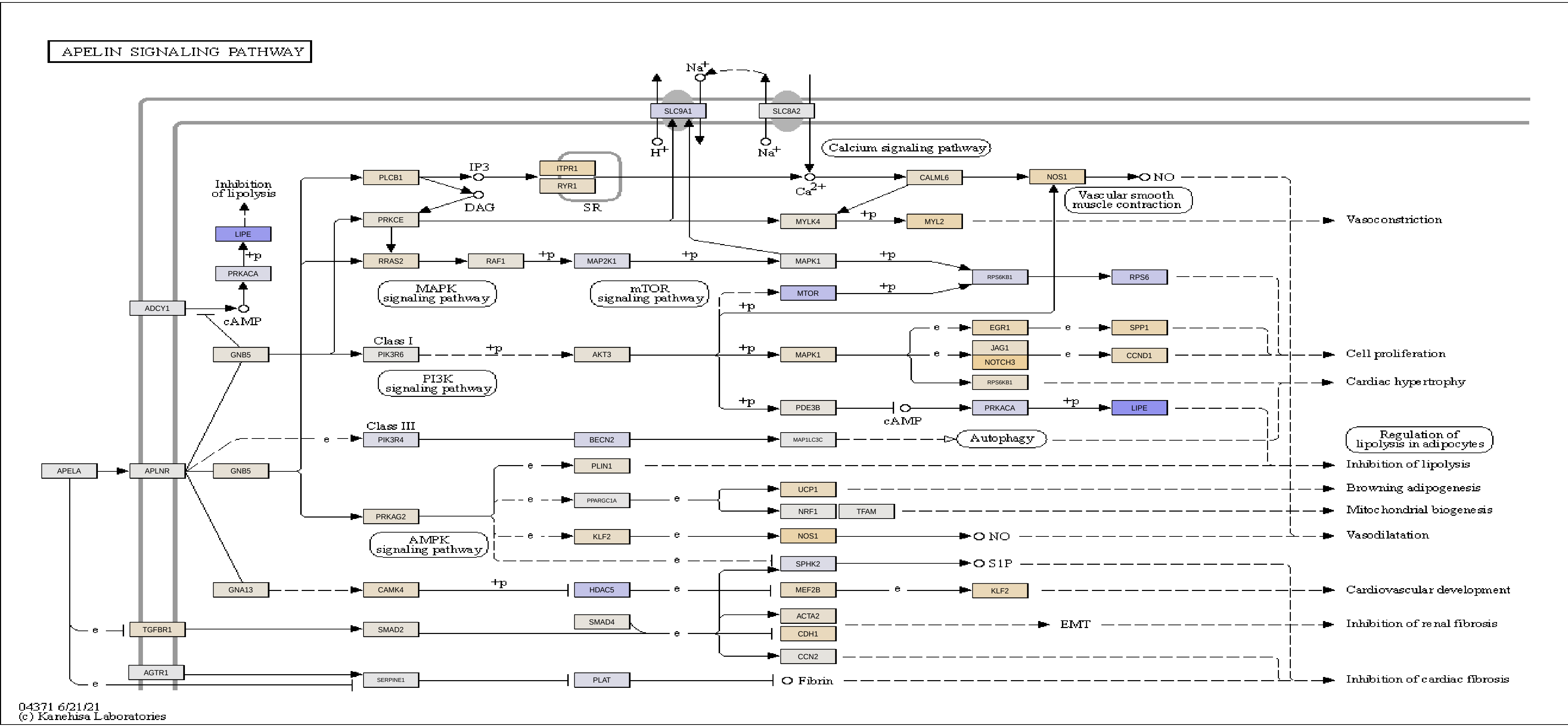
APELIN SIGNALING PATHWAY
Na+
SLC9A1
SLC8A2
H+
Na+
Calcium signaling pathway
PLCB1
IP3
ITPR1
RYR1
SR
DAG
Ca2+
CALML6
NOS1
NO
Vascular smooth muscle contraction
Inhibition of lipolysis
LIPE
+p
PRKACA
PRKCE
MYLK4
MYL2
Vasoconstriction
RRAS2
RAF1
MAP2K1
MAPK1
RPS6KB1
RPS6
MAPK signaling pathway
mTOR signaling pathway
MTOR
ADCY1
cAMP
GNB5
Class I
PIK3R6
AKT3
EGR1
SPP1
JAG1
NOTCH3
CCND1
Cell proliferation
Cardiac hypertrophy
PI3K signaling pathway
PDE3B
LIPE
Class III
PIK3R4
BECN2
MAP1LC3C
Autophagy
Regulation of lipolysis in adipocytes
APELA
APLNR
PLIN1
Inhibition of lipolysis
PPARGC1A
UCP1
Browning adipogenesis
NRF1
TFAM
Mitochondrial biogenesis
PRKAG2
AMPK signaling pathway
KLF2
Vasodilatation
SPHK2
S1P
GNA13
CAMK4
HDAC5
MEF2B
Cardiovascular development
ACTA2
EMT
Inhibition of renal fibrosis
CDH1
TGFBR1
SMAD2
SMAD4
CCN2
AGTR1
SERPINE1
PLAT
Fibrin
Inhibition of cardiac fibrosis
e
04371 6/21/21
(c) Kanehisa Laboratories

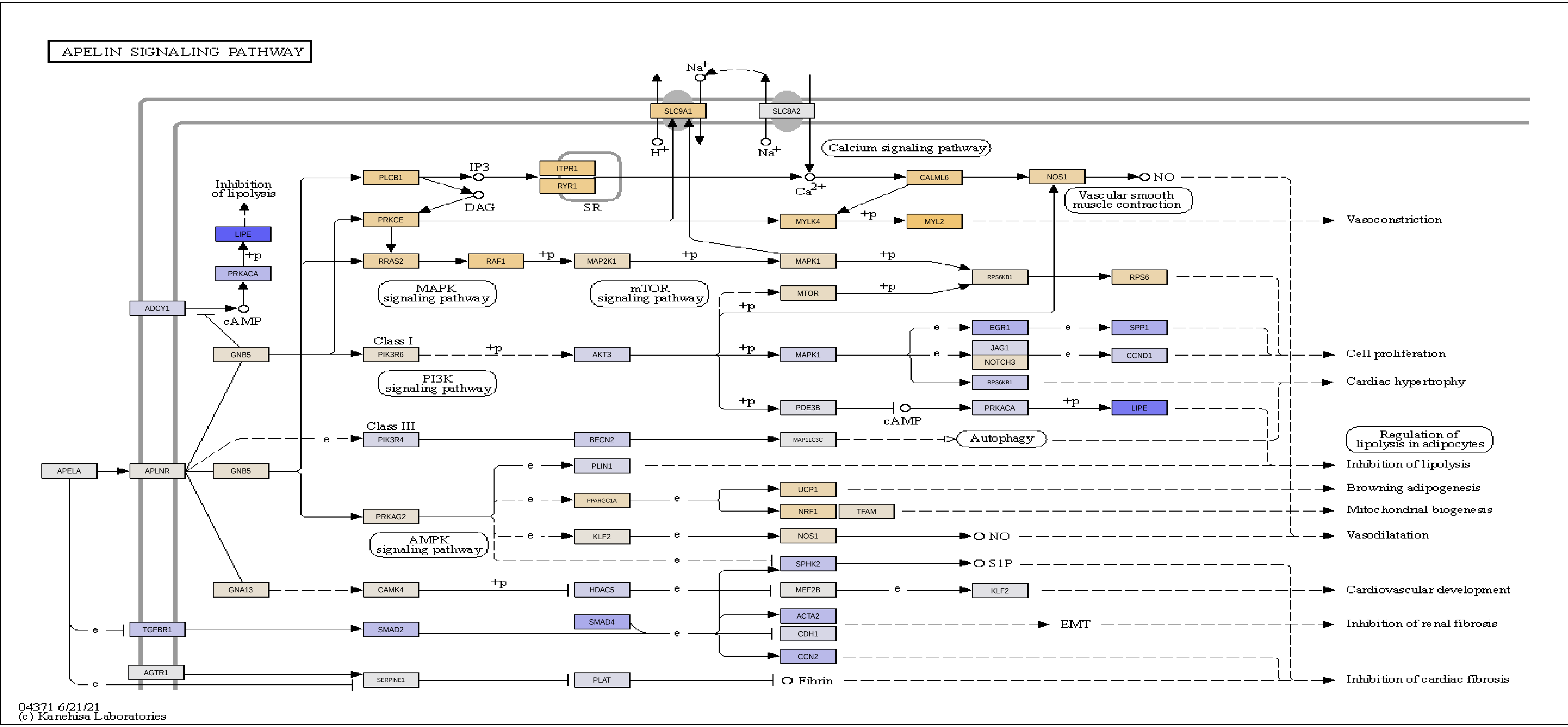
APELIN SIGNALING PATHWAY
APELA
APLNR
ADCY1
cAMP
PRKACA
LIPE
Inhibition of lipolysis
GNB5
GNA13
TGFBR1
AGTR1
PLCB1
IP3
DAG
ITPR1
RYR1
SR
PRKCE
RRAS2
RAF1
MAP2K1
MAPK signaling pathway
mTOR signaling pathway
Class I
PIK3R6
AKT3
PI3K signaling pathway
Class III
PIK3R4
BECN2
PRKAG2
AMPK signaling pathway
CAMK4
HDAC5
SMAD2
SMAD4
SERPINE1
PLAT
SLC9A1
SLC8A2
Na+
H+
Calcium signaling pathway
Ca^{2+}
CALML6
NOS1
NO
Vascular smooth muscle contraction
MYLK4
MYL2
MAPK1
RPS6KB1
RPS6
MTOR
EGR1
SPP1
JAG1
NOTCH3
CCND1
PDE3B
LIPE
MAP1LC3C
Autophagy
PLIN1
PPARGC1A
UCP1
NRF1
TFAM
KLF2
S1P
SPHK2
MEF2B
ACTA2
CDH1
CCN2
EMT
Fibrin
Vasoconstriction
Cell proliferation
Cardiac hypertrophy
Regulation of lipolysis in adipocytes
Inhibition of lipolysis
Browning adipogenesis
Mitochondrial biogenesis
Vasodilatation
Cardiovascular development
Inhibition of renal fibrosis
Inhibition of cardiac fibrosis
04371 6/21/21
(c) Kanehisa Laboratories
