# Supplementary material for: The Transcriptome and Methylome of the Developing and Aging Brain and Their Relations to Gliomas and Psychological Disorders
Source: Cells. 2022 Jan 21;11(3):362. doi: 10.3390/cells11030362 (PMC8834030; doi:10.3390/cells11030362)

C−type lectin receptor signaling path

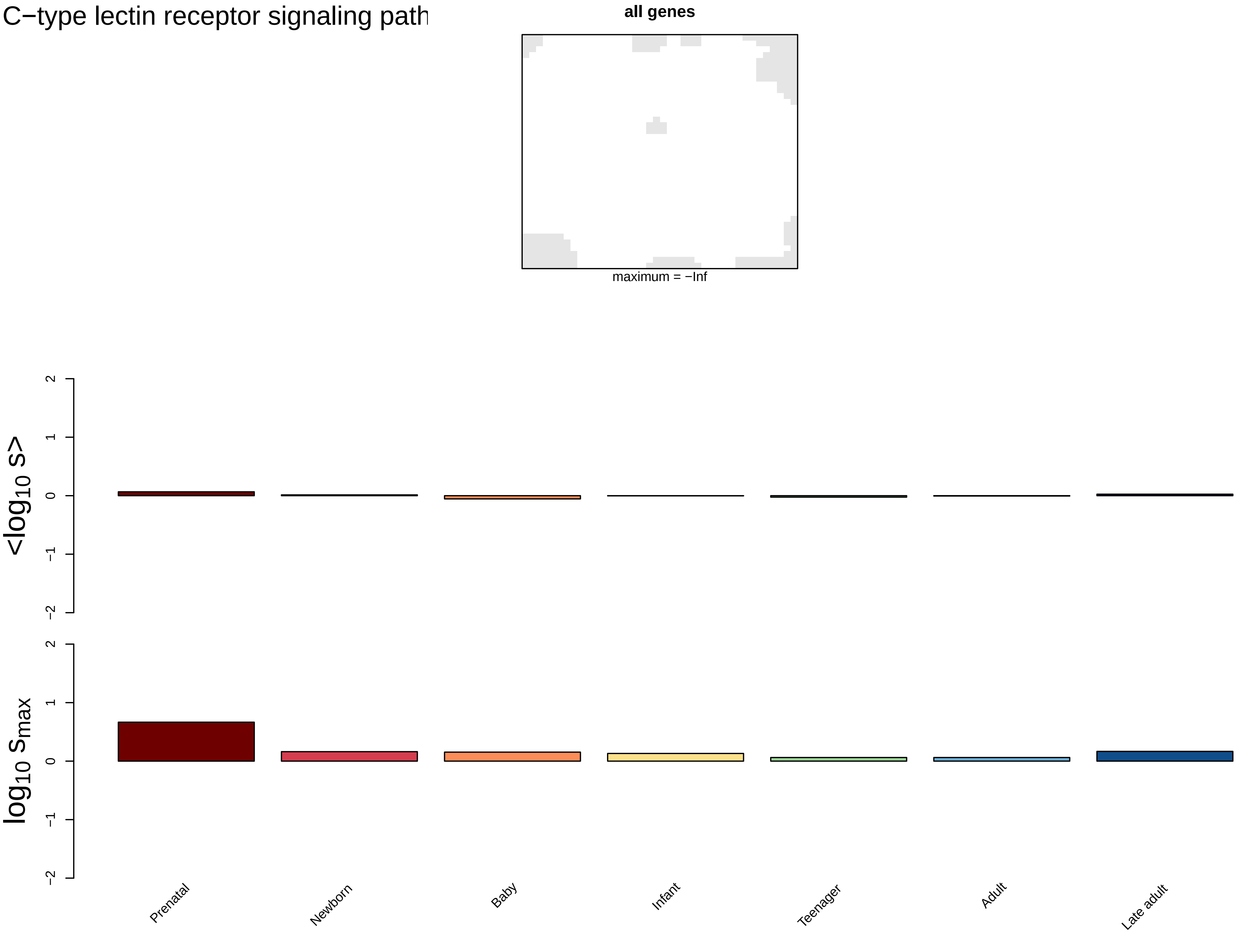

C_type_lectin_receptor_signaling_pathway
genes with data

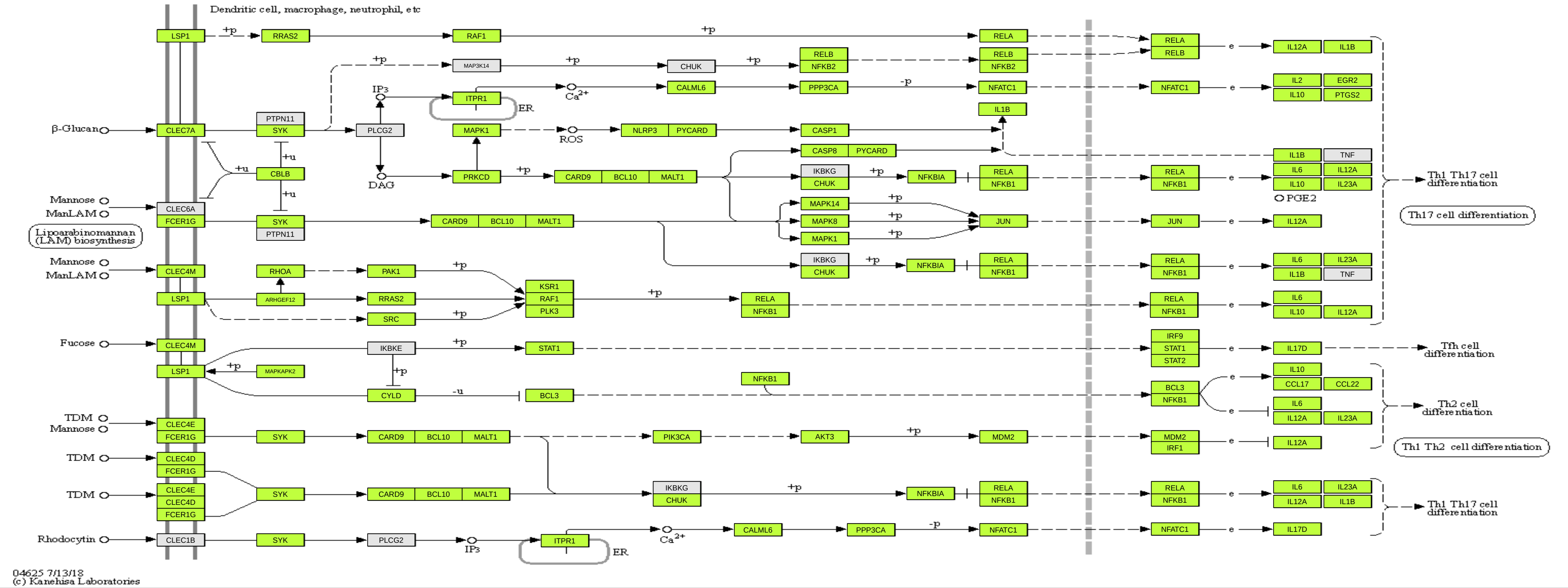

C_type_lectin_receptor_signaling_pathway
sink nodes

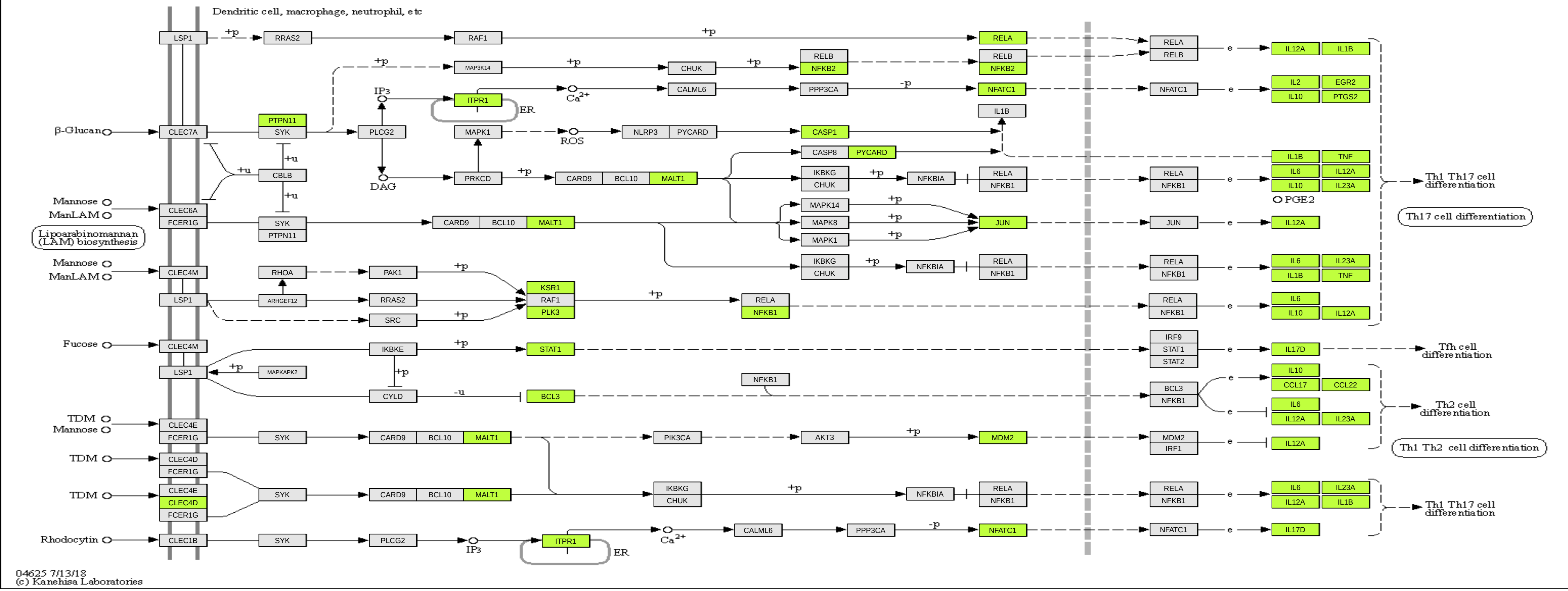

C_type_lectin_receptor_signaling_pathway
Prenatal

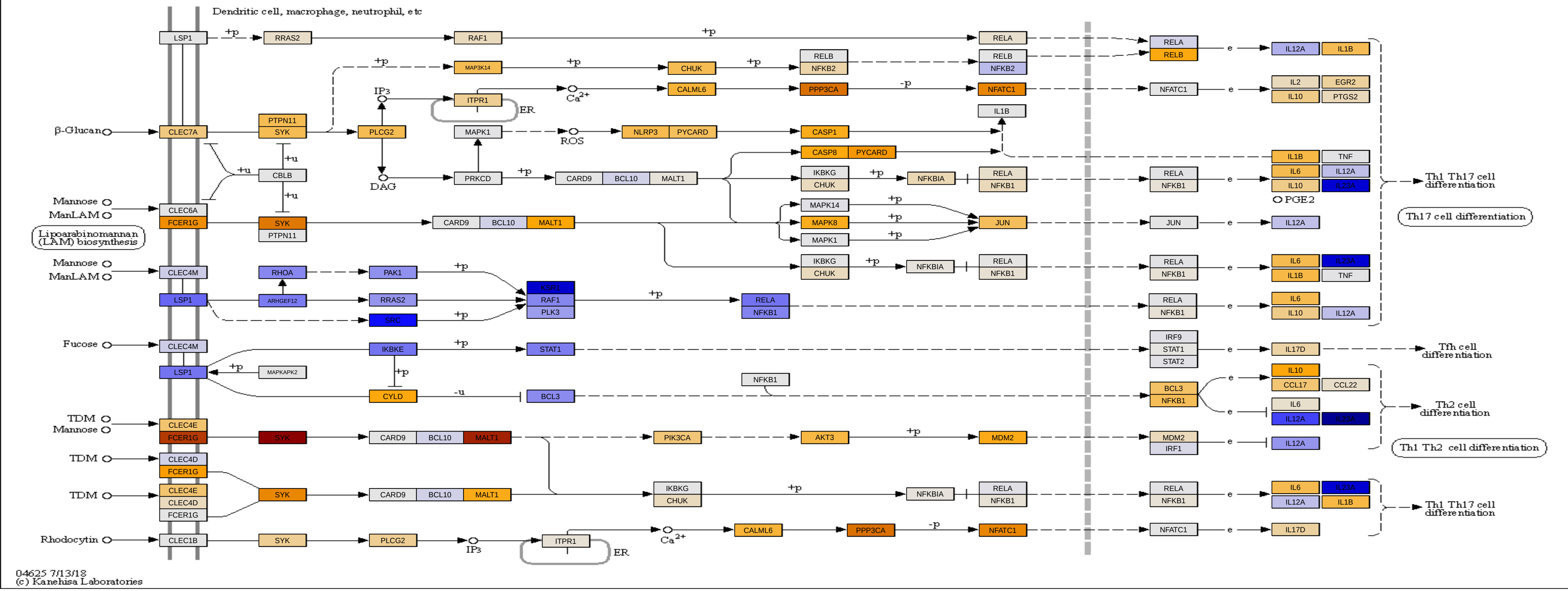

C_type_lectin_receptor_signaling_pathway
Newborn

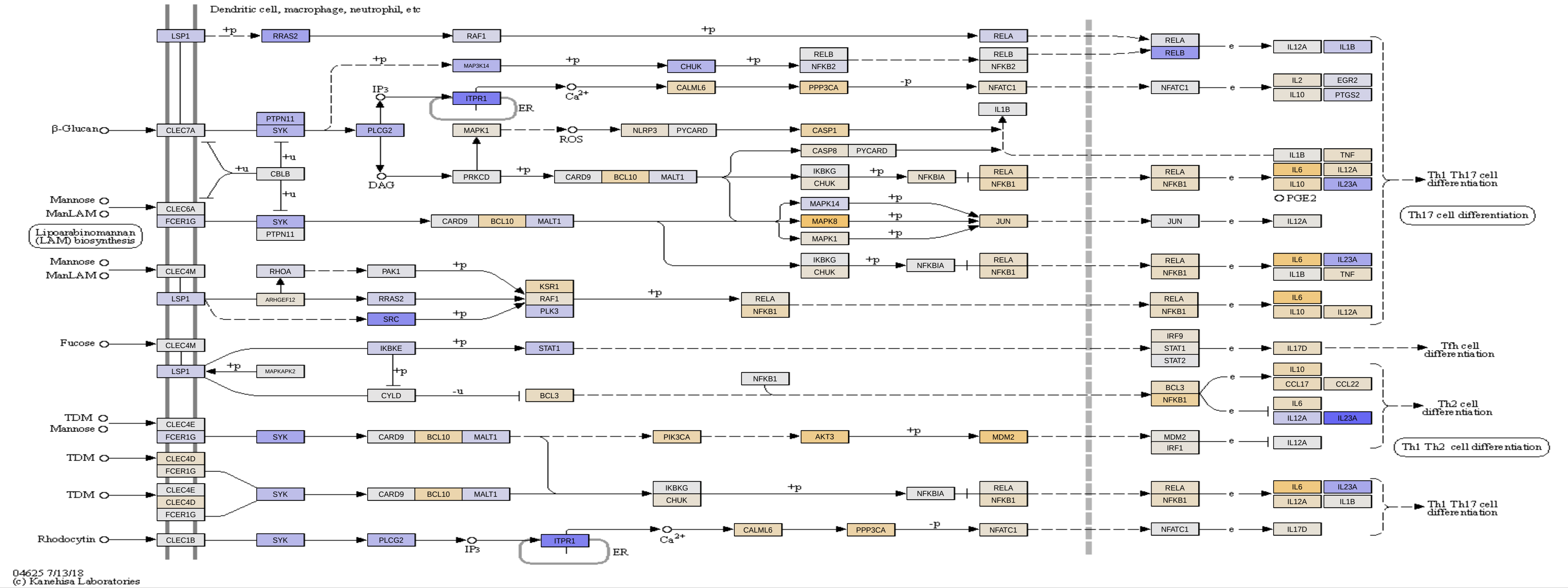

C_type_lectin_receptor_signaling_pathway
Baby

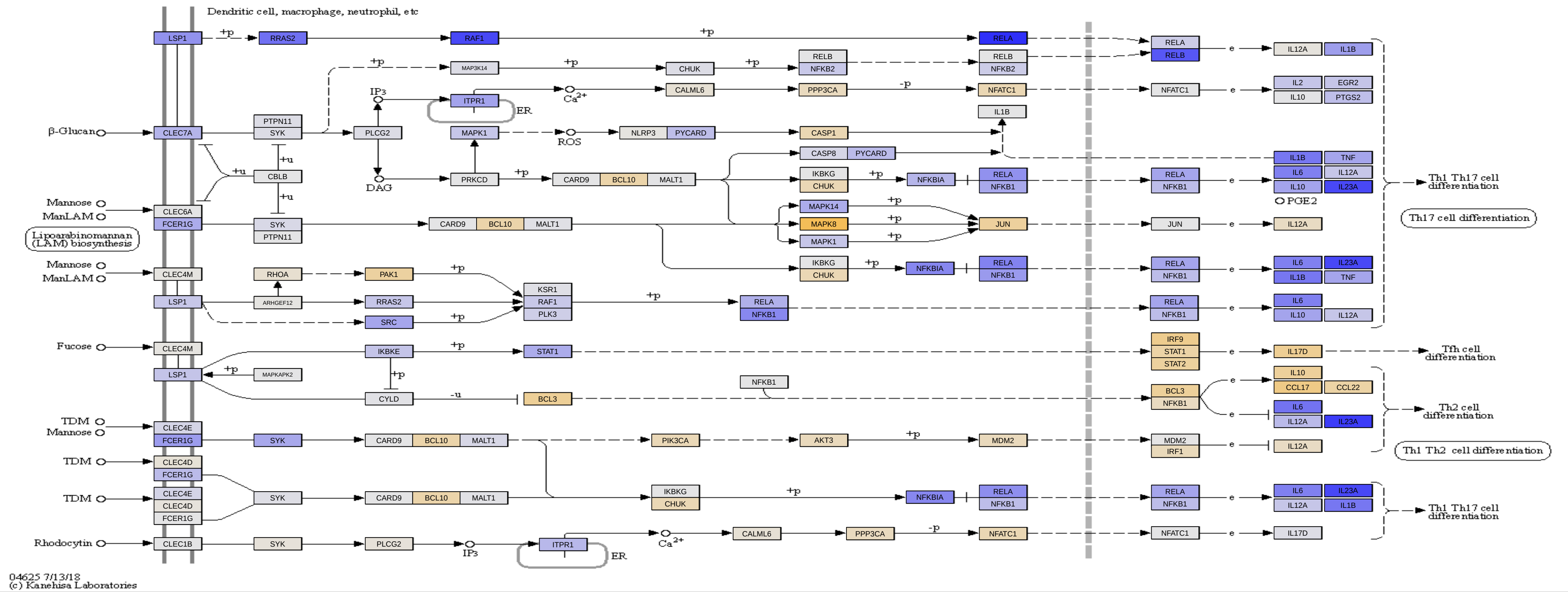

C_type_lectin_receptor_signaling_pathway
Infant

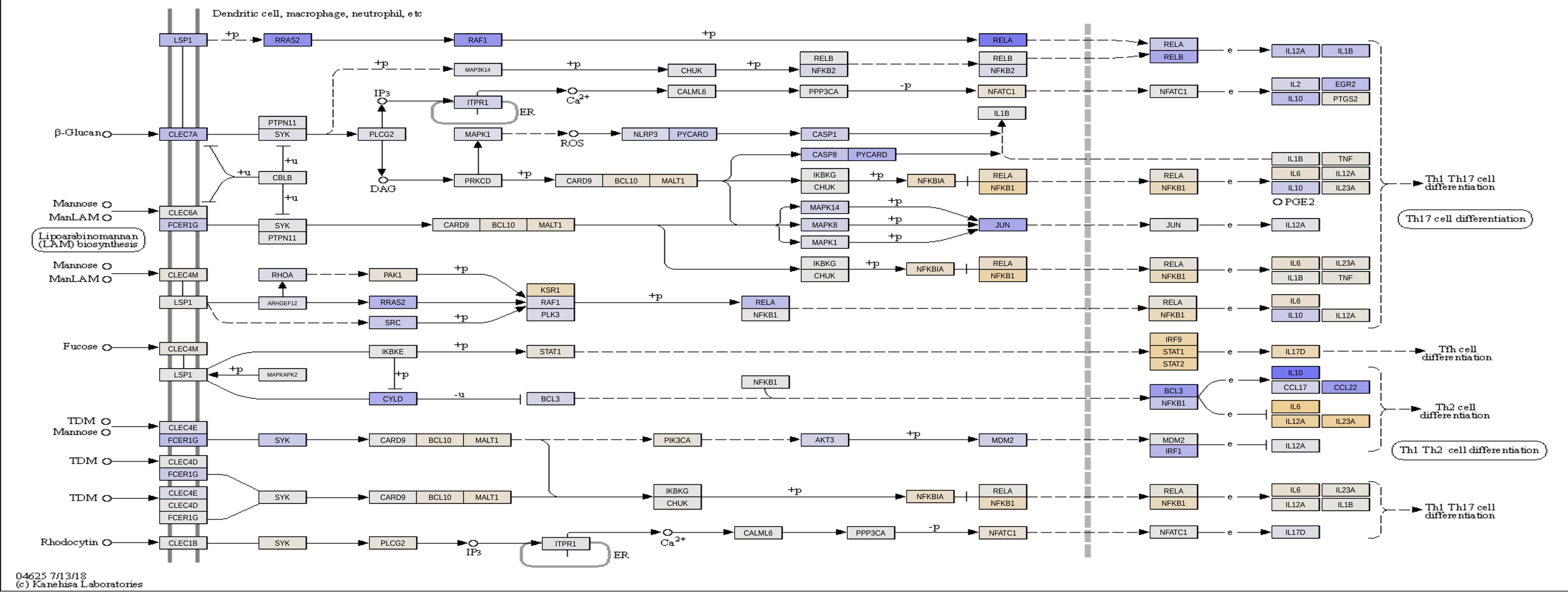

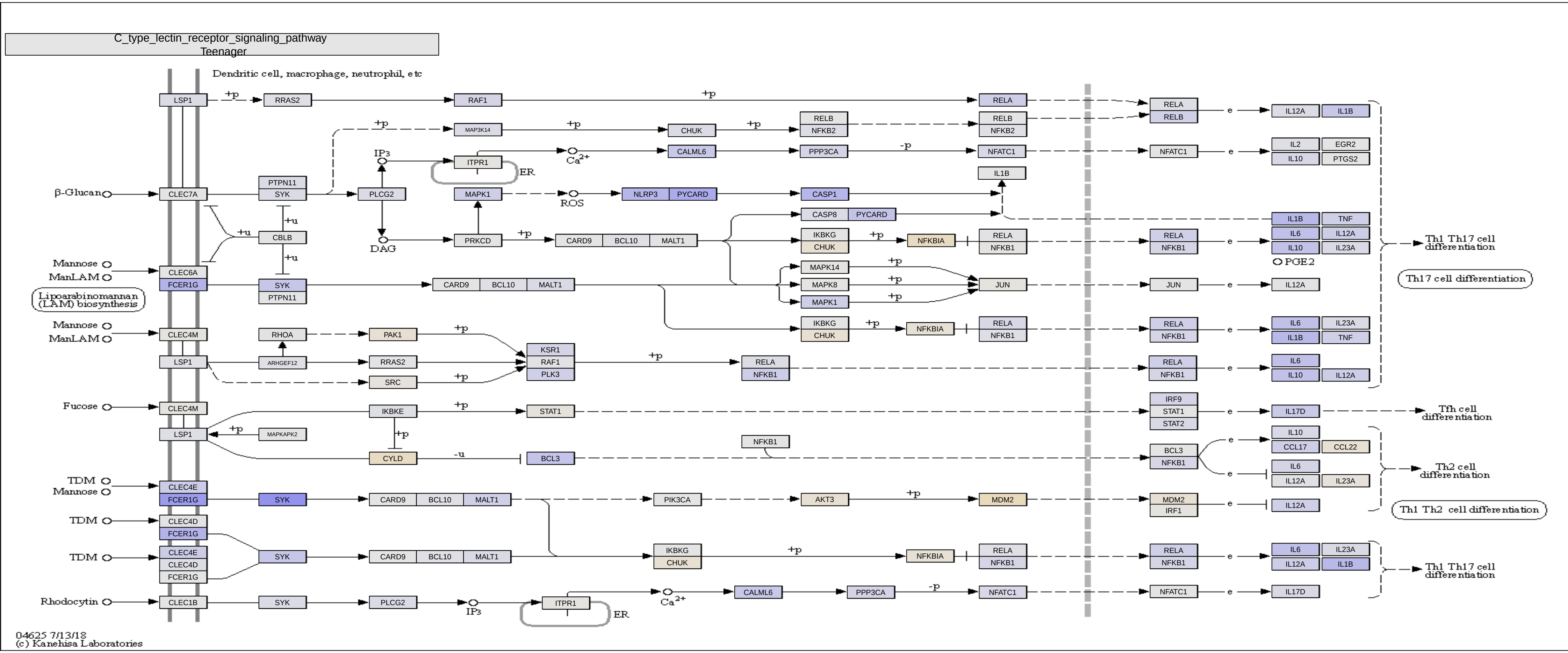
C_type_lectin_receptor_signaling_pathway
Teenager
Dendritic cell, macrophage, neutrophil, etc
β-Glucan
Mannose
ManLAM
Lipoarabinomannan (LAM) biosynthesis
Fucose
TDM
Rhodocytin
LSP1
RRAS2
RAF1
RELA
RELB
NFKB2
MAP3K14
CHUK
IP3
ITPR1
ER
Ca2+
CALML6
PPP3CA
NFATC1
PTPN11
SYK
CLEC7A
PLCG2
MAPK1
ROS
NLRP3
PYCARD
CASP1
IL1B
CASP8
CBLB
DAG
PRKCD
CARD9
BCL10
MALT1
IKBKG
NFKBIA
NFKB1
CLEC6A
FCER1G
MAPK14
MAPK8
JUN
CLEC4M
RHOA
PAK1
KSR1
PLK3
ARHGEF12
SRC
IKBKE
STAT1
MAPKAPK2
CYLD
BCL3
CLEC4E
PIK3CA
AKT3
MDM2
CLEC4D
CLEC1B
IRF9
STAT2
IRF1
IL12A
IL2
EGR2
IL10
PTGS2
TNF
IL6
IL23A
PGE2
IL17D
CCL17
CCL22
Th1 Th17 cell differentiation
Th17 cell differentiation
Tfh cell differentiation
Th2 cell differentiation
Th1 Th2 cell differentiation
04625 7/13/18
(c) Kanehisa Laboratories

C_type_lectin_receptor_signaling_pathway
Adult

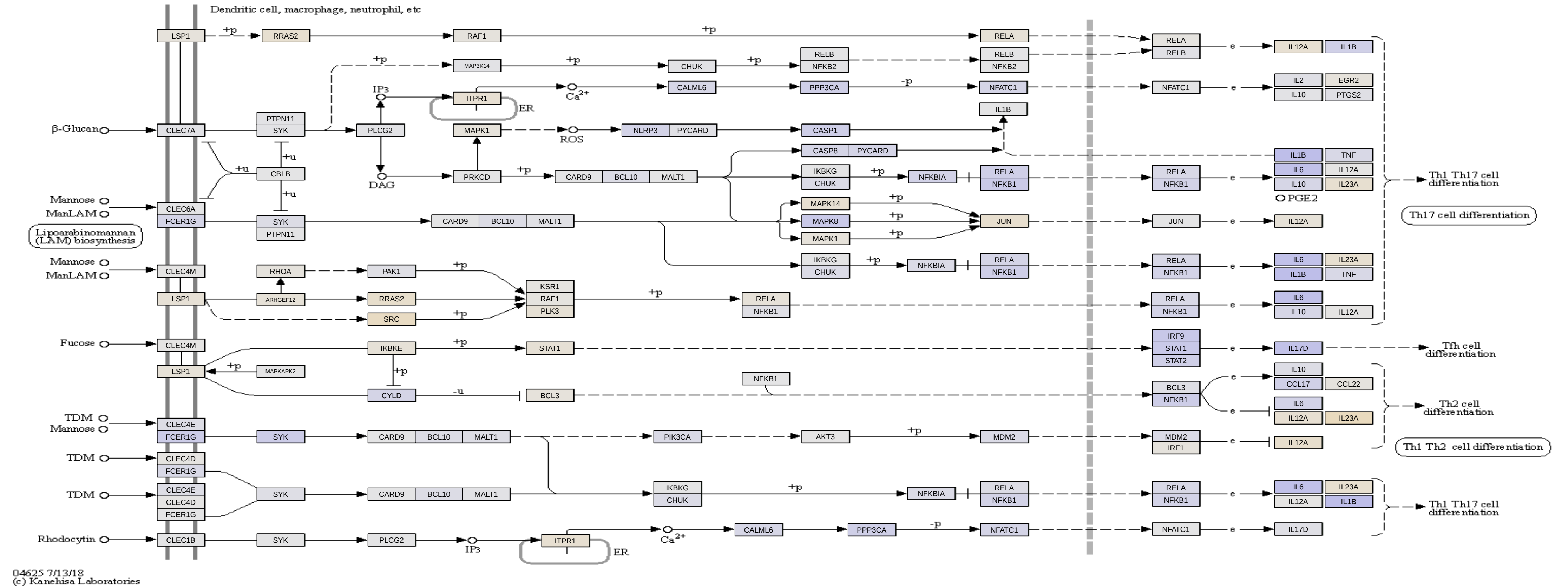

C_type_lectin_receptor_signaling_pathway
Late adult

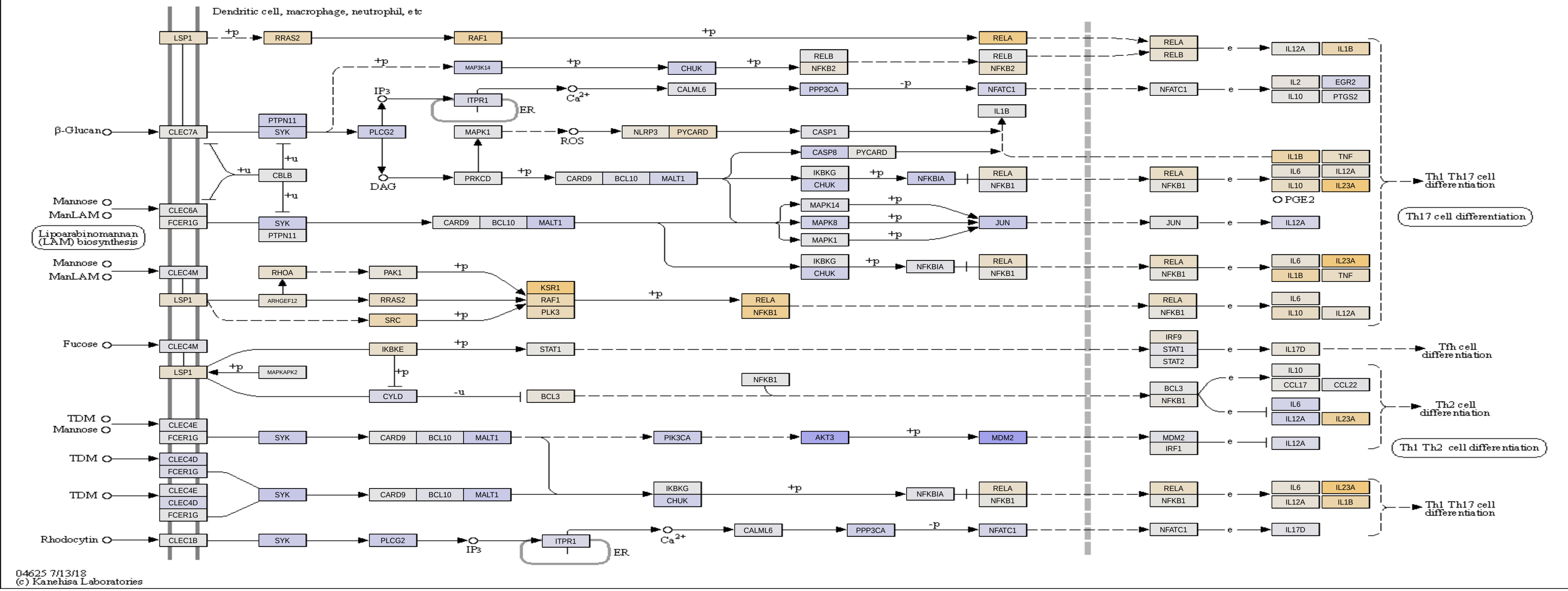

Supplement: Supplementary file 1 [file cells-11-00362-s001.zip › Suppl-Material-S4-Pathways-PSF_Methylation/C_type_lectin_receptor_signaling_pathway.pdf]
