# Supplementary material for: The Transcriptome and Methylome of the Developing and Aging Brain and Their Relations to Gliomas and Psychological Disorders
Source: Cells. 2022 Jan 21;11(3):362. doi: 10.3390/cells11030362 (PMC8834030; doi:10.3390/cells11030362)
Supplement: Supplementary file 1 [file cells-11-00362-s001.zip › Suppl-Material-S4-Pathways-PSF_Methylation/cAMP_signaling_pathway.pdf]

cAMP signaling pathway

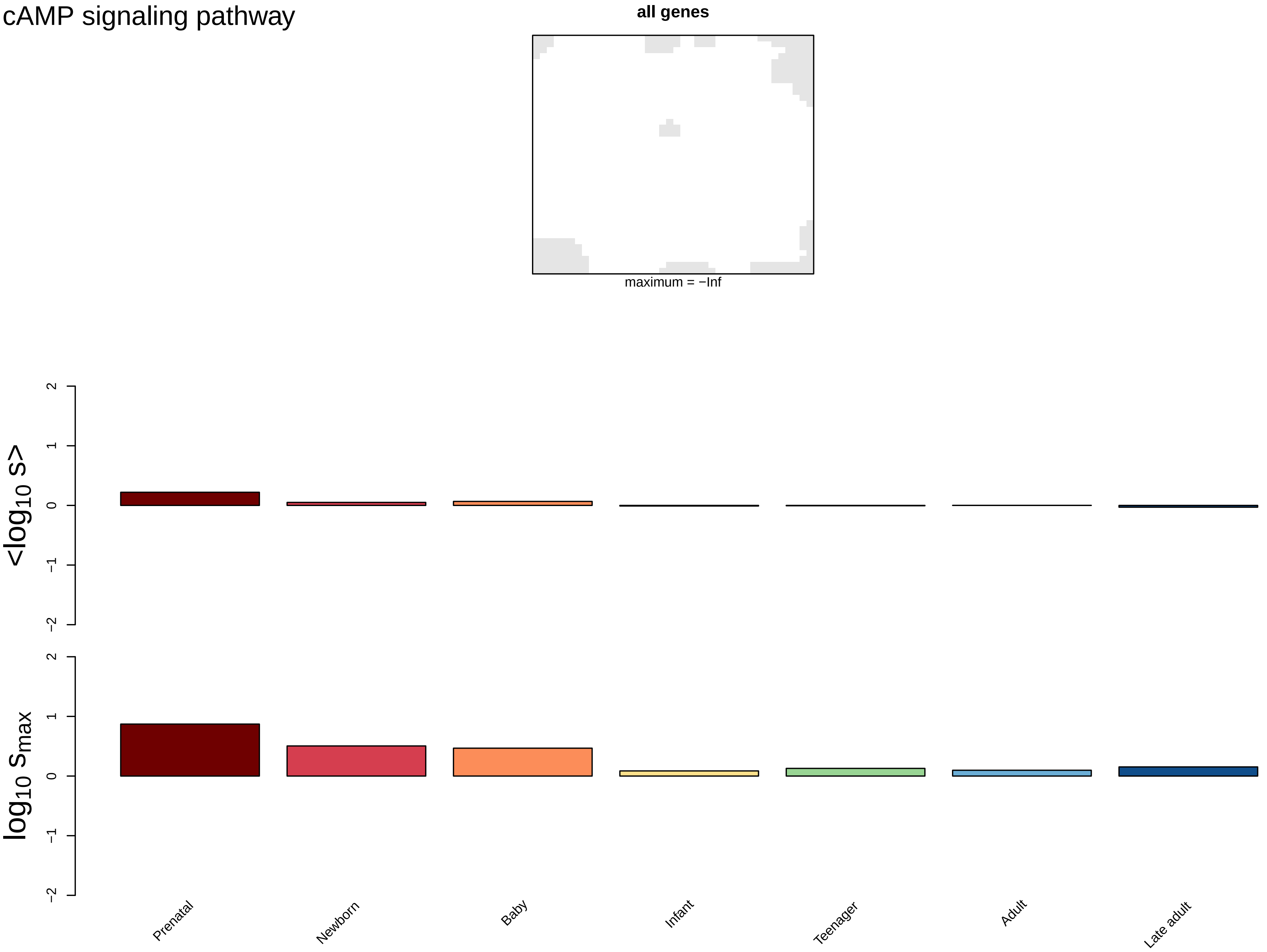

cAMP_signaling_pathway
genes with data

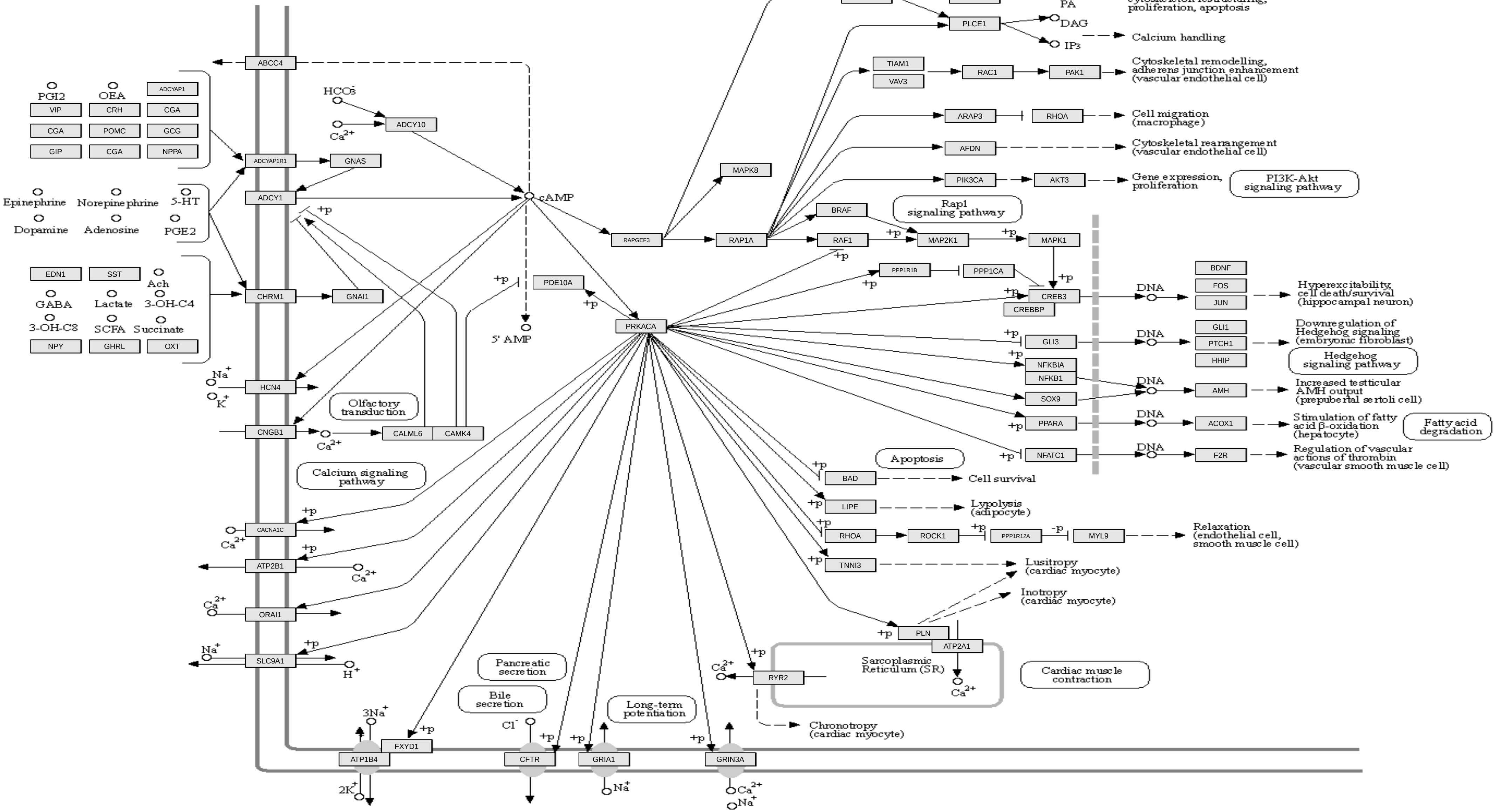

04024 6/4/21
(c) Kanehisa Laboratories

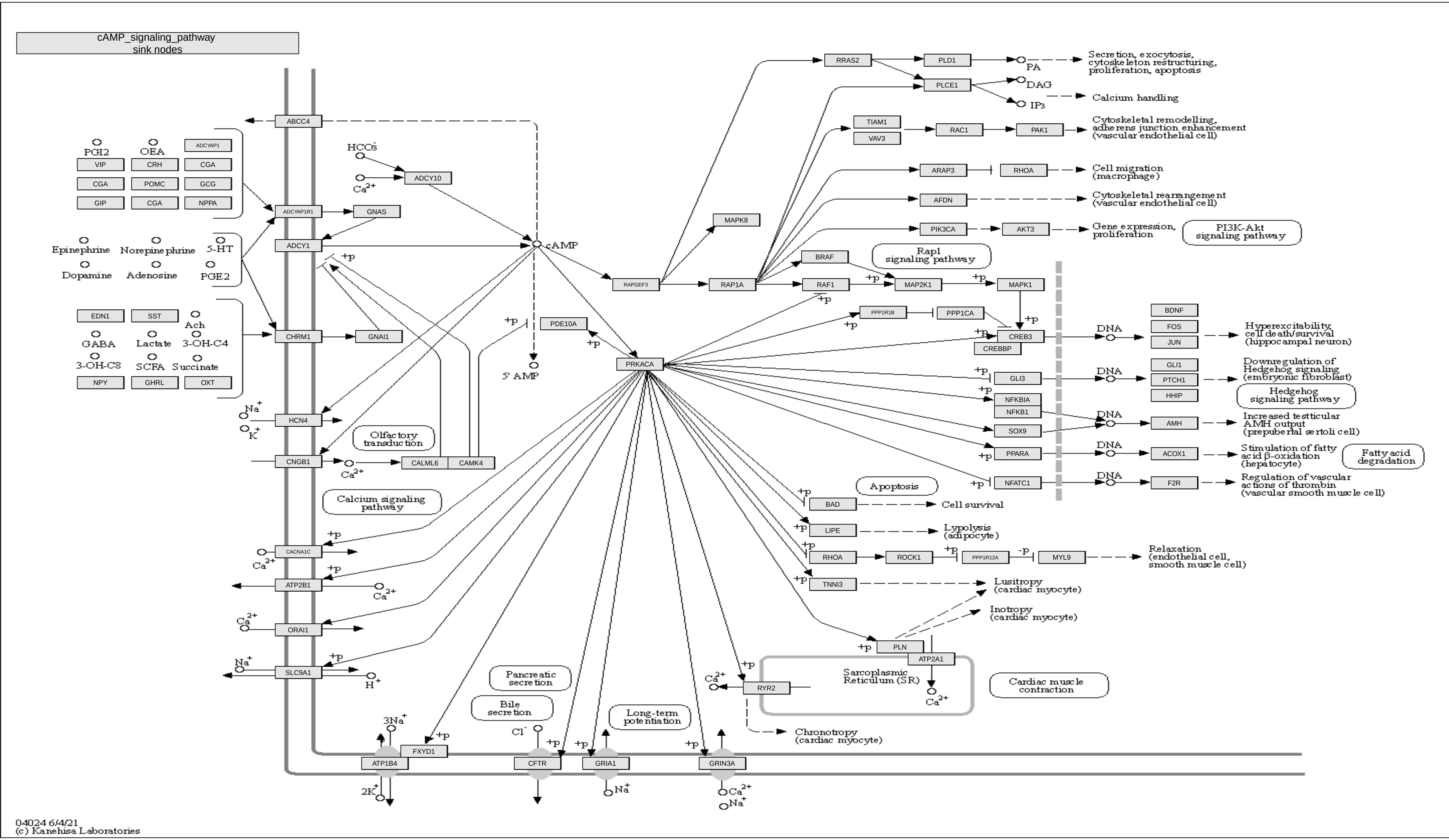
cAMP_signaling_pathway
sink nodes
ABCC4
PGI2
OEA
ADCYAP1
VIP
CRH
CGA
CGA
POMC
GCG
GIP
CGA
NPPA
HCO3-
Ca2+
ADCY10
ADCYAP1R1
GNAS
ADCY1
cAMP
Epinephrine
Norepinephrine
5-HT
Dopamine
Adenosine
PGE2
+p
EDN1
SST
Ach
GABA
Lactate
3-OH-C4
3-OH-C8
SCFA
Succinate
NPY
GHRL
OXT
CHRM1
GNAI1
PDE10A
5' AMP
PRKACA
Na+
K+
HCN4
Olfactory transduction
CNGB1
Ca2+
CALML6
CAMK4
Calcium signaling pathway
CACNA1C
ATP2B1
ORAI1
SLC9A1
H+
3Na+
2K+
FXYD1
ATP1B4
Pancreatic secretion
Bile secretion
Cl-
CFTR
Long-term potentiation
GRIA1
GRIN3A
RAPGEF3
MAPK8
RAP1A
RRAS2
PLD1
PA
PLCE1
DAG
IP3
Secretion, exocytosis, cytoskeleton restructuring, proliferation, apoptosis
Calcium handling
TIAM1
VAV3
RAC1
PAK1
Cytoskeletal remodelling, adherens junction enhancement (vascular endothelial cell)
ARAP3
RHOA
Cell migration (macrophage)
AFDN
Cytoskeletal rearrangement (vascular endothelial cell)
PIK3CA
AKT3
Gene expression, proliferation
PI3K-Akt signaling pathway
BRAF
Rap1 signaling pathway
RAF1
MAP2K1
MAPK1
PPP1R1B
PPP1CA
CREB3
CREBBP
DNA
BDNF
FOS
JUN
Hyperexcitability, cell death/survival (hippocampal neuron)
GLI3
GLI1
PTCH1
HHIP
Downregulation of Hedgehog signaling (embryonic fibroblast)
Hedgehog signaling pathway
NFKBIA
NFKB1
SOX9
AMH
Increased testicular AMH output (prepubertal sertoli cell)
PPARA
ACOX1
Stimulation of fatty acid β-oxidation (hepatocyte)
Fatty acid degradation
NFATC1
F2R
Regulation of vascular actions of thrombin (vascular smooth muscle cell)
Apoptosis
BAD
Cell survival
LIPE
Lypolysis (adipocyte)
RHOA
ROCK1
PPP1R12A
-p
MYL9
Relaxation (endothelial cell, smooth muscle cell)
TNNI3
Lusitropy (cardiac myocyte)
Inotropy (cardiac myocyte)
PLN
ATP2A1
Sarcoplasmic Reticulum (SR)
Cardiac muscle contraction
RYR2
Chronotropy (cardiac myocyte)
04024 6/4/21
(c) Kanehisa Laboratories

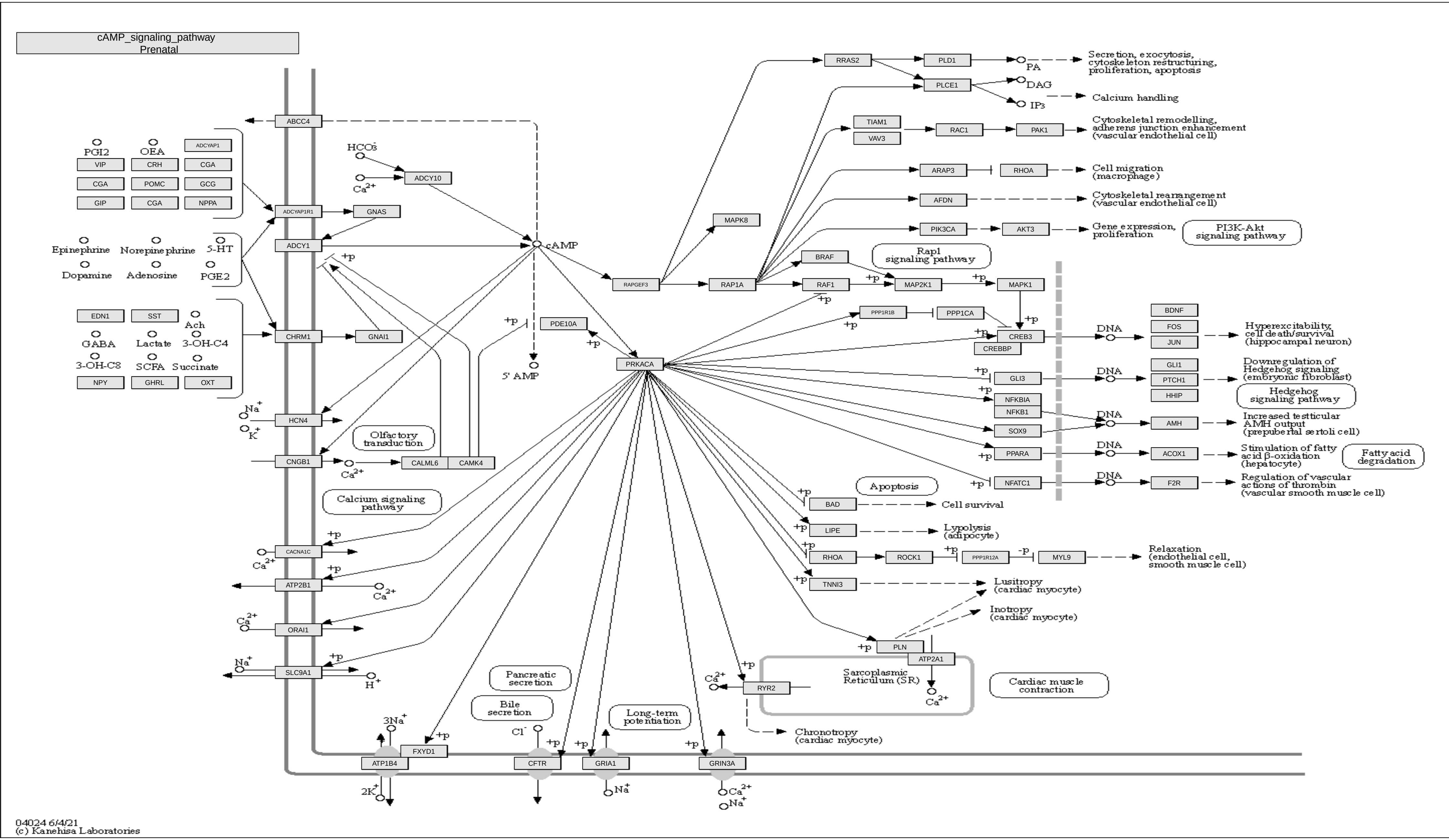
cAMP_signaling_pathway
Prenatal
ABCC4
PGI2
OEA
ADCYAP1
VIP
CRH
CGA
CGA
POMC
GCG
GIP
CGA
NPPA
HCO3-
Ca2+
ADCY10
ADCYAP1R1
GNAS
ADCY1
cAMP
Epinephrine
Norepinephrine
5-HT
Dopamine
Adenosine
PGE2
+p
EDN1
SST
Ach
GABA
Lactate
3-OH-C4
3-OH-C8
SCFA
Succinate
NPY
GHRL
OXT
CHRM1
GNAI1
PDE10A
5' AMP
PRKACA
Na+
K+
HCN4
CNGB1
Olfactory transduction
CALML6
CAMK4
Calcium signaling pathway
CACNA1C
ATP2B1
ORAI1
SLC9A1
H+
3Na+
2K+
ATP1B4
FXYD1
Pancreatic secretion
Bile secretion
Cl-
CFTR
GRIA1
Long-term potentiation
GRIN3A
RYR2
Sarcoplasmic Reticulum (SR)
PLN
ATP2A1
Cardiac muscle contraction
Chronotropy (cardiac myocyte)
Lusitropy (cardiac myocyte)
Inotropy (cardiac myocyte)
RRAS2
PLD1
PA
Secretion, exocytosis, cytoskeleton restructuring, proliferation, apoptosis
PLCE1
DAG
IP3
Calcium handling
TIAM1
VAV3
RAC1
PAK1
Cytoskeletal remodelling, adherens junction enhancement (vascular endothelial cell)
ARAP3
RHOA
Cell migration (macrophage)
AFDN
Cytoskeletal rearrangement (vascular endothelial cell)
MAPK8
PIK3CA
AKT3
Gene expression, proliferation
PI3K-Akt signaling pathway
BRAF
Rap1 signaling pathway
RAPGEF3
RAP1A
RAF1
MAP2K1
MAPK1
PPP1R1B
PPP1CA
CREB3
CREBBP
DNA
BDNF
FOS
JUN
Hyperexcitability, cell death/survival (hippocampal neuron)
GLI3
GLI1
PTCH1
HHIP
Downregulation of Hedgehog signaling (embryonic fibroblast)
Hedgehog signaling pathway
NFKBIA
NFKB1
SOX9
AMH
Increased testicular AMH output (prepubertal sertoli cell)
PPARA
ACOX1
Stimulation of fatty acid β-oxidation (hepatocyte)
Fatty acid degradation
NFATC1
F2R
Regulation of vascular actions of thrombin (vascular smooth muscle cell)
BAD
Apoptosis
Cell survival
LIPE
Lypolysis (adipocyte)
ROCK1
PPP1R12A
-p
MYL9
Relaxation (endothelial cell, smooth muscle cell)
TNNI3
04024 6/4/21
(c) Kanehisa Laboratories

cAMP_signaling_pathway
Newborn

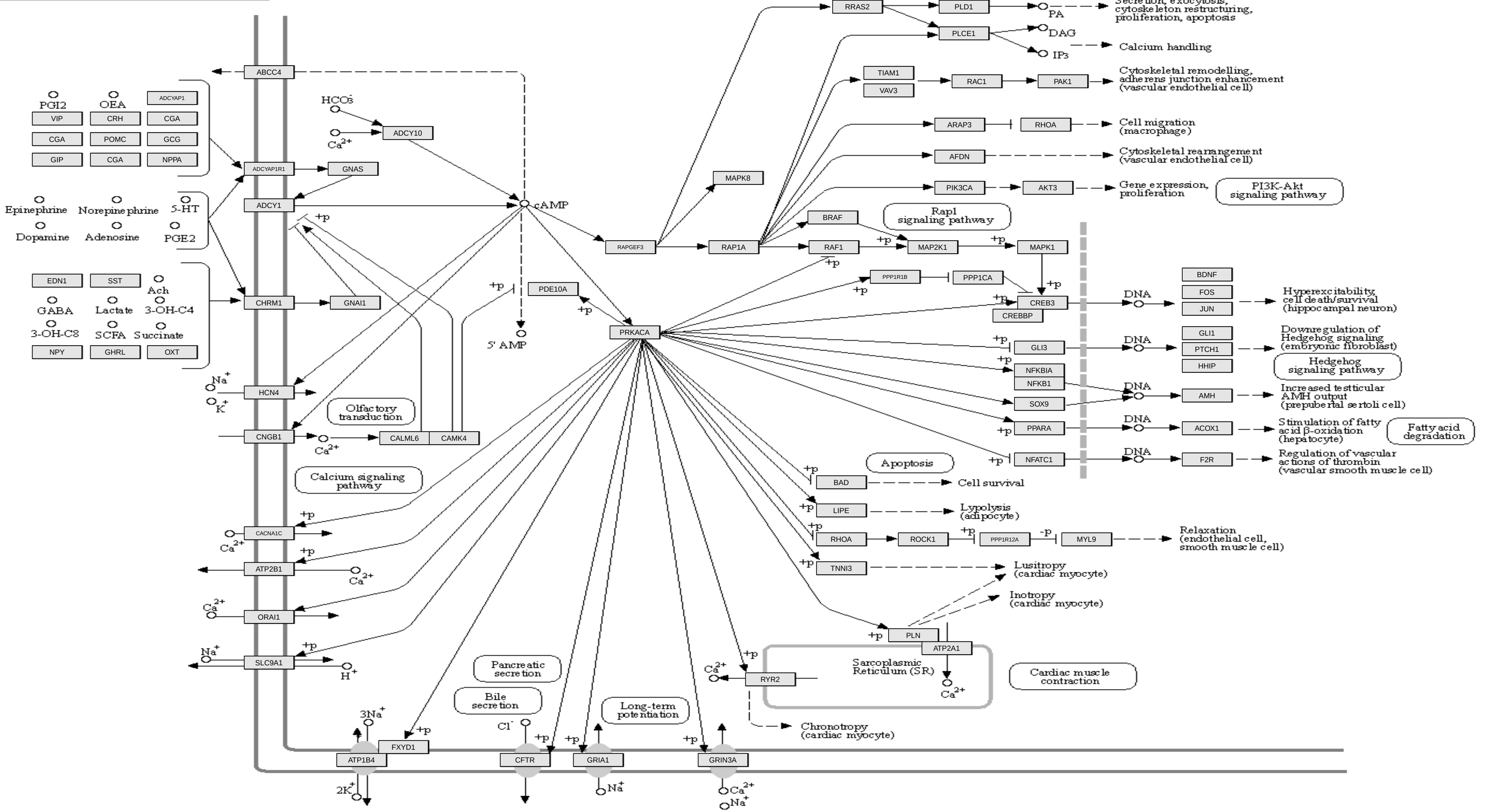

04024 6/4/21
(c) Kanehisa Laboratories

cAMP_signaling_pathway
Baby

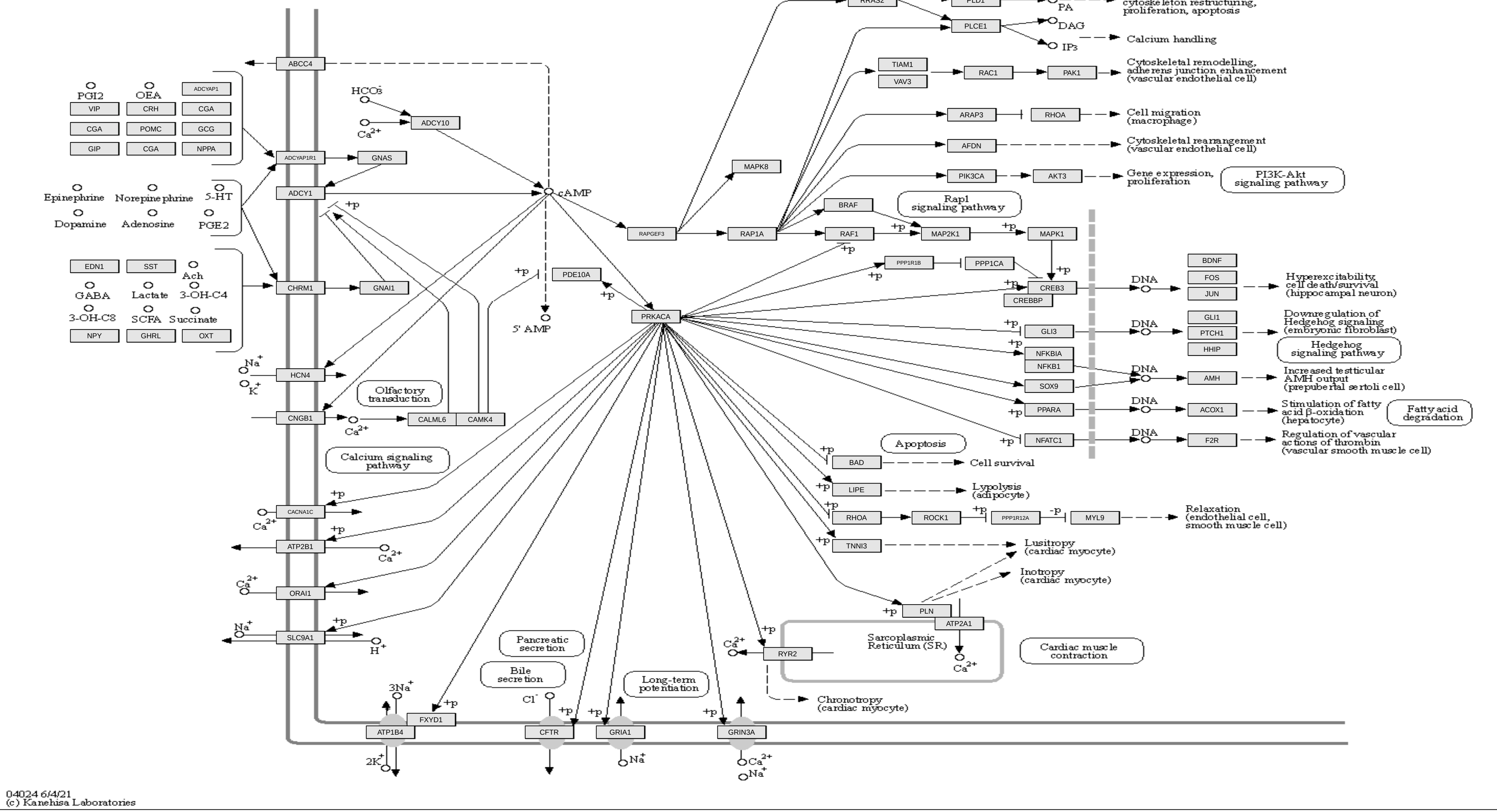

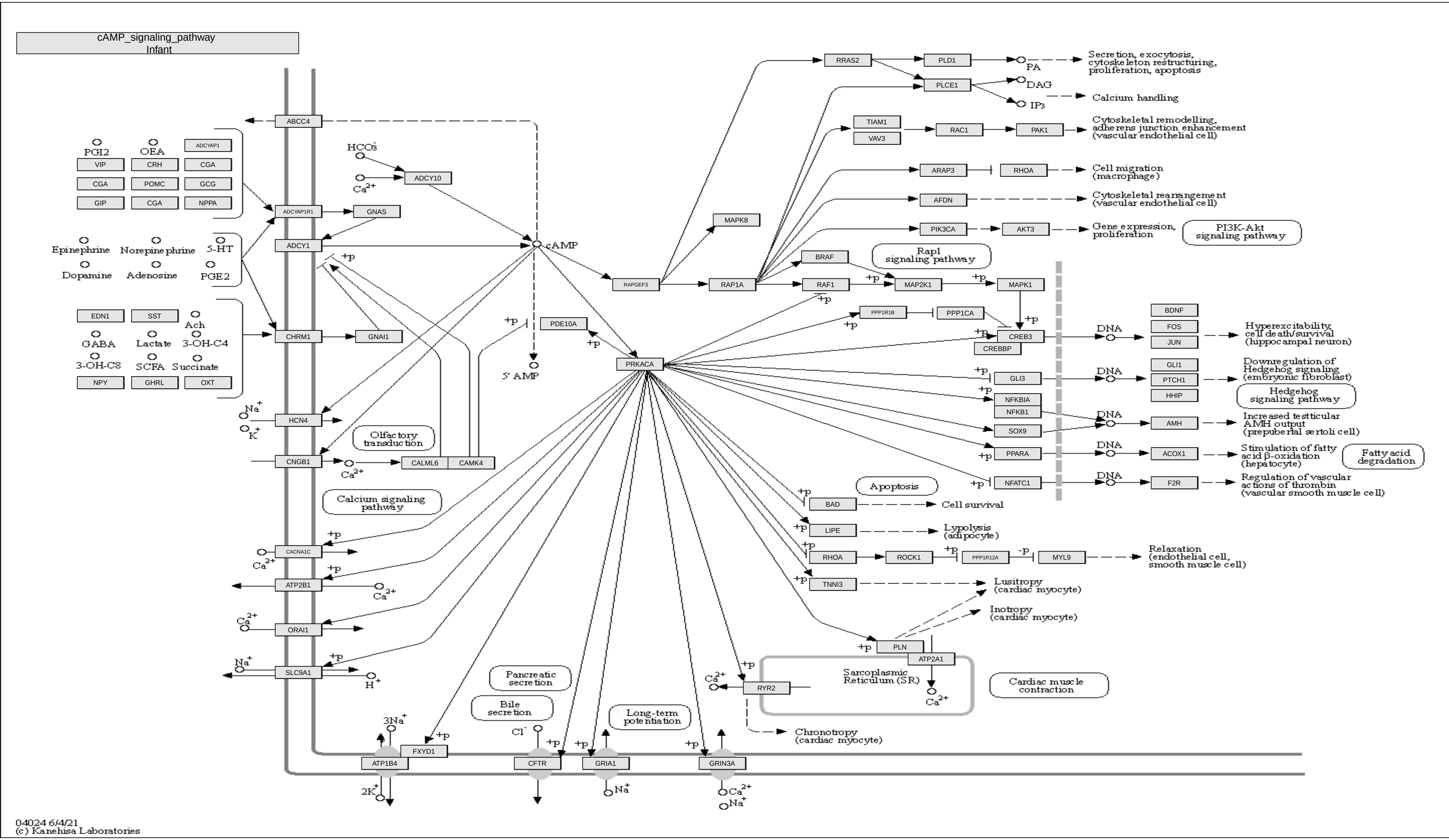
cAMP_signaling_pathway
Infant
ABCC4
PGI2
OEA
ADCYAP1
VIP
CRH
CGA
CGA
POMC
GCG
GIP
CGA
NPPA
HCO3-
Ca2+
ADCY10
ADCYAP1R1
GNAS
ADCY1
cAMP
Epinephrine
Norepinephrine
5-HT
Dopamine
Adenosine
PGE2
+p
EDN1
SST
Ach
GABA
Lactate
3-OH-C4
3-OH-C8
SCFA
Succinate
NPY
GHRL
OXT
CHRM1
GNAI1
PDE10A
5' AMP
PRKACA
Na+
K+
HCN4
Olfactory transduction
CNGB1
Ca2+
CALML6
CAMK4
Calcium signaling pathway
CACNA1C
ATP2B1
ORAI1
SLC9A1
H+
3Na+
2K+
ATP1B4
FXYD1
Pancreatic secretion
Bile secretion
Cl-
CFTR
Long-term potentiation
GRIA1
GRIN3A
RAPGEF3
MAPK8
RAP1A
RRAS2
PLD1
PA
PLCE1
DAG
IP3
Secretion, exocytosis, cytoskeleton restructuring, proliferation, apoptosis
Calcium handling
TIAM1
VAV3
RAC1
PAK1
Cytoskeletal remodelling, adherens junction enhancement (vascular endothelial cell)
ARAP3
RHOA
Cell migration (macrophage)
AFDN
Cytoskeletal rearrangement (vascular endothelial cell)
PIK3CA
AKT3
Gene expression, proliferation
PI3K-Akt signaling pathway
BRAF
Rap1 signaling pathway
RAF1
MAP2K1
MAPK1
PPP1R1B
PPP1CA
CREB3
CREBBP
DNA
BDNF
FOS
JUN
Hyperexcitability, cell death/survival (hippocampal neuron)
GLI3
GLI1
PTCH1
HHIP
Downregulation of Hedgehog signaling (embryonic fibroblast)
Hedgehog signaling pathway
NFKBIA
NFKB1
SOX9
AMH
Increased testicular AMH output (prepubertal sertoli cell)
PPARA
ACOX1
Stimulation of fatty acid β-oxidation (hepatocyte)
Fatty acid degradation
NFATC1
F2R
Regulation of vascular actions of thrombin (vascular smooth muscle cell)
Apoptosis
BAD
Cell survival
LIPE
Lypolysis (adipocyte)
RHOA
ROCK1
PPP1R12A
-p
MYL9
Relaxation (endothelial cell, smooth muscle cell)
TNNI3
Lusitropy (cardiac myocyte)
Inotropy (cardiac myocyte)
PLN
ATP2A1
Sarcoplasmic Reticulum (SR)
RYR2
Cardiac muscle contraction
Chronotropy (cardiac myocyte)
04024 6/4/21
(c) Kanehisa Laboratories

cAMP_signaling_pathway
Teenager

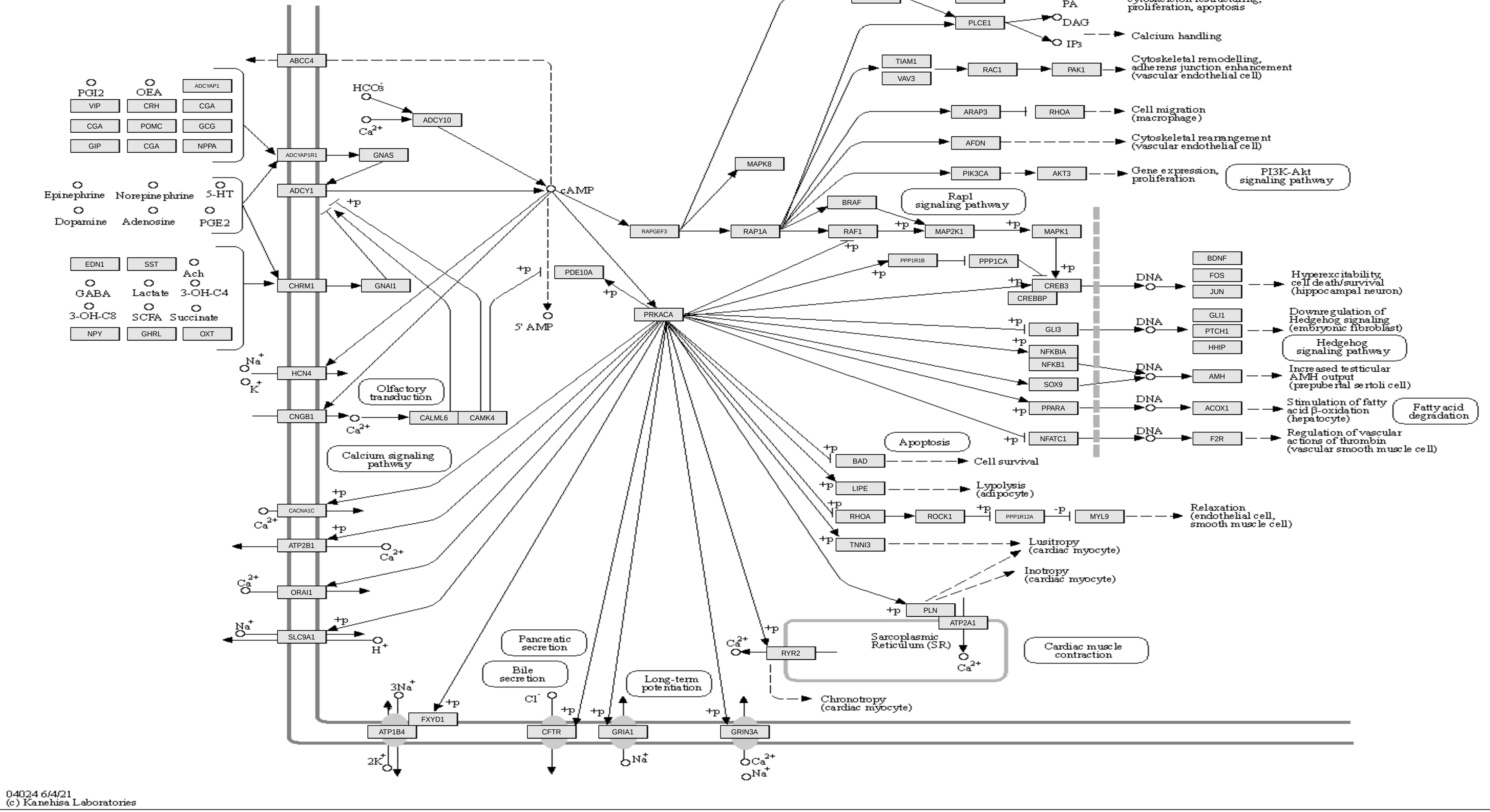

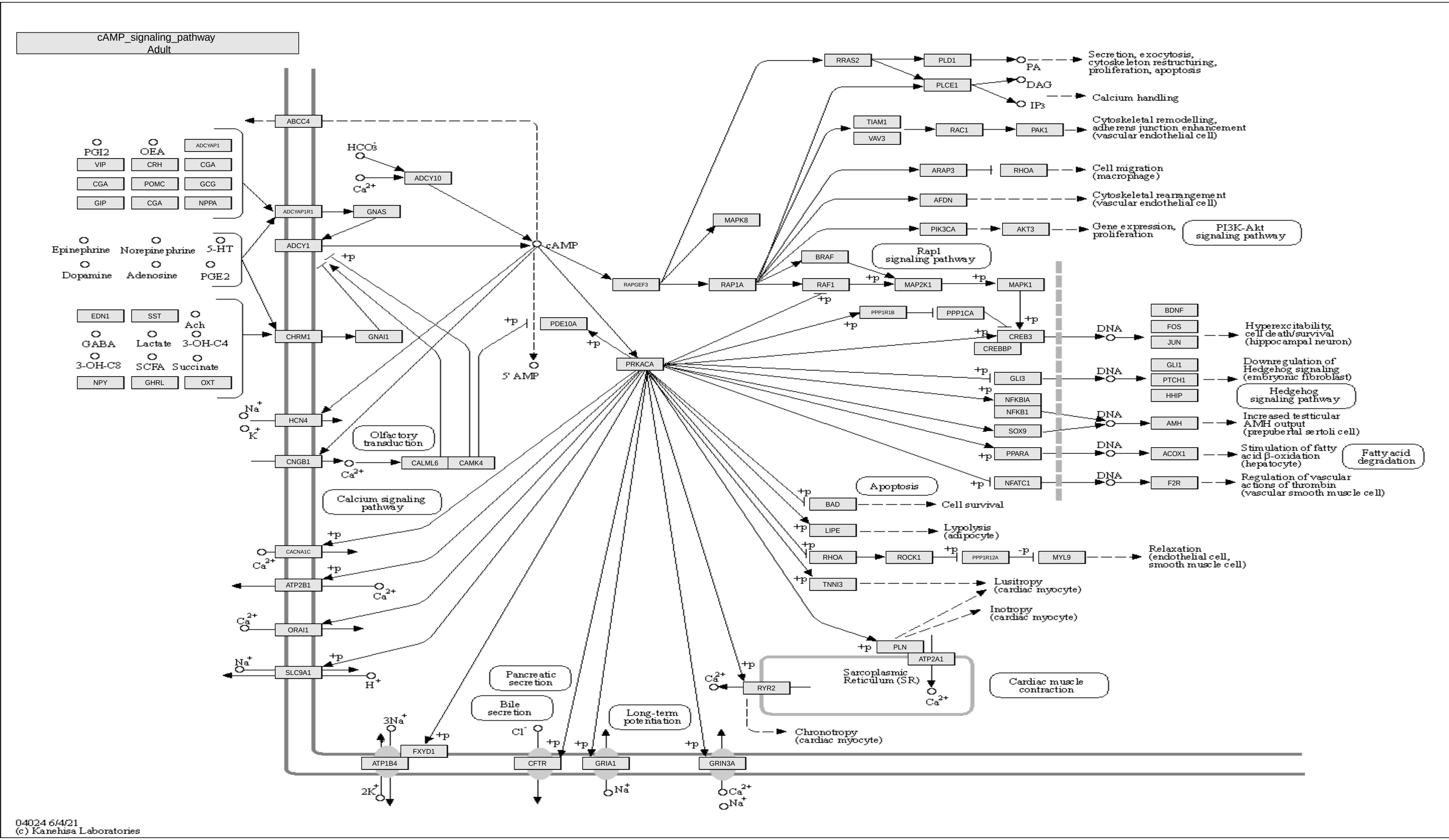
cAMP_signaling_pathway
Adult
ABCC4
PGI2
OEA
ADCYAP1
VIP
CRH
CGA
CGA
POMC
GCG
GIP
CGA
NPPA
HCO3-
Ca2+
ADCY10
ADCYAP1R1
GNAS
Epinephrine
Norepinephrine
5-HT
Dopamine
Adenosine
PGE2
ADCY1
cAMP
+p
EDN1
SST
Ach
GABA
Lactate
3-OH-C4
3-OH-C8
SCFA
Succinate
NPY
GHRL
OXT
CHRM1
GNAI1
PDE10A
5' AMP
Na+
K+
HCN4
Olfactory transduction
CNGB1
CALML6
CAMK4
Calcium signaling pathway
CACNA1C
ATP2B1
ORAI1
SLC9A1
H+
3Na+
2K+
ATP1B4
FXYD1
Pancreatic secretion
Bile secretion
Cl-
CFTR
Long-term potentiation
GRIA1
GRIN3A
RYR2
Chronotropy (cardiac myocyte)
Sarcoplasmic Reticulum (SR)
PLN
ATP2A1
Cardiac muscle contraction
PRKACA
RAPGEF3
MAPK8
RRAS2
PLD1
PA
Secretion, exocytosis, cytoskeleton restructuring, proliferation, apoptosis
PLCE1
DAG
IP3
Calcium handling
TIAM1
VAV3
RAC1
PAK1
Cytoskeletal remodelling, adherens junction enhancement (vascular endothelial cell)
ARAP3
RHOA
Cell migration (macrophage)
AFDN
Cytoskeletal rearrangement (vascular endothelial cell)
PIK3CA
AKT3
Gene expression, proliferation
PI3K-Akt signaling pathway
BRAF
Rap1 signaling pathway
RAP1A
RAF1
MAP2K1
MAPK1
PPP1R1B
PPP1CA
CREB3
CREBBP
DNA
BDNF
FOS
JUN
Hyperexcitability, cell death/survival (hippocampal neuron)
GLI3
GLI1
PTCH1
HHIP
Downregulation of Hedgehog signaling (embryonic fibroblast)
Hedgehog signaling pathway
NFKBIA
NFKB1
SOX9
AMH
Increased testicular AMH output (prepubertal sertoli cell)
PPARA
ACOX1
Stimulation of fatty acid β-oxidation (hepatocyte)
Fatty acid degradation
NFATC1
F2R
Regulation of vascular actions of thrombin (vascular smooth muscle cell)
Apoptosis
BAD
Cell survival
LIPE
Lypolysis (adipocyte)
RHOA
ROCK1
PPP1R12A
-p
MYL9
Relaxation (endothelial cell, smooth muscle cell)
TNNI3
Lusitropy (cardiac myocyte)
Inotropy (cardiac myocyte)
04024 6/4/21
(c) Kanehisa Laboratories

cAMP_signaling_pathway
Late adult

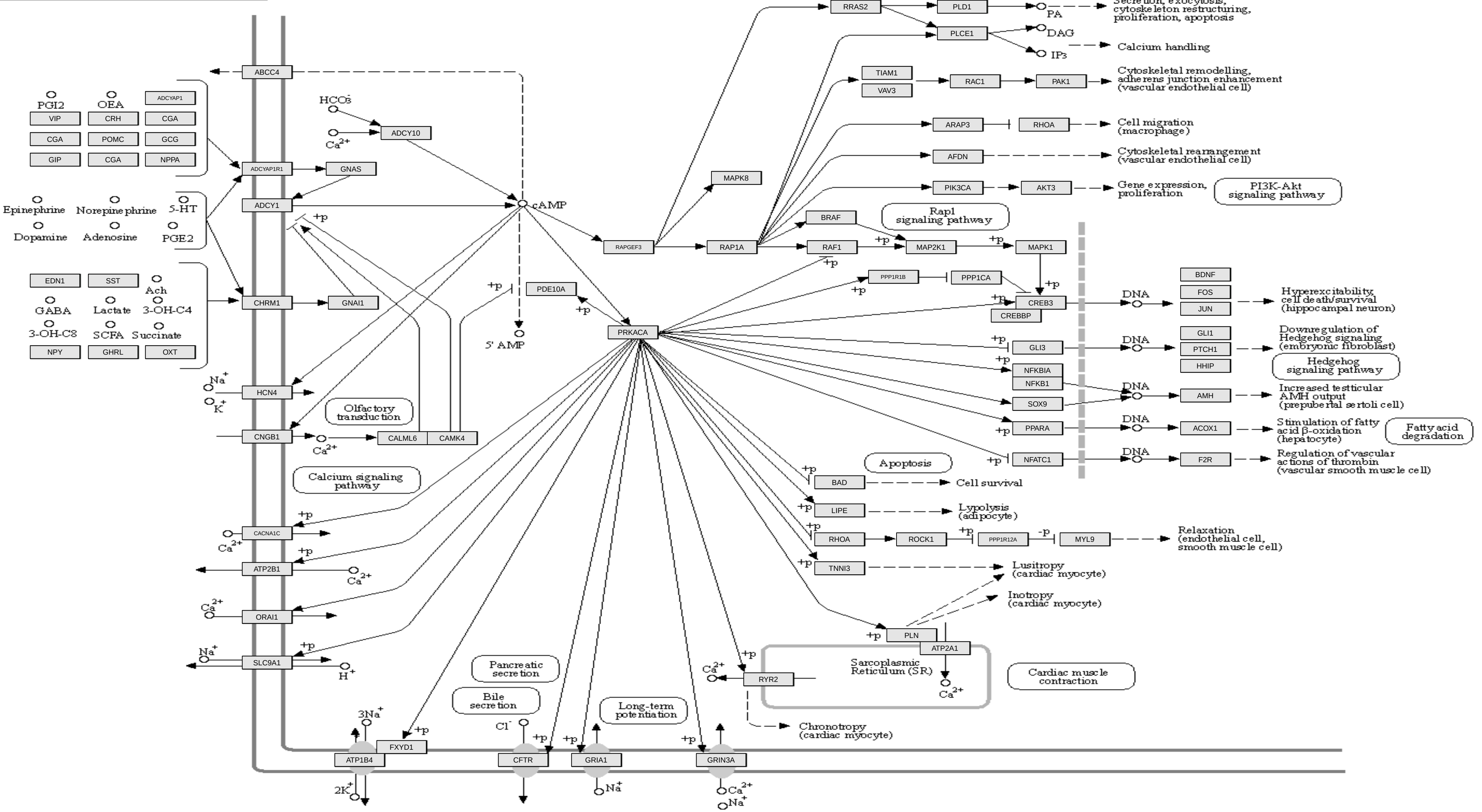

04024 6/4/21
(c) Kanehisa Laboratories
