# Supplementary material for: The Transcriptome and Methylome of the Developing and Aging Brain and Their Relations to Gliomas and Psychological Disorders
Source: Cells. 2022 Jan 21;11(3):362. doi: 10.3390/cells11030362 (PMC8834030; doi:10.3390/cells11030362)
Supplement: Supplementary file 1 [file cells-11-00362-s001.zip › Suppl-Material-S4-Pathways-PSF_Methylation/Chemokine_signaling_pathway.pdf]

Chemokine signaling pathway

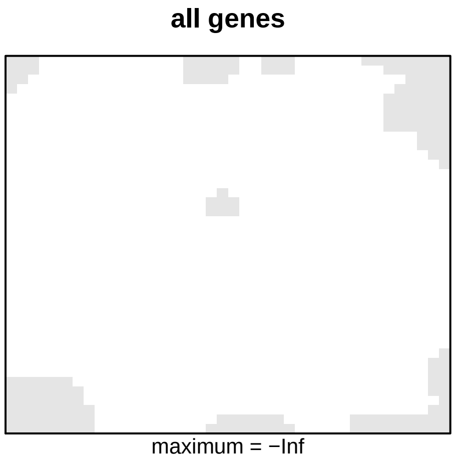

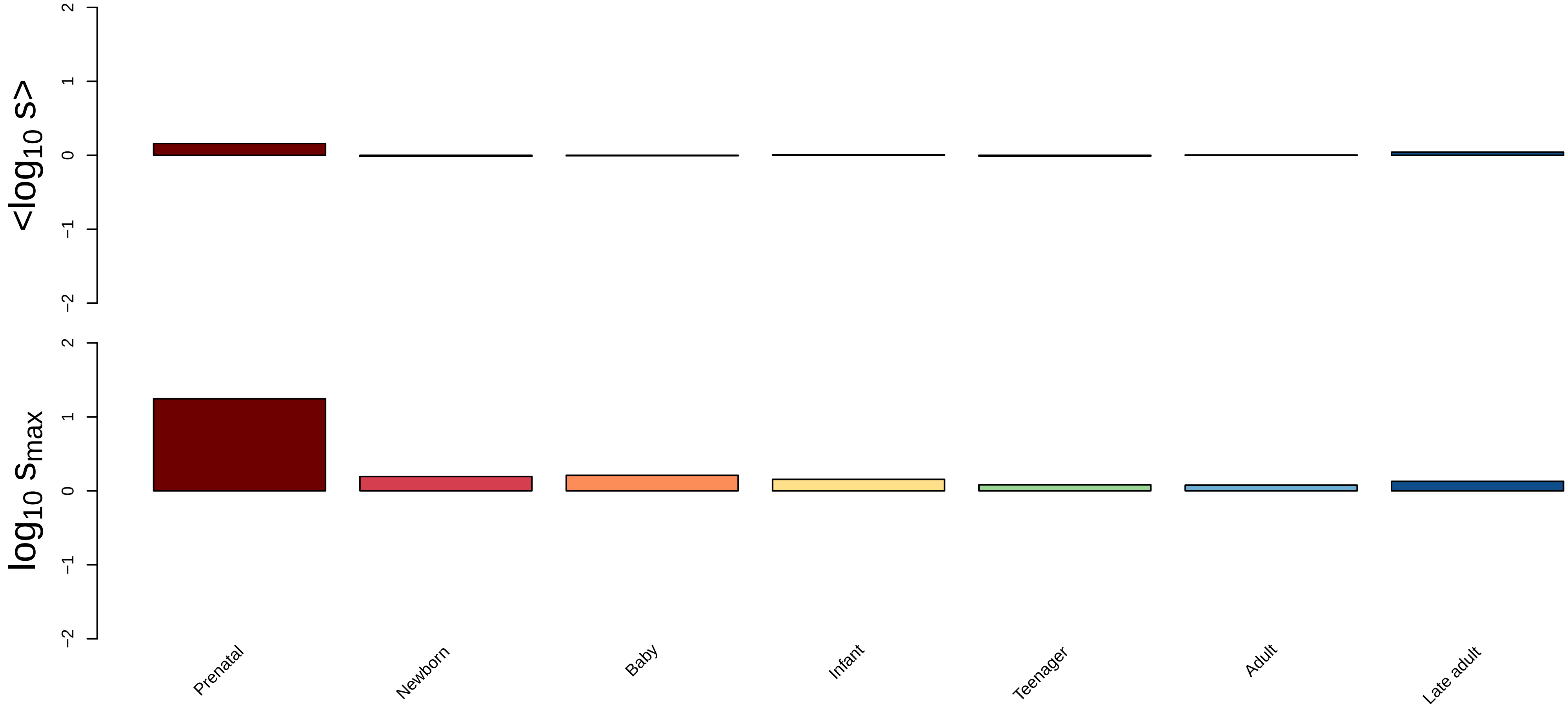

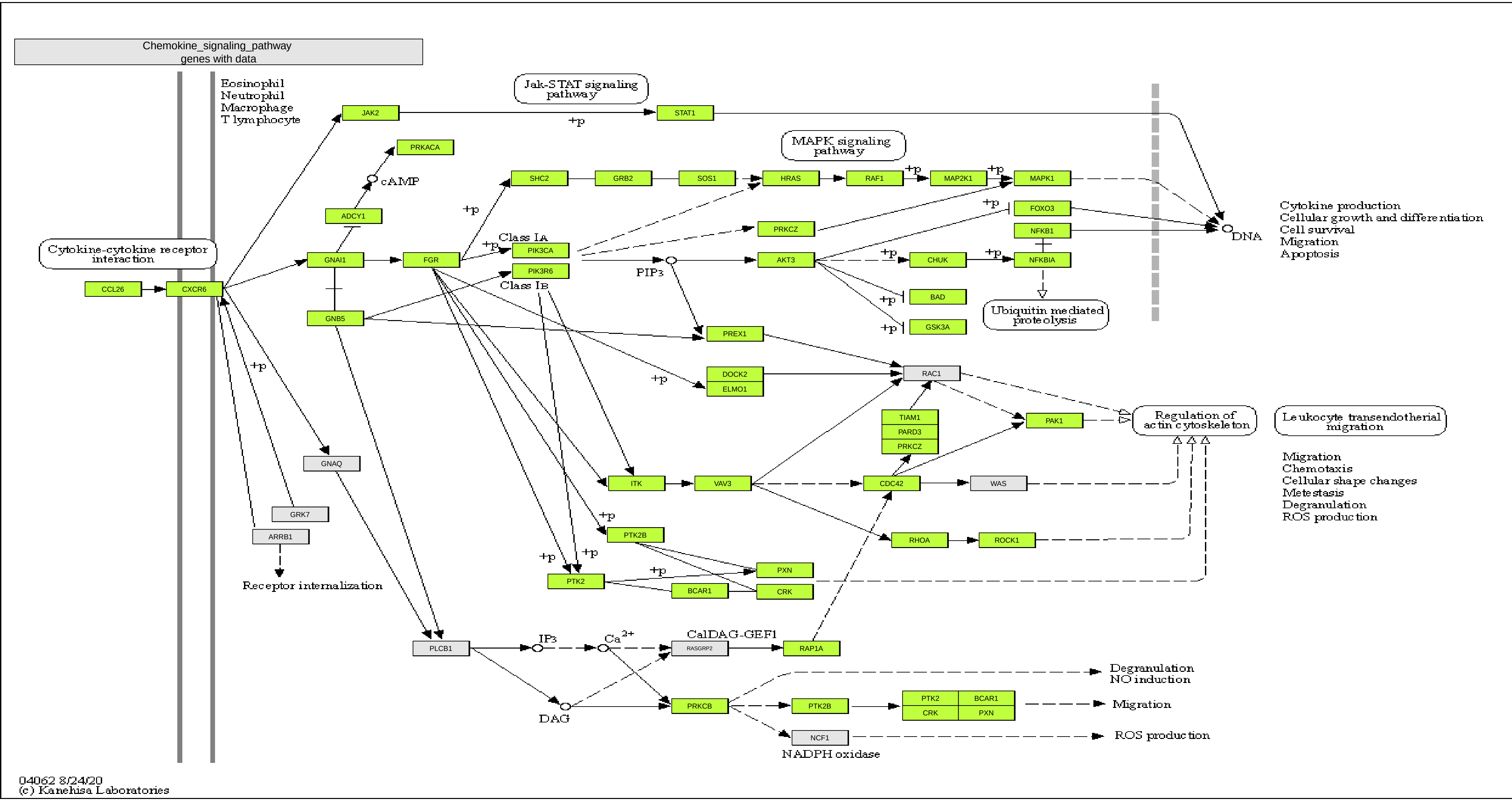
Chemokine_signaling_pathway
genes with data
Eosinophil
Neutrophil
Macrophage
T lymphocyte
Jak-STAT signaling pathway
JAK2
STAT1
+p
PRKACA
cAMP
ADCY1
MAPK signaling pathway
SHC2
GRB2
SOS1
HRAS
RAF1
MAP2K1
MAPK1
FOXO3
NFKB1
PRKCZ
Class IA
PIK3CA
PIK3R6
Class IB
PIP3
AKT3
CHUK
NFKBIA
BAD
GSK3A
Ubiquitin mediated proteolysis
DNA
Cytokine production
Cellular growth and differentiation
Cell survival
Migration
Apoptosis
Cytokine-cytokine receptor interaction
CCL26
CXCR6
GNAI1
FGR
GNB5
PREX1
DOCK2
ELMO1
RAC1
TIAM1
PARD3
PRKCZ
PAK1
Regulation of actin cytoskeleton
Leukocyte transendothelial migration
Migration
Chemotaxis
Cellular shape changes
Metestasis
Degranulation
ROS production
GNAQ
GRK7
ARRB1
Receptor internalization
ITK
VAV3
CDC42
WAS
RHOA
ROCK1
PTK2B
PXN
CRK
PTK2
BCAR1
PLCB1
IP3
Ca2+
CalDAG-GEF1
RASGRP2
RAP1A
DAG
PRKCB
PTK2B
PTK2
BCAR1
CRK
PXN
NCF1
NADPH oxidase
Degranulation
NO induction
Migration
ROS production
04062 8/24/20
(c) Kanehisa Laboratories

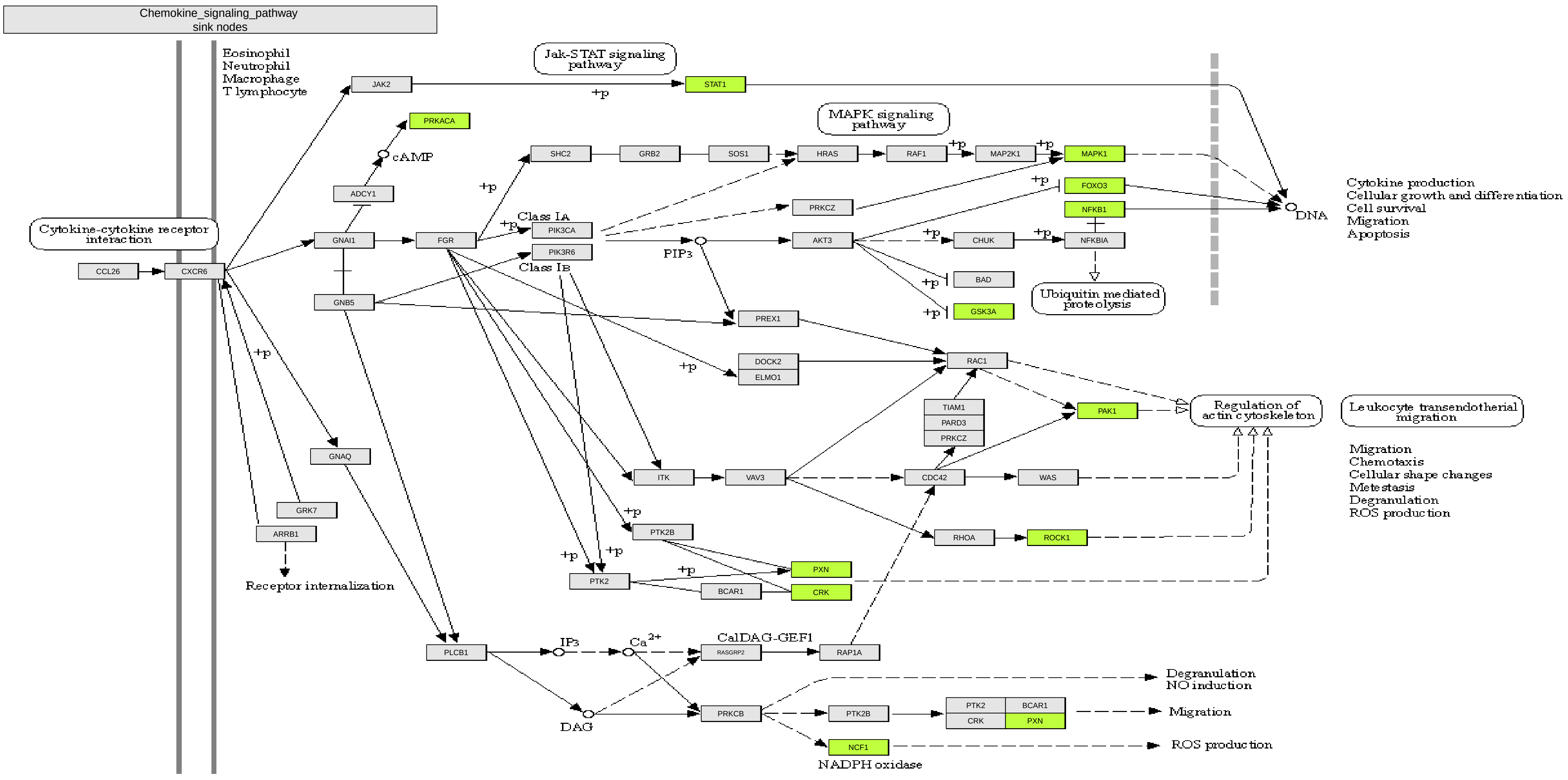

04062 8/24/20

(c) Kanehisa Laboratories

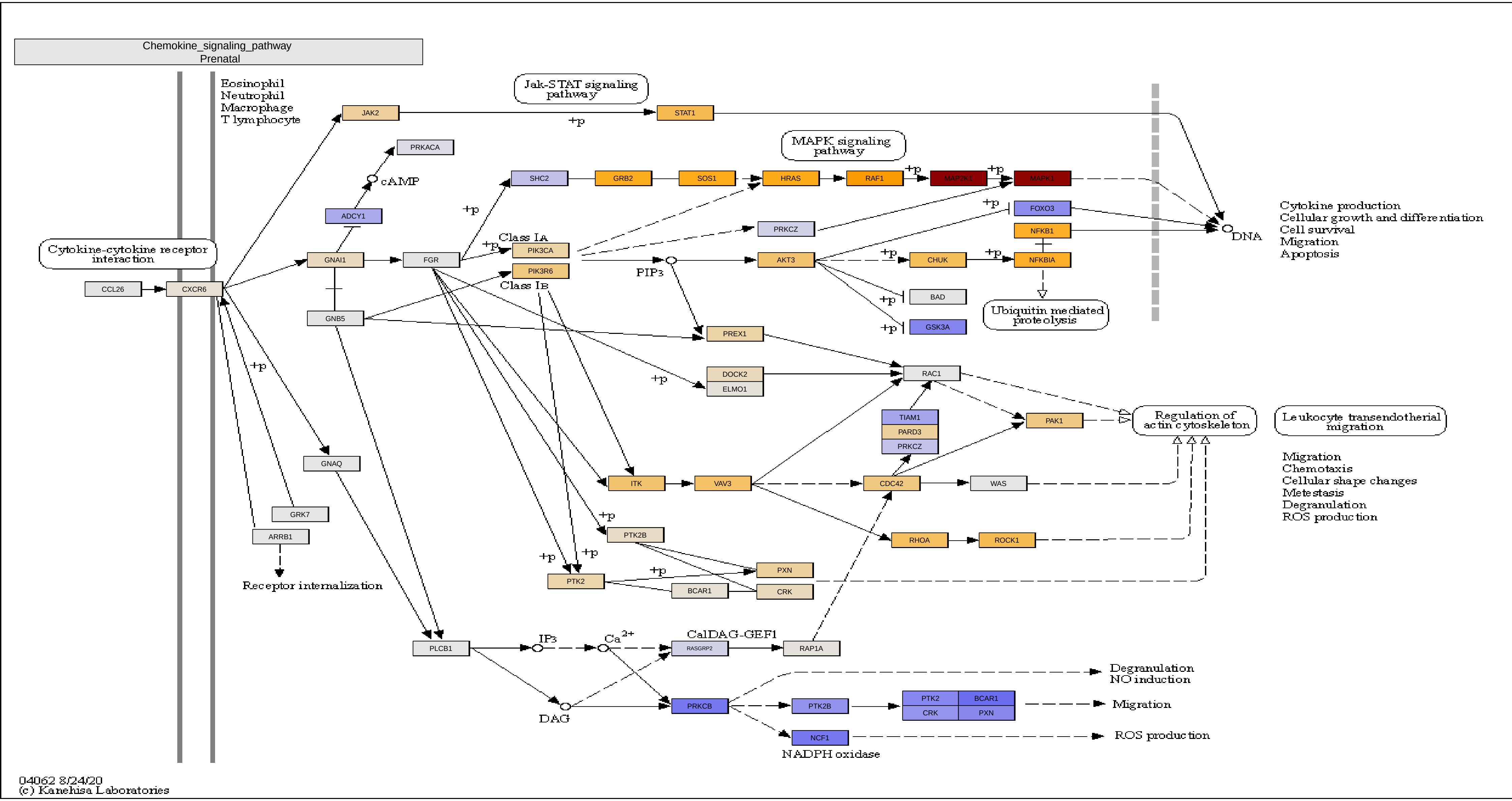
Chemokine_signaling_pathway
Prenatal
Eosinophil
Neutrophil
Macrophage
T lymphocyte
Jak-STAT signaling pathway
MAPK signaling pathway
Cytokine-cytokine receptor interaction
CCL26
CXCR6
GNAI1
GNB5
FGR
ADCY1
cAMP
PRKACA
JAK2
STAT1
SHC2
GRB2
SOS1
HRAS
RAF1
MAP2K1
MAPK1
PRKCZ
FOXO3
NFKB1
NFKBIA
CHUK
AKT3
BAD
GSK3A
PIK3CA
PIK3R6
Class IA
Class IB
PIP3
PREX1
DOCK2
ELMO1
RAC1
TIAM1
PARD3
PRKCZ
PAK1
CDC42
WAS
ITK
VAV3
RHOA
ROCK1
PTK2B
PTK2
PXN
CRK
BCAR1
GNAQ
GRK7
ARRB1
Receptor internalization
PLCB1
IP3
Ca2+
CalDAG-GEF1
RASGRP2
RAP1A
DAG
PRKCB
PTK2B
NCF1
NADPH oxidase
PTK2
BCAR1
CRK
PXN
DNA
Cytokine production
Cellular growth and differentiation
Cell survival
Migration
Apoptosis
Ubiquitin mediated proteolysis
Regulation of actin cytoskeleton
Leukocyte transendothelial migration
Migration
Chemotaxis
Cellular shape changes
Metestasis
Degranulation
ROS production
Degranulation
NO induction
Migration
ROS production
04062 8/24/20
(c) Kanehisa Laboratories

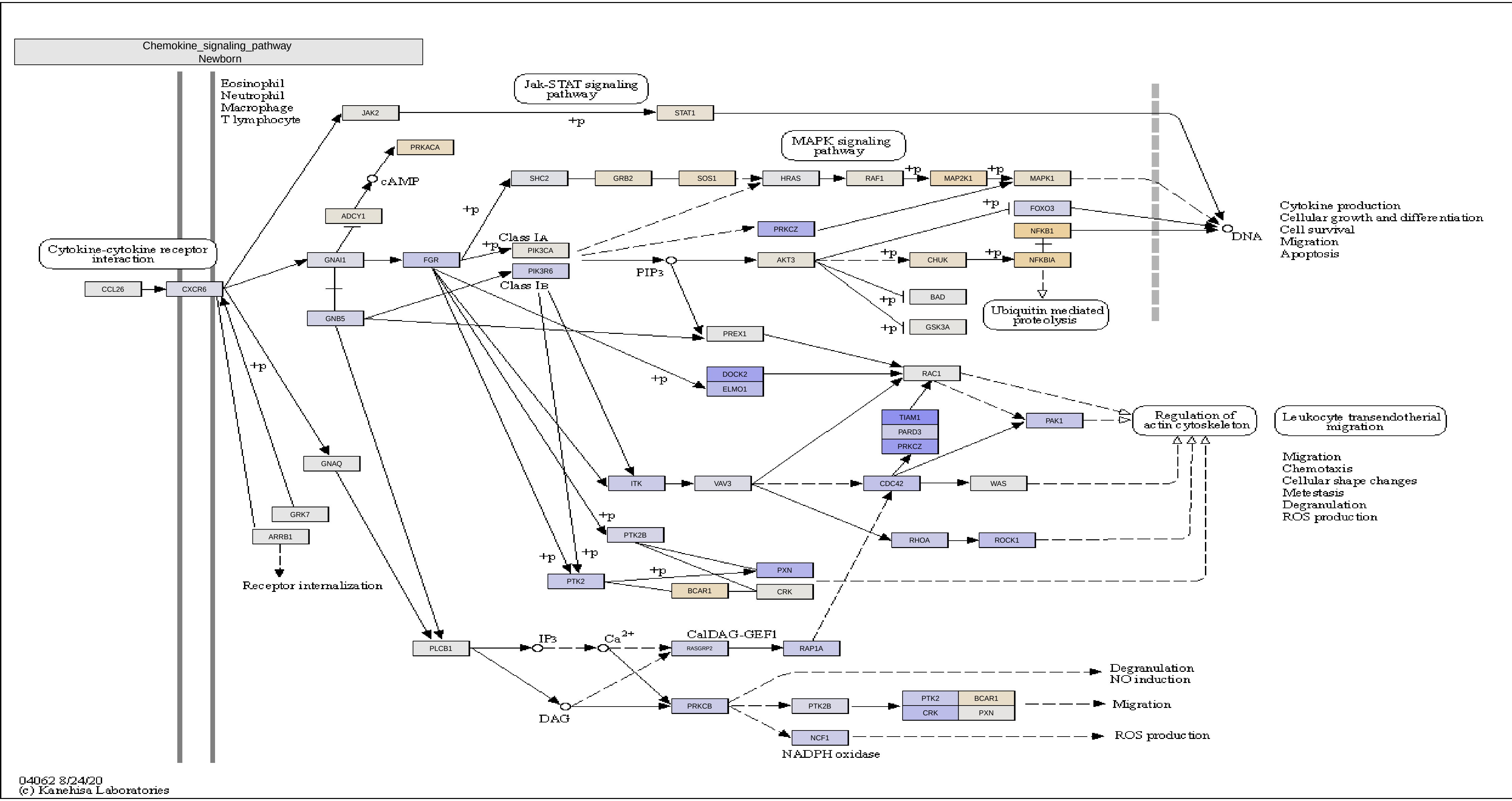
Chemokine_signaling_pathway
Newborn
Eosinophil
Neutrophil
Macrophage
T lymphocyte
Jak-STAT signaling pathway
JAK2
STAT1
+p
PRKACA
cAMP
ADCY1
MAPK signaling pathway
SHC2
GRB2
SOS1
HRAS
RAF1
MAP2K1
MAPK1
+p
FOXO3
NFKB1
PRKCZ
Class IA
PIK3CA
PIK3R6
Class IB
GNAI1
FGR
PIP3
AKT3
CHUK
NFKBIA
BAD
GSK3A
Ubiquitin mediated proteolysis
DNA
Cytokine production
Cellular growth and differentiation
Cell survival
Migration
Apoptosis
Cytokine-cytokine receptor interaction
CCL26
CXCR6
GNB5
PREX1
RAC1
DOCK2
ELMO1
TIAM1
PARD3
PRKCZ
PAK1
Regulation of actin cytoskeleton
Leukocyte transendothelial migration
Migration
Chemotaxis
Cellular shape changes
Metestasis
Degranulation
ROS production
GNAQ
GRK7
ARRB1
Receptor internalization
ITK
VAV3
CDC42
WAS
PTK2B
RHOA
ROCK1
PXN
PTK2
BCAR1
CRK
PLCB1
IP3
Ca2+
CalDAG-GEF1
RASGRP2
RAP1A
DAG
PRKCB
PTK2B
NCF1
NADPH oxidase
Degranulation
NO induction
Migration
ROS production
04062 8/24/20
(c) Kanehisa Laboratories

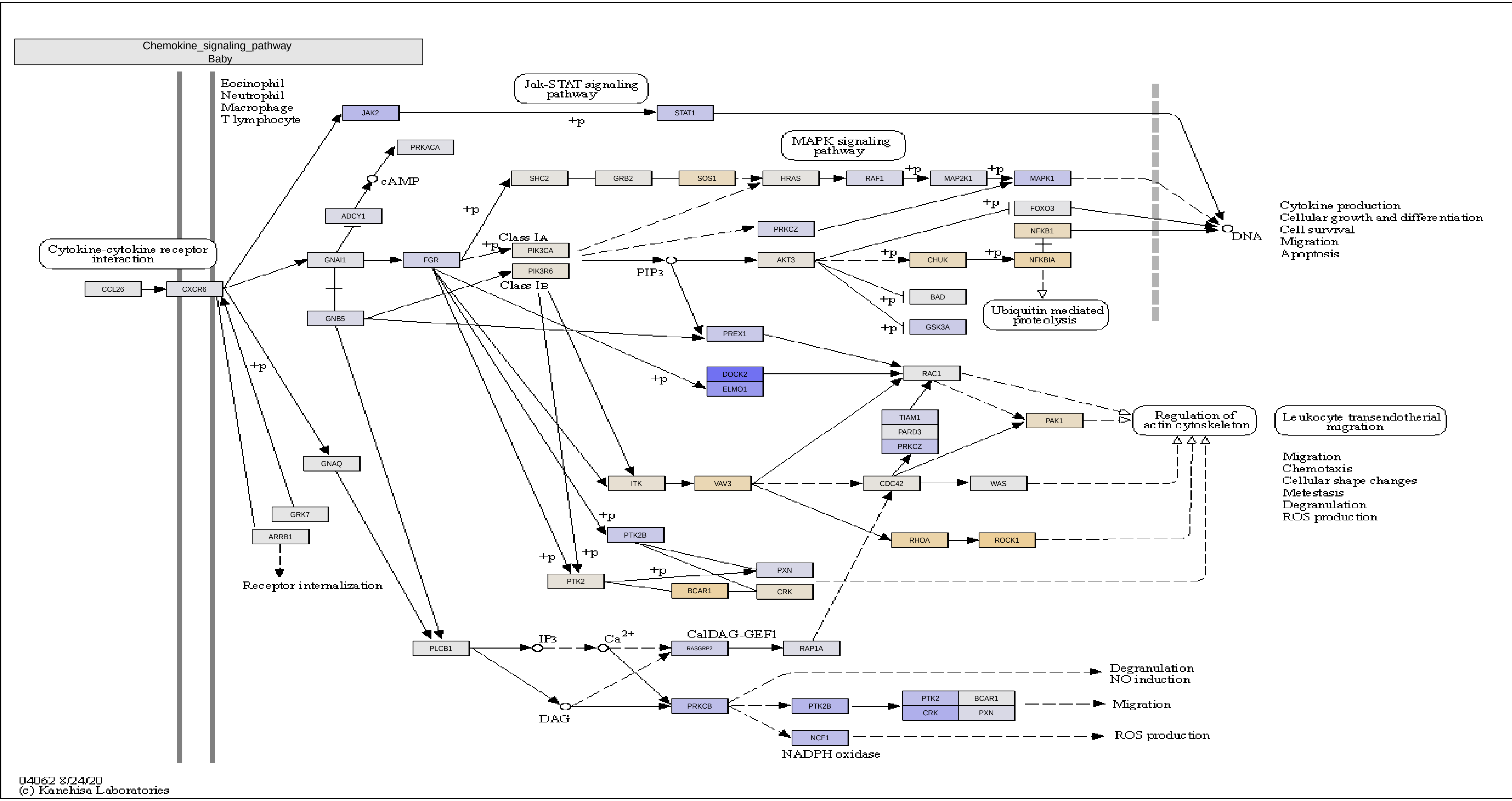
Chemokine_signaling_pathway
Baby
Eosinophil
Neutrophil
Macrophage
T lymphocyte
Jak-STAT signaling pathway
JAK2
STAT1
+p
PRKACA
cAMP
ADCY1
MAPK signaling pathway
SHC2
GRB2
SOS1
HRAS
RAF1
MAP2K1
MAPK1
+p
FOXO3
NFKB1
PRKCZ
Class IA
PIK3CA
PIK3R6
Class IB
PIP3
AKT3
CHUK
NFKBIA
BAD
GSK3A
Ubiquitin mediated proteolysis
DNA
Cytokine production
Cellular growth and differentiation
Cell survival
Migration
Apoptosis
Cytokine-cytokine receptor interaction
CCL26
CXCR6
GNAI1
FGR
GNB5
PREX1
DOCK2
ELMO1
RAC1
TIAM1
PARD3
PRKCZ
PAK1
Regulation of actin cytoskeleton
Leukocyte transendothelial migration
Migration
Chemotaxis
Cellular shape changes
Metestasis
Degranulation
ROS production
GNAQ
GRK7
ARRB1
Receptor internalization
ITK
VAV3
CDC42
WAS
RHOA
ROCK1
PTK2B
PXN
PTK2
BCAR1
CRK
PLCB1
IP3
Ca2+
CalDAG-GEF1
RASGRP2
RAP1A
DAG
PRKCB
PTK2B
NCF1
NADPH oxidase
Degranulation
NO induction
Migration
ROS production
04062 8/24/20
(c) Kanehisa Laboratories

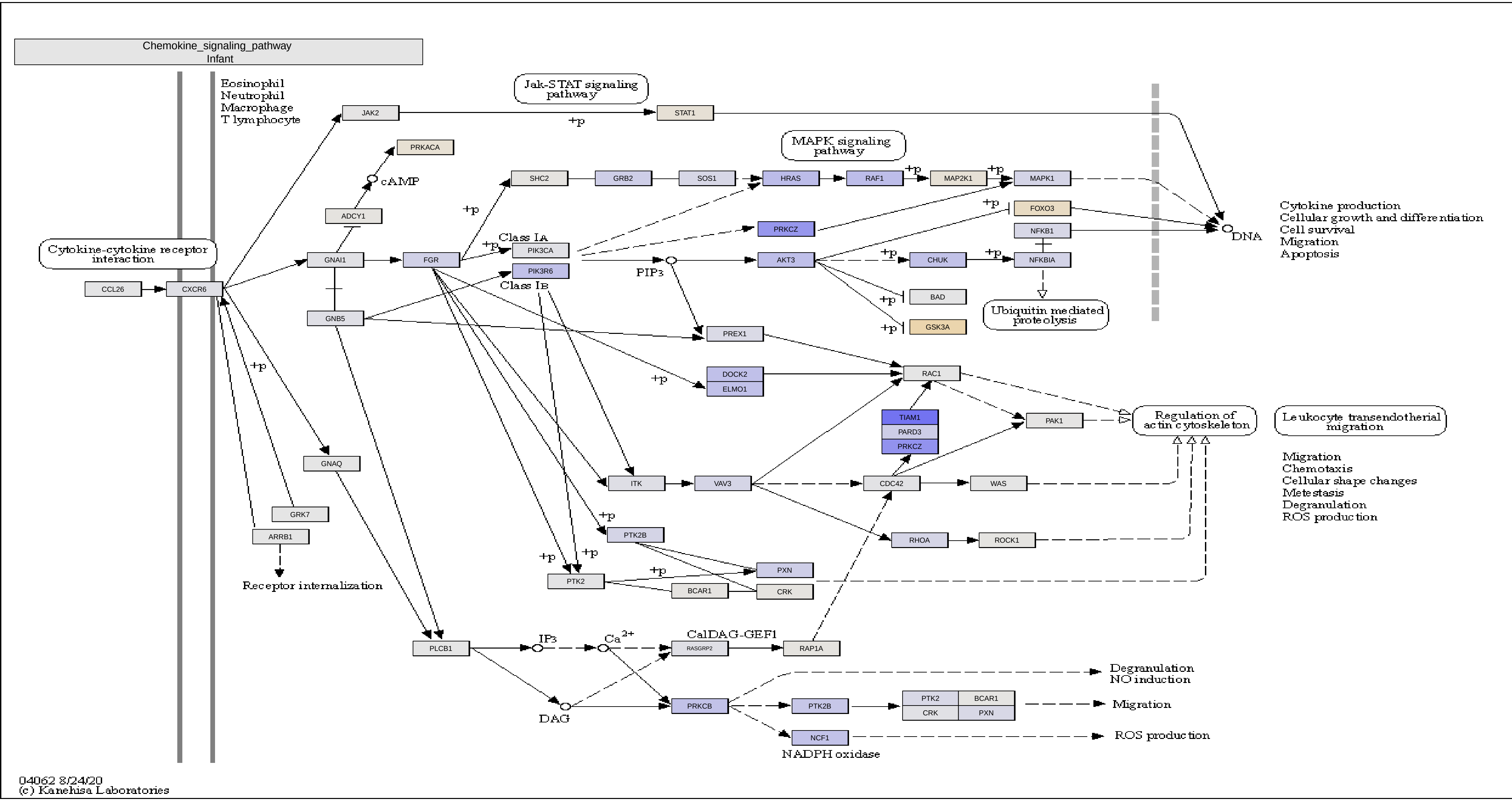
Chemokine_signaling_pathway
Infant
Eosinophil
Neutrophil
Macrophage
T lymphocyte
Jak-STAT signaling pathway
MAPK signaling pathway
Cytokine-cytokine receptor interaction
Ubiquitin mediated proteolysis
Regulation of actin cytoskeleton
Leukocyte transendothelial migration
CCL26
CXCR6
JAK2
STAT1
PRKACA
cAMP
ADCY1
GNAI1
GNB5
FGR
SHC2
GRB2
SOS1
HRAS
RAF1
MAP2K1
MAPK1
PRKCZ
FOXO3
NFKB1
NFKBIA
CHUK
AKT3
BAD
GSK3A
Class IA
PIK3CA
PIK3R6
Class IB
PIP3
PREX1
DOCK2
ELMO1
RAC1
PAK1
TIAM1
PARD3
PRKCZ
CDC42
WAS
RHOA
ROCK1
ITK
VAV3
PTK2B
PXN
CRK
BCAR1
PTK2
GNAQ
GRK7
ARRB1
Receptor internalization
PLCB1
IP3
Ca2+
CalDAG-GEF1
RASGRP2
RAP1A
DAG
PRKCB
PTK2B
NCF1
NADPH oxidase
PTK2
BCAR1
CRK
PXN
+p
DNA
Cytokine production
Cellular growth and differentiation
Cell survival
Migration
Apoptosis
Migration
Chemotaxis
Cellular shape changes
Metestasis
Degranulation
ROS production
Degranulation
NO induction
Migration
ROS production
04062 8/24/20
(c) Kanehisa Laboratories

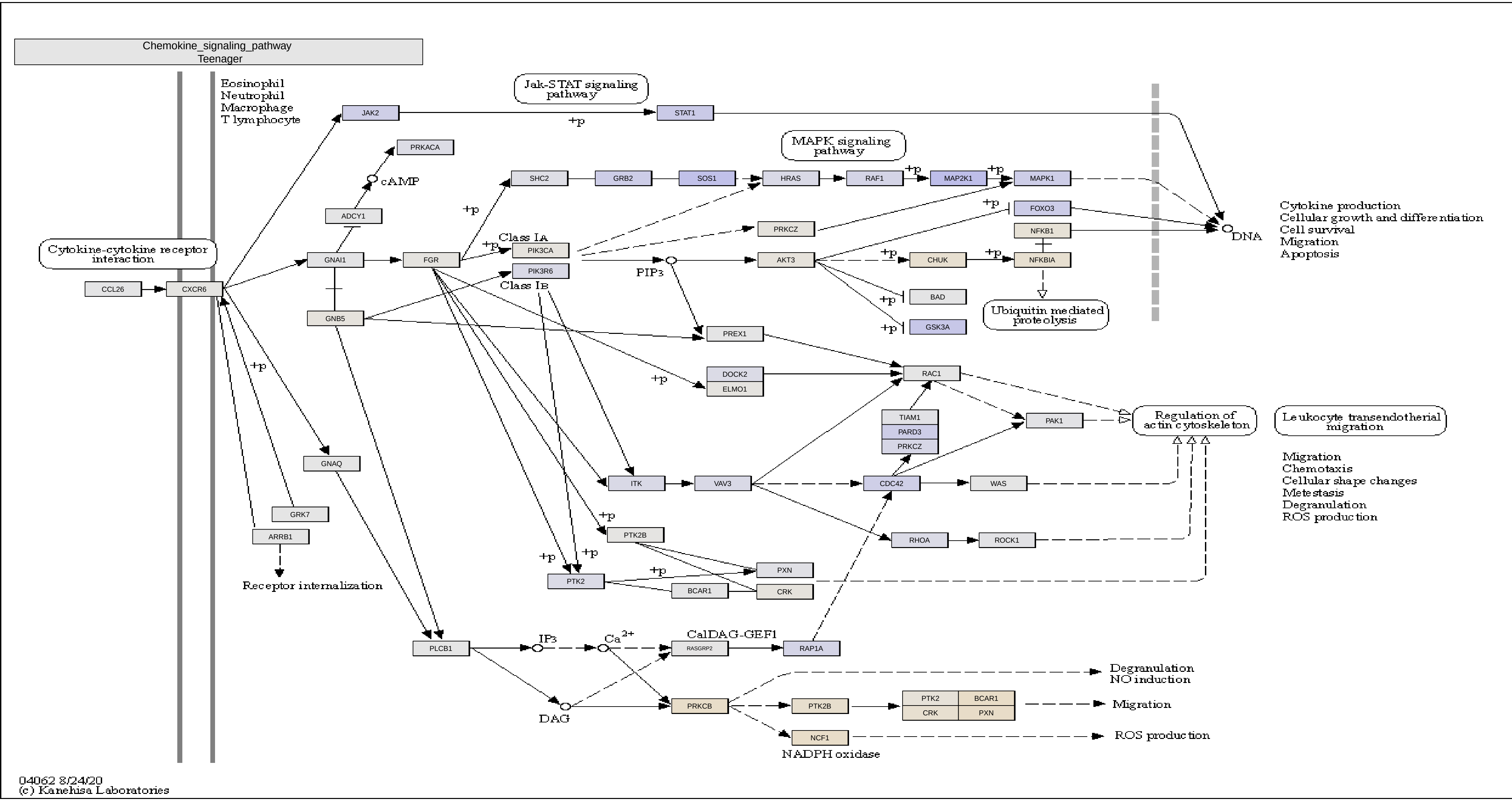
Chemokine_signaling_pathway
Teenager
Eosinophil
Neutrophil
Macrophage
T lymphocyte
Jak-STAT signaling pathway
JAK2
STAT1
+p
PRKACA
cAMP
ADCY1
MAPK signaling pathway
SHC2
GRB2
SOS1
HRAS
RAF1
MAP2K1
MAPK1
FOXO3
NFKB1
PRKCZ
Class IA
PIK3CA
PIK3R6
Class IB
PIP3
AKT3
CHUK
NFKBIA
BAD
GSK3A
Ubiquitin mediated proteolysis
DNA
Cytokine production
Cellular growth and differentiation
Cell survival
Migration
Apoptosis
Cytokine-cytokine receptor interaction
CCL26
CXCR6
GNAI1
FGR
GNB5
PREX1
RAC1
DOCK2
ELMO1
TIAM1
PARD3
PRKCZ
PAK1
Regulation of actin cytoskeleton
Leukocyte transendothelial migration
Migration
Chemotaxis
Cellular shape changes
Metestasis
Degranulation
ROS production
GNAQ
GRK7
ARRB1
Receptor internalization
ITK
VAV3
CDC42
WAS
RHOA
ROCK1
PTK2B
PXN
PTK2
BCAR1
CRK
PLCB1
IP3
Ca^{2+}
CalDAG-GEF1
RASGRP2
RAP1A
DAG
PRKCB
PTK2B
NCF1
NADPH oxidase
PTK2
BCAR1
CRK
PXN
Degranulation
NO induction
Migration
ROS production
04062 8/24/20
(c) Kanehisa Laboratories

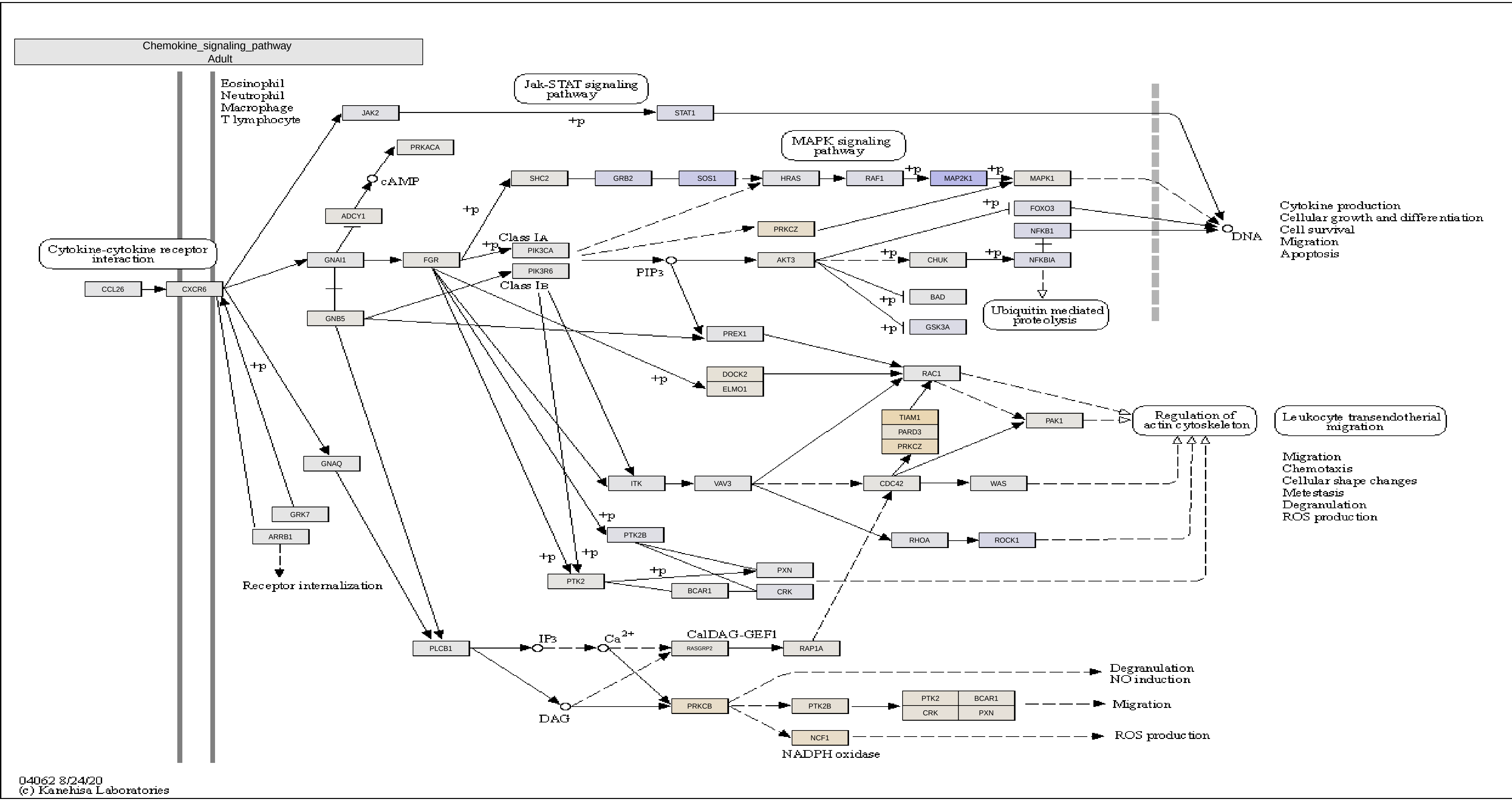
Chemokine_signaling_pathway
Adult
Eosinophil
Neutrophil
Macrophage
T lymphocyte
Jak-STAT signaling pathway
JAK2
STAT1
+p
PRKACA
cAMP
ADCY1
MAPK signaling pathway
SHC2
GRB2
SOS1
HRAS
RAF1
MAP2K1
MAPK1
+p
PRKCZ
FOXO3
NFKB1
Class IA
PIK3CA
PIK3R6
Class IB
PIP3
AKT3
CHUK
NFKBIA
BAD
GSK3A
Ubiquitin mediated proteolysis
DNA
Cytokine production
Cellular growth and differentiation
Cell survival
Migration
Apoptosis
Cytokine-cytokine receptor interaction
CCL26
CXCR6
GNAI1
FGR
GNB5
PREX1
DOCK2
ELMO1
RAC1
TIAM1
PARD3
PRKCZ
PAK1
Regulation of actin cytoskeleton
Leukocyte transendothelial migration
Migration
Chemotaxis
Cellular shape changes
Metestasis
Degranulation
ROS production
GNAQ
GRK7
ARRB1
Receptor internalization
ITK
VAV3
CDC42
WAS
RHOA
ROCK1
PTK2B
PXN
CRK
PTK2
BCAR1
PLCB1
IP3
Ca2+
CalDAG-GEF1
RASGRP2
RAP1A
DAG
PRKCB
PTK2B
PTK2
BCAR1
CRK
PXN
NCF1
NADPH oxidase
Degranulation
NO induction
Migration
ROS production
04062 8/24/20
(c) Kanehisa Laboratories

Chemokine_signaling_pathway
Late adult

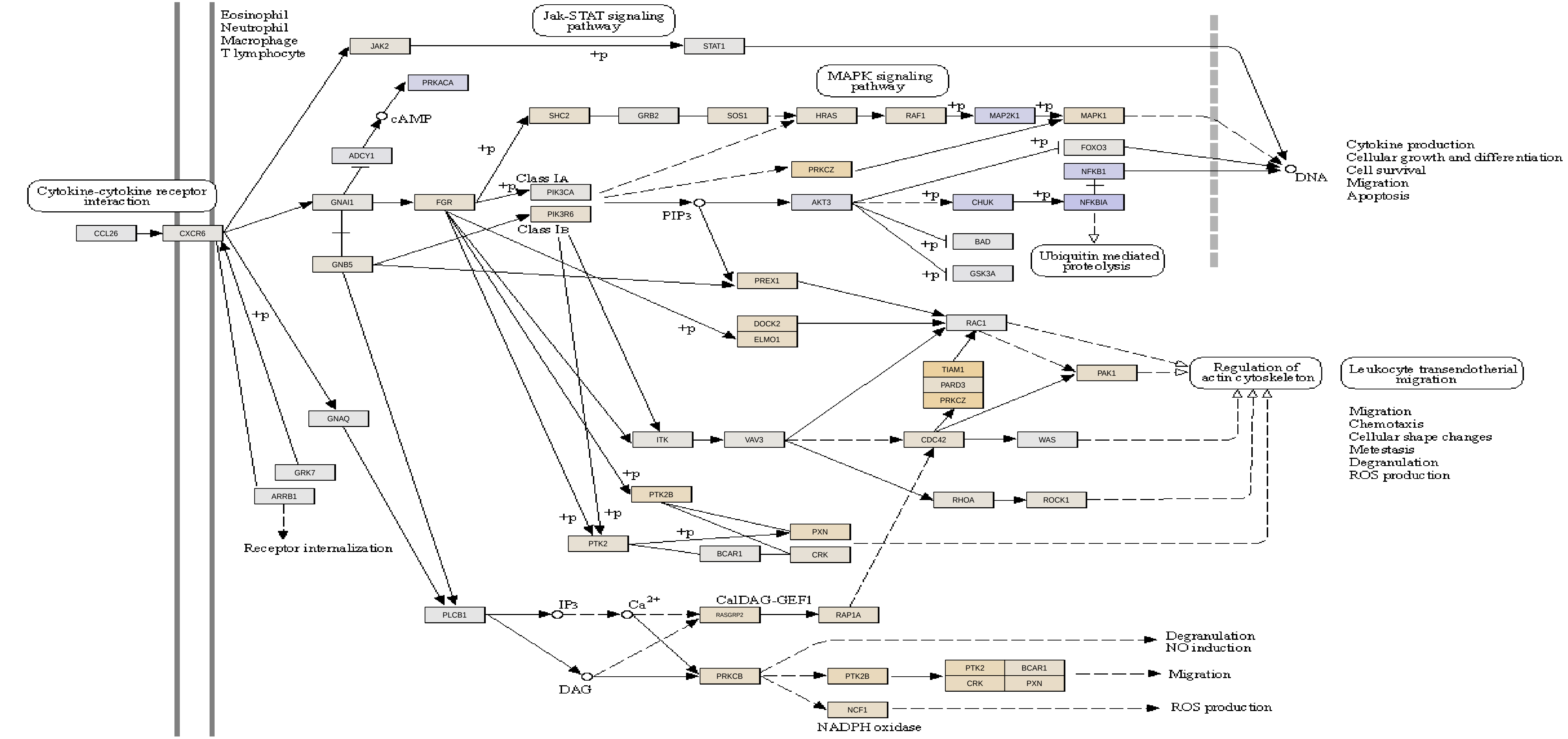

04062 8/24/20
(c) Kanehisa Laboratories
