# Supplementary material for: The Transcriptome and Methylome of the Developing and Aging Brain and Their Relations to Gliomas and Psychological Disorders
Source: Cells. 2022 Jan 21;11(3):362. doi: 10.3390/cells11030362 (PMC8834030; doi:10.3390/cells11030362)
Supplement: Supplementary file 1 [file cells-11-00362-s001.zip › Suppl-Material-S4-Pathways-PSF_Methylation/FoxO_signaling_pathway.pdf]

FoxO signaling pathway

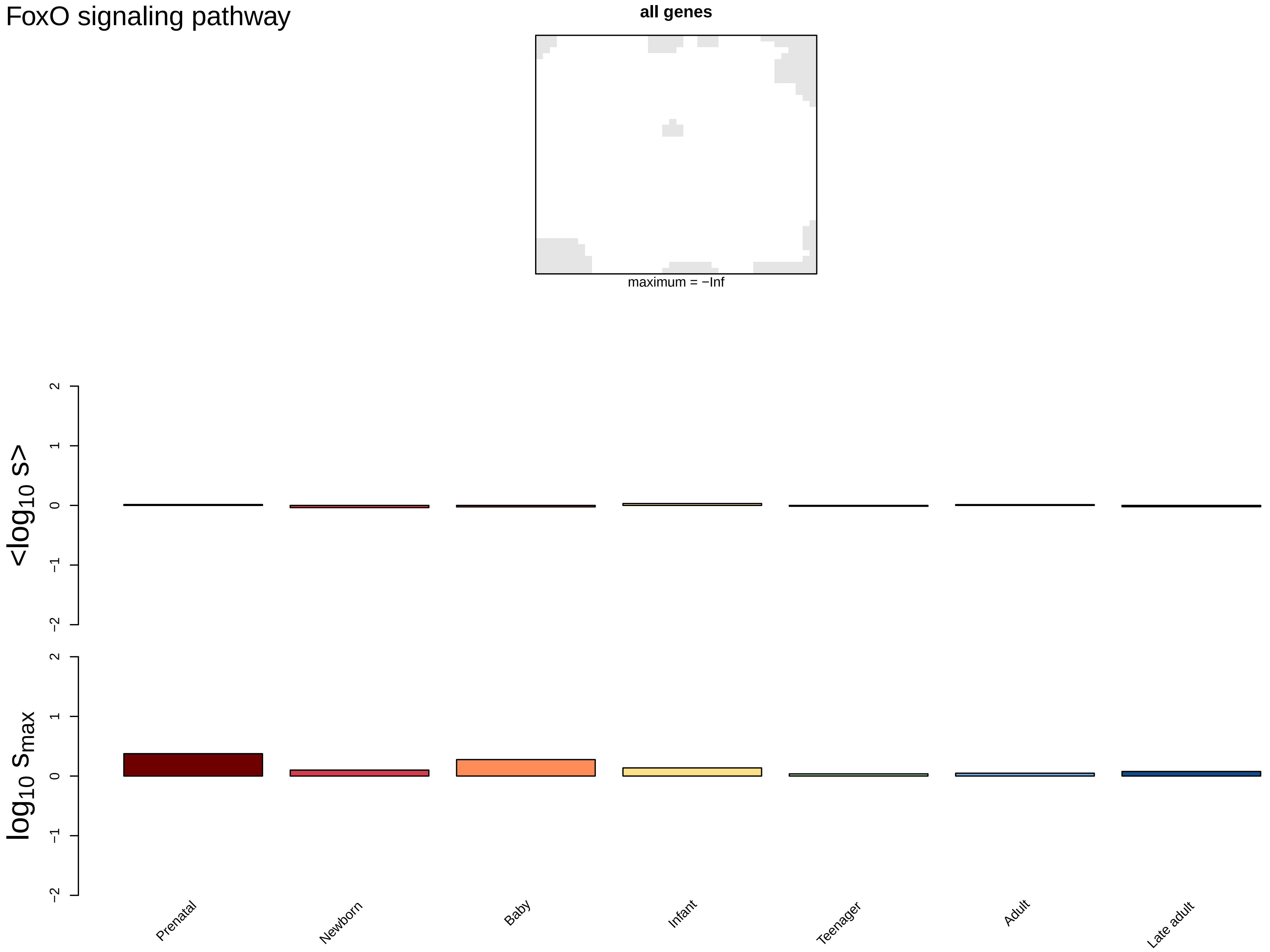

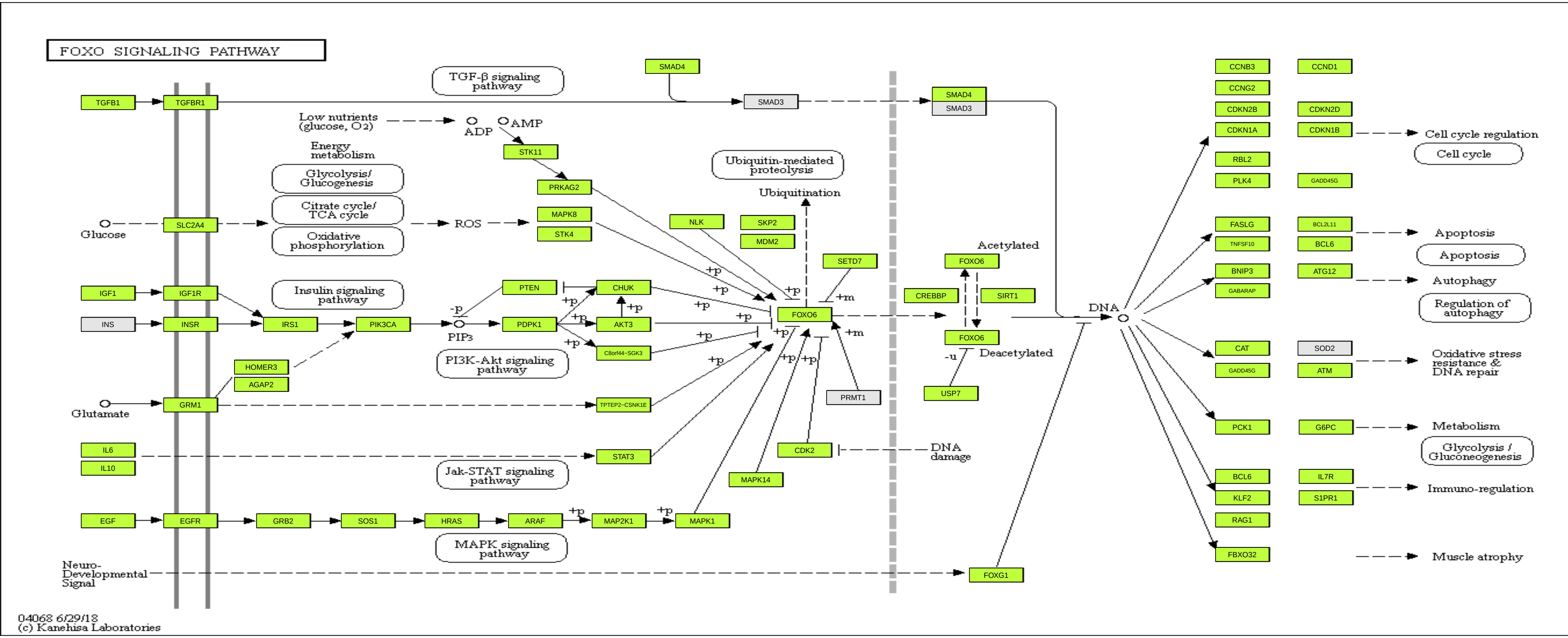
FOXO SIGNALING PATHWAY
TGF-β signaling pathway
SMAD4
TGFB1
TGFBR1
SMAD3
SMAD4
SMAD3
Low nutrients (glucose, O2)
ADP
AMP
STK11
Energy metabolism
Glycolysis/ Gluconeogenesis
Citrate cycle/ TCA cycle
Oxidative phosphorylation
PRKAG2
Ubiquitin-mediated proteolysis
Ubiquitination
Glucose
SLC2A4
ROS
MAPK8
STK4
NLK
SKP2
MDM2
SETD7
IGF1
IGF1R
Insulin signaling pathway
PTEN
CHUK
INS
INSR
IRS1
PIK3CA
PIP3
PDPK1
AKT3
FOXO6
C8orf44-SGK3
PI3K-Akt signaling pathway
HOMER3
AGAP2
Glutamate
GRM1
TPTEP2-CSNK1E
PRMT1
IL6
IL10
STAT3
CDK2
DNA damage
Jak-STAT signaling pathway
MAPK14
EGF
EGFR
GRB2
SOS1
HRAS
ARAF
MAP2K1
MAPK1
MAPK signaling pathway
Neuro-Developmental Signal
FOXG1
Acetylated
FOXO6
CREBBP
SIRT1
FOXO6
Deacetylated
USP7
DNA
CCNB3
CCND1
CCNG2
CDKN2B
CDKN2D
CDKN1A
CDKN1B
Cell cycle regulation
Cell cycle
RBL2
PLK4
GADD45G
FASLG
TNFSF10
BCL2L11
BCL6
Apoptosis
Apoptosis
BNIP3
GABARAP
ATG12
Autophagy
Regulation of autophagy
CAT
GADD45G
SOD2
ATM
Oxidative stress resistance & DNA repair
PCK1
G6PC
Metabolism
Glycolysis / Gluconeogenesis
BCL6
KLF2
IL7R
S1PR1
Immuno-regulation
RAG1
FBXO32
Muscle atrophy
04068 6/29/18
(c) Kanehisa Laboratories

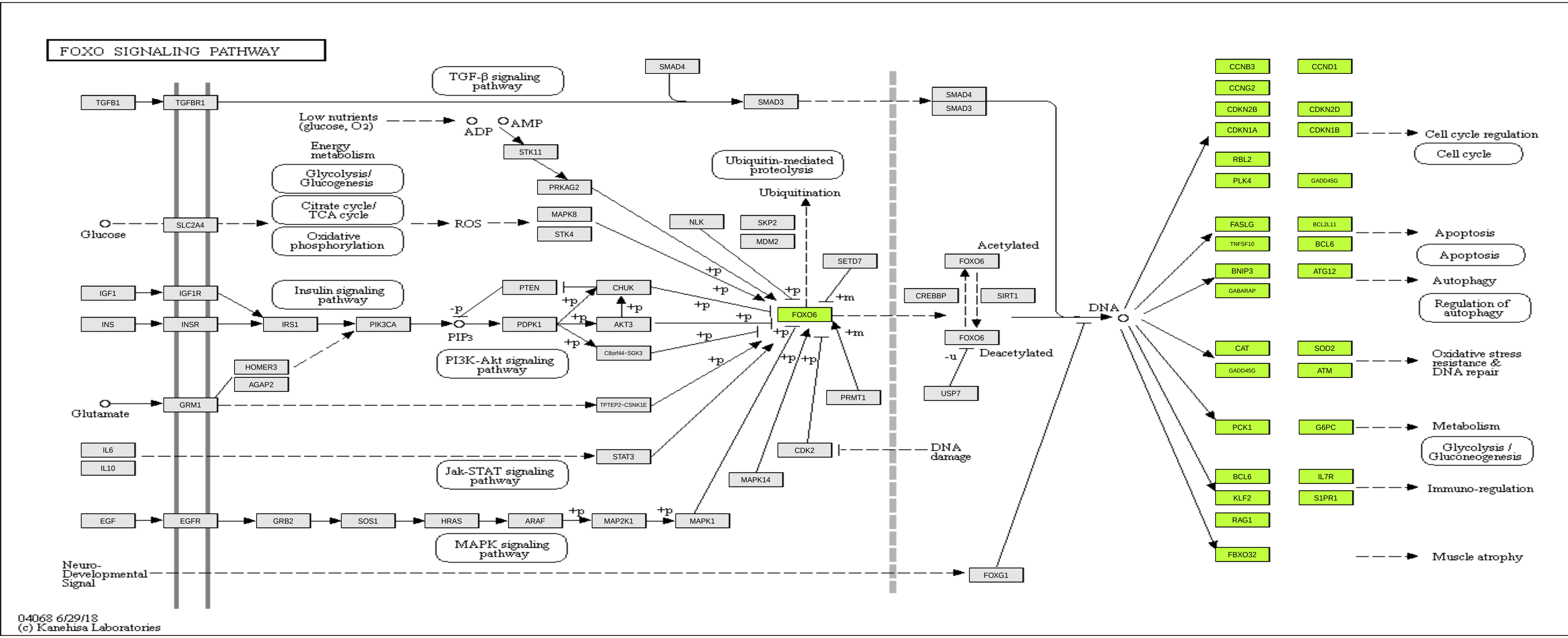
FOXO SIGNALING PATHWAY
TGFB1
TGFBR1
TGF-β signaling pathway
SMAD4
SMAD3
SMAD4
SMAD3
Low nutrients (glucose, O2)
ADP
AMP
STK11
Energy metabolism
Glycolysis/ Glucogenesis
Citrate cycle/ TCA cycle
Oxidative phosphorylation
PRKAG2
Glucose
SLC2A4
ROS
MAPK8
STK4
NLK
SKP2
MDM2
Ubiquitin-mediated proteolysis
Ubiquitination
SETD7
IGF1
IGF1R
Insulin signaling pathway
INS
INSR
IRS1
PIK3CA
-p
PIP3
PTEN
PDPK1
CHUK
AKT3
C8orf44-SGK3
+p
+m
FOXO6
HOMER3
AGAP2
PI3K-Akt signaling pathway
Glutamate
GRM1
TPTEP2-CSNK1E
PRMT1
IL6
IL10
STAT3
Jak-STAT signaling pathway
CDK2
DNA damage
MAPK14
EGF
EGFR
GRB2
SOS1
HRAS
ARAF
MAP2K1
MAPK1
MAPK signaling pathway
Neuro- Developmental Signal
FOXG1
Acetylated
FOXO6
CREBBP
SIRT1
FOXO6
-u
Deacetylated
USP7
DNA
CCNB3
CCND1
CCNG2
CDKN2B
CDKN2D
CDKN1A
CDKN1B
Cell cycle regulation
Cell cycle
RBL2
PLK4
GADD45G
FASLG
BCL2L11
TNFSF10
BCL6
Apoptosis
Apoptosis
BNIP3
ATG12
GABARAP
Autophagy
Regulation of autophagy
CAT
SOD2
GADD45G
ATM
Oxidative stress resistance & DNA repair
PCK1
G6PC
Metabolism
Glycolysis / Gluconeogenesis
BCL6
IL7R
KLF2
S1PR1
Immuno-regulation
RAG1
FBXO32
Muscle atrophy
04068 6/29/18
(c) Kanehisa Laboratories

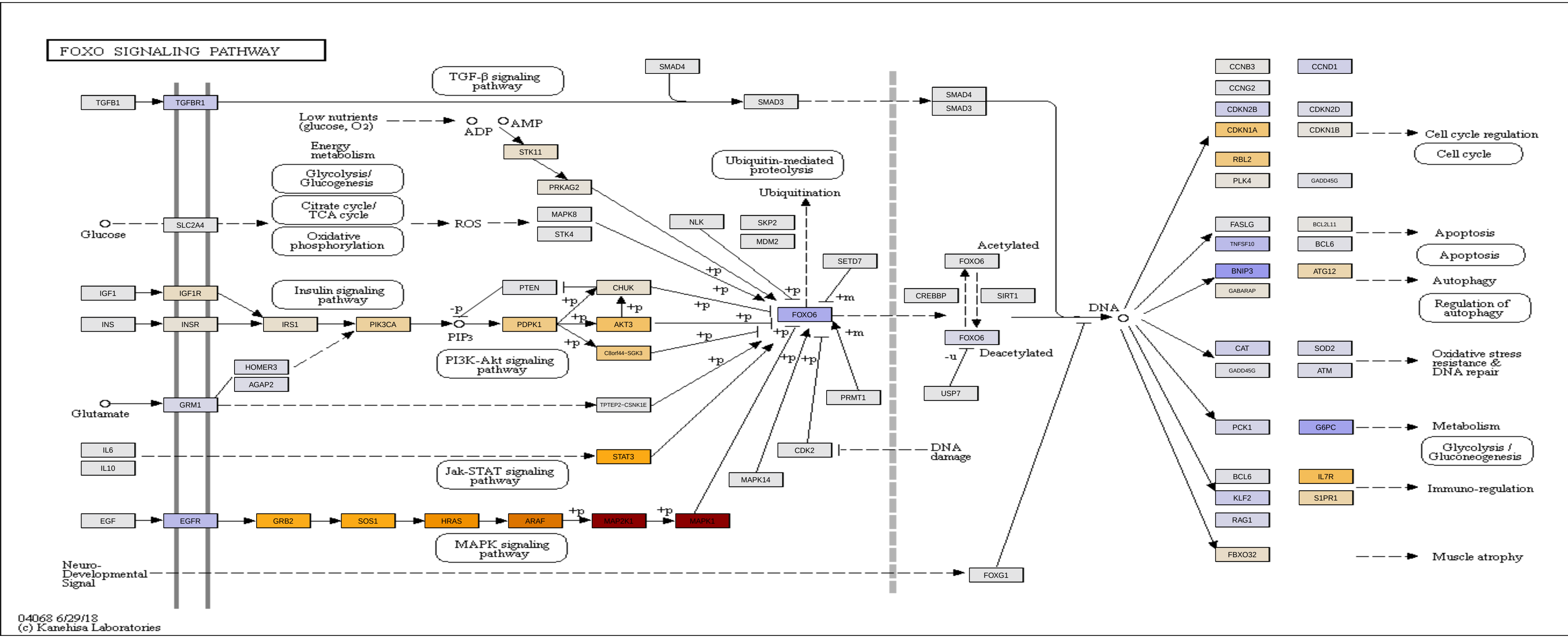
FOXO SIGNALING PATHWAY
TGFB1
TGFBR1
TGF-β signaling pathway
SMAD4
SMAD3
SMAD4
SMAD3
Low nutrients (glucose, O2)
ADP
AMP
STK11
Energy metabolism
Glycolysis/ Glucogenesis
Citrate cycle/ TCA cycle
Oxidative phosphorylation
PRKAG2
Glucose
SLC2A4
ROS
MAPK8
STK4
Ubiquitin-mediated proteolysis
Ubiquitination
NLK
SKP2
MDM2
SETD7
IGF1
IGF1R
Insulin signaling pathway
INS
INSR
IRS1
PIK3CA
-p
PIP3
PTEN
CHUK
PDPK1
AKT3
C8orf44-SGK3
+p
+m
FOXO6
PI3K-Akt signaling pathway
HOMER3
AGAP2
Glutamate
GRM1
TPTEP2-CSNK1E
IL6
IL10
STAT3
Jak-STAT signaling pathway
CDK2
DNA damage
MAPK14
PRMT1
EGF
EGFR
GRB2
SOS1
HRAS
ARAF
MAP2K1
MAPK1
MAPK signaling pathway
Neuro-Developmental Signal
Acetylated
FOXO6
CREBBP
SIRT1
FOXO6
-u
Deacetylated
USP7
FOXG1
DNA
CCNB3
CCNG2
CDKN2B
CDKN1A
RBL2
PLK4
CCND1
CDKN2D
CDKN1B
GADD45G
Cell cycle regulation
Cell cycle
FASLG
TNFSF10
BCL2L11
BCL6
Apoptosis
Apoptosis
BNIP3
GABARAP
ATG12
Autophagy
Regulation of autophagy
CAT
GADD45G
SOD2
ATM
Oxidative stress resistance & DNA repair
PCK1
G6PC
Metabolism
Glycolysis / Gluconeogenesis
BCL6
KLF2
RAG1
IL7R
S1PR1
Immuno-regulation
FBXO32
Muscle atrophy
04068 6/29/18
(c) Kanehisa Laboratories

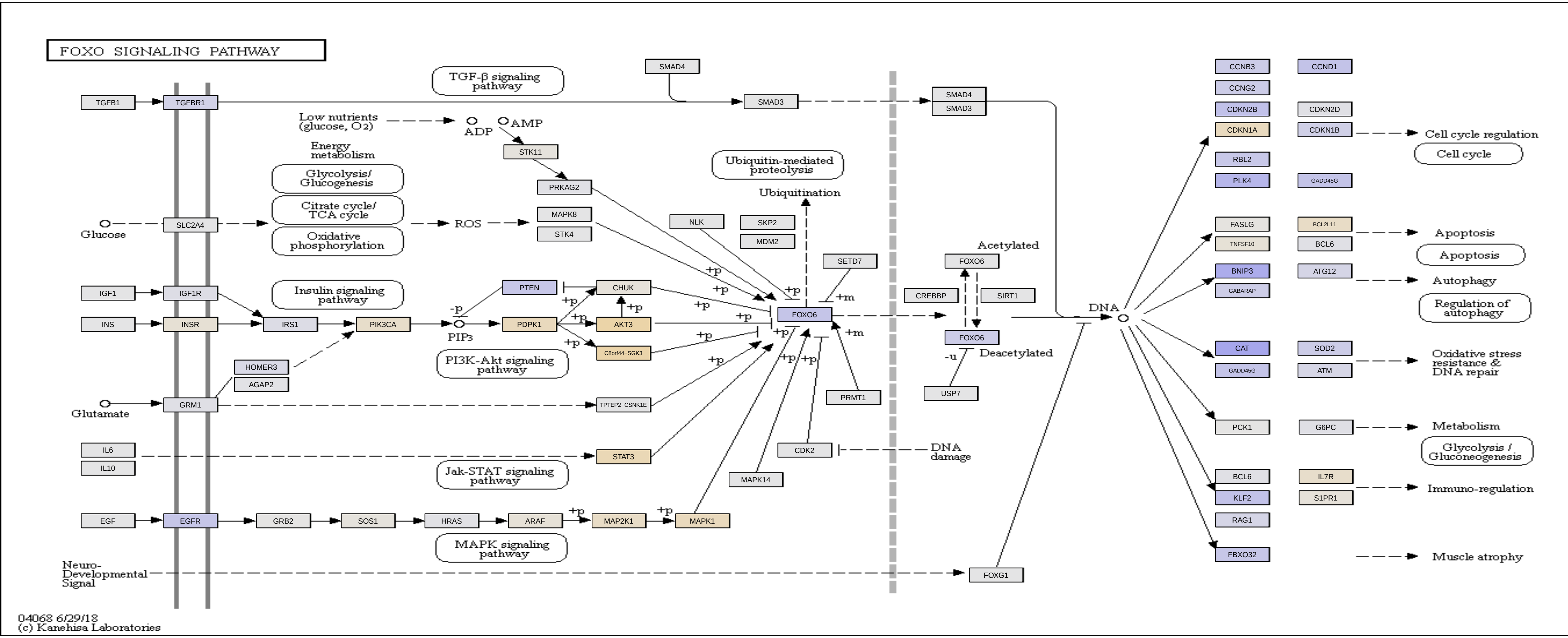
FOXO SIGNALING PATHWAY
TGF-β signaling pathway
SMAD4
TGFB1
TGFBR1
SMAD3
SMAD4
SMAD3
Low nutrients (glucose, O2)
ADP
AMP
STK11
Energy metabolism
Glycolysis/ Glucogenesis
Citrate cycle/ TCA cycle
Oxidative phosphorylation
PRKAG2
MAPK8
STK4
ROS
Glucose
SLC2A4
Ubiquitin-mediated proteolysis
Ubiquitination
NLK
SKP2
MDM2
SETD7
IGF1
IGF1R
Insulin signaling pathway
INS
INSR
IRS1
PIK3CA
PTEN
CHUK
PIP3
PDPK1
AKT3
C8orf44-SGK3
PI3K-Akt signaling pathway
FOXO6
+p
-p
+m
HOMER3
AGAP2
Glutamate
GRM1
TPTEP2-CSNK1E
IL6
IL10
STAT3
Jak-STAT signaling pathway
CDK2
DNA damage
MAPK14
PRMT1
EGF
EGFR
GRB2
SOS1
HRAS
ARAF
MAP2K1
MAPK1
MAPK signaling pathway
Neuro-Developmental Signal
FOXG1
CREBBP
SIRT1
Acetylated
Deacetylated
-u
USP7
DNA
CCNB3
CCND1
CCNG2
CDKN2B
CDKN2D
CDKN1A
CDKN1B
RBL2
PLK4
GADD45G
Cell cycle regulation
Cell cycle
FASLG
BCL2L11
TNFSF10
BCL6
Apoptosis
Apoptosis
BNIP3
ATG12
GABARAP
Autophagy
Regulation of autophagy
CAT
SOD2
GADD45G
ATM
Oxidative stress resistance & DNA repair
PCK1
G6PC
Metabolism
Glycolysis / Gluconeogenesis
BCL6
IL7R
KLF2
S1PR1
Immuno-regulation
RAG1
FBXO32
Muscle atrophy
04068 6/29/18
(c) Kanehisa Laboratories

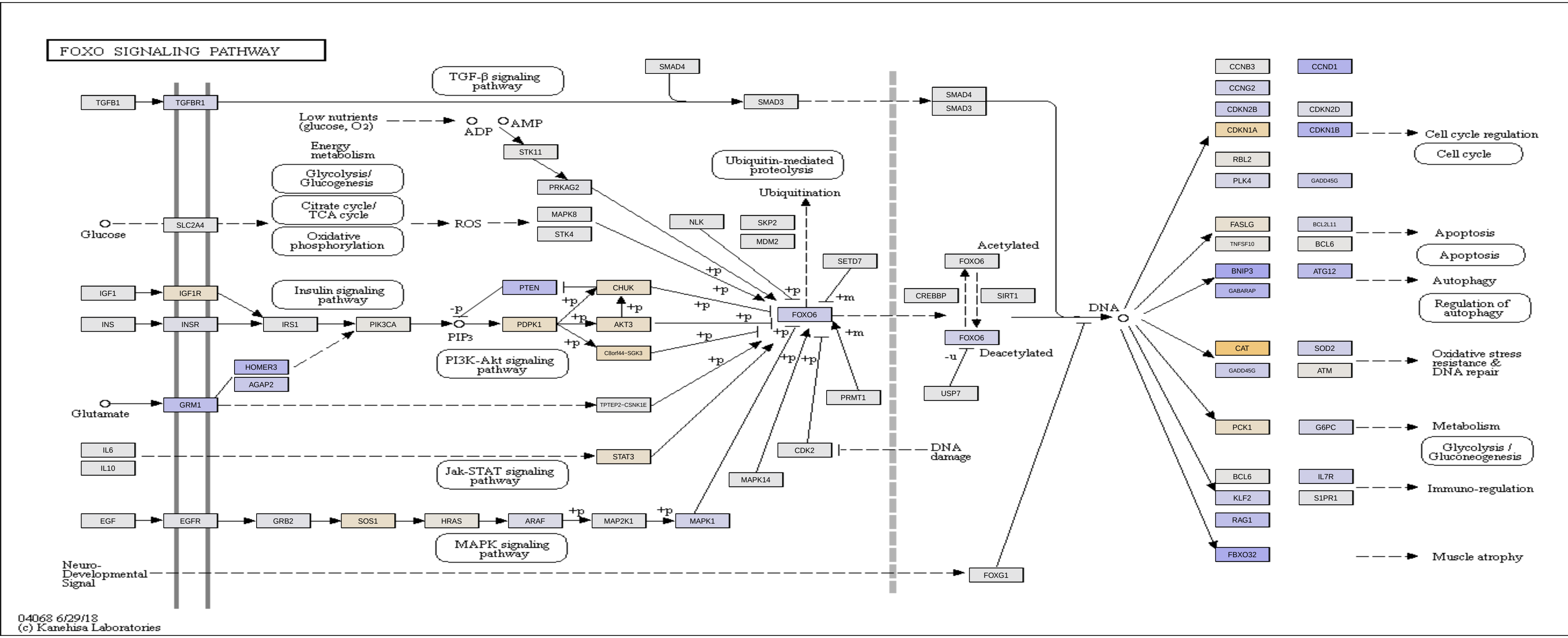
FOXO SIGNALING PATHWAY
TGFB1
TGFBR1
TGF-β signaling pathway
SMAD4
SMAD3
SMAD4
SMAD3
Low nutrients (glucose, O2)
ADP
AMP
STK11
Energy metabolism
Glycolysis/ Glucogenesis
Citrate cycle/ TCA cycle
Oxidative phosphorylation
Glucose
SLC2A4
ROS
PRKAG2
MAPK8
STK4
NLK
SKP2
MDM2
Ubiquitin-mediated proteolysis
Ubiquitination
SETD7
IGF1
IGF1R
Insulin signaling pathway
INS
INSR
IRS1
PIK3CA
PIP3
-p
PTEN
CHUK
PDPK1
AKT3
C8orf44-SGK3
+p
+m
FOXO6
PI3K-Akt signaling pathway
HOMER3
AGAP2
Glutamate
GRM1
TPTEP2-CSNK1E
PRMT1
IL6
IL10
STAT3
Jak-STAT signaling pathway
CDK2
DNA damage
MAPK14
EGF
EGFR
GRB2
SOS1
HRAS
ARAF
MAP2K1
MAPK1
MAPK signaling pathway
Neuro-Developmental Signal
FOXG1
CREBBP
SIRT1
FOXO6
Acetylated
FOXO6
Deacetylated
-u
USP7
DNA
CCNB3
CCND1
CCNG2
CDKN2B
CDKN2D
CDKN1A
CDKN1B
Cell cycle regulation
Cell cycle
RBL2
PLK4
GADD45G
FASLG
BCL2L11
TNFSF10
BCL6
Apoptosis
Apoptosis
BNIP3
ATG12
GABARAP
Autophagy
Regulation of autophagy
CAT
SOD2
GADD45G
ATM
Oxidative stress resistance & DNA repair
PCK1
G6PC
Metabolism
Glycolysis / Gluconeogenesis
BCL6
IL7R
KLF2
S1PR1
Immuno-regulation
RAG1
FBXO32
Muscle atrophy
04068 6/29/18
(c) Kanehisa Laboratories

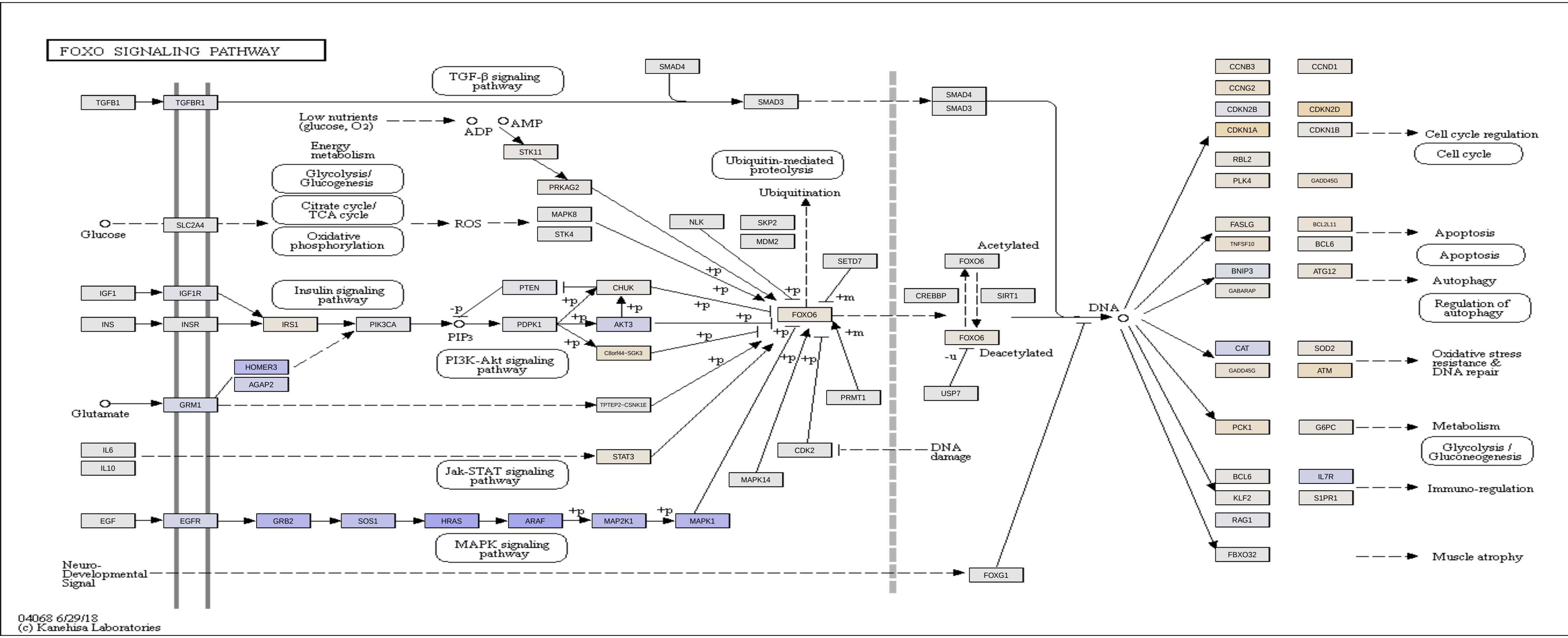
FOXO SIGNALING PATHWAY
TGFB1
TGFBR1
TGF-β signaling pathway
SMAD4
SMAD3
SMAD4
SMAD3
Low nutrients (glucose, O2)
ADP
AMP
STK11
PRKAG2
Energy metabolism
Glycolysis/ Glucogenesis
Citrate cycle/ TCA cycle
Oxidative phosphorylation
ROS
MAPK8
STK4
Glucose
SLC2A4
IGF1
IGF1R
Insulin signaling pathway
INS
INSR
IRS1
PIK3CA
PIP3
PTEN
CHUK
PDPK1
AKT3
C8orf44-SGK3
PI3K-Akt signaling pathway
HOMER3
AGAP2
Glutamate
GRM1
TPTEP2-CSNK1E
IL6
IL10
STAT3
Jak-STAT signaling pathway
EGF
EGFR
GRB2
SOS1
HRAS
ARAF
MAP2K1
MAPK1
MAPK signaling pathway
Neuro-Developmental Signal
Ubiquitin-mediated proteolysis
Ubiquitination
NLK
SKP2
MDM2
SETD7
FOXO6
CDK2
MAPK14
PRMT1
DNA damage
CREBBP
SIRT1
FOXO6
Acetylated
FOXO6
Deacetylated
USP7
FOXG1
DNA
CCNB3
CCND1
CCNG2
CDKN2B
CDKN2D
CDKN1A
CDKN1B
Cell cycle regulation
Cell cycle
RBL2
PLK4
GADD45G
FASLG
BCL2L11
Apoptosis
TNFSF10
BCL6
Apoptosis
BNIP3
ATG12
Autophagy
GABARAP
Regulation of autophagy
CAT
SOD2
Oxidative stress resistance & DNA repair
GADD45G
ATM
PCK1
G6PC
Metabolism
Glycolysis / Gluconeogenesis
BCL6
IL7R
Immuno-regulation
KLF2
S1PR1
RAG1
FBXO32
Muscle atrophy
04068 6/29/18
(c) Kanehisa Laboratories

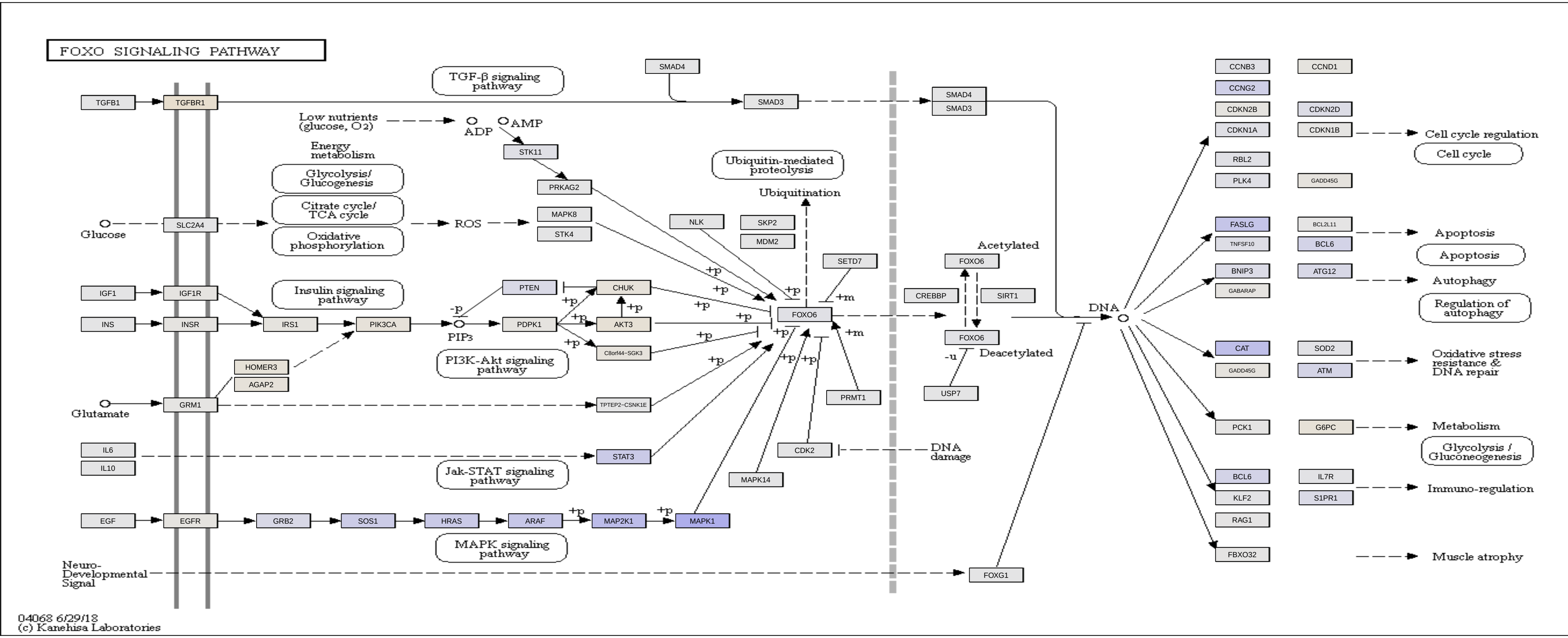
FOXO SIGNALING PATHWAY
TGF-β signaling pathway
TGFB1
TGFBR1
SMAD4
SMAD3
Low nutrients (glucose, O2)
ADP
AMP
STK11
PRKAG2
Energy metabolism
Glycolysis/ Glucogenesis
Citrate cycle/ TCA cycle
Oxidative phosphorylation
Glucose
SLC2A4
ROS
MAPK8
STK4
Ubiquitin-mediated proteolysis
Ubiquitination
NLK
SKP2
MDM2
SETD7
IGF1
IGF1R
INS
INSR
IRS1
PIK3CA
Insulin signaling pathway
PTEN
CHUK
PDPK1
AKT3
C8orf44-SGK3
PIP3
PI3K-Akt signaling pathway
FOXO6
HOMER3
AGAP2
Glutamate
GRM1
TPTEP2-CSNK1E
IL6
IL10
STAT3
Jak-STAT signaling pathway
CDK2
DNA damage
PRMT1
MAPK14
EGF
EGFR
GRB2
SOS1
HRAS
ARAF
MAP2K1
MAPK1
MAPK signaling pathway
Neuro-Developmental Signal
FOXG1
Acetylated
CREBBP
SIRT1
Deacetylated
USP7
DNA
CCNB3
CCND1
CCNG2
CDKN2B
CDKN2D
CDKN1A
CDKN1B
Cell cycle regulation
Cell cycle
RBL2
PLK4
GADD45G
FASLG
BCL2L11
TNFSF10
BCL6
Apoptosis
BNIP3
ATG12
GABARAP
Autophagy
Regulation of autophagy
CAT
SOD2
ATM
Oxidative stress resistance & DNA repair
PCK1
G6PC
Metabolism
Glycolysis / Gluconeogenesis
IL7R
KLF2
S1PR1
Immuno-regulation
RAG1
FBXO32
Muscle atrophy
04068 6/29/18
(c) Kanehisa Laboratories

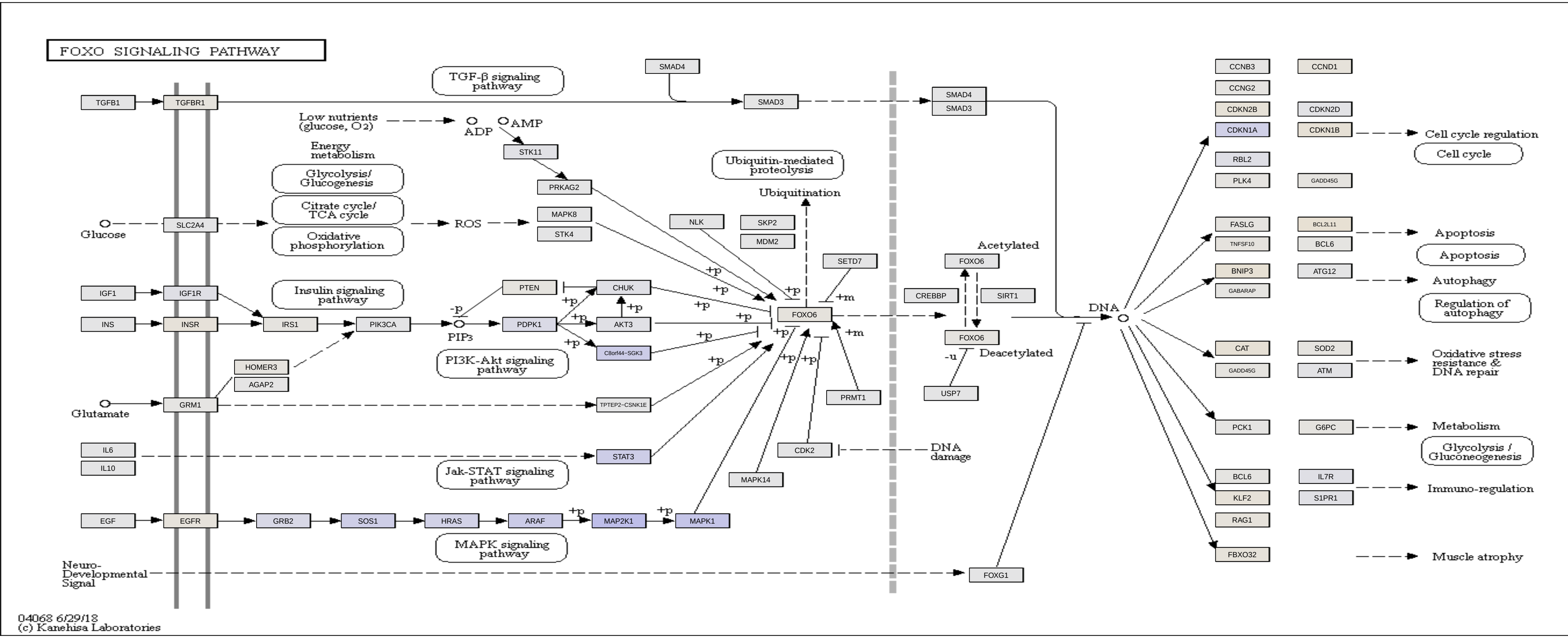
FOXO SIGNALING PATHWAY
TGF-β signaling pathway
SMAD4
TGFB1
TGFBR1
SMAD3
SMAD4
SMAD3
Low nutrients (glucose, O2)
ADP
AMP
STK11
Energy metabolism
Glycolysis/ Glucogenesis
Citrate cycle/ TCA cycle
Oxidative phosphorylation
PRKAG2
Glucose
SLC2A4
ROS
MAPK8
STK4
NLK
SKP2
MDM2
Ubiquitin-mediated proteolysis
Ubiquitination
SETD7
IGF1
IGF1R
Insulin signaling pathway
INS
INSR
IRS1
PIK3CA
PIP3
PTEN
CHUK
PDPK1
AKT3
C8orf44-SGK3
PI3K-Akt signaling pathway
FOXO6
HOMER3
AGAP2
Glutamate
GRM1
TPTEP2-CSNK1E
IL6
IL10
STAT3
Jak-STAT signaling pathway
CDK2
DNA damage
MAPK14
PRMT1
EGF
EGFR
GRB2
SOS1
HRAS
ARAF
MAP2K1
MAPK1
MAPK signaling pathway
Neuro-Developmental Signal
Acetylated
FOXO6
CREBBP
SIRT1
FOXO6
Deacetylated
USP7
FOXG1
DNA
CCNB3
CCND1
CCNG2
CDKN2B
CDKN2D
CDKN1A
CDKN1B
Cell cycle regulation
Cell cycle
RBL2
PLK4
GADD45G
FASLG
BCL2L11
TNFSF10
BCL6
Apoptosis
Apoptosis
BNIP3
ATG12
GABARAP
Autophagy
Regulation of autophagy
CAT
SOD2
GADD45G
ATM
Oxidative stress resistance & DNA repair
PCK1
G6PC
Metabolism
Glycolysis / Gluconeogenesis
BCL6
IL7R
KLF2
S1PR1
Immuno-regulation
RAG1
FBXO32
Muscle atrophy
04068 6/29/18
(c) Kanehisa Laboratories

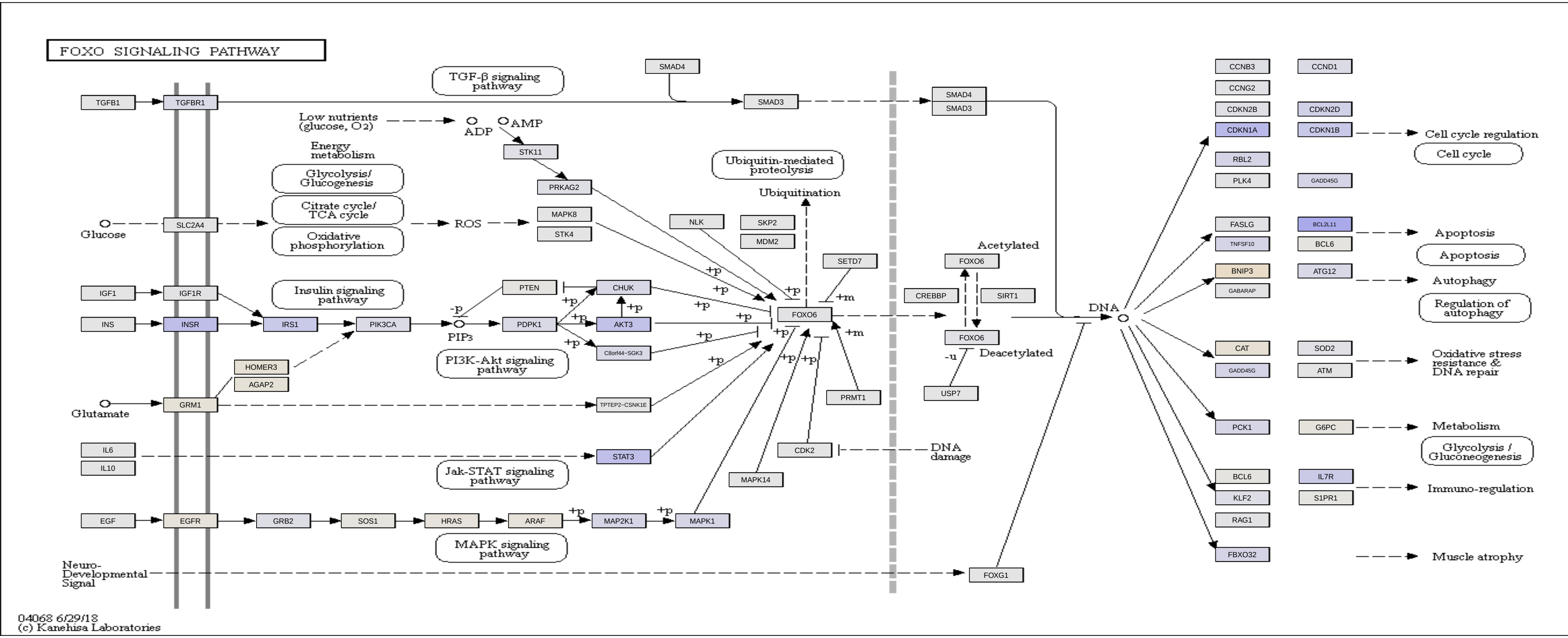
FOXO SIGNALING PATHWAY
TGF-β signaling pathway
SMAD4
TGFB1
TGFBR1
SMAD3
SMAD4
SMAD3
Low nutrients (glucose, O2)
ADP
AMP
STK11
Energy metabolism
Glycolysis/ Glucogenesis
Citrate cycle/ TCA cycle
Oxidative phosphorylation
PRKAG2
Glucose
SLC2A4
ROS
MAPK8
STK4
NLK
SKP2
MDM2
Ubiquitin-mediated proteolysis
Ubiquitination
SETD7
IGF1
IGF1R
INS
INSR
IRS1
PIK3CA
Insulin signaling pathway
PIP3
PTEN
CHUK
PDPK1
AKT3
C8orf44-SGK3
PI3K-Akt signaling pathway
FOXO6
+p
+m
-p
-u
HOMER3
AGAP2
Glutamate
GRM1
TPTEP2-CSNK1E
IL6
IL10
STAT3
Jak-STAT signaling pathway
CDK2
DNA damage
MAPK14
PRMT1
EGF
EGFR
GRB2
SOS1
HRAS
ARAF
MAP2K1
MAPK1
MAPK signaling pathway
Neuro-Developmental Signal
FOXG1
CREBBP
SIRT1
Acetylated
Deacetylated
USP7
DNA
CCNB3
CCND1
CCNG2
CDKN2B
CDKN2D
CDKN1A
CDKN1B
RBL2
PLK4
GADD45G
Cell cycle regulation
Cell cycle
FASLG
BCL2L11
TNFSF10
BCL6
Apoptosis
Apoptosis
BNIP3
ATG12
GABARAP
Autophagy
Regulation of autophagy
CAT
SOD2
GADD45G
ATM
Oxidative stress resistance & DNA repair
PCK1
G6PC
Metabolism
Glycolysis / Gluconeogenesis
BCL6
IL7R
KLF2
S1PR1
Immuno-regulation
RAG1
FBXO32
Muscle atrophy
04068 6/29/18
(c) Kanehisa Laboratories
