# Supplementary material for: The Transcriptome and Methylome of the Developing and Aging Brain and Their Relations to Gliomas and Psychological Disorders
Source: Cells. 2022 Jan 21;11(3):362. doi: 10.3390/cells11030362 (PMC8834030; doi:10.3390/cells11030362)
Supplement: Supplementary file 1 [file cells-11-00362-s001.zip › Suppl-Material-S4-Pathways-PSF_Methylation/GnRH_signaling_pathway.pdf]

GnRH signaling pathway

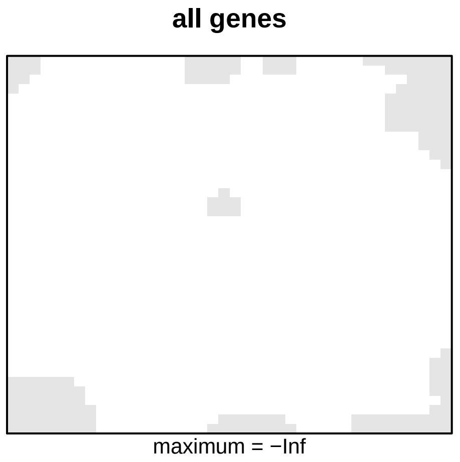

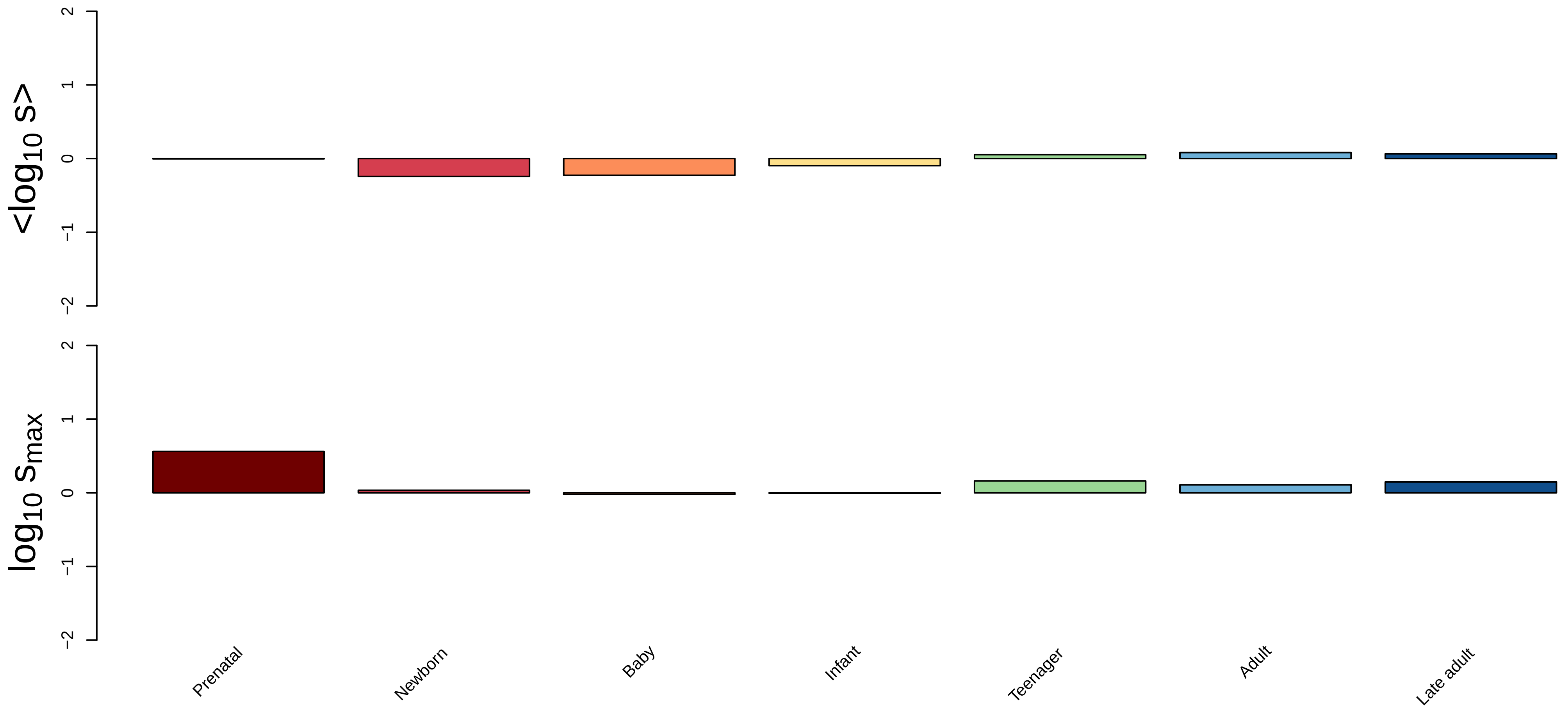

GnRH_signaling_pathway
genes with data

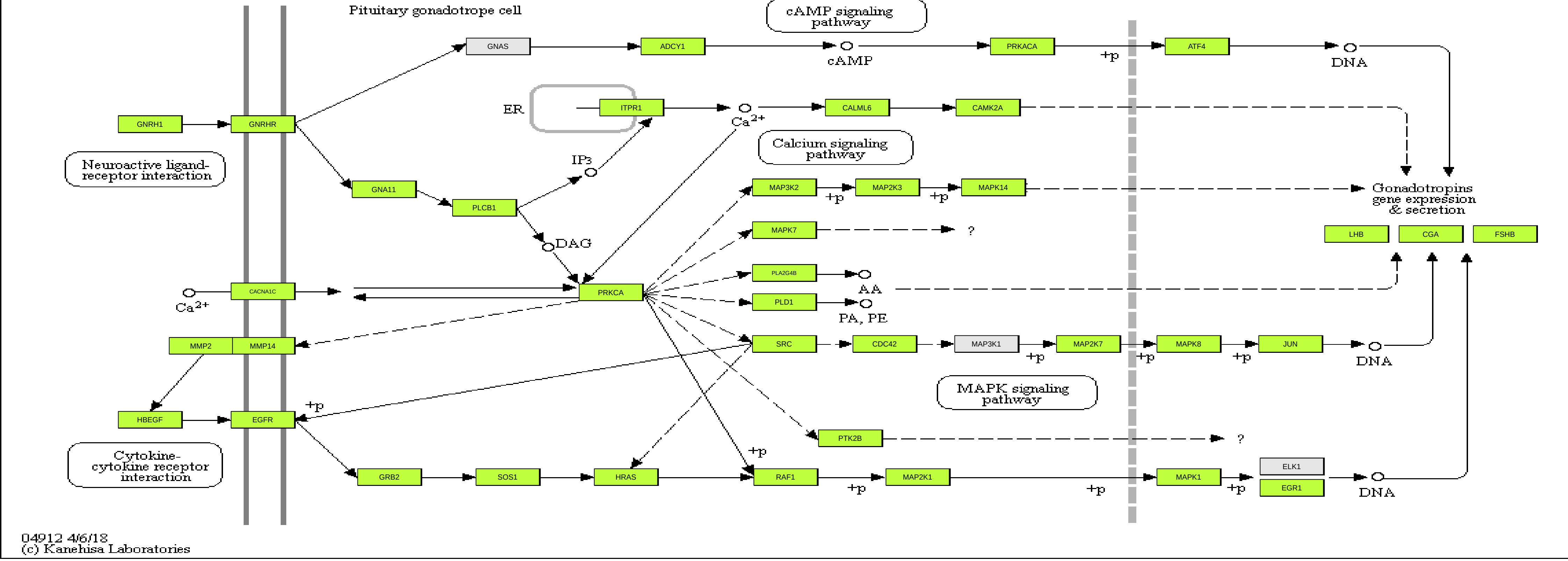

GnRH_signaling_pathway
sink nodes

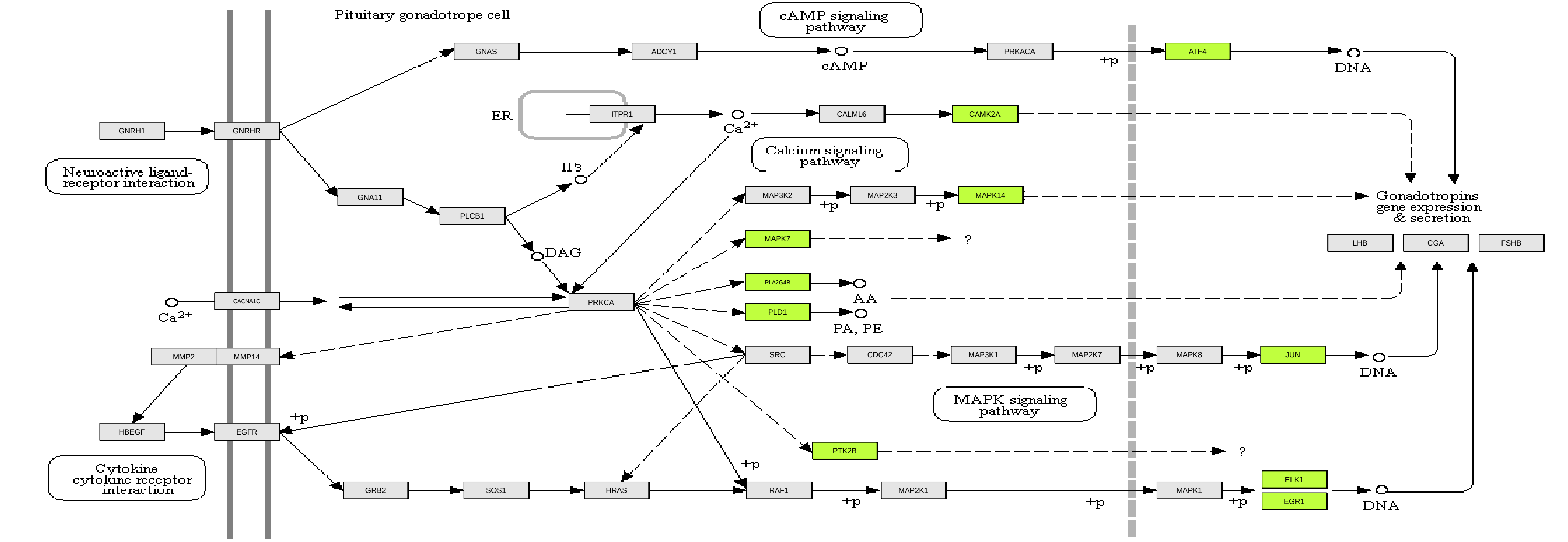

04912 4/6/18
(c) Kanehisa Laboratories

GnRH_signaling_pathway
Prenatal

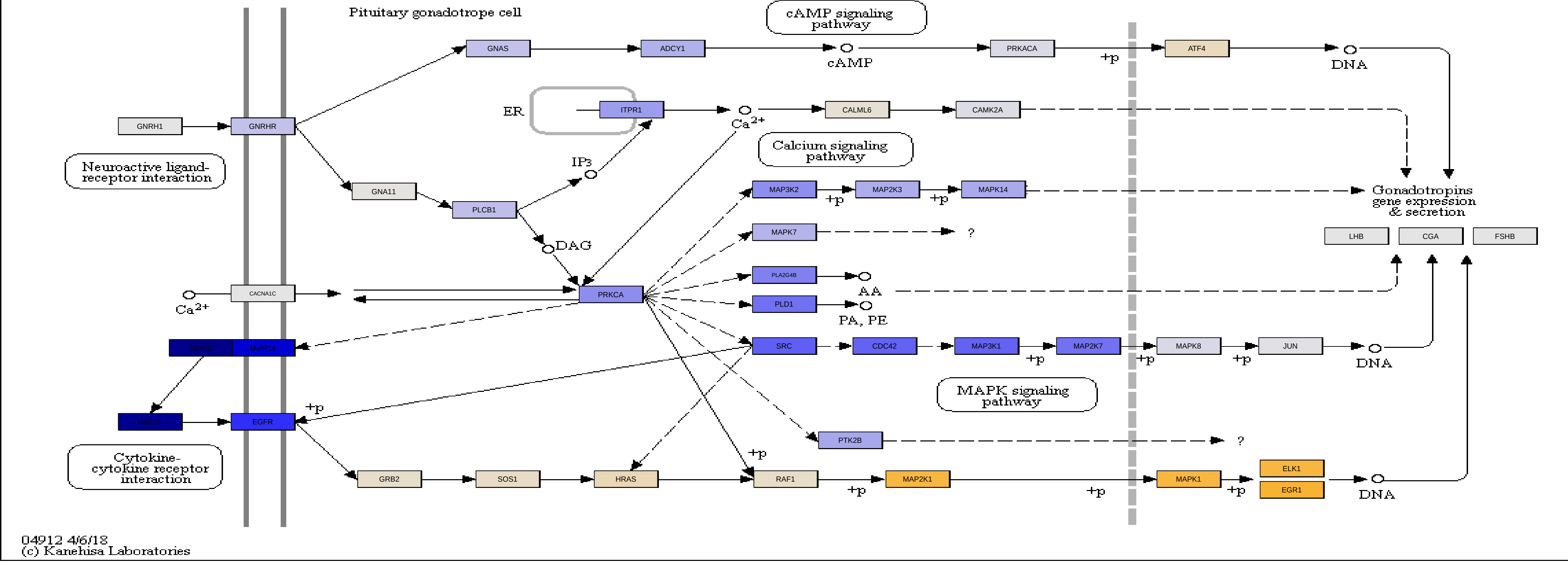

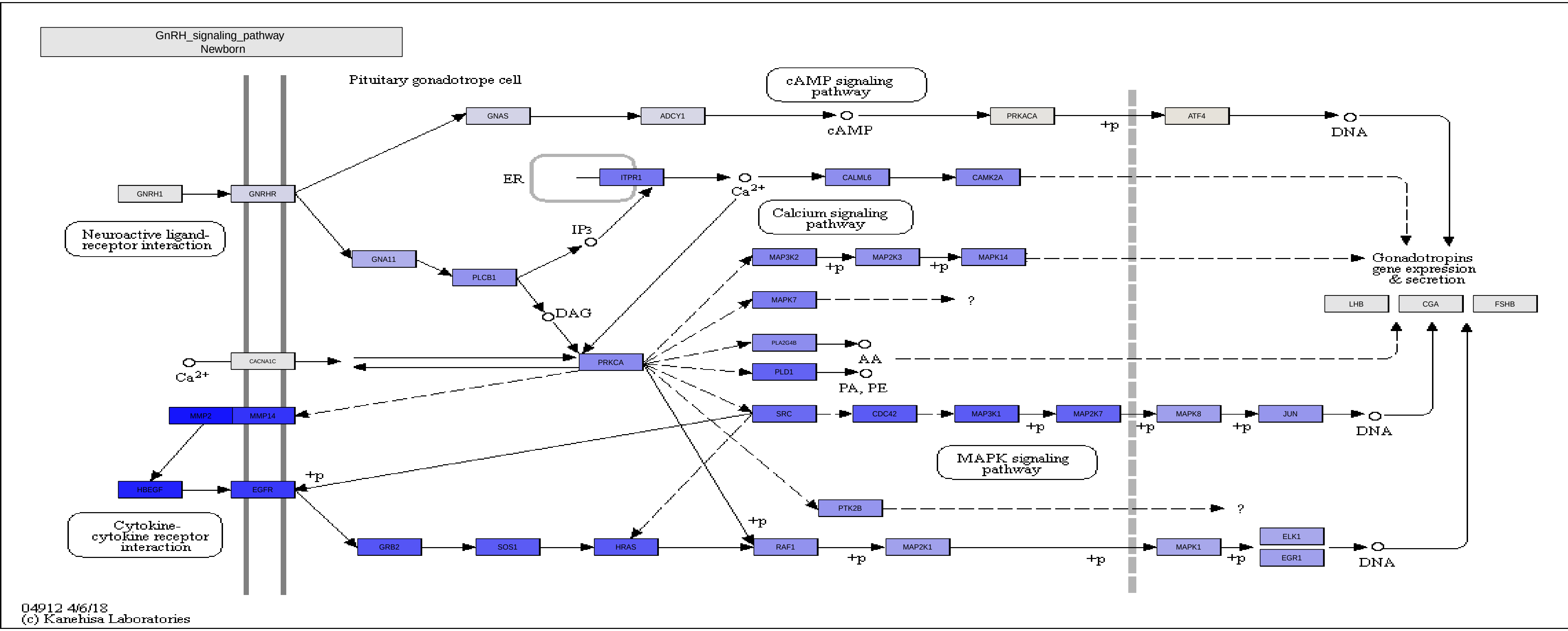

GnRH_signaling_pathway
Newborn
Pituitary gonadotrope cell
cAMP signaling pathway
GNAS
ADCY1
cAMP
PRKACA
+p
ATF4
DNA
ER
ITPR1
Ca2+
CALML6
CAMK2A
GNRH1
GNRHR
Neuroactive ligand-receptor interaction
Calcium signaling pathway
IP3
GNA11
PLCB1
DAG
MAP3K2
+p
MAP2K3
+p
MAPK14
Gonadotropins gene expression & secretion
MAPK7
?
LHB
CGA
FSHB
PLA2G4B
AA
CACNA1C
Ca2+
PRKCA
PLD1
PA, PE
MMP2
MMP14
SRC
CDC42
MAP3K1
+p
MAP2K7
+p
MAPK8
+p
JUN
DNA
MAPK signaling pathway
HBEGF
EGFR
+p
PTK2B
?
+p
ELK1
Cytokine-cytokine receptor interaction
GRB2
SOS1
HRAS
RAF1
+p
MAP2K1
+p
MAPK1
+p
EGR1
DNA
04912 4/6/18
(c) Kanehisa Laboratories

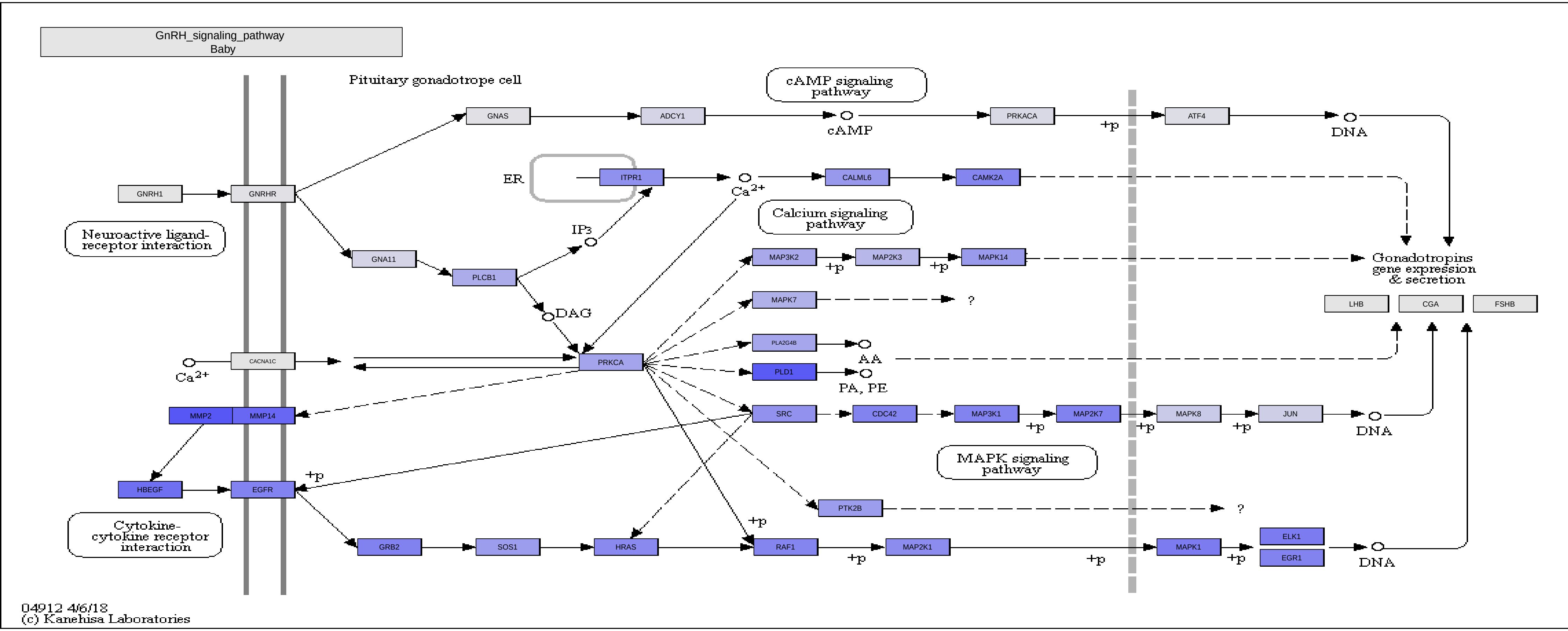

GnRH_signaling_pathway
Baby
Pituitary gonadotrope cell
cAMP signaling pathway
GNAS
ADCY1
cAMP
PRKACA
+p
ATF4
DNA
ER
ITPR1
Ca2+
CALML6
CAMK2A
GNRH1
GNRHR
Calcium signaling pathway
Neuroactive ligand-receptor interaction
IP3
GNA11
PLCB1
MAP3K2
MAP2K3
MAPK14
Gonadotropins gene expression & secretion
DAG
MAPK7
?
LHB
CGA
FSHB
PLA2G4B
AA
Ca2+
CACNA1C
PRKCA
PLD1
PA, PE
MMP2
MMP14
SRC
CDC42
MAP3K1
MAP2K7
MAPK8
JUN
DNA
HBEGF
EGFR
MAPK signaling pathway
PTK2B
Cytokine-cytokine receptor interaction
GRB2
SOS1
HRAS
RAF1
MAP2K1
MAPK1
ELK1
EGR1
04912 4/6/18
(c) Kanehisa Laboratories

GnRH_signaling_pathway
Infant

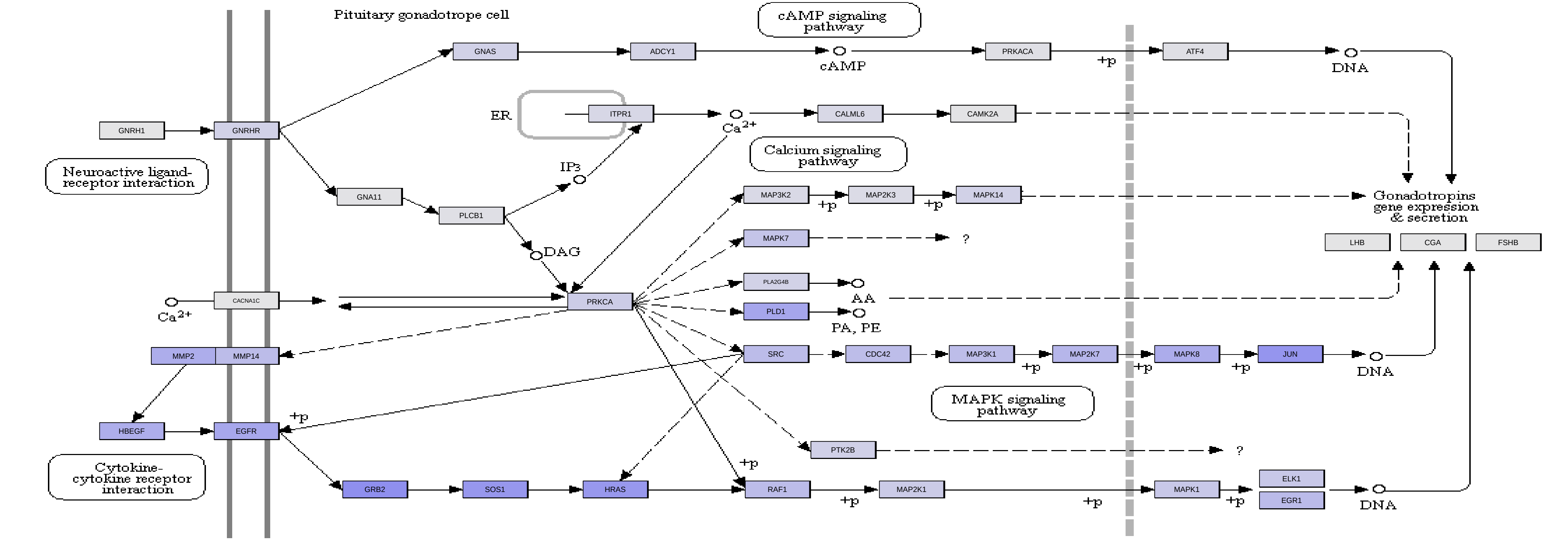

04912 4/6/18
(c) Kanehisa Laboratories

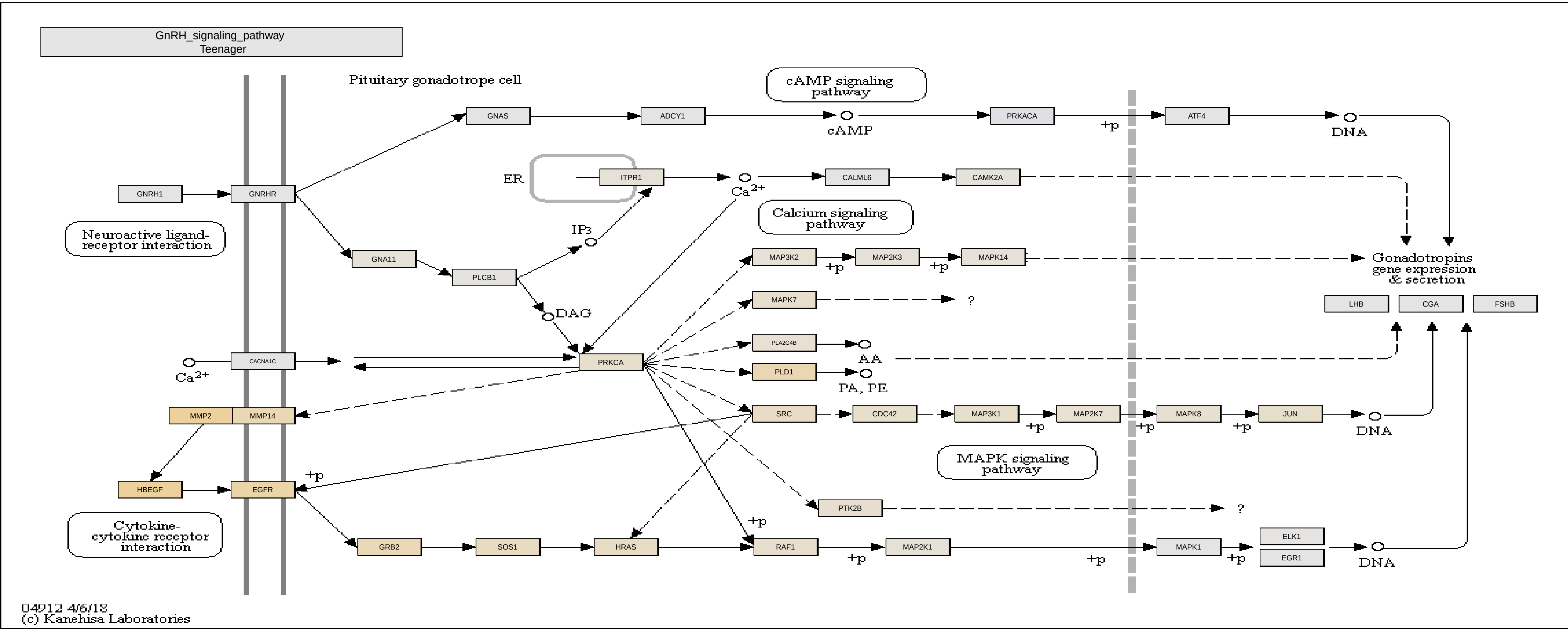
GnRH_signaling_pathway
Teenager
Pituitary gonadotrope cell
cAMP signaling pathway
GNAS
ADCY1
cAMP
PRKACA
+p
ATF4
DNA
ER
ITPR1
Ca2+
CALML6
CAMK2A
GNRH1
GNRHR
Calcium signaling pathway
Neuroactive ligand-receptor interaction
IP3
GNA11
PLCB1
MAP3K2
MAP2K3
MAPK14
Gonadotropins gene expression & secretion
MAPK7
?
LHB
CGA
FSHB
DAG
PLA2G4B
AA
CACNA1C
PRKCA
PLD1
PA, PE
MMP2
MMP14
SRC
CDC42
MAP3K1
MAP2K7
MAPK8
JUN
MAPK signaling pathway
HBEGF
EGFR
PTK2B
Cytokine-cytokine receptor interaction
GRB2
SOS1
HRAS
RAF1
MAP2K1
MAPK1
ELK1
EGR1
04912 4/6/18
(c) Kanehisa Laboratories

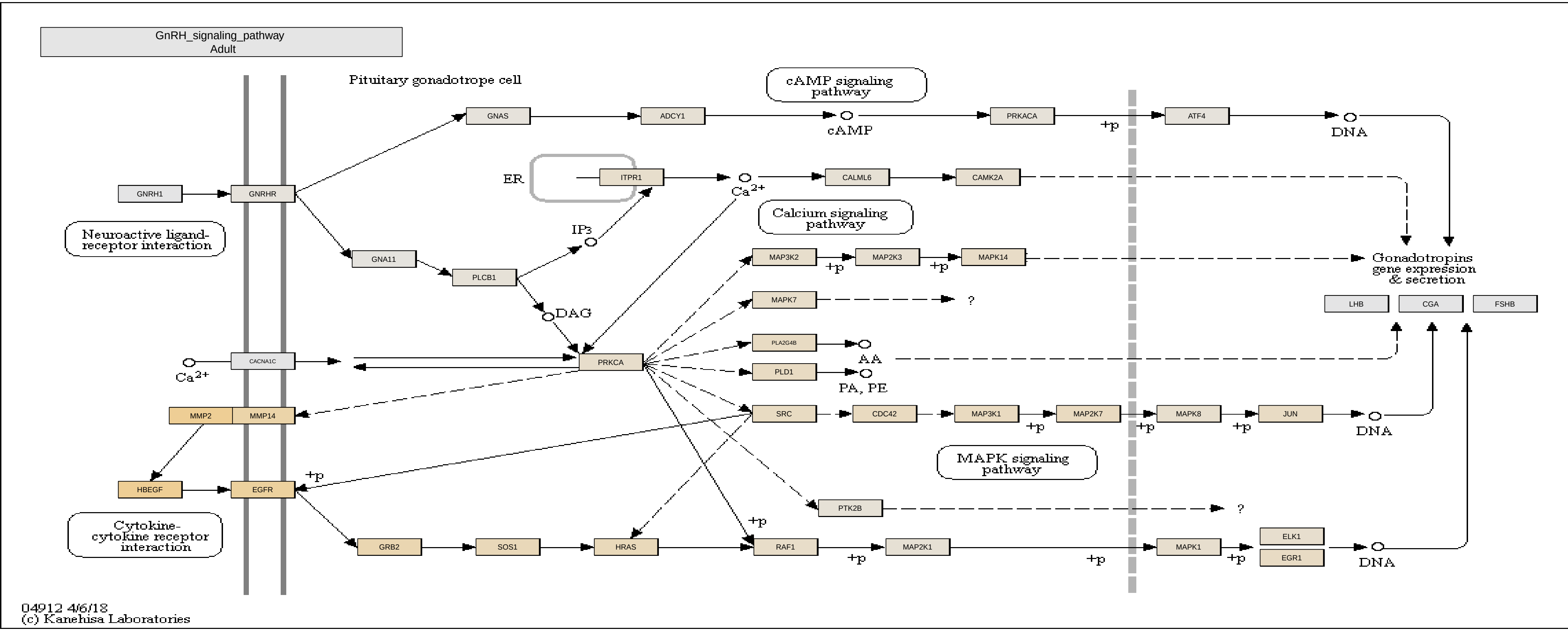
GnRH_signaling_pathway
Adult
Pituitary gonadotrope cell
cAMP signaling pathway
GNAS
ADCY1
cAMP
PRKACA
+p
ATF4
DNA
ER
ITPR1
Ca2+
CALML6
CAMK2A
GNRH1
GNRHR
Neuroactive ligand-receptor interaction
Calcium signaling pathway
IP3
GNA11
PLCB1
DAG
MAP3K2
+p
MAP2K3
+p
MAPK14
Gonadotropins gene expression & secretion
LHB
CGA
FSHB
MAPK7
?
PLA2G4B
AA
CACNA1C
Ca2+
PRKCA
PLD1
PA, PE
MMP2
MMP14
SRC
CDC42
MAP3K1
MAP2K7
MAPK8
JUN
DNA
MAPK signaling pathway
HBEGF
EGFR
+p
PTK2B
?
Cytokine-cytokine receptor interaction
GRB2
SOS1
HRAS
RAF1
MAP2K1
MAPK1
ELK1
EGR1
DNA
04912 4/6/18
(c) Kanehisa Laboratories

GnRH_signaling_pathway
Late adult

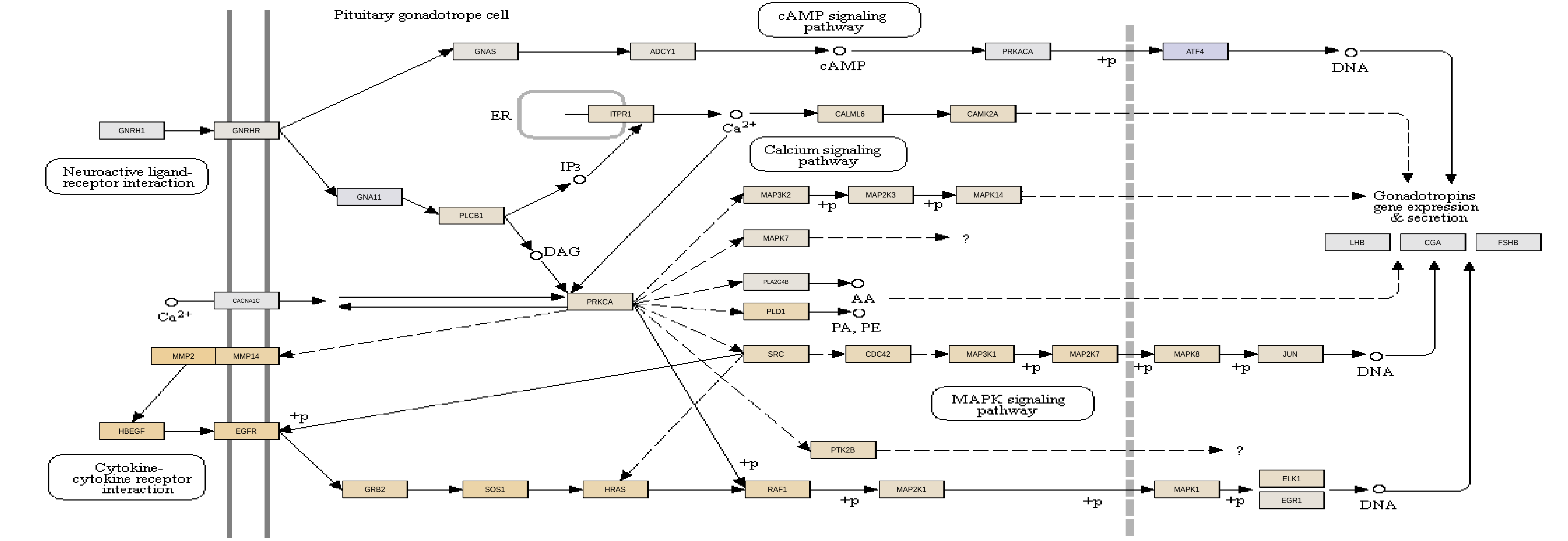

04912 4/6/18
(c) Kanehisa Laboratories
