# Supplementary material for: The Transcriptome and Methylome of the Developing and Aging Brain and Their Relations to Gliomas and Psychological Disorders
Source: Cells. 2022 Jan 21;11(3):362. doi: 10.3390/cells11030362 (PMC8834030; doi:10.3390/cells11030362)
Supplement: Supplementary file 1 [file cells-11-00362-s001.zip › Suppl-Material-S4-Pathways-PSF_Methylation/Hippo_signaling_pathway.pdf]

Hippo signaling pathway

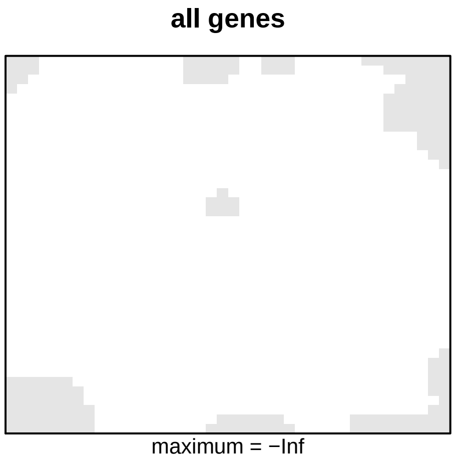

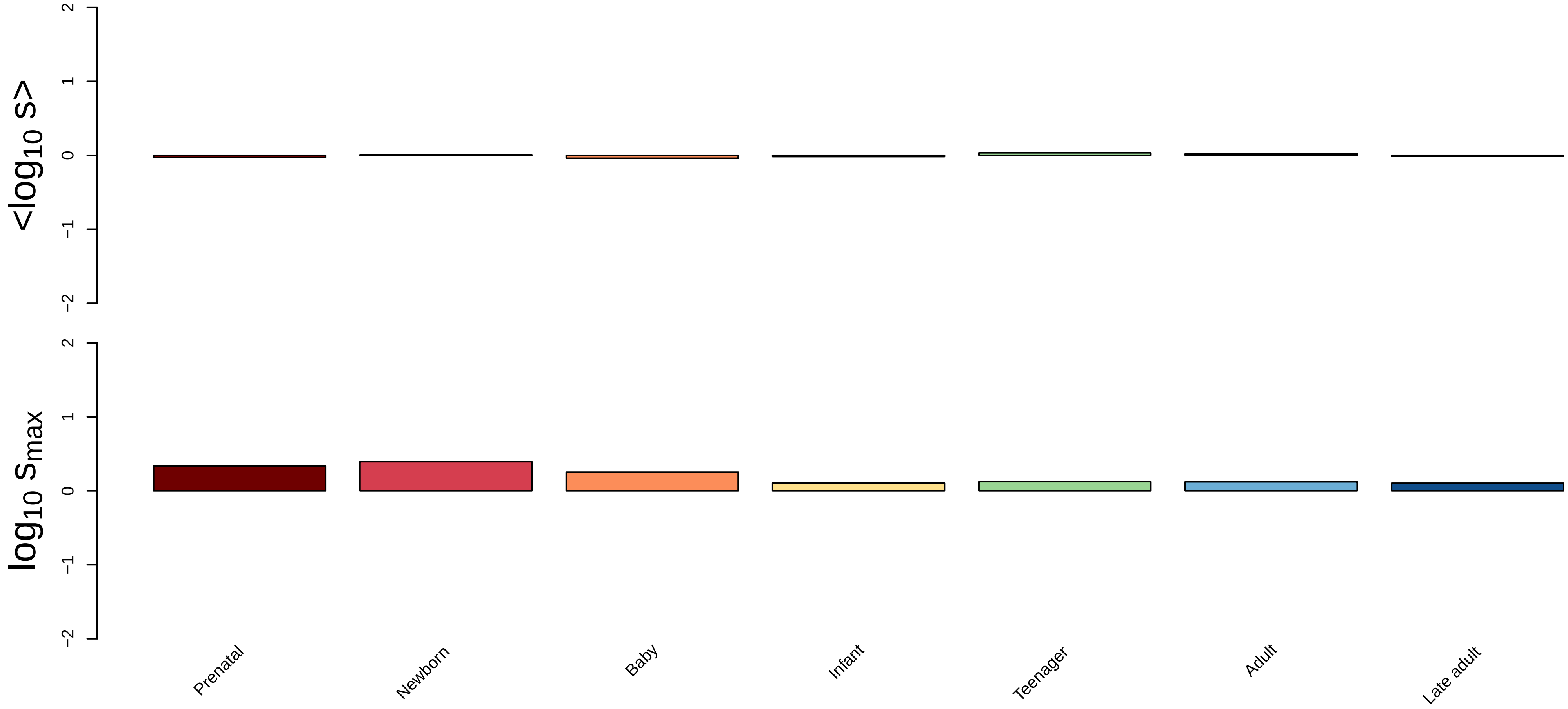

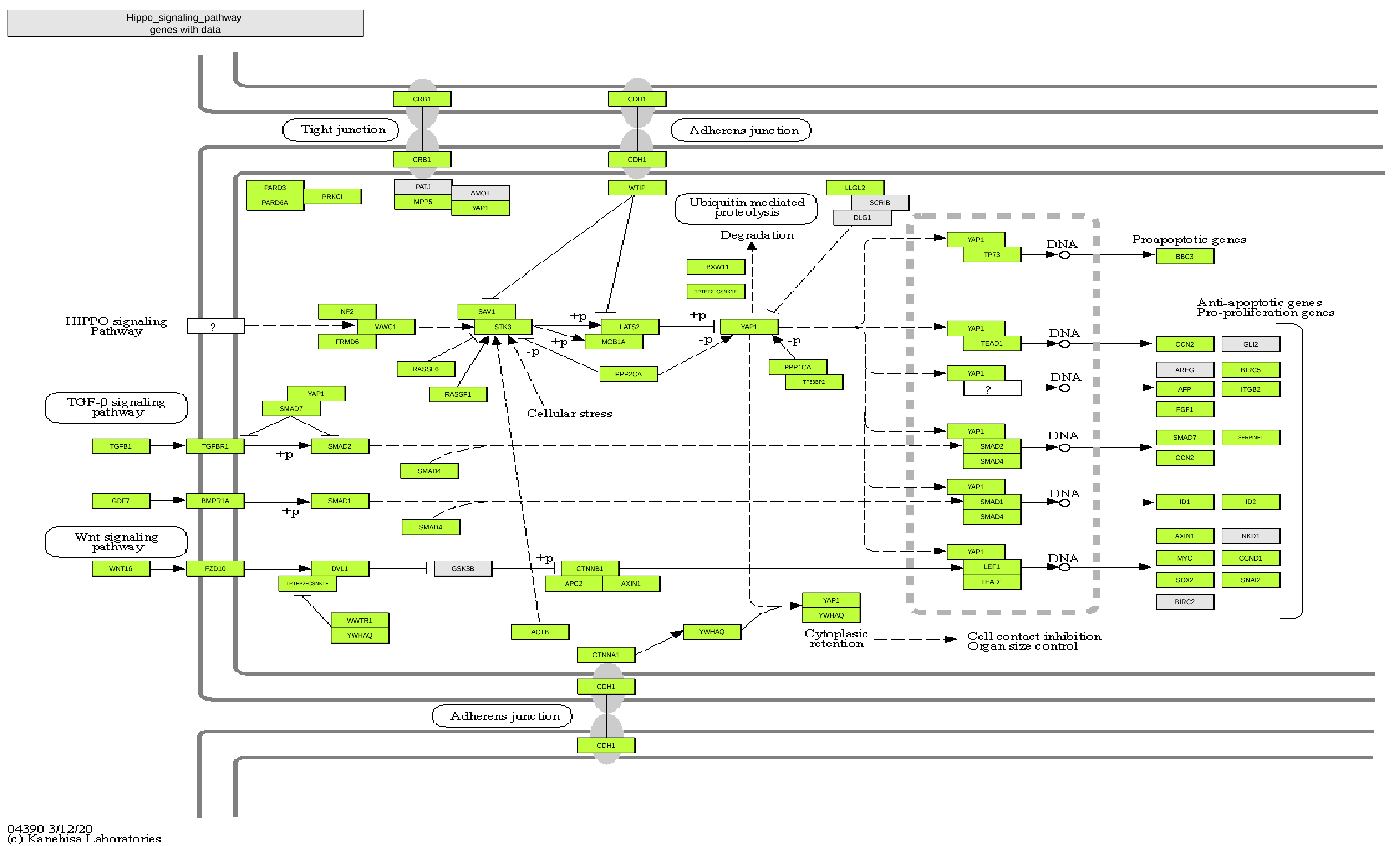
Hippo_signaling_pathway
genes with data
CRB1
CDH1
Tight junction
Adherens junction
CRB1
CDH1
PARD3
PARD6A
PRKCI
PATJ
MPP5
AMOT
YAP1
WTIP
Ubiquitin mediated proteolysis
Degradation
LLGL2
SCRIB
DLG1
FBXW11
TPTEP2-CSNK1E
HIPPO signaling Pathway
?
NF2
WWC1
FRMD6
SAV1
STK3
+p
LATS2
MOB1A
+p
YAP1
-p
-p
PPP1CA
TP53BP2
PPP2CA
RASSF6
RASSF1
-p
Cellular stress
YAP1
TP73
DNA
Proapoptotic genes
BBC3
Anti-apoptotic genes
Pro-proliferation genes
YAP1
TEAD1
DNA
CCN2
GLI2
AREG
BIRC5
YAP1
?
DNA
AFP
ITGB2
FGF1
TGF-β signaling pathway
YAP1
SMAD7
TGFB1
TGFBR1
+p
SMAD2
SMAD4
YAP1
SMAD2
SMAD4
DNA
SMAD7
SERPINE1
CCN2
GDF7
BMPR1A
+p
SMAD1
SMAD4
YAP1
SMAD1
SMAD4
DNA
ID1
ID2
Wnt signaling pathway
WNT16
FZD10
DVL1
TPTEP2-CSNK1E
GSK3B
+p
CTNNB1
APC2
AXIN1
AXIN1
NKD1
YAP1
LEF1
TEAD1
DNA
MYC
CCND1
SOX2
SNAI2
BIRC2
WWTR1
YWHAQ
ACTB
YAP1
YWHAQ
YWHAQ
CTNNA1
Cytoplasic retention
Cell contact inhibition
Organ size control
CDH1
Adherens junction
CDH1
04390 3/12/20
(c) Kanehisa Laboratories

Hippo_signaling_pathway
sink nodes

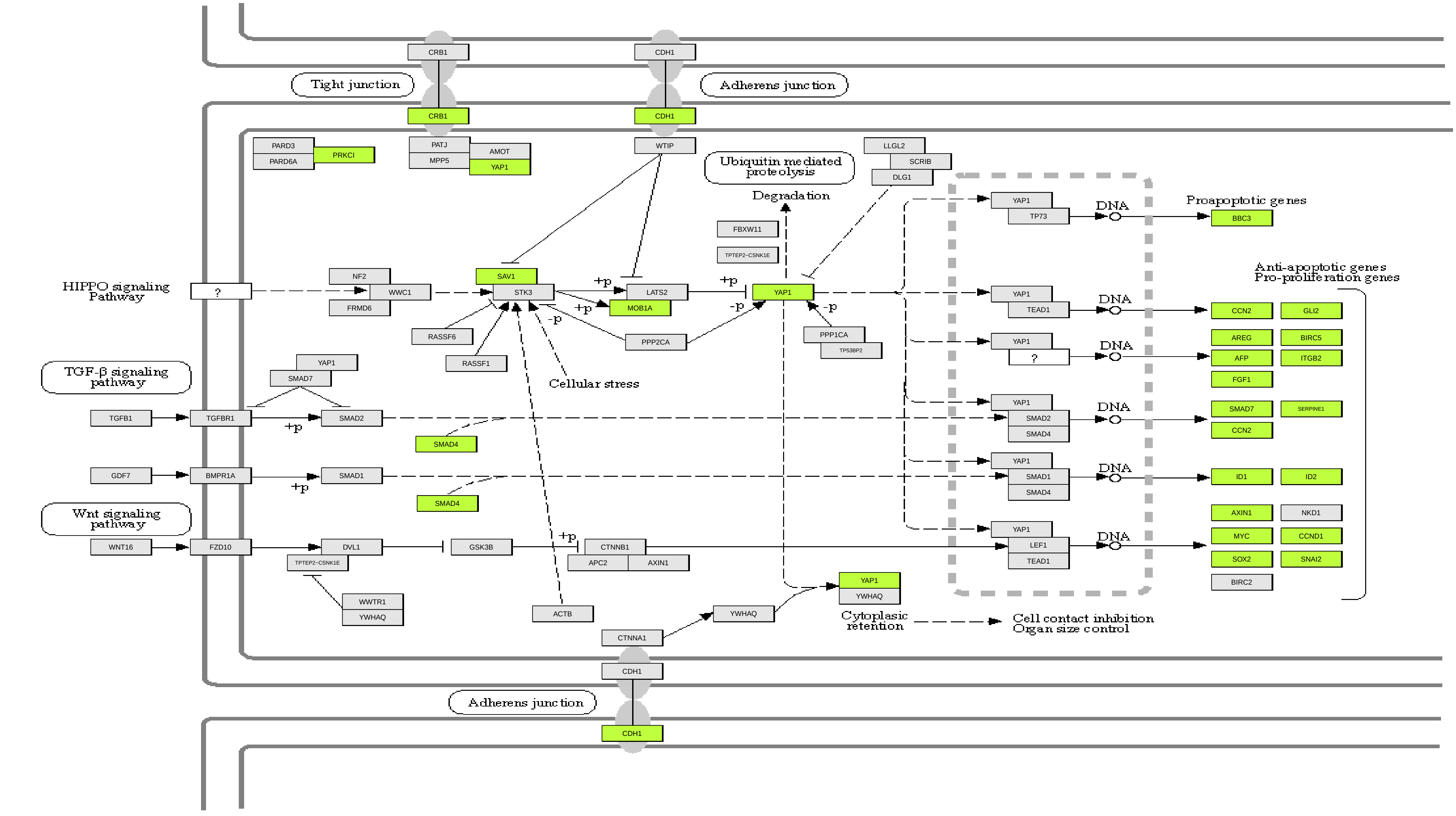

04390 3/12/20
(c) Kanehisa Laboratories

Hippo_signaling_pathway
Prenatal

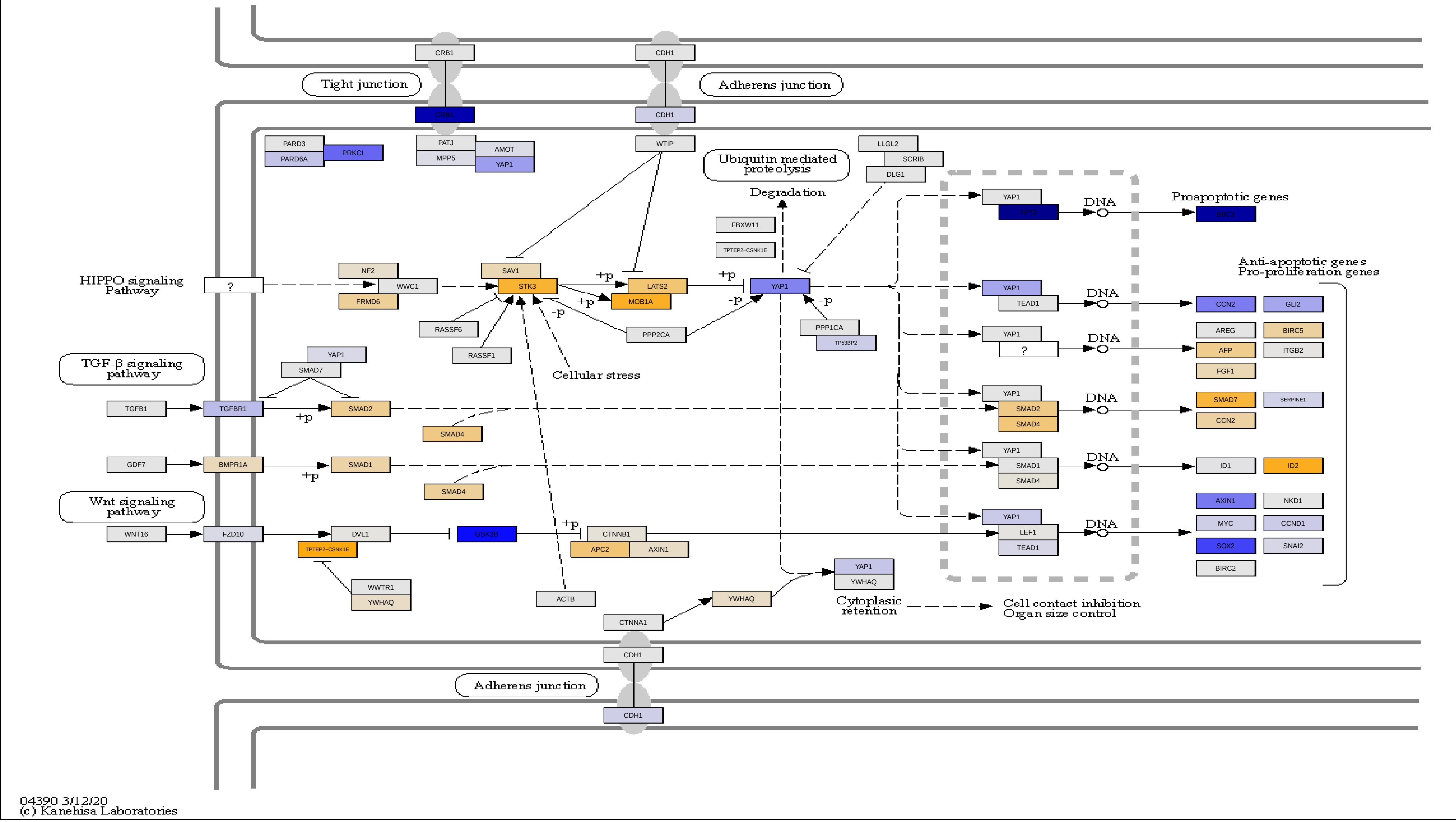

Hippo_signaling_pathway
Newborn

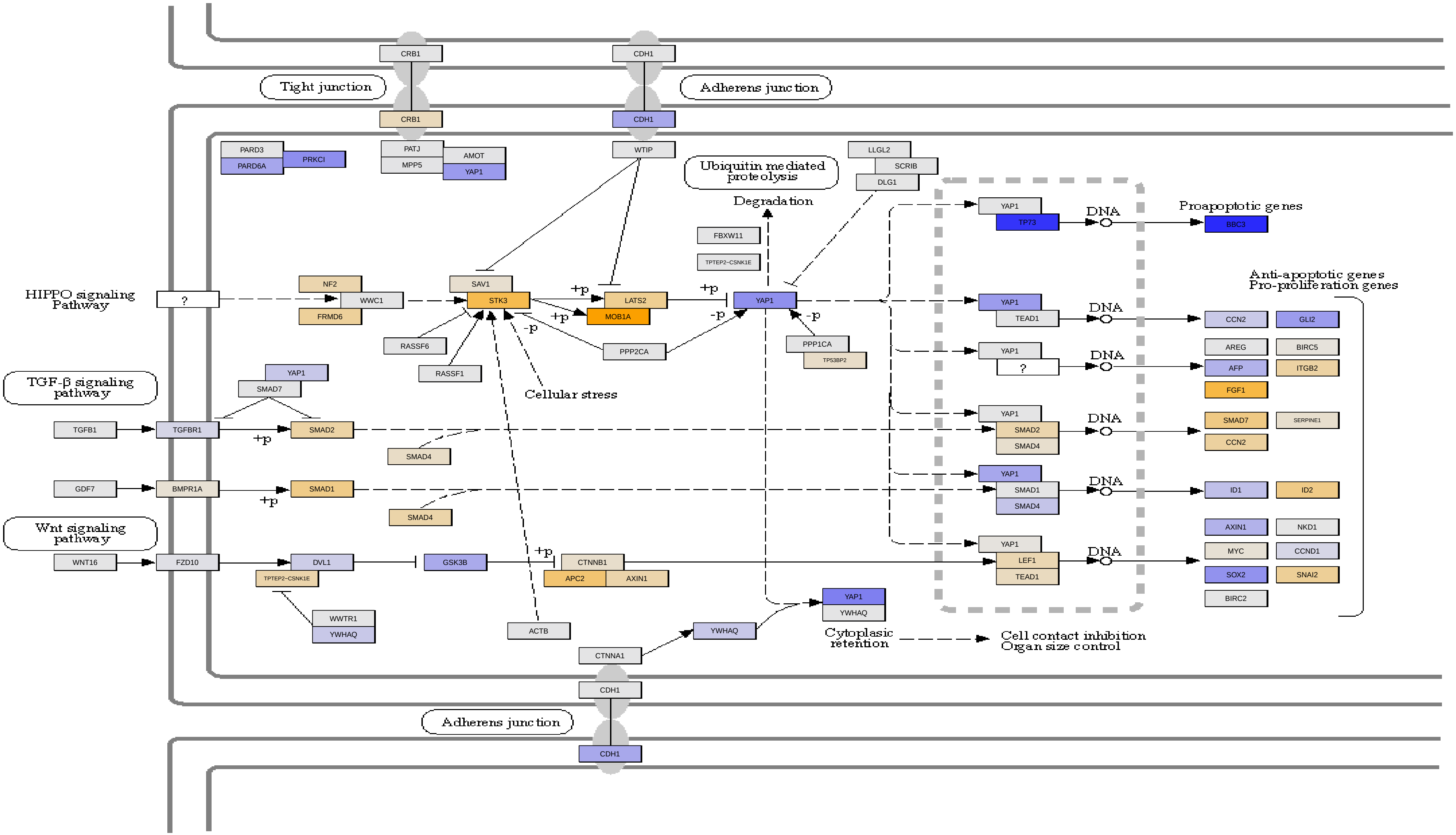

04390 3/12/20
(c) Kanehisa Laboratories

Hippo_signaling_pathway
Baby

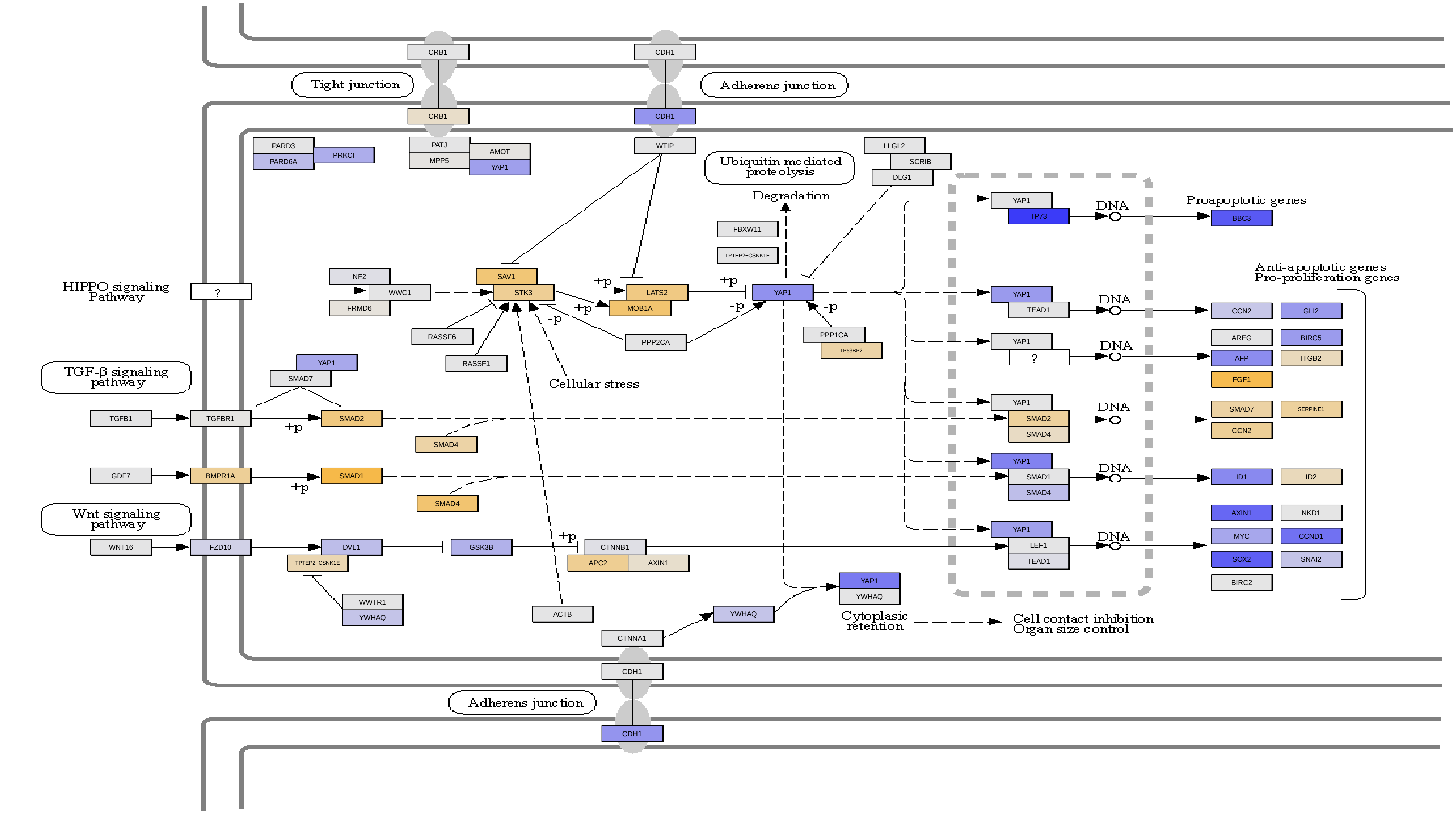

04390 3/12/20
(c) Kanehisa Laboratories

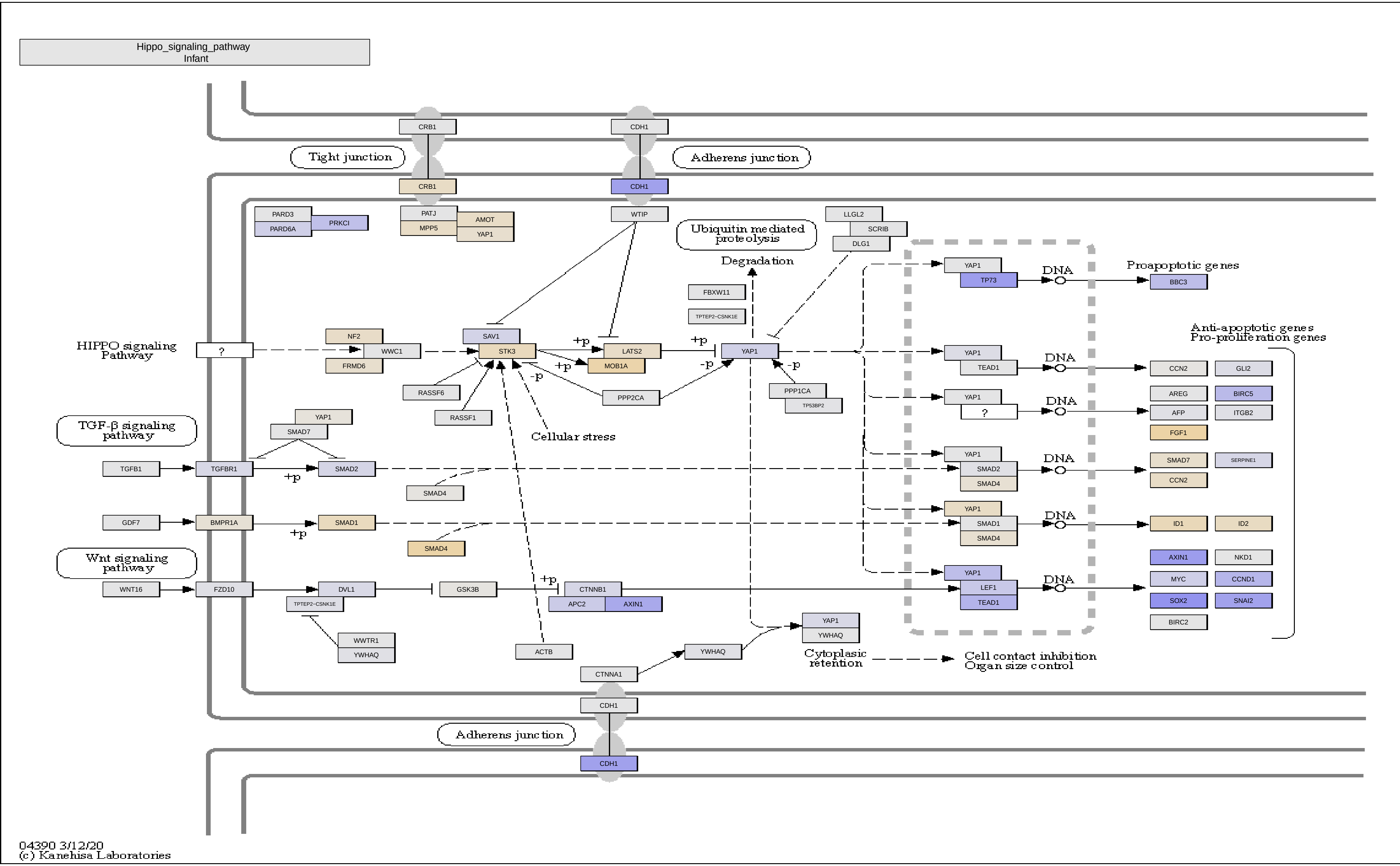
Hippo_signaling_pathway
Infant
CRB1
CDH1
Tight junction
Adherens junction
CRB1
CDH1
PARD3
PARD6A
PRKCI
PATJ
MPP5
AMOT
YAP1
WTIP
Ubiquitin mediated proteolysis
Degradation
LLGL2
SCRIB
DLG1
YAP1
TP73
DNA
Proapoptotic genes
BBC3
FBXW11
TPTEP2-CSNK1E
Anti-apoptotic genes
Pro-proliferation genes
HIPPO signaling Pathway
?
NF2
WWC1
FRMD6
SAV1
STK3
+p
LATS2
MOB1A
+p
YAP1
-p
-p
YAP1
TEAD1
DNA
CCN2
GLI2
RASSF6
RASSF1
-p
PPP2CA
PPP1CA
TP53BP2
YAP1
?
DNA
AREG
BIRC5
AFP
ITGB2
FGF1
Cellular stress
TGF-β signaling pathway
YAP1
SMAD7
TGFB1
TGFBR1
+p
SMAD2
SMAD4
YAP1
SMAD2
SMAD4
DNA
SMAD7
SERPINE1
CCN2
GDF7
BMPR1A
+p
SMAD1
SMAD4
YAP1
SMAD1
SMAD4
DNA
ID1
ID2
Wnt signaling pathway
WNT16
FZD10
DVL1
TPTEP2-CSNK1E
GSK3B
+p
CTNNB1
APC2
AXIN1
YAP1
LEF1
TEAD1
DNA
AXIN1
NKD1
MYC
CCND1
SOX2
SNAI2
BIRC2
WWTR1
YWHAQ
ACTB
YAP1
YWHAQ
YWHAQ
Cytoplasic retention
Cell contact inhibition
Organ size control
CTNNA1
CDH1
Adherens junction
CDH1
04390 3/12/20
(c) Kanehisa Laboratories

Hippo_signaling_pathway
Teenager

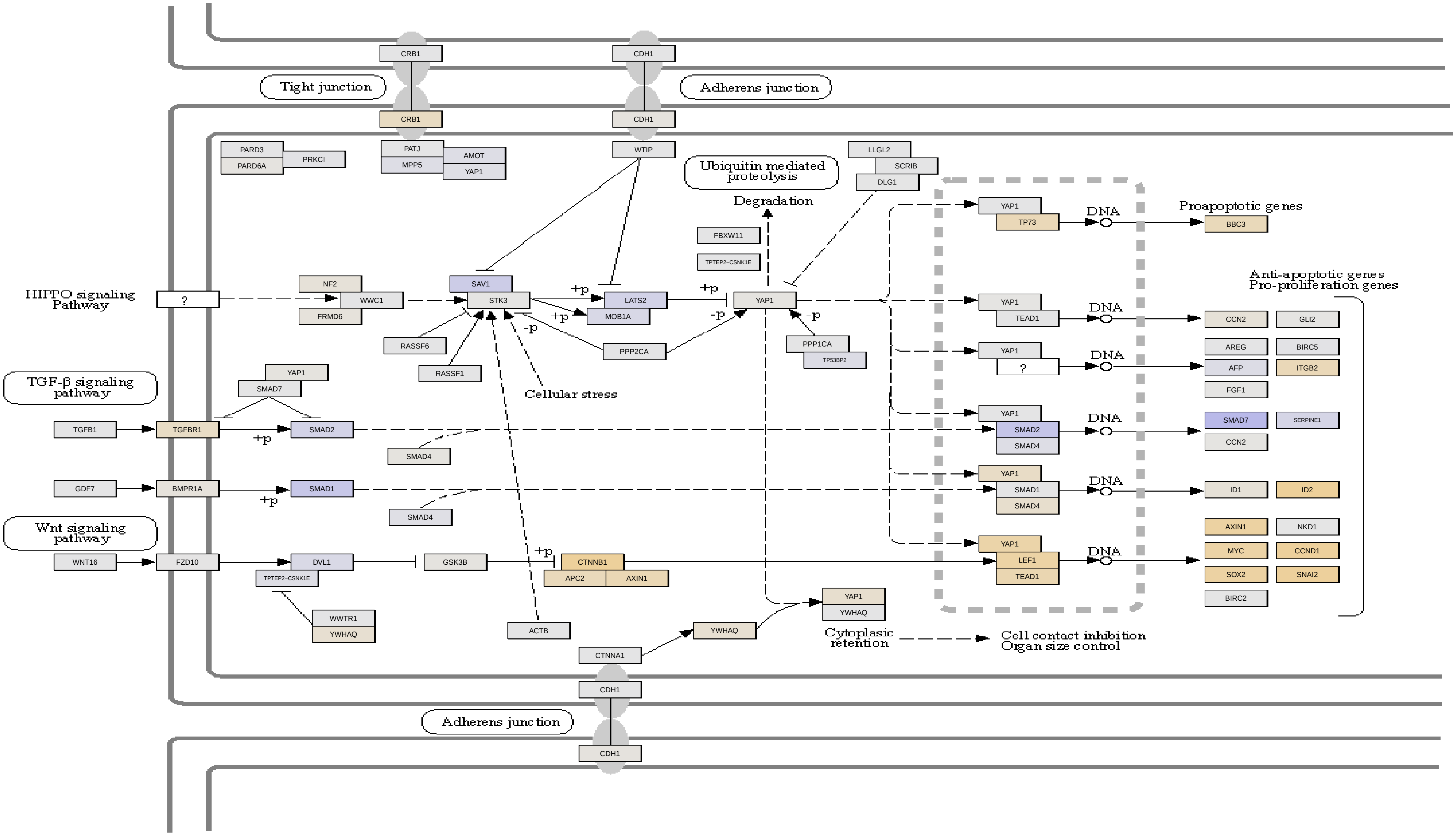

04390 3/12/20
(c) Kanehisa Laboratories

Hippo_signaling_pathway
Adult

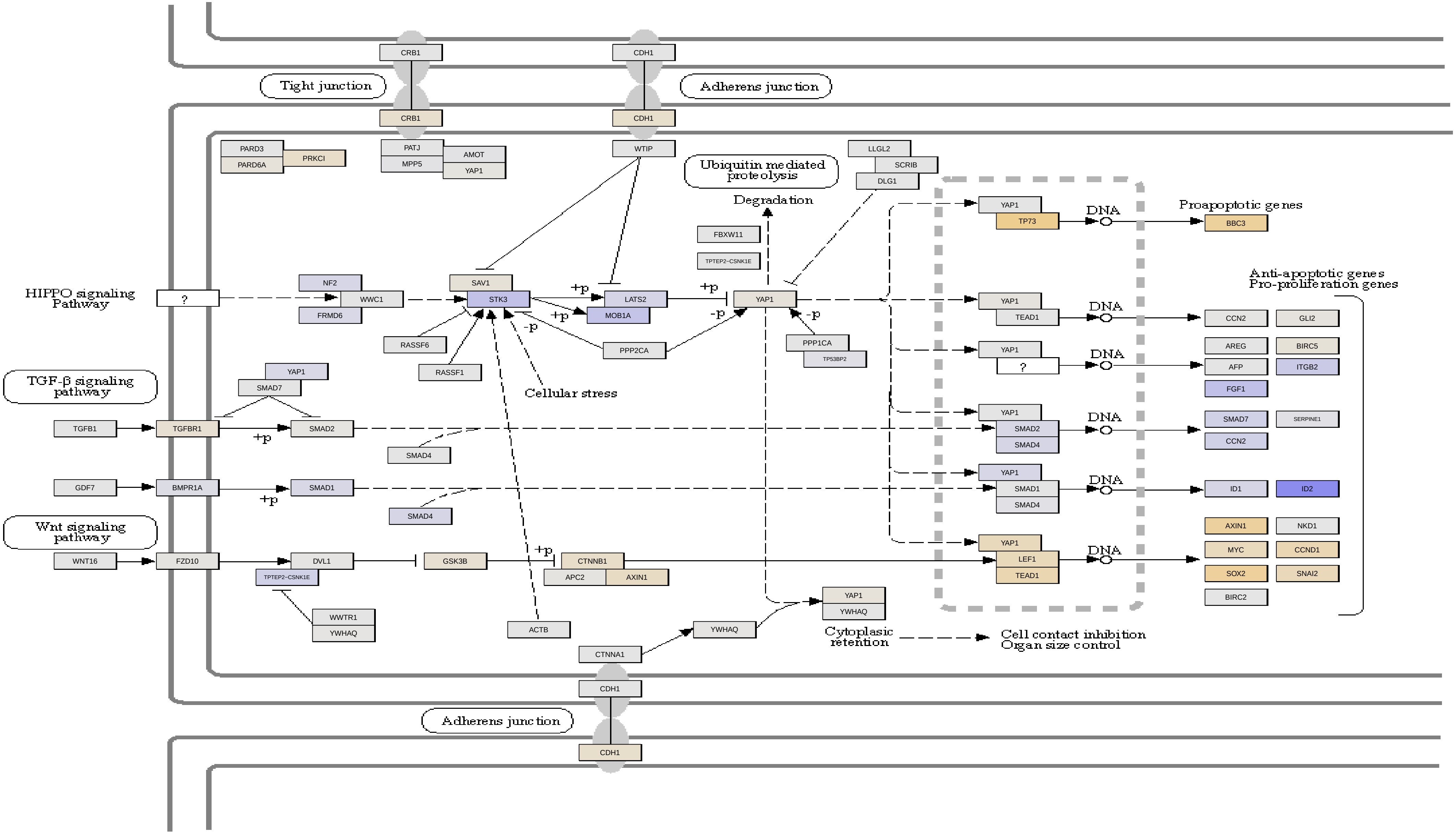

04390 3/12/20
(c) Kanehisa Laboratories

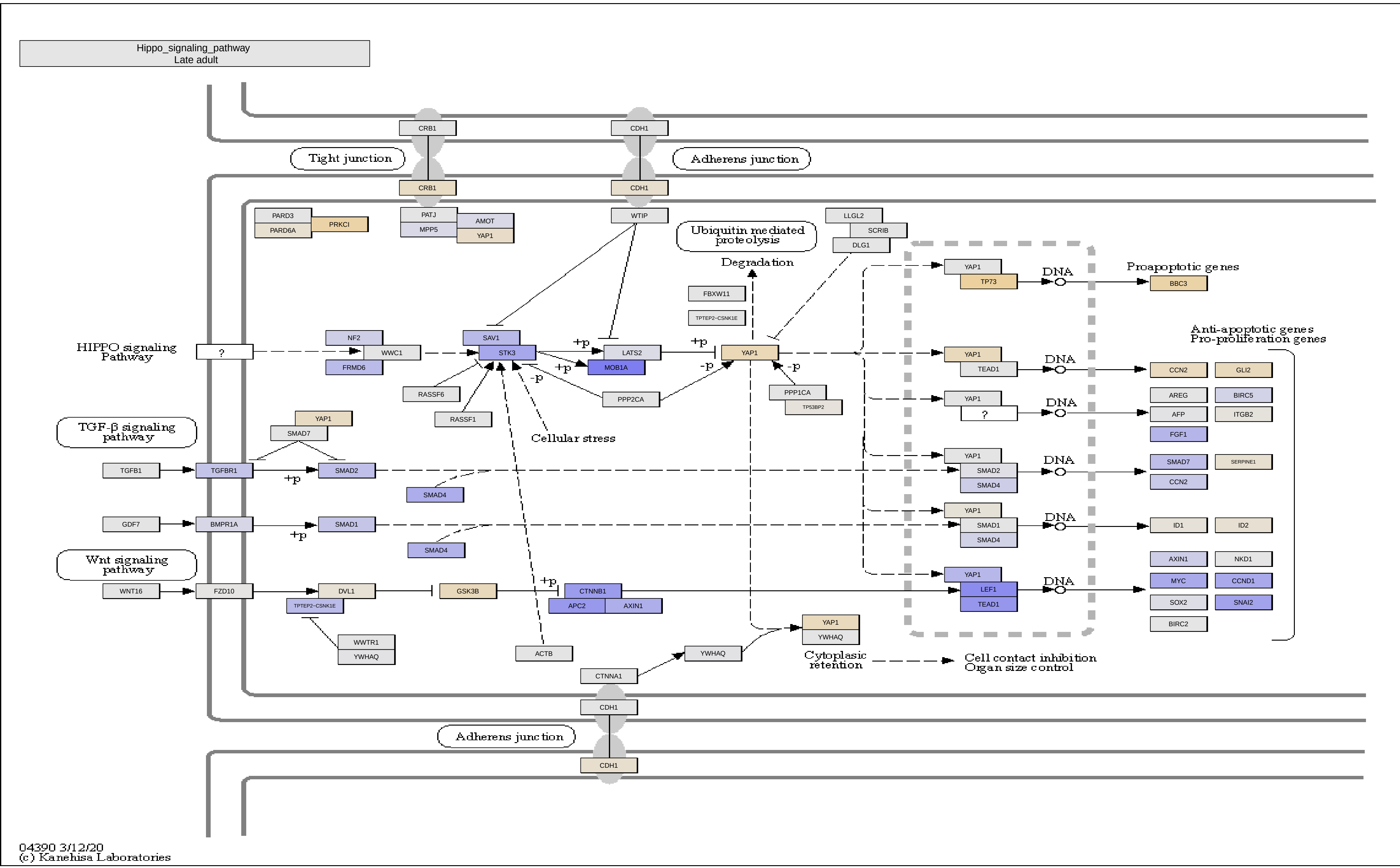

Hippo_signaling_pathway
Late adult
CRB1
CDH1
Tight junction
Adherens junction
CRB1
CDH1
PARD3
PARD6A
PRKCI
PATJ
MPP5
AMOT
YAP1
WTIP
Ubiquitin mediated proteolysis
Degradation
LLGL2
SCRIB
DLG1
YAP1
TP73
DNA
Proapoptotic genes
BBC3
FBXW11
TPTEP2-CSNK1E
Anti-apoptotic genes
Pro-proliferation genes
HIPPO signaling Pathway
?
NF2
WWC1
FRMD6
SAV1
STK3
+p
LATS2
MOB1A
+p
-p
YAP1
-p
YAP1
TEAD1
DNA
CCN2
GLI2
RASSF6
RASSF1
PPP2CA
PPP1CA
TP53BP2
YAP1
?
DNA
AREG
BIRC5
AFP
ITGB2
FGF1
YAP1
SMAD7
TGF-β signaling pathway
Cellular stress
TGFB1
TGFBR1
SMAD2
SMAD4
YAP1
SMAD2
SMAD4
DNA
SMAD7
SERPINE1
CCN2
GDF7
BMPR1A
SMAD1
SMAD4
YAP1
SMAD1
SMAD4
DNA
ID1
ID2
Wnt signaling pathway
WNT16
FZD10
DVL1
TPTEP2-CSNK1E
GSK3B
CTNNB1
APC2
AXIN1
YAP1
LEF1
TEAD1
DNA
AXIN1
NKD1
MYC
CCND1
SOX2
SNAI2
BIRC2
WWTR1
YWHAQ
ACTB
YWHAQ
YAP1
YWHAQ
Cytoplasic retention
Cell contact inhibition
Organ size control
CTNNA1
CDH1
Adherens junction
CDH1
04390 3/12/20
(c) Kanehisa Laboratories
