# Supplementary material for: The Transcriptome and Methylome of the Developing and Aging Brain and Their Relations to Gliomas and Psychological Disorders
Source: Cells. 2022 Jan 21;11(3):362. doi: 10.3390/cells11030362 (PMC8834030; doi:10.3390/cells11030362)
Supplement: Supplementary file 1 [file cells-11-00362-s001.zip › Suppl-Material-S4-Pathways-PSF_Methylation/JAK_STAT_signaling_pathway.pdf]

JAK−STAT signaling pathway

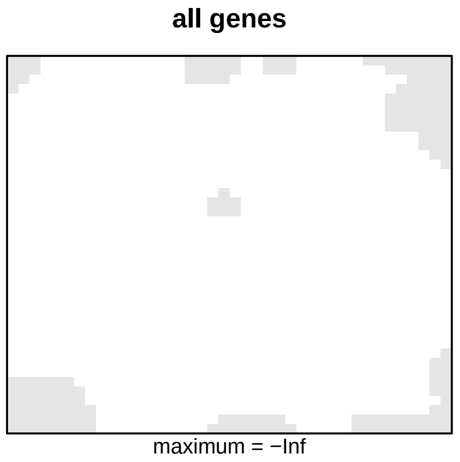

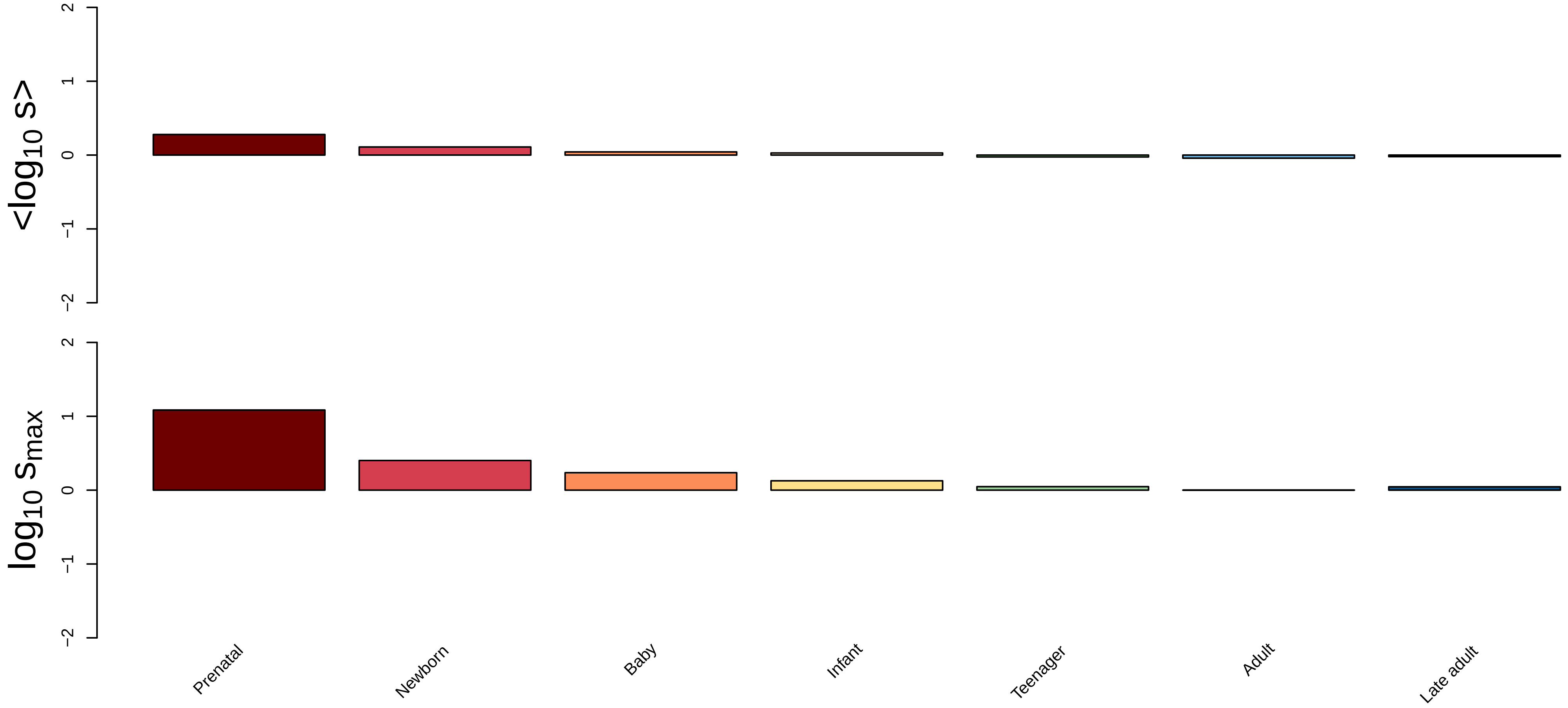

JAK_STAT_signaling_pathway
genes with data

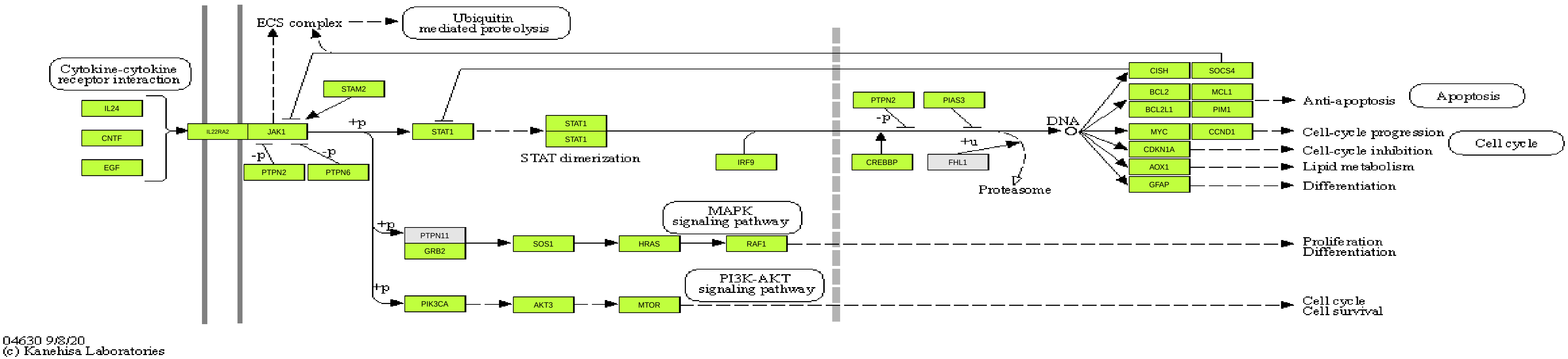

JAK_STAT_signaling_pathway
sink nodes

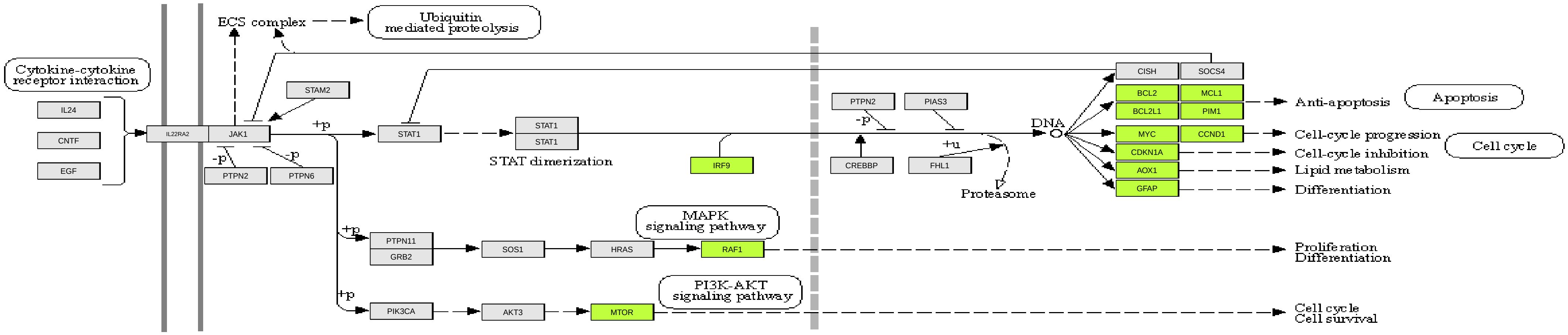

04630 9/8/20
(c) Kanehisa Laboratories

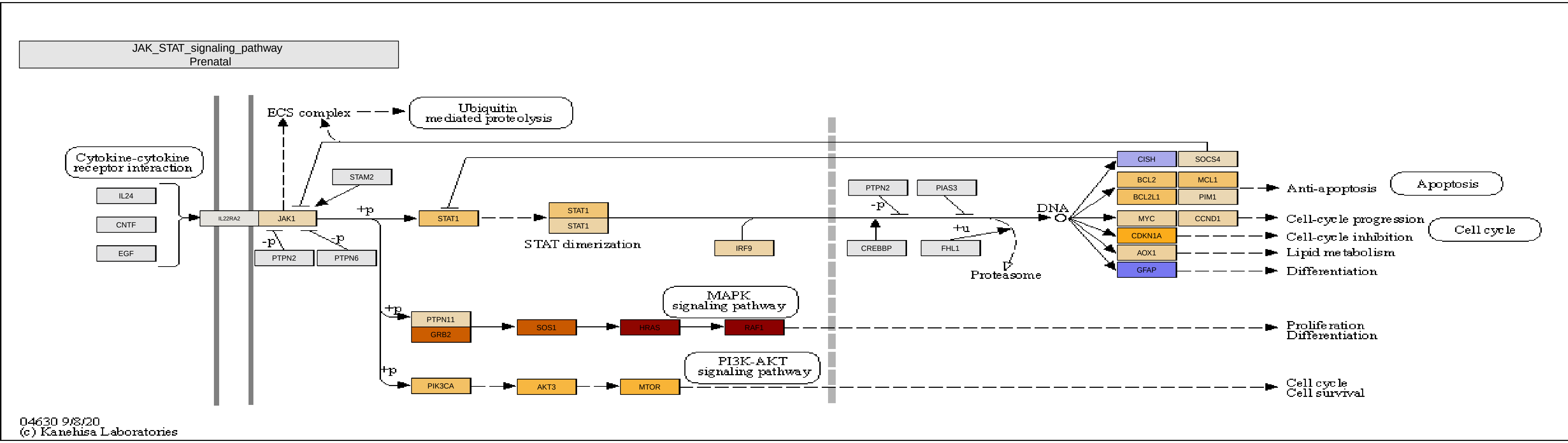
JAK_STAT_signaling_pathway
Prenatal
ECS complex
Ubiquitin mediated proteolysis
Cytokine-cytokine receptor interaction
IL24
CNTF
EGF
IL22RA2
JAK1
STAM2
+p
-p
PTPN2
PTPN6
STAT1
STAT1
STAT1
STAT dimerization
IRF9
PTPN2
PIAS3
CREBBP
FHL1
+u
Proteasome
DNA
CISH
SOCS4
BCL2
MCL1
BCL2L1
PIM1
MYC
CCND1
CDKN1A
AOX1
GFAP
Anti-apoptosis
Apoptosis
Cell-cycle progression
Cell-cycle inhibition
Cell cycle
Lipid metabolism
Differentiation
MAPK signaling pathway
PTPN11
GRB2
SOS1
HRAS
RAF1
Proliferation
Differentiation
PI3K-AKT signaling pathway
PIK3CA
AKT3
MTOR
Cell cycle
Cell survival
04630 9/8/20
(c) Kanehisa Laboratories

JAK_STAT_signaling_pathway
Newborn

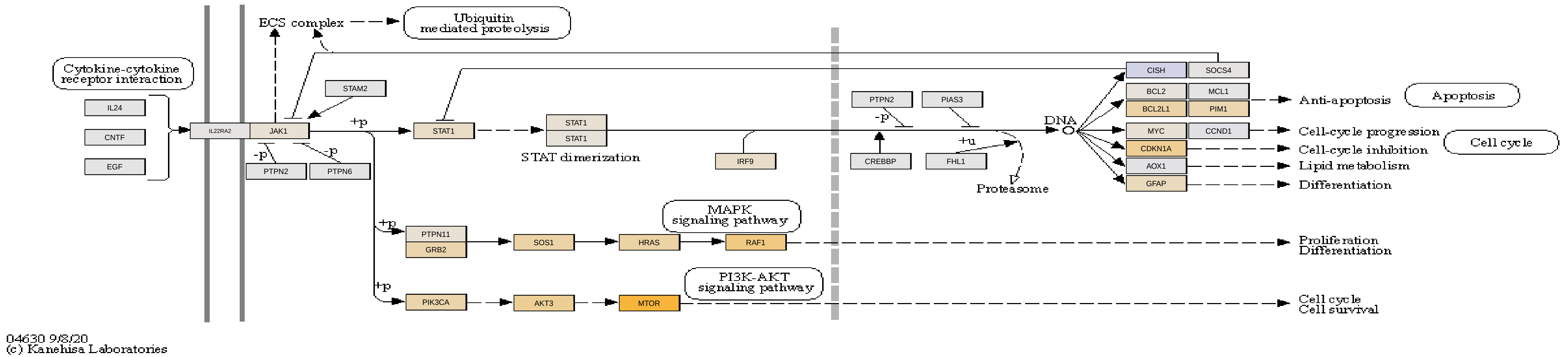

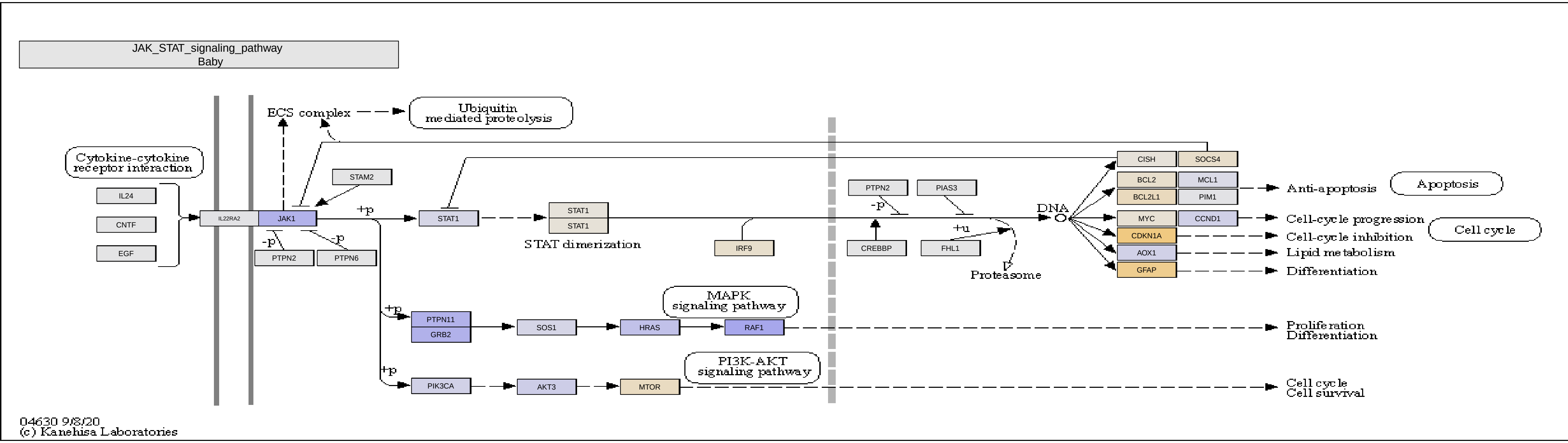

JAK_STAT_signaling_pathway
Baby
ECS complex
Ubiquitin mediated proteolysis
Cytokine-cytokine receptor interaction
IL24
CNTF
EGF
IL22RA2
JAK1
STAM2
PTPN2
PTPN6
+p
-p
STAT1
STAT dimerization
IRF9
PTPN2
PIAS3
CREBBP
FHL1
+u
Proteasome
DNA
CISH
SOCS4
BCL2
MCL1
BCL2L1
PIM1
MYC
CCND1
CDKN1A
AOX1
GFAP
Anti-apoptosis
Apoptosis
Cell-cycle progression
Cell-cycle inhibition
Lipid metabolism
Differentiation
Cell cycle
PTPN11
GRB2
SOS1
HRAS
RAF1
MAPK signaling pathway
Proliferation
Differentiation
PIK3CA
AKT3
MTOR
PI3K-AKT signaling pathway
Cell cycle
Cell survival
04630 9/8/20
(c) Kanehisa Laboratories

JAK_STAT_signaling_pathway
Infant

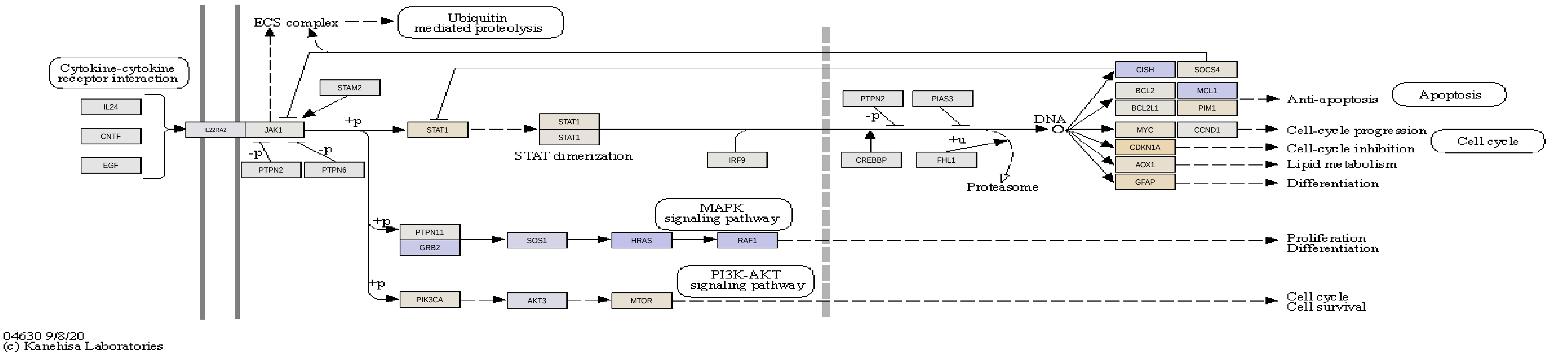

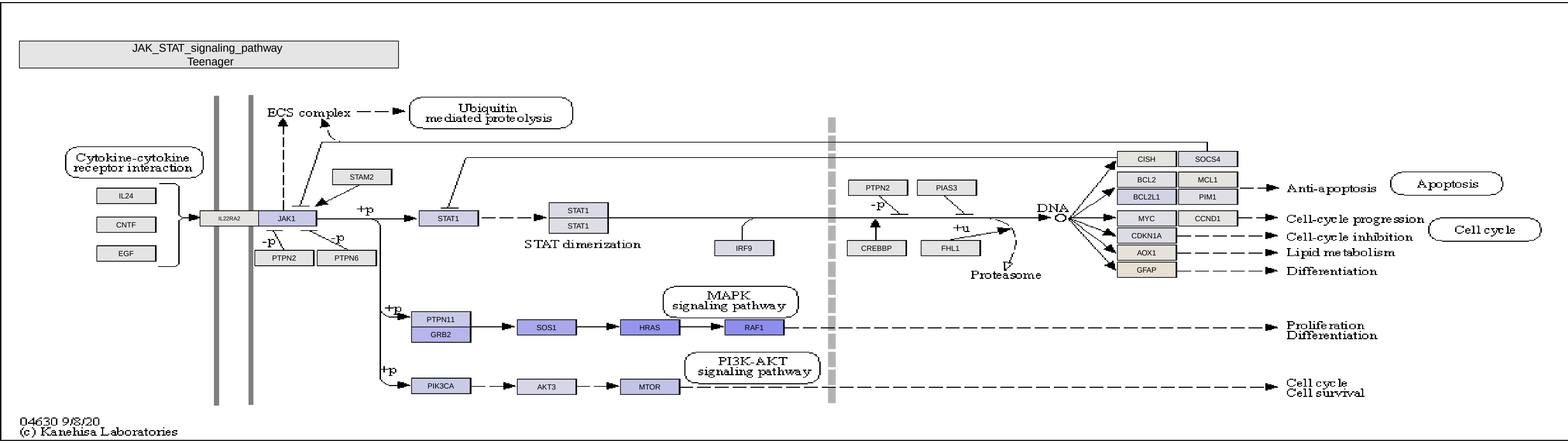
JAK_STAT_signaling_pathway
Teenager
ECS complex
Ubiquitin
mediated proteolysis
Cytokine-cytokine
receptor interaction
IL24
CNTF
EGF
IL22RA2
JAK1
STAM2
+p
-p
-p
PTPN2
PTPN6
STAT1
STAT1
STAT1
STAT dimerization
IRF9
PTPN2
PIAS3
-p
CREBBP
FHL1
+u
Proteasome
DNA
CISH
SOCS4
BCL2
MCL1
BCL2L1
PIM1
MYC
CCND1
CDKN1A
AOX1
GFAP
Anti-apoptosis
Apoptosis
Cell-cycle progression
Cell-cycle inhibition
Cell cycle
Lipid metabolism
Differentiation
+p
PTPN11
GRB2
SOS1
HRAS
RAF1
MAPK
signaling pathway
Proliferation
Differentiation
+p
PIK3CA
AKT3
MTOR
PI3K-AKT
signaling pathway
Cell cycle
Cell survival
04630 9/8/20
(c) Kanehisa Laboratories

JAK_STAT_signaling_pathway
Adult

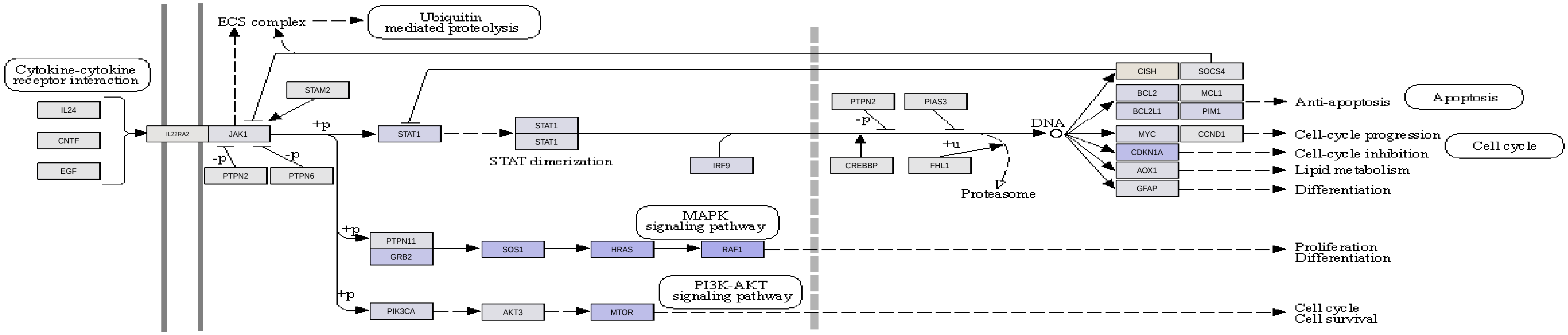

04630 9/8/20
(c) Kanehisa Laboratories

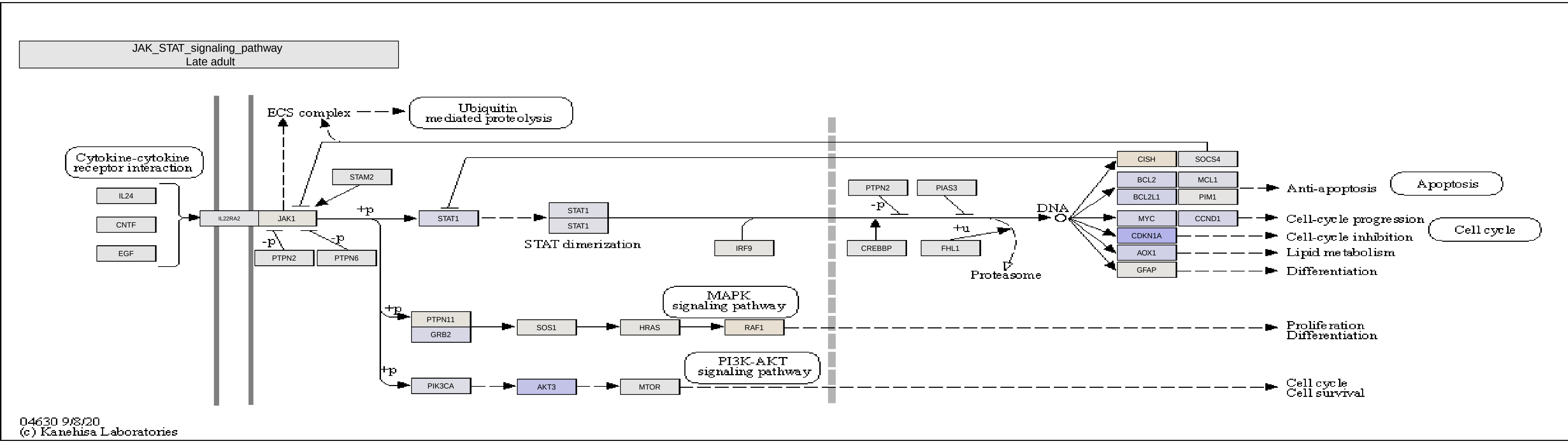
JAK_STAT_signaling_pathway
Late adult
ECS complex
Ubiquitin mediated proteolysis
Cytokine-cytokine receptor interaction
IL24
CNTF
EGF
IL22RA2
JAK1
STAM2
PTPN2
PTPN6
STAT1
STAT1
STAT1
STAT dimerization
IRF9
PTPN2
PIAS3
CREBBP
FHL1
Proteasome
DNA
CISH
SOCS4
BCL2
MCL1
BCL2L1
PIM1
MYC
CCND1
CDKN1A
AOX1
GFAP
Anti-apoptosis
Apoptosis
Cell-cycle progression
Cell-cycle inhibition
Lipid metabolism
Differentiation
Cell cycle
MAPK signaling pathway
PTPN11
GRB2
SOS1
HRAS
RAF1
Proliferation
Differentiation
PI3K-AKT signaling pathway
PIK3CA
AKT3
MTOR
Cell cycle
Cell survival
04630 9/8/20
(c) Kanehisa Laboratories
