# Supplementary material for: The Transcriptome and Methylome of the Developing and Aging Brain and Their Relations to Gliomas and Psychological Disorders
Source: Cells. 2022 Jan 21;11(3):362. doi: 10.3390/cells11030362 (PMC8834030; doi:10.3390/cells11030362)
Supplement: Supplementary file 1 [file cells-11-00362-s001.zip › Suppl-Material-S4-Pathways-PSF_Methylation/MAPK_signaling_pathway.pdf]

MAPK signaling pathway

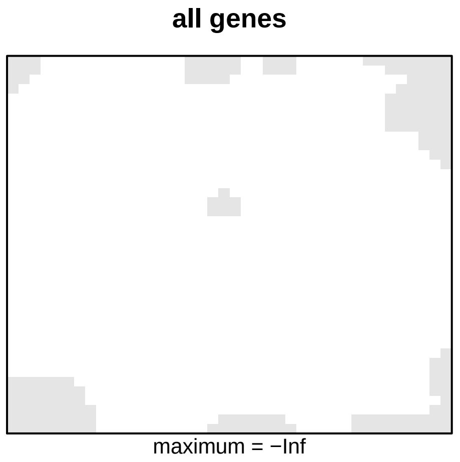

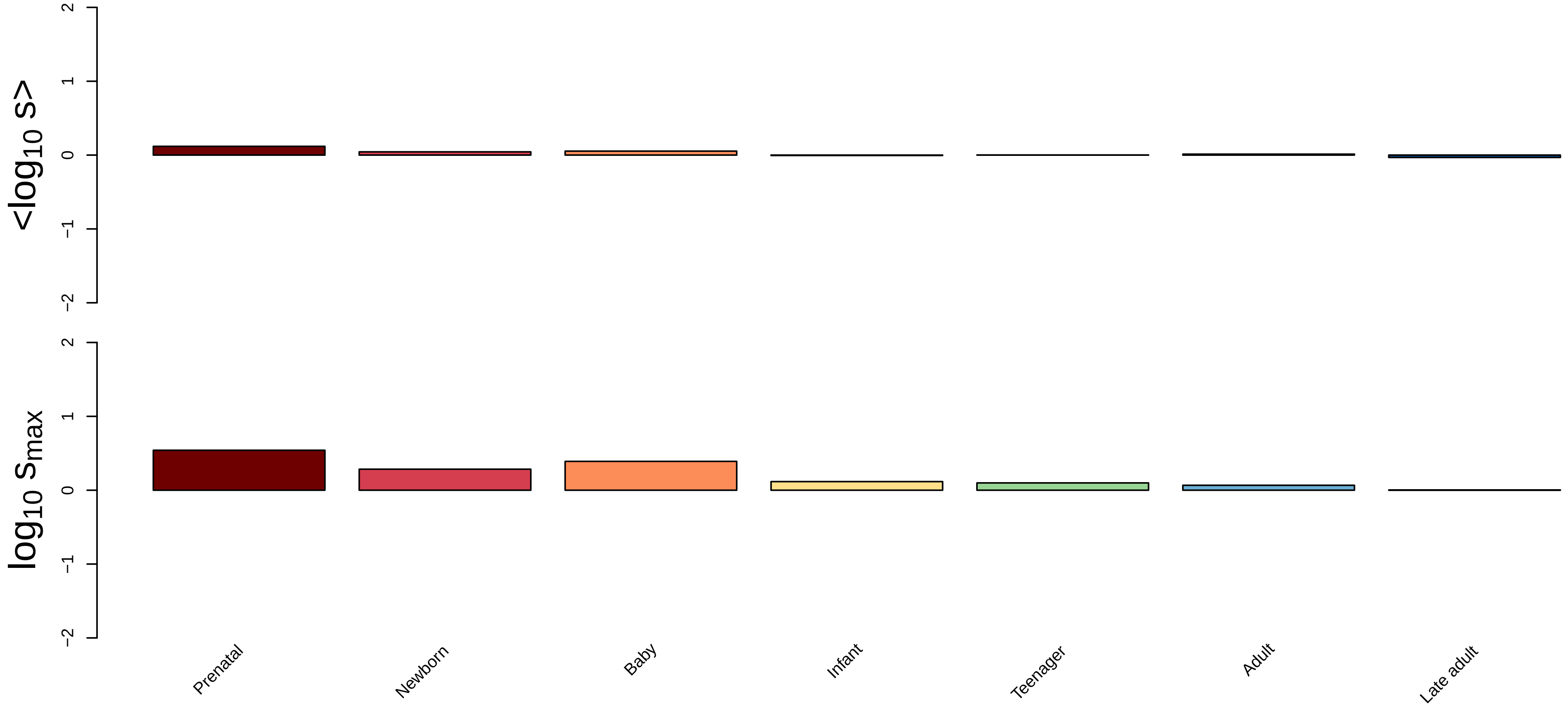

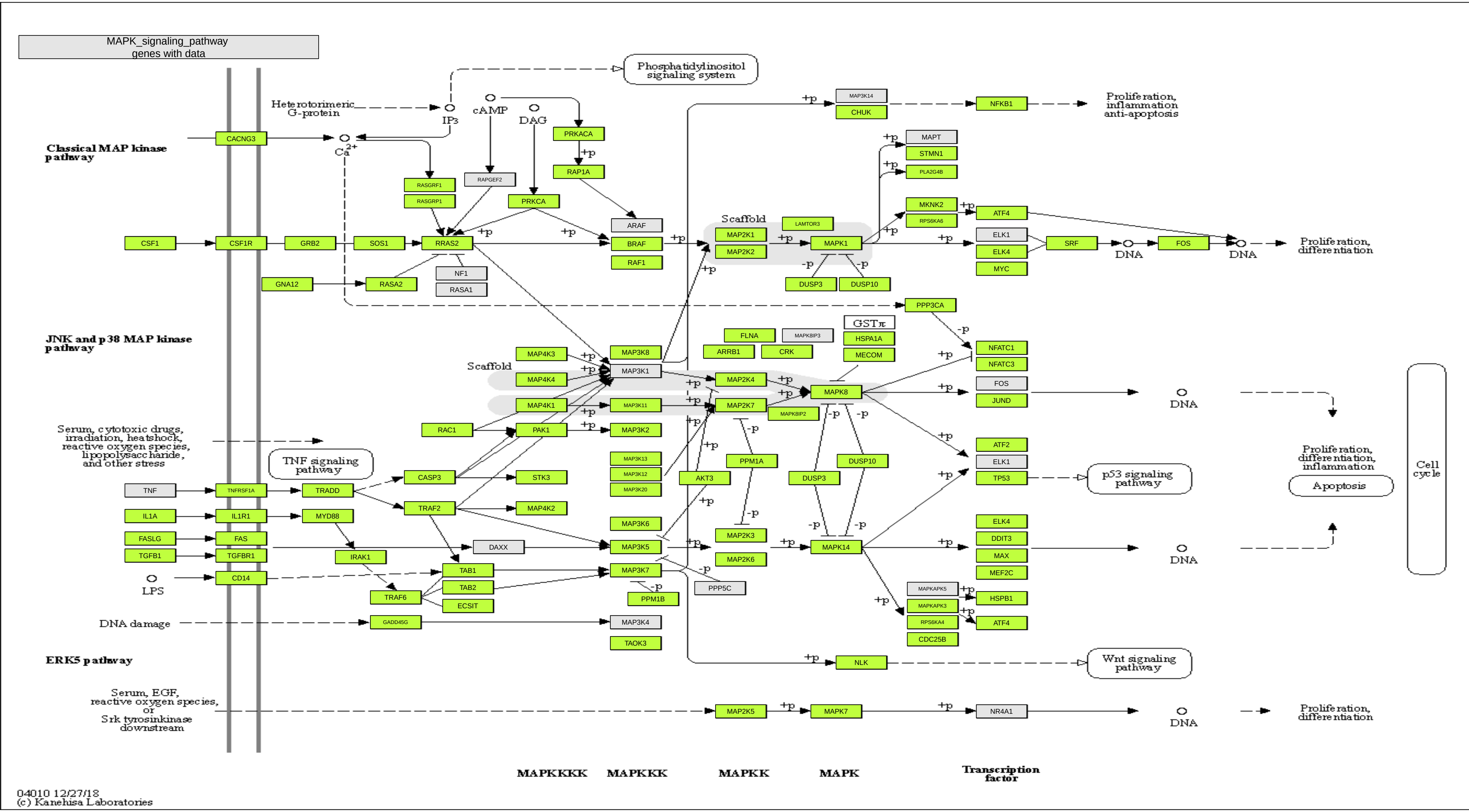
MAPK_signaling_pathway
genes with data
Phosphatidylinositol signaling system
Heterotorimeric G-protein
IP3
cAMP
DAG
Ca2+
Classical MAP kinase pathway
CACNG3
PRKACA
RAP1A
RASGRF1
RASGRP1
RAPGEF2
PRKCA
ARAF
BRAF
RAF1
CSF1
CSF1R
GRB2
SOS1
RRAS2
NF1
RASA1
GNA12
RASA2
Scaffold
LAMTOR3
MAP2K1
MAP2K2
MAPK1
DUSP3
DUSP10
MAP3K14
CHUK
NFKB1
Proliferation, inflammation anti-apoptosis
MAPT
STMN1
PLA2G4B
MKNK2
RPS6KA6
ATF4
ELK1
ELK4
MYC
SRF
FOS
DNA
Proliferation, differentiation
PPP3CA
NFATC1
NFATC3
JNK and p38 MAP kinase pathway
MAP4K3
MAP4K4
MAP4K1
MAP3K8
MAP3K1
MAP3K11
MAP3K2
FLNA
MAPK8IP3
GSTπ
HSPA1A
MECOM
ARRB1
CRK
MAP2K4
MAP2K7
MAPK8IP2
MAPK8
JUND
RAC1
PAK1
Serum, cytotoxic drugs, irradiation, heatshock, reactive oxygen species, lipopolysaccharide, and other stress
TNF signaling pathway
MAP3K13
MAP3K12
MAP3K20
PPM1A
AKT3
DUSP3
DUSP10
ATF2
ELK1
TP53
p53 signaling pathway
Proliferation, differentiation, inflammation
Apoptosis
Cell cycle
TNF
TNFRSF1A
TRADD
CASP3
STK3
IL1A
IL1R1
MYD88
TRAF2
MAP4K2
MAP3K6
MAP3K5
DAXX
FASLG
FAS
TGFB1
TGFBR1
IRAK1
MAP2K3
MAP2K6
MAPK14
ELK4
DDIT3
MAX
MEF2C
LPS
CD14
TAB1
TAB2
TRAF6
ECSIT
MAP3K7
PPM1B
PPP5C
MAPKAPK5
MAPKAPK3
RPS6KA4
CDC25B
HSPB1
ATF4
DNA damage
GADD45G
MAP3K4
TAOK3
NLK
Wnt signaling pathway
ERK5 pathway
Serum, EGF, reactive oxygen species, or Srk tyrosinkinase downstream
MAP2K5
MAPK7
NR4A1
+p
-p
MAPKKKK
MAPKKK
MAPKK
MAPK
Transcription factor
04010 12/27/18
(c) Kanehisa Laboratories

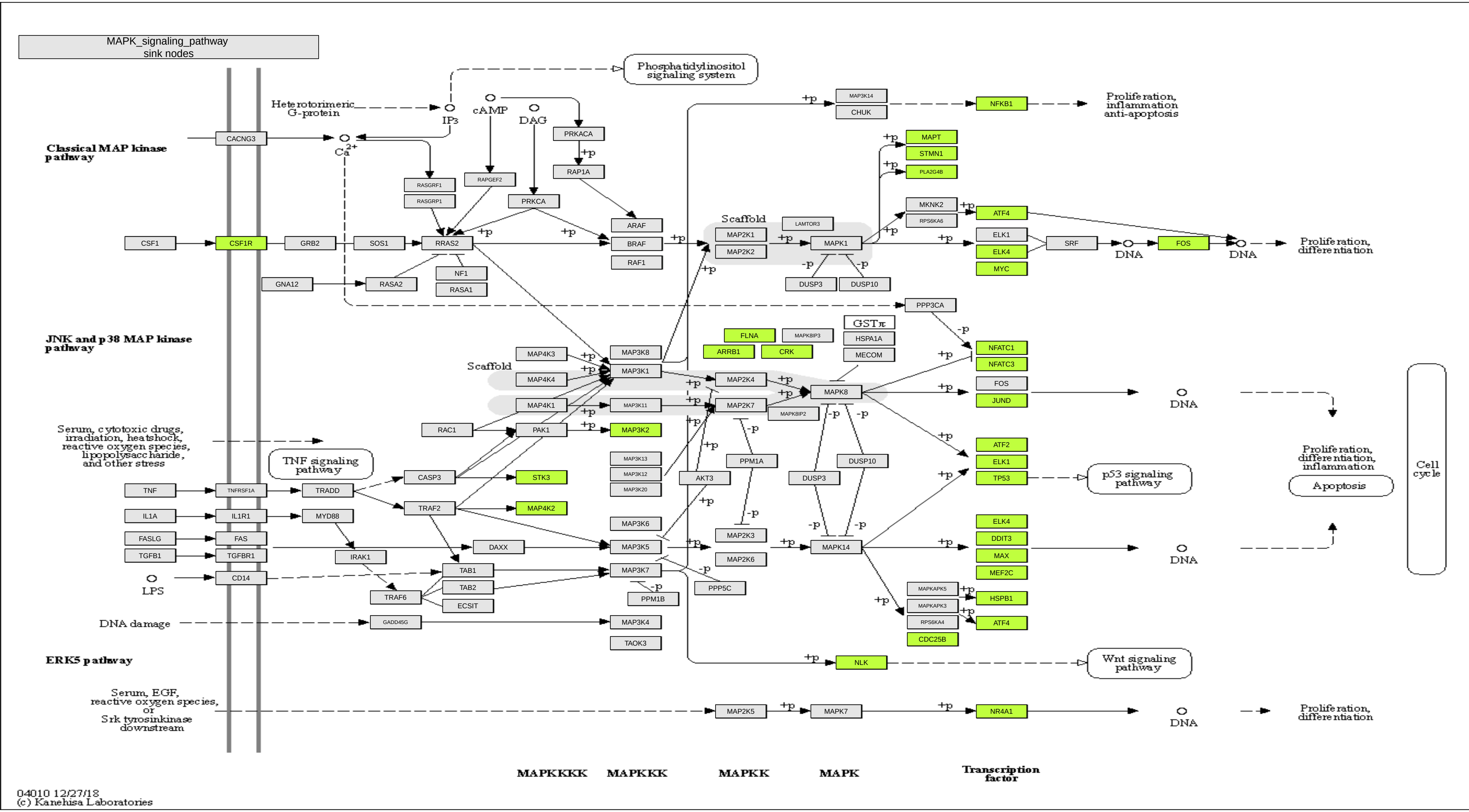
MAPK_signaling_pathway
sink nodes
Classical MAP kinase pathway
JNK and p38 MAP kinase pathway
ERK5 pathway
Heterotorimeric G-protein
IP3
cAMP
DAG
Ca2+
Phosphatidylinositol signaling system
CACNG3
PRKACA
RAP1A
RASGRF1
RASGRP1
RAPGEF2
PRKCA
CSF1
CSF1R
GRB2
SOS1
RRAS2
ARAF
BRAF
RAF1
NF1
RASA1
GNA12
RASA2
Scaffold
LAMTOR3
MAP2K1
MAP2K2
MAPK1
DUSP3
DUSP10
MAP3K14
CHUK
NFKB1
Proliferation, inflammation anti-apoptosis
MAPT
STMN1
PLA2G4B
MKNK2
RPS6KA6
ATF4
ELK1
ELK4
MYC
SRF
FOS
DNA
Proliferation, differentiation
PPP3CA
NFATC1
NFATC3
FOS
JUND
FLNA
MAPK8IP3
ARRB1
CRK
GST π
HSPA1A
MECOM
MAP4K3
MAP4K4
MAP4K1
MAP3K8
MAP3K1
MAP3K11
MAP3K2
MAP2K4
MAP2K7
MAPK8IP2
MAPK8
RAC1
PAK1
Serum, cytotoxic drugs, irradiation, heatshock, reactive oxygen species, lipopolysaccharide, and other stress
TNF signaling pathway
CASP3
STK3
MAP3K13
MAP3K12
MAP3K20
AKT3
PPM1A
DUSP10
DUSP3
ATF2
ELK1
TP53
p53 signaling pathway
Proliferation, differentiation, inflammation
Apoptosis
Cell cycle
TNF
TNFRSF1A
TRADD
TRAF2
MAP4K2
IL1A
IL1R1
MYD88
FASLG
FAS
TGFB1
TGFBR1
LPS
CD14
IRAK1
DAXX
MAP3K6
MAP3K5
MAP2K3
MAP2K6
MAPK14
ELK4
DDIT3
MAX
MEF2C
TAB1
TAB2
TRAF6
ECSIT
MAP3K7
PPM1B
PPP5C
MAPKAPK5
MAPKAPK3
RPS6KA4
HSPB1
ATF4
CDC25B
DNA damage
GADD45G
MAP3K4
TAOK3
NLK
Wnt signaling pathway
Serum, EGF, reactive oxygen species, or Srk tyrosinkinase downstream
MAP2K5
MAPK7
NR4A1
+p
-p
MAPKKKK
MAPKKK
MAPKK
MAPK
Transcription factor
04010 12/27/18
(c) Kanehisa Laboratories

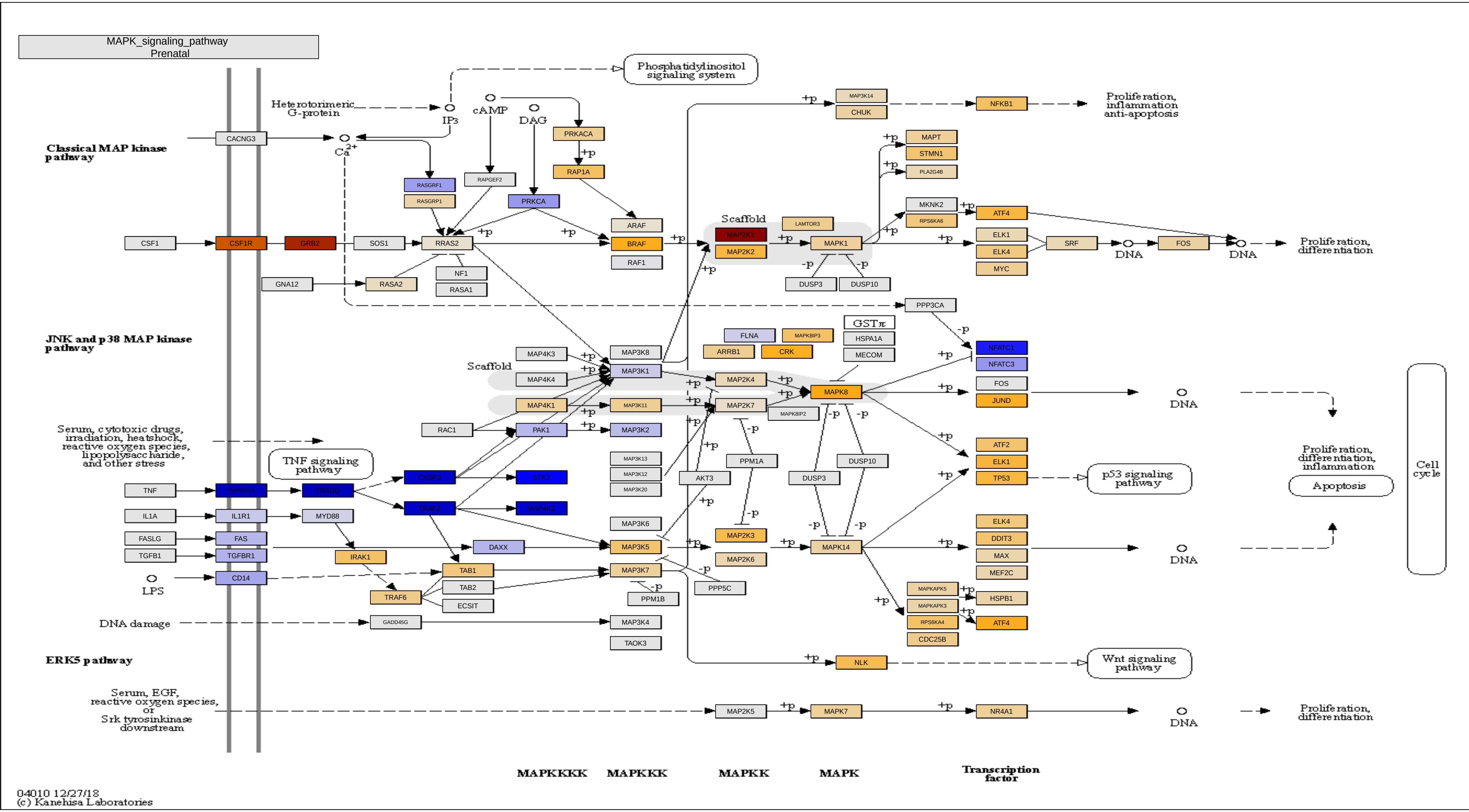
MAPK_signaling_pathway
Prenatal
Classical MAP kinase pathway
JNK and p38 MAP kinase pathway
ERK5 pathway
Heterotorimeric G-protein
IP3
cAMP
DAG
Ca2+
Phosphatidylinositol signaling system
CACNG3
RASGRF1
RASGRP1
RAPGEF2
PRKCA
PRKACA
RAP1A
CSF1
CSF1R
GRB2
SOS1
RRAS2
ARAF
BRAF
RAF1
NF1
RASA1
GNA12
RASA2
Scaffold
MAP2K1
MAP2K2
LAMTOR3
MAPK1
DUSP3
DUSP10
MAP3K14
CHUK
NFKB1
Proliferation, inflammation anti-apoptosis
MAPT
STMN1
PLA2G4B
MKNK2
RPS6KA6
ATF4
ELK1
ELK4
MYC
SRF
FOS
DNA
Proliferation, differentiation
PPP3CA
NFATC1
NFATC3
FOS
JUND
Serum, cytotoxic drugs, irradiation, heatshock, reactive oxygen species, lipopolysaccharide, and other stress
TNF signaling pathway
MAP4K3
MAP4K4
MAP4K1
MAP3K8
MAP3K1
MAP3K11
MAP3K2
MAP3K13
MAP3K12
MAP3K20
MAP3K6
MAP3K5
MAP3K7
MAP3K4
TAOK3
FLNA
MAPK8IP3
ARRB1
CRK
GSTπ
HSPA1A
MECOM
MAP2K4
MAP2K7
MAPK8IP2
MAPK8
PPM1A
AKT3
DUSP3
DUSP10
ATF2
ELK1
TP53
p53 signaling pathway
Proliferation, differentiation, inflammation
Apoptosis
Cell cycle
RAC1
PAK1
CASP3
STK3
TNF
TNFRSF1A
TRADD
TRAF2
MAP4K2
IL1A
IL1R1
MYD88
IRAK1
FASLG
FAS
DAXX
TGFB1
TGFBR1
LPS
CD14
TAB1
TAB2
TRAF6
ECSIT
PPM1B
PPP5C
MAP2K3
MAP2K6
MAPK14
ELK4
DDIT3
MAX
MEF2C
MAPKAPK5
MAPKAPK3
RPS6KA4
CDC25B
HSPB1
ATF4
DNA damage
GADD45G
NLK
Wnt signaling pathway
Serum, EGF, reactive oxygen species, or Srk tyrosinkinase downstream
MAP2K5
MAPK7
NR4A1
+p
-p
MAPKKKK
MAPKKK
MAPKK
MAPK
Transcription factor
04010 12/27/18
(c) Kanehisa Laboratories

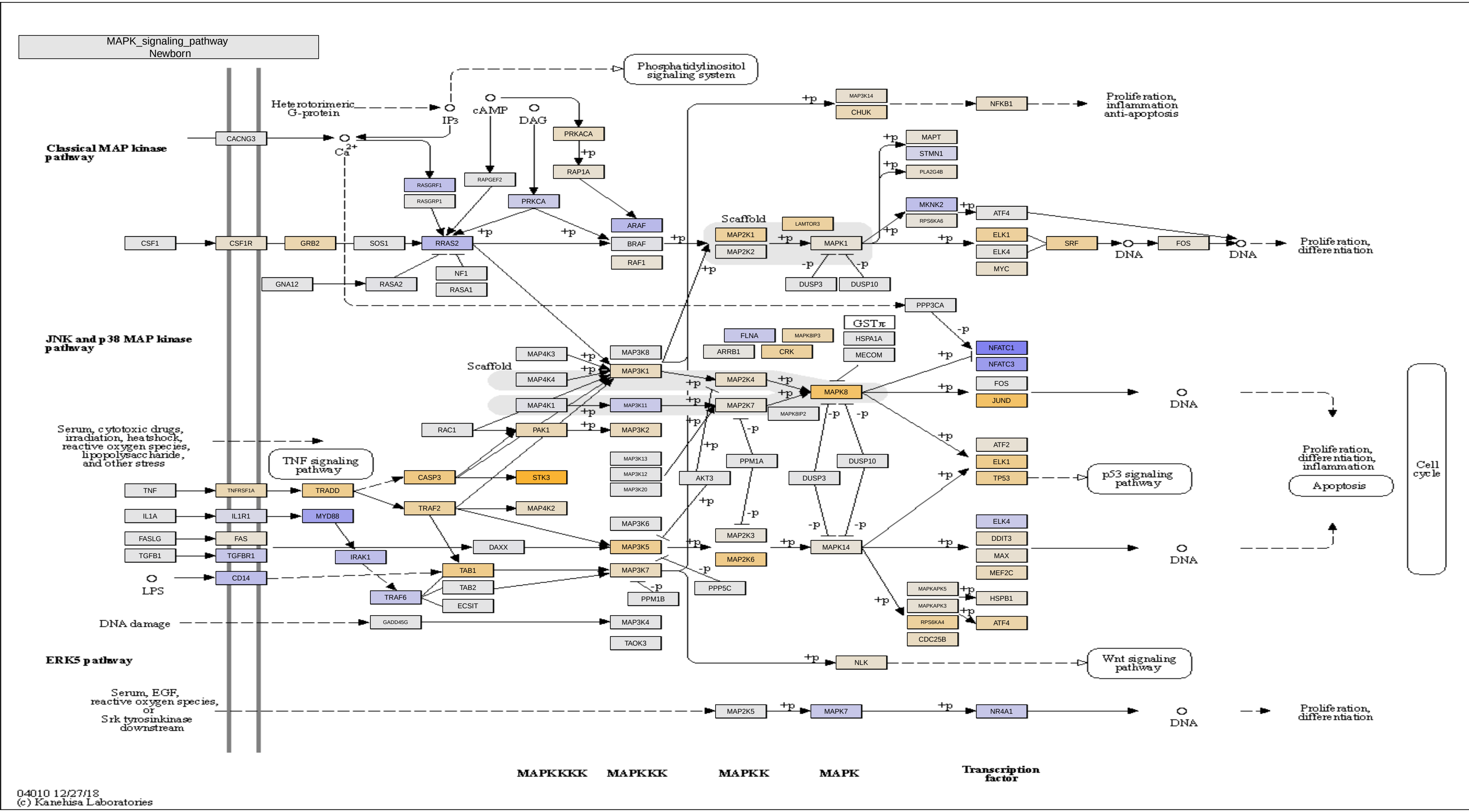
MAPK_signaling_pathway
Newborn
Classical MAP kinase pathway
JNK and p38 MAP kinase pathway
ERK5 pathway
Heterotorimeric G-protein
IP3
cAMP
DAG
Ca^{2+}
Phosphatidylinositol signaling system
CACNG3
CSF1
CSF1R
GRB2
SOS1
RRAS2
RASGRF1
RASGRP1
RAPGEF2
PRKCA
PRKACA
RAP1A
ARAF
BRAF
RAF1
GNA12
RASA2
NF1
RASA1
Scaffold
LAMTOR3
MAP2K1
MAP2K2
MAPK1
DUSP3
DUSP10
MAP3K14
CHUK
NFKB1
Proliferation, inflammation anti-apoptosis
MAPT
STMN1
PLA2G4B
MKNK2
RPS6KA6
ATF4
ELK1
ELK4
MYC
SRF
FOS
DNA
Proliferation, differentiation
PPP3CA
Serum, cytotoxic drugs, irradiation, heatshock, reactive oxygen species, lipopolysaccharide, and other stress
TNF signaling pathway
MAP4K3
MAP4K4
MAP4K1
MAP3K8
MAP3K1
MAP3K11
MAP3K2
MAP3K13
MAP3K12
MAP3K20
MAP3K6
MAP3K5
MAP3K7
MAP3K4
TAOK3
RAC1
PAK1
CASP3
STK3
TRAF2
MAP4K2
TNF
TNFRSF1A
TRADD
IL1A
IL1R1
MYD88
FASLG
FAS
DAXX
TGFB1
TGFBR1
IRAK1
LPS
CD14
TAB1
TAB2
TRAF6
ECSIT
PPM1B
DNA damage
GADD45G
FLNA
MAPK8IP3
ARRB1
CRK
GSTπ
HSPA1A
MECOM
MAP2K4
MAP2K7
MAPK8IP2
MAPK8
PPM1A
AKT3
DUSP10
DUSP3
MAP2K3
MAP2K6
PPP5C
MAPK14
NFATC1
NFATC3
FOS
JUND
ATF2
ELK1
TP53
p53 signaling pathway
Proliferation, differentiation, inflammation
Apoptosis
Cell cycle
ELK4
DDIT3
MAX
MEF2C
MAPKAPK5
MAPKAPK3
RPS6KA4
CDC25B
HSPB1
ATF4
NLK
Wnt signaling pathway
Serum, EGF, reactive oxygen species, or Srk tyrosinkinase downstream
MAP2K5
MAPK7
NR4A1
+p
-p
MAPKKKK
MAPKKK
MAPKK
MAPK
Transcription factor
04010 12/27/18
(c) Kanehisa Laboratories

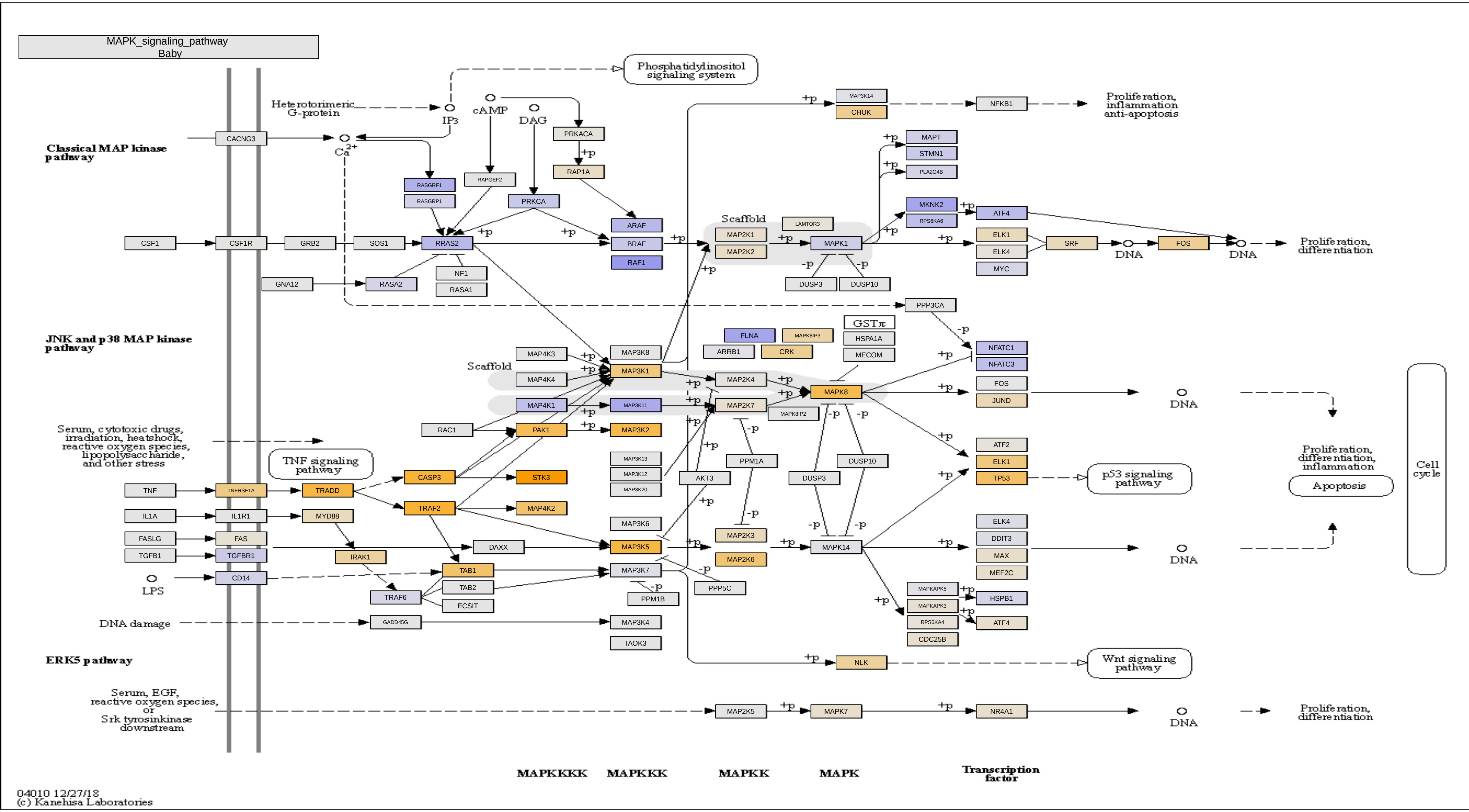

MAPK_signaling_pathway
Baby
Phosphatidylinositol signaling system
Heterotorimeric G-protein
IP3
cAMP
DAG
Ca2+
Classical MAP kinase pathway
CACNG3
PRKACA
RAP1A
RASGRF1
RASGRP1
RAPGEF2
PRKCA
CSF1
CSF1R
GRB2
SOS1
RRAS2
ARAF
BRAF
RAF1
GNA12
RASA2
NF1
RASA1
Scaffold
LAMTOR3
MAP2K1
MAP2K2
MAPK1
DUSP3
DUSP10
MAP3K14
CHUK
NFKB1
Proliferation, inflammation anti-apoptosis
MAPT
STMN1
PLA2G4B
MKNK2
RPS6KA6
ATF4
ELK1
ELK4
MYC
SRF
FOS
DNA
Proliferation, differentiation
PPP3CA
JNK and p38 MAP kinase pathway
MAP4K3
MAP4K4
MAP4K1
MAP3K8
MAP3K1
MAP3K11
FLNA
MAPK8IP3
ARRB1
CRK
GSTπ
HSPA1A
MECOM
MAP2K4
MAP2K7
MAPK8IP2
MAPK8
NFATC1
NFATC3
FOS
JUND
RAC1
PAK1
MAP3K2
Serum, cytotoxic drugs, irradiation, heatshock, reactive oxygen species, lipopolysaccharide, and other stress
TNF signaling pathway
CASP3
STK3
MAP3K13
MAP3K12
MAP3K20
AKT3
PPM1A
DUSP10
DUSP3
ATF2
ELK1
TP53
p53 signaling pathway
Proliferation, differentiation, inflammation
Apoptosis
Cell cycle
TNF
TNFRSF1A
TRADD
IL1A
IL1R1
MYD88
TRAF2
MAP4K2
MAP3K6
FASLG
FAS
TGFB1
TGFBR1
DAXX
MAP3K5
IRAK1
MAP2K3
MAP2K6
MAPK14
ELK4
DDIT3
MAX
MEF2C
LPS
CD14
TAB1
TAB2
TRAF6
ECSIT
MAP3K7
PPM1B
PPP5C
MAPKAPK5
MAPKAPK3
RPS6KA4
CDC25B
HSPB1
ATF4
DNA damage
GADD45G
MAP3K4
TAOK3
NLK
Wnt signaling pathway
ERK5 pathway
Serum, EGF, reactive oxygen species, or Srk tyrosinkinase downstream
MAP2K5
MAPK7
NR4A1
+p
-p
MAPKKKK
MAPKKK
MAPKK
MAPK
Transcription factor
04010 12/27/18
(c) Kanehisa Laboratories

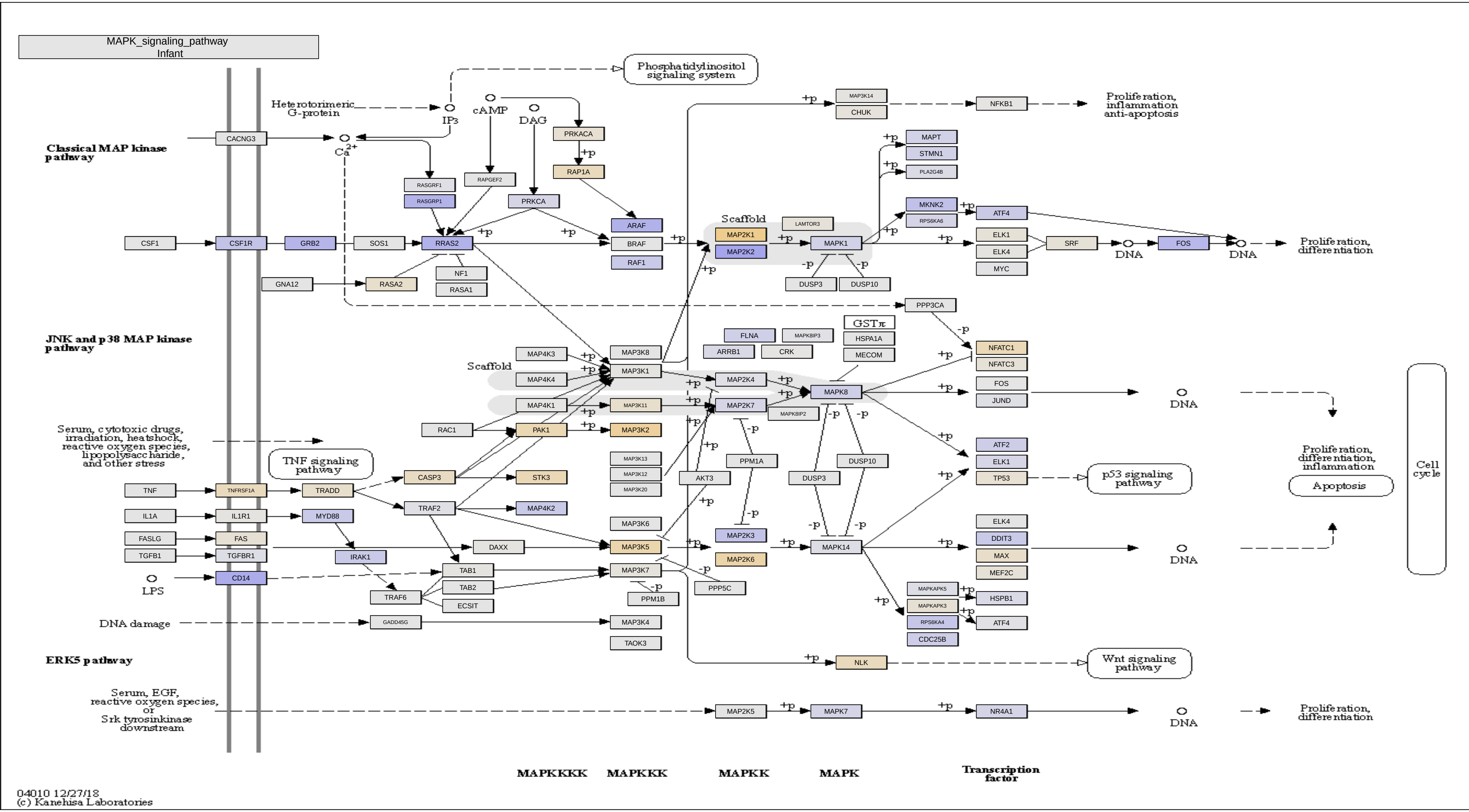

MAPK_signaling_pathway
Infant
Phosphatidylinositol signaling system
Heterotorimeric G-protein
IP3
cAMP
DAG
Ca2+
Classical MAP kinase pathway
CACNG3
PRKACA
RAP1A
RASGRF1
RASGRP1
RAPGEF2
PRKCA
CSF1
CSF1R
GRB2
SOS1
RRAS2
ARAF
BRAF
RAF1
NF1
RASA1
GNA12
RASA2
Scaffold
LAMTOR3
MAP2K1
MAP2K2
MAPK1
DUSP3
DUSP10
MAP3K14
CHUK
NFKB1
Proliferation, inflammation anti-apoptosis
MAPT
STMN1
PLA2G4B
MKNK2
RPS6KA6
ATF4
ELK1
ELK4
MYC
SRF
FOS
DNA
Proliferation, differentiation
PPP3CA
NFATC1
NFATC3
JNK and p38 MAP kinase pathway
MAP4K3
MAP4K4
MAP4K1
MAP3K8
MAP3K1
MAP3K11
MAP3K2
FLNA
MAPK8IP3
ARRB1
CRK
GSTπ
HSPA1A
MECOM
MAP2K4
MAP2K7
MAPK8IP2
MAPK8
FOS
JUND
RAC1
PAK1
Serum, cytotoxic drugs, irradiation, heatshock, reactive oxygen species, lipopolysaccharide, and other stress
TNF signaling pathway
CASP3
STK3
MAP3K13
MAP3K12
MAP3K20
PPM1A
AKT3
DUSP10
DUSP3
ATF2
ELK1
TP53
p53 signaling pathway
Proliferation, differentiation, inflammation
Apoptosis
Cell cycle
TNF
TNFRSF1A
TRADD
TRAF2
MAP4K2
IL1A
IL1R1
MYD88
FASLG
FAS
TGFB1
TGFBR1
IRAK1
DAXX
MAP3K6
MAP3K5
MAP2K3
MAP2K6
MAPK14
ELK4
DDIT3
MAX
MEF2C
LPS
CD14
TAB1
TAB2
TRAF6
ECSIT
MAP3K7
PPM1B
PPP5C
MAPKAPK5
MAPKAPK3
RPS6KA4
CDC25B
HSPB1
ATF4
DNA damage
GADD45G
MAP3K4
TAOK3
NLK
Wnt signaling pathway
ERK5 pathway
Serum, EGF, reactive oxygen species, or Srk tyrosinkinase downstream
MAP2K5
MAPK7
NR4A1
+p
-p
MAPKKKK
MAPKKK
MAPKK
MAPK
Transcription factor
04010 12/27/18
(c) Kanehisa Laboratories

MAPK_signaling_pathway
Teenager

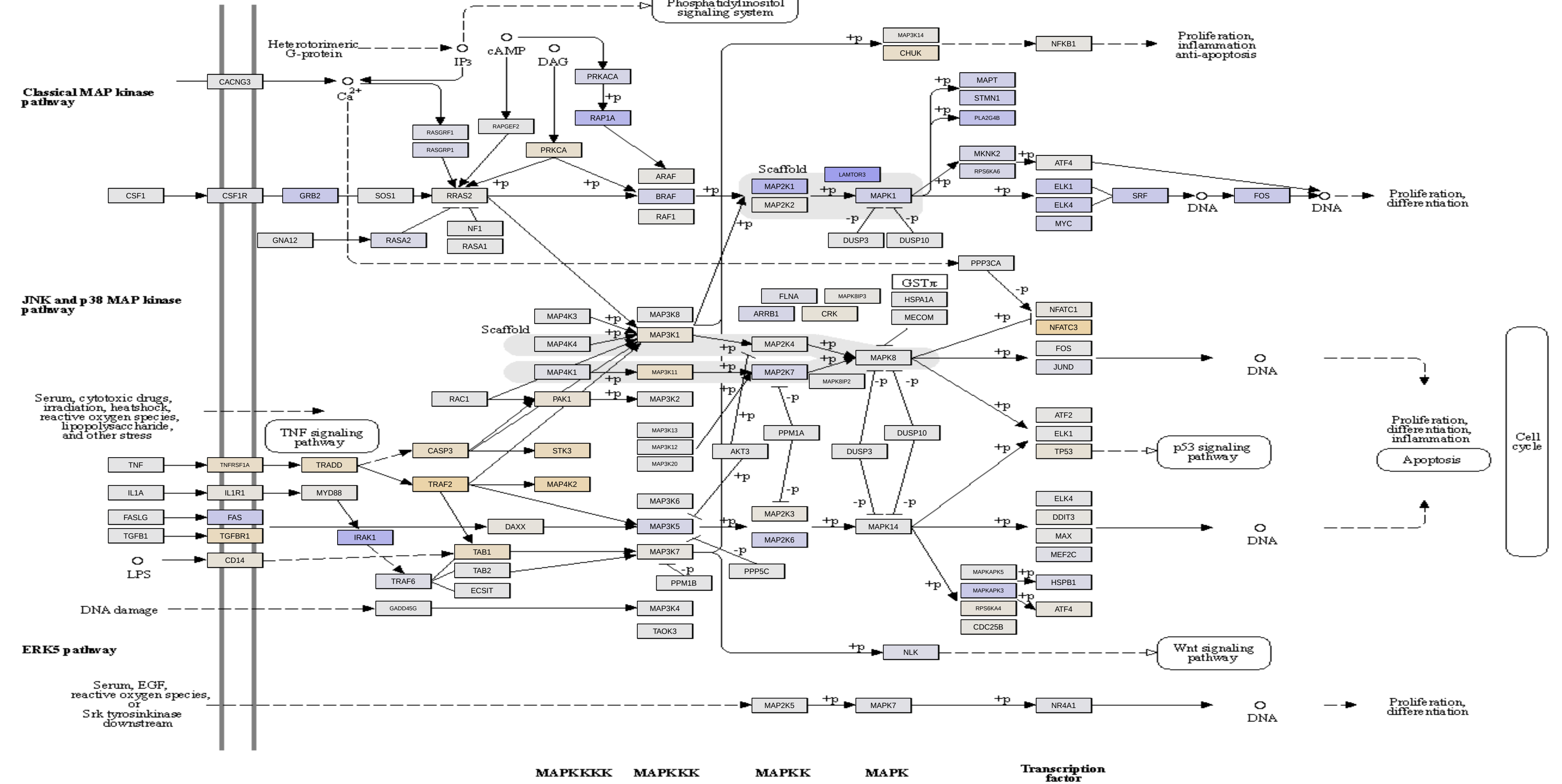

04010 12/27/18
(c) Kanehisa Laboratories

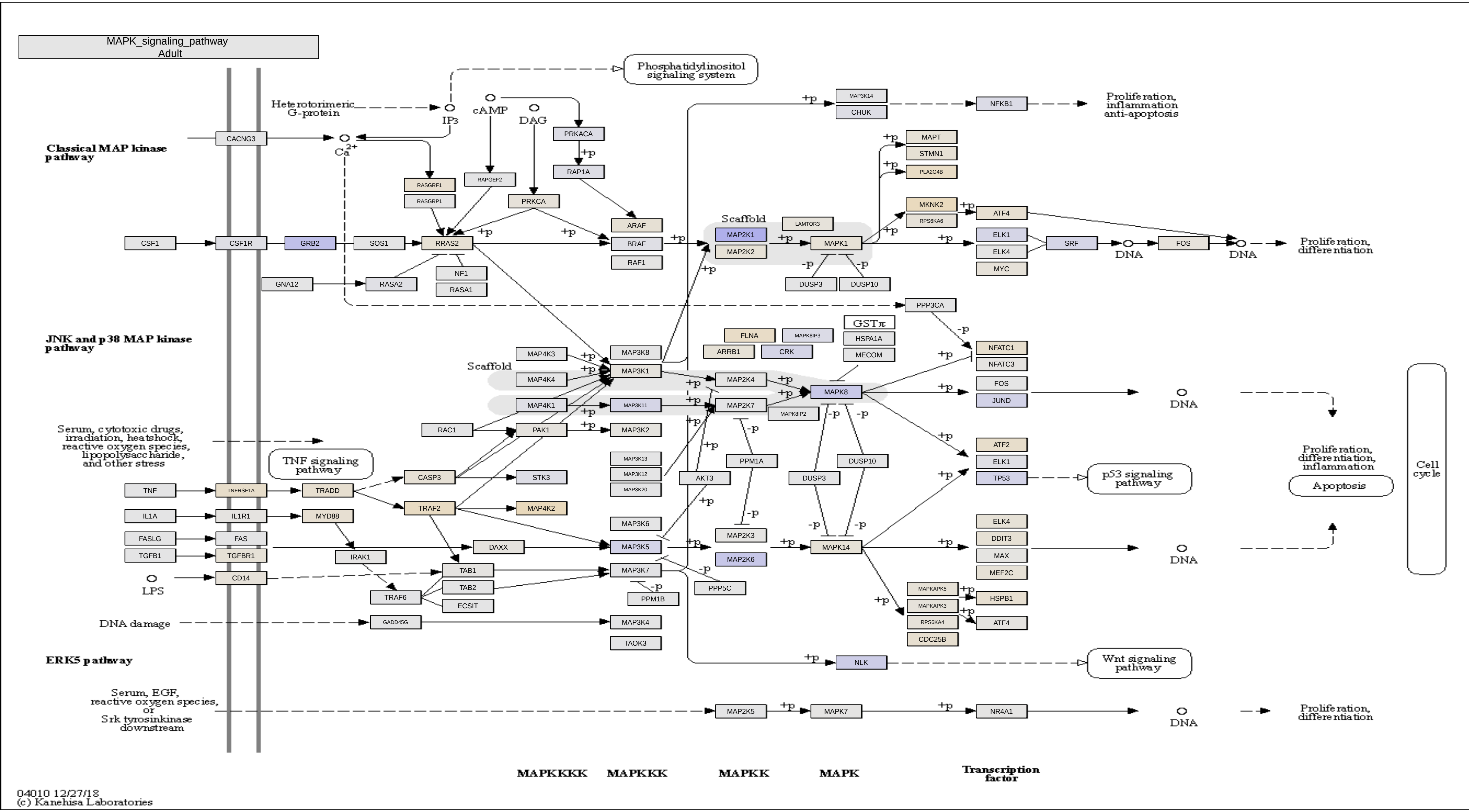
MAPK_signaling_pathway
Adult
Classical MAP kinase pathway
JNK and p38 MAP kinase pathway
ERK5 pathway
Heterotorimeric G-protein
IP3
cAMP
DAG
Ca2+
Phosphatidylinositol signaling system
CACNG3
PRKACA
RAP1A
RASGRF1
RASGRP1
RAPGEF2
PRKCA
ARAF
BRAF
RAF1
CSF1
CSF1R
GRB2
SOS1
RRAS2
NF1
RASA1
GNA12
RASA2
Scaffold
LAMTOR3
MAP2K1
MAP2K2
MAPK1
DUSP3
DUSP10
MAP3K14
CHUK
NFKB1
Proliferation, inflammation anti-apoptosis
MAPT
STMN1
PLA2G4B
MKNK2
RPS6KA6
ATF4
ELK1
ELK4
MYC
SRF
FOS
DNA
Proliferation, differentiation
PPP3CA
NFATC1
NFATC3
FOS
JUND
FLNA
MAPK8IP3
ARRB1
CRK
GSTπ
HSPA1A
MECOM
MAP4K3
MAP4K4
MAP4K1
MAP3K8
MAP3K1
MAP3K11
MAP2K4
MAP2K7
MAPK8IP2
MAPK8
RAC1
PAK1
MAP3K2
MAP3K13
MAP3K12
MAP3K20
AKT3
PPM1A
DUSP10
DUSP3
Serum, cytotoxic drugs, irradiation, heatshock, reactive oxygen species, lipopolysaccharide, and other stress
TNF signaling pathway
CASP3
STK3
TNF
TNFRSF1A
TRADD
TRAF2
MAP4K2
IL1A
IL1R1
MYD88
FASLG
FAS
TGFB1
TGFBR1
LPS
CD14
IRAK1
DAXX
MAP3K6
MAP3K5
MAP2K3
MAP2K6
MAPK14
TAB1
TAB2
TRAF6
ECSIT
MAP3K7
PPM1B
PPP5C
ATF2
ELK1
TP53
p53 signaling pathway
Proliferation, differentiation, inflammation
Apoptosis
Cell cycle
ELK4
DDIT3
MAX
MEF2C
MAPKAPK5
MAPKAPK3
RPS6KA4
CDC25B
HSPB1
ATF4
DNA damage
GADD45G
MAP3K4
TAOK3
NLK
Wnt signaling pathway
Serum, EGF, reactive oxygen species, or Srk tyrosinkinase downstream
MAP2K5
MAPK7
NR4A1
MAPKKKK
MAPKKK
MAPKK
MAPK
Transcription factor
+p
-p
04010 12/27/18
(c) Kanehisa Laboratories

MAPK_signaling_pathway
Late adult

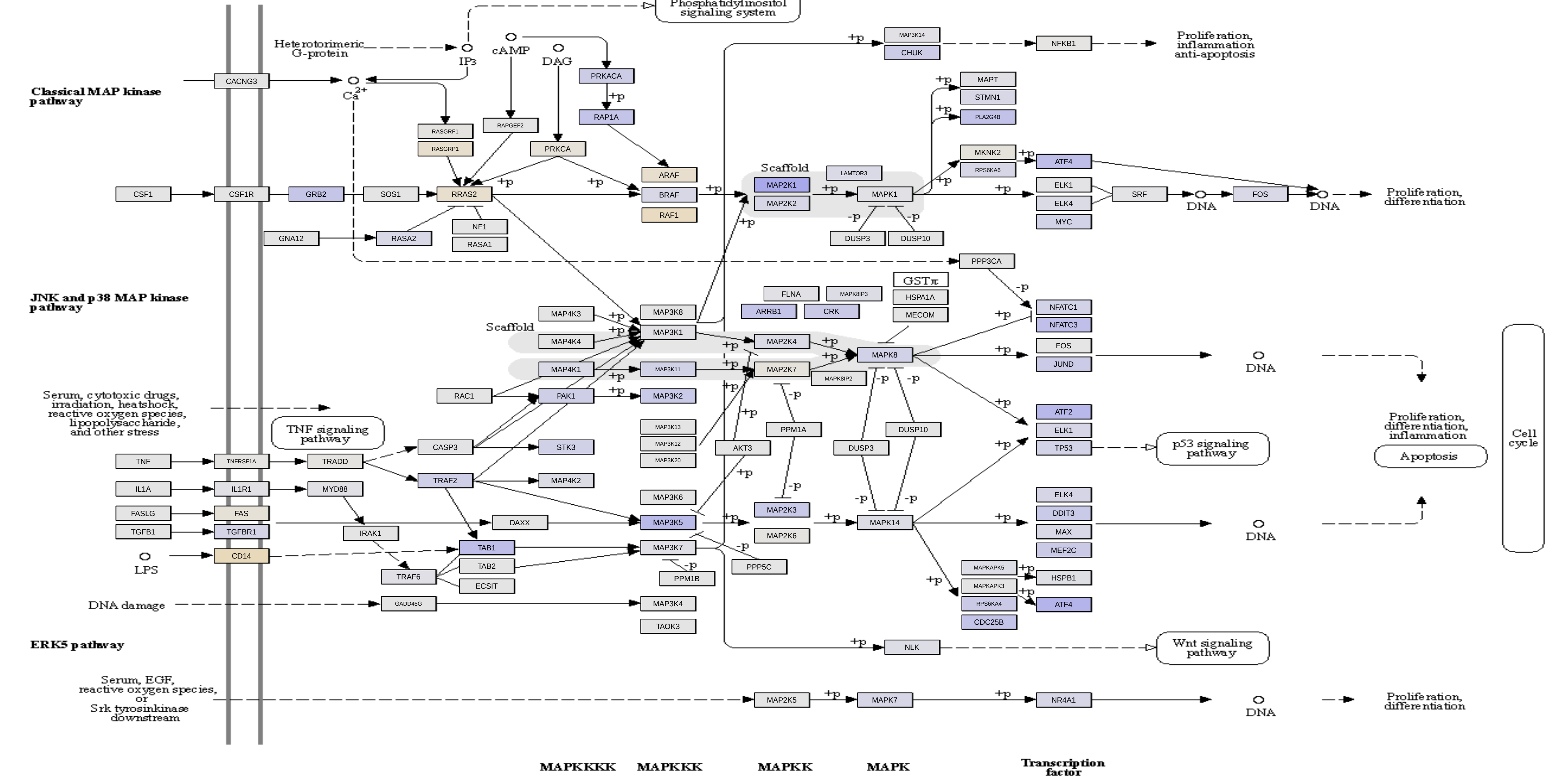

04010 12/27/18
(c) Kanehisa Laboratories
