# Supplementary material for: The Transcriptome and Methylome of the Developing and Aging Brain and Their Relations to Gliomas and Psychological Disorders
Source: Cells. 2022 Jan 21;11(3):362. doi: 10.3390/cells11030362 (PMC8834030; doi:10.3390/cells11030362)
Supplement: Supplementary file 1 [file cells-11-00362-s001.zip › Suppl-Material-S4-Pathways-PSF_Methylation/Neurotrophin_signaling_pathway.pdf]

Neurotrophin signaling pathway

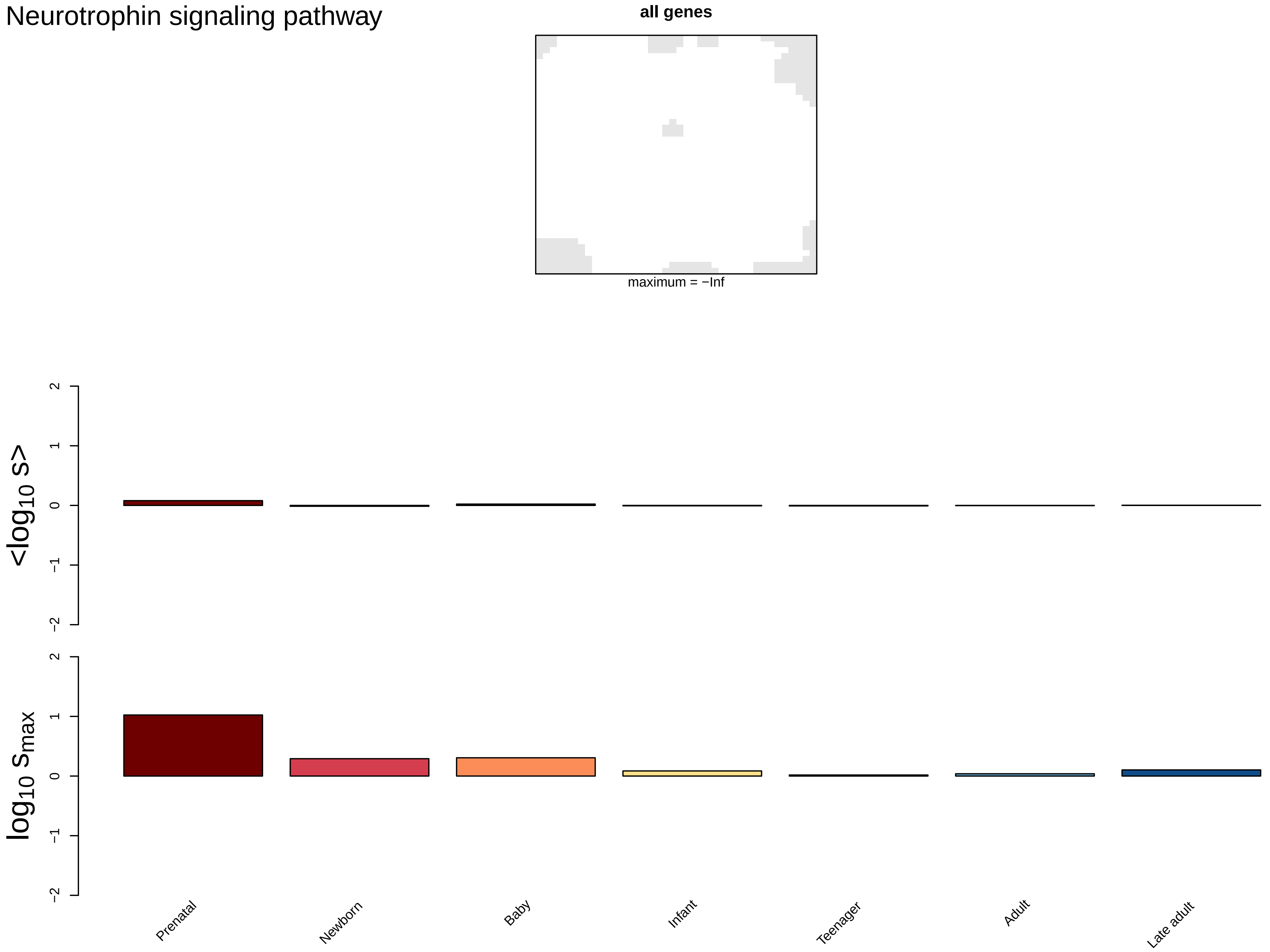

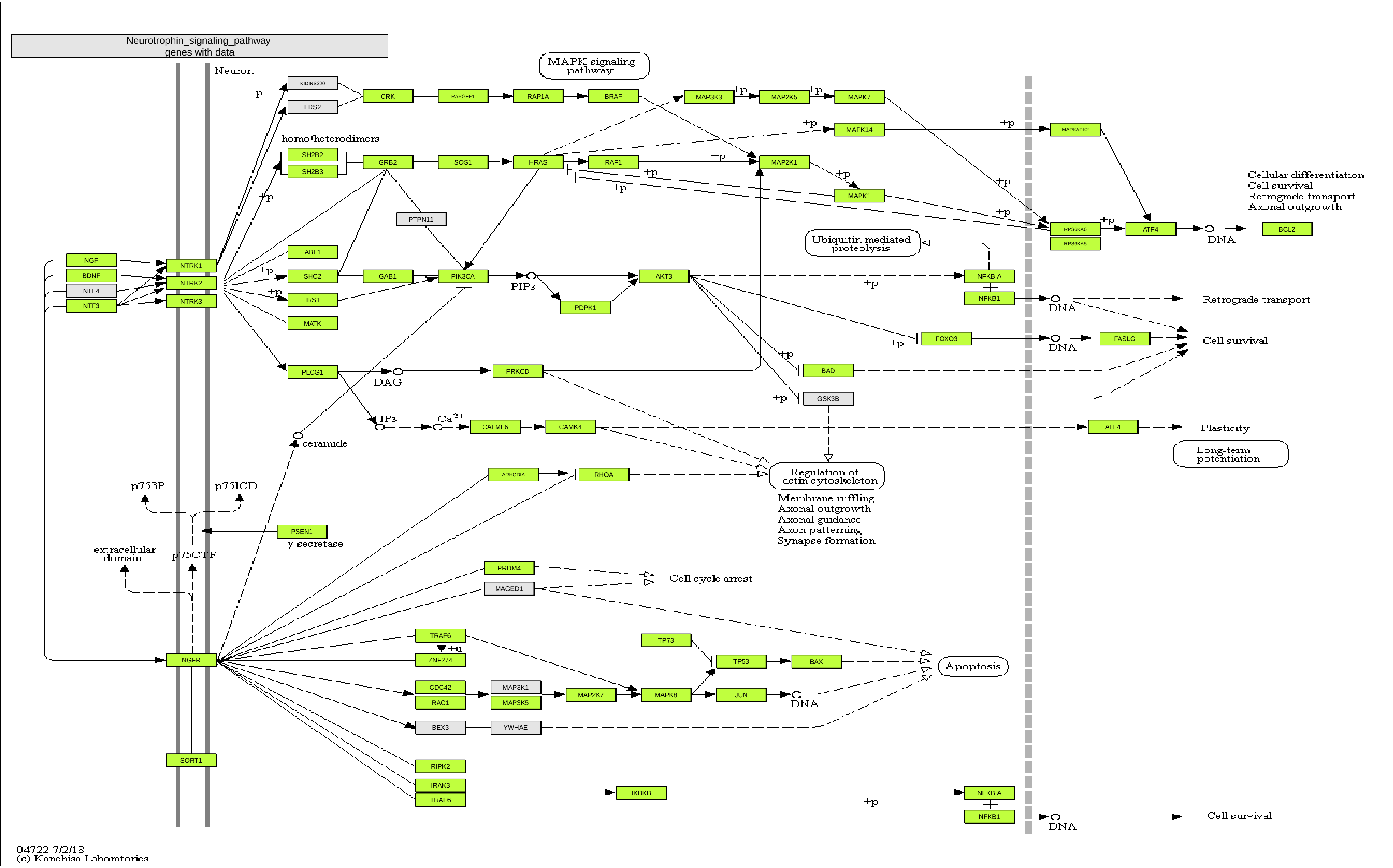
Neurotrophin_signaling_pathway
genes with data
Neuron
MAPK signaling pathway
KIDINS220
FRS2
CRK
RAPGEF1
RAP1A
BRAF
MAP3K3
MAP2K5
MAPK7
MAPK14
MAPKAPK2
homo/heterodimers
SH2B2
SH2B3
GRB2
SOS1
HRAS
RAF1
MAP2K1
MAPK1
PTPN11
RPS6KA6
RPS6KA5
ATF4
DNA
BCL2
Cellular differentiation
Cell survival
Retrograde transport
Axonal outgrowth
Ubiquitin mediated proteolysis
NGF
BDNF
NTF4
NTF3
NTRK1
NTRK2
NTRK3
ABL1
SHC2
GAB1
PIK3CA
PIP3
AKT3
NFKBIA
NFKB1
IRS1
PDPK1
MATK
FOXO3
FASLG
Retrograde transport
Cell survival
BAD
GSK3B
PLCG1
DAG
PRKCD
IP3
Ca2+
CALML6
CAMK4
ATF4
Plasticity
Long-term potentiation
ceramide
ARHGDIA
RHOA
Regulation of actin cytoskeleton
Membrane ruffling
Axonal outgrowth
Axonal guidance
Axon patterning
Synapse formation
p75βP
p75ICD
PSEN1
γ-secretase
extracellular domain
p75CTF
PRDM4
MAGED1
Cell cycle arrest
TRAF6
ZNF274
+u
TP73
TP53
BAX
Apoptosis
NGFR
CDC42
RAC1
MAP3K1
MAP3K5
MAP2K7
MAPK8
JUN
BEX3
YWHAE
SORT1
RIPK2
IRAK3
TRAF6
IKBKB
NFKBIA
NFKB1
Cell survival
+p
04722 7/2/18
(c) Kanehisa Laboratories

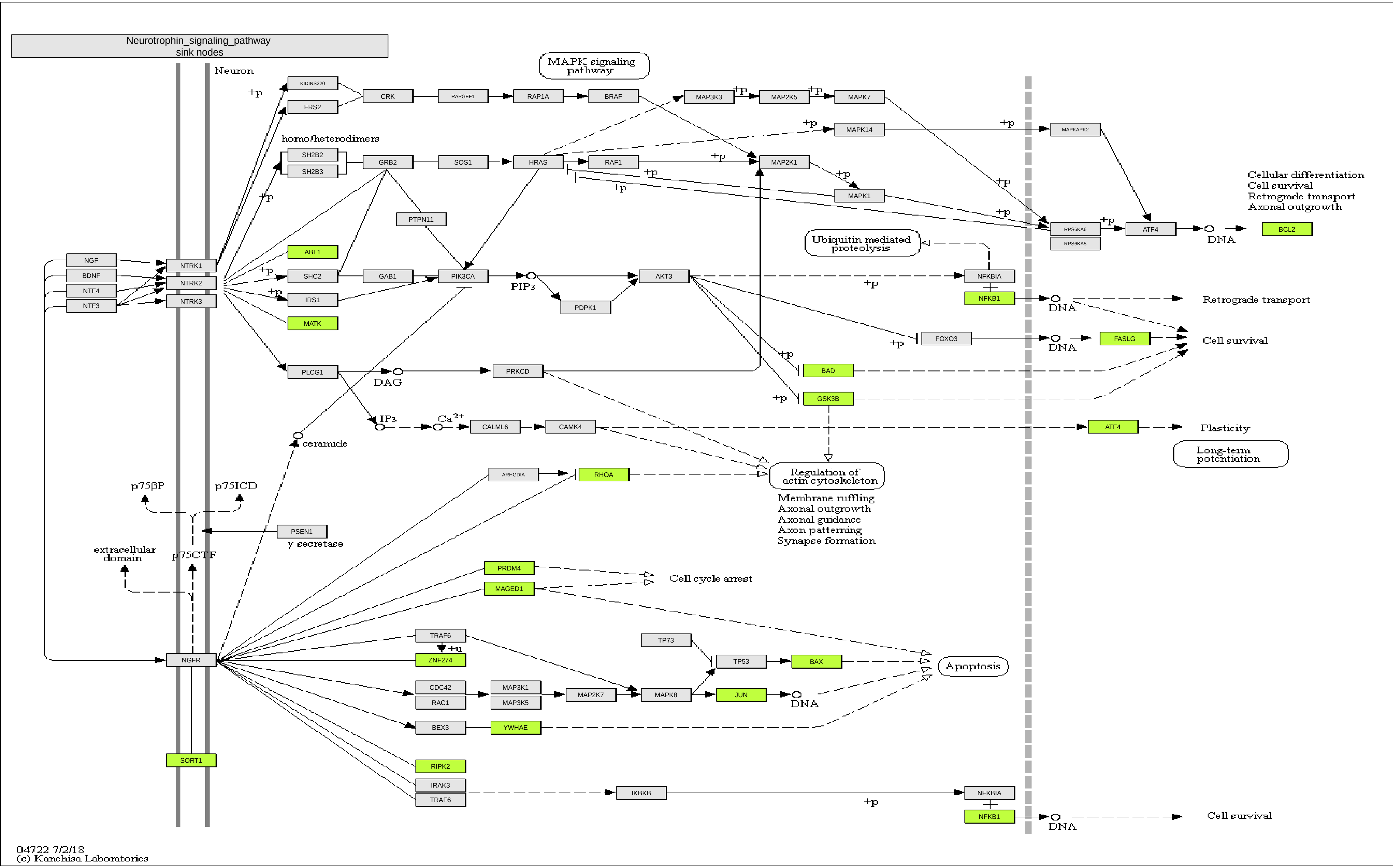
Neurotrophin_signaling_pathway
sink nodes
Neuron
MAPK signaling pathway
KIDINS220
FRS2
CRK
RAPGEF1
RAP1A
BRAF
MAP3K3
MAP2K5
MAPK7
MAPK14
MAPKAPK2
homo/heterodimers
SH2B2
SH2B3
GRB2
SOS1
HRAS
RAF1
MAP2K1
MAPK1
PTPN11
RPS6KA6
RPS6KA5
ATF4
DNA
BCL2
Cellular differentiation
Cell survival
Retrograde transport
Axonal outgrowth
Ubiquitin mediated proteolysis
NGF
BDNF
NTF4
NTF3
NTRK1
NTRK2
NTRK3
ABL1
SHC2
GAB1
PIK3CA
PIP3
PDPK1
AKT3
NFKBIA
NFKB1
IRS1
MATK
FOXO3
FASLG
Retrograde transport
Cell survival
PLCG1
DAG
PRKCD
BAD
GSK3B
IP3
Ca2+
CALML6
CAMK4
ATF4
Plasticity
Long-term potentiation
ceramide
Regulation of actin cytoskeleton
Membrane ruffling
Axonal outgrowth
Axonal guidance
Axon patterning
Synapse formation
ARHGDIA
RHOA
p75βP
p75ICD
PSEN1
γ-secretase
extracellular domain
p75CTF
PRDM4
MAGED1
Cell cycle arrest
TRAF6
ZNF274
+u
TP73
TP53
BAX
Apoptosis
CDC42
RAC1
MAP3K1
MAP3K5
MAP2K7
MAPK8
JUN
NGFR
BEX3
YWHAE
SORT1
RIPK2
IRAK3
TRAF6
IKBKB
NFKBIA
NFKB1
Cell survival
+p
04722 7/2/18
(c) Kanehisa Laboratories

Neurotrophin_signaling_pathway
Prenatal

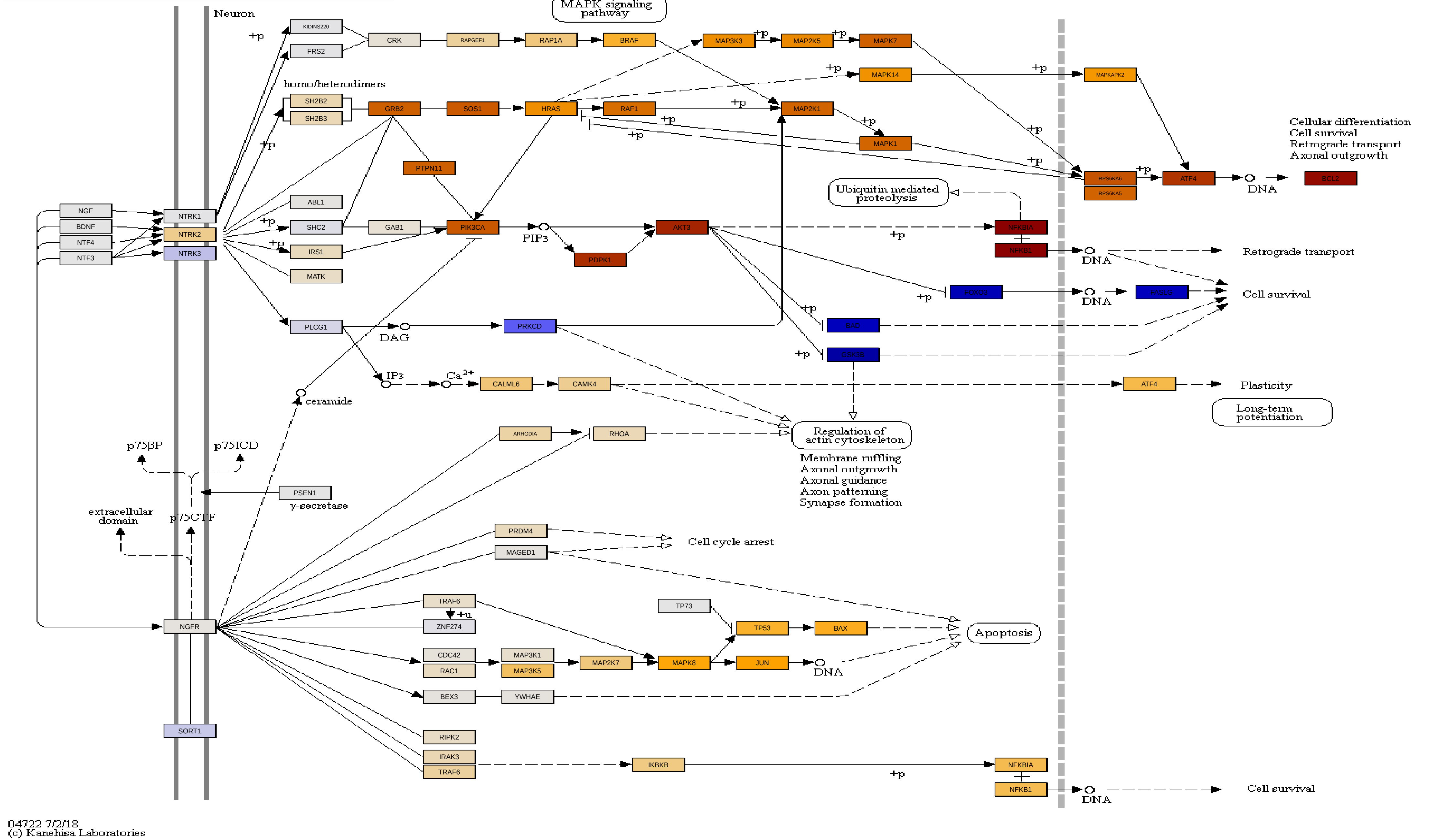

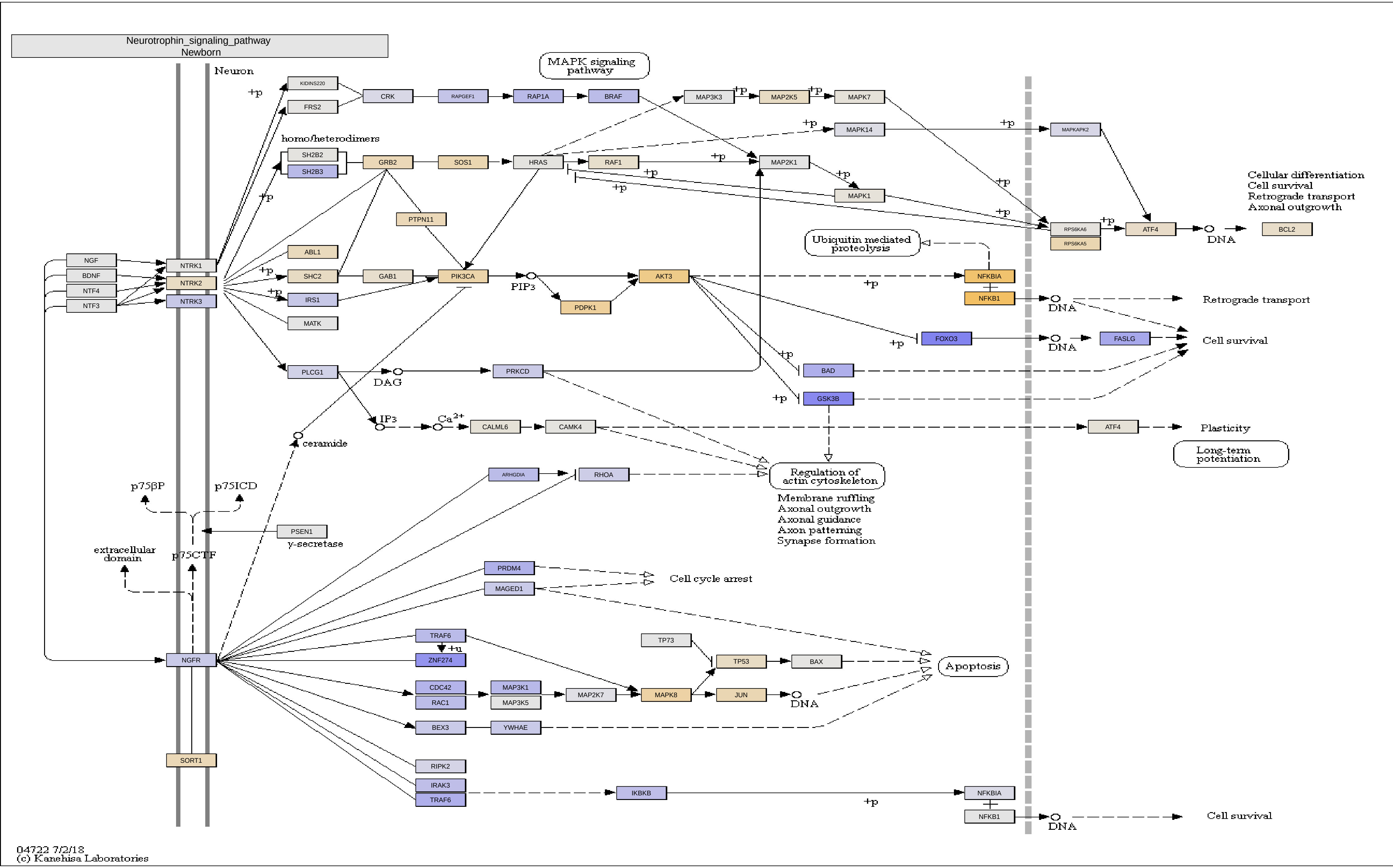
Neurotrophin_signaling_pathway
Newborn
Neuron
MAPK signaling pathway
KIDINS220
FRS2
CRK
RAPGEF1
RAP1A
BRAF
MAP3K3
MAP2K5
MAPK7
MAPK14
MAPKAPK2
homo/heterodimers
SH2B2
SH2B3
GRB2
SOS1
HRAS
RAF1
MAP2K1
MAPK1
RPS6KA6
RPS6KA5
ATF4
DNA
BCL2
Cellular differentiation
Cell survival
Retrograde transport
Axonal outgrowth
PTPN11
ABL1
SHC2
GAB1
PIK3CA
PIP3
PDPK1
AKT3
Ubiquitin mediated proteolysis
NFKBIA
NFKB1
Retrograde transport
FOXO3
FASLG
Cell survival
BAD
GSK3B
NGF
BDNF
NTF4
NTF3
NTRK1
NTRK2
NTRK3
IRS1
MATK
PLCG1
DAG
PRKCD
IP3
Ca2+
CALML6
CAMK4
Plasticity
Long-term potentiation
ceramide
ARHGDIA
RHOA
Regulation of actin cytoskeleton
Membrane ruffling
Axonal outgrowth
Axonal guidance
Axon patterning
Synapse formation
p75βP
p75ICD
PSEN1
γ-secretase
extracellular domain
p75CTF
PRDM4
MAGED1
Cell cycle arrest
TRAF6
ZNF274
+u
TP73
TP53
BAX
Apoptosis
NGFR
CDC42
RAC1
MAP3K1
MAP3K5
MAP2K7
MAPK8
JUN
BEX3
YWHAE
SORT1
RIPK2
IRAK3
IKBKB
+p
Cell survival
04722 7/2/18
(c) Kanehisa Laboratories

Neurotrophin_signaling_pathway
Baby

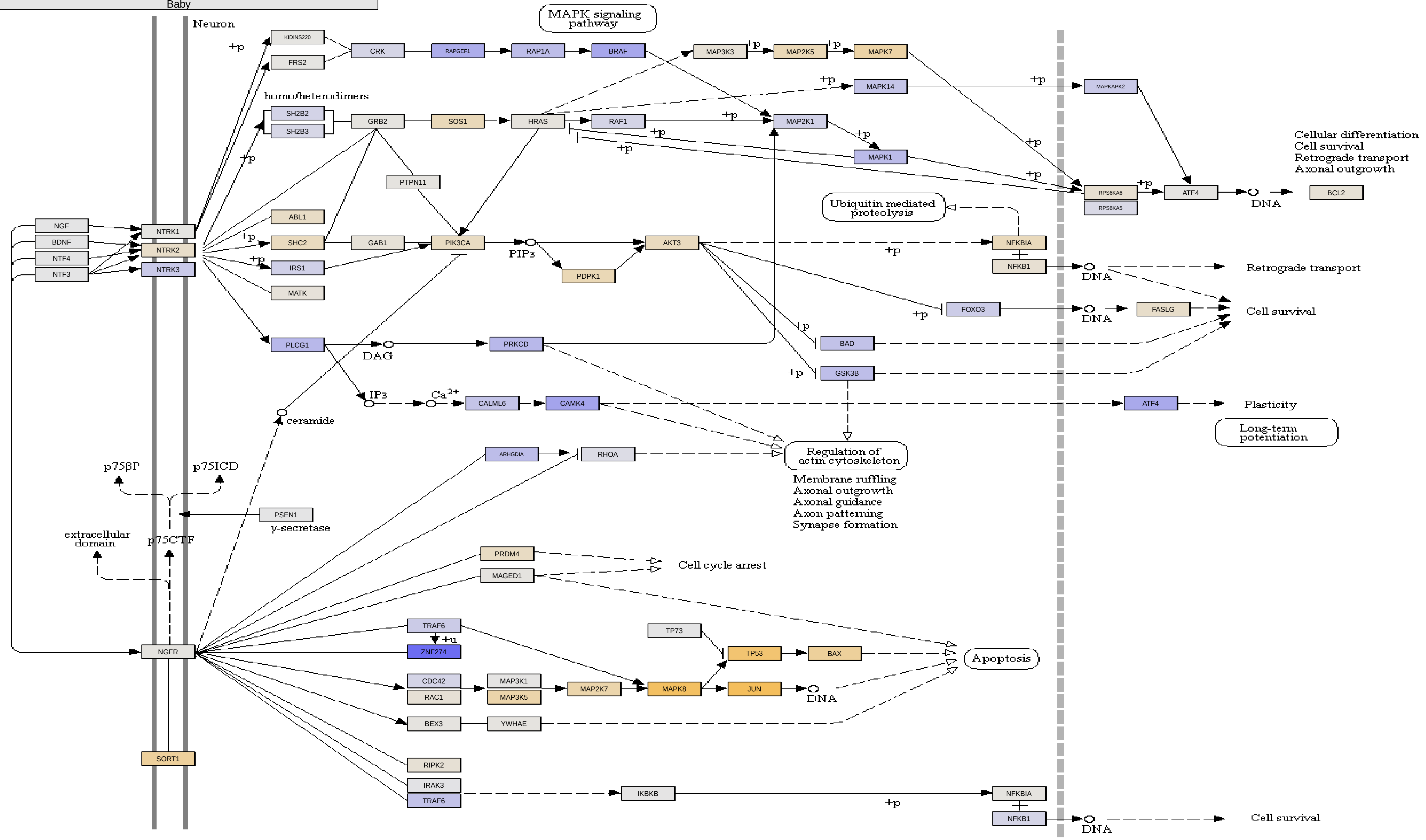

04722 7/2/18
(c) Kanehisa Laboratories

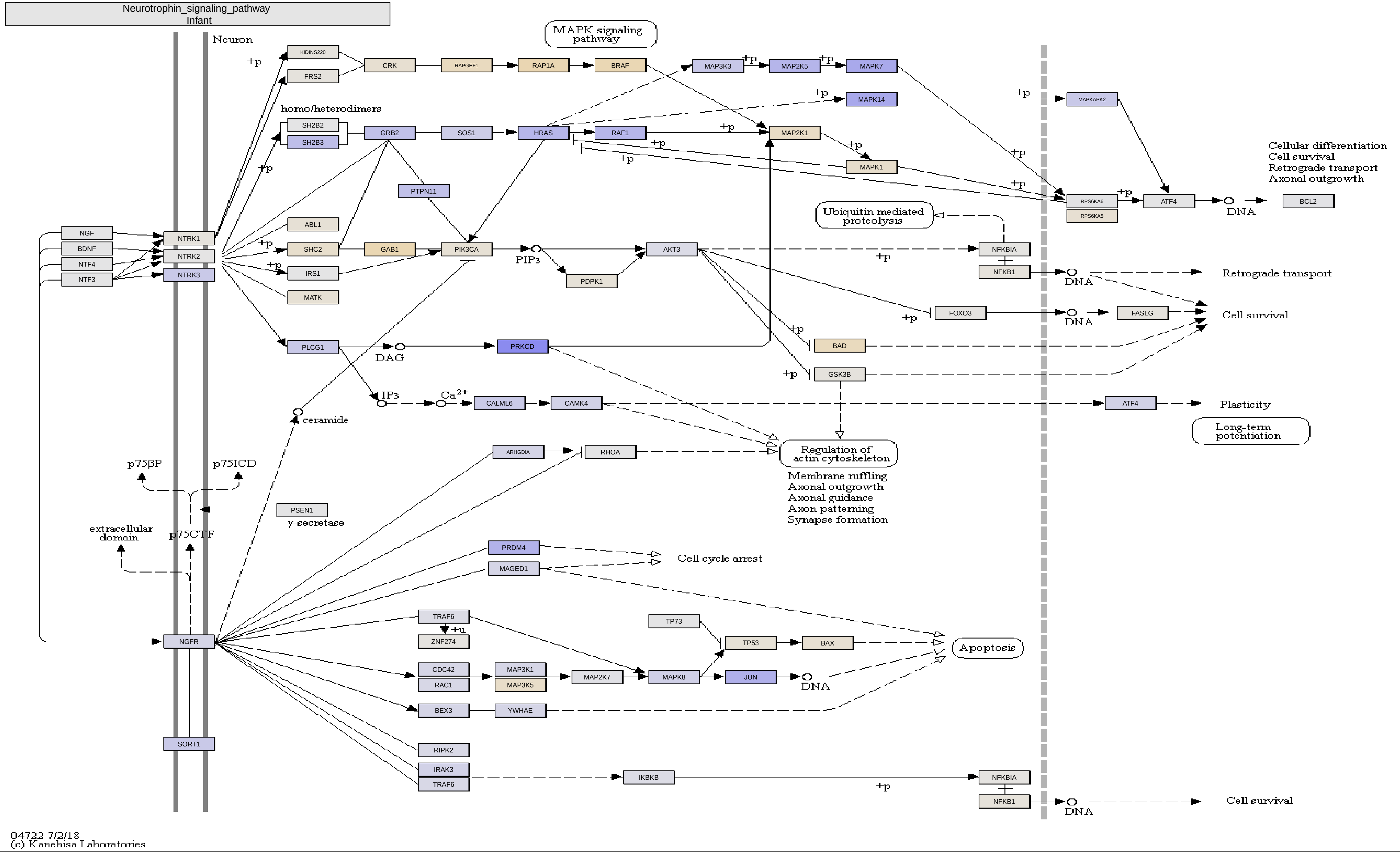

Neurotrophin_signaling_pathway
Infant
Neuron
MAPK signaling pathway
homo/heterodimers
NGF
BDNF
NTF4
NTF3
NTRK1
NTRK2
NTRK3
KIDINS220
FRS2
CRK
RAPGEF1
RAP1A
BRAF
MAP3K3
MAP2K5
MAPK7
MAPK14
MAPKAPK2
SH2B2
SH2B3
GRB2
SOS1
HRAS
RAF1
MAP2K1
MAPK1
PTPN11
ABL1
SHC2
GAB1
PIK3CA
PIP3
PDPK1
AKT3
IRS1
MATK
PLCG1
DAG
PRKCD
IP3
Ca2+
CALML6
CAMK4
ceramide
RPS6KA6
RPS6KA5
ATF4
DNA
BCL2
Cellular differentiation
Cell survival
Retrograde transport
Axonal outgrowth
Ubiquitin mediated proteolysis
NFKBIA
NFKB1
Retrograde transport
FOXO3
FASLG
Cell survival
BAD
GSK3B
Plasticity
Long-term potentiation
Regulation of actin cytoskeleton
Membrane ruffling
Axonal outgrowth
Axonal guidance
Axon patterning
Synapse formation
ARHGDIA
RHOA
p75βP
p75ICD
PSEN1
γ-secretase
extracellular domain
p75CTF
PRDM4
MAGED1
Cell cycle arrest
NGFR
TRAF6
ZNF274
+u
TP73
TP53
BAX
Apoptosis
CDC42
RAC1
MAP3K1
MAP3K5
MAP2K7
MAPK8
JUN
BEX3
YWHAE
SORT1
RIPK2
IRAK3
TRAF6
IKBKB
NFKBIA
NFKB1
Cell survival
+p
04722 7/2/18
(c) Kanehisa Laboratories

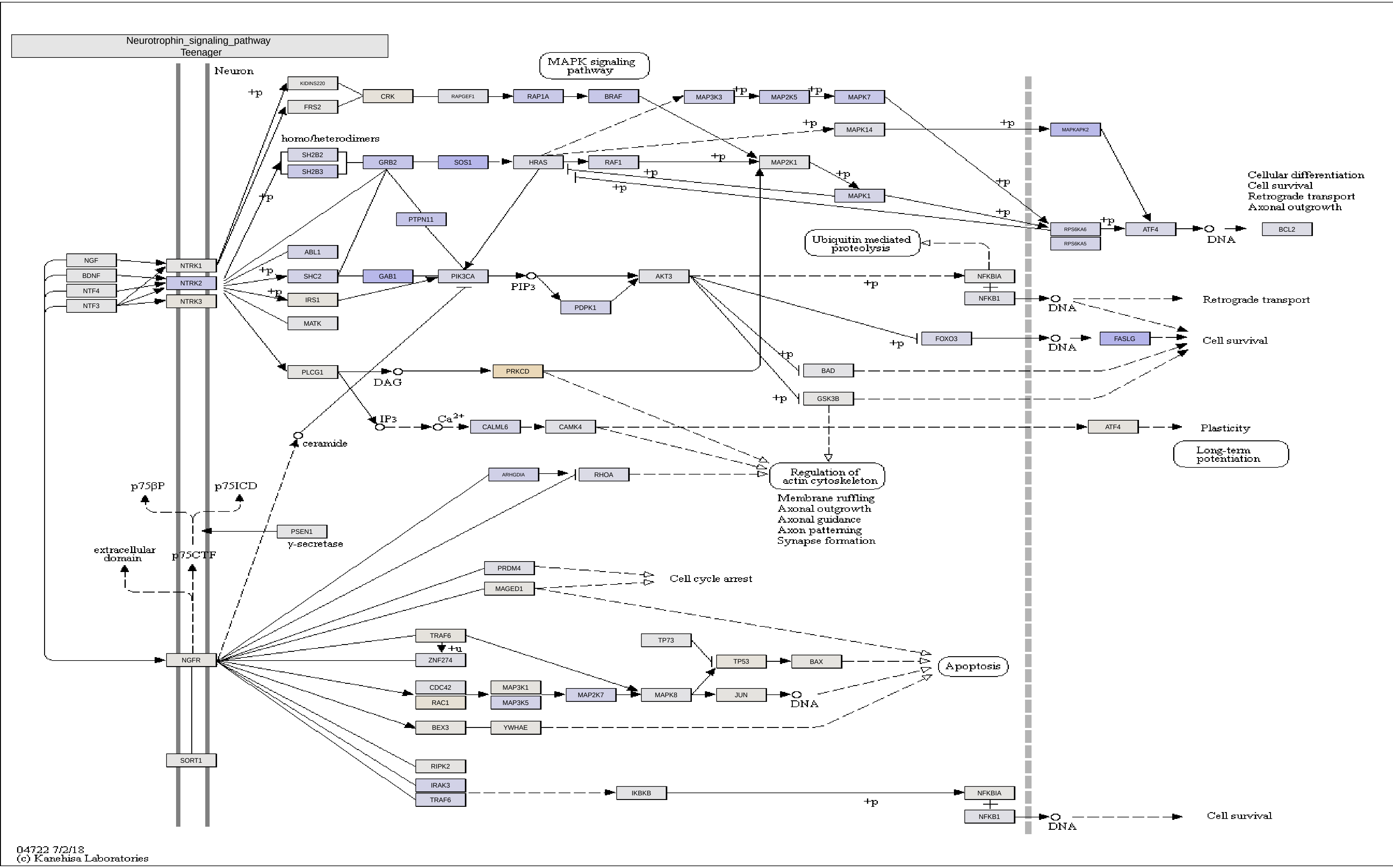
Neurotrophin_signaling_pathway
Teenager
Neuron
MAPK signaling pathway
KIDINS220
FRS2
CRK
RAPGEF1
RAP1A
BRAF
MAP3K3
MAP2K5
MAPK7
MAPK14
MAPKAPK2
homo/heterodimers
SH2B2
SH2B3
GRB2
SOS1
HRAS
RAF1
MAP2K1
MAPK1
PTPN11
RPS6KA6
RPS6KA5
ATF4
DNA
BCL2
Cellular differentiation
Cell survival
Retrograde transport
Axonal outgrowth
Ubiquitin mediated proteolysis
NGF
BDNF
NTF4
NTF3
NTRK1
NTRK2
NTRK3
ABL1
SHC2
GAB1
PIK3CA
PIP3
AKT3
NFKBIA
NFKB1
PDPK1
IRS1
MATK
FOXO3
FASLG
Retrograde transport
Cell survival
PLCG1
DAG
PRKCD
BAD
GSK3B
IP3
Ca2+
CALML6
CAMK4
ATF4
Plasticity
Long-term potentiation
ceramide
ARHGDIA
RHOA
Regulation of actin cytoskeleton
Membrane ruffling
Axonal outgrowth
Axonal guidance
Axon patterning
Synapse formation
p75βP
p75ICD
PSEN1
γ-secretase
extracellular domain
p75CTF
PRDM4
MAGED1
Cell cycle arrest
TRAF6
+u
ZNF274
TP73
TP53
BAX
Apoptosis
NGFR
CDC42
RAC1
MAP3K1
MAP3K5
MAP2K7
MAPK8
JUN
BEX3
YWHAE
SORT1
RIPK2
IRAK3
TRAF6
IKBKB
NFKBIA
NFKB1
Cell survival
+p
04722 7/2/18
(c) Kanehisa Laboratories

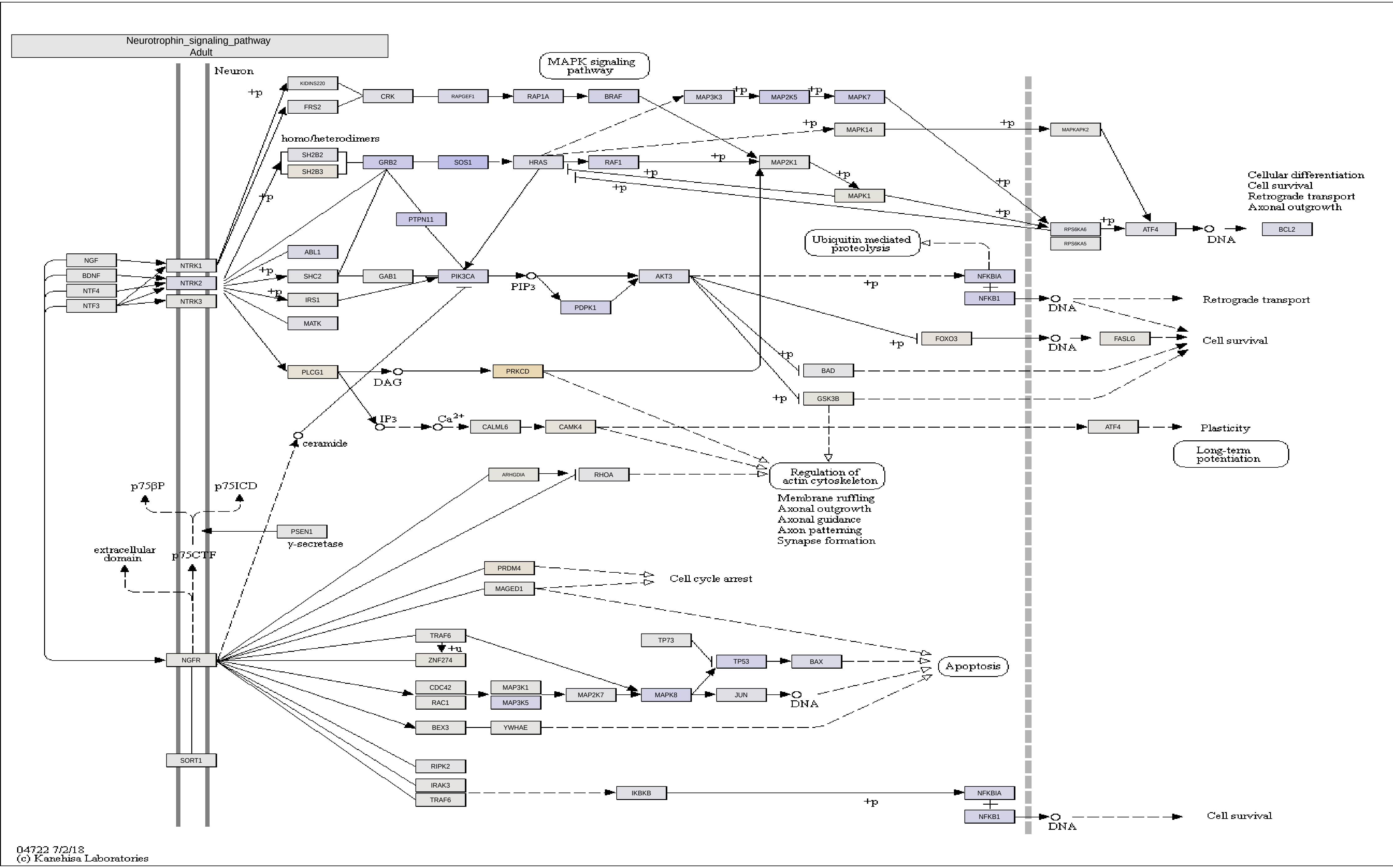
Neurotrophin_signaling_pathway
Adult
Neuron
MAPK signaling pathway
KIDINS220
FRS2
CRK
RAPGEF1
RAP1A
BRAF
MAP3K3
MAP2K5
MAPK7
MAPK14
MAPKAPK2
homo/heterodimers
SH2B2
SH2B3
GRB2
SOS1
HRAS
RAF1
MAP2K1
MAPK1
PTPN11
RPS6KA6
RPS6KA5
ATF4
DNA
BCL2
Cellular differentiation
Cell survival
Retrograde transport
Axonal outgrowth
Ubiquitin mediated proteolysis
NGF
BDNF
NTF4
NTF3
NTRK1
NTRK2
NTRK3
ABL1
SHC2
GAB1
PIK3CA
AKT3
NFKBIA
NFKB1
IRS1
MATK
PIP3
PDPK1
Retrograde transport
Cell survival
FOXO3
FASLG
BAD
GSK3B
PLCG1
DAG
PRKCD
IP3
Ca2+
CALML6
CAMK4
Plasticity
Long-term potentiation
ceramide
ARHGDIA
RHOA
Regulation of actin cytoskeleton
Membrane ruffling
Axonal outgrowth
Axonal guidance
Axon patterning
Synapse formation
p75βP
p75ICD
PSEN1
γ-secretase
extracellular domain
p75CTF
PRDM4
MAGED1
Cell cycle arrest
TRAF6
ZNF274
TP73
TP53
BAX
Apoptosis
NGFR
CDC42
RAC1
MAP3K1
MAP3K5
MAP2K7
MAPK8
JUN
BEX3
YWHAE
SORT1
RIPK2
IRAK3
TRAF6
IKBKB
NFKBIA
NFKB1
Cell survival
+p
+u
04722 7/2/18
(c) Kanehisa Laboratories

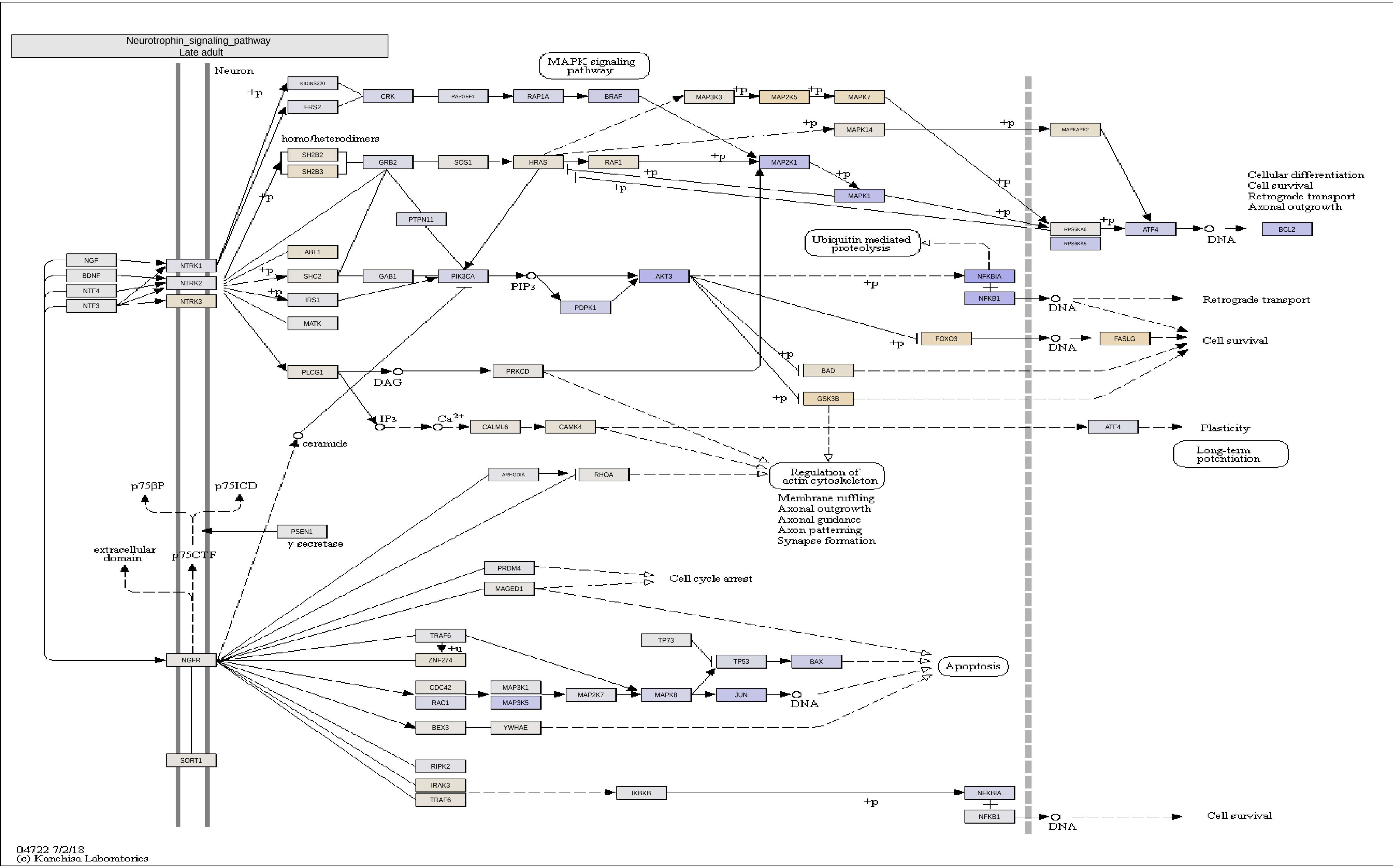

Neurotrophin_signaling_pathway
Late adult
Neuron
MAPK signaling pathway
KIDINS220
FRS2
CRK
RAPGEF1
RAP1A
BRAF
MAP3K3
MAP2K5
MAPK7
MAPK14
MAPKAPK2
homo/heterodimers
SH2B2
SH2B3
GRB2
SOS1
HRAS
RAF1
MAP2K1
MAPK1
RPS6KA6
RPS6KA5
ATF4
DNA
BCL2
Cellular differentiation
Cell survival
Retrograde transport
Axonal outgrowth
PTPN11
ABL1
Ubiquitin mediated proteolysis
NGF
BDNF
NTF4
NTF3
NTRK1
NTRK2
NTRK3
SHC2
GAB1
PIK3CA
PIP3
AKT3
NFKBIA
NFKB1
PDPK1
IRS1
MATK
Retrograde transport
Cell survival
FOXO3
FASLG
PLCG1
DAG
PRKCD
BAD
GSK3B
IP3
Ca2+
CALML6
CAMK4
ATF4
Plasticity
Long-term potentiation
ceramide
ARHGDIA
RHOA
Regulation of actin cytoskeleton
Membrane ruffling
Axonal outgrowth
Axonal guidance
Axon patterning
Synapse formation
p75βP
p75ICD
PSEN1
γ-secretase
extracellular domain
p75CTF
PRDM4
MAGED1
Cell cycle arrest
TRAF6
+u
ZNF274
TP73
TP53
BAX
Apoptosis
NGFR
CDC42
RAC1
MAP3K1
MAP3K5
MAP2K7
MAPK8
JUN
BEX3
YWHAE
SORT1
RIPK2
IRAK3
TRAF6
IKBKB
NFKBIA
NFKB1
Cell survival
+p
04722 7/2/18
(c) Kanehisa Laboratories
