# Supplementary material for: The Transcriptome and Methylome of the Developing and Aging Brain and Their Relations to Gliomas and Psychological Disorders
Source: Cells. 2022 Jan 21;11(3):362. doi: 10.3390/cells11030362 (PMC8834030; doi:10.3390/cells11030362)
Supplement: Supplementary file 1 [file cells-11-00362-s001.zip › Suppl-Material-S4-Pathways-PSF_Methylation/NF_kappa_B_signaling_pathway.pdf]

# NF−kappa B signaling pathway

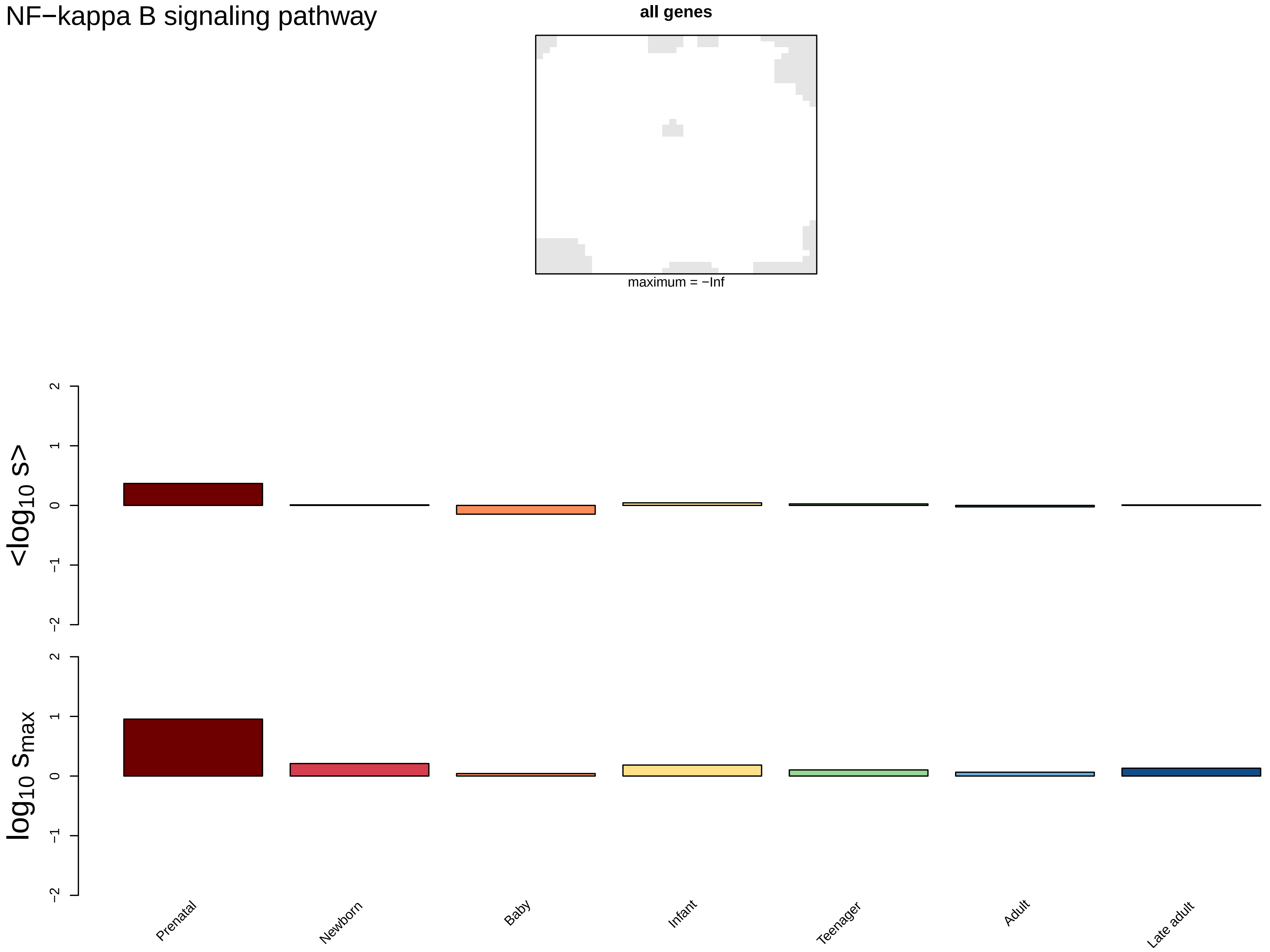

NF_kappa_B_signaling_pathway
genes with data

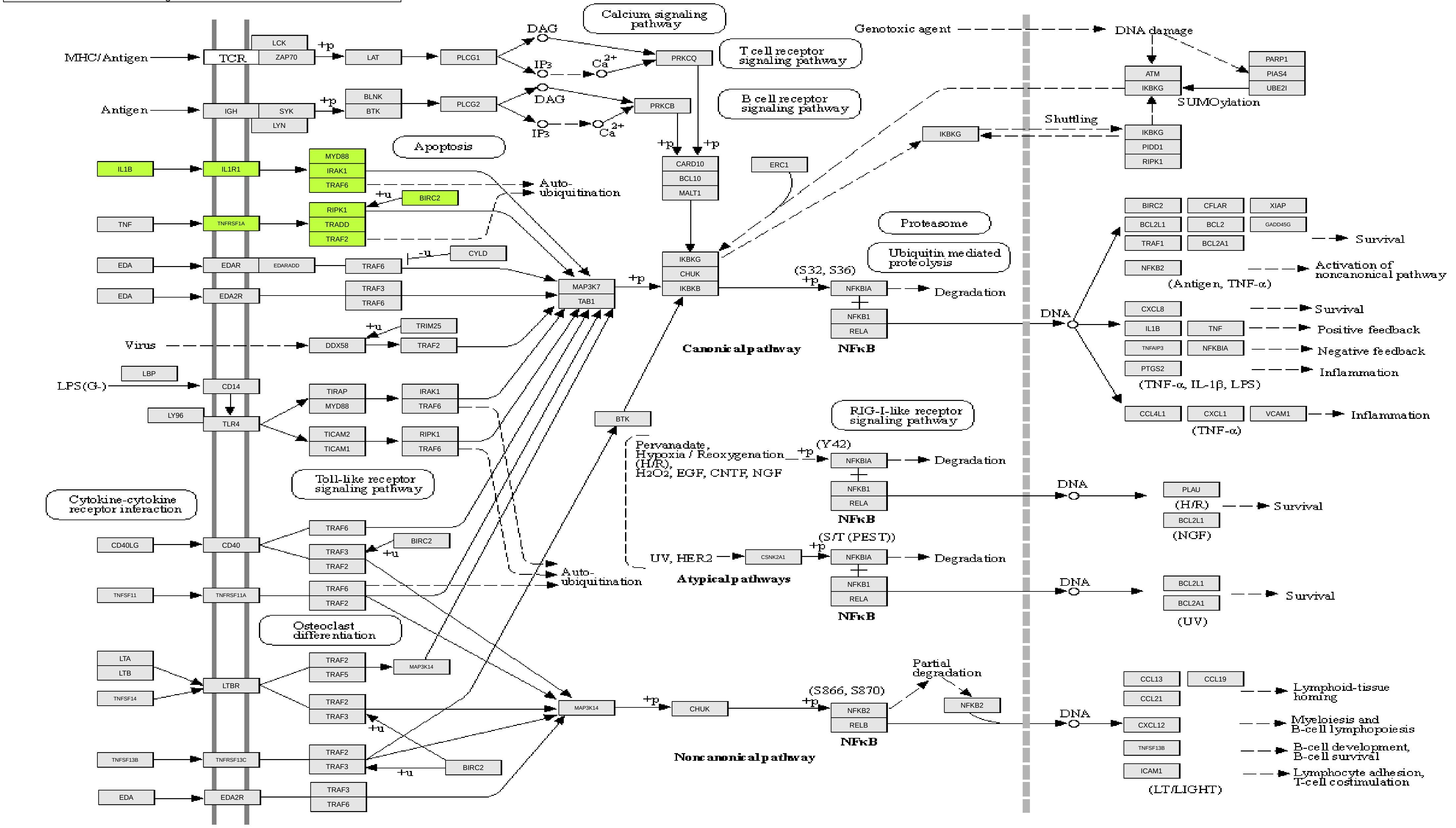


04064 4/12/19
(c) Kanehisa Laboratories

NF_kappa_B_signaling_pathway
sink nodes

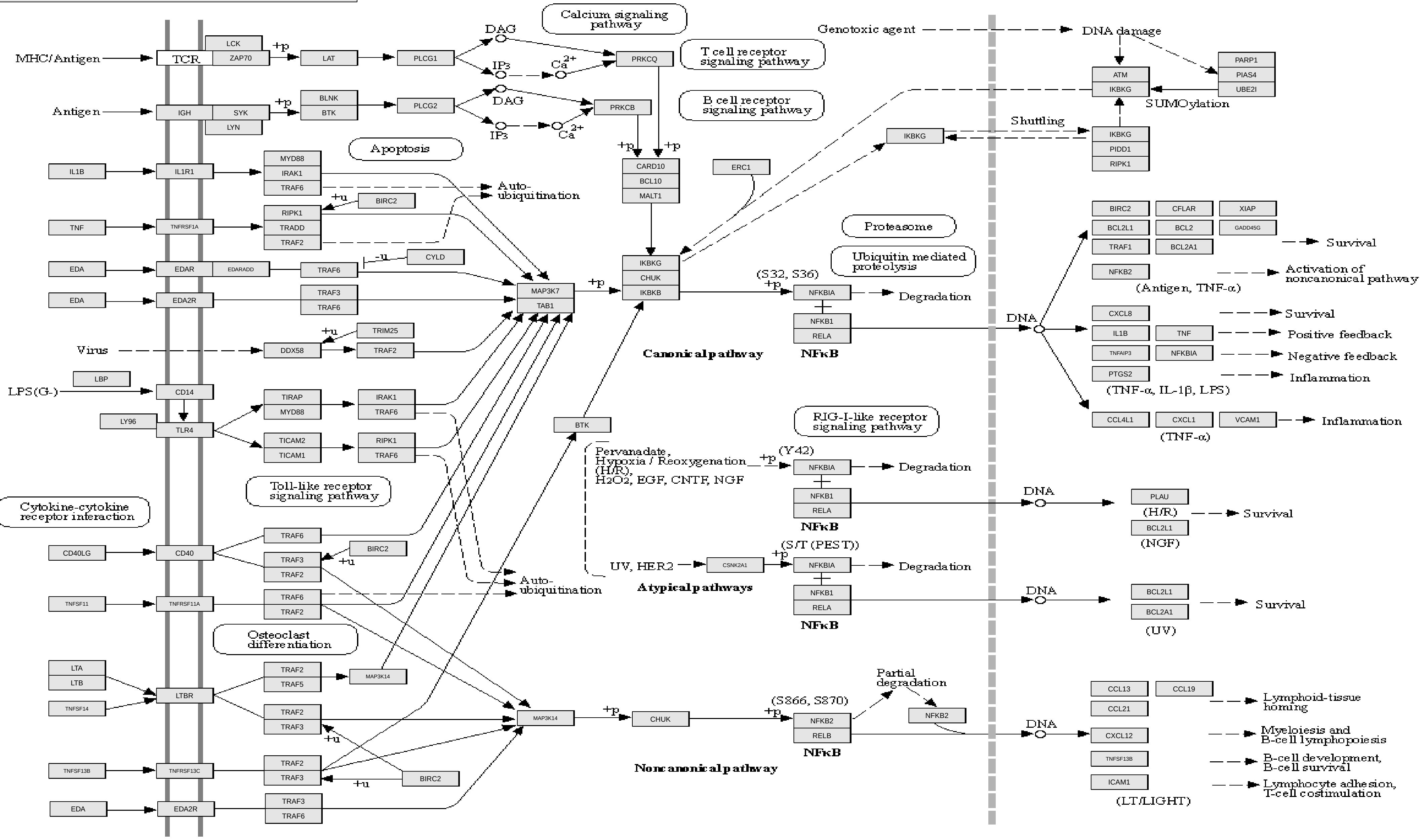


04064 4/12/19
(c) Kanehisa Laboratories

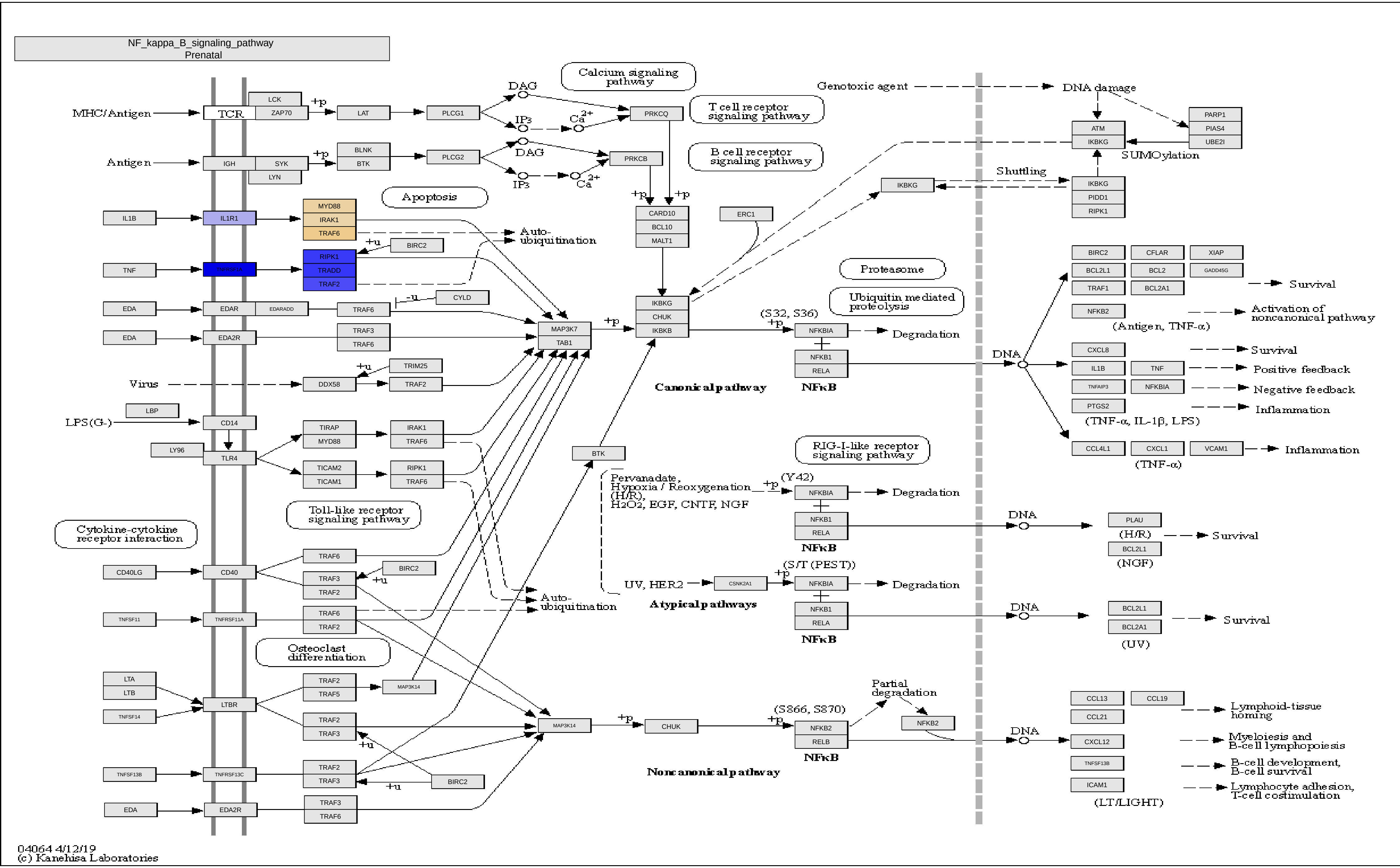
NF_kappa_B_signaling_pathway
Prenatal
MHC/Antigen
TCR
LCK
ZAP70
+p
LAT
PLCG1
DAG
IP3
Ca2+
PRKCQ
Calcium signaling pathway
T cell receptor signaling pathway
B cell receptor signaling pathway
Antigen
IGH
SYK
LYN
BLNK
BTK
PLCG2
PRKCB
IL1B
IL1R1
MYD88
IRAK1
TRAF6
Apoptosis
Auto-ubiquitination
+u
BIRC2
TNF
TNFRSF1A
RIPK1
TRADD
TRAF2
EDA
EDAR
EDARADD
TRAF6
-u
CYLD
EDA2R
TRAF3
MAP3K7
TAB1
Virus
DDX58
TRIM25
LPS(G-)
LBP
CD14
LY96
TLR4
TIRAP
TICAM2
TICAM1
RIPK1
Toll-like receptor signaling pathway
Cytokine-cytokine receptor interaction
CD40LG
CD40
TNFSF11
TNFRSF11A
Osteoclast differentiation
LTA
LTB
TNFSF14
LTBR
TRAF5
MAP3K14
TNFSF13B
TNFRSF13C
CARD10
BCL10
MALT1
ERC1
IKBKG
CHUK
IKBKB
BTK
Canonical pathway
(S32, S36)
NFKBIA
NFKB1
RELA
NFκB
Degradation
Proteasome
Ubiquitin mediated proteolysis
RIG-I-like receptor signaling pathway
Pervanadate, Hypoxia / Reoxygenation (H/R), H2O2, EGF, CNTF, NGF
(Y42)
UV, HER2
CSNK2A1
(S/T (PEST))
Atypical pathways
(S866, S870)
NFKB2
RELB
Partial degradation
Noncanonical pathway
Genotoxic agent
DNA damage
ATM
PARP1
PIAS4
UBE2I
SUMOylation
Shuttling
PIDD1
DNA
BIRC2
CFLAR
XIAP
BCL2L1
BCL2
GADD45G
TRAF1
BCL2A1
Survival
Activation of noncanonical pathway
(Antigen, TNF-α)
CXCL8
IL1B
TNF
Positive feedback
TNFAIP3
Negative feedback
PTGS2
Inflammation
(TNF-α, IL-1β, LPS)
CCL4L1
CXCL1
VCAM1
(TNF-α)
PLAU
(H/R)
(NGF)
(UV)
CCL13
CCL19
CCL21
CXCL12
ICAM1
Lymphoid-tissue homing
Myeloiesis and B-cell lymphopoiesis
B-cell development, B-cell survival
Lymphocyte adhesion, T-cell costimulation
(LT/LIGHT)
04064 4/12/19
(c) Kanehisa Laboratories

NF_kappa_B_signaling_pathway
Newborn

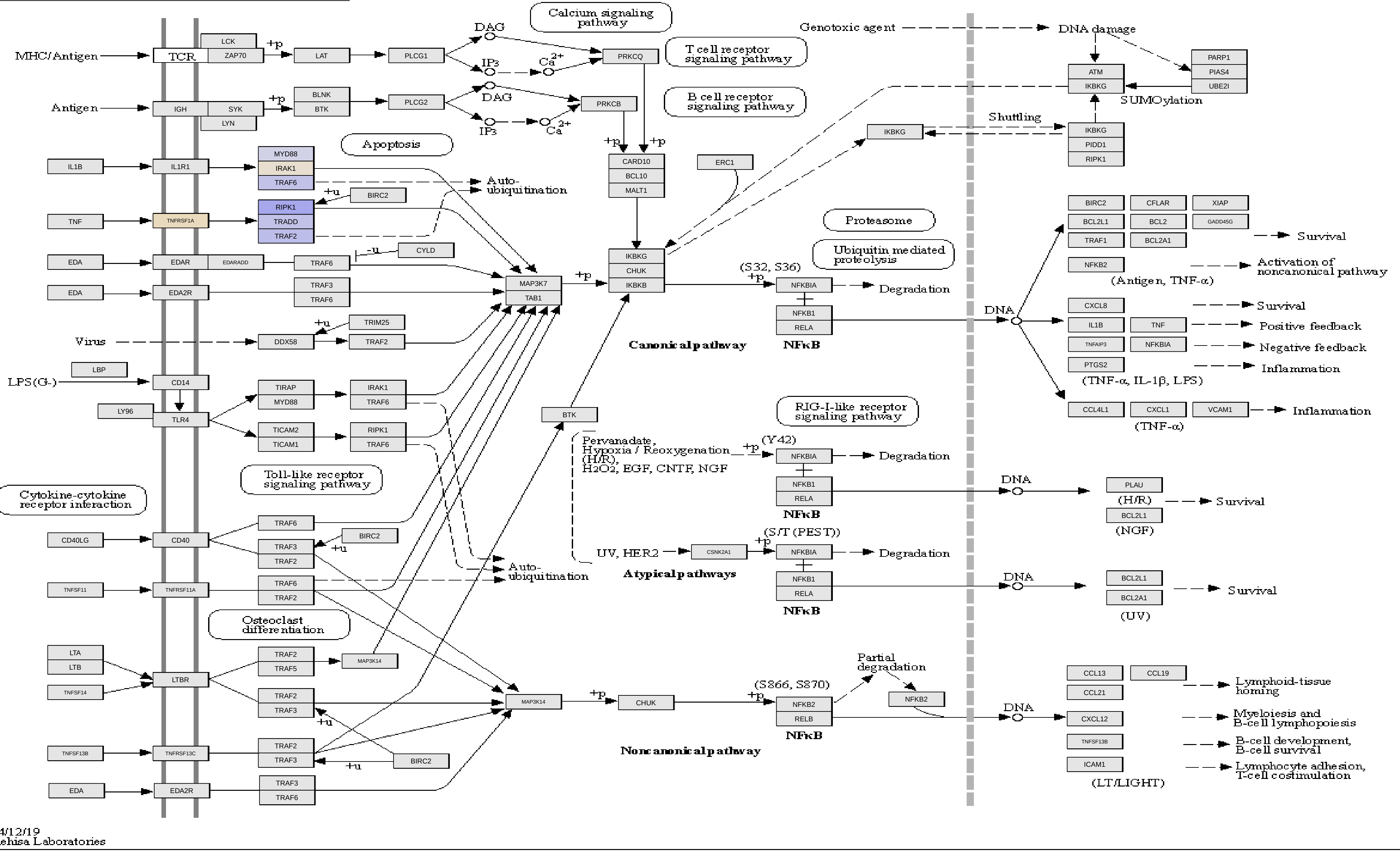

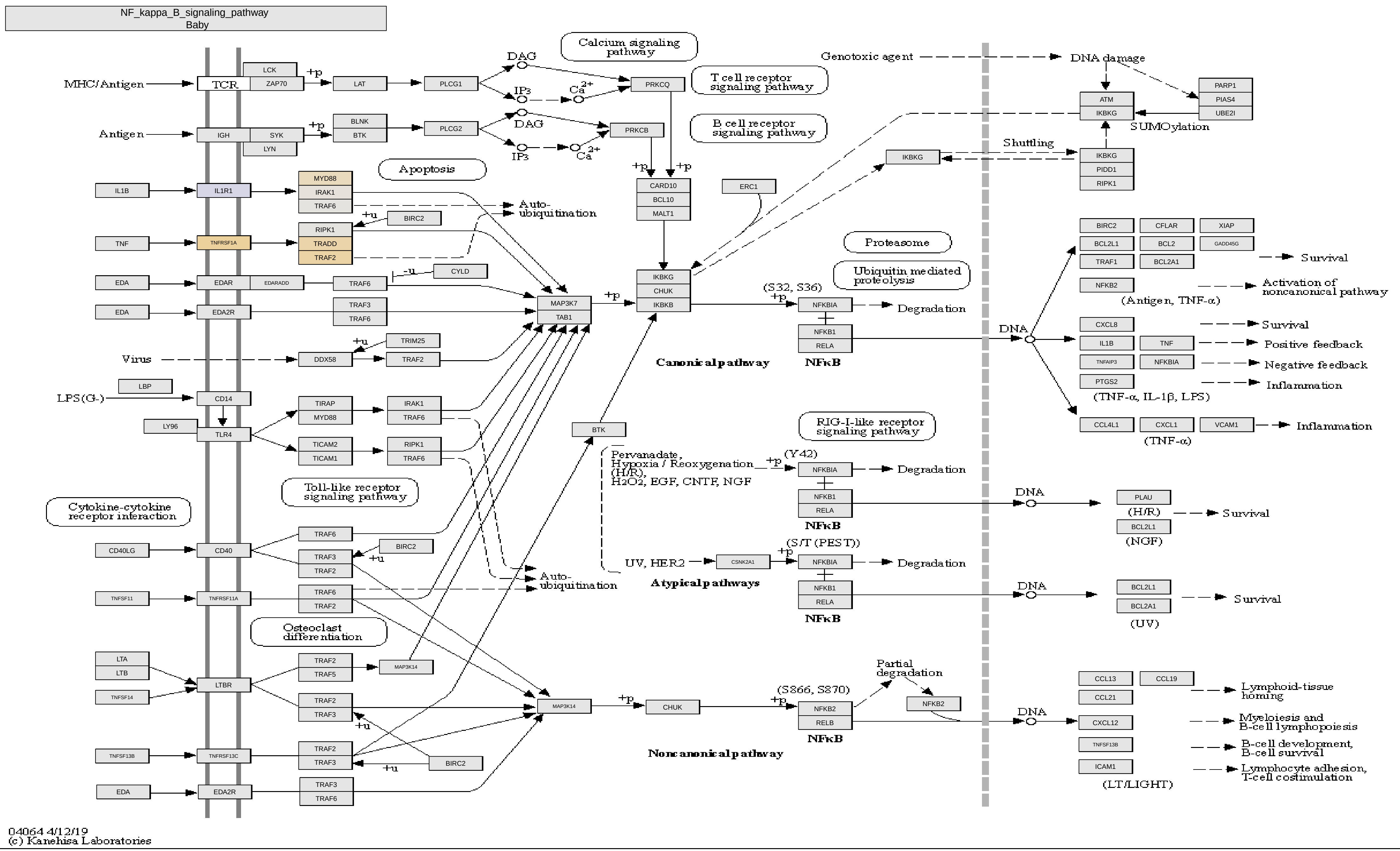
NF_kappa_B_signaling_pathway
Baby
MHC/Antigen
TCR
LCK
ZAP70
LAT
PLCG1
DAG
IP3
Ca2+
PRKCQ
Calcium signaling pathway
T cell receptor signaling pathway
B cell receptor signaling pathway
Antigen
IGH
SYK
LYN
BLNK
BTK
PLCG2
PRKCB
Genotoxic agent
DNA damage
ATM
IKBKG
PARP1
PIAS4
UBE2I
SUMOylation
Shuttling
PIDD1
RIPK1
Apoptosis
IL1B
IL1R1
MYD88
IRAK1
TRAF6
Auto-ubiquitination
BIRC2
TNF
TNFRSF1A
TRADD
TRAF2
CARD10
BCL10
MALT1
ERC1
EDA
EDAR
EDARADD
CYLD
EDA2R
TRAF3
MAP3K7
TAB1
CHUK
IKBKB
NFKBIA
NFKB1
RELA
Degradation
Proteasome
Ubiquitin mediated proteolysis
(S32, S36)
Canonical pathway
NFκB
Virus
DDX58
TRIM25
LPS(G-)
LBP
CD14
LY96
TLR4
TIRAP
TICAM2
TICAM1
Toll-like receptor signaling pathway
BTK
RIG-I-like receptor signaling pathway
Pervanadate, Hypoxia / Reoxygenation (H/R), H2O2, EGF, CNTF, NGF
(Y42)
UV, HER2
CSNK2A1
(S/T (PEST))
Atypical pathways
Cytokine-cytokine receptor interaction
CD40LG
CD40
TNFSF11
TNFRSF11A
Osteoclast differentiation
LTA
LTB
TNFSF14
LTBR
TRAF5
MAP3K14
TNFSF13B
TNFRSF13C
(S866, S870)
Partial degradation
NFKB2
RELB
Noncanonical pathway
DNA
BIRC2
CFLAR
XIAP
BCL2L1
BCL2
GADD45G
TRAF1
BCL2A1
Survival
NFKB2
Activation of noncanonical pathway
(Antigen, TNF-α)
CXCL8
IL1B
TNF
TNFAIP3
NFKBIA
PTGS2
Positive feedback
Negative feedback
Inflammation
(TNF-α, IL-1β, LPS)
CCL4L1
CXCL1
VCAM1
(TNF-α)
PLAU
(H/R)
BCL2L1
(NGF)
BCL2A1
(UV)
CCL13
CCL19
CCL21
CXCL12
TNFSF13B
ICAM1
Lymphoid-tissue homing
Myeloiesis and B-cell lymphopoiesis
B-cell development, B-cell survival
Lymphocyte adhesion, T-cell costimulation
(LT/LIGHT)
04064 4/12/19
(c) Kanehisa Laboratories

NF_kappa_B_signaling_pathway
Infant

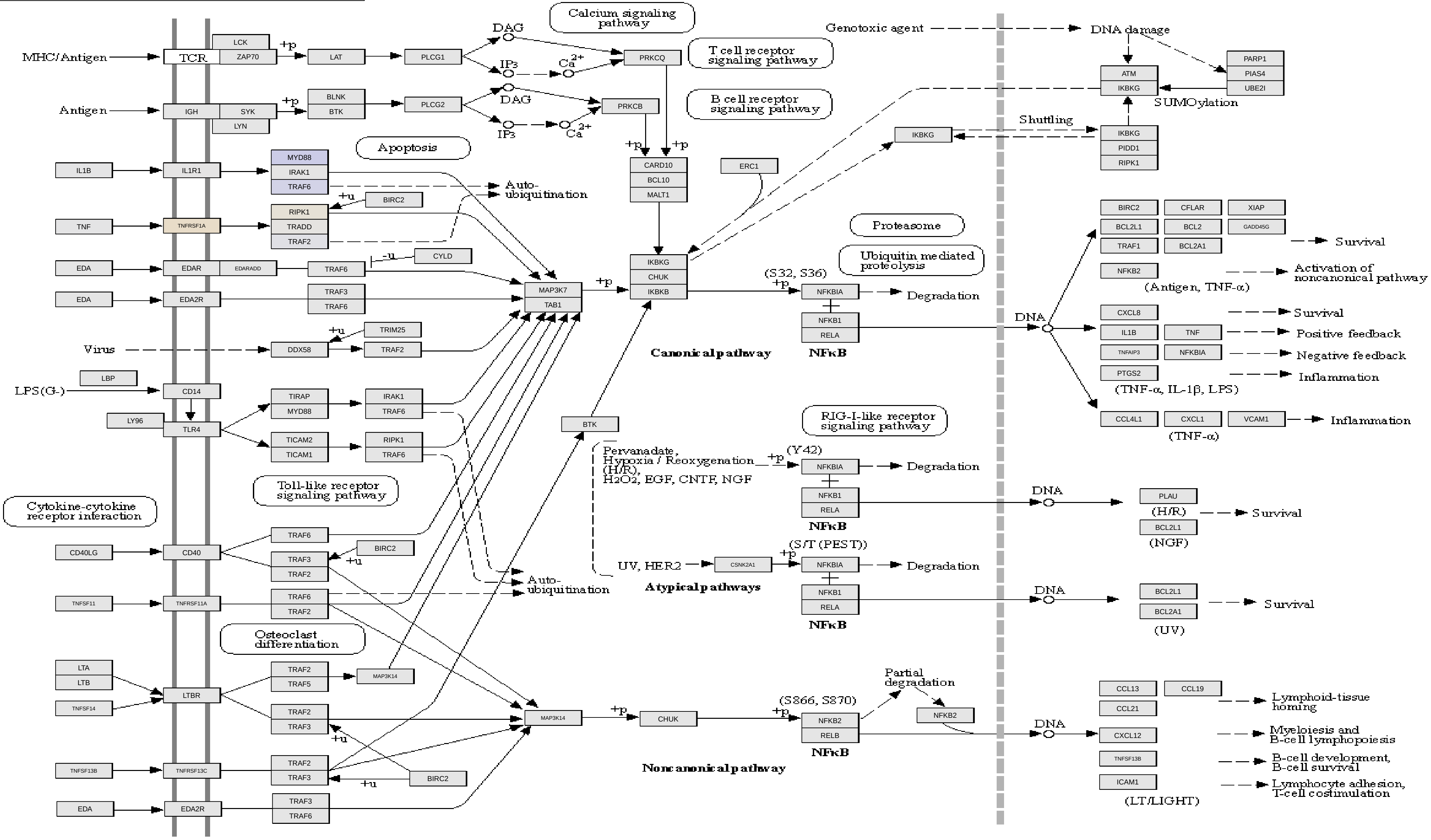


04064 4/12/19
(c) Kanehisa Laboratories

NF_kappa_B_signaling_pathway
Teenager

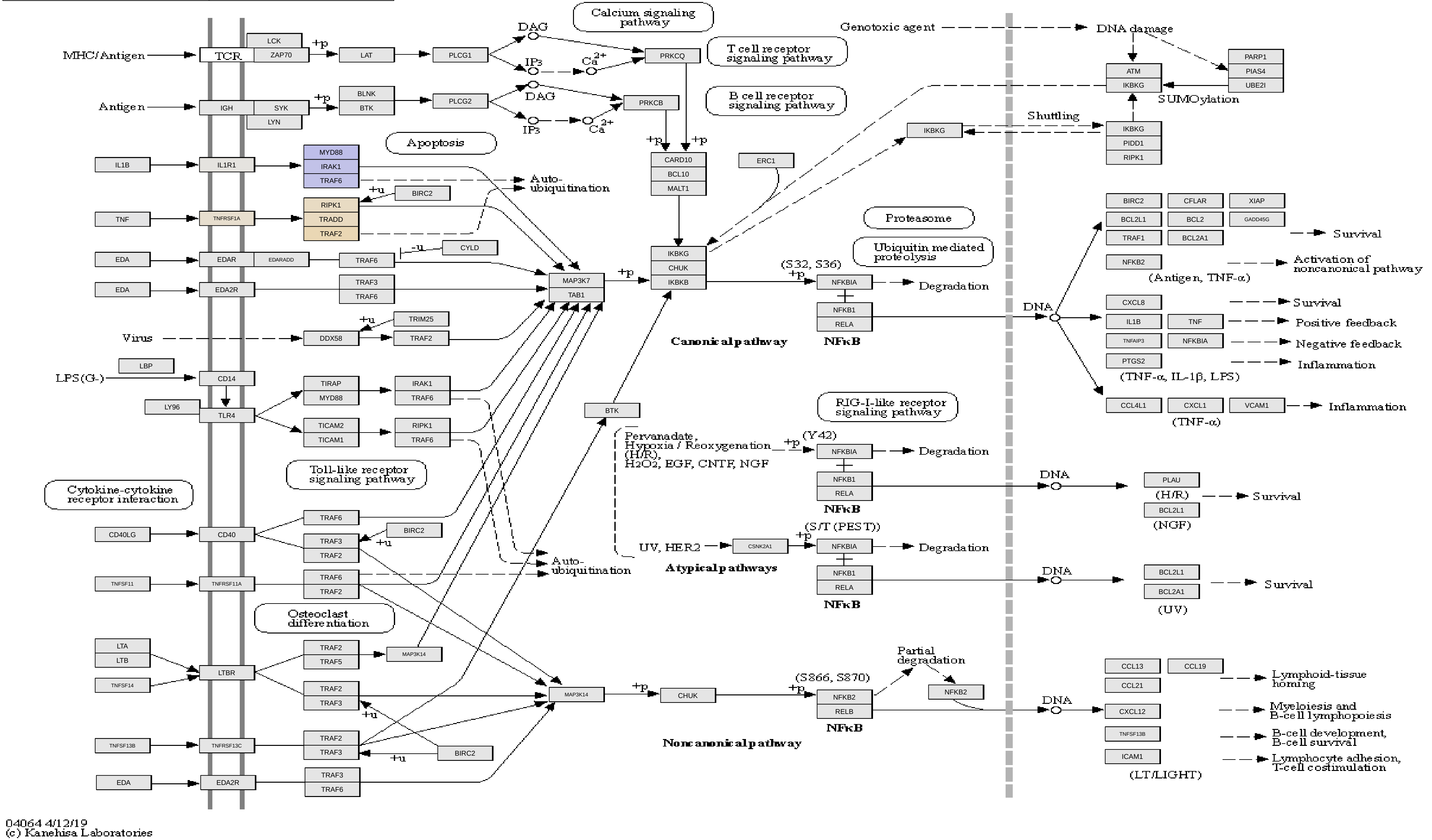

NF_kappa_B_signaling_pathway
Adult

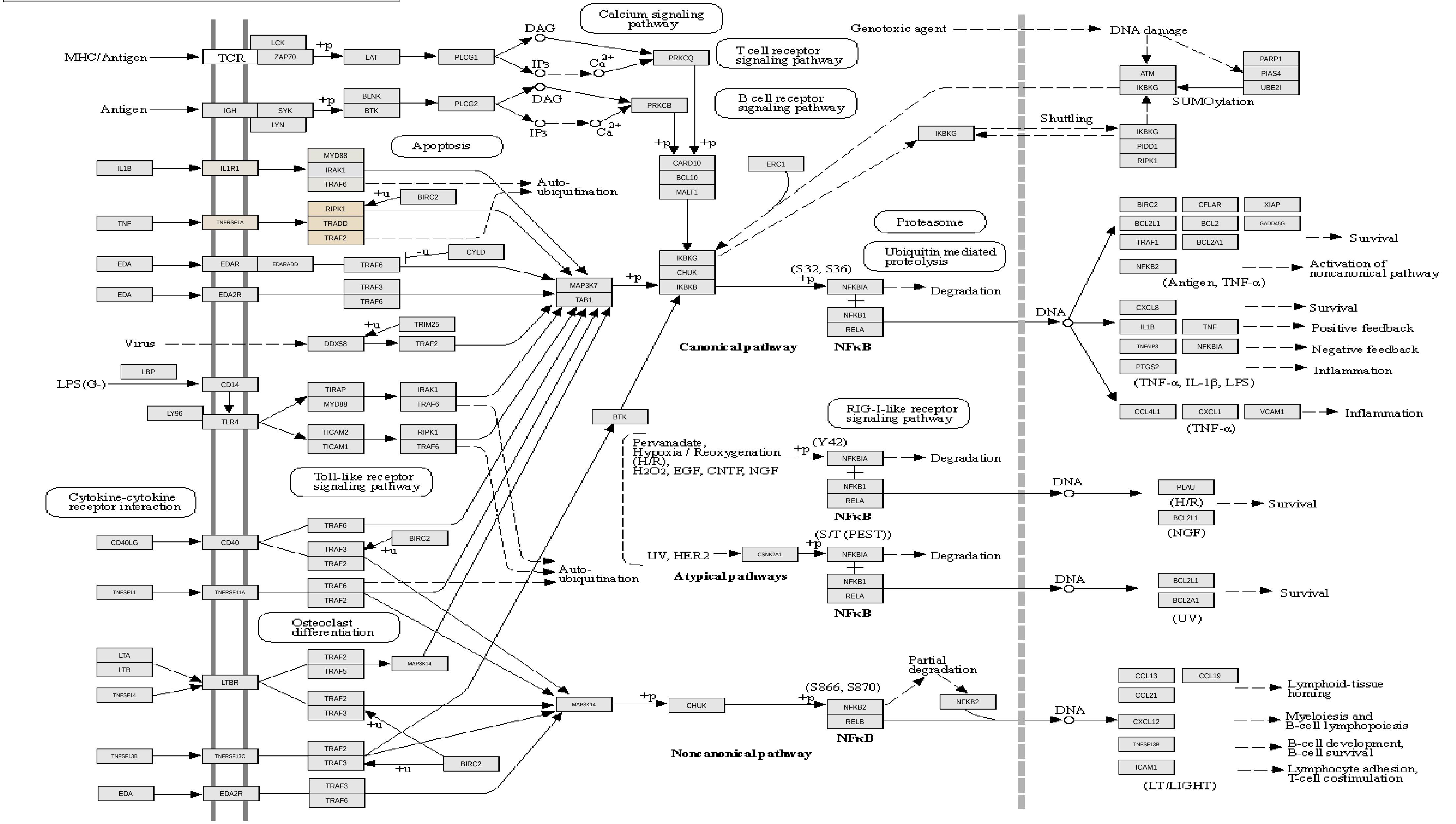


04064 4/12/19
(c) Kanehisa Laboratories

NF_kappa_B_signaling_pathway
Late adult

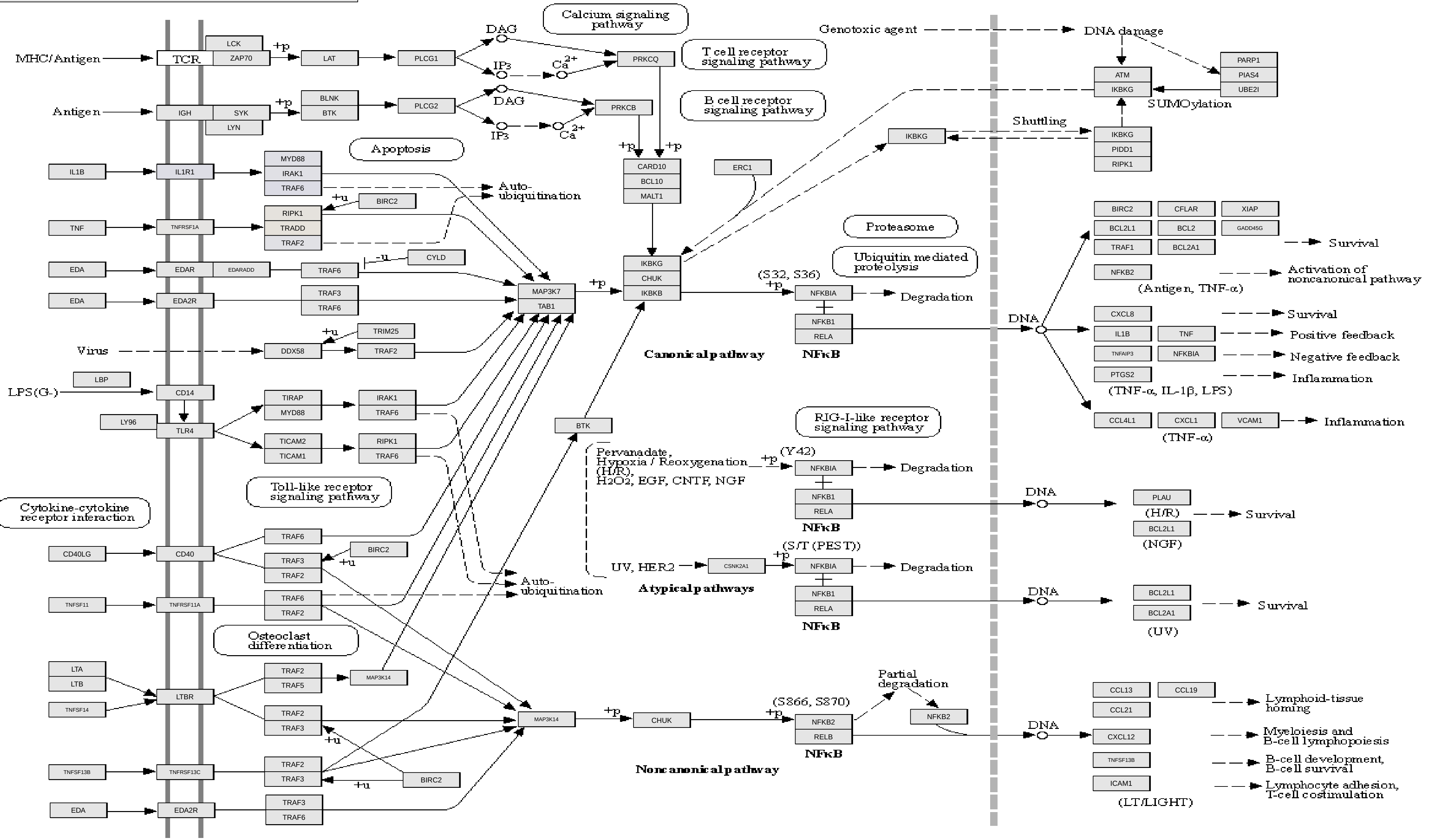


04064 4/12/19
(c) Kanehisa Laboratories
