# Supplementary material for: The Transcriptome and Methylome of the Developing and Aging Brain and Their Relations to Gliomas and Psychological Disorders
Source: Cells. 2022 Jan 21;11(3):362. doi: 10.3390/cells11030362 (PMC8834030; doi:10.3390/cells11030362)

# NOD−like receptor signaling pathway

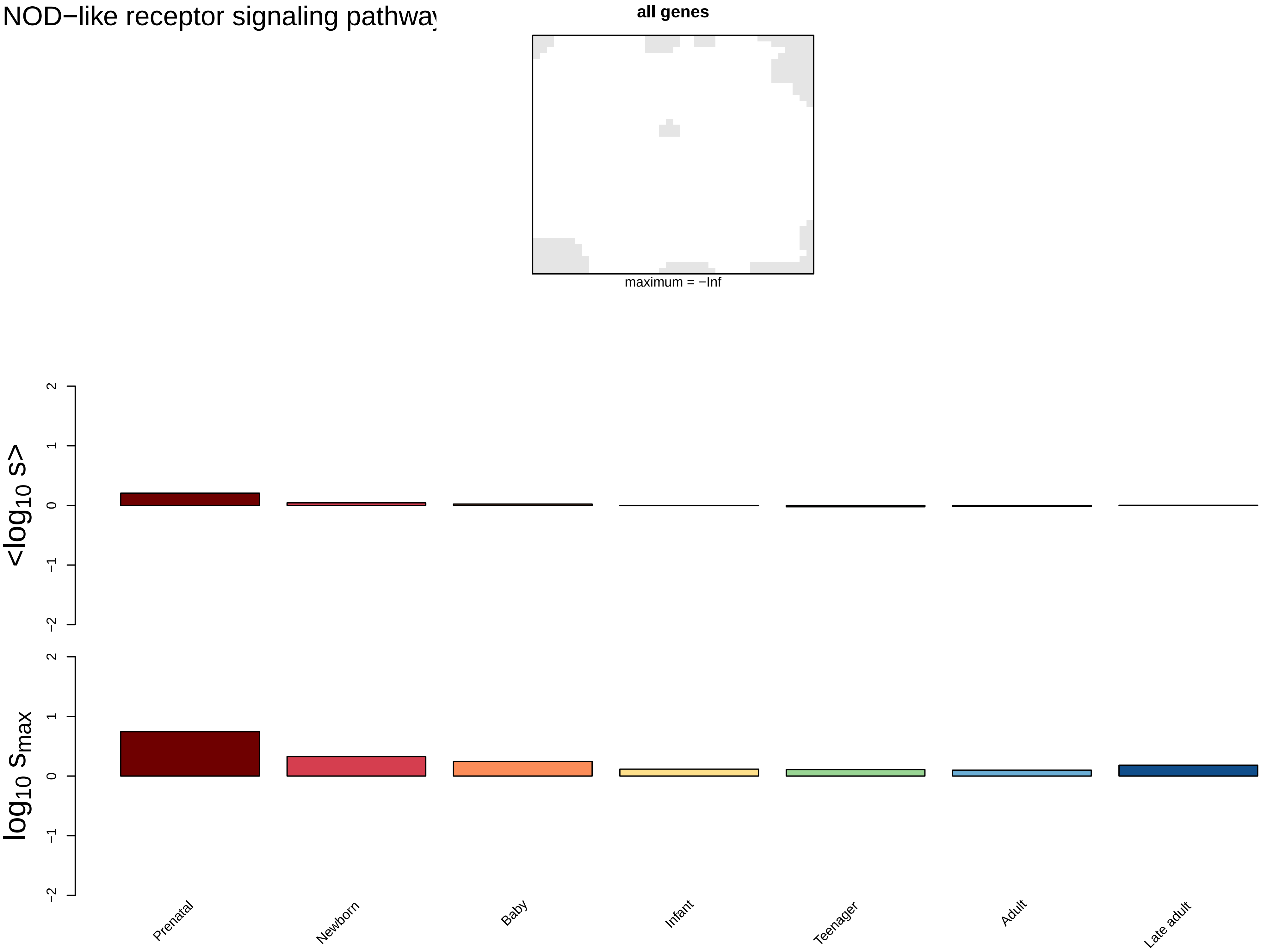

NOD_like_receptor_signaling_pathway
genes with data

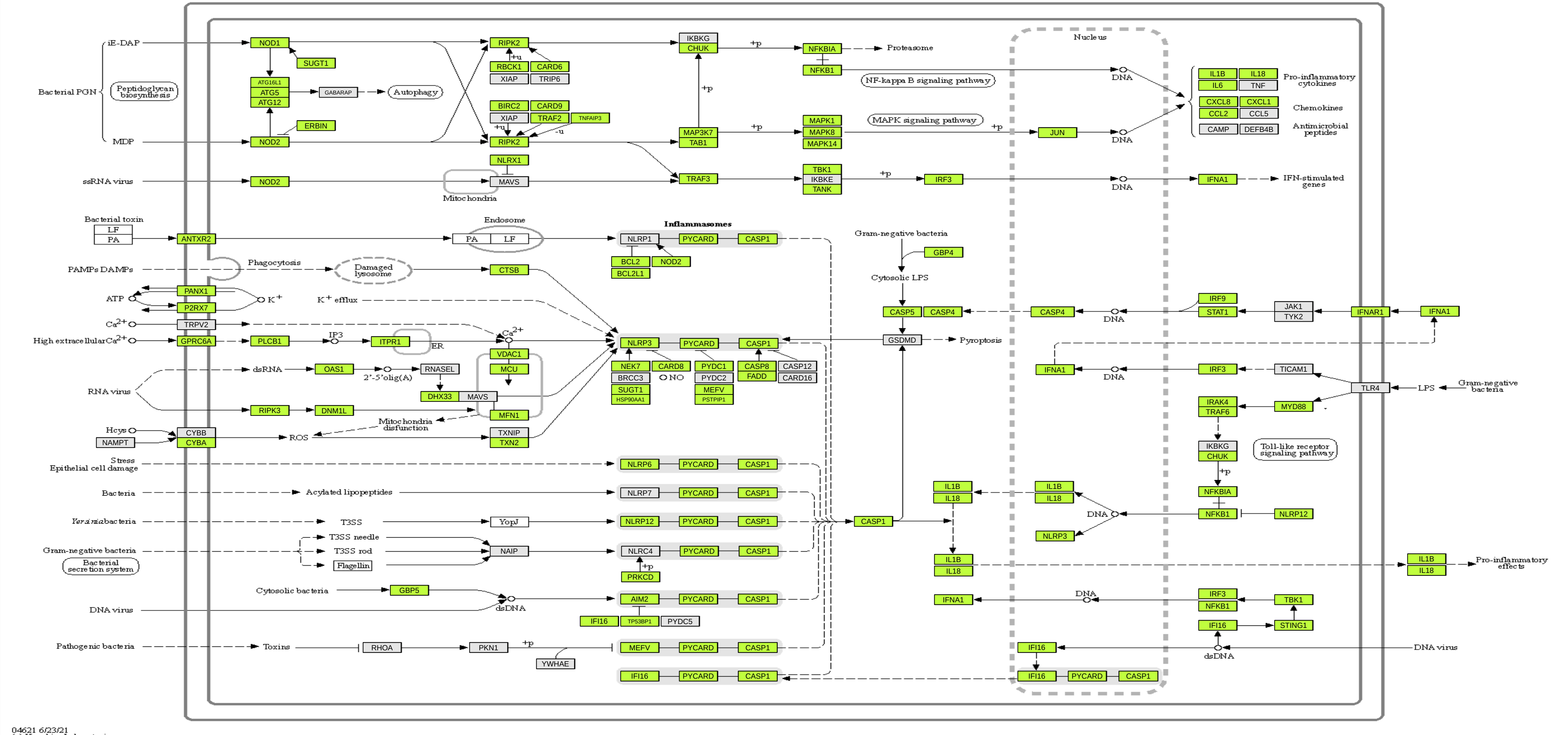

04621 6/23/21
(c) Kanehisa Laboratories

NOD_like_receptor_signaling_pathway
sink nodes

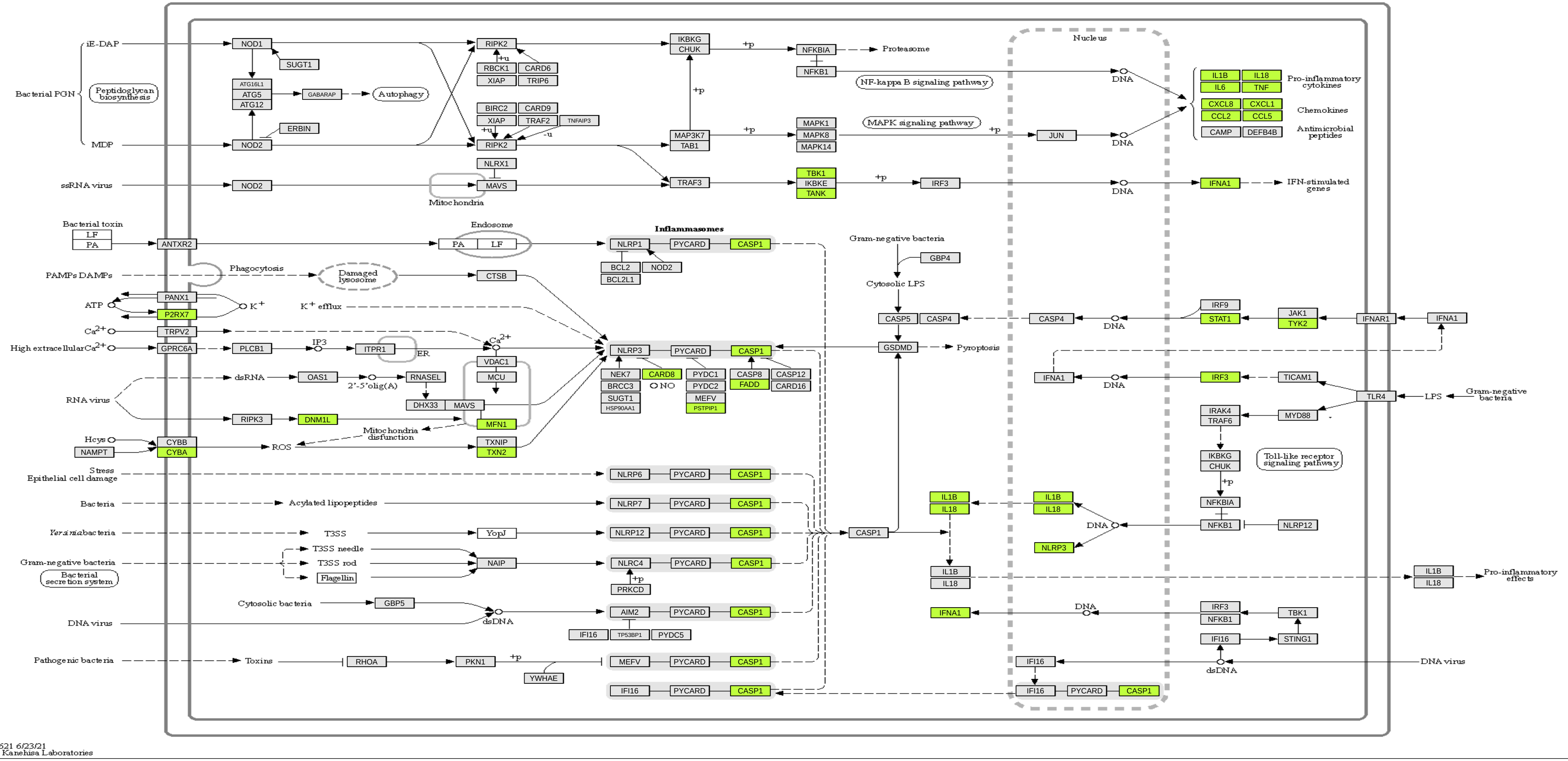

NOD_like_receptor_signaling_pathway
Prenatal

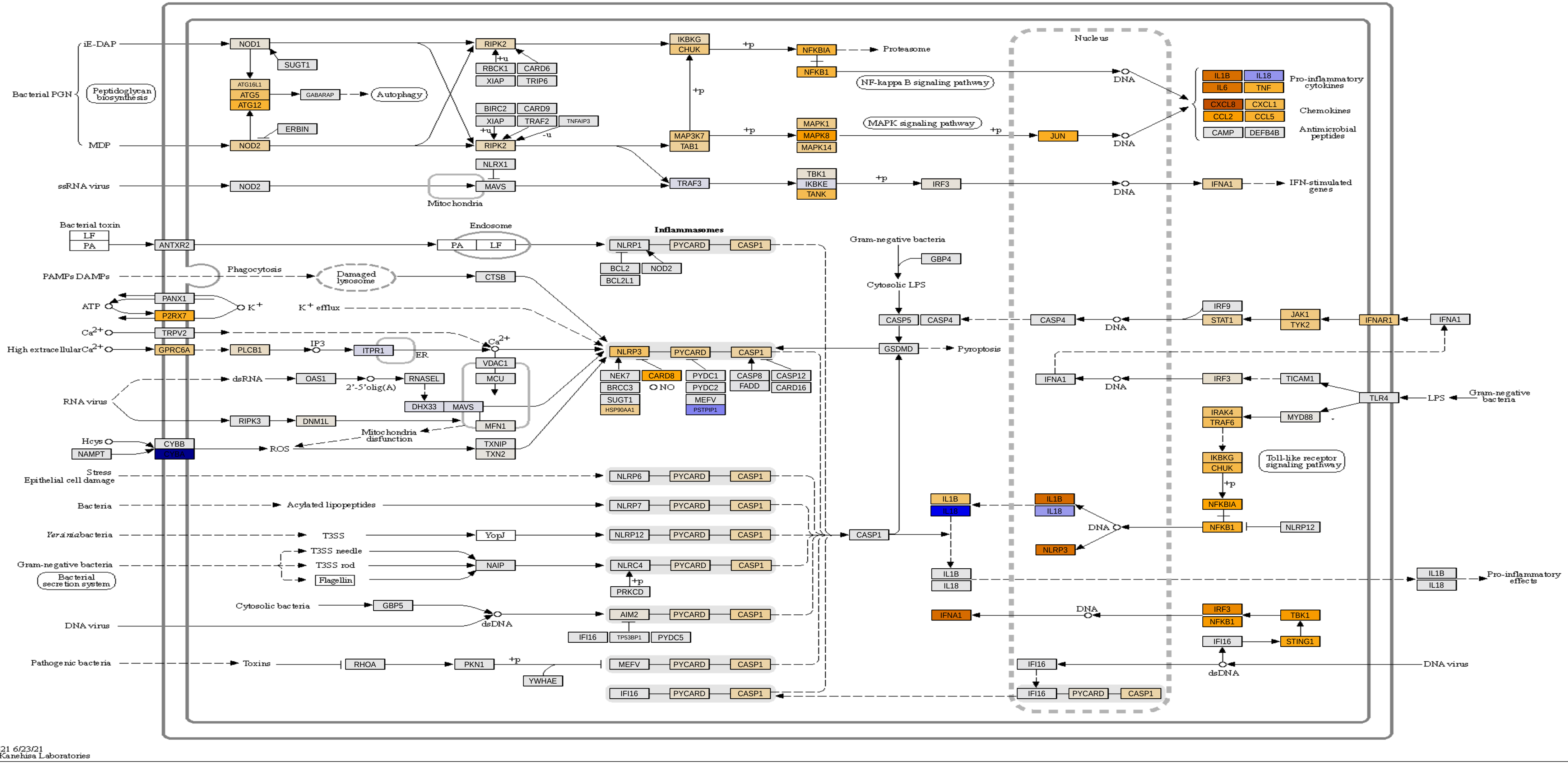

NOD_like_receptor_signaling_pathway
Newborn

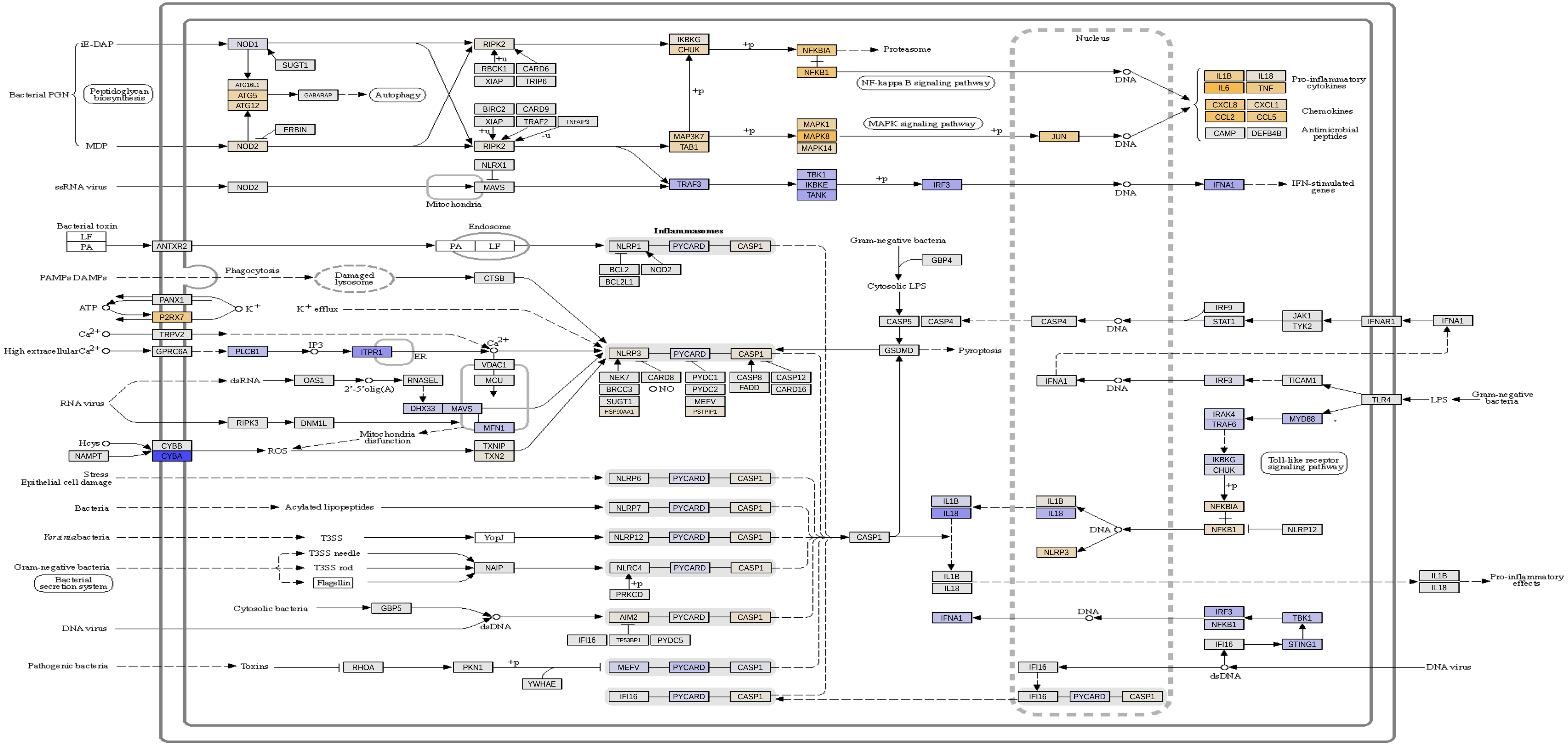


04621 6/23/21
(c) Kanehisa Laboratories

NOD_like_receptor_signaling_pathway
Baby

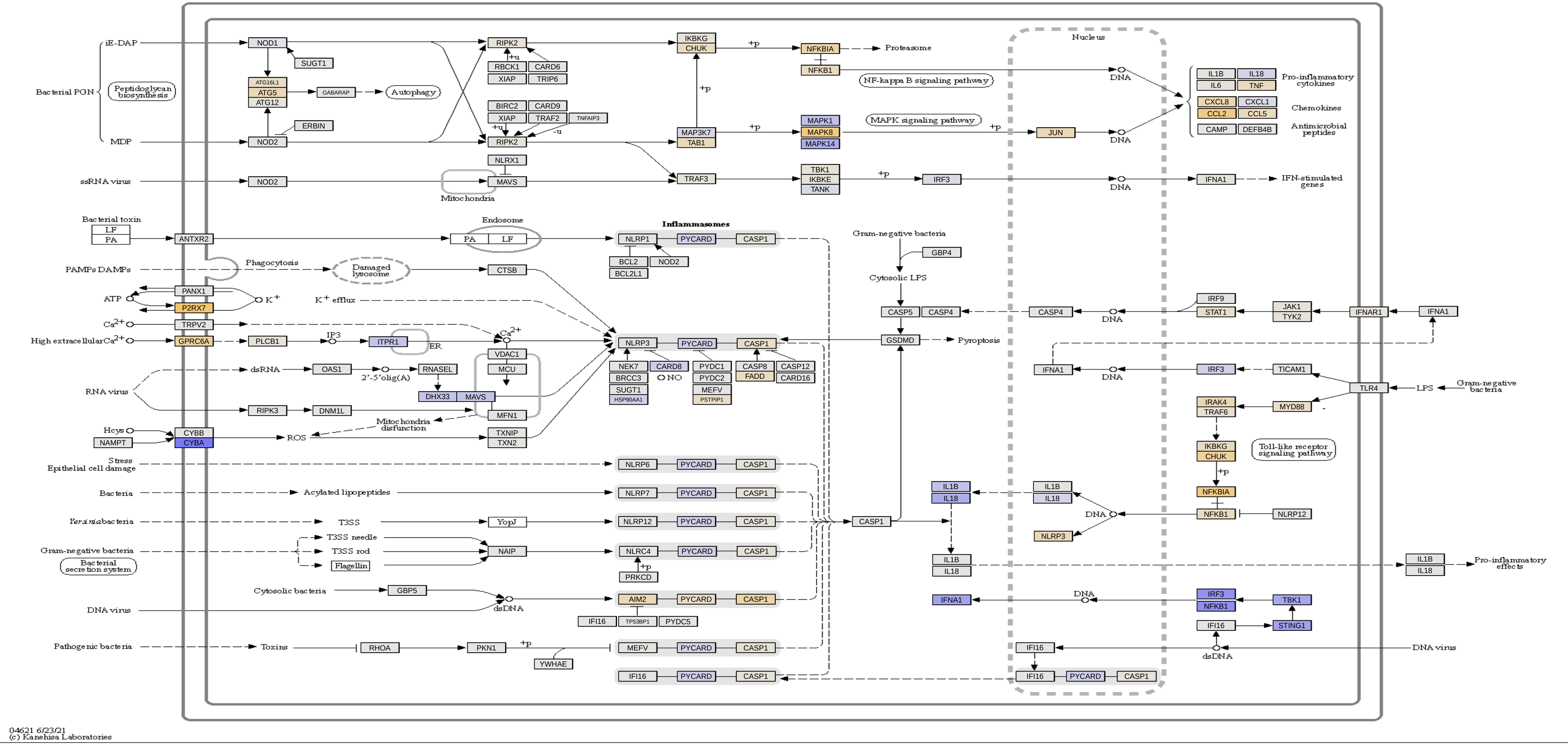

NOD_like_receptor_signaling_pathway
Infant

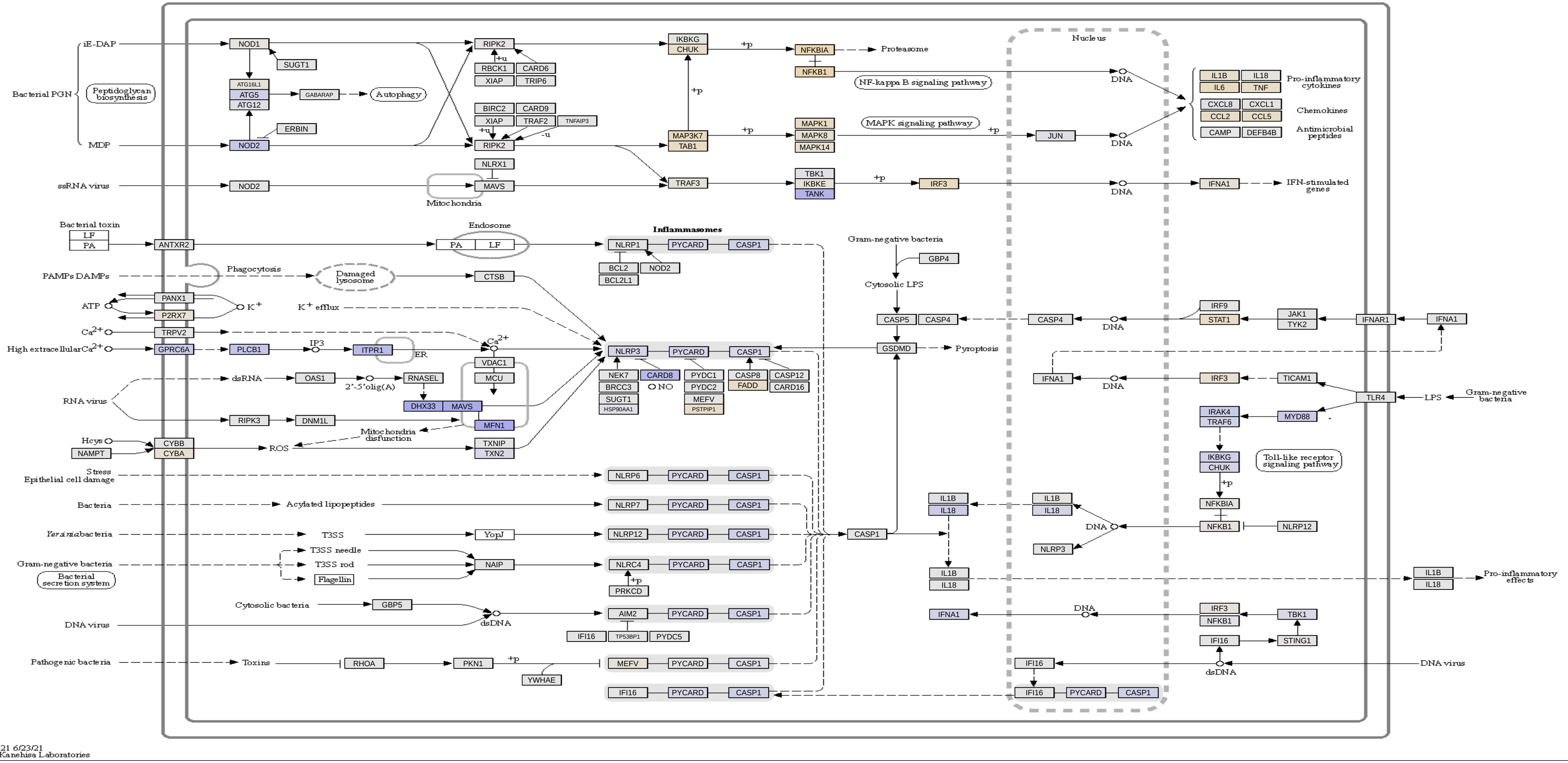

NOD_like_receptor_signaling_pathway
Teenager

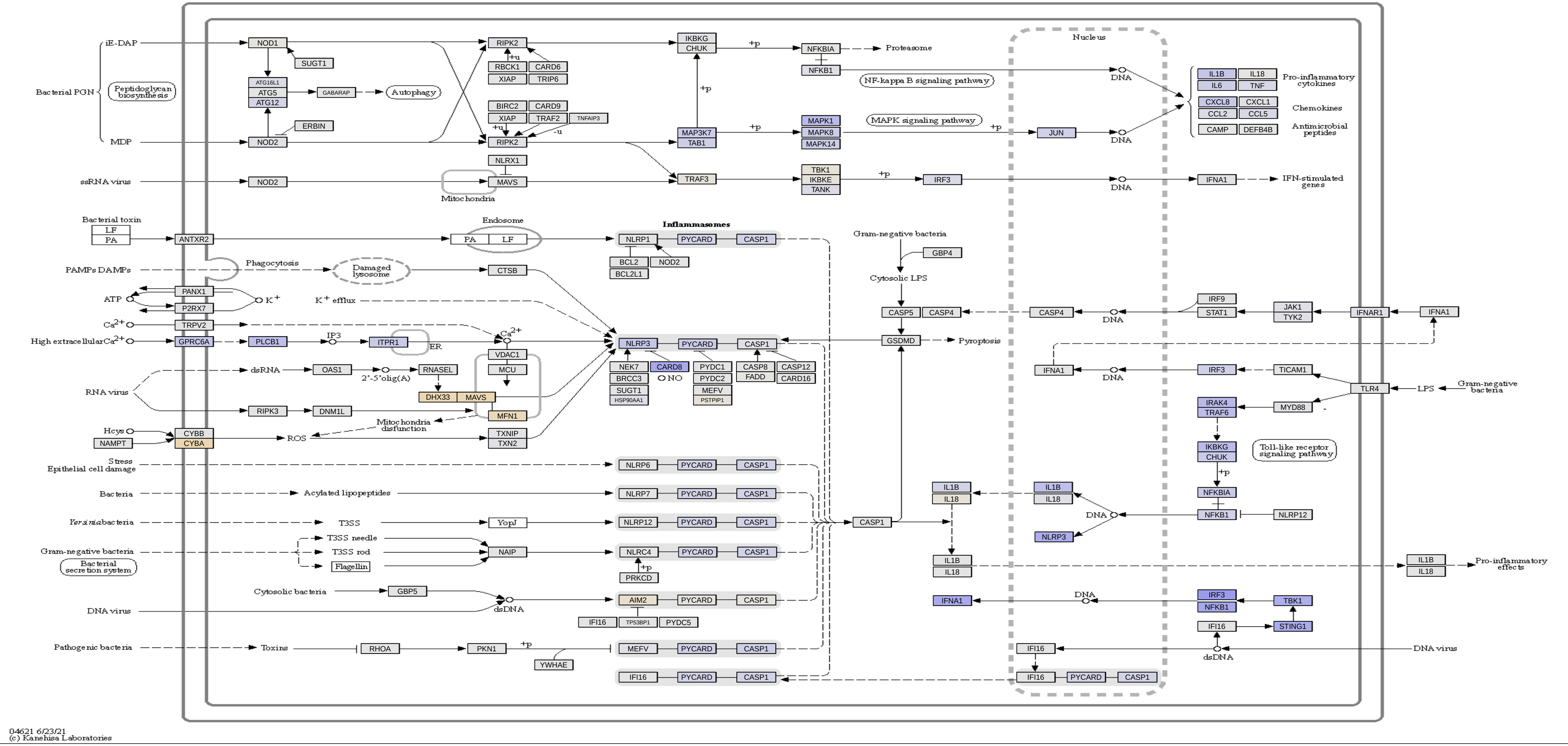

NOD_like_receptor_signaling_pathway
Adult

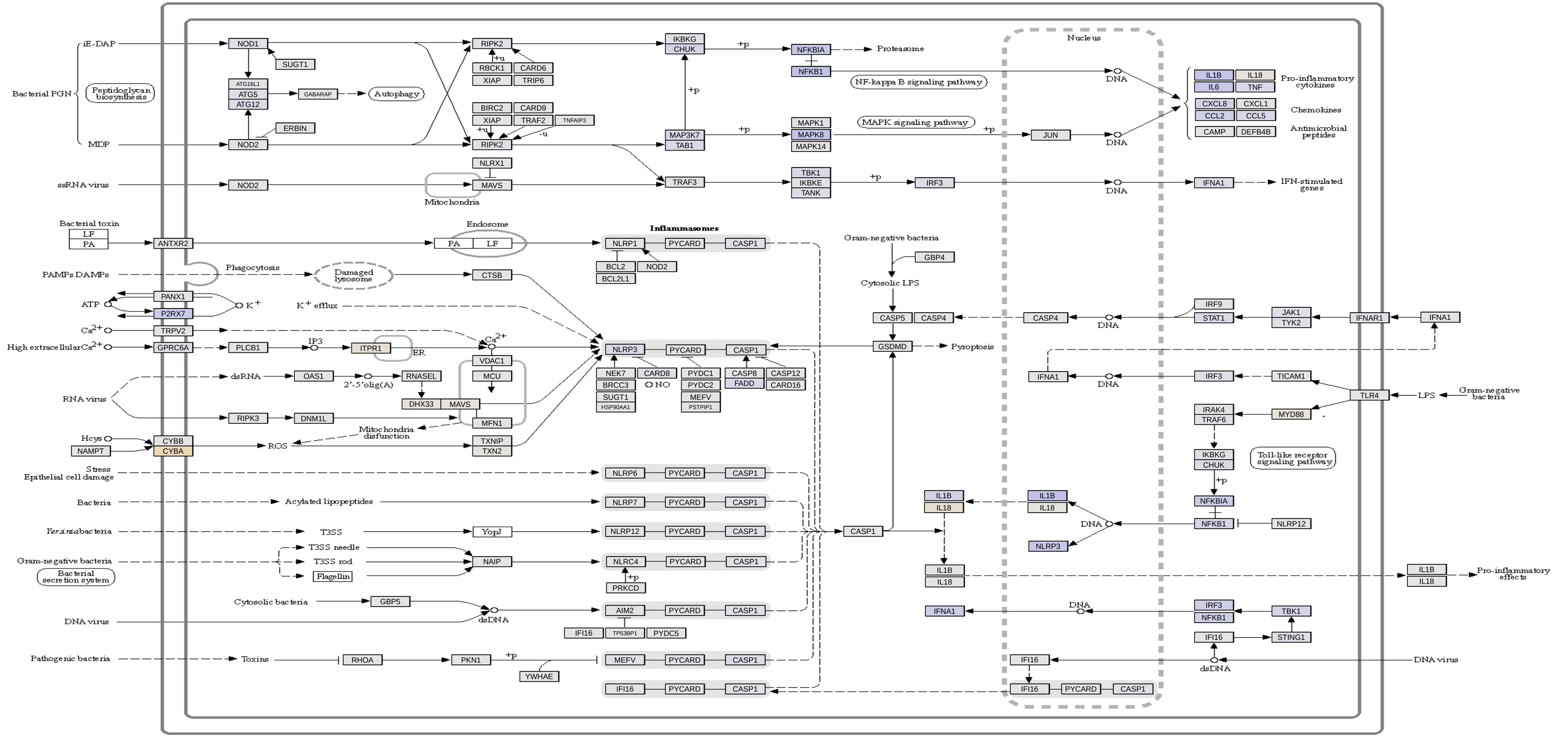


04621 6/23/21
(c) Kanehisa Laboratories

NOD_like_receptor_signaling_pathway
Late adult

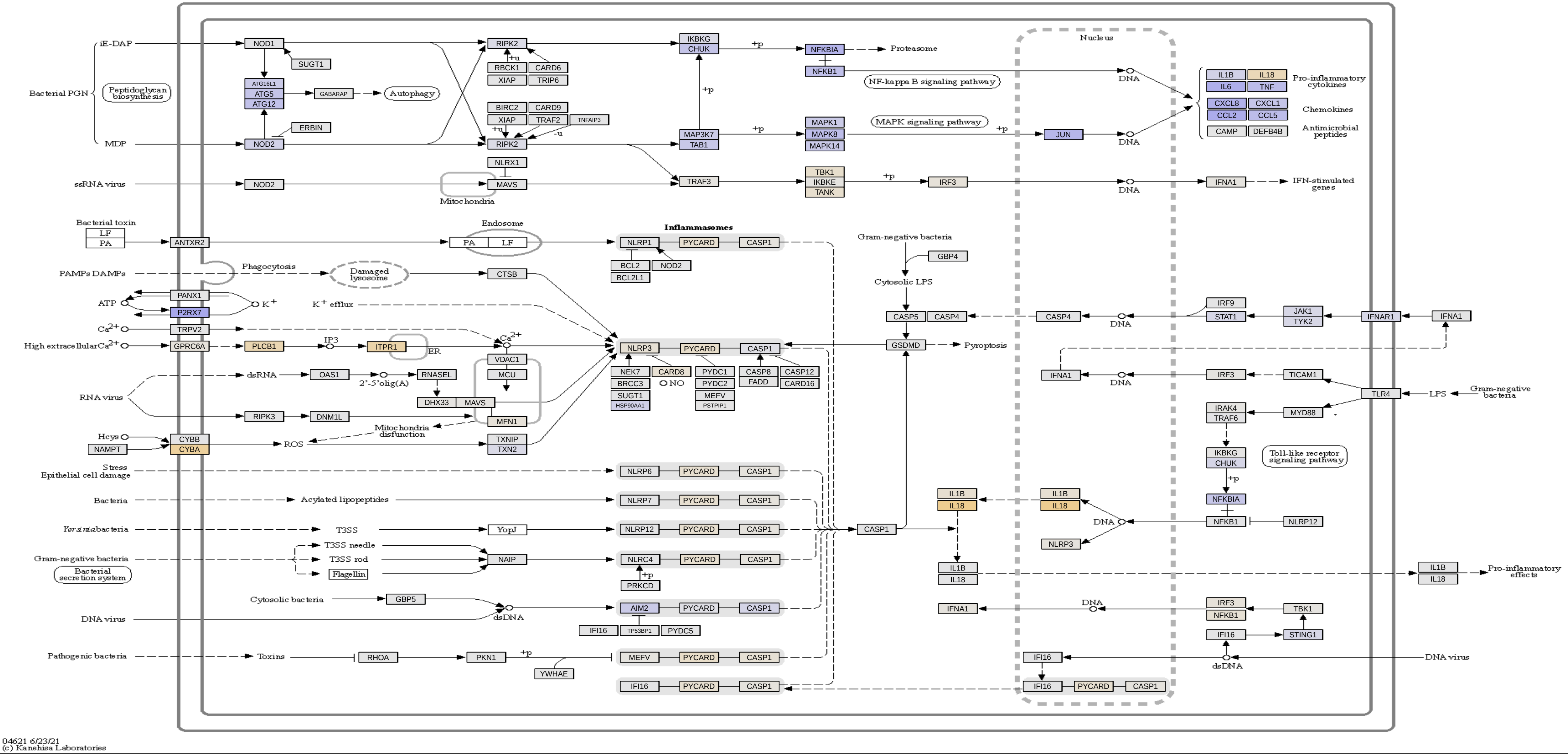

Supplement: Supplementary file 1 [file cells-11-00362-s001.zip › Suppl-Material-S4-Pathways-PSF_Methylation/NOD_like_receptor_signaling_pathway.pdf]
