# Supplementary material for: The Transcriptome and Methylome of the Developing and Aging Brain and Their Relations to Gliomas and Psychological Disorders
Source: Cells. 2022 Jan 21;11(3):362. doi: 10.3390/cells11030362 (PMC8834030; doi:10.3390/cells11030362)
Supplement: Supplementary file 1 [file cells-11-00362-s001.zip › Suppl-Material-S4-Pathways-PSF_Methylation/Notch_signaling_pathway.pdf]

Notch signaling pathway

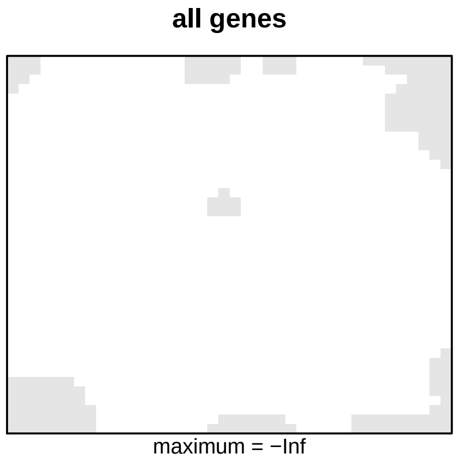

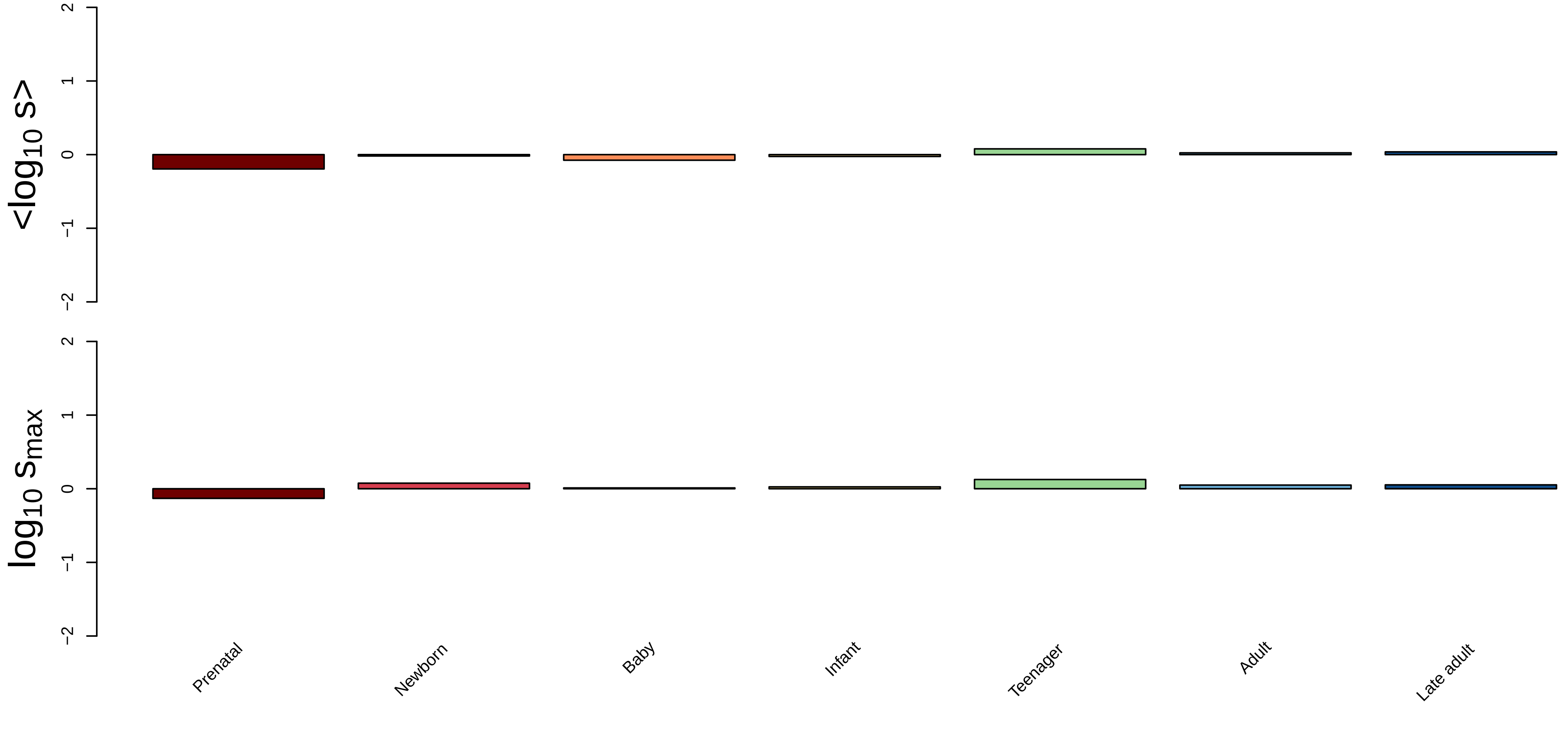

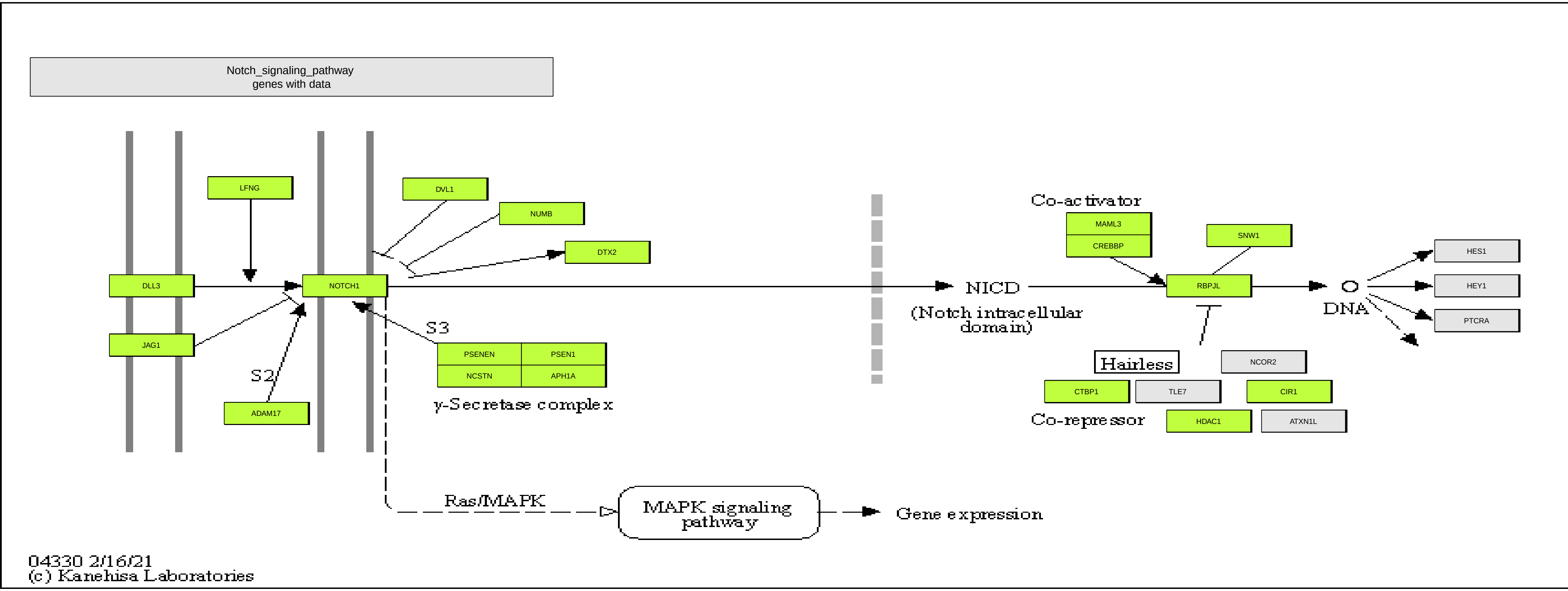

Notch_signaling_pathway
genes with data
LFNG
DVL1
NUMB
DTX2
DLL3
NOTCH1
JAG1
S2
ADAM17
S3
PSENEN
PSEN1
NCSTN
APH1A
γ-Secretase complex
NICD
(Notch intracellular domain)
Co-activator
MAML3
CREBBP
SNW1
RBPJL
DNA
HES1
HEY1
PTCRA
Hairless
NCOR2
CTBP1
TLE7
CIR1
Co-repressor
HDAC1
ATXN1L
Ras/MAPK
MAPK signaling pathway
Gene expression
04330 2/16/21
(c) Kanehisa Laboratories

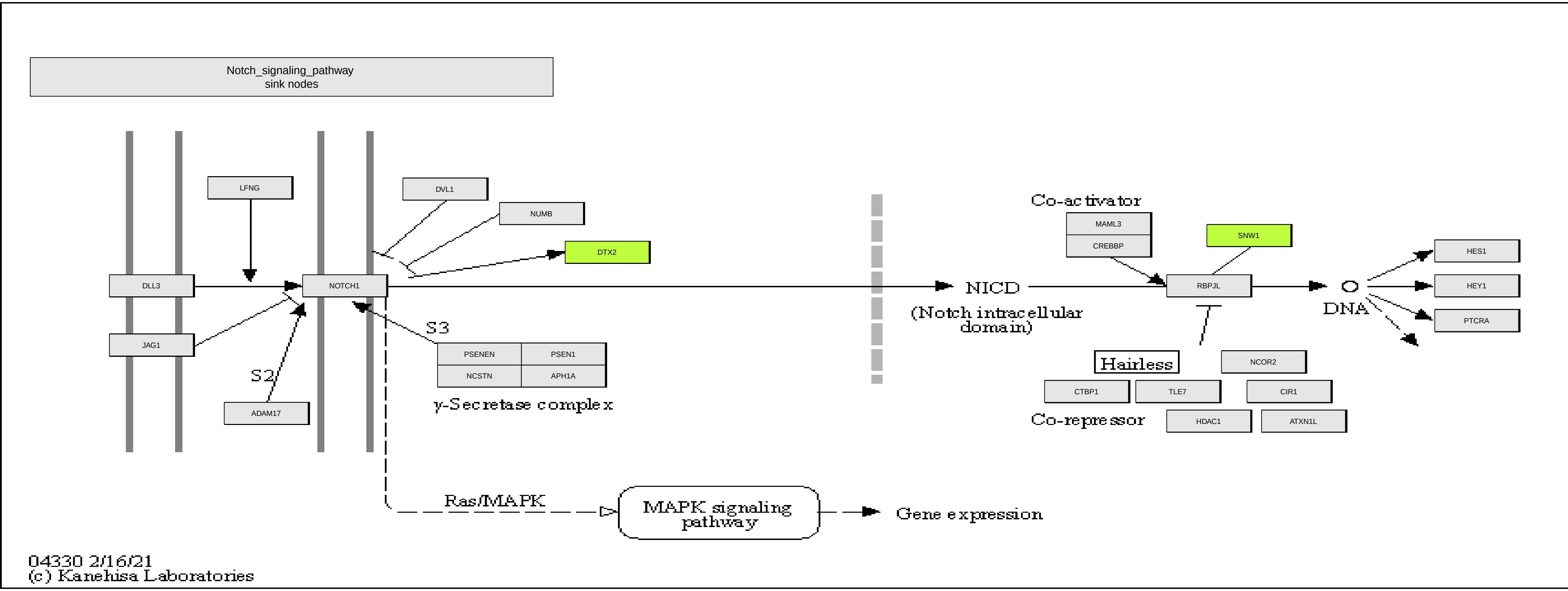

Notch_signaling_pathway
sink nodes
LFNG
DVL1
NUMB
DTX2
DLL3
NOTCH1
JAG1
S2
ADAM17
S3
PSENEN
PSEN1
NCSTN
APH1A
γ-Secretase complex
NICD
(Notch intracellular domain)
Co-activator
MAML3
CREBBP
SNW1
RBPJL
DNA
HES1
HEY1
PTCRA
Hairless
NCOR2
CTBP1
TLE7
CIR1
Co-repressor
HDAC1
ATXN1L
Ras/MAPK
MAPK signaling pathway
Gene expression
04330 2/16/21
(c) Kanehisa Laboratories

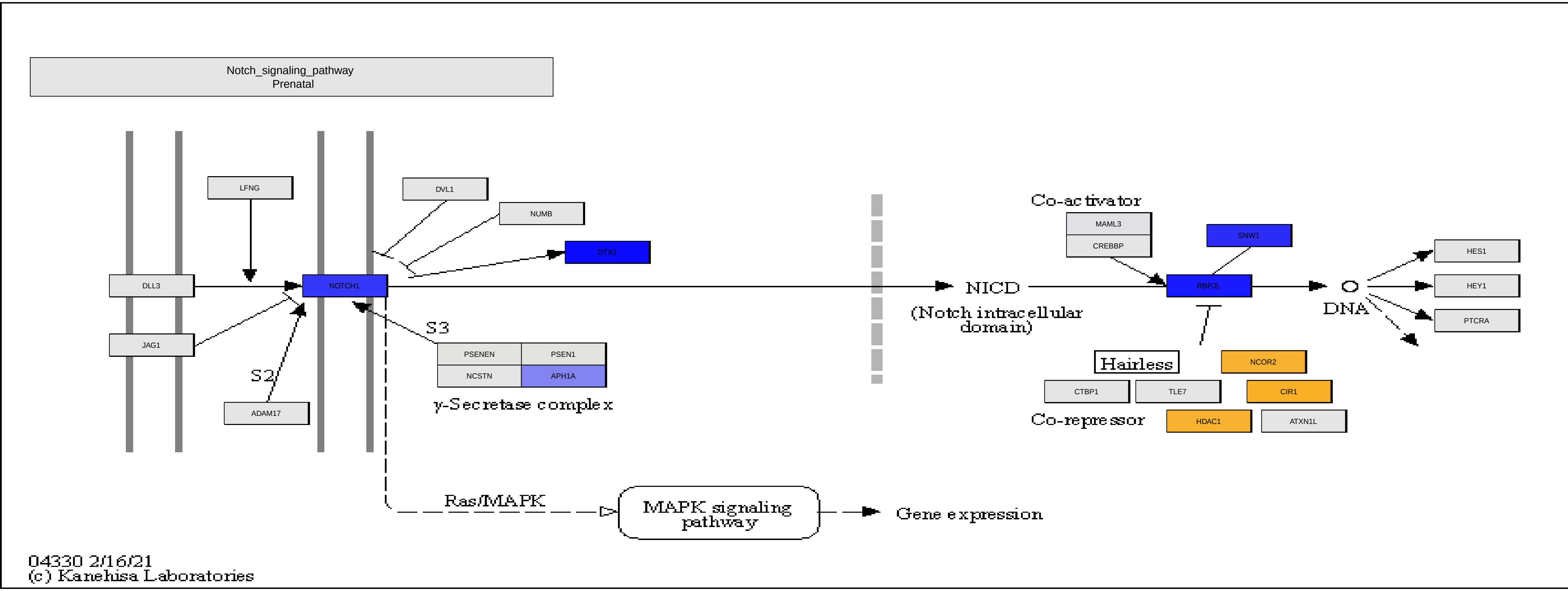
Notch_signaling_pathway
Prenatal
LFNG
DVL1
NUMB
DTX2
DLL3
NOTCH1
JAG1
S2
ADAM17
S3
PSENEN
PSEN1
NCSTN
APH1A
γ-Secretase complex
Ras/MAPK
MAPK signaling pathway
Gene expression
NICD
(Notch intracellular domain)
Co-activator
MAML3
CREBBP
SNW1
RBPJL
DNA
HES1
HEY1
PTCRA
Hairless
NCOR2
CTBP1
TLE7
CIR1
Co-repressor
HDAC1
ATXN1L
04330 2/16/21
(c) Kanehisa Laboratories

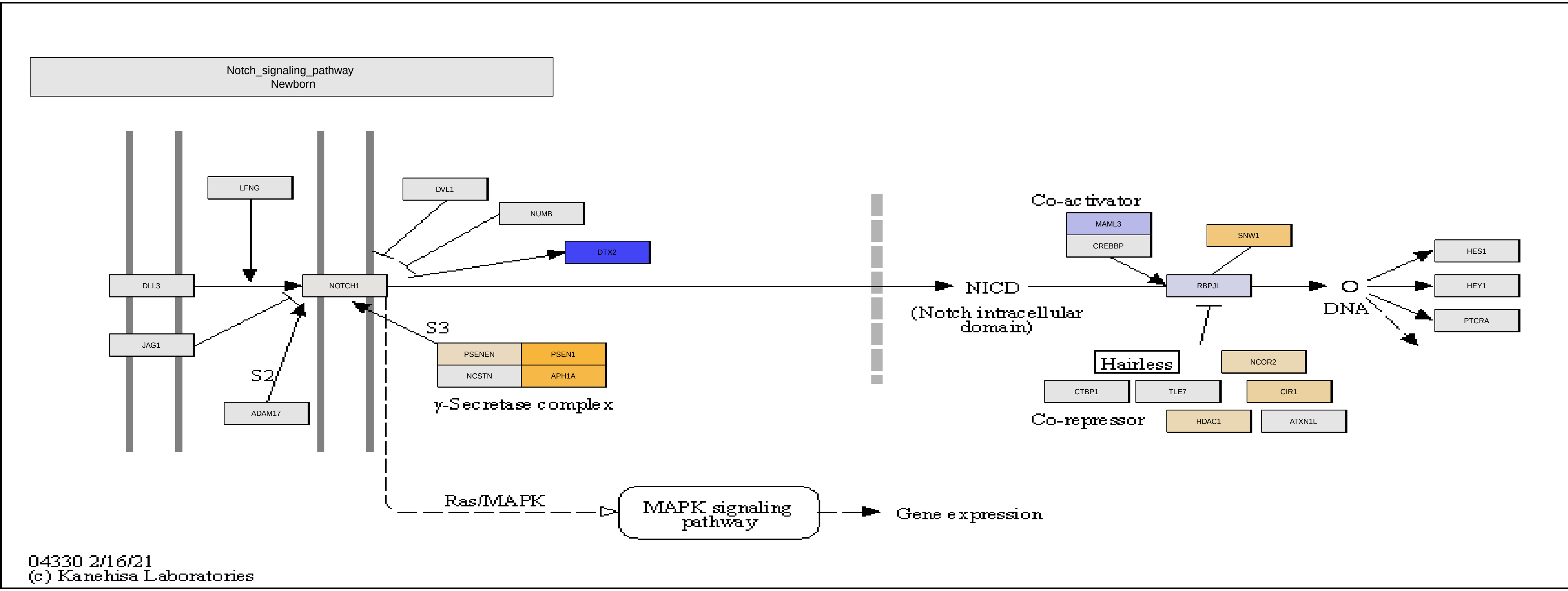
Notch_signaling_pathway
Newborn
LFNG
DVL1
NUMB
DTX2
DLL3
NOTCH1
JAG1
S2
ADAM17
S3
PSENEN
PSEN1
NCSTN
APH1A
γ-Secretase complex
NICD
(Notch intracellular domain)
Co-activator
MAML3
CREBBP
SNW1
RBPJL
DNA
HES1
HEY1
PTCRA
Hairless
NCOR2
CTBP1
TLE7
CIR1
Co-repressor
HDAC1
ATXN1L
Ras/MAPK
MAPK signaling pathway
Gene expression
04330 2/16/21
(c) Kanehisa Laboratories

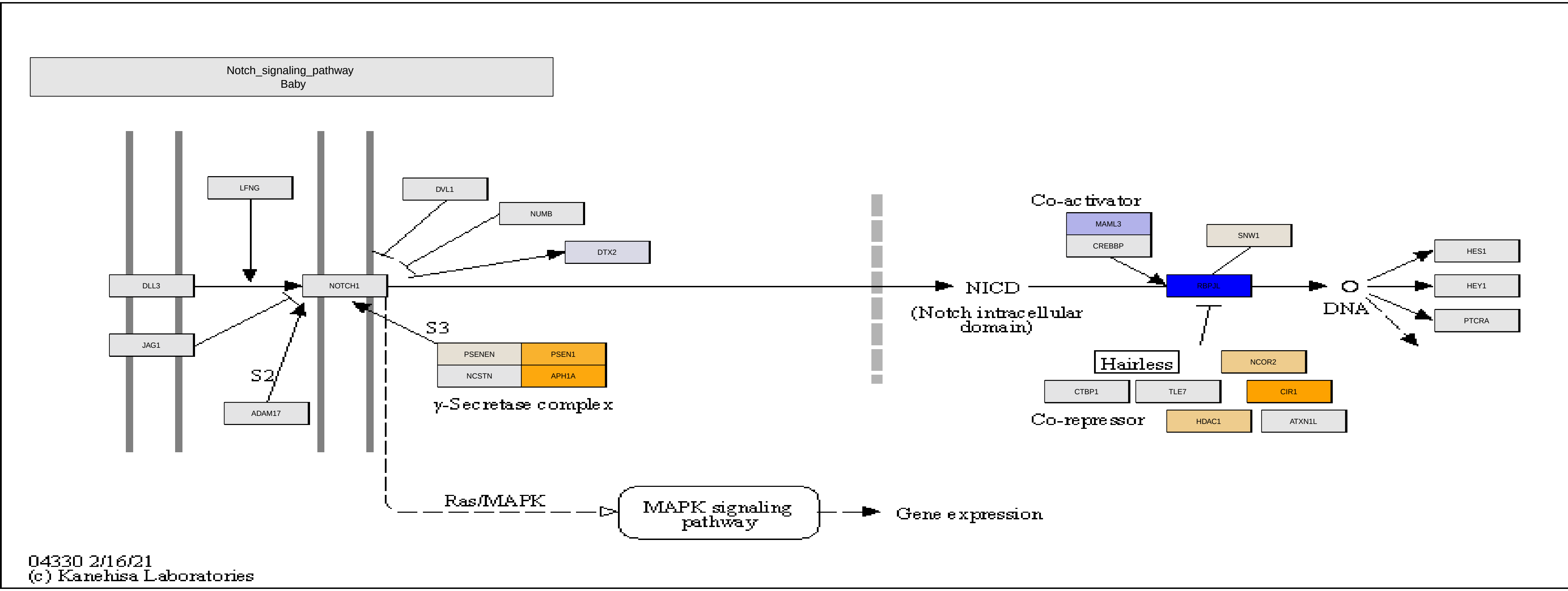
Notch_signaling_pathway
Baby
LFNG
DVL1
NUMB
DTX2
DLL3
NOTCH1
JAG1
S2
ADAM17
S3
PSENEN
PSEN1
NCSTN
APH1A
γ-Secretase complex
NICD
(Notch intracellular domain)
Co-activator
MAML3
CREBBP
SNW1
RBPJL
Hairless
NCOR2
CTBP1
TLE7
CIR1
Co-repressor
HDAC1
ATXN1L
DNA
HES1
HEY1
PTCRA
Ras/MAPK
MAPK signaling pathway
Gene expression
04330 2/16/21
(c) Kanehisa Laboratories

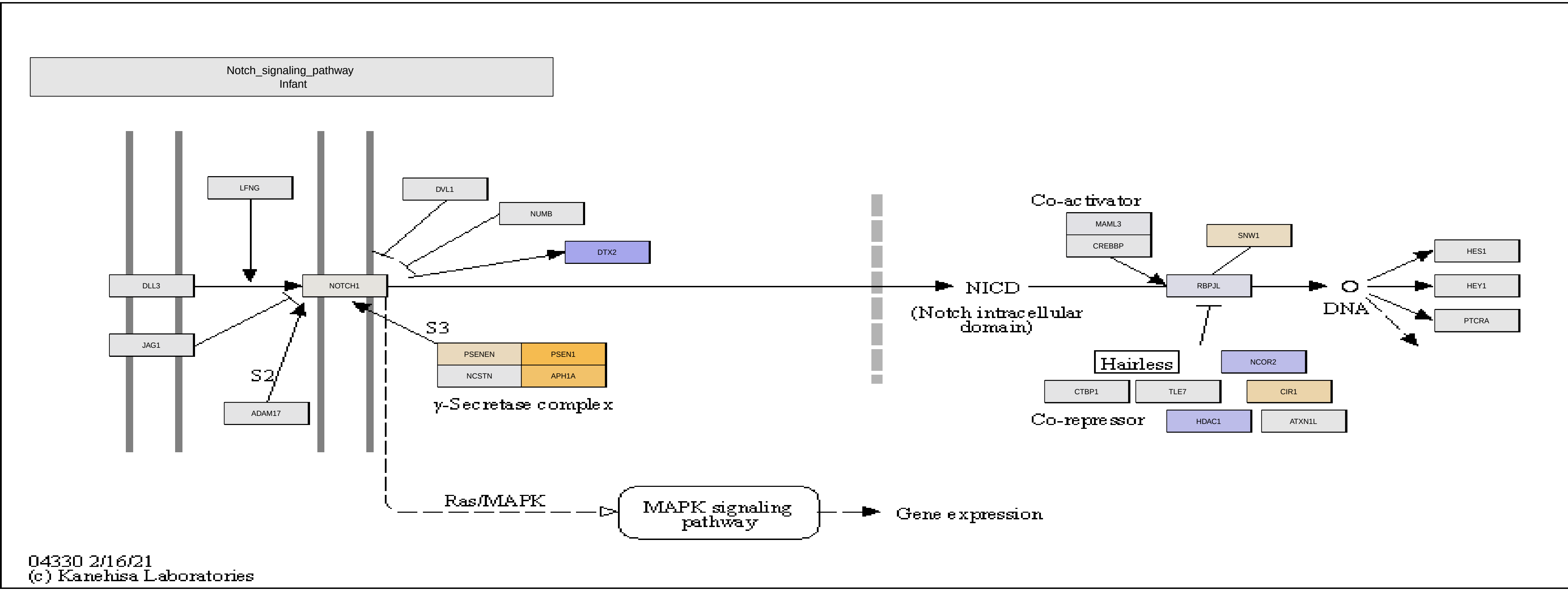
Notch_signaling_pathway
Infant
LFNG
DVL1
NUMB
DTX2
DLL3
NOTCH1
JAG1
S2
ADAM17
S3
PSENEN
PSEN1
NCSTN
APH1A
γ-Secretase complex
NICD
(Notch intracellular domain)
Co-activator
MAML3
CREBBP
SNW1
RBPJL
DNA
HES1
HEY1
PTCRA
Hairless
NCOR2
CTBP1
TLE7
CIR1
Co-repressor
HDAC1
ATXN1L
Ras/MAPK
MAPK signaling pathway
Gene expression
04330 2/16/21
(c) Kanehisa Laboratories

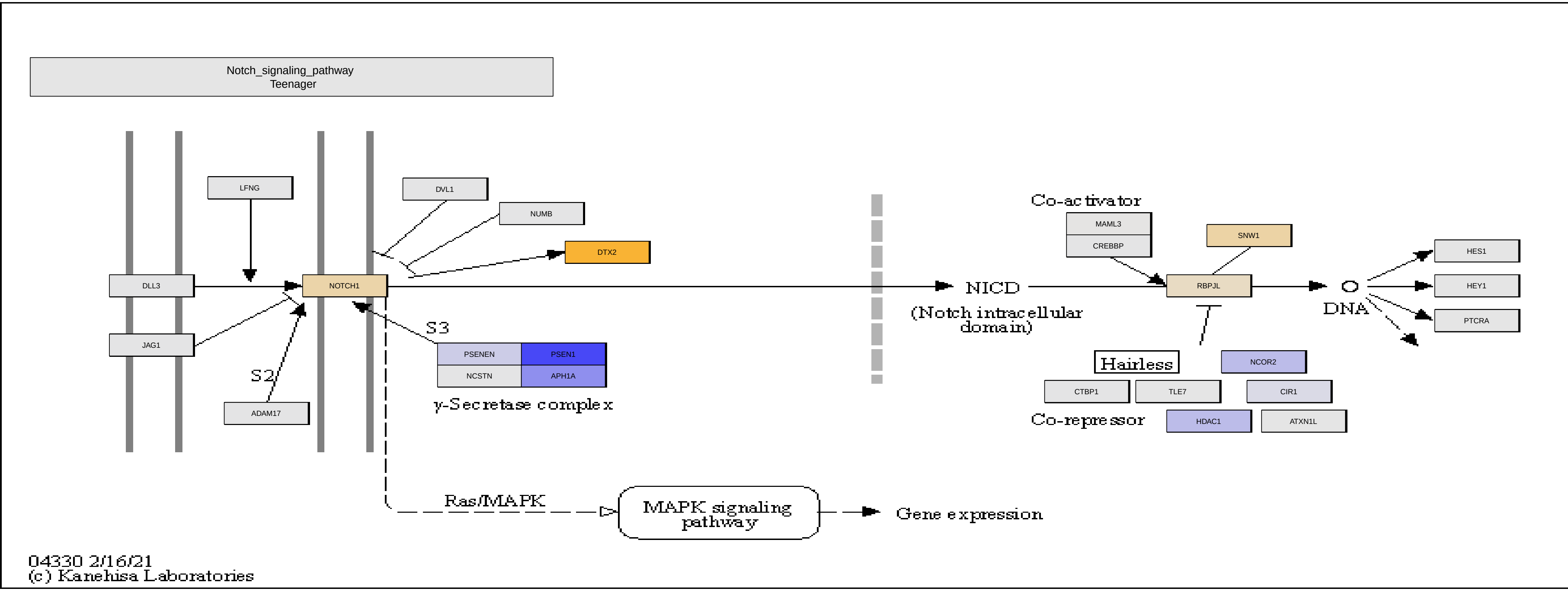

Notch_signaling_pathway
Teenager
LFNG
DVL1
NUMB
DTX2
DLL3
NOTCH1
JAG1
S2
ADAM17
S3
PSENEN
PSEN1
NCSTN
APH1A
γ-Secretase complex
NICD
(Notch intracellular domain)
Co-activator
MAML3
CREBBP
SNW1
RBPJL
DNA
HES1
HEY1
PTCRA
Hairless
NCOR2
CTBP1
TLE7
CIR1
Co-repressor
HDAC1
ATXN1L
Ras/MAPK
MAPK signaling pathway
Gene expression
04330 2/16/21
(c) Kanehisa Laboratories

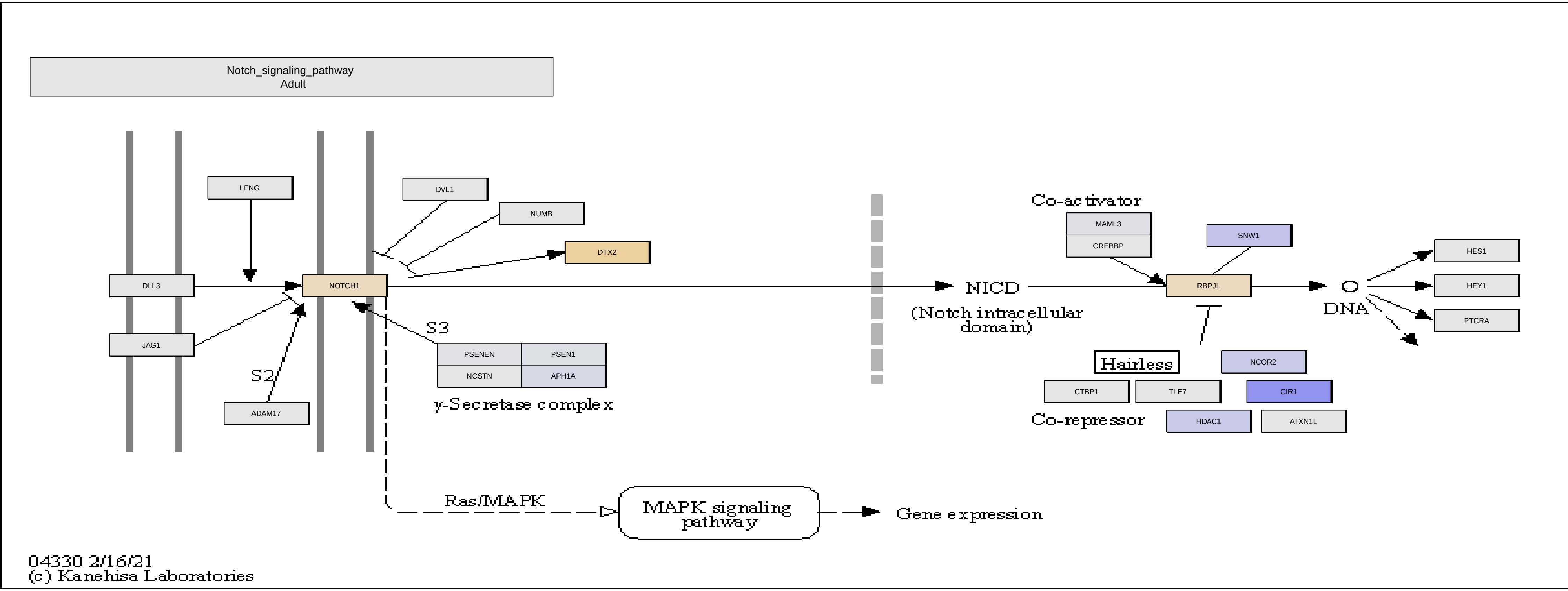
Notch_signaling_pathway
Adult
LFNG
DVL1
NUMB
DTX2
DLL3
NOTCH1
JAG1
S2
ADAM17
S3
PSENEN
PSEN1
NCSTN
APH1A
γ-Secretase complex
NICD
(Notch intracellular domain)
Co-activator
MAML3
CREBBP
SNW1
RBPJL
HES1
HEY1
PTCRA
DNA
Hairless
NCOR2
CTBP1
TLE7
CIR1
Co-repressor
HDAC1
ATXN1L
Ras/MAPK
MAPK signaling pathway
Gene expression
04330 2/16/21
(c) Kanehisa Laboratories

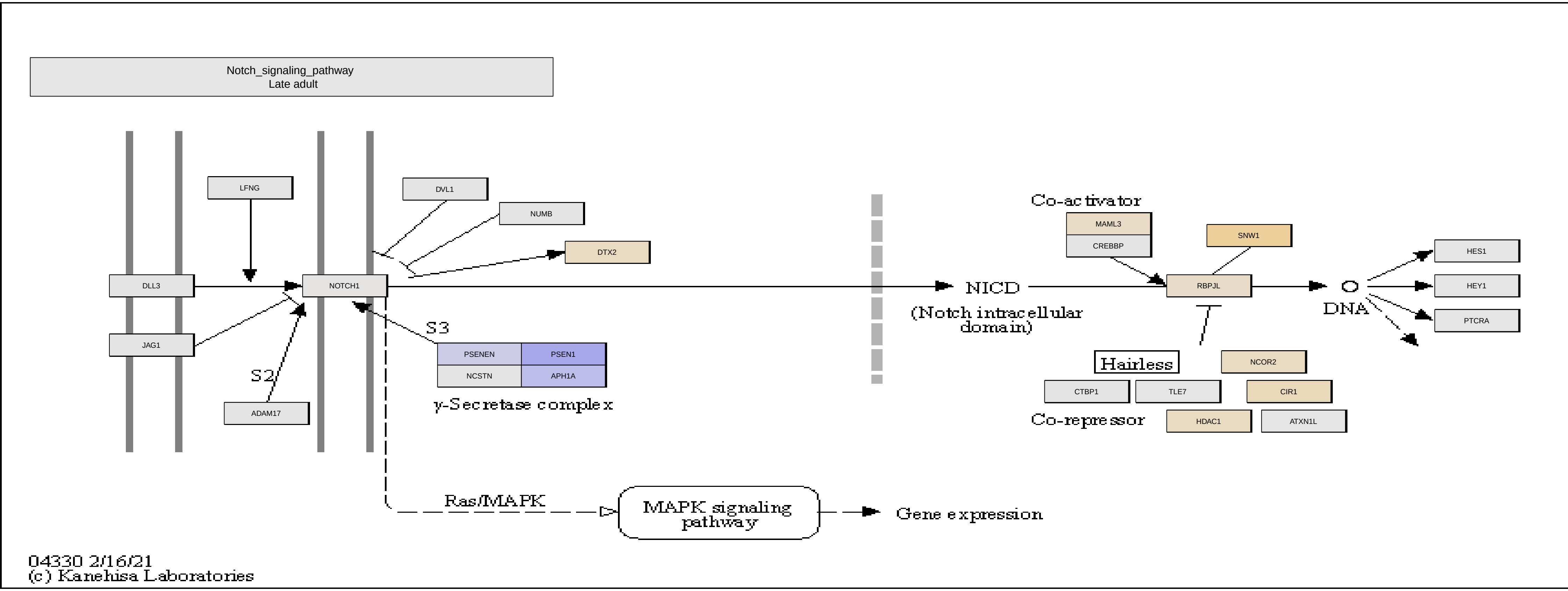
Notch_signaling_pathway
Late adult
LFNG
DVL1
NUMB
DTX2
DLL3
NOTCH1
JAG1
S2
ADAM17
S3
PSENEN
PSEN1
NCSTN
APH1A
γ-Secretase complex
NICD
(Notch intracellular domain)
Co-activator
MAML3
CREBBP
SNW1
RBPJL
DNA
HES1
HEY1
PTCRA
Hairless
NCOR2
CTBP1
TLE7
CIR1
Co-repressor
HDAC1
ATXN1L
Ras/MAPK
MAPK signaling pathway
Gene expression
04330 2/16/21
(c) Kanehisa Laboratories
