# Supplementary material for: The Transcriptome and Methylome of the Developing and Aging Brain and Their Relations to Gliomas and Psychological Disorders
Source: Cells. 2022 Jan 21;11(3):362. doi: 10.3390/cells11030362 (PMC8834030; doi:10.3390/cells11030362)
Supplement: Supplementary file 1 [file cells-11-00362-s001.zip › Suppl-Material-S4-Pathways-PSF_Methylation/p53_signaling_pathway.pdf]

p53 signaling pathway

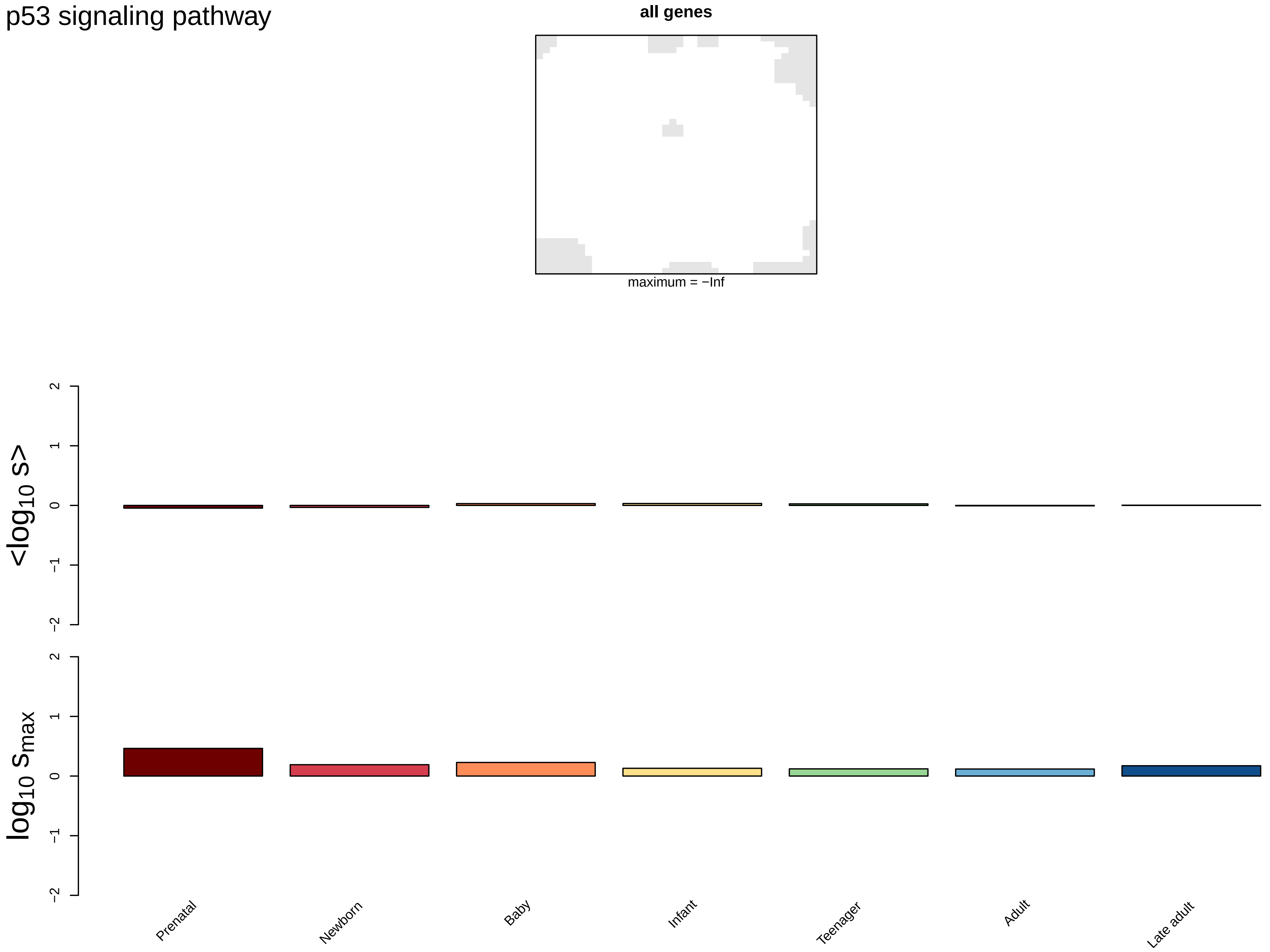

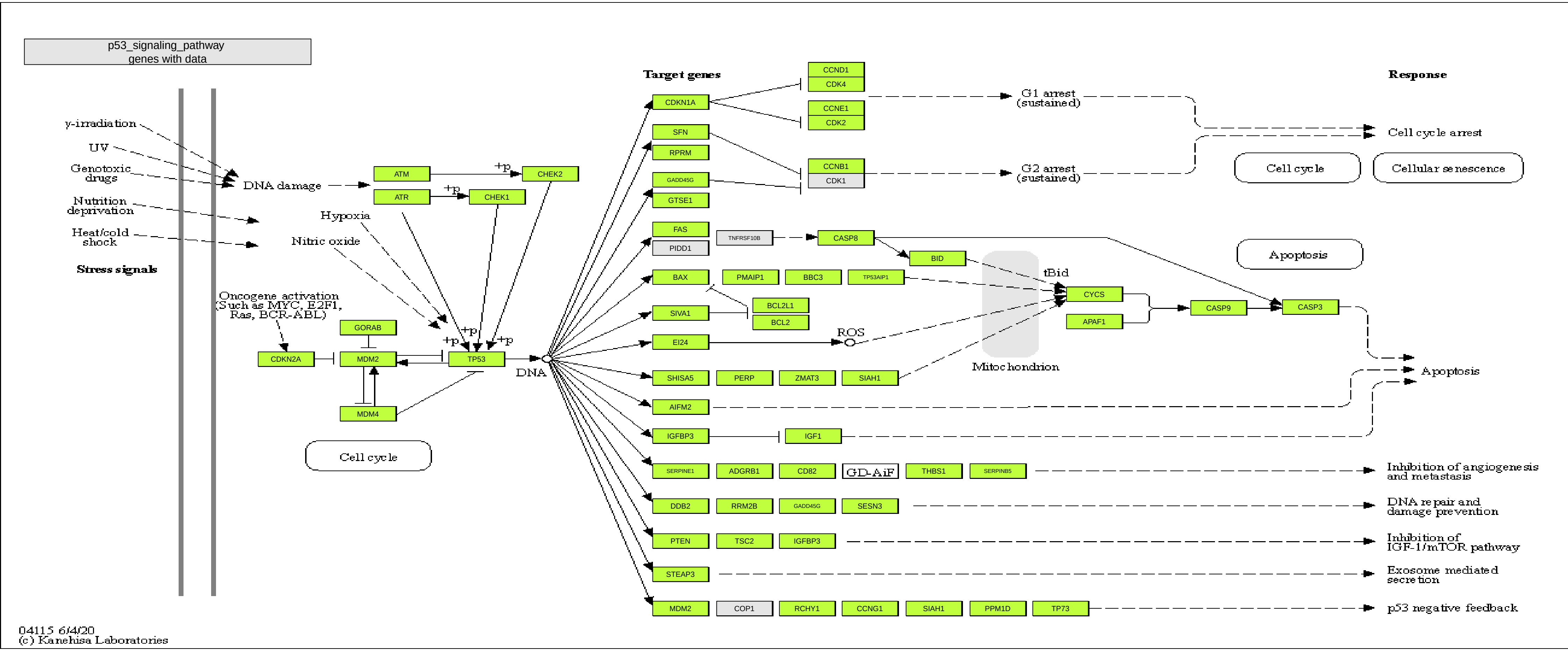
p53_signaling_pathway
genes with data
γ-irradiation
UV
Genotoxic drugs
Nutrition deprivation
Heat/cold shock
Stress signals
DNA damage
ATM
ATR
CHEK2
CHEK1
+p
Hypoxia
Nitric oxide
Oncogene activation (Such as MYC, E2F1, Ras, BCR-ABL)
GORAB
CDKN2A
MDM2
TP53
MDM4
DNA
Cell cycle
Target genes
CDKN1A
SFN
RPRM
GADD45G
GTSE1
FAS
PIDD1
TNFRSF10B
CASP8
BID
BAX
PMAIP1
BBC3
TP53AIP1
BCL2L1
BCL2
SIVA1
EI24
ROS
SHISA5
PERP
ZMAT3
SIAH1
AIFM2
IGFBP3
IGF1
SERPINE1
ADGRB1
CD82
GD-AiF
THBS1
SERPINB5
DDB2
RRM2B
GADD45G
SESN3
PTEN
TSC2
IGFBP3
STEAP3
MDM2
COP1
RCHY1
CCNG1
SIAH1
PPM1D
TP73
CCND1
CDK4
CCNE1
CDK2
CCNB1
CDK1
G1 arrest (sustained)
G2 arrest (sustained)
tBid
CYCS
APAF1
CASP9
CASP3
Mitochondrion
Response
Cell cycle arrest
Cell cycle
Cellular senescence
Apoptosis
Apoptosis
Inhibition of angiogenesis and metastasis
DNA repair and damage prevention
Inhibition of IGF-1/mTOR pathway
Exosome mediated secretion
p53 negative feedback
04115 6/4/20
(c) Kanehisa Laboratories

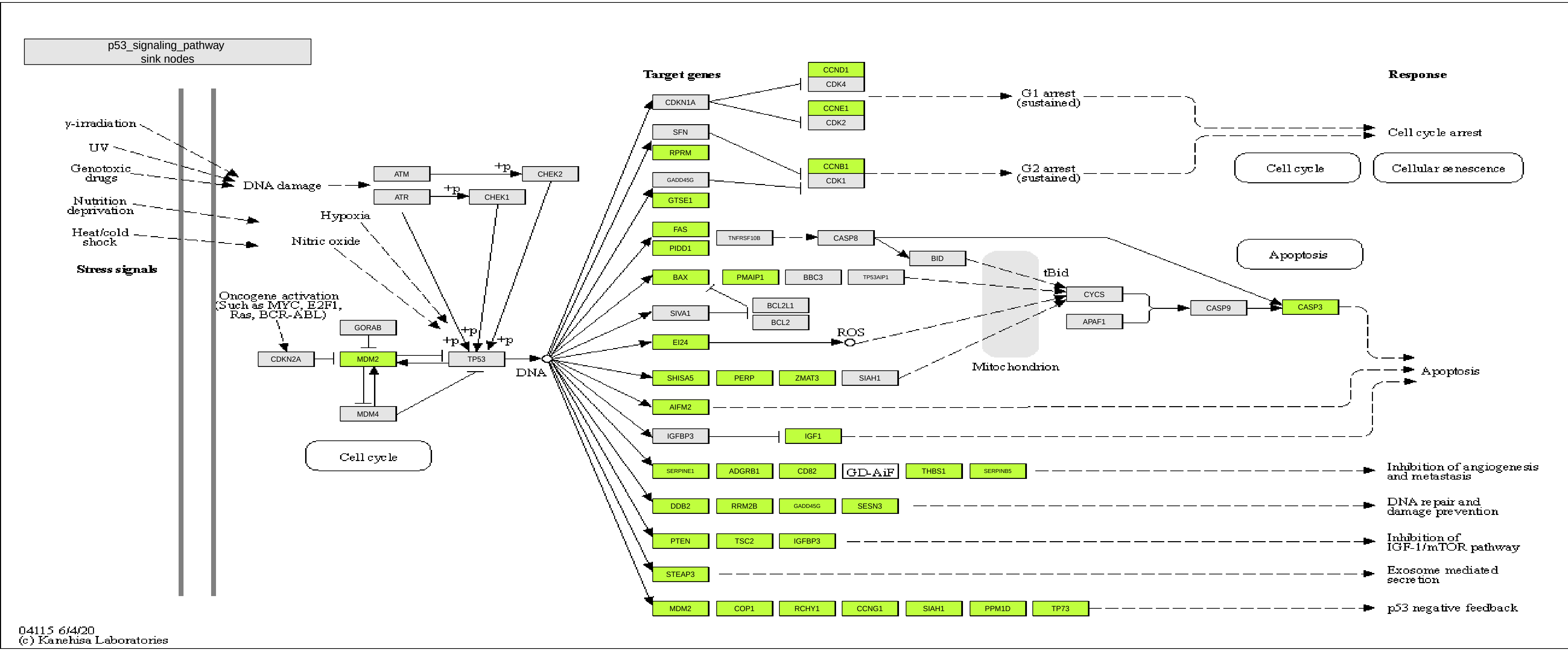
p53_signaling_pathway
sink nodes
γ-irradiation
UV
Genotoxic drugs
Nutrition deprivation
Heat/cold shock
Stress signals
DNA damage
Hypoxia
Nitric oxide
Oncogene activation (Such as MYC, E2F1, Ras, BCR-ABL)
ATM
ATR
CHEK2
CHEK1
+p
GORAB
CDKN2A
MDM2
TP53
MDM4
Cell cycle
DNA
Target genes
CDKN1A
SFN
RPRM
GADD45G
GTSE1
FAS
PIDD1
BAX
SIVA1
EI24
SHISA5
AIFM2
IGFBP3
SERPINE1
DDB2
PTEN
STEAP3
CCND1
CDK4
CCNE1
CDK2
CCNB1
CDK1
TNFRSF10B
CASP8
BID
PMAIP1
BBC3
TP53AIP1
BCL2L1
BCL2
ROS
PERP
ZMAT3
SIAH1
IGF1
ADGRB1
CD82
GD-AiF
THBS1
SERPINB5
RRM2B
GADD45G
SESN3
TSC2
IGFBP3
COP1
RCHY1
CCNG1
SIAH1
PPM1D
TP73
G1 arrest (sustained)
G2 arrest (sustained)
tBid
CYCS
APAF1
CASP9
CASP3
Mitochondrion
Response
Cell cycle arrest
Cell cycle
Cellular senescence
Apoptosis
Apoptosis
Inhibition of angiogenesis and metastasis
DNA repair and damage prevention
Inhibition of IGF-1/mTOR pathway
Exosome mediated secretion
p53 negative feedback
04115 6/4/20
(c) Kanehisa Laboratories

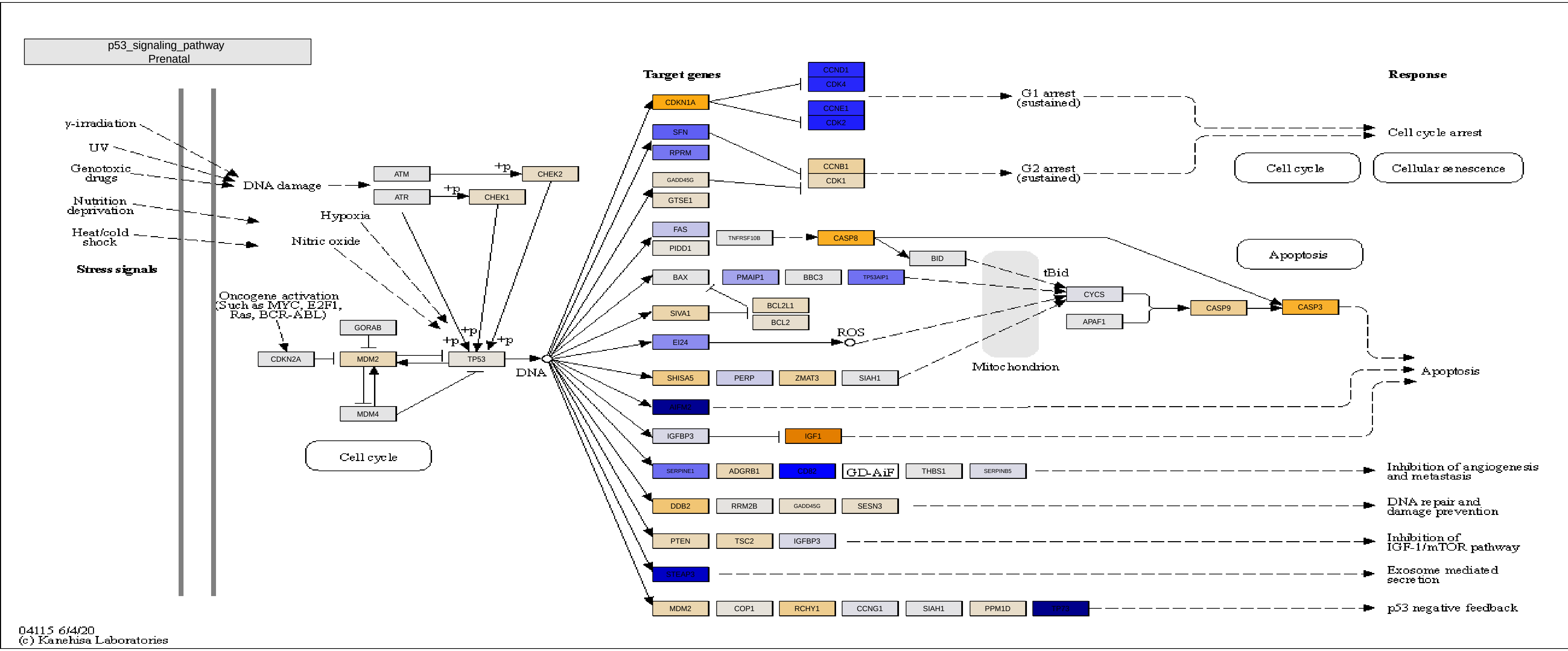

p53_signaling_pathway
Prenatal
γ-irradiation
UV
Genotoxic drugs
Nutrition deprivation
Heat/cold shock
Stress signals
DNA damage
Hypoxia
Nitric oxide
Oncogene activation (Such as MYC, E2F1, Ras, BCR-ABL)
ATM
ATR
CHEK2
CHEK1
+p
GORAB
CDKN2A
MDM2
MDM4
TP53
DNA
Cell cycle
Target genes
CDKN1A
SFN
RPRM
GADD45G
GTSE1
CCND1
CDK4
CCNE1
CDK2
CCNB1
CDK1
G1 arrest (sustained)
G2 arrest (sustained)
Response
Cell cycle arrest
Cell cycle
Cellular senescence
FAS
PIDD1
TNFRSF10B
CASP8
BID
tBid
BAX
PMAIP1
BBC3
TP53AIP1
BCL2L1
BCL2
SIVA1
EI24
ROS
CYCS
APAF1
CASP9
CASP3
Mitochondrion
Apoptosis
SHISA5
PERP
ZMAT3
SIAH1
AIFM2
IGFBP3
IGF1
SERPINE1
ADGRB1
CD82
GD-AiF
THBS1
SERPINB5
Inhibition of angiogenesis and metastasis
DDB2
RRM2B
GADD45G
SESN3
DNA repair and damage prevention
PTEN
TSC2
IGFBP3
Inhibition of IGF-1/mTOR pathway
STEAP3
Exosome mediated secretion
MDM2
COP1
RCHY1
CCNG1
SIAH1
PPM1D
TP73
p53 negative feedback
04115 6/4/20
(c) Kanehisa Laboratories

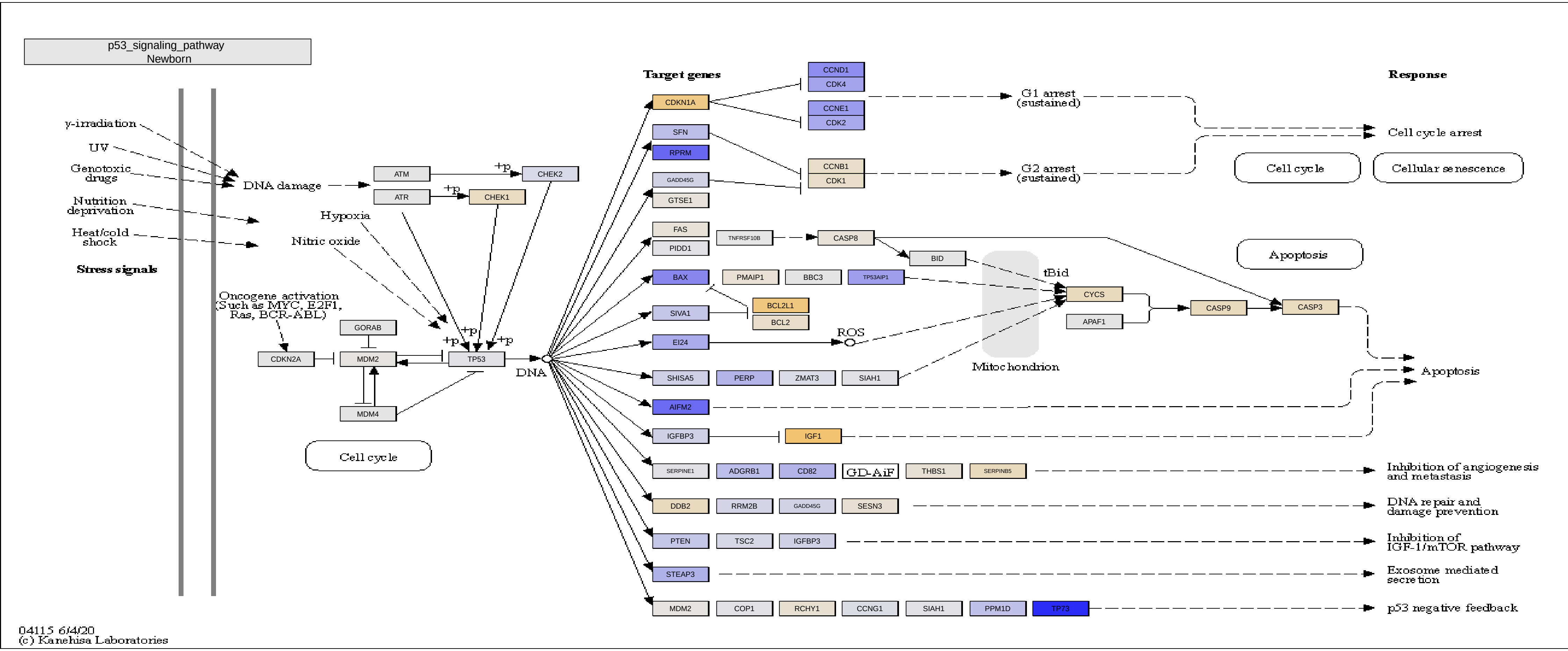
p53_signaling_pathway
Newborn
Target genes
Response
γ-irradiation
UV
Genotoxic drugs
Nutrition deprivation
Heat/cold shock
Stress signals
DNA damage
Hypoxia
Nitric oxide
Oncogene activation
(Such as MYC, E2F1, Ras, BCR-ABL)
ATM
ATR
CHEK2
CHEK1
+p
GORAB
CDKN2A
MDM2
MDM4
TP53
DNA
Cell cycle
CDKN1A
SFN
RPRM
GADD45G
GTSE1
CCND1
CDK4
CCNE1
CDK2
CCNB1
CDK1
G1 arrest (sustained)
G2 arrest (sustained)
Cell cycle arrest
Cell cycle
Cellular senescence
FAS
PIDD1
TNFRSF10B
CASP8
BID
BAX
PMAIP1
BBC3
TP53AIP1
BCL2L1
BCL2
SIVA1
EI24
ROS
tBid
CYCS
APAF1
CASP9
CASP3
Apoptosis
Mitochondrion
SHISA5
PERP
ZMAT3
SIAH1
AIFM2
IGFBP3
IGF1
Apoptosis
SERPINE1
ADGRB1
CD82
GD-AiF
THBS1
SERPINB5
Inhibition of angiogenesis and metastasis
DDB2
RRM2B
GADD45G
SESN3
DNA repair and damage prevention
PTEN
TSC2
IGFBP3
Inhibition of IGF-1/mTOR pathway
STEAP3
Exosome mediated secretion
MDM2
COP1
RCHY1
CCNG1
SIAH1
PPM1D
TP73
p53 negative feedback
04115 6/4/20
(c) Kanehisa Laboratories

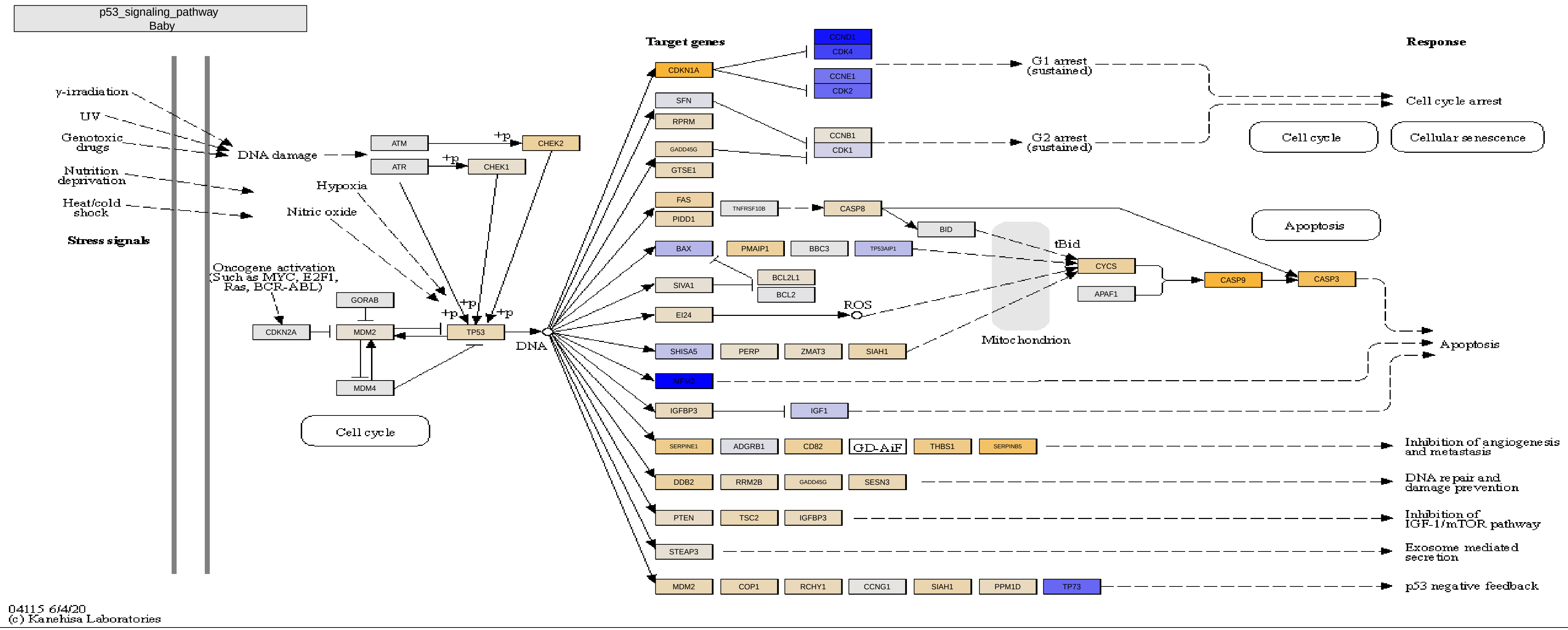
p53_signaling_pathway
Baby
Target genes
Response
γ-irradiation
UV
Genotoxic drugs
Nutrition deprivation
Heat/cold shock
Stress signals
DNA damage
Hypoxia
Nitric oxide
ATM
ATR
CHEK2
CHEK1
+p
Oncogene activation (Such as MYC, E2F1, Ras, BCR-ABL)
GORAB
CDKN2A
MDM2
MDM4
TP53
DNA
Cell cycle
CDKN1A
SFN
RPRM
GADD45G
GTSE1
CCND1
CDK4
CCNE1
CDK2
CCNB1
CDK1
G1 arrest (sustained)
G2 arrest (sustained)
Cell cycle arrest
Cell cycle
Cellular senescence
FAS
PIDD1
TNFRSF10B
CASP8
BID
tBid
BAX
PMAIP1
BBC3
TP53AIP1
CYCS
APAF1
CASP9
CASP3
Apoptosis
SIVA1
BCL2L1
BCL2
EI24
ROS
Mitochondrion
SHISA5
PERP
ZMAT3
SIAH1
AIFM2
IGFBP3
IGF1
Apoptosis
SERPINE1
ADGRB1
CD82
GD-AiF
THBS1
SERPINB5
Inhibition of angiogenesis and metastasis
DDB2
RRM2B
GADD45G
SESN3
DNA repair and damage prevention
PTEN
TSC2
IGFBP3
Inhibition of IGF-1/mTOR pathway
STEAP3
Exosome mediated secretion
MDM2
COP1
RCHY1
CCNG1
SIAH1
PPM1D
TP73
p53 negative feedback
04115 6/4/20
(c) Kanehisa Laboratories

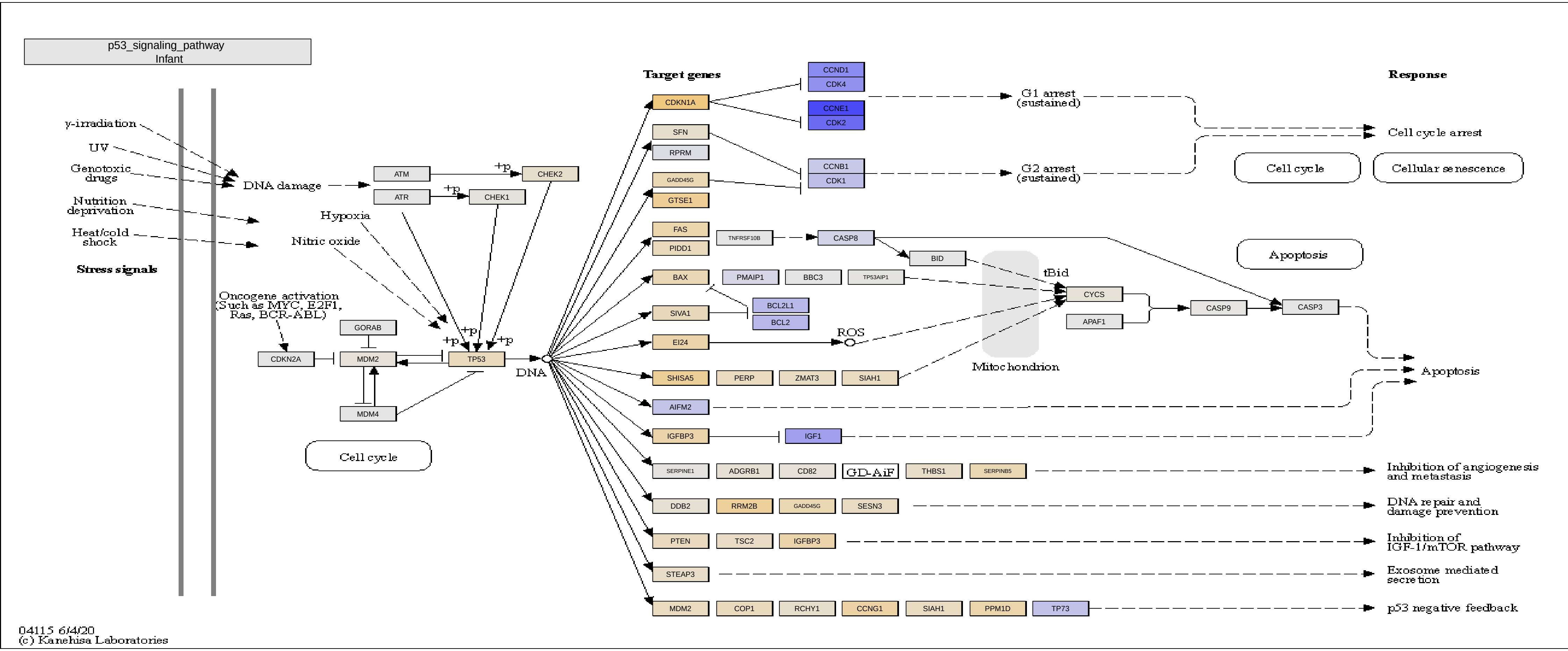
p53_signaling_pathway
Infant
γ-irradiation
UV
Genotoxic drugs
Nutrition deprivation
Heat/cold shock
Stress signals
DNA damage
ATM
ATR
CHEK2
CHEK1
+p
Hypoxia
Nitric oxide
Oncogene activation (Such as MYC, E2F1, Ras, BCR-ABL)
GORAB
CDKN2A
MDM2
TP53
MDM4
DNA
Cell cycle
Target genes
CDKN1A
SFN
RPRM
GADD45G
GTSE1
FAS
PIDD1
BAX
SIVA1
EI24
SHISA5
PERP
ZMAT3
SIAH1
AIFM2
IGFBP3
SERPINE1
ADGRB1
CD82
GD-AiF
THBS1
SERPINB5
DDB2
RRM2B
GADD45G
SESN3
PTEN
TSC2
IGFBP3
STEAP3
MDM2
COP1
RCHY1
CCNG1
SIAH1
PPM1D
TP73
CCND1
CDK4
CCNE1
CDK2
CCNB1
CDK1
TNFRSF10B
CASP8
BID
PMAIP1
BBC3
TP53AIP1
BCL2L1
BCL2
IGF1
ROS
tBid
CYCS
APAF1
CASP9
CASP3
Mitochondrion
G1 arrest (sustained)
G2 arrest (sustained)
Response
Cell cycle arrest
Cell cycle
Cellular senescence
Apoptosis
Apoptosis
Inhibition of angiogenesis and metastasis
DNA repair and damage prevention
Inhibition of IGF-1/mTOR pathway
Exosome mediated secretion
p53 negative feedback
04115 6/4/20
(c) Kanehisa Laboratories

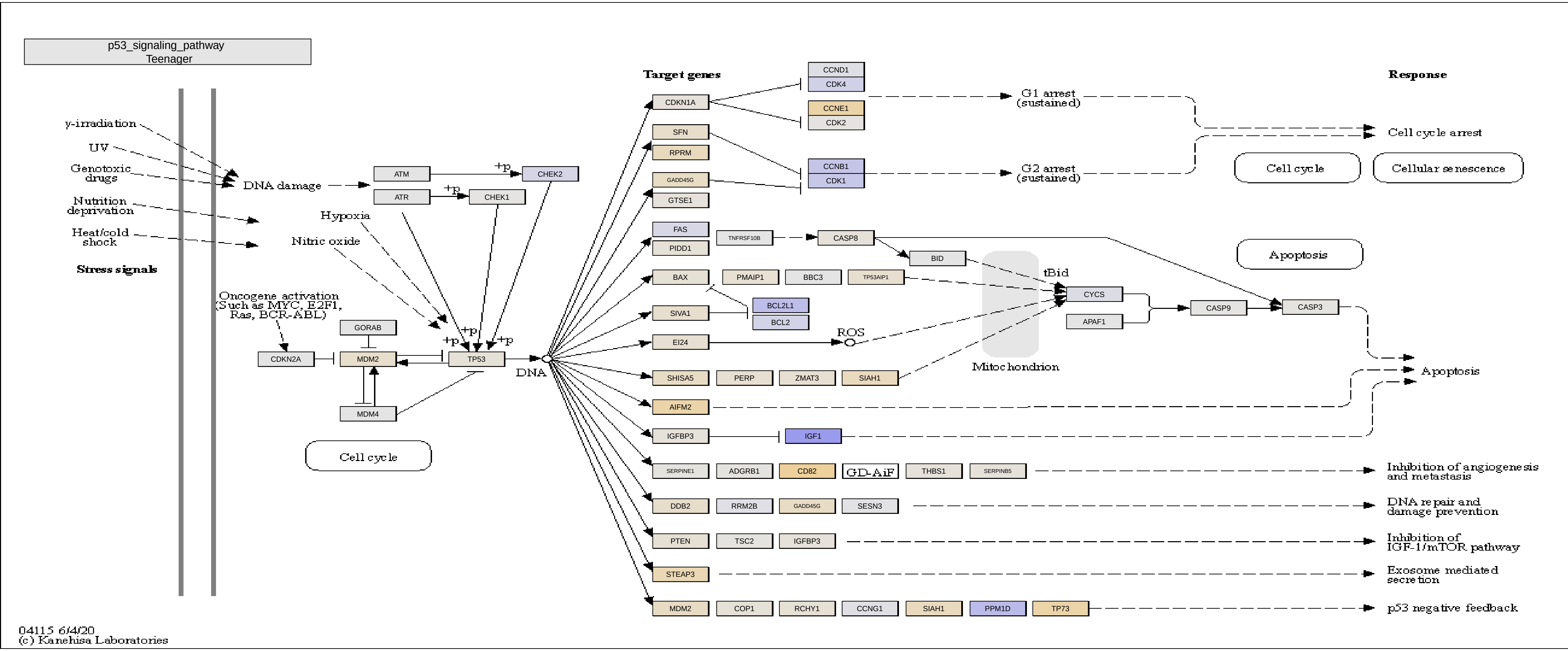
p53_signaling_pathway
Teenager
Stress signals
γ-irradiation
UV
Genotoxic drugs
Nutrition deprivation
Heat/cold shock
DNA damage
ATM
ATR
CHEK2
CHEK1
+p
Hypoxia
Nitric oxide
Oncogene activation (Such as MYC, E2F1, Ras, BCR-ABL)
CDKN2A
GORAB
MDM2
MDM4
TP53
DNA
Cell cycle
Target genes
CDKN1A
SFN
RPRM
GADD45G
GTSE1
FAS
PIDD1
BAX
SIVA1
EI24
SHISA5
AIFM2
IGFBP3
SERPINE1
DDB2
PTEN
STEAP3
MDM2
CCND1
CDK4
CCNE1
CDK2
CCNB1
CDK1
G1 arrest (sustained)
G2 arrest (sustained)
Response
Cell cycle arrest
Cell cycle
Cellular senescence
TNFRSF10B
CASP8
BID
tBid
PMAIP1
BBC3
TP53AIP1
BCL2L1
BCL2
ROS
CYCS
APAF1
CASP9
CASP3
Mitochondrion
Apoptosis
PERP
ZMAT3
SIAH1
IGF1
ADGRB1
CD82
GD-AiF
THBS1
SERPINB5
Inhibition of angiogenesis and metastasis
RRM2B
GADD45G
SESN3
DNA repair and damage prevention
TSC2
IGFBP3
Inhibition of IGF-1/mTOR pathway
Exosome mediated secretion
COP1
RCHY1
CCNG1
SIAH1
PPM1D
TP73
p53 negative feedback
04115 6/4/20
(c) Kanehisa Laboratories

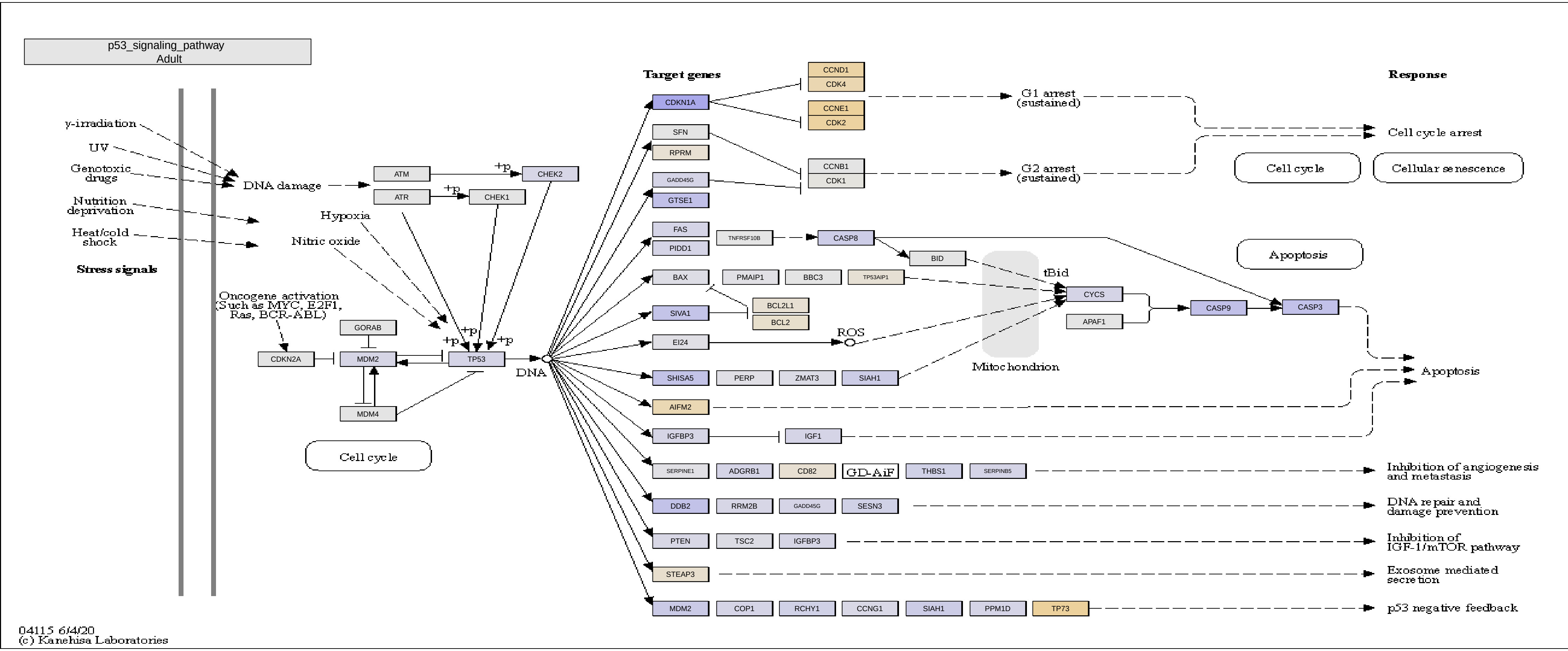

p53_signaling_pathway
Adult
γ-irradiation
UV
Genotoxic drugs
Nutrition deprivation
Heat/cold shock
Stress signals
DNA damage
ATM
ATR
CHEK2
CHEK1
+p
Hypoxia
Nitric oxide
Oncogene activation (Such as MYC, E2F1, Ras, BCR-ABL)
CDKN2A
GORAB
MDM2
MDM4
TP53
DNA
Cell cycle
Target genes
CDKN1A
SFN
RPRM
GADD45G
GTSE1
FAS
PIDD1
TNFRSF10B
CASP8
BID
BAX
PMAIP1
BBC3
TP53AIP1
BCL2L1
BCL2
SIVA1
EI24
ROS
tBid
Mitochondrion
CYCS
APAF1
CASP9
CASP3
SHISA5
PERP
ZMAT3
SIAH1
AIFM2
IGFBP3
IGF1
SERPINE1
ADGRB1
CD82
GD-AiF
THBS1
SERPINB5
DDB2
RRM2B
GADD45G
SESN3
PTEN
TSC2
IGFBP3
STEAP3
MDM2
COP1
RCHY1
CCNG1
SIAH1
PPM1D
TP73
CCND1
CDK4
CCNE1
CDK2
CCNB1
CDK1
G1 arrest (sustained)
G2 arrest (sustained)
Response
Cell cycle arrest
Cell cycle
Cellular senescence
Apoptosis
Apoptosis
Inhibition of angiogenesis and metastasis
DNA repair and damage prevention
Inhibition of IGF-1/mTOR pathway
Exosome mediated secretion
p53 negative feedback
04115 6/4/20
(c) Kanehisa Laboratories

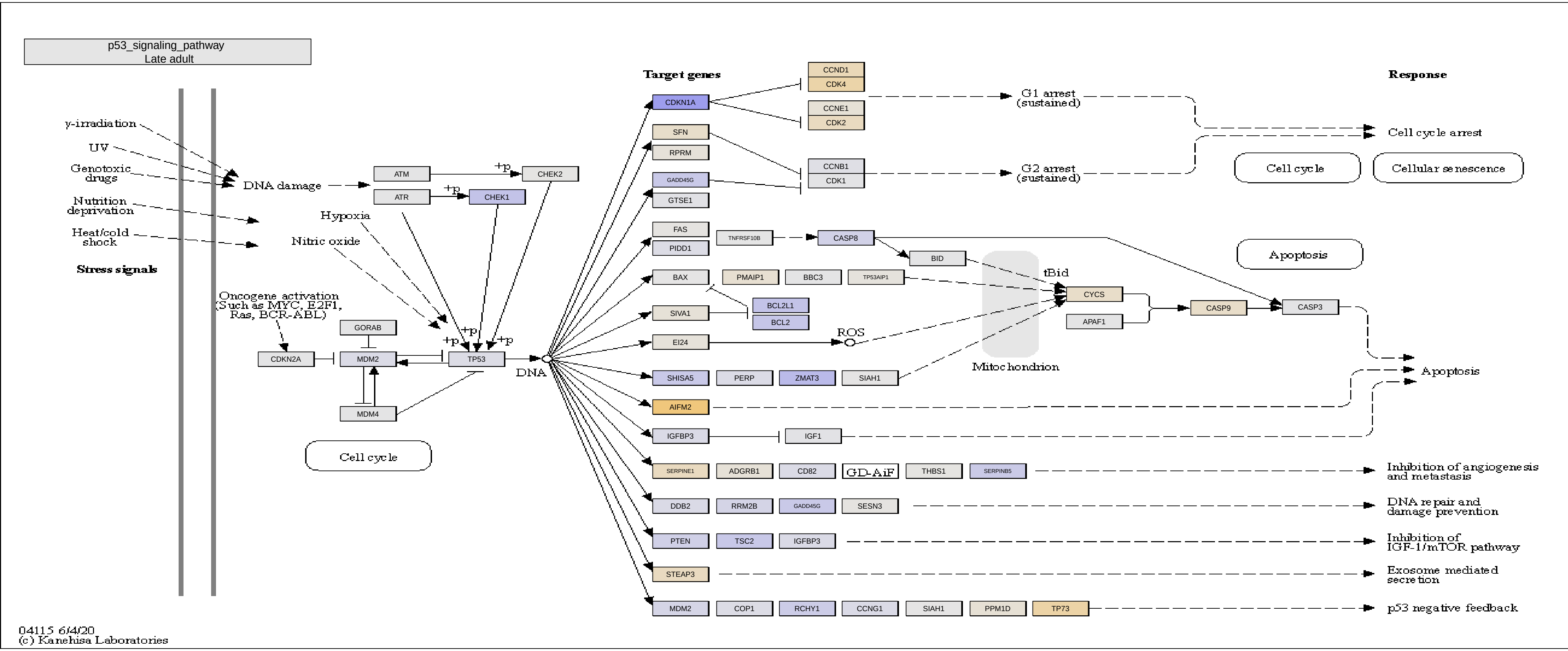
p53_signaling_pathway
Late adult
Stress signals
γ-irradiation
UV
Genotoxic drugs
Nutrition deprivation
Heat/cold shock
DNA damage
Hypoxia
Nitric oxide
Oncogene activation (Such as MYC, E2F1, Ras, BCR-ABL)
ATM
ATR
CHEK2
CHEK1
+p
GORAB
CDKN2A
MDM2
MDM4
TP53
DNA
Cell cycle
Target genes
CDKN1A
SFN
RPRM
GADD45G
GTSE1
FAS
PIDD1
TNFRSF10B
CASP8
BID
BAX
PMAIP1
BBC3
TP53AIP1
SIVA1
BCL2L1
BCL2
EI24
ROS
SHISA5
PERP
ZMAT3
SIAH1
AIFM2
IGFBP3
IGF1
SERPINE1
ADGRB1
CD82
GD-AiF
THBS1
SERPINB5
DDB2
RRM2B
GADD45G
SESN3
PTEN
TSC2
IGFBP3
STEAP3
MDM2
COP1
RCHY1
CCNG1
SIAH1
PPM1D
TP73
CCND1
CDK4
CCNE1
CDK2
CCNB1
CDK1
G1 arrest (sustained)
G2 arrest (sustained)
tBid
CYCS
APAF1
CASP9
CASP3
Mitochondrion
Response
Cell cycle arrest
Cell cycle
Cellular senescence
Apoptosis
Apoptosis
Inhibition of angiogenesis and metastasis
DNA repair and damage prevention
Inhibition of IGF-1/mTOR pathway
Exosome mediated secretion
p53 negative feedback
04115 6/4/20
(c) Kanehisa Laboratories
