# Supplementary material for: The Transcriptome and Methylome of the Developing and Aging Brain and Their Relations to Gliomas and Psychological Disorders
Source: Cells. 2022 Jan 21;11(3):362. doi: 10.3390/cells11030362 (PMC8834030; doi:10.3390/cells11030362)
Supplement: Supplementary file 1 [file cells-11-00362-s001.zip › Suppl-Material-S4-Pathways-PSF_Methylation/Phosphatidylinositol_signaling_system.pdf]

Phosphatidylinositol signaling systen

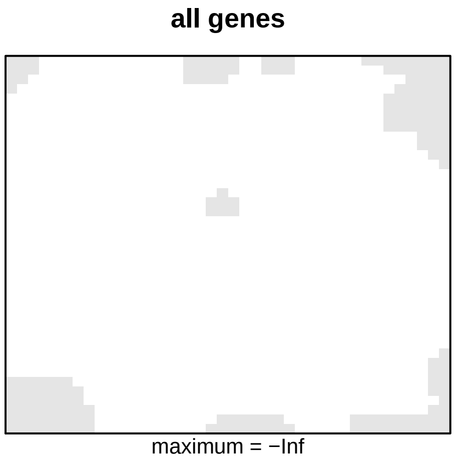

Phosphatidylinositol_signaling_system
genes with data

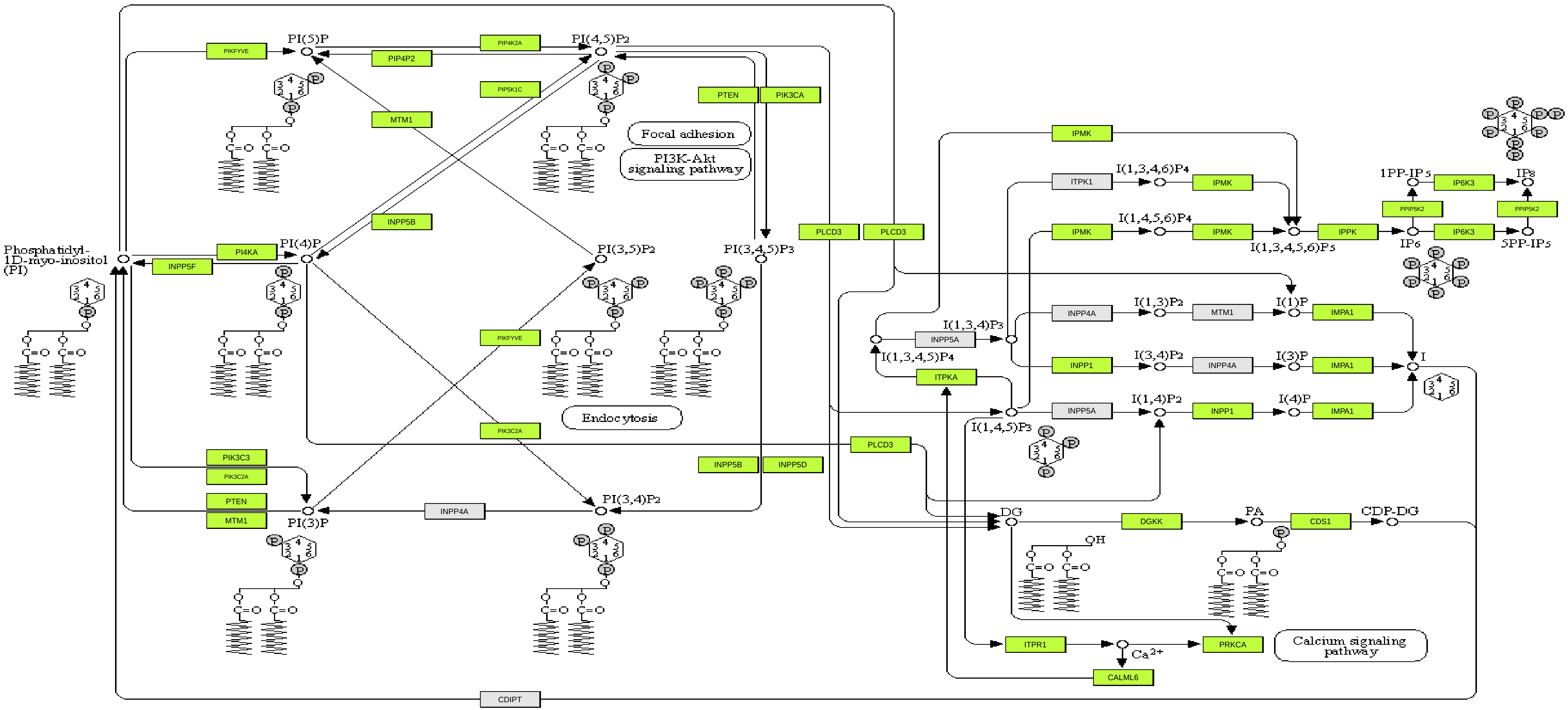

04070 7/7/21
(c) Kanehisa Laboratories

Phosphatidylinositol_signaling_system
sink nodes

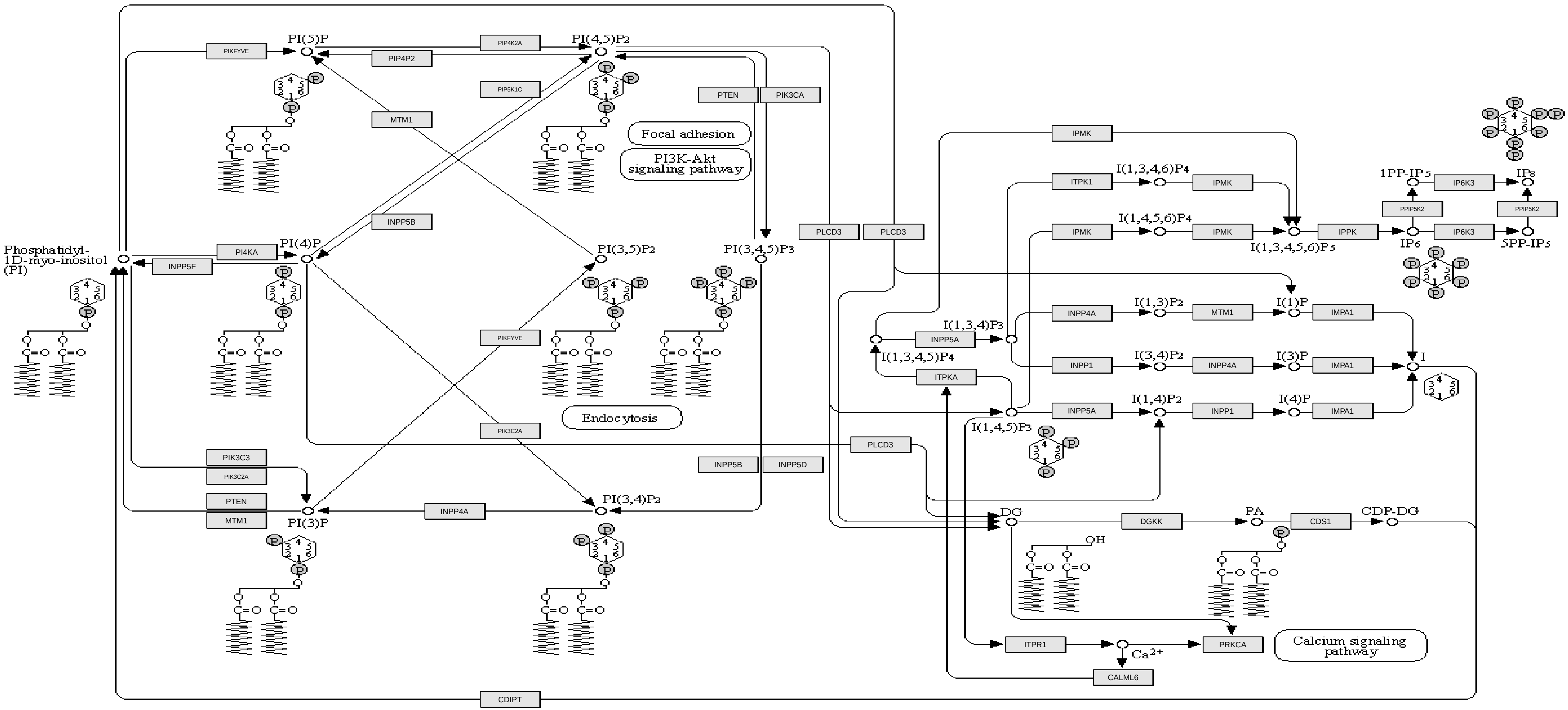

04070 7/7/21
(c) Kanehisa Laboratories

Phosphatidylinositol_signaling_system
Prenatal

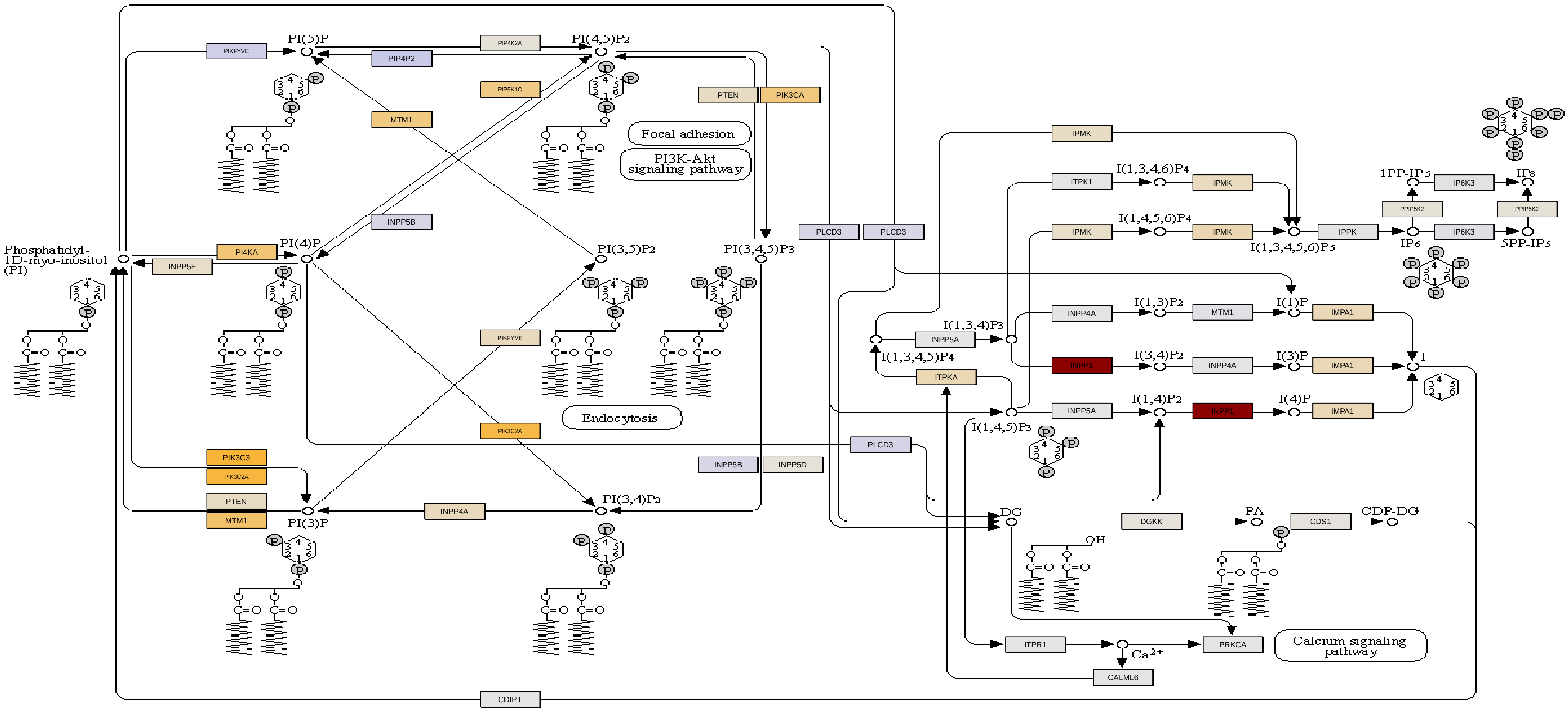

04070 7/7/21
(c) Kanehisa Laboratories

Phosphatidylinositol_signaling_system
Newborn

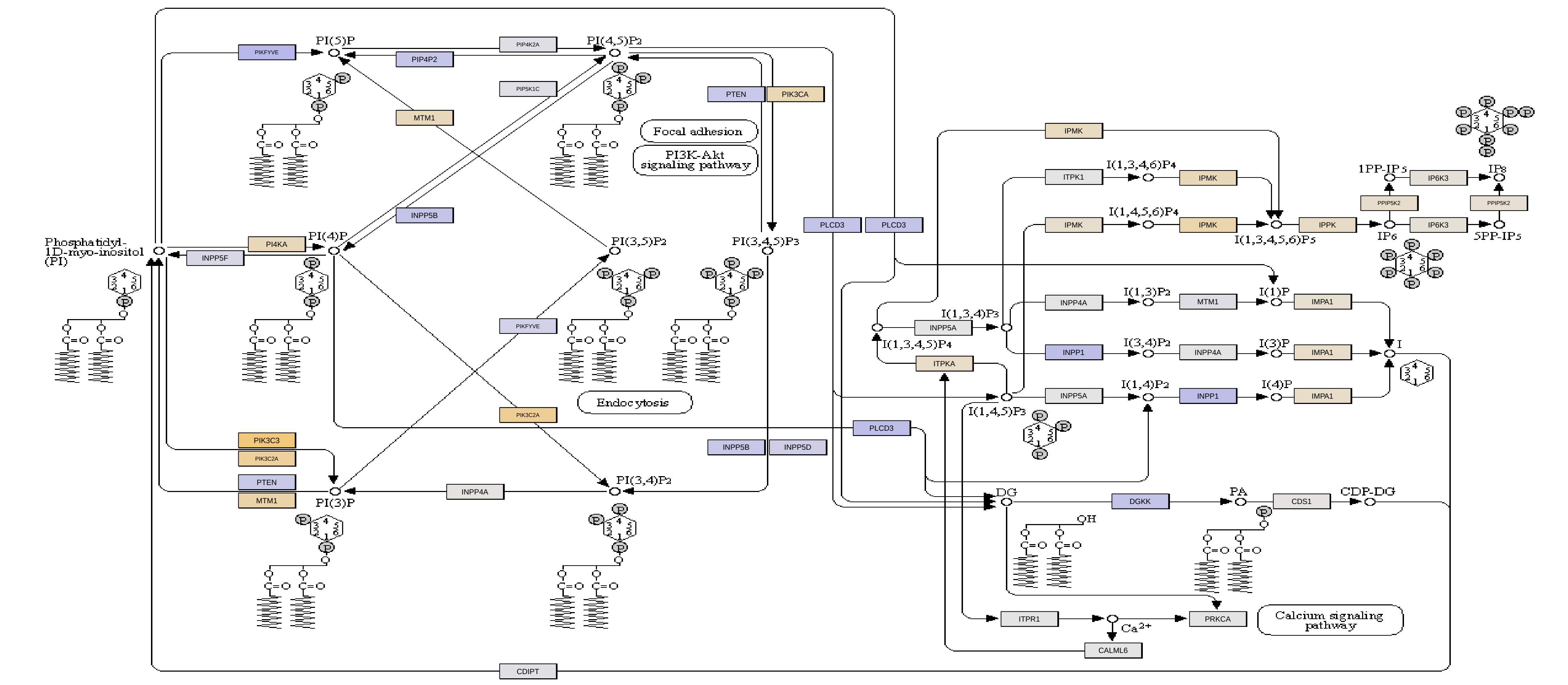

04070 7/7/21
(c) Kanehisa Laboratories

Phosphatidylinositol_signaling_system
Baby

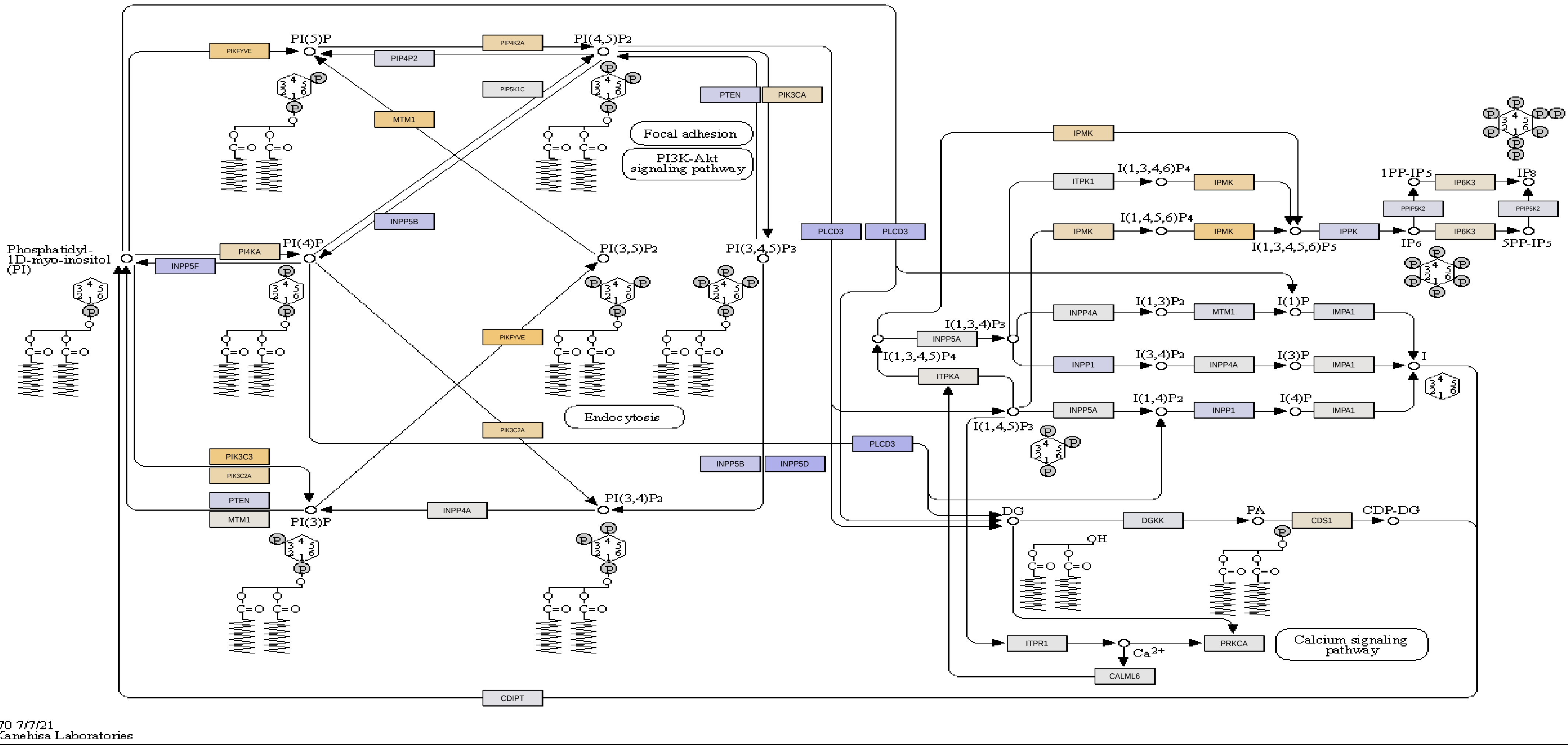

Phosphatidylinositol_signaling_system
Infant

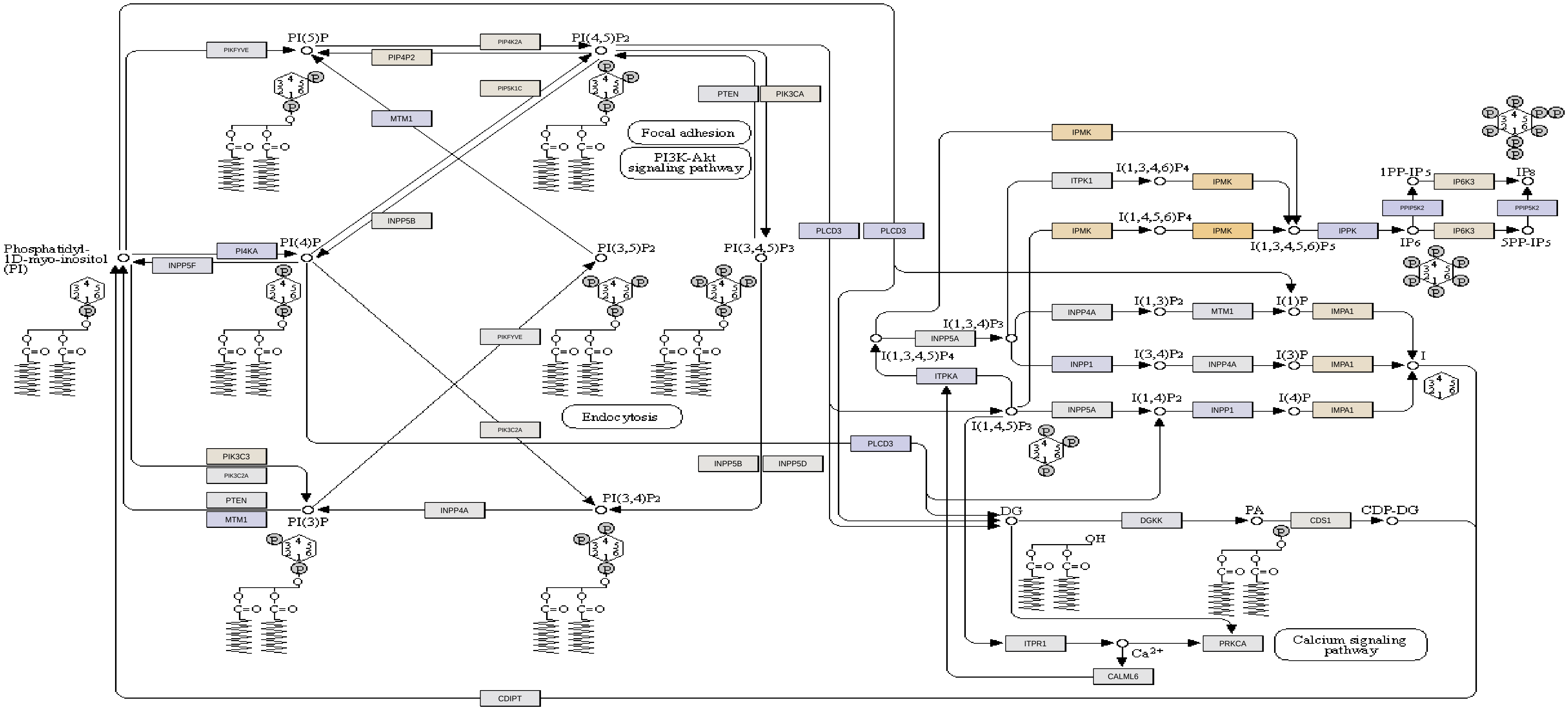

04070 7/7/21
(c) Kanehisa Laboratories

Phosphatidylinositol_signaling_system
Teenager

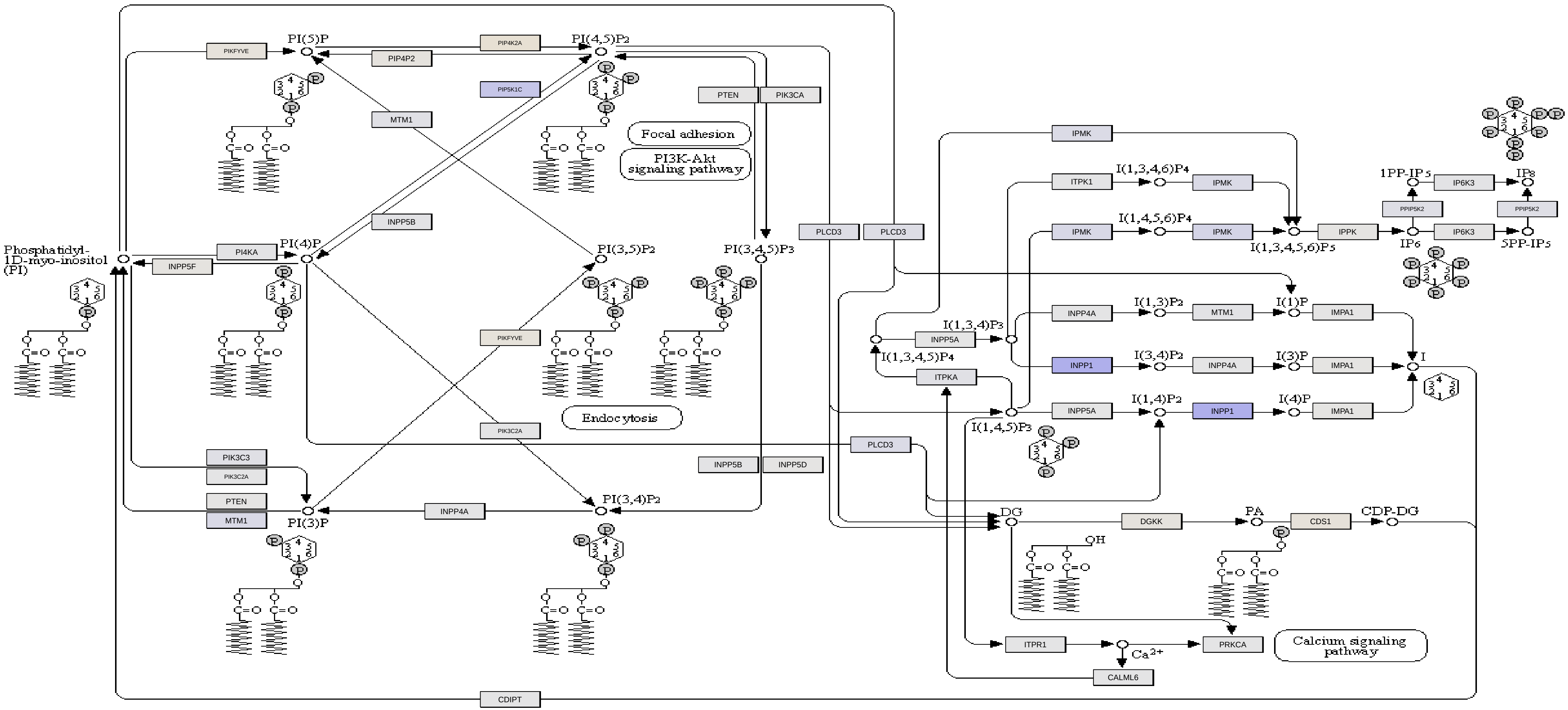

04070 7/7/21
(c) Kanehisa Laboratories

Phosphatidylinositol_signaling_system
Adult

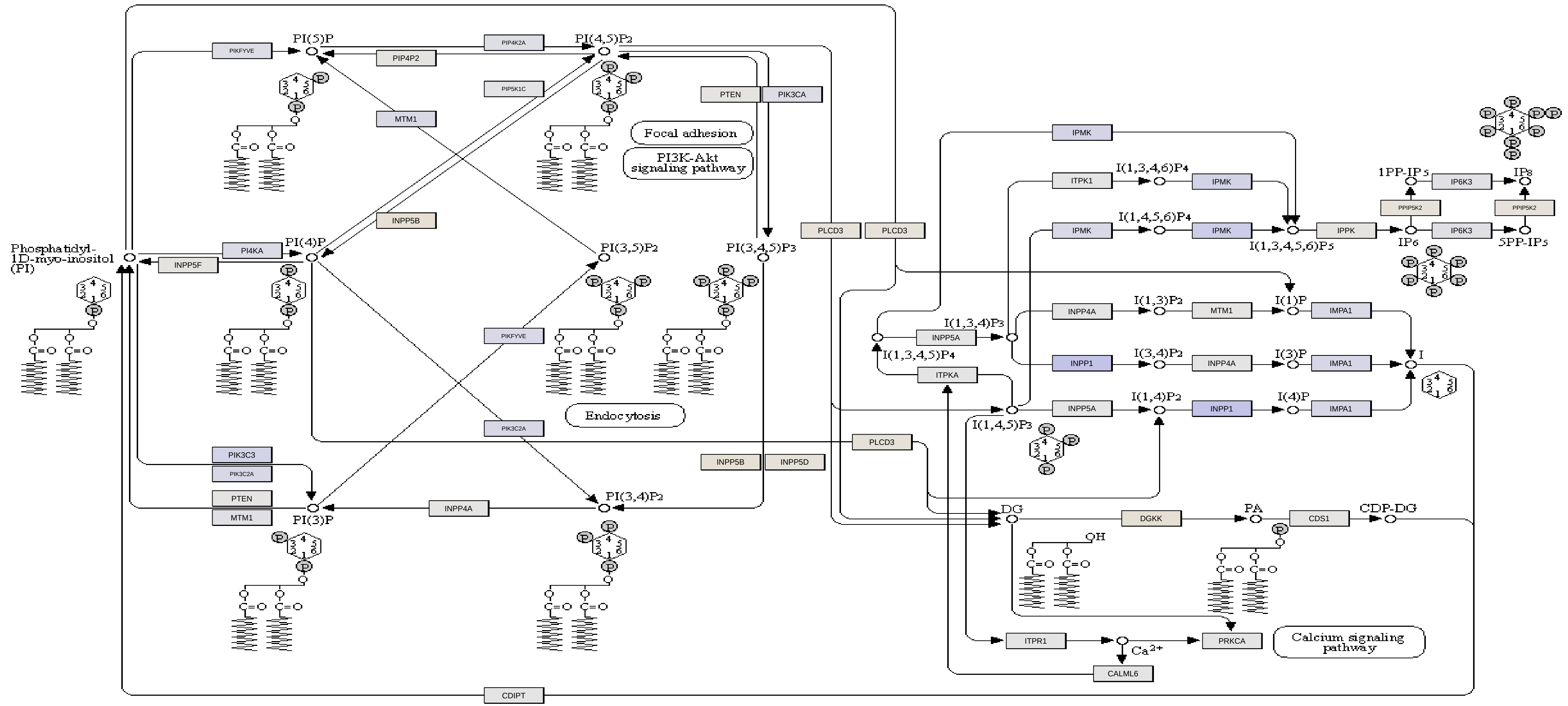

04070 7/7/21
(c) Kanehisa Laboratories

Phosphatidylinositol_signaling_system
Late adult

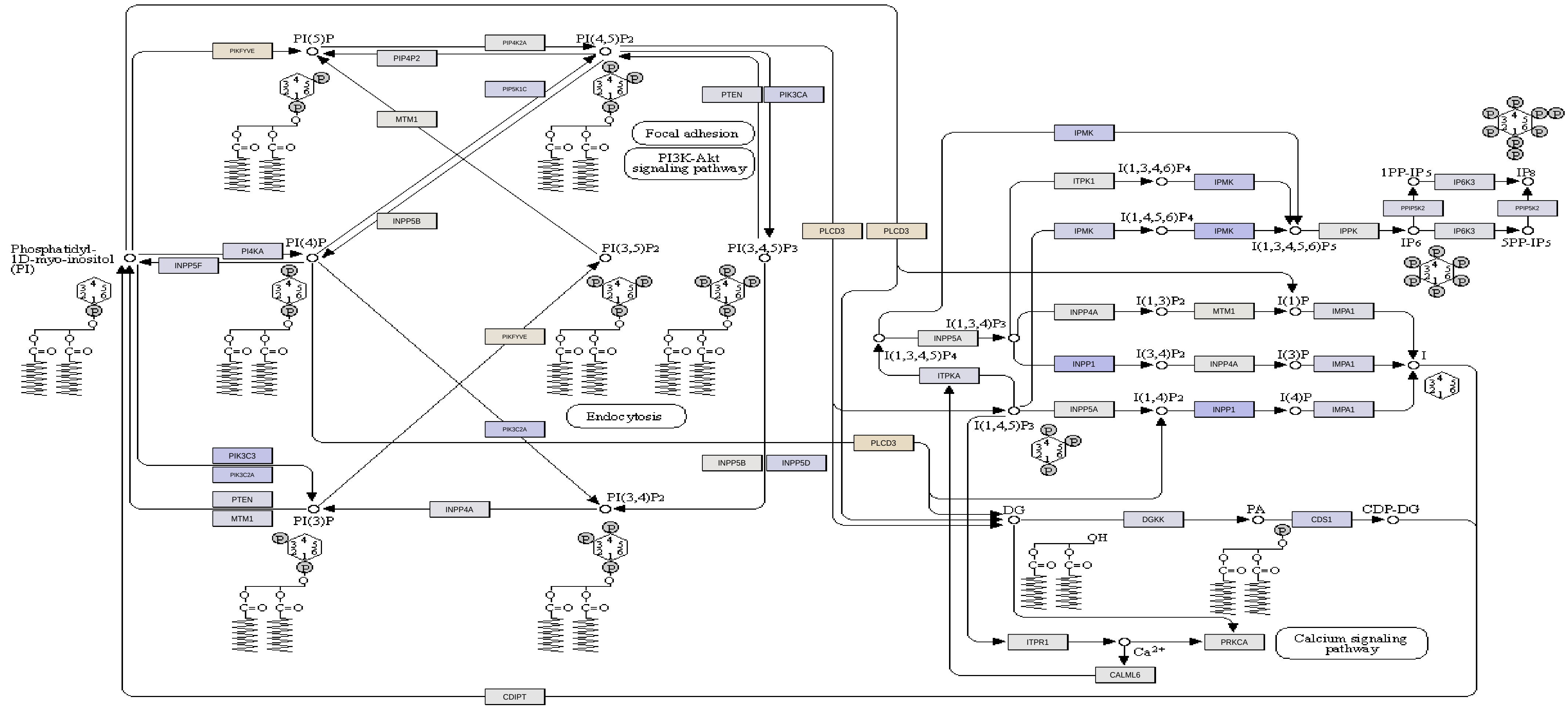

04070 7/7/21
(c) Kanehisa Laboratories
