# Supplementary material for: The Transcriptome and Methylome of the Developing and Aging Brain and Their Relations to Gliomas and Psychological Disorders
Source: Cells. 2022 Jan 21;11(3):362. doi: 10.3390/cells11030362 (PMC8834030; doi:10.3390/cells11030362)

Phospholipase D signaling pathway

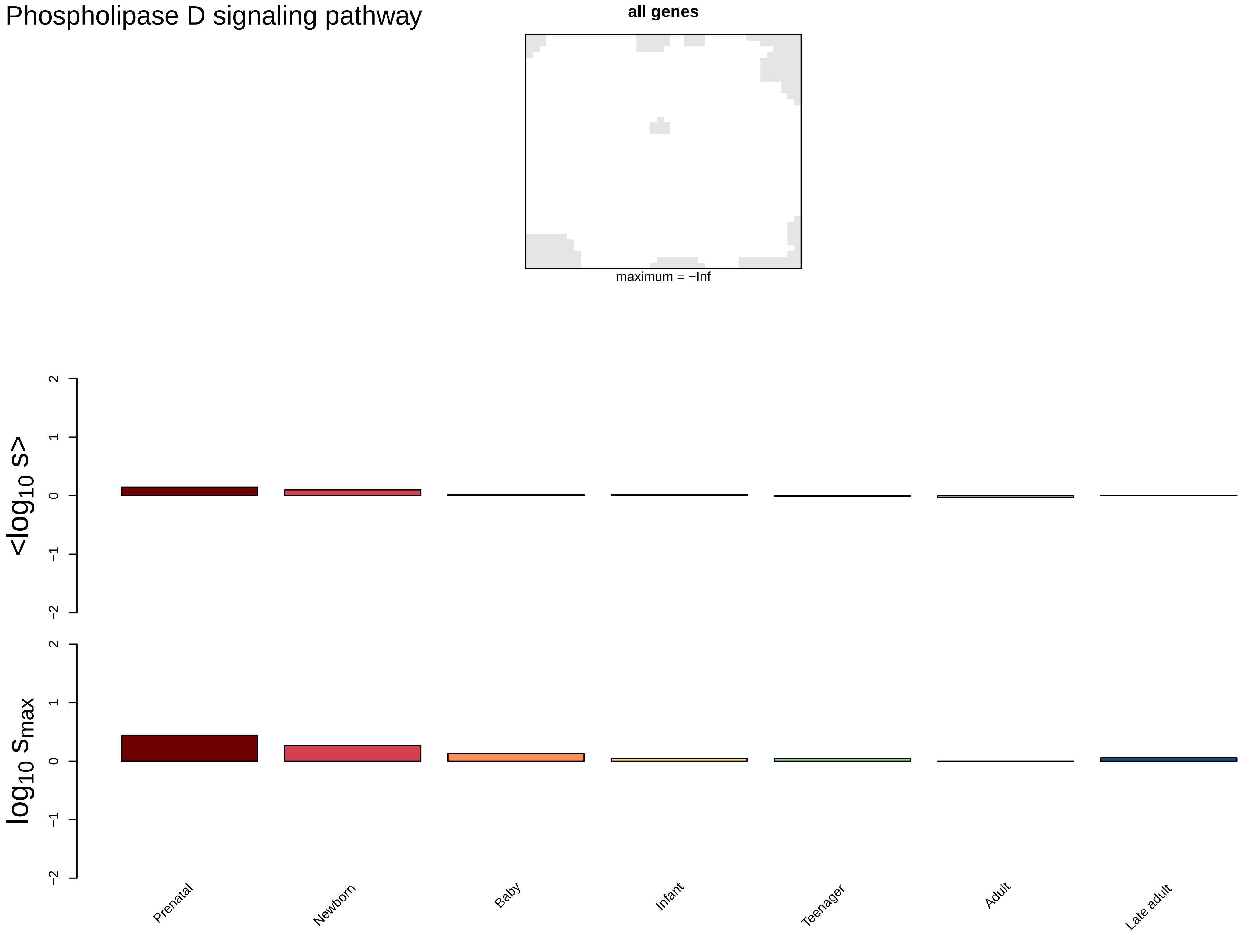

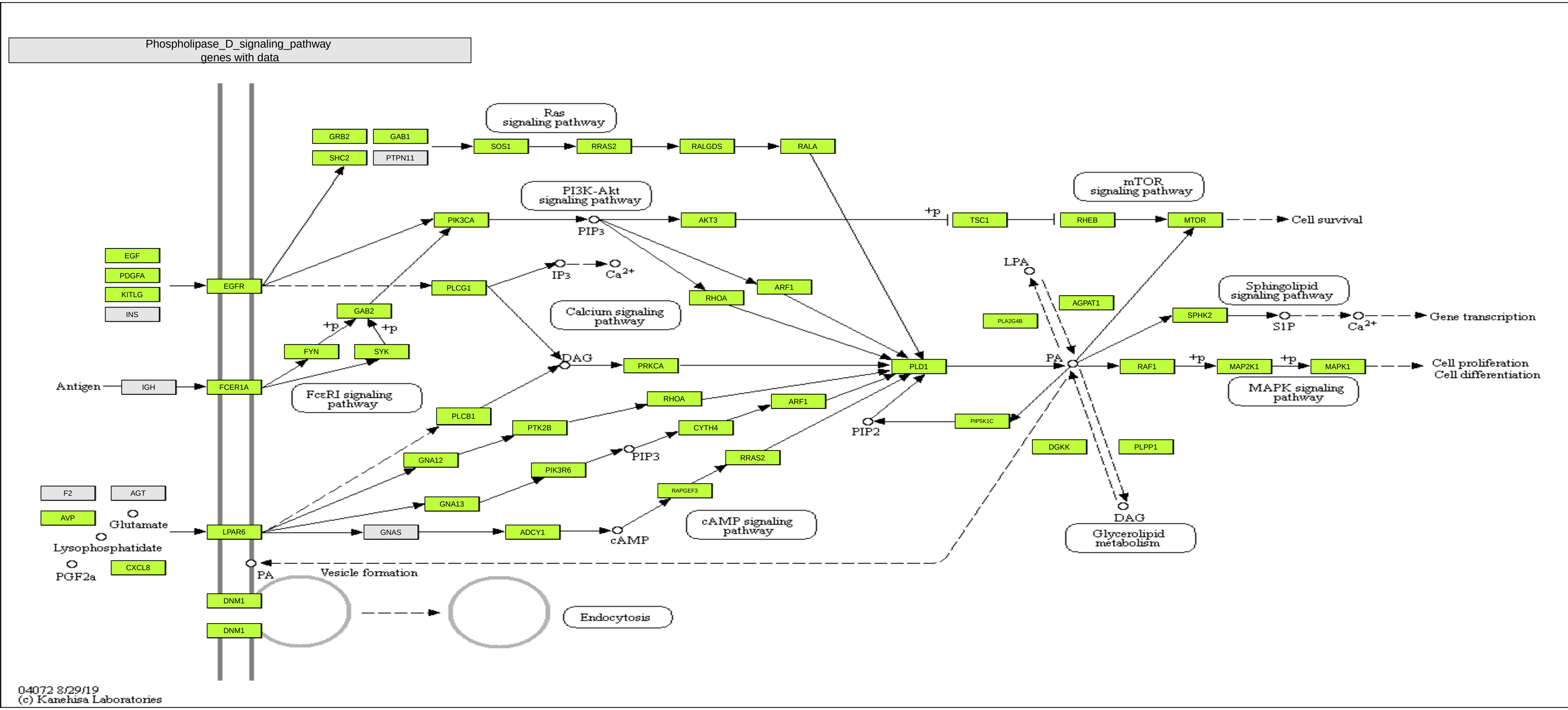

Phospholipase_D_signaling_pathway
genes with data
Ras signaling pathway
GRB2
GAB1
SHC2
PTPN11
SOS1
RRAS2
RALGDS
RALA
PI3K-Akt signaling pathway
PIK3CA
PIP3
AKT3
+p
TSC1
RHEB
MTOR
mTOR signaling pathway
Cell survival
EGF
PDGFA
KITLG
INS
EGFR
PLCG1
IP3
Ca2+
GAB2
+p
FYN
SYK
Calcium signaling pathway
RHOA
ARF1
LPA
AGPAT1
PLA2G4B
Sphingolipid signaling pathway
SPHK2
S1P
Gene transcription
DAG
PRKCA
PLD1
PA
RAF1
MAP2K1
MAPK1
Cell proliferation
Cell differentiation
MAPK signaling pathway
Antigen
IGH
FCER1A
FcεRI signaling pathway
PLCB1
PTK2B
CYTH4
PIP2
PIP5K1C
DGKK
PLPP1
GNA12
PIK3R6
PIP3
RRAS2
F2
AGT
AVP
Glutamate
Lysophosphatidate
PGF2a
CXCL8
LPAR6
GNA13
RAPGEF3
GNAS
ADCY1
cAMP
cAMP signaling pathway
Glycerolipid metabolism
PA
Vesicle formation
DNM1
DNM1
Endocytosis
04072 8/29/19
(c) Kanehisa Laboratories

Phospholipase_D_signaling_pathway
sink nodes

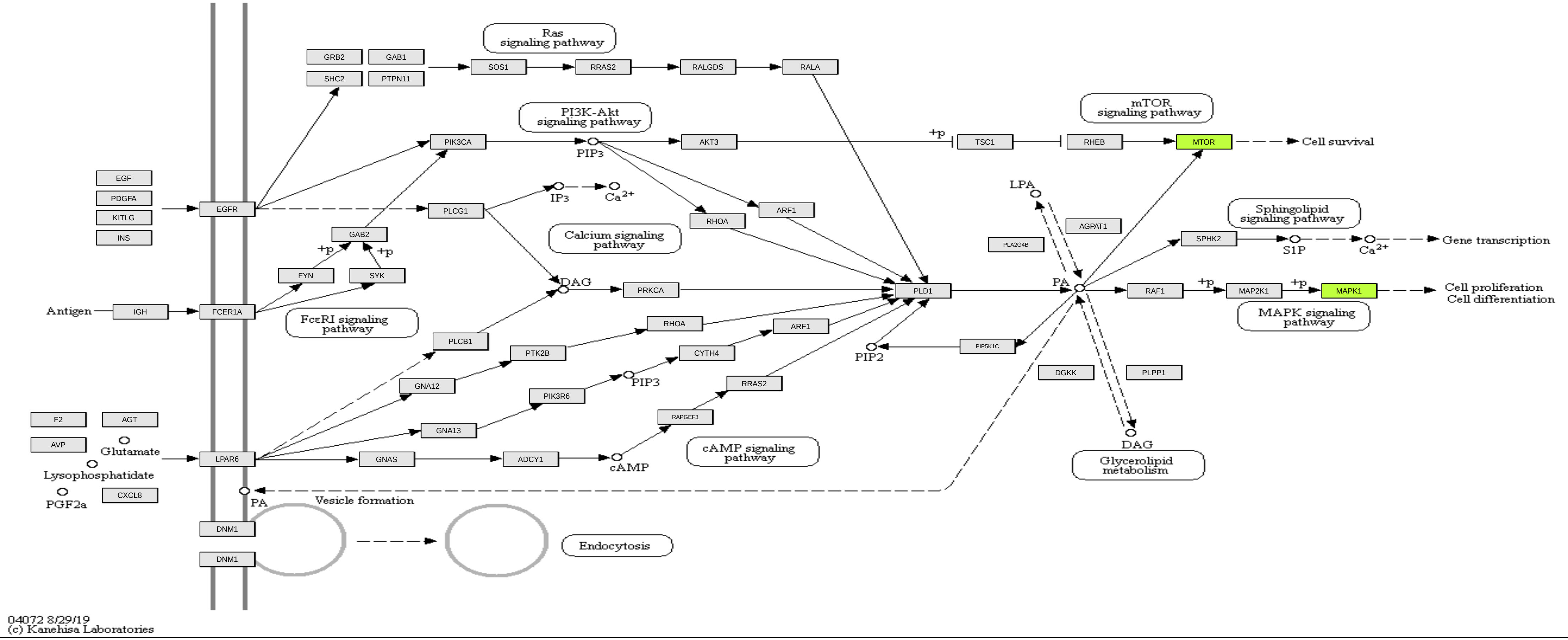

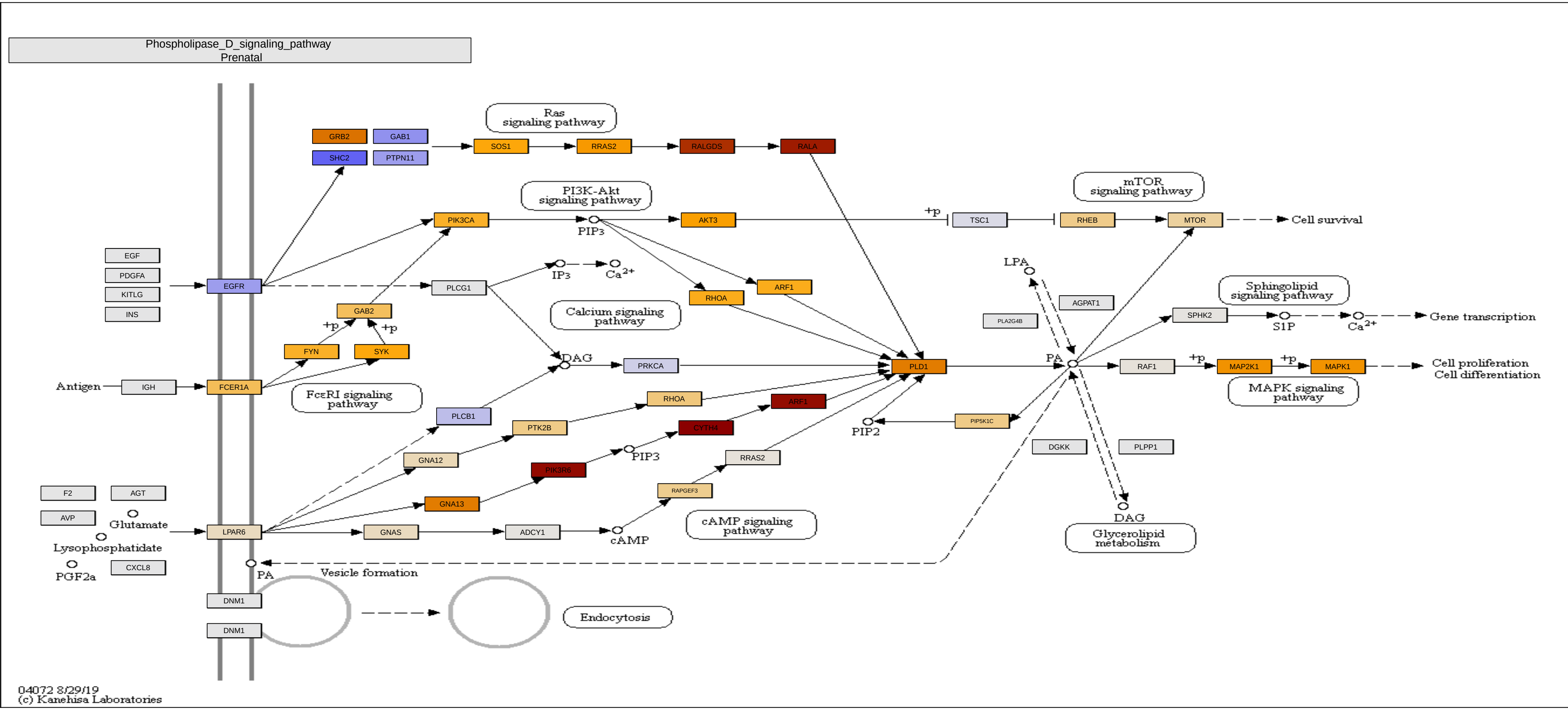

Phospholipase_D_signaling_pathway
Prenatal
Ras signaling pathway
GRB2
GAB1
SHC2
PTPN11
SOS1
RRAS2
RALGDS
RALA
PI3K-Akt signaling pathway
mTOR signaling pathway
PIK3CA
PIP3
AKT3
+p
TSC1
RHEB
MTOR
Cell survival
EGF
PDGFA
KITLG
INS
EGFR
PLCG1
IP3
Ca2+
RHOA
ARF1
LPA
AGPAT1
PLA2G4B
Sphingolipid signaling pathway
SPHK2
S1P
Ca2+
Gene transcription
GAB2
+p
+p
Calcium signaling pathway
FYN
SYK
DAG
PRKCA
PLD1
PA
RAF1
+p
MAP2K1
+p
MAPK1
Cell proliferation
Cell differentiation
Antigen
IGH
FCER1A
FcεRI signaling pathway
PLCB1
RHOA
ARF1
MAPK signaling pathway
PTK2B
CYTH4
PIP5K1C
PIP2
GNA12
PIP3
DGKK
PLPP1
PIK3R6
RRAS2
F2
AGT
RAPGEF3
GNA13
AVP
Glutamate
Lysophosphatidate
LPAR6
GNAS
ADCY1
cAMP
cAMP signaling pathway
DAG
Glycerolipid metabolism
PGF2a
CXCL8
PA
Vesicle formation
DNM1
DNM1
Endocytosis
04072 8/29/19
(c) Kanehisa Laboratories

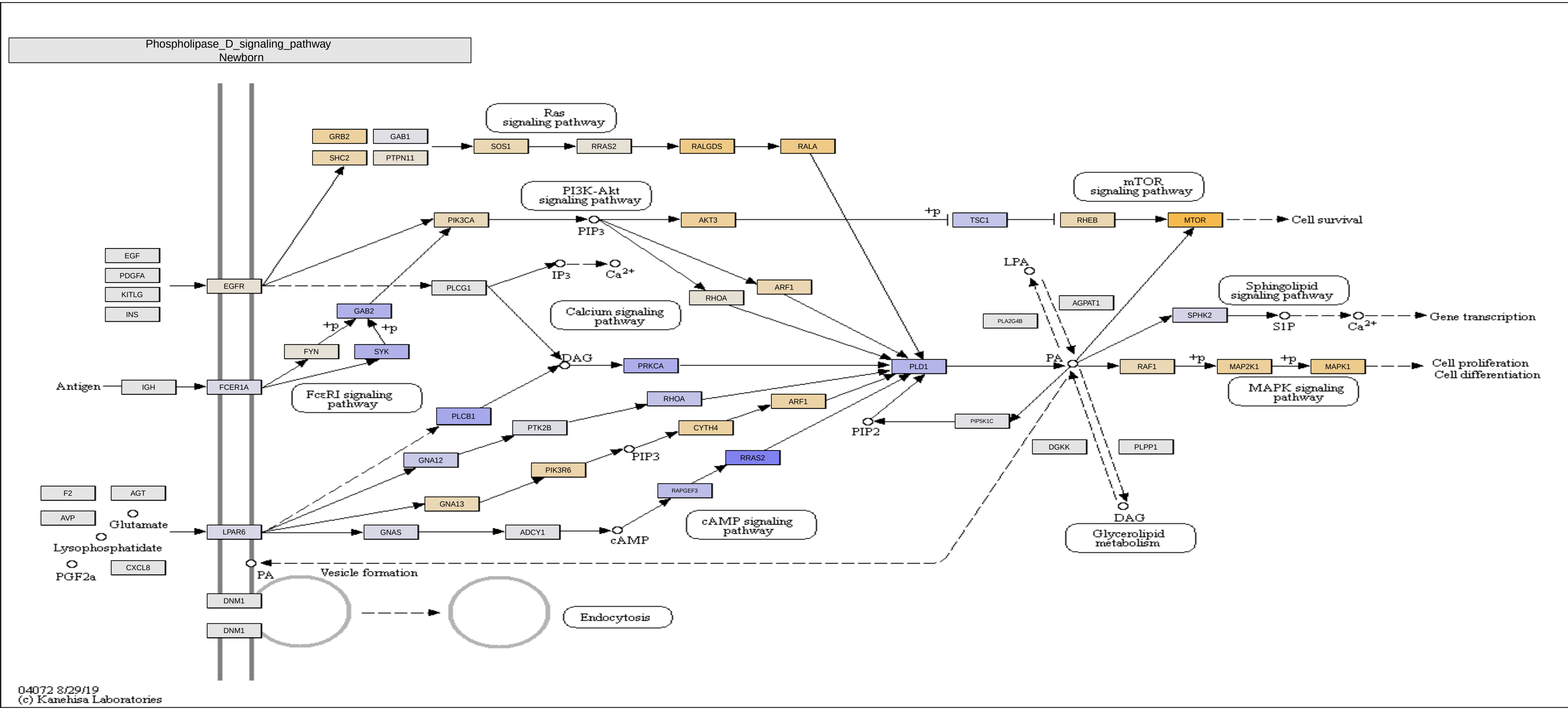
Phospholipase_D_signaling_pathway
Newborn
Ras signaling pathway
GRB2
GAB1
SHC2
PTPN11
SOS1
RRAS2
RALGDS
RALA
PI3K-Akt signaling pathway
PIK3CA
PIP3
AKT3
+p
TSC1
RHEB
MTOR
mTOR signaling pathway
Cell survival
EGF
PDGFA
KITLG
INS
EGFR
PLCG1
IP3
Ca2+
RHOA
ARF1
LPA
AGPAT1
PLA2G4B
Sphingolipid signaling pathway
SPHK2
S1P
Gene transcription
GAB2
+p
FYN
SYK
Calcium signaling pathway
DAG
PRKCA
PLD1
PA
RAF1
MAP2K1
MAPK1
Cell proliferation
Cell differentiation
MAPK signaling pathway
Antigen
IGH
FCER1A
FcεRI signaling pathway
PLCB1
RHOA
ARF1
PTK2B
CYTH4
PIP2
PIP5K1C
GNA12
PIK3R6
PIP3
RRAS2
DGKK
PLPP1
F2
AGT
AVP
Glutamate
GNA13
RAPGEF3
Lysophosphatidate
LPAR6
GNAS
ADCY1
cAMP
cAMP signaling pathway
DAG
Glycerolipid metabolism
PGF2a
CXCL8
PA
Vesicle formation
DNM1
DNM1
Endocytosis
04072 8/29/19
(c) Kanehisa Laboratories

Phospholipase_D_signaling_pathway
Baby

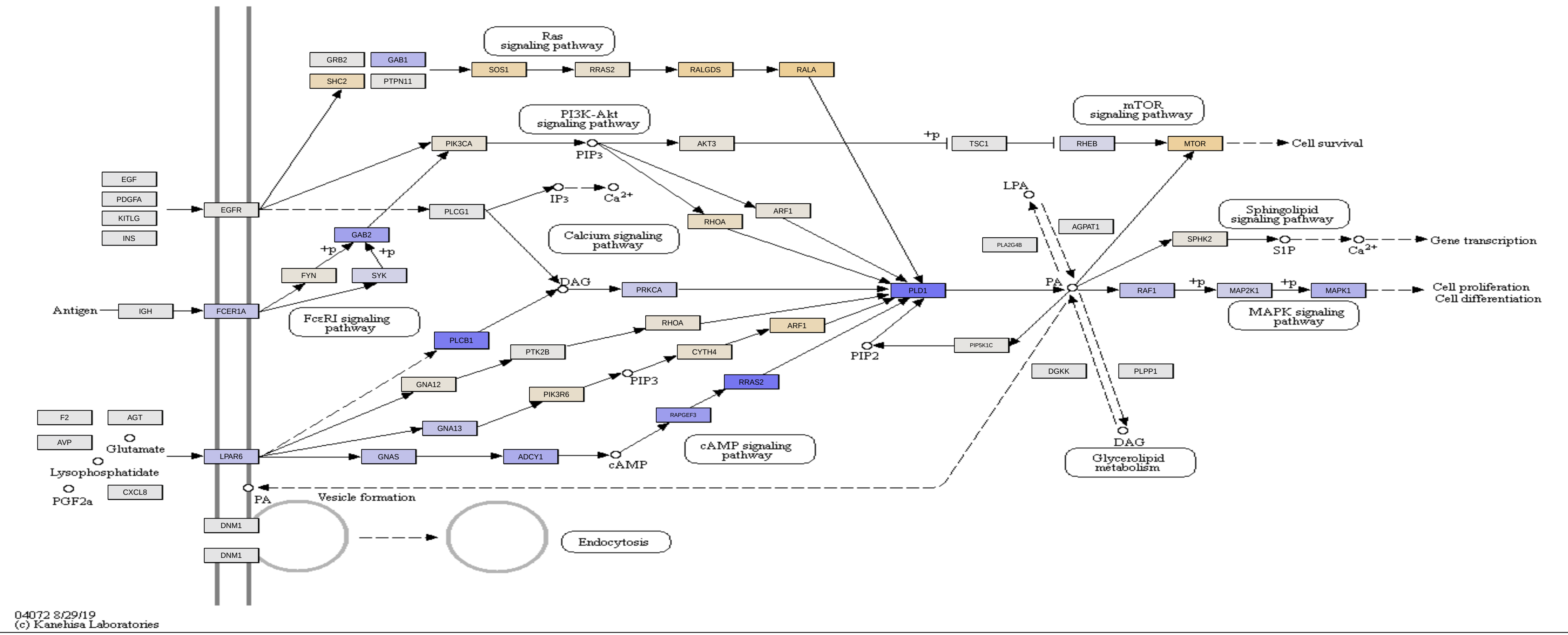

Phospholipase_D_signaling_pathway
Infant

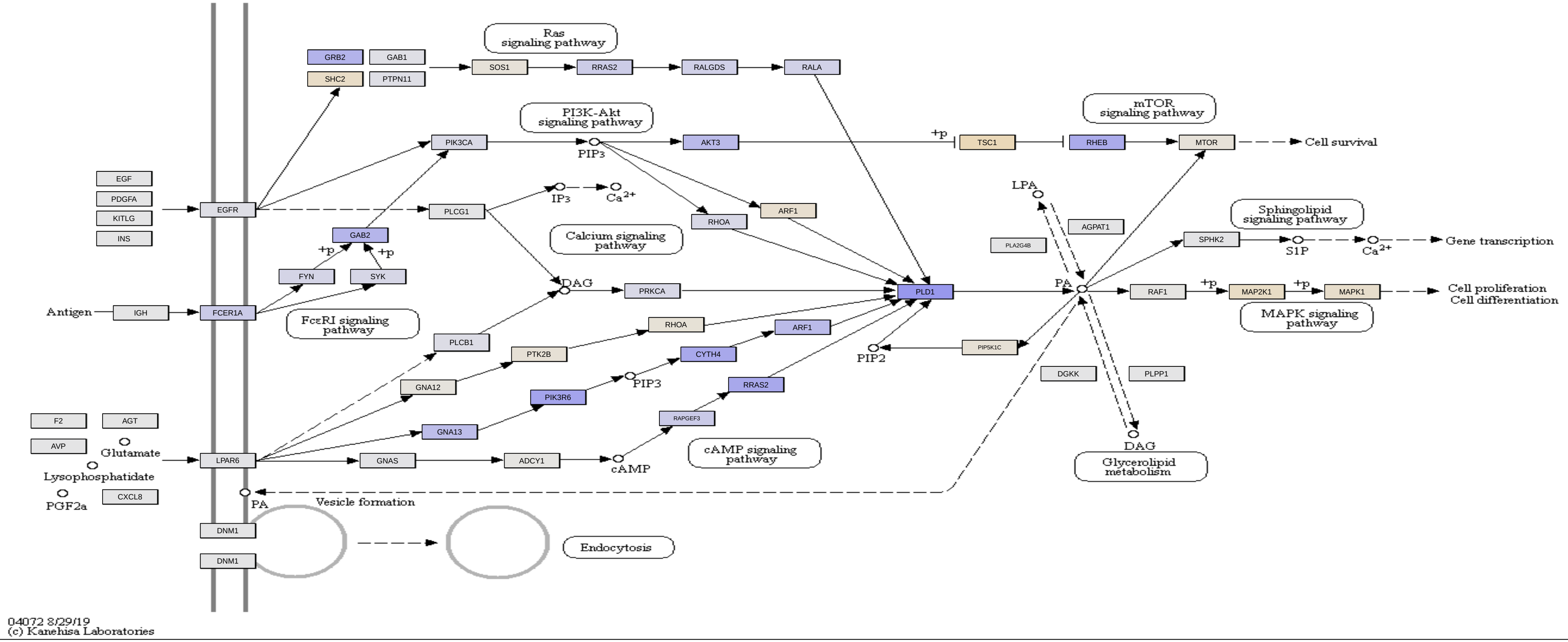

Phospholipase_D_signaling_pathway
Teenager

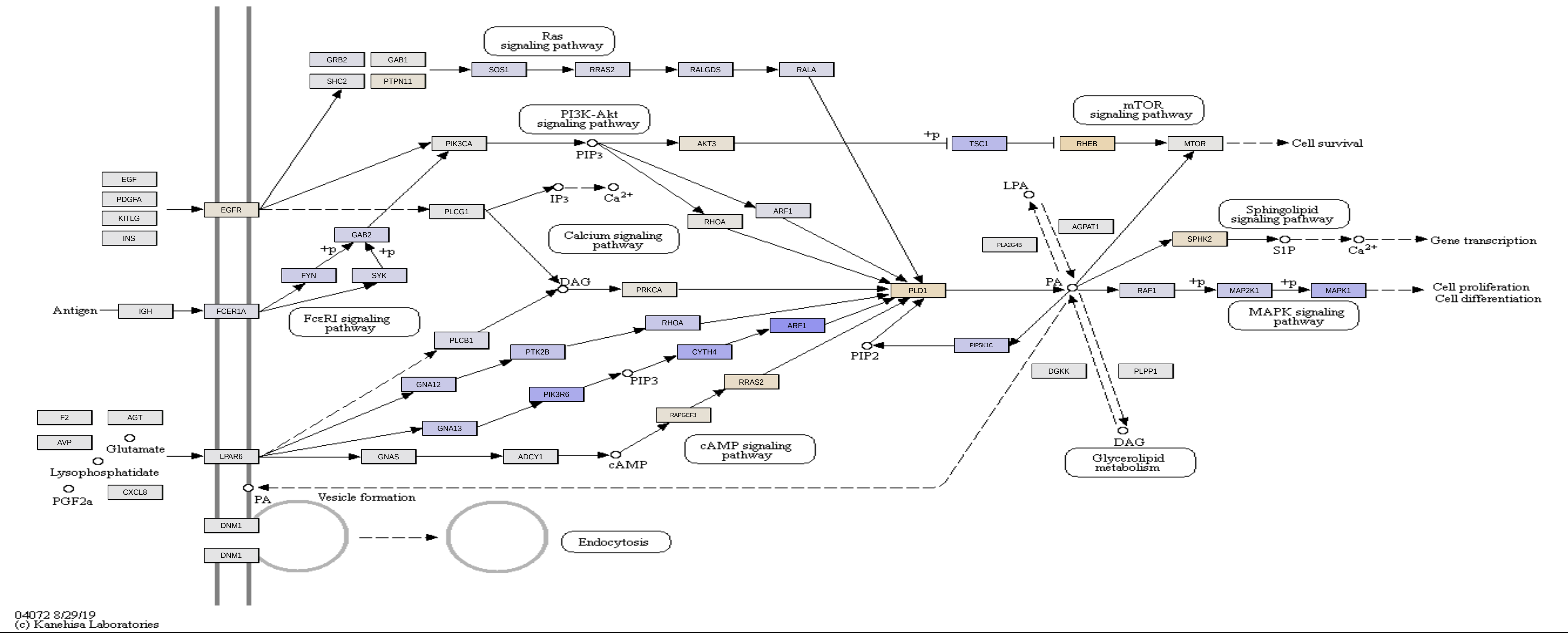

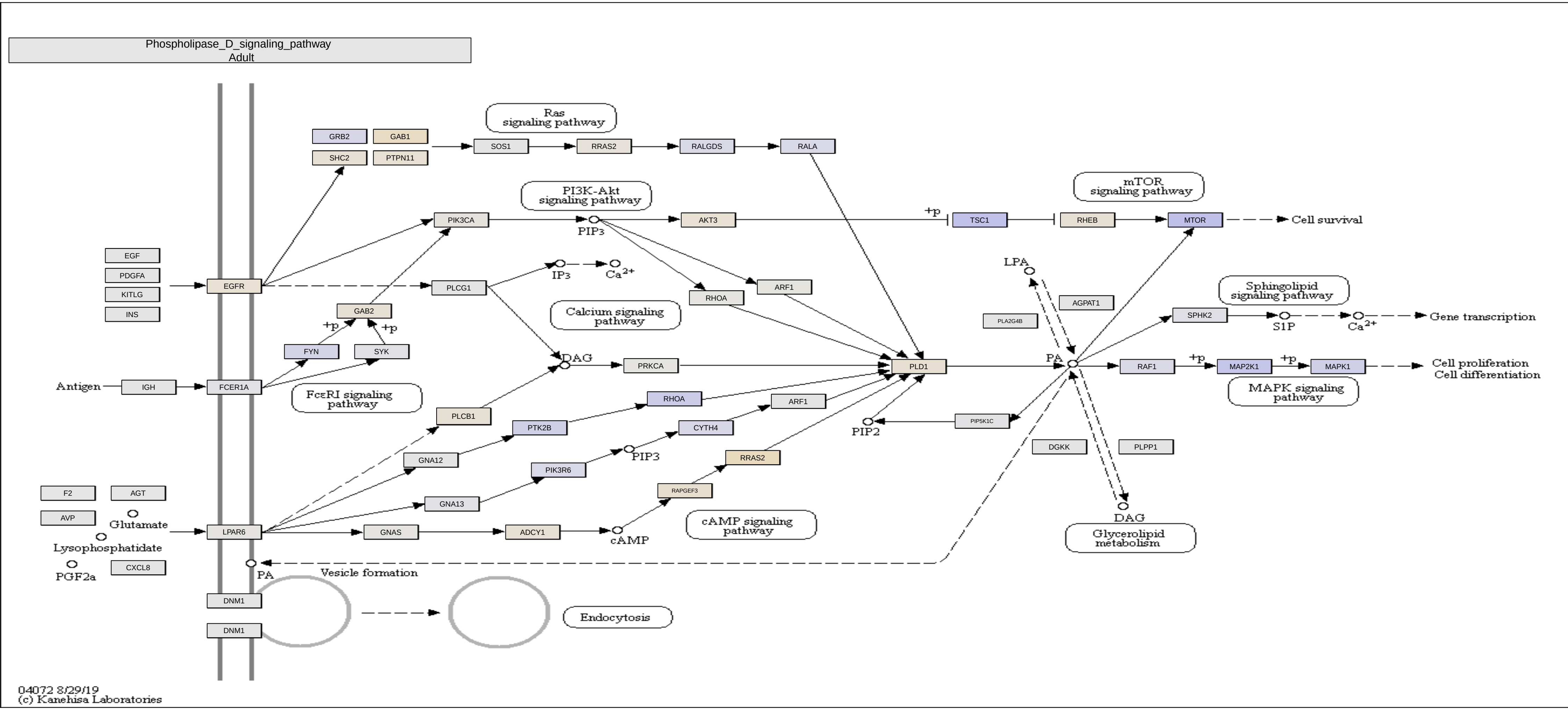

(c) Kanehisa Laboratories

Phospholipase_D_signaling_pathway
Late adult

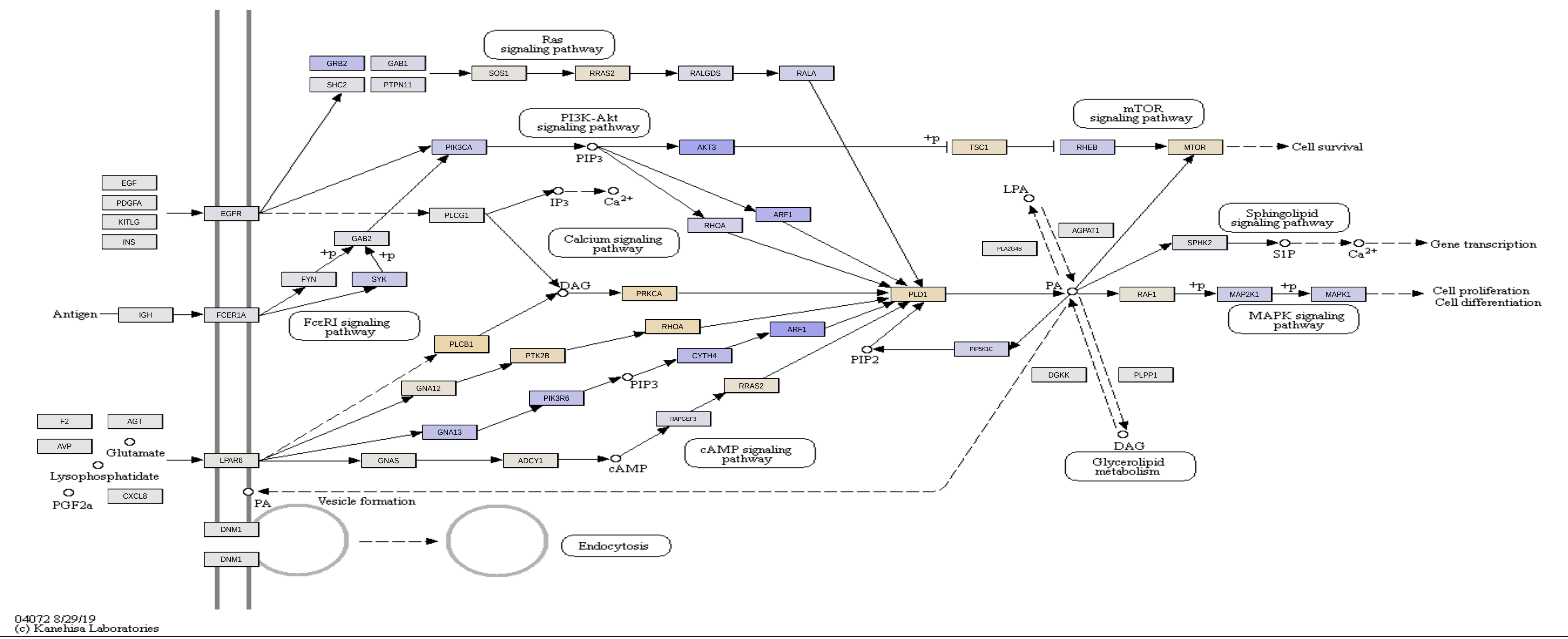

Supplement: Supplementary file 1 [file cells-11-00362-s001.zip › Suppl-Material-S4-Pathways-PSF_Methylation/Phospholipase_D_signaling_pathway.pdf]
