# Supplementary material for: The Transcriptome and Methylome of the Developing and Aging Brain and Their Relations to Gliomas and Psychological Disorders
Source: Cells. 2022 Jan 21;11(3):362. doi: 10.3390/cells11030362 (PMC8834030; doi:10.3390/cells11030362)
Supplement: Supplementary file 1 [file cells-11-00362-s001.zip › Suppl-Material-S4-Pathways-PSF_Methylation/Ras_signaling_pathway.pdf]

Ras signaling pathway

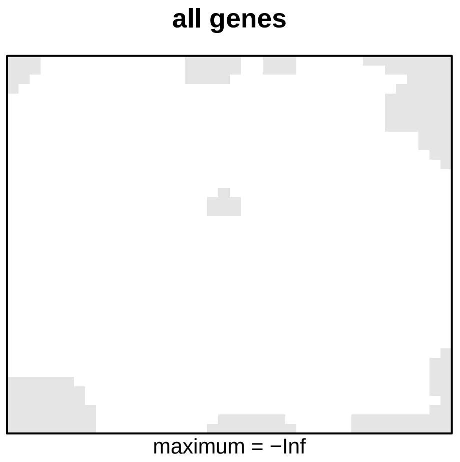

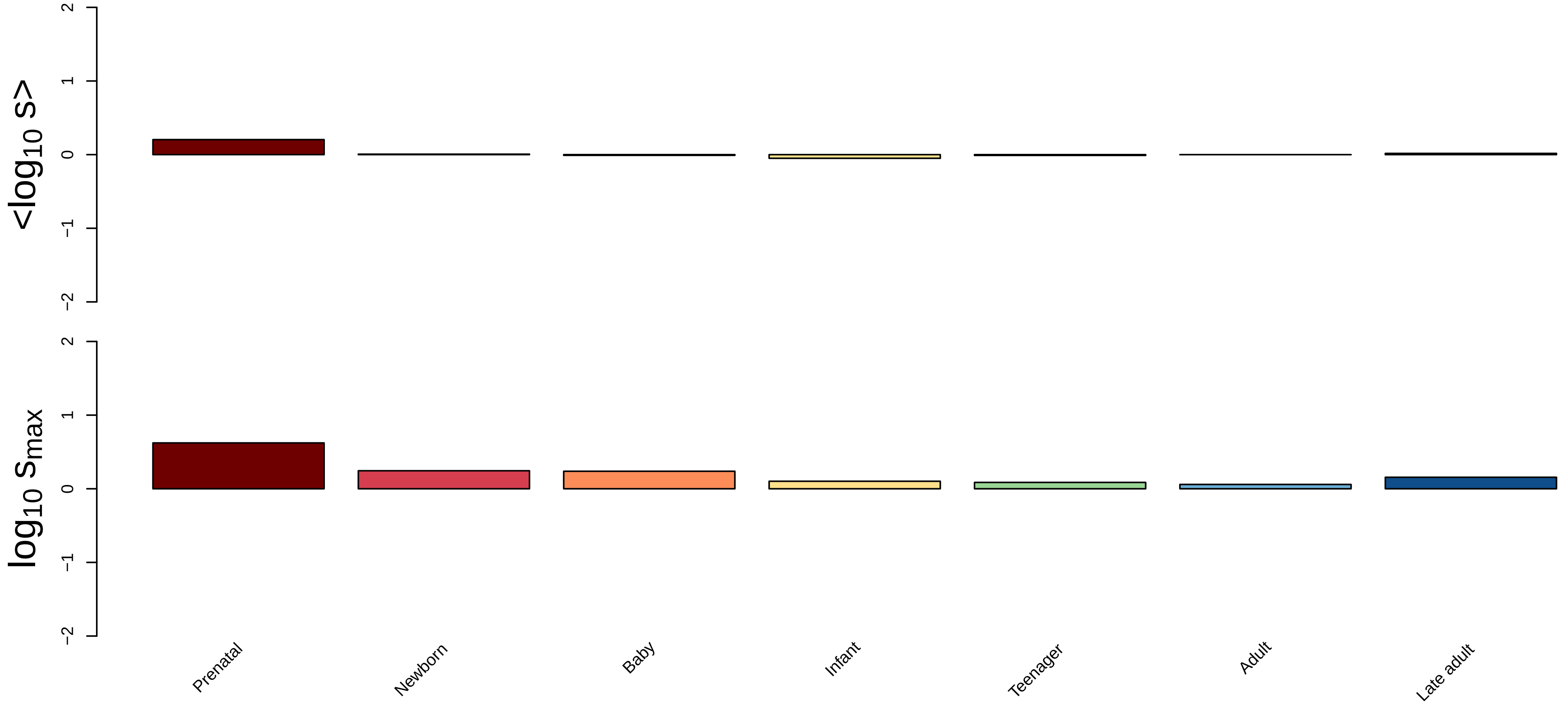

Ras_signaling_pathway
genes with data

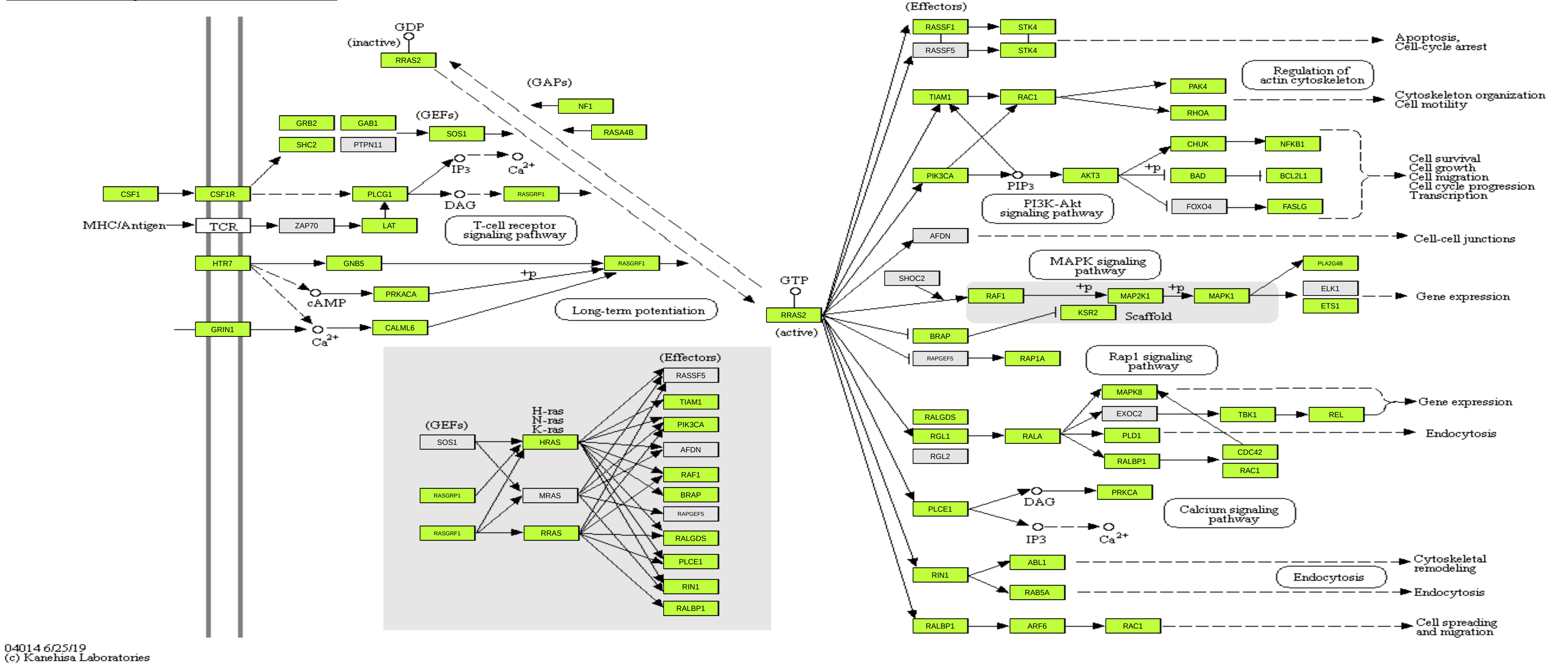

Ras_signaling_pathway
sink nodes

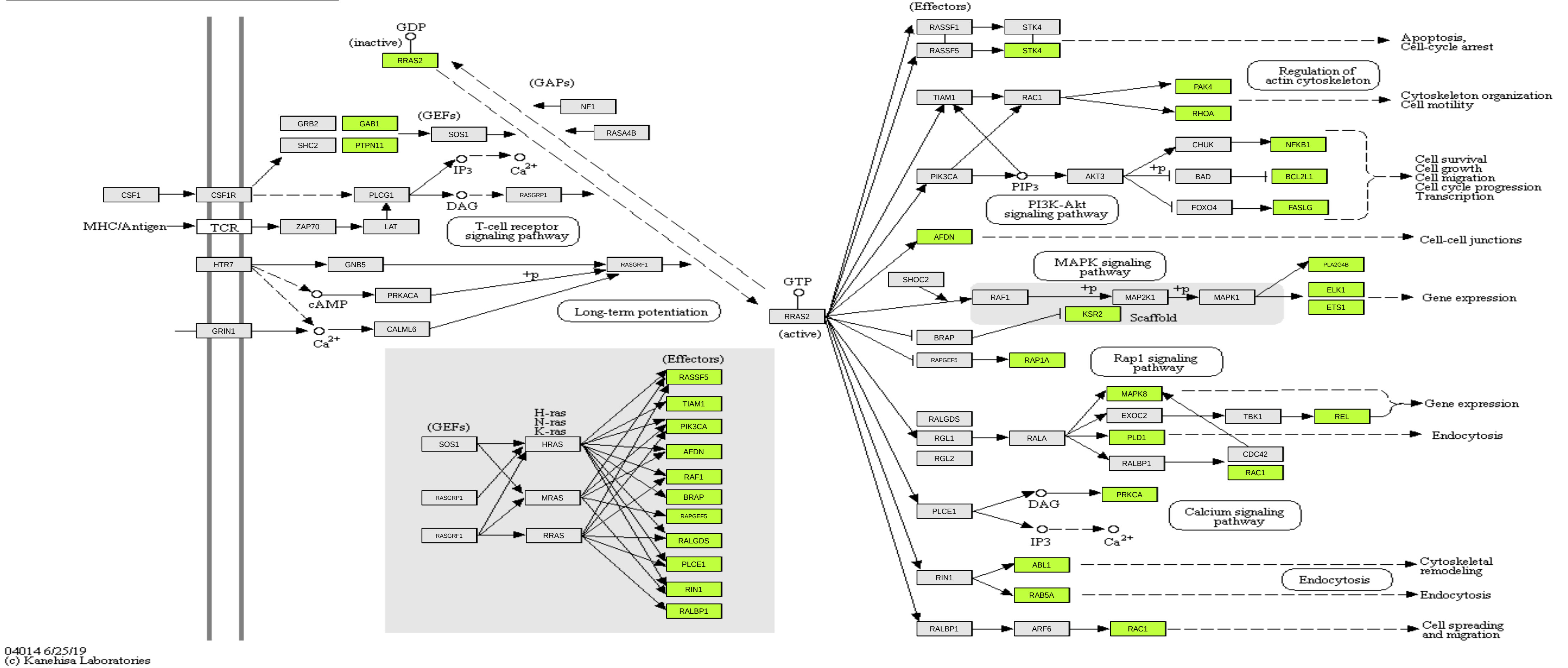

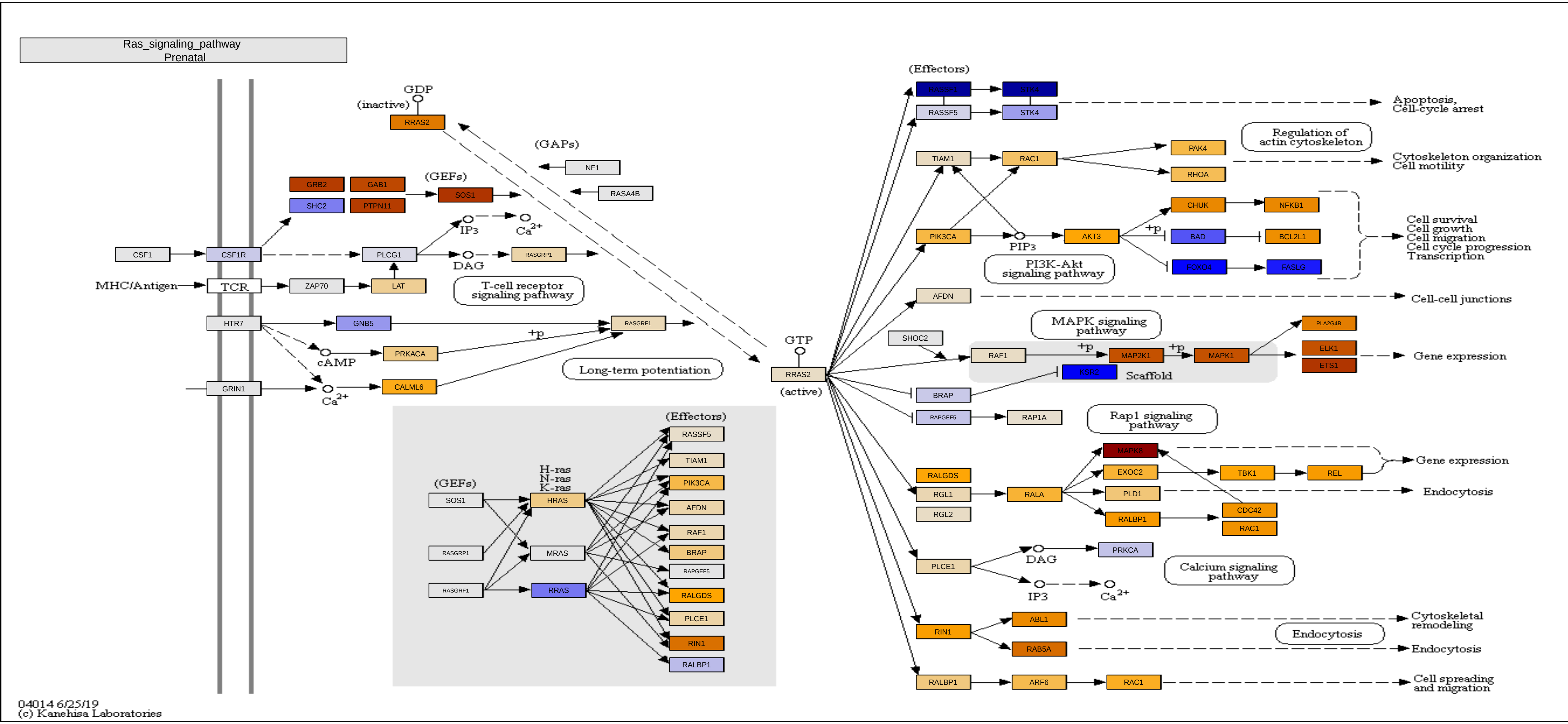

Ras_signaling_pathway
Prenatal
GDP
(inactive)
RRAS2
(GAPs)
NF1
RASA4B
(GEFs)
GRB2
GAB1
SHC2
PTPN11
SOS1
IP3
Ca2+
CSF1
CSF1R
PLCG1
DAG
RASGRP1
MHC/Antigen
TCR
ZAP70
LAT
T-cell receptor signaling pathway
HTR7
GNB5
RASGRF1
+p
cAMP
PRKACA
Long-term potentiation
GRIN1
Ca2+
CALML6
(Effectors)
RASSF5
TIAM1
PIK3CA
AFDN
RAF1
BRAP
RAPGEF5
RALGDS
PLCE1
RIN1
RALBP1
(GEFs)
SOS1
RASGRP1
RASGRF1
H-ras
N-ras
K-ras
HRAS
MRAS
RRAS
GTP
RRAS2
(active)
(Effectors)
RASSF5
STK4
Apoptosis, Cell-cycle arrest
TIAM1
RAC1
PAK4
RHOA
Regulation of actin cytoskeleton
Cytoskeleton organization
Cell motility
CHUK
NFKB1
PIK3CA
PIP3
AKT3
+p
BAD
BCL2L1
FOXO4
FASLG
Cell survival
Cell growth
Cell migration
Cell cycle progression
Transcription
PI3K-Akt signaling pathway
AFDN
Cell-cell junctions
MAPK signaling pathway
PLA2G4B
SHOC2
RAF1
+p
MAP2K1
+p
MAPK1
ELK1
ETS1
Gene expression
KSR2
Scaffold
BRAP
RAPGEF5
RAP1A
Rap1 signaling pathway
MAPK8
EXOC2
TBK1
REL
Gene expression
RALGDS
RGL1
RALA
PLD1
Endocytosis
RGL2
CDC42
RALBP1
RAC1
PLCE1
DAG
PRKCA
Calcium signaling pathway
IP3
Ca2+
RIN1
ABL1
Cytoskeletal remodeling
Endocytosis
RAB5A
Endocytosis
RALBP1
ARF6
RAC1
Cell spreading and migration
04014 6/25/19
(c) Kanehisa Laboratories

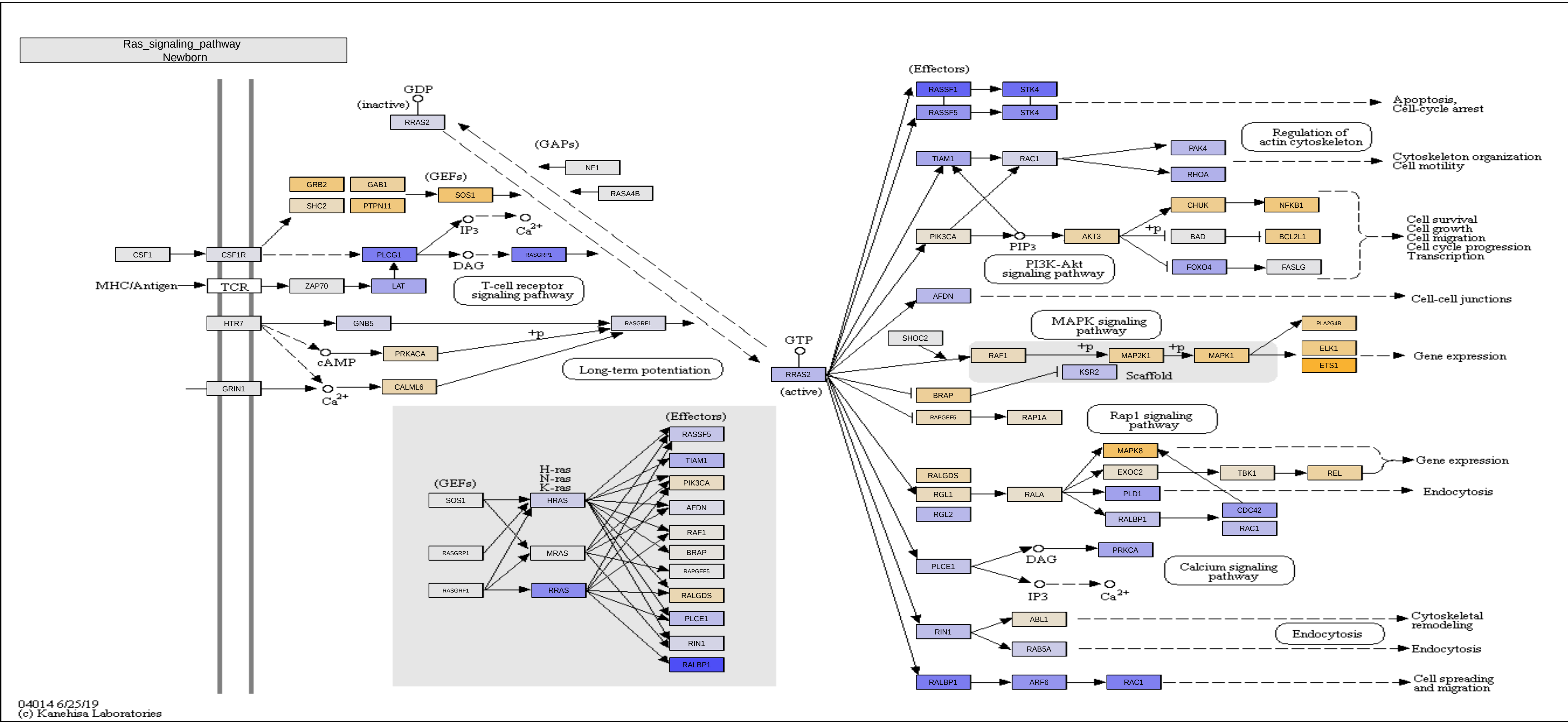
Ras_signaling_pathway
Newborn
(Effectors)
RASSF1
STK4
RASSF5
STK4
Apoptosis,
Cell-cycle arrest
GDP
(inactive)
RRAS2
(GAPs)
NF1
RASA4B
TIAM1
RAC1
PAK4
RHOA
Regulation of actin cytoskeleton
Cytoskeleton organization
Cell motility
GRB2
GAB1
SHC2
PTPN11
(GEFs)
SOS1
CHUK
NFKB1
BAD
BCL2L1
FOXO4
FASLG
PIK3CA
PIP3
AKT3
+p
PI3K-Akt signaling pathway
Cell survival
Cell growth
Cell migration
Cell cycle progression
Transcription
CSF1
CSF1R
PLCG1
IP3
Ca2+
DAG
RASGRP1
MHC/Antigen
TCR
ZAP70
LAT
T-cell receptor signaling pathway
AFDN
Cell-cell junctions
HTR7
GNB5
RASGRF1
+p
cAMP
PRKACA
Long-term potentiation
GTP
RRAS2
(active)
SHOC2
MAPK signaling pathway
RAF1
MAP2K1
MAPK1
PLA2G4B
ELK1
ETS1
Gene expression
KSR2
Scaffold
BRAP
RAPGEF5
RAP1A
Rap1 signaling pathway
GRIN1
CALML6
(Effectors)
RASSF5
TIAM1
PIK3CA
AFDN
RAF1
BRAP
RAPGEF5
RALGDS
PLCE1
RIN1
RALBP1
H-ras
N-ras
K-ras
(GEFs)
SOS1
HRAS
RASGRP1
MRAS
RASGRF1
RRAS
MAPK8
EXOC2
TBK1
REL
Gene expression
RALGDS
RGL1
RALA
PLD1
Endocytosis
RGL2
RALBP1
CDC42
RAC1
PLCE1
DAG
PRKCA
IP3
Calcium signaling pathway
RIN1
ABL1
RAB5A
Cytoskeletal remodeling
Endocytosis
RALBP1
ARF6
RAC1
Cell spreading and migration
04014 6/25/19
(c) Kanehisa Laboratories

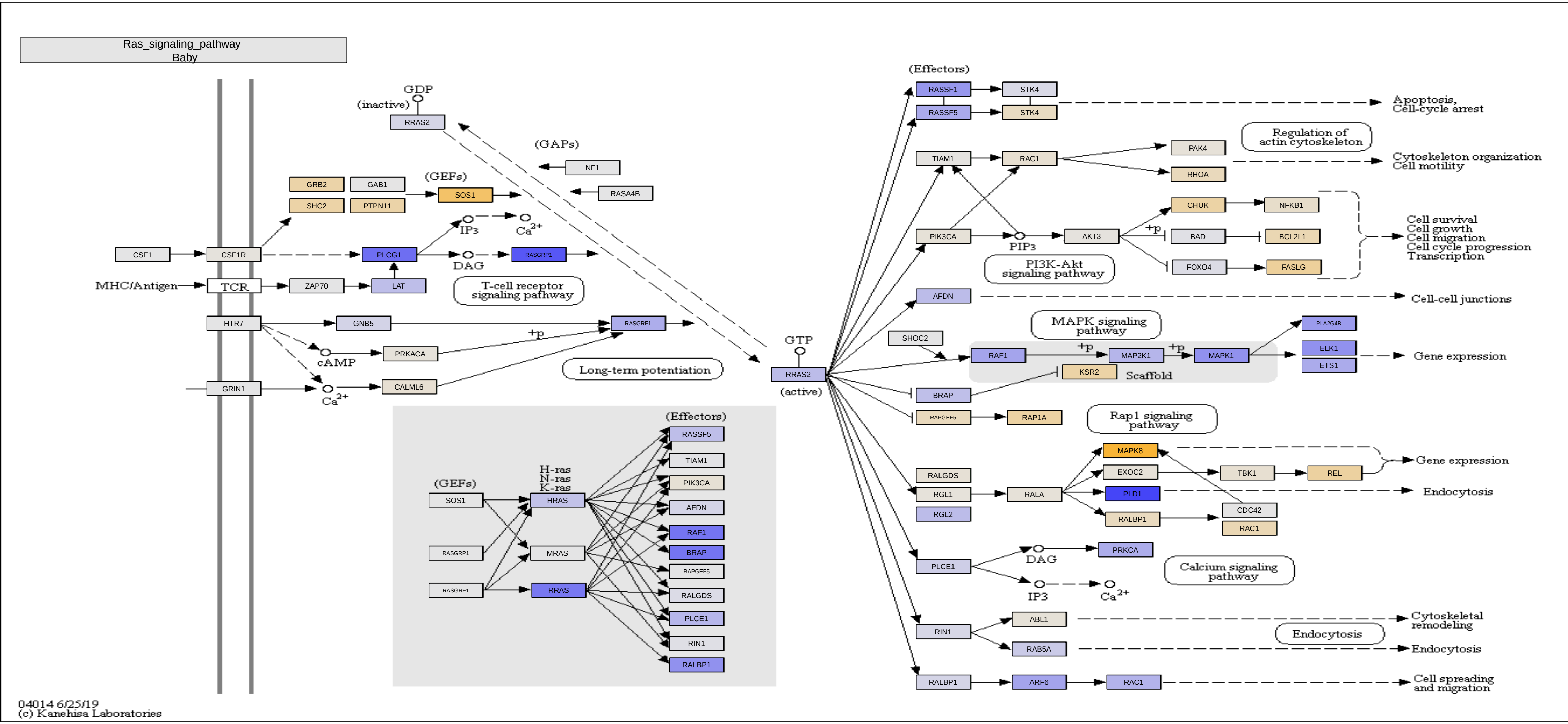
Ras_signaling_pathway
Baby
GDP
(inactive)
RRAS2
(GAPs)
NF1
RASA4B
GRB2
GAB1
SHC2
PTPN11
(GEFs)
SOS1
IP3
Ca2+
CSF1
CSF1R
PLCG1
DAG
RASGRP1
MHC/Antigen
TCR
ZAP70
LAT
T-cell receptor signaling pathway
HTR7
GNB5
RASGRF1
+p
PRKACA
cAMP
Long-term potentiation
GRIN1
CALML6
Ca2+
(Effectors)
RASSF5
TIAM1
PIK3CA
AFDN
RAF1
BRAP
RAPGEF5
RALGDS
PLCE1
RIN1
RALBP1
(GEFs)
H-ras
N-ras
K-ras
SOS1
HRAS
RASGRP1
MRAS
RASGRF1
RRAS
GTP
RRAS2
(active)
(Effectors)
RASSF1
STK4
RASSF5
STK4
Apoptosis, Cell-cycle arrest
TIAM1
RAC1
PAK4
RHOA
Regulation of actin cytoskeleton
Cytoskeleton organization Cell motility
CHUK
NFKB1
PIK3CA
PIP3
AKT3
+p
BAD
BCL2L1
FOXO4
FASLG
Cell survival Cell growth Cell migration Cell cycle progression Transcription
PI3K-Akt signaling pathway
AFDN
Cell-cell junctions
MAPK signaling pathway
SHOC2
RAF1
+p
MAP2K1
+p
MAPK1
PLA2G4B
ELK1
ETS1
Gene expression
KSR2
Scaffold
BRAP
RAPGEF5
RAP1A
Rap1 signaling pathway
MAPK8
EXOC2
TBK1
REL
Gene expression
RALGDS
RGL1
RALA
PLD1
Endocytosis
RGL2
RALBP1
CDC42
RAC1
PLCE1
DAG
PRKCA
IP3
Ca2+
Calcium signaling pathway
RIN1
ABL1
Cytoskeletal remodeling
Endocytosis
RAB5A
Endocytosis
RALBP1
ARF6
RAC1
Cell spreading and migration
04014 6/25/19
(c) Kanehisa Laboratories

Ras_signaling_pathway
Infant

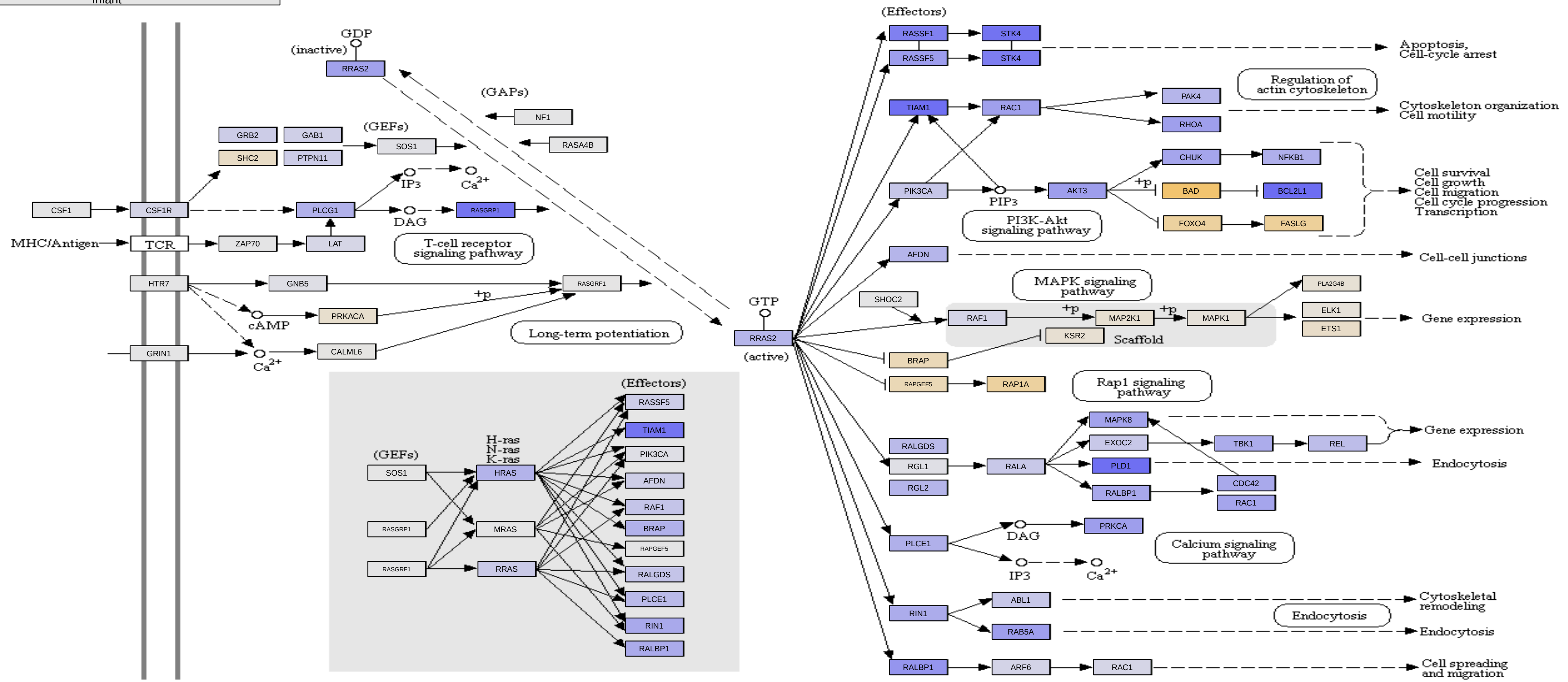

04014 6/25/19
(c) Kanehisa Laboratories

Ras_signaling_pathway
Teenager

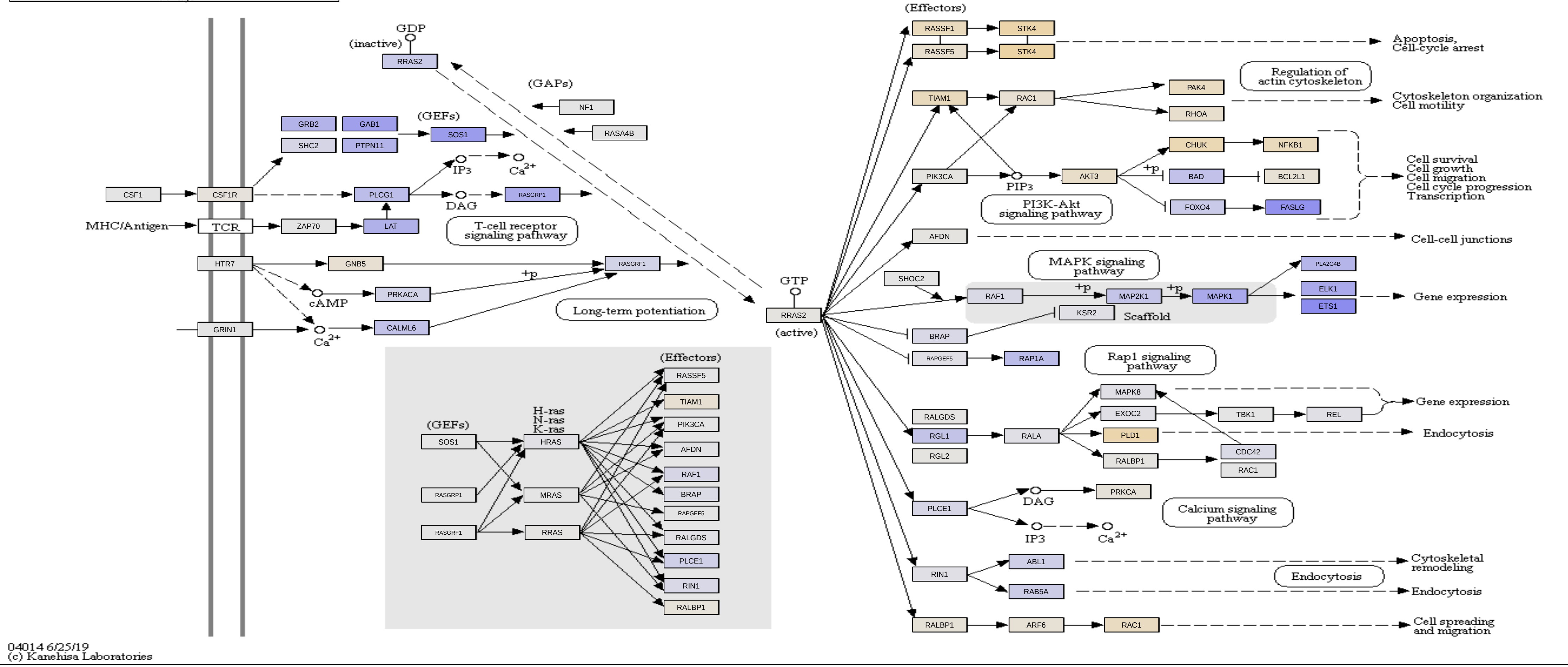

Ras_signaling_pathway
Adult

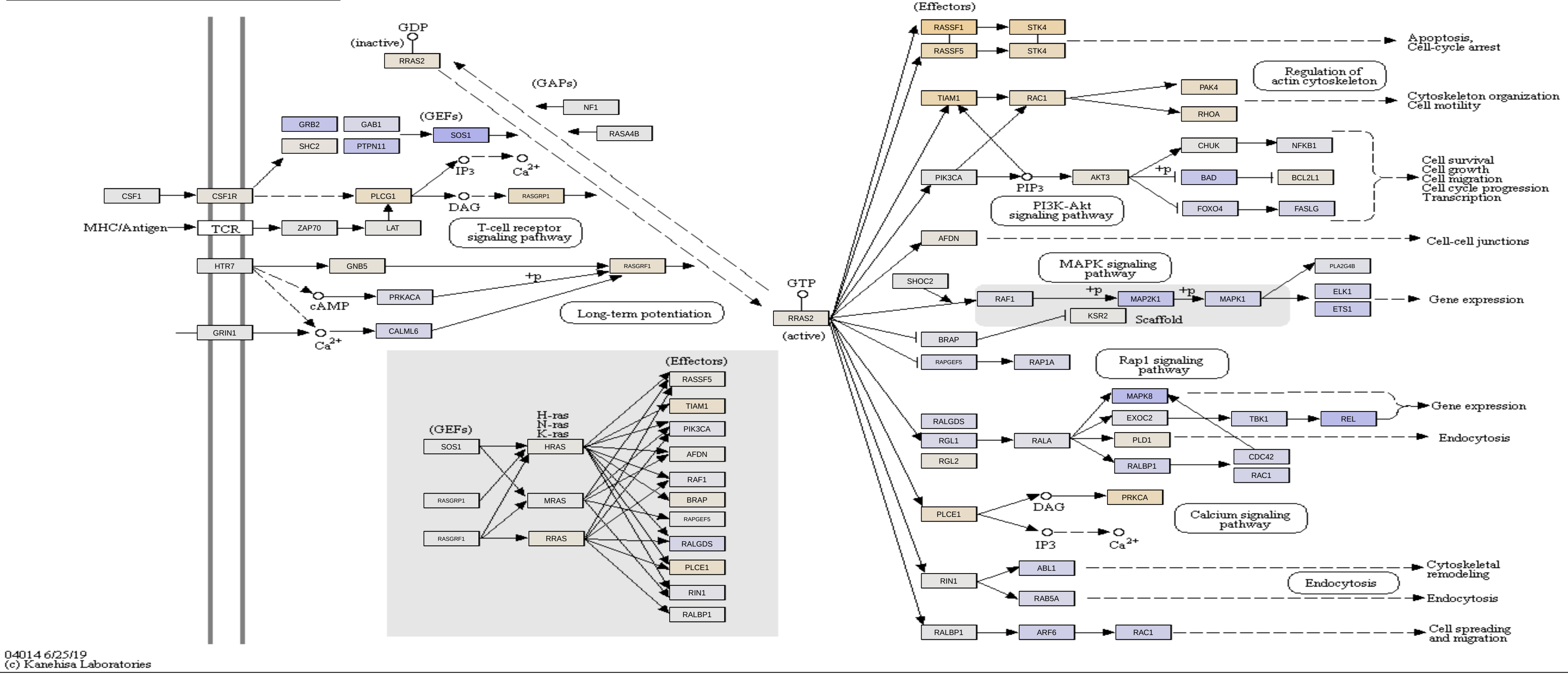

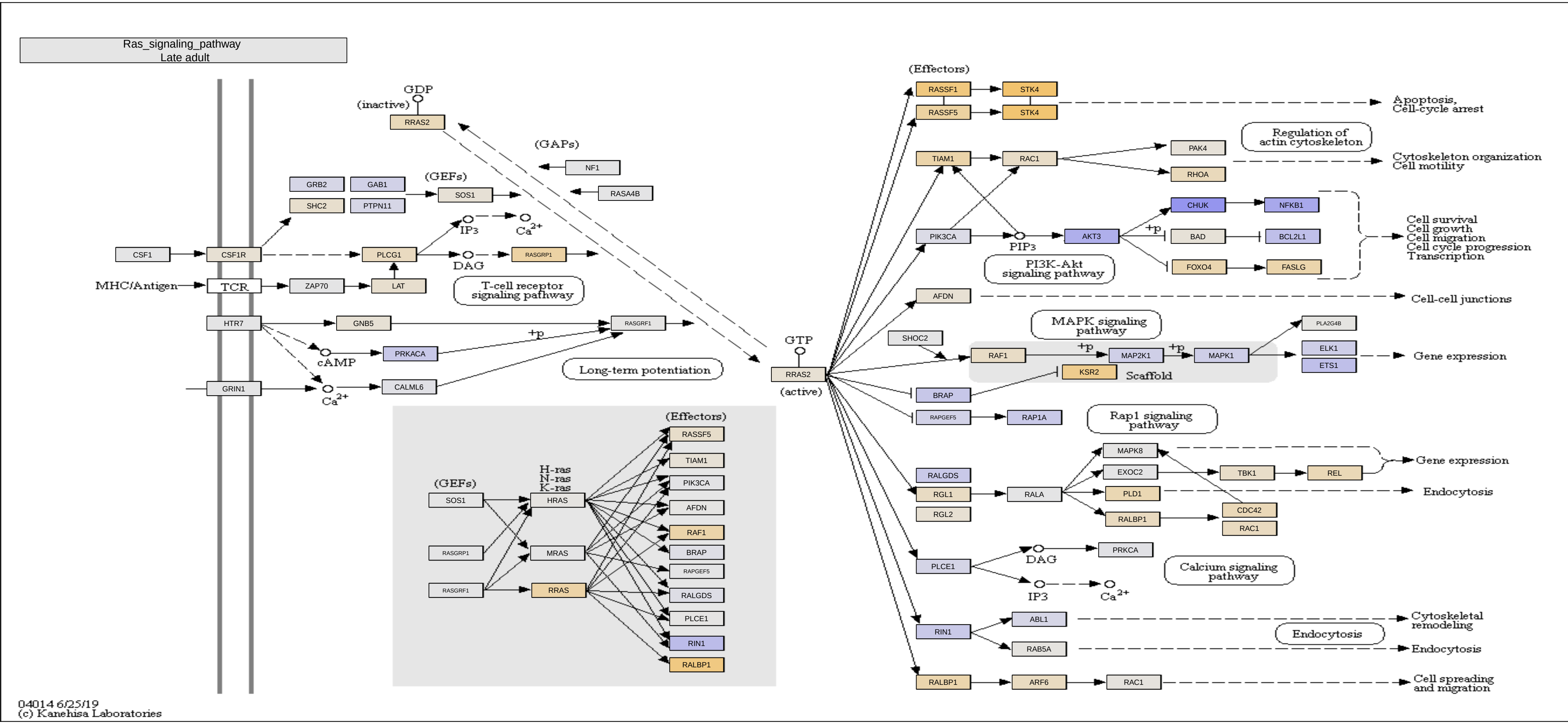
Ras_signaling_pathway
Late adult
GDP
(inactive)
RRAS2
(GAPs)
NF1
RASA4B
GRB2
GAB1
SHC2
PTPN11
(GEFs)
SOS1
IP3
Ca2+
CSF1
CSF1R
PLCG1
DAG
RASGRP1
MHC/Antigen
TCR
ZAP70
LAT
T-cell receptor signaling pathway
HTR7
GNB5
RASGRF1
+p
cAMP
PRKACA
Long-term potentiation
GRIN1
Ca2+
CALML6
GTP
RRAS2
(active)
(Effectors)
RASSF5
TIAM1
PIK3CA
AFDN
RAF1
BRAP
RAPGEF5
RALGDS
PLCE1
RIN1
RALBP1
H-ras
N-ras
K-ras
SOS1
HRAS
RASGRP1
MRAS
RASGRF1
RRAS
(Effectors)
RASSF1
STK4
RASSF5
STK4
Apoptosis, Cell-cycle arrest
TIAM1
RAC1
PAK4
RHOA
Regulation of actin cytoskeleton
Cytoskeleton organization Cell motility
CHUK
NFKB1
PIK3CA
PIP3
AKT3
+p
BAD
BCL2L1
FOXO4
FASLG
PI3K-Akt signaling pathway
Cell survival Cell growth Cell migration Cell cycle progression Transcription
AFDN
Cell-cell junctions
MAPK signaling pathway
SHOC2
RAF1
+p
MAP2K1
+p
MAPK1
PLA2G4B
ELK1
ETS1
Gene expression
KSR2
Scaffold
BRAP
RAPGEF5
RAP1A
Rap1 signaling pathway
MAPK8
EXOC2
TBK1
REL
Gene expression
RALGDS
RGL1
RALA
PLD1
Endocytosis
RGL2
RALBP1
CDC42
RAC1
PLCE1
DAG
PRKCA
IP3
Ca2+
Calcium signaling pathway
RIN1
ABL1
Cytoskeletal remodeling
Endocytosis
RAB5A
Endocytosis
RALBP1
ARF6
RAC1
Cell spreading and migration
04014 6/25/19
(c) Kanehisa Laboratories
