# Supplementary material for: The Transcriptome and Methylome of the Developing and Aging Brain and Their Relations to Gliomas and Psychological Disorders
Source: Cells. 2022 Jan 21;11(3):362. doi: 10.3390/cells11030362 (PMC8834030; doi:10.3390/cells11030362)
Supplement: Supplementary file 1 [file cells-11-00362-s001.zip › Suppl-Material-S4-Pathways-PSF_Methylation/T_cell_receptor_signaling_pathway.pdf]

T cell receptor signaling pathway

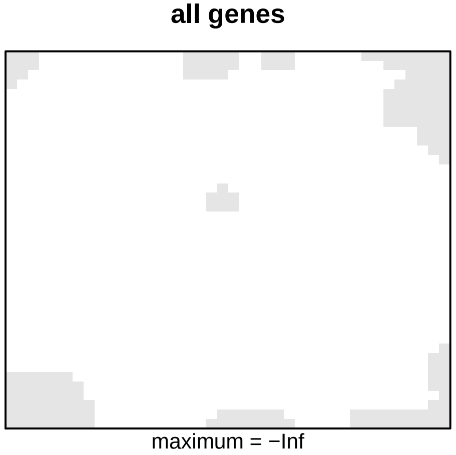

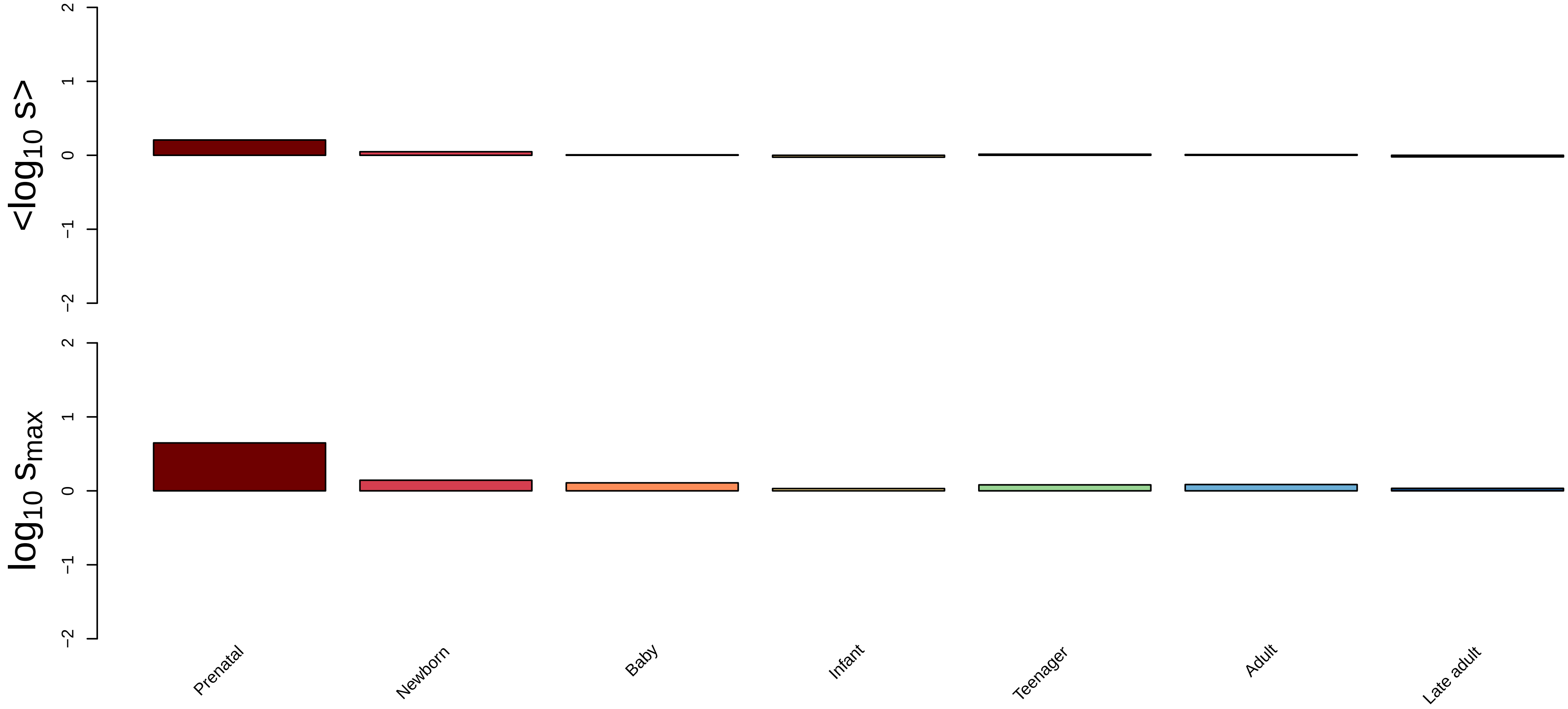

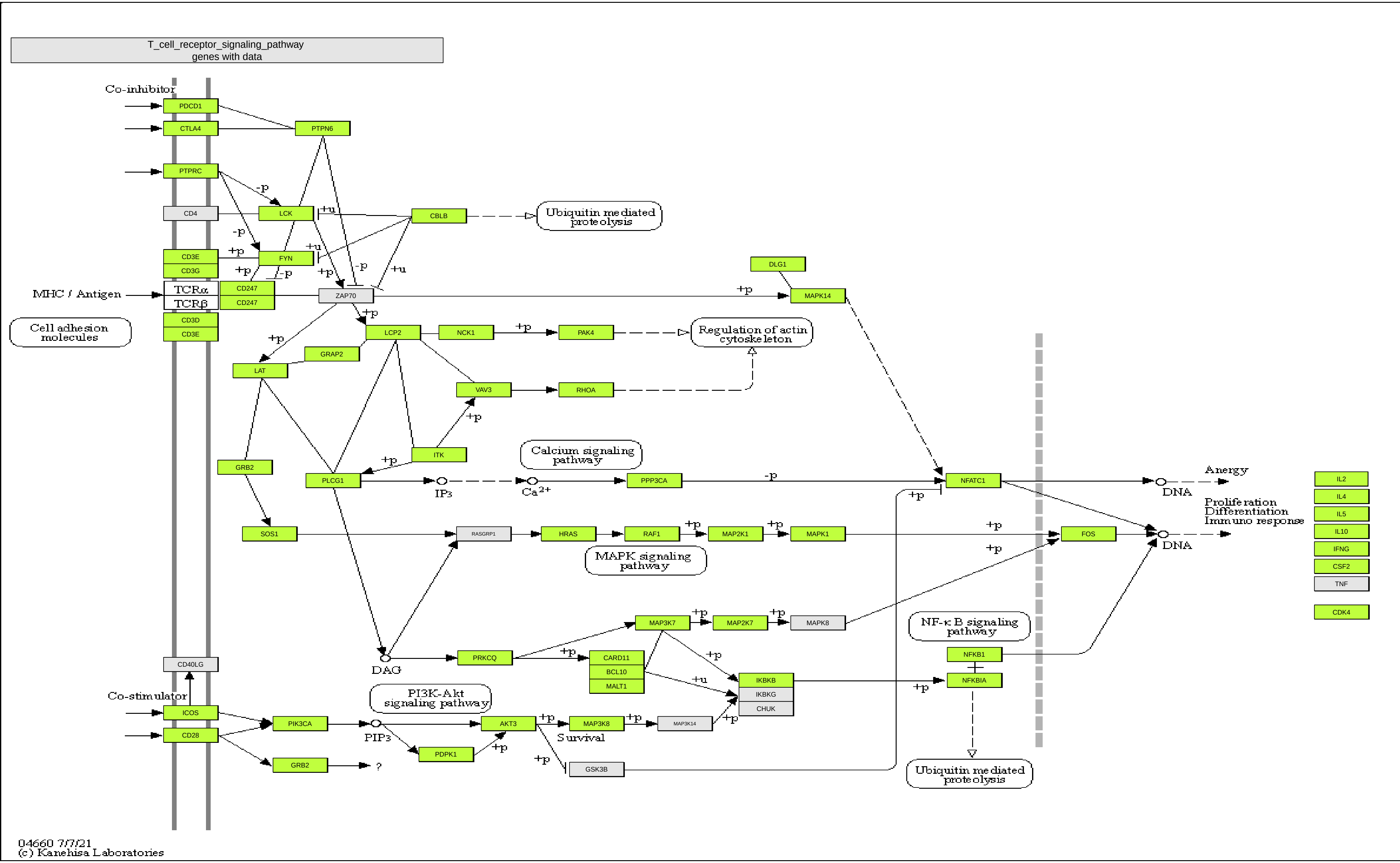

T_cell_receptor_signaling_pathway
genes with data
Co-inhibitor
PDCD1
CTLA4
PTPN6
PTPRC
CD4
LCK
CBLB
Ubiquitin mediated proteolysis
CD3E
CD3G
FYN
MHC / Antigen
TCRα
TCRβ
CD247
CD247
ZAP70
DLG1
MAPK14
CD3D
CD3E
Cell adhesion molecules
LCP2
NCK1
PAK4
Regulation of actin cytoskeleton
GRAP2
LAT
VAV3
RHOA
ITK
Calcium signaling pathway
GRB2
PLCG1
IP3
Ca2+
PPP3CA
NFATC1
Anergy
DNA
Proliferation
Differentiation
Immuno response
IL2
IL4
IL5
IL10
IFNG
CSF2
TNF
CDK4
SOS1
RASGRP1
HRAS
RAF1
MAP2K1
MAPK1
FOS
DNA
MAPK signaling pathway
MAP3K7
MAP2K7
MAPK8
NF-κB signaling pathway
NFKB1
NFKBIA
DAG
PRKCQ
CARD11
BCL10
MALT1
IKBKB
IKBKG
CHUK
CD40LG
Co-stimulator
PI3K-Akt signaling pathway
ICOS
CD28
PIK3CA
PIP3
AKT3
MAP3K8
MAP3K14
Survival
PDPK1
GSK3B
GRB2
?
Ubiquitin mediated proteolysis
04660 7/7/21
(c) Kanehisa Laboratories

T_cell_receptor_signaling_pathway
sink nodes

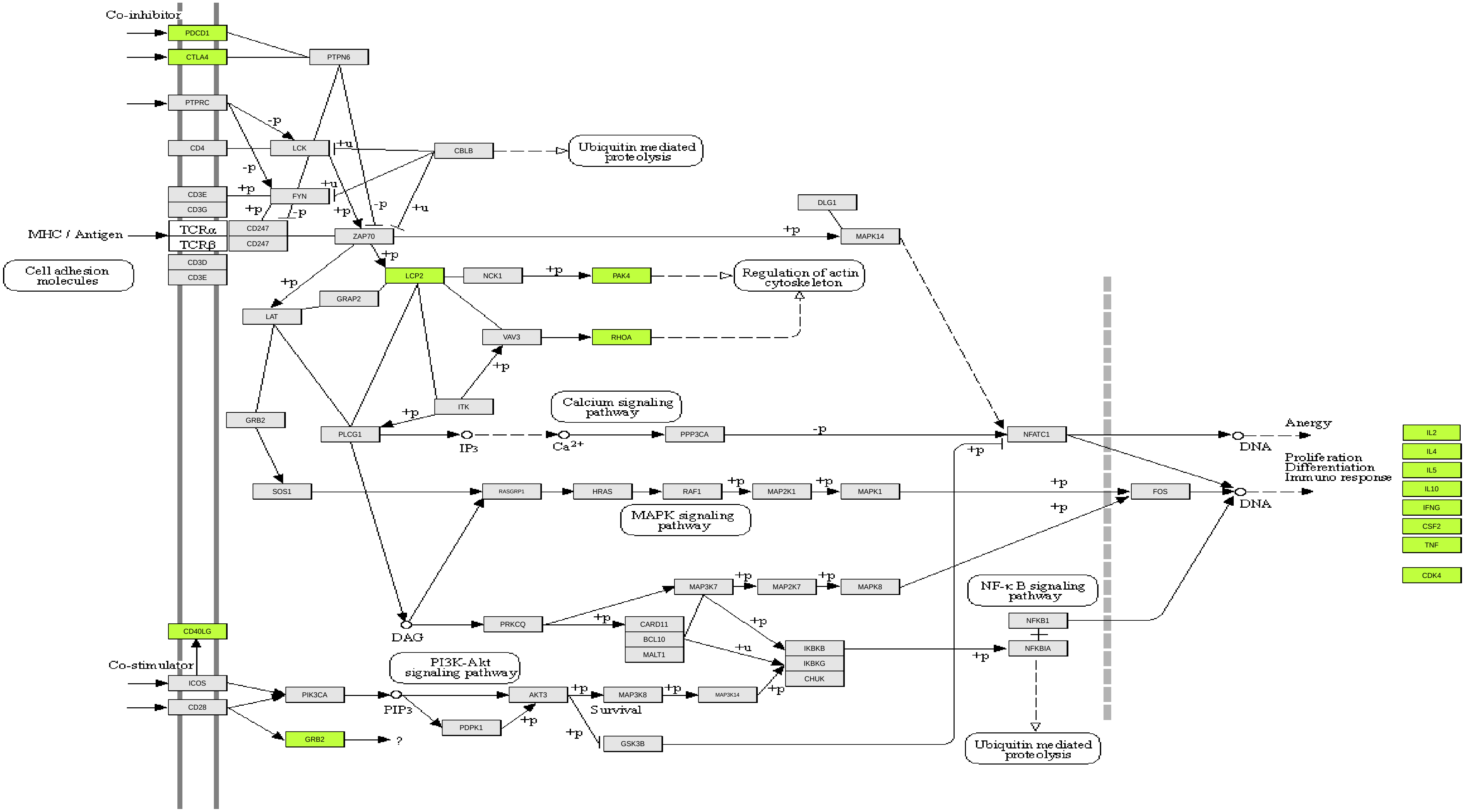

04660 7/7/21
(c) Kanehisa Laboratories

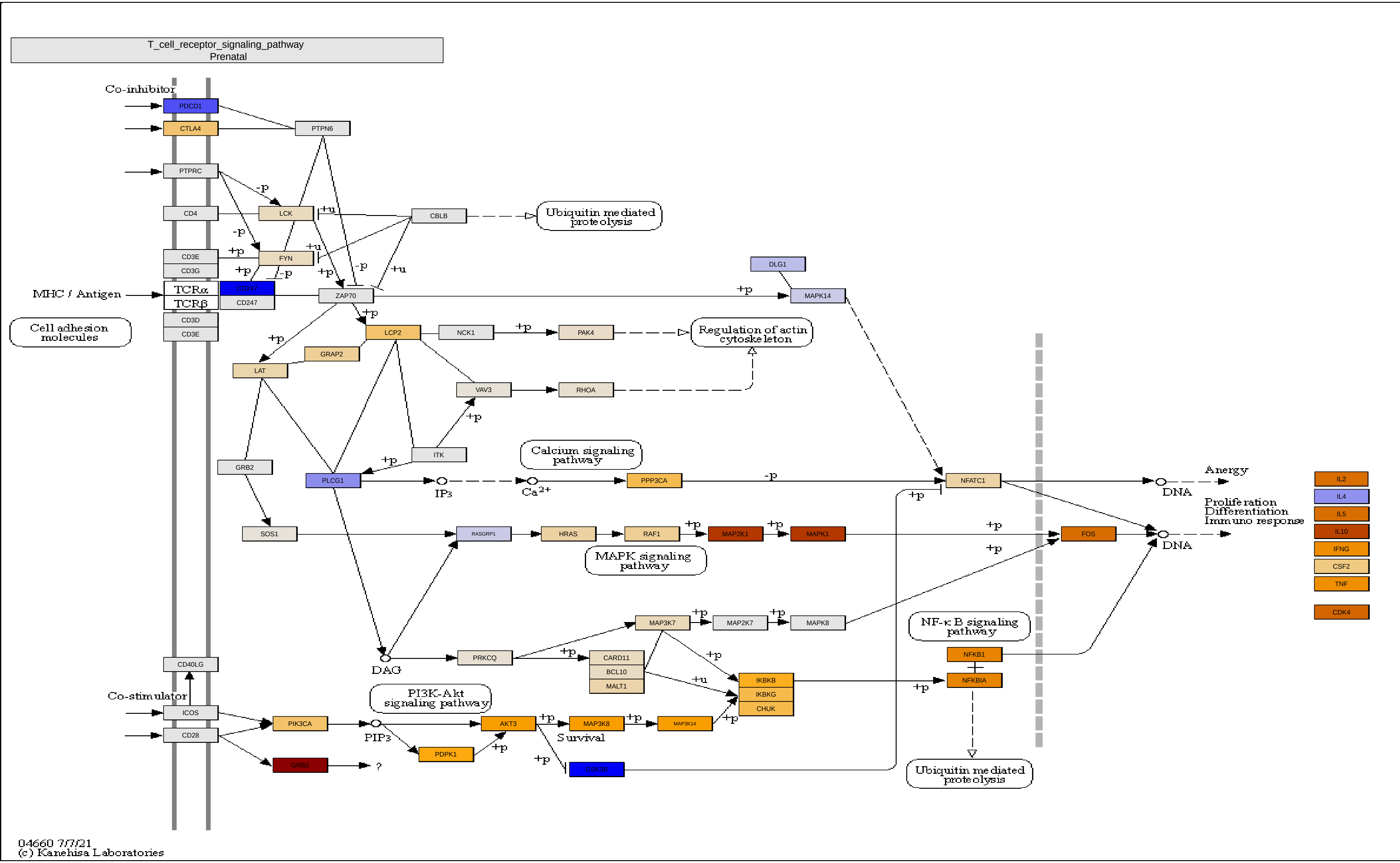
T_cell_receptor_signaling_pathway
Prenatal
Co-inhibitor
PDCD1
CTLA4
PTPN6
PTPRC
CD4
LCK
CBLB
Ubiquitin mediated proteolysis
CD3E
CD3G
FYN
DLG1
MHC / Antigen
TCRα
TCRβ
CD247
CD247
ZAP70
MAPK14
CD3D
CD3E
Cell adhesion molecules
LCP2
NCK1
PAK4
Regulation of actin cytoskeleton
GRAP2
LAT
VAV3
RHOA
ITK
Calcium signaling pathway
GRB2
PLCG1
IP3
Ca2+
PPP3CA
NFATC1
DNA
Anergy
Proliferation
Differentiation
Immuno response
IL2
IL4
IL5
IL10
IFNG
CSF2
TNF
CDK4
SOS1
RASGRP1
HRAS
RAF1
MAP2K1
MAPK1
FOS
MAPK signaling pathway
MAP3K7
MAP2K7
MAPK8
NF-κB signaling pathway
NFKB1
NFKBIA
CD40LG
PRKCQ
CARD11
BCL10
MALT1
IKBKB
IKBKG
CHUK
DAG
PI3K-Akt signaling pathway
Co-stimulator
ICOS
CD28
PIK3CA
PIP3
AKT3
MAP3K8
MAP3K14
Survival
PDPK1
GRB2
GSK3B
Ubiquitin mediated proteolysis
04660 7/7/21
(c) Kanehisa Laboratories

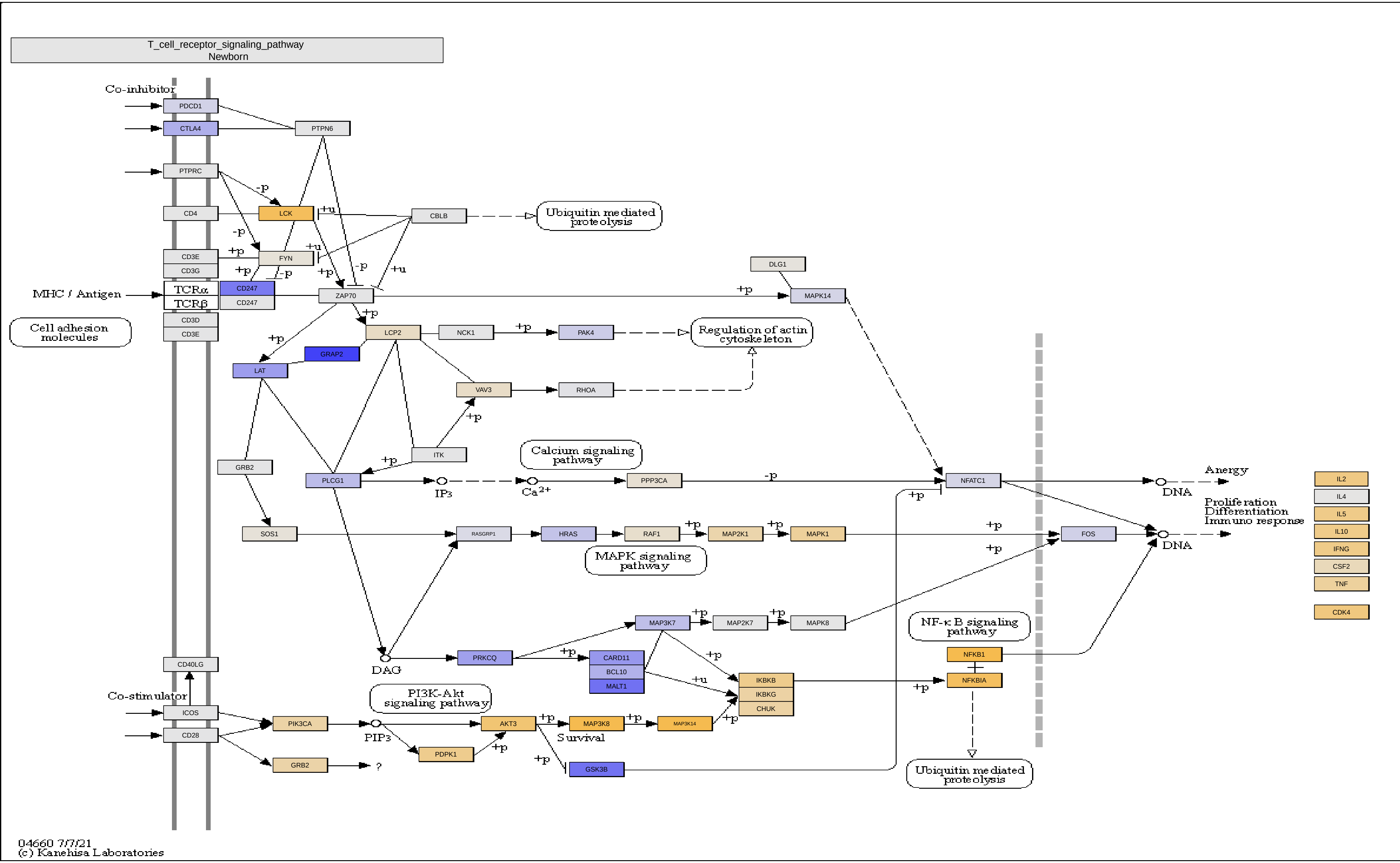
T_cell_receptor_signaling_pathway
Newborn
Co-inhibitor
PDCD1
CTLA4
PTPN6
PTPRC
-p
LCK
CD4
+u
CBLB
Ubiquitin mediated proteolysis
-p
+p
CD3E
FYN
+u
CD3G
+p
-p
+p
-p
+u
DLG1
MHC / Antigen
TCRα
TCRβ
CD247
CD247
ZAP70
+p
MAPK14
CD3D
CD3E
Cell adhesion molecules
+p
+p
LCP2
NCK1
+p
PAK4
Regulation of actin cytoskeleton
GRAP2
LAT
VAV3
RHOA
+p
ITK
Calcium signaling pathway
+p
GRB2
PLCG1
IP3
Ca2+
PPP3CA
-p
NFATC1
+p
Anergy
DNA
Proliferation
Differentiation
Immuno response
IL2
IL4
IL5
IL10
IFNG
CSF2
TNF
CDK4
SOS1
RASGRP1
HRAS
RAF1
+p
MAP2K1
+p
MAPK1
+p
FOS
DNA
+p
MAPK signaling pathway
MAP3K7
+p
MAP2K7
+p
MAPK8
NF-κB signaling pathway
CD40LG
+p
DAG
PRKCQ
+p
CARD11
BCL10
MALT1
+u
IKBKB
IKBKG
CHUK
NFKB1
NFKBIA
+p
Co-stimulator
PI3K-Akt signaling pathway
ICOS
PIK3CA
PIP3
AKT3
+p
MAP3K8
+p
MAP3K14
+p
Survival
CD28
PDPK1
+p
+p
GRB2
?
GSK3B
Ubiquitin mediated proteolysis
04660 7/7/21
(c) Kanehisa Laboratories

T_cell_receptor_signaling_pathway
Baby

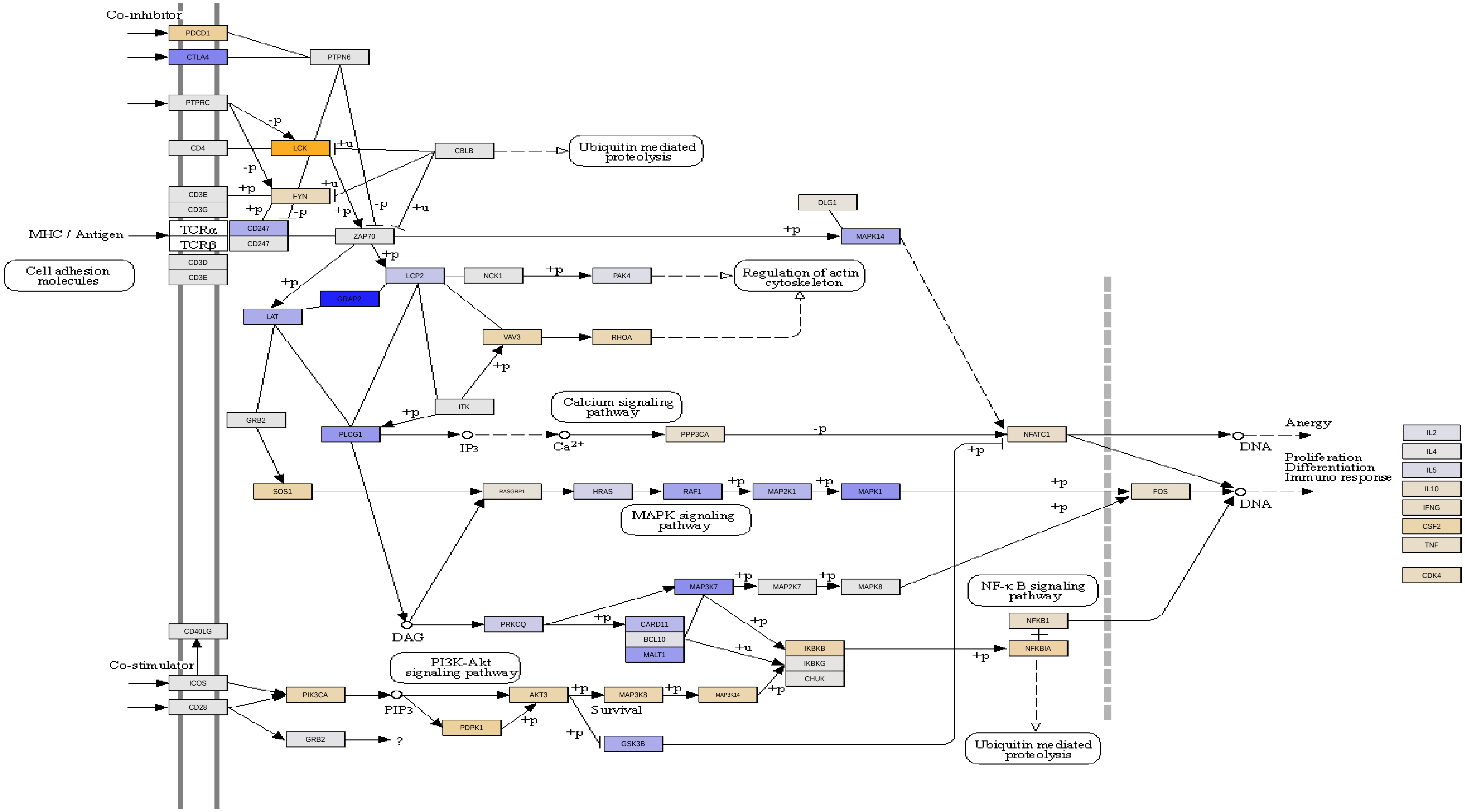

04660 7/7/21
(c) Kanehisa Laboratories

T_cell_receptor_signaling_pathway
Infant

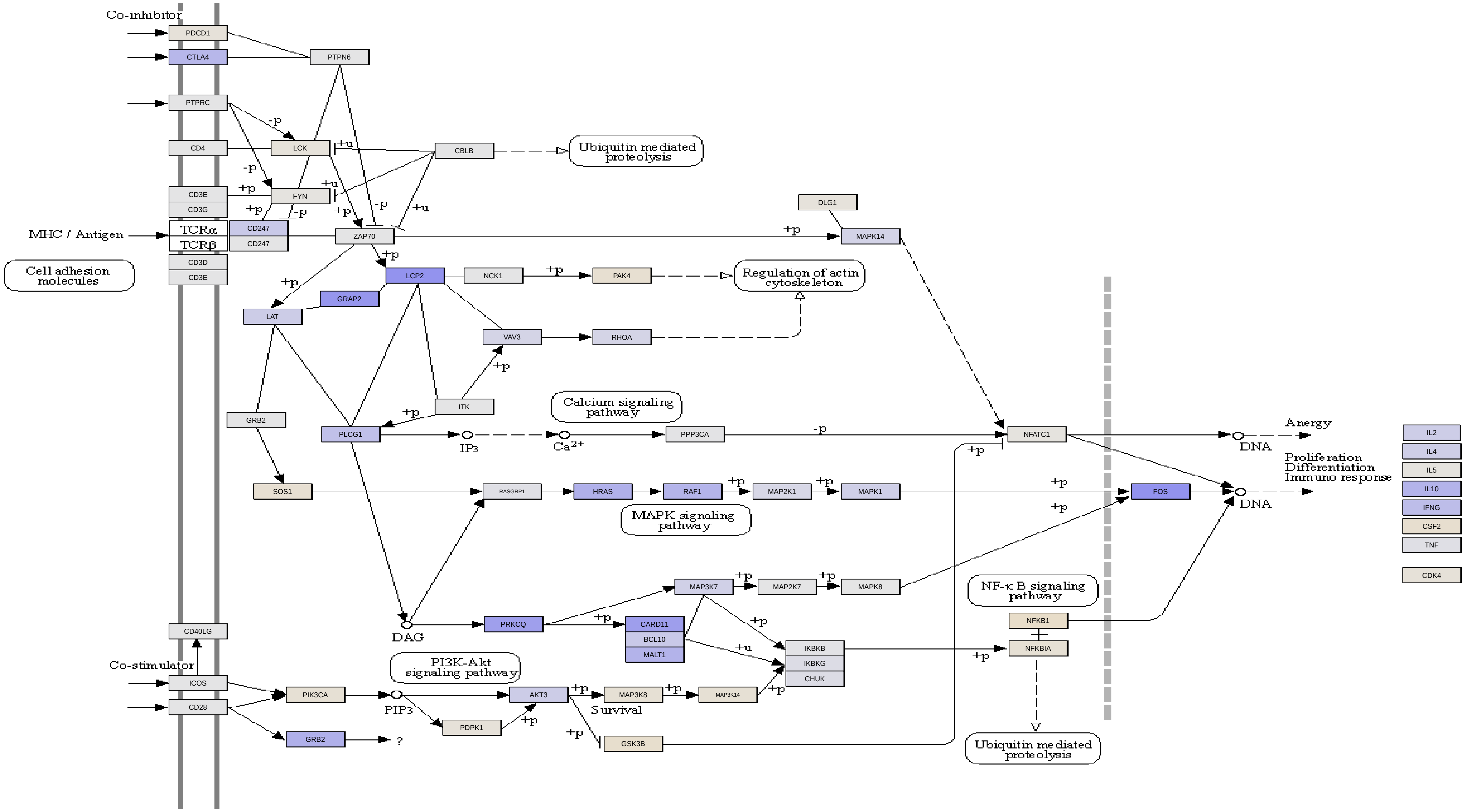

04660 7/7/21
(c) Kanehisa Laboratories

T_cell_receptor_signaling_pathway
Teenager

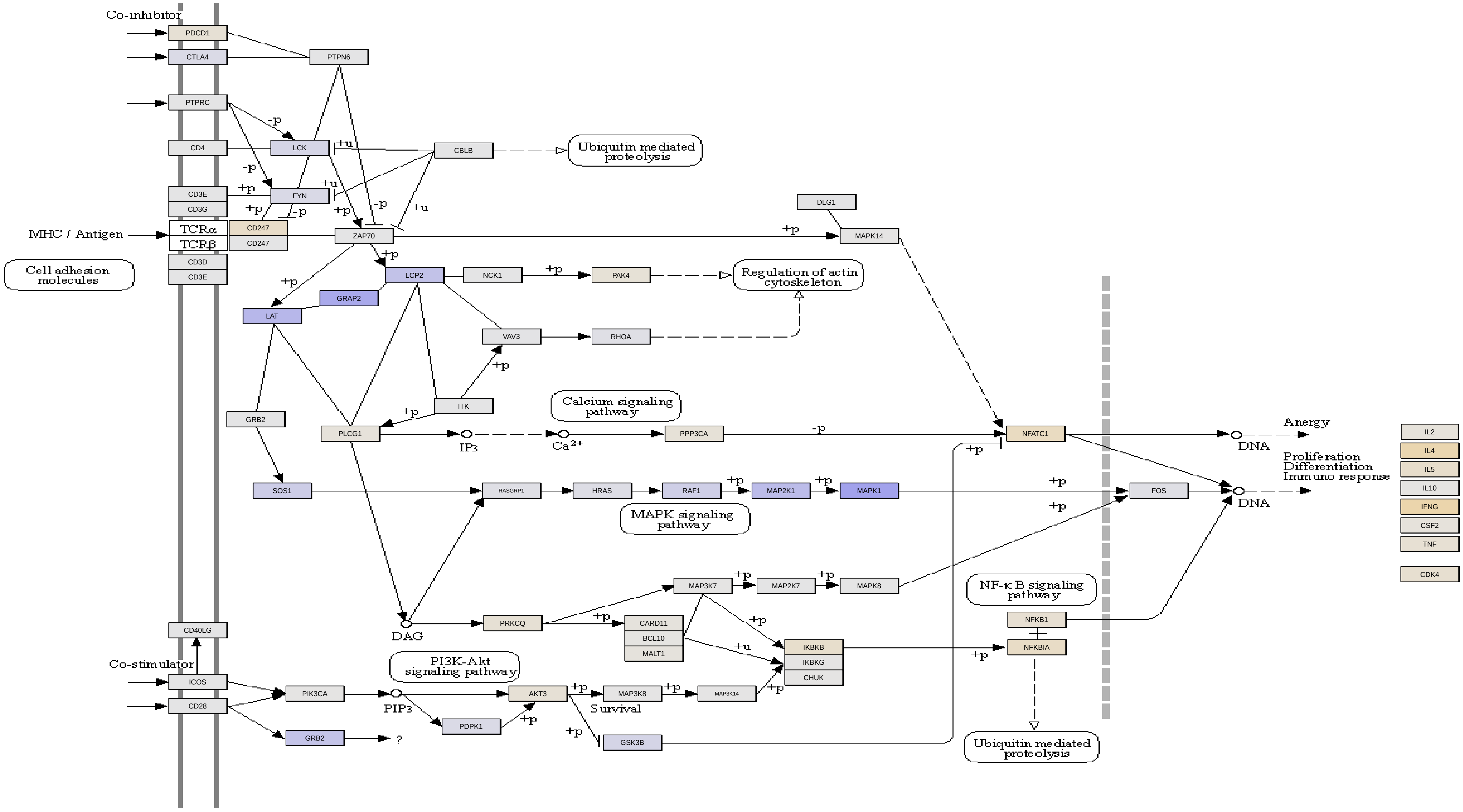

04660 7/7/21
(c) Kanehisa Laboratories

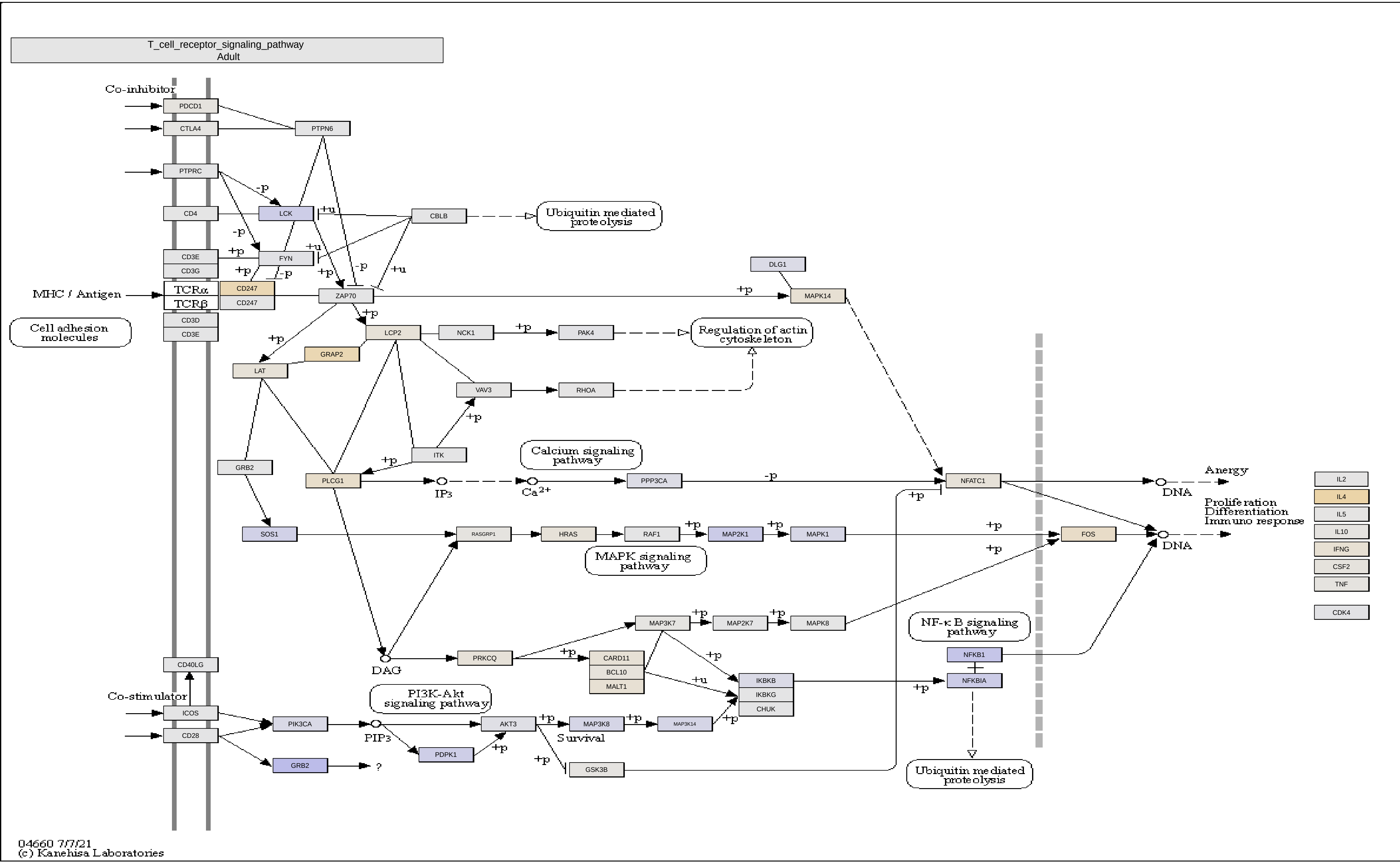
T_cell_receptor_signaling_pathway
Adult
Co-inhibitor
PDCD1
CTLA4
PTPN6
PTPRC
CD4
LCK
CBLB
Ubiquitin mediated proteolysis
CD3E
CD3G
FYN
MHC / Antigen
TCRα
TCRβ
CD247
CD247
ZAP70
DLG1
MAPK14
CD3D
CD3E
Cell adhesion molecules
LCP2
NCK1
PAK4
Regulation of actin cytoskeleton
GRAP2
LAT
VAV3
RHOA
ITK
GRB2
Calcium signaling pathway
PLCG1
IP3
Ca2+
PPP3CA
NFATC1
Anergy
DNA
Proliferation
Differentiation
Immuno response
SOS1
RASGRP1
HRAS
RAF1
MAP2K1
MAPK1
FOS
MAPK signaling pathway
MAP3K7
MAP2K7
MAPK8
NF-κB signaling pathway
NFKB1
NFKBIA
DAG
PRKCQ
CARD11
BCL10
MALT1
IKBKB
IKBKG
CHUK
CD40LG
Co-stimulator
PI3K-Akt signaling pathway
ICOS
CD28
PIK3CA
PIP3
AKT3
MAP3K8
MAP3K14
Survival
PDPK1
GRB2
?
GSK3B
Ubiquitin mediated proteolysis
IL2
IL4
IL5
IL10
IFNG
CSF2
TNF
CDK4
04660 7/7/21
(c) Kanehisa Laboratories

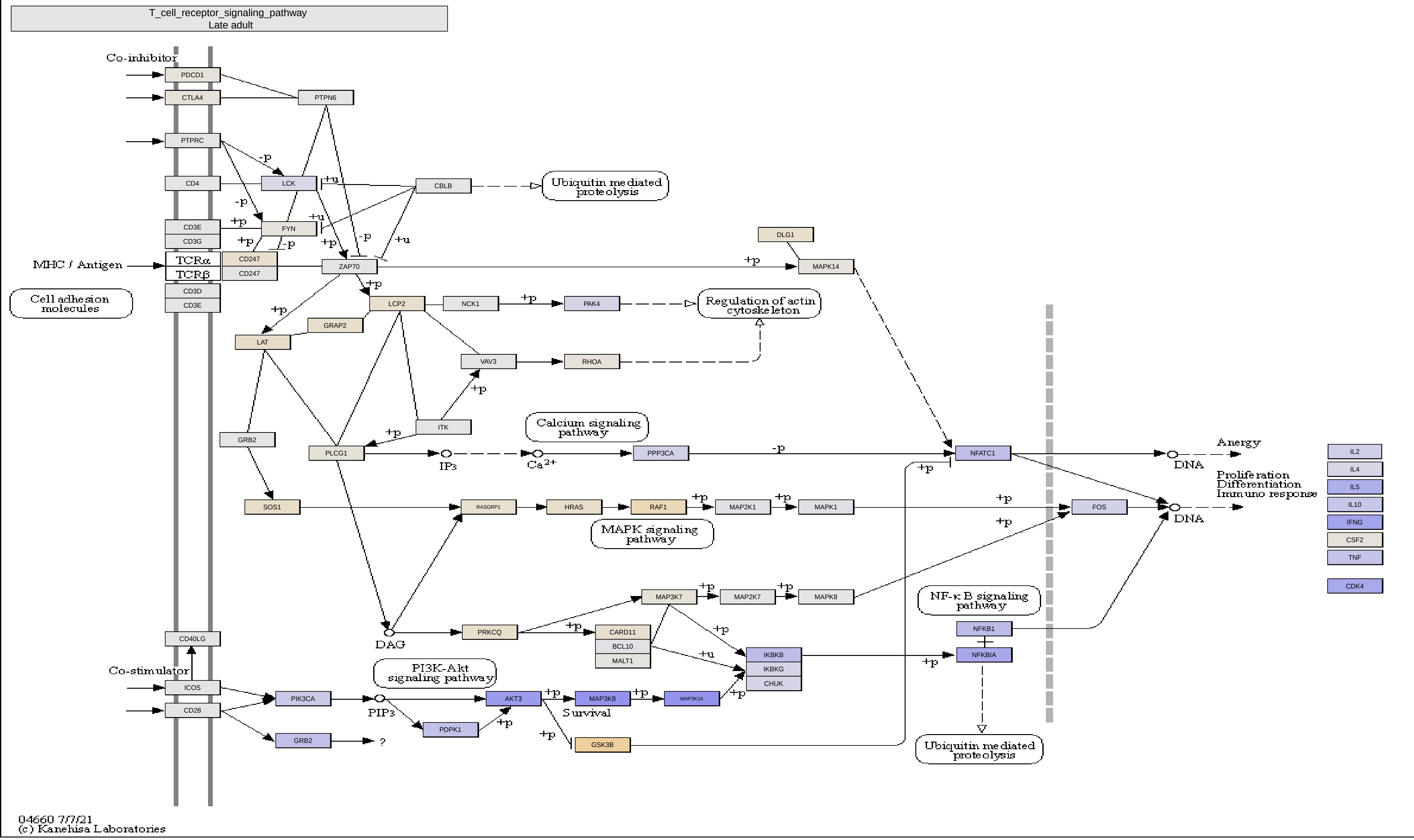

T_cell_receptor_signaling_pathway
Late adult
Co-inhibitor
PDCD1
CTLA4
PTPN6
PTPRC
CD4
LCK
CBLB
Ubiquitin mediated proteolysis
CD3E
CD3G
FYN
MHC / Antigen
TCRα
TCRβ
CD247
CD247
ZAP70
DLG1
MAPK14
CD3D
CD3E
Cell adhesion molecules
LCP2
NCK1
PAK4
Regulation of actin cytoskeleton
GRAP2
LAT
VAV3
RHOA
ITK
Calcium signaling pathway
GRB2
PLCG1
IP3
Ca2+
PPP3CA
NFATC1
Anergy
DNA
Proliferation
Differentiation
Immuno response
SOS1
RASGRP1
HRAS
RAF1
MAP2K1
MAPK1
FOS
MAPK signaling pathway
IL2
IL4
IL5
IL10
IFNG
CSF2
TNF
CDK4
MAP3K7
MAP2K7
MAPK8
NF-κB signaling pathway
DAG
PRKCQ
CARD11
BCL10
MALT1
NFKB1
IKBKB
IKBKG
CHUK
NFKBIA
CD40LG
Co-stimulator
ICOS
CD28
PI3K-Akt signaling pathway
PIK3CA
PIP3
AKT3
MAP3K8
MAP3K14
Survival
PDPK1
GRB2
GSK3B
Ubiquitin mediated proteolysis
04660 7/7/21
(c) Kanehisa Laboratories
