# Supplementary material for: The Transcriptome and Methylome of the Developing and Aging Brain and Their Relations to Gliomas and Psychological Disorders
Source: Cells. 2022 Jan 21;11(3):362. doi: 10.3390/cells11030362 (PMC8834030; doi:10.3390/cells11030362)
Supplement: Supplementary file 1 [file cells-11-00362-s001.zip › Suppl-Material-S4-Pathways-PSF_Methylation/TGF_beta_signaling_pathway.pdf]

TGF−beta signaling pathway

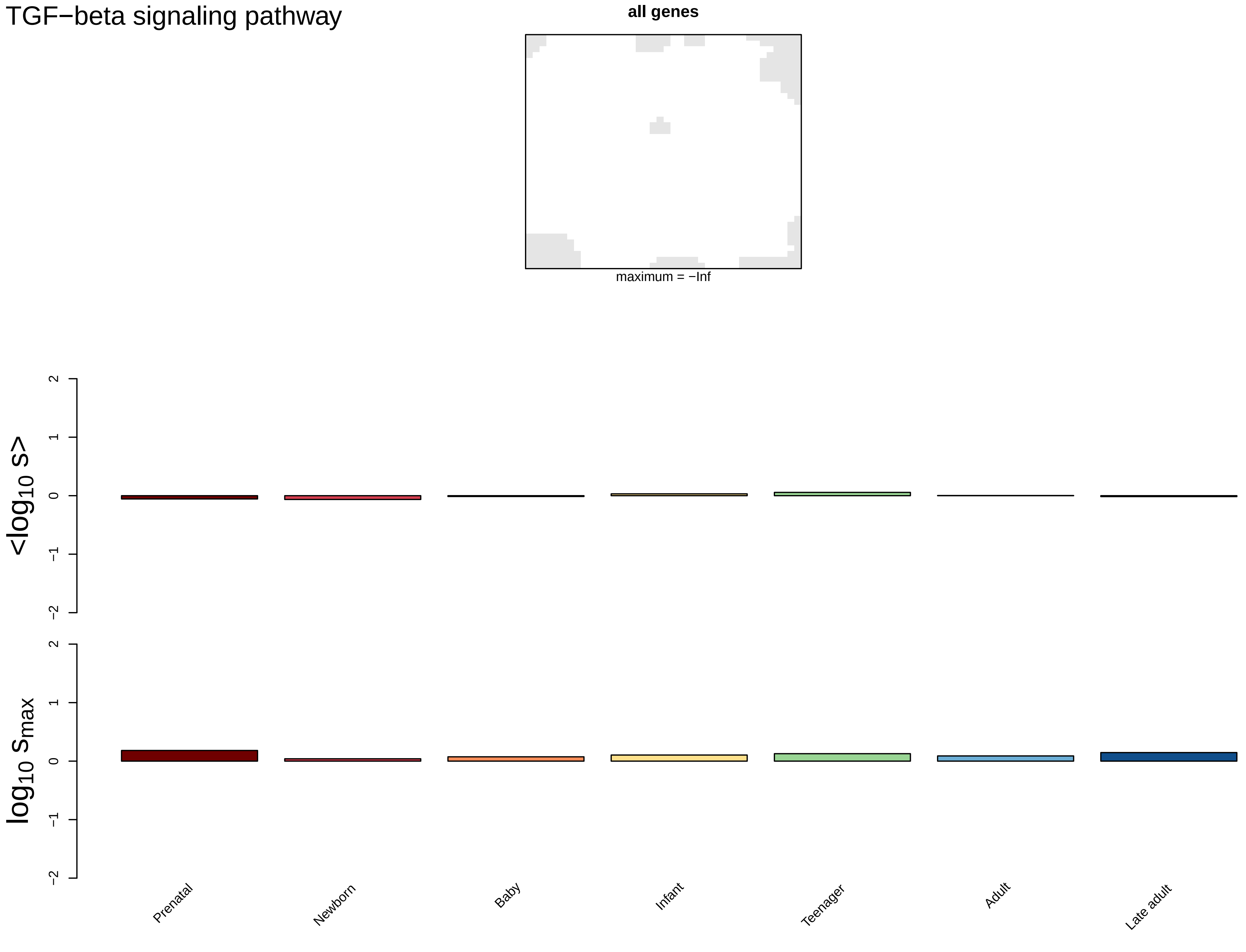

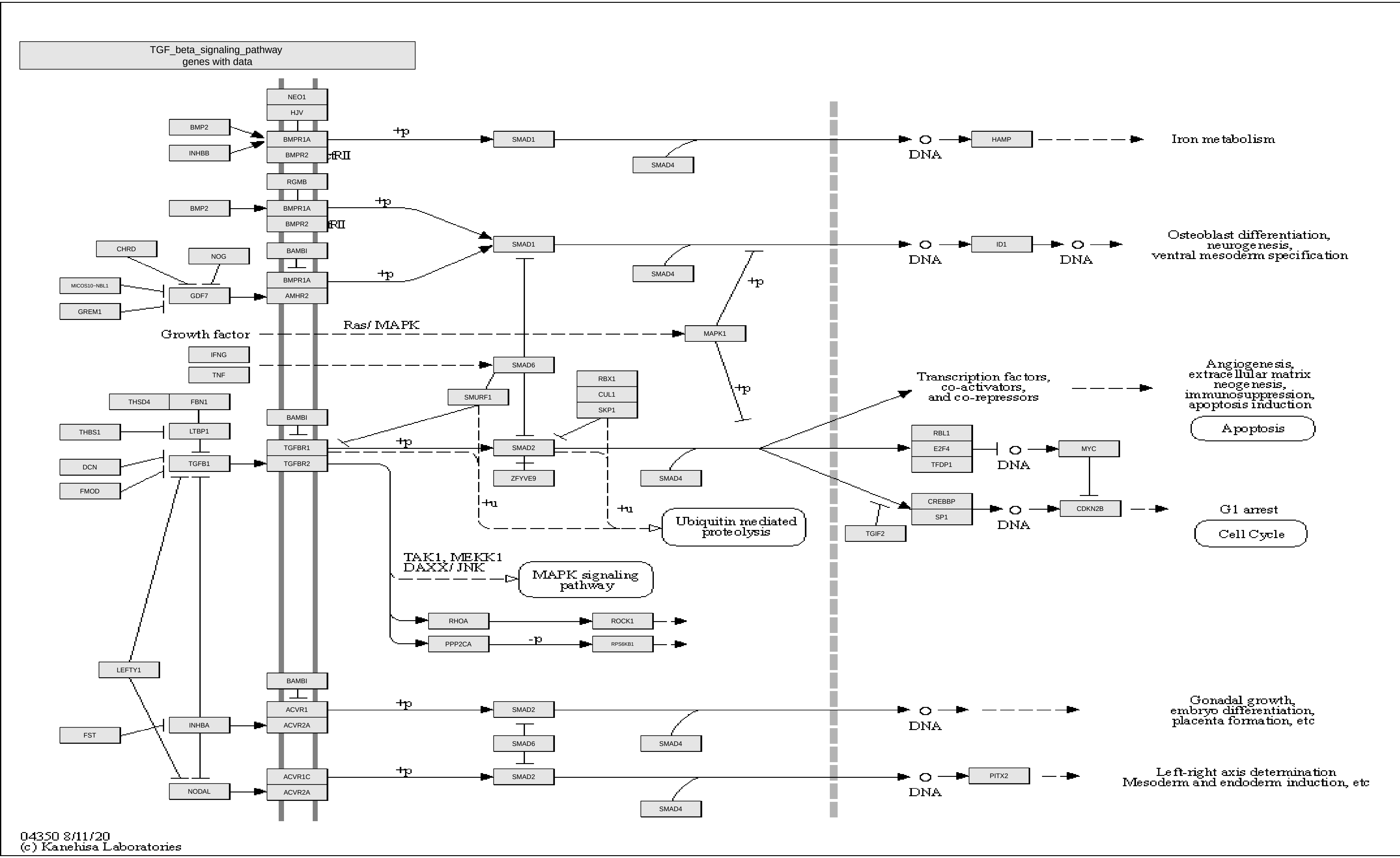
TGF_beta_signaling_pathway
genes with data
NEO1
HJV
BMP2
INHBB
BMPR1A
BMPR2
+p
SMAD1
SMAD4
DNA
HAMP
Iron metabolism
RGMB
BMP2
BMPR1A
BMPR2
+p
SMAD1
SMAD4
DNA
ID1
DNA
Osteoblast differentiation,
neurogenesis,
ventral mesoderm specification
CHRD
NOG
BAMBI
MICOS10-NBL1
GDF7
BMPR1A
AMHR2
+p
GREM1
+p
Growth factor
Ras/ MAPK
MAPK1
+p
IFNG
TNF
SMAD6
RBX1
CUL1
SKP1
SMURF1
Transcription factors,
co-activators,
and co-repressors
Angiogenesis,
extracellular matrix
neogenesis,
immunosuppression,
apoptosis induction
Apoptosis
THSD4
FBN1
BAMBI
THBS1
LTBP1
TGFBR1
TGFBR2
+p
SMAD2
RBL1
E2F4
TFDP1
DNA
MYC
DCN
TGFB1
ZFYVE9
SMAD4
FMOD
CREBBP
SP1
DNA
CDKN2B
G1 arrest
Cell Cycle
+u
+u
Ubiquitin mediated
proteolysis
TGIF2
TAK1, MEKK1
DAXX/ JNK
MAPK signaling
pathway
RHOA
ROCK1
PPP2CA
-p
RPS6KB1
LEFTY1
BAMBI
INHBA
ACVR1
ACVR2A
+p
SMAD2
DNA
Gonadal growth,
embryo differentiation,
placenta formation, etc
FST
SMAD6
SMAD4
NODAL
ACVR1C
ACVR2A
+p
SMAD2
DNA
PITX2
Left-right axis determination
Mesoderm and endoderm induction, etc
SMAD4
04350 8/11/20
(c) Kanehisa Laboratories

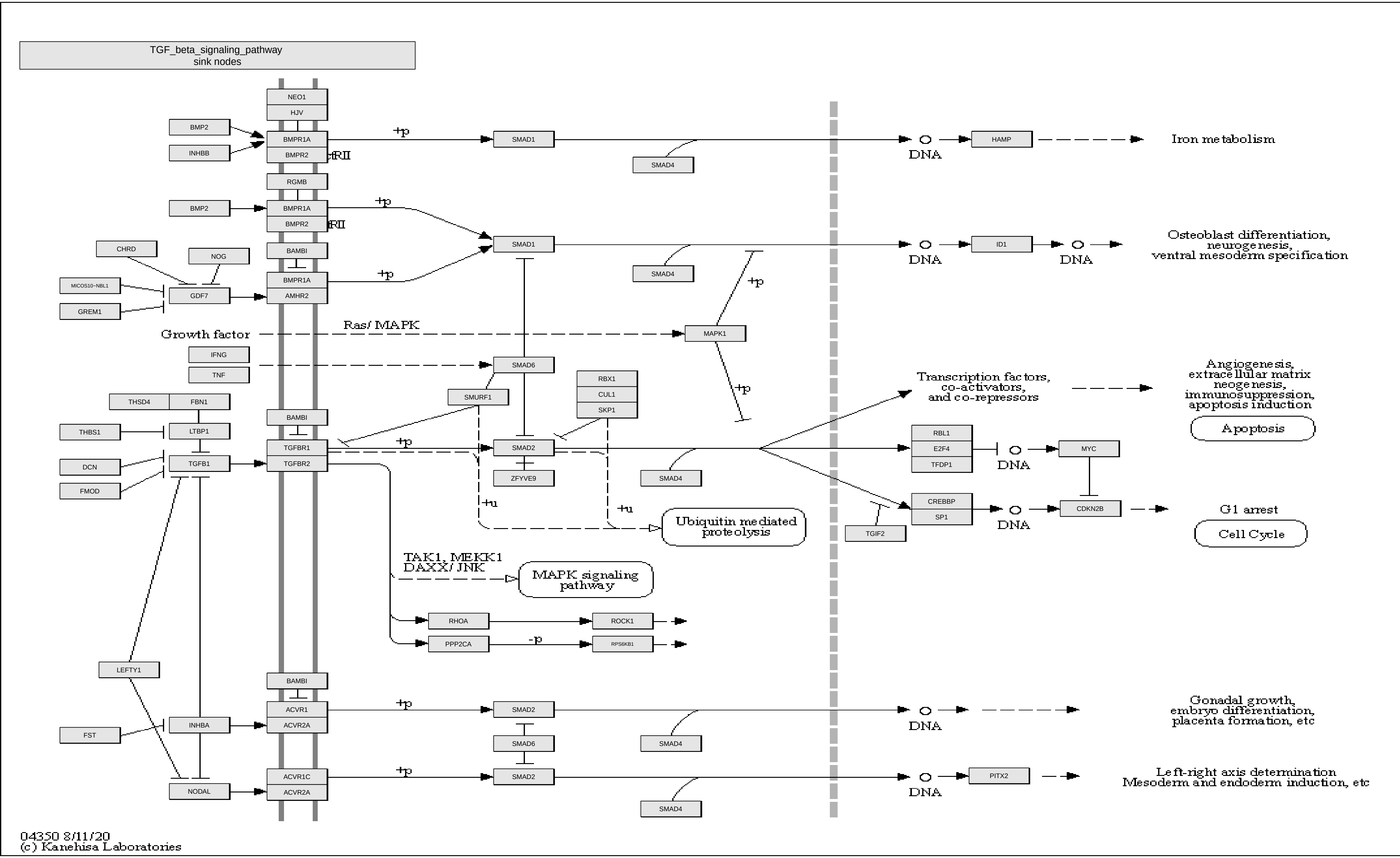
TGF_beta_signaling_pathway
sink nodes
NEO1
HJV
BMP2
INHBB
BMPR1A
BMPR2
RGMB
BAMBI
AMHR2
CHRD
NOG
MICOS10−NBL1
GREM1
GDF7
+p
SMAD1
SMAD4
DNA
HAMP
Iron metabolism
ID1
Osteoblast differentiation,
neurogenesis,
ventral mesoderm specification
Growth factor
Ras/ MAPK
MAPK1
IFNG
TNF
SMAD6
SMURF1
RBX1
CUL1
SKP1
THSD4
FBN1
THBS1
LTBP1
DCN
FMOD
TGFB1
TGFBR1
TGFBR2
SMAD2
ZFYVE9
+u
Ubiquitin mediated
proteolysis
Transcription factors,
co-activators,
and co-repressors
Angiogenesis,
extracellular matrix
neogenesis,
immunosuppression,
apoptosis induction
Apoptosis
RBL1
E2F4
TFDP1
MYC
CREBBP
SP1
TGIF2
CDKN2B
G1 arrest
Cell Cycle
TAK1, MEKK1
DAXX/ JNK
MAPK signaling
pathway
RHOA
ROCK1
PPP2CA
-p
RPS6KB1
LEFTY1
INHBA
FST
NODAL
ACVR1
ACVR2A
ACVR1C
Gonadal growth,
embryo differentiation,
placenta formation, etc
PITX2
Left-right axis determination
Mesoderm and endoderm induction, etc
04350 8/11/20
(c) Kanehisa Laboratories

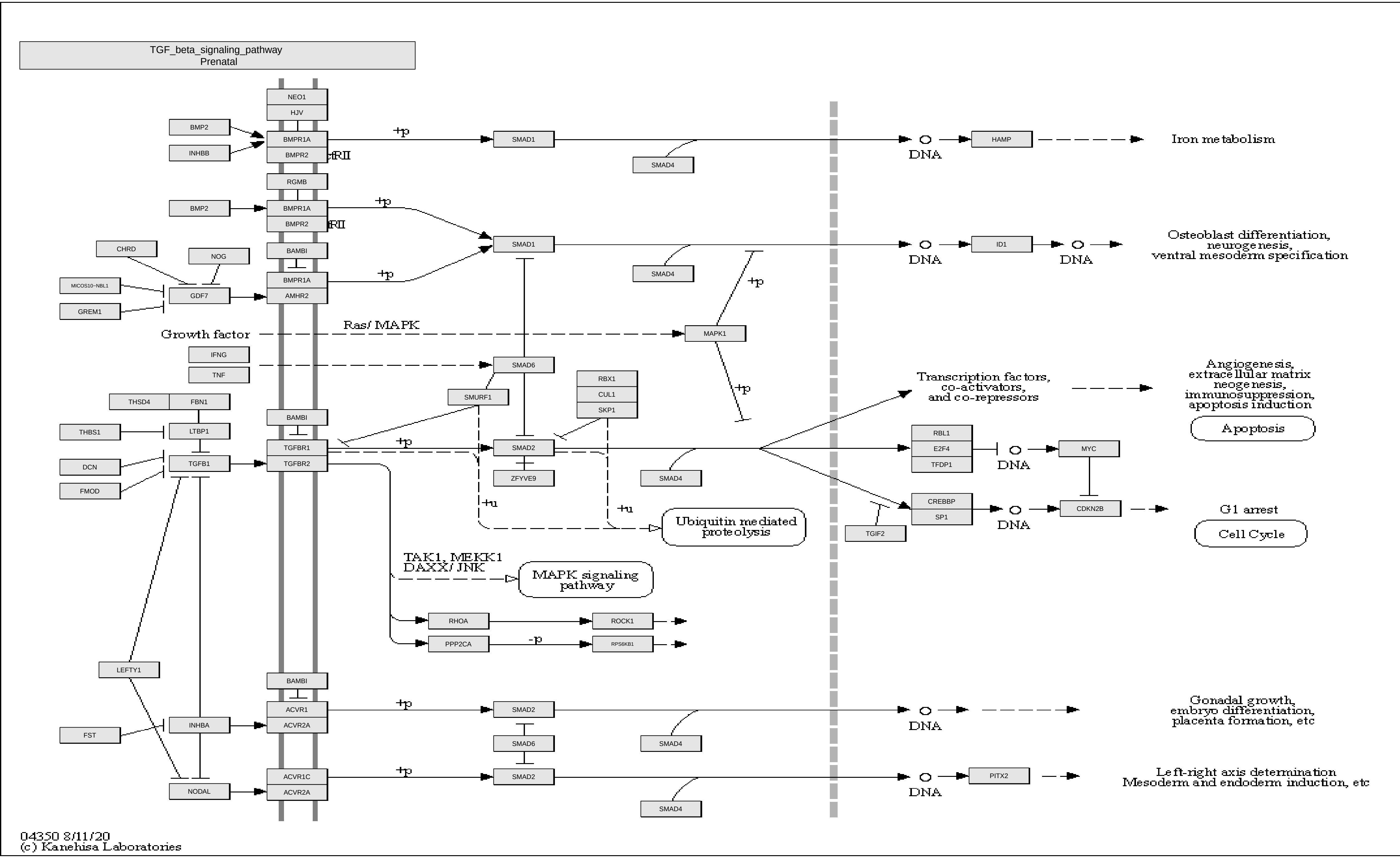
TGF_beta_signaling_pathway
Prenatal
NEO1
HJV
BMP2
INHBB
BMPR1A
BMPR2
+p
SMAD1
SMAD4
DNA
HAMP
Iron metabolism
RGMB
BMP2
BMPR1A
BMPR2
+p
SMAD1
SMAD4
DNA
ID1
DNA
Osteoblast differentiation,
neurogenesis,
ventral mesoderm specification
CHRD
NOG
BAMBI
MICOS10-NBL1
GDF7
BMPR1A
AMHR2
GREM1
+p
+p
Growth factor
Ras/ MAPK
MAPK1
+p
IFNG
TNF
SMAD6
SMURF1
RBX1
CUL1
SKP1
Transcription factors,
co-activators,
and co-repressors
Angiogenesis,
extracellular matrix
neogenesis,
immunosuppression,
apoptosis induction
Apoptosis
THSD4
FBN1
BAMBI
THBS1
LTBP1
TGFBR1
TGFBR2
+p
SMAD2
ZFYVE9
SMAD4
RBL1
E2F4
TFDP1
DNA
MYC
DCN
TGFB1
FMOD
CREBBP
SP1
DNA
CDKN2B
G1 arrest
Cell Cycle
TGIF2
+u
+u
Ubiquitin mediated proteolysis
TAK1, MEKK1
DAXX/ JNK
MAPK signaling pathway
RHOA
ROCK1
PPP2CA
-p
RPS6KB1
LEFTY1
BAMBI
ACVR1
ACVR2A
+p
SMAD2
SMAD6
SMAD4
DNA
Gonadal growth,
embryo differentiation,
placenta formation, etc
INHBA
FST
NODAL
ACVR1C
ACVR2A
+p
SMAD2
SMAD4
DNA
PITX2
Left-right axis determination
Mesoderm and endoderm induction, etc
04350 8/11/20
(c) Kanehisa Laboratories

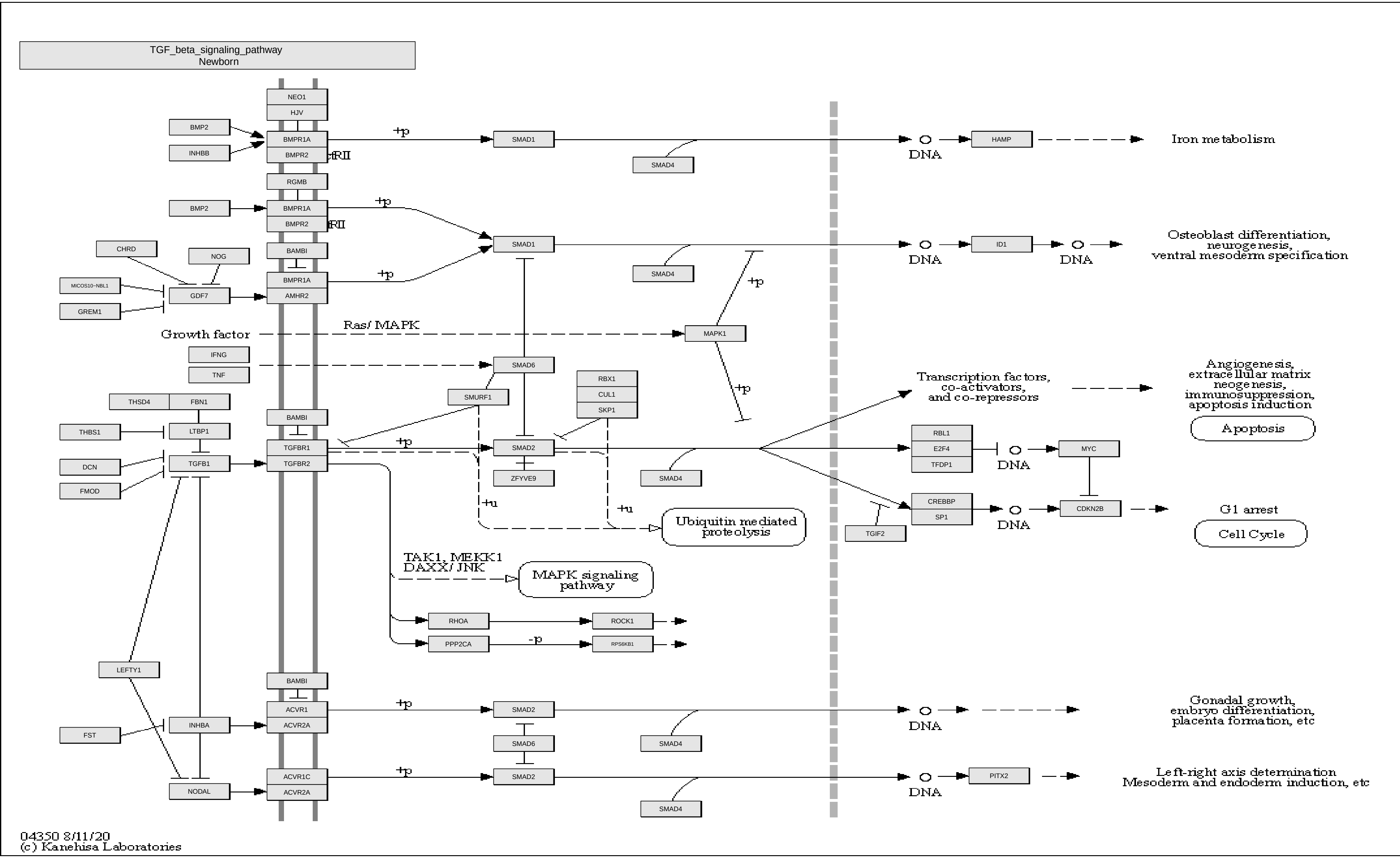
TGF_beta_signaling_pathway
Newborn
NEO1
HJV
BMP2
INHBB
BMPR1A
BMPR2
RGMB
BMP2
BMPR1A
BMPR2
BAMBI
CHRD
NOG
MICOS10−NBL1
GREM1
GDF7
BMPR1A
AMHR2
Growth factor
Ras/ MAPK
IFNG
TNF
THSD4
FBN1
THBS1
LTBP1
DCN
FMOD
TGFB1
BAMBI
TGFBR1
TGFBR2
SMAD1
SMAD4
SMAD1
SMAD4
MAPK1
SMAD6
SMURF1
RBX1
CUL1
SKP1
SMAD2
ZFYVE9
SMAD4
+p
+u
Ubiquitin mediated proteolysis
TAK1, MEKK1
DAXX/ JNK
MAPK signaling pathway
RHOA
ROCK1
PPP2CA
RPS6KB1
-p
LEFTY1
BAMBI
FST
INHBA
ACVR1
ACVR2A
SMAD2
SMAD6
SMAD4
NODAL
ACVR1C
ACVR2A
SMAD2
SMAD4
DNA
HAMP
Iron metabolism
ID1
Osteoblast differentiation,
neurogenesis,
ventral mesoderm specification
Transcription factors,
co-activators,
and co-repressors
Angiogenesis,
extracellular matrix
neogenesis,
immunosuppression,
apoptosis induction
Apoptosis
RBL1
E2F4
TFDP1
MYC
CREBBP
SP1
TGIF2
CDKN2B
G1 arrest
Cell Cycle
Gonadal growth,
embryo differentiation,
placenta formation, etc
PITX2
Left-right axis determination
Mesoderm and endoderm induction, etc
04350 8/11/20
(c) Kanehisa Laboratories

TGF_beta_signaling_pathway
Baby

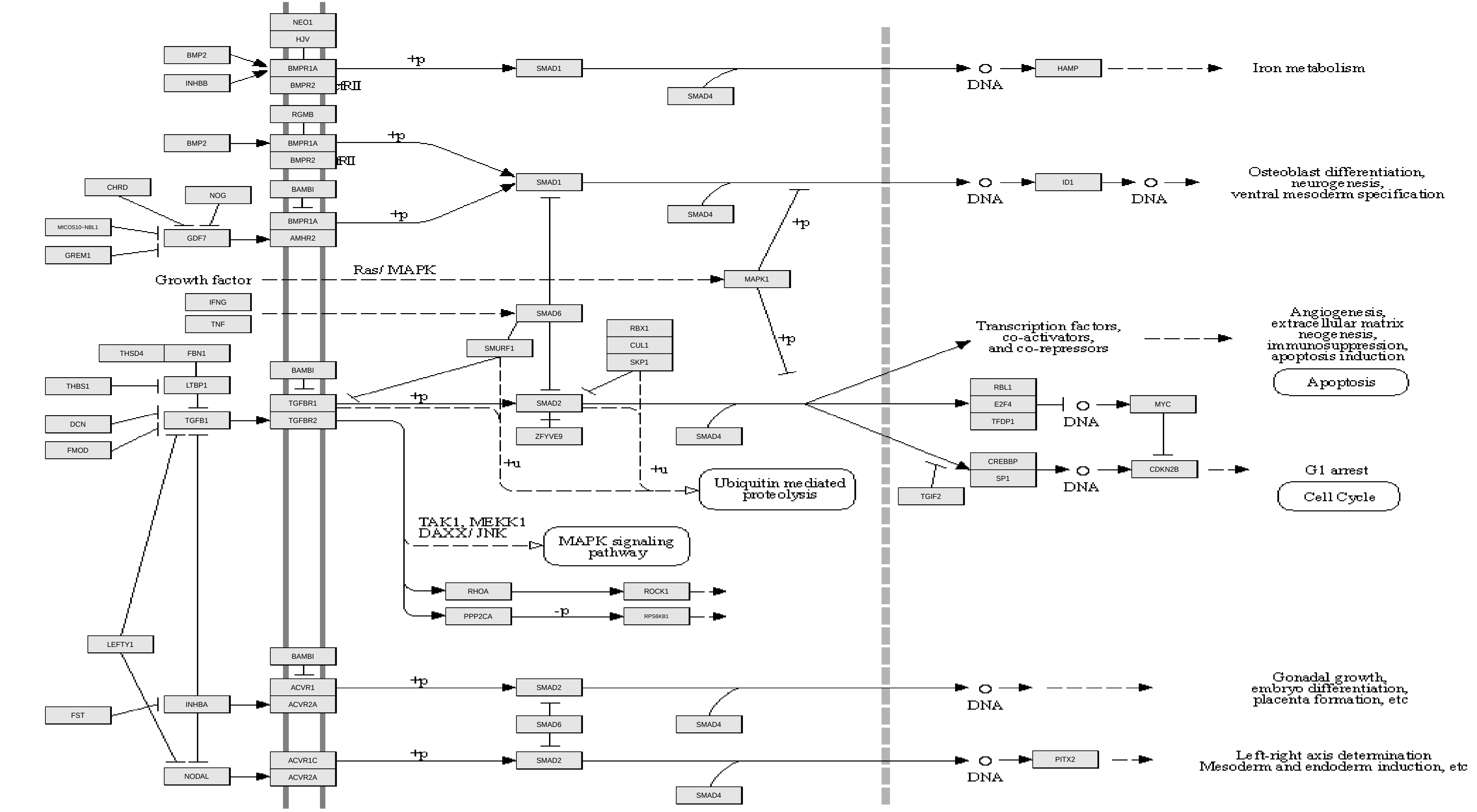

04350 8/11/20
(c) Kanehisa Laboratories

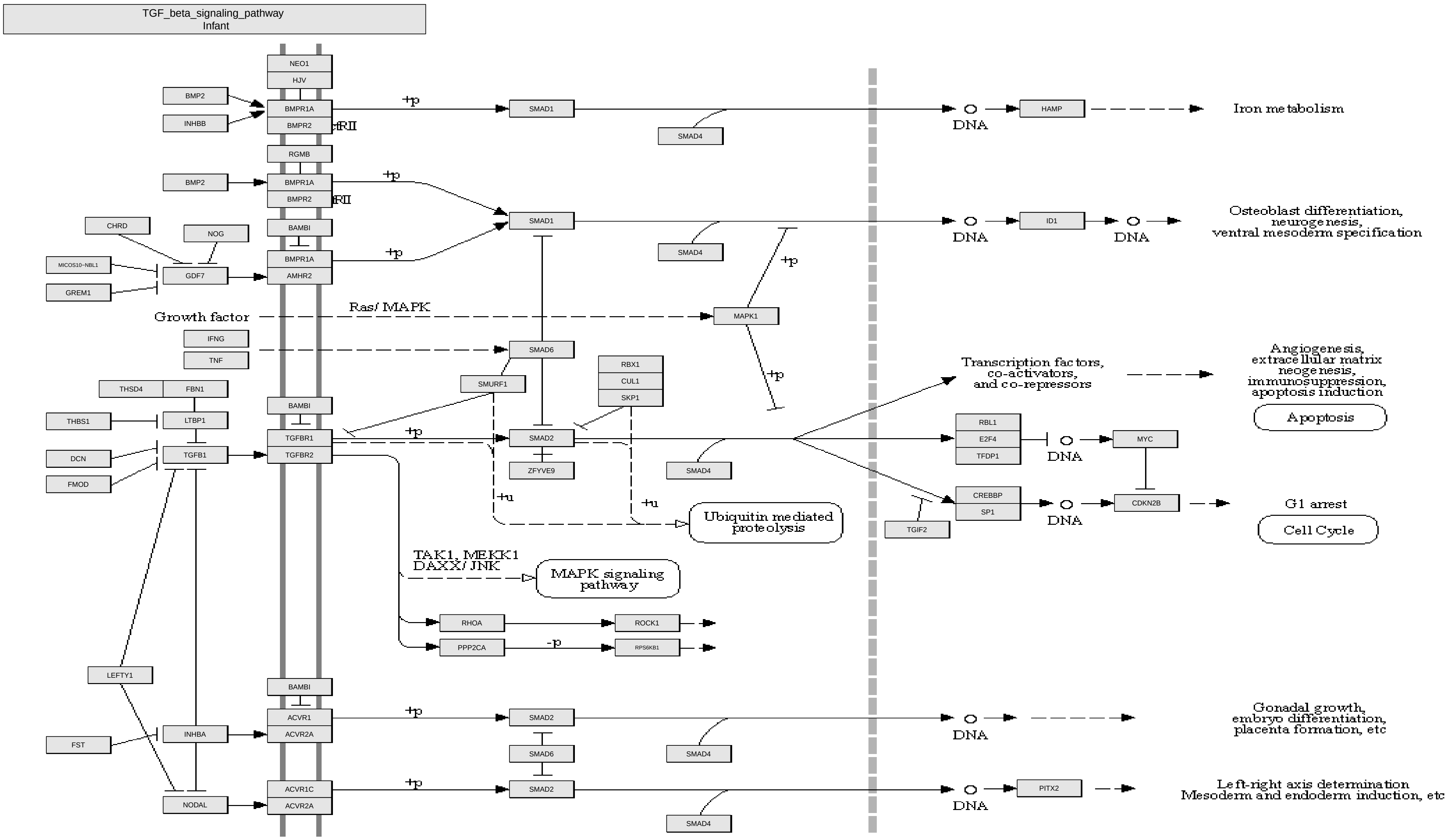

04350 8/11/20

(c) Kanehisa Laboratories

TGF_beta_signaling_pathway
Teenager

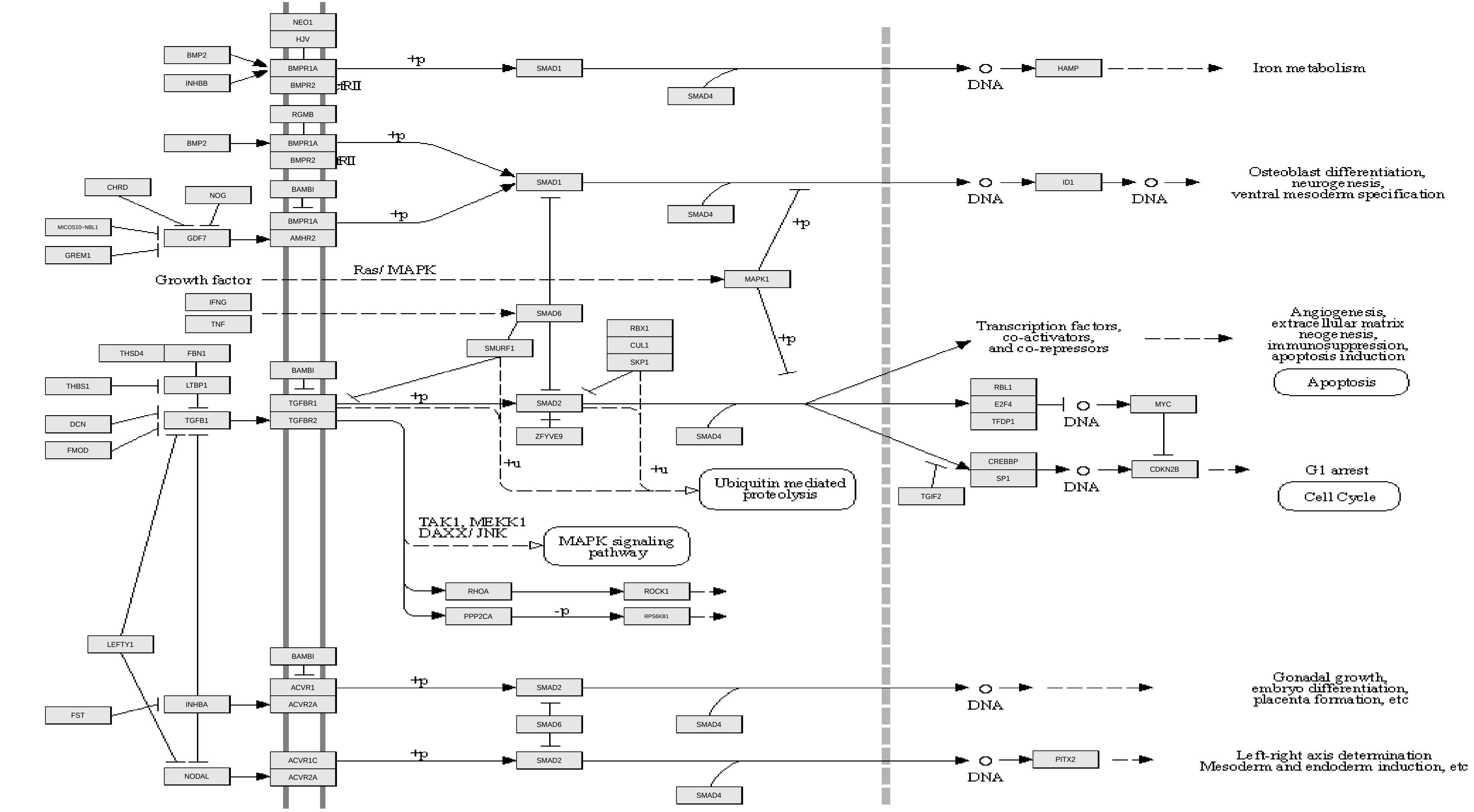

04350 8/11/20
(c) Kanehisa Laboratories

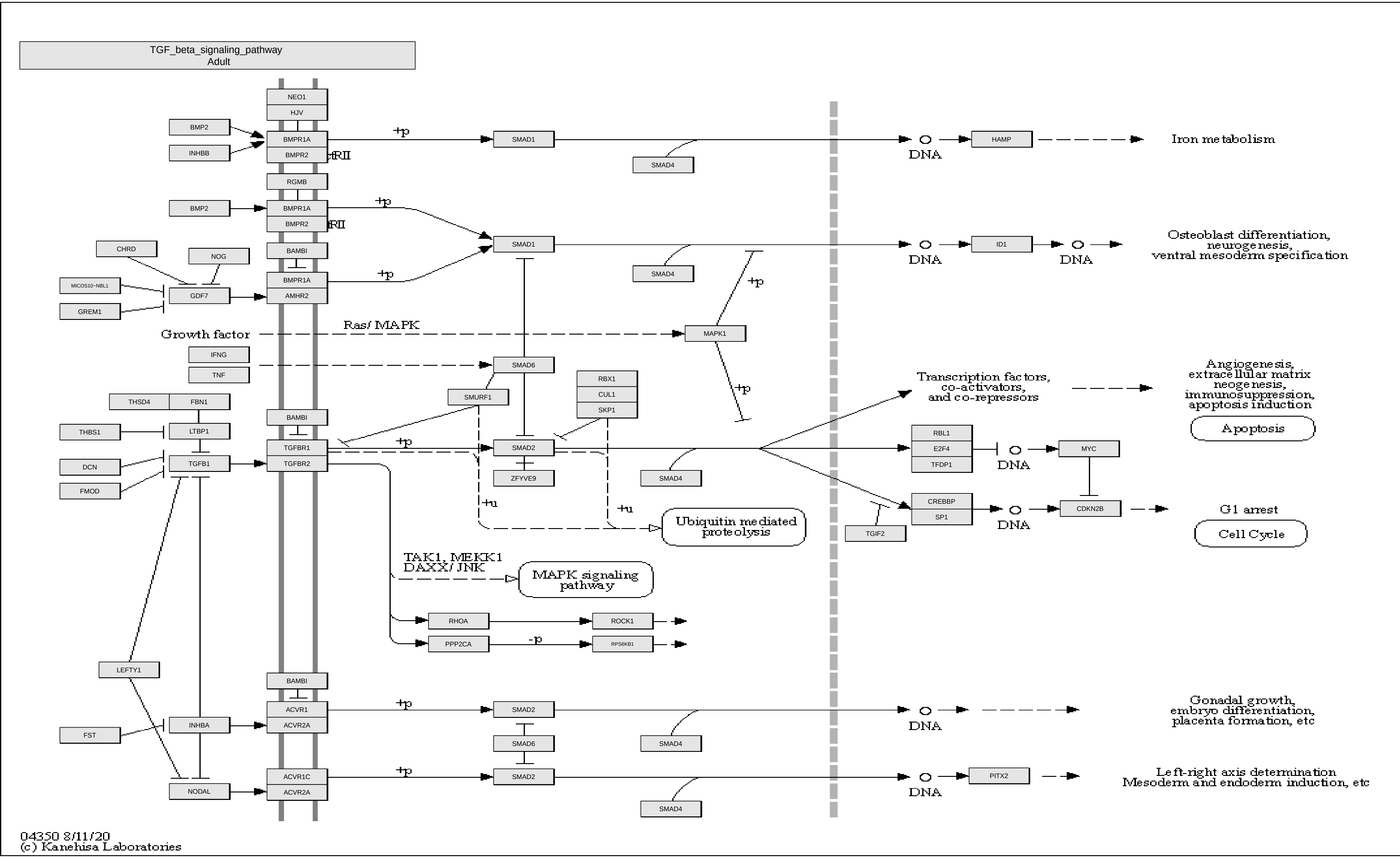
TGF_beta_signaling_pathway
Adult
NEO1
HJV
BMP2
INHBB
BMPR1A
BMPR2
+p
SMAD1
SMAD4
DNA
HAMP
Iron metabolism
RGMB
BMP2
BMPR1A
BMPR2
+p
SMAD1
SMAD4
DNA
ID1
DNA
Osteoblast differentiation, neurogenesis, ventral mesoderm specification
CHRD
NOG
BAMBI
MICOS10-NBL1
GREM1
GDF7
BMPR1A
AMHR2
+p
+p
Growth factor
Ras/ MAPK
MAPK1
+p
IFNG
TNF
SMAD6
SMURF1
RBX1
CUL1
SKP1
Transcription factors, co-activators, and co-repressors
Angiogenesis, extracellular matrix neogenesis, immunosuppression, apoptosis induction
Apoptosis
THSD4
FBN1
THBS1
LTBP1
BAMBI
TGFBR1
TGFBR2
+p
SMAD2
ZFYVE9
SMAD4
RBL1
E2F4
TFDP1
DNA
MYC
DCN
FMOD
TGFB1
+u
+u
Ubiquitin mediated proteolysis
CREBBP
SP1
DNA
CDKN2B
G1 arrest
Cell Cycle
TGIF2
TAK1, MEKK1
DAXX/ JNK
MAPK signaling pathway
RHOA
ROCK1
PPP2CA
-p
RPS6KB1
LEFTY1
BAMBI
INHBA
ACVR1
ACVR2A
+p
SMAD2
SMAD6
SMAD4
DNA
Gonadal growth, embryo differentiation, placenta formation, etc
FST
NODAL
ACVR1C
ACVR2A
+p
SMAD2
SMAD4
DNA
PITX2
Left-right axis determination
Mesoderm and endoderm induction, etc
04350 8/11/20
(c) Kanehisa Laboratories

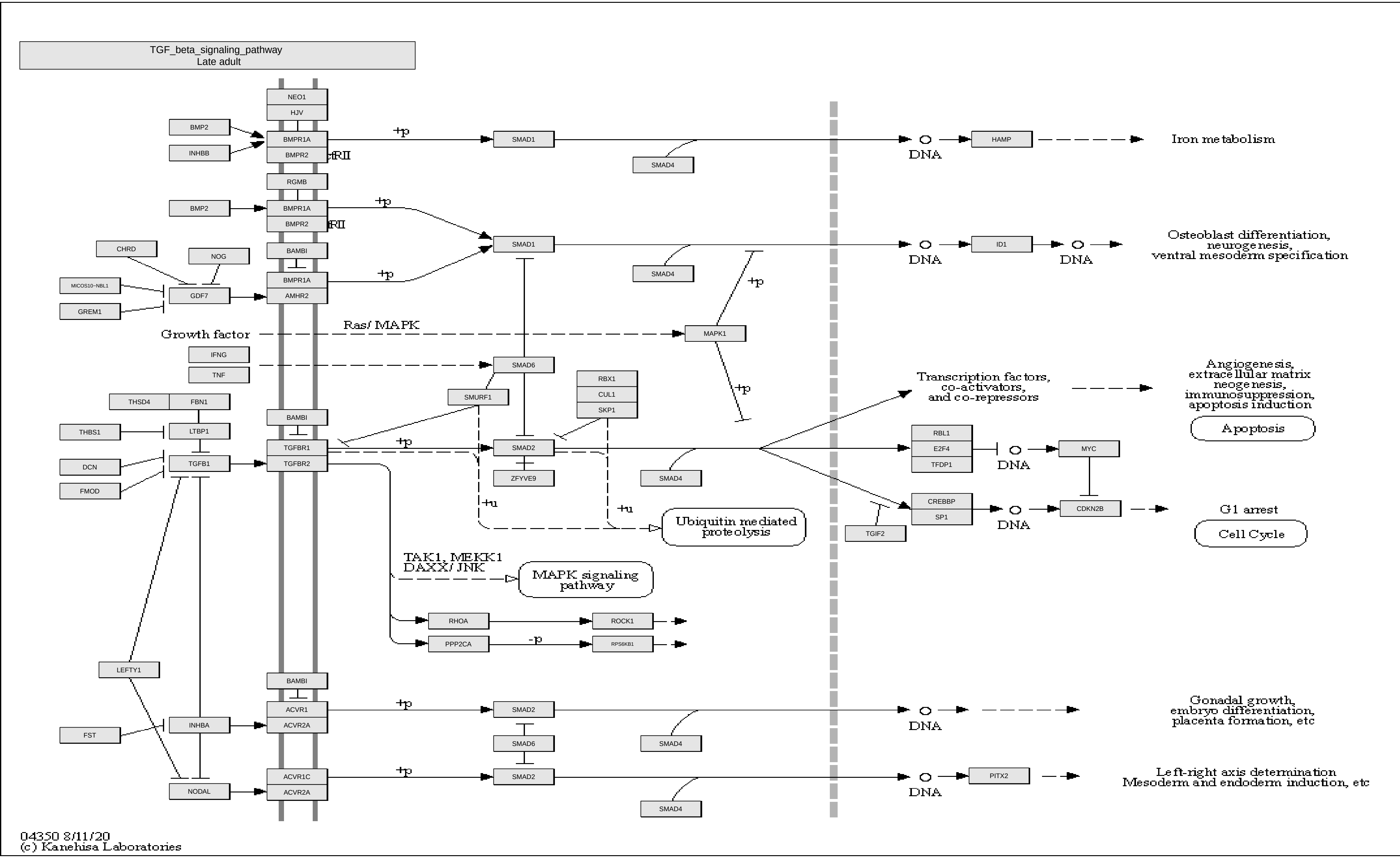
TGF_beta_signaling_pathway
Late adult
NEO1
HJV
BMP2
INHBB
BMPR1A
BMPR2
+p
SMAD1
SMAD4
DNA
HAMP
Iron metabolism
RGMB
BMP2
BMPR1A
BMPR2
BAMBI
CHRD
NOG
MICOS10-NBL1
GREM1
GDF7
BMPR1A
AMHR2
ID1
Osteoblast differentiation,
neurogenesis,
ventral mesoderm specification
Growth factor
Ras/ MAPK
MAPK1
IFNG
TNF
SMAD6
SMURF1
RBX1
CUL1
SKP1
Transcription factors,
co-activators,
and co-repressors
Angiogenesis,
extracellular matrix
neogenesis,
immunosuppression,
apoptosis induction
Apoptosis
THSD4
FBN1
THBS1
LTBP1
BAMBI
TGFBR1
TGFBR2
DCN
FMOD
TGFB1
SMAD2
ZFYVE9
RBL1
E2F4
TFDP1
MYC
CREBBP
SP1
CDKN2B
TGIF2
G1 arrest
Cell Cycle
+u
Ubiquitin mediated
proteolysis
TAK1, MEKK1
DAXX/ JNK
MAPK signaling
pathway
RHOA
ROCK1
PPP2CA
-p
RPS6KB1
LEFTY1
BAMBI
ACVR1
ACVR2A
INHBA
FST
Gonadal growth,
embryo differentiation,
placenta formation, etc
NODAL
ACVR1C
ACVR2A
PITX2
Left-right axis determination
Mesoderm and endoderm induction, etc
04350 8/11/20
(c) Kanehisa Laboratories
