# Supplementary material for: The Transcriptome and Methylome of the Developing and Aging Brain and Their Relations to Gliomas and Psychological Disorders
Source: Cells. 2022 Jan 21;11(3):362. doi: 10.3390/cells11030362 (PMC8834030; doi:10.3390/cells11030362)
Supplement: Supplementary file 1 [file cells-11-00362-s001.zip › Suppl-Material-S4-Pathways-PSF_Methylation/TNF_signaling_pathway.pdf]

TNF signaling pathway

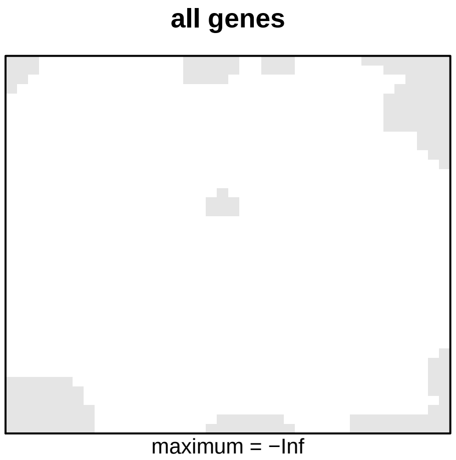

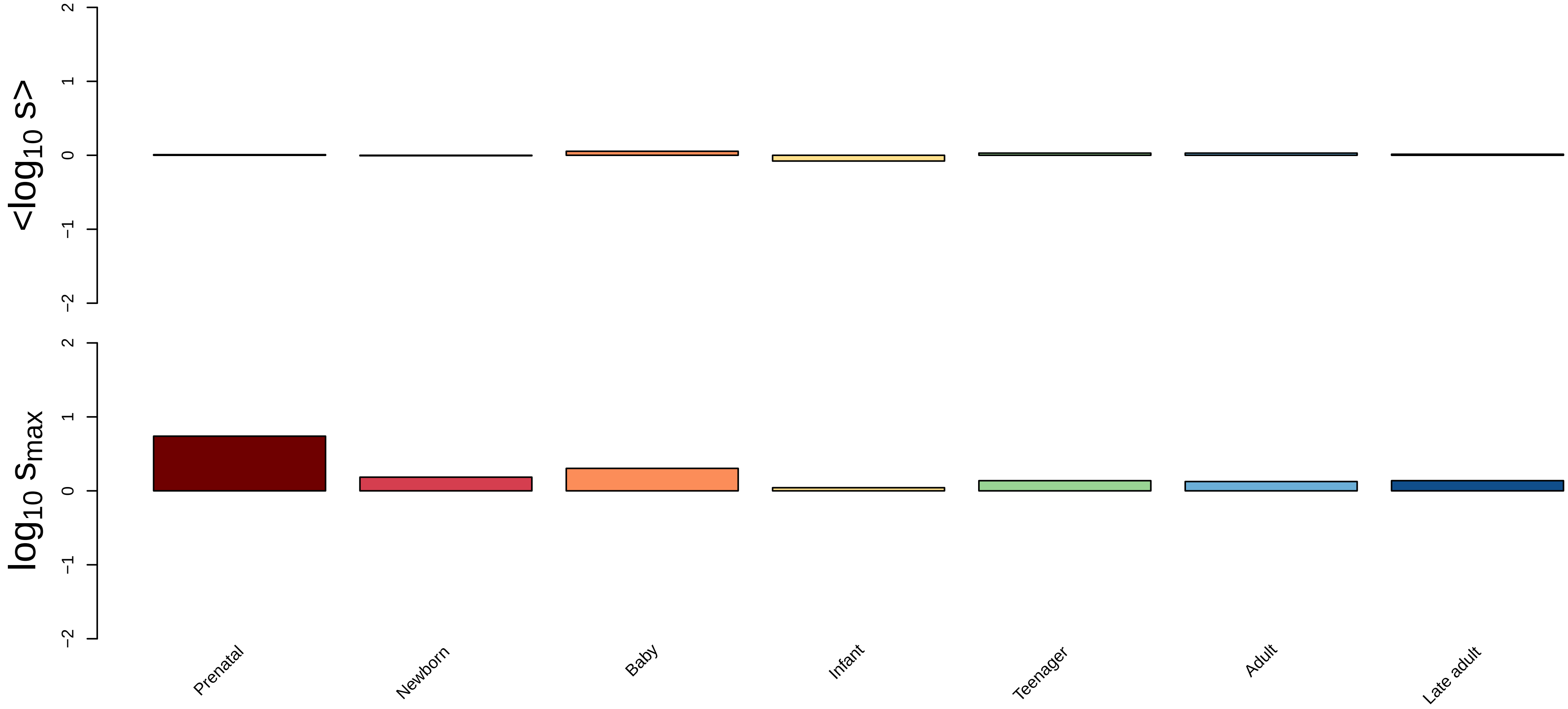

TNF_signaling_pathway
genes with data

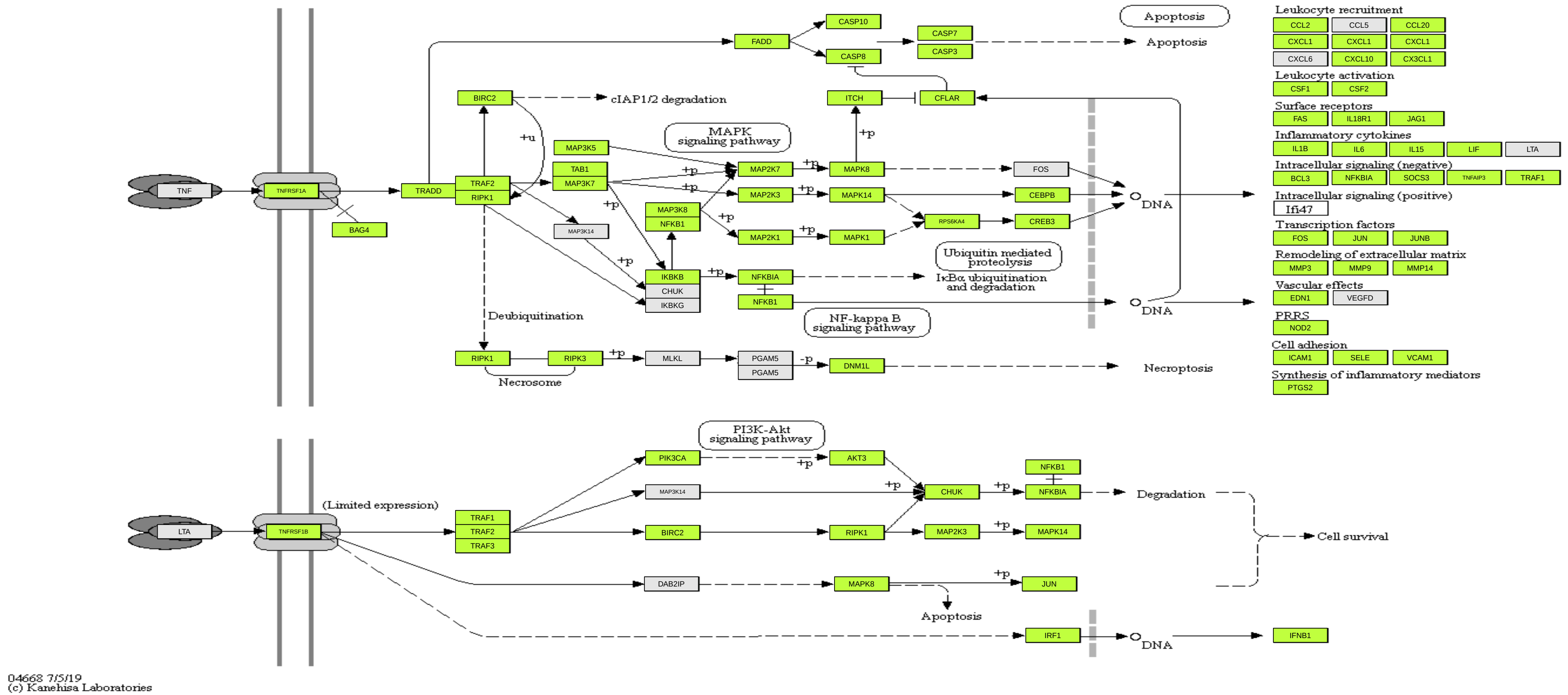

TNF_signaling_pathway
sink nodes

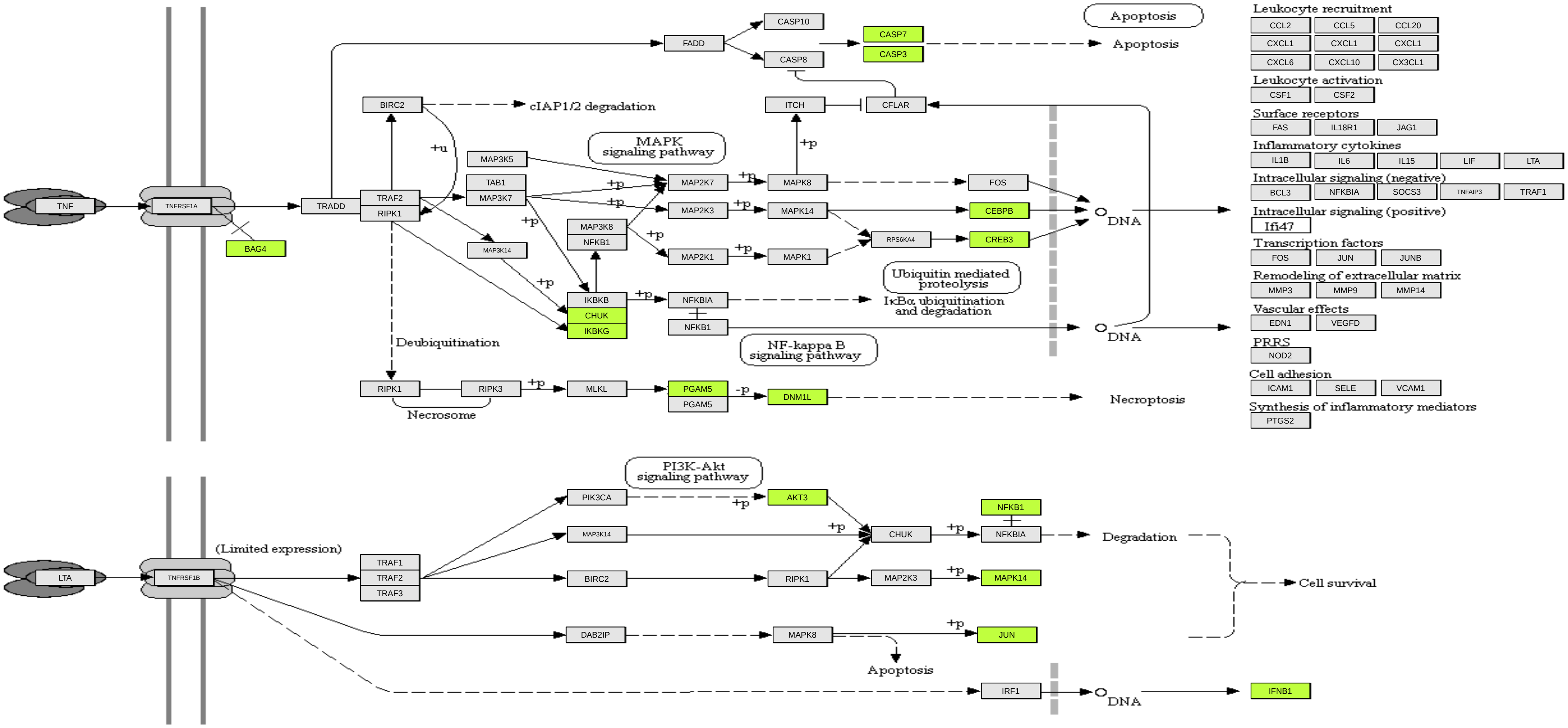

04668 7/5/19
(c) Kanehisa Laboratories

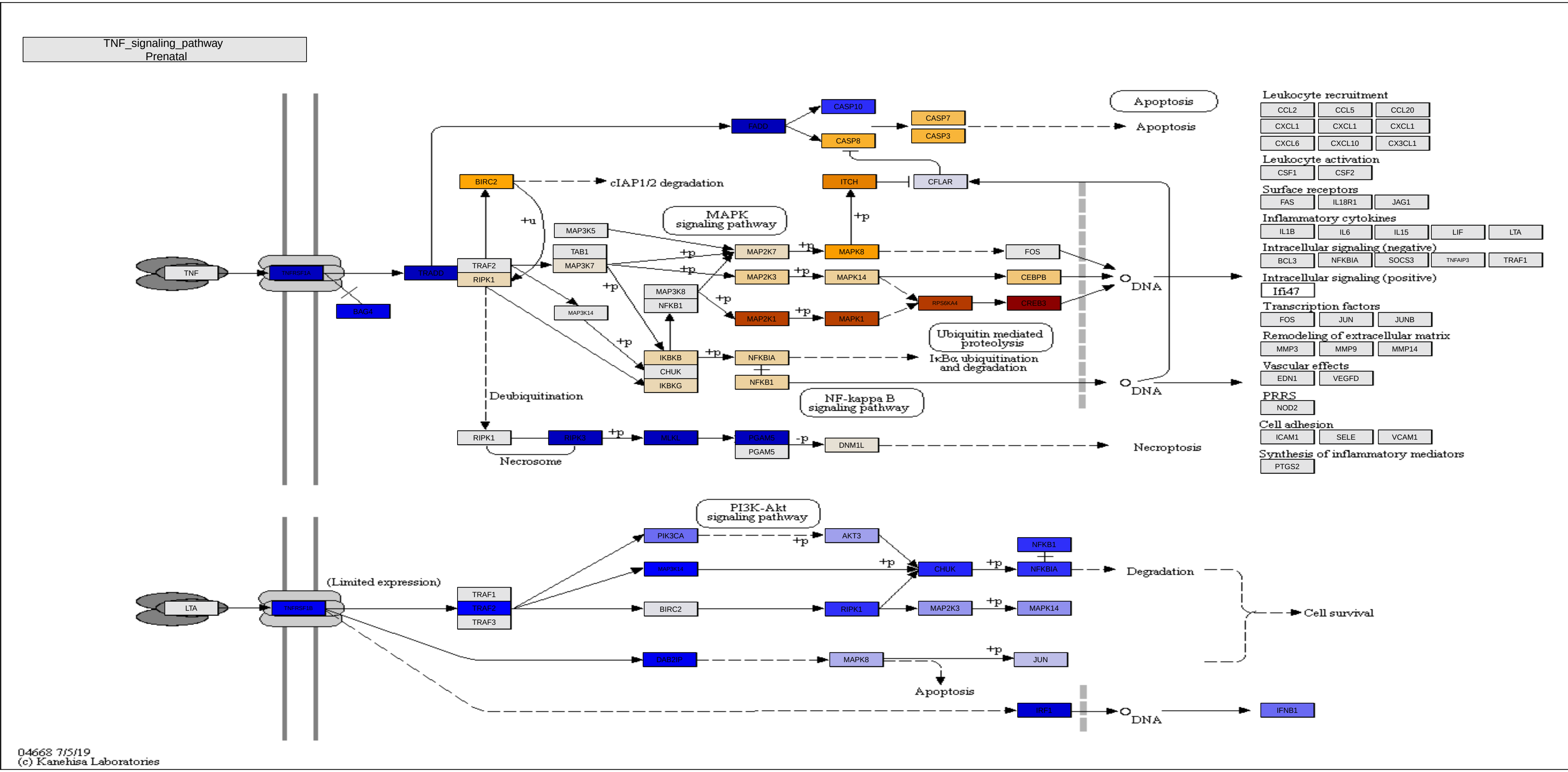

TNF_signaling_pathway
Prenatal
TNF
LTA
(Limited expression)
TRAF1
TRAF3
TRAF2
RIPK1
BIRC2
cIAP1/2 degradation
+u
MAP3K5
TAB1
MAP3K7
MAP3K14
MAP3K8
NFKB1
IKBKB
CHUK
IKBKG
NFKBIA
MAPK
signaling pathway
MAP2K7
MAP2K3
MAP2K1
MAPK8
MAPK14
MAPK1
ITCH
CFLAR
CASP10
CASP8
CASP7
CASP3
Apoptosis
FOS
CEBPB
DNA
Ubiquitin mediated
proteolysis
IκBα ubiquitination
and degradation
NF-kappa B
signaling pathway
Deubiquitination
Necrosome
PGAM5
DNM1L
Necroptosis
+p
-p
PI3K-Akt
signaling pathway
PIK3CA
AKT3
CHUK
NFKB1
NFKBIA
Degradation
Cell survival
RIPK1
MAP2K3
MAPK14
MAPK8
JUN
Leukocyte recruitment
CCL2
CCL5
CCL20
CXCL1
CXCL6
CXCL10
CX3CL1
Leukocyte activation
CSF1
CSF2
Surface receptors
FAS
IL18R1
JAG1
Inflammatory cytokines
IL1B
IL6
IL15
LIF
LTA
Intracellular signaling (negative)
BCL3
NFKBIA
SOCS3
TNFAIP3
TRAF1
Intracellular signaling (positive)
Ifi47
Transcription factors
FOS
JUN
JUNB
Remodeling of extracellular matrix
MMP3
MMP9
MMP14
Vascular effects
EDN1
VEGFD
PRRS
NOD2
Cell adhesion
ICAM1
SELE
VCAM1
Synthesis of inflammatory mediators
PTGS2
IFNB1
04668 7/5/19
(c) Kanehisa Laboratories

TNF_signaling_pathway
Newborn

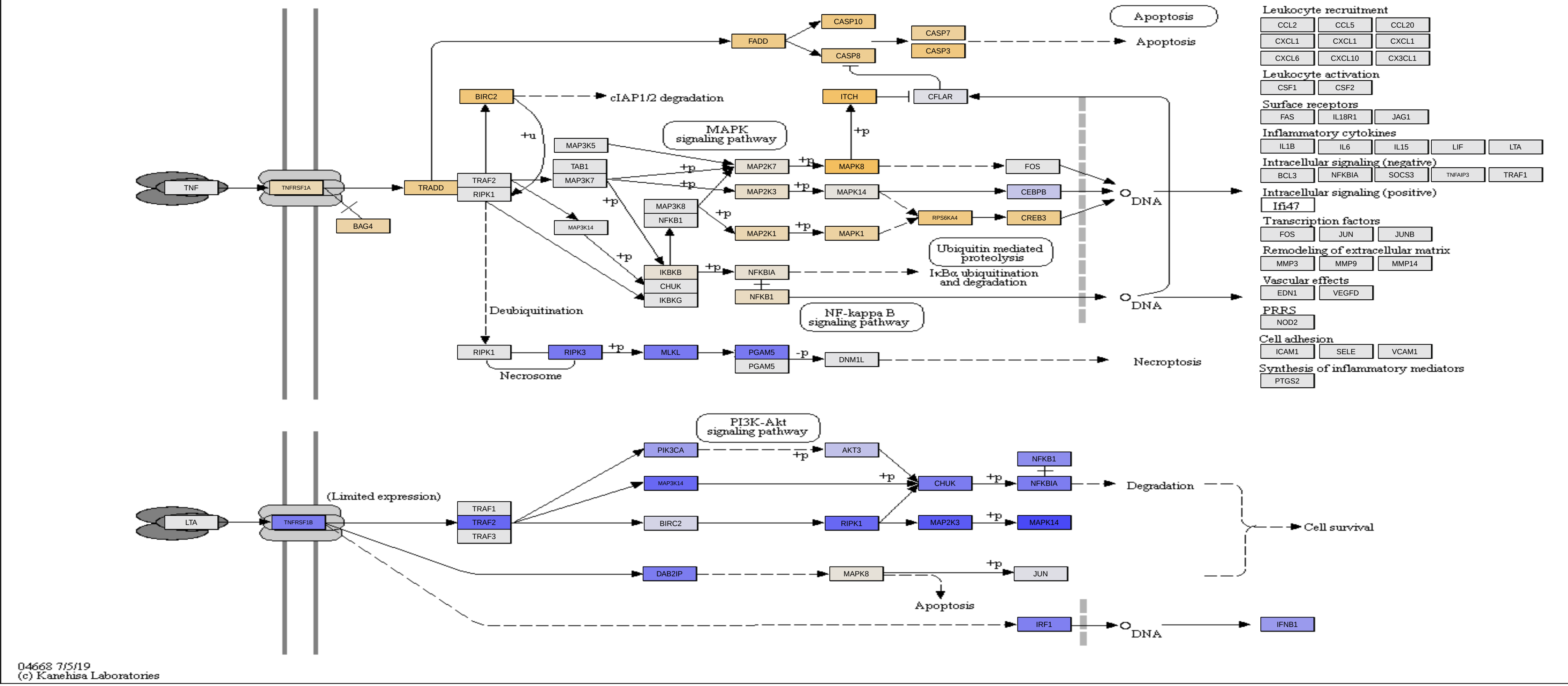

TNF_signaling_pathway
Baby

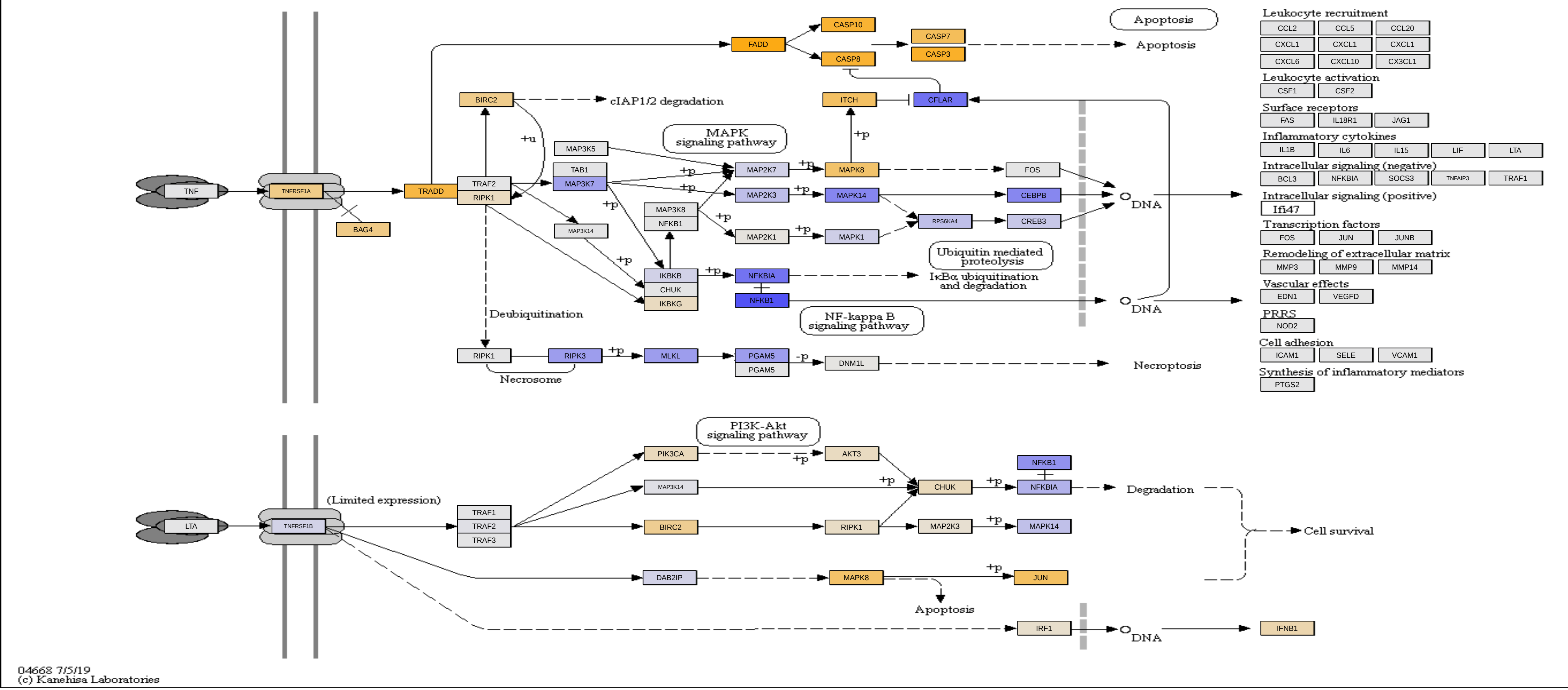

TNF_signaling_pathway
Infant

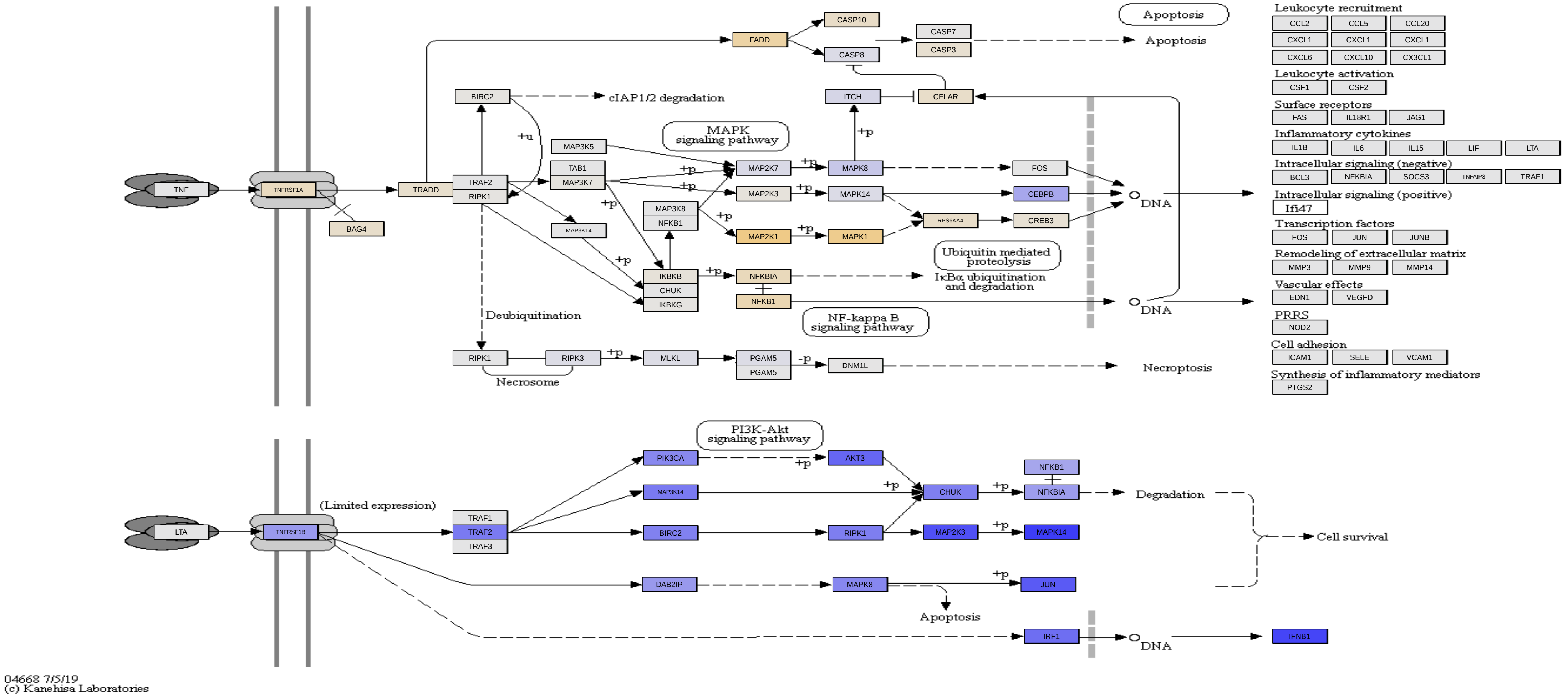

TNF_signaling_pathway
Teenager

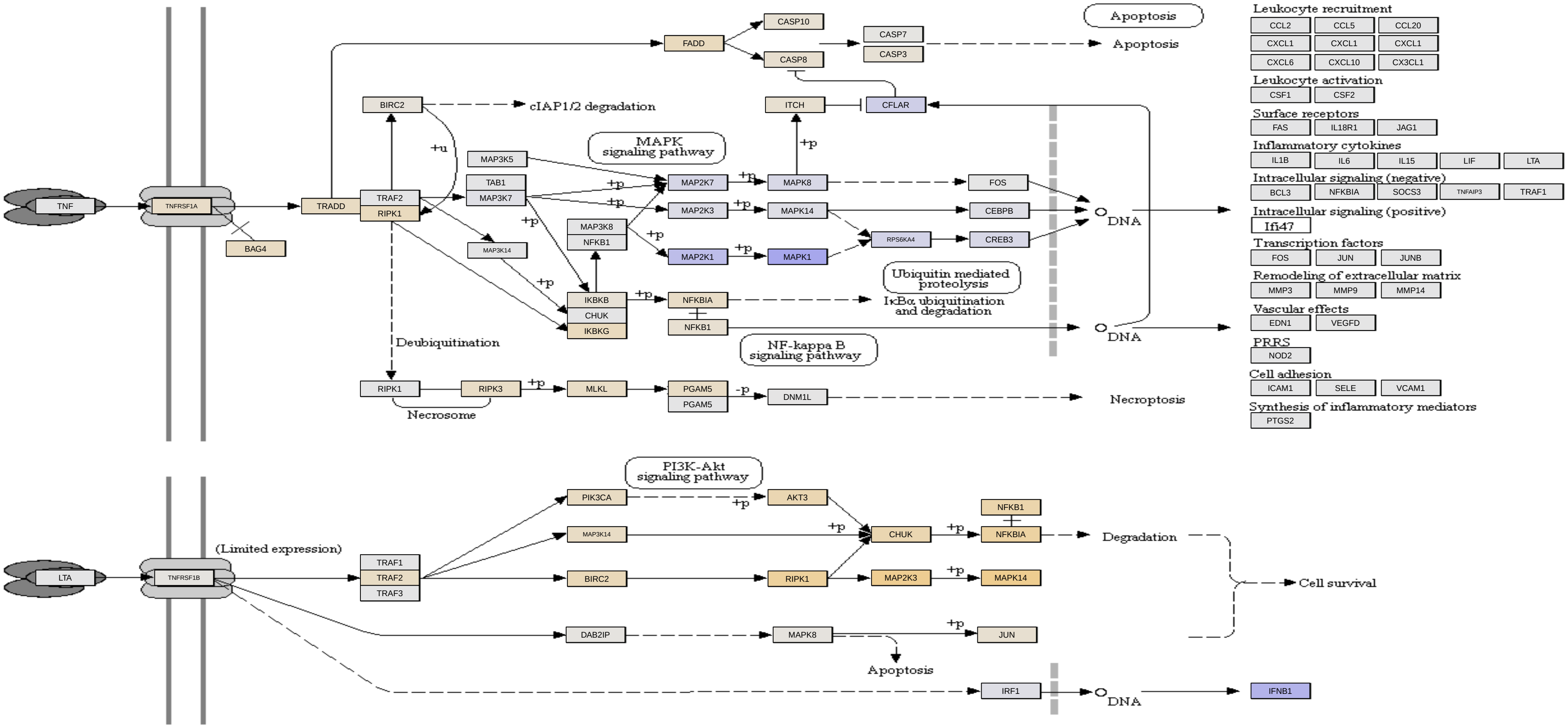

04668 7/5/19
(c) Kanehisa Laboratories

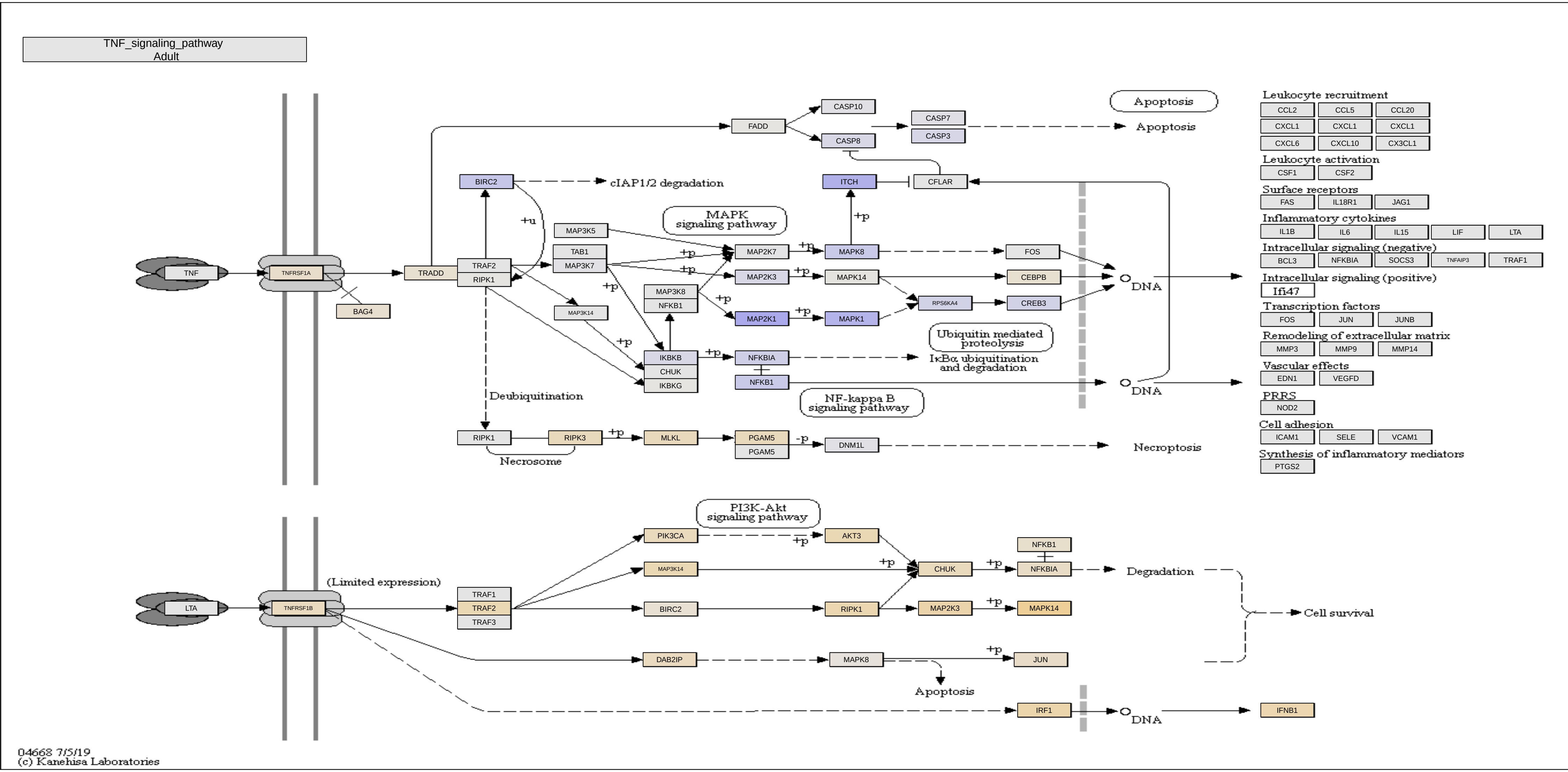
TNF_signaling_pathway
Adult
TNF
TNFRSF1A
BAG4
TRADD
TRAF2
RIPK1
BIRC2
cIAP1/2 degradation
+u
FADD
CASP10
CASP8
CASP7
CASP3
Apoptosis
Apoptosis
ITCH
CFLAR
MAP3K5
TAB1
MAP3K7
MAP3K14
MAPK signaling pathway
MAP2K7
MAP2K3
MAP2K1
MAPK8
MAPK14
MAPK1
FOS
CEBPB
RPS6KA4
CREB3
DNA
MAP3K8
NFKB1
IKBKB
CHUK
IKBKG
NFKBIA
NFKB1
Ubiquitin mediated proteolysis
IκBα ubiquitination and degradation
NF-kappa B signaling pathway
DNA
+p
Deubiquitination
RIPK1
RIPK3
MLKL
PGAM5
PGAM5
-p
DNM1L
Necrosome
Necroptosis
Leukocyte recruitment
CCL2
CCL5
CCL20
CXCL1
CXCL1
CXCL1
CXCL6
CXCL10
CX3CL1
Leukocyte activation
CSF1
CSF2
Surface receptors
FAS
IL18R1
JAG1
Inflammatory cytokines
IL1B
IL6
IL15
LIF
LTA
Intracellular signaling (negative)
BCL3
NFKBIA
SOCS3
TNFAIP3
TRAF1
Intracellular signaling (positive)
Ifi47
Transcription factors
FOS
JUN
JUNB
Remodeling of extracellular matrix
MMP3
MMP9
MMP14
Vascular effects
EDN1
VEGFD
PRRS
NOD2
Cell adhesion
ICAM1
SELE
VCAM1
Synthesis of inflammatory mediators
PTGS2
LTA
TNFRSF1B
(Limited expression)
TRAF1
TRAF2
TRAF3
PI3K-Akt signaling pathway
PIK3CA
AKT3
MAP3K14
CHUK
NFKB1
NFKBIA
Degradation
BIRC2
RIPK1
MAP2K3
MAPK14
Cell survival
DAB2IP
MAPK8
JUN
Apoptosis
IRF1
DNA
IFNB1
04668 7/5/19
(c) Kanehisa Laboratories

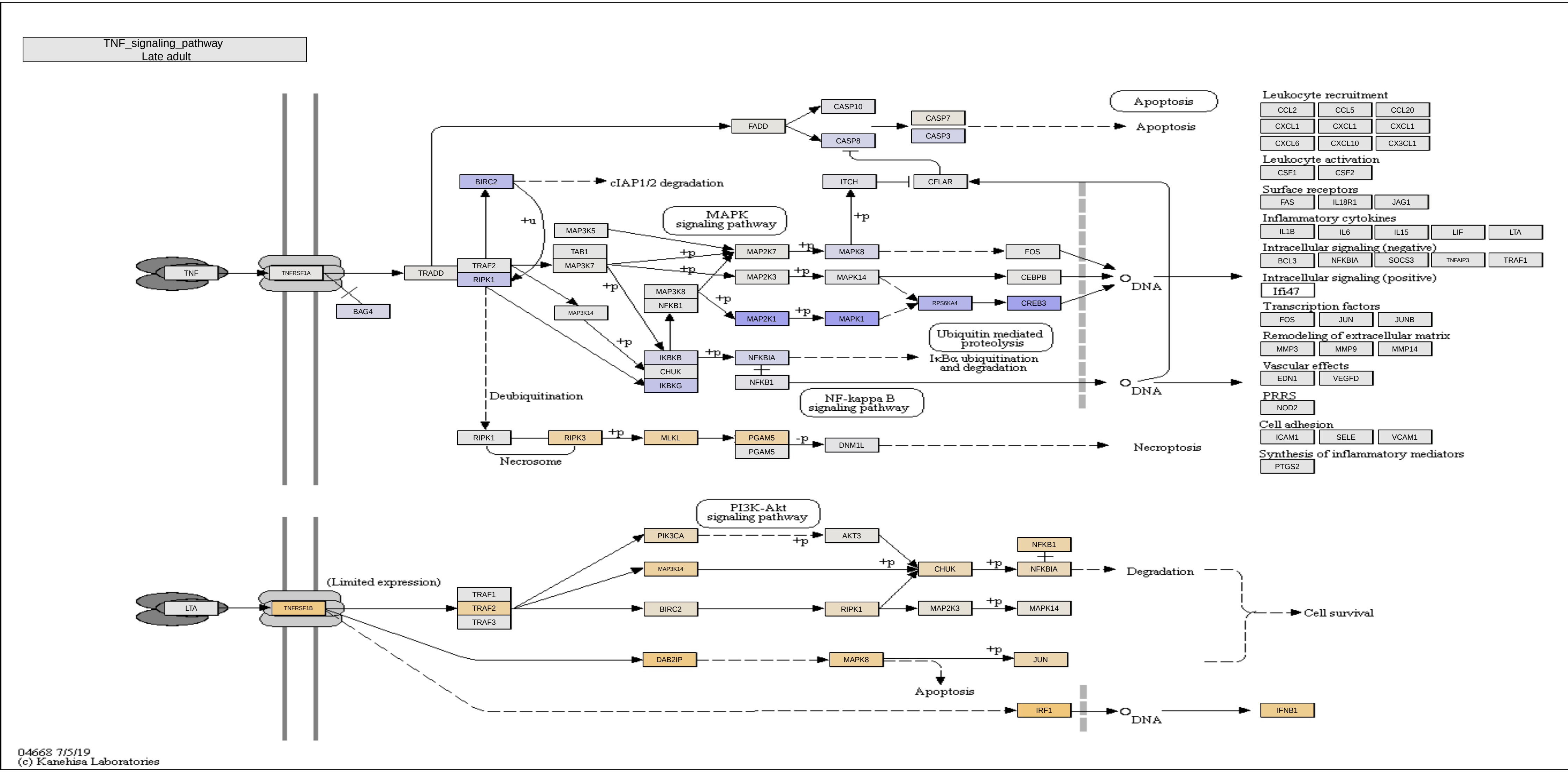
TNF_signaling_pathway
Late adult
TNF
TNFRSF1A
BAG4
TRADD
TRAF2
RIPK1
BIRC2
cIAP1/2 degradation
+u
MAP3K5
TAB1
MAP3K7
MAP3K14
MAP3K8
NFKB1
IKBKB
CHUK
IKBKG
MAPK
signaling pathway
MAP2K7
MAP2K3
MAP2K1
MAPK8
MAPK14
MAPK1
FADD
CASP10
CASP8
CASP7
CASP3
Apoptosis
ITCH
CFLAR
FOS
CEBPB
RPS6KA4
CREB3
DNA
Ubiquitin mediated
proteolysis
NFKBIA
NFKB1
IκBα ubiquitination
and degradation
NF-kappa B
signaling pathway
Deubiquitination
RIPK1
RIPK3
MLKL
PGAM5
PGAM5
DNM1L
Necrosome
Necroptosis
+p
-p
Leukocyte recruitment
CCL2
CCL5
CCL20
CXCL1
CXCL1
CXCL1
CXCL6
CXCL10
CX3CL1
Leukocyte activation
CSF1
CSF2
Surface receptors
FAS
IL18R1
JAG1
Inflammatory cytokines
IL1B
IL6
IL15
LIF
LTA
Intracellular signaling (negative)
BCL3
NFKBIA
SOCS3
TNFAIP3
TRAF1
Intracellular signaling (positive)
Ifi47
Transcription factors
FOS
JUN
JUNB
Remodeling of extracellular matrix
MMP3
MMP9
MMP14
Vascular effects
EDN1
VEGFD
PRRS
NOD2
Cell adhesion
ICAM1
SELE
VCAM1
Synthesis of inflammatory mediators
PTGS2
PI3K-Akt
signaling pathway
LTA
TNFRSF1B
(Limited expression)
TRAF1
TRAF2
TRAF3
PIK3CA
MAP3K14
BIRC2
DAB2IP
AKT3
CHUK
RIPK1
MAP2K3
MAPK8
NFKB1
NFKBIA
MAPK14
JUN
Degradation
Cell survival
Apoptosis
IRF1
DNA
IFNB1
04668 7/5/19
(c) Kanehisa Laboratories
