# Supplementary figures and images for: The Transcriptome and Methylome of the Developing and Aging Brain and Their Relations to Gliomas and Psychological Disorders
Source: Cells. 2022 Jan 21;11(3):362. doi: 10.3390/cells11030362 (PMC8834030; doi:10.3390/cells11030362)

mean signal

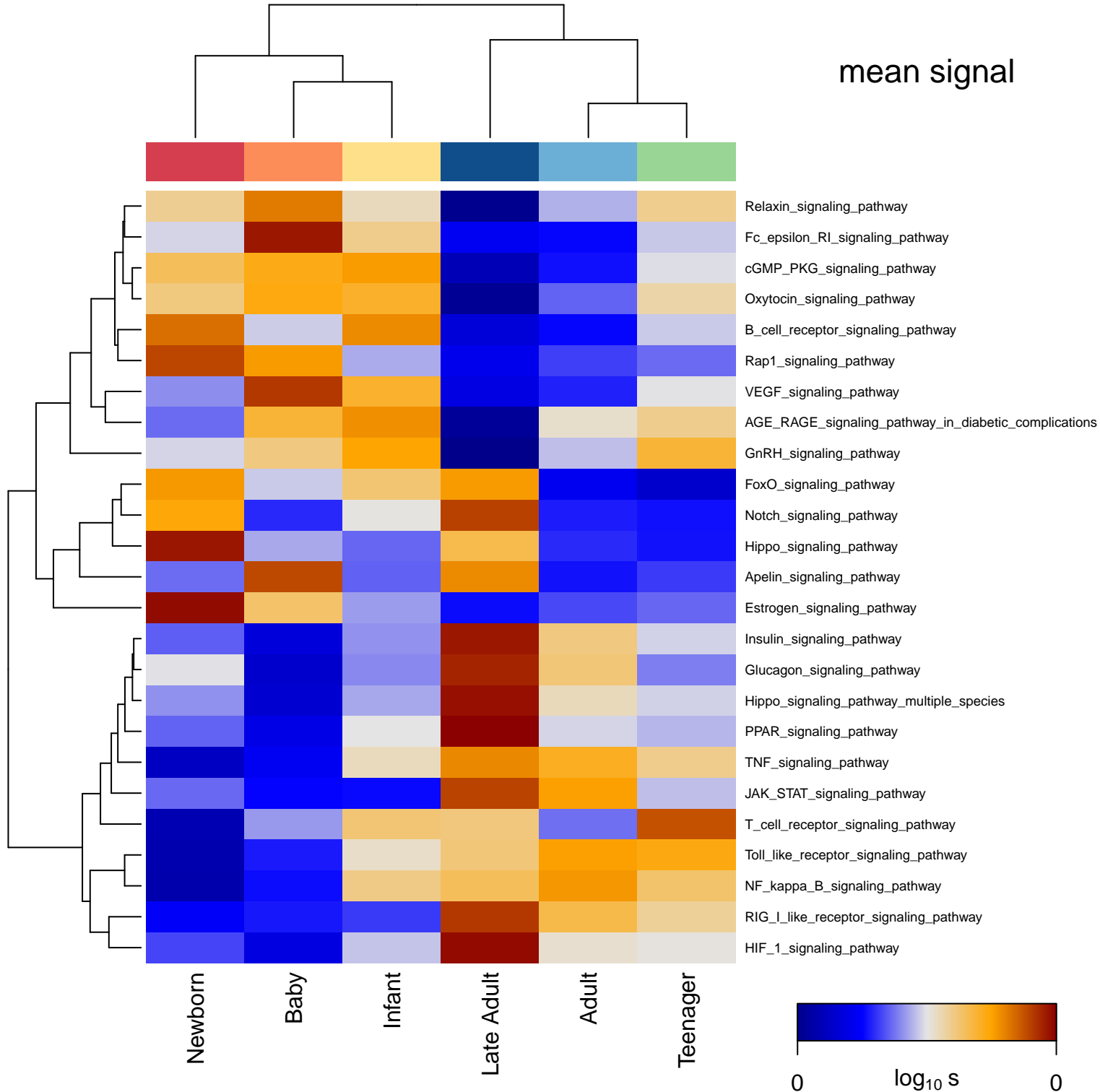

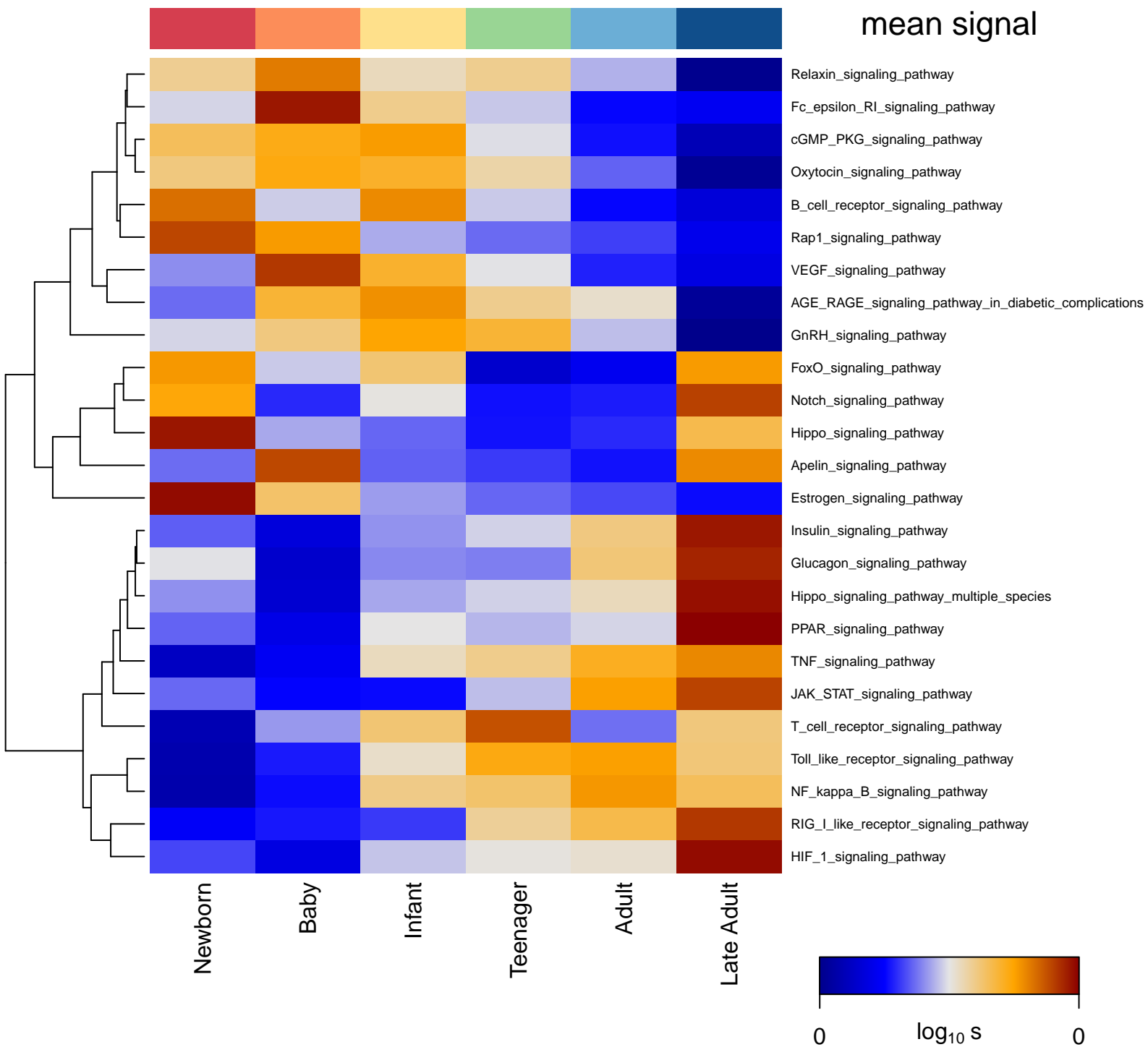

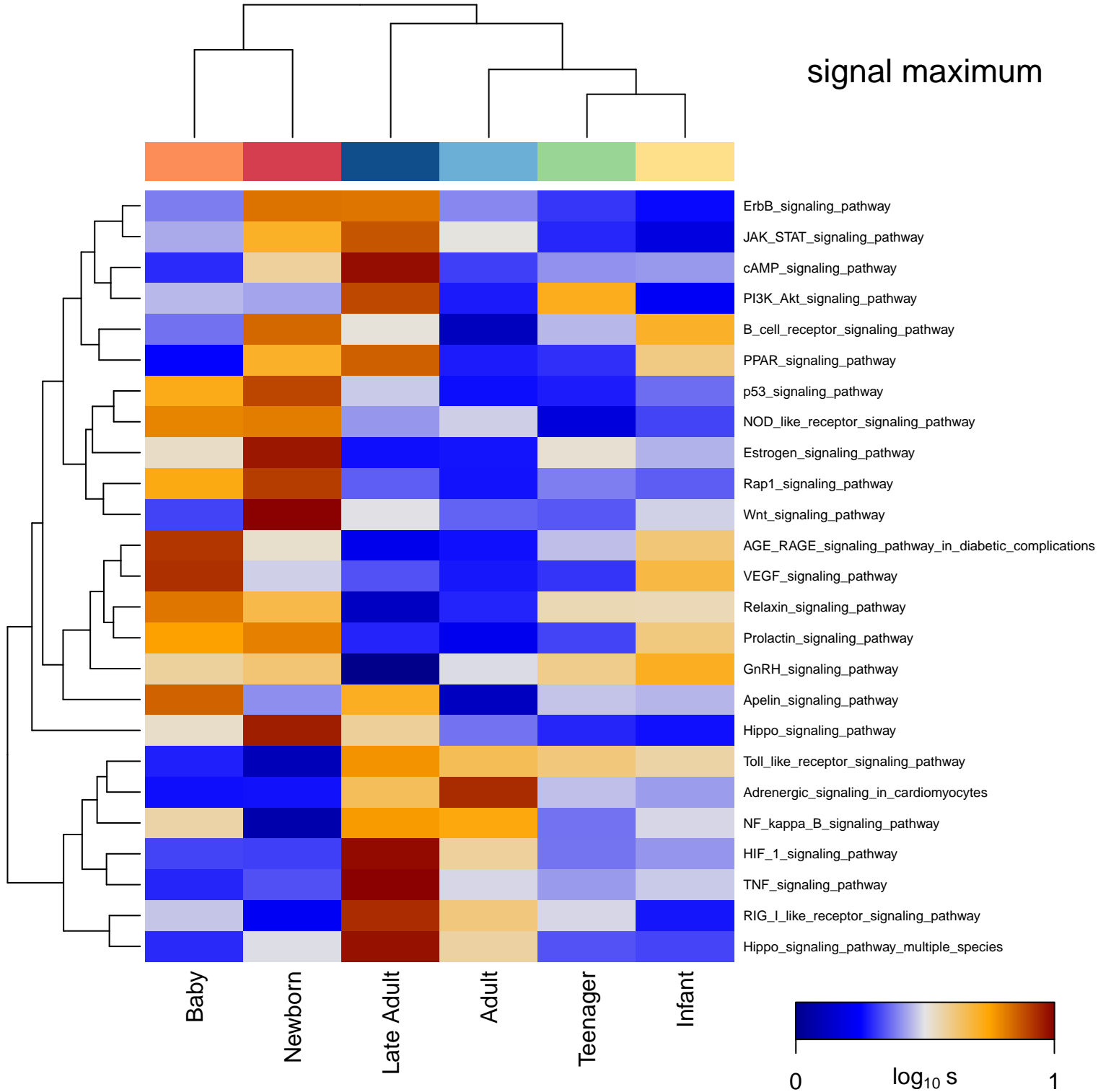

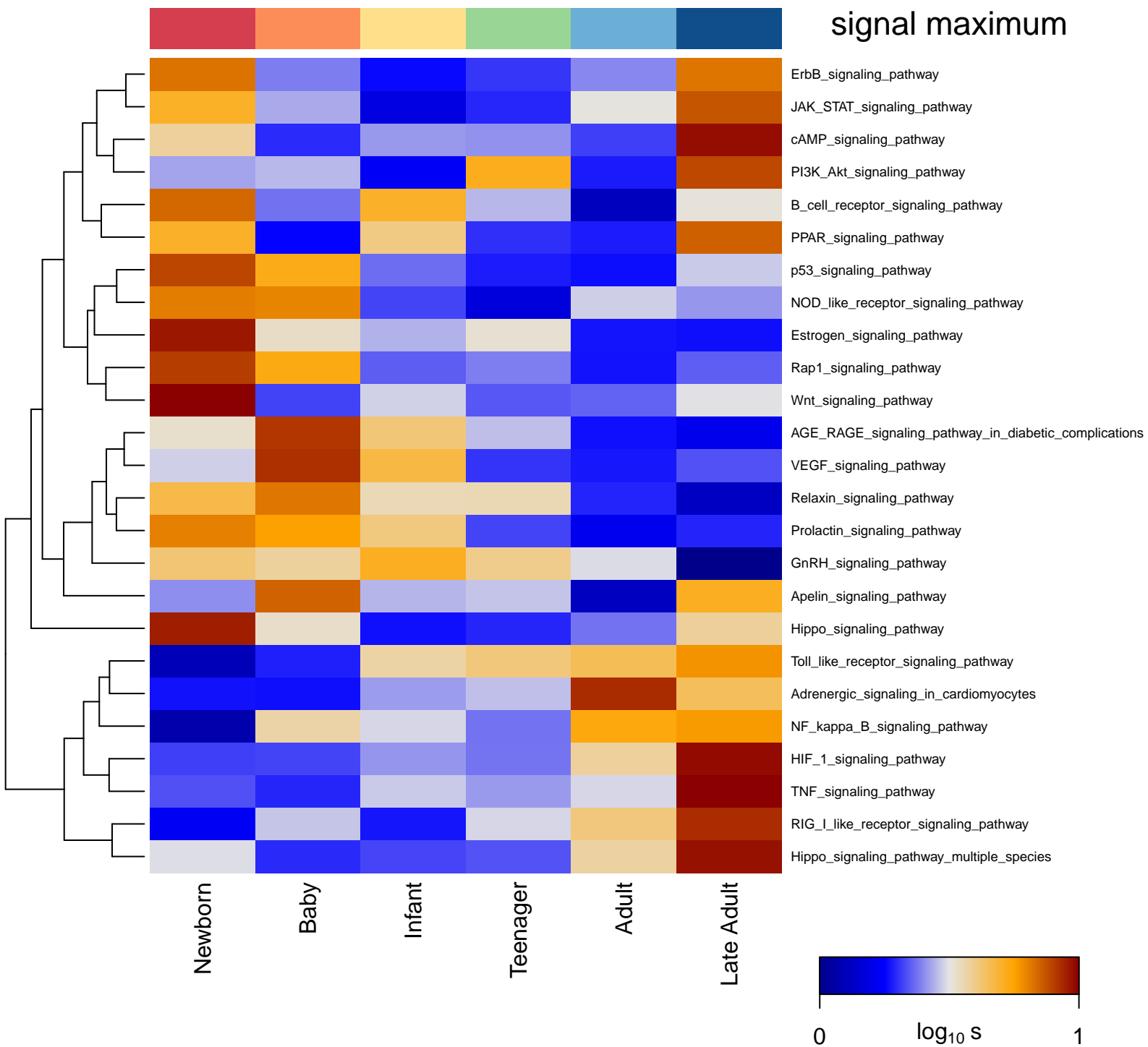

Supplement: Supplementary file 1 [file cells-11-00362-s001.zip › Suppl-Material-S3-Pathways-PSF_Expression/0verview Heatmaps.pdf]

Epithelial cell signaling in Helicobact

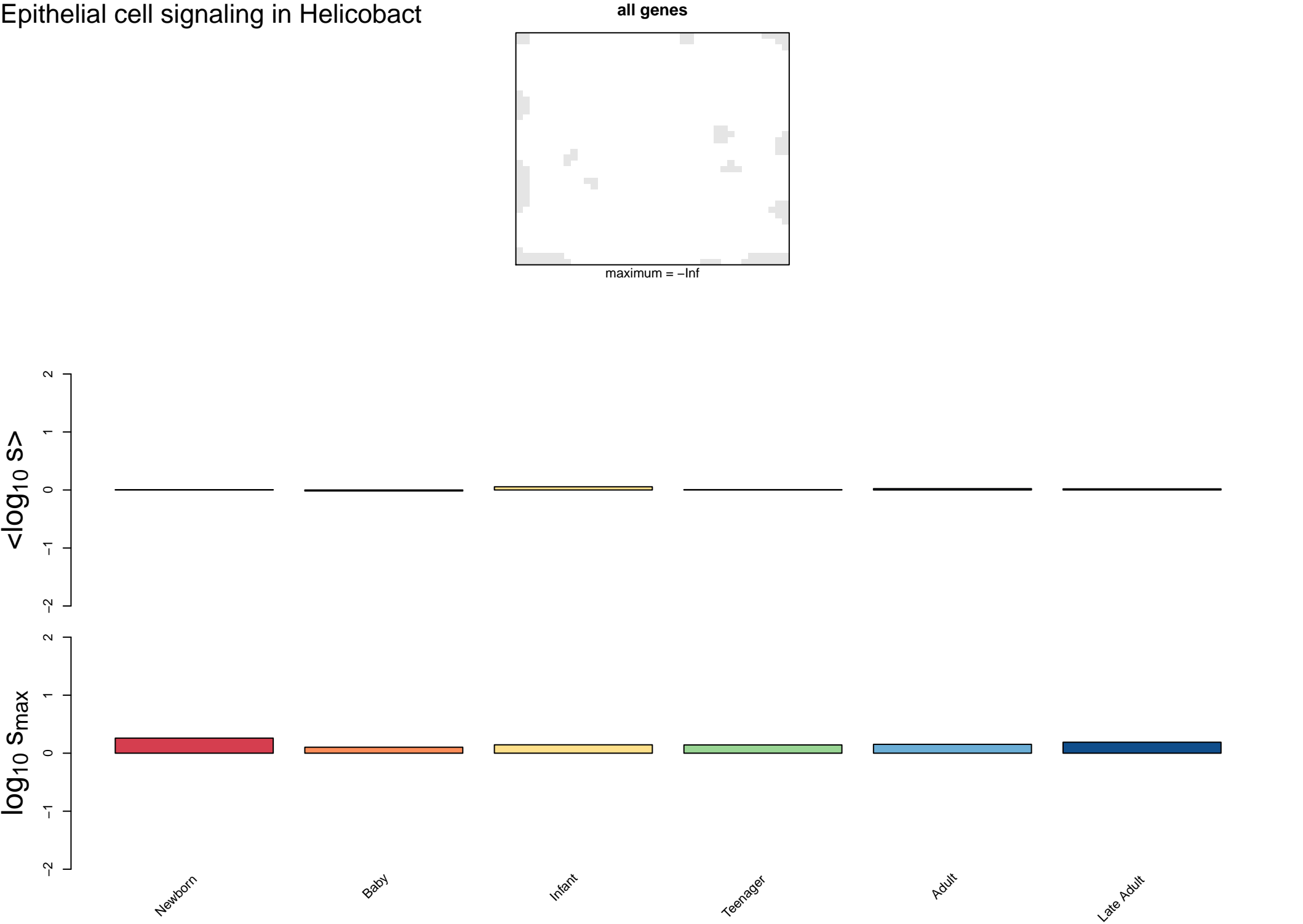

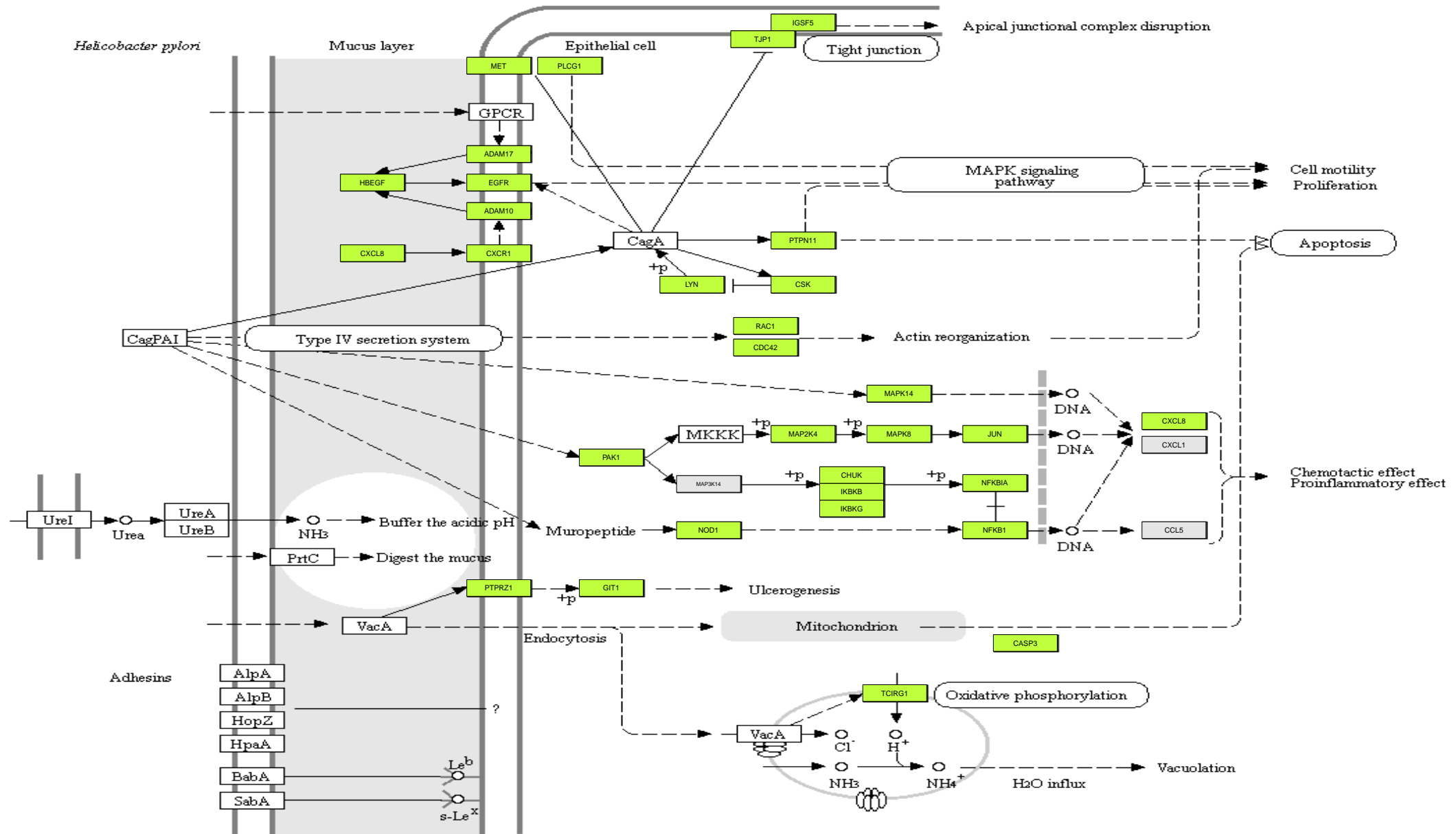

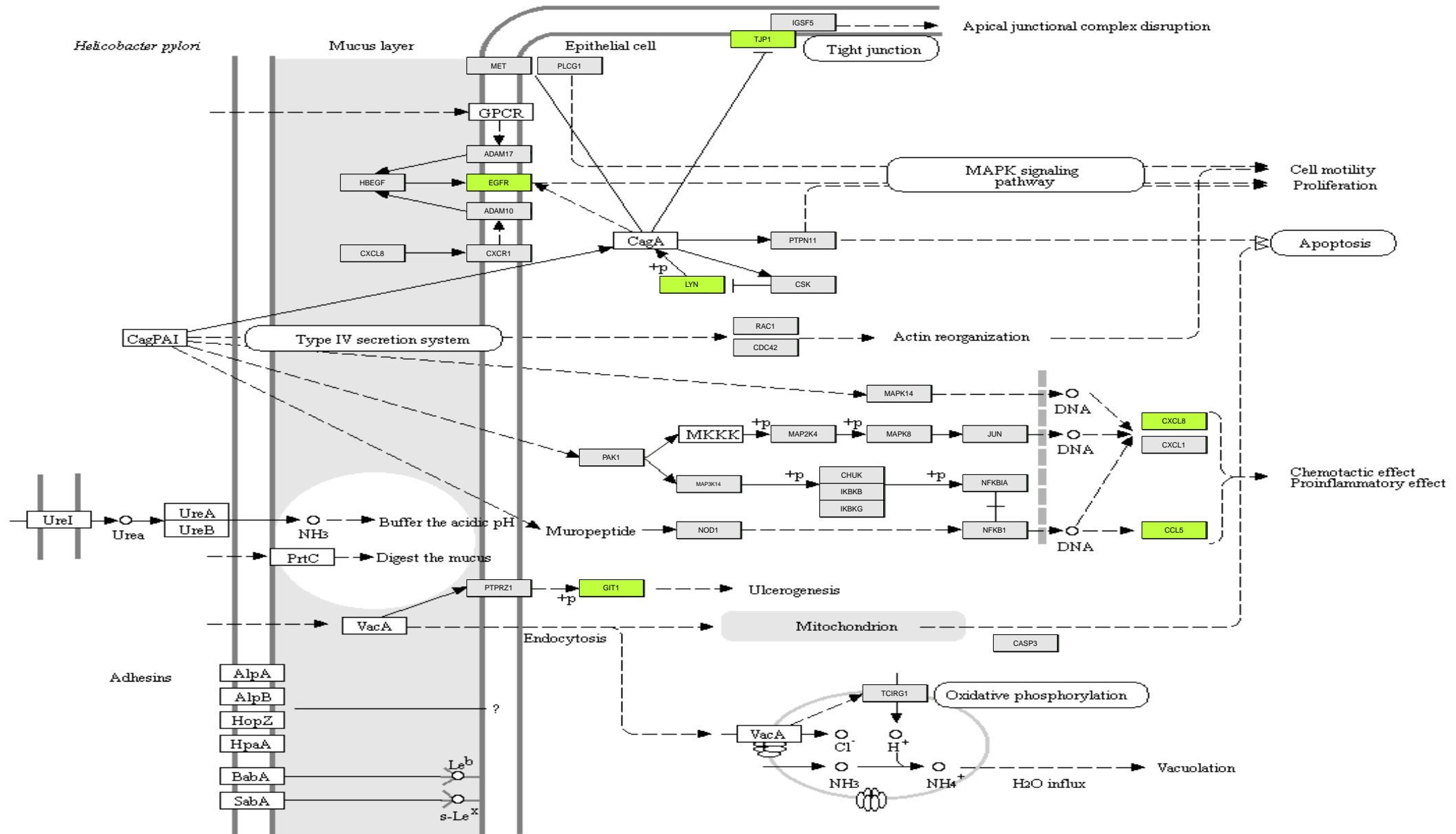

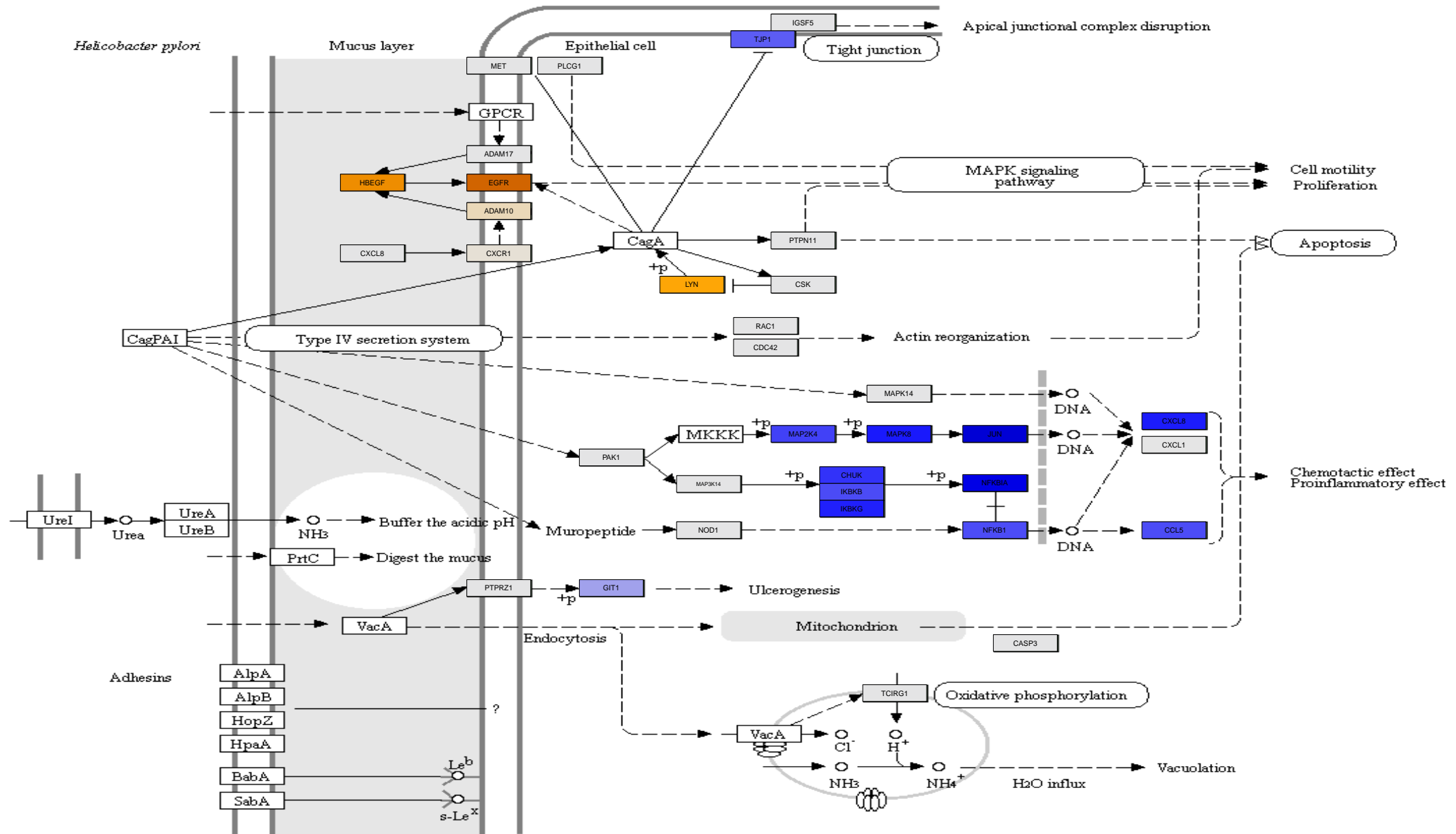

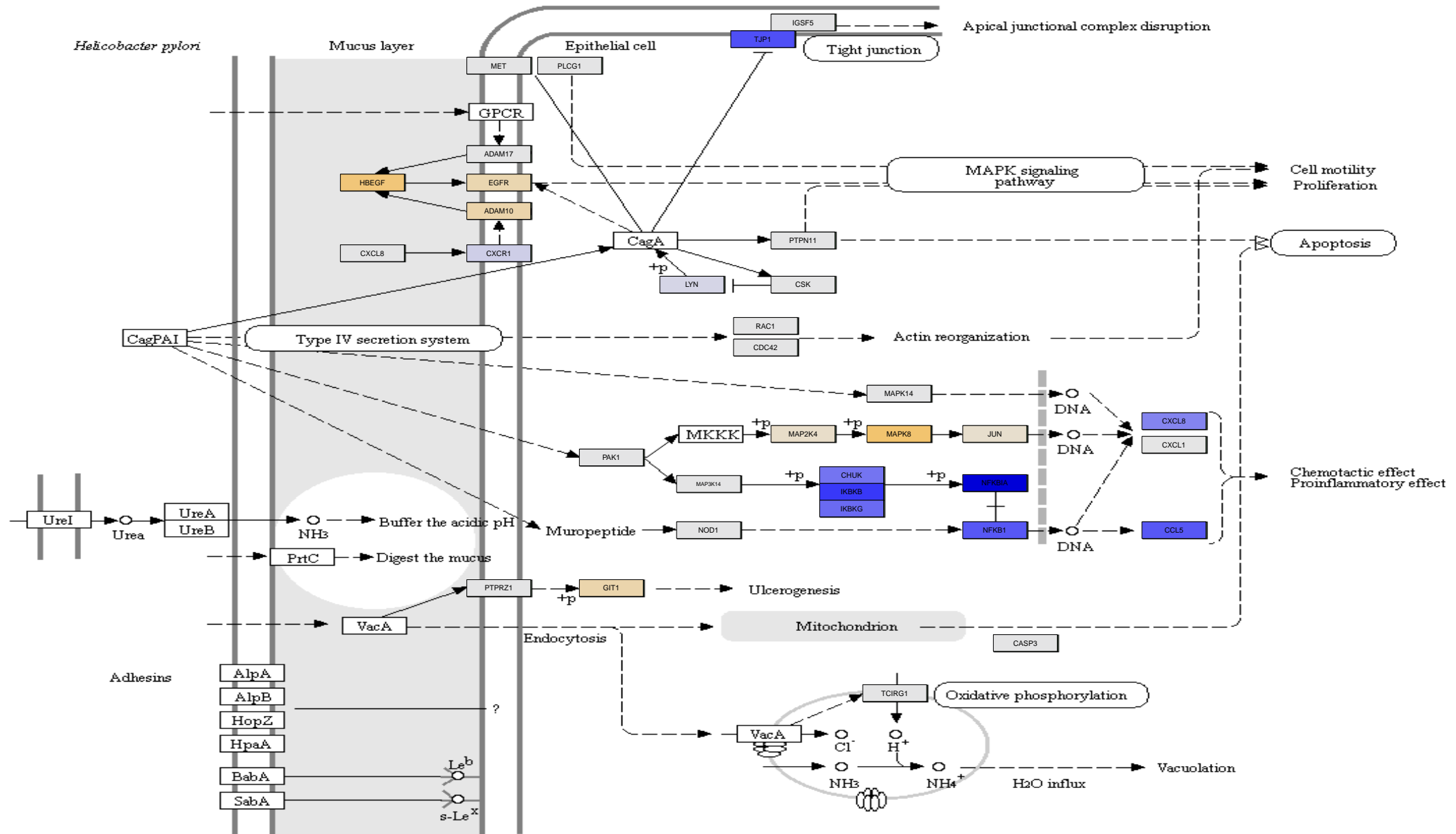

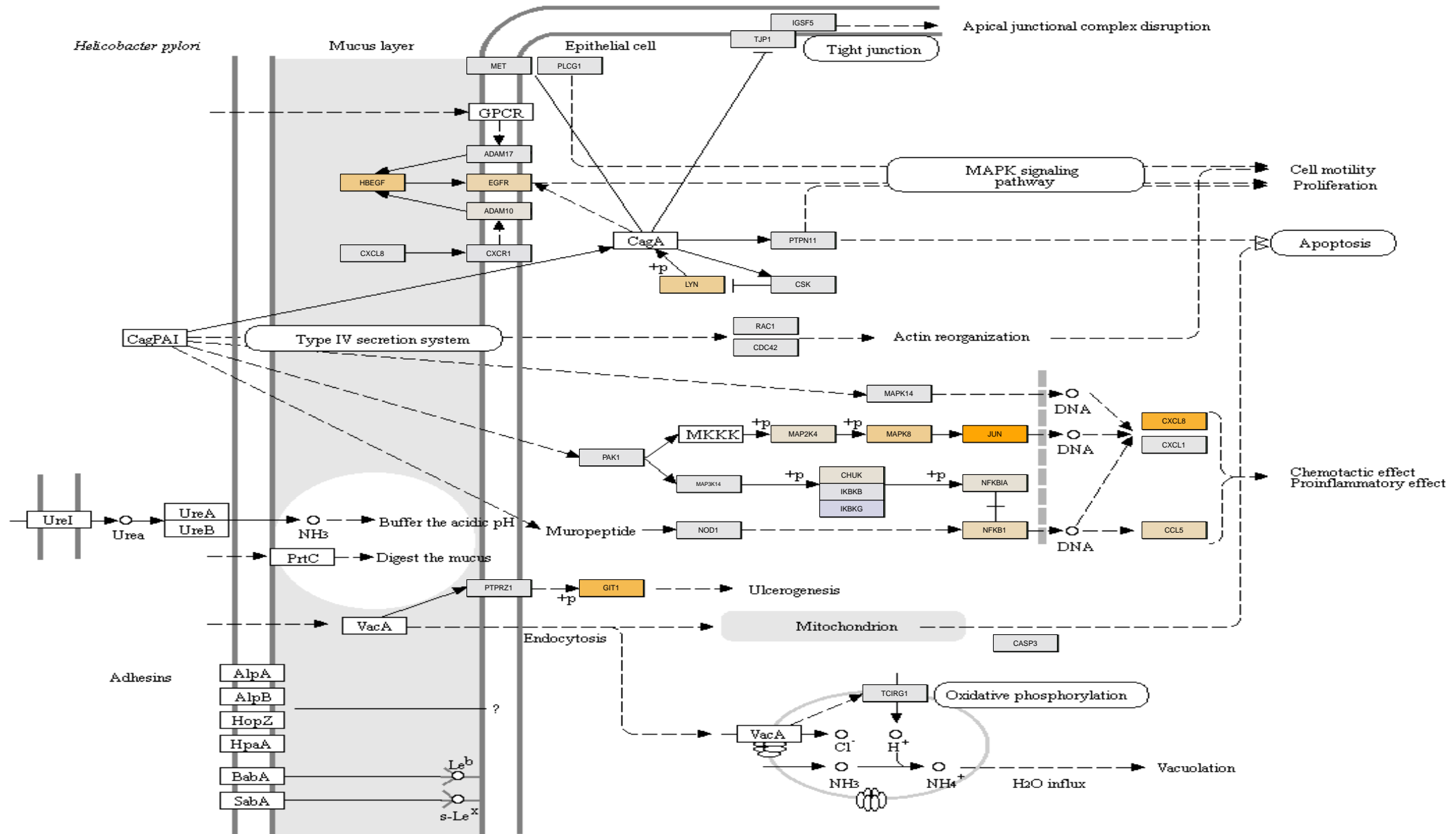

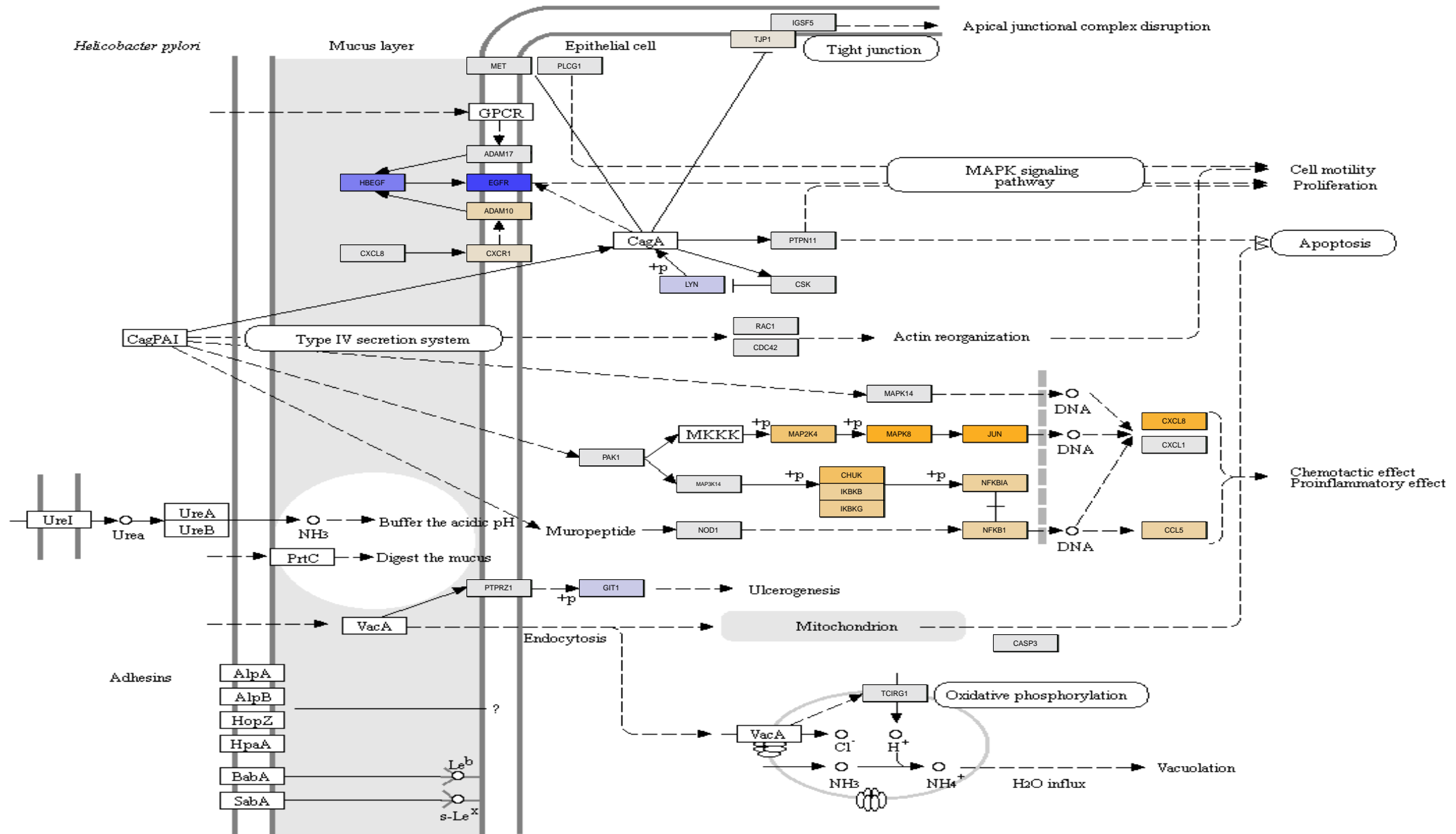

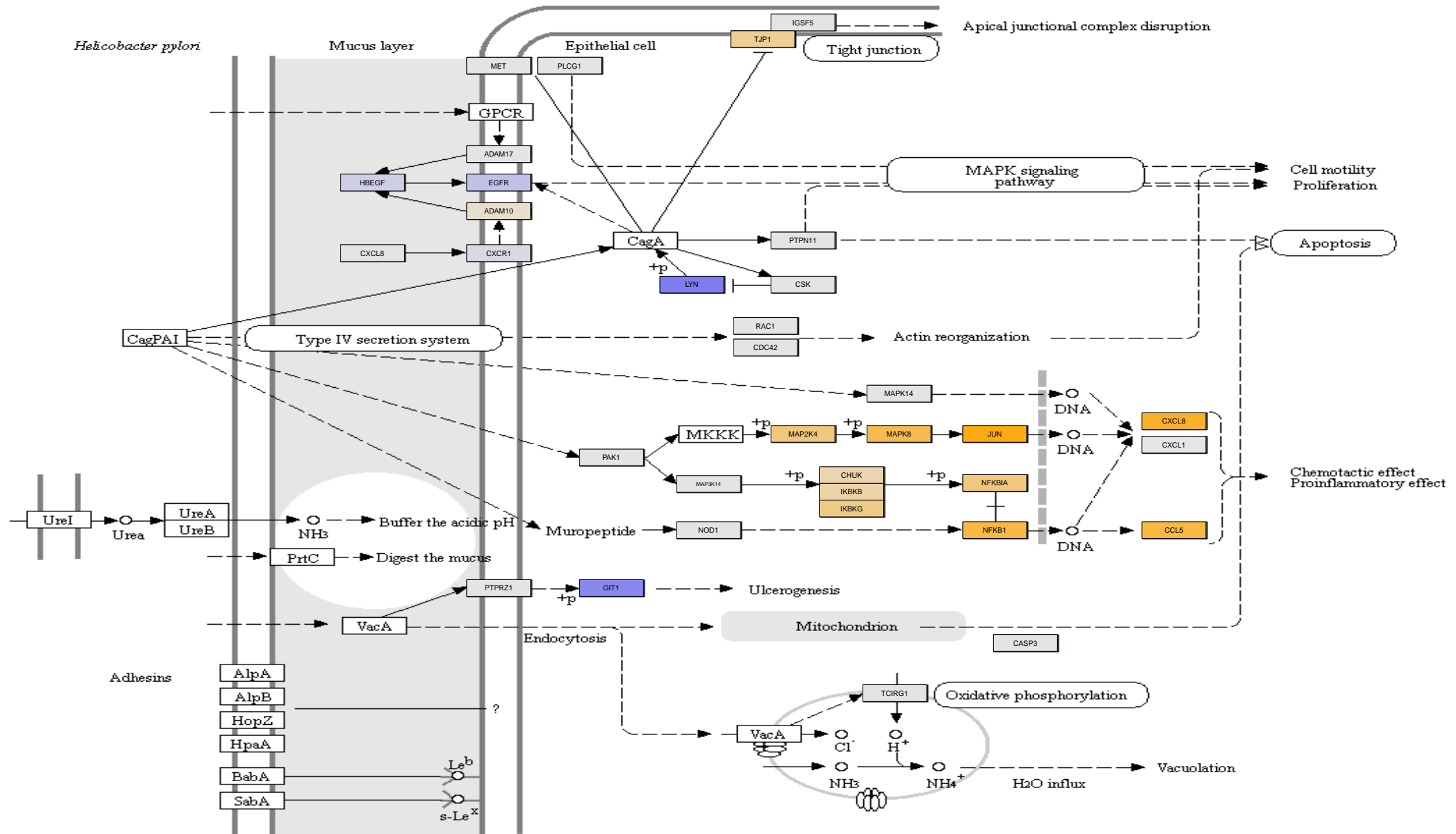

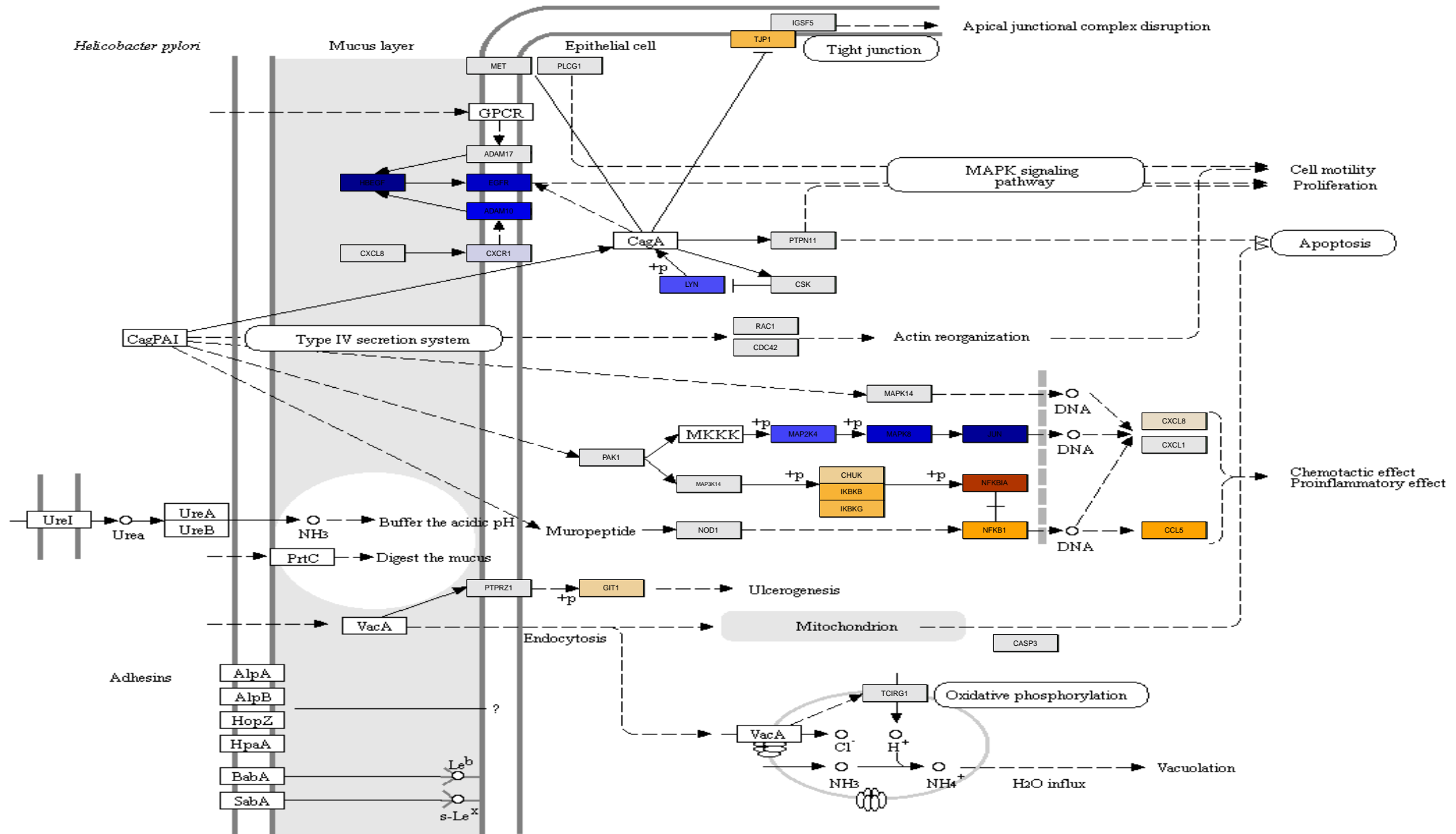

Supplement: Supplementary file 1 [file cells-11-00362-s001.zip › Suppl-Material-S3-Pathways-PSF_Expression/Epithelial_cell_signaling_in_Helicobacter_pylori_infection.pdf]

MAPK signaling pathway

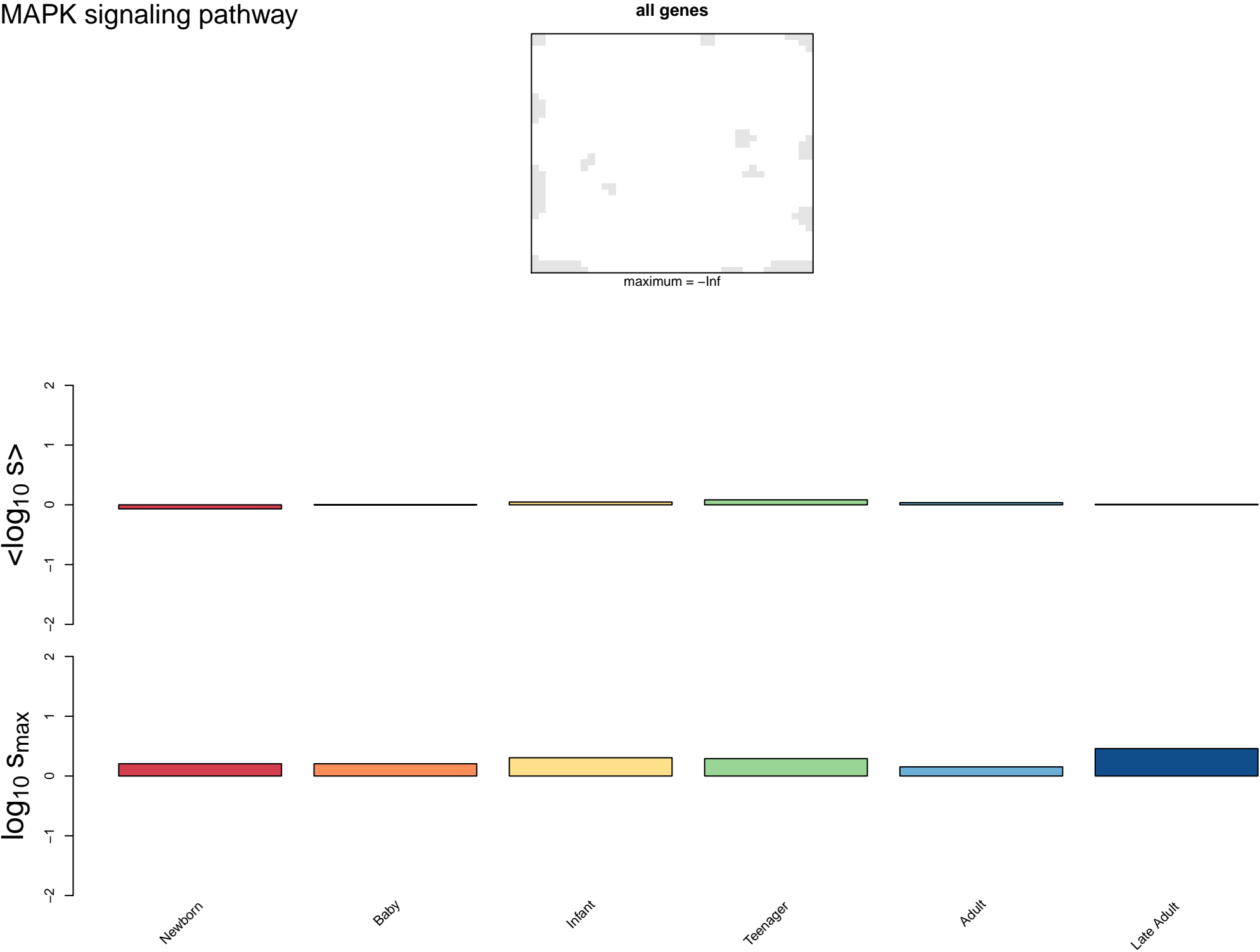







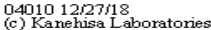

MAPK signaling pathway  
Infant

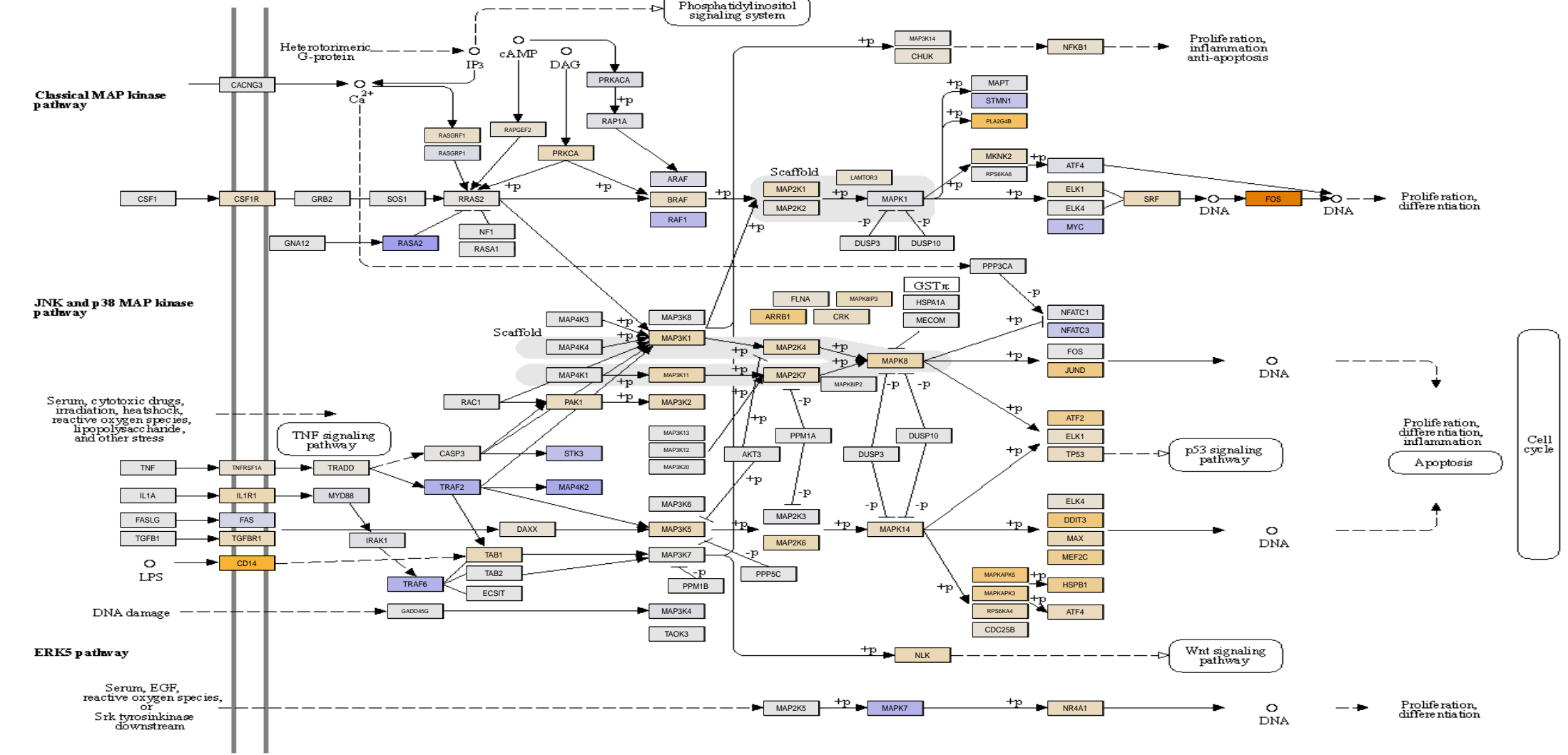

MAPK signaling pathway  
Teenager

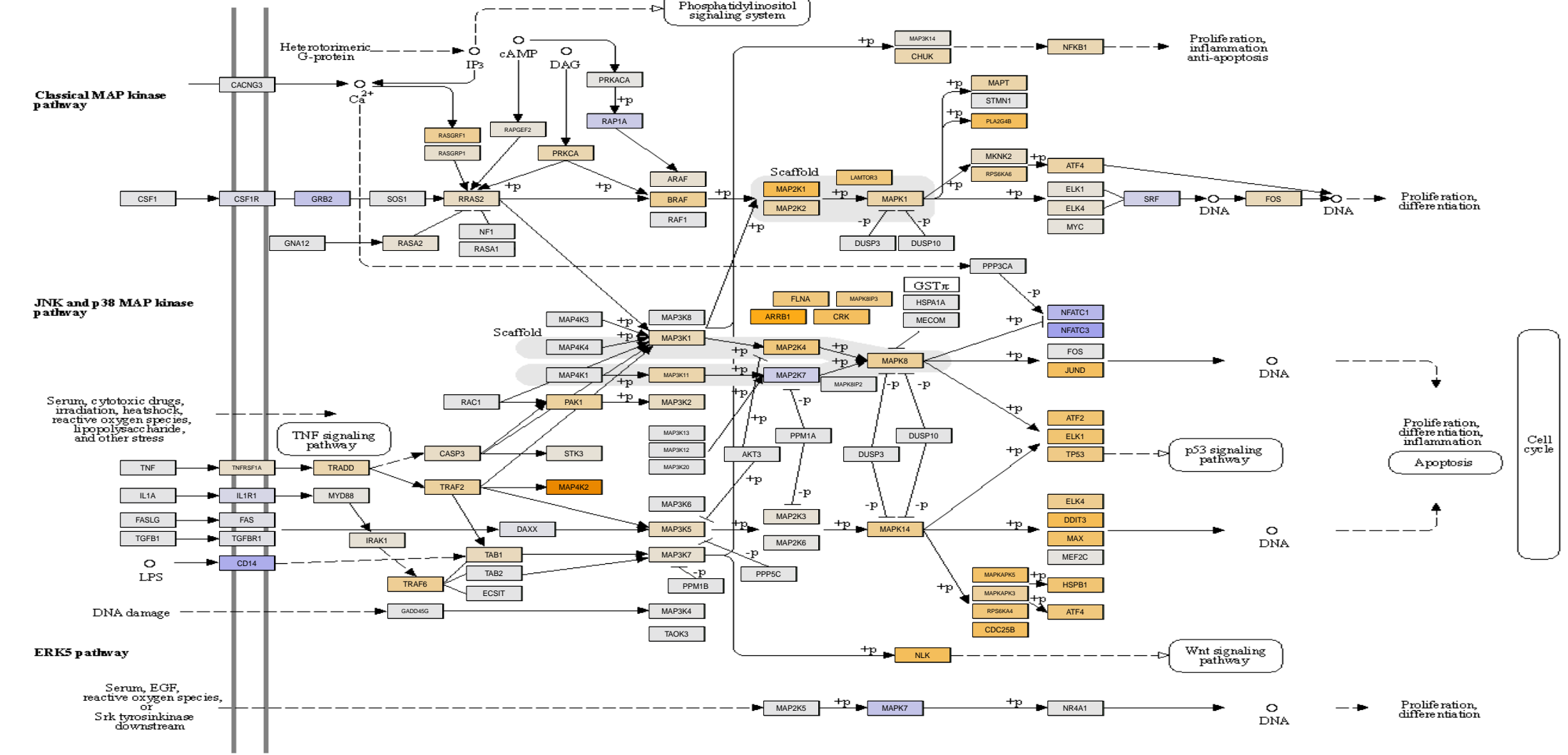

Supplement: Supplementary file 1 [file cells-11-00362-s001.zip › Suppl-Material-S3-Pathways-PSF_Expression/MAPK_signaling_pathway.pdf]

Phosphatidylinositol signaling system

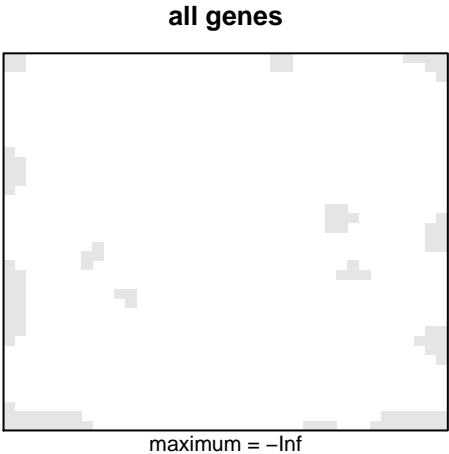











Phosphatidylinositol\_signaling\_system  
Teenager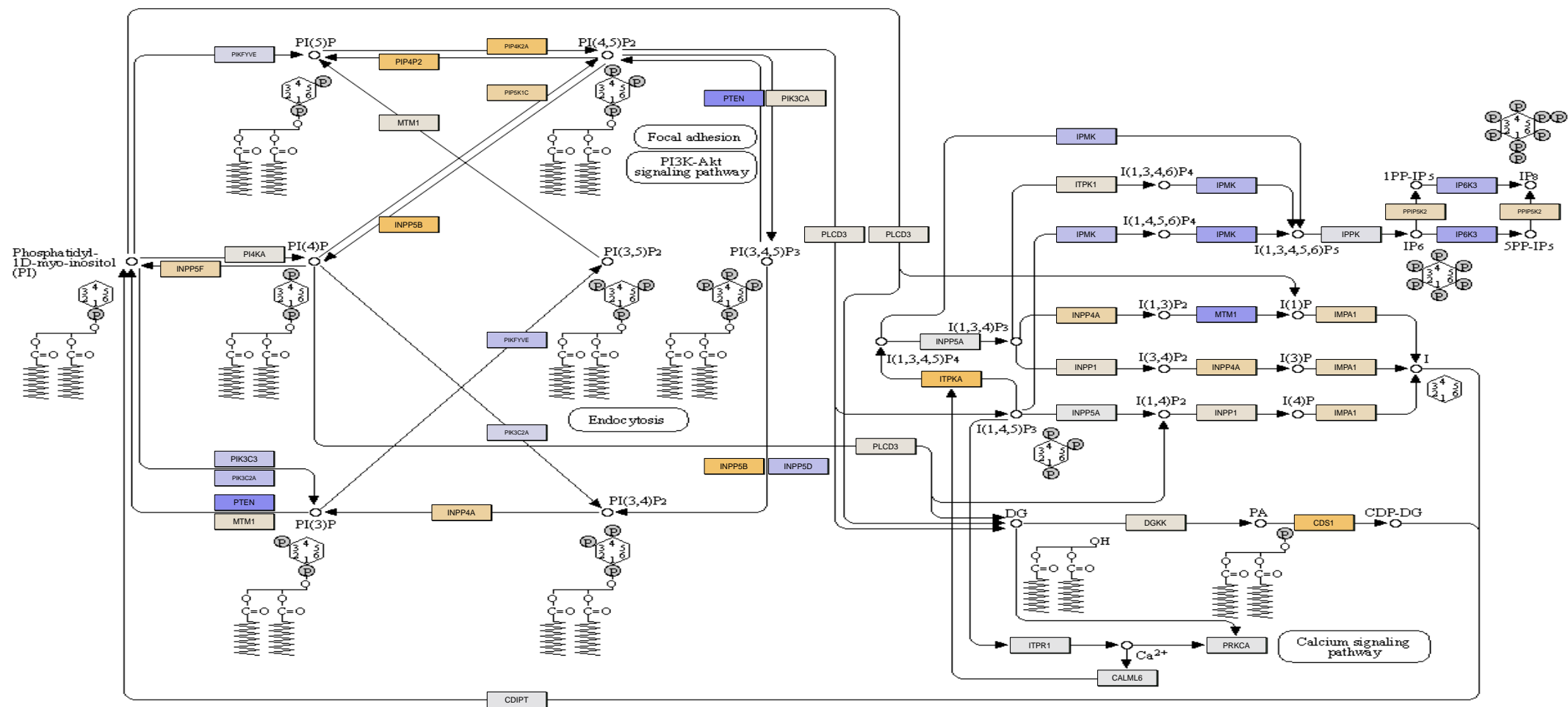

Supplement: Supplementary file 1 [file cells-11-00362-s001.zip › Suppl-Material-S3-Pathways-PSF_Expression/Phosphatidylinositol_signaling_system.pdf]

all genes

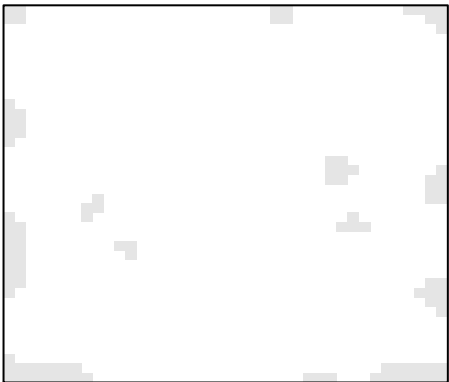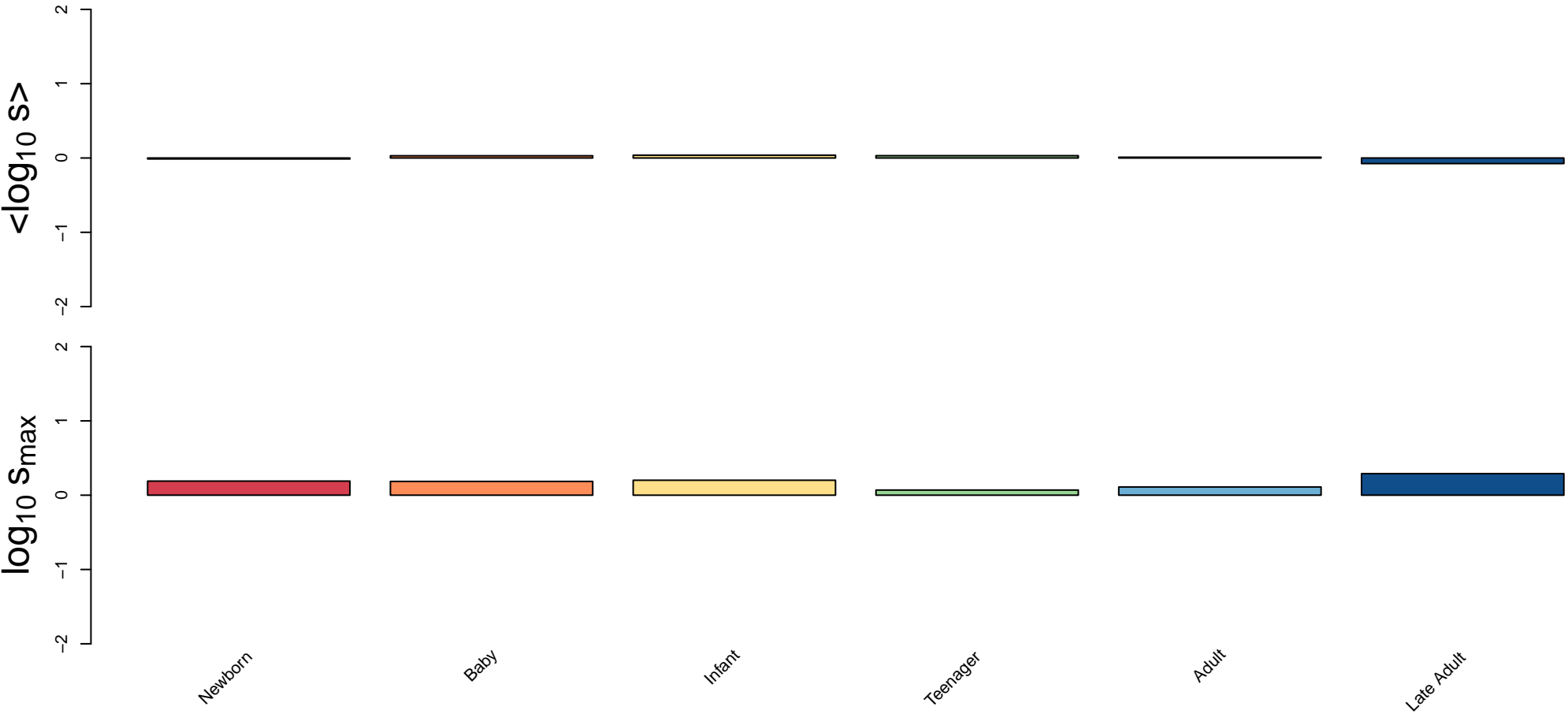

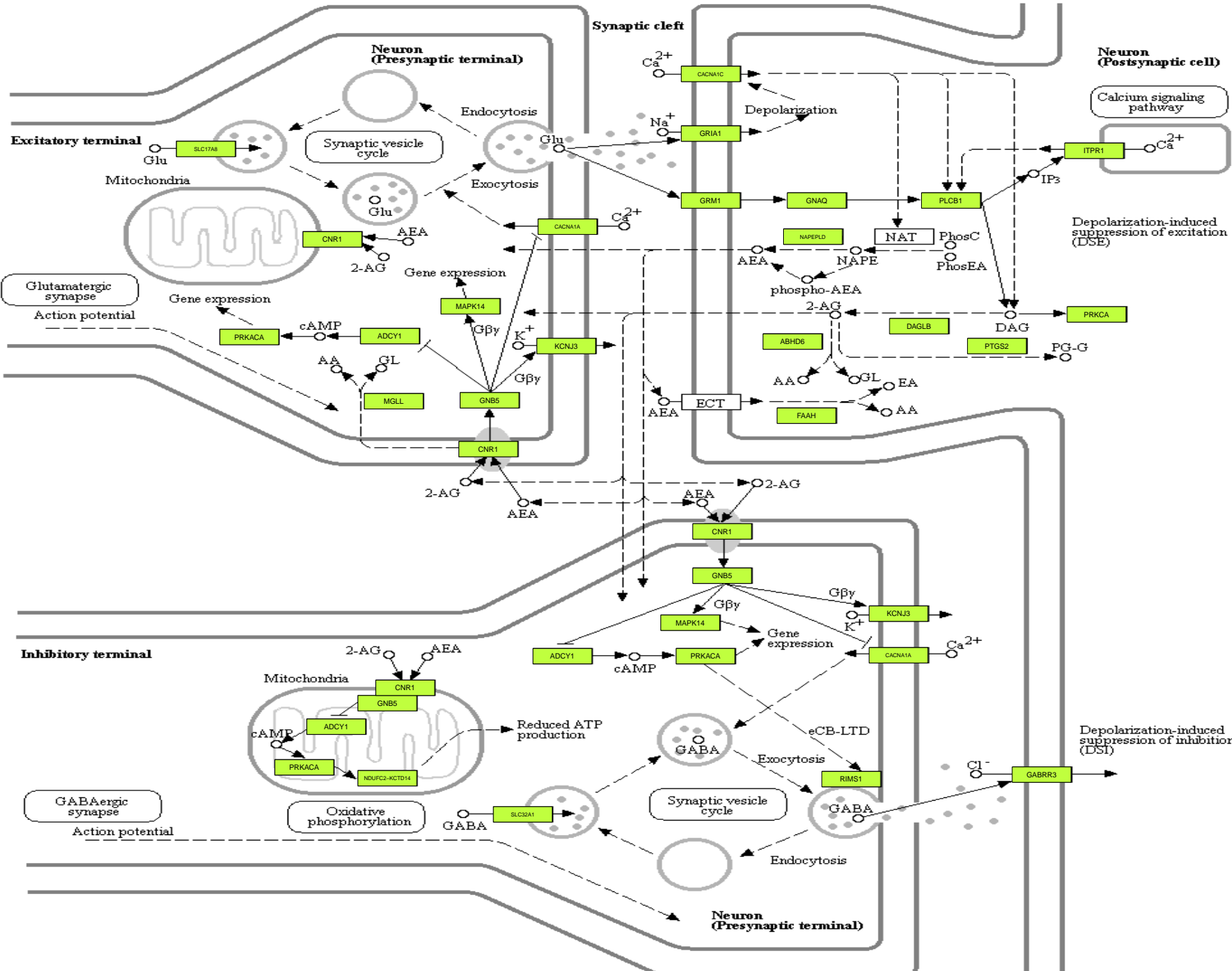

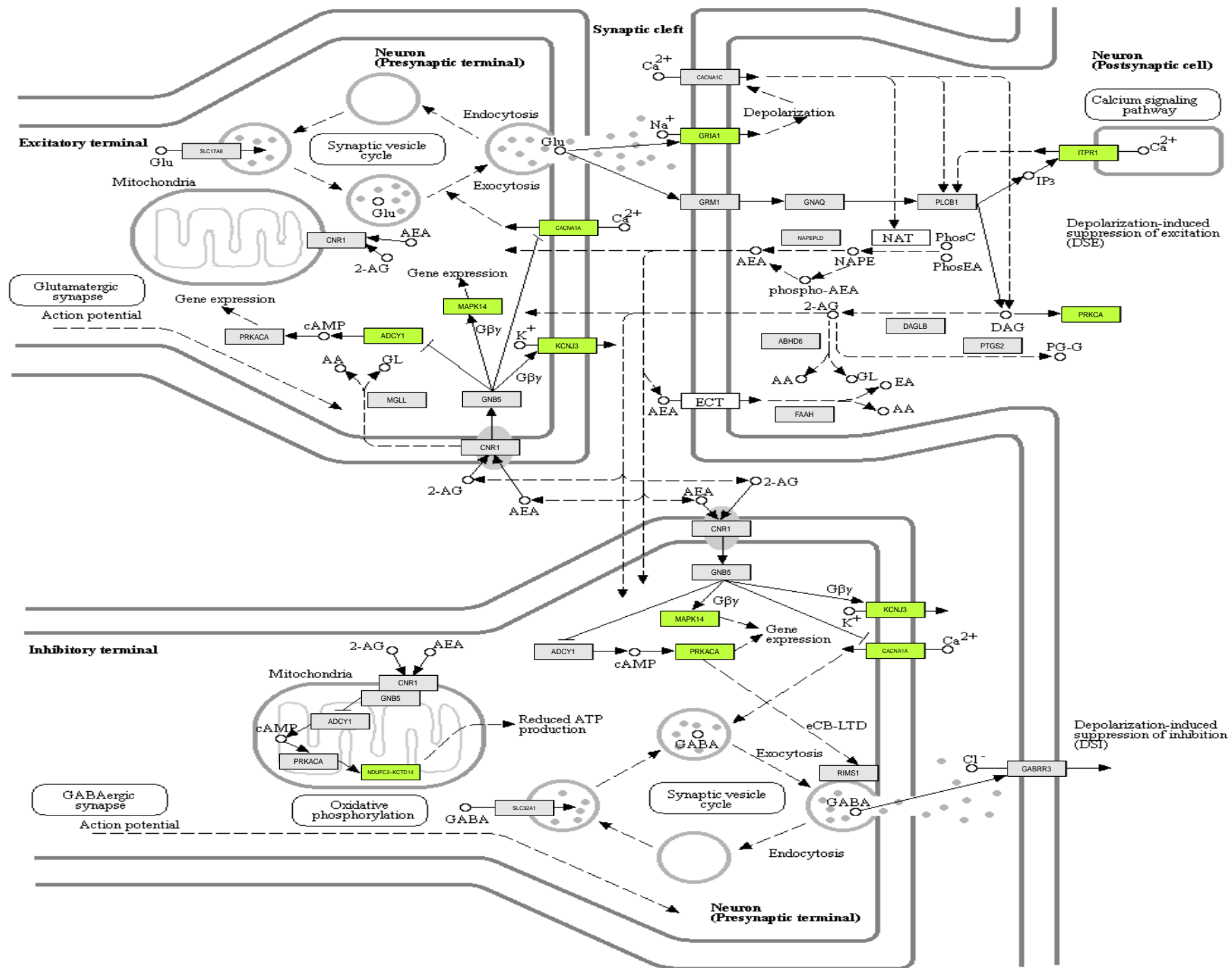

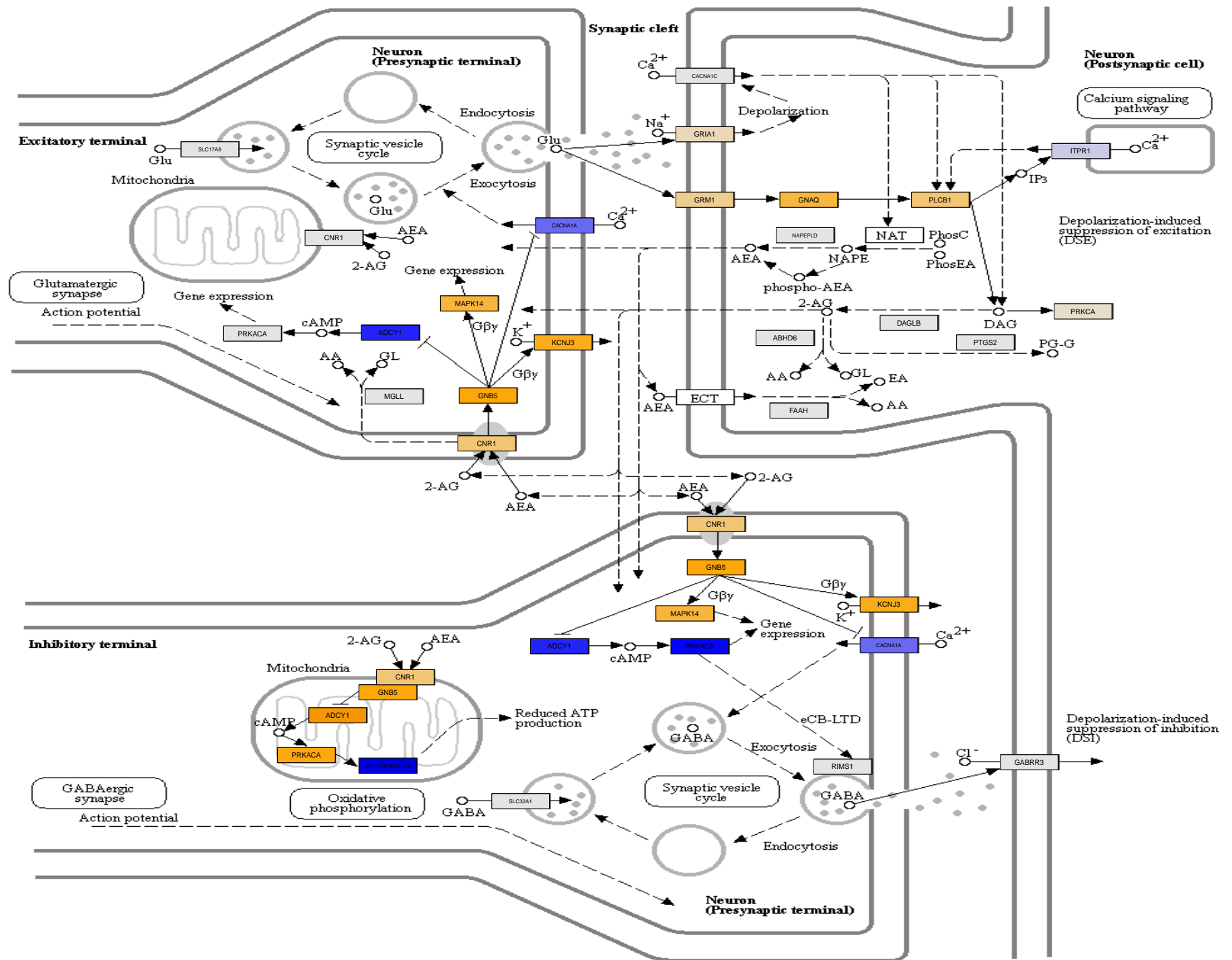

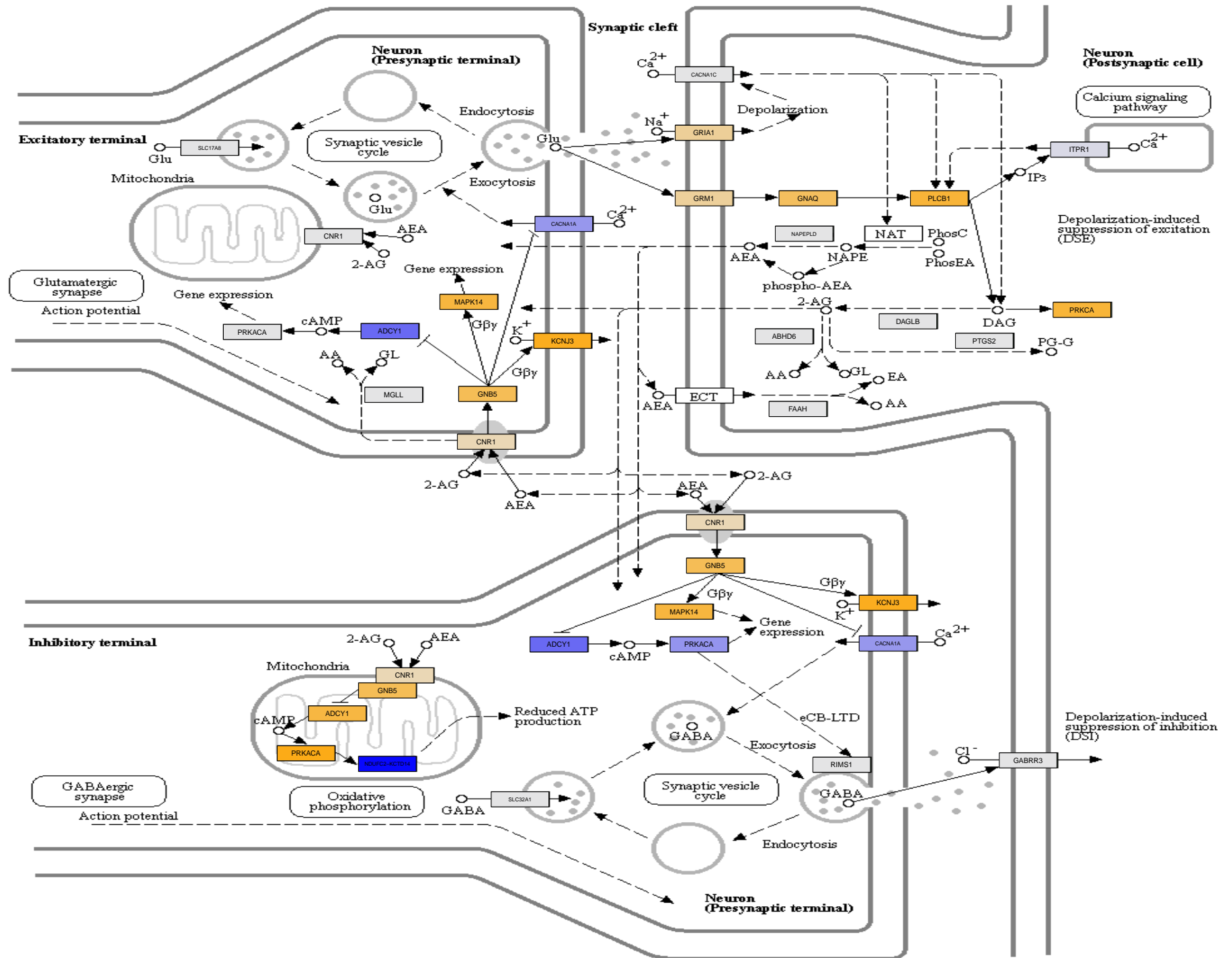

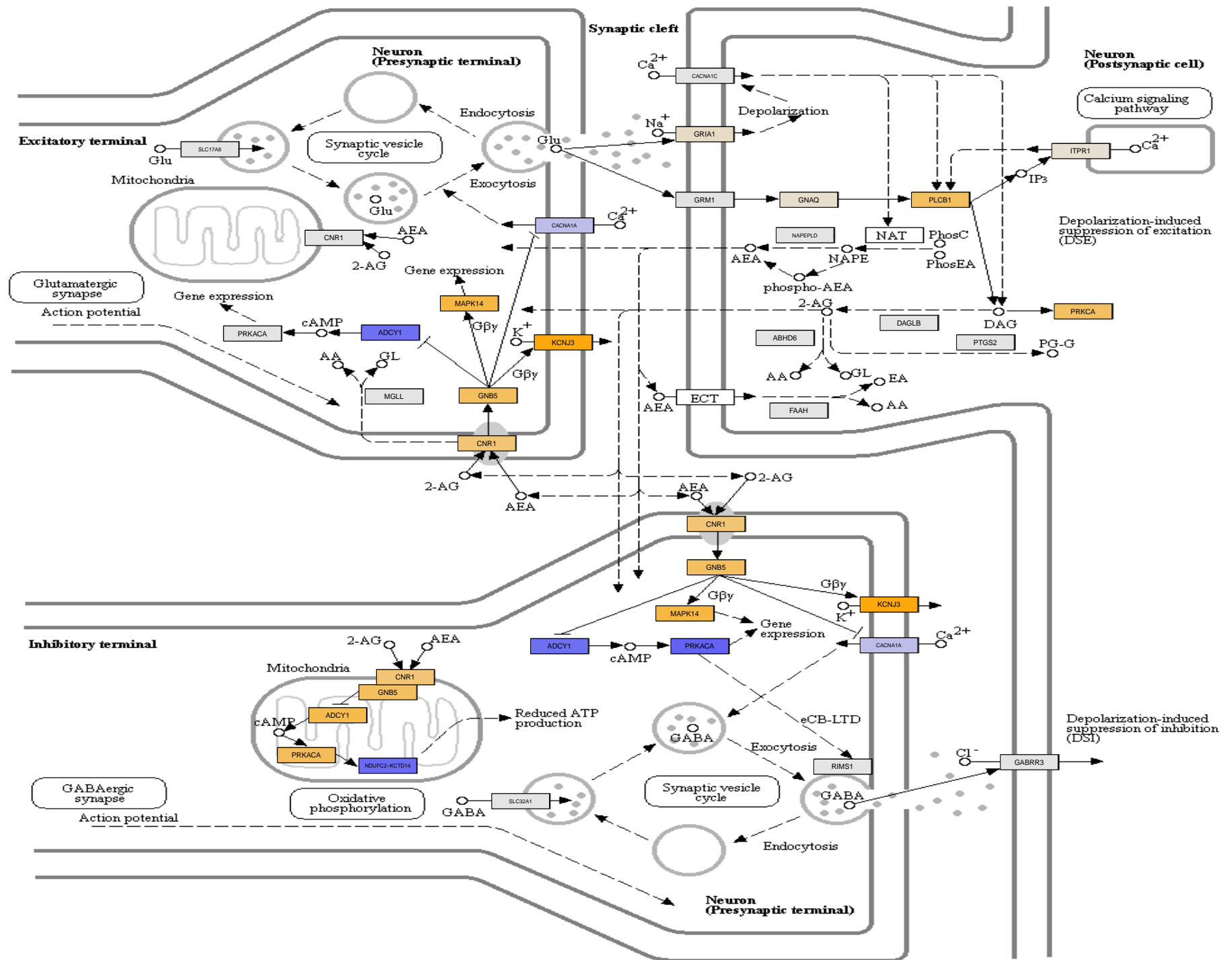

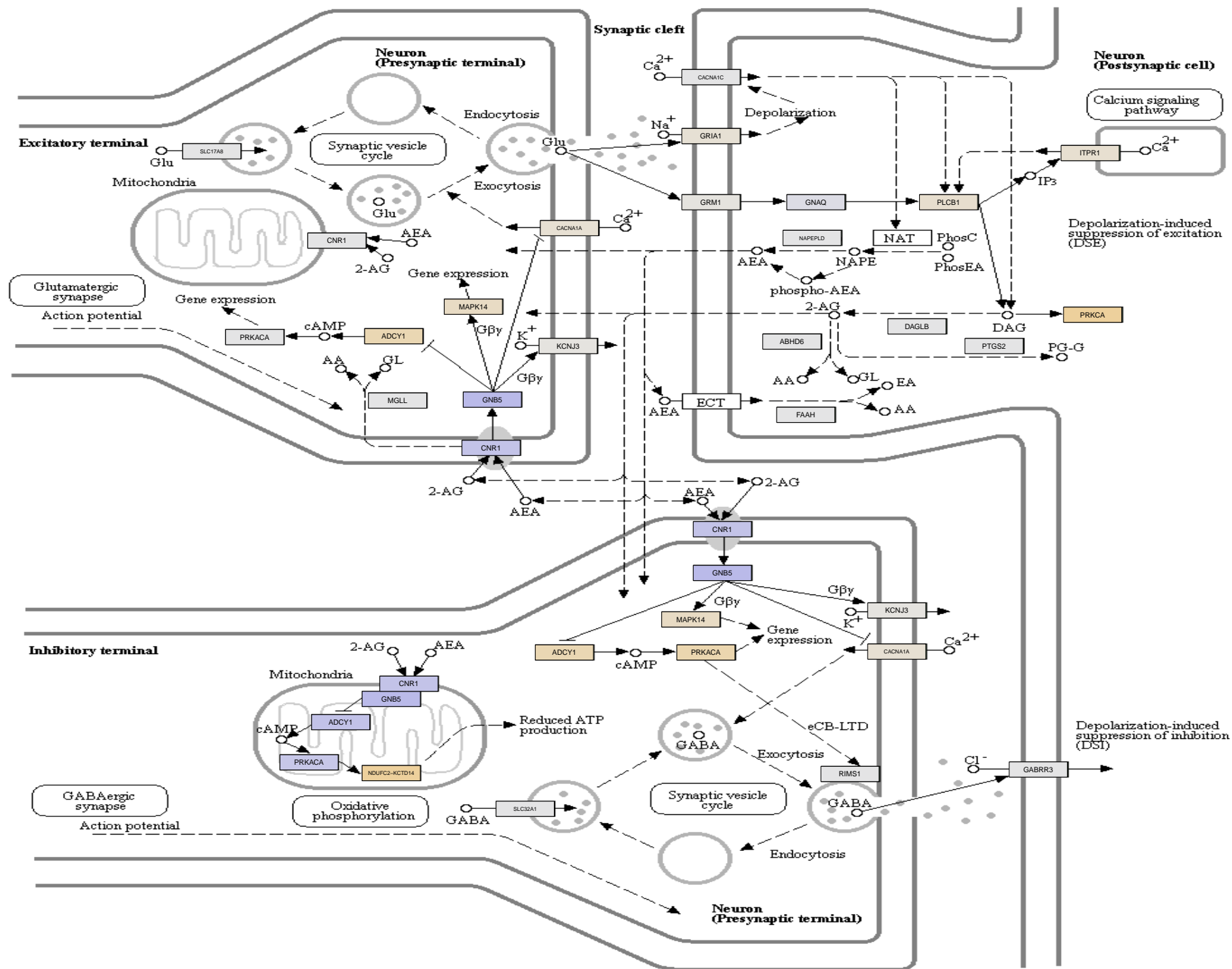

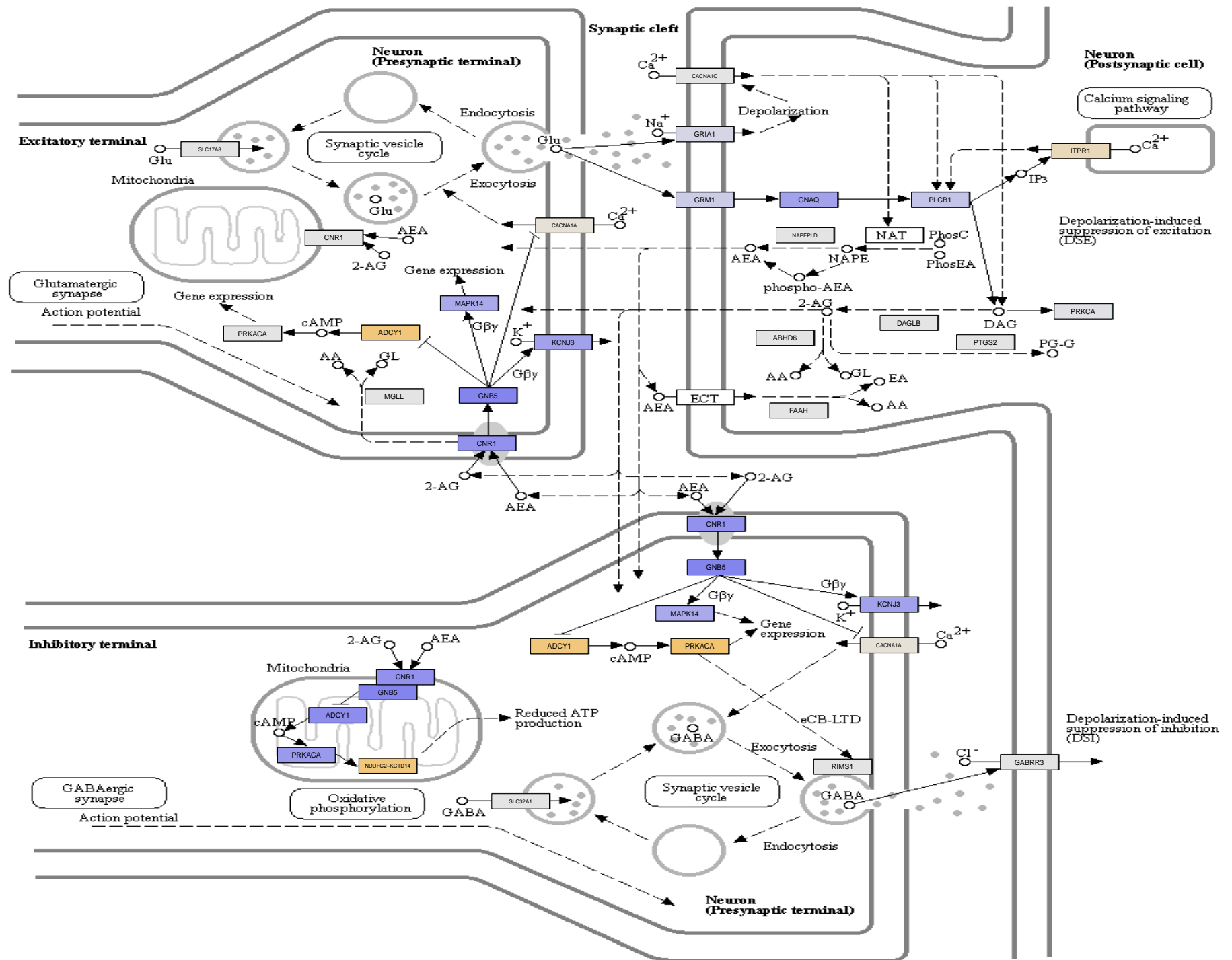

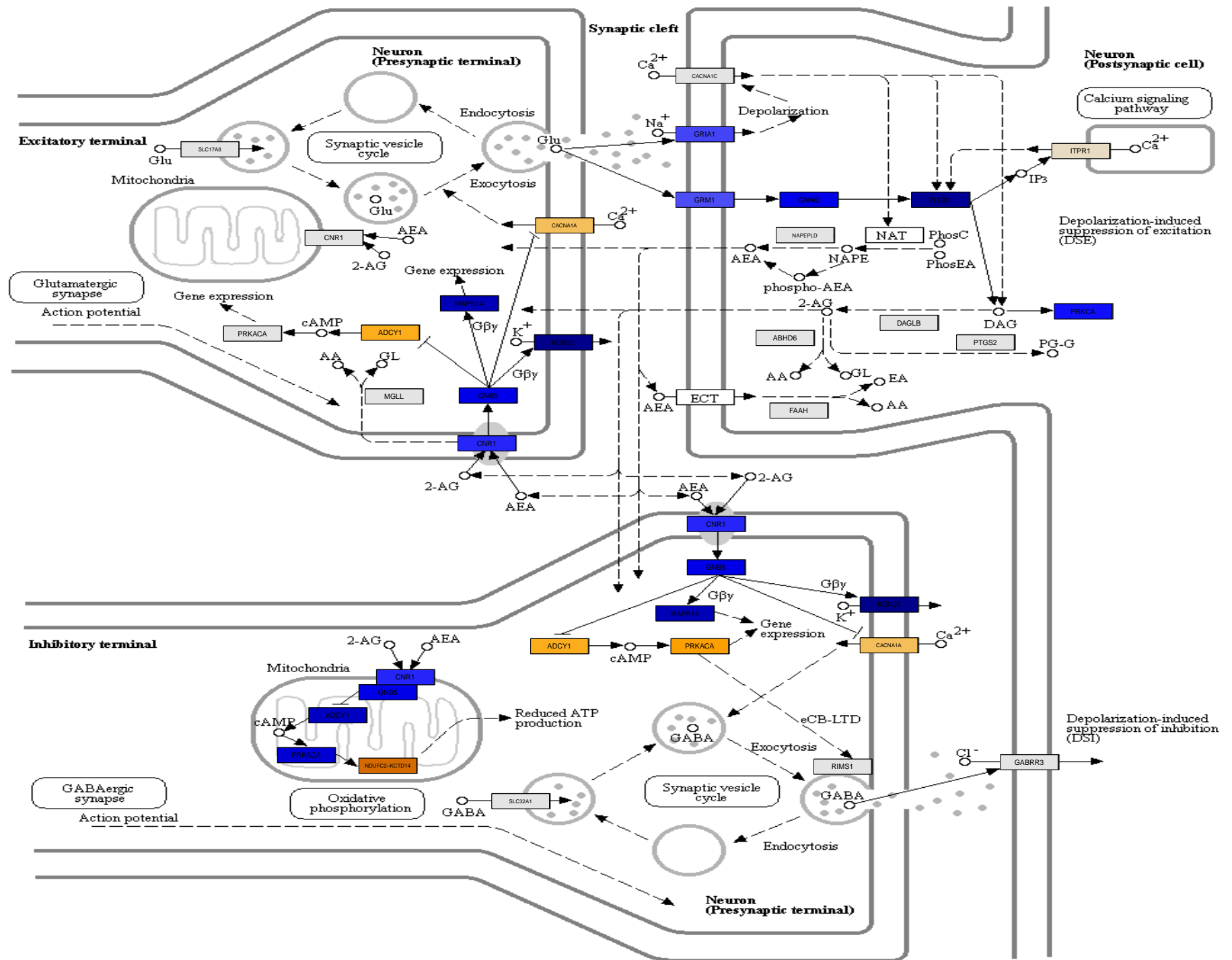

Supplement: Supplementary file 1 [file cells-11-00362-s001.zip › Suppl-Material-S3-Pathways-PSF_Expression/Retrograde_endocannabinoid_signaling.pdf]

Sphingolipid signaling pathway

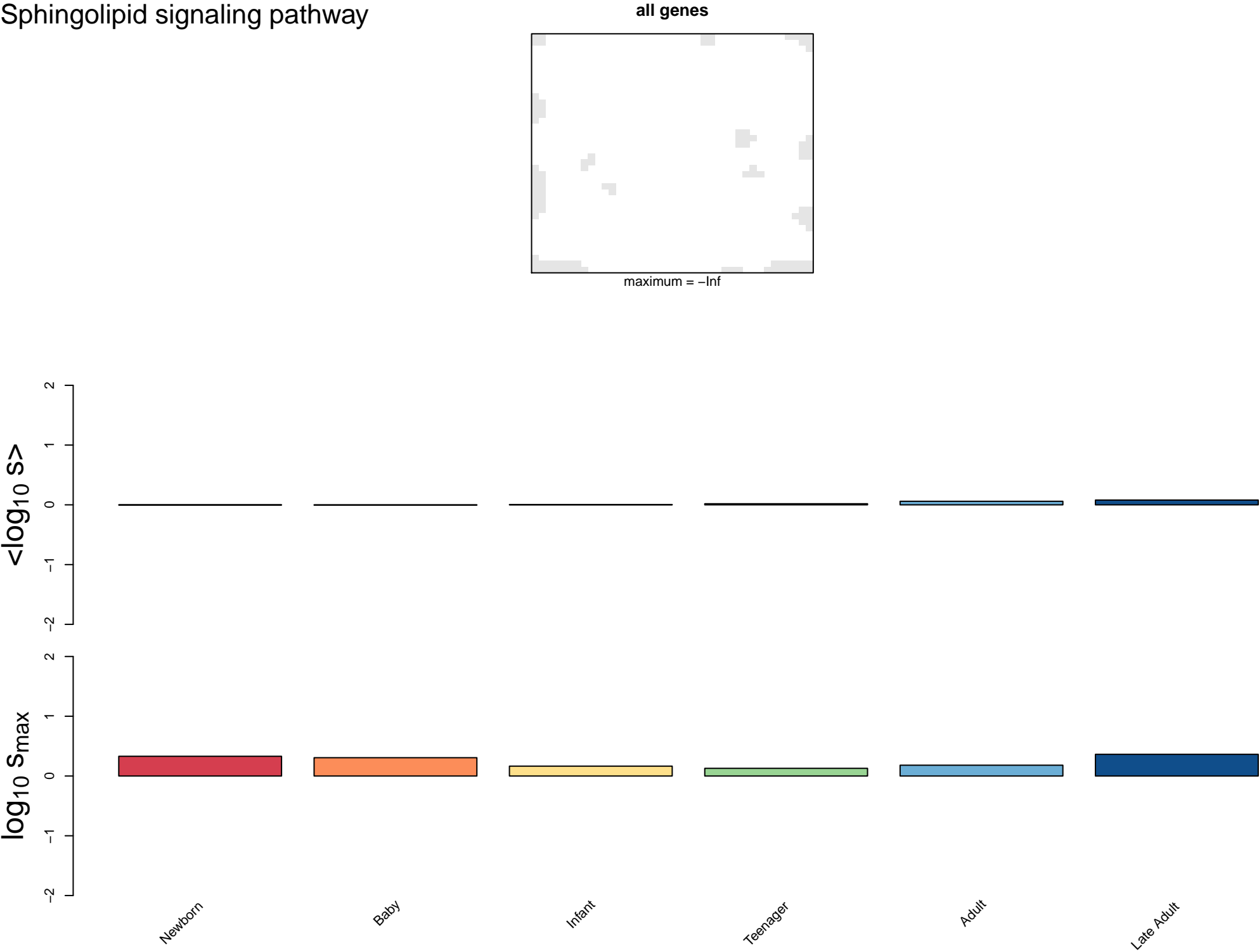

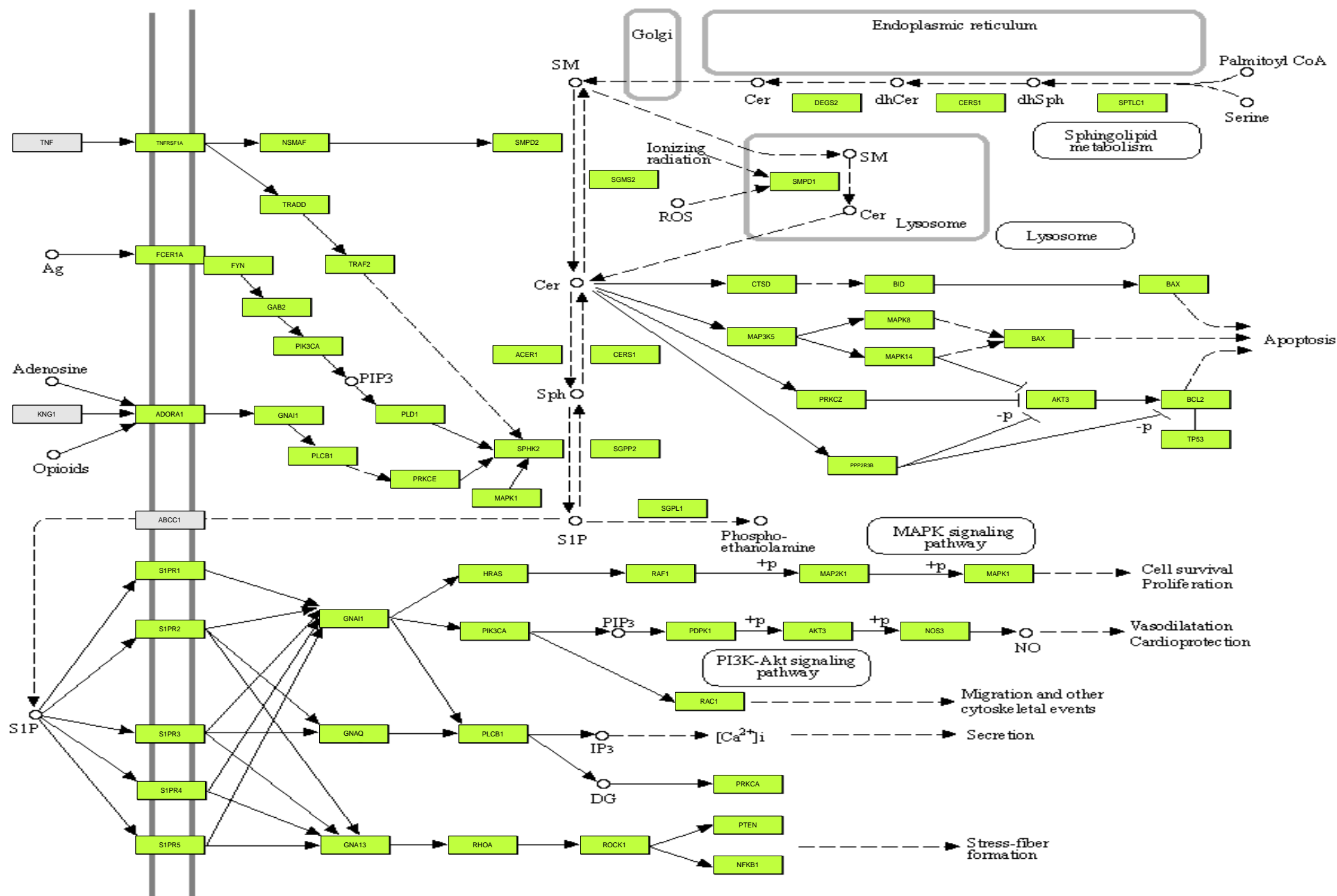

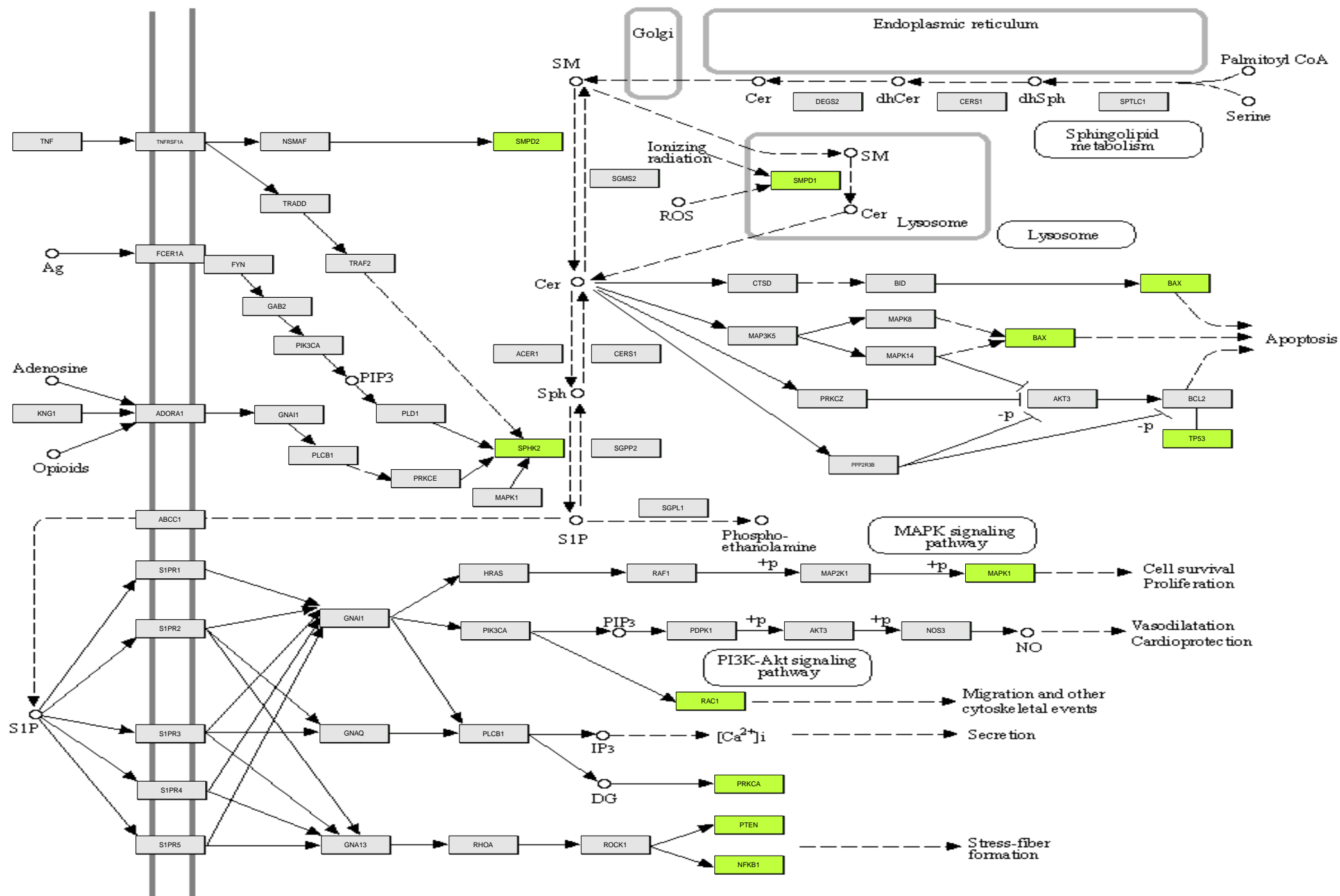

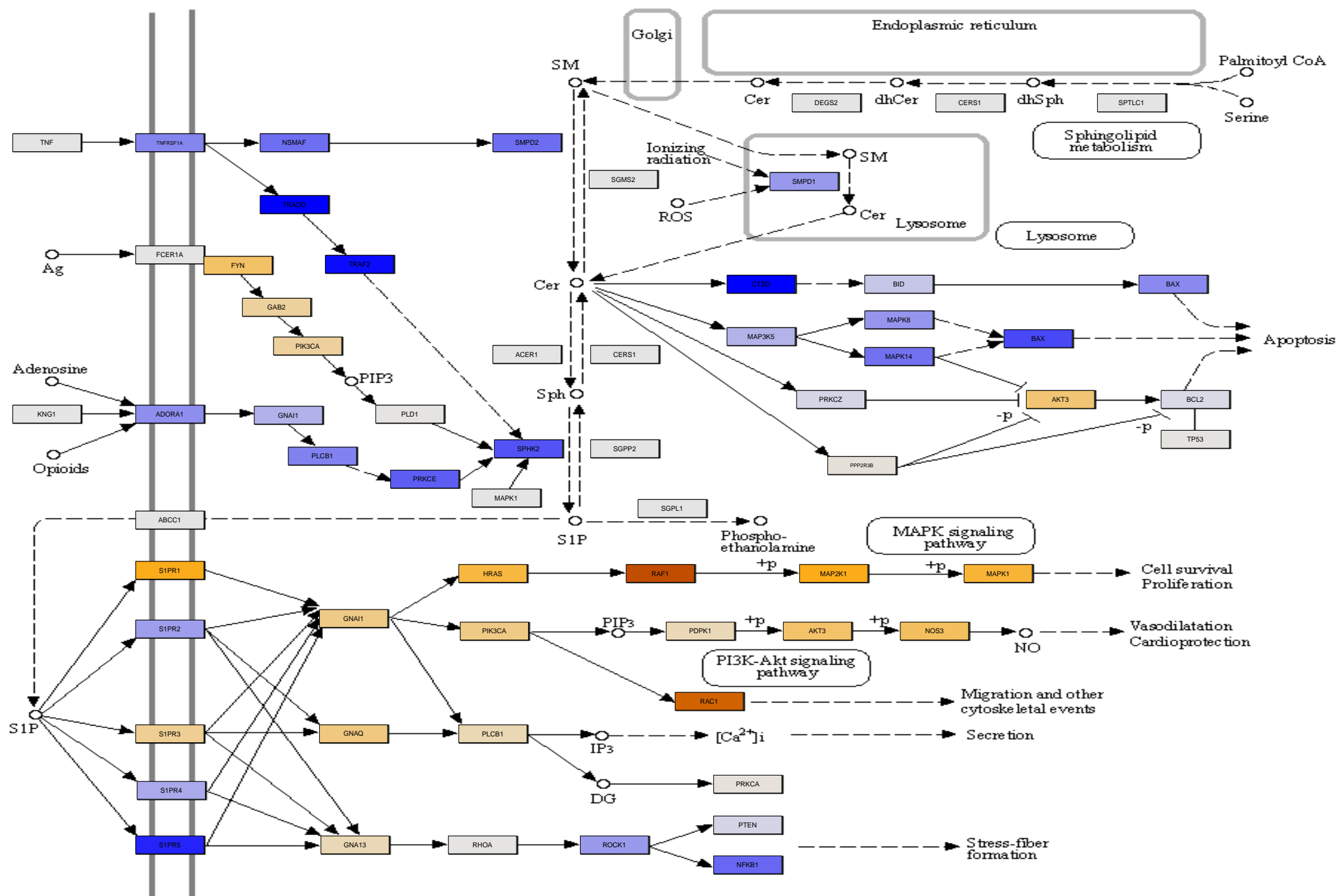

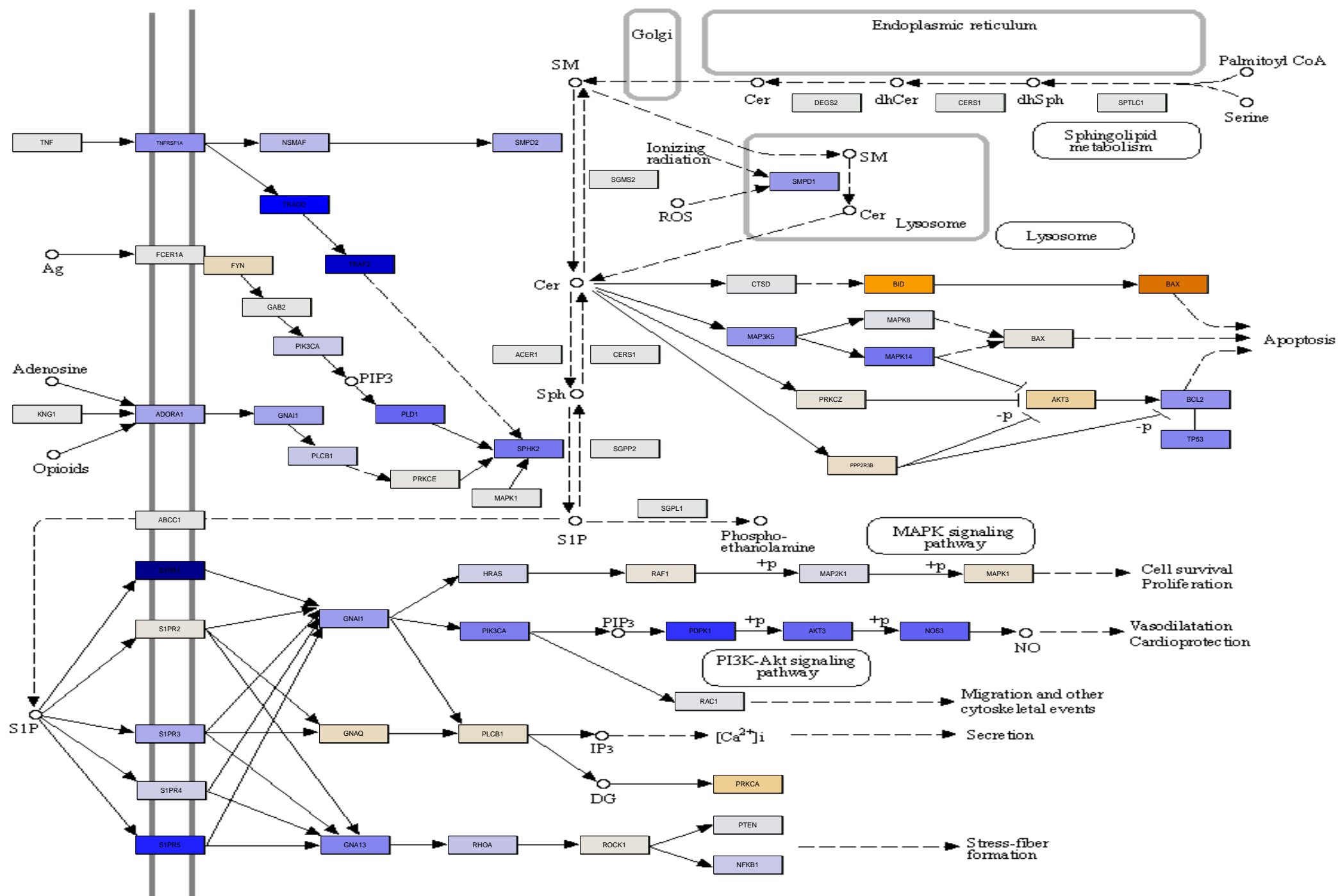

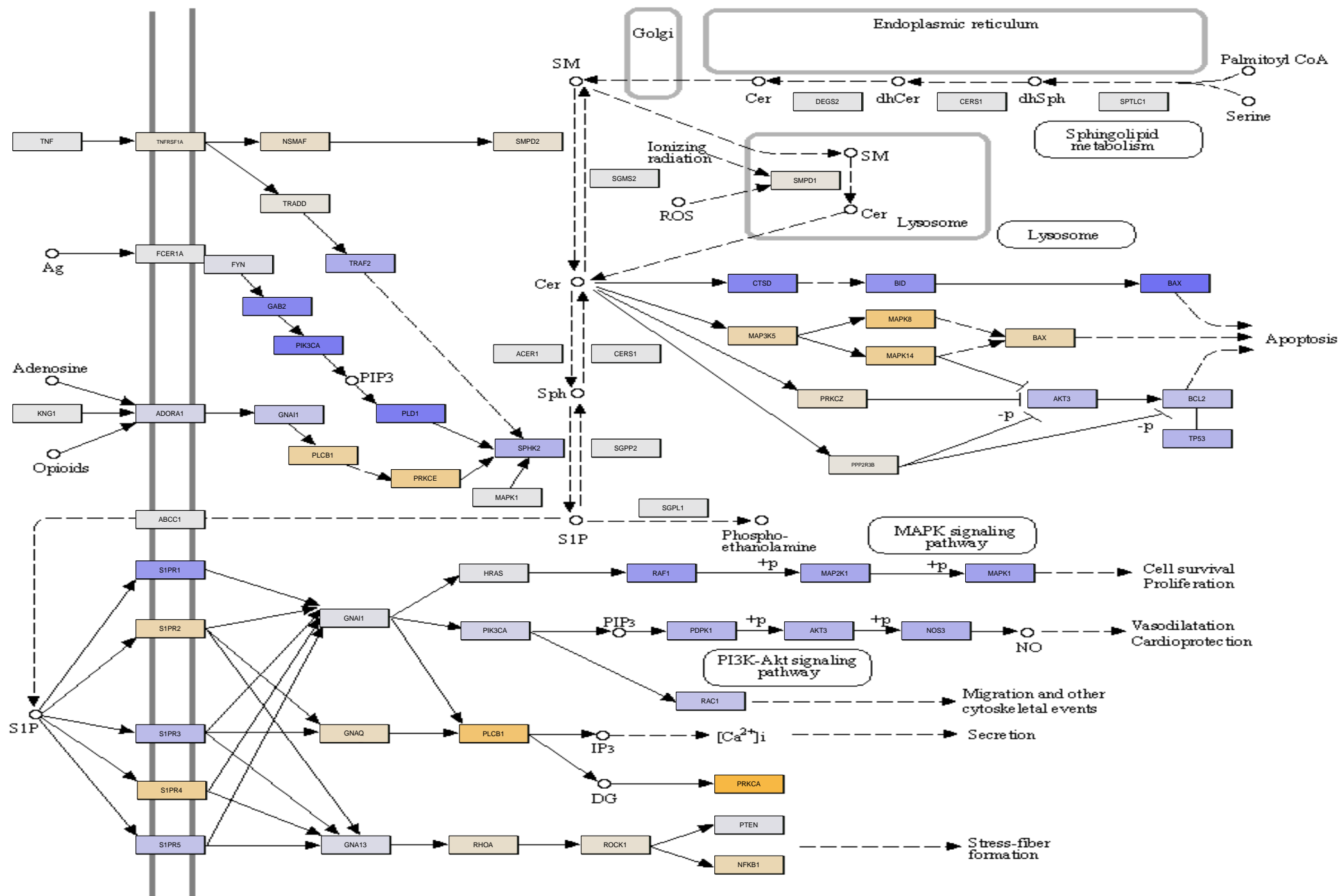

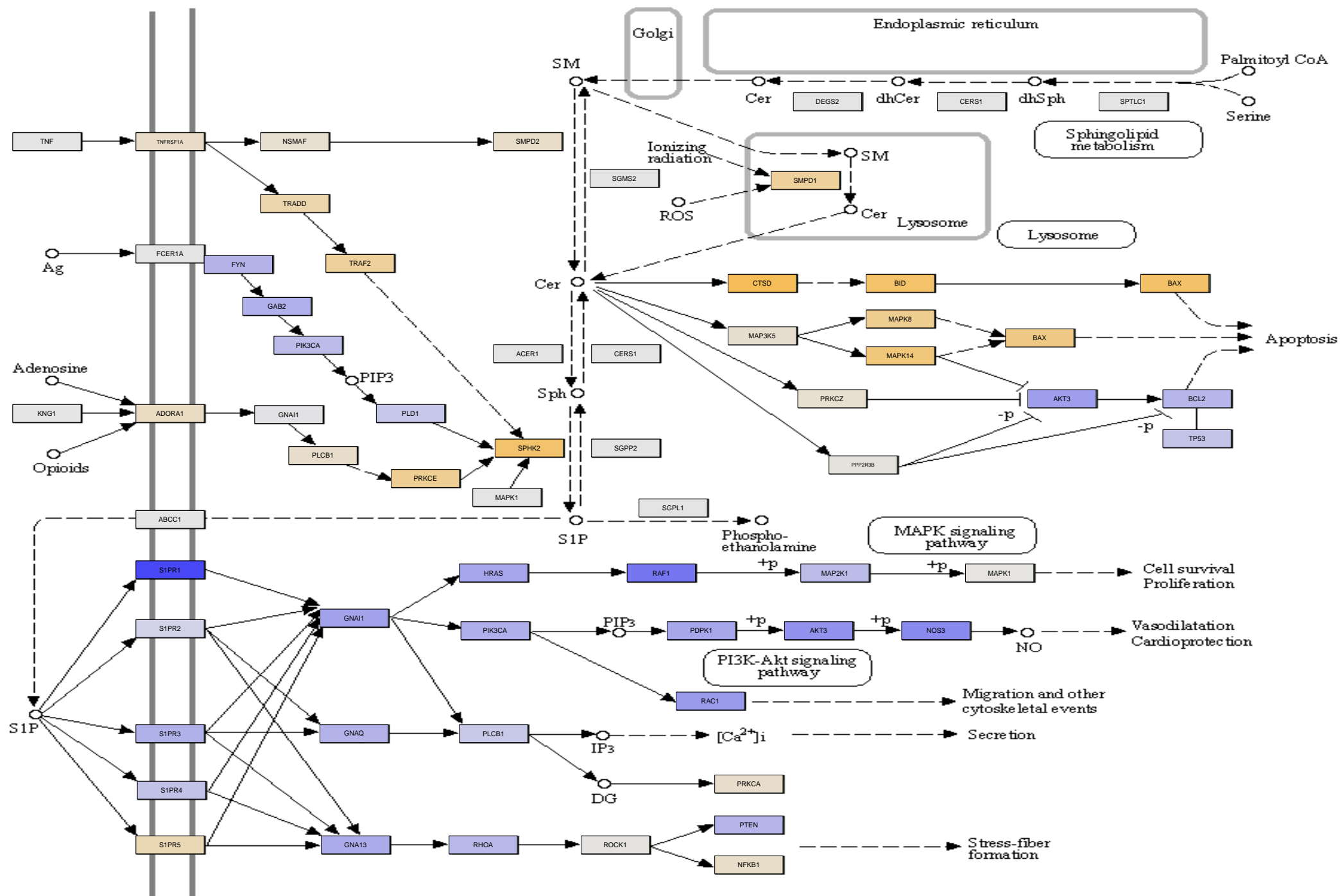

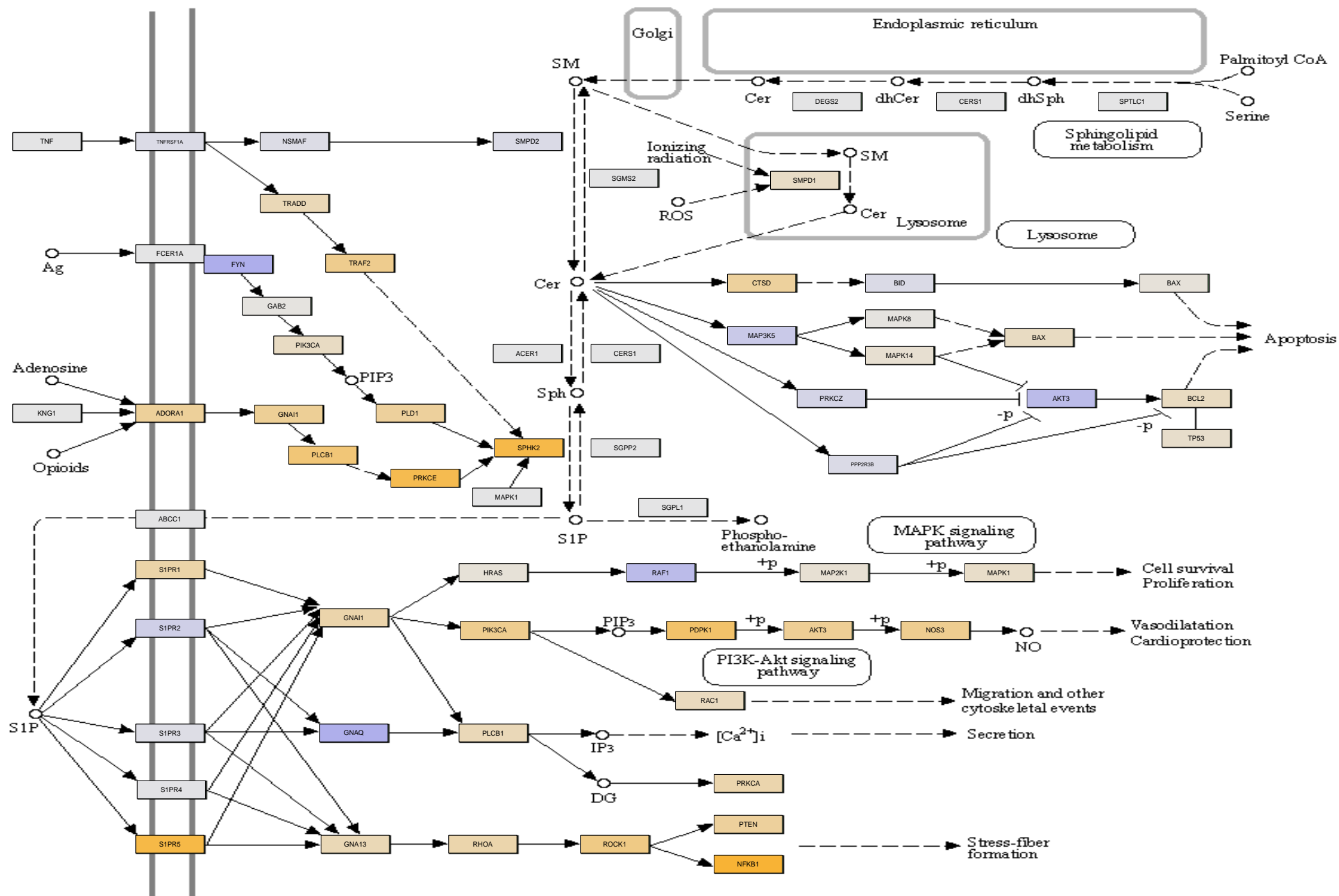

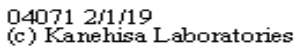

Supplement: Supplementary file 1 [file cells-11-00362-s001.zip › Suppl-Material-S3-Pathways-PSF_Expression/Sphingolipid_signaling_pathway.pdf]

Thyroid hormone signaling pathway

all genes

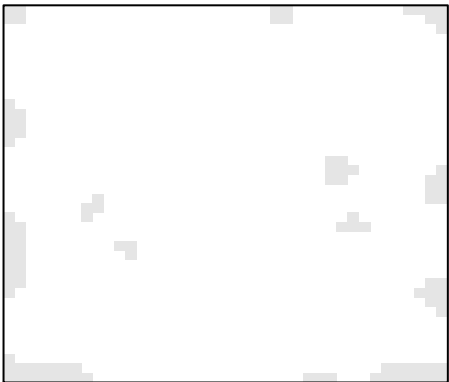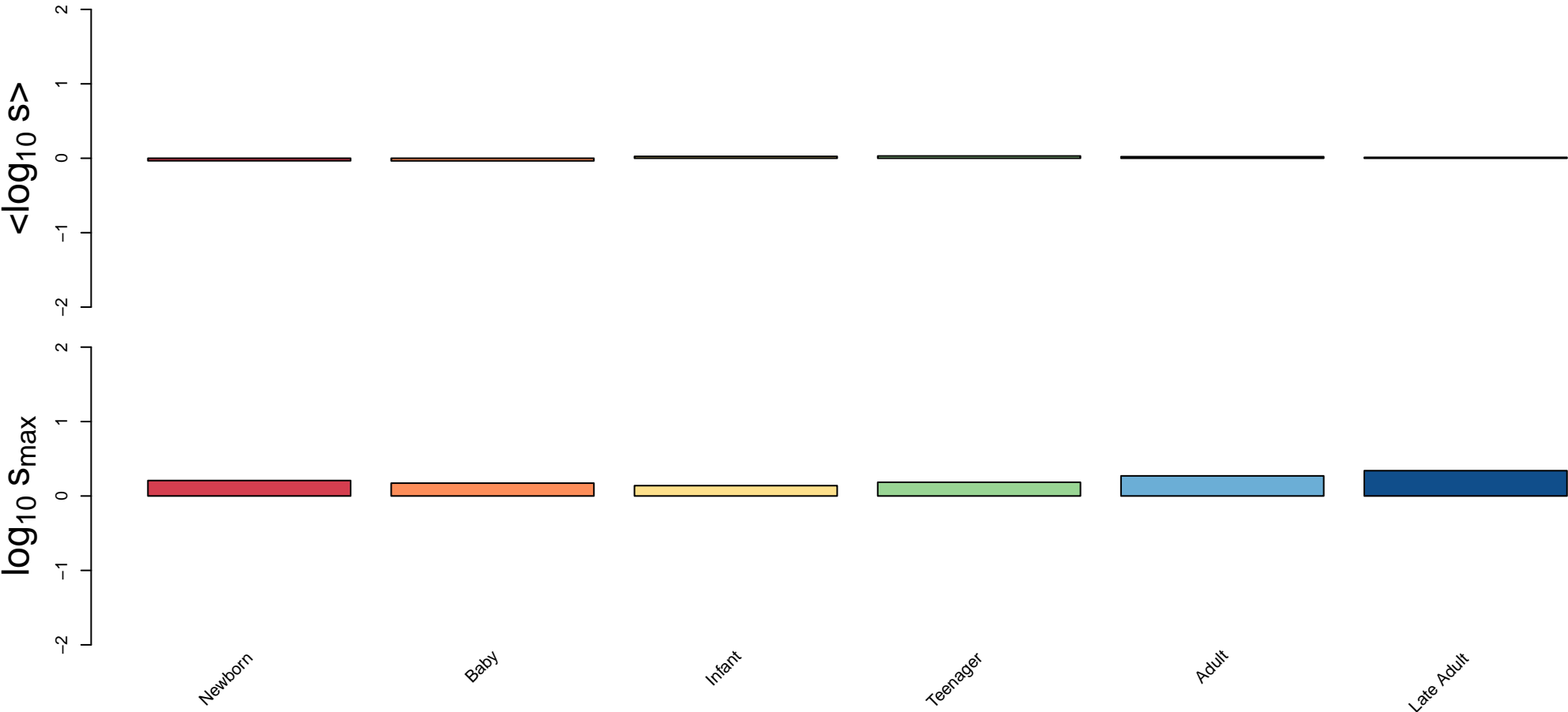

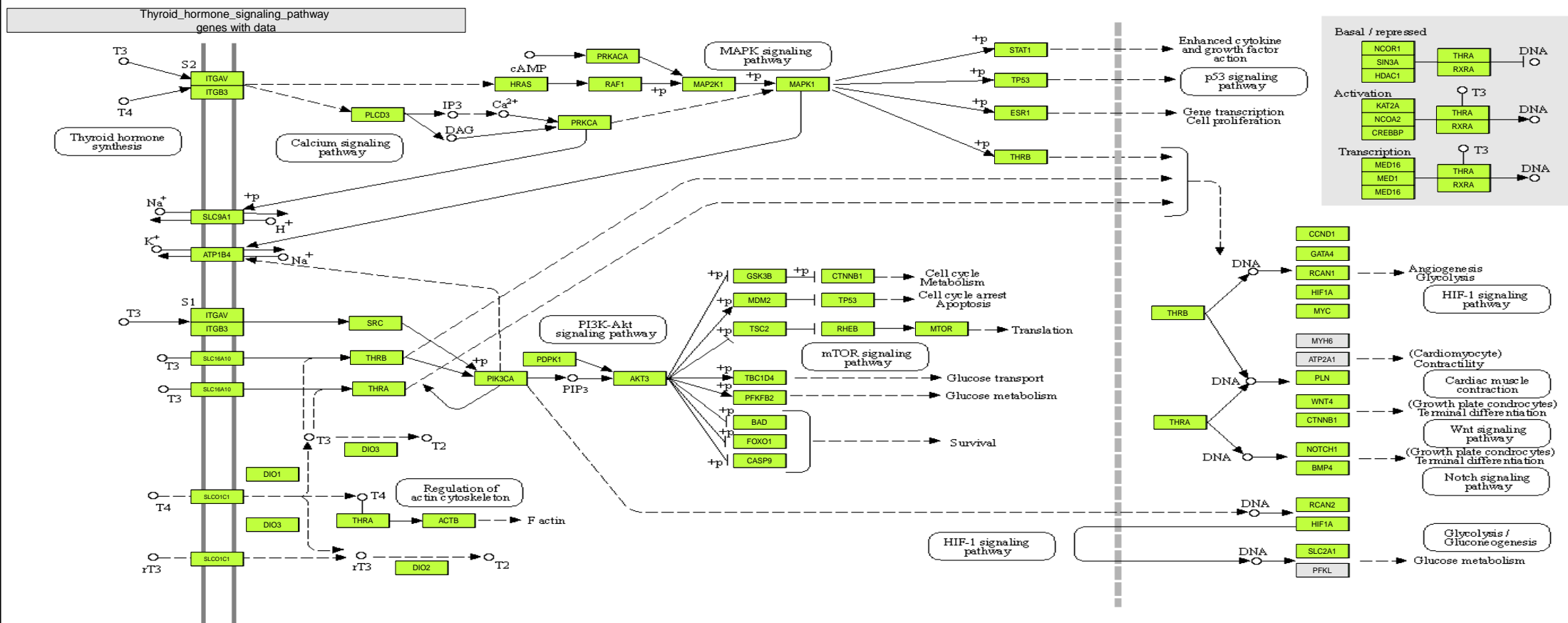

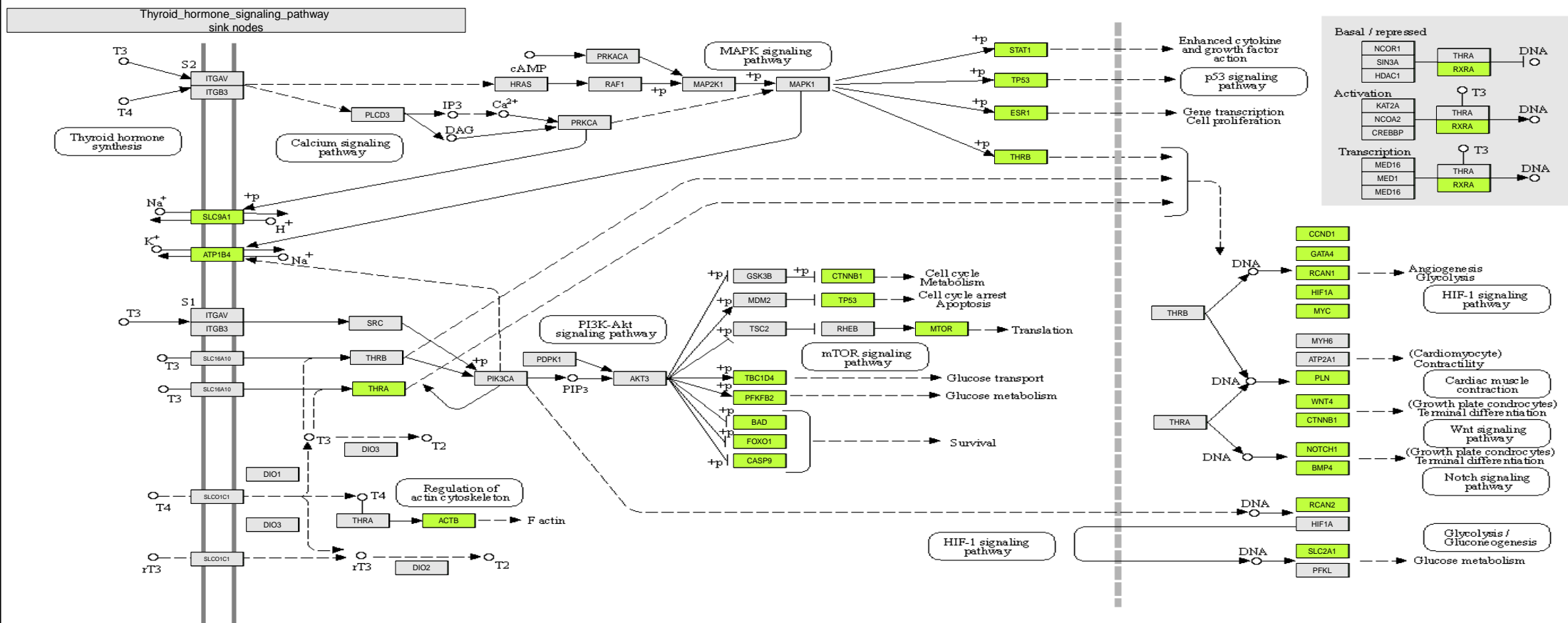

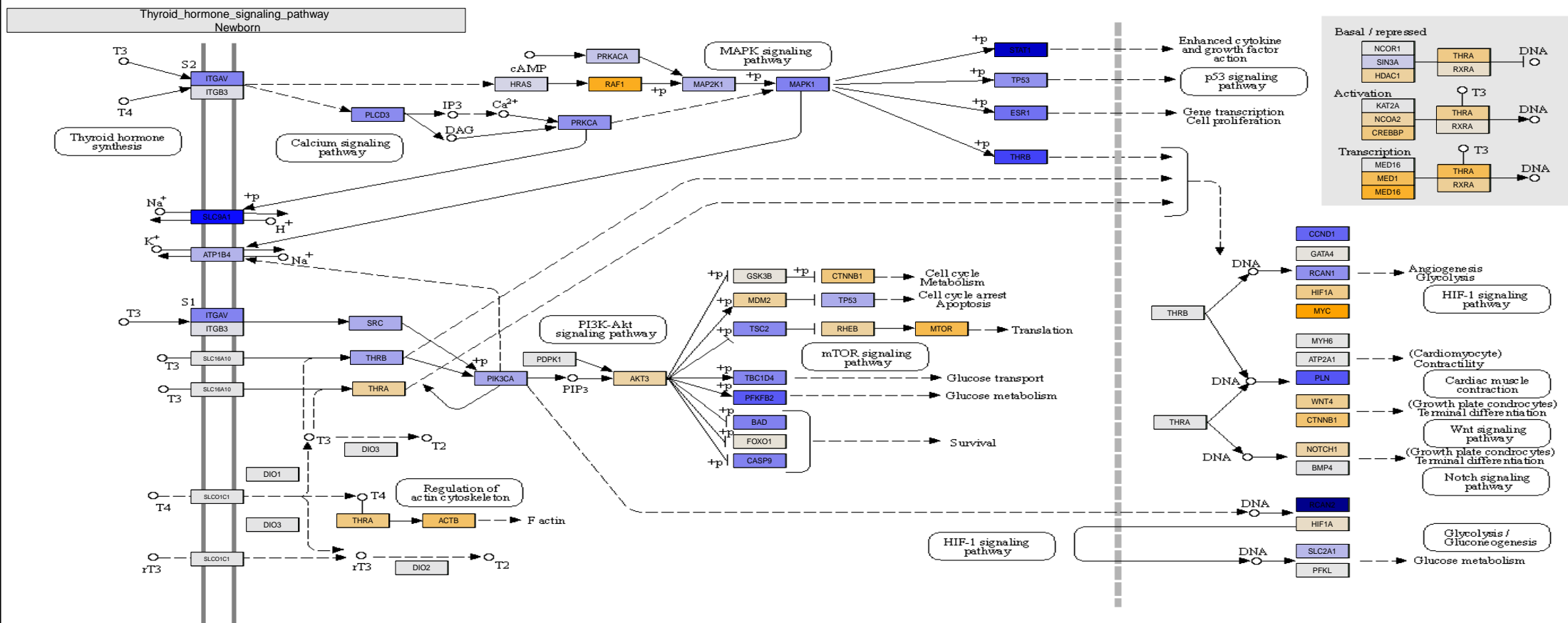

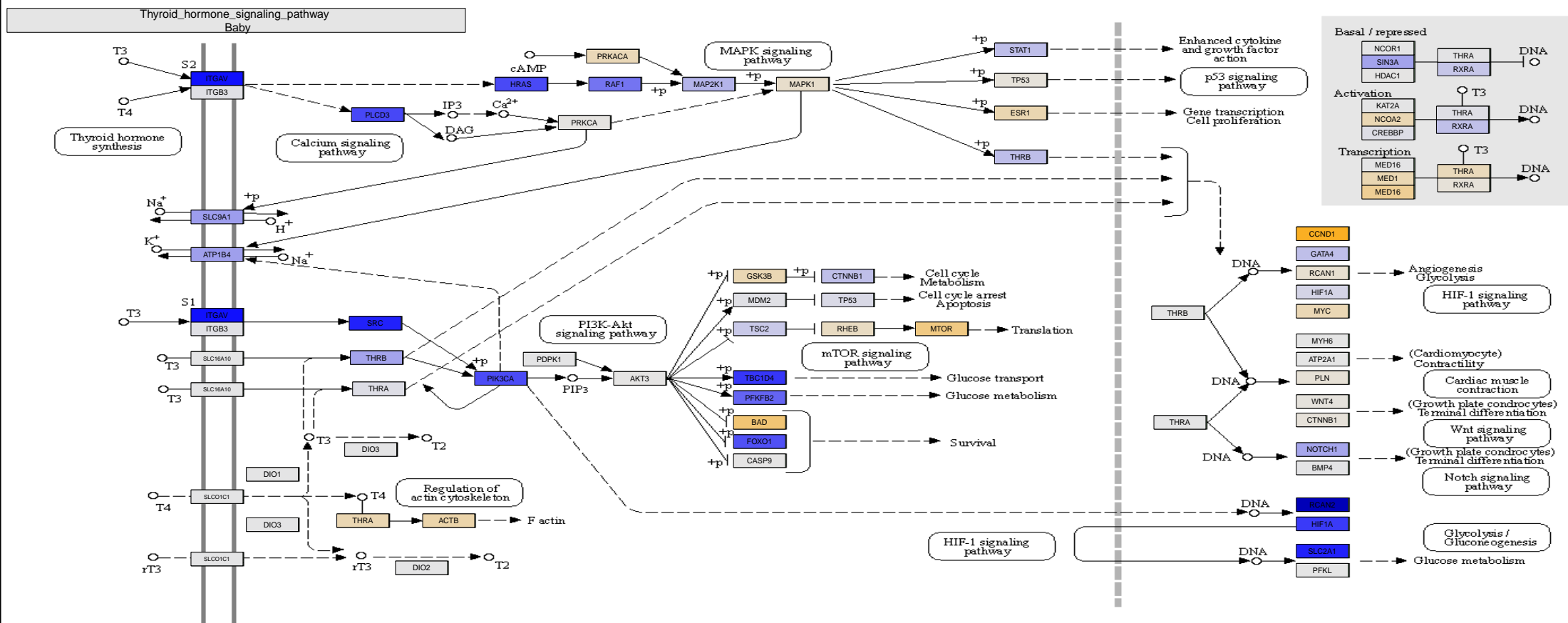

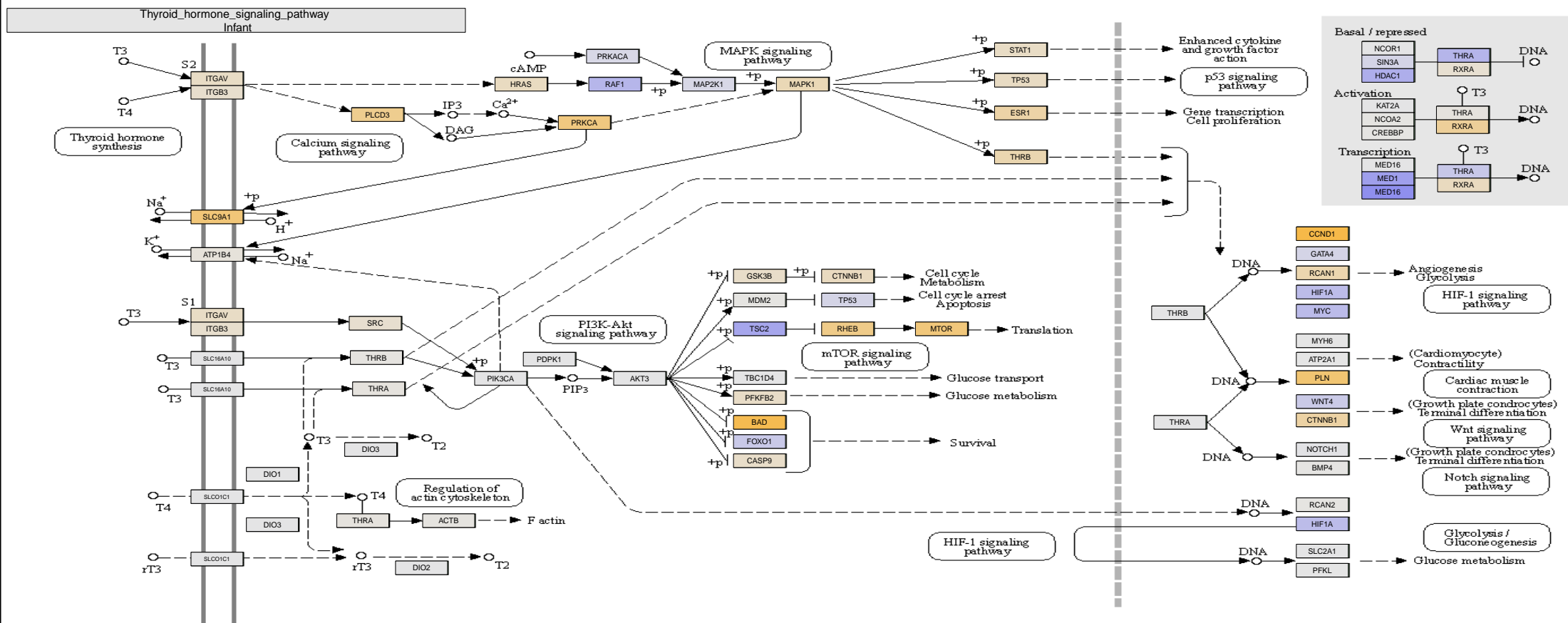

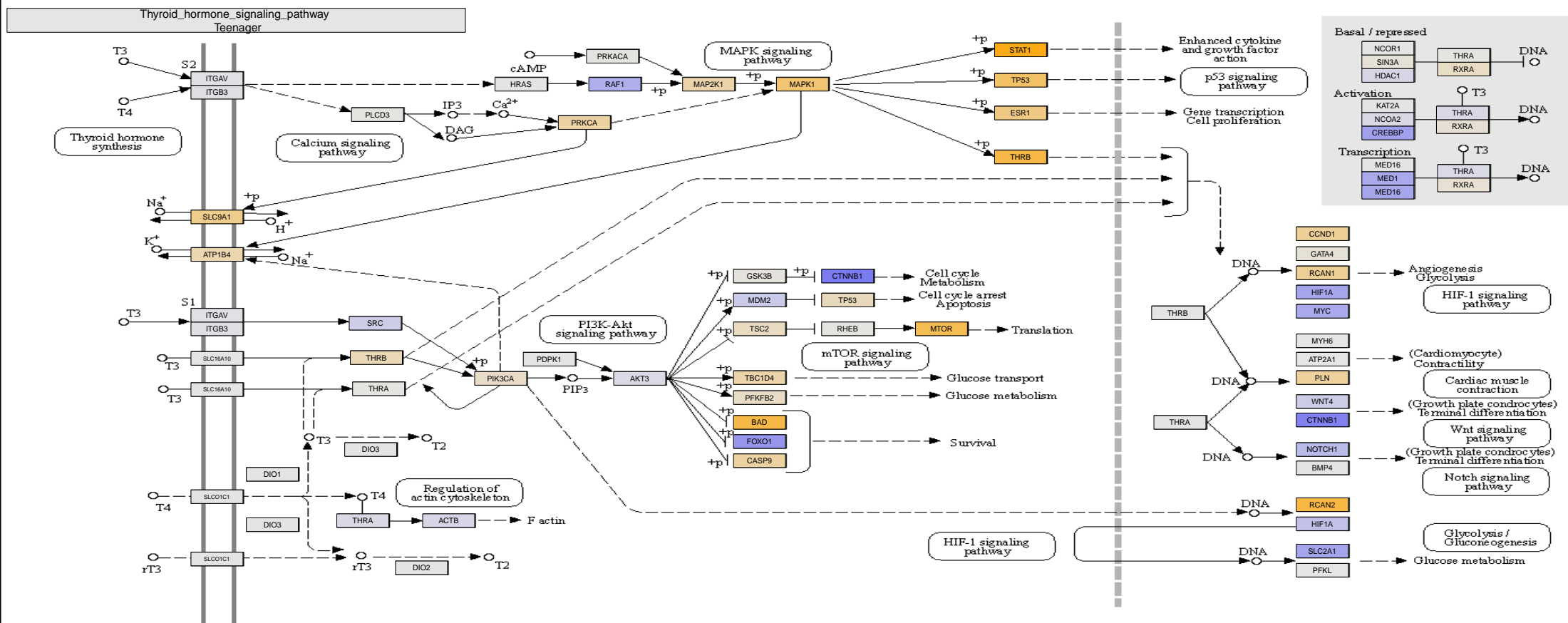

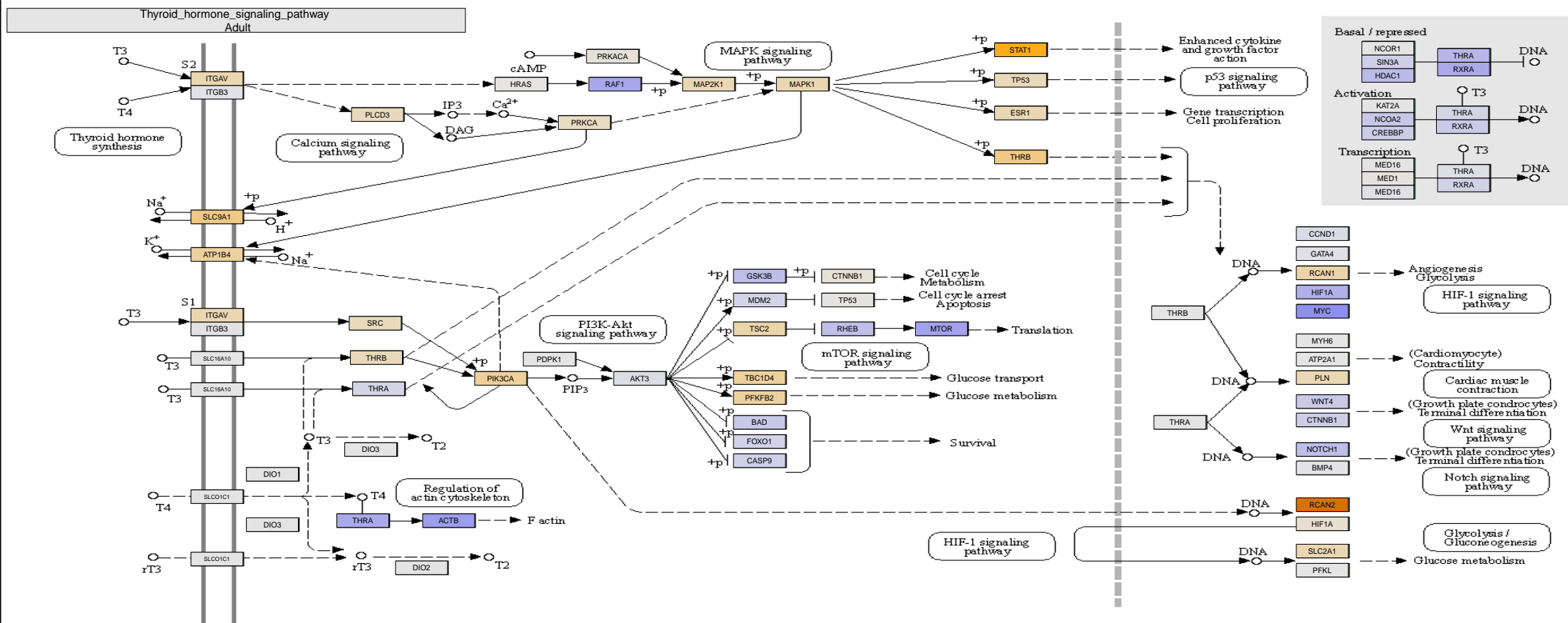

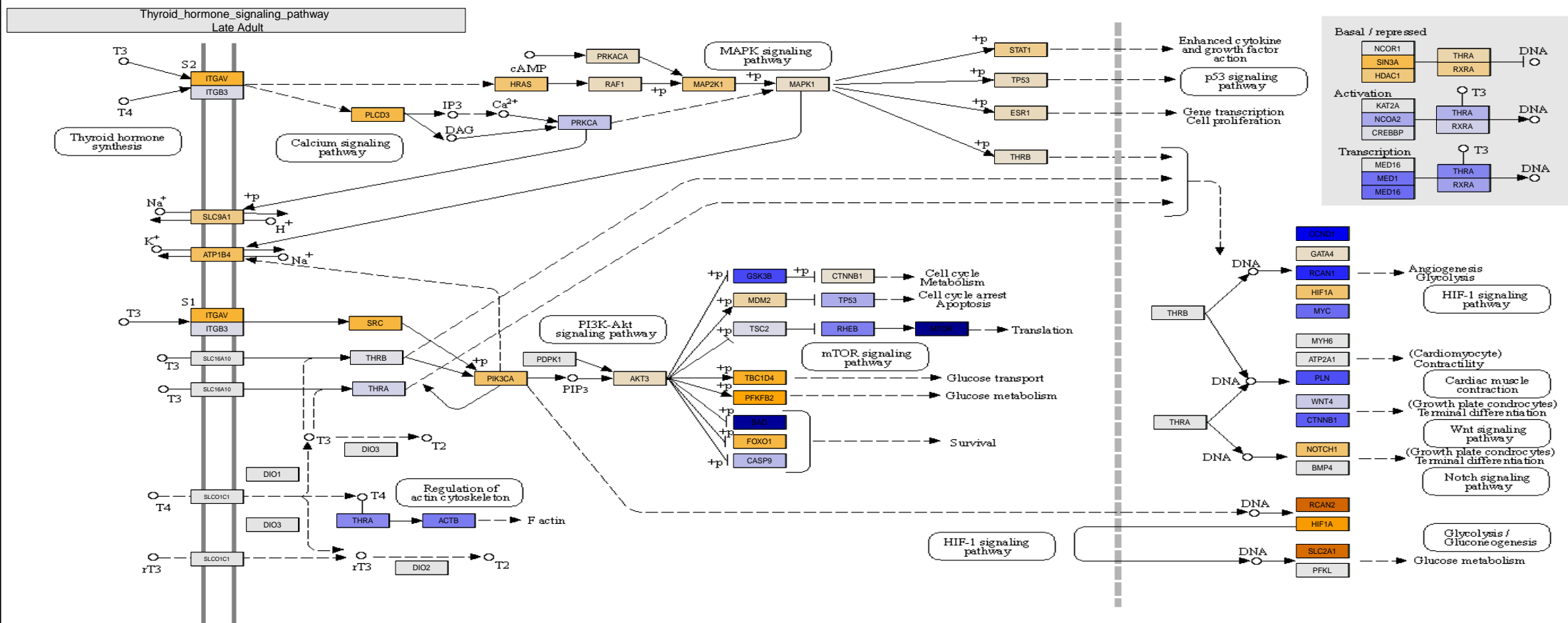

Supplement: Supplementary file 1 [file cells-11-00362-s001.zip › Suppl-Material-S3-Pathways-PSF_Expression/Thyroid_hormone_signaling_pathway.pdf]

Toll-like receptor signaling pathway

all genes

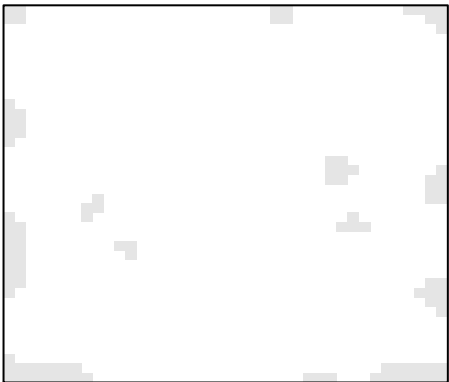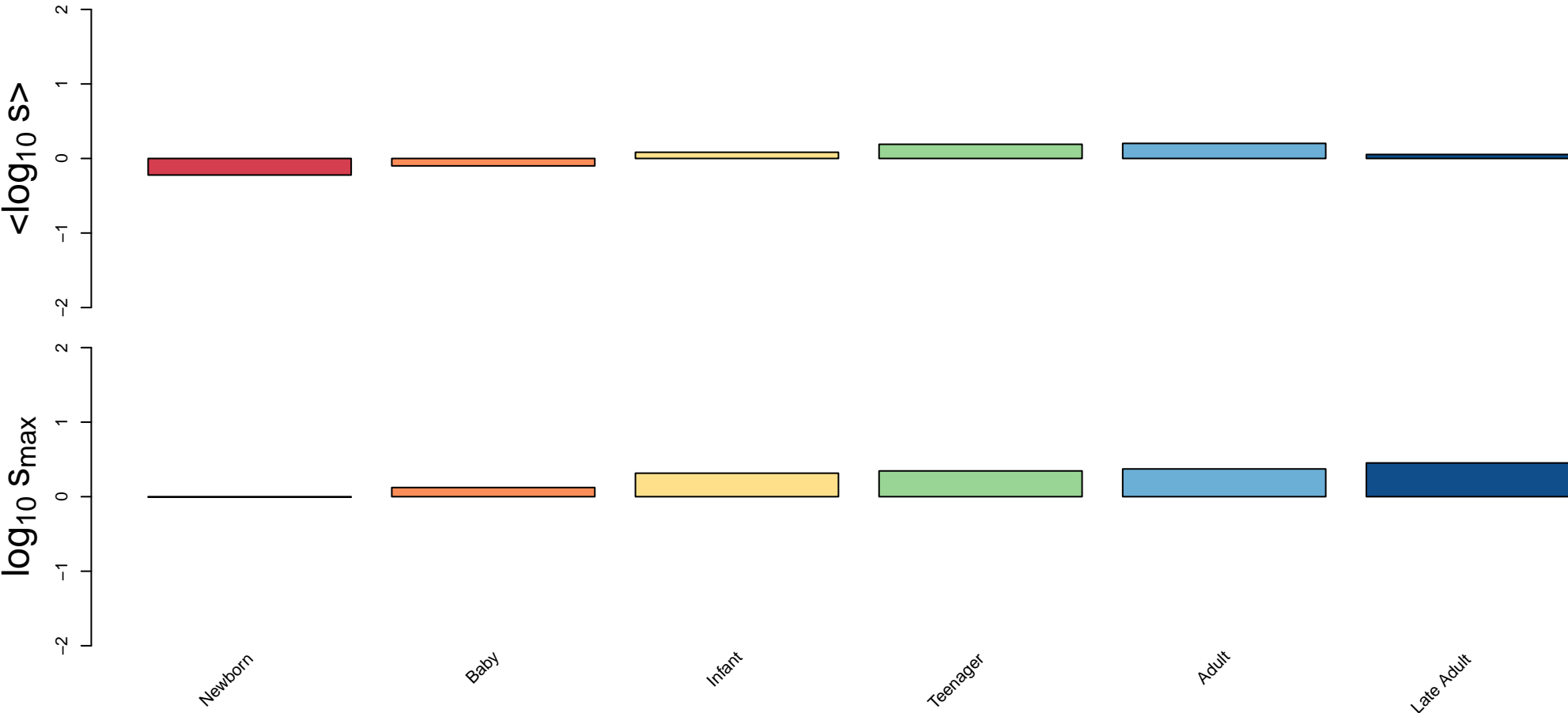

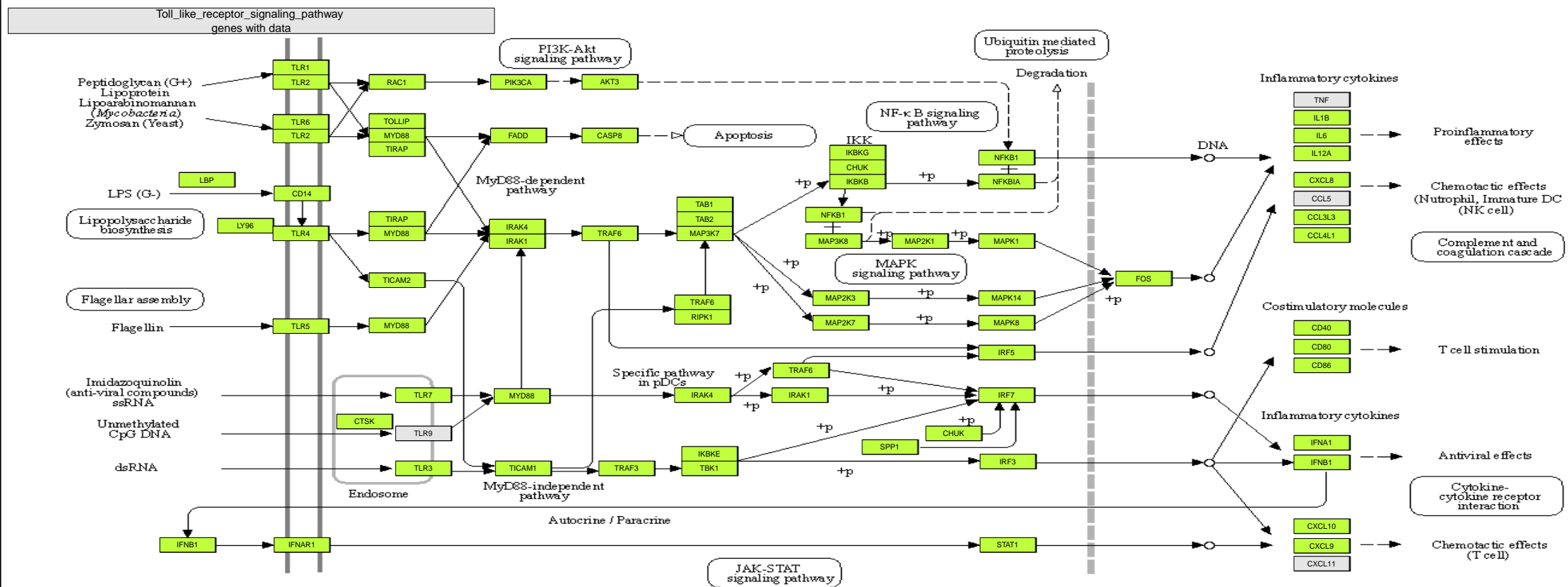

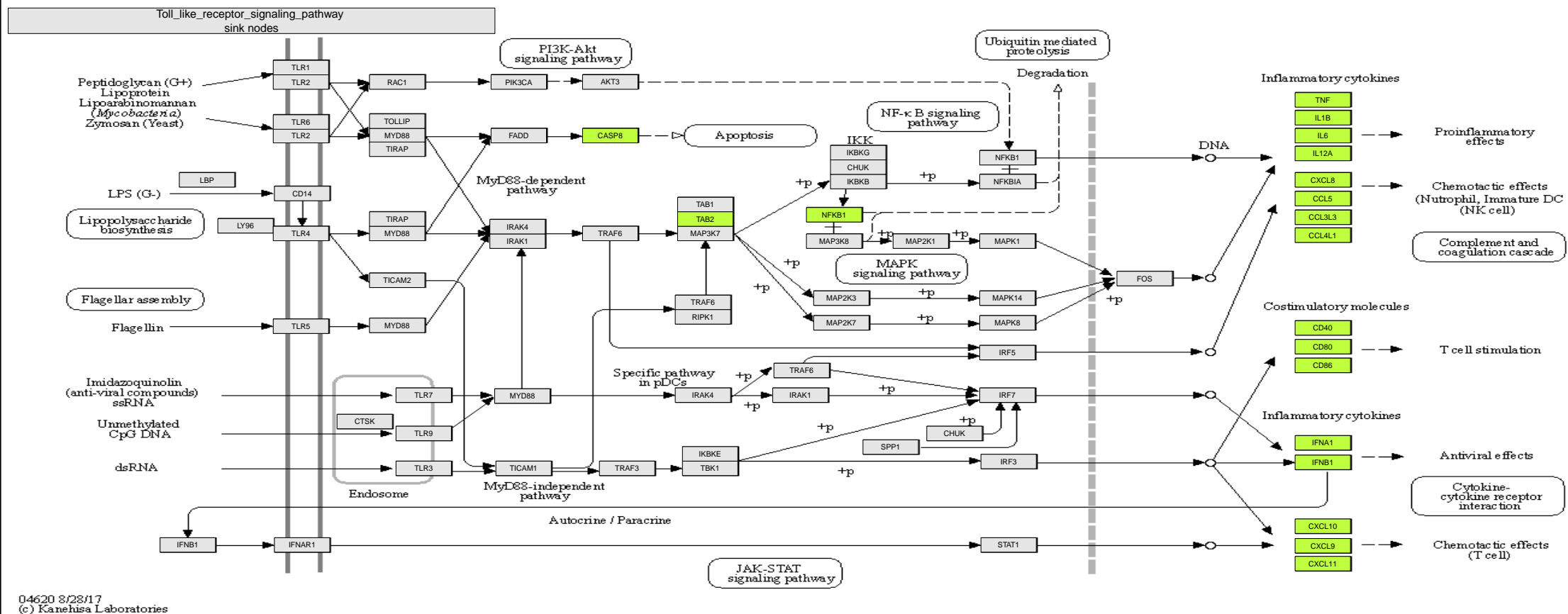

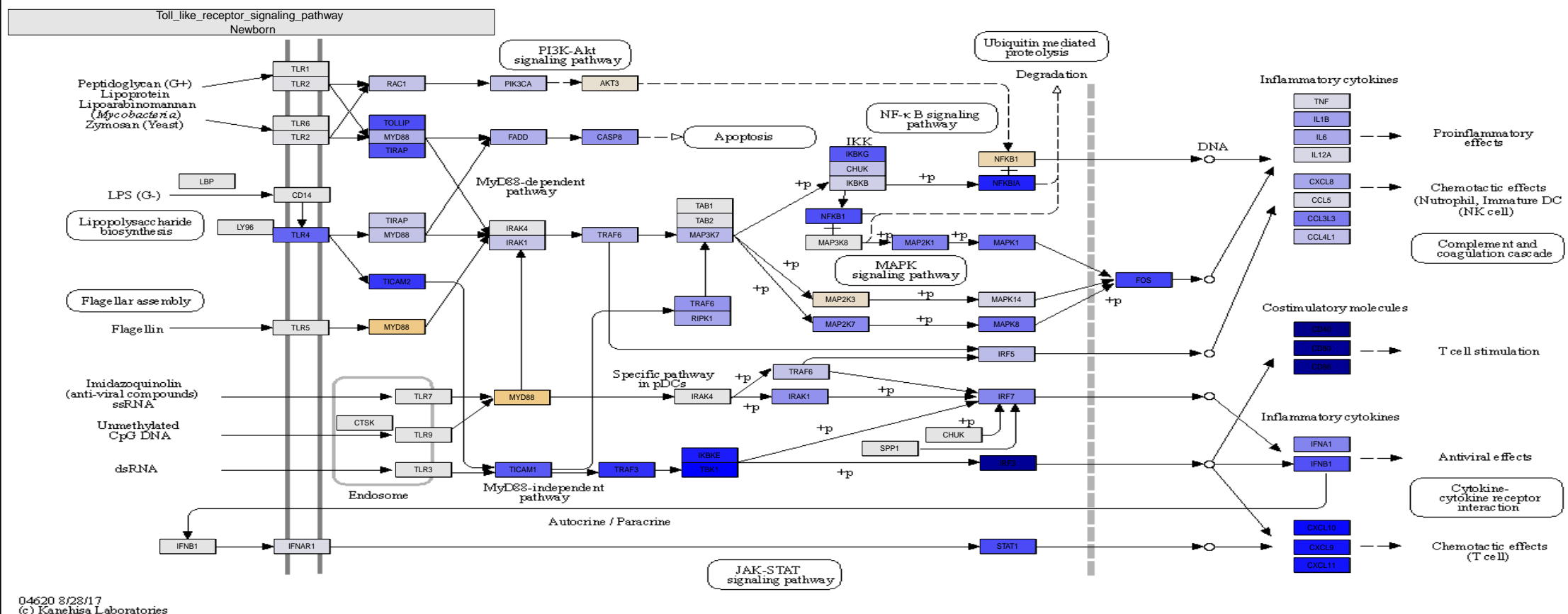

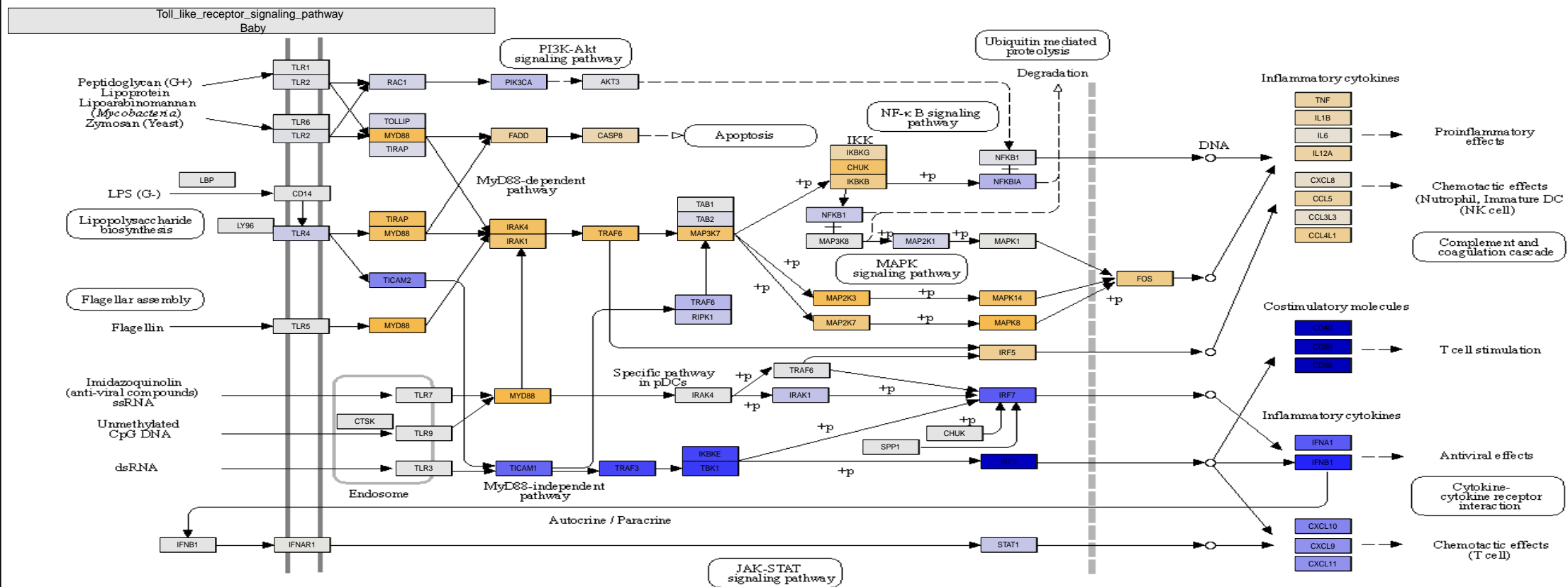

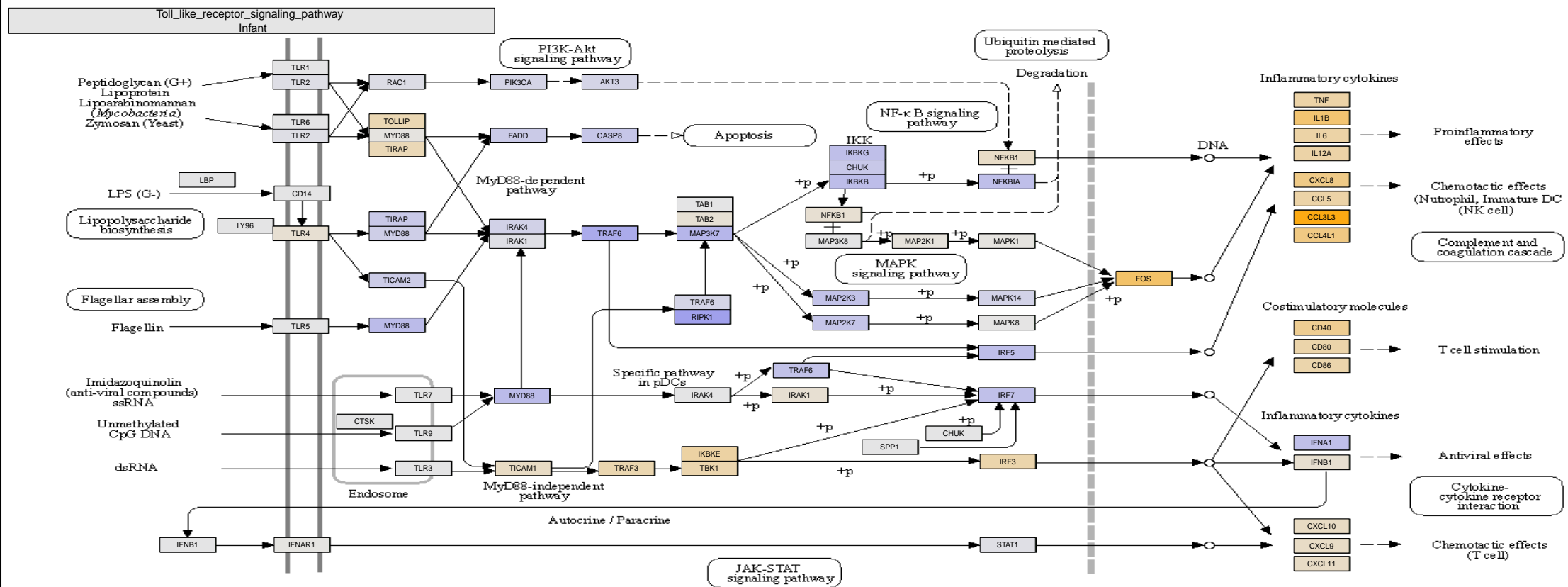

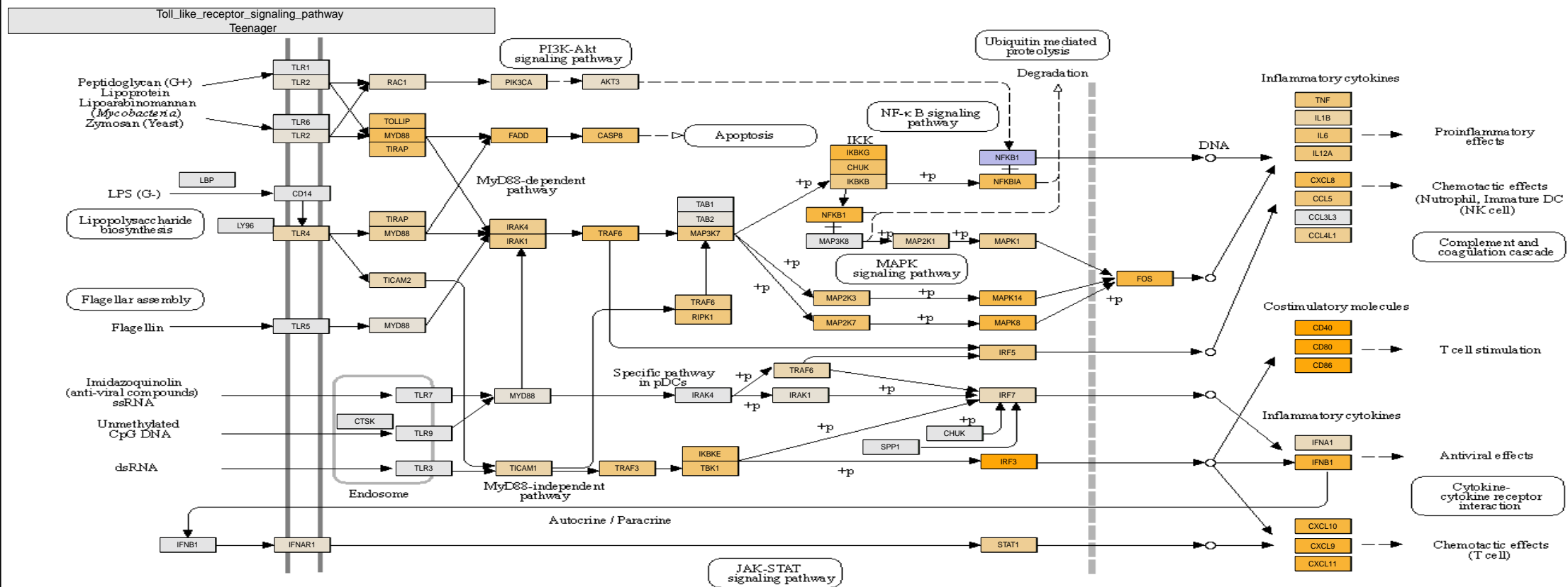

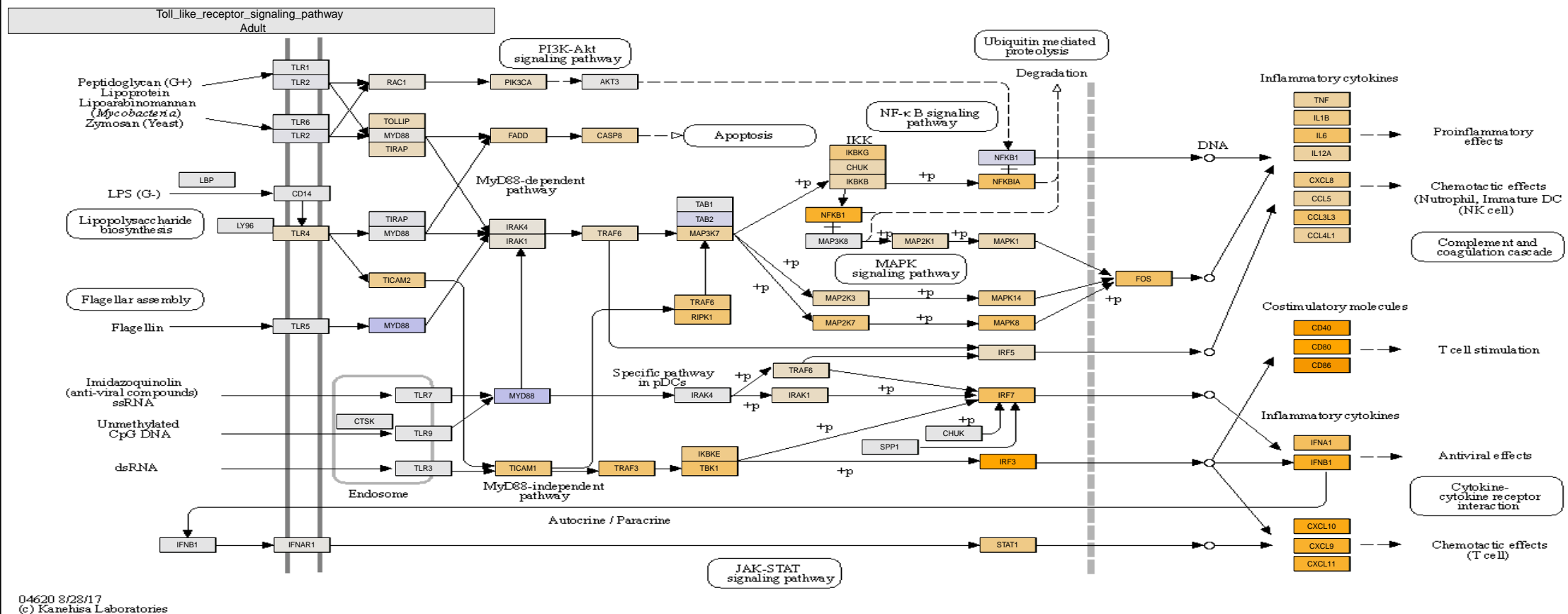

Supplement: Supplementary file 1 [file cells-11-00362-s001.zip › Suppl-Material-S3-Pathways-PSF_Expression/Toll_like_receptor_signaling_pathway.pdf]

Wnt signaling pathway

all genes

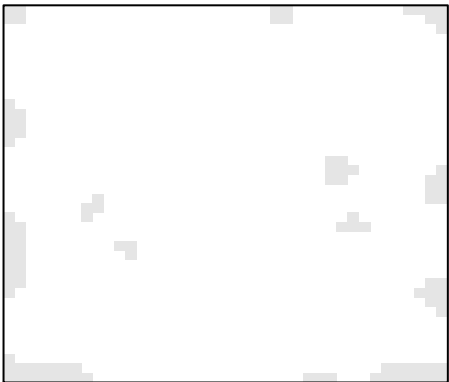

maximum = -Inf

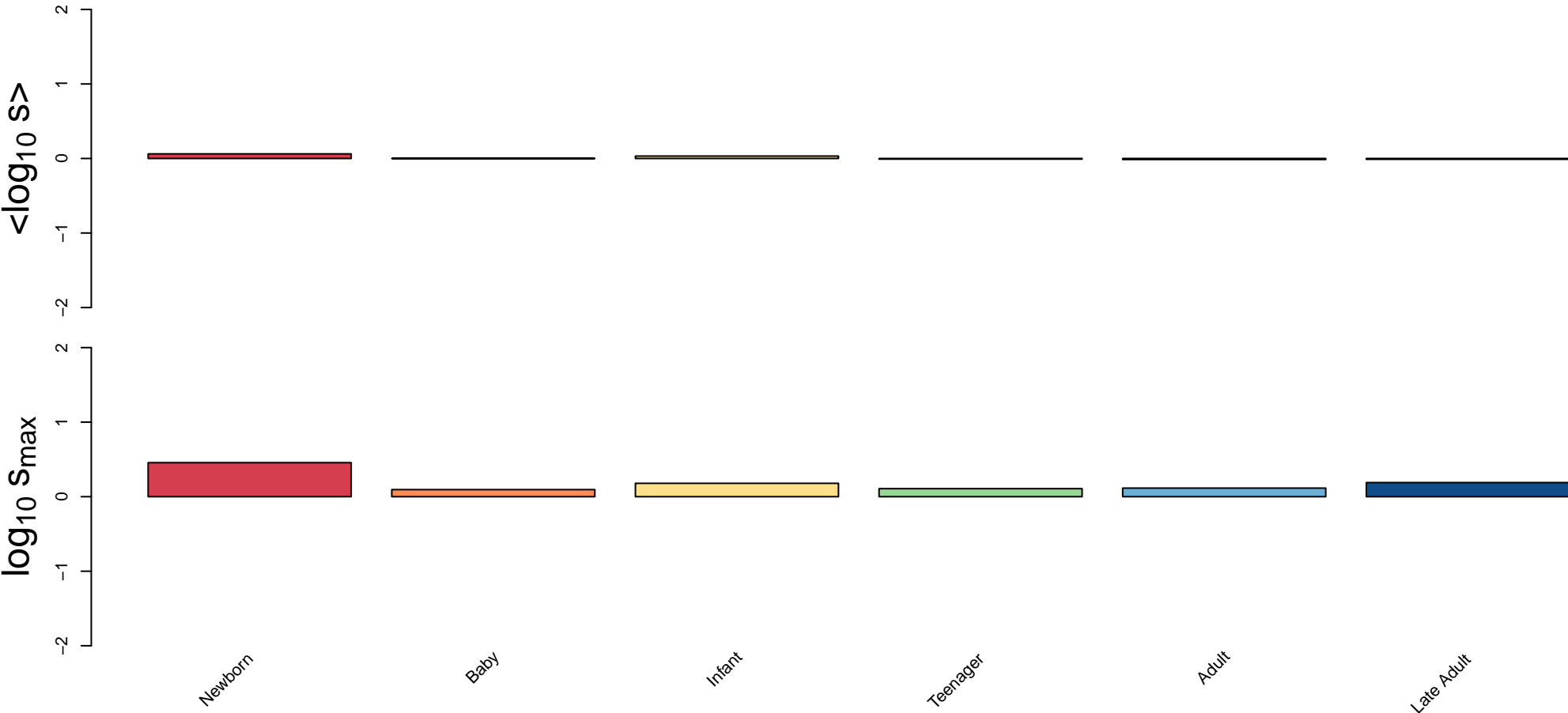

Wnt\_signaling\_pathway  
genes with data

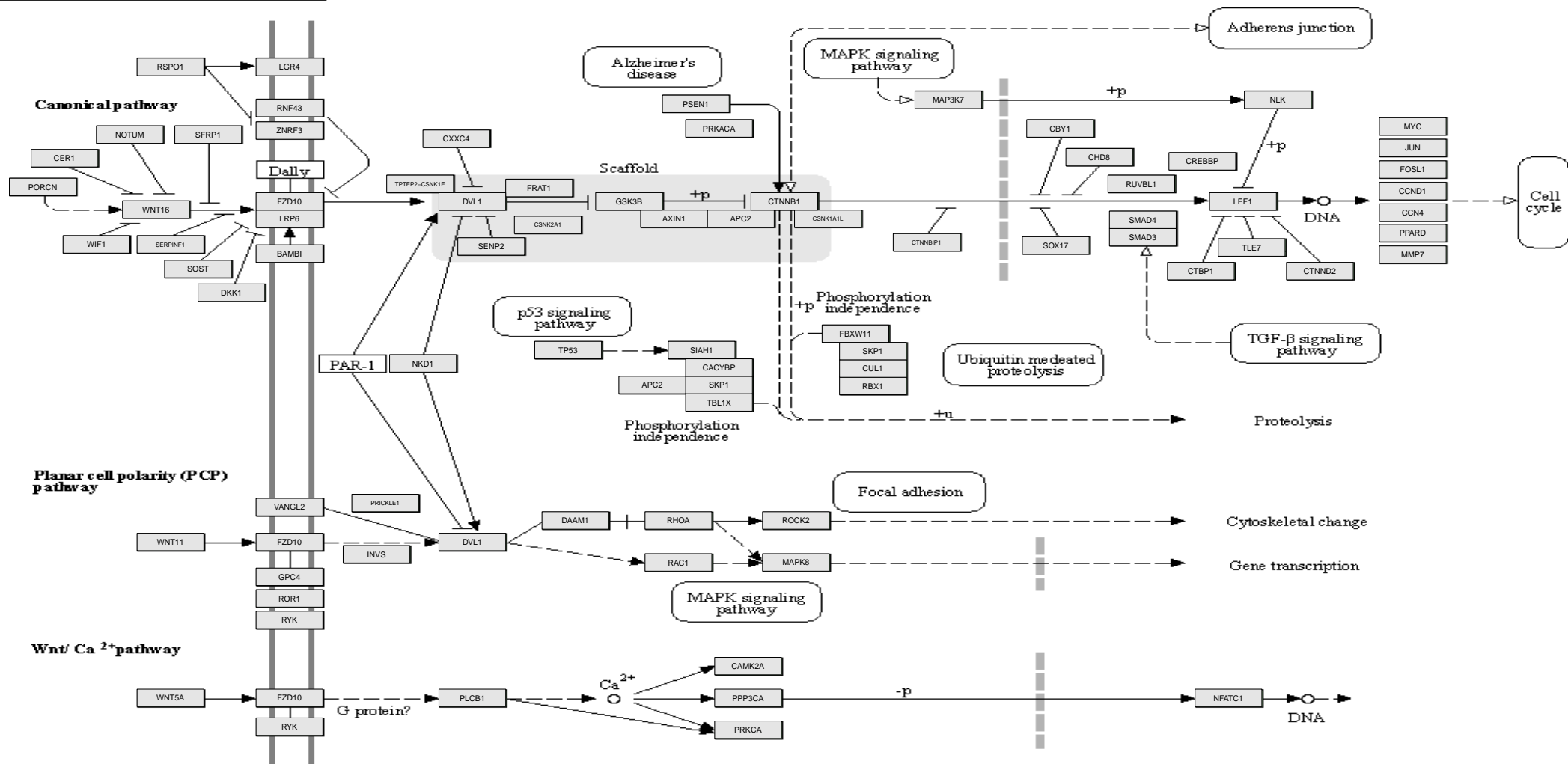

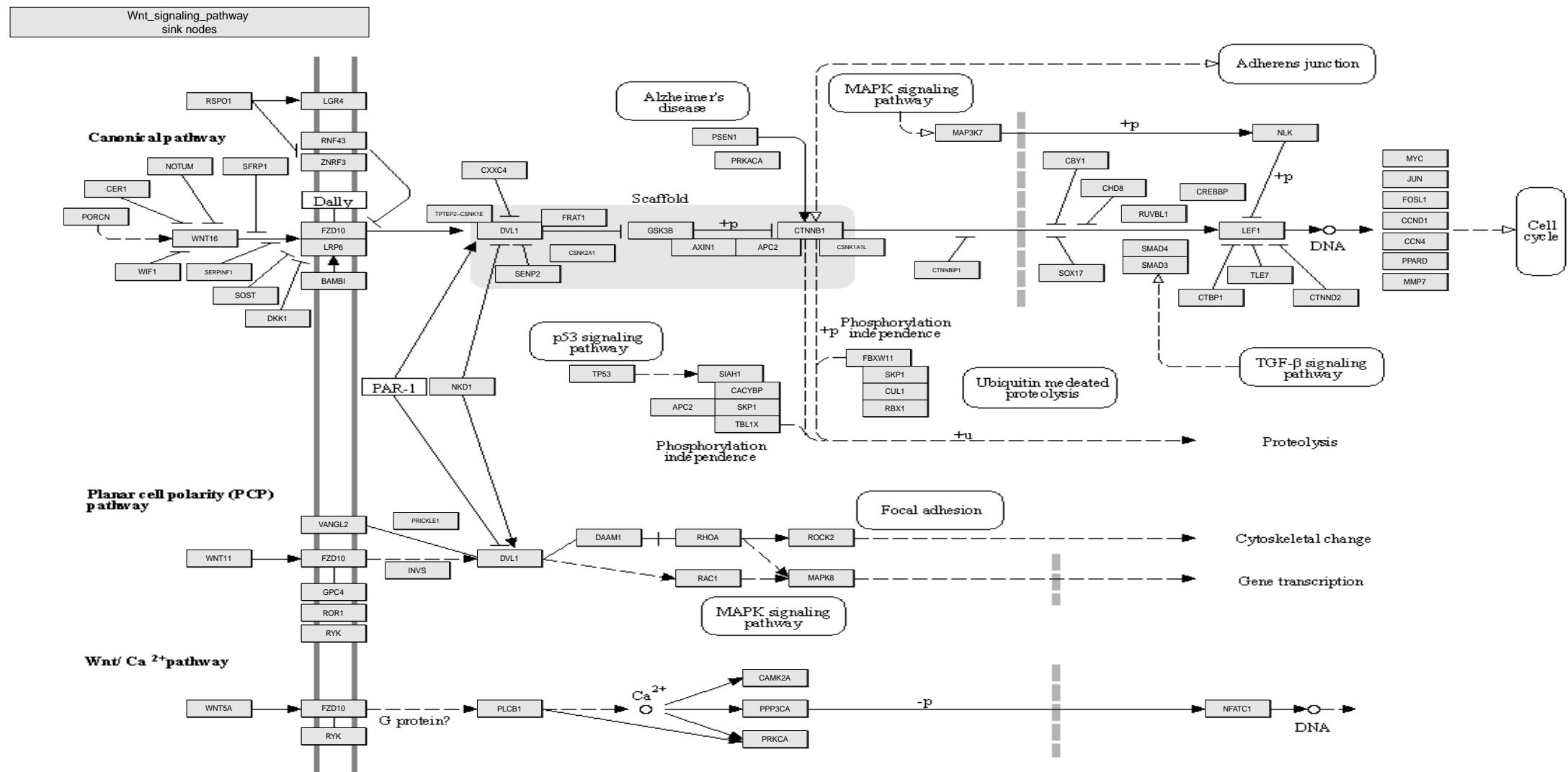

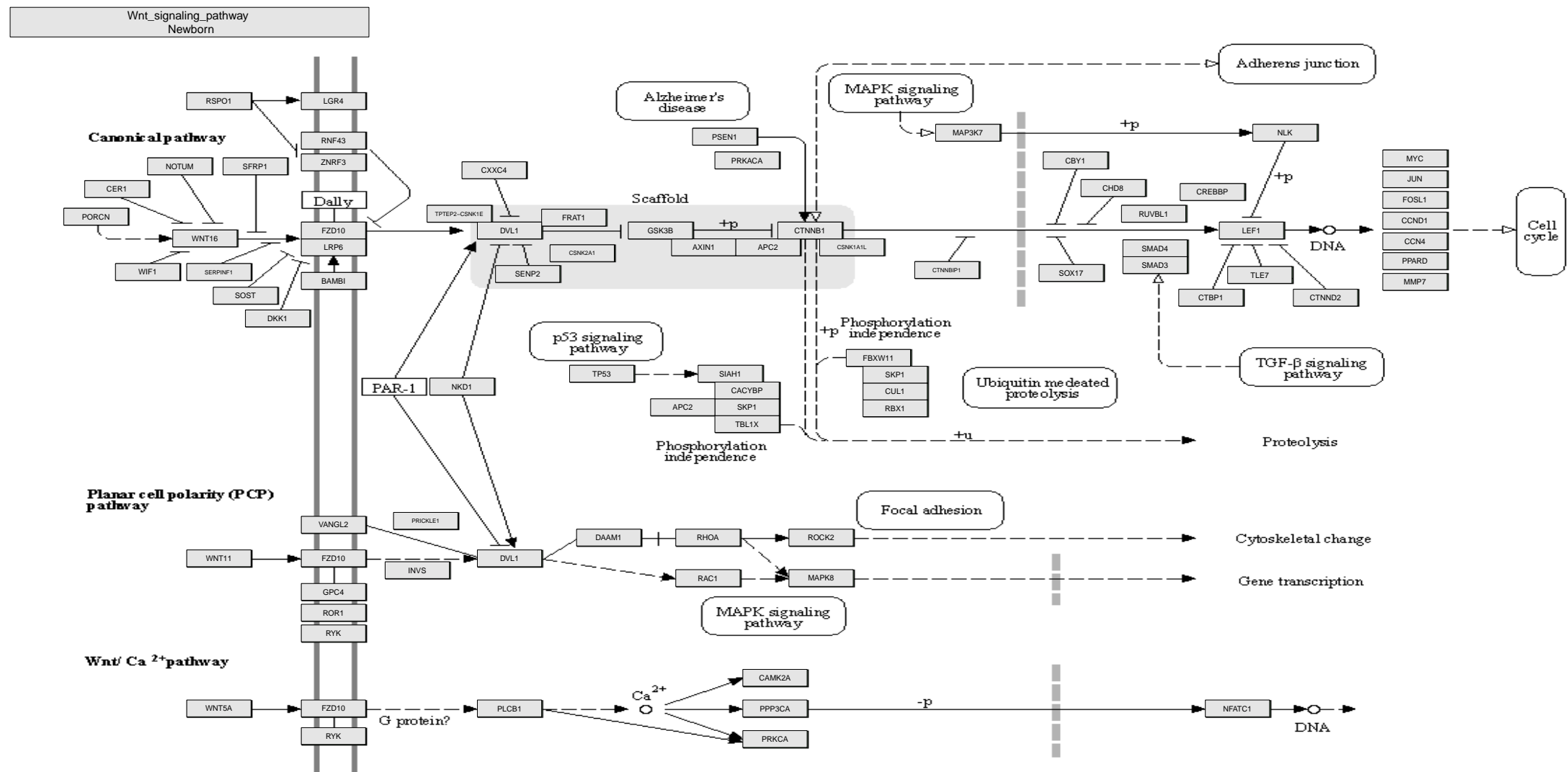

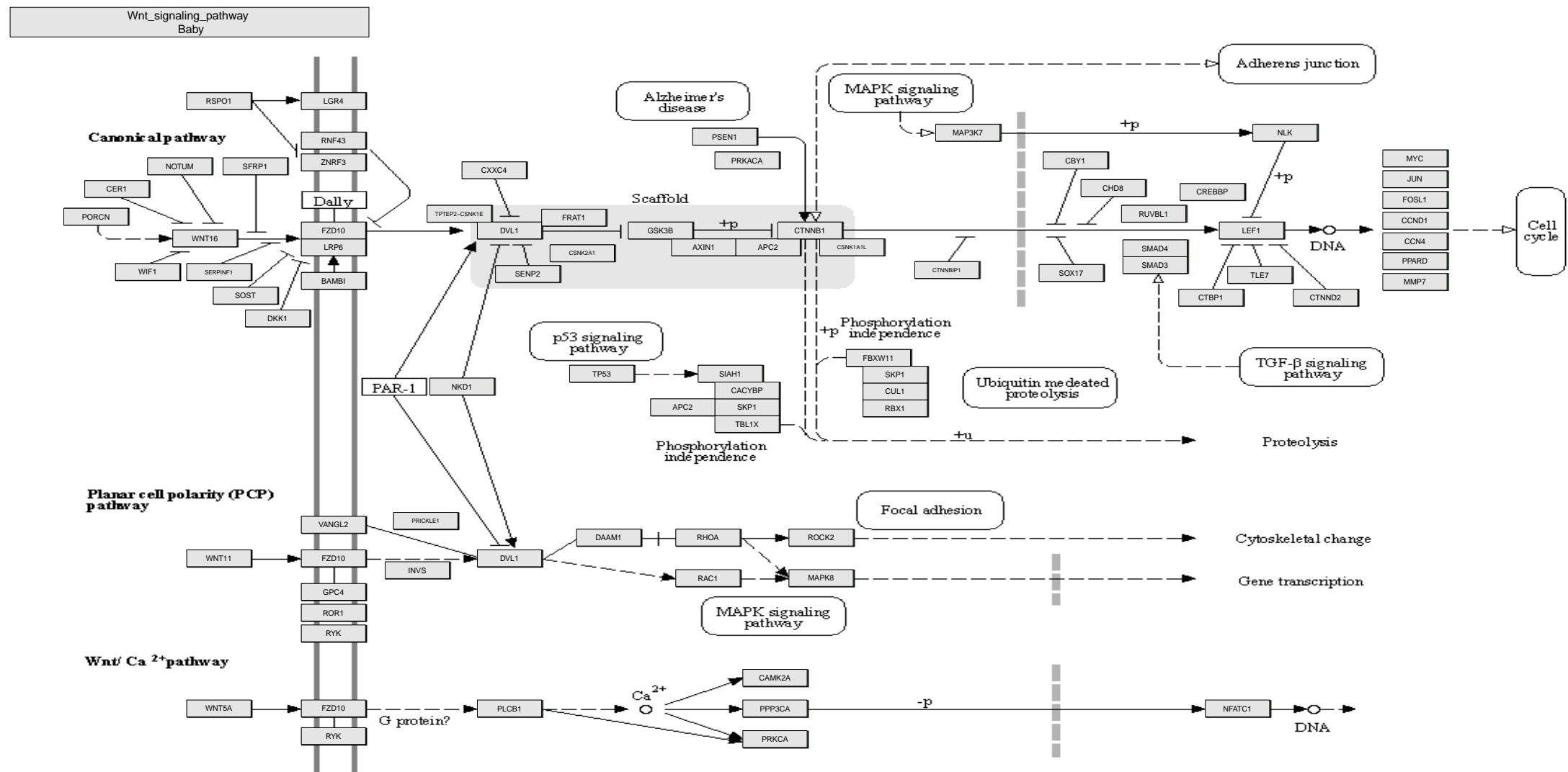

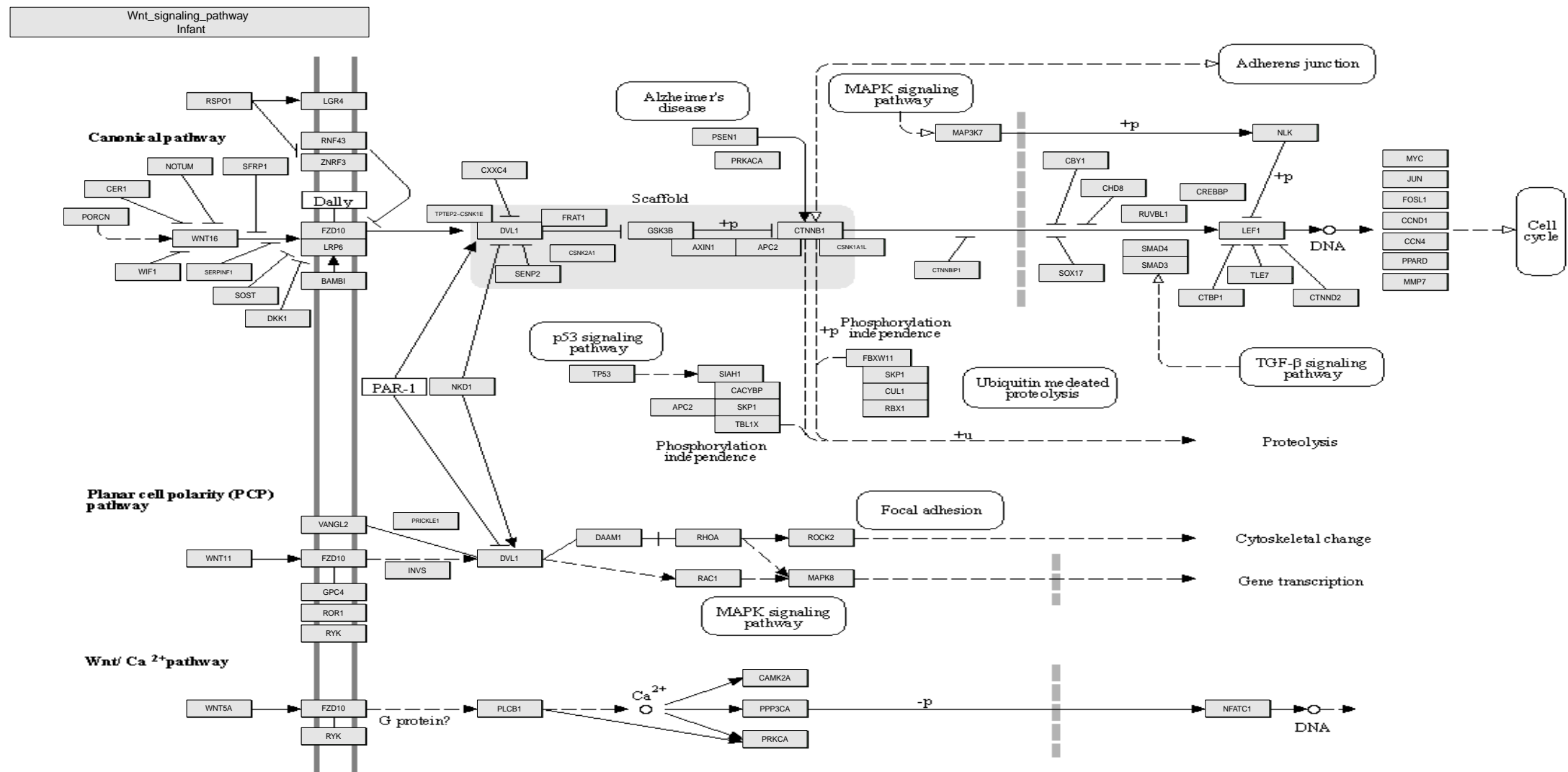

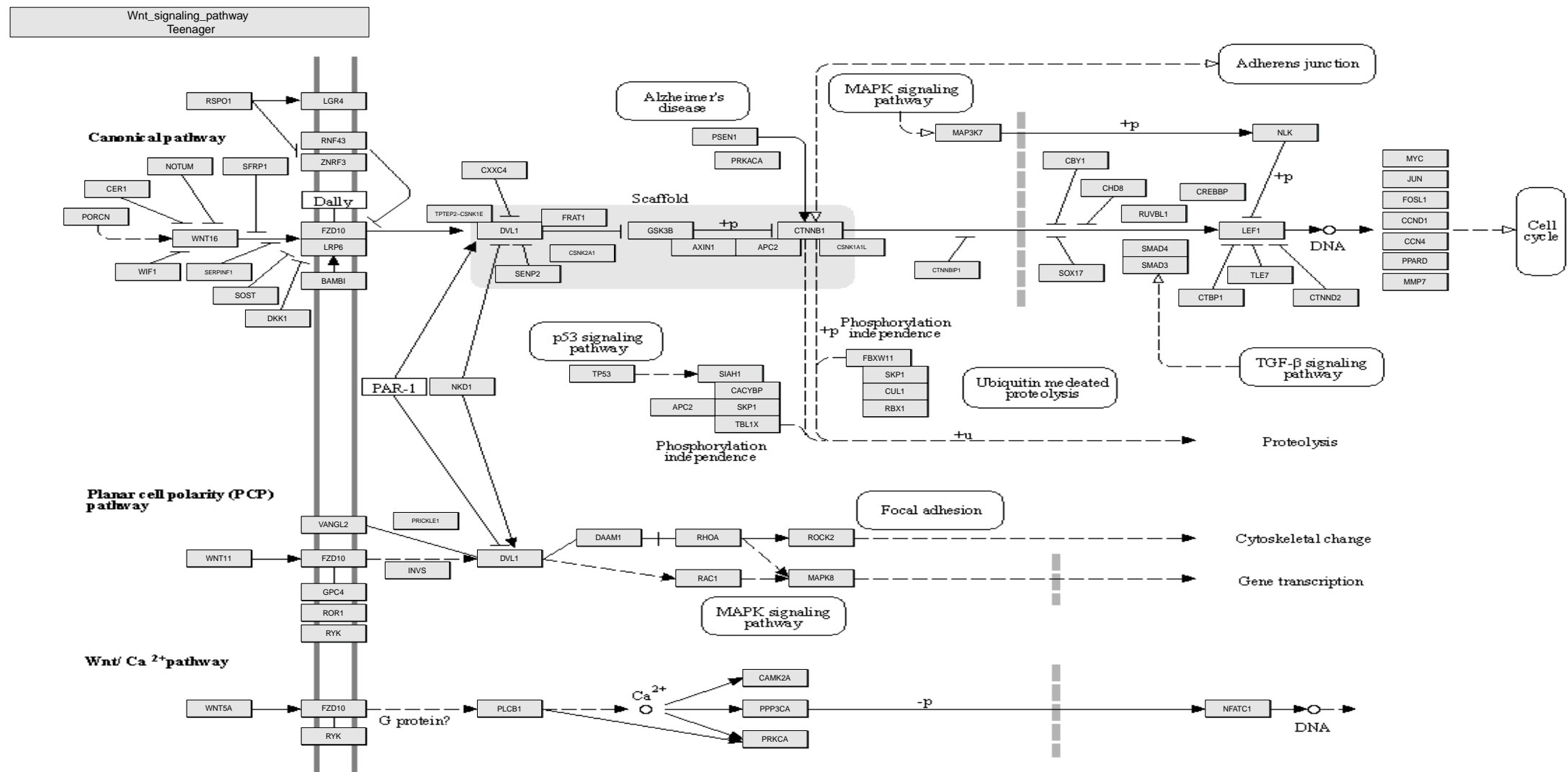

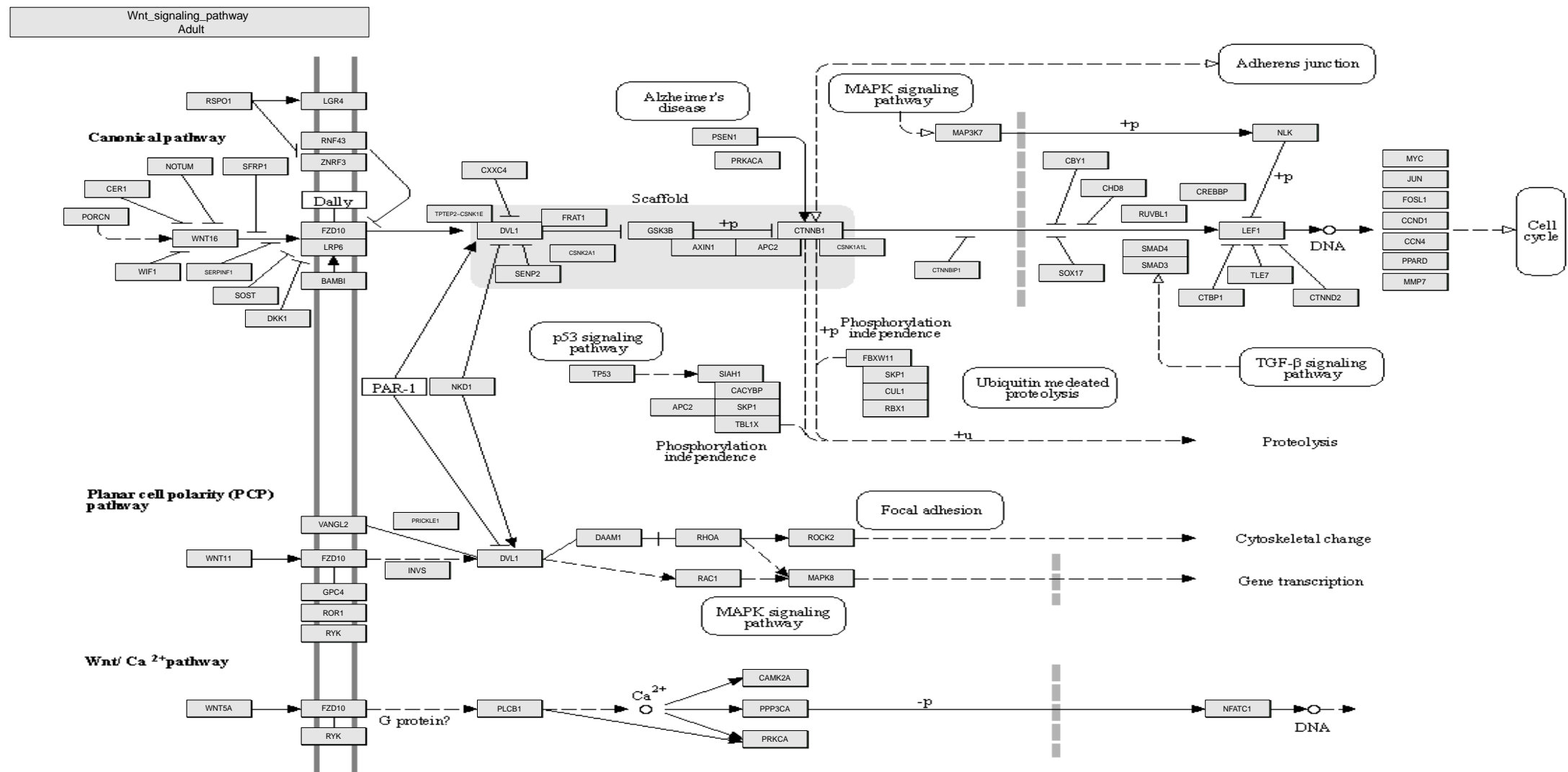

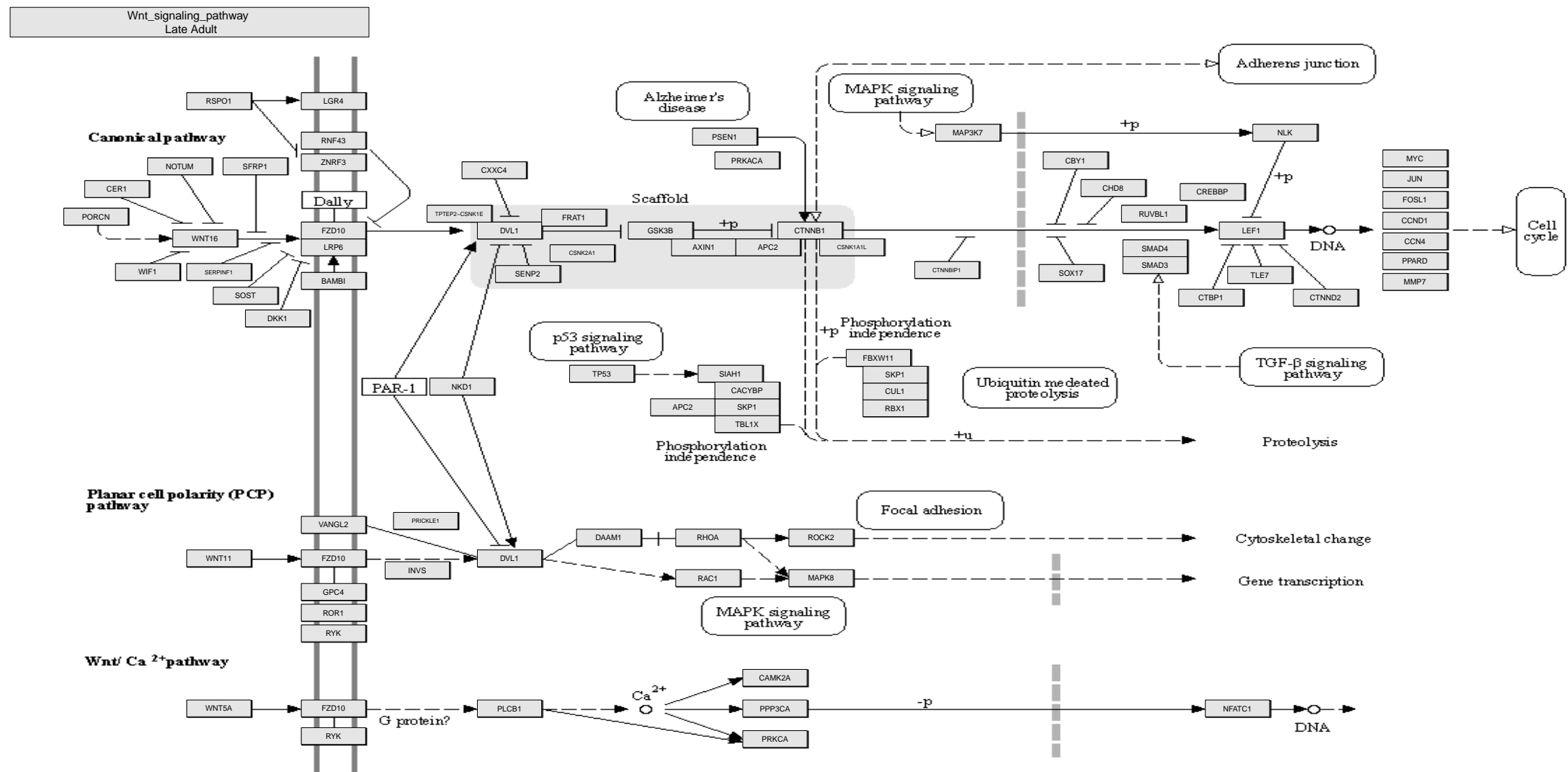

Supplement: Supplementary file 1 [file cells-11-00362-s001.zip › Suppl-Material-S3-Pathways-PSF_Expression/Wnt_signaling_pathway.pdf]

mean signal

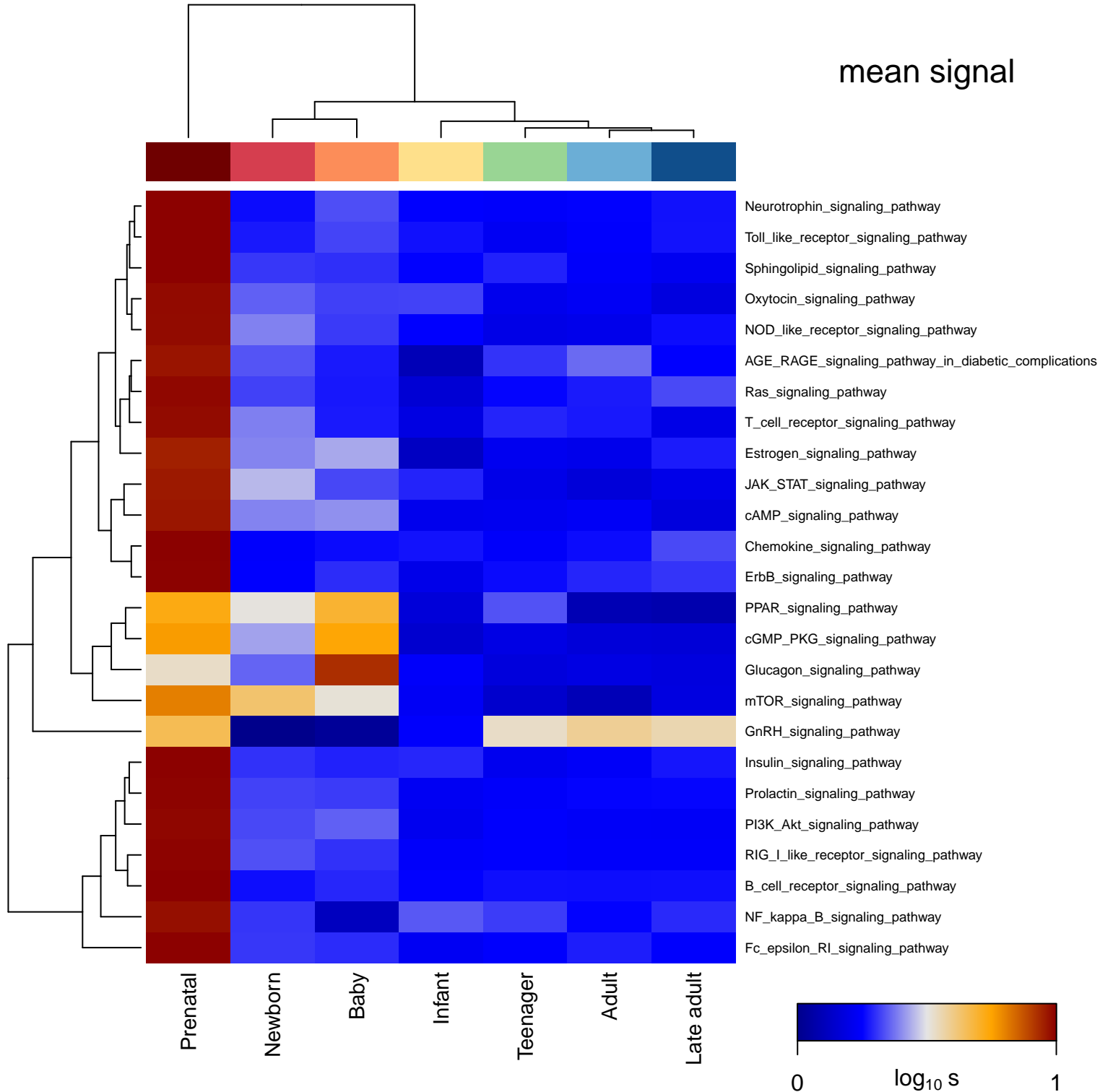

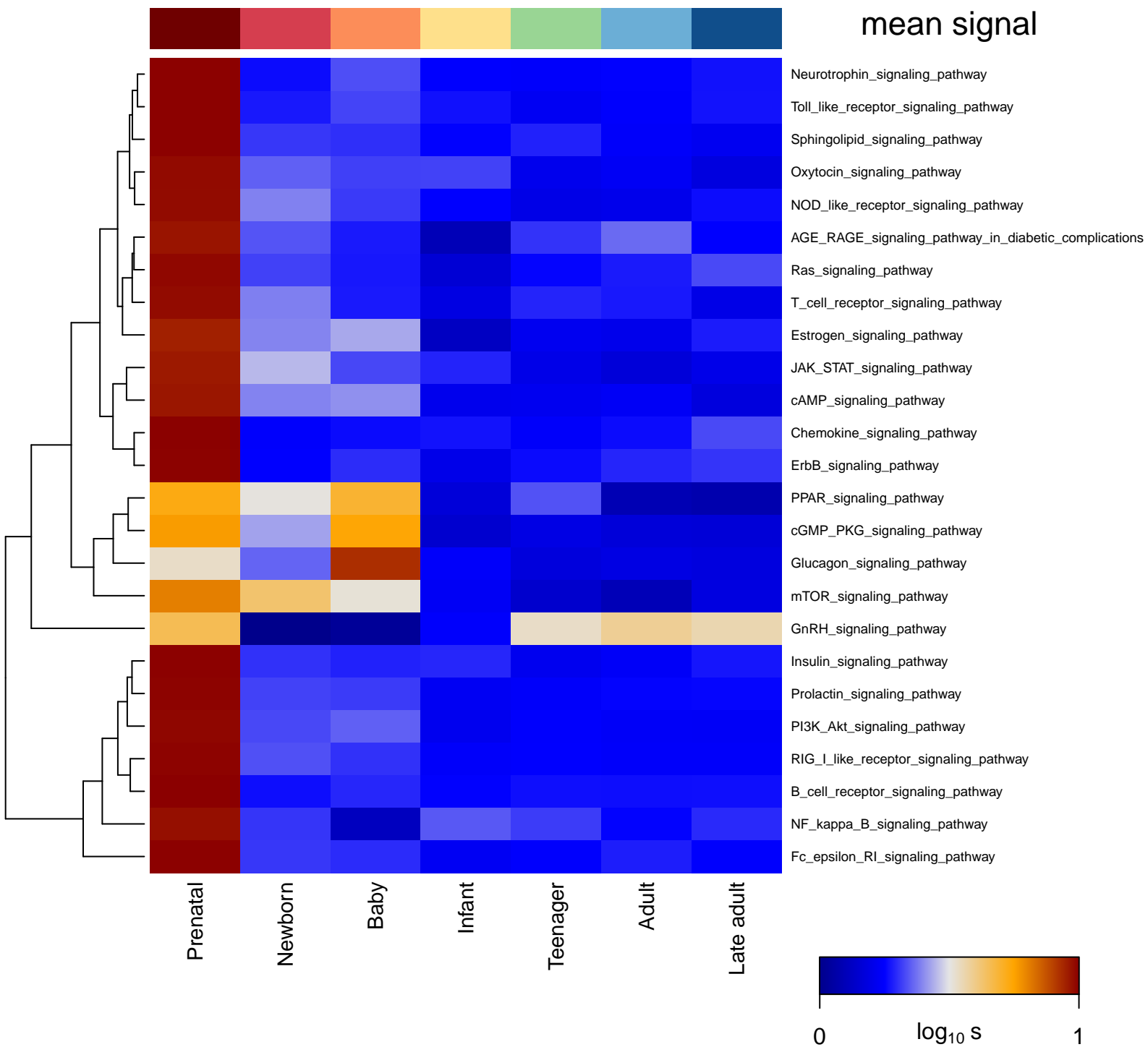

signal maximum

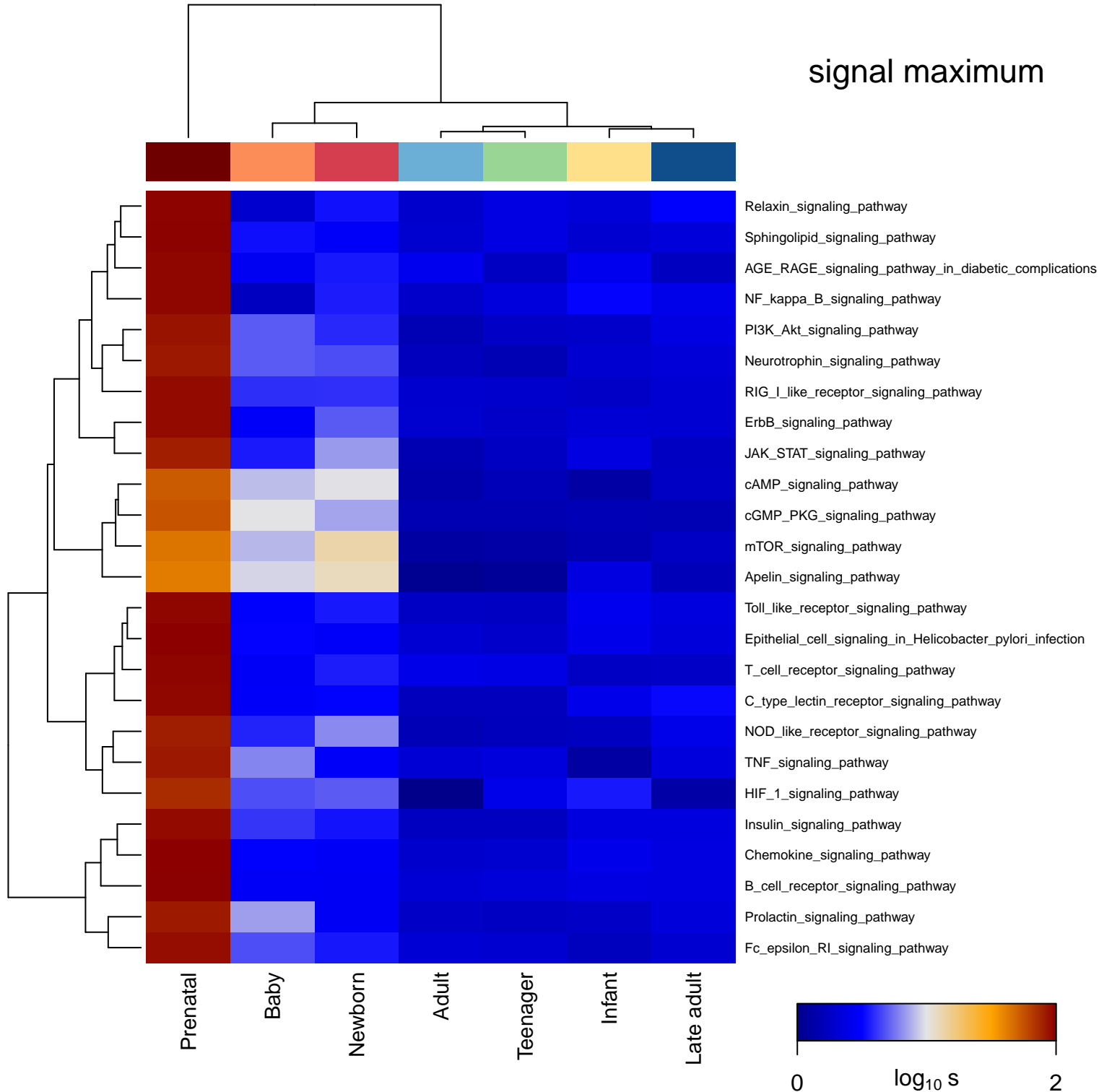

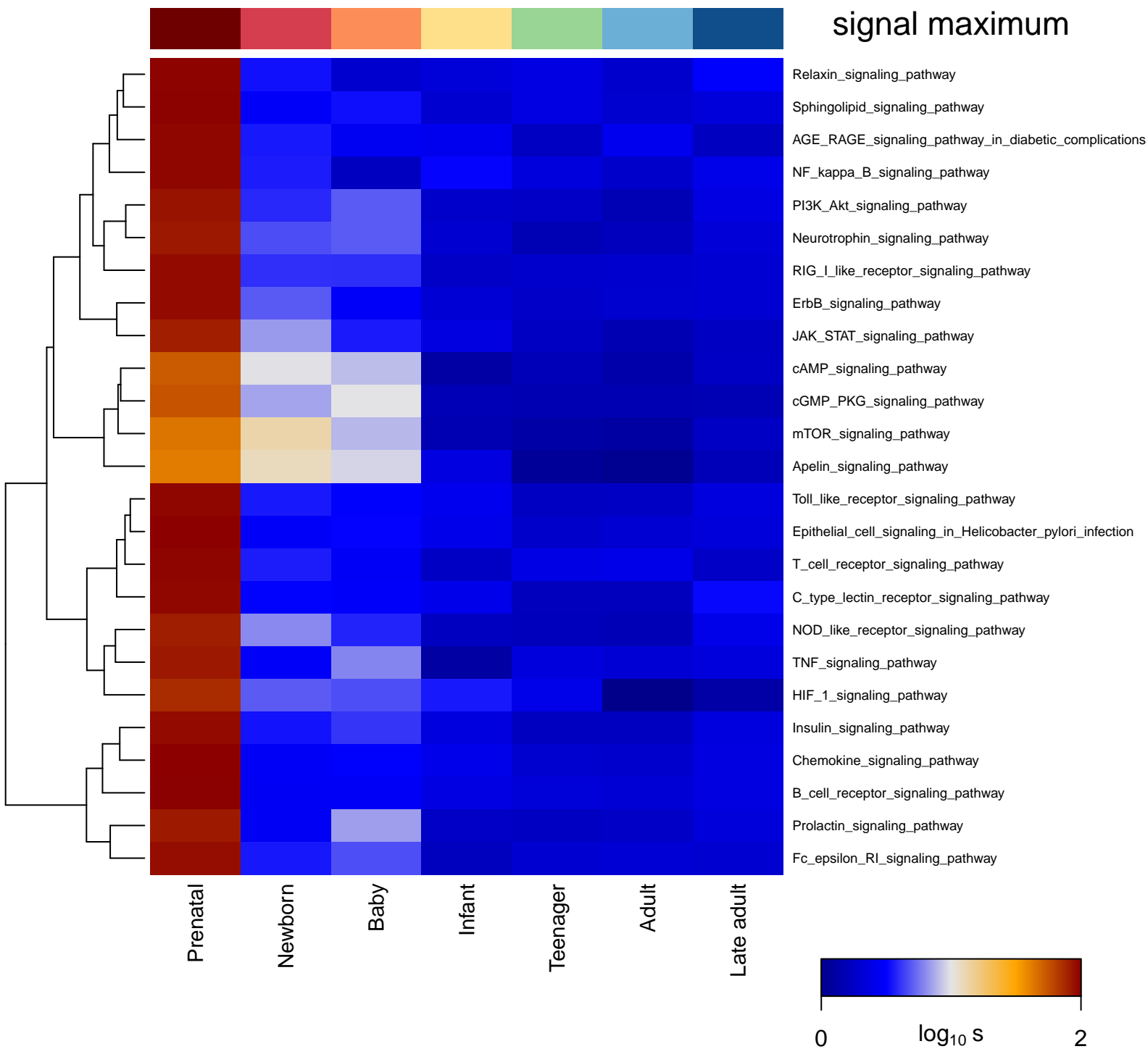

Supplement: Supplementary file 1 [file cells-11-00362-s001.zip › Suppl-Material-S4-Pathways-PSF_Methylation/0verview Heatmaps.pdf]

Epithelial cell signaling in Helicobact

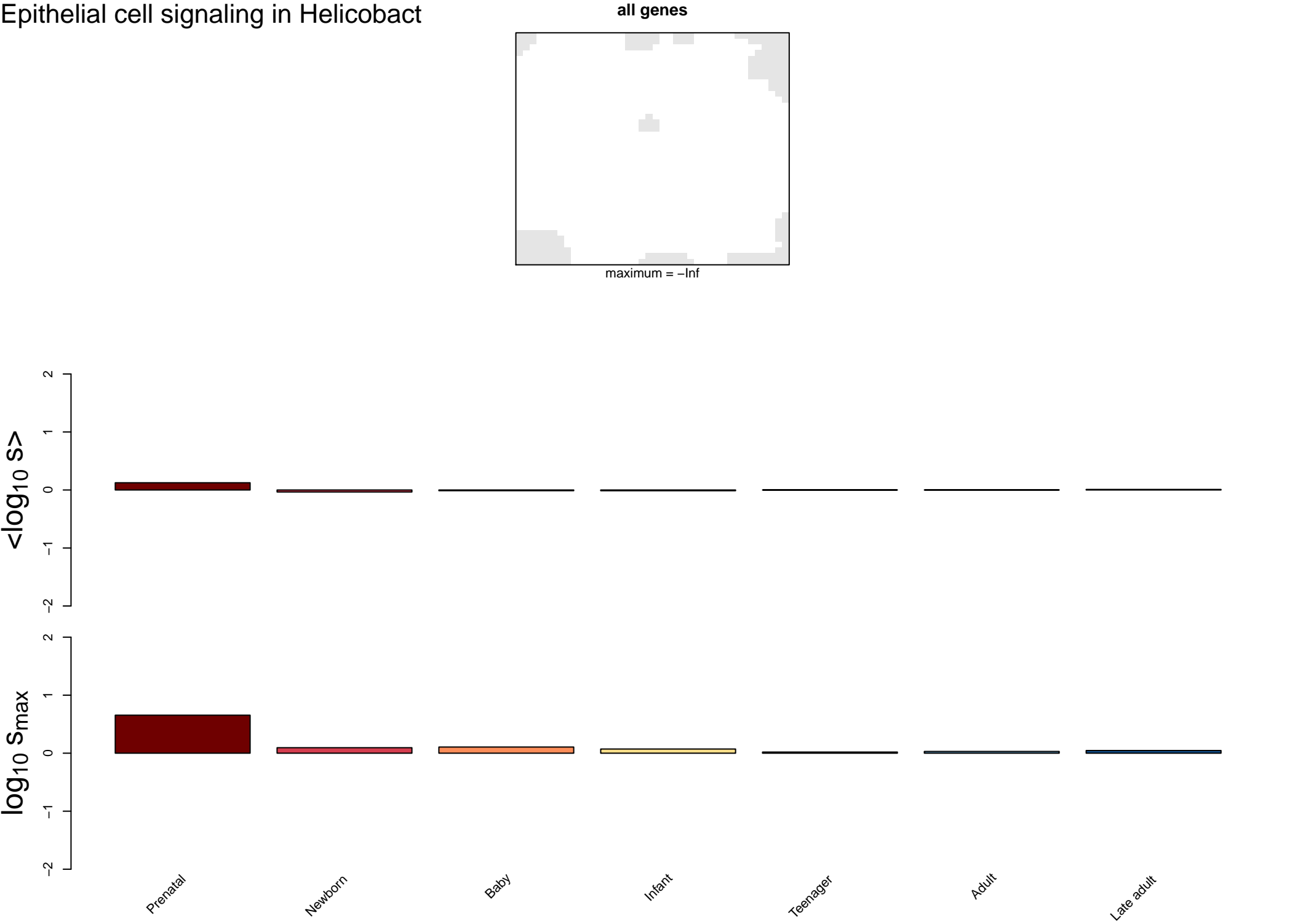

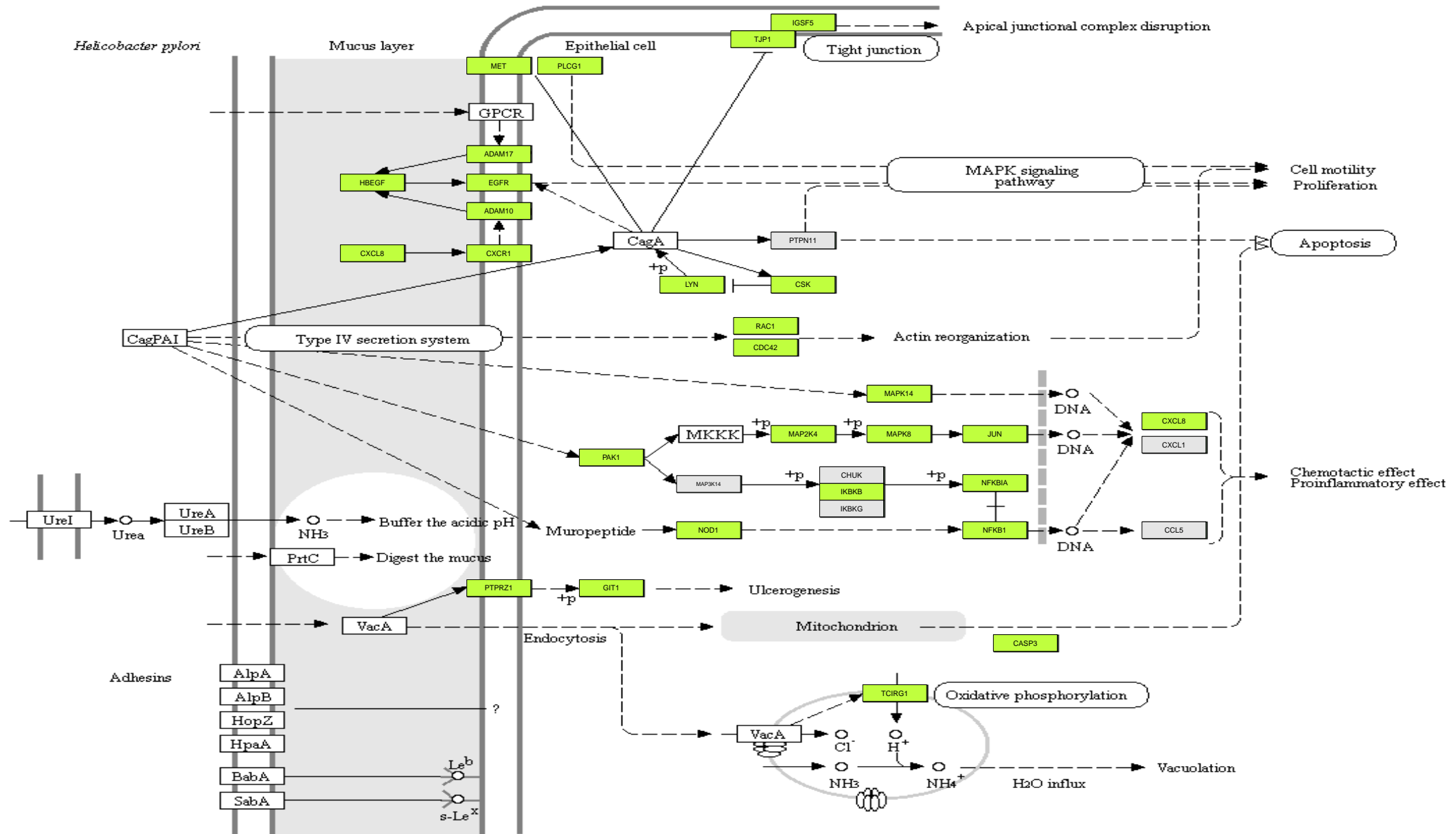

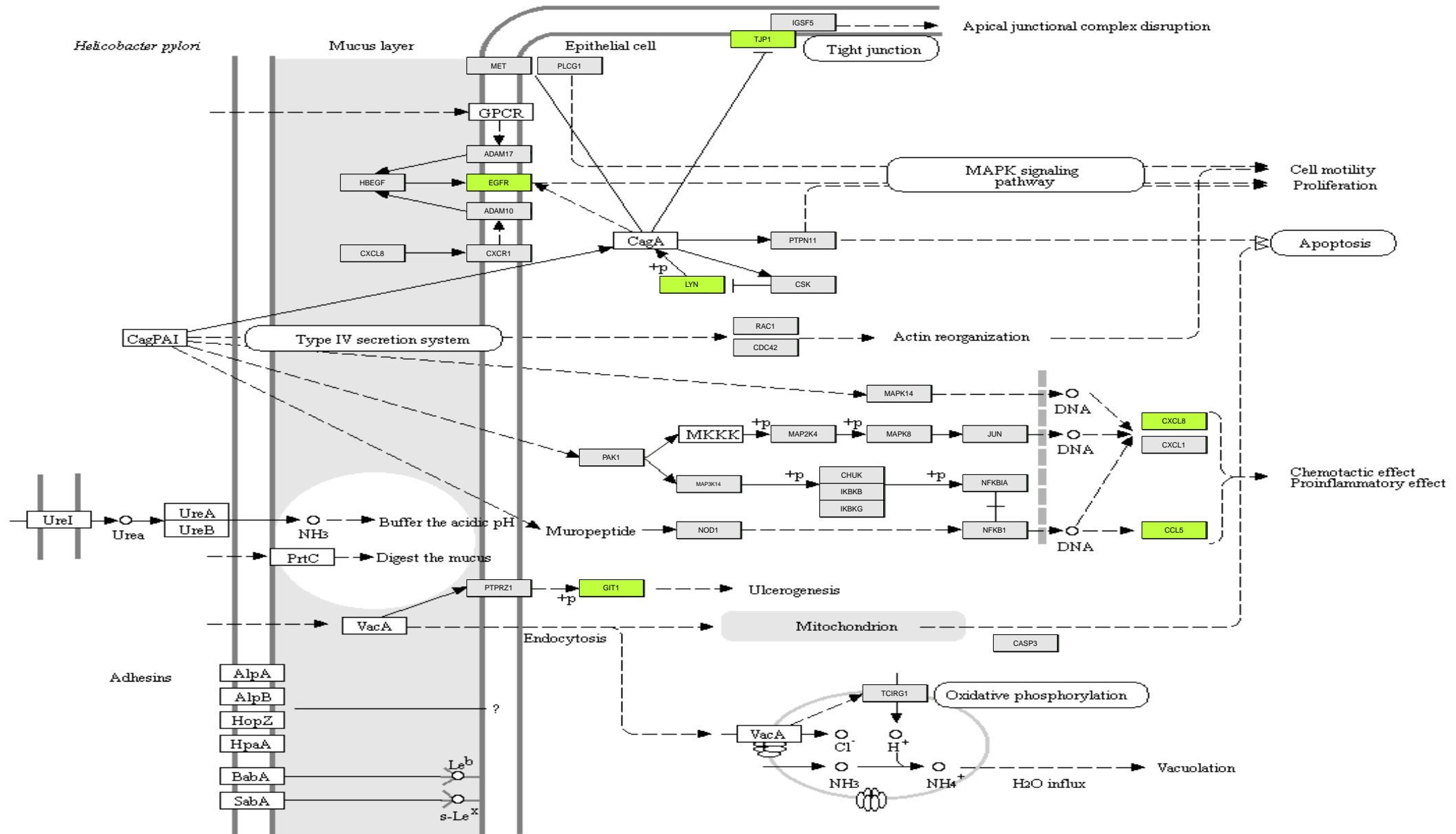

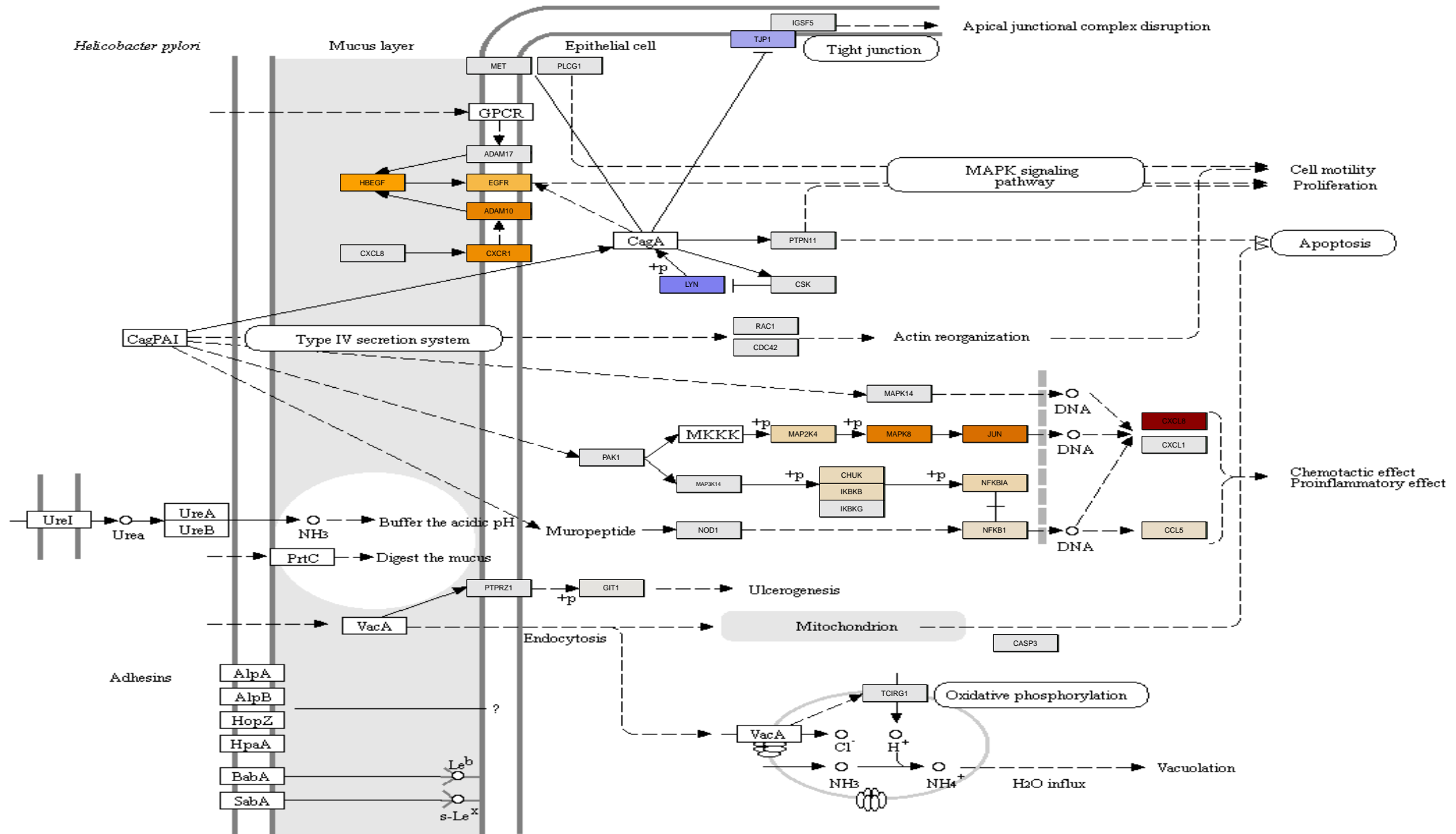

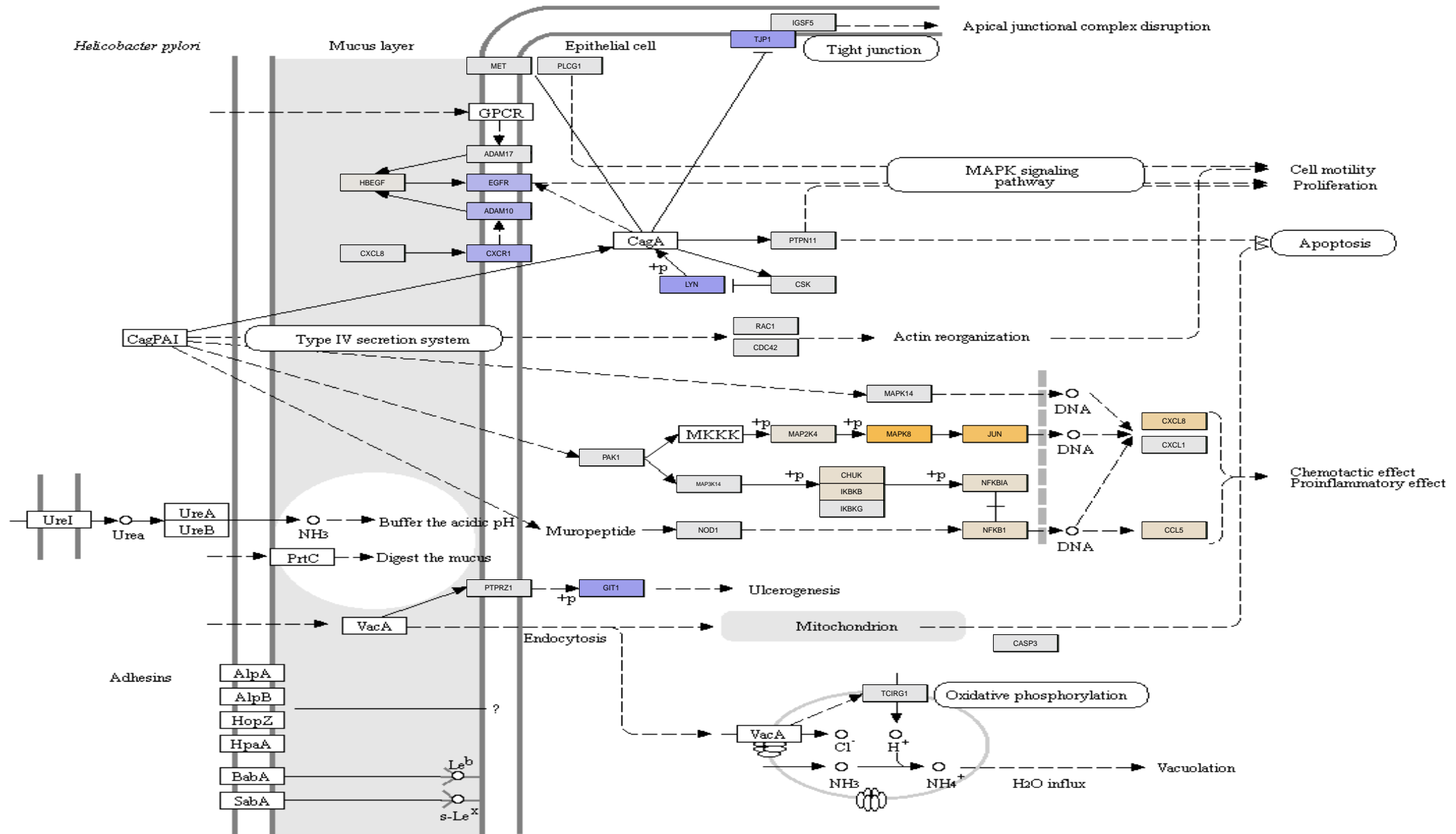

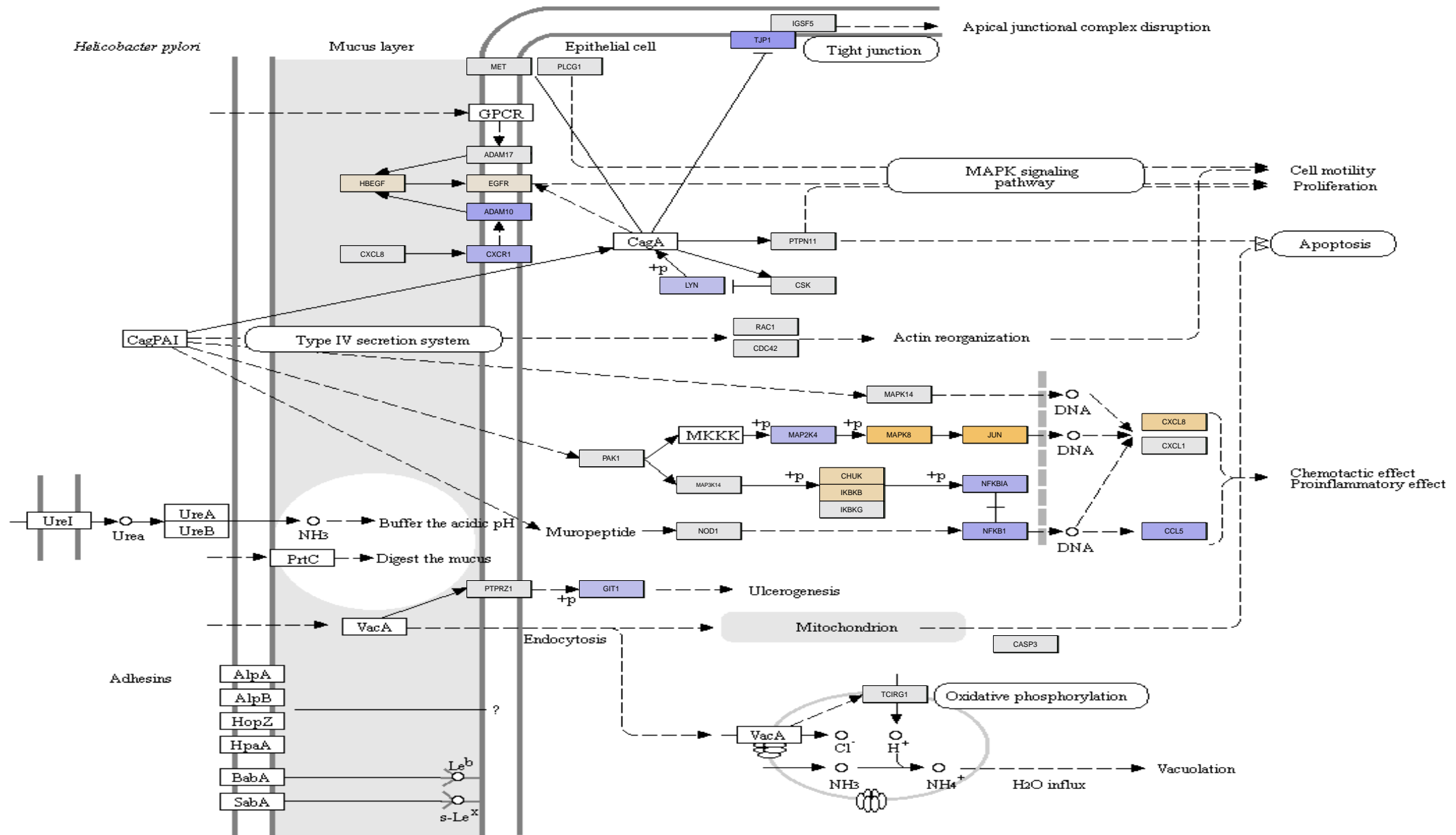

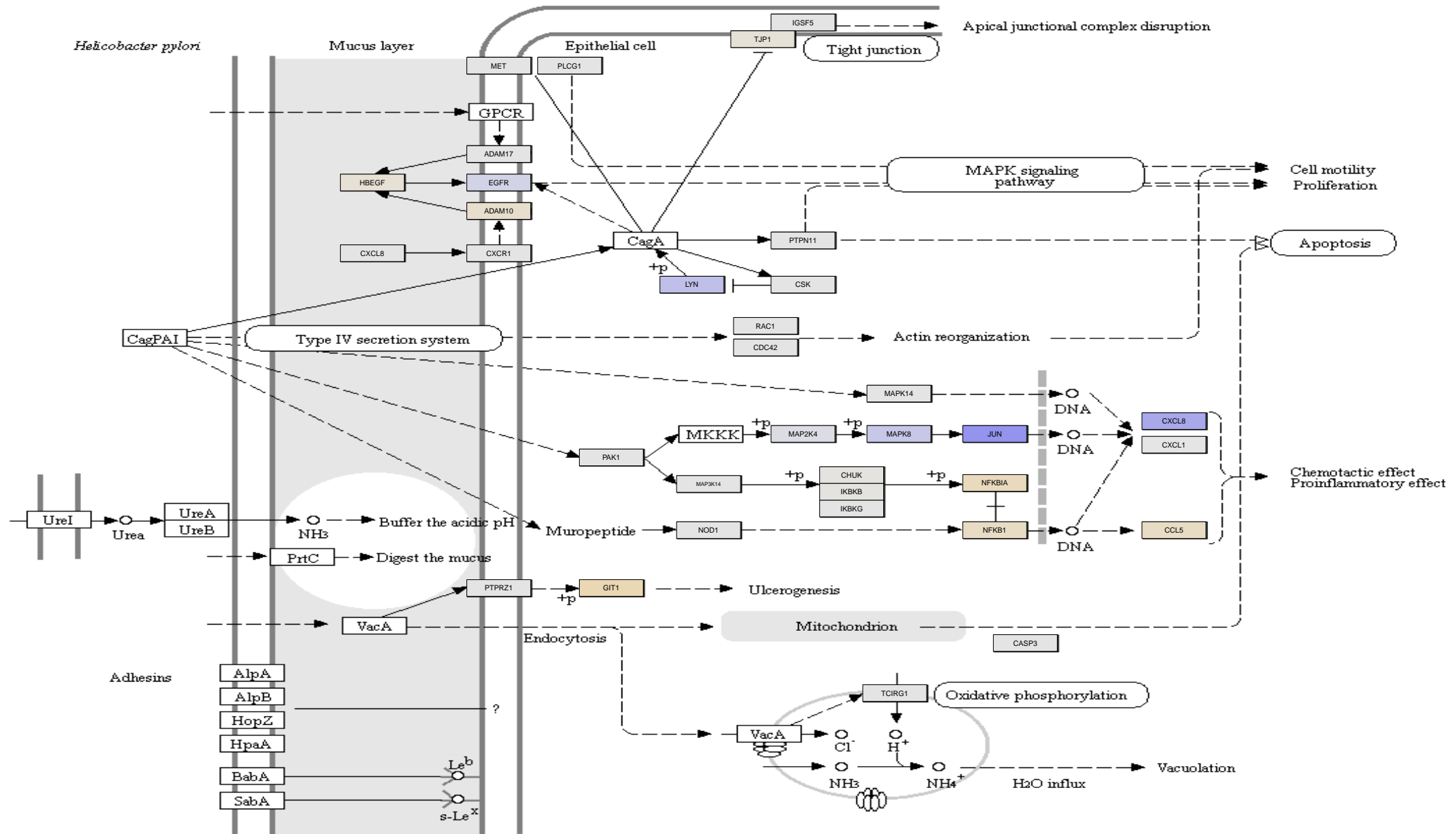

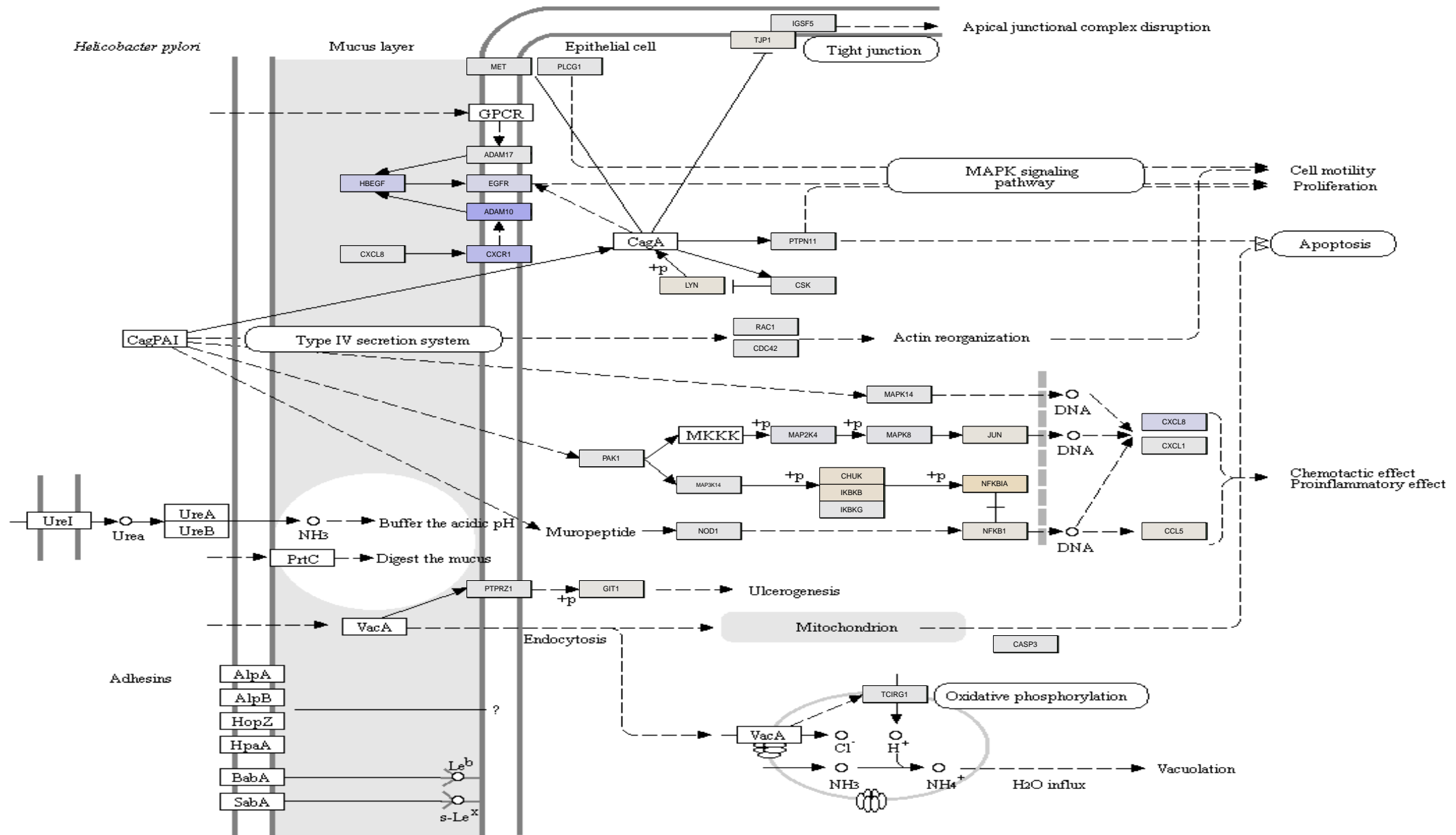

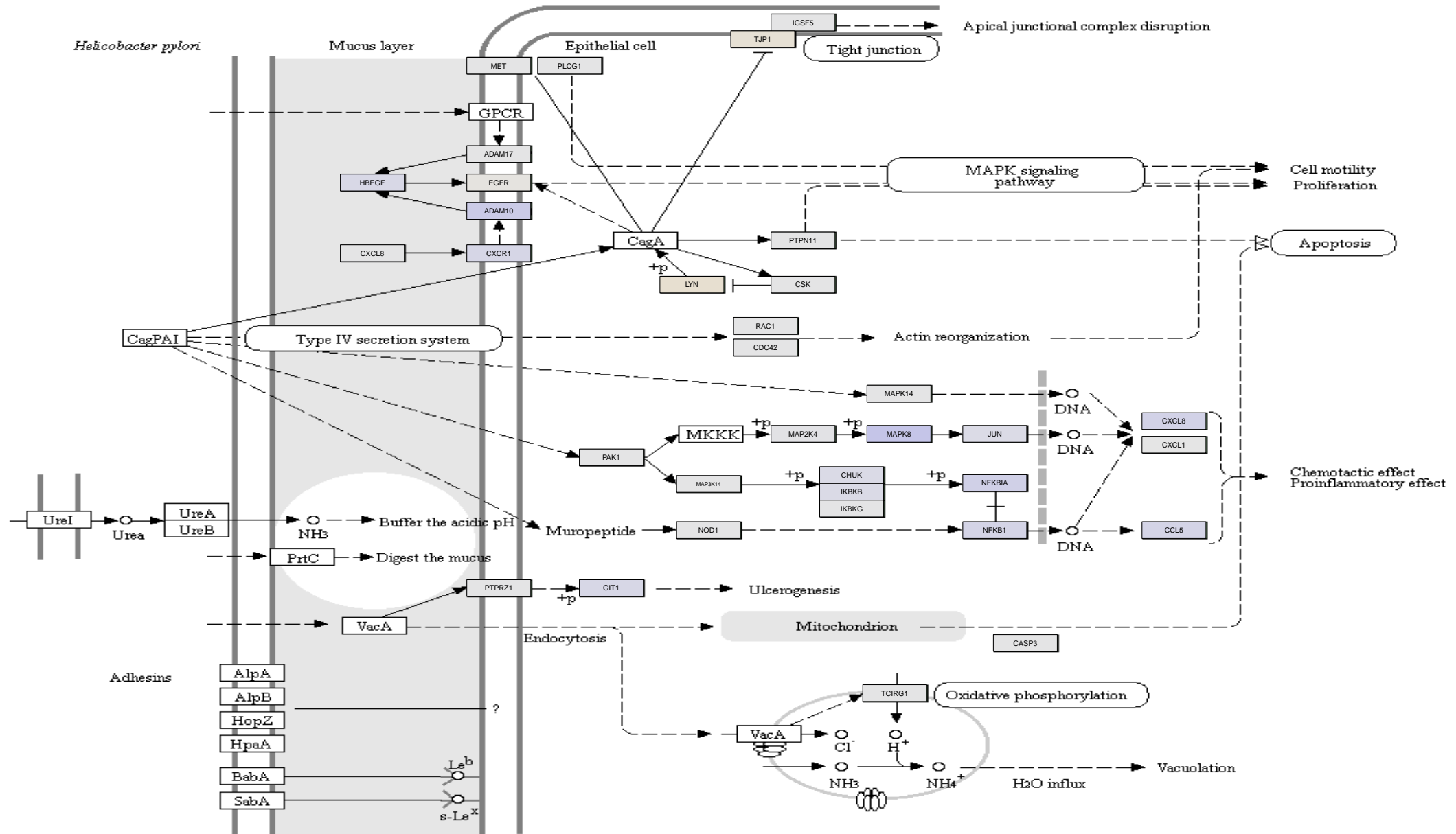

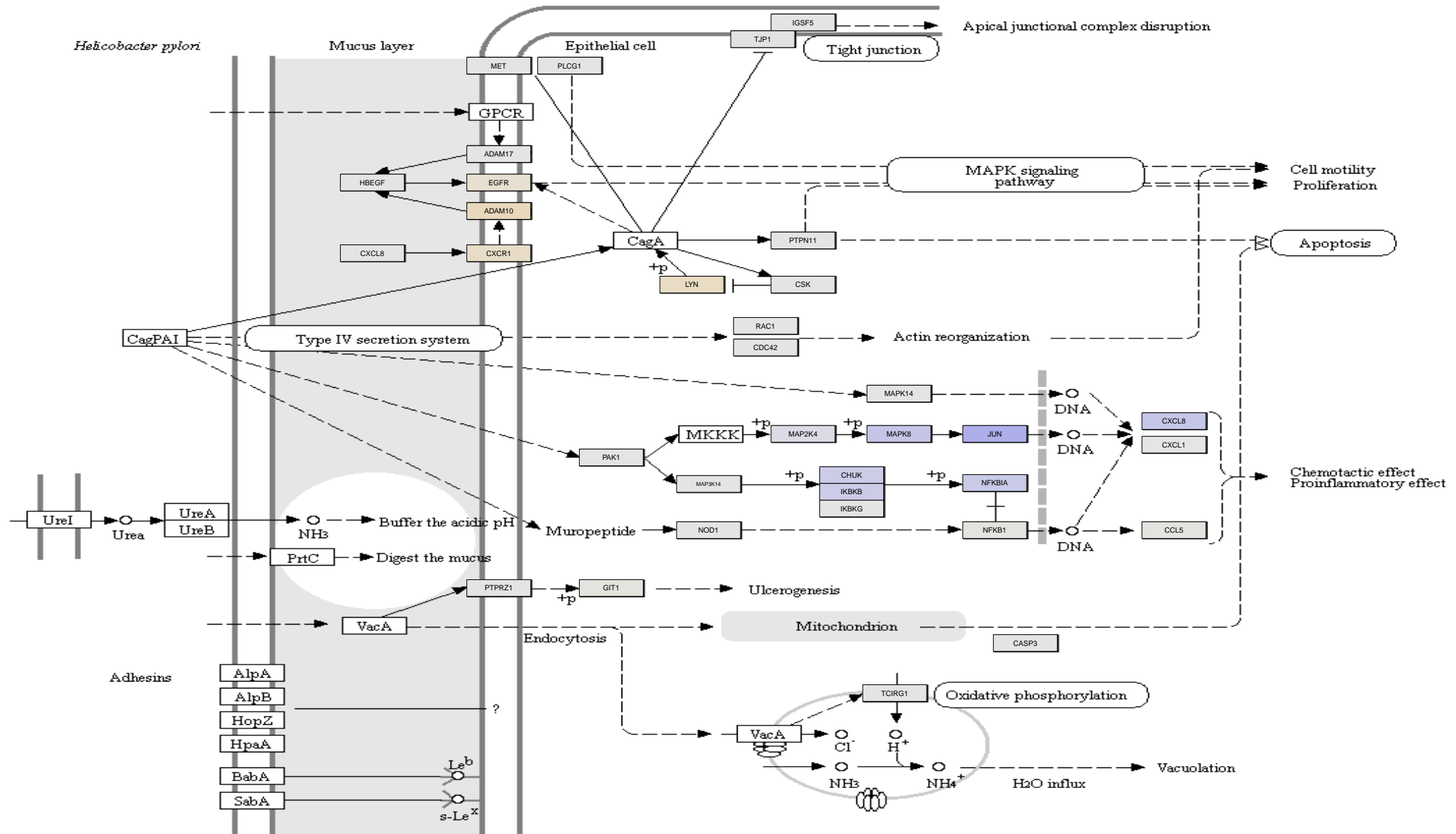

Supplement: Supplementary file 1 [file cells-11-00362-s001.zip › Suppl-Material-S4-Pathways-PSF_Methylation/Epithelial_cell_signaling_in_Helicobacter_pylori_infection.pdf]

Hedgehog signaling pathway

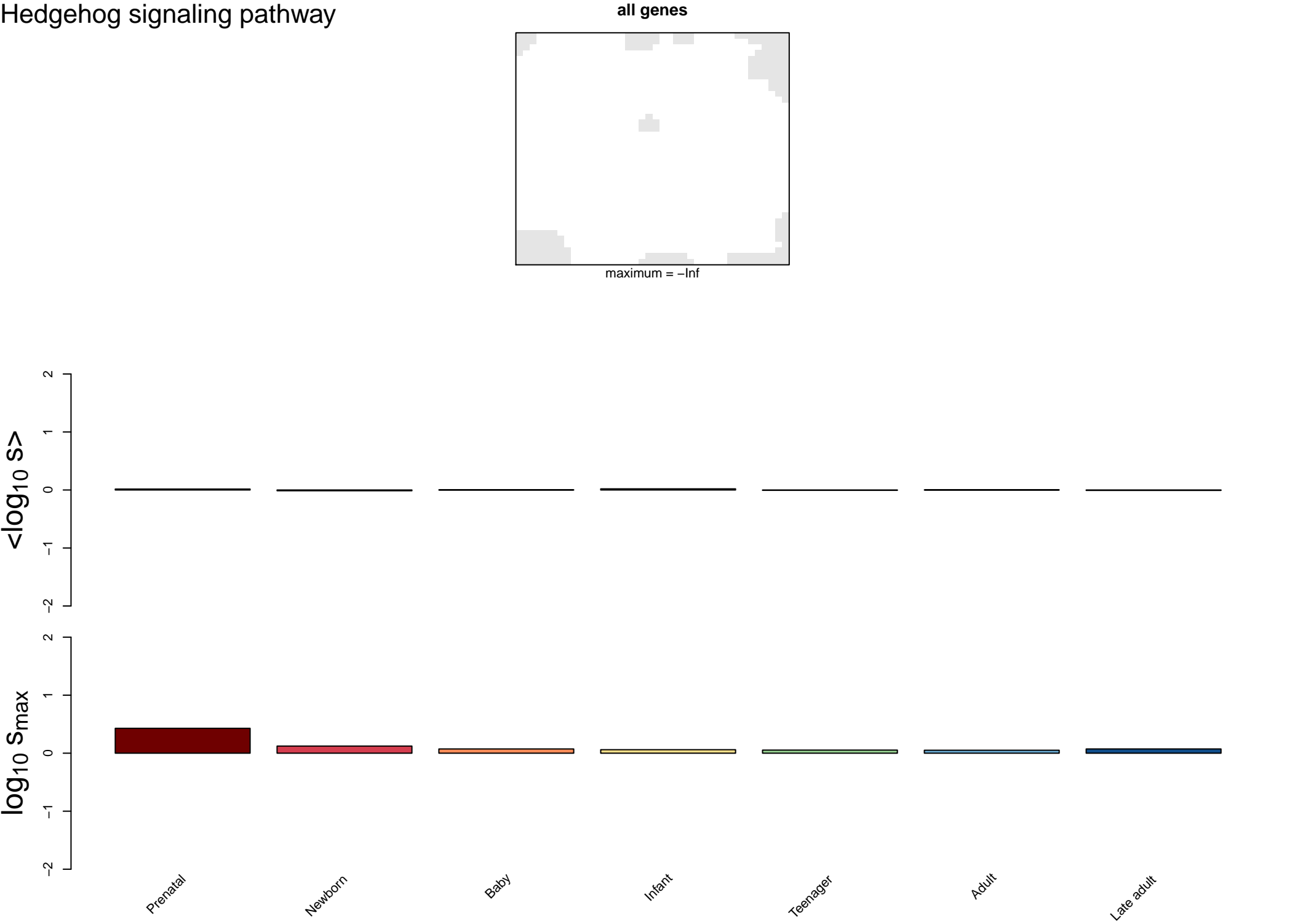

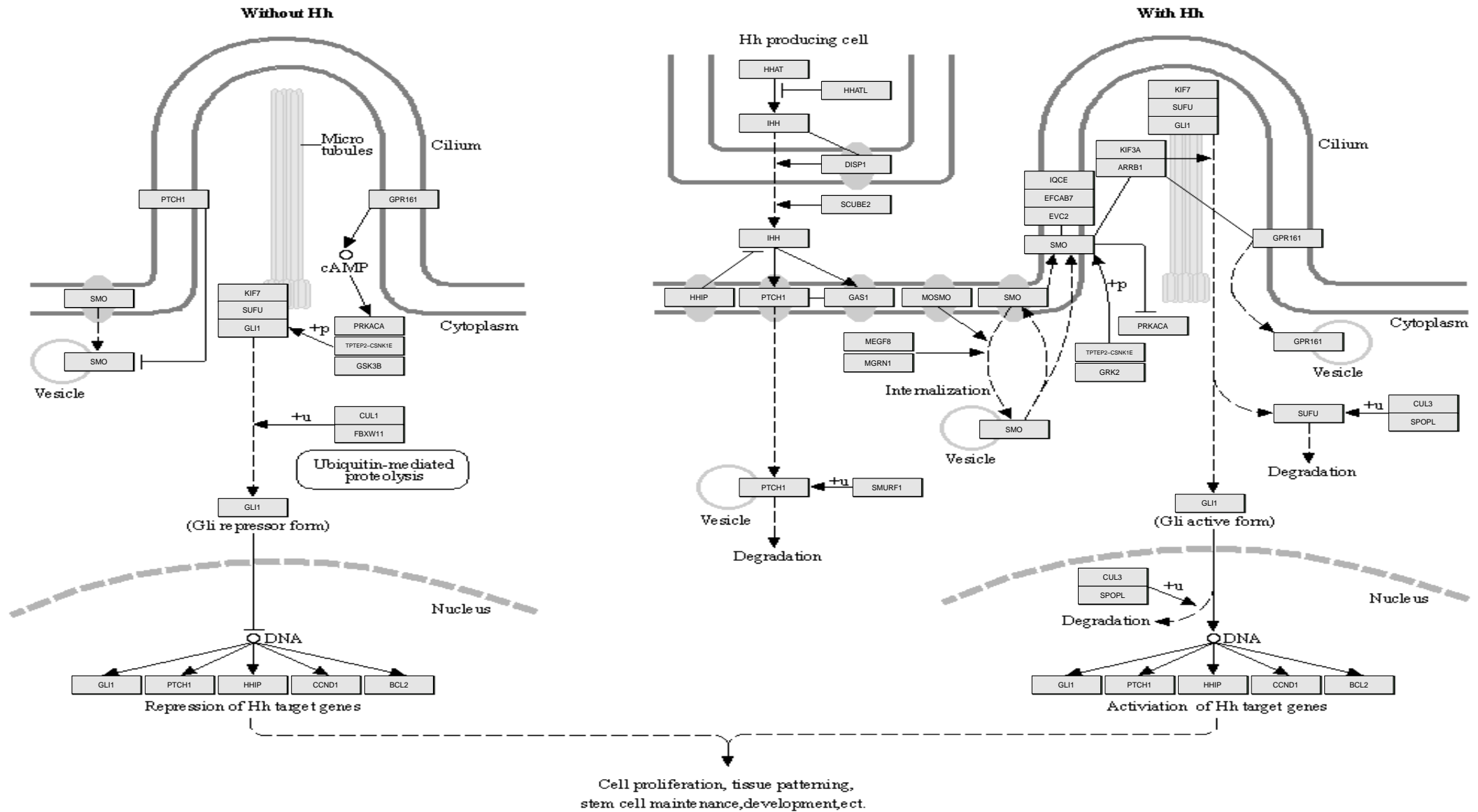

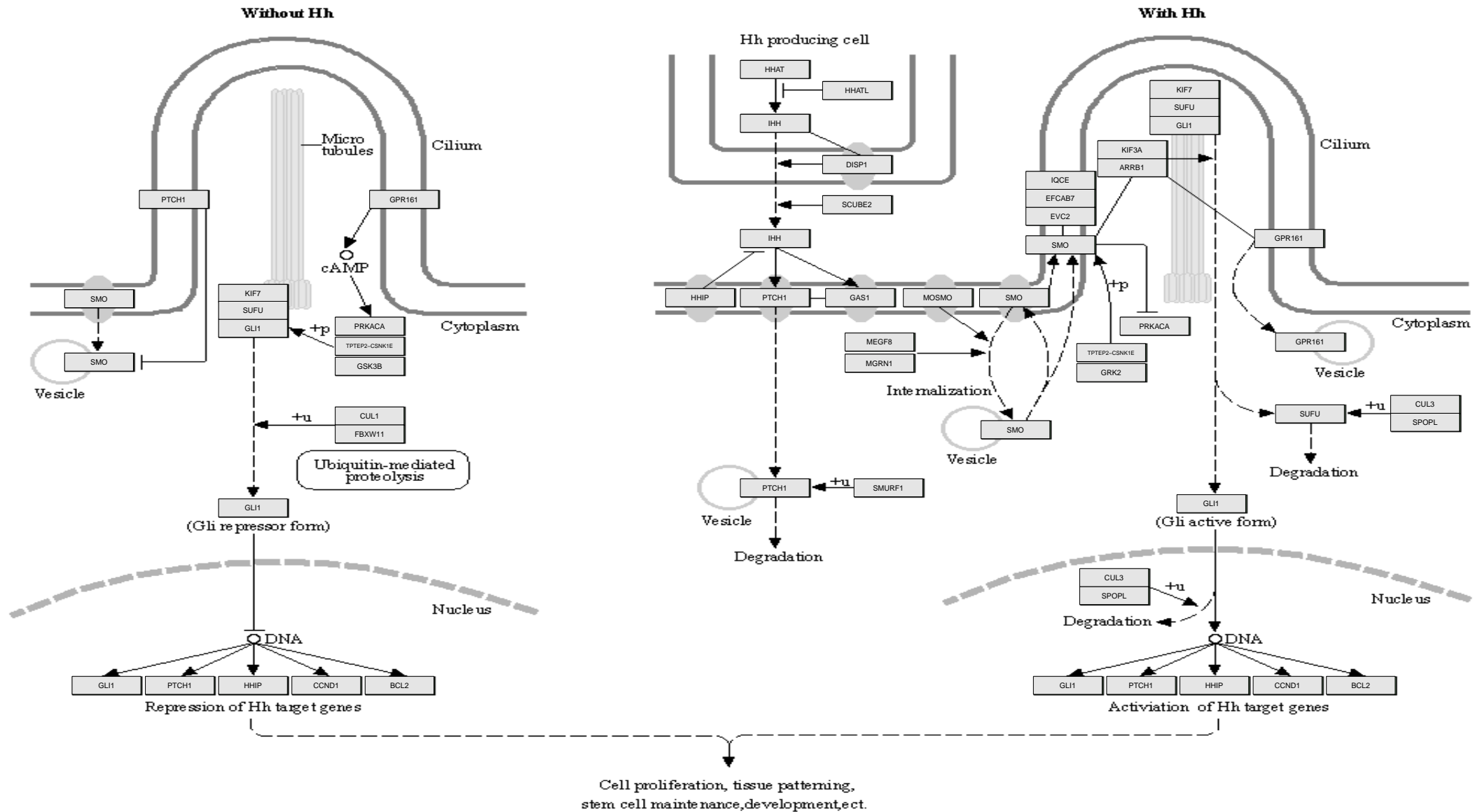

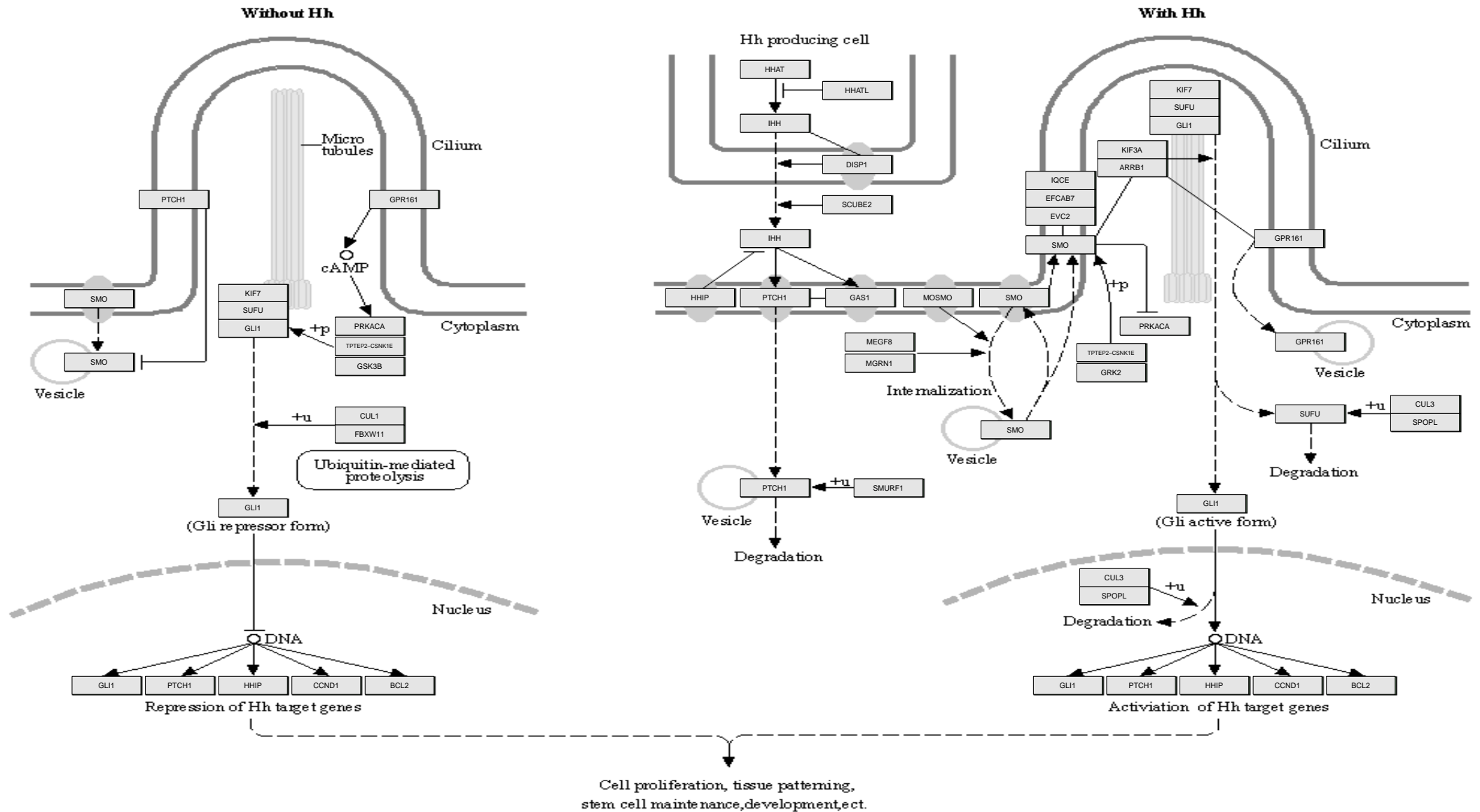

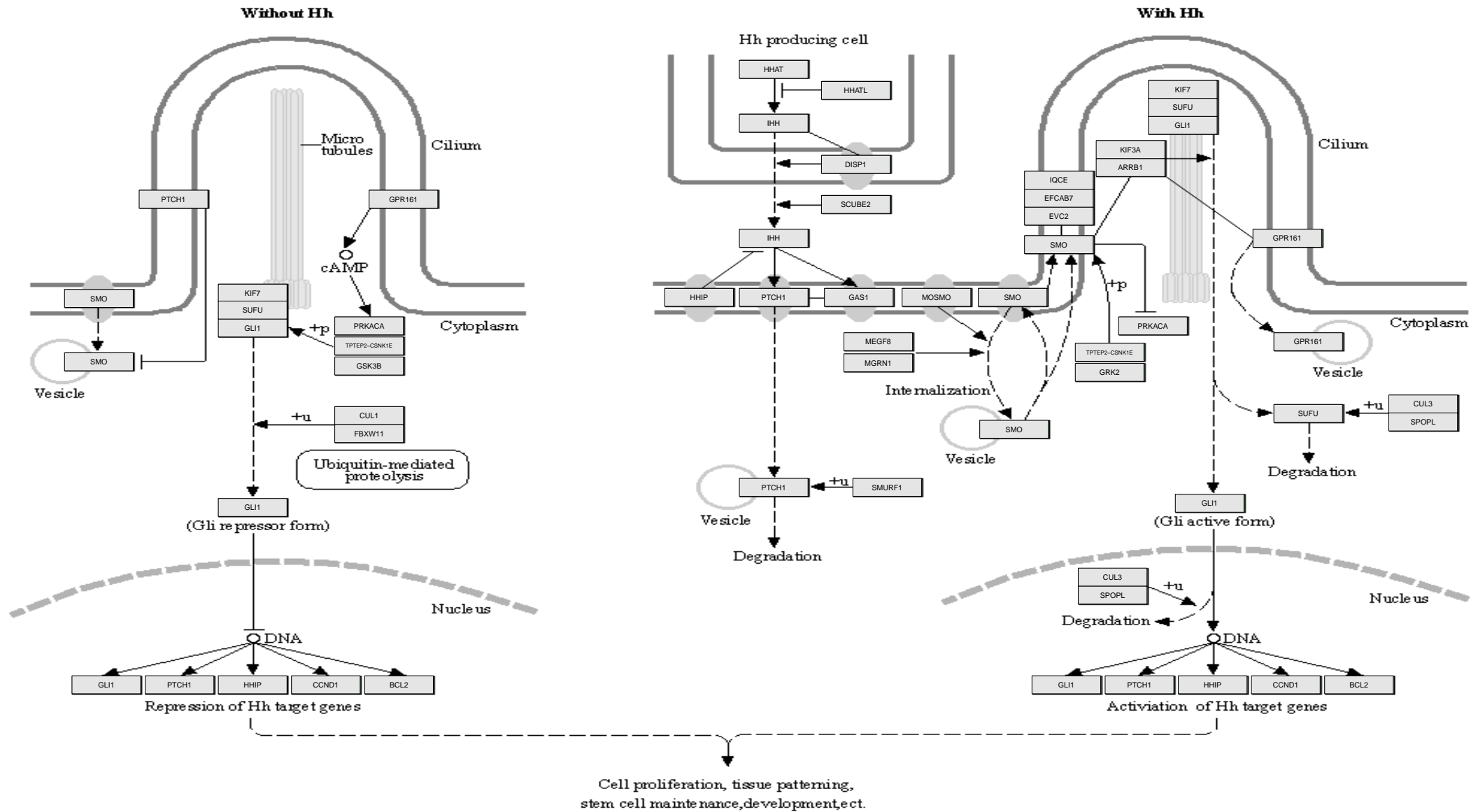

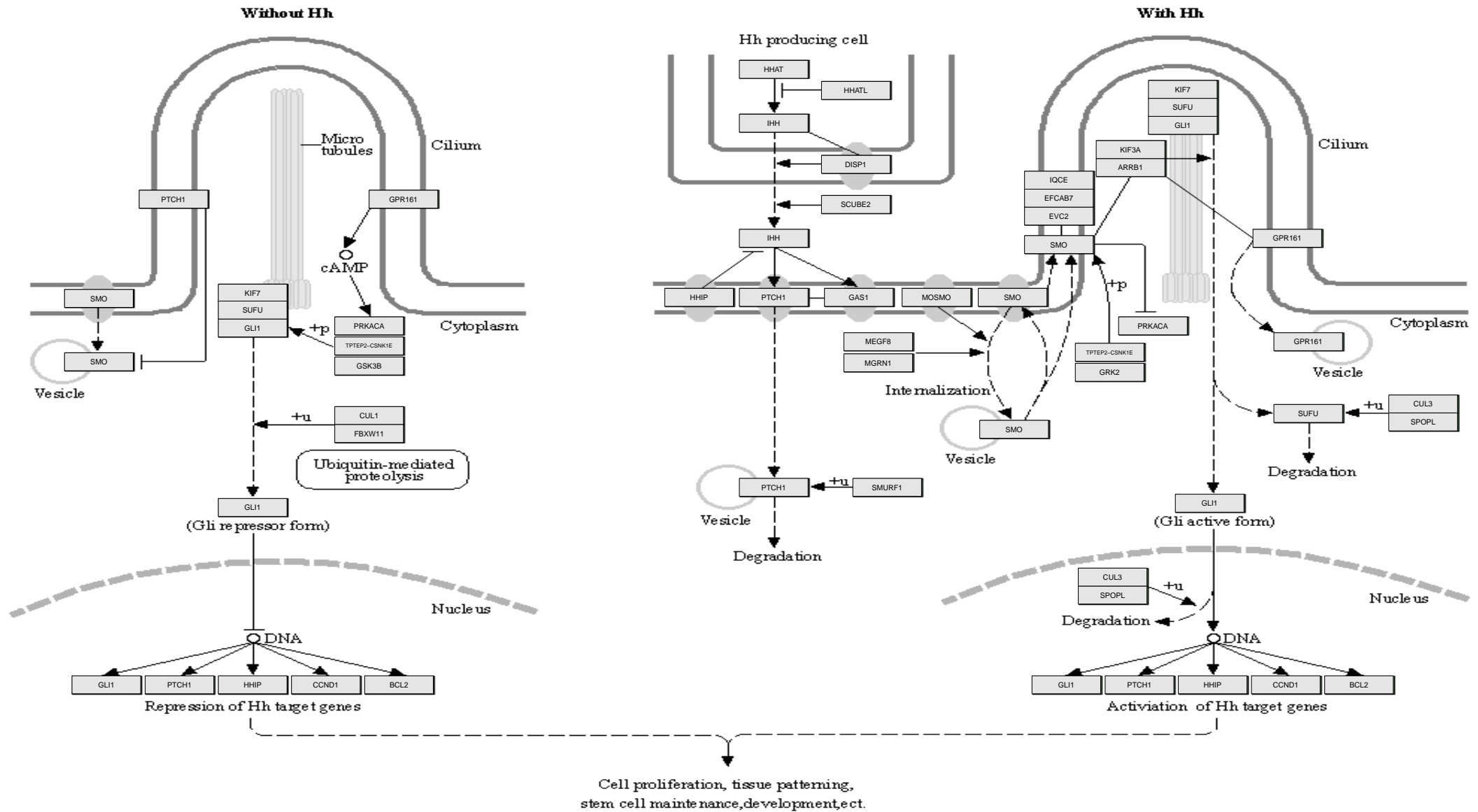

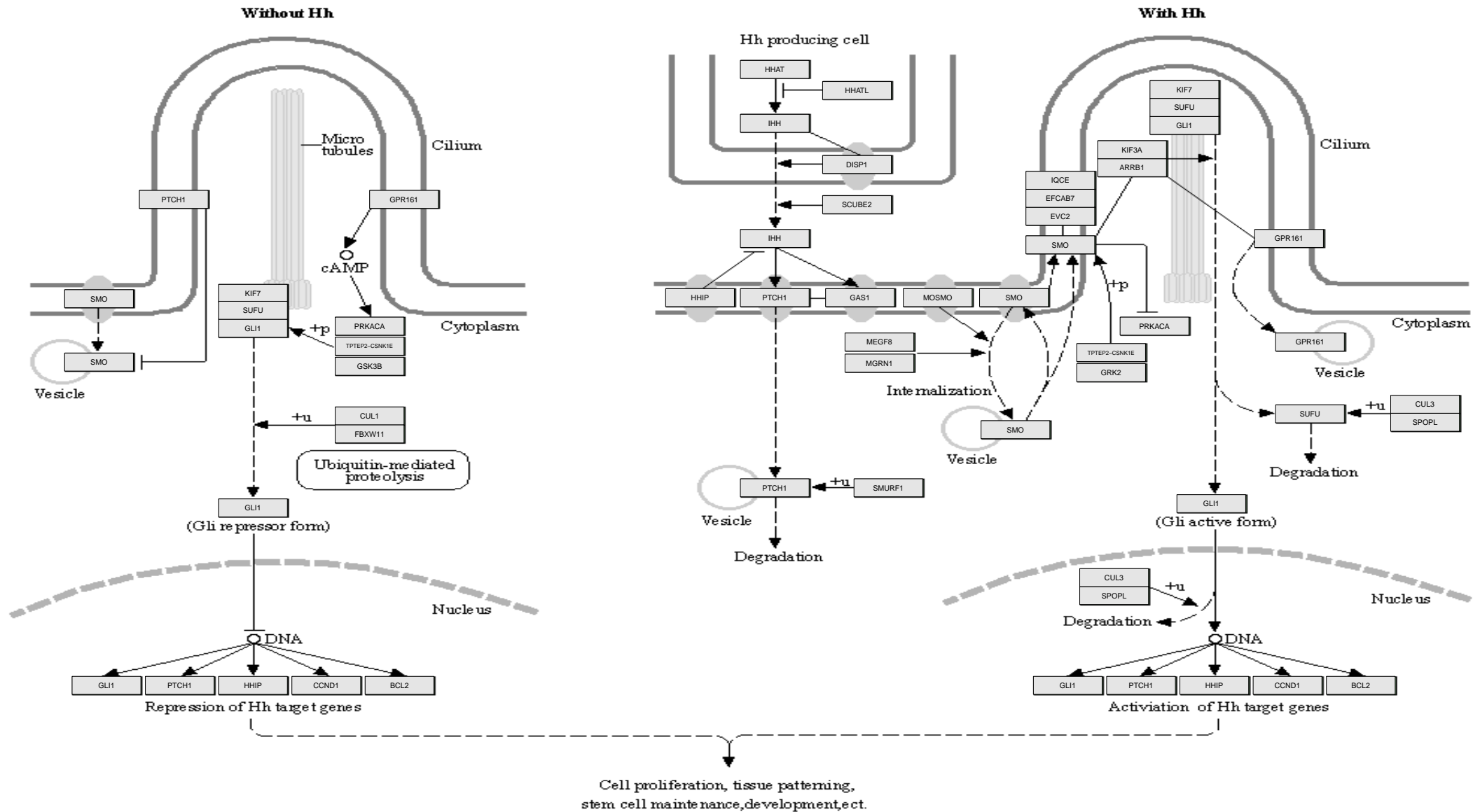

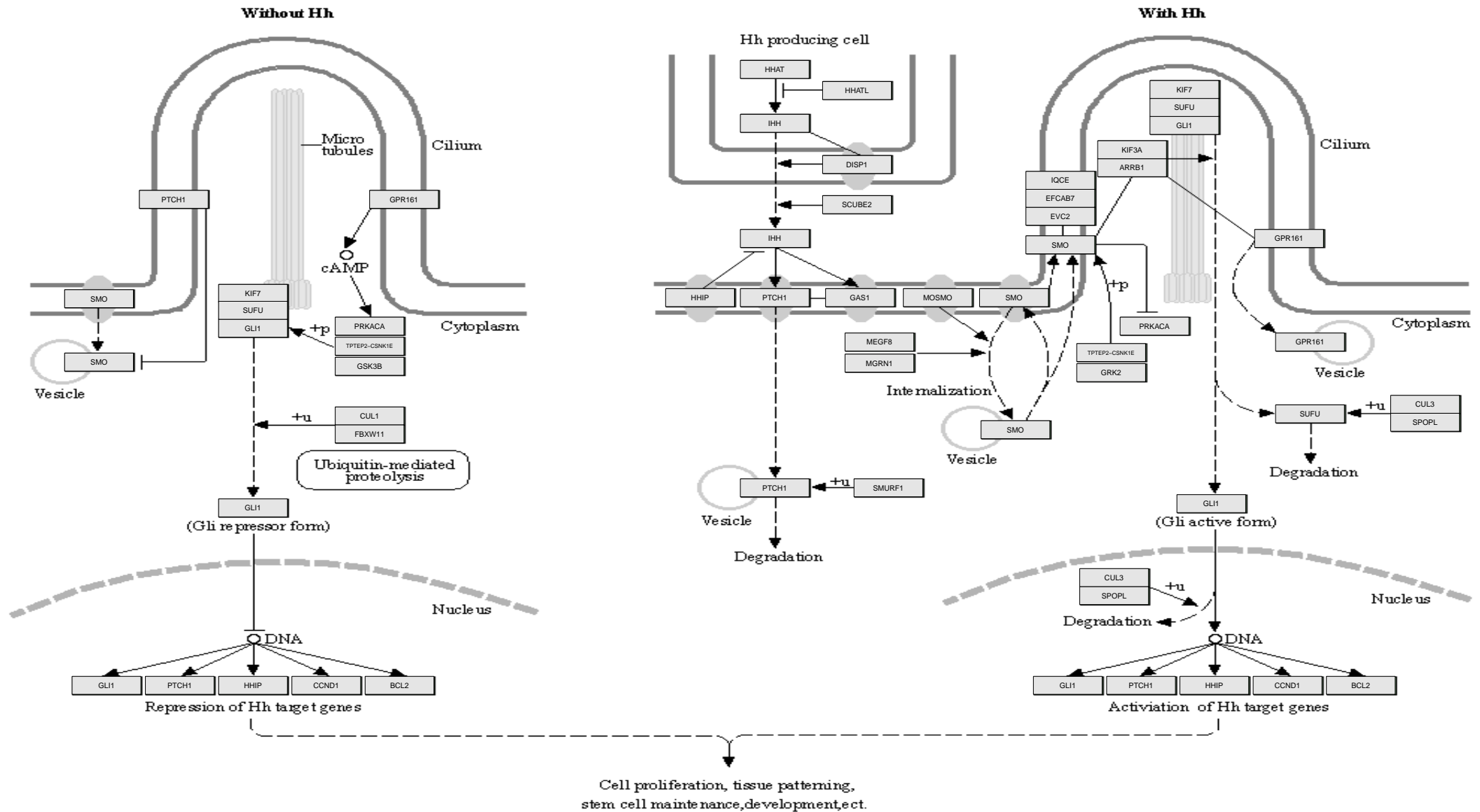

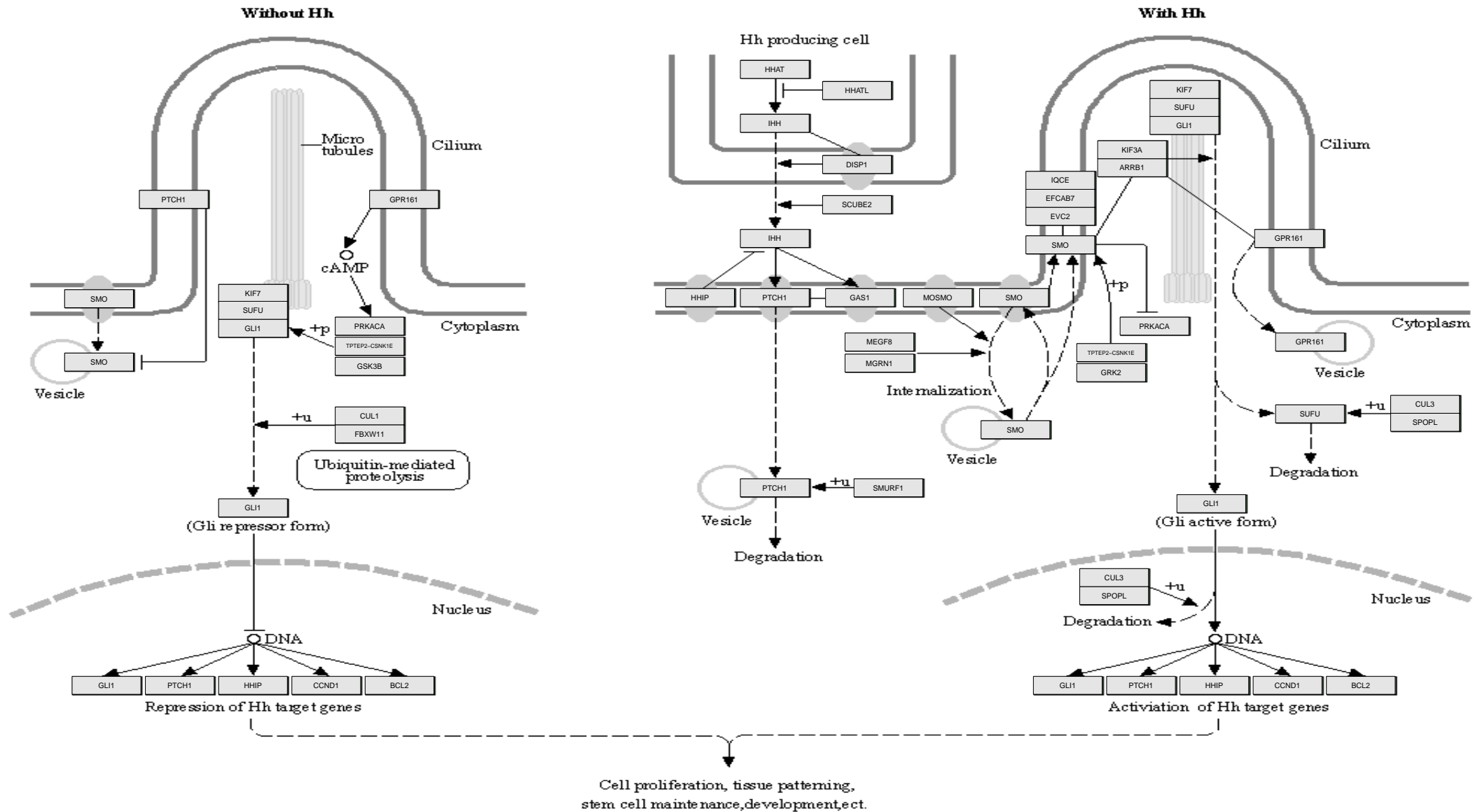

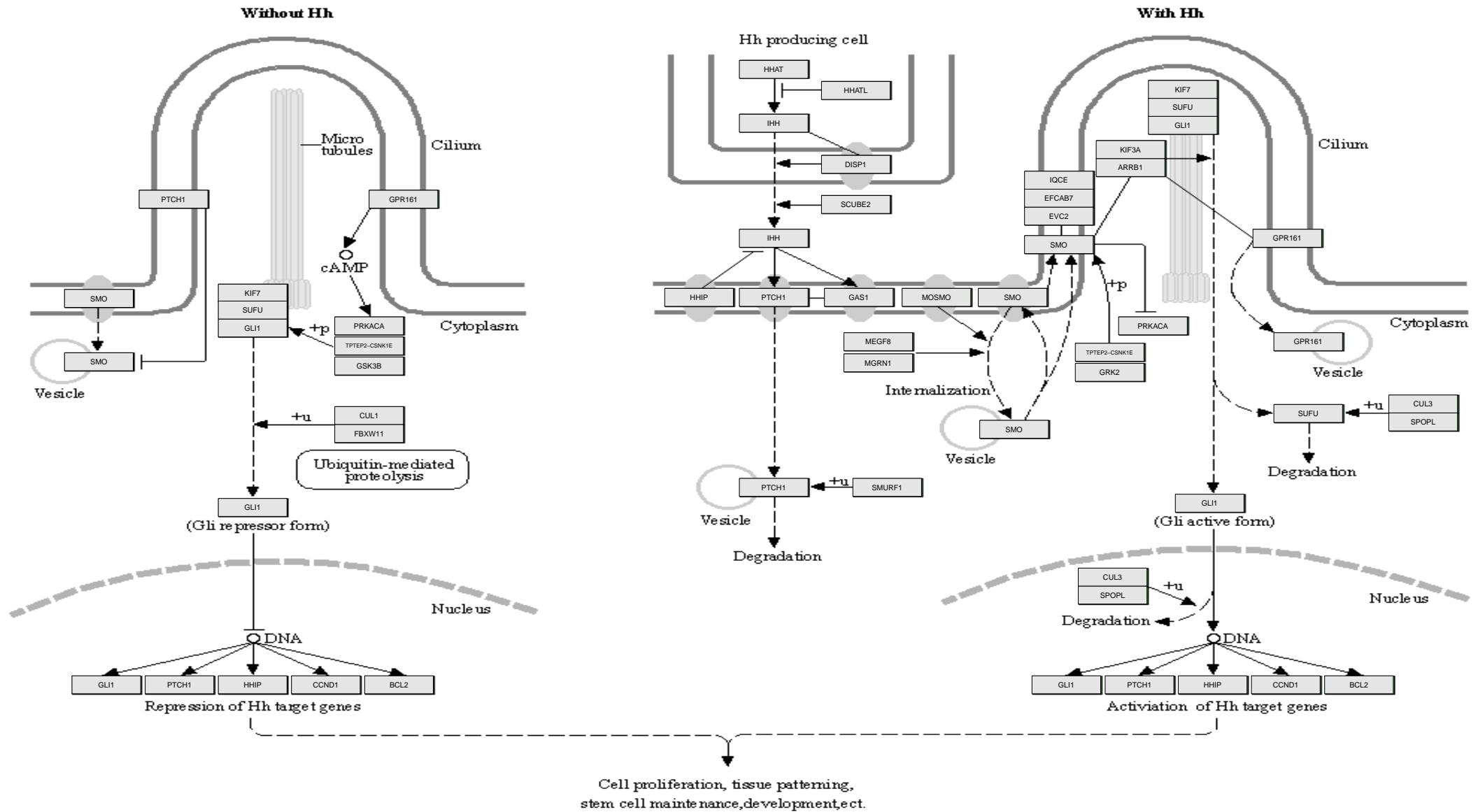

Supplement: Supplementary file 1 [file cells-11-00362-s001.zip › Suppl-Material-S4-Pathways-PSF_Methylation/Hedgehog_signaling_pathway.pdf]

all genes

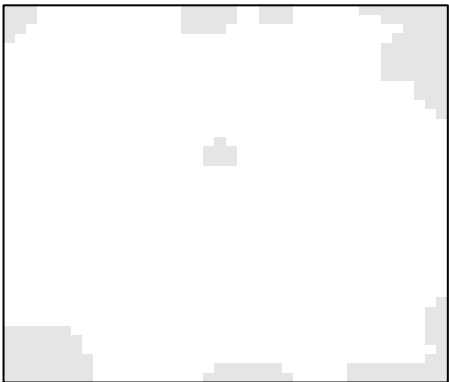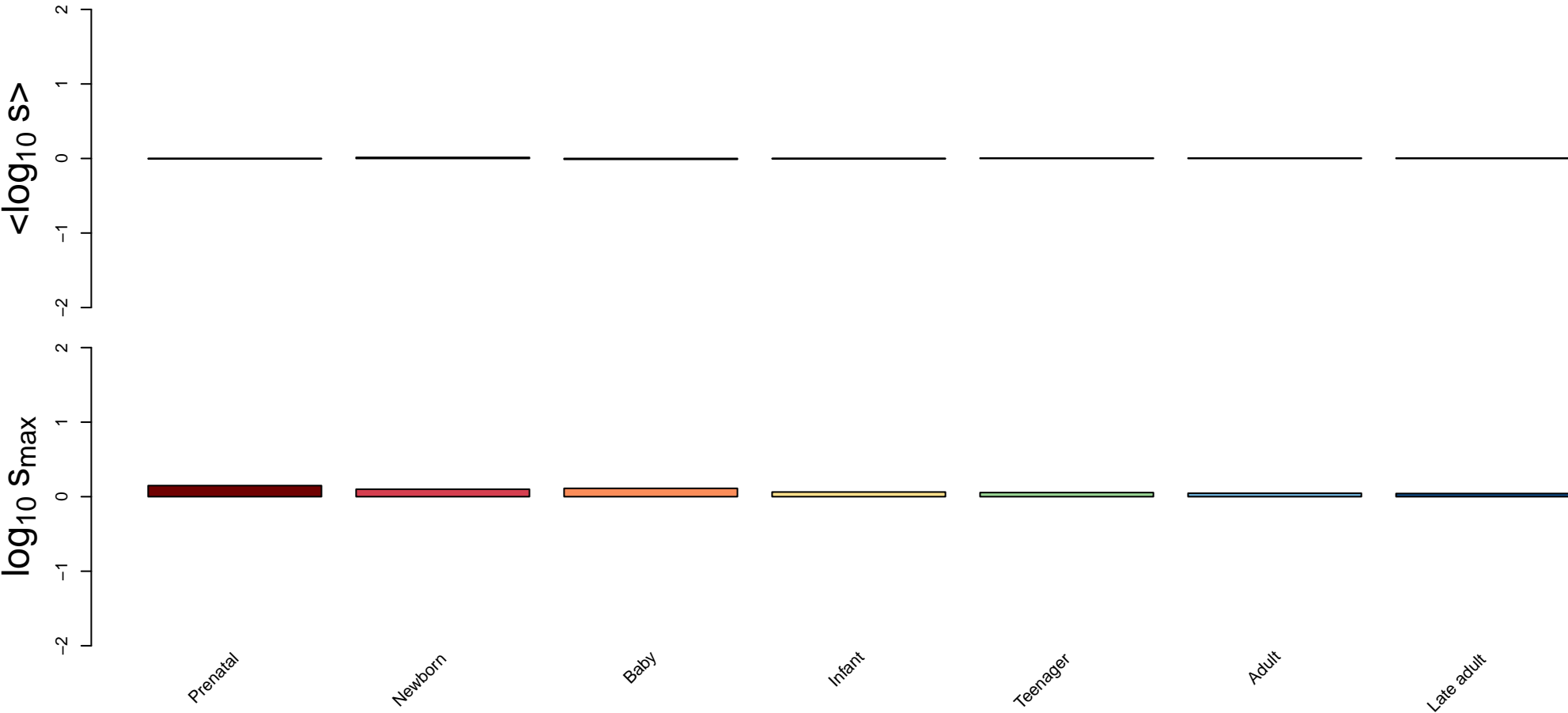

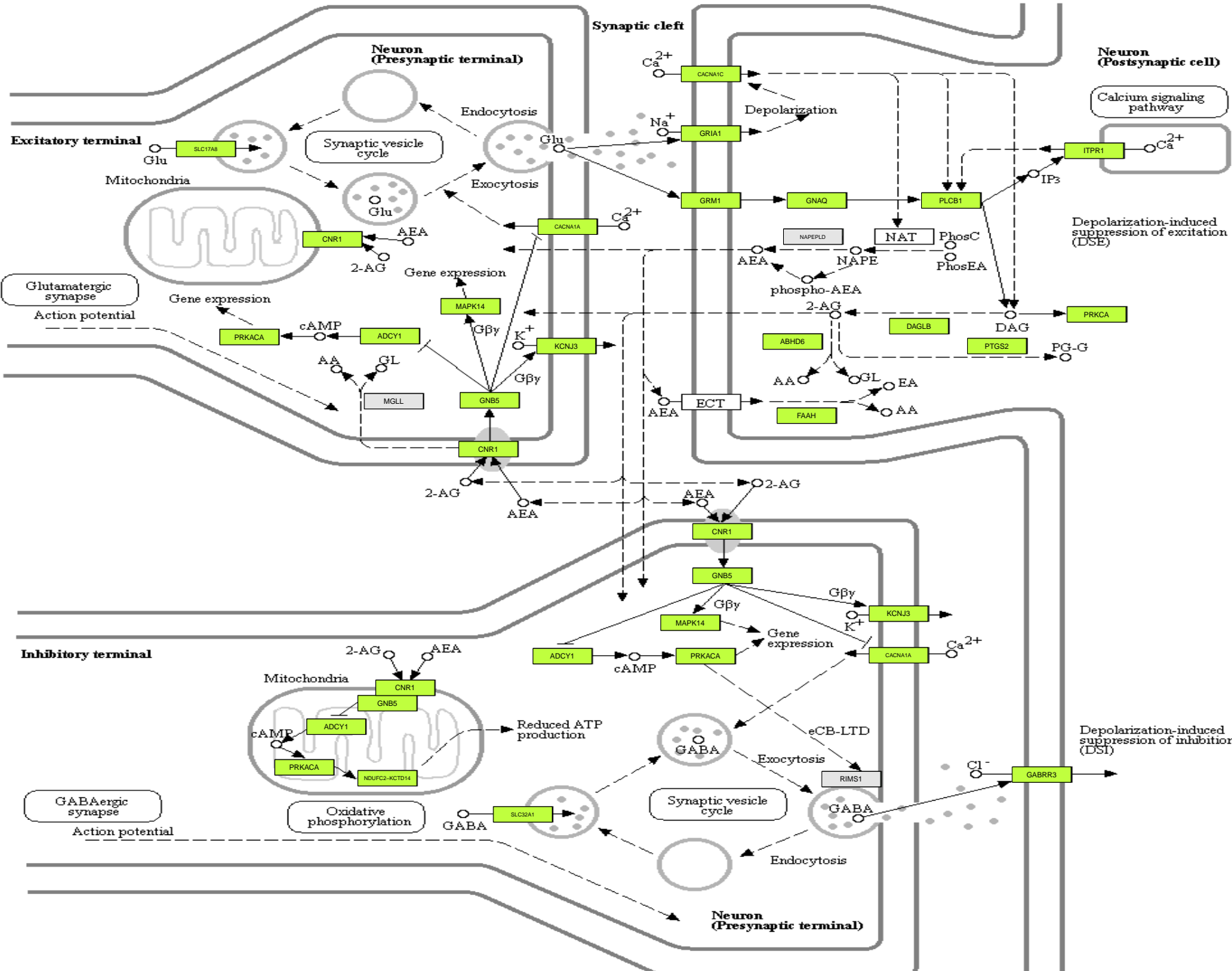

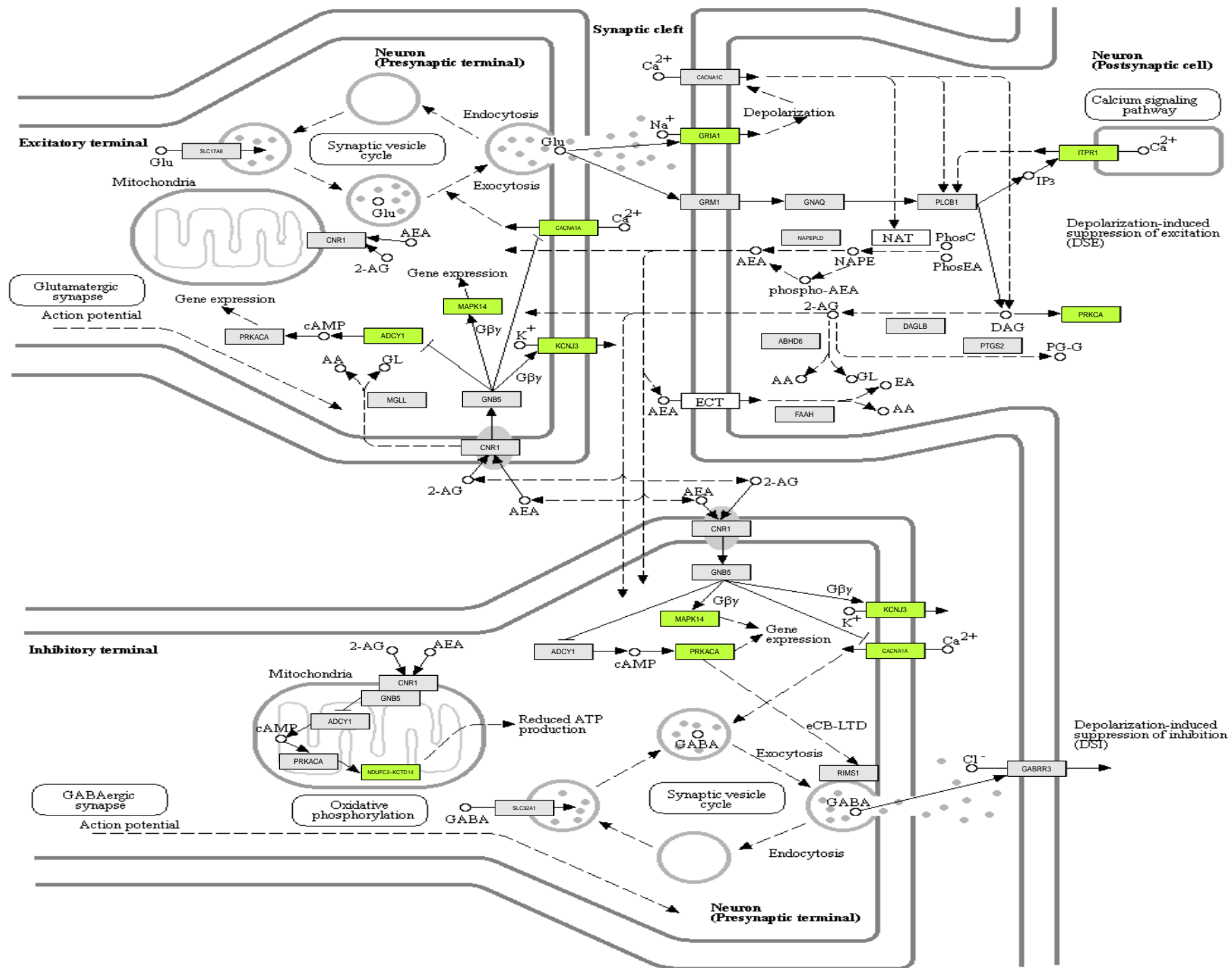

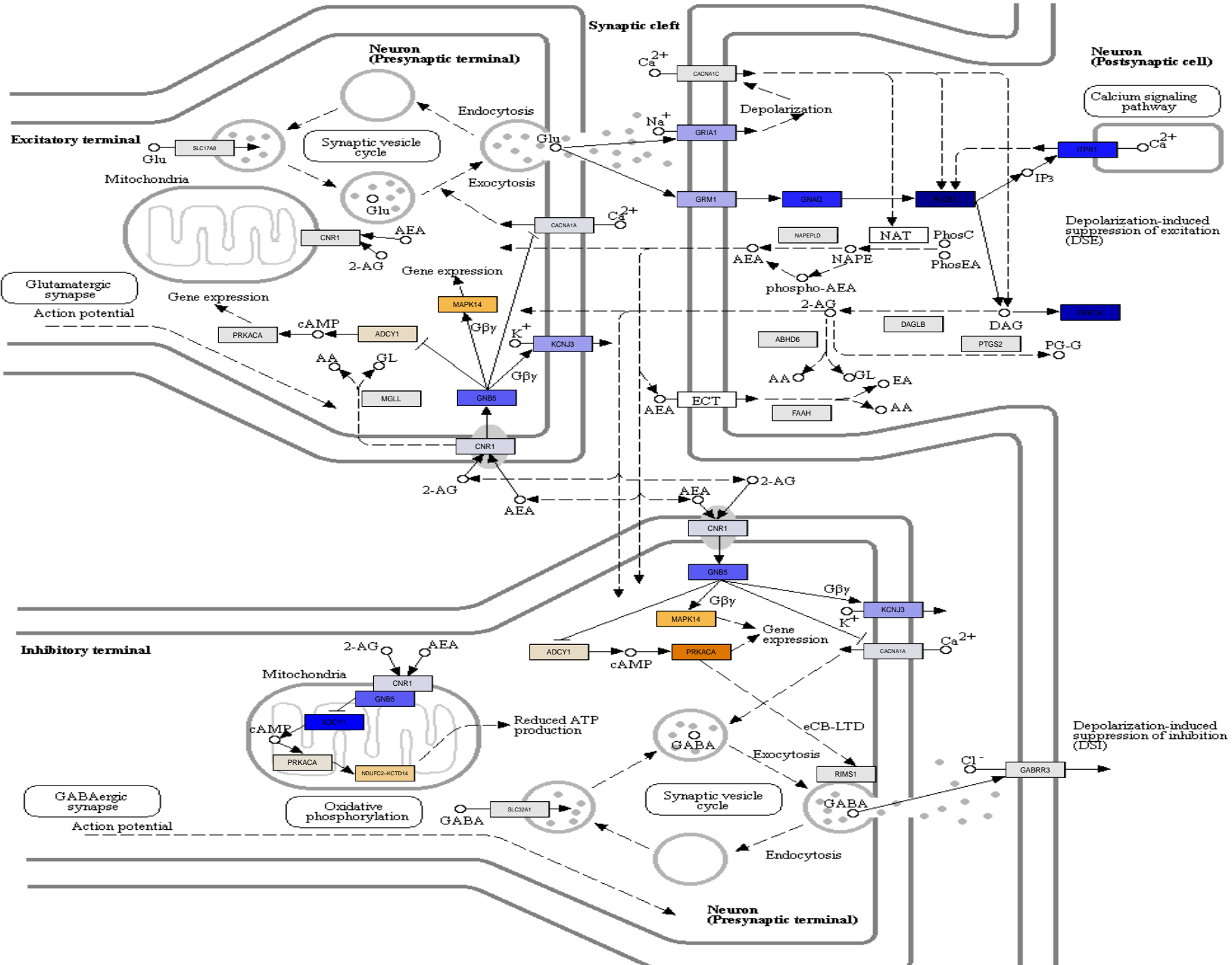

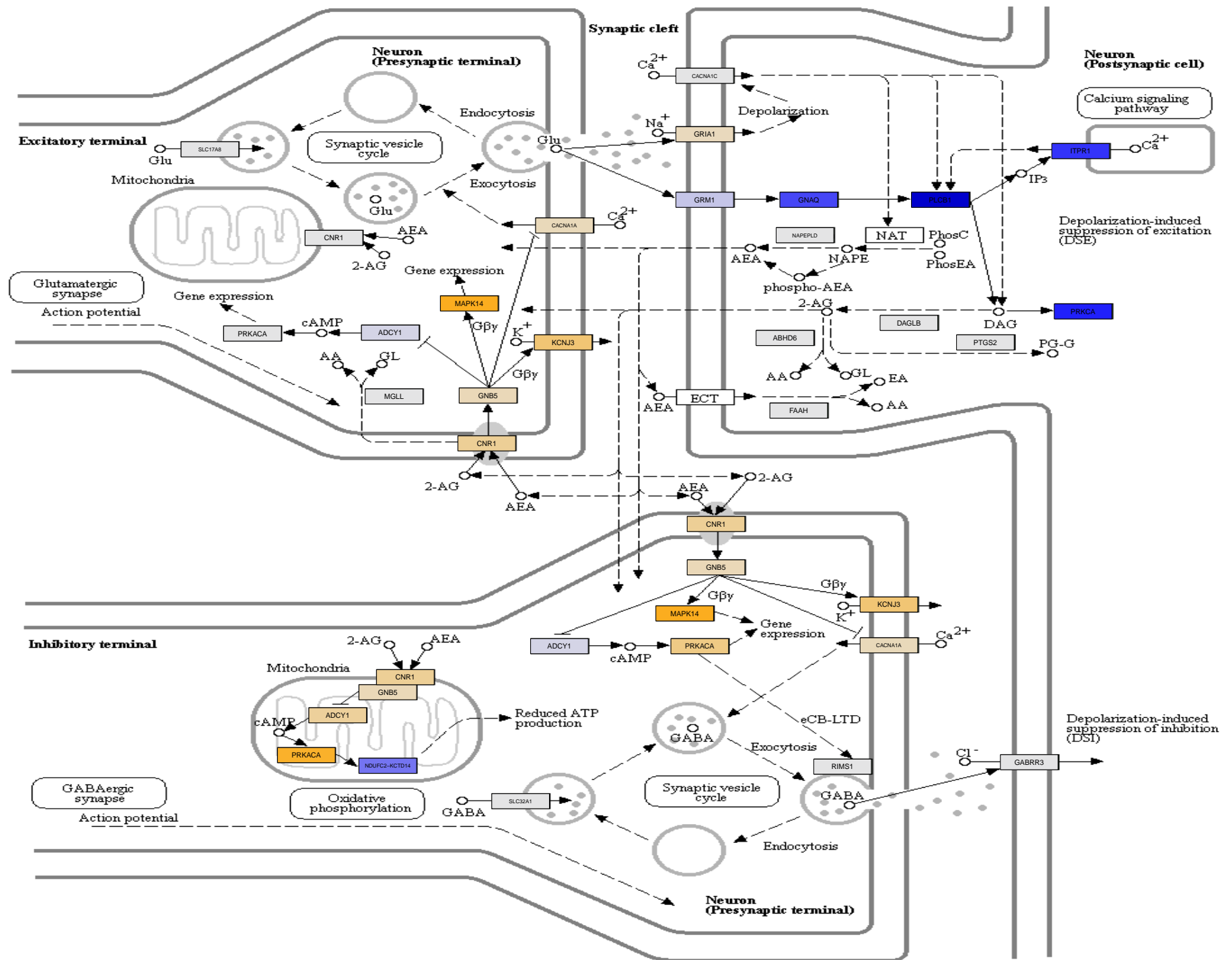

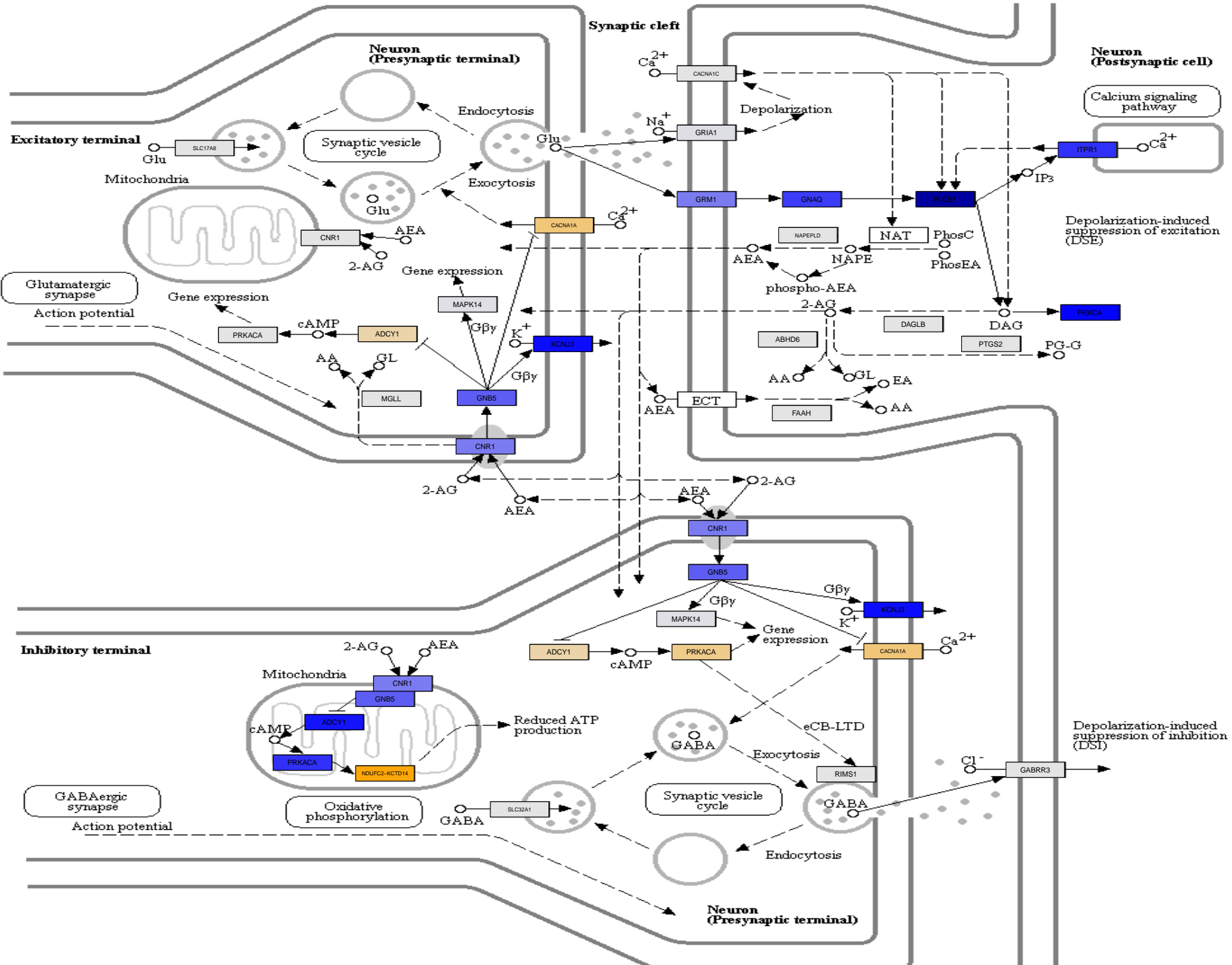

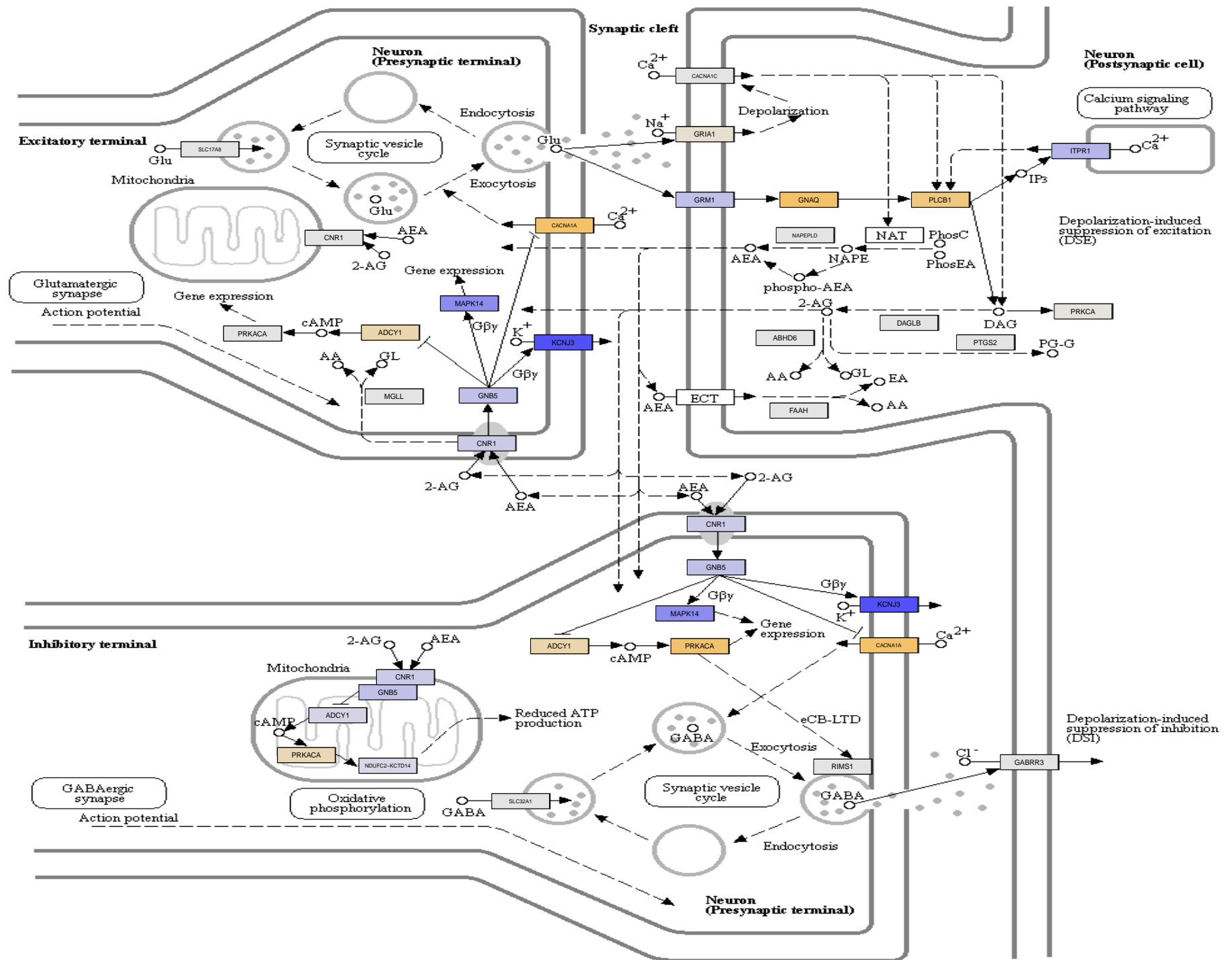

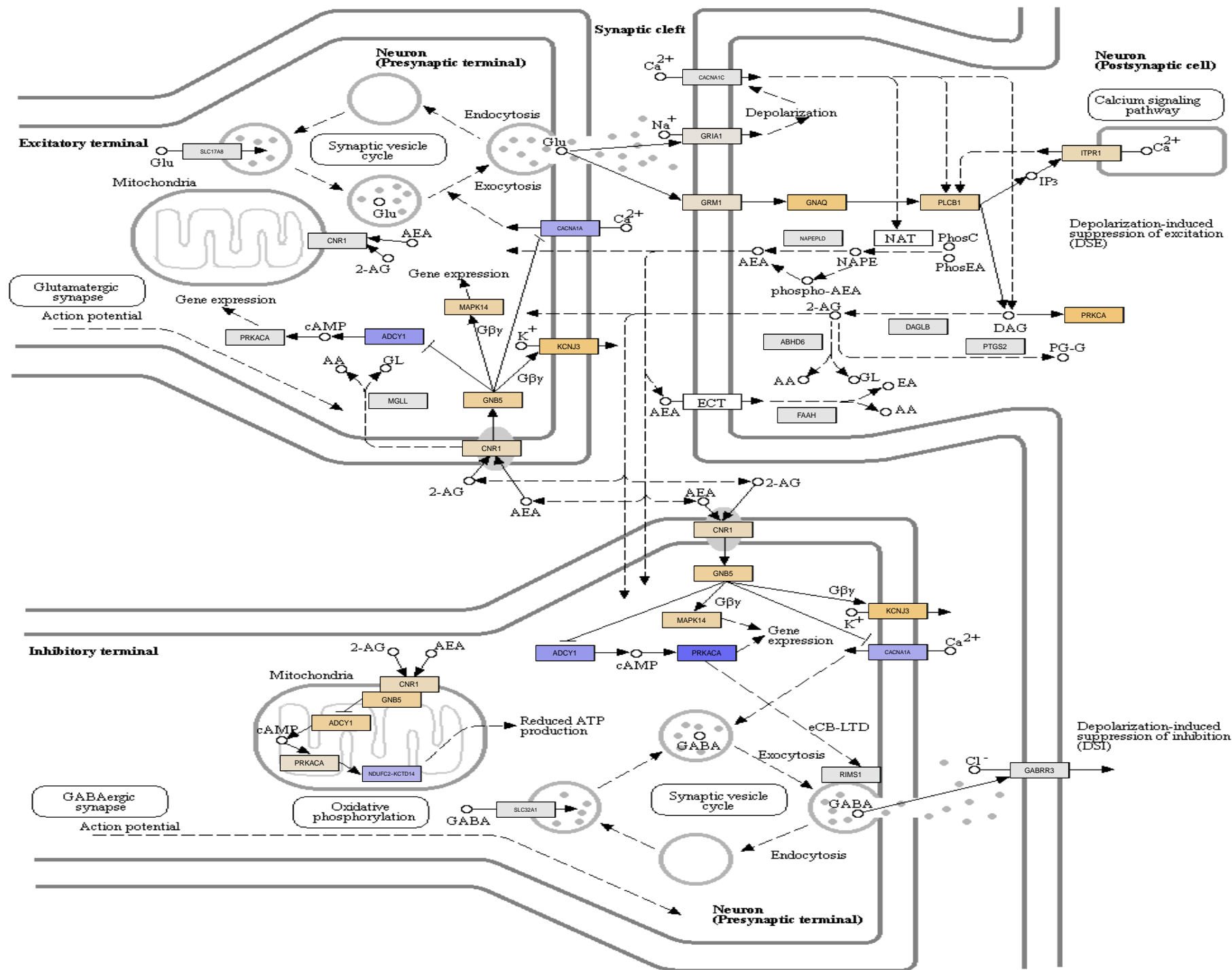

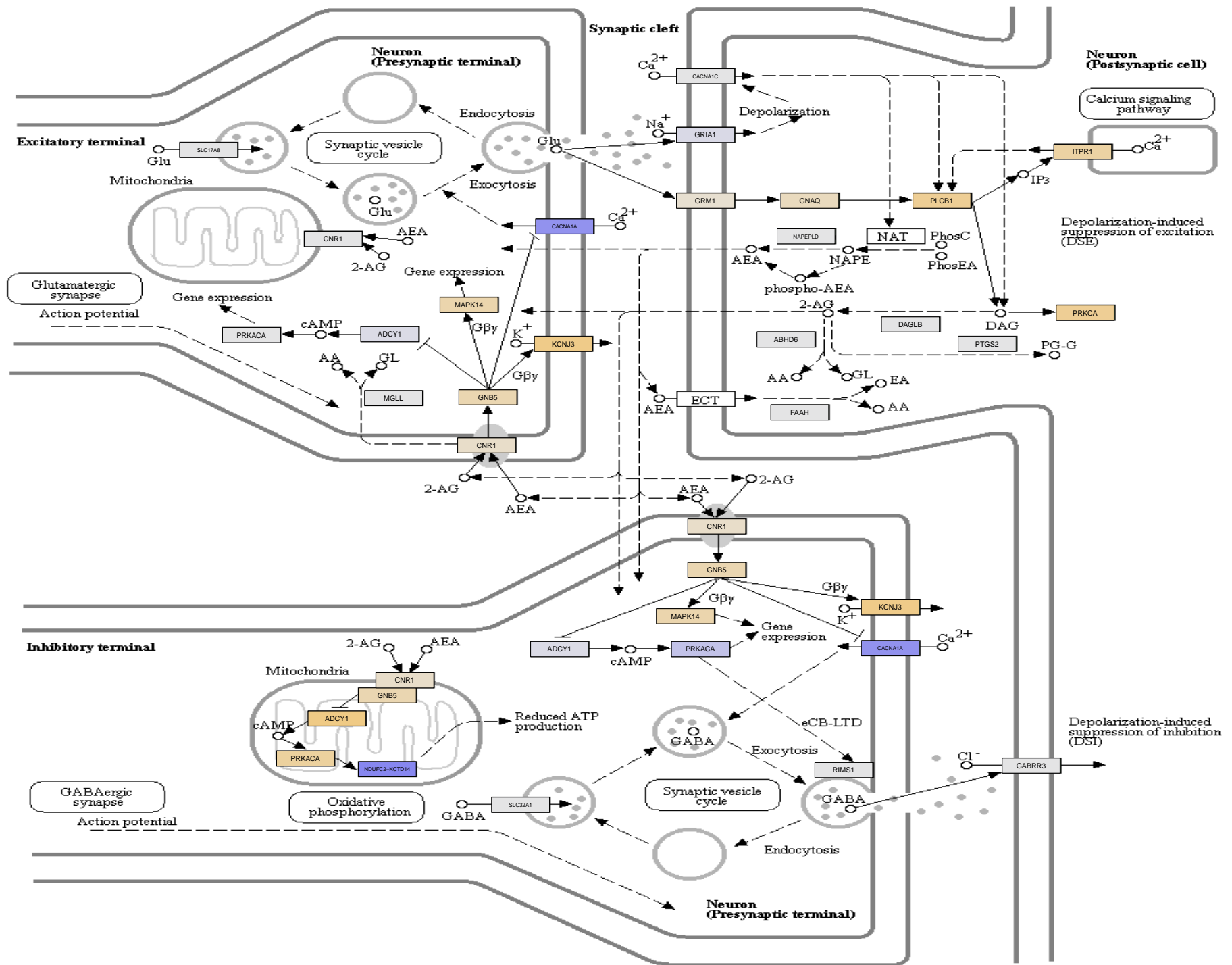

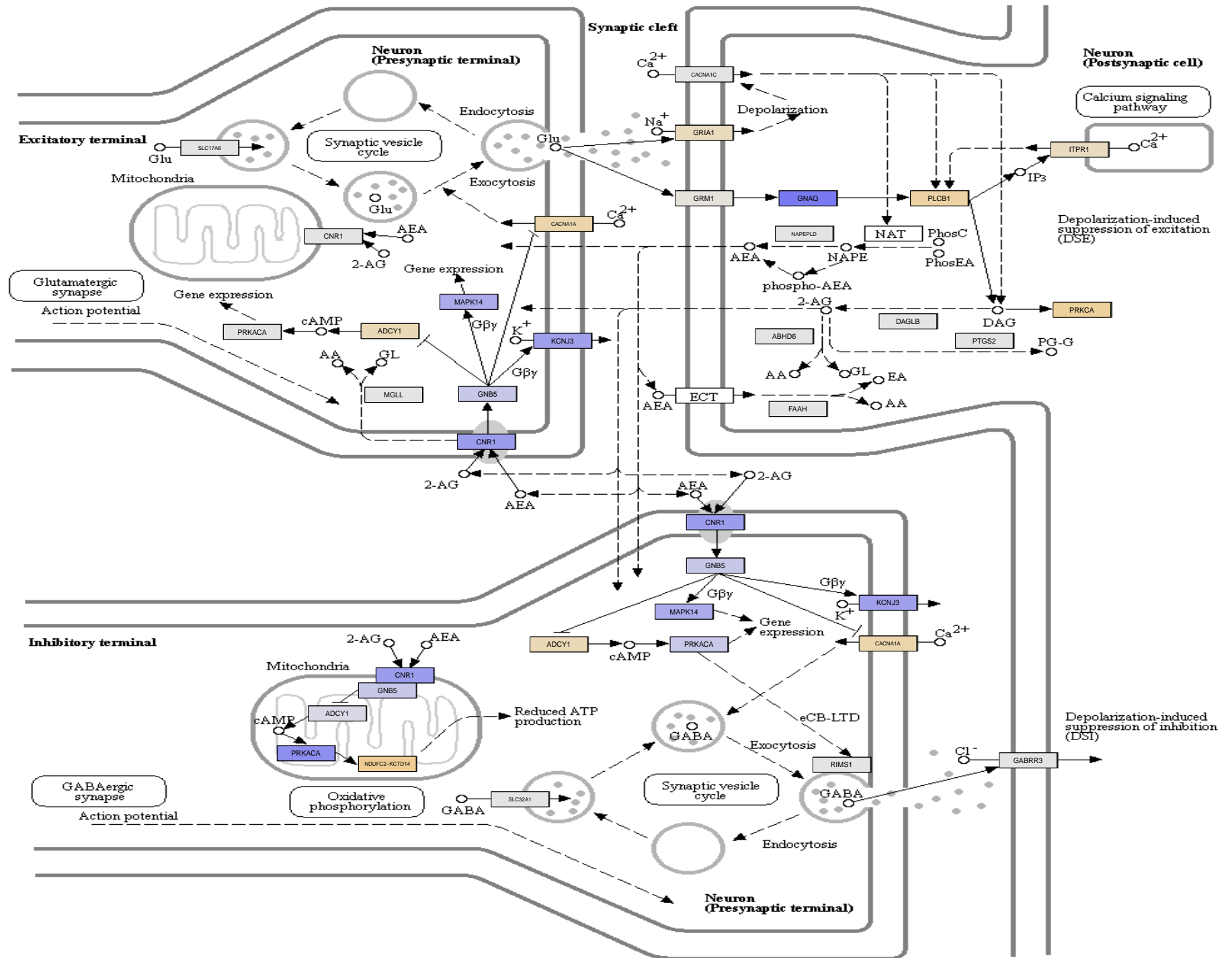

Supplement: Supplementary file 1 [file cells-11-00362-s001.zip › Suppl-Material-S4-Pathways-PSF_Methylation/Retrograde_endocannabinoid_signaling.pdf]

Sphingolipid signaling pathway

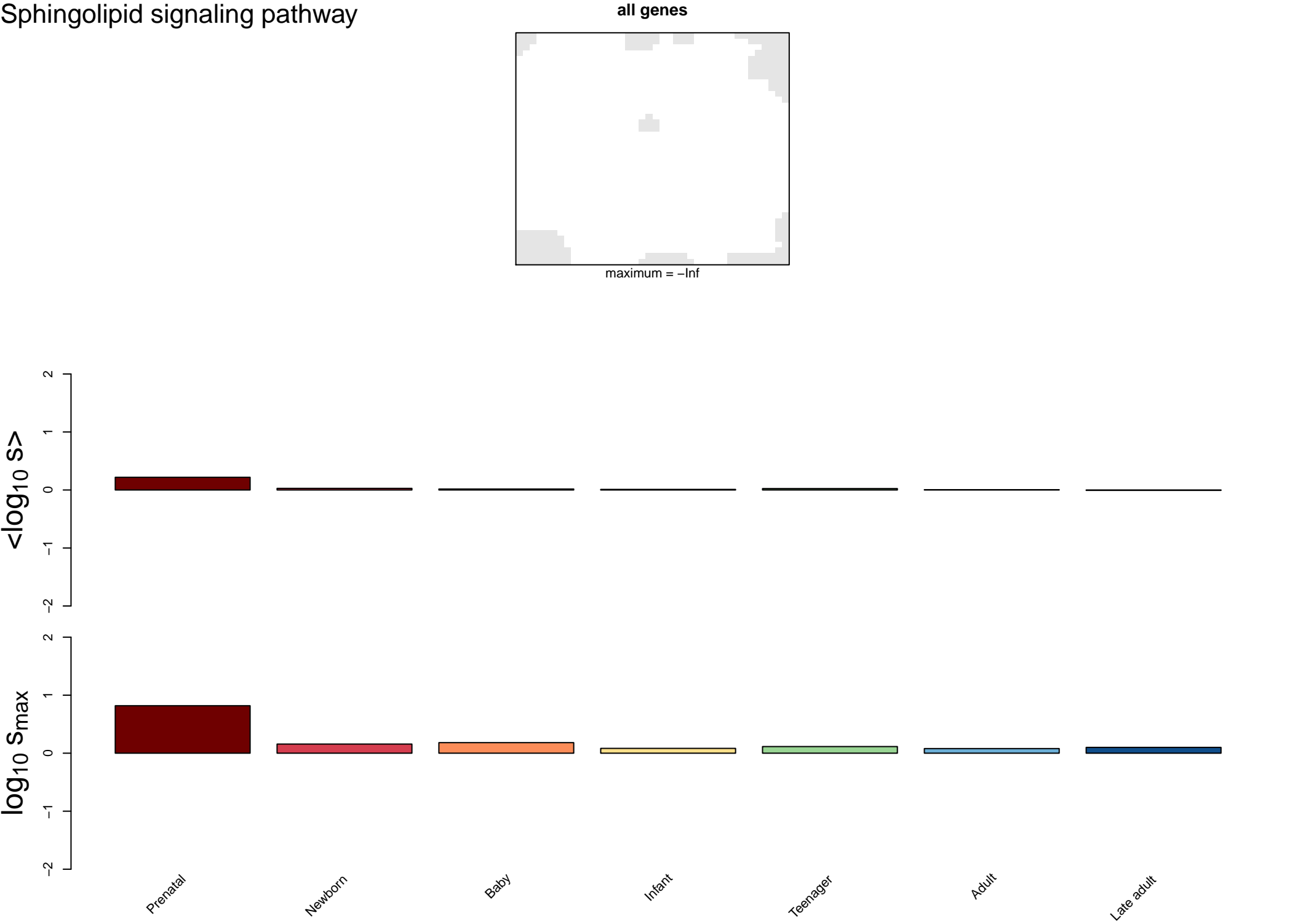

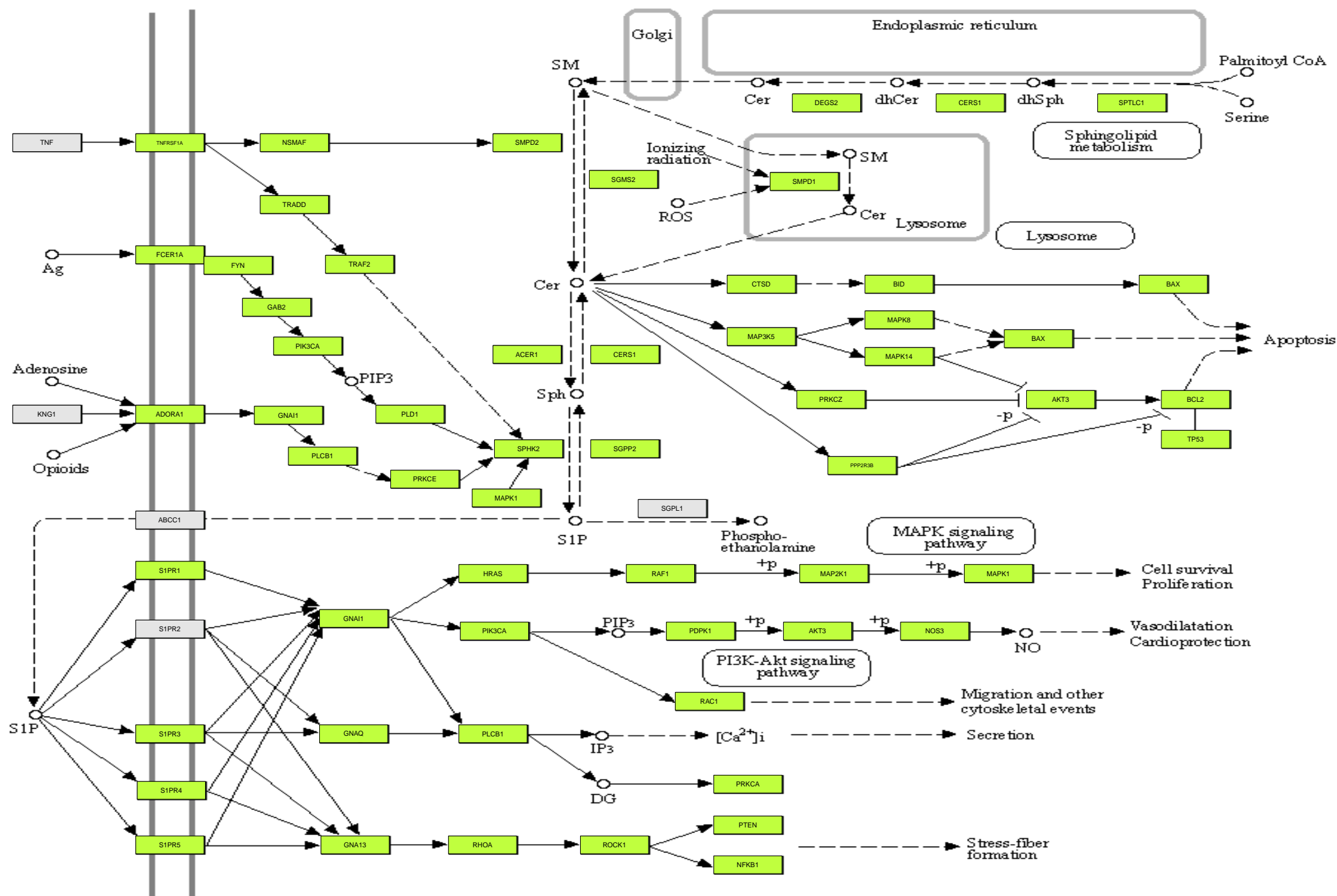

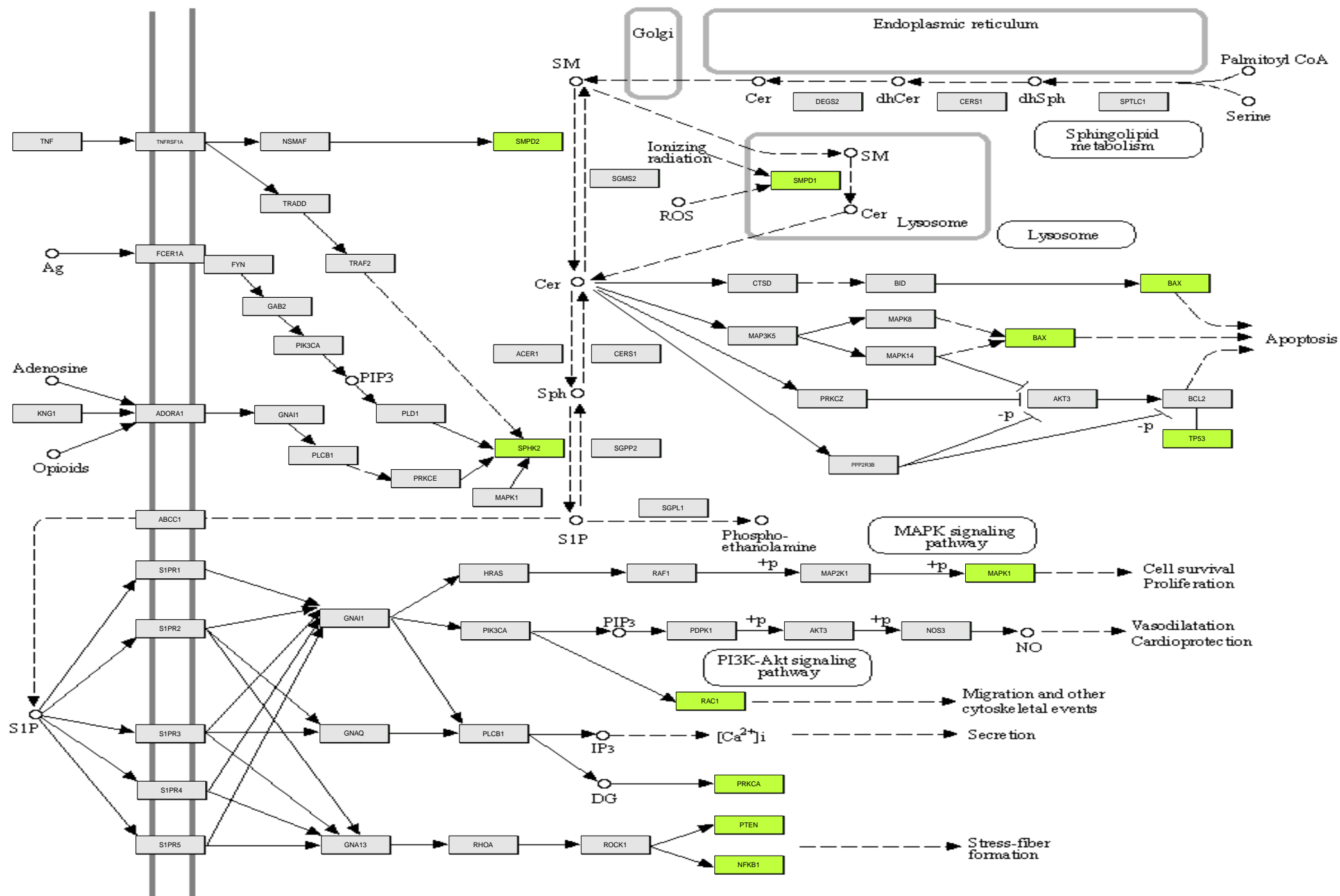

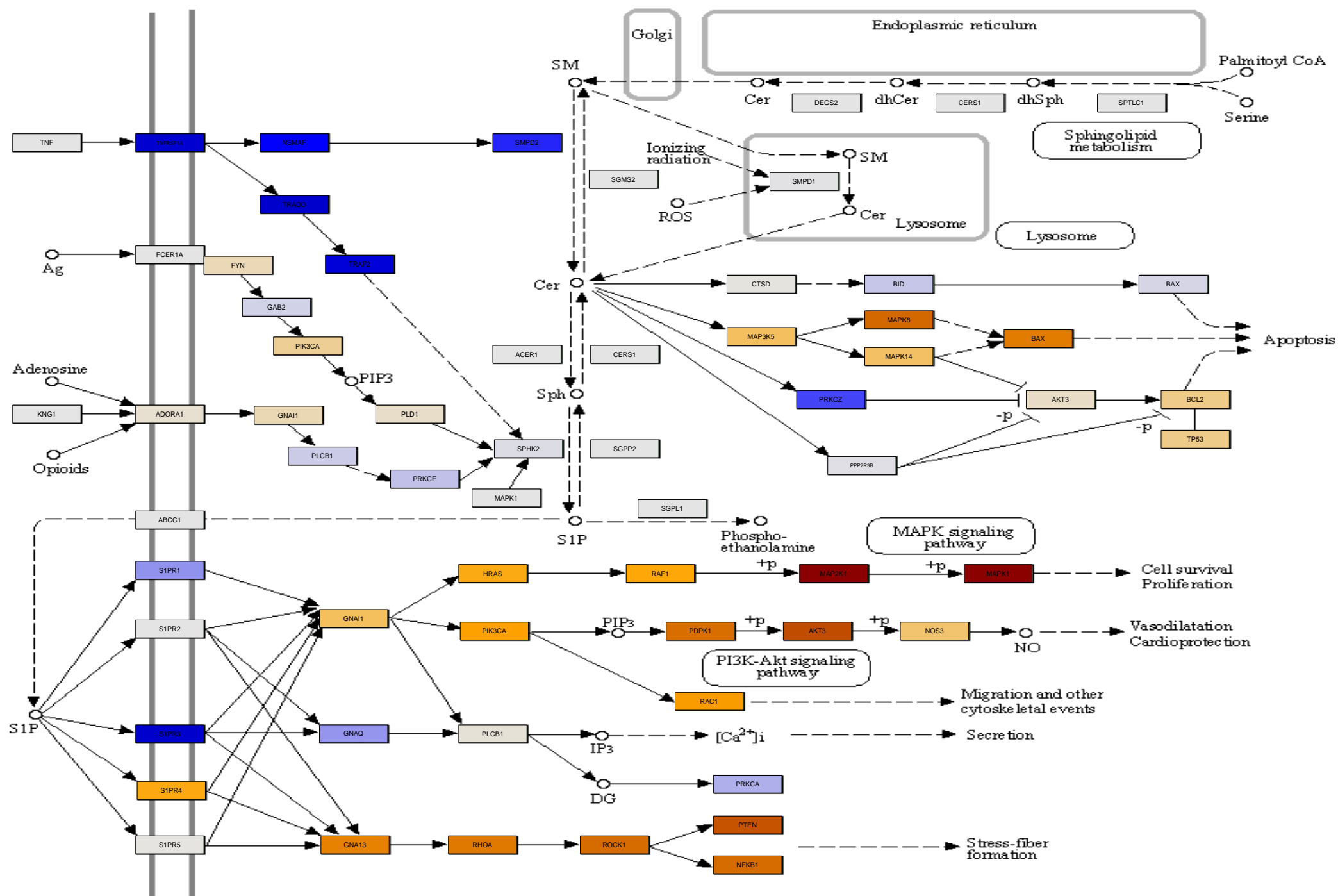

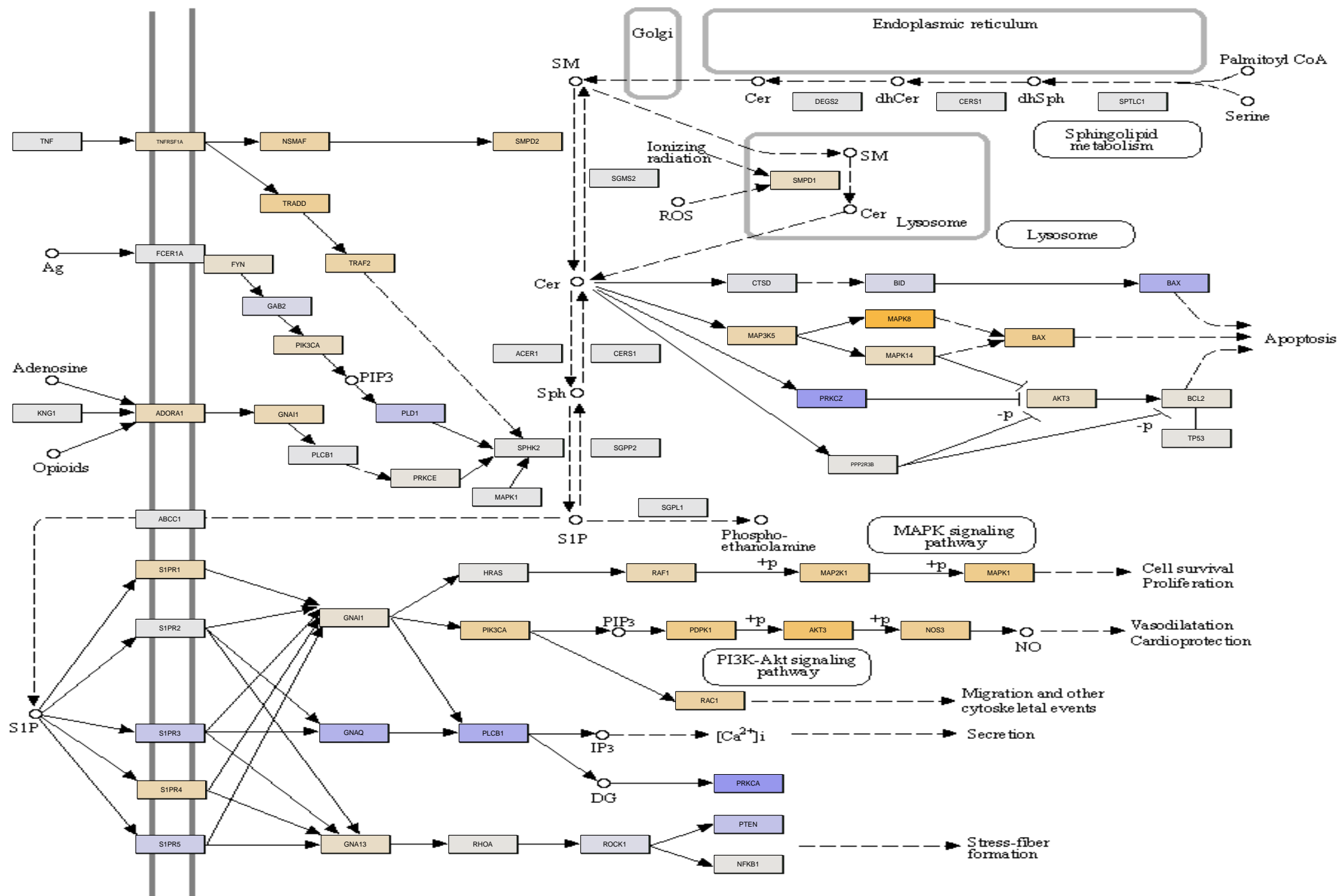

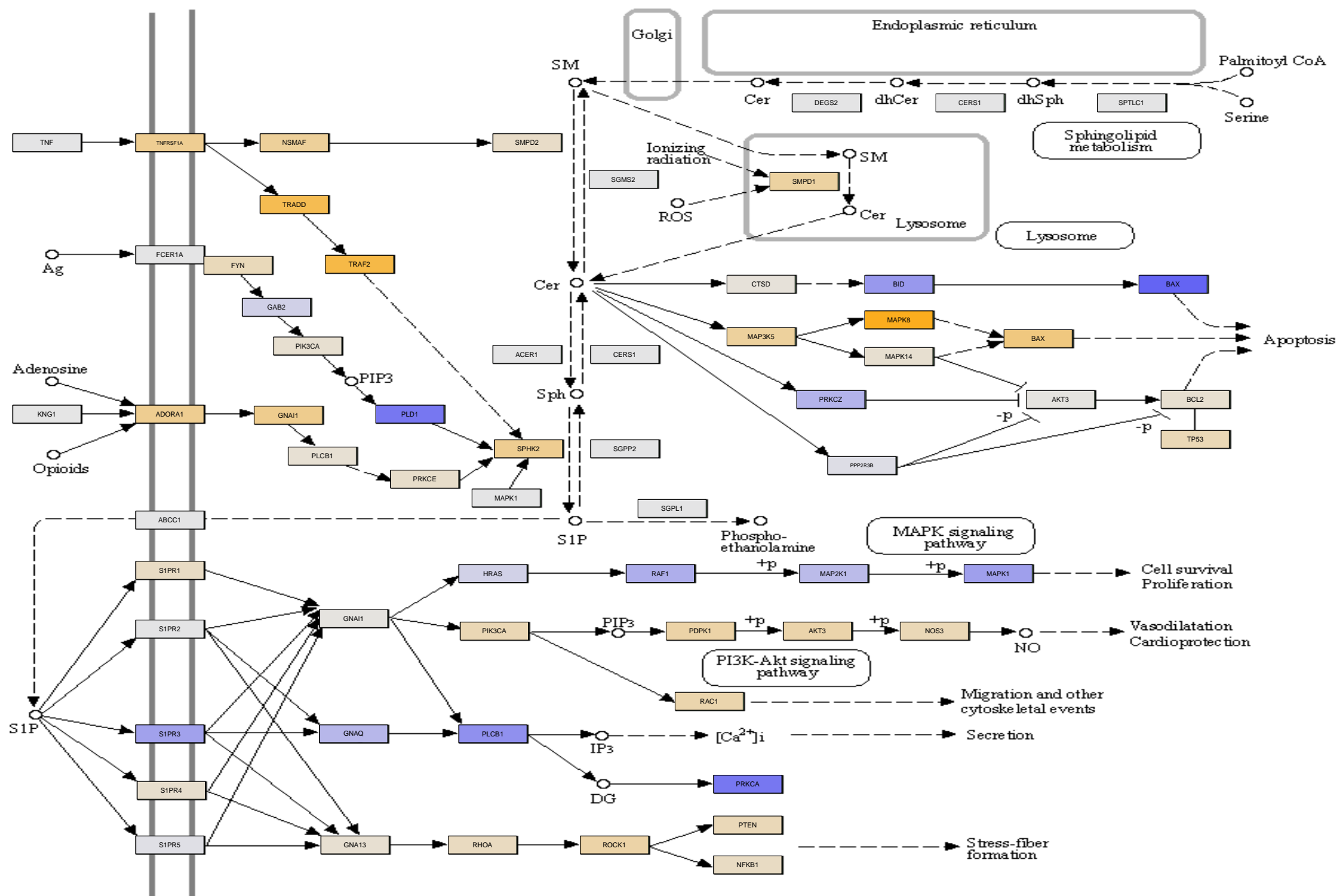

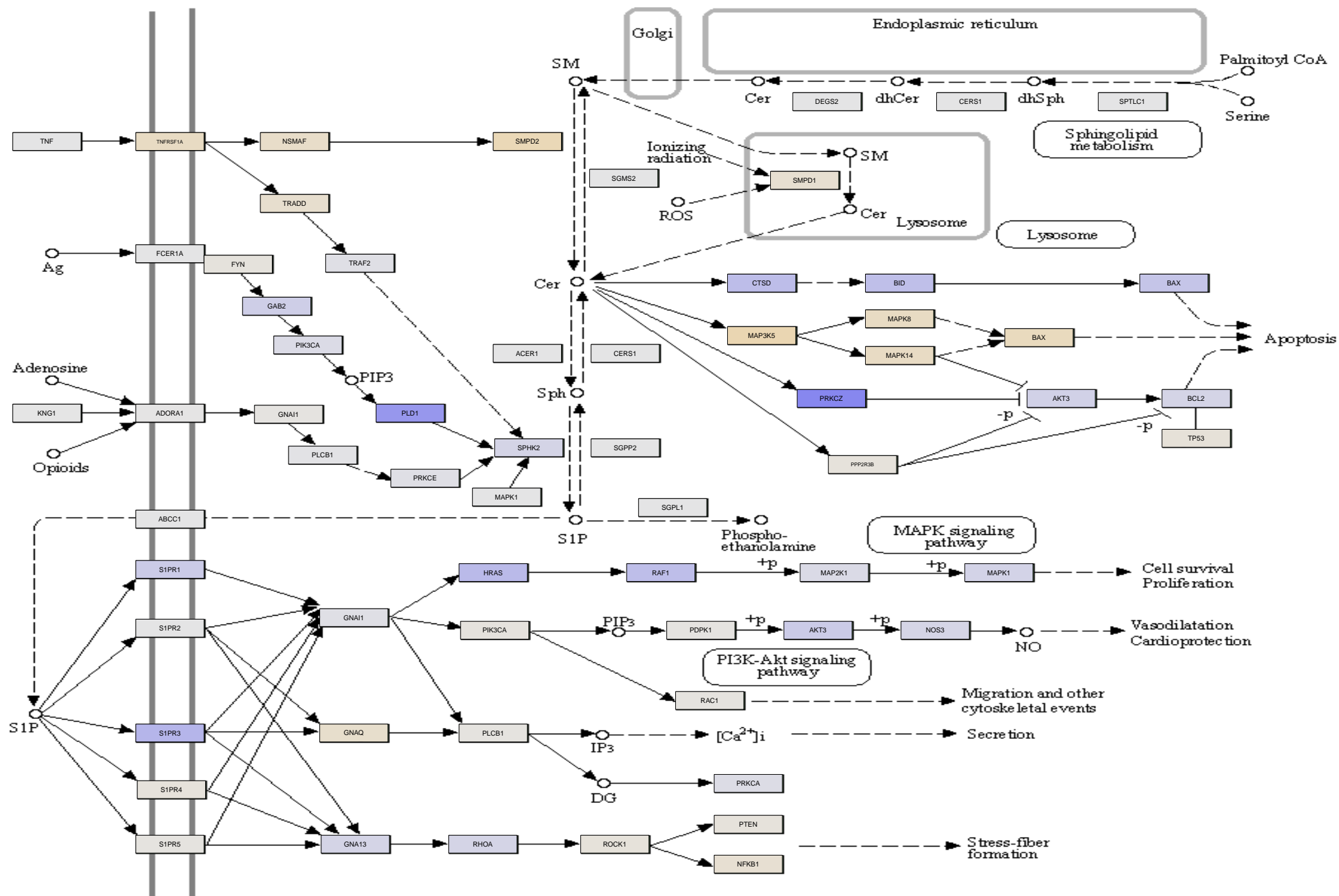

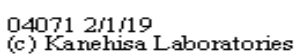

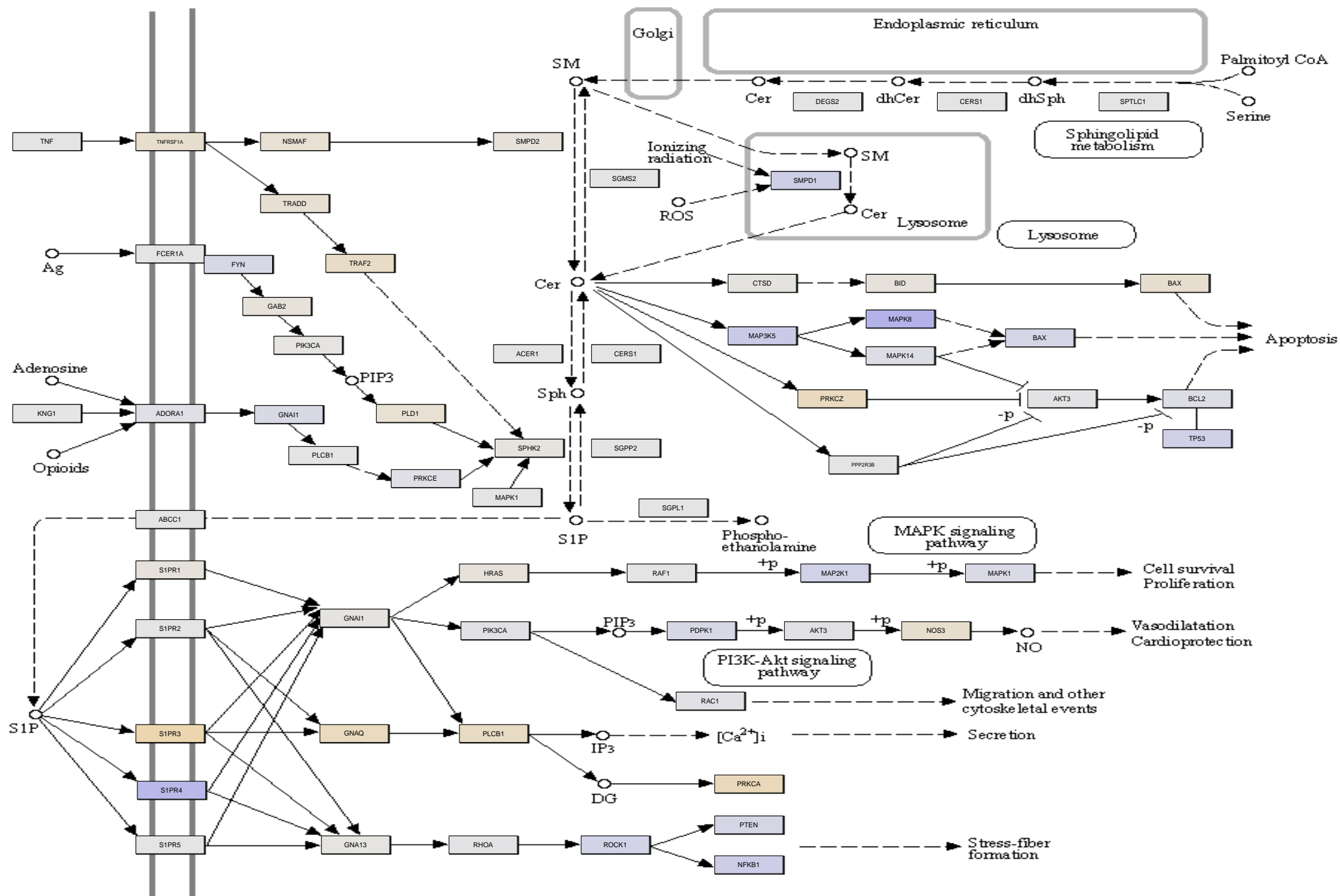

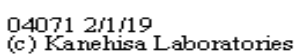

Supplement: Supplementary file 1 [file cells-11-00362-s001.zip › Suppl-Material-S4-Pathways-PSF_Methylation/Sphingolipid_signaling_pathway.pdf]

# Thyroid hormone signaling pathway

all genes

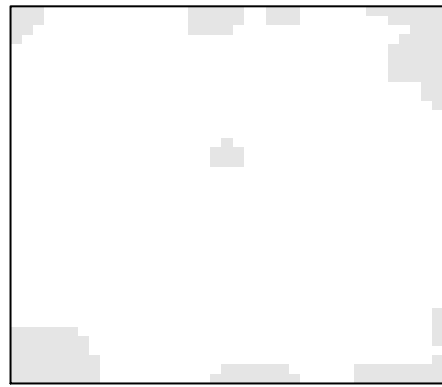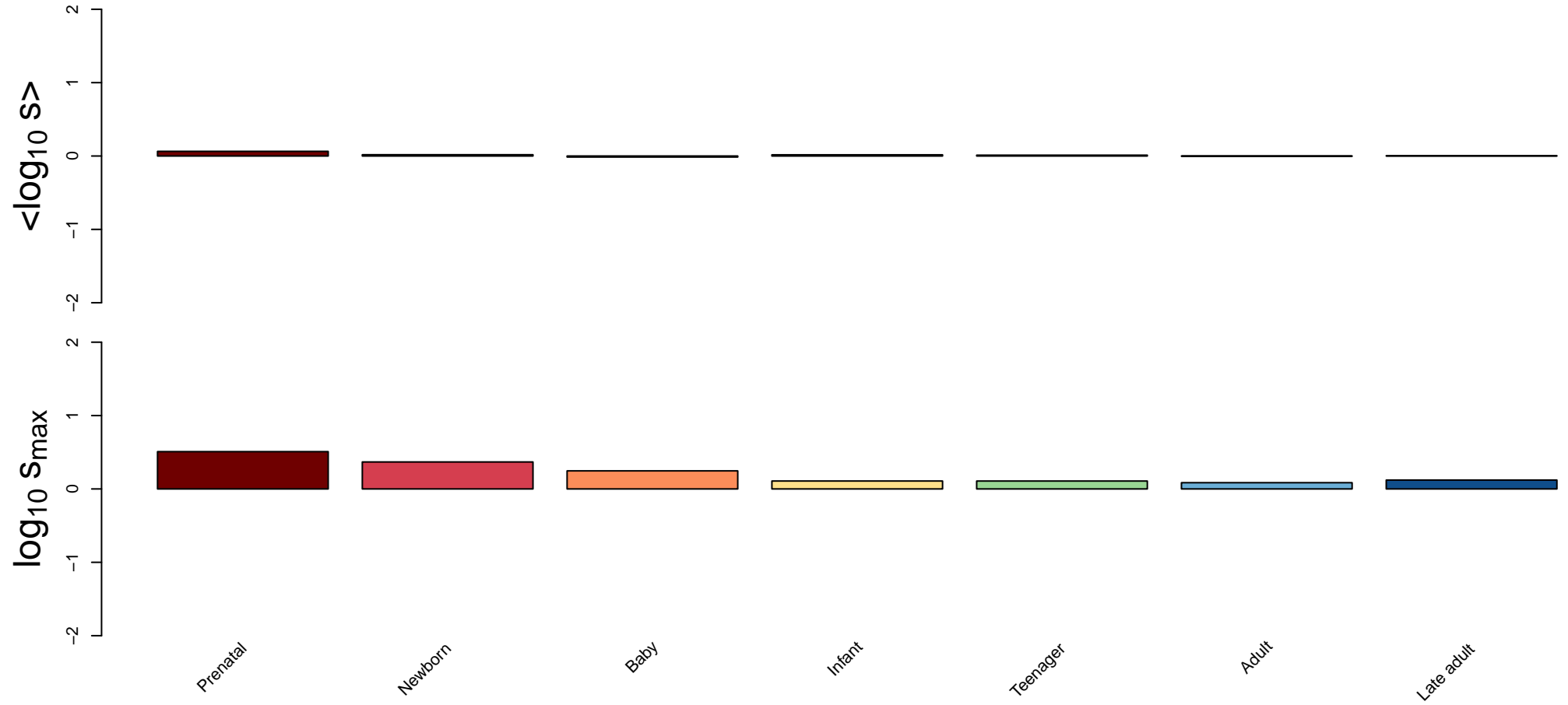



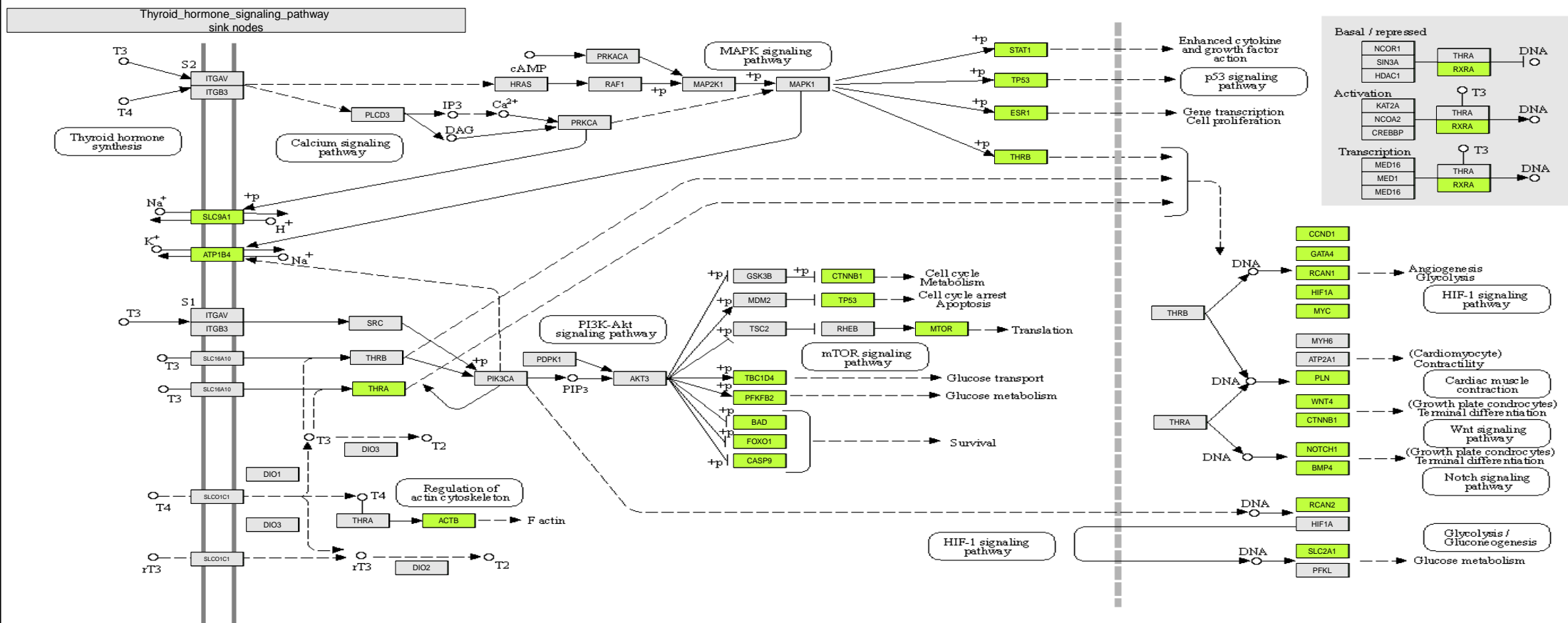

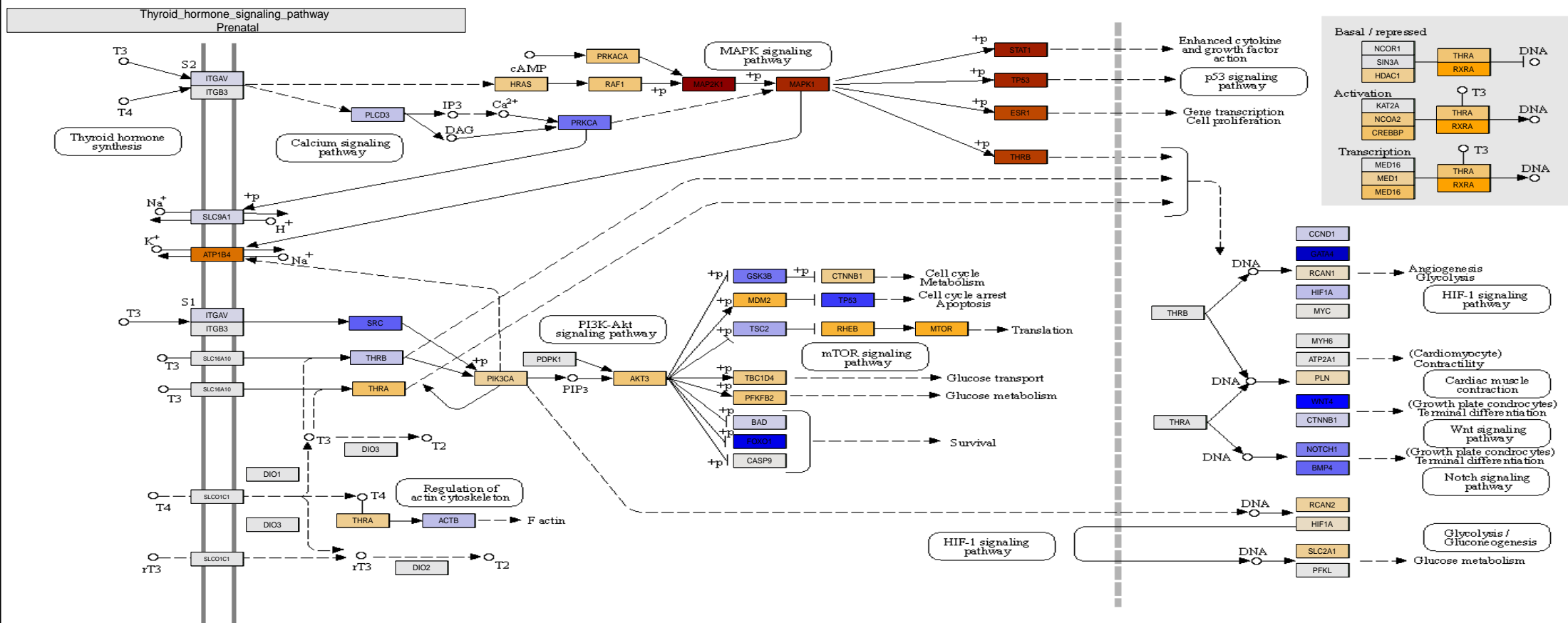

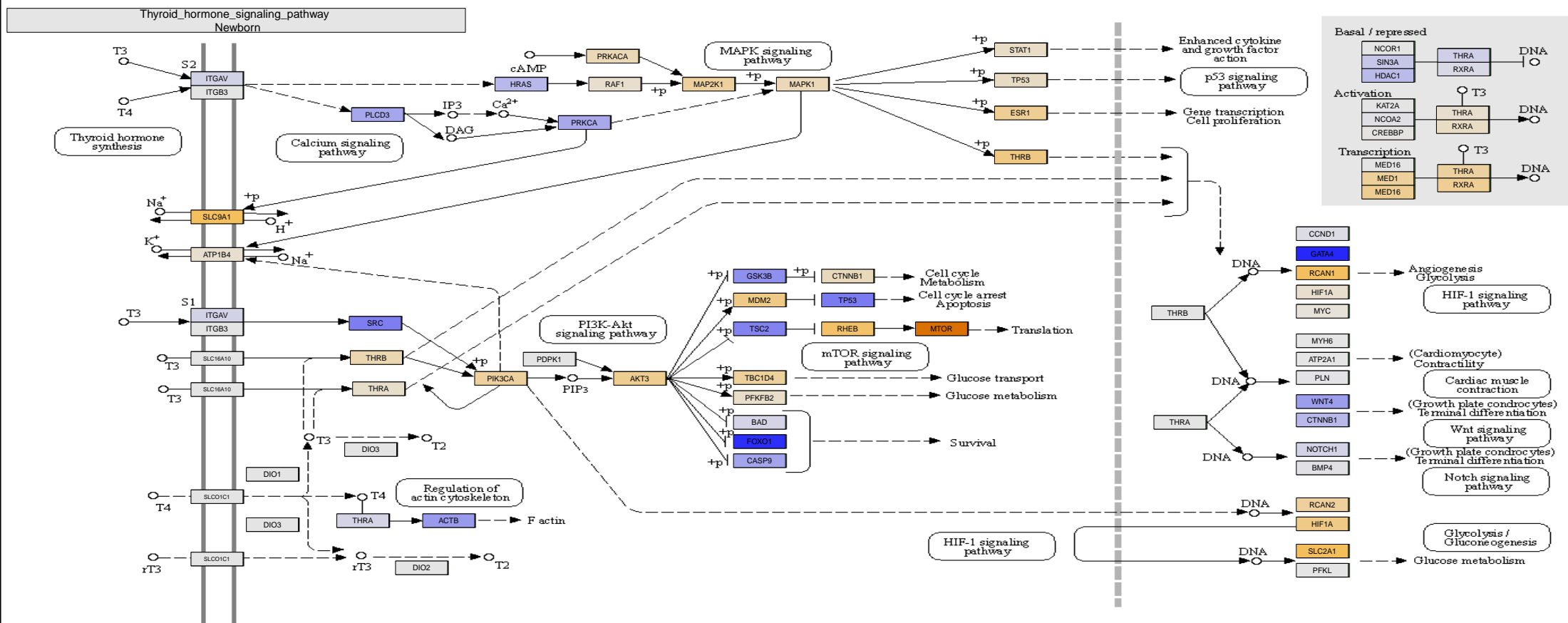

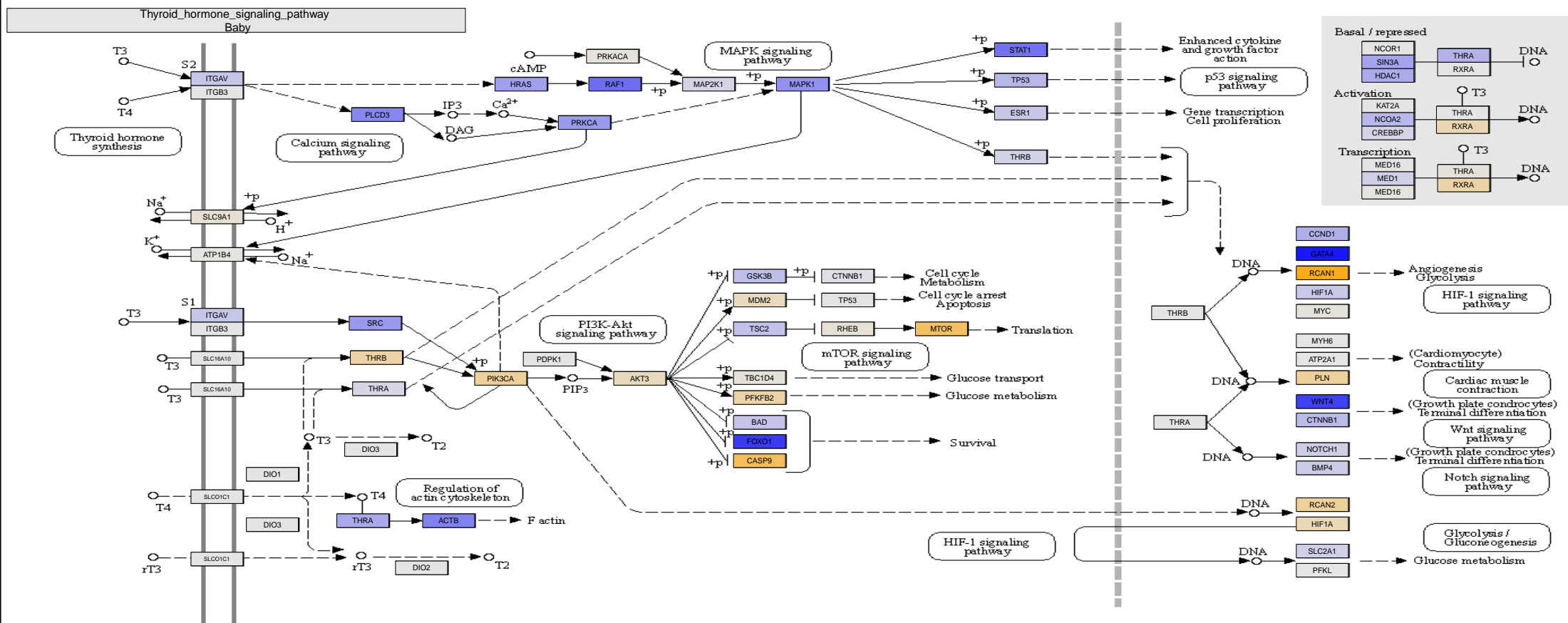

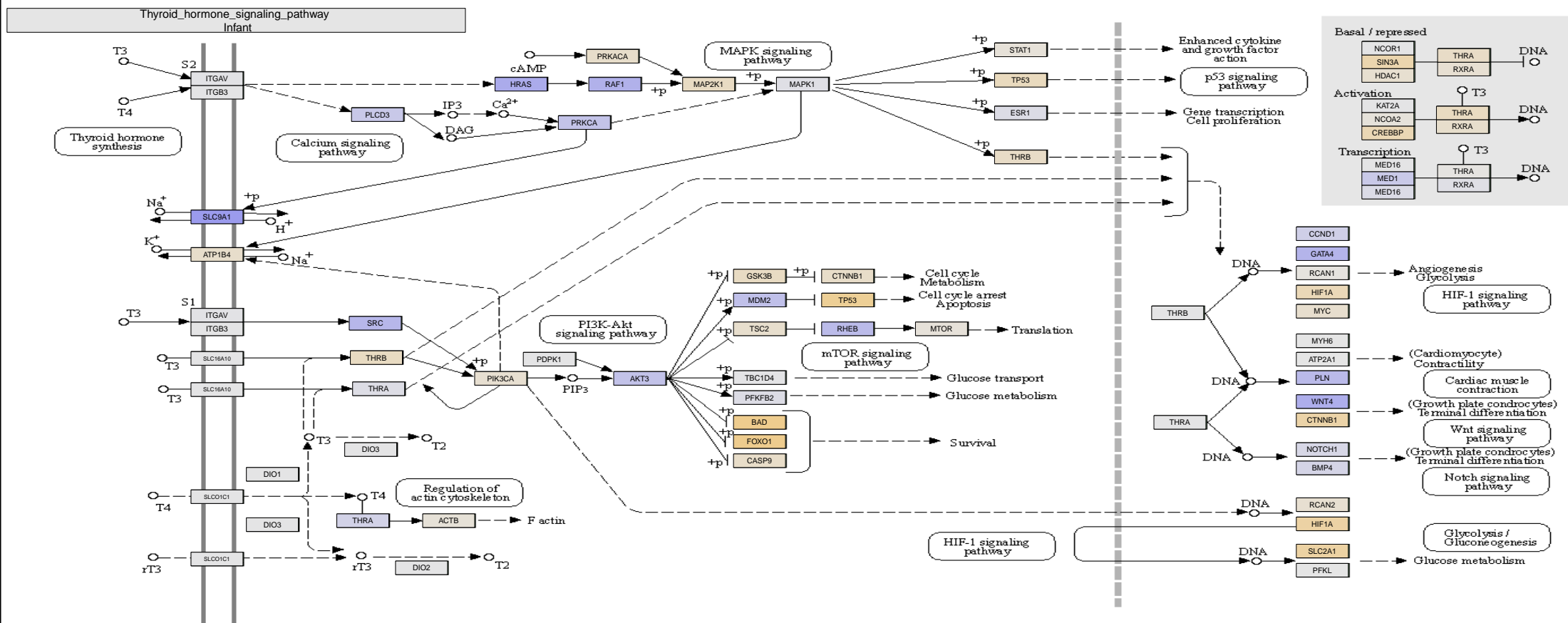

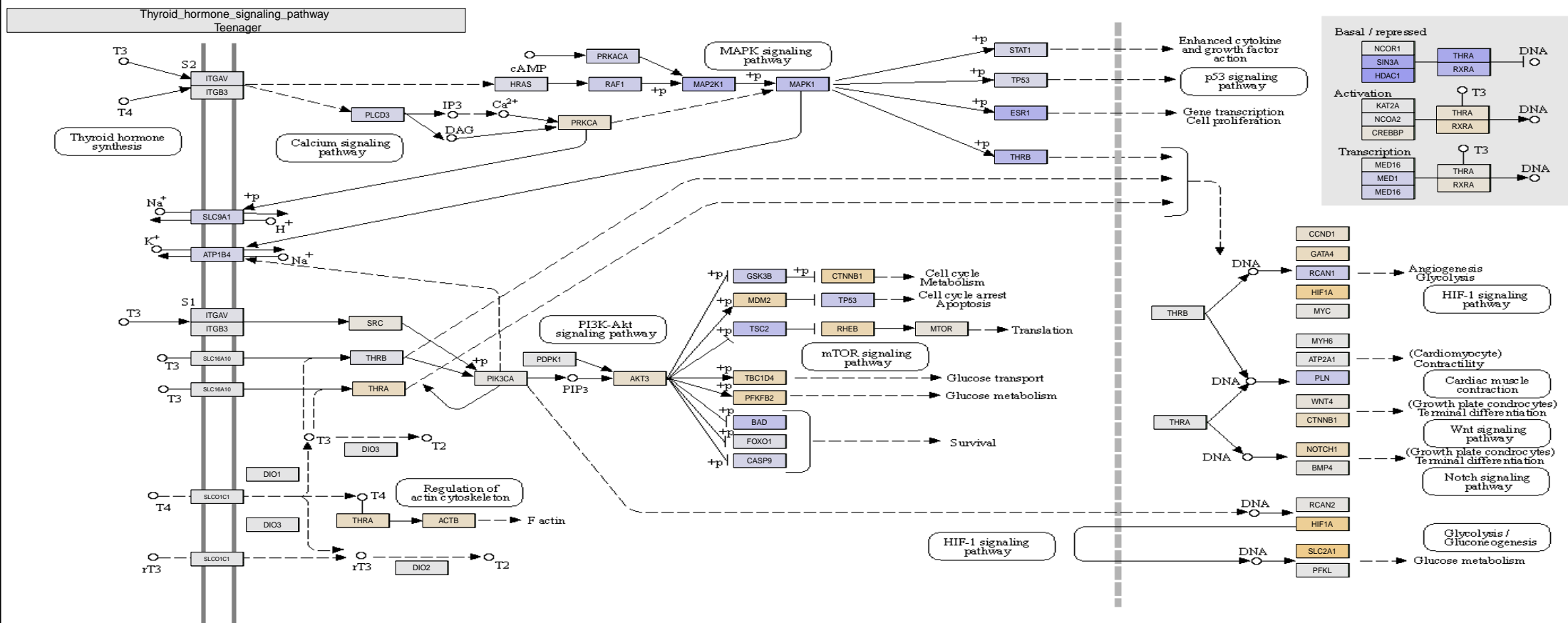

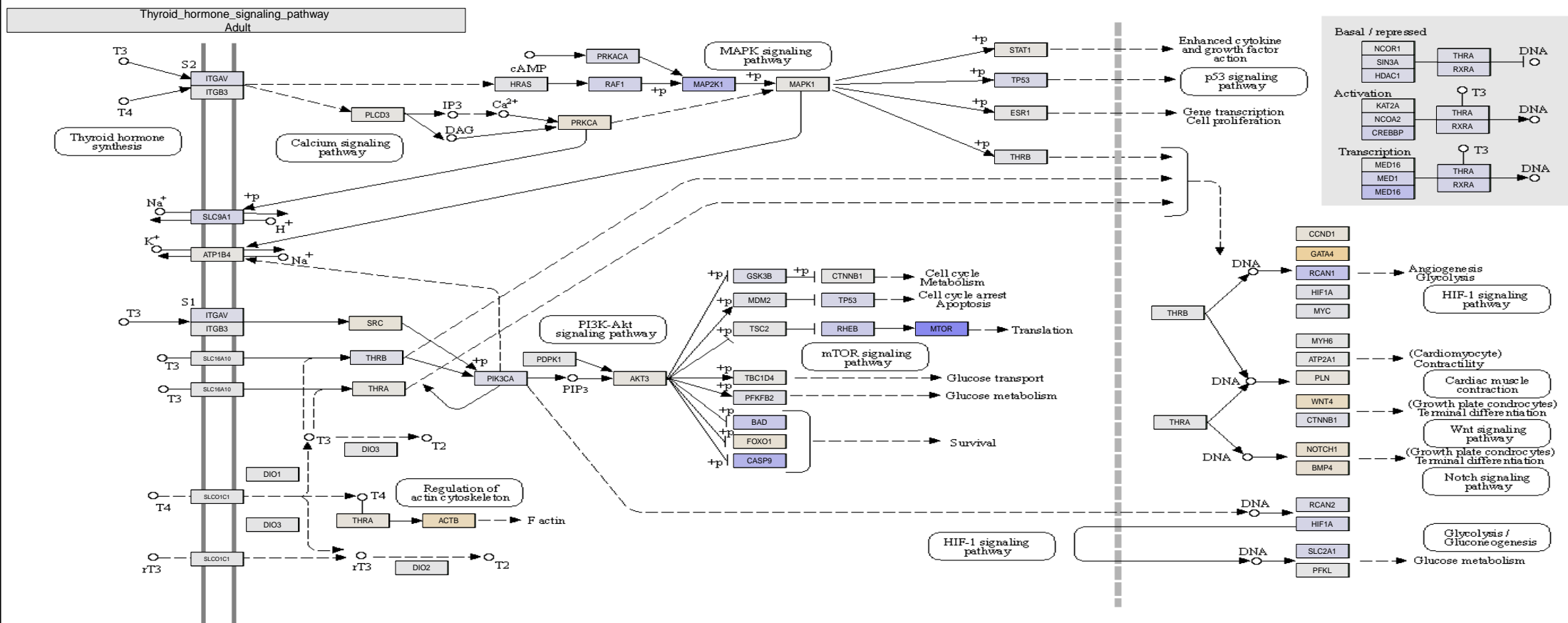

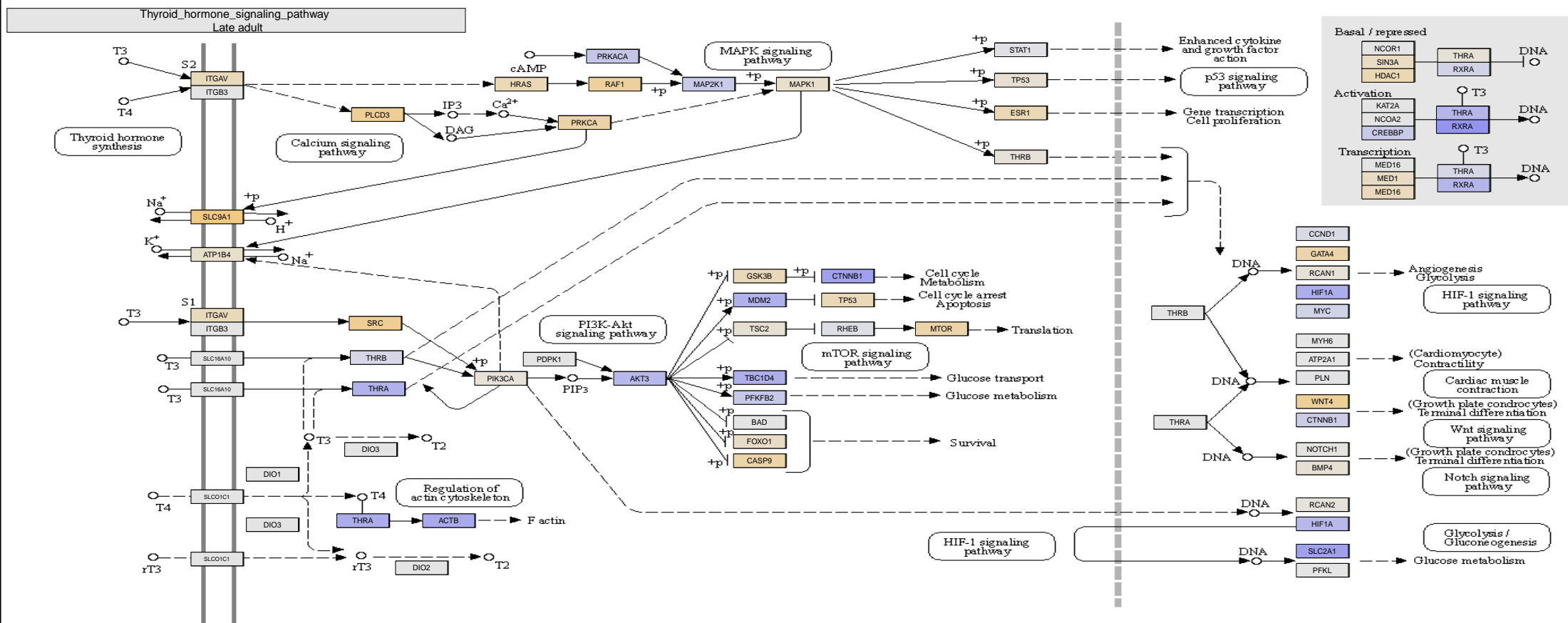

Supplement: Supplementary file 1 [file cells-11-00362-s001.zip › Suppl-Material-S4-Pathways-PSF_Methylation/Thyroid_hormone_signaling_pathway.pdf]

# Toll-like receptor signaling pathway

all genes

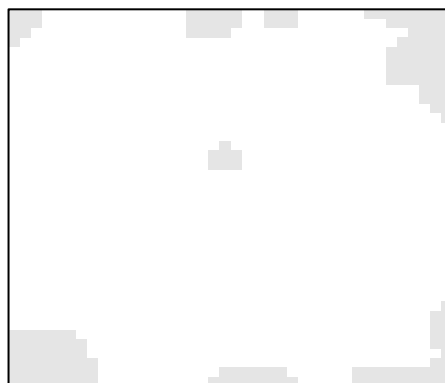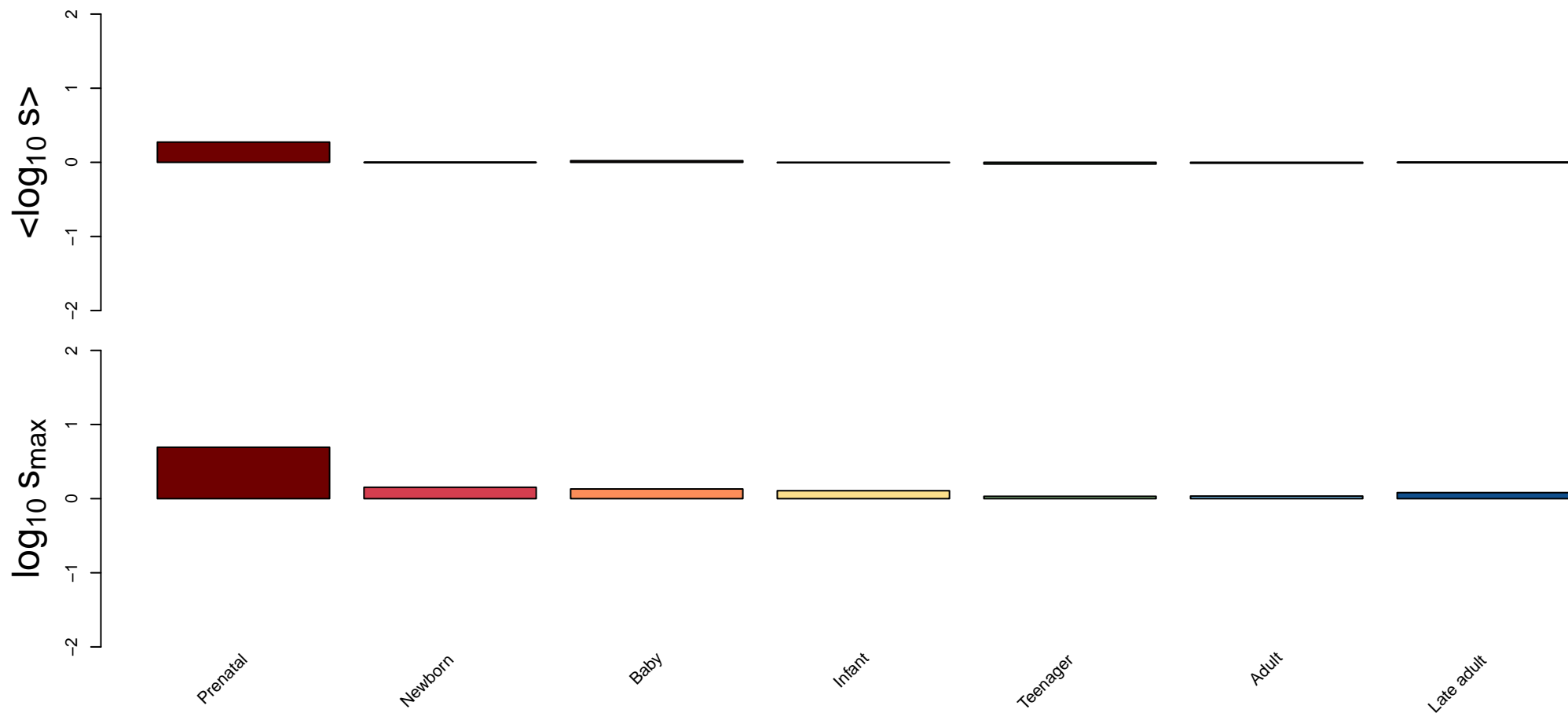



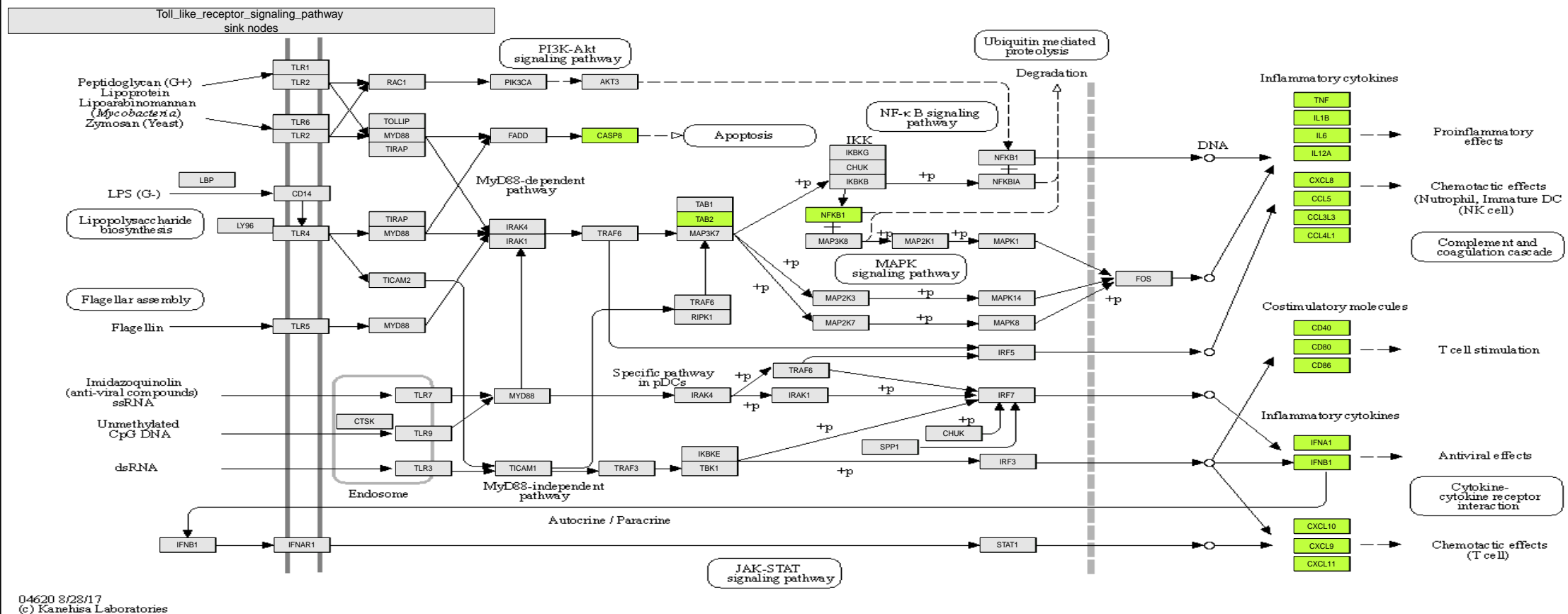

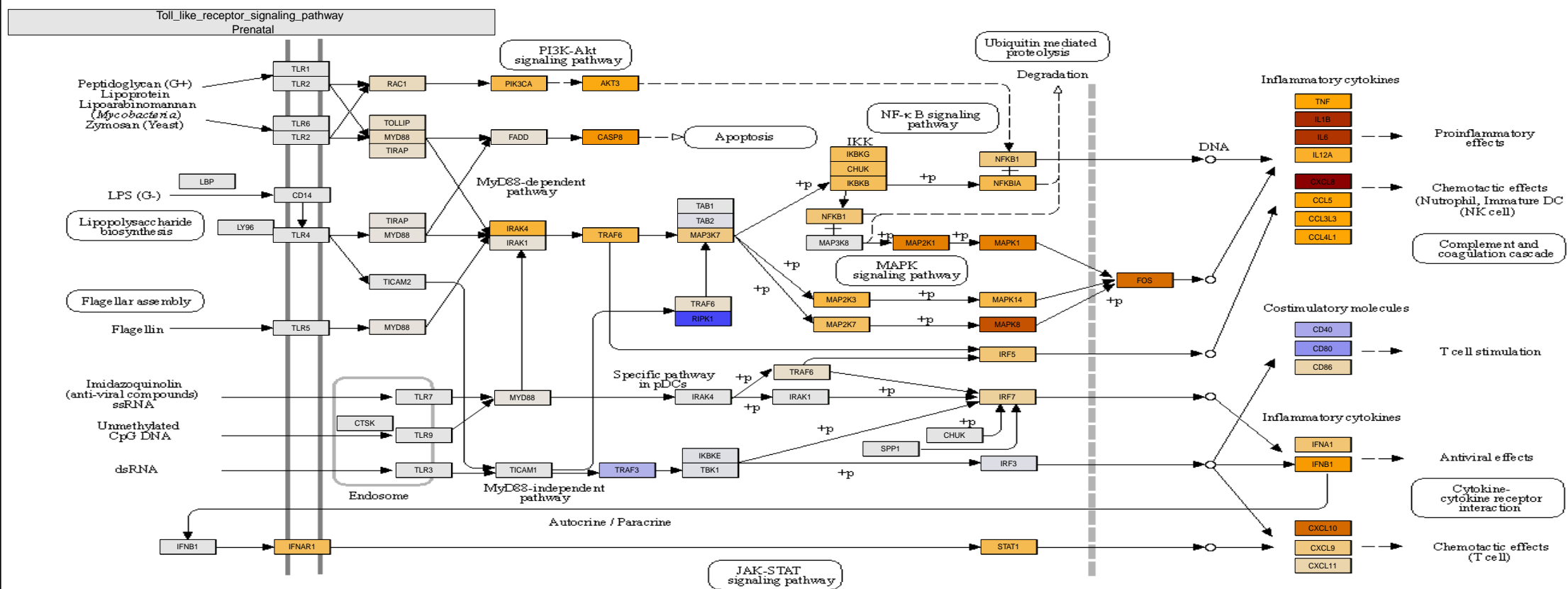

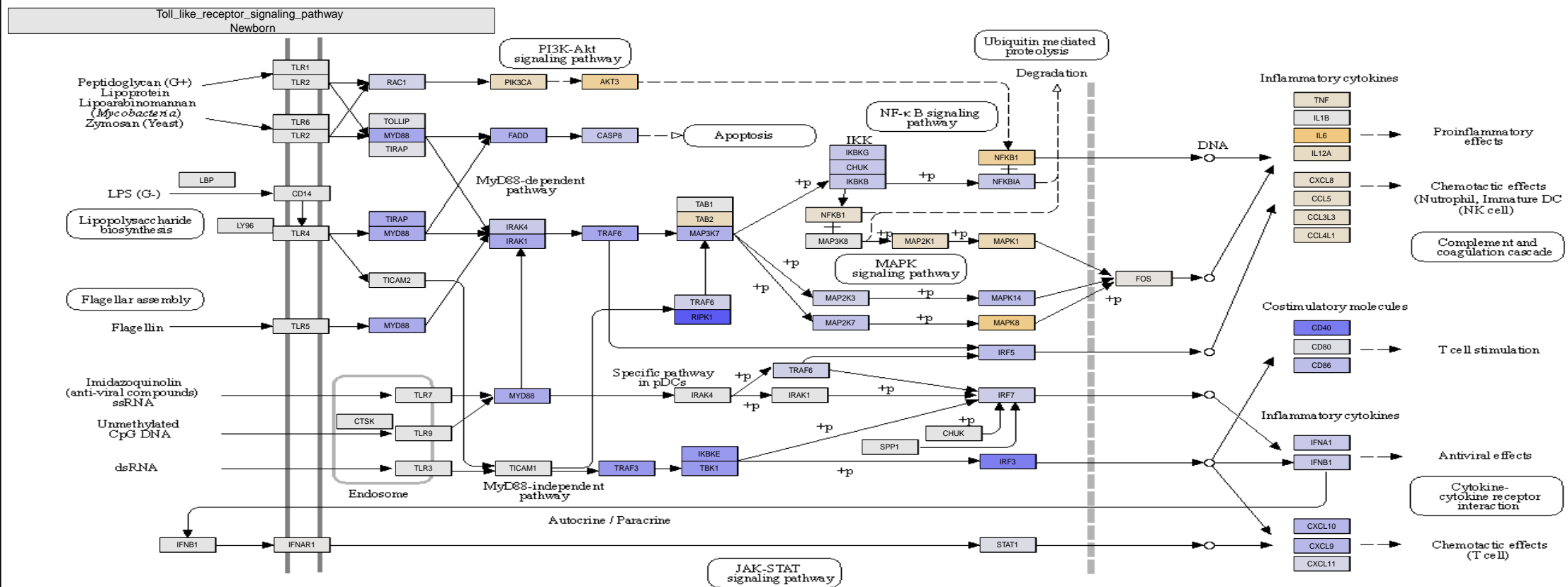

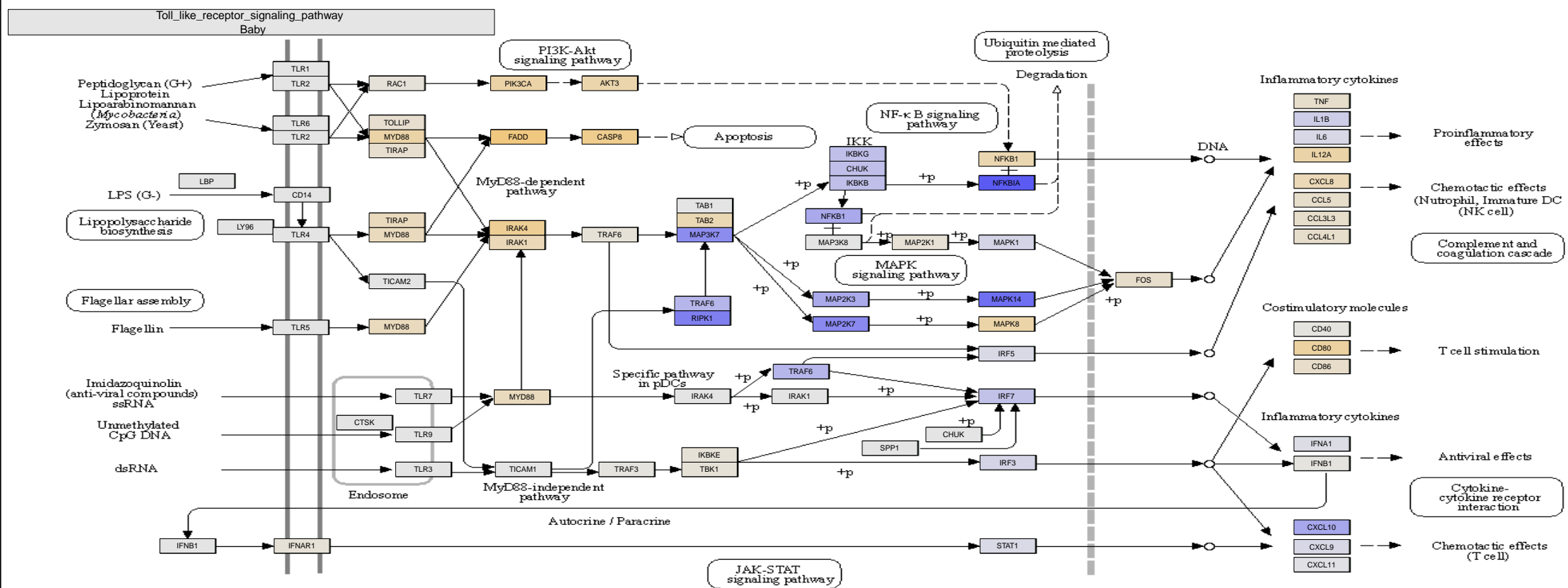

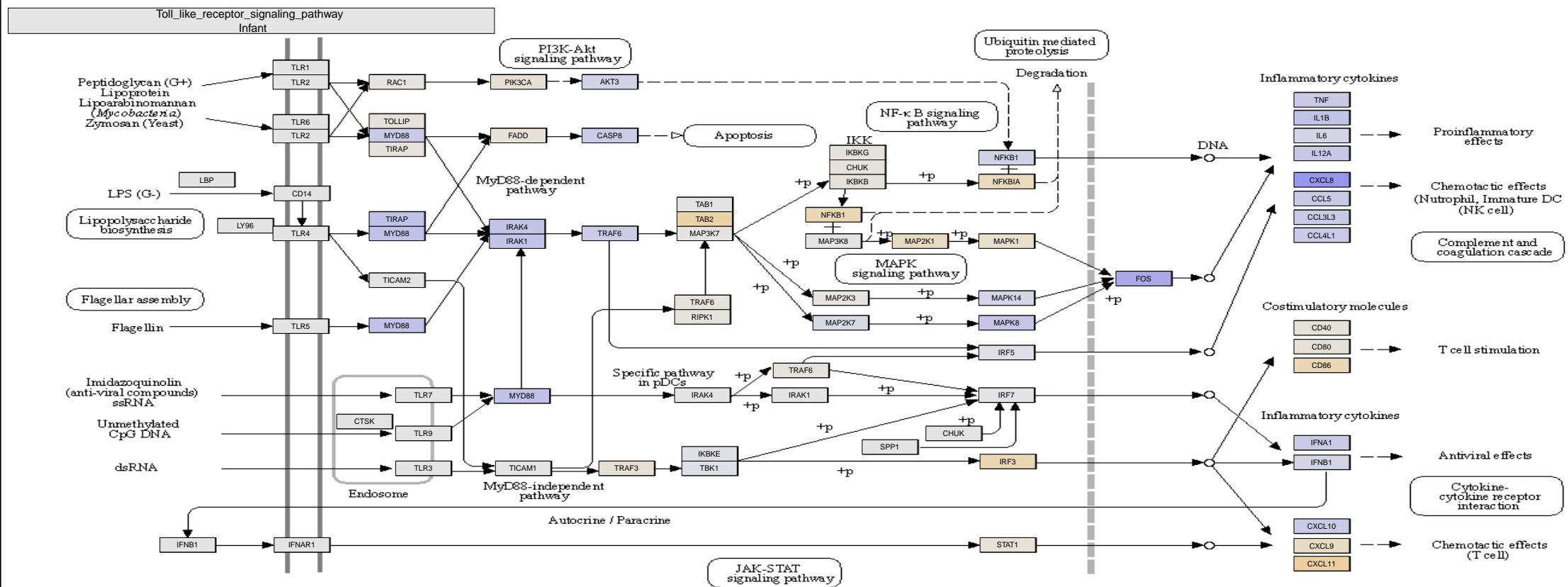

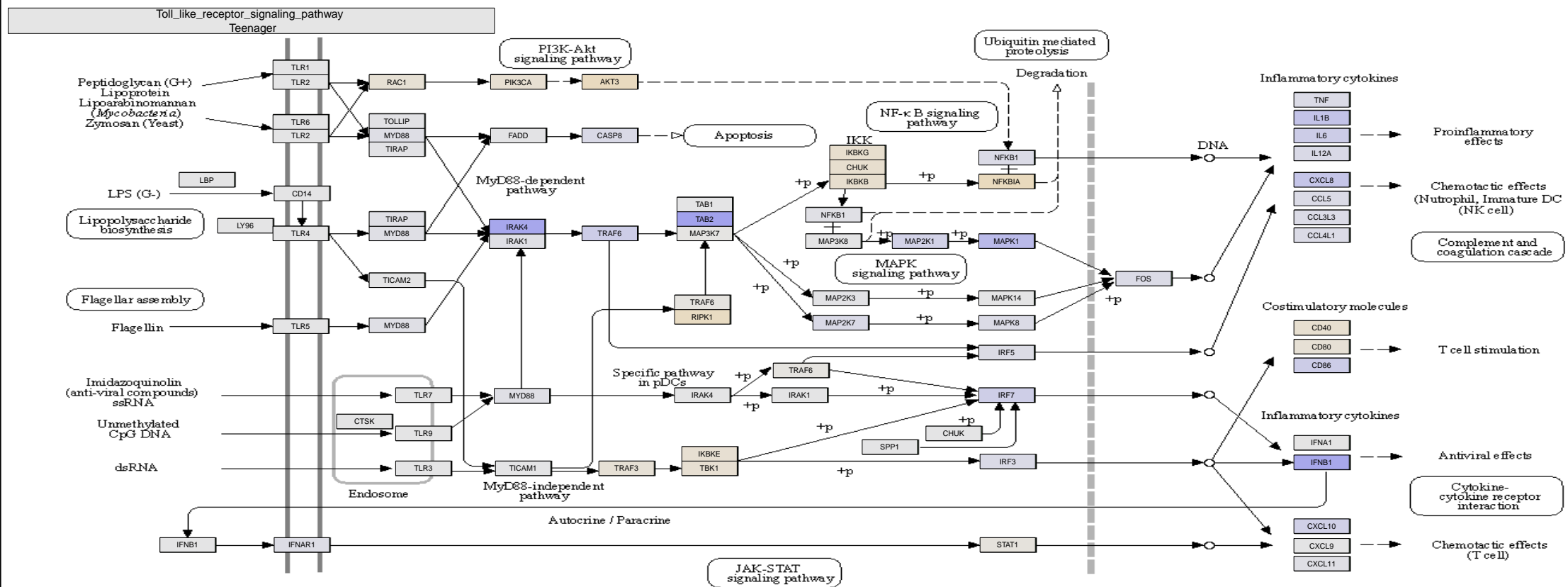

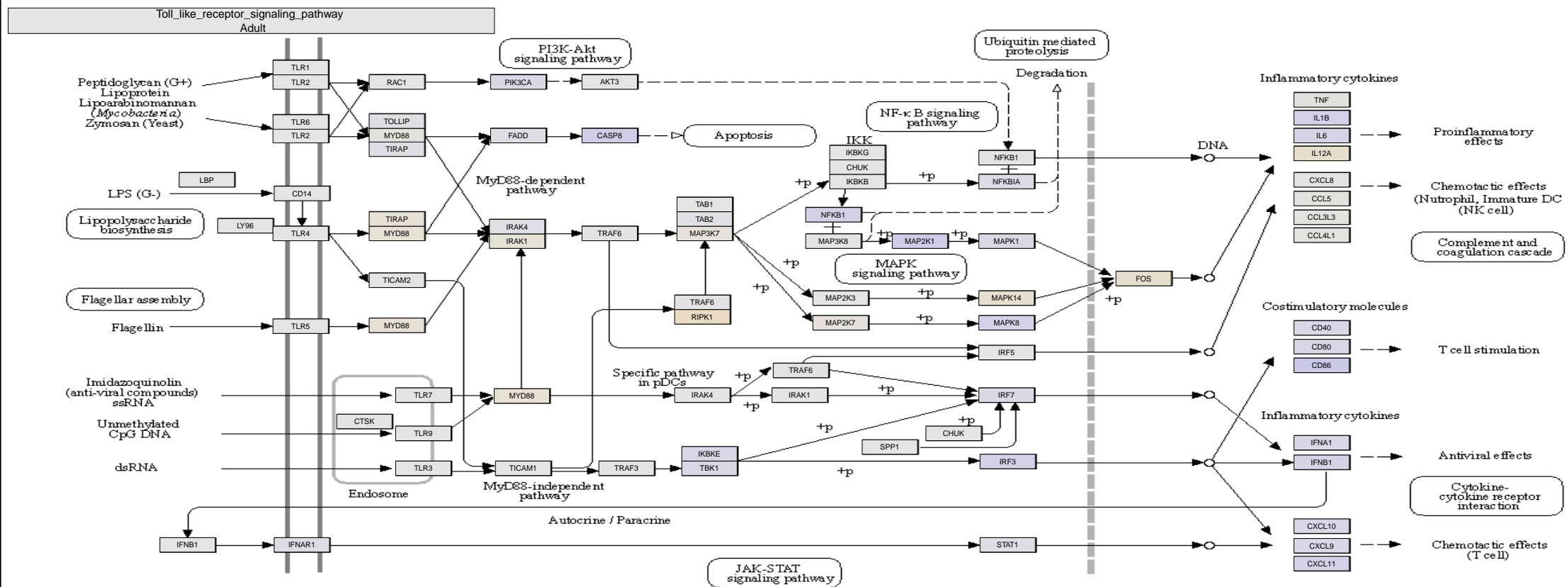

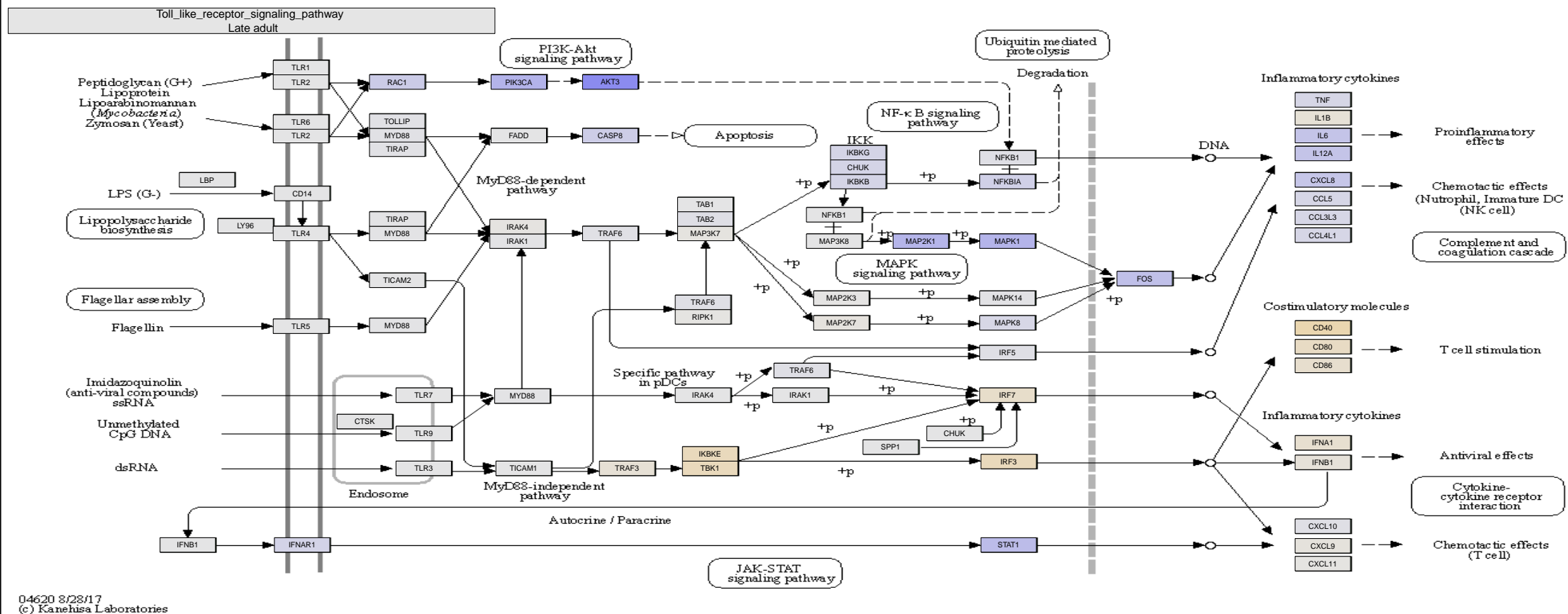

Supplement: Supplementary file 1 [file cells-11-00362-s001.zip › Suppl-Material-S4-Pathways-PSF_Methylation/Toll_like_receptor_signaling_pathway.pdf]

Wnt signaling pathway

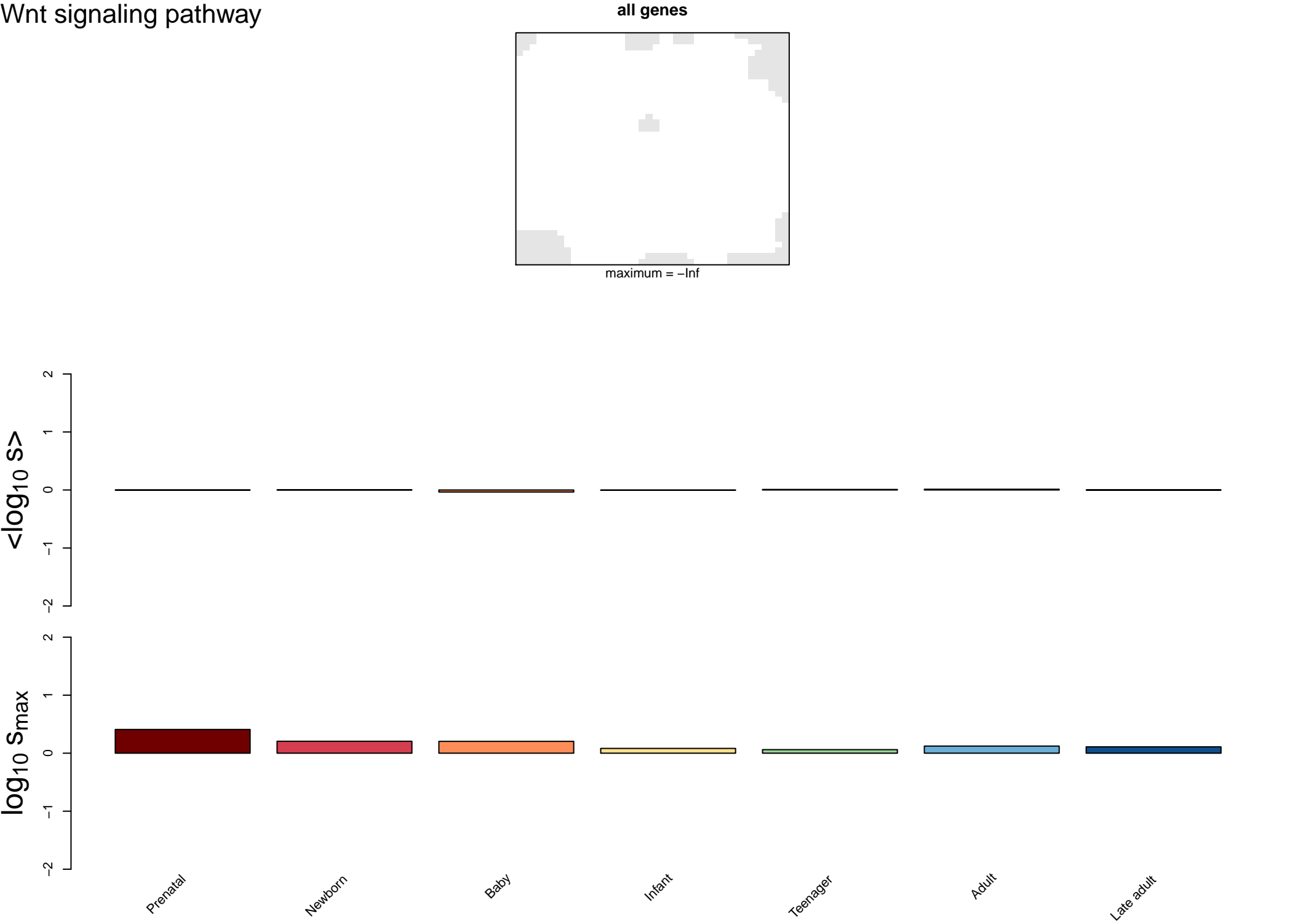

Wnt\_signaling\_pathway  
genes with data

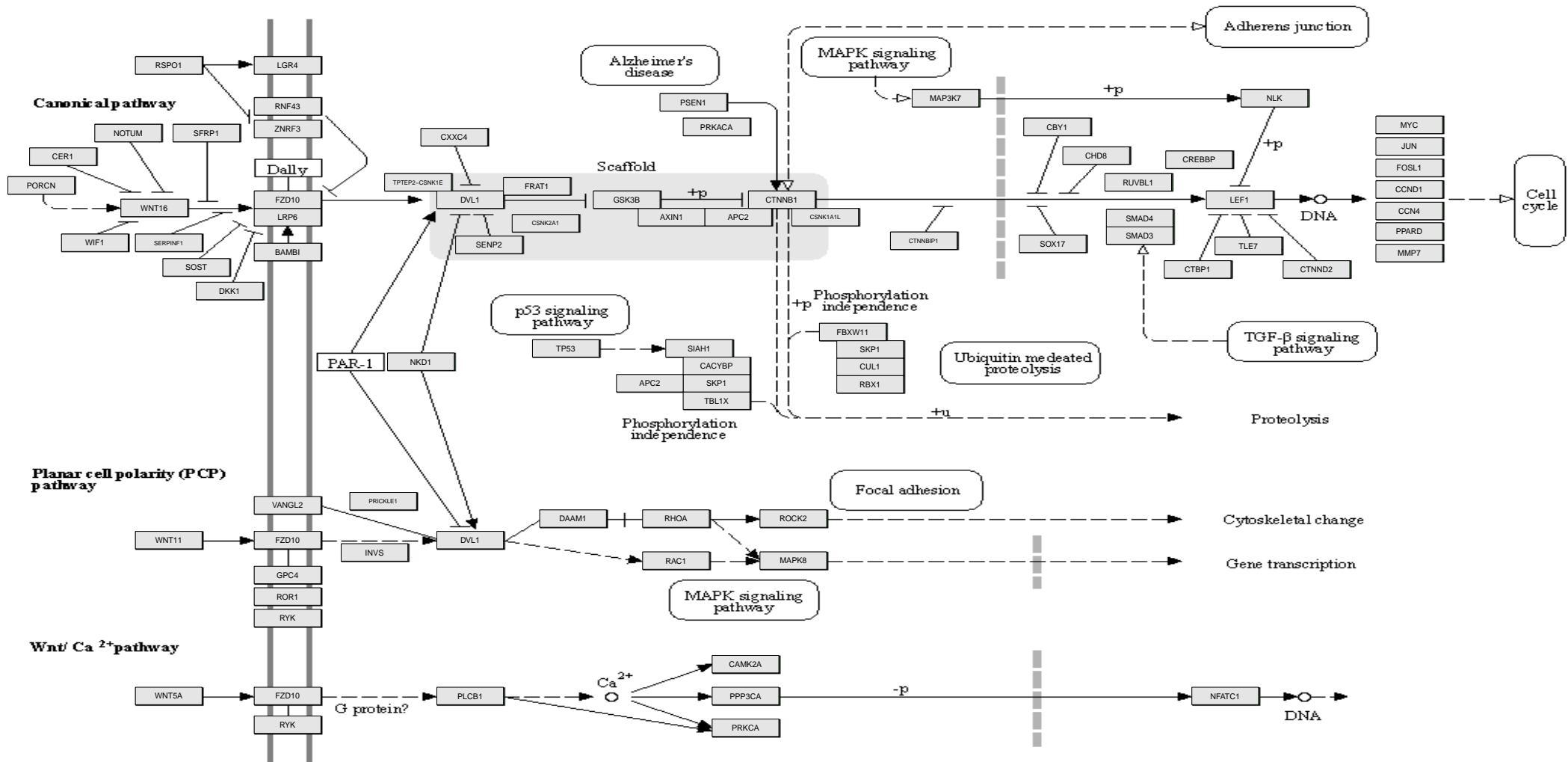

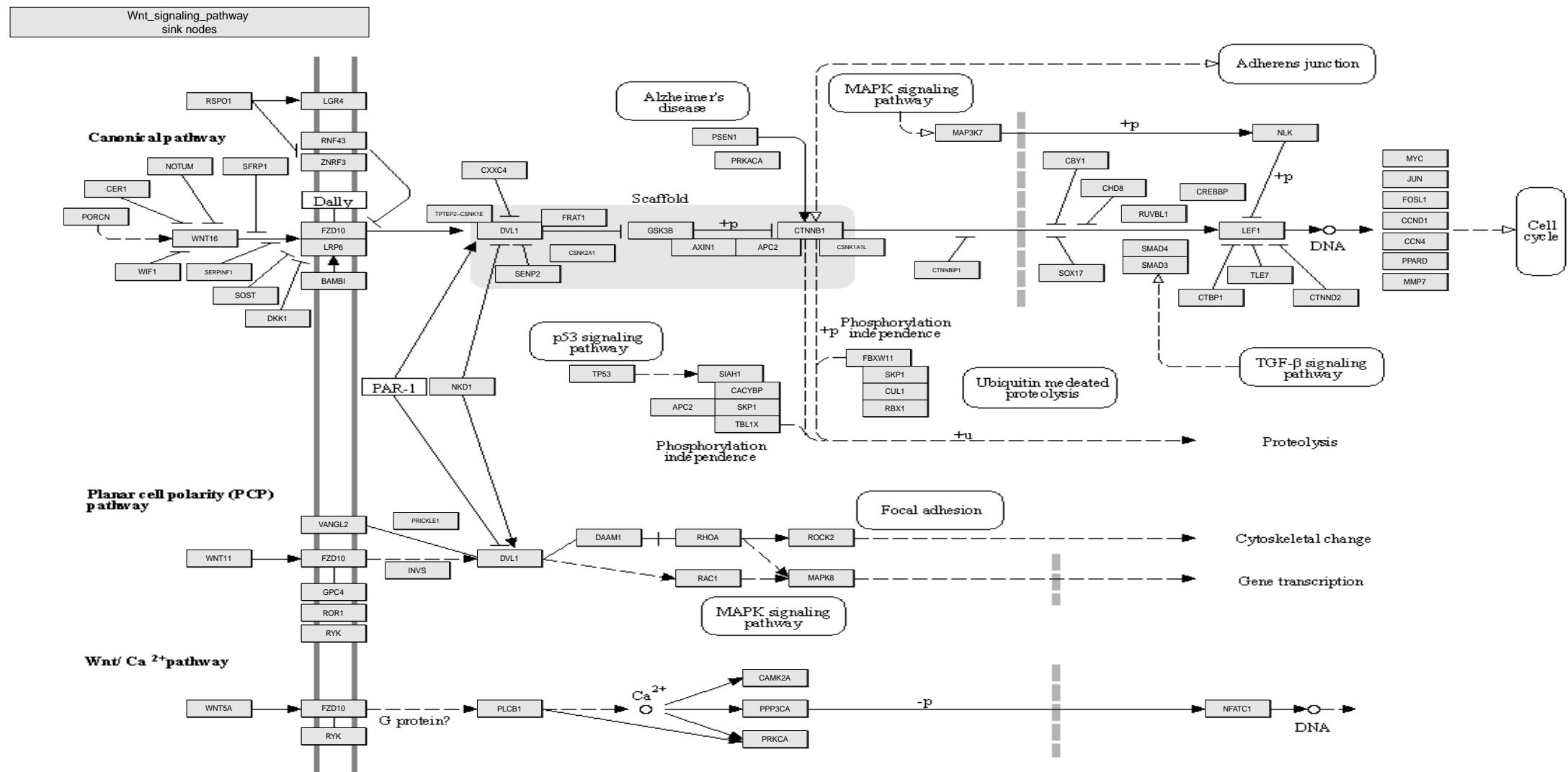

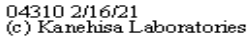

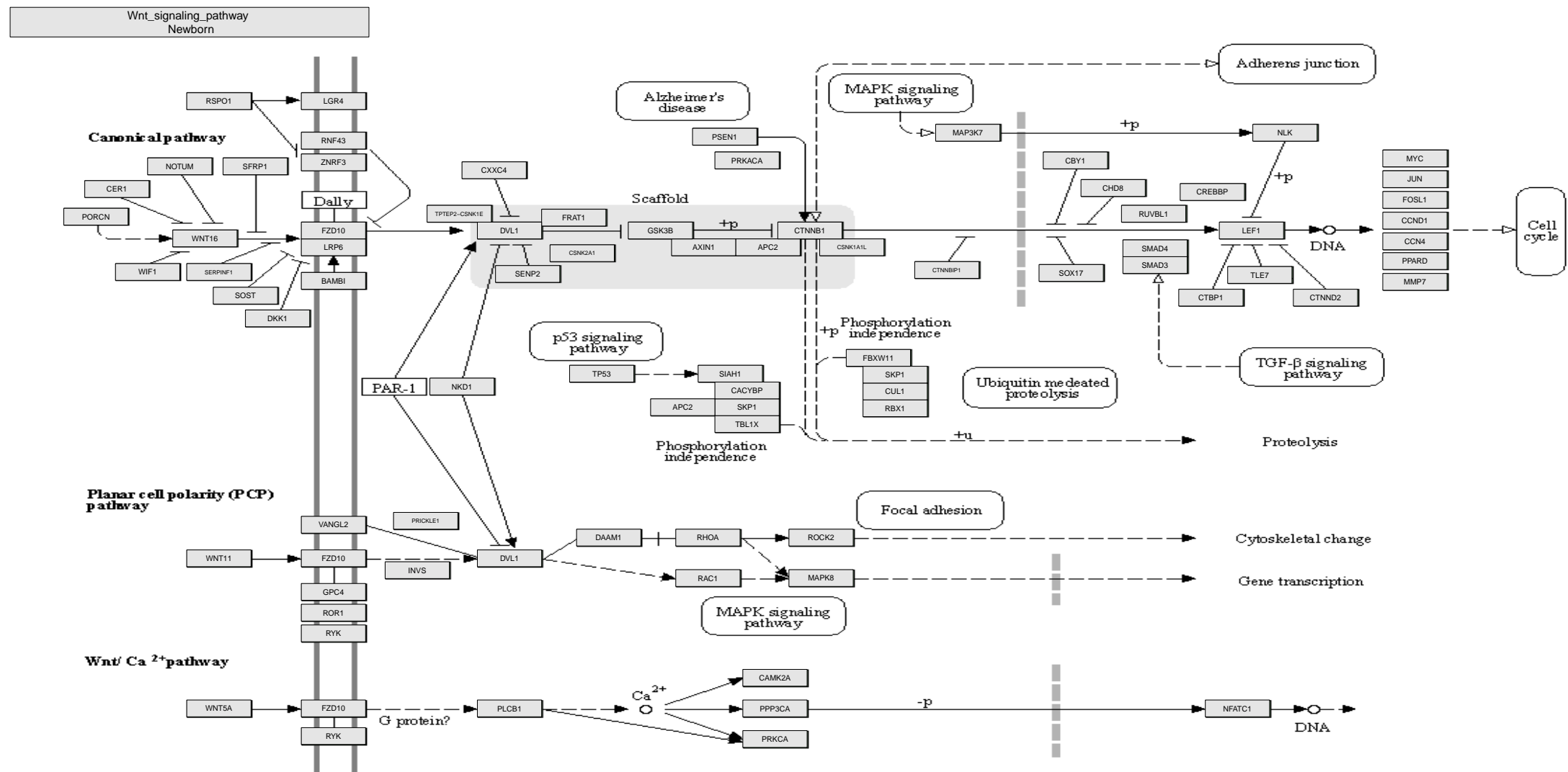

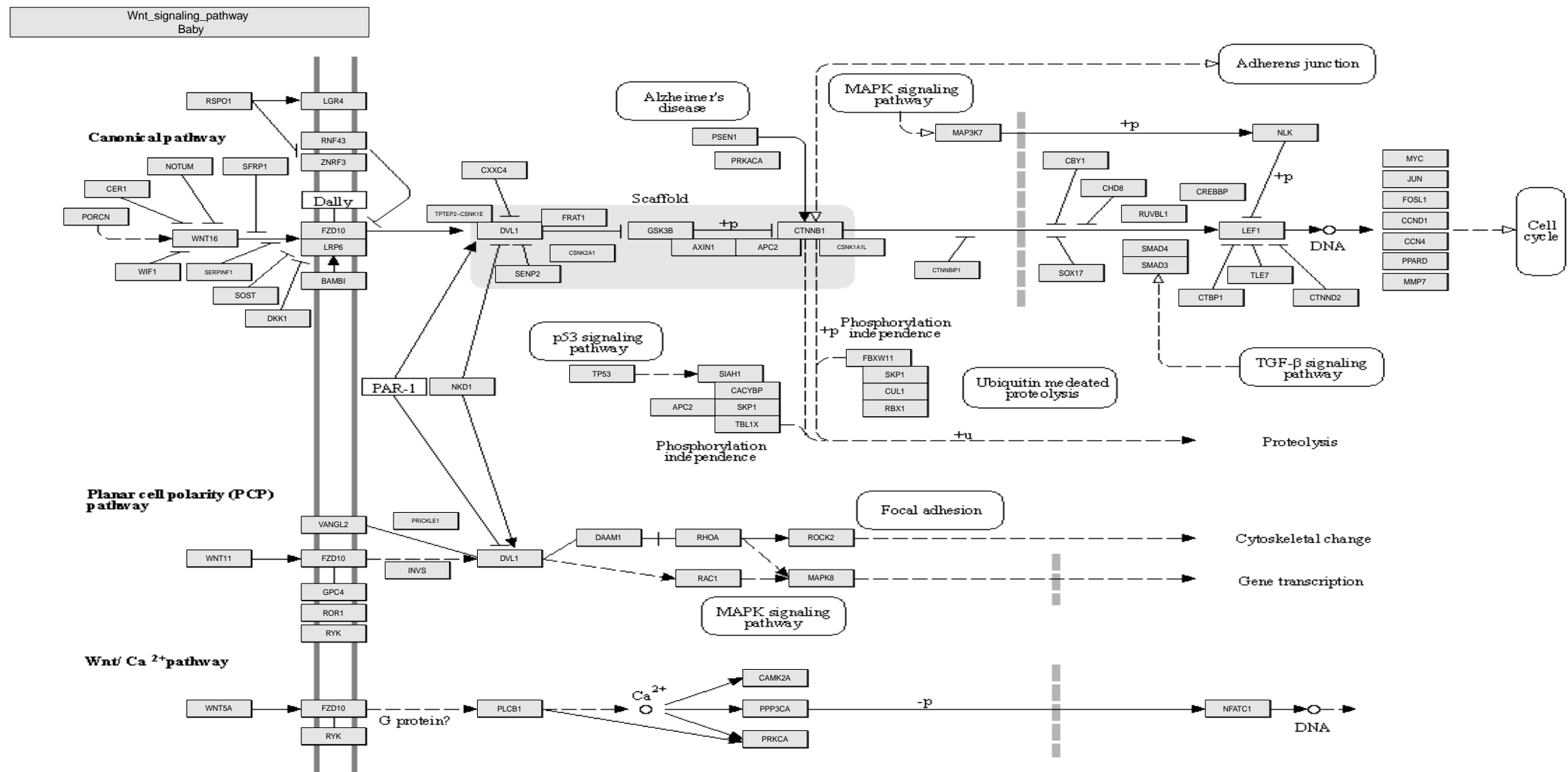

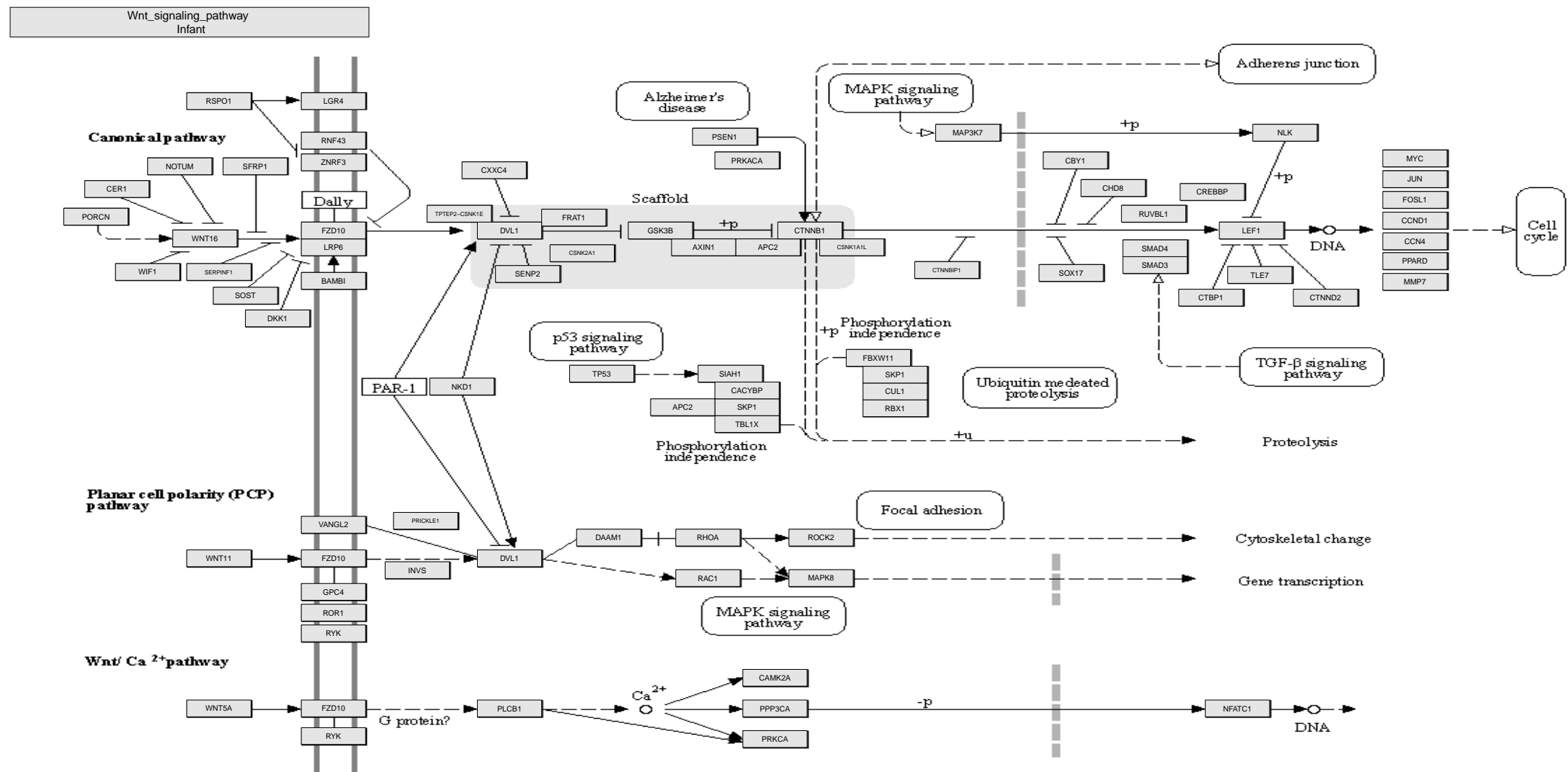



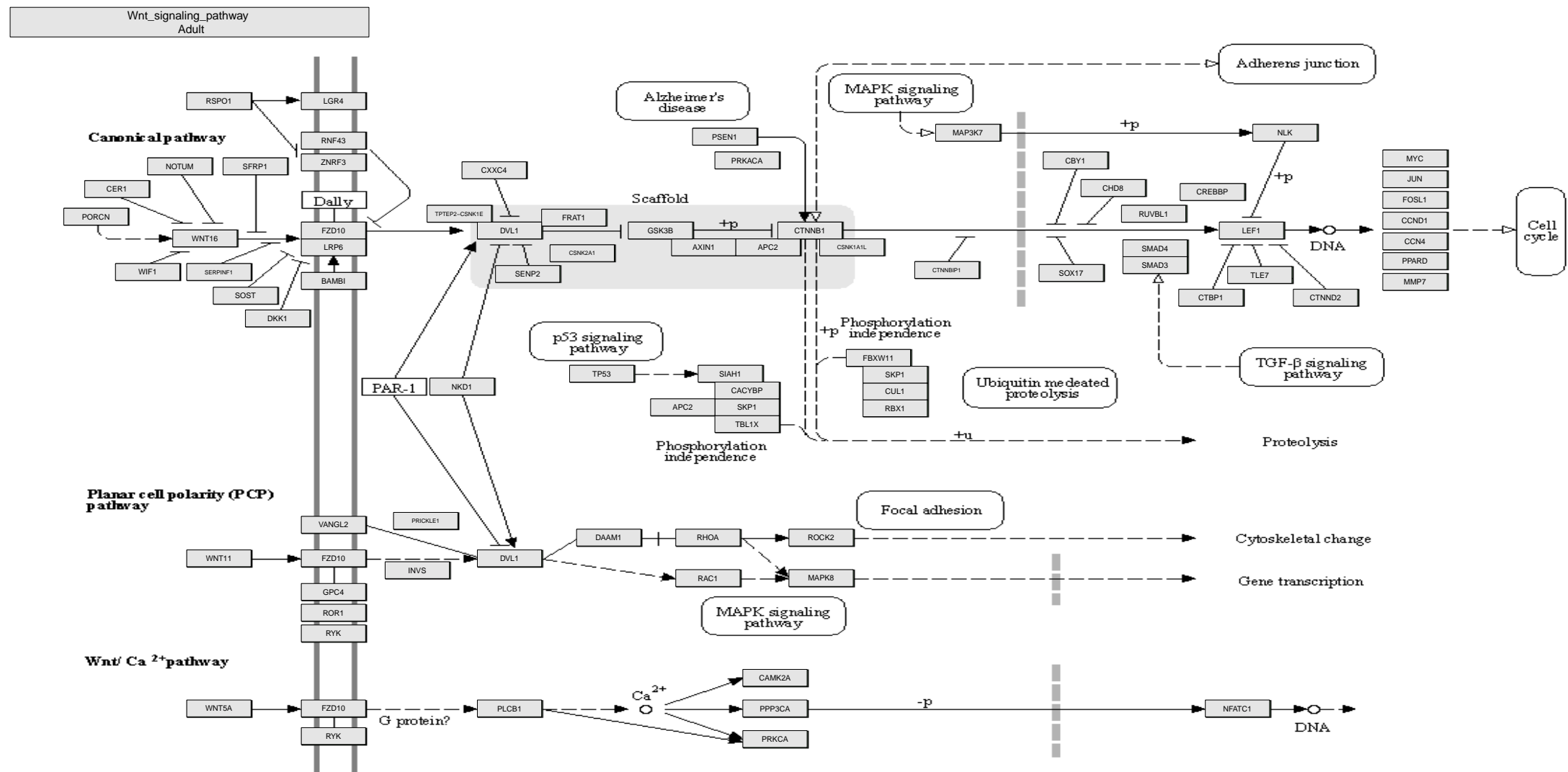

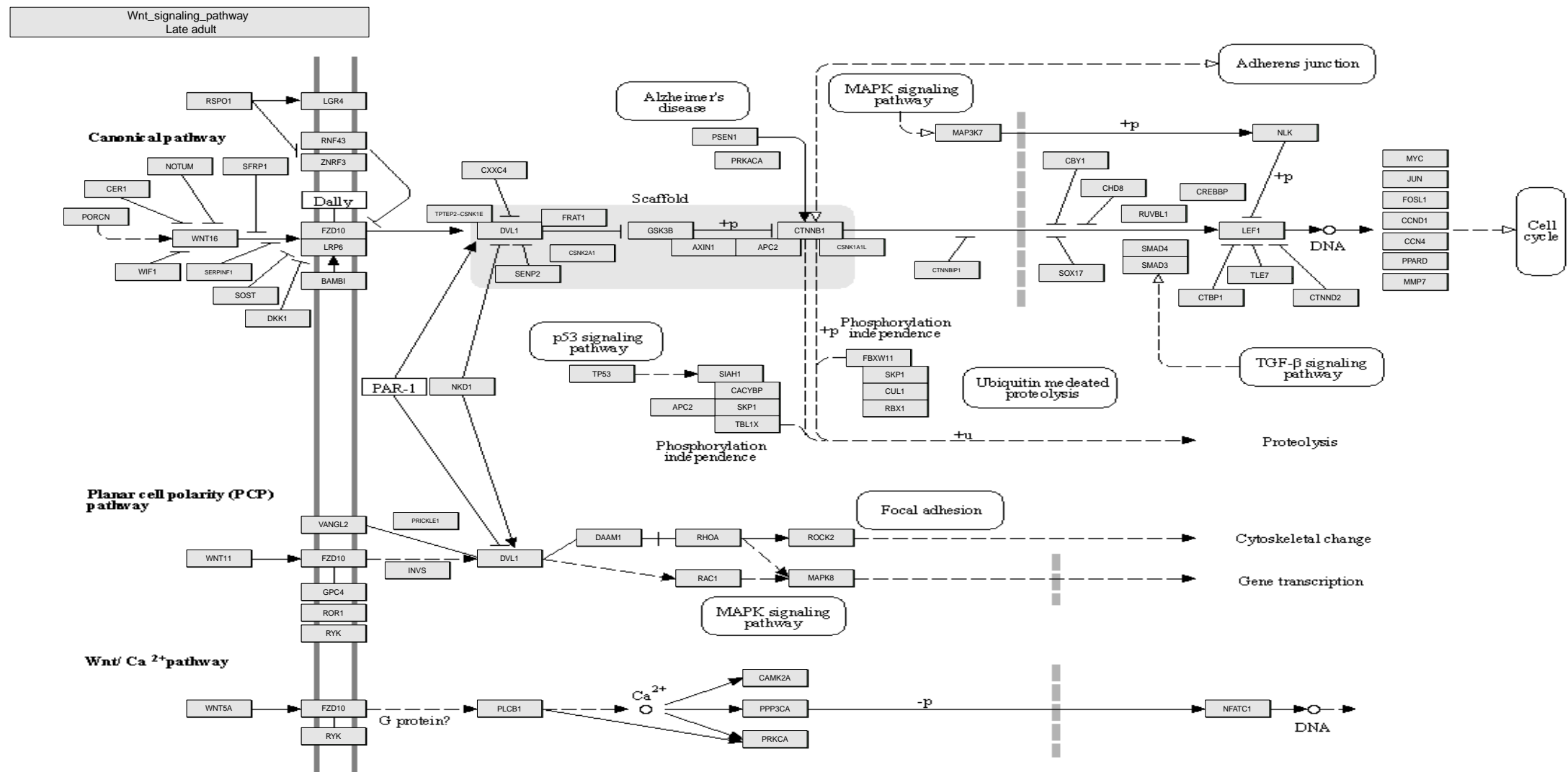

Supplement: Supplementary file 1 [file cells-11-00362-s001.zip › Suppl-Material-S4-Pathways-PSF_Methylation/Wnt_signaling_pathway.pdf]
